# Machine Learning-Based Screening for Potential Singlet Fission Chromophores: The Challenge of Imbalanced Data Sets

**DOI:** 10.1021/acs.jpclett.3c02365

**Published:** 2023-11-03

**Authors:** Lyuben Borislavov, Miroslava Nedyalkova, Alia Tadjer, Onder Aydemir, Julia Romanova

**Affiliations:** † Institute of General and Inorganic Chemistry, Bulgarian Academy of Sciences, 11 Akad. Georgi Bonchev str., 1113 Sofia, Bulgaria; ‡ Chemistry Department, University of Fribourg, Chemin du Musée 9, 1700 Fribourg, Switzerland; § Faculty of Chemistry and Pharmacy, Sofia University, 1 James Bourchier Blvd., 1164 Sofia, Bulgaria; ∥ Faculty of Engineering, Department of Electrical & Electronics Engineering, Karadeniz Technical University, 61080 Trabzon, Turkey

## Abstract

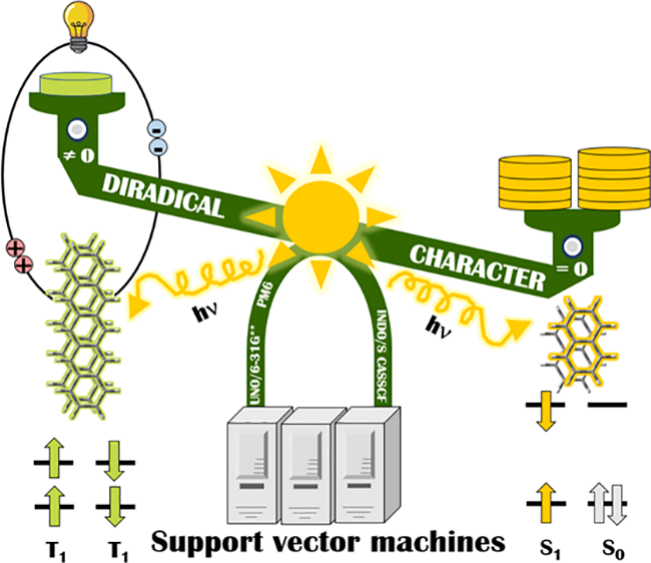

Excitation with one photon of a singlet fission (SF) material generates two triplet excitons, thus doubling the solar cell efficiency. Therefore, the SF molecules are regarded as new generation organic photovoltaics, but it is hard to identify them. Recently, it was demonstrated that molecules of low-to-intermediate diradical character (DRC) are potential SF chromophores. This prompts a low-cost strategy for finding new SF candidates by computational high-throughput workflows. We propose a machine learning aided screening for SF entrants based on their DRC. Our data set comprises 469 784 compounds extracted from the PubChem database, structurally rich but inherently imbalanced regarding DRC values. We developed well performing classification models that can retrieve potential SF chromophores. The latter (∼4%) were analyzed by K-means clustering to reveal qualitative structure–property relationships and to extract strategies for molecular design. The developed screening procedure and data set can be easily adapted for applications of diradicaloids in photonics and spintronics.

The commercially available solar cells use mainly silicon as the photoactive material, which transforms one photon into two free charge carriers.^1^ The disadvantage of the silicon-based photovoltaics is the detailed-balance limit,^2^ which determines that maximum 33% of the incoming solar energy can be converted to electricity. In 2010 Michl et al. demonstrated that the detailed-balance limit can be overcome by exploiting singlet fission (SF)—a photophysical phenomenon observed in some organic chromophores.^3,4^ In SF, a chromophore in a singlet excited state interacts with a neighboring chromophore in the ground state, producing two triplet excitons, thus generating four free charge carriers. Such an increase in the number of free charge carriers per photon can double the solar cells efficiency. At the molecular level, the SF propensity of a chromophore is determined by the energy differences between its first triplet (T_1_), first singlet (S_1_), and second triplet excited states (T_2_). SF occurs spontaneously if the molecule satisfies the following thermodynamic feasibility conditions: 2E(T_1_) – E(S_1_) ≤ 0 and 2E(T_1_) – E(T_2_) ≤ 0, where E is the excitation energy to the respective excited state.^3,4^ Recently, endothermic SF is also experimentally observed in the cases where 2E(T_1_) – E(S_1_) is slightly positive ∼0.2 eV.^5^ However, most of the existing molecules possess a relatively high-energy T_1_ and the feasibility conditions are unfulfilled even when considering slightly endothermic criteria. The hunt for molecules capable of SF is additionally complicated by the requirements to any photoactive solar cell material, namely, an absorption maximum at about 2 eV and air/moisture stability.^3^ Moreover, since the thermal losses in SF are proportional to |2E(T_1_) – E(S_1_)|, this energy difference should be slightly negative and close to zero.^3^

Recently, Nakano and co-workers demonstrated that among organic compounds of low-to-intermediate diradical character (DRC), good candidates can be found that simultaneously satisfy the feasibility conditions and minimize the thermal losses in SF.^6,7^ Therefore, nowadays, DRC is used as a key quantity in the design of SF based photovoltaic materials. DRC can be defined in the framework of the multiconfigurational self-consistent field theory as twice the weight of the doubly excited configuration in the ground state,^8^ and it is not a directly experimentally observable quantity.^9^ DRC is a measure of the open-shell character of the molecules and is related to the energy of T_1_. DRC varies between 0 for closed-shell systems and 1 for ideal diradicals, while the intermediate values correspond to diradicaloids. A classic example for the impact of DRC on the SF propensity can be found in the acenes family. The DRC of anthracene is low,^9^ and it undergoes endothermic SF.^10^ For pentacene, with DRC approaching the intermediate region, the process is exothermic, and this large conjugated hydrocarbon is among the most successful SF materials reported so far.^11^

In brief, the SF chromophores are rare and simultaneously precious treasures that can boost the development of next-generation solar cell technologies. Therefore, we urgently need efficient, computationally inexpensive screening procedures and molecular design strategies for the discovery of new SF chromophores. The bottleneck in the quantum-chemical calculations of SF chromophores is the estimation of the excited states energies.^12^ The standard time-dependent density functional theory^13^ (TD-DFT) approach is questionable for molecules with low to intermediate DRC because of spin contamination^14,15^ and triplet instability^16,17^ problems. The recently developed spin-flip formulation of the method^18,19^ and the Tamm–Dankoff approximation^20^ (TDA) can overcome this problem. However, all TD-DFT approaches are sensitive to the choice of functional, which imposes extensive benchmarking. The alternative approach is to estimate the excitation energies by high-level multiconfigurational methods like CASPT2 and RASPT2.^21−23^ However, the CASPT2/RASPT2 excited-state calculations are resource-consuming, require human-inspected input (active space selection), and are limited to molecules with up to 16/22 π-electrons. Fortunately, the DRC is also associated with the SF propensity, and it can be easily calculated by using a combination of the Yamaguchi’s spin-projection Hartree–Fock scheme^8^ (broken symmetry solution) and natural orbitals.^24,25^ Therefore, an obvious solution of the high-throughput screening problem is to use the DRC as a qualitative criterion for extraction of potential SF chromophores from existing databases.

So far, there have been several studies on the high-throughput screening of large databases for potential SF chromophores. The trend started in 2019, when Perkinson et al. employed a high-throughput procedure to screen 4482 anthracene derivatives and selected 88 (2%) as potential SF candidates.^26^ In the same year, Padula et al. reported a TD-DFT based screening of the Cambridge Structural Database (CCSD) for potential SF materials and found “few needles in a haystack”, namely, 262 (0.7%) SF candidates out of 40K compounds.^27^ As a continuation of this work, in 2021 the same group performed another large-scale computational study which strongly supported the positive relationship between SF propensity and multiple DRC in the selected 262 candidates.^28^ In this work, DRC was calculated with the resource demanding CASSCF method. Meanwhile, the group of Corminboeuf demonstrated a new strategy for design of intermolecular SF materials and screened existing copolymer materials by using high-throughput TDA calculations of donor–acceptor dimers.^29,30^ Recently, Lopez-Carballeira et al. also applied TD-DFT based screening protocol for finding new SF materials in CCSD databases and reported that only 254 molecules (0.87%) match the feasibility conditions out of which only 24 (0.08%) are of practical concern.^31^

Nowadays, we live in the data science era and new evidence is collected daily for the effectiveness of machine learning (ML) algorithms in the discovery of new advanced materials.^32−39^ However, the scientific papers combining ML and SF are intermittent. In 2019, Schröder et al.^40^ used ML to explore the quantum dynamics in pentacene dimers. Later in 2021, Ma and co-workers^41^ developed general transferable multilevel attention neural network for prediction of properties like the energy of HOMO and tested it with the 262 SF candidates of Troisi et al.^27^ The authors reported that the prediction power of their approach is significantly decreased for the SF data set. Again in 2021, Zhu et al. optimized deep neural networks for prediction of excitation energies in SF anthracene-based candidates.^42^ The following three investigations are from 2022. Weber and co-workers used quantum chemical calculations and ML approaches to explore design rules for singlet fission in 4 million indigoid derivatives.^43^ Walsh et al. combined an SF data set and ML to calibrate successfully a high-throughput technique (extended tight binding based simplified TDA approximation) against a higher accuracy one like TD-DFT.^44^ Marom and co-workers employed machine-learning algorithms to generate computationally efficient models that can predict the many-body perturbation theory thermodynamic driving force for SF in a data set of 101 polycyclic aromatic hydrocarbons (PAHs).^45^

Here, we present an ML-based screening procedure for the recognition of potential SF materials from general-purpose chemical databases like PubChem^46^ depending on their DRC. We deployed binary classification ML algorithms, namely, a class weighted support vector machine (SVM) and a cost-sensitive decision tree (DT) to build models that can successfully select prominent SF candidates despite the imbalanced nature of the data set. Molecules meeting the following criteria are extracted from the PubChem database:^46^ to contain between 5 and 28 non-hydrogen atoms (B, C, Si, N, O, S, Se, and F), to possess molecular mass up to 350 Da and low rotable bond count. The feature space covers chemometrics descriptors plus quantum-chemical descriptors for excited states obtained with the recently implemented semiempirical CASSCF method.^47^ The “observable”, DRC, is obtained with the Yamaguchi’s spin-projection scheme.^8^ The preselected potential SF candidates with appropriate E(S_1_) are 17759, and they were subject to cluster analysis and chemical classification for derivation of structure–property relationships.

The overview on the structure and properties of the 469 784 compounds included in the data set can be done by looking at the histograms with respect to key quantum-chemical and chemometrics descriptors (Figure 1). The major part of the entries contains about 10 carbon atoms. The other most abundant elements in the data set are oxygen and nitrogen: generally, up to 3 oxygens and up to 3 nitrogens per compound. About 210 000 of all molecules have no aromatic bonds. These are either compounds with zero DRC, which are *a priori* not suitable for SF, or nonaromatic, antiaromatic, polyenic, and quinoid structures, which in principle can possess nonzero DRC and serve as SF materials. The rest of the compounds have between 5 and 35 aromatic bonds and are consequently benzene derivatives and polycyclic systems. The latter is consistent with the most abundant ring number 2-4.

**Figure 1:**
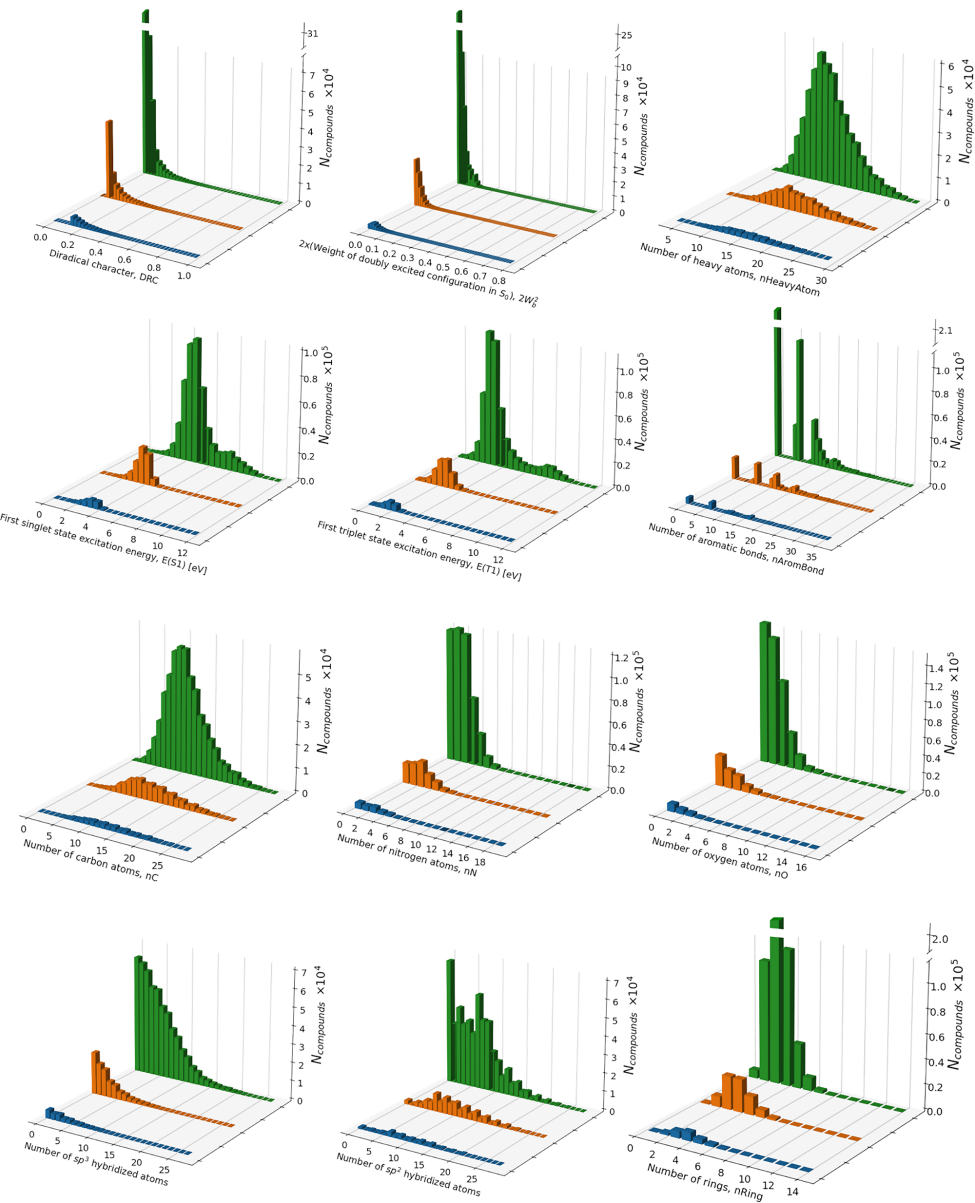
Histograms representing the distribution of DRC and key descriptors in the whole data set of 469 784 compounds (green), as well as in the diradicaloids in DS05 (orange) and in DS13 (blue).

The histogram in Figure 1 shows that the whole data set embraces molecules with DRC between 0.00 and 1.00 but only 318 compounds possess DRC greater than 0.60. To define a binary classification problem, we must divide the whole data set into two classes. *Class 0* is reserved for closed-shell molecules without DRC, and most probably is unsuitable for SF. *Class 1* includes diradicaloids with nonzero DRC, which are possible SF candidates. To separate the classes, we used two DRC thresholds, namely, ≥0.05 and ≥0.13. These give rise to two differently imbalanced data sets, denoted as DS05 and DS13, respectively, used to train our models. The choice of the thresholds is motivated as follows. In DS05, all molecules with DRC below 0.05 can be regarded as closed-shell systems belonging to *Class 0*. Like this, DS05 comprises 384 835 (81.02%) compounds with zero DRC (*Class 0*) and 84 949 (18.08%) molecules with DRC ≥ 0.05 (*Class 1*). In DS13 the threshold is tighter, and it is set with respect to the anthracene DRC = 0.13, obtained with our computational protocol (SI, Table S3). DS13 contains 450 122 (95.82%) compounds with zero DRC (*Class 0*) and 19 662 (4.18%) molecules with DRC ≥ 0.13 (*Class 1*). It is obvious that DS05 and DS13 are imbalanced. As pointed out in the introduction, this imbalanced situation represents an intrinsic particularity of the problem since chromophores with nonzero DRC and SF propensity are known to be scarce. Therefore, both the formulation of the problem and the data set analysis reveal that one should consider the class disparity in the development of successful binary classification models for finding new SF chromophores from general purpose databases.

The S_1_ and T_1_ energies of the compounds in the data set span the wide range of 0–12 eV with maxima in the E(S_1_) and E(T_1_) distribution around 3 and 2 eV, respectively. Here, it is important to note that the computational approach that we use to extract qualitative trends is expected to overestimate E(S_1_) and E(T_1_) (SI, Table S3).^48^ More accurate values for E(S_1_) can be obtained when the configurational and active spaces in the INDO/S CASSCF (2,2) calculations are increased and are selected manually. Nevertheless, even at this relatively low theoretical level, we can find compounds that possess E(S_1_) in the UV–visible range (1.5–3 eV) and are therefore potential SF chromophores for practical photovoltaic application.

Comparison of the ML models as a function of the data set threshold and models’ hyperparameters is represented in Table 1 and Figure 2. The performance of the methods is judged based on the value of PAM (polygon area metric),^49^ which combines all six metrics suitable for measuring the performance of models trained with imbalanced data sets (SI, Sections 2.4–2.5). In all cases the SVM outperforms the DT and the model performance is almost insensitive to the input quantum chemical descriptors E(T_1_) or E (S_1_). The latter is expected since the histograms of these two quantities show qualitatively identical patterns. At first sight, the SVM model with 1:1 class weight (unweighted) for DS05 seems to perform relatively well giving PAM 70.92%, but a careful examination of the metrics reveals that it has lower sensitivity and is therefore unsuitable to find the rare molecules with nonzero DRCs (numerous false negatives). For the more imbalanced DS13 the sensitivity is even lower. The SVM models are improved when the class weights for each data set are optimized by a grid search. Further optimization of the SVM hyperparameters improves the performance (SI, Section 2.4). The best SVM models for DS05 and DS13 have PAM equal to 75.63% and 74.67%, respectively.

**Table 1:** Performance of the ML Models [%] (SI, Section 2.5) on the Test Set as a Function of the Data Set Threshold, Hyperparameters^a^ and Input Quantum Chemical Descriptors (E(T_1_) or E(S_1_)^b^)

	Model											
	SVM (S_1_)						SVM (T_1_)		DT (S_1_)		DT (T_1_)	
Data set	DS05	DS13	DS05	DS05	DS13	DS13	DS05	DS13	DS05	DS13	DS05	DS13
class weights	1:1	1:1	1:1.5	1:1.5	1:2	1:2	1:1.5	1:2				
gamma	0.1	0.1	0.1	0.1	0.1	0.05	0.1	0.05				
C	1.0	1.0	1.0	5.0	1.0	2.0	5.0	2.0				
MD									14	10	14	10
Metric%												
PAM	70.92	65.53	74.00	75.63	72.05	74.67	75.25	73.29	64.85	64.34	64.58	65.41
CA	93.70	98.23	93.68	94.14	98.30	98.36	94.09	98.28	91.83	97.91	91.82	98.01
SE	77.27	68.39	83.67	84.76	78.48	82.24	84.25	80.83	73.43	69.8	72.93	70.53
SP	97.33	99.54	95.89	96.22	99.17	99.07	96.27	99.05	95.9	99.15	96.00	99.22
AUC	87.30	83.96	89.78	90.49	88.82	90.65	90.26	89.94	84.66	84.47	84.46	84.88
JI	68.95	61.89	70.57	72.38	66.00	67.85	72.07	66.4	61.95	58.44	61.75	59.9
FM	70.92	65.53	74.00	83.98	72.05	80.84	83.77	79.81	76.50	73.77	76.35	74.92

a Class weights, gamma—RBF-kernel width, C—cost parameter, and MD—max depth.

b Metric—polygon area metric (PAM), classification accuracy (CA), sensitivity (SE), specificity (SP), area under curve (AUC), Jaccard Index (J), and F-measure (FM). The underlined values of the hyperparameters are optimized by 4-fold cross validation over the training set (SI, Section 2.4).

**Figure 2:**
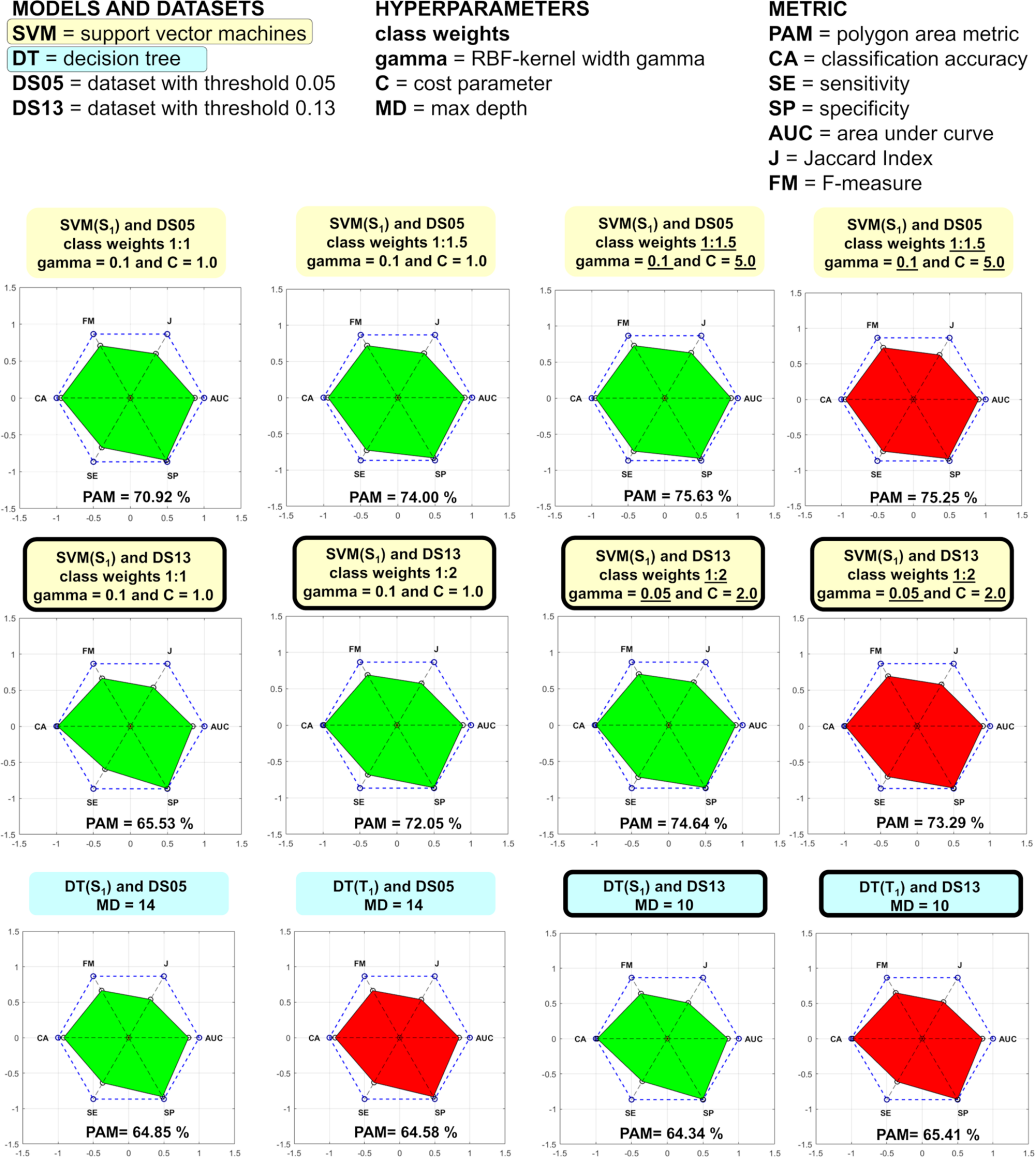
Graphical representation of the performance of the ML models on the test set as a function of the data set threshold (DS13 labels are framed), hyperparameters and input quantum chemical descriptors (either E(T_1_) or E(S_1_) as they correlate). The underlined values of the hyperparameters are optimized by 4-fold cross validation over the training set (SI, Section 2.4).

From a molecular design perspective, it is interesting to analyze the structure and properties of the molecules with nonzero DRC and this is done by comparison of the histograms with the descriptors for the whole data set with histograms for the subset of molecules with DRC ≥ 0.05 and DRC ≥ 0.13 (Figure 1). Regarding the number of heavy atoms, we observe a slight shift in the maximum to higher number of heavy atoms for molecules with nonzero DRC. Such behavior is expected since in principle DRC is proportional to the heavy atom content in conjugated systems. The trend is more pronounced when looking into the carbon atom content. For this quantity, we observe a shift of the peak toward higher values when going from the entire data set to DS13. A clear-cut confirmation of the DRC dependence on the conjugation length can be found in the distributions of the fraction of sp^2^ and sp^3^ carbons. In the whole data set the sp^2^ fraction is with maximum at 0 but it peaks beyond 5 for the subsets with nonzero DRC. The situation with the sp^3^ fraction is reverse.

One of the most important requirements to the SF chromophores is to absorb in the UV–vis region and to possess a relatively low energy E(T_1_). When comparing the excitation energies histograms (Figure 1), we find a gradual shift of the E(S_1_) and E(T_1_) distributions toward lower values with DRC growth. In the whole data set the E(S_1_) distribution has a maximum at above 4 eV, while in DS05 and DS13 the peak is located below 4 eV. Such a decrease in the excitation energies and their shift toward the UV–vis region agrees with the structure of the compounds with nonzero DRC, which, as discussed above, is characterized with better π-conjugation.

Finally, we performed K-means clustering to gain deeper insight into the structure–property relationships of the most promising SF candidates—those with DRC ≥ 0.13 and E(S_1_) in the 1.5–3.0 eV region, ideally around 2 eV. Since with the small active and configurational space E(S_1_) is expected to be overestimated (SI, Table S3), we explore the energy range between 2.0–4.1 eV, where 4.1 eV is the upper limit imposed by E(S_1_) of anthracene and 2.0 is the value 1.5 eV incremented to account for the error of the computed E(S_1_) for anthracene. Among all 469 784 compounds, only 17 759 satisfy the imposed criteria, and they represent 90.32% of all molecules with DRC ≥ 0.13.

The 17 759 molecules were subject to K-means clustering with 16 structural chemometrics descriptors, which divides them into two very well distinguishable clusters (Figure 3). The members of *Cluster1* are characterized with a relatively high content of aromatic bonds, 6-membered rings, fused rings, and heavy atoms among which dominate the sp^2^-carbons. The prevailing features in *Cluster2* are opposite, and its members have lower mean values for the number of aromatic bonds, 6-membered rings, fused cycles, heavy atoms, and sp^2^-carbons. The behavior of the other descriptors reveals that *Cluster 2* is richer in nitrogen, oxygen, and single bonds. It is worth noting that, although clearly distinguishable with respect to the structural patterns, both clusters share almost identical mean DRC values.

**Figure 3:**
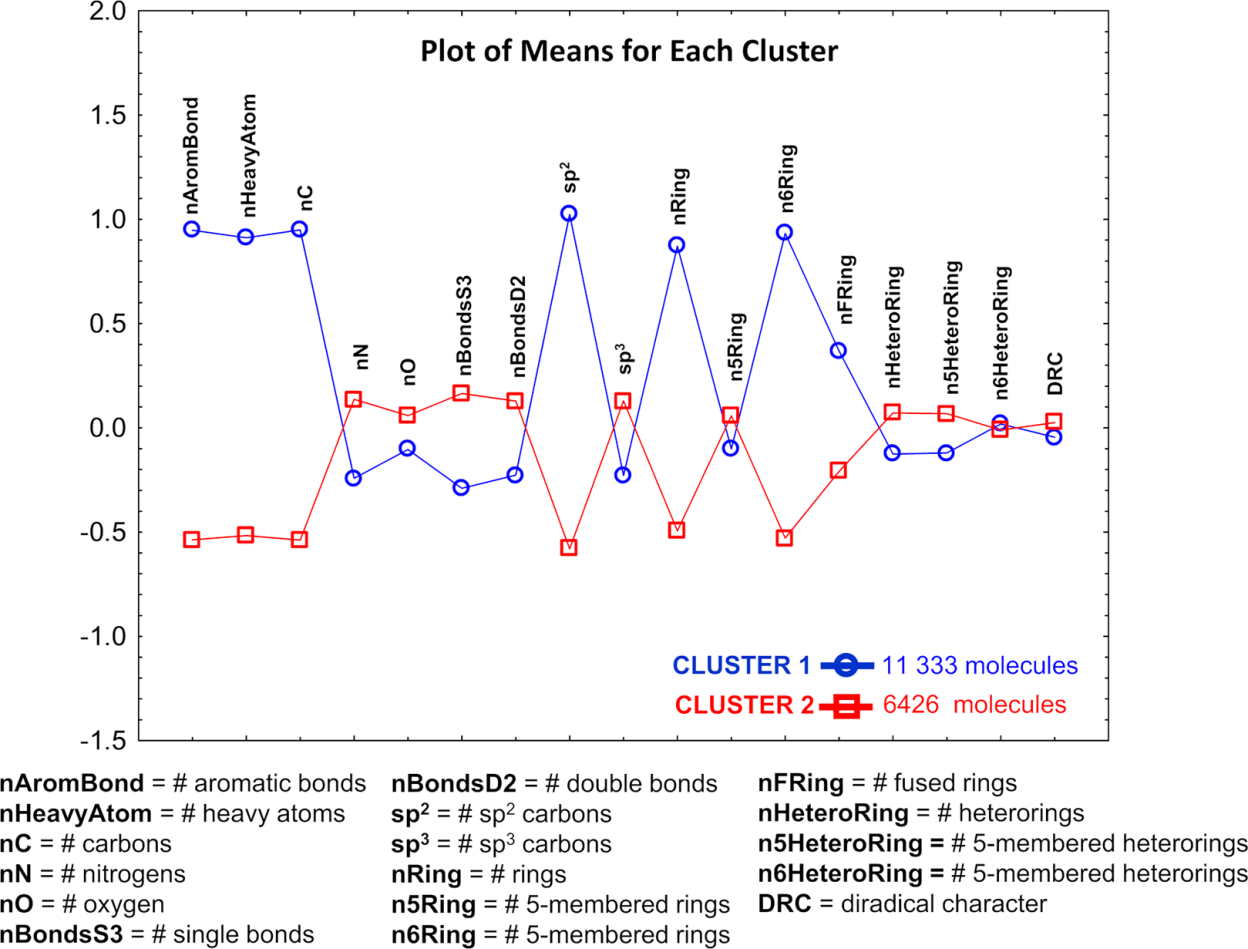
K-means cluster analysis of 17 759 molecules with DRC ≥ 0.13 and E(S_1_) in the range 2.0–4.1 eV. Structural descriptors underlying the clustering: 0 corresponds to the mean value of each descriptor for all molecules, while positive and negative values correspond to deviation from the mean value.

Figure 4 summarizes families of potential SF chromophores belonging to *Cluster 1* or 2. Careful examination of the members in the clusters (SI) confirms the derived structure-property relationships. In *Cluster 1* one can find the classical examples for SF chromophores—anthracene, tetracene, and pentacene—but also other PAHs like benzotetracene, perylene, benzoperylene, etc. Therefore, *Cluster 1* contains mainly PAHs and their doped or functionalized derivatives. Following the descriptors pattern and the structural differences between the clusters (Figure 3), it is obvious that most of the diradicaloids in *Cluster 1* are stabilized by the presence of Clar’s sextets.^50^*Cluster 2* is composed mainly of smaller in size molecules of quinoid type with 1–3 cycles (rarely 4), high heteroatoms/carbon ratio, and mixed heteroatomic content. Members of *Cluster 2* are also polyenes and molecules with nonaromatic and antiaromatic rings.

**Figure 4:**
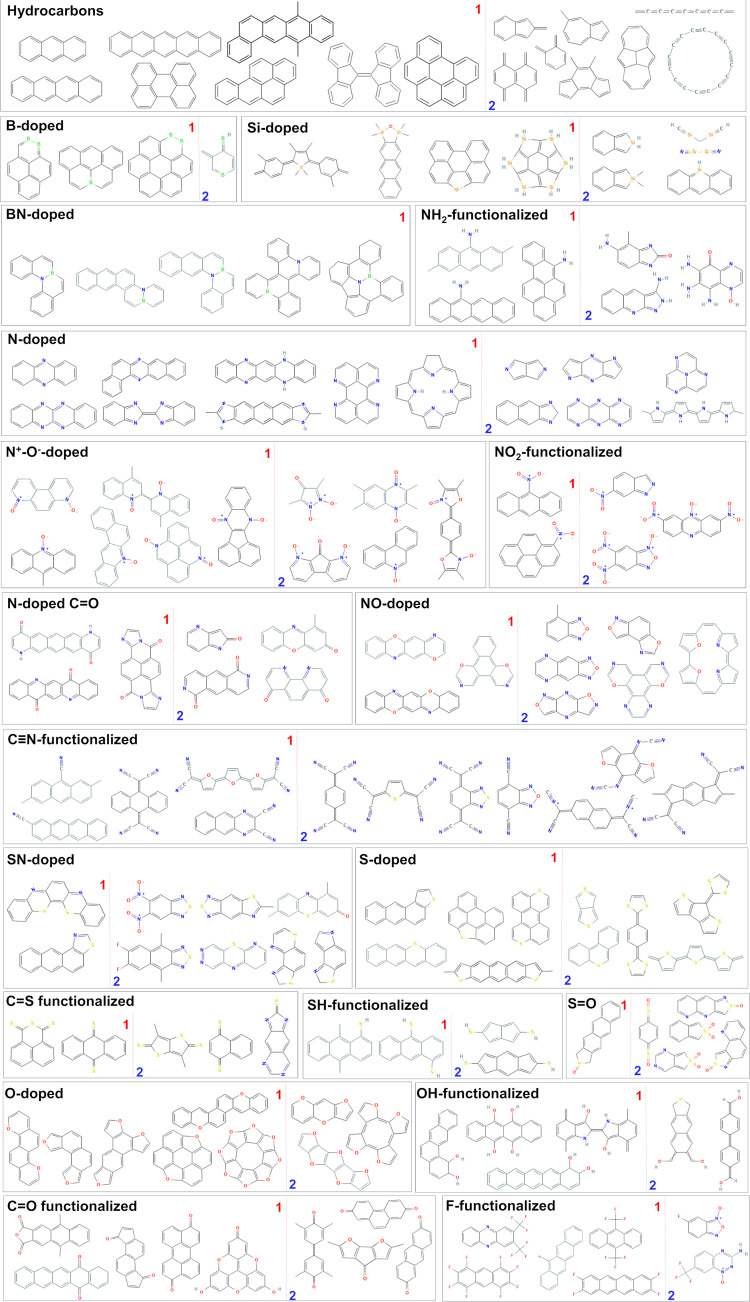
Summary of the families of potential SF chromophores belonging to *Cluster 1* (left from label 1) or *Cluster 2* (right from label 2). The 2D structure images are extracted from PubChem.^46^

As can be seen, albeit simple, the computational approach confirms and naturally summarizes many of the published molecular design strategies in the SF area.^4,27,51−64^ The hunted diradicaloids belong to a wide assortment of structural motifs in organic conjugated compounds, and from a DRC perspective the design strategies are broad and worth further investigation. Our approach significantly shortlists the possible SF candidates to 4% but at the same time the good catch of 17 759 compounds shows that the DRC criterion is not as tight as the feasibility conditions. Therefore, for a strict selection of potential photovoltaics candidates, subsequent high-level quantum chemical estimation of the SF thermodynamic requirements is needed. Brief discussion on the limitations and applicability of the model can be found in the SI (Page S21).

In summary, we demonstrate binary classification models to screen general purpose data sets for potential SF candidates based on their DRC. The advantage of our ML approach is that the training data set is simultaneously large enough and structurally diverse and includes input features, quickly obtainable even on a desktop. The ML simulations reveal that well performing models for sieving of SF molecules from general-purpose chemical databases should consider the imbalanced character of the data—a specificity, which originates from the experimentally known fact that these chromophores are relatively rare troves. As a result of the screening procedure, several thousand potential chromophores were preselected based on their DRC and were subject to cluster analysis to explore structure–propertiy relationships. The K-means clustering confirms and logically summarizes many of the published molecular design strategies in the singlet fission area, which demonstrates that data-oriented approaches can be applied successfully in the singlet fission domain. The study confirms the suitability of known SF materials, shortlists new potential compounds, and sifts structures for further computational workflows aiming at multiconfigurational estimation of the feasibility conditions or combinatorial generation of intramolecular SF chromophores. Moreover, since the diradicaloids are attractive in many other application areas of organic molecules,^86−90^ the developed screening procedure is expected to serve as an inspiration in the design of materials beyond the SF domain. The best optimized ML model is implemented in a user-friendly web application,^91^ where chemists can check the SF potential of newly designed molecules.

## Computational Methods

Step by step data set preparation, computational protocols, ML models training, metric explanation, and k-means analysis details can be found in the SI, Sections 1–5. The molecules were extracted from the PubChem database^46^ and contain between 5 and 28 heavy atoms (B, C, Si, N, O, S, Se, and F) and have molecular mass up to 350 Da and low rotable bond count. After structural refinement, the Cartesian coordinates of the compounds were obtained from the SMILES codes by using OPENBABEL.^65^ The structures were subject to geometry optimization and frequency analysis at the PM6 level,^66^ INDO/S CASSCF (2,2) excited states calculations,^47^ chemometrics features generation, and DRC computations with spin projected PUHF/6-31G** method.^8^ The final data set of 469 784 compounds and their descriptors were used to train a class-weighted support-vector machine,^67−70^ and a cost-sensitive decision tree^71−75^ models to sort out potential SF chromophores. The scikit-learn^76^ (version 1.3.0) and LIBSVM^77^ libraries were used to perform the ML classification. The models are optimized and compared by using a metric suitable to judge the performance of imbalanced classification such as the polygon area metric^49^ (SI, Section 2.5), which constructs a polygon in a regular hexagon with six widely used metrics. The semiempirical and DRC calculations are done by OPENMOPAC^78^ and Gaussian 09,^79^ respectively. The chemometrics descriptors are generated by PaDel,^80^ the ML metrics are visualized by using MATLAB,^81^ and the K-means clustering^82,83^ is performed with STATISTICA.^84^ All data and ML codes are open-source^85^ and available on GitHub (SI, Section 6).

## Supporting Information Available

Detailed information on the data set construction, computational procedure, ML models, and statistical approaches (Sections 1–5,7) and links to open access data sets and codes (Section 6) (PDF)
The full record URLs to the 2D structure images of 17 759 compounds extracted from PubChem (PDF)
