# Supplementary material for: Machine Learning-Based Screening for Potential Singlet Fission Chromophores: The Challenge of Imbalanced Data Sets
Source: J Phys Chem Lett. 2023 Nov 3;14(45):10103–12. doi: 10.1021/acs.jpclett.3c02365 (PMC10659028; doi:10.1021/acs.jpclett.3c02365)
Supplement: Supplementary file 1 — jz3c02365_si_001.pdf [file jz3c02365_si_001.pdf]

SUPPORTING INFORMATION

Machine Learning-Based Screening for Potential Singlet Fission Chromophores: The Challenge of Imbalanced Data Sets

Lyuben Borislavov[1], Miroslava Nedyalkova[2,3], Alia Tadjer[3], Onder Aydemir[4], Julia Romanova[3,]*

[1] Institute of General and Inorganic Chemistry, 11 Akad. Georgi Bonchev str., 1113 Sofia, Bulgaria

[2] Chemistry Department, University of Fribourg, Chemin du Musée 9, 1700 Fribourg, Switzerland

[3] Faculty of Chemistry and Pharmacy, Sofia University, 1 James Bourchier blvd. 1164 Sofia, Bulgaria

[4] Faculty of Engineering, Department of Electrical & Electronics Engineering, Karadeniz Technical University, 61080 Trabzon, Turkey

Corresponding Author: *jromanova@chem.uni-sofia.bg

CONTENT

1. COMPUTATIONAL PROTOCOL AND DATASET CONSTRUCTION… S2
1.1. Compounds extraction from Pubchem…………………………………….. S2
1.2. Semi-empirical descriptors………………………………………………. S3
1.3. Chemometric descriptors………………………………………………… S4
1.4. Diradical character (DRC)……………………………………………….. S6

2. MACHINE LEARNING APPROACHES……………………………….. S7
2.1.Class-weighted support vector machine………………………………… S7
2.2.Cost-sensitive decision tree………………………………………………. S8
2.3.Reduction of the feature space…………………………………………… S8
2.4.Metric…………………………………………………………………….... S12
2.5.Models training………………………………………………………... S12

3. BENCHMARK ON DRC CALCULATIONS…………………………….. S17

4. RESULTS ON ACENES…………………………………………………. S19

5. K-MEANS CLUSTERING ANALYSIS…………………………………. S19

6. SUMMARY OF OPEN ACCESS DATA AND CODES…………………… S20

7. DISCUSSION ON THE LIMITATIONS AND APPLICABILITY OF THE ML MODEL……………………………………….. S21

Important note: The references numbering in the manuscript and in the SI is identical.

1. COMPUTATIONAL PROTOCOL AND DATASET CONSTRUCTION

The step-by-step protocol for dataset construction is represented in **Scheme S1**. Detailed information about each computational and refinement step is provided in subsections 1.1.-1.4.

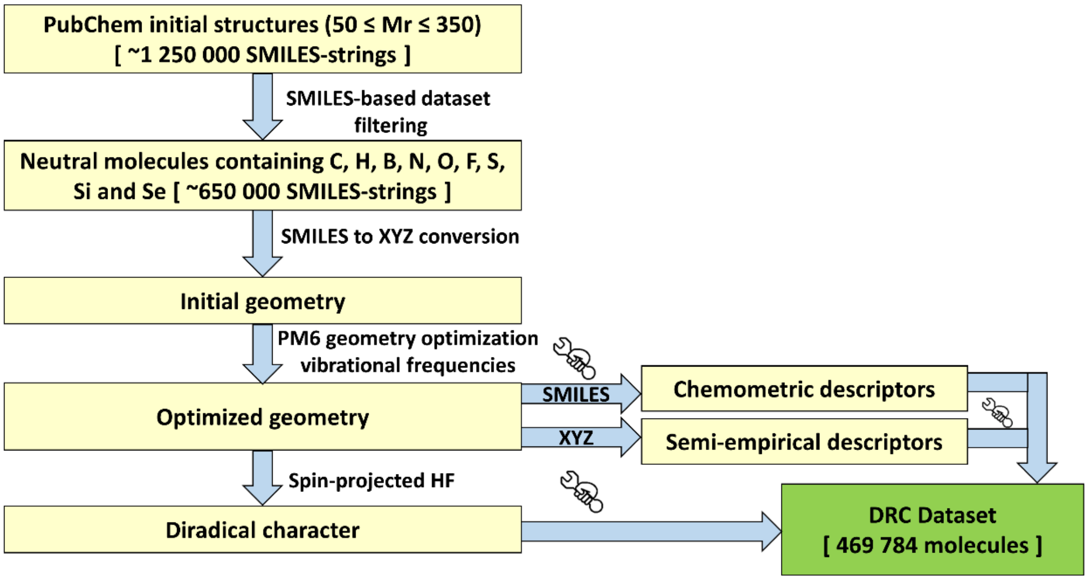

Scheme S1. Dataset generation and computational approach.

1.1. Compounds extraction from Pubchem[46]

The SMILES codes of approximately 1 250 000 molecules were extracted from the PubChem database, which is the world's largest open-access collection of chemical compounds. We have selected all chemicals with up to 28 heavy atoms, molecular weight between 50 and 350 Da, neutral charge and low rotable bond count. The target rotable bond count is kept low in order to hit relatively planar molecular systems, i.e. conjugated ones, and to avoid conformationally complex compounds because the effect of conformation on the singlet fission propensity of the molecules is out of the scope of this study. In particular, the PubChem query is:

Query "((((5[HeavyAtomCount] : 28[HeavyAtomCount]) AND 000000000000[TotalFormalCharge] : 000000000000[TotalFormalCharge]) AND 0[RotatableBondCount] : 0[RotatableBondCount]) AND

0000050.000000[MolecularWeight] : 0000350.000000[MolecularWeight]) AND 0000010.0[Complexity] : 0002000.0[Complexity]'

Next, by using the SMILES codes the structures were refined as follows:

- Compounds containing any chemical element different from C, O, N, Si, S, Se, F, B, and H were discarded
- All salts (ionic bond is present – SMILES string ".") and compounds containing quadrupole bonds (SMILES string "$") were discarded
- All chiral compounds (SMILES string "@") were discarded

At this refinement step approximately 650 000 molecules remained. Their SMILES strings were subsequently converted to Cartesian coordinates utilizing the OpenBabel[65] software package. The Cartesian representations of the compounds were used to obtain the semi-empirical features, while chemometric descriptors were computed from the corresponding SMILES strings.

1.2. Semi-empirical descriptors

The molecular structures of all compounds have been optimized at the semi-empirical level with the PM6 method[66]. In the next step all structures were subject to frequency analysis with the same method in order to confirm that they represent minima on the potential energy surface. The optimized geometries with imaginary frequencies were discarded from the dataset.

The vertical excitation energies were calculated on top of the PM6 geometries by using the complete active space self-consistent field theory (CASSCF) in combination with the semi-empirical INDO/S method.[47] Two orbitals and two electrons, i.e. HOMO and LUMO, are included in the active space (2,2). This minimal active space allows us to estimate only the excitation energy to the first (S_1) and second (S_2) singlet excited states and the corresponding oscillator strengths, as well as the excitation energy to the first triplet excited state (T_1). On the other hand, its size is sufficient to estimate extremely quickly the weight of the doubly excited configuration (W_b) in the ground state at the semi-empirical level, which is proportional to the DRC.

At this stage compounds with negative first triplet excitation energies were discarded, since their diradical character is too large for SF application. Additionally, the molecules for which an error or warning messages appeared in the output files of the excited-state calculation were discarded.

All semi-empirical calculations were performed with the OPENMOPAC program.[78]

1.3. Chemometric descriptors

Chemometric descriptors related to the chemical composition and topology of the compounds in the dataset were generated from the corresponding SMILES string representations using the PaDel software package[80]. Descriptors related to aromaticity and conjugation were also selected since they correlate with the diradical character. Mean value, median and mode were calculated for all chemometric descriptors in the dataset. If for 60 % or more of the molecules the value of a given descriptor was equal to the descriptor mode, then the descriptor was discarded.

Table S1: Complete list of descriptors in the dataset.

Descriptor	Description
S1(CAS2-1) [eV]	First singlet state excitation energy
DS2(CAS2-1) [eV]	First doubly excited state excitation energy
INDOS-OscStr(CAS2-1)	S0->S1 oscillator strength
CI-Coef**2(CAS2-1)	Coefficient of the doubly excited configuration in the ground state
T1(CAS2-1) [eV]	First triplet state excitation energy
ALogP	Ghose-Crippen LogKow
ALogP2	Square of ALogP
AMR	Molar refractivity
apol	Sum of the atomic polarizabilities (including implicit hydrogens)
naAromAtom	Number of aromatic atoms
nAromBond	Number of aromatic bonds
nAtom	Number of atoms
nHeavyAtom	Number of heavy atoms (i.e. non-hydrogen)
nH	Number of hydrogen atoms
nC	Number of carbon atoms
nN	Number of nitrogen atoms
nO	Number of oxygen atoms

nBonds	Number of bonds (excluding bonds with hydrogen)
nBonds2	Total number of bonds (including bonds to hydrogens)
nBondsS	Number of single bonds (including bonds with hydrogen)
nBondsS2	Total number of single bonds (including bonds to hydrogens, excluding aromatic bonds)
nBondsS3	Total number of single bonds (excluding bonds to hydrogens and aromatic bonds)
nBondsD	Number of double bonds
nBondsD2	Total number of double bonds (excluding aromatic bonds)
nBondsM	Total number of bonds that have bond order greater than one (aromatic bonds have bond order 1.5).
C1SP2	Doubly bound carbon bound to a single other carbon
C2SP2	Doubly bound carbon bound to two other carbons
C3SP2	Doubly bound carbon bound to three other carbons
C1SP3	Singly bound carbon bound to a single other carbon
C2SP3	Singly bound carbon bound to two other carbons
Sv	Sum of atomic van der Waals volumes (scaled on carbon atom)
Sse	Sum of atomic Sanderson electronegativities (scaled on carbon atom)
Spe	Sum of atomic Pauling electronegativities (scaled on carbon atom)
Sare	Sum of atomic Allred-Rochow electronegativities (scaled on carbon atom)
Sp	Sum of atomic polarizabilities (scaled on carbon atom)
Si	Sum of first ionization potentials (scaled on carbon atom)
Mv	Mean atomic van der Waals volumes (scaled on carbon atom)
Mse	Mean atomic Sanderson electronegativities (scaled on carbon atom)
Mpe	Mean atomic Pauling electronegativities (scaled on carbon atom)
Mare	Mean atomic Allred-Rochow electronegativities (scaled on carbon atom)
Mp	Mean atomic polarizabilities (scaled on carbon atom)
Mi	Mean first ionization potentials (scaled on carbon atom)
ECCEN	A topological descriptor combining distance and adjacency information
hmax	Maximum H E-State
hmin	Minimum H E-State
nRing	Number of rings
n5Ring	Number of 5-membered rings
n6Ring	Number of 6-membered rings
nFRing	Number of fused rings
nTRing	Number of rings (includes counts from fused rings)

nT5Ring	Number of 5-membered rings (includes counts from fused rings)
nT6Ring	Number of 6-membered rings (includes counts from fused rings)
nHeteroRing	Number of rings containing heteroatoms (N, O, P, S, or halogens)
n5HeteroRing	Number of 5-membered rings containing heteroatoms (N, O, P, S, or halogens)
n6HeteroRing	Number of 6-membered rings containing heteroatoms (N, O, P, S, or halogens)
nT5HeteroRing	Number of 5-membered rings (includes counts from fused rings) containing heteroatoms (N, O, P, S, or halogens)
nT6HeteroRing	Number of 6-membered rings (includes counts from fused rings) containing heteroatoms (N, O, P, S, or halogens)
nRotBt	Number of rotable bonds, including terminal bonds
RotBtFrac	Fraction of rotable bonds, including terminal bonds
topoRadius	Topological radius (minimum atom eccentricity)
topoDiameter	Topological diameter (maximum atom eccentricity)
topoShape	Petitjean topological shape index

1.4.Diradical character (DRC)

Spin-projected Hartree-Fock (PUHF) calculations[8] with 6-31G** basis set were performed using the PM6 geometries as input. In the spin-projected PUHF theory the diradical character (y_0, i=0), related to HONO and LUNO, and the tetraradical character (y_1, i=1), associated with HONO-1 and LUNO+1, can be estimated as:

$$y_i = 1 - \frac{2T_i}{1 + T_i^2}$$

where T_i represents the overlap integral between the corresponding orbital pairs. T_i is calculated from the UHF natural orbitals occupation numbers (n) of the UHF natural orbitals[24-25]:

$$T_i = \frac{n_{HONO-i} - n_{LUNO+i}}{2}$$

It is very important to note that in order to ensure convergence of the broken-symmetry solution, the following keywords were used to calculate the diradical character: *# 6-31G** UHF pop=NO guess=mix nosymm stable=opt*. The additional keywords *nosymm* and *stable=opt* (wavefunction RHF → UHF stability check) definitely increase the computational time but without them many diradicaloids remain hidden.

Since the PUHF/6-31G** computations are usually performed on top of structures optimized at the density functional level of theory, we have performed a benchmark study to assess our approach based on PM6 geometries in quantitative aspect. For more information, please see Section 3 of the SI.

All spin-projected calculations of the diradical character were done by using Gaussian 09[79].

After all refinements described in Sections 1.1-1.4, the dataset contains 469 784 compounds.
The final dataset is freely available online: https://github.com/ml4sf/ml4sf-500kdataset (https://github.com/ml4sf/ml4sf-500kdataset/blob/master/datasets/Dataset470K.csv.lrz)

2. MACHINE LEARNING APPROACHES

Machine learning approaches have successfully been used to address many regression and classification problems in chemistry[85]. In the present study we developed a data-driven high-throughput screening procedure that is able to identify potential singlet-fission chromophores based on their diradical character. Since molecules that could undergo singlet-fission comprise a fairly small fraction of the chemical space, the machine learning approaches used must be able to handle the inherited class imbalance of the dataset. Therefore, in this study were trained and tested classification algorithms that are known to perform quite well on imbalanced datasets[68,72] such as cost-sensitive decision trees and class-weighted support vector machines.

2.1. Class-weighted support vector machines (SVM)

The support vector machines (SVMs) are robust algorithms for non-probabilistic binary classification that are known to exhibit high generalization ability[67]. SVMs map input data (in our case semi-empirical and chemometric descriptors) to some high-dimensional feature space by using an *a priori* known non-linear mapping and subsequently perform linear separation in that feature space. SVMs have been successfully applied to different structure-property relationships problems in chemistry[69] and their class-weighted version is known to perform fairly well on imbalanced datasets[70].

In the present study a class-weighted SVM with a radial basis function (RBF) kernel as implemented in the scikit-learn library[76] was used. The scikit-learn implementation of SVMs on its turn is based on the LIBSVM library[77].

2.2. Cost-sensitive decision tree (DT)

First introduced in 1987 the classification DT[71]is a hierarchical supervised machine learning model that logically combines a sequence of decisions based on simple tests and their possible outcomes. This is achieved by optimizing the simple test condition threshold during the training process[74]. Compared to other widely used machine learning algorithms, DTs are simpler to understand and can be visualized. The DT has been extensively utilized for classification in the field of qualitative structure-property relationships studies[73] and its cost-sensitive version performs well in the case of imbalanced datasets problems. [75]

In the present study a cost-sensitive decision tree algorithm with a Gini classification criterion as implemented in the scikit-learn library was used. [76]

The codes for the models described in Sections 2.1-2.2 are freely available online:
Class-weighted support vector machines:
https://github.com/ml4sf/ml4sf-500kdataset/tree/master/svm
Cost-sensitive decision tree:
https://github.com/ml4sf/ml4sf-500kdataset/tree/master/dtree

2.3.Reduction of the feature space

The feature space for training the models was selected based on the correlation matrix for the final dataset, represented in **Figure S1**. The models were trained mainly with mutually independent descriptors, which correlate well with the "observable" – the diradical character (**Table S2**).

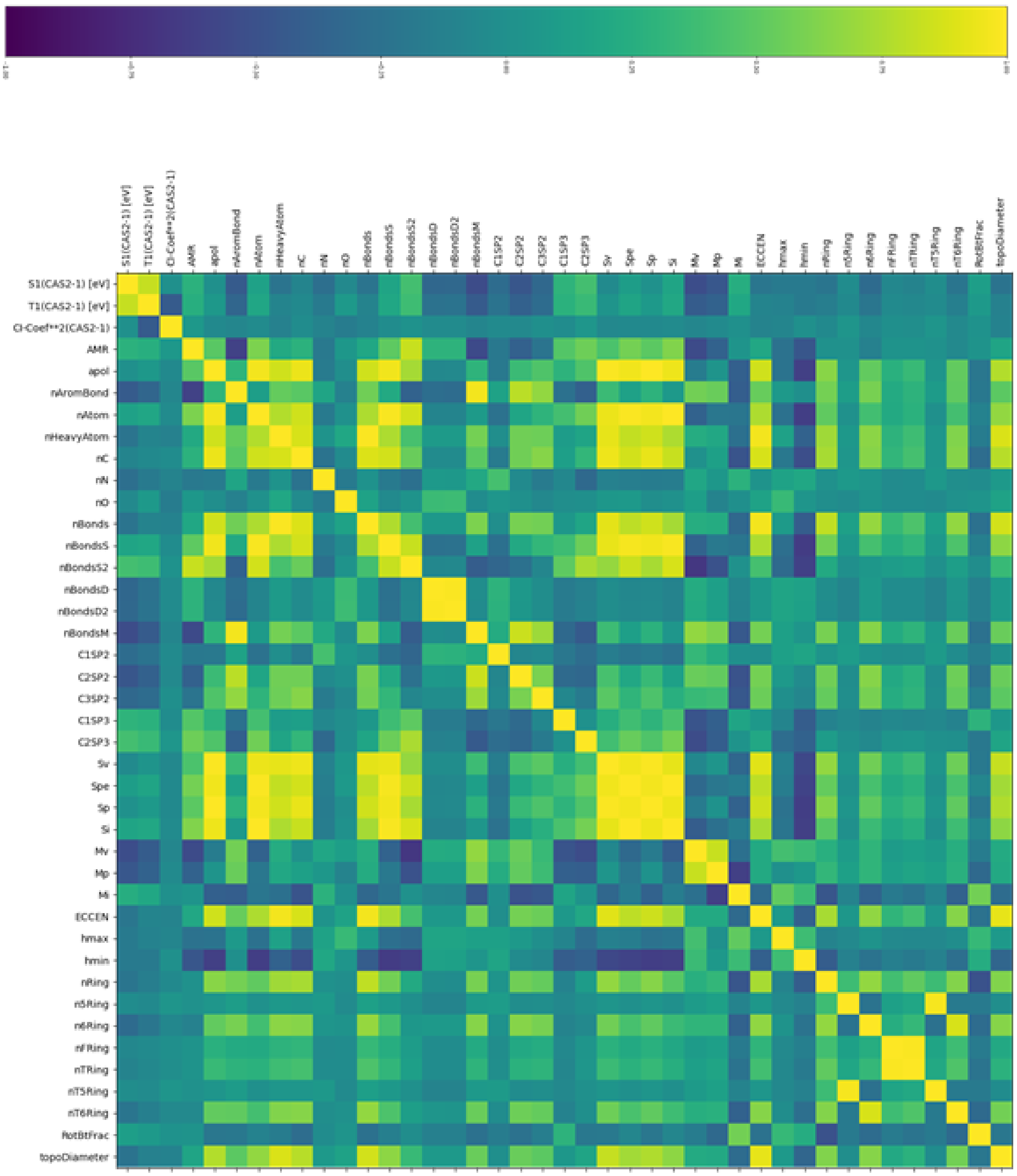

Figure S1 The correlation matrix of the descriptors used to train the SVM and DT models. Positive and strong correlation (+1) is for the yellow end, while negative and strong correlation (-1) is reserved for the blue end. The feature space includes mainly uncorrelated descriptors.

Table S2: DRC – descriptor correlation values. The descriptors used for performing machine learning training are marked in green.

Descriptor	Correlation (Descriptor, DRC)
S1(CAS2-1) [eV]	-0.443
DS2(CAS2-1) [eV]	-0.396
INDOS-OscStr(CAS2-1)	0.177
CI-Coef**2(CAS2-1)	0.337
T1(CAS2-1) [eV]	-0.396
ALogP	0.091
ALogp2	-0.021
AMR	-0.129
apol	0.134
naAromAtom	0.323
nAromBond	0.328
nAtom	0.045
nHeavyAtom	0.234
nH	-0.090
nC	0.233
nN	0.063
nO	-0.041
nBonds	0.253
nBonds2	0.073
nBondsS	0.050
nBondsS2	-0.139
nBondsS3	-0.179
nBondsD	0.117
nBondsD2	0.106
nBondsM	0.373
C1SP2	0.036
C2SP2	0.382
C3SP2	0.329
C1SP3	-0.192
C2SP3	-0.149

Sv	0.160
Sse	0.047
Spe	0.055
Sare	0.053
Sp	0.129
Si	0.026
Mv	0.275
Mse	-0.005
Mpe	0.033
Mare	0.020
Mp	0.260
Mi	-0.219
ECCEN	0.252
hmax	0.009
hmin	0.052
nRing	0.272
n5Ring	-0.010
n6Ring	0.302
nFRing	0.147
nTRing	0.177
nT5Ring	-0.009
nT6Ring	0.276
nHeteroRing	0.001
n5HeteroRing	-0.012
n6HeteroRing	0.044
nT5HeteroRing	-0.012
nT6HeteroRing	0.034
nRotBt	-0.069
RotBtFrac	-0.128
topoRadius	0.175
topoDiameter	0.231
topoShape	0.078
DRC	1.000

2.4.Metric

Performance evaluation of a classification model is a central task in the field of pattern recognition and machine learning. To do this, various kinds of performance metrics, including classification accuracy (CA), sensitivity (SE), specificity (SP), area under curve (AUC), Jaccard Index (J), and F-measure (FM), have been used by the researchers. While the CA might be enough to assess a classification model for a dataset with a balanced label distribution, it might be often a complex task for the imbalanced datasets. Because the considered dataset in this study has an imbalanced label distribution, we calculate the polygon area metric (PAM), which constructs a polygon in a regular hexagon with six widely used metrics (CA, SE, SP, J, AUC and FM) and calculates the area of this polygon as an evaluation metric between 0 and 1.[49] Moreover, PAM not only yields a visual graph, which helps for a detailed model assessment in terms of the considered six metrics, but it also provides a rapid comparison of the classification model performance using only one scalar metric value: the greater the polygon area occupied in the hexagon, the better the performance of the classifier model and vice versa. The metric is visualized with MATLAB. [81]

2.5. Models training

In the models training, the datasets were split into a training set containing 75% of the compounds and a test set comprising 25% of the molecules. The class ratio in the training and test sets is similar (for DS13: training set 4.18% molecules with DRC > 0.13, test set: 4.21% molecules with DRC > 0.13; for DS05: training set 18.07 % molecules with DRC > 0.05, test set: 18.11% molecules with DRC > 0.05).

2.5.1. Decision tree (DT)

The maximal allowed depth (MD) of a decision determines whether the decision tree underfits or overfits the data. The optimal value of MD was estimated by means of a grid search: MD was varied from 2 to 38 in steps of 2 and the mean value of 4-fold cross validation F-metric (FMCV (mean)) was used as a model performance metric during the hyperparameter optimization (**Table S3**). The values of FM metric over the training set were considered to take into account overfitting (i.e. in case the overfitting FM metric over the training set is considerably larger than the mean of cross-validation FM metric).

Table S3. Decision tree maximal allowed depth (MD) optimization for DS05 and DS13 datasets. The FM (train) is the F-measure over the training set, F1CV*i* (*i* = 1, 2, 3, 4) is the F-measure of the *i*-th cross-validation fold, FMCV (mean) is the mean of F1CV1, F1CV2, F1CV3 and F1CV4. Descriptors used: ['S1(CAS2-1) [eV]', 'CI-Coef**2(CAS2-1)', 'AMR', 'apol', 'nAromBond', 'nAtom', 'nHeavyAtom', 'nC', 'nN', 'nO', 'nBonds', 'nBondsS', 'nBondsS2', 'nBondsD', 'nBondsD2', 'nBondsM', 'C1SP2', 'C2SP2', 'C3SP2', 'C1SP3', 'C2SP3', 'Sv', 'Spe', 'Sp', 'Si', 'Mv', 'Mp', 'Mi', 'ECCEN', 'hmax', 'hmin', 'nRing', 'n5Ring', 'n6Ring', 'nFRing', 'nTRing', 'nT5Ring', 'nT6Ring', 'RotBtFrac', 'topoDiameter'].

DS05						
**MD**	**FM (train)**	**F1CV1**	**F1CV2**	**F1CV3**	**F1CV4**	**FMCV (mean)**
2	0.613	0.617	0.610	0.613	0.611	0.613
4	0.585	0.583	0.581	0.585	0.586	0.584
6	0.693	0.684	0.642	0.680	0.686	0.673
8	0.716	0.706	0.703	0.717	0.713	0.710
10	0.759	0.740	0.741	0.736	0.736	0.738
12	0.798	0.746	0.747	0.748	0.744	0.746
**14**	**0.845**	**0.759**	**0.752**	**0.755**	**0.749**	**0.754**
16	0.888	0.759	0.753	0.754	0.755	0.755
18	0.927	0.753	0.751	0.751	0.754	0.752
20	0.956	0.749	0.750	0.750	0.749	0.749
22	0.976	0.748	0.746	0.748	0.749	0.748
24	0.988	0.743	0.746	0.745	0.745	0.745
26	0.994	0.742	0.744	0.746	0.745	0.744
28	0.998	0.741	0.746	0.743	0.745	0.744
30	0.999	0.740	0.744	0.744	0.744	0.743
32	1.000	0.742	0.743	0.746	0.747	0.745
34	1.000	0.740	0.745	0.743	0.745	0.743
36	1.000	0.739	0.741	0.745	0.747	0.743
38	1.000	0.742	0.742	0.743	0.745	0.743

DS13						
**MD**	**FM (train)**	**F1CV1**	**F1CV2**	**F1CV3**	**F1CV4**	**FMCV (mean)**
2	0.540	0.538	0.544	0.527	0.543	0.538
4	0.580	0.574	0.581	0.544	0.576	0.569
6	0.647	0.653	0.615	0.632	0.658	0.640
8	0.732	0.723	0.691	0.693	0.709	0.704
**10**	**0.794**	**0.731**	**0.716**	**0.720**	**0.723**	**0.723**
12	0.852	0.735	0.720	0.720	0.727	0.725
14	0.905	0.726	0.712	0.718	0.721	0.719
16	0.944	0.722	0.712	0.715	0.715	0.716
18	0.968	0.721	0.705	0.712	0.717	0.714
20	0.982	0.718	0.697	0.702	0.710	0.707
22	0.989	0.713	0.696	0.704	0.708	0.706
24	0.994	0.711	0.696	0.701	0.709	0.704
26	0.997	0.708	0.696	0.695	0.703	0.701
28	0.998	0.712	0.691	0.697	0.701	0.700
30	0.999	0.709	0.692	0.691	0.705	0.699
32	1.000	0.711	0.691	0.693	0.702	0.699
34	1.000	0.707	0.689	0.695	0.702	0.698
36	1.000	0.705	0.695	0.696	0.702	0.699
38	1.000	0.708	0.696	0.697	0.702	0.701

The optimal MD value for DS05 is 14: although the value of FMCV (mean) is by 0.001 smaller than the maximal FMCV (mean) value, the difference between FMCV (mean) and FM (train) is smaller compared to the case of MD = 16 (which corresponds to the maximal FMCV-value) which means that less overfitting occurs when MD = 14.

The optimal MD value for DS13 is 10. The reasons for choosing MD = 10 rather than MD = 12 are similar to the reasons for selecting MD for DS05 (discussed above).

2.5.2. Support vector machine (SVM)

The class weights, cost parameter (C) and the RBF-kernel gamma parameter were optimized by grid search to attain maximal FM value.

In particular, we trained SVM-models with Class 0 to Class 1 ratio 1:1, 1:2, 1:3, 1:4, 1:9, 1:12, 1:14 for DS13 and 1:1, 1:1.5, 1:2, 1:3 and 1:4 for DS05. The gamma parameter was varied in the 0.05 - 0.30 range with steps of 0.05. The cost parameter (C) was set to 0.50, 1.00, 2.00, 5.00 or 10.00. The mean value of the 4-fold cross validation F-metric (FMCV (mean)) was used as a model performance metric during the hyperparameter optimization (**Table S4**). The FM metric values over the training set were considered to take in account overfitting (i.e. in case the overfitting F-metric over the training set is considerably larger than the mean of the cross-validation F-metric).

Table S4. SVM models training and optimization of the hyperparameters for DS05 and DS13 datasets – W0 and W1 are the weights of Class 0 and Class 1, RBF-kernel gamma parameter and cost parameter (C). FM (train) is F-measure over the training set, F1CV*i* (*i* = 1, 2, 3, 4) F-measure of the *i*-th cross-validation fold, FMCV (mean) is the mean of F1CV1, F1CV2, F1CV3 and F1CV4. Descriptors used: ['S1(CAS2-1) [eV]', 'CI-Coef**2(CAS2-1)', 'AMR', 'apol', 'nAromBond', 'nAtom', 'nHeavyAtom', 'nC', 'nN', 'nO', 'nBonds', 'nBondsS', 'nBondsS2', 'nBondsD', 'nBondsD2', 'nBondsM', 'C1SP2', 'C2SP2', 'C3SP2', 'C1SP3', 'C2SP3', 'Sv', 'Spe', 'Sp', 'Si', 'Mv', 'Mp', 'Mi', 'ECCEN', 'hmax', 'hmin', 'nRing', 'n5Ring', 'n6Ring', 'nFRing', 'nTRing', 'nT5Ring', 'nT6Ring', 'RotBtFrac', 'topoDiameter'].

**DS05, weights variation**									
W0	W1	gamma	C	FM (train)	F1CV1	F1CV2	F1CV3	F1CV4	FMCV (mean)
1	1	0.10	1.00	0.856	0.802	0.806	0.804	0.806	0.804
**1**	**1.5**	**0.10**	**1.00**	**0.870**	**0.819**	**0.818**	**0.820**	**0.818**	**0.819**
1	2	0.10	1.00	0.870	0.821	0.817	0.819	0.818	0.819
1	3	0.10	1.00	0.862	0.815	0.808	0.812	0.807	0.811
1	4	0.10	1.00	0.851	0.806	0.799	0.803	0.798	0.801
**DS05, weights 1:1.5, gamma parameter variation**									
W0	W1	gamma	C	FM (train)	F1CV1	F1CV2	F1CV3	F1CV4	FMCV (mean)
1	1.5	0.05	1.00	0.835	0.808	0.809	0.809	0.807	0.808
**1**	**1.5**	**0.10**	**1.00**	**0.870**	**0.819**	**0.818**	**0.820**	**0.818**	**0.819**
1	1.5	0.15	1.00	0.894	0.819	0.819	0.819	0.816	0.818
1	1.5	0.20	1.00	0.912	0.814	0.814	0.812	0.81	0.812
1	1.5	0.25	1.00	0.925	0.803	0.805	0.801	0.802	0.803
1	1.5	0.30	1.00	0.935	0.793	0.793	0.792	0.791	0.792

DS05, weights 1:1.5, gamma parameter 0.10, C variation									
W0	W1	gamma	C	FM (train)	F1CV1	F1CV2	F1CV3	F1CV4	FMCV (mean)
1	1.5	0.10	0.50	0.850	0.809	0.807	0.809	0.808	0.808
1	1.5	0.10	1.00	0.870	0.819	0.818	0.820	0.818	0.819
1	1.5	0.10	2.00	0.891	0.825	0.825	0.825	0.825	0.825
**1**	**1.5**	**0.10**	**5.00**	**0.916**	**0.830**	**0.829**	**0.828**	**0.828**	**0.829**
1	1.5	0.10	10.00	0.933	0.830	0.828	0.829	0.828	0.829
**DS05, weights 1:1.5, gamma parameter 0.15, C variation**									
W0	W1	gamma	C	FM (train)	F1CV1	F1CV2	F1CV3	F1CV4	FMCV (mean)
1	1.5	0.15	0.50	0.869	0.808	0.808	0.806	0.805	0.807
1	1.5	0.15	1.00	0.894	0.819	0.819	0.819	0.816	0.818
1	1.5	0.15	2.00	0.917	0.824	0.824	0.823	0.821	0.823
**1**	**1.5**	**0.15**	**5.00**	**0.943**	**0.825**	**0.824**	**0.824**	**0.822**	**0.824**
1	1.5	0.15	10.00	0.958	0.822	0.822	0.823	0.821	0.822
**DS13, weights variation**									
W0	W1	gamma	C	FM (train)	F1CV1	F1CV2	F1CV3	F1CV4	FMCV (mean)
1	1	0.10	1.00	0.854	0.763	0.744	0.740	0.749	0.749
**1**	**2**	**0.10**	**1.00**	**0.892**	**0.792**	**0.780**	**0.775**	**0.785**	**0.783**
1	3	0.10	1.00	0.891	0.790	0.781	0.780	0.782	0.783
1	4	0.10	1.00	0.887	0.787	0.779	0.778	0.779	0.781
1	9	0.10	1.00	0.866	0.768	0.759	0.756	0.759	0.760
1	12	0.10	1.00	0.860	0.762	0.763	0.750	0.752	0.757
1	14	0.10	1.00	0.856	0.759	0.752	0.747	0.748	0.752
1	24	0.10	1.00	0.849	0.751	0.742	0.741	0.743	0.849
**DS13, weights 1:2, gamma parameter variation**									
W0	W1	gamma	C	FM (train)	F1CV1	F1CV2	F1CV3	F1CV4	FMCV (mean)
1	2	0.01	1.00	0.777	0.768	0.756	0.758	0.766	0.762
**1**	**2**	**0.05**	**1.00**	**0.846**	**0.794**	**0.786**	**0.787**	**0.794**	**0.79**
1	2	0.10	1.00	0.892	0.792	0.780	0.775	0.785	0.783
1	2	0.15	1.00	0.921	0.775	0.759	0.751	0.760	0.761
1	2	0.20	1.00	0.940	0.747	0.735	0.724	0.735	0.735
1	2	0.25	1.00	0.952	0.712	0.702	0.694	0.703	0.703
1	2	0.30	1.00	0.960	0.683	0.673	0.662	0.671	0.672
**DS13, weights 1:3, gamma parameter variation**									
W0	W1	gamma	C	FM (train)	F1CV1	F1CV2	F1CV3	F1CV4	FMCV (mean)
1	3	0.01	1.00	0.774	0.763	0.754	0.759	0.765	0.760
**1**	**3**	**0.05**	**1.00**	**0.844**	**0.792**	**0.787**	**0.784**	**0.795**	**0.789**
1	3	0.10	1.00	0.891	0.790	0.781	0.780	0.782	0.783
1	3	0.15	1.00	0.923	0.775	0.763	0.757	0.762	0.764
1	3	0.20	1.00	0.942	0.748	0.739	0.726	0.738	0.738
1	3	0.25	1.00	0.954	0.717	0.711	0.697	0.707	0.708
1	3	0.30	1.00	0.962	0.686	0.675	0.665	0.673	0.675
**DS13, weights 1:2, gamma parameter 0.05, C variation**									
W0	W1	gamma	C	FM (train)	F1CV1	F1CV2	F1CV3	F1CV4	FMCV (mean)
1	2	0.05	0.50	0.825	0.787	0.776	0.777	0.784	0.781
1	2	0.05	1.00	0.846	0.794	0.786	0.787	0.794	0.790
**1**	**2**	**0.05**	**2.00**	**0.867**	**0.802**	**0.791**	**0.790**	**0.802**	**0.796**
1	2	0.05	5.00	0.893	0.806	0.792	0.793	0.806	0.799
1	2	0.05	10.00	0.914	0.803	0.788	0.796	0.800	0.797

NB: C = 2.00 was selected for DS13 since the difference between FMCV (mean) and FM (train) is smaller compared to the case when C = 5.00, which means that setting C to 2.00 yields a less overfitted model.

A reasonable choice of a derivative descriptor is also the energy difference $2E(T_1)$ - $E(S_1)$. However, our results demonstrate strong mutual correlation between $E(S_1)$ and $E(T_1)$, hence, between $2E(T_1)$ - $E(S_1)$ and the individual excitation energies. In other words, we can simultaneously build good classification models based on $E(S_1)$, $E(T_1)$ or $2E(T_1)$ - $E(S_1)$. To avoid overfitting, one of the three descriptors should be included in the model. Therefore, we trained also classification models based on $2E(T_1)$ - $E(S_1)$:1) SVM model (DS13, 1:2 ratio, gamma=0.05 and C=2) and 2) DT model (DS13 and MD=10). The PAM values for 1) is 74.85% and for 2) is 63.76%, i.e. the performance of the models with $2E(T_1)$ - $E(S_1)$ is slightly lower than or identical with the corresponding models with $E(S_1)$. Since the calculation of one excitation energy is cheaper than the computation of two, the model based on $E(S_1)$ is preferred and reported.

Only the best performing models are represented in the main manuscript. The SVM/DS13 model is implemented in an user-friendly web application (https://singletfission.chem.uni-sofia.bg/), which requires only *.mol file as an input to predict the SF propensity of a molecule based on its DRC.

3. BENCHMARK ON DRC CALCULATIONS

Usually, the DRC is calculated on top of geometries optimized with the DFT method. Therefore, we have performed a benchmark study in order to estimate the quality of the DRC obtained with the spin-projection UHF scheme on top of the PM6 geometries. The benchmark set contains 172 molecules with a non-zero DRC extracted from PubChem and included in our datasets. Our results show good agreement between the DRCs estimated using PM6 and B3LYP/6-31G** optimized geometries (**Figure S2**). In less than 5% the difference between DRC(B3LYP) and DRC(PM6) is larger than 0.1, which is owed mainly to the well-known fact that the semiempirical methods overstate planarity in conjugated systems, which does not fully apply to diradicaloids, where partial non-planarity may aid spin separation. The mean average error is 0.03 and in most of the cases

DRC(B3LYP) is larger than DRC(PM6). This confirms the reliability of the PUHF//PM6 computational protocol, which also has the advantage of much lower computational cost.

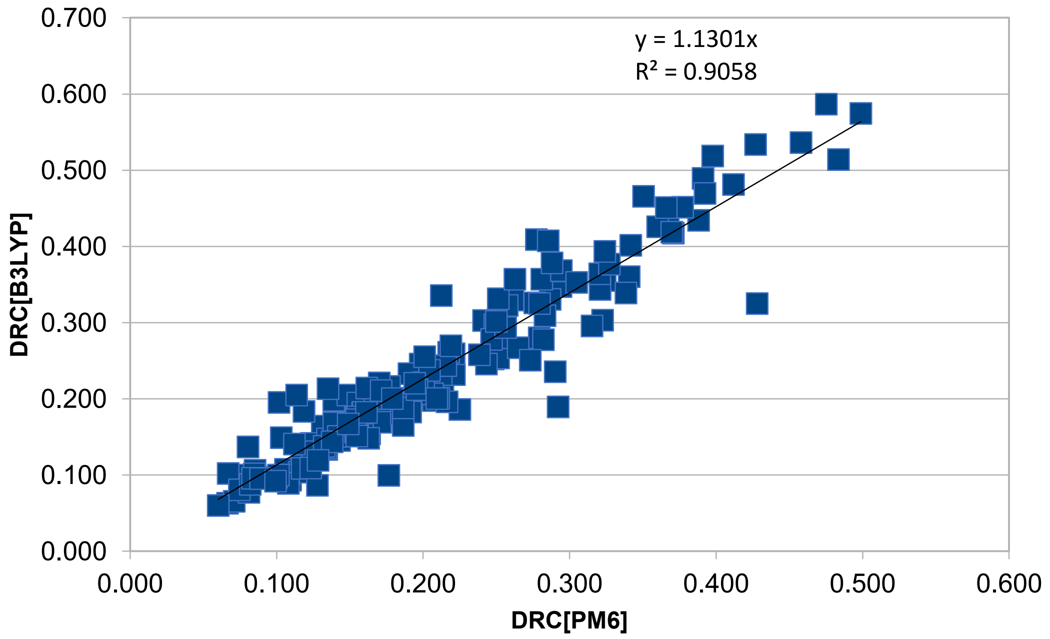

Figure S2. Correlation between PUHF/6-31G** results for the DRC, calculated with PM6 and with B3LYP/6-31G** optimized geometries. The benchmark contains 172 molecules with non-zero DRCs extracted from PubChem and included in our datasets.

The benchmark dataset with 172 compounds is freely available online: https://github.com/ml4sf/ml4sf-500kdataset (https://github.com/ml4sf/ml4sf-500kdataset/blob/master/geom-benchmark/GeomBenchmark.csv)

4. RESULTS FOR ACENES

The acenes (in particular anthracene) are used as reference molecules in our study. Therefore, for readers' convenience, some of their quantum chemical data are reported in **Table S5** and compared with experimental ones.

Table S5. DRC and $E(T_1)/E(S_1)$ [eV] – diradical character and excitation energies to the first excited triplet/singlet state obtained with INDO/S CASSCF(2,2) on top of PM6 geometries. SE/MSE – sign error and mean sign error with respect to reported in the literature excitation energies to the first excited triplet/singlet state $E(T_1)^{exp}/E(S_1)^{exp}$. The experimental data are taken from reference 48.

**PubChemPID**	**8418**	**7080**	**8671**	**MSE**
**Molecule**	**Anthracene**	**Tetracene**	**Pentacene**	
**$E(T_1)$**	2.31	1.91	1.66	
**$E(T_1)^{exp,47}$**	1.87	1.27	0.86	
**SE $E(T_1)$**	0.44	0.64	0.80	0.63
**$E(S_1)$**	4.07	3.48	3.09	
**$E(S_1)^{exp,47}$**	3.60	2.88	2.37	
**SE $E(S_1)$**	0.47	0.6	0.72	0.60
**DRC^{PM6}**	0.131	0.243	0.356	

The benchmark dataset for the impact of the PM6 geometry with 172 compounds is freely available online: https://github.com/ml4sf/ml4sf-500kdataset/blob/master/geom-benchmark/GeomBenchmark.csv.

5. K-MEANS CLUSTERING ANALYSIS

Among the supervised pattern recognition techniques used in machine learning, K-means clustering is one of the best documented. The K-means algorithm (where K is the number of the desired clusters) illustrates how variables are linked to the respective objects (or samples) by attributing a distance.[82-83] One cluster contains only objects with similar distances within the variables used in the chemical space, meaning that objects can be assigned to the same cluster because of one variable but with a great distance between each other, as the objects do not show similarities. The clusters separate the objects in a very coherent way, allowing derivation of

conclusions. The number of clusters into which the objects of interest should be partitioned must be determined *a priori*. The hypothesis for the predetermined number of clusters follows the expert opinion or specific reasons of the researcher (preliminary information, preliminary testing etc.). To achieve this objective, this statistical method partitions the objects of interest into patterns of similarity (clusters) whose number matches the preliminary hypothesis. The within-group distances (typically squared Euclidean distances) are minimized using an algorithm. It is necessary to rely on cluster centers (centroids) to find groups with similar spatial distributions in order to make comparisons. The K-means clustering was performed with STATISTICA. [84]

Lists with the members of Cluster 1 and Cluster 2 are also available online. The images of the compounds extracted from the PubChem database can be found here: https://github.com/ml4sf/ml4sf-500kdataset/tree/master/cluster-analysis

6. SUMMARY OF OPEN ACCESS DATA AND CODES

The final dataset is freely available online: https://github.com/ml4sf/ml4sf-500kdataset/blob/master/datasets/Dataset470K.csv.lrz.

The codes for the class-weighted support vector machines: https://github.com/ml4sf/ml4sf-500kdataset/tree/master/svm.

The code for the cost-sensitive decision tree: https://github.com/ml4sf/ml4sf-500kdataset/tree/master/dtree.

The benchmark dataset for the impact of the PM6 geometry with 172 compounds is freely available online: https://github.com/ml4sf/ml4sf-500kdataset/blob/master/geom-benchmark/GeomBenchmark.csv.

Lists with the members of Cluster 1 and Cluster 2 are also available online. The images of the compounds extracted from the PubChem database can be found here: https://github.com/ml4sf/ml4sf-500kdataset/tree/master/cluster-analysis. Note that the number of the structure images of the compounds extracted from PubChem is slightly smaller than the number of molecules in the corresponding clusters (csv files). This is probably due to changes/limitations of the PubChem web page/database.

User-friendly web application implementing the SVM/DS13 model: https://singletfission.chem.uni-sofia.bg/. We have created a web application which internally uses OPENMOPAC[78] and PaDEL[80] to calculate the descriptors based on an optimized molecular geometry. The descriptors are afterwards fed to the SVM/DS13 model to perform classification of a desired molecule based on its DRC.

7. DISCUSSION ON THE LIMITATIONS AND APPLICABILITY OF THE ML MODEL

In principle, different approximations for the theoretical estimation of the diradical character exist. In a qualitative aspect, diradical characters obtained by different computational schemes should mutually correlate. On the other hand, the machine learning is built upon statistics and works through qualitative trends. Therefore, in principle, the choice of the approximation for calculation of DRC should not sizably affect the quality of the classification model. In a quantitative aspect, however, one cannot expect a perfect match between diradical characters obtained by different computational schemes. Nevertheless, classification models can be derived on DRC obtained at different level of theory but in this case there is not strict definition of DRC ranges appropriate for singlet fission application yet. And since the Nakano's rule for singlet fission chromophores[6] is usually applied with the Yamaguchi's PUHF scheme[8], one should stick to this kind of approximation. In case another DRC approximation is more appropriate or the target application is different from singlet fission, the readers can use our database and the model but after its optimization.

Another important aspect is that using DRC as the only proxy to find SF chromophores has its limitations. A more accurate classification of a given material as a potential SF chromophore should be done after very precise and high-level multiconfigurational calculations of the excitation energies $E(S_1)$, $E(T_1)$ and $E(T_2)$. Although, even then, the experimental result can be different from the theoretical estimate because there are many factors, which affect the singlet fission propensity (such as solvent, crystal packing, etc.).

And finally, we tested our model on few known SF materials, which, because of their molecular size, were not part of the training and test sets: perylene diimide (CID: 66475), terrylene (CID: 123026), 1,3-diphenylisobenzofuran (CID: 21649) and zeaxanthin (CID 5280899). The SVM/DS13 model is able to correctly classify three out of four molecules as potential SF materials based on their DRC. The failed example is the zeaxanthin (CID 5280899). The misclassification of the zeaxanthin is expected since according to Figure 3 the model is trained on diradicaloids with high number of aromatic bonds and cycles (Cluster 1), as well as on diradicaloids with high number of double bonds and relatively high heteroatom/carbon ratio (Cluster 2).
