# Supplementary material for: Machine Learning-Based Screening for Potential Singlet Fission Chromophores: The Challenge of Imbalanced Data Sets
Source: J Phys Chem Lett. 2023 Nov 3;14(45):10103–12. doi: 10.1021/acs.jpclett.3c02365 (PMC10659028; doi:10.1021/acs.jpclett.3c02365)
Supplement: Supplementary file 2 — jz3c02365_si_003.pdf [file jz3c02365_si_003.pdf]

https://pubchem.ncbi.nlm.nih.gov/compound/9994827#section=2D-Structure
https://pubchem.ncbi.nlm.nih.gov/compound/9991699#section=2D-Structure
https://pubchem.ncbi.nlm.nih.gov/compound/9988871#section=2D-Structure
https://pubchem.ncbi.nlm.nih.gov/compound/9971235#section=2D-Structure
https://pubchem.ncbi.nlm.nih.gov/compound/9970666#section=2D-Structure
https://pubchem.ncbi.nlm.nih.gov/compound/9964272#section=2D-Structure
https://pubchem.ncbi.nlm.nih.gov/compound/9926867#section=2D-Structure
https://pubchem.ncbi.nlm.nih.gov/compound/9925923#section=2D-Structure
https://pubchem.ncbi.nlm.nih.gov/compound/9900267#section=2D-Structure
https://pubchem.ncbi.nlm.nih.gov/compound/9900266#section=2D-Structure
https://pubchem.ncbi.nlm.nih.gov/compound/98583#section=2D-Structure
https://pubchem.ncbi.nlm.nih.gov/compound/9839260#section=2D-Structure
https://pubchem.ncbi.nlm.nih.gov/compound/9819709#section=2D-Structure
https://pubchem.ncbi.nlm.nih.gov/compound/9816148#section=2D-Structure
https://pubchem.ncbi.nlm.nih.gov/compound/9796839#section=2D-Structure
https://pubchem.ncbi.nlm.nih.gov/compound/9794694#section=2D-Structure
https://pubchem.ncbi.nlm.nih.gov/compound/97548601#section=2D-Structure
https://pubchem.ncbi.nlm.nih.gov/compound/97548505#section=2D-Structure
https://pubchem.ncbi.nlm.nih.gov/compound/97548504#section=2D-Structure
https://pubchem.ncbi.nlm.nih.gov/compound/97037260#section=2D-Structure
https://pubchem.ncbi.nlm.nih.gov/compound/97037254#section=2D-Structure
https://pubchem.ncbi.nlm.nih.gov/compound/96896#section=2D-Structure
https://pubchem.ncbi.nlm.nih.gov/compound/96470099#section=2D-Structure
https://pubchem.ncbi.nlm.nih.gov/compound/95731#section=2D-Structure
https://pubchem.ncbi.nlm.nih.gov/compound/9543288#section=2D-Structure
https://pubchem.ncbi.nlm.nih.gov/compound/9543287#section=2D-Structure
https://pubchem.ncbi.nlm.nih.gov/compound/9543286#section=2D-Structure
https://pubchem.ncbi.nlm.nih.gov/compound/94719#section=2D-Structure
https://pubchem.ncbi.nlm.nih.gov/compound/94183#section=2D-Structure
https://pubchem.ncbi.nlm.nih.gov/compound/94030#section=2D-Structure
https://pubchem.ncbi.nlm.nih.gov/compound/92209351#section=2D-Structure
https://pubchem.ncbi.nlm.nih.gov/compound/92209350#section=2D-Structure
https://pubchem.ncbi.nlm.nih.gov/compound/92029999#section=2D-Structure
https://pubchem.ncbi.nlm.nih.gov/compound/92029388#section=2D-Structure
https://pubchem.ncbi.nlm.nih.gov/compound/91972158#section=2D-Structure
https://pubchem.ncbi.nlm.nih.gov/compound/91932460#section=2D-Structure
https://pubchem.ncbi.nlm.nih.gov/compound/91929102#section=2D-Structure
https://pubchem.ncbi.nlm.nih.gov/compound/91896215#section=2D-Structure
https://pubchem.ncbi.nlm.nih.gov/compound/9187#section=2D-Structure
https://pubchem.ncbi.nlm.nih.gov/compound/91866351#section=2D-Structure
https://pubchem.ncbi.nlm.nih.gov/compound/9185#section=2D-Structure
https://pubchem.ncbi.nlm.nih.gov/compound/91810447#section=2D-Structure
https://pubchem.ncbi.nlm.nih.gov/compound/91806324#section=2D-Structure
https://pubchem.ncbi.nlm.nih.gov/compound/91801289#section=2D-Structure
https://pubchem.ncbi.nlm.nih.gov/compound/91752336#section=2D-Structure
https://pubchem.ncbi.nlm.nih.gov/compound/91743232#section=2D-Structure
https://pubchem.ncbi.nlm.nih.gov/compound/91741436#section=2D-Structure
https://pubchem.ncbi.nlm.nih.gov/compound/91740555#section=2D-Structure
https://pubchem.ncbi.nlm.nih.gov/compound/91739151#section=2D-Structure
https://pubchem.ncbi.nlm.nih.gov/compound/91737329#section=2D-Structure
https://pubchem.ncbi.nlm.nih.gov/compound/91665934#section=2D-Structure
https://pubchem.ncbi.nlm.nih.gov/compound/91665829#section=2D-Structure
https://pubchem.ncbi.nlm.nih.gov/compound/91665824#section=2D-Structure
https://pubchem.ncbi.nlm.nih.gov/compound/91665823#section=2D-Structure
https://pubchem.ncbi.nlm.nih.gov/compound/91665802#section=2D-Structure

https://pubchem.ncbi.nlm.nih.gov/compound/91665748#section=2D-Structure
https://pubchem.ncbi.nlm.nih.gov/compound/91665532#section=2D-Structure
https://pubchem.ncbi.nlm.nih.gov/compound/91613307#section=2D-Structure
https://pubchem.ncbi.nlm.nih.gov/compound/91609781#section=2D-Structure
https://pubchem.ncbi.nlm.nih.gov/compound/91608578#section=2D-Structure
https://pubchem.ncbi.nlm.nih.gov/compound/91608046#section=2D-Structure
https://pubchem.ncbi.nlm.nih.gov/compound/91607446#section=2D-Structure
https://pubchem.ncbi.nlm.nih.gov/compound/91602851#section=2D-Structure
https://pubchem.ncbi.nlm.nih.gov/compound/91599021#section=2D-Structure
https://pubchem.ncbi.nlm.nih.gov/compound/91593402#section=2D-Structure
https://pubchem.ncbi.nlm.nih.gov/compound/91585522#section=2D-Structure
https://pubchem.ncbi.nlm.nih.gov/compound/91577878#section=2D-Structure
https://pubchem.ncbi.nlm.nih.gov/compound/91576971#section=2D-Structure
https://pubchem.ncbi.nlm.nih.gov/compound/91574874#section=2D-Structure
https://pubchem.ncbi.nlm.nih.gov/compound/91566318#section=2D-Structure
https://pubchem.ncbi.nlm.nih.gov/compound/91563348#section=2D-Structure
https://pubchem.ncbi.nlm.nih.gov/compound/91560342#section=2D-Structure
https://pubchem.ncbi.nlm.nih.gov/compound/91558442#section=2D-Structure
https://pubchem.ncbi.nlm.nih.gov/compound/91549809#section=2D-Structure
https://pubchem.ncbi.nlm.nih.gov/compound/91546525#section=2D-Structure
https://pubchem.ncbi.nlm.nih.gov/compound/91546480#section=2D-Structure
https://pubchem.ncbi.nlm.nih.gov/compound/91544548#section=2D-Structure
https://pubchem.ncbi.nlm.nih.gov/compound/91544429#section=2D-Structure
https://pubchem.ncbi.nlm.nih.gov/compound/91543322#section=2D-Structure
https://pubchem.ncbi.nlm.nih.gov/compound/91542422#section=2D-Structure
https://pubchem.ncbi.nlm.nih.gov/compound/91539863#section=2D-Structure
https://pubchem.ncbi.nlm.nih.gov/compound/91534543#section=2D-Structure
https://pubchem.ncbi.nlm.nih.gov/compound/91532432#section=2D-Structure
https://pubchem.ncbi.nlm.nih.gov/compound/91529527#section=2D-Structure
https://pubchem.ncbi.nlm.nih.gov/compound/91526277#section=2D-Structure
https://pubchem.ncbi.nlm.nih.gov/compound/91523634#section=2D-Structure
https://pubchem.ncbi.nlm.nih.gov/compound/91510780#section=2D-Structure
https://pubchem.ncbi.nlm.nih.gov/compound/91502775#section=2D-Structure
https://pubchem.ncbi.nlm.nih.gov/compound/91499278#section=2D-Structure
https://pubchem.ncbi.nlm.nih.gov/compound/91498865#section=2D-Structure
https://pubchem.ncbi.nlm.nih.gov/compound/91496189#section=2D-Structure
https://pubchem.ncbi.nlm.nih.gov/compound/91493919#section=2D-Structure
https://pubchem.ncbi.nlm.nih.gov/compound/91493802#section=2D-Structure
https://pubchem.ncbi.nlm.nih.gov/compound/91489497#section=2D-Structure
https://pubchem.ncbi.nlm.nih.gov/compound/91481822#section=2D-Structure
https://pubchem.ncbi.nlm.nih.gov/compound/91475595#section=2D-Structure
https://pubchem.ncbi.nlm.nih.gov/compound/91473170#section=2D-Structure
https://pubchem.ncbi.nlm.nih.gov/compound/91469840#section=2D-Structure
https://pubchem.ncbi.nlm.nih.gov/compound/91464532#section=2D-Structure
https://pubchem.ncbi.nlm.nih.gov/compound/9146#section=2D-Structure
https://pubchem.ncbi.nlm.nih.gov/compound/91458679#section=2D-Structure
https://pubchem.ncbi.nlm.nih.gov/compound/91457356#section=2D-Structure
https://pubchem.ncbi.nlm.nih.gov/compound/91455906#section=2D-Structure
https://pubchem.ncbi.nlm.nih.gov/compound/91455819#section=2D-Structure
https://pubchem.ncbi.nlm.nih.gov/compound/91453903#section=2D-Structure
https://pubchem.ncbi.nlm.nih.gov/compound/91451661#section=2D-Structure
https://pubchem.ncbi.nlm.nih.gov/compound/9145#section=2D-Structure
https://pubchem.ncbi.nlm.nih.gov/compound/91448077#section=2D-Structure
https://pubchem.ncbi.nlm.nih.gov/compound/91446904#section=2D-Structure
https://pubchem.ncbi.nlm.nih.gov/compound/91446731#section=2D-Structure

https://pubchem.ncbi.nlm.nih.gov/compound/91440579#section=2D-Structure
https://pubchem.ncbi.nlm.nih.gov/compound/91439019#section=2D-Structure
https://pubchem.ncbi.nlm.nih.gov/compound/91437520#section=2D-Structure
https://pubchem.ncbi.nlm.nih.gov/compound/91437253#section=2D-Structure
https://pubchem.ncbi.nlm.nih.gov/compound/91432177#section=2D-Structure
https://pubchem.ncbi.nlm.nih.gov/compound/91430125#section=2D-Structure
https://pubchem.ncbi.nlm.nih.gov/compound/91423791#section=2D-Structure
https://pubchem.ncbi.nlm.nih.gov/compound/91422639#section=2D-Structure
https://pubchem.ncbi.nlm.nih.gov/compound/91421538#section=2D-Structure
https://pubchem.ncbi.nlm.nih.gov/compound/9142#section=2D-Structure
https://pubchem.ncbi.nlm.nih.gov/compound/91417476#section=2D-Structure
https://pubchem.ncbi.nlm.nih.gov/compound/91411349#section=2D-Structure
https://pubchem.ncbi.nlm.nih.gov/compound/91408498#section=2D-Structure
https://pubchem.ncbi.nlm.nih.gov/compound/91403415#section=2D-Structure
https://pubchem.ncbi.nlm.nih.gov/compound/91402077#section=2D-Structure
https://pubchem.ncbi.nlm.nih.gov/compound/91402012#section=2D-Structure
https://pubchem.ncbi.nlm.nih.gov/compound/91391137#section=2D-Structure
https://pubchem.ncbi.nlm.nih.gov/compound/91378888#section=2D-Structure
https://pubchem.ncbi.nlm.nih.gov/compound/91373273#section=2D-Structure
https://pubchem.ncbi.nlm.nih.gov/compound/91372494#section=2D-Structure
https://pubchem.ncbi.nlm.nih.gov/compound/91370851#section=2D-Structure
https://pubchem.ncbi.nlm.nih.gov/compound/9137#section=2D-Structure
https://pubchem.ncbi.nlm.nih.gov/compound/91367147#section=2D-Structure
https://pubchem.ncbi.nlm.nih.gov/compound/91366482#section=2D-Structure
https://pubchem.ncbi.nlm.nih.gov/compound/91364652#section=2D-Structure
https://pubchem.ncbi.nlm.nih.gov/compound/91355582#section=2D-Structure
https://pubchem.ncbi.nlm.nih.gov/compound/91345773#section=2D-Structure
https://pubchem.ncbi.nlm.nih.gov/compound/91339273#section=2D-Structure
https://pubchem.ncbi.nlm.nih.gov/compound/91336900#section=2D-Structure
https://pubchem.ncbi.nlm.nih.gov/compound/91331446#section=2D-Structure
https://pubchem.ncbi.nlm.nih.gov/compound/91328198#section=2D-Structure
https://pubchem.ncbi.nlm.nih.gov/compound/91327543#section=2D-Structure
https://pubchem.ncbi.nlm.nih.gov/compound/91320239#section=2D-Structure
https://pubchem.ncbi.nlm.nih.gov/compound/91318007#section=2D-Structure
https://pubchem.ncbi.nlm.nih.gov/compound/91309906#section=2D-Structure
https://pubchem.ncbi.nlm.nih.gov/compound/91305938#section=2D-Structure
https://pubchem.ncbi.nlm.nih.gov/compound/91299882#section=2D-Structure
https://pubchem.ncbi.nlm.nih.gov/compound/91295493#section=2D-Structure
https://pubchem.ncbi.nlm.nih.gov/compound/91295325#section=2D-Structure
https://pubchem.ncbi.nlm.nih.gov/compound/91293631#section=2D-Structure
https://pubchem.ncbi.nlm.nih.gov/compound/91292344#section=2D-Structure
https://pubchem.ncbi.nlm.nih.gov/compound/91270259#section=2D-Structure
https://pubchem.ncbi.nlm.nih.gov/compound/91265829#section=2D-Structure
https://pubchem.ncbi.nlm.nih.gov/compound/9126#section=2D-Structure
https://pubchem.ncbi.nlm.nih.gov/compound/91256472#section=2D-Structure
https://pubchem.ncbi.nlm.nih.gov/compound/91243860#section=2D-Structure
https://pubchem.ncbi.nlm.nih.gov/compound/91242555#section=2D-Structure
https://pubchem.ncbi.nlm.nih.gov/compound/91241798#section=2D-Structure
https://pubchem.ncbi.nlm.nih.gov/compound/91235212#section=2D-Structure
https://pubchem.ncbi.nlm.nih.gov/compound/91234333#section=2D-Structure
https://pubchem.ncbi.nlm.nih.gov/compound/91229019#section=2D-Structure
https://pubchem.ncbi.nlm.nih.gov/compound/91221926#section=2D-Structure
https://pubchem.ncbi.nlm.nih.gov/compound/91218340#section=2D-Structure
https://pubchem.ncbi.nlm.nih.gov/compound/91210596#section=2D-Structure
https://pubchem.ncbi.nlm.nih.gov/compound/91210039#section=2D-Structure

https://pubchem.ncbi.nlm.nih.gov/compound/91206393#section=2D-Structure
https://pubchem.ncbi.nlm.nih.gov/compound/91204115#section=2D-Structure
https://pubchem.ncbi.nlm.nih.gov/compound/91197146#section=2D-Structure
https://pubchem.ncbi.nlm.nih.gov/compound/91192056#section=2D-Structure
https://pubchem.ncbi.nlm.nih.gov/compound/91190806#section=2D-Structure
https://pubchem.ncbi.nlm.nih.gov/compound/9119#section=2D-Structure
https://pubchem.ncbi.nlm.nih.gov/compound/91189796#section=2D-Structure
https://pubchem.ncbi.nlm.nih.gov/compound/91185500#section=2D-Structure
https://pubchem.ncbi.nlm.nih.gov/compound/91181692#section=2D-Structure
https://pubchem.ncbi.nlm.nih.gov/compound/91180993#section=2D-Structure
https://pubchem.ncbi.nlm.nih.gov/compound/9118#section=2D-Structure
https://pubchem.ncbi.nlm.nih.gov/compound/91179481#section=2D-Structure
https://pubchem.ncbi.nlm.nih.gov/compound/91177231#section=2D-Structure
https://pubchem.ncbi.nlm.nih.gov/compound/91175291#section=2D-Structure
https://pubchem.ncbi.nlm.nih.gov/compound/9117#section=2D-Structure
https://pubchem.ncbi.nlm.nih.gov/compound/91166244#section=2D-Structure
https://pubchem.ncbi.nlm.nih.gov/compound/91160201#section=2D-Structure
https://pubchem.ncbi.nlm.nih.gov/compound/91149172#section=2D-Structure
https://pubchem.ncbi.nlm.nih.gov/compound/91125887#section=2D-Structure
https://pubchem.ncbi.nlm.nih.gov/compound/91119220#section=2D-Structure
https://pubchem.ncbi.nlm.nih.gov/compound/91118761#section=2D-Structure
https://pubchem.ncbi.nlm.nih.gov/compound/91114371#section=2D-Structure
https://pubchem.ncbi.nlm.nih.gov/compound/91110781#section=2D-Structure
https://pubchem.ncbi.nlm.nih.gov/compound/9111#section=2D-Structure
https://pubchem.ncbi.nlm.nih.gov/compound/91108965#section=2D-Structure
https://pubchem.ncbi.nlm.nih.gov/compound/91106553#section=2D-Structure
https://pubchem.ncbi.nlm.nih.gov/compound/911047#section=2D-Structure
https://pubchem.ncbi.nlm.nih.gov/compound/9110#section=2D-Structure
https://pubchem.ncbi.nlm.nih.gov/compound/91095009#section=2D-Structure
https://pubchem.ncbi.nlm.nih.gov/compound/91092204#section=2D-Structure
https://pubchem.ncbi.nlm.nih.gov/compound/91090194#section=2D-Structure
https://pubchem.ncbi.nlm.nih.gov/compound/9109#section=2D-Structure
https://pubchem.ncbi.nlm.nih.gov/compound/9108#section=2D-Structure
https://pubchem.ncbi.nlm.nih.gov/compound/91074990#section=2D-Structure
https://pubchem.ncbi.nlm.nih.gov/compound/91071133#section=2D-Structure
https://pubchem.ncbi.nlm.nih.gov/compound/9107#section=2D-Structure
https://pubchem.ncbi.nlm.nih.gov/compound/9106#section=2D-Structure
https://pubchem.ncbi.nlm.nih.gov/compound/91059290#section=2D-Structure
https://pubchem.ncbi.nlm.nih.gov/compound/91057853#section=2D-Structure
https://pubchem.ncbi.nlm.nih.gov/compound/91045167#section=2D-Structure
https://pubchem.ncbi.nlm.nih.gov/compound/91042007#section=2D-Structure
https://pubchem.ncbi.nlm.nih.gov/compound/91040857#section=2D-Structure
https://pubchem.ncbi.nlm.nih.gov/compound/91034232#section=2D-Structure
https://pubchem.ncbi.nlm.nih.gov/compound/91032165#section=2D-Structure
https://pubchem.ncbi.nlm.nih.gov/compound/9103#section=2D-Structure
https://pubchem.ncbi.nlm.nih.gov/compound/91021005#section=2D-Structure
https://pubchem.ncbi.nlm.nih.gov/compound/91020799#section=2D-Structure
https://pubchem.ncbi.nlm.nih.gov/compound/91019899#section=2D-Structure
https://pubchem.ncbi.nlm.nih.gov/compound/91011142#section=2D-Structure
https://pubchem.ncbi.nlm.nih.gov/compound/91009654#section=2D-Structure
https://pubchem.ncbi.nlm.nih.gov/compound/91004047#section=2D-Structure
https://pubchem.ncbi.nlm.nih.gov/compound/90994966#section=2D-Structure
https://pubchem.ncbi.nlm.nih.gov/compound/90990921#section=2D-Structure
https://pubchem.ncbi.nlm.nih.gov/compound/90980274#section=2D-Structure
https://pubchem.ncbi.nlm.nih.gov/compound/90978136#section=2D-Structure

https://pubchem.ncbi.nlm.nih.gov/compound/90978098#section=2D-Structure
https://pubchem.ncbi.nlm.nih.gov/compound/90971488#section=2D-Structure
https://pubchem.ncbi.nlm.nih.gov/compound/90969900#section=2D-Structure
https://pubchem.ncbi.nlm.nih.gov/compound/90963081#section=2D-Structure
https://pubchem.ncbi.nlm.nih.gov/compound/90962670#section=2D-Structure
https://pubchem.ncbi.nlm.nih.gov/compound/90962095#section=2D-Structure
https://pubchem.ncbi.nlm.nih.gov/compound/90957775#section=2D-Structure
https://pubchem.ncbi.nlm.nih.gov/compound/90951169#section=2D-Structure
https://pubchem.ncbi.nlm.nih.gov/compound/90944304#section=2D-Structure
https://pubchem.ncbi.nlm.nih.gov/compound/90935015#section=2D-Structure
https://pubchem.ncbi.nlm.nih.gov/compound/90934201#section=2D-Structure
https://pubchem.ncbi.nlm.nih.gov/compound/90925628#section=2D-Structure
https://pubchem.ncbi.nlm.nih.gov/compound/90922040#section=2D-Structure
https://pubchem.ncbi.nlm.nih.gov/compound/90920311#section=2D-Structure
https://pubchem.ncbi.nlm.nih.gov/compound/90920197#section=2D-Structure
https://pubchem.ncbi.nlm.nih.gov/compound/90914236#section=2D-Structure
https://pubchem.ncbi.nlm.nih.gov/compound/90908635#section=2D-Structure
https://pubchem.ncbi.nlm.nih.gov/compound/90904436#section=2D-Structure
https://pubchem.ncbi.nlm.nih.gov/compound/90901131#section=2D-Structure
https://pubchem.ncbi.nlm.nih.gov/compound/90888840#section=2D-Structure
https://pubchem.ncbi.nlm.nih.gov/compound/90885381#section=2D-Structure
https://pubchem.ncbi.nlm.nih.gov/compound/90881016#section=2D-Structure
https://pubchem.ncbi.nlm.nih.gov/compound/90872293#section=2D-Structure
https://pubchem.ncbi.nlm.nih.gov/compound/90871236#section=2D-Structure
https://pubchem.ncbi.nlm.nih.gov/compound/90867169#section=2D-Structure
https://pubchem.ncbi.nlm.nih.gov/compound/90866434#section=2D-Structure
https://pubchem.ncbi.nlm.nih.gov/compound/90861481#section=2D-Structure
https://pubchem.ncbi.nlm.nih.gov/compound/90858067#section=2D-Structure
https://pubchem.ncbi.nlm.nih.gov/compound/90856317#section=2D-Structure
https://pubchem.ncbi.nlm.nih.gov/compound/90843427#section=2D-Structure
https://pubchem.ncbi.nlm.nih.gov/compound/90818880#section=2D-Structure
https://pubchem.ncbi.nlm.nih.gov/compound/90804417#section=2D-Structure
https://pubchem.ncbi.nlm.nih.gov/compound/90796466#section=2D-Structure
https://pubchem.ncbi.nlm.nih.gov/compound/90791710#section=2D-Structure
https://pubchem.ncbi.nlm.nih.gov/compound/90791377#section=2D-Structure
https://pubchem.ncbi.nlm.nih.gov/compound/90781732#section=2D-Structure
https://pubchem.ncbi.nlm.nih.gov/compound/90771596#section=2D-Structure
https://pubchem.ncbi.nlm.nih.gov/compound/90765734#section=2D-Structure
https://pubchem.ncbi.nlm.nih.gov/compound/90762111#section=2D-Structure
https://pubchem.ncbi.nlm.nih.gov/compound/90761597#section=2D-Structure
https://pubchem.ncbi.nlm.nih.gov/compound/90761044#section=2D-Structure
https://pubchem.ncbi.nlm.nih.gov/compound/90760410#section=2D-Structure
https://pubchem.ncbi.nlm.nih.gov/compound/90759541#section=2D-Structure
https://pubchem.ncbi.nlm.nih.gov/compound/90758934#section=2D-Structure
https://pubchem.ncbi.nlm.nih.gov/compound/90758230#section=2D-Structure
https://pubchem.ncbi.nlm.nih.gov/compound/90757133#section=2D-Structure
https://pubchem.ncbi.nlm.nih.gov/compound/90755956#section=2D-Structure
https://pubchem.ncbi.nlm.nih.gov/compound/90755751#section=2D-Structure
https://pubchem.ncbi.nlm.nih.gov/compound/90755104#section=2D-Structure
https://pubchem.ncbi.nlm.nih.gov/compound/90752121#section=2D-Structure
https://pubchem.ncbi.nlm.nih.gov/compound/90751907#section=2D-Structure
https://pubchem.ncbi.nlm.nih.gov/compound/90749770#section=2D-Structure
https://pubchem.ncbi.nlm.nih.gov/compound/90749464#section=2D-Structure
https://pubchem.ncbi.nlm.nih.gov/compound/90745817#section=2D-Structure
https://pubchem.ncbi.nlm.nih.gov/compound/90744591#section=2D-Structure

https://pubchem.ncbi.nlm.nih.gov/compound/90742468#section=2D-Structure
https://pubchem.ncbi.nlm.nih.gov/compound/90741429#section=2D-Structure
https://pubchem.ncbi.nlm.nih.gov/compound/90737873#section=2D-Structure
https://pubchem.ncbi.nlm.nih.gov/compound/90736145#section=2D-Structure
https://pubchem.ncbi.nlm.nih.gov/compound/90735338#section=2D-Structure
https://pubchem.ncbi.nlm.nih.gov/compound/90733360#section=2D-Structure
https://pubchem.ncbi.nlm.nih.gov/compound/90719000#section=2D-Structure
https://pubchem.ncbi.nlm.nih.gov/compound/90710923#section=2D-Structure
https://pubchem.ncbi.nlm.nih.gov/compound/90707620#section=2D-Structure
https://pubchem.ncbi.nlm.nih.gov/compound/90704239#section=2D-Structure
https://pubchem.ncbi.nlm.nih.gov/compound/90703815#section=2D-Structure
https://pubchem.ncbi.nlm.nih.gov/compound/90697554#section=2D-Structure
https://pubchem.ncbi.nlm.nih.gov/compound/90694609#section=2D-Structure
https://pubchem.ncbi.nlm.nih.gov/compound/90693137#section=2D-Structure
https://pubchem.ncbi.nlm.nih.gov/compound/90692396#section=2D-Structure
https://pubchem.ncbi.nlm.nih.gov/compound/90692022#section=2D-Structure
https://pubchem.ncbi.nlm.nih.gov/compound/90691198#section=2D-Structure
https://pubchem.ncbi.nlm.nih.gov/compound/90686724#section=2D-Structure
https://pubchem.ncbi.nlm.nih.gov/compound/90685512#section=2D-Structure
https://pubchem.ncbi.nlm.nih.gov/compound/90658323#section=2D-Structure
https://pubchem.ncbi.nlm.nih.gov/compound/90480785#section=2D-Structure
https://pubchem.ncbi.nlm.nih.gov/compound/90471722#section=2D-Structure
https://pubchem.ncbi.nlm.nih.gov/compound/90470165#section=2D-Structure
https://pubchem.ncbi.nlm.nih.gov/compound/90456485#section=2D-Structure
https://pubchem.ncbi.nlm.nih.gov/compound/90443782#section=2D-Structure
https://pubchem.ncbi.nlm.nih.gov/compound/90443781#section=2D-Structure
https://pubchem.ncbi.nlm.nih.gov/compound/90439950#section=2D-Structure
https://pubchem.ncbi.nlm.nih.gov/compound/90439931#section=2D-Structure
https://pubchem.ncbi.nlm.nih.gov/compound/90439926#section=2D-Structure
https://pubchem.ncbi.nlm.nih.gov/compound/90439925#section=2D-Structure
https://pubchem.ncbi.nlm.nih.gov/compound/90439924#section=2D-Structure
https://pubchem.ncbi.nlm.nih.gov/compound/90439922#section=2D-Structure
https://pubchem.ncbi.nlm.nih.gov/compound/90435186#section=2D-Structure
https://pubchem.ncbi.nlm.nih.gov/compound/90401434#section=2D-Structure
https://pubchem.ncbi.nlm.nih.gov/compound/90343846#section=2D-Structure
https://pubchem.ncbi.nlm.nih.gov/compound/90305852#section=2D-Structure
https://pubchem.ncbi.nlm.nih.gov/compound/90303571#section=2D-Structure
https://pubchem.ncbi.nlm.nih.gov/compound/90303514#section=2D-Structure
https://pubchem.ncbi.nlm.nih.gov/compound/90294469#section=2D-Structure
https://pubchem.ncbi.nlm.nih.gov/compound/90278208#section=2D-Structure
https://pubchem.ncbi.nlm.nih.gov/compound/90260149#section=2D-Structure
https://pubchem.ncbi.nlm.nih.gov/compound/90260148#section=2D-Structure
https://pubchem.ncbi.nlm.nih.gov/compound/90260093#section=2D-Structure
https://pubchem.ncbi.nlm.nih.gov/compound/90249793#section=2D-Structure
https://pubchem.ncbi.nlm.nih.gov/compound/90249787#section=2D-Structure
https://pubchem.ncbi.nlm.nih.gov/compound/90241784#section=2D-Structure
https://pubchem.ncbi.nlm.nih.gov/compound/90240076#section=2D-Structure
https://pubchem.ncbi.nlm.nih.gov/compound/90239319#section=2D-Structure
https://pubchem.ncbi.nlm.nih.gov/compound/90238352#section=2D-Structure
https://pubchem.ncbi.nlm.nih.gov/compound/90238262#section=2D-Structure
https://pubchem.ncbi.nlm.nih.gov/compound/90220594#section=2D-Structure
https://pubchem.ncbi.nlm.nih.gov/compound/90219642#section=2D-Structure
https://pubchem.ncbi.nlm.nih.gov/compound/90206884#section=2D-Structure
https://pubchem.ncbi.nlm.nih.gov/compound/90193203#section=2D-Structure
https://pubchem.ncbi.nlm.nih.gov/compound/90189876#section=2D-Structure

https://pubchem.ncbi.nlm.nih.gov/compound/90181522#section=2D-Structure
https://pubchem.ncbi.nlm.nih.gov/compound/90164420#section=2D-Structure
https://pubchem.ncbi.nlm.nih.gov/compound/90155766#section=2D-Structure
https://pubchem.ncbi.nlm.nih.gov/compound/90153516#section=2D-Structure
https://pubchem.ncbi.nlm.nih.gov/compound/90147210#section=2D-Structure
https://pubchem.ncbi.nlm.nih.gov/compound/90147208#section=2D-Structure
https://pubchem.ncbi.nlm.nih.gov/compound/90147206#section=2D-Structure
https://pubchem.ncbi.nlm.nih.gov/compound/90146104#section=2D-Structure
https://pubchem.ncbi.nlm.nih.gov/compound/90132430#section=2D-Structure
https://pubchem.ncbi.nlm.nih.gov/compound/90123963#section=2D-Structure
https://pubchem.ncbi.nlm.nih.gov/compound/90123883#section=2D-Structure
https://pubchem.ncbi.nlm.nih.gov/compound/90122341#section=2D-Structure
https://pubchem.ncbi.nlm.nih.gov/compound/90120855#section=2D-Structure
https://pubchem.ncbi.nlm.nih.gov/compound/90120854#section=2D-Structure
https://pubchem.ncbi.nlm.nih.gov/compound/90120853#section=2D-Structure
https://pubchem.ncbi.nlm.nih.gov/compound/90106504#section=2D-Structure
https://pubchem.ncbi.nlm.nih.gov/compound/90092693#section=2D-Structure
https://pubchem.ncbi.nlm.nih.gov/compound/90084879#section=2D-Structure
https://pubchem.ncbi.nlm.nih.gov/compound/90084877#section=2D-Structure
https://pubchem.ncbi.nlm.nih.gov/compound/90074035#section=2D-Structure
https://pubchem.ncbi.nlm.nih.gov/compound/90019323#section=2D-Structure
https://pubchem.ncbi.nlm.nih.gov/compound/90003497#section=2D-Structure
https://pubchem.ncbi.nlm.nih.gov/compound/89973#section=2D-Structure
https://pubchem.ncbi.nlm.nih.gov/compound/89971680#section=2D-Structure
https://pubchem.ncbi.nlm.nih.gov/compound/89956072#section=2D-Structure
https://pubchem.ncbi.nlm.nih.gov/compound/89949767#section=2D-Structure
https://pubchem.ncbi.nlm.nih.gov/compound/89948867#section=2D-Structure
https://pubchem.ncbi.nlm.nih.gov/compound/89948865#section=2D-Structure
https://pubchem.ncbi.nlm.nih.gov/compound/89948840#section=2D-Structure
https://pubchem.ncbi.nlm.nih.gov/compound/89948816#section=2D-Structure
https://pubchem.ncbi.nlm.nih.gov/compound/89948793#section=2D-Structure
https://pubchem.ncbi.nlm.nih.gov/compound/89948787#section=2D-Structure
https://pubchem.ncbi.nlm.nih.gov/compound/89948785#section=2D-Structure
https://pubchem.ncbi.nlm.nih.gov/compound/89948783#section=2D-Structure
https://pubchem.ncbi.nlm.nih.gov/compound/89948779#section=2D-Structure
https://pubchem.ncbi.nlm.nih.gov/compound/89939747#section=2D-Structure
https://pubchem.ncbi.nlm.nih.gov/compound/89939746#section=2D-Structure
https://pubchem.ncbi.nlm.nih.gov/compound/89924722#section=2D-Structure
https://pubchem.ncbi.nlm.nih.gov/compound/89924594#section=2D-Structure
https://pubchem.ncbi.nlm.nih.gov/compound/89924439#section=2D-Structure
https://pubchem.ncbi.nlm.nih.gov/compound/89914379#section=2D-Structure
https://pubchem.ncbi.nlm.nih.gov/compound/89914037#section=2D-Structure
https://pubchem.ncbi.nlm.nih.gov/compound/89891835#section=2D-Structure
https://pubchem.ncbi.nlm.nih.gov/compound/89878396#section=2D-Structure
https://pubchem.ncbi.nlm.nih.gov/compound/89876013#section=2D-Structure
https://pubchem.ncbi.nlm.nih.gov/compound/89871485#section=2D-Structure
https://pubchem.ncbi.nlm.nih.gov/compound/89871484#section=2D-Structure
https://pubchem.ncbi.nlm.nih.gov/compound/89870071#section=2D-Structure
https://pubchem.ncbi.nlm.nih.gov/compound/89827614#section=2D-Structure
https://pubchem.ncbi.nlm.nih.gov/compound/89827606#section=2D-Structure
https://pubchem.ncbi.nlm.nih.gov/compound/89822224#section=2D-Structure
https://pubchem.ncbi.nlm.nih.gov/compound/89815105#section=2D-Structure
https://pubchem.ncbi.nlm.nih.gov/compound/89815104#section=2D-Structure
https://pubchem.ncbi.nlm.nih.gov/compound/89808899#section=2D-Structure
https://pubchem.ncbi.nlm.nih.gov/compound/89805065#section=2D-Structure

https://pubchem.ncbi.nlm.nih.gov/compound/89798109#section=2D-Structure
https://pubchem.ncbi.nlm.nih.gov/compound/89784851#section=2D-Structure
https://pubchem.ncbi.nlm.nih.gov/compound/89781084#section=2D-Structure
https://pubchem.ncbi.nlm.nih.gov/compound/89780144#section=2D-Structure
https://pubchem.ncbi.nlm.nih.gov/compound/89761618#section=2D-Structure
https://pubchem.ncbi.nlm.nih.gov/compound/89761616#section=2D-Structure
https://pubchem.ncbi.nlm.nih.gov/compound/89761505#section=2D-Structure
https://pubchem.ncbi.nlm.nih.gov/compound/89760886#section=2D-Structure
https://pubchem.ncbi.nlm.nih.gov/compound/89706643#section=2D-Structure
https://pubchem.ncbi.nlm.nih.gov/compound/89706625#section=2D-Structure
https://pubchem.ncbi.nlm.nih.gov/compound/89704506#section=2D-Structure
https://pubchem.ncbi.nlm.nih.gov/compound/89701021#section=2D-Structure
https://pubchem.ncbi.nlm.nih.gov/compound/89700986#section=2D-Structure
https://pubchem.ncbi.nlm.nih.gov/compound/89700599#section=2D-Structure
https://pubchem.ncbi.nlm.nih.gov/compound/89697083#section=2D-Structure
https://pubchem.ncbi.nlm.nih.gov/compound/89697082#section=2D-Structure
https://pubchem.ncbi.nlm.nih.gov/compound/89690026#section=2D-Structure
https://pubchem.ncbi.nlm.nih.gov/compound/89661983#section=2D-Structure
https://pubchem.ncbi.nlm.nih.gov/compound/89661603#section=2D-Structure
https://pubchem.ncbi.nlm.nih.gov/compound/89657431#section=2D-Structure
https://pubchem.ncbi.nlm.nih.gov/compound/89656511#section=2D-Structure
https://pubchem.ncbi.nlm.nih.gov/compound/89652264#section=2D-Structure
https://pubchem.ncbi.nlm.nih.gov/compound/89649113#section=2D-Structure
https://pubchem.ncbi.nlm.nih.gov/compound/89649#section=2D-Structure
https://pubchem.ncbi.nlm.nih.gov/compound/89647905#section=2D-Structure
https://pubchem.ncbi.nlm.nih.gov/compound/89645946#section=2D-Structure
https://pubchem.ncbi.nlm.nih.gov/compound/89644672#section=2D-Structure
https://pubchem.ncbi.nlm.nih.gov/compound/89643381#section=2D-Structure
https://pubchem.ncbi.nlm.nih.gov/compound/89643359#section=2D-Structure
https://pubchem.ncbi.nlm.nih.gov/compound/89639279#section=2D-Structure
https://pubchem.ncbi.nlm.nih.gov/compound/89590324#section=2D-Structure
https://pubchem.ncbi.nlm.nih.gov/compound/89590317#section=2D-Structure
https://pubchem.ncbi.nlm.nih.gov/compound/89590316#section=2D-Structure
https://pubchem.ncbi.nlm.nih.gov/compound/89577823#section=2D-Structure
https://pubchem.ncbi.nlm.nih.gov/compound/89572629#section=2D-Structure
https://pubchem.ncbi.nlm.nih.gov/compound/89571947#section=2D-Structure
https://pubchem.ncbi.nlm.nih.gov/compound/89570176#section=2D-Structure
https://pubchem.ncbi.nlm.nih.gov/compound/89570175#section=2D-Structure
https://pubchem.ncbi.nlm.nih.gov/compound/89567822#section=2D-Structure
https://pubchem.ncbi.nlm.nih.gov/compound/89546169#section=2D-Structure
https://pubchem.ncbi.nlm.nih.gov/compound/89519339#section=2D-Structure
https://pubchem.ncbi.nlm.nih.gov/compound/89513198#section=2D-Structure
https://pubchem.ncbi.nlm.nih.gov/compound/89507556#section=2D-Structure
https://pubchem.ncbi.nlm.nih.gov/compound/89497489#section=2D-Structure
https://pubchem.ncbi.nlm.nih.gov/compound/89497434#section=2D-Structure
https://pubchem.ncbi.nlm.nih.gov/compound/89497433#section=2D-Structure
https://pubchem.ncbi.nlm.nih.gov/compound/89494584#section=2D-Structure
https://pubchem.ncbi.nlm.nih.gov/compound/89494583#section=2D-Structure
https://pubchem.ncbi.nlm.nih.gov/compound/89483163#section=2D-Structure
https://pubchem.ncbi.nlm.nih.gov/compound/89469021#section=2D-Structure
https://pubchem.ncbi.nlm.nih.gov/compound/89428281#section=2D-Structure
https://pubchem.ncbi.nlm.nih.gov/compound/89422377#section=2D-Structure
https://pubchem.ncbi.nlm.nih.gov/compound/89419599#section=2D-Structure
https://pubchem.ncbi.nlm.nih.gov/compound/89411447#section=2D-Structure
https://pubchem.ncbi.nlm.nih.gov/compound/89402939#section=2D-Structure

https://pubchem.ncbi.nlm.nih.gov/compound/89402932#section=2D-Structure
https://pubchem.ncbi.nlm.nih.gov/compound/89402789#section=2D-Structure
https://pubchem.ncbi.nlm.nih.gov/compound/89394593#section=2D-Structure
https://pubchem.ncbi.nlm.nih.gov/compound/89377119#section=2D-Structure
https://pubchem.ncbi.nlm.nih.gov/compound/89376765#section=2D-Structure
https://pubchem.ncbi.nlm.nih.gov/compound/89374805#section=2D-Structure
https://pubchem.ncbi.nlm.nih.gov/compound/89374804#section=2D-Structure
https://pubchem.ncbi.nlm.nih.gov/compound/89374802#section=2D-Structure
https://pubchem.ncbi.nlm.nih.gov/compound/89363921#section=2D-Structure
https://pubchem.ncbi.nlm.nih.gov/compound/89354882#section=2D-Structure
https://pubchem.ncbi.nlm.nih.gov/compound/89346727#section=2D-Structure
https://pubchem.ncbi.nlm.nih.gov/compound/89343685#section=2D-Structure
https://pubchem.ncbi.nlm.nih.gov/compound/89343386#section=2D-Structure
https://pubchem.ncbi.nlm.nih.gov/compound/89321694#section=2D-Structure
https://pubchem.ncbi.nlm.nih.gov/compound/89310858#section=2D-Structure
https://pubchem.ncbi.nlm.nih.gov/compound/89309502#section=2D-Structure
https://pubchem.ncbi.nlm.nih.gov/compound/89309487#section=2D-Structure
https://pubchem.ncbi.nlm.nih.gov/compound/89309480#section=2D-Structure
https://pubchem.ncbi.nlm.nih.gov/compound/89301071#section=2D-Structure
https://pubchem.ncbi.nlm.nih.gov/compound/89289252#section=2D-Structure
https://pubchem.ncbi.nlm.nih.gov/compound/89287817#section=2D-Structure
https://pubchem.ncbi.nlm.nih.gov/compound/89264071#section=2D-Structure
https://pubchem.ncbi.nlm.nih.gov/compound/89256751#section=2D-Structure
https://pubchem.ncbi.nlm.nih.gov/compound/89242573#section=2D-Structure
https://pubchem.ncbi.nlm.nih.gov/compound/89242568#section=2D-Structure
https://pubchem.ncbi.nlm.nih.gov/compound/89223279#section=2D-Structure
https://pubchem.ncbi.nlm.nih.gov/compound/89207007#section=2D-Structure
https://pubchem.ncbi.nlm.nih.gov/compound/89177416#section=2D-Structure
https://pubchem.ncbi.nlm.nih.gov/compound/89177014#section=2D-Structure
https://pubchem.ncbi.nlm.nih.gov/compound/89172530#section=2D-Structure
https://pubchem.ncbi.nlm.nih.gov/compound/89161802#section=2D-Structure
https://pubchem.ncbi.nlm.nih.gov/compound/89157421#section=2D-Structure
https://pubchem.ncbi.nlm.nih.gov/compound/89152522#section=2D-Structure
https://pubchem.ncbi.nlm.nih.gov/compound/89152126#section=2D-Structure
https://pubchem.ncbi.nlm.nih.gov/compound/89150152#section=2D-Structure
https://pubchem.ncbi.nlm.nih.gov/compound/89150033#section=2D-Structure
https://pubchem.ncbi.nlm.nih.gov/compound/89150027#section=2D-Structure
https://pubchem.ncbi.nlm.nih.gov/compound/89143763#section=2D-Structure
https://pubchem.ncbi.nlm.nih.gov/compound/89136973#section=2D-Structure
https://pubchem.ncbi.nlm.nih.gov/compound/89136752#section=2D-Structure
https://pubchem.ncbi.nlm.nih.gov/compound/89120023#section=2D-Structure
https://pubchem.ncbi.nlm.nih.gov/compound/89117633#section=2D-Structure
https://pubchem.ncbi.nlm.nih.gov/compound/89117632#section=2D-Structure
https://pubchem.ncbi.nlm.nih.gov/compound/89117628#section=2D-Structure
https://pubchem.ncbi.nlm.nih.gov/compound/89117561#section=2D-Structure
https://pubchem.ncbi.nlm.nih.gov/compound/89117288#section=2D-Structure
https://pubchem.ncbi.nlm.nih.gov/compound/89117283#section=2D-Structure
https://pubchem.ncbi.nlm.nih.gov/compound/89117278#section=2D-Structure
https://pubchem.ncbi.nlm.nih.gov/compound/89117274#section=2D-Structure
https://pubchem.ncbi.nlm.nih.gov/compound/89117268#section=2D-Structure
https://pubchem.ncbi.nlm.nih.gov/compound/89117264#section=2D-Structure
https://pubchem.ncbi.nlm.nih.gov/compound/89117258#section=2D-Structure
https://pubchem.ncbi.nlm.nih.gov/compound/89117250#section=2D-Structure
https://pubchem.ncbi.nlm.nih.gov/compound/89117239#section=2D-Structure
https://pubchem.ncbi.nlm.nih.gov/compound/89117227#section=2D-Structure

https://pubchem.ncbi.nlm.nih.gov/compound/89117224#section=2D-Structure
https://pubchem.ncbi.nlm.nih.gov/compound/89117214#section=2D-Structure
https://pubchem.ncbi.nlm.nih.gov/compound/89117198#section=2D-Structure
https://pubchem.ncbi.nlm.nih.gov/compound/89117195#section=2D-Structure
https://pubchem.ncbi.nlm.nih.gov/compound/89117187#section=2D-Structure
https://pubchem.ncbi.nlm.nih.gov/compound/89116930#section=2D-Structure
https://pubchem.ncbi.nlm.nih.gov/compound/89104908#section=2D-Structure
https://pubchem.ncbi.nlm.nih.gov/compound/89086145#section=2D-Structure
https://pubchem.ncbi.nlm.nih.gov/compound/89070064#section=2D-Structure
https://pubchem.ncbi.nlm.nih.gov/compound/89059092#section=2D-Structure
https://pubchem.ncbi.nlm.nih.gov/compound/89059090#section=2D-Structure
https://pubchem.ncbi.nlm.nih.gov/compound/89056965#section=2D-Structure
https://pubchem.ncbi.nlm.nih.gov/compound/89054818#section=2D-Structure
https://pubchem.ncbi.nlm.nih.gov/compound/89053130#section=2D-Structure
https://pubchem.ncbi.nlm.nih.gov/compound/89051047#section=2D-Structure
https://pubchem.ncbi.nlm.nih.gov/compound/89035605#section=2D-Structure
https://pubchem.ncbi.nlm.nih.gov/compound/89026481#section=2D-Structure
https://pubchem.ncbi.nlm.nih.gov/compound/89016786#section=2D-Structure
https://pubchem.ncbi.nlm.nih.gov/compound/89012459#section=2D-Structure
https://pubchem.ncbi.nlm.nih.gov/compound/89012264#section=2D-Structure
https://pubchem.ncbi.nlm.nih.gov/compound/88997861#section=2D-Structure
https://pubchem.ncbi.nlm.nih.gov/compound/88997094#section=2D-Structure
https://pubchem.ncbi.nlm.nih.gov/compound/88997071#section=2D-Structure
https://pubchem.ncbi.nlm.nih.gov/compound/88997065#section=2D-Structure
https://pubchem.ncbi.nlm.nih.gov/compound/88996963#section=2D-Structure
https://pubchem.ncbi.nlm.nih.gov/compound/88996961#section=2D-Structure
https://pubchem.ncbi.nlm.nih.gov/compound/88996891#section=2D-Structure
https://pubchem.ncbi.nlm.nih.gov/compound/88996890#section=2D-Structure
https://pubchem.ncbi.nlm.nih.gov/compound/88976177#section=2D-Structure
https://pubchem.ncbi.nlm.nih.gov/compound/88960456#section=2D-Structure
https://pubchem.ncbi.nlm.nih.gov/compound/88958401#section=2D-Structure
https://pubchem.ncbi.nlm.nih.gov/compound/88955323#section=2D-Structure
https://pubchem.ncbi.nlm.nih.gov/compound/88948519#section=2D-Structure
https://pubchem.ncbi.nlm.nih.gov/compound/88946521#section=2D-Structure
https://pubchem.ncbi.nlm.nih.gov/compound/88938377#section=2D-Structure
https://pubchem.ncbi.nlm.nih.gov/compound/88937790#section=2D-Structure
https://pubchem.ncbi.nlm.nih.gov/compound/88931000#section=2D-Structure
https://pubchem.ncbi.nlm.nih.gov/compound/88929946#section=2D-Structure
https://pubchem.ncbi.nlm.nih.gov/compound/8892542#section=2D-Structure
https://pubchem.ncbi.nlm.nih.gov/compound/88925319#section=2D-Structure
https://pubchem.ncbi.nlm.nih.gov/compound/88915093#section=2D-Structure
https://pubchem.ncbi.nlm.nih.gov/compound/88911753#section=2D-Structure
https://pubchem.ncbi.nlm.nih.gov/compound/88908592#section=2D-Structure
https://pubchem.ncbi.nlm.nih.gov/compound/88908591#section=2D-Structure
https://pubchem.ncbi.nlm.nih.gov/compound/88877706#section=2D-Structure
https://pubchem.ncbi.nlm.nih.gov/compound/88861954#section=2D-Structure
https://pubchem.ncbi.nlm.nih.gov/compound/88861945#section=2D-Structure
https://pubchem.ncbi.nlm.nih.gov/compound/88861944#section=2D-Structure
https://pubchem.ncbi.nlm.nih.gov/compound/88861869#section=2D-Structure
https://pubchem.ncbi.nlm.nih.gov/compound/88848357#section=2D-Structure
https://pubchem.ncbi.nlm.nih.gov/compound/88847540#section=2D-Structure
https://pubchem.ncbi.nlm.nih.gov/compound/88842885#section=2D-Structure
https://pubchem.ncbi.nlm.nih.gov/compound/88838587#section=2D-Structure
https://pubchem.ncbi.nlm.nih.gov/compound/88838541#section=2D-Structure
https://pubchem.ncbi.nlm.nih.gov/compound/88838291#section=2D-Structure

https://pubchem.ncbi.nlm.nih.gov/compound/88838186#section=2D-Structure
https://pubchem.ncbi.nlm.nih.gov/compound/88838068#section=2D-Structure
https://pubchem.ncbi.nlm.nih.gov/compound/88837479#section=2D-Structure
https://pubchem.ncbi.nlm.nih.gov/compound/88836305#section=2D-Structure
https://pubchem.ncbi.nlm.nih.gov/compound/88833084#section=2D-Structure
https://pubchem.ncbi.nlm.nih.gov/compound/88831023#section=2D-Structure
https://pubchem.ncbi.nlm.nih.gov/compound/88819687#section=2D-Structure
https://pubchem.ncbi.nlm.nih.gov/compound/88819571#section=2D-Structure
https://pubchem.ncbi.nlm.nih.gov/compound/88818685#section=2D-Structure
https://pubchem.ncbi.nlm.nih.gov/compound/88818270#section=2D-Structure
https://pubchem.ncbi.nlm.nih.gov/compound/88816989#section=2D-Structure
https://pubchem.ncbi.nlm.nih.gov/compound/88814225#section=2D-Structure
https://pubchem.ncbi.nlm.nih.gov/compound/88813538#section=2D-Structure
https://pubchem.ncbi.nlm.nih.gov/compound/88813361#section=2D-Structure
https://pubchem.ncbi.nlm.nih.gov/compound/88813293#section=2D-Structure
https://pubchem.ncbi.nlm.nih.gov/compound/88812553#section=2D-Structure
https://pubchem.ncbi.nlm.nih.gov/compound/88811158#section=2D-Structure
https://pubchem.ncbi.nlm.nih.gov/compound/88809160#section=2D-Structure
https://pubchem.ncbi.nlm.nih.gov/compound/88798973#section=2D-Structure
https://pubchem.ncbi.nlm.nih.gov/compound/88797335#section=2D-Structure
https://pubchem.ncbi.nlm.nih.gov/compound/88792392#section=2D-Structure
https://pubchem.ncbi.nlm.nih.gov/compound/88790143#section=2D-Structure
https://pubchem.ncbi.nlm.nih.gov/compound/88789989#section=2D-Structure
https://pubchem.ncbi.nlm.nih.gov/compound/88785537#section=2D-Structure
https://pubchem.ncbi.nlm.nih.gov/compound/88780805#section=2D-Structure
https://pubchem.ncbi.nlm.nih.gov/compound/88767708#section=2D-Structure
https://pubchem.ncbi.nlm.nih.gov/compound/88767334#section=2D-Structure
https://pubchem.ncbi.nlm.nih.gov/compound/88763798#section=2D-Structure
https://pubchem.ncbi.nlm.nih.gov/compound/88757615#section=2D-Structure
https://pubchem.ncbi.nlm.nih.gov/compound/88755044#section=2D-Structure
https://pubchem.ncbi.nlm.nih.gov/compound/88744717#section=2D-Structure
https://pubchem.ncbi.nlm.nih.gov/compound/88742059#section=2D-Structure
https://pubchem.ncbi.nlm.nih.gov/compound/88735633#section=2D-Structure
https://pubchem.ncbi.nlm.nih.gov/compound/88731892#section=2D-Structure
https://pubchem.ncbi.nlm.nih.gov/compound/88731887#section=2D-Structure
https://pubchem.ncbi.nlm.nih.gov/compound/88731112#section=2D-Structure
https://pubchem.ncbi.nlm.nih.gov/compound/88729670#section=2D-Structure
https://pubchem.ncbi.nlm.nih.gov/compound/88726368#section=2D-Structure
https://pubchem.ncbi.nlm.nih.gov/compound/88718915#section=2D-Structure
https://pubchem.ncbi.nlm.nih.gov/compound/88713744#section=2D-Structure
https://pubchem.ncbi.nlm.nih.gov/compound/88710871#section=2D-Structure
https://pubchem.ncbi.nlm.nih.gov/compound/88708143#section=2D-Structure
https://pubchem.ncbi.nlm.nih.gov/compound/88698515#section=2D-Structure
https://pubchem.ncbi.nlm.nih.gov/compound/88694743#section=2D-Structure
https://pubchem.ncbi.nlm.nih.gov/compound/88684861#section=2D-Structure
https://pubchem.ncbi.nlm.nih.gov/compound/88683070#section=2D-Structure
https://pubchem.ncbi.nlm.nih.gov/compound/88674292#section=2D-Structure
https://pubchem.ncbi.nlm.nih.gov/compound/88657796#section=2D-Structure
https://pubchem.ncbi.nlm.nih.gov/compound/88657788#section=2D-Structure
https://pubchem.ncbi.nlm.nih.gov/compound/88636042#section=2D-Structure
https://pubchem.ncbi.nlm.nih.gov/compound/88633534#section=2D-Structure
https://pubchem.ncbi.nlm.nih.gov/compound/88633316#section=2D-Structure
https://pubchem.ncbi.nlm.nih.gov/compound/88624841#section=2D-Structure
https://pubchem.ncbi.nlm.nih.gov/compound/88621#section=2D-Structure
https://pubchem.ncbi.nlm.nih.gov/compound/88616553#section=2D-Structure

https://pubchem.ncbi.nlm.nih.gov/compound/88616507#section=2D-Structure
https://pubchem.ncbi.nlm.nih.gov/compound/88613006#section=2D-Structure
https://pubchem.ncbi.nlm.nih.gov/compound/88612672#section=2D-Structure
https://pubchem.ncbi.nlm.nih.gov/compound/88608860#section=2D-Structure
https://pubchem.ncbi.nlm.nih.gov/compound/88607546#section=2D-Structure
https://pubchem.ncbi.nlm.nih.gov/compound/88593901#section=2D-Structure
https://pubchem.ncbi.nlm.nih.gov/compound/88573772#section=2D-Structure
https://pubchem.ncbi.nlm.nih.gov/compound/88572135#section=2D-Structure
https://pubchem.ncbi.nlm.nih.gov/compound/88562564#section=2D-Structure
https://pubchem.ncbi.nlm.nih.gov/compound/88558444#section=2D-Structure
https://pubchem.ncbi.nlm.nih.gov/compound/88554302#section=2D-Structure
https://pubchem.ncbi.nlm.nih.gov/compound/88553801#section=2D-Structure
https://pubchem.ncbi.nlm.nih.gov/compound/88553800#section=2D-Structure
https://pubchem.ncbi.nlm.nih.gov/compound/88542282#section=2D-Structure
https://pubchem.ncbi.nlm.nih.gov/compound/88539488#section=2D-Structure
https://pubchem.ncbi.nlm.nih.gov/compound/88537879#section=2D-Structure
https://pubchem.ncbi.nlm.nih.gov/compound/88537846#section=2D-Structure
https://pubchem.ncbi.nlm.nih.gov/compound/88537829#section=2D-Structure
https://pubchem.ncbi.nlm.nih.gov/compound/88537614#section=2D-Structure
https://pubchem.ncbi.nlm.nih.gov/compound/88537601#section=2D-Structure
https://pubchem.ncbi.nlm.nih.gov/compound/88537600#section=2D-Structure
https://pubchem.ncbi.nlm.nih.gov/compound/88537537#section=2D-Structure
https://pubchem.ncbi.nlm.nih.gov/compound/88533726#section=2D-Structure
https://pubchem.ncbi.nlm.nih.gov/compound/88533725#section=2D-Structure
https://pubchem.ncbi.nlm.nih.gov/compound/88532370#section=2D-Structure
https://pubchem.ncbi.nlm.nih.gov/compound/88526073#section=2D-Structure
https://pubchem.ncbi.nlm.nih.gov/compound/88522513#section=2D-Structure
https://pubchem.ncbi.nlm.nih.gov/compound/88520940#section=2D-Structure
https://pubchem.ncbi.nlm.nih.gov/compound/88519712#section=2D-Structure
https://pubchem.ncbi.nlm.nih.gov/compound/88508816#section=2D-Structure
https://pubchem.ncbi.nlm.nih.gov/compound/88503390#section=2D-Structure
https://pubchem.ncbi.nlm.nih.gov/compound/88502298#section=2D-Structure
https://pubchem.ncbi.nlm.nih.gov/compound/88496956#section=2D-Structure
https://pubchem.ncbi.nlm.nih.gov/compound/88489931#section=2D-Structure
https://pubchem.ncbi.nlm.nih.gov/compound/88489929#section=2D-Structure
https://pubchem.ncbi.nlm.nih.gov/compound/88481957#section=2D-Structure
https://pubchem.ncbi.nlm.nih.gov/compound/88479077#section=2D-Structure
https://pubchem.ncbi.nlm.nih.gov/compound/88477746#section=2D-Structure
https://pubchem.ncbi.nlm.nih.gov/compound/88470140#section=2D-Structure
https://pubchem.ncbi.nlm.nih.gov/compound/88469341#section=2D-Structure
https://pubchem.ncbi.nlm.nih.gov/compound/88468087#section=2D-Structure
https://pubchem.ncbi.nlm.nih.gov/compound/88464402#section=2D-Structure
https://pubchem.ncbi.nlm.nih.gov/compound/88458070#section=2D-Structure
https://pubchem.ncbi.nlm.nih.gov/compound/88457848#section=2D-Structure
https://pubchem.ncbi.nlm.nih.gov/compound/88456039#section=2D-Structure
https://pubchem.ncbi.nlm.nih.gov/compound/88455222#section=2D-Structure
https://pubchem.ncbi.nlm.nih.gov/compound/88454067#section=2D-Structure
https://pubchem.ncbi.nlm.nih.gov/compound/88450441#section=2D-Structure
https://pubchem.ncbi.nlm.nih.gov/compound/88443015#section=2D-Structure
https://pubchem.ncbi.nlm.nih.gov/compound/88429716#section=2D-Structure
https://pubchem.ncbi.nlm.nih.gov/compound/88413917#section=2D-Structure
https://pubchem.ncbi.nlm.nih.gov/compound/88407216#section=2D-Structure
https://pubchem.ncbi.nlm.nih.gov/compound/88404740#section=2D-Structure
https://pubchem.ncbi.nlm.nih.gov/compound/88404079#section=2D-Structure
https://pubchem.ncbi.nlm.nih.gov/compound/88403809#section=2D-Structure

https://pubchem.ncbi.nlm.nih.gov/compound/88396674#section=2D-Structure
https://pubchem.ncbi.nlm.nih.gov/compound/88392924#section=2D-Structure
https://pubchem.ncbi.nlm.nih.gov/compound/88383933#section=2D-Structure
https://pubchem.ncbi.nlm.nih.gov/compound/88383841#section=2D-Structure
https://pubchem.ncbi.nlm.nih.gov/compound/88381598#section=2D-Structure
https://pubchem.ncbi.nlm.nih.gov/compound/88376116#section=2D-Structure
https://pubchem.ncbi.nlm.nih.gov/compound/88362867#section=2D-Structure
https://pubchem.ncbi.nlm.nih.gov/compound/88359939#section=2D-Structure
https://pubchem.ncbi.nlm.nih.gov/compound/88338129#section=2D-Structure
https://pubchem.ncbi.nlm.nih.gov/compound/88337095#section=2D-Structure
https://pubchem.ncbi.nlm.nih.gov/compound/88330237#section=2D-Structure
https://pubchem.ncbi.nlm.nih.gov/compound/88317535#section=2D-Structure
https://pubchem.ncbi.nlm.nih.gov/compound/88317522#section=2D-Structure
https://pubchem.ncbi.nlm.nih.gov/compound/88314468#section=2D-Structure
https://pubchem.ncbi.nlm.nih.gov/compound/88293768#section=2D-Structure
https://pubchem.ncbi.nlm.nih.gov/compound/88288023#section=2D-Structure
https://pubchem.ncbi.nlm.nih.gov/compound/88280299#section=2D-Structure
https://pubchem.ncbi.nlm.nih.gov/compound/88276792#section=2D-Structure
https://pubchem.ncbi.nlm.nih.gov/compound/88264492#section=2D-Structure
https://pubchem.ncbi.nlm.nih.gov/compound/88264026#section=2D-Structure
https://pubchem.ncbi.nlm.nih.gov/compound/88263708#section=2D-Structure
https://pubchem.ncbi.nlm.nih.gov/compound/88262616#section=2D-Structure
https://pubchem.ncbi.nlm.nih.gov/compound/88262338#section=2D-Structure
https://pubchem.ncbi.nlm.nih.gov/compound/88256365#section=2D-Structure
https://pubchem.ncbi.nlm.nih.gov/compound/88253194#section=2D-Structure
https://pubchem.ncbi.nlm.nih.gov/compound/88251487#section=2D-Structure
https://pubchem.ncbi.nlm.nih.gov/compound/88240288#section=2D-Structure
https://pubchem.ncbi.nlm.nih.gov/compound/88236407#section=2D-Structure
https://pubchem.ncbi.nlm.nih.gov/compound/88233770#section=2D-Structure
https://pubchem.ncbi.nlm.nih.gov/compound/88225814#section=2D-Structure
https://pubchem.ncbi.nlm.nih.gov/compound/88218975#section=2D-Structure
https://pubchem.ncbi.nlm.nih.gov/compound/88218469#section=2D-Structure
https://pubchem.ncbi.nlm.nih.gov/compound/88216924#section=2D-Structure
https://pubchem.ncbi.nlm.nih.gov/compound/88211357#section=2D-Structure
https://pubchem.ncbi.nlm.nih.gov/compound/88200077#section=2D-Structure
https://pubchem.ncbi.nlm.nih.gov/compound/88195794#section=2D-Structure
https://pubchem.ncbi.nlm.nih.gov/compound/88193853#section=2D-Structure
https://pubchem.ncbi.nlm.nih.gov/compound/88193851#section=2D-Structure
https://pubchem.ncbi.nlm.nih.gov/compound/88193063#section=2D-Structure
https://pubchem.ncbi.nlm.nih.gov/compound/88189400#section=2D-Structure
https://pubchem.ncbi.nlm.nih.gov/compound/88188107#section=2D-Structure
https://pubchem.ncbi.nlm.nih.gov/compound/88174654#section=2D-Structure
https://pubchem.ncbi.nlm.nih.gov/compound/88170777#section=2D-Structure
https://pubchem.ncbi.nlm.nih.gov/compound/88166162#section=2D-Structure
https://pubchem.ncbi.nlm.nih.gov/compound/88158599#section=2D-Structure
https://pubchem.ncbi.nlm.nih.gov/compound/88158381#section=2D-Structure
https://pubchem.ncbi.nlm.nih.gov/compound/88157630#section=2D-Structure
https://pubchem.ncbi.nlm.nih.gov/compound/88157232#section=2D-Structure
https://pubchem.ncbi.nlm.nih.gov/compound/88156507#section=2D-Structure
https://pubchem.ncbi.nlm.nih.gov/compound/88156438#section=2D-Structure
https://pubchem.ncbi.nlm.nih.gov/compound/88155690#section=2D-Structure
https://pubchem.ncbi.nlm.nih.gov/compound/88148186#section=2D-Structure
https://pubchem.ncbi.nlm.nih.gov/compound/88145313#section=2D-Structure
https://pubchem.ncbi.nlm.nih.gov/compound/88133631#section=2D-Structure
https://pubchem.ncbi.nlm.nih.gov/compound/88132978#section=2D-Structure

https://pubchem.ncbi.nlm.nih.gov/compound/88119905#section=2D-Structure
https://pubchem.ncbi.nlm.nih.gov/compound/88118565#section=2D-Structure
https://pubchem.ncbi.nlm.nih.gov/compound/88116811#section=2D-Structure
https://pubchem.ncbi.nlm.nih.gov/compound/88099577#section=2D-Structure
https://pubchem.ncbi.nlm.nih.gov/compound/88095096#section=2D-Structure
https://pubchem.ncbi.nlm.nih.gov/compound/88092975#section=2D-Structure
https://pubchem.ncbi.nlm.nih.gov/compound/88085816#section=2D-Structure
https://pubchem.ncbi.nlm.nih.gov/compound/88085561#section=2D-Structure
https://pubchem.ncbi.nlm.nih.gov/compound/88085484#section=2D-Structure
https://pubchem.ncbi.nlm.nih.gov/compound/88085196#section=2D-Structure
https://pubchem.ncbi.nlm.nih.gov/compound/88085188#section=2D-Structure
https://pubchem.ncbi.nlm.nih.gov/compound/88082099#section=2D-Structure
https://pubchem.ncbi.nlm.nih.gov/compound/88080326#section=2D-Structure
https://pubchem.ncbi.nlm.nih.gov/compound/88078278#section=2D-Structure
https://pubchem.ncbi.nlm.nih.gov/compound/88077557#section=2D-Structure
https://pubchem.ncbi.nlm.nih.gov/compound/88075676#section=2D-Structure
https://pubchem.ncbi.nlm.nih.gov/compound/88072747#section=2D-Structure
https://pubchem.ncbi.nlm.nih.gov/compound/88066114#section=2D-Structure
https://pubchem.ncbi.nlm.nih.gov/compound/88053216#section=2D-Structure
https://pubchem.ncbi.nlm.nih.gov/compound/88047121#section=2D-Structure
https://pubchem.ncbi.nlm.nih.gov/compound/88031226#section=2D-Structure
https://pubchem.ncbi.nlm.nih.gov/compound/88029958#section=2D-Structure
https://pubchem.ncbi.nlm.nih.gov/compound/88022943#section=2D-Structure
https://pubchem.ncbi.nlm.nih.gov/compound/88022686#section=2D-Structure
https://pubchem.ncbi.nlm.nih.gov/compound/88022685#section=2D-Structure
https://pubchem.ncbi.nlm.nih.gov/compound/88022211#section=2D-Structure
https://pubchem.ncbi.nlm.nih.gov/compound/88022114#section=2D-Structure
https://pubchem.ncbi.nlm.nih.gov/compound/88021912#section=2D-Structure
https://pubchem.ncbi.nlm.nih.gov/compound/88020311#section=2D-Structure
https://pubchem.ncbi.nlm.nih.gov/compound/88018613#section=2D-Structure
https://pubchem.ncbi.nlm.nih.gov/compound/88017551#section=2D-Structure
https://pubchem.ncbi.nlm.nih.gov/compound/88008564#section=2D-Structure
https://pubchem.ncbi.nlm.nih.gov/compound/87999593#section=2D-Structure
https://pubchem.ncbi.nlm.nih.gov/compound/87998227#section=2D-Structure
https://pubchem.ncbi.nlm.nih.gov/compound/87990220#section=2D-Structure
https://pubchem.ncbi.nlm.nih.gov/compound/87973944#section=2D-Structure
https://pubchem.ncbi.nlm.nih.gov/compound/87968598#section=2D-Structure
https://pubchem.ncbi.nlm.nih.gov/compound/87965561#section=2D-Structure
https://pubchem.ncbi.nlm.nih.gov/compound/87964336#section=2D-Structure
https://pubchem.ncbi.nlm.nih.gov/compound/87962872#section=2D-Structure
https://pubchem.ncbi.nlm.nih.gov/compound/87956770#section=2D-Structure
https://pubchem.ncbi.nlm.nih.gov/compound/87955890#section=2D-Structure
https://pubchem.ncbi.nlm.nih.gov/compound/87949218#section=2D-Structure
https://pubchem.ncbi.nlm.nih.gov/compound/87948547#section=2D-Structure
https://pubchem.ncbi.nlm.nih.gov/compound/87924143#section=2D-Structure
https://pubchem.ncbi.nlm.nih.gov/compound/87916984#section=2D-Structure
https://pubchem.ncbi.nlm.nih.gov/compound/87916960#section=2D-Structure
https://pubchem.ncbi.nlm.nih.gov/compound/87916952#section=2D-Structure
https://pubchem.ncbi.nlm.nih.gov/compound/87916933#section=2D-Structure
https://pubchem.ncbi.nlm.nih.gov/compound/87916914#section=2D-Structure
https://pubchem.ncbi.nlm.nih.gov/compound/87916912#section=2D-Structure
https://pubchem.ncbi.nlm.nih.gov/compound/87916908#section=2D-Structure
https://pubchem.ncbi.nlm.nih.gov/compound/87916894#section=2D-Structure
https://pubchem.ncbi.nlm.nih.gov/compound/87916885#section=2D-Structure
https://pubchem.ncbi.nlm.nih.gov/compound/87916864#section=2D-Structure

https://pubchem.ncbi.nlm.nih.gov/compound/87916859#section=2D-Structure
https://pubchem.ncbi.nlm.nih.gov/compound/87916829#section=2D-Structure
https://pubchem.ncbi.nlm.nih.gov/compound/87916821#section=2D-Structure
https://pubchem.ncbi.nlm.nih.gov/compound/87916795#section=2D-Structure
https://pubchem.ncbi.nlm.nih.gov/compound/87896477#section=2D-Structure
https://pubchem.ncbi.nlm.nih.gov/compound/87888990#section=2D-Structure
https://pubchem.ncbi.nlm.nih.gov/compound/87882567#section=2D-Structure
https://pubchem.ncbi.nlm.nih.gov/compound/87881085#section=2D-Structure
https://pubchem.ncbi.nlm.nih.gov/compound/87877627#section=2D-Structure
https://pubchem.ncbi.nlm.nih.gov/compound/87876067#section=2D-Structure
https://pubchem.ncbi.nlm.nih.gov/compound/87872657#section=2D-Structure
https://pubchem.ncbi.nlm.nih.gov/compound/87856905#section=2D-Structure
https://pubchem.ncbi.nlm.nih.gov/compound/87850839#section=2D-Structure
https://pubchem.ncbi.nlm.nih.gov/compound/87842723#section=2D-Structure
https://pubchem.ncbi.nlm.nih.gov/compound/87837583#section=2D-Structure
https://pubchem.ncbi.nlm.nih.gov/compound/87837581#section=2D-Structure
https://pubchem.ncbi.nlm.nih.gov/compound/87836269#section=2D-Structure
https://pubchem.ncbi.nlm.nih.gov/compound/87830456#section=2D-Structure
https://pubchem.ncbi.nlm.nih.gov/compound/87828901#section=2D-Structure
https://pubchem.ncbi.nlm.nih.gov/compound/87828177#section=2D-Structure
https://pubchem.ncbi.nlm.nih.gov/compound/87813276#section=2D-Structure
https://pubchem.ncbi.nlm.nih.gov/compound/87813274#section=2D-Structure
https://pubchem.ncbi.nlm.nih.gov/compound/87806022#section=2D-Structure
https://pubchem.ncbi.nlm.nih.gov/compound/87806019#section=2D-Structure
https://pubchem.ncbi.nlm.nih.gov/compound/87806018#section=2D-Structure
https://pubchem.ncbi.nlm.nih.gov/compound/87800803#section=2D-Structure
https://pubchem.ncbi.nlm.nih.gov/compound/87796329#section=2D-Structure
https://pubchem.ncbi.nlm.nih.gov/compound/87789792#section=2D-Structure
https://pubchem.ncbi.nlm.nih.gov/compound/87780745#section=2D-Structure
https://pubchem.ncbi.nlm.nih.gov/compound/87780306#section=2D-Structure
https://pubchem.ncbi.nlm.nih.gov/compound/87763981#section=2D-Structure
https://pubchem.ncbi.nlm.nih.gov/compound/87763980#section=2D-Structure
https://pubchem.ncbi.nlm.nih.gov/compound/87758879#section=2D-Structure
https://pubchem.ncbi.nlm.nih.gov/compound/87754172#section=2D-Structure
https://pubchem.ncbi.nlm.nih.gov/compound/87749921#section=2D-Structure
https://pubchem.ncbi.nlm.nih.gov/compound/87744208#section=2D-Structure
https://pubchem.ncbi.nlm.nih.gov/compound/87743318#section=2D-Structure
https://pubchem.ncbi.nlm.nih.gov/compound/87734#section=2D-Structure
https://pubchem.ncbi.nlm.nih.gov/compound/87729970#section=2D-Structure
https://pubchem.ncbi.nlm.nih.gov/compound/87727374#section=2D-Structure
https://pubchem.ncbi.nlm.nih.gov/compound/87727196#section=2D-Structure
https://pubchem.ncbi.nlm.nih.gov/compound/87724136#section=2D-Structure
https://pubchem.ncbi.nlm.nih.gov/compound/87715895#section=2D-Structure
https://pubchem.ncbi.nlm.nih.gov/compound/87715562#section=2D-Structure
https://pubchem.ncbi.nlm.nih.gov/compound/87709726#section=2D-Structure
https://pubchem.ncbi.nlm.nih.gov/compound/87708343#section=2D-Structure
https://pubchem.ncbi.nlm.nih.gov/compound/87697612#section=2D-Structure
https://pubchem.ncbi.nlm.nih.gov/compound/87693797#section=2D-Structure
https://pubchem.ncbi.nlm.nih.gov/compound/87680128#section=2D-Structure
https://pubchem.ncbi.nlm.nih.gov/compound/87679825#section=2D-Structure
https://pubchem.ncbi.nlm.nih.gov/compound/87679653#section=2D-Structure
https://pubchem.ncbi.nlm.nih.gov/compound/87678241#section=2D-Structure
https://pubchem.ncbi.nlm.nih.gov/compound/87665733#section=2D-Structure
https://pubchem.ncbi.nlm.nih.gov/compound/87654119#section=2D-Structure
https://pubchem.ncbi.nlm.nih.gov/compound/87652944#section=2D-Structure

https://pubchem.ncbi.nlm.nih.gov/compound/87652073#section=2D-Structure
https://pubchem.ncbi.nlm.nih.gov/compound/87651472#section=2D-Structure
https://pubchem.ncbi.nlm.nih.gov/compound/87651301#section=2D-Structure
https://pubchem.ncbi.nlm.nih.gov/compound/87645565#section=2D-Structure
https://pubchem.ncbi.nlm.nih.gov/compound/87644087#section=2D-Structure
https://pubchem.ncbi.nlm.nih.gov/compound/87642837#section=2D-Structure
https://pubchem.ncbi.nlm.nih.gov/compound/87641731#section=2D-Structure
https://pubchem.ncbi.nlm.nih.gov/compound/87630604#section=2D-Structure
https://pubchem.ncbi.nlm.nih.gov/compound/87629629#section=2D-Structure
https://pubchem.ncbi.nlm.nih.gov/compound/87628970#section=2D-Structure
https://pubchem.ncbi.nlm.nih.gov/compound/87627279#section=2D-Structure
https://pubchem.ncbi.nlm.nih.gov/compound/87622378#section=2D-Structure
https://pubchem.ncbi.nlm.nih.gov/compound/87621368#section=2D-Structure
https://pubchem.ncbi.nlm.nih.gov/compound/87621202#section=2D-Structure
https://pubchem.ncbi.nlm.nih.gov/compound/87616693#section=2D-Structure
https://pubchem.ncbi.nlm.nih.gov/compound/87616086#section=2D-Structure
https://pubchem.ncbi.nlm.nih.gov/compound/87591622#section=2D-Structure
https://pubchem.ncbi.nlm.nih.gov/compound/87590087#section=2D-Structure
https://pubchem.ncbi.nlm.nih.gov/compound/87589535#section=2D-Structure
https://pubchem.ncbi.nlm.nih.gov/compound/87589534#section=2D-Structure
https://pubchem.ncbi.nlm.nih.gov/compound/87589227#section=2D-Structure
https://pubchem.ncbi.nlm.nih.gov/compound/87588561#section=2D-Structure
https://pubchem.ncbi.nlm.nih.gov/compound/87586132#section=2D-Structure
https://pubchem.ncbi.nlm.nih.gov/compound/87574557#section=2D-Structure
https://pubchem.ncbi.nlm.nih.gov/compound/87567156#section=2D-Structure
https://pubchem.ncbi.nlm.nih.gov/compound/87565757#section=2D-Structure
https://pubchem.ncbi.nlm.nih.gov/compound/87564525#section=2D-Structure
https://pubchem.ncbi.nlm.nih.gov/compound/87561150#section=2D-Structure
https://pubchem.ncbi.nlm.nih.gov/compound/87560740#section=2D-Structure
https://pubchem.ncbi.nlm.nih.gov/compound/87557735#section=2D-Structure
https://pubchem.ncbi.nlm.nih.gov/compound/87548660#section=2D-Structure
https://pubchem.ncbi.nlm.nih.gov/compound/87536144#section=2D-Structure
https://pubchem.ncbi.nlm.nih.gov/compound/87532517#section=2D-Structure
https://pubchem.ncbi.nlm.nih.gov/compound/87529460#section=2D-Structure
https://pubchem.ncbi.nlm.nih.gov/compound/87512128#section=2D-Structure
https://pubchem.ncbi.nlm.nih.gov/compound/87512127#section=2D-Structure
https://pubchem.ncbi.nlm.nih.gov/compound/87510015#section=2D-Structure
https://pubchem.ncbi.nlm.nih.gov/compound/87510014#section=2D-Structure
https://pubchem.ncbi.nlm.nih.gov/compound/87509773#section=2D-Structure
https://pubchem.ncbi.nlm.nih.gov/compound/87509772#section=2D-Structure
https://pubchem.ncbi.nlm.nih.gov/compound/87502110#section=2D-Structure
https://pubchem.ncbi.nlm.nih.gov/compound/87498486#section=2D-Structure
https://pubchem.ncbi.nlm.nih.gov/compound/87498462#section=2D-Structure
https://pubchem.ncbi.nlm.nih.gov/compound/87497187#section=2D-Structure
https://pubchem.ncbi.nlm.nih.gov/compound/87488330#section=2D-Structure
https://pubchem.ncbi.nlm.nih.gov/compound/87477349#section=2D-Structure
https://pubchem.ncbi.nlm.nih.gov/compound/87475289#section=2D-Structure
https://pubchem.ncbi.nlm.nih.gov/compound/87469589#section=2D-Structure
https://pubchem.ncbi.nlm.nih.gov/compound/87462#section=2D-Structure
https://pubchem.ncbi.nlm.nih.gov/compound/87456489#section=2D-Structure
https://pubchem.ncbi.nlm.nih.gov/compound/87452991#section=2D-Structure
https://pubchem.ncbi.nlm.nih.gov/compound/87438498#section=2D-Structure
https://pubchem.ncbi.nlm.nih.gov/compound/87438497#section=2D-Structure
https://pubchem.ncbi.nlm.nih.gov/compound/87436806#section=2D-Structure
https://pubchem.ncbi.nlm.nih.gov/compound/87436462#section=2D-Structure

https://pubchem.ncbi.nlm.nih.gov/compound/87426054#section=2D-Structure
https://pubchem.ncbi.nlm.nih.gov/compound/87425767#section=2D-Structure
https://pubchem.ncbi.nlm.nih.gov/compound/87425648#section=2D-Structure
https://pubchem.ncbi.nlm.nih.gov/compound/87421094#section=2D-Structure
https://pubchem.ncbi.nlm.nih.gov/compound/87411260#section=2D-Structure
https://pubchem.ncbi.nlm.nih.gov/compound/87407229#section=2D-Structure
https://pubchem.ncbi.nlm.nih.gov/compound/87402228#section=2D-Structure
https://pubchem.ncbi.nlm.nih.gov/compound/87401701#section=2D-Structure
https://pubchem.ncbi.nlm.nih.gov/compound/87394384#section=2D-Structure
https://pubchem.ncbi.nlm.nih.gov/compound/87390098#section=2D-Structure
https://pubchem.ncbi.nlm.nih.gov/compound/87382626#section=2D-Structure
https://pubchem.ncbi.nlm.nih.gov/compound/87382375#section=2D-Structure
https://pubchem.ncbi.nlm.nih.gov/compound/87380874#section=2D-Structure
https://pubchem.ncbi.nlm.nih.gov/compound/87377118#section=2D-Structure
https://pubchem.ncbi.nlm.nih.gov/compound/87377114#section=2D-Structure
https://pubchem.ncbi.nlm.nih.gov/compound/87367678#section=2D-Structure
https://pubchem.ncbi.nlm.nih.gov/compound/87361580#section=2D-Structure
https://pubchem.ncbi.nlm.nih.gov/compound/87350071#section=2D-Structure
https://pubchem.ncbi.nlm.nih.gov/compound/87329973#section=2D-Structure
https://pubchem.ncbi.nlm.nih.gov/compound/87327760#section=2D-Structure
https://pubchem.ncbi.nlm.nih.gov/compound/87327690#section=2D-Structure
https://pubchem.ncbi.nlm.nih.gov/compound/87325194#section=2D-Structure
https://pubchem.ncbi.nlm.nih.gov/compound/87318815#section=2D-Structure
https://pubchem.ncbi.nlm.nih.gov/compound/87311427#section=2D-Structure
https://pubchem.ncbi.nlm.nih.gov/compound/87311369#section=2D-Structure
https://pubchem.ncbi.nlm.nih.gov/compound/87308505#section=2D-Structure
https://pubchem.ncbi.nlm.nih.gov/compound/87308504#section=2D-Structure
https://pubchem.ncbi.nlm.nih.gov/compound/87302903#section=2D-Structure
https://pubchem.ncbi.nlm.nih.gov/compound/87293528#section=2D-Structure
https://pubchem.ncbi.nlm.nih.gov/compound/87277136#section=2D-Structure
https://pubchem.ncbi.nlm.nih.gov/compound/87271744#section=2D-Structure
https://pubchem.ncbi.nlm.nih.gov/compound/87271743#section=2D-Structure
https://pubchem.ncbi.nlm.nih.gov/compound/87265264#section=2D-Structure
https://pubchem.ncbi.nlm.nih.gov/compound/87259420#section=2D-Structure
https://pubchem.ncbi.nlm.nih.gov/compound/87257049#section=2D-Structure
https://pubchem.ncbi.nlm.nih.gov/compound/87251064#section=2D-Structure
https://pubchem.ncbi.nlm.nih.gov/compound/87250039#section=2D-Structure
https://pubchem.ncbi.nlm.nih.gov/compound/87246726#section=2D-Structure
https://pubchem.ncbi.nlm.nih.gov/compound/87242385#section=2D-Structure
https://pubchem.ncbi.nlm.nih.gov/compound/87237001#section=2D-Structure
https://pubchem.ncbi.nlm.nih.gov/compound/87231976#section=2D-Structure
https://pubchem.ncbi.nlm.nih.gov/compound/87231692#section=2D-Structure
https://pubchem.ncbi.nlm.nih.gov/compound/87231515#section=2D-Structure
https://pubchem.ncbi.nlm.nih.gov/compound/87231253#section=2D-Structure
https://pubchem.ncbi.nlm.nih.gov/compound/87229211#section=2D-Structure
https://pubchem.ncbi.nlm.nih.gov/compound/87221186#section=2D-Structure
https://pubchem.ncbi.nlm.nih.gov/compound/87219930#section=2D-Structure
https://pubchem.ncbi.nlm.nih.gov/compound/87216096#section=2D-Structure
https://pubchem.ncbi.nlm.nih.gov/compound/87199346#section=2D-Structure
https://pubchem.ncbi.nlm.nih.gov/compound/87197031#section=2D-Structure
https://pubchem.ncbi.nlm.nih.gov/compound/87194565#section=2D-Structure
https://pubchem.ncbi.nlm.nih.gov/compound/87194048#section=2D-Structure
https://pubchem.ncbi.nlm.nih.gov/compound/87184295#section=2D-Structure
https://pubchem.ncbi.nlm.nih.gov/compound/87183084#section=2D-Structure
https://pubchem.ncbi.nlm.nih.gov/compound/87174172#section=2D-Structure

https://pubchem.ncbi.nlm.nih.gov/compound/87166280#section=2D-Structure
https://pubchem.ncbi.nlm.nih.gov/compound/87164739#section=2D-Structure
https://pubchem.ncbi.nlm.nih.gov/compound/87162997#section=2D-Structure
https://pubchem.ncbi.nlm.nih.gov/compound/87162801#section=2D-Structure
https://pubchem.ncbi.nlm.nih.gov/compound/87161356#section=2D-Structure
https://pubchem.ncbi.nlm.nih.gov/compound/87160965#section=2D-Structure
https://pubchem.ncbi.nlm.nih.gov/compound/87158691#section=2D-Structure
https://pubchem.ncbi.nlm.nih.gov/compound/87149463#section=2D-Structure
https://pubchem.ncbi.nlm.nih.gov/compound/87149150#section=2D-Structure
https://pubchem.ncbi.nlm.nih.gov/compound/87148329#section=2D-Structure
https://pubchem.ncbi.nlm.nih.gov/compound/87147682#section=2D-Structure
https://pubchem.ncbi.nlm.nih.gov/compound/87139974#section=2D-Structure
https://pubchem.ncbi.nlm.nih.gov/compound/87139855#section=2D-Structure
https://pubchem.ncbi.nlm.nih.gov/compound/87139780#section=2D-Structure
https://pubchem.ncbi.nlm.nih.gov/compound/87139650#section=2D-Structure
https://pubchem.ncbi.nlm.nih.gov/compound/87131615#section=2D-Structure
https://pubchem.ncbi.nlm.nih.gov/compound/87129022#section=2D-Structure
https://pubchem.ncbi.nlm.nih.gov/compound/87127386#section=2D-Structure
https://pubchem.ncbi.nlm.nih.gov/compound/87127290#section=2D-Structure
https://pubchem.ncbi.nlm.nih.gov/compound/87127289#section=2D-Structure
https://pubchem.ncbi.nlm.nih.gov/compound/87119535#section=2D-Structure
https://pubchem.ncbi.nlm.nih.gov/compound/87111693#section=2D-Structure
https://pubchem.ncbi.nlm.nih.gov/compound/87107540#section=2D-Structure
https://pubchem.ncbi.nlm.nih.gov/compound/87097137#section=2D-Structure
https://pubchem.ncbi.nlm.nih.gov/compound/87091753#section=2D-Structure
https://pubchem.ncbi.nlm.nih.gov/compound/87087960#section=2D-Structure
https://pubchem.ncbi.nlm.nih.gov/compound/87081164#section=2D-Structure
https://pubchem.ncbi.nlm.nih.gov/compound/87073319#section=2D-Structure
https://pubchem.ncbi.nlm.nih.gov/compound/87073257#section=2D-Structure
https://pubchem.ncbi.nlm.nih.gov/compound/87068996#section=2D-Structure
https://pubchem.ncbi.nlm.nih.gov/compound/87064953#section=2D-Structure
https://pubchem.ncbi.nlm.nih.gov/compound/87061525#section=2D-Structure
https://pubchem.ncbi.nlm.nih.gov/compound/87059627#section=2D-Structure
https://pubchem.ncbi.nlm.nih.gov/compound/87059105#section=2D-Structure
https://pubchem.ncbi.nlm.nih.gov/compound/86735206#section=2D-Structure
https://pubchem.ncbi.nlm.nih.gov/compound/86718300#section=2D-Structure
https://pubchem.ncbi.nlm.nih.gov/compound/8671#section=2D-Structure
https://pubchem.ncbi.nlm.nih.gov/compound/86702564#section=2D-Structure
https://pubchem.ncbi.nlm.nih.gov/compound/86690507#section=2D-Structure
https://pubchem.ncbi.nlm.nih.gov/compound/86685012#section=2D-Structure
https://pubchem.ncbi.nlm.nih.gov/compound/86650349#section=2D-Structure
https://pubchem.ncbi.nlm.nih.gov/compound/865242#section=2D-Structure
https://pubchem.ncbi.nlm.nih.gov/compound/865240#section=2D-Structure
https://pubchem.ncbi.nlm.nih.gov/compound/865239#section=2D-Structure
https://pubchem.ncbi.nlm.nih.gov/compound/865237#section=2D-Structure
https://pubchem.ncbi.nlm.nih.gov/compound/86386#section=2D-Structure
https://pubchem.ncbi.nlm.nih.gov/compound/86293752#section=2D-Structure
https://pubchem.ncbi.nlm.nih.gov/compound/86271907#section=2D-Structure
https://pubchem.ncbi.nlm.nih.gov/compound/86271906#section=2D-Structure
https://pubchem.ncbi.nlm.nih.gov/compound/86271814#section=2D-Structure
https://pubchem.ncbi.nlm.nih.gov/compound/86271812#section=2D-Structure
https://pubchem.ncbi.nlm.nih.gov/compound/86270594#section=2D-Structure
https://pubchem.ncbi.nlm.nih.gov/compound/86270592#section=2D-Structure
https://pubchem.ncbi.nlm.nih.gov/compound/86270504#section=2D-Structure
https://pubchem.ncbi.nlm.nih.gov/compound/86268849#section=2D-Structure

https://pubchem.ncbi.nlm.nih.gov/compound/86268645#section=2D-Structure
https://pubchem.ncbi.nlm.nih.gov/compound/86268440#section=2D-Structure
https://pubchem.ncbi.nlm.nih.gov/compound/86255069#section=2D-Structure
https://pubchem.ncbi.nlm.nih.gov/compound/86250212#section=2D-Structure
https://pubchem.ncbi.nlm.nih.gov/compound/86248239#section=2D-Structure
https://pubchem.ncbi.nlm.nih.gov/compound/86246732#section=2D-Structure
https://pubchem.ncbi.nlm.nih.gov/compound/86230350#section=2D-Structure
https://pubchem.ncbi.nlm.nih.gov/compound/86221937#section=2D-Structure
https://pubchem.ncbi.nlm.nih.gov/compound/86209409#section=2D-Structure
https://pubchem.ncbi.nlm.nih.gov/compound/86209346#section=2D-Structure
https://pubchem.ncbi.nlm.nih.gov/compound/86204601#section=2D-Structure
https://pubchem.ncbi.nlm.nih.gov/compound/86199127#section=2D-Structure
https://pubchem.ncbi.nlm.nih.gov/compound/86181989#section=2D-Structure
https://pubchem.ncbi.nlm.nih.gov/compound/86181893#section=2D-Structure
https://pubchem.ncbi.nlm.nih.gov/compound/86181474#section=2D-Structure
https://pubchem.ncbi.nlm.nih.gov/compound/86181400#section=2D-Structure
https://pubchem.ncbi.nlm.nih.gov/compound/86178899#section=2D-Structure
https://pubchem.ncbi.nlm.nih.gov/compound/86178898#section=2D-Structure
https://pubchem.ncbi.nlm.nih.gov/compound/86175536#section=2D-Structure
https://pubchem.ncbi.nlm.nih.gov/compound/86167504#section=2D-Structure
https://pubchem.ncbi.nlm.nih.gov/compound/86156430#section=2D-Structure
https://pubchem.ncbi.nlm.nih.gov/compound/86150101#section=2D-Structure
https://pubchem.ncbi.nlm.nih.gov/compound/86149135#section=2D-Structure
https://pubchem.ncbi.nlm.nih.gov/compound/86142682#section=2D-Structure
https://pubchem.ncbi.nlm.nih.gov/compound/86138861#section=2D-Structure
https://pubchem.ncbi.nlm.nih.gov/compound/86131399#section=2D-Structure
https://pubchem.ncbi.nlm.nih.gov/compound/86126564#section=2D-Structure
https://pubchem.ncbi.nlm.nih.gov/compound/86107660#section=2D-Structure
https://pubchem.ncbi.nlm.nih.gov/compound/86103978#section=2D-Structure
https://pubchem.ncbi.nlm.nih.gov/compound/86103975#section=2D-Structure
https://pubchem.ncbi.nlm.nih.gov/compound/86094688#section=2D-Structure
https://pubchem.ncbi.nlm.nih.gov/compound/86094687#section=2D-Structure
https://pubchem.ncbi.nlm.nih.gov/compound/86094639#section=2D-Structure
https://pubchem.ncbi.nlm.nih.gov/compound/86091416#section=2D-Structure
https://pubchem.ncbi.nlm.nih.gov/compound/86083807#section=2D-Structure
https://pubchem.ncbi.nlm.nih.gov/compound/86082125#section=2D-Structure
https://pubchem.ncbi.nlm.nih.gov/compound/86065776#section=2D-Structure
https://pubchem.ncbi.nlm.nih.gov/compound/86054201#section=2D-Structure
https://pubchem.ncbi.nlm.nih.gov/compound/86044621#section=2D-Structure
https://pubchem.ncbi.nlm.nih.gov/compound/86038643#section=2D-Structure
https://pubchem.ncbi.nlm.nih.gov/compound/86033799#section=2D-Structure
https://pubchem.ncbi.nlm.nih.gov/compound/86028708#section=2D-Structure
https://pubchem.ncbi.nlm.nih.gov/compound/86022432#section=2D-Structure
https://pubchem.ncbi.nlm.nih.gov/compound/86021816#section=2D-Structure
https://pubchem.ncbi.nlm.nih.gov/compound/86021739#section=2D-Structure
https://pubchem.ncbi.nlm.nih.gov/compound/86020204#section=2D-Structure
https://pubchem.ncbi.nlm.nih.gov/compound/85997156#section=2D-Structure
https://pubchem.ncbi.nlm.nih.gov/compound/85995800#section=2D-Structure
https://pubchem.ncbi.nlm.nih.gov/compound/85990645#section=2D-Structure
https://pubchem.ncbi.nlm.nih.gov/compound/85989472#section=2D-Structure
https://pubchem.ncbi.nlm.nih.gov/compound/85988346#section=2D-Structure
https://pubchem.ncbi.nlm.nih.gov/compound/85980878#section=2D-Structure
https://pubchem.ncbi.nlm.nih.gov/compound/85980365#section=2D-Structure
https://pubchem.ncbi.nlm.nih.gov/compound/85980358#section=2D-Structure
https://pubchem.ncbi.nlm.nih.gov/compound/85971926#section=2D-Structure

https://pubchem.ncbi.nlm.nih.gov/compound/85966155#section=2D-Structure
https://pubchem.ncbi.nlm.nih.gov/compound/85956589#section=2D-Structure
https://pubchem.ncbi.nlm.nih.gov/compound/85952659#section=2D-Structure
https://pubchem.ncbi.nlm.nih.gov/compound/85939688#section=2D-Structure
https://pubchem.ncbi.nlm.nih.gov/compound/85924962#section=2D-Structure
https://pubchem.ncbi.nlm.nih.gov/compound/85915940#section=2D-Structure
https://pubchem.ncbi.nlm.nih.gov/compound/85915837#section=2D-Structure
https://pubchem.ncbi.nlm.nih.gov/compound/85909543#section=2D-Structure
https://pubchem.ncbi.nlm.nih.gov/compound/85908018#section=2D-Structure
https://pubchem.ncbi.nlm.nih.gov/compound/85887759#section=2D-Structure
https://pubchem.ncbi.nlm.nih.gov/compound/85886989#section=2D-Structure
https://pubchem.ncbi.nlm.nih.gov/compound/85886323#section=2D-Structure
https://pubchem.ncbi.nlm.nih.gov/compound/85878187#section=2D-Structure
https://pubchem.ncbi.nlm.nih.gov/compound/85868427#section=2D-Structure
https://pubchem.ncbi.nlm.nih.gov/compound/85868394#section=2D-Structure
https://pubchem.ncbi.nlm.nih.gov/compound/85844002#section=2D-Structure
https://pubchem.ncbi.nlm.nih.gov/compound/85842348#section=2D-Structure
https://pubchem.ncbi.nlm.nih.gov/compound/85835775#section=2D-Structure
https://pubchem.ncbi.nlm.nih.gov/compound/85835744#section=2D-Structure
https://pubchem.ncbi.nlm.nih.gov/compound/85826760#section=2D-Structure
https://pubchem.ncbi.nlm.nih.gov/compound/85817672#section=2D-Structure
https://pubchem.ncbi.nlm.nih.gov/compound/85815308#section=2D-Structure
https://pubchem.ncbi.nlm.nih.gov/compound/85815244#section=2D-Structure
https://pubchem.ncbi.nlm.nih.gov/compound/85809719#section=2D-Structure
https://pubchem.ncbi.nlm.nih.gov/compound/85809093#section=2D-Structure
https://pubchem.ncbi.nlm.nih.gov/compound/85807289#section=2D-Structure
https://pubchem.ncbi.nlm.nih.gov/compound/85807279#section=2D-Structure
https://pubchem.ncbi.nlm.nih.gov/compound/85806567#section=2D-Structure
https://pubchem.ncbi.nlm.nih.gov/compound/85803566#section=2D-Structure
https://pubchem.ncbi.nlm.nih.gov/compound/85802691#section=2D-Structure
https://pubchem.ncbi.nlm.nih.gov/compound/85800532#section=2D-Structure
https://pubchem.ncbi.nlm.nih.gov/compound/85800529#section=2D-Structure
https://pubchem.ncbi.nlm.nih.gov/compound/85800527#section=2D-Structure
https://pubchem.ncbi.nlm.nih.gov/compound/85800526#section=2D-Structure
https://pubchem.ncbi.nlm.nih.gov/compound/85787285#section=2D-Structure
https://pubchem.ncbi.nlm.nih.gov/compound/85784039#section=2D-Structure
https://pubchem.ncbi.nlm.nih.gov/compound/85778609#section=2D-Structure
https://pubchem.ncbi.nlm.nih.gov/compound/85776993#section=2D-Structure
https://pubchem.ncbi.nlm.nih.gov/compound/85776396#section=2D-Structure
https://pubchem.ncbi.nlm.nih.gov/compound/85773529#section=2D-Structure
https://pubchem.ncbi.nlm.nih.gov/compound/85765792#section=2D-Structure
https://pubchem.ncbi.nlm.nih.gov/compound/85759582#section=2D-Structure
https://pubchem.ncbi.nlm.nih.gov/compound/85756169#section=2D-Structure
https://pubchem.ncbi.nlm.nih.gov/compound/85755813#section=2D-Structure
https://pubchem.ncbi.nlm.nih.gov/compound/85751975#section=2D-Structure
https://pubchem.ncbi.nlm.nih.gov/compound/85743743#section=2D-Structure
https://pubchem.ncbi.nlm.nih.gov/compound/85719967#section=2D-Structure
https://pubchem.ncbi.nlm.nih.gov/compound/85719964#section=2D-Structure
https://pubchem.ncbi.nlm.nih.gov/compound/85718311#section=2D-Structure
https://pubchem.ncbi.nlm.nih.gov/compound/85702113#section=2D-Structure
https://pubchem.ncbi.nlm.nih.gov/compound/85700475#section=2D-Structure
https://pubchem.ncbi.nlm.nih.gov/compound/85693968#section=2D-Structure
https://pubchem.ncbi.nlm.nih.gov/compound/85655501#section=2D-Structure
https://pubchem.ncbi.nlm.nih.gov/compound/85640925#section=2D-Structure
https://pubchem.ncbi.nlm.nih.gov/compound/85629914#section=2D-Structure

https://pubchem.ncbi.nlm.nih.gov/compound/85624118#section=2D-Structure
https://pubchem.ncbi.nlm.nih.gov/compound/85618745#section=2D-Structure
https://pubchem.ncbi.nlm.nih.gov/compound/856152#section=2D-Structure
https://pubchem.ncbi.nlm.nih.gov/compound/85612865#section=2D-Structure
https://pubchem.ncbi.nlm.nih.gov/compound/85610296#section=2D-Structure
https://pubchem.ncbi.nlm.nih.gov/compound/85609101#section=2D-Structure
https://pubchem.ncbi.nlm.nih.gov/compound/85598040#section=2D-Structure
https://pubchem.ncbi.nlm.nih.gov/compound/85580855#section=2D-Structure
https://pubchem.ncbi.nlm.nih.gov/compound/85565814#section=2D-Structure
https://pubchem.ncbi.nlm.nih.gov/compound/85565408#section=2D-Structure
https://pubchem.ncbi.nlm.nih.gov/compound/85563143#section=2D-Structure
https://pubchem.ncbi.nlm.nih.gov/compound/85533457#section=2D-Structure
https://pubchem.ncbi.nlm.nih.gov/compound/85527993#section=2D-Structure
https://pubchem.ncbi.nlm.nih.gov/compound/85526211#section=2D-Structure
https://pubchem.ncbi.nlm.nih.gov/compound/85525283#section=2D-Structure
https://pubchem.ncbi.nlm.nih.gov/compound/85388#section=2D-Structure
https://pubchem.ncbi.nlm.nih.gov/compound/85387#section=2D-Structure
https://pubchem.ncbi.nlm.nih.gov/compound/85364#section=2D-Structure
https://pubchem.ncbi.nlm.nih.gov/compound/85358#section=2D-Structure
https://pubchem.ncbi.nlm.nih.gov/compound/85280093#section=2D-Structure
https://pubchem.ncbi.nlm.nih.gov/compound/85168619#section=2D-Structure
https://pubchem.ncbi.nlm.nih.gov/compound/851060#section=2D-Structure
https://pubchem.ncbi.nlm.nih.gov/compound/849498#section=2D-Structure
https://pubchem.ncbi.nlm.nih.gov/compound/8418#section=2D-Structure
https://pubchem.ncbi.nlm.nih.gov/compound/82687#section=2D-Structure
https://pubchem.ncbi.nlm.nih.gov/compound/821508#section=2D-Structure
https://pubchem.ncbi.nlm.nih.gov/compound/82143#section=2D-Structure
https://pubchem.ncbi.nlm.nih.gov/compound/81992#section=2D-Structure
https://pubchem.ncbi.nlm.nih.gov/compound/81271#section=2D-Structure
https://pubchem.ncbi.nlm.nih.gov/compound/799128#section=2D-Structure
https://pubchem.ncbi.nlm.nih.gov/compound/78388402#section=2D-Structure
https://pubchem.ncbi.nlm.nih.gov/compound/78303945#section=2D-Structure
https://pubchem.ncbi.nlm.nih.gov/compound/780996#section=2D-Structure
https://pubchem.ncbi.nlm.nih.gov/compound/77994132#section=2D-Structure
https://pubchem.ncbi.nlm.nih.gov/compound/77620847#section=2D-Structure
https://pubchem.ncbi.nlm.nih.gov/compound/77519122#section=2D-Structure
https://pubchem.ncbi.nlm.nih.gov/compound/76643388#section=2D-Structure
https://pubchem.ncbi.nlm.nih.gov/compound/76626519#section=2D-Structure
https://pubchem.ncbi.nlm.nih.gov/compound/76601692#section=2D-Structure
https://pubchem.ncbi.nlm.nih.gov/compound/76601658#section=2D-Structure
https://pubchem.ncbi.nlm.nih.gov/compound/76574#section=2D-Structure
https://pubchem.ncbi.nlm.nih.gov/compound/76155727#section=2D-Structure
https://pubchem.ncbi.nlm.nih.gov/compound/76139#section=2D-Structure
https://pubchem.ncbi.nlm.nih.gov/compound/76138#section=2D-Structure
https://pubchem.ncbi.nlm.nih.gov/compound/75595901#section=2D-Structure
https://pubchem.ncbi.nlm.nih.gov/compound/74841#section=2D-Structure
https://pubchem.ncbi.nlm.nih.gov/compound/74223648#section=2D-Structure
https://pubchem.ncbi.nlm.nih.gov/compound/74047702#section=2D-Structure
https://pubchem.ncbi.nlm.nih.gov/compound/74046952#section=2D-Structure
https://pubchem.ncbi.nlm.nih.gov/compound/74037981#section=2D-Structure
https://pubchem.ncbi.nlm.nih.gov/compound/74036397#section=2D-Structure
https://pubchem.ncbi.nlm.nih.gov/compound/739715#section=2D-Structure
https://pubchem.ncbi.nlm.nih.gov/compound/73949147#section=2D-Structure
https://pubchem.ncbi.nlm.nih.gov/compound/73891496#section=2D-Structure
https://pubchem.ncbi.nlm.nih.gov/compound/73707662#section=2D-Structure

https://pubchem.ncbi.nlm.nih.gov/compound/73699497#section=2D-Structure
https://pubchem.ncbi.nlm.nih.gov/compound/73294076#section=2D-Structure
https://pubchem.ncbi.nlm.nih.gov/compound/73294075#section=2D-Structure
https://pubchem.ncbi.nlm.nih.gov/compound/73291953#section=2D-Structure
https://pubchem.ncbi.nlm.nih.gov/compound/73212330#section=2D-Structure
https://pubchem.ncbi.nlm.nih.gov/compound/73212253#section=2D-Structure
https://pubchem.ncbi.nlm.nih.gov/compound/72770#section=2D-Structure
https://pubchem.ncbi.nlm.nih.gov/compound/72715156#section=2D-Structure
https://pubchem.ncbi.nlm.nih.gov/compound/72715155#section=2D-Structure
https://pubchem.ncbi.nlm.nih.gov/compound/72715062#section=2D-Structure
https://pubchem.ncbi.nlm.nih.gov/compound/72714970#section=2D-Structure
https://pubchem.ncbi.nlm.nih.gov/compound/72649332#section=2D-Structure
https://pubchem.ncbi.nlm.nih.gov/compound/72603498#section=2D-Structure
https://pubchem.ncbi.nlm.nih.gov/compound/72603497#section=2D-Structure
https://pubchem.ncbi.nlm.nih.gov/compound/72603494#section=2D-Structure
https://pubchem.ncbi.nlm.nih.gov/compound/72449799#section=2D-Structure
https://pubchem.ncbi.nlm.nih.gov/compound/72390434#section=2D-Structure
https://pubchem.ncbi.nlm.nih.gov/compound/72279867#section=2D-Structure
https://pubchem.ncbi.nlm.nih.gov/compound/72258099#section=2D-Structure
https://pubchem.ncbi.nlm.nih.gov/compound/71775319#section=2D-Structure
https://pubchem.ncbi.nlm.nih.gov/compound/71752416#section=2D-Structure
https://pubchem.ncbi.nlm.nih.gov/compound/71724703#section=2D-Structure
https://pubchem.ncbi.nlm.nih.gov/compound/71724701#section=2D-Structure
https://pubchem.ncbi.nlm.nih.gov/compound/71724669#section=2D-Structure
https://pubchem.ncbi.nlm.nih.gov/compound/71724668#section=2D-Structure
https://pubchem.ncbi.nlm.nih.gov/compound/71723446#section=2D-Structure
https://pubchem.ncbi.nlm.nih.gov/compound/71723443#section=2D-Structure
https://pubchem.ncbi.nlm.nih.gov/compound/71723297#section=2D-Structure
https://pubchem.ncbi.nlm.nih.gov/compound/71696710#section=2D-Structure
https://pubchem.ncbi.nlm.nih.gov/compound/71538123#section=2D-Structure
https://pubchem.ncbi.nlm.nih.gov/compound/71535894#section=2D-Structure
https://pubchem.ncbi.nlm.nih.gov/compound/71512357#section=2D-Structure
https://pubchem.ncbi.nlm.nih.gov/compound/71512145#section=2D-Structure
https://pubchem.ncbi.nlm.nih.gov/compound/71512144#section=2D-Structure
https://pubchem.ncbi.nlm.nih.gov/compound/71484403#section=2D-Structure
https://pubchem.ncbi.nlm.nih.gov/compound/71446893#section=2D-Structure
https://pubchem.ncbi.nlm.nih.gov/compound/71446600#section=2D-Structure
https://pubchem.ncbi.nlm.nih.gov/compound/71440296#section=2D-Structure
https://pubchem.ncbi.nlm.nih.gov/compound/71440137#section=2D-Structure
https://pubchem.ncbi.nlm.nih.gov/compound/71436416#section=2D-Structure
https://pubchem.ncbi.nlm.nih.gov/compound/71436161#section=2D-Structure
https://pubchem.ncbi.nlm.nih.gov/compound/71436083#section=2D-Structure
https://pubchem.ncbi.nlm.nih.gov/compound/71425434#section=2D-Structure
https://pubchem.ncbi.nlm.nih.gov/compound/71421267#section=2D-Structure
https://pubchem.ncbi.nlm.nih.gov/compound/71420917#section=2D-Structure
https://pubchem.ncbi.nlm.nih.gov/compound/71420915#section=2D-Structure
https://pubchem.ncbi.nlm.nih.gov/compound/71419663#section=2D-Structure
https://pubchem.ncbi.nlm.nih.gov/compound/71406476#section=2D-Structure
https://pubchem.ncbi.nlm.nih.gov/compound/71406287#section=2D-Structure
https://pubchem.ncbi.nlm.nih.gov/compound/71405769#section=2D-Structure
https://pubchem.ncbi.nlm.nih.gov/compound/71405165#section=2D-Structure
https://pubchem.ncbi.nlm.nih.gov/compound/71404652#section=2D-Structure
https://pubchem.ncbi.nlm.nih.gov/compound/71401864#section=2D-Structure
https://pubchem.ncbi.nlm.nih.gov/compound/71401355#section=2D-Structure
https://pubchem.ncbi.nlm.nih.gov/compound/71397460#section=2D-Structure

https://pubchem.ncbi.nlm.nih.gov/compound/71380417#section=2D-Structure
https://pubchem.ncbi.nlm.nih.gov/compound/71376357#section=2D-Structure
https://pubchem.ncbi.nlm.nih.gov/compound/71374977#section=2D-Structure
https://pubchem.ncbi.nlm.nih.gov/compound/71371690#section=2D-Structure
https://pubchem.ncbi.nlm.nih.gov/compound/71371518#section=2D-Structure
https://pubchem.ncbi.nlm.nih.gov/compound/71371099#section=2D-Structure
https://pubchem.ncbi.nlm.nih.gov/compound/71362136#section=2D-Structure
https://pubchem.ncbi.nlm.nih.gov/compound/71360152#section=2D-Structure
https://pubchem.ncbi.nlm.nih.gov/compound/71359095#section=2D-Structure
https://pubchem.ncbi.nlm.nih.gov/compound/71359071#section=2D-Structure
https://pubchem.ncbi.nlm.nih.gov/compound/71359023#section=2D-Structure
https://pubchem.ncbi.nlm.nih.gov/compound/71358961#section=2D-Structure
https://pubchem.ncbi.nlm.nih.gov/compound/71358934#section=2D-Structure
https://pubchem.ncbi.nlm.nih.gov/compound/71358931#section=2D-Structure
https://pubchem.ncbi.nlm.nih.gov/compound/71358886#section=2D-Structure
https://pubchem.ncbi.nlm.nih.gov/compound/71358731#section=2D-Structure
https://pubchem.ncbi.nlm.nih.gov/compound/71358710#section=2D-Structure
https://pubchem.ncbi.nlm.nih.gov/compound/71358647#section=2D-Structure
https://pubchem.ncbi.nlm.nih.gov/compound/71358618#section=2D-Structure
https://pubchem.ncbi.nlm.nih.gov/compound/71358609#section=2D-Structure
https://pubchem.ncbi.nlm.nih.gov/compound/71355924#section=2D-Structure
https://pubchem.ncbi.nlm.nih.gov/compound/71348814#section=2D-Structure
https://pubchem.ncbi.nlm.nih.gov/compound/71347422#section=2D-Structure
https://pubchem.ncbi.nlm.nih.gov/compound/71345869#section=2D-Structure
https://pubchem.ncbi.nlm.nih.gov/compound/71345321#section=2D-Structure
https://pubchem.ncbi.nlm.nih.gov/compound/71344945#section=2D-Structure
https://pubchem.ncbi.nlm.nih.gov/compound/71343241#section=2D-Structure
https://pubchem.ncbi.nlm.nih.gov/compound/71342942#section=2D-Structure
https://pubchem.ncbi.nlm.nih.gov/compound/71340033#section=2D-Structure
https://pubchem.ncbi.nlm.nih.gov/compound/71338329#section=2D-Structure
https://pubchem.ncbi.nlm.nih.gov/compound/71336750#section=2D-Structure
https://pubchem.ncbi.nlm.nih.gov/compound/71336149#section=2D-Structure
https://pubchem.ncbi.nlm.nih.gov/compound/71335616#section=2D-Structure
https://pubchem.ncbi.nlm.nih.gov/compound/71335262#section=2D-Structure
https://pubchem.ncbi.nlm.nih.gov/compound/71333856#section=2D-Structure
https://pubchem.ncbi.nlm.nih.gov/compound/71333820#section=2D-Structure
https://pubchem.ncbi.nlm.nih.gov/compound/71329949#section=2D-Structure
https://pubchem.ncbi.nlm.nih.gov/compound/71329658#section=2D-Structure
https://pubchem.ncbi.nlm.nih.gov/compound/71326808#section=2D-Structure
https://pubchem.ncbi.nlm.nih.gov/compound/71325161#section=2D-Structure
https://pubchem.ncbi.nlm.nih.gov/compound/71323683#section=2D-Structure
https://pubchem.ncbi.nlm.nih.gov/compound/71306948#section=2D-Structure
https://pubchem.ncbi.nlm.nih.gov/compound/71261419#section=2D-Structure
https://pubchem.ncbi.nlm.nih.gov/compound/71261415#section=2D-Structure
https://pubchem.ncbi.nlm.nih.gov/compound/71261414#section=2D-Structure
https://pubchem.ncbi.nlm.nih.gov/compound/71246288#section=2D-Structure
https://pubchem.ncbi.nlm.nih.gov/compound/71222258#section=2D-Structure
https://pubchem.ncbi.nlm.nih.gov/compound/71222255#section=2D-Structure
https://pubchem.ncbi.nlm.nih.gov/compound/71222251#section=2D-Structure
https://pubchem.ncbi.nlm.nih.gov/compound/71197510#section=2D-Structure
https://pubchem.ncbi.nlm.nih.gov/compound/71195541#section=2D-Structure
https://pubchem.ncbi.nlm.nih.gov/compound/71192122#section=2D-Structure
https://pubchem.ncbi.nlm.nih.gov/compound/71190464#section=2D-Structure
https://pubchem.ncbi.nlm.nih.gov/compound/71187763#section=2D-Structure
https://pubchem.ncbi.nlm.nih.gov/compound/71175557#section=2D-Structure

https://pubchem.ncbi.nlm.nih.gov/compound/71166123#section=2D-Structure
https://pubchem.ncbi.nlm.nih.gov/compound/71142861#section=2D-Structure
https://pubchem.ncbi.nlm.nih.gov/compound/71132081#section=2D-Structure
https://pubchem.ncbi.nlm.nih.gov/compound/71128497#section=2D-Structure
https://pubchem.ncbi.nlm.nih.gov/compound/71089280#section=2D-Structure
https://pubchem.ncbi.nlm.nih.gov/compound/71088544#section=2D-Structure
https://pubchem.ncbi.nlm.nih.gov/compound/71073311#section=2D-Structure
https://pubchem.ncbi.nlm.nih.gov/compound/71054485#section=2D-Structure
https://pubchem.ncbi.nlm.nih.gov/compound/71052843#section=2D-Structure
https://pubchem.ncbi.nlm.nih.gov/compound/71035#section=2D-Structure
https://pubchem.ncbi.nlm.nih.gov/compound/71009724#section=2D-Structure
https://pubchem.ncbi.nlm.nih.gov/compound/70999999#section=2D-Structure
https://pubchem.ncbi.nlm.nih.gov/compound/70992345#section=2D-Structure
https://pubchem.ncbi.nlm.nih.gov/compound/70963415#section=2D-Structure
https://pubchem.ncbi.nlm.nih.gov/compound/70957951#section=2D-Structure
https://pubchem.ncbi.nlm.nih.gov/compound/70957910#section=2D-Structure
https://pubchem.ncbi.nlm.nih.gov/compound/70957863#section=2D-Structure
https://pubchem.ncbi.nlm.nih.gov/compound/70957861#section=2D-Structure
https://pubchem.ncbi.nlm.nih.gov/compound/70904454#section=2D-Structure
https://pubchem.ncbi.nlm.nih.gov/compound/70882730#section=2D-Structure
https://pubchem.ncbi.nlm.nih.gov/compound/70865182#section=2D-Structure
https://pubchem.ncbi.nlm.nih.gov/compound/70823189#section=2D-Structure
https://pubchem.ncbi.nlm.nih.gov/compound/70823156#section=2D-Structure
https://pubchem.ncbi.nlm.nih.gov/compound/70810982#section=2D-Structure
https://pubchem.ncbi.nlm.nih.gov/compound/7080#section=2D-Structure
https://pubchem.ncbi.nlm.nih.gov/compound/70651784#section=2D-Structure
https://pubchem.ncbi.nlm.nih.gov/compound/70649325#section=2D-Structure
https://pubchem.ncbi.nlm.nih.gov/compound/70646713#section=2D-Structure
https://pubchem.ncbi.nlm.nih.gov/compound/70641450#section=2D-Structure
https://pubchem.ncbi.nlm.nih.gov/compound/70633261#section=2D-Structure
https://pubchem.ncbi.nlm.nih.gov/compound/70633100#section=2D-Structure
https://pubchem.ncbi.nlm.nih.gov/compound/70632886#section=2D-Structure
https://pubchem.ncbi.nlm.nih.gov/compound/70603918#section=2D-Structure
https://pubchem.ncbi.nlm.nih.gov/compound/70597236#section=2D-Structure
https://pubchem.ncbi.nlm.nih.gov/compound/70587086#section=2D-Structure
https://pubchem.ncbi.nlm.nih.gov/compound/70580370#section=2D-Structure
https://pubchem.ncbi.nlm.nih.gov/compound/70567817#section=2D-Structure
https://pubchem.ncbi.nlm.nih.gov/compound/70566310#section=2D-Structure
https://pubchem.ncbi.nlm.nih.gov/compound/70565817#section=2D-Structure
https://pubchem.ncbi.nlm.nih.gov/compound/70538671#section=2D-Structure
https://pubchem.ncbi.nlm.nih.gov/compound/70536460#section=2D-Structure
https://pubchem.ncbi.nlm.nih.gov/compound/70529340#section=2D-Structure
https://pubchem.ncbi.nlm.nih.gov/compound/70526210#section=2D-Structure
https://pubchem.ncbi.nlm.nih.gov/compound/70512121#section=2D-Structure
https://pubchem.ncbi.nlm.nih.gov/compound/70499049#section=2D-Structure
https://pubchem.ncbi.nlm.nih.gov/compound/70473368#section=2D-Structure
https://pubchem.ncbi.nlm.nih.gov/compound/70452495#section=2D-Structure
https://pubchem.ncbi.nlm.nih.gov/compound/70431198#section=2D-Structure
https://pubchem.ncbi.nlm.nih.gov/compound/70425774#section=2D-Structure
https://pubchem.ncbi.nlm.nih.gov/compound/70425378#section=2D-Structure
https://pubchem.ncbi.nlm.nih.gov/compound/70416310#section=2D-Structure
https://pubchem.ncbi.nlm.nih.gov/compound/70404923#section=2D-Structure
https://pubchem.ncbi.nlm.nih.gov/compound/70390557#section=2D-Structure
https://pubchem.ncbi.nlm.nih.gov/compound/70389894#section=2D-Structure
https://pubchem.ncbi.nlm.nih.gov/compound/70383753#section=2D-Structure

https://pubchem.ncbi.nlm.nih.gov/compound/70380968#section=2D-Structure
https://pubchem.ncbi.nlm.nih.gov/compound/70362451#section=2D-Structure
https://pubchem.ncbi.nlm.nih.gov/compound/70361885#section=2D-Structure
https://pubchem.ncbi.nlm.nih.gov/compound/70356687#section=2D-Structure
https://pubchem.ncbi.nlm.nih.gov/compound/70334318#section=2D-Structure
https://pubchem.ncbi.nlm.nih.gov/compound/70299775#section=2D-Structure
https://pubchem.ncbi.nlm.nih.gov/compound/70296229#section=2D-Structure
https://pubchem.ncbi.nlm.nih.gov/compound/70293427#section=2D-Structure
https://pubchem.ncbi.nlm.nih.gov/compound/70293417#section=2D-Structure
https://pubchem.ncbi.nlm.nih.gov/compound/70284237#section=2D-Structure
https://pubchem.ncbi.nlm.nih.gov/compound/70283778#section=2D-Structure
https://pubchem.ncbi.nlm.nih.gov/compound/70283776#section=2D-Structure
https://pubchem.ncbi.nlm.nih.gov/compound/70240864#section=2D-Structure
https://pubchem.ncbi.nlm.nih.gov/compound/70234122#section=2D-Structure
https://pubchem.ncbi.nlm.nih.gov/compound/70225127#section=2D-Structure
https://pubchem.ncbi.nlm.nih.gov/compound/70202270#section=2D-Structure
https://pubchem.ncbi.nlm.nih.gov/compound/70182485#section=2D-Structure
https://pubchem.ncbi.nlm.nih.gov/compound/70178652#section=2D-Structure
https://pubchem.ncbi.nlm.nih.gov/compound/70162302#section=2D-Structure
https://pubchem.ncbi.nlm.nih.gov/compound/70159821#section=2D-Structure
https://pubchem.ncbi.nlm.nih.gov/compound/70117138#section=2D-Structure
https://pubchem.ncbi.nlm.nih.gov/compound/70071877#section=2D-Structure
https://pubchem.ncbi.nlm.nih.gov/compound/69996782#section=2D-Structure
https://pubchem.ncbi.nlm.nih.gov/compound/69994617#section=2D-Structure
https://pubchem.ncbi.nlm.nih.gov/compound/69989147#section=2D-Structure
https://pubchem.ncbi.nlm.nih.gov/compound/69982498#section=2D-Structure
https://pubchem.ncbi.nlm.nih.gov/compound/69963758#section=2D-Structure
https://pubchem.ncbi.nlm.nih.gov/compound/69919445#section=2D-Structure
https://pubchem.ncbi.nlm.nih.gov/compound/69852481#section=2D-Structure
https://pubchem.ncbi.nlm.nih.gov/compound/69826808#section=2D-Structure
https://pubchem.ncbi.nlm.nih.gov/compound/69817173#section=2D-Structure
https://pubchem.ncbi.nlm.nih.gov/compound/69807665#section=2D-Structure
https://pubchem.ncbi.nlm.nih.gov/compound/69780305#section=2D-Structure
https://pubchem.ncbi.nlm.nih.gov/compound/69774914#section=2D-Structure
https://pubchem.ncbi.nlm.nih.gov/compound/69736534#section=2D-Structure
https://pubchem.ncbi.nlm.nih.gov/compound/69724737#section=2D-Structure
https://pubchem.ncbi.nlm.nih.gov/compound/69715280#section=2D-Structure
https://pubchem.ncbi.nlm.nih.gov/compound/69699125#section=2D-Structure
https://pubchem.ncbi.nlm.nih.gov/compound/69652923#section=2D-Structure
https://pubchem.ncbi.nlm.nih.gov/compound/69642493#section=2D-Structure
https://pubchem.ncbi.nlm.nih.gov/compound/69631322#section=2D-Structure
https://pubchem.ncbi.nlm.nih.gov/compound/69625552#section=2D-Structure
https://pubchem.ncbi.nlm.nih.gov/compound/69592425#section=2D-Structure
https://pubchem.ncbi.nlm.nih.gov/compound/69543578#section=2D-Structure
https://pubchem.ncbi.nlm.nih.gov/compound/69478155#section=2D-Structure
https://pubchem.ncbi.nlm.nih.gov/compound/69474724#section=2D-Structure
https://pubchem.ncbi.nlm.nih.gov/compound/69444045#section=2D-Structure
https://pubchem.ncbi.nlm.nih.gov/compound/69413164#section=2D-Structure
https://pubchem.ncbi.nlm.nih.gov/compound/69413162#section=2D-Structure
https://pubchem.ncbi.nlm.nih.gov/compound/69412698#section=2D-Structure
https://pubchem.ncbi.nlm.nih.gov/compound/69398398#section=2D-Structure
https://pubchem.ncbi.nlm.nih.gov/compound/69373814#section=2D-Structure
https://pubchem.ncbi.nlm.nih.gov/compound/69368350#section=2D-Structure
https://pubchem.ncbi.nlm.nih.gov/compound/69363200#section=2D-Structure
https://pubchem.ncbi.nlm.nih.gov/compound/69353364#section=2D-Structure

https://pubchem.ncbi.nlm.nih.gov/compound/69345033#section=2D-Structure
https://pubchem.ncbi.nlm.nih.gov/compound/69312384#section=2D-Structure
https://pubchem.ncbi.nlm.nih.gov/compound/69276035#section=2D-Structure
https://pubchem.ncbi.nlm.nih.gov/compound/69267335#section=2D-Structure
https://pubchem.ncbi.nlm.nih.gov/compound/69264958#section=2D-Structure
https://pubchem.ncbi.nlm.nih.gov/compound/69216356#section=2D-Structure
https://pubchem.ncbi.nlm.nih.gov/compound/69198813#section=2D-Structure
https://pubchem.ncbi.nlm.nih.gov/compound/69194986#section=2D-Structure
https://pubchem.ncbi.nlm.nih.gov/compound/69192844#section=2D-Structure
https://pubchem.ncbi.nlm.nih.gov/compound/69188674#section=2D-Structure
https://pubchem.ncbi.nlm.nih.gov/compound/69170#section=2D-Structure
https://pubchem.ncbi.nlm.nih.gov/compound/6916872#section=2D-Structure
https://pubchem.ncbi.nlm.nih.gov/compound/69069032#section=2D-Structure
https://pubchem.ncbi.nlm.nih.gov/compound/69065159#section=2D-Structure
https://pubchem.ncbi.nlm.nih.gov/compound/69010754#section=2D-Structure
https://pubchem.ncbi.nlm.nih.gov/compound/69003983#section=2D-Structure
https://pubchem.ncbi.nlm.nih.gov/compound/68973634#section=2D-Structure
https://pubchem.ncbi.nlm.nih.gov/compound/68962170#section=2D-Structure
https://pubchem.ncbi.nlm.nih.gov/compound/68941607#section=2D-Structure
https://pubchem.ncbi.nlm.nih.gov/compound/68941522#section=2D-Structure
https://pubchem.ncbi.nlm.nih.gov/compound/68927547#section=2D-Structure
https://pubchem.ncbi.nlm.nih.gov/compound/68910860#section=2D-Structure
https://pubchem.ncbi.nlm.nih.gov/compound/68901209#section=2D-Structure
https://pubchem.ncbi.nlm.nih.gov/compound/68891890#section=2D-Structure
https://pubchem.ncbi.nlm.nih.gov/compound/68886568#section=2D-Structure
https://pubchem.ncbi.nlm.nih.gov/compound/68850773#section=2D-Structure
https://pubchem.ncbi.nlm.nih.gov/compound/68826518#section=2D-Structure
https://pubchem.ncbi.nlm.nih.gov/compound/68825512#section=2D-Structure
https://pubchem.ncbi.nlm.nih.gov/compound/68823094#section=2D-Structure
https://pubchem.ncbi.nlm.nih.gov/compound/68747259#section=2D-Structure
https://pubchem.ncbi.nlm.nih.gov/compound/68685997#section=2D-Structure
https://pubchem.ncbi.nlm.nih.gov/compound/68656790#section=2D-Structure
https://pubchem.ncbi.nlm.nih.gov/compound/68656730#section=2D-Structure
https://pubchem.ncbi.nlm.nih.gov/compound/68633111#section=2D-Structure
https://pubchem.ncbi.nlm.nih.gov/compound/68604400#section=2D-Structure
https://pubchem.ncbi.nlm.nih.gov/compound/68586527#section=2D-Structure
https://pubchem.ncbi.nlm.nih.gov/compound/68586140#section=2D-Structure
https://pubchem.ncbi.nlm.nih.gov/compound/68586035#section=2D-Structure
https://pubchem.ncbi.nlm.nih.gov/compound/68582924#section=2D-Structure
https://pubchem.ncbi.nlm.nih.gov/compound/68581881#section=2D-Structure
https://pubchem.ncbi.nlm.nih.gov/compound/68574858#section=2D-Structure
https://pubchem.ncbi.nlm.nih.gov/compound/68574526#section=2D-Structure
https://pubchem.ncbi.nlm.nih.gov/compound/68562853#section=2D-Structure
https://pubchem.ncbi.nlm.nih.gov/compound/68559498#section=2D-Structure
https://pubchem.ncbi.nlm.nih.gov/compound/68545588#section=2D-Structure
https://pubchem.ncbi.nlm.nih.gov/compound/68493774#section=2D-Structure
https://pubchem.ncbi.nlm.nih.gov/compound/68479454#section=2D-Structure
https://pubchem.ncbi.nlm.nih.gov/compound/68479453#section=2D-Structure
https://pubchem.ncbi.nlm.nih.gov/compound/68427716#section=2D-Structure
https://pubchem.ncbi.nlm.nih.gov/compound/68422300#section=2D-Structure
https://pubchem.ncbi.nlm.nih.gov/compound/68416077#section=2D-Structure
https://pubchem.ncbi.nlm.nih.gov/compound/68376941#section=2D-Structure
https://pubchem.ncbi.nlm.nih.gov/compound/68339090#section=2D-Structure
https://pubchem.ncbi.nlm.nih.gov/compound/68251485#section=2D-Structure
https://pubchem.ncbi.nlm.nih.gov/compound/68251473#section=2D-Structure

https://pubchem.ncbi.nlm.nih.gov/compound/68251225#section=2D-Structure
https://pubchem.ncbi.nlm.nih.gov/compound/68251204#section=2D-Structure
https://pubchem.ncbi.nlm.nih.gov/compound/68251191#section=2D-Structure
https://pubchem.ncbi.nlm.nih.gov/compound/68242428#section=2D-Structure
https://pubchem.ncbi.nlm.nih.gov/compound/68227487#section=2D-Structure
https://pubchem.ncbi.nlm.nih.gov/compound/68214839#section=2D-Structure
https://pubchem.ncbi.nlm.nih.gov/compound/68205880#section=2D-Structure
https://pubchem.ncbi.nlm.nih.gov/compound/68204894#section=2D-Structure
https://pubchem.ncbi.nlm.nih.gov/compound/68203732#section=2D-Structure
https://pubchem.ncbi.nlm.nih.gov/compound/68178057#section=2D-Structure
https://pubchem.ncbi.nlm.nih.gov/compound/68170998#section=2D-Structure
https://pubchem.ncbi.nlm.nih.gov/compound/6817#section=2D-Structure
https://pubchem.ncbi.nlm.nih.gov/compound/68168344#section=2D-Structure
https://pubchem.ncbi.nlm.nih.gov/compound/68145978#section=2D-Structure
https://pubchem.ncbi.nlm.nih.gov/compound/68063#section=2D-Structure
https://pubchem.ncbi.nlm.nih.gov/compound/68040637#section=2D-Structure
https://pubchem.ncbi.nlm.nih.gov/compound/68024307#section=2D-Structure
https://pubchem.ncbi.nlm.nih.gov/compound/68023090#section=2D-Structure
https://pubchem.ncbi.nlm.nih.gov/compound/68017875#section=2D-Structure
https://pubchem.ncbi.nlm.nih.gov/compound/68017874#section=2D-Structure
https://pubchem.ncbi.nlm.nih.gov/compound/68012806#section=2D-Structure
https://pubchem.ncbi.nlm.nih.gov/compound/67997547#section=2D-Structure
https://pubchem.ncbi.nlm.nih.gov/compound/67993958#section=2D-Structure
https://pubchem.ncbi.nlm.nih.gov/compound/67993434#section=2D-Structure
https://pubchem.ncbi.nlm.nih.gov/compound/67993294#section=2D-Structure
https://pubchem.ncbi.nlm.nih.gov/compound/67975215#section=2D-Structure
https://pubchem.ncbi.nlm.nih.gov/compound/67946678#section=2D-Structure
https://pubchem.ncbi.nlm.nih.gov/compound/67939174#section=2D-Structure
https://pubchem.ncbi.nlm.nih.gov/compound/67938688#section=2D-Structure
https://pubchem.ncbi.nlm.nih.gov/compound/67917615#section=2D-Structure
https://pubchem.ncbi.nlm.nih.gov/compound/67917091#section=2D-Structure
https://pubchem.ncbi.nlm.nih.gov/compound/67915938#section=2D-Structure
https://pubchem.ncbi.nlm.nih.gov/compound/67910781#section=2D-Structure
https://pubchem.ncbi.nlm.nih.gov/compound/67901529#section=2D-Structure
https://pubchem.ncbi.nlm.nih.gov/compound/67877455#section=2D-Structure
https://pubchem.ncbi.nlm.nih.gov/compound/67858853#section=2D-Structure
https://pubchem.ncbi.nlm.nih.gov/compound/67857773#section=2D-Structure
https://pubchem.ncbi.nlm.nih.gov/compound/67856285#section=2D-Structure
https://pubchem.ncbi.nlm.nih.gov/compound/67840930#section=2D-Structure
https://pubchem.ncbi.nlm.nih.gov/compound/67811857#section=2D-Structure
https://pubchem.ncbi.nlm.nih.gov/compound/67802260#section=2D-Structure
https://pubchem.ncbi.nlm.nih.gov/compound/67779814#section=2D-Structure
https://pubchem.ncbi.nlm.nih.gov/compound/67766807#section=2D-Structure
https://pubchem.ncbi.nlm.nih.gov/compound/67725901#section=2D-Structure
https://pubchem.ncbi.nlm.nih.gov/compound/67725559#section=2D-Structure
https://pubchem.ncbi.nlm.nih.gov/compound/67725417#section=2D-Structure
https://pubchem.ncbi.nlm.nih.gov/compound/67718152#section=2D-Structure
https://pubchem.ncbi.nlm.nih.gov/compound/67713046#section=2D-Structure
https://pubchem.ncbi.nlm.nih.gov/compound/67683501#section=2D-Structure
https://pubchem.ncbi.nlm.nih.gov/compound/67673847#section=2D-Structure
https://pubchem.ncbi.nlm.nih.gov/compound/67662838#section=2D-Structure
https://pubchem.ncbi.nlm.nih.gov/compound/67654253#section=2D-Structure
https://pubchem.ncbi.nlm.nih.gov/compound/67597910#section=2D-Structure
https://pubchem.ncbi.nlm.nih.gov/compound/67596687#section=2D-Structure
https://pubchem.ncbi.nlm.nih.gov/compound/67594522#section=2D-Structure

https://pubchem.ncbi.nlm.nih.gov/compound/67579752#section=2D-Structure
https://pubchem.ncbi.nlm.nih.gov/compound/67579475#section=2D-Structure
https://pubchem.ncbi.nlm.nih.gov/compound/67547861#section=2D-Structure
https://pubchem.ncbi.nlm.nih.gov/compound/67538801#section=2D-Structure
https://pubchem.ncbi.nlm.nih.gov/compound/67513983#section=2D-Structure
https://pubchem.ncbi.nlm.nih.gov/compound/67503451#section=2D-Structure
https://pubchem.ncbi.nlm.nih.gov/compound/67487#section=2D-Structure
https://pubchem.ncbi.nlm.nih.gov/compound/67467290#section=2D-Structure
https://pubchem.ncbi.nlm.nih.gov/compound/67462#section=2D-Structure
https://pubchem.ncbi.nlm.nih.gov/compound/67461#section=2D-Structure
https://pubchem.ncbi.nlm.nih.gov/compound/67455#section=2D-Structure
https://pubchem.ncbi.nlm.nih.gov/compound/67454#section=2D-Structure
https://pubchem.ncbi.nlm.nih.gov/compound/67453#section=2D-Structure
https://pubchem.ncbi.nlm.nih.gov/compound/67431720#section=2D-Structure
https://pubchem.ncbi.nlm.nih.gov/compound/67409547#section=2D-Structure
https://pubchem.ncbi.nlm.nih.gov/compound/67399073#section=2D-Structure
https://pubchem.ncbi.nlm.nih.gov/compound/67352112#section=2D-Structure
https://pubchem.ncbi.nlm.nih.gov/compound/67330130#section=2D-Structure
https://pubchem.ncbi.nlm.nih.gov/compound/67260170#section=2D-Structure
https://pubchem.ncbi.nlm.nih.gov/compound/67259750#section=2D-Structure
https://pubchem.ncbi.nlm.nih.gov/compound/67258931#section=2D-Structure
https://pubchem.ncbi.nlm.nih.gov/compound/67258388#section=2D-Structure
https://pubchem.ncbi.nlm.nih.gov/compound/67244572#section=2D-Structure
https://pubchem.ncbi.nlm.nih.gov/compound/67243056#section=2D-Structure
https://pubchem.ncbi.nlm.nih.gov/compound/67242399#section=2D-Structure
https://pubchem.ncbi.nlm.nih.gov/compound/67240931#section=2D-Structure
https://pubchem.ncbi.nlm.nih.gov/compound/67240493#section=2D-Structure
https://pubchem.ncbi.nlm.nih.gov/compound/67182847#section=2D-Structure
https://pubchem.ncbi.nlm.nih.gov/compound/67182845#section=2D-Structure
https://pubchem.ncbi.nlm.nih.gov/compound/671258#section=2D-Structure
https://pubchem.ncbi.nlm.nih.gov/compound/67117305#section=2D-Structure
https://pubchem.ncbi.nlm.nih.gov/compound/67110097#section=2D-Structure
https://pubchem.ncbi.nlm.nih.gov/compound/67108287#section=2D-Structure
https://pubchem.ncbi.nlm.nih.gov/compound/67042596#section=2D-Structure
https://pubchem.ncbi.nlm.nih.gov/compound/66895203#section=2D-Structure
https://pubchem.ncbi.nlm.nih.gov/compound/66895202#section=2D-Structure
https://pubchem.ncbi.nlm.nih.gov/compound/66829051#section=2D-Structure
https://pubchem.ncbi.nlm.nih.gov/compound/66764701#section=2D-Structure
https://pubchem.ncbi.nlm.nih.gov/compound/66750694#section=2D-Structure
https://pubchem.ncbi.nlm.nih.gov/compound/66743343#section=2D-Structure
https://pubchem.ncbi.nlm.nih.gov/compound/66723768#section=2D-Structure
https://pubchem.ncbi.nlm.nih.gov/compound/66711702#section=2D-Structure
https://pubchem.ncbi.nlm.nih.gov/compound/66709631#section=2D-Structure
https://pubchem.ncbi.nlm.nih.gov/compound/66693253#section=2D-Structure
https://pubchem.ncbi.nlm.nih.gov/compound/66673992#section=2D-Structure
https://pubchem.ncbi.nlm.nih.gov/compound/66649713#section=2D-Structure
https://pubchem.ncbi.nlm.nih.gov/compound/66648896#section=2D-Structure
https://pubchem.ncbi.nlm.nih.gov/compound/66623628#section=2D-Structure
https://pubchem.ncbi.nlm.nih.gov/compound/66618960#section=2D-Structure
https://pubchem.ncbi.nlm.nih.gov/compound/66612494#section=2D-Structure
https://pubchem.ncbi.nlm.nih.gov/compound/66588786#section=2D-Structure
https://pubchem.ncbi.nlm.nih.gov/compound/66561952#section=2D-Structure
https://pubchem.ncbi.nlm.nih.gov/compound/66552883#section=2D-Structure
https://pubchem.ncbi.nlm.nih.gov/compound/6618499#section=2D-Structure
https://pubchem.ncbi.nlm.nih.gov/compound/6612188#section=2D-Structure

https://pubchem.ncbi.nlm.nih.gov/compound/659228#section=2D-Structure
https://pubchem.ncbi.nlm.nih.gov/compound/65730#section=2D-Structure
https://pubchem.ncbi.nlm.nih.gov/compound/65106#section=2D-Structure
https://pubchem.ncbi.nlm.nih.gov/compound/6455215#section=2D-Structure
https://pubchem.ncbi.nlm.nih.gov/compound/6455096#section=2D-Structure
https://pubchem.ncbi.nlm.nih.gov/compound/6454336#section=2D-Structure
https://pubchem.ncbi.nlm.nih.gov/compound/6453902#section=2D-Structure
https://pubchem.ncbi.nlm.nih.gov/compound/6453897#section=2D-Structure
https://pubchem.ncbi.nlm.nih.gov/compound/6453176#section=2D-Structure
https://pubchem.ncbi.nlm.nih.gov/compound/6452601#section=2D-Structure
https://pubchem.ncbi.nlm.nih.gov/compound/6452502#section=2D-Structure
https://pubchem.ncbi.nlm.nih.gov/compound/6451843#section=2D-Structure
https://pubchem.ncbi.nlm.nih.gov/compound/6451842#section=2D-Structure
https://pubchem.ncbi.nlm.nih.gov/compound/6451620#section=2D-Structure
https://pubchem.ncbi.nlm.nih.gov/compound/6451416#section=2D-Structure
https://pubchem.ncbi.nlm.nih.gov/compound/6451370#section=2D-Structure
https://pubchem.ncbi.nlm.nih.gov/compound/6451363#section=2D-Structure
https://pubchem.ncbi.nlm.nih.gov/compound/6432614#section=2D-Structure
https://pubchem.ncbi.nlm.nih.gov/compound/6431290#section=2D-Structure
https://pubchem.ncbi.nlm.nih.gov/compound/6431165#section=2D-Structure
https://pubchem.ncbi.nlm.nih.gov/compound/6428580#section=2D-Structure
https://pubchem.ncbi.nlm.nih.gov/compound/6428557#section=2D-Structure
https://pubchem.ncbi.nlm.nih.gov/compound/6428551#section=2D-Structure
https://pubchem.ncbi.nlm.nih.gov/compound/6428547#section=2D-Structure
https://pubchem.ncbi.nlm.nih.gov/compound/6428178#section=2D-Structure
https://pubchem.ncbi.nlm.nih.gov/compound/6427872#section=2D-Structure
https://pubchem.ncbi.nlm.nih.gov/compound/6427869#section=2D-Structure
https://pubchem.ncbi.nlm.nih.gov/compound/6416199#section=2D-Structure
https://pubchem.ncbi.nlm.nih.gov/compound/6399209#section=2D-Structure
https://pubchem.ncbi.nlm.nih.gov/compound/6395596#section=2D-Structure
https://pubchem.ncbi.nlm.nih.gov/compound/639472#section=2D-Structure
https://pubchem.ncbi.nlm.nih.gov/compound/638636#section=2D-Structure
https://pubchem.ncbi.nlm.nih.gov/compound/6325610#section=2D-Structure
https://pubchem.ncbi.nlm.nih.gov/compound/629650#section=2D-Structure
https://pubchem.ncbi.nlm.nih.gov/compound/629493#section=2D-Structure
https://pubchem.ncbi.nlm.nih.gov/compound/629492#section=2D-Structure
https://pubchem.ncbi.nlm.nih.gov/compound/629094#section=2D-Structure
https://pubchem.ncbi.nlm.nih.gov/compound/629093#section=2D-Structure
https://pubchem.ncbi.nlm.nih.gov/compound/629086#section=2D-Structure
https://pubchem.ncbi.nlm.nih.gov/compound/628135#section=2D-Structure
https://pubchem.ncbi.nlm.nih.gov/compound/62743#section=2D-Structure
https://pubchem.ncbi.nlm.nih.gov/compound/627429#section=2D-Structure
https://pubchem.ncbi.nlm.nih.gov/compound/626296#section=2D-Structure
https://pubchem.ncbi.nlm.nih.gov/compound/626012#section=2D-Structure
https://pubchem.ncbi.nlm.nih.gov/compound/625907#section=2D-Structure
https://pubchem.ncbi.nlm.nih.gov/compound/625888#section=2D-Structure
https://pubchem.ncbi.nlm.nih.gov/compound/624823#section=2D-Structure
https://pubchem.ncbi.nlm.nih.gov/compound/624635#section=2D-Structure
https://pubchem.ncbi.nlm.nih.gov/compound/624498#section=2D-Structure
https://pubchem.ncbi.nlm.nih.gov/compound/624490#section=2D-Structure
https://pubchem.ncbi.nlm.nih.gov/compound/624488#section=2D-Structure
https://pubchem.ncbi.nlm.nih.gov/compound/624352#section=2D-Structure
https://pubchem.ncbi.nlm.nih.gov/compound/622001#section=2D-Structure
https://pubchem.ncbi.nlm.nih.gov/compound/621380#section=2D-Structure
https://pubchem.ncbi.nlm.nih.gov/compound/621379#section=2D-Structure

https://pubchem.ncbi.nlm.nih.gov/compound/619007#section=2D-Structure
https://pubchem.ncbi.nlm.nih.gov/compound/618855#section=2D-Structure
https://pubchem.ncbi.nlm.nih.gov/compound/616679#section=2D-Structure
https://pubchem.ncbi.nlm.nih.gov/compound/615584#section=2D-Structure
https://pubchem.ncbi.nlm.nih.gov/compound/614239#section=2D-Structure
https://pubchem.ncbi.nlm.nih.gov/compound/613262#section=2D-Structure
https://pubchem.ncbi.nlm.nih.gov/compound/613183#section=2D-Structure
https://pubchem.ncbi.nlm.nih.gov/compound/611430#section=2D-Structure
https://pubchem.ncbi.nlm.nih.gov/compound/611395#section=2D-Structure
https://pubchem.ncbi.nlm.nih.gov/compound/611364#section=2D-Structure
https://pubchem.ncbi.nlm.nih.gov/compound/611177#section=2D-Structure
https://pubchem.ncbi.nlm.nih.gov/compound/610387#section=2D-Structure
https://pubchem.ncbi.nlm.nih.gov/compound/609490#section=2D-Structure
https://pubchem.ncbi.nlm.nih.gov/compound/609407#section=2D-Structure
https://pubchem.ncbi.nlm.nih.gov/compound/608855#section=2D-Structure
https://pubchem.ncbi.nlm.nih.gov/compound/608357#section=2D-Structure
https://pubchem.ncbi.nlm.nih.gov/compound/607255#section=2D-Structure
https://pubchem.ncbi.nlm.nih.gov/compound/606676#section=2D-Structure
https://pubchem.ncbi.nlm.nih.gov/compound/606380#section=2D-Structure
https://pubchem.ncbi.nlm.nih.gov/compound/606332#section=2D-Structure
https://pubchem.ncbi.nlm.nih.gov/compound/605519#section=2D-Structure
https://pubchem.ncbi.nlm.nih.gov/compound/601692#section=2D-Structure
https://pubchem.ncbi.nlm.nih.gov/compound/6015640#section=2D-Structure
https://pubchem.ncbi.nlm.nih.gov/compound/60147943#section=2D-Structure
https://pubchem.ncbi.nlm.nih.gov/compound/60147942#section=2D-Structure
https://pubchem.ncbi.nlm.nih.gov/compound/60147940#section=2D-Structure
https://pubchem.ncbi.nlm.nih.gov/compound/60139181#section=2D-Structure
https://pubchem.ncbi.nlm.nih.gov/compound/60139172#section=2D-Structure
https://pubchem.ncbi.nlm.nih.gov/compound/60138268#section=2D-Structure
https://pubchem.ncbi.nlm.nih.gov/compound/60134593#section=2D-Structure
https://pubchem.ncbi.nlm.nih.gov/compound/60134305#section=2D-Structure
https://pubchem.ncbi.nlm.nih.gov/compound/60134277#section=2D-Structure
https://pubchem.ncbi.nlm.nih.gov/compound/60134212#section=2D-Structure
https://pubchem.ncbi.nlm.nih.gov/compound/60134205#section=2D-Structure
https://pubchem.ncbi.nlm.nih.gov/compound/60134200#section=2D-Structure
https://pubchem.ncbi.nlm.nih.gov/compound/60134194#section=2D-Structure
https://pubchem.ncbi.nlm.nih.gov/compound/60134179#section=2D-Structure
https://pubchem.ncbi.nlm.nih.gov/compound/60134169#section=2D-Structure
https://pubchem.ncbi.nlm.nih.gov/compound/60116275#section=2D-Structure
https://pubchem.ncbi.nlm.nih.gov/compound/60116255#section=2D-Structure
https://pubchem.ncbi.nlm.nih.gov/compound/60114124#section=2D-Structure
https://pubchem.ncbi.nlm.nih.gov/compound/60106170#section=2D-Structure
https://pubchem.ncbi.nlm.nih.gov/compound/60106151#section=2D-Structure
https://pubchem.ncbi.nlm.nih.gov/compound/60105584#section=2D-Structure
https://pubchem.ncbi.nlm.nih.gov/compound/60103366#section=2D-Structure
https://pubchem.ncbi.nlm.nih.gov/compound/60101478#section=2D-Structure
https://pubchem.ncbi.nlm.nih.gov/compound/60093776#section=2D-Structure
https://pubchem.ncbi.nlm.nih.gov/compound/60092445#section=2D-Structure
https://pubchem.ncbi.nlm.nih.gov/compound/60055057#section=2D-Structure
https://pubchem.ncbi.nlm.nih.gov/compound/60042398#section=2D-Structure
https://pubchem.ncbi.nlm.nih.gov/compound/60042391#section=2D-Structure
https://pubchem.ncbi.nlm.nih.gov/compound/60042384#section=2D-Structure
https://pubchem.ncbi.nlm.nih.gov/compound/60042383#section=2D-Structure
https://pubchem.ncbi.nlm.nih.gov/compound/60042367#section=2D-Structure
https://pubchem.ncbi.nlm.nih.gov/compound/60042366#section=2D-Structure

https://pubchem.ncbi.nlm.nih.gov/compound/60042338#section=2D-Structure
https://pubchem.ncbi.nlm.nih.gov/compound/60042325#section=2D-Structure
https://pubchem.ncbi.nlm.nih.gov/compound/60042314#section=2D-Structure
https://pubchem.ncbi.nlm.nih.gov/compound/59994811#section=2D-Structure
https://pubchem.ncbi.nlm.nih.gov/compound/59979920#section=2D-Structure
https://pubchem.ncbi.nlm.nih.gov/compound/59946660#section=2D-Structure
https://pubchem.ncbi.nlm.nih.gov/compound/59946648#section=2D-Structure
https://pubchem.ncbi.nlm.nih.gov/compound/59941416#section=2D-Structure
https://pubchem.ncbi.nlm.nih.gov/compound/59941391#section=2D-Structure
https://pubchem.ncbi.nlm.nih.gov/compound/59941386#section=2D-Structure
https://pubchem.ncbi.nlm.nih.gov/compound/59931160#section=2D-Structure
https://pubchem.ncbi.nlm.nih.gov/compound/59916360#section=2D-Structure
https://pubchem.ncbi.nlm.nih.gov/compound/59916358#section=2D-Structure
https://pubchem.ncbi.nlm.nih.gov/compound/59916356#section=2D-Structure
https://pubchem.ncbi.nlm.nih.gov/compound/59898148#section=2D-Structure
https://pubchem.ncbi.nlm.nih.gov/compound/59882811#section=2D-Structure
https://pubchem.ncbi.nlm.nih.gov/compound/59882365#section=2D-Structure
https://pubchem.ncbi.nlm.nih.gov/compound/59881413#section=2D-Structure
https://pubchem.ncbi.nlm.nih.gov/compound/59866361#section=2D-Structure
https://pubchem.ncbi.nlm.nih.gov/compound/59863477#section=2D-Structure
https://pubchem.ncbi.nlm.nih.gov/compound/59859439#section=2D-Structure
https://pubchem.ncbi.nlm.nih.gov/compound/59858644#section=2D-Structure
https://pubchem.ncbi.nlm.nih.gov/compound/59854145#section=2D-Structure
https://pubchem.ncbi.nlm.nih.gov/compound/59832560#section=2D-Structure
https://pubchem.ncbi.nlm.nih.gov/compound/59832555#section=2D-Structure
https://pubchem.ncbi.nlm.nih.gov/compound/59832180#section=2D-Structure
https://pubchem.ncbi.nlm.nih.gov/compound/59831142#section=2D-Structure
https://pubchem.ncbi.nlm.nih.gov/compound/59831138#section=2D-Structure
https://pubchem.ncbi.nlm.nih.gov/compound/59811844#section=2D-Structure
https://pubchem.ncbi.nlm.nih.gov/compound/59811670#section=2D-Structure
https://pubchem.ncbi.nlm.nih.gov/compound/59798821#section=2D-Structure
https://pubchem.ncbi.nlm.nih.gov/compound/59787207#section=2D-Structure
https://pubchem.ncbi.nlm.nih.gov/compound/59775894#section=2D-Structure
https://pubchem.ncbi.nlm.nih.gov/compound/59775489#section=2D-Structure
https://pubchem.ncbi.nlm.nih.gov/compound/59773751#section=2D-Structure
https://pubchem.ncbi.nlm.nih.gov/compound/59771955#section=2D-Structure
https://pubchem.ncbi.nlm.nih.gov/compound/59757460#section=2D-Structure
https://pubchem.ncbi.nlm.nih.gov/compound/59747627#section=2D-Structure
https://pubchem.ncbi.nlm.nih.gov/compound/59742107#section=2D-Structure
https://pubchem.ncbi.nlm.nih.gov/compound/59742048#section=2D-Structure
https://pubchem.ncbi.nlm.nih.gov/compound/59742043#section=2D-Structure
https://pubchem.ncbi.nlm.nih.gov/compound/59742042#section=2D-Structure
https://pubchem.ncbi.nlm.nih.gov/compound/59742036#section=2D-Structure
https://pubchem.ncbi.nlm.nih.gov/compound/59742031#section=2D-Structure
https://pubchem.ncbi.nlm.nih.gov/compound/59742019#section=2D-Structure
https://pubchem.ncbi.nlm.nih.gov/compound/59742009#section=2D-Structure
https://pubchem.ncbi.nlm.nih.gov/compound/59741999#section=2D-Structure
https://pubchem.ncbi.nlm.nih.gov/compound/59741980#section=2D-Structure
https://pubchem.ncbi.nlm.nih.gov/compound/59741949#section=2D-Structure
https://pubchem.ncbi.nlm.nih.gov/compound/59741947#section=2D-Structure
https://pubchem.ncbi.nlm.nih.gov/compound/59741941#section=2D-Structure
https://pubchem.ncbi.nlm.nih.gov/compound/59722390#section=2D-Structure
https://pubchem.ncbi.nlm.nih.gov/compound/597208#section=2D-Structure
https://pubchem.ncbi.nlm.nih.gov/compound/59709513#section=2D-Structure
https://pubchem.ncbi.nlm.nih.gov/compound/59709511#section=2D-Structure

https://pubchem.ncbi.nlm.nih.gov/compound/59701935#section=2D-Structure
https://pubchem.ncbi.nlm.nih.gov/compound/59683893#section=2D-Structure
https://pubchem.ncbi.nlm.nih.gov/compound/59683889#section=2D-Structure
https://pubchem.ncbi.nlm.nih.gov/compound/59650222#section=2D-Structure
https://pubchem.ncbi.nlm.nih.gov/compound/59642675#section=2D-Structure
https://pubchem.ncbi.nlm.nih.gov/compound/59642459#section=2D-Structure
https://pubchem.ncbi.nlm.nih.gov/compound/5963143#section=2D-Structure
https://pubchem.ncbi.nlm.nih.gov/compound/59628570#section=2D-Structure
https://pubchem.ncbi.nlm.nih.gov/compound/59623609#section=2D-Structure
https://pubchem.ncbi.nlm.nih.gov/compound/59621931#section=2D-Structure
https://pubchem.ncbi.nlm.nih.gov/compound/59565908#section=2D-Structure
https://pubchem.ncbi.nlm.nih.gov/compound/59561087#section=2D-Structure
https://pubchem.ncbi.nlm.nih.gov/compound/59561078#section=2D-Structure
https://pubchem.ncbi.nlm.nih.gov/compound/59520035#section=2D-Structure
https://pubchem.ncbi.nlm.nih.gov/compound/59520032#section=2D-Structure
https://pubchem.ncbi.nlm.nih.gov/compound/59513167#section=2D-Structure
https://pubchem.ncbi.nlm.nih.gov/compound/59510195#section=2D-Structure
https://pubchem.ncbi.nlm.nih.gov/compound/59510180#section=2D-Structure
https://pubchem.ncbi.nlm.nih.gov/compound/59510178#section=2D-Structure
https://pubchem.ncbi.nlm.nih.gov/compound/59510168#section=2D-Structure
https://pubchem.ncbi.nlm.nih.gov/compound/59510160#section=2D-Structure
https://pubchem.ncbi.nlm.nih.gov/compound/59510153#section=2D-Structure
https://pubchem.ncbi.nlm.nih.gov/compound/59510134#section=2D-Structure
https://pubchem.ncbi.nlm.nih.gov/compound/59510107#section=2D-Structure
https://pubchem.ncbi.nlm.nih.gov/compound/59510103#section=2D-Structure
https://pubchem.ncbi.nlm.nih.gov/compound/59510068#section=2D-Structure
https://pubchem.ncbi.nlm.nih.gov/compound/59510061#section=2D-Structure
https://pubchem.ncbi.nlm.nih.gov/compound/59510046#section=2D-Structure
https://pubchem.ncbi.nlm.nih.gov/compound/59502520#section=2D-Structure
https://pubchem.ncbi.nlm.nih.gov/compound/59502518#section=2D-Structure
https://pubchem.ncbi.nlm.nih.gov/compound/59502508#section=2D-Structure
https://pubchem.ncbi.nlm.nih.gov/compound/59502504#section=2D-Structure
https://pubchem.ncbi.nlm.nih.gov/compound/59502501#section=2D-Structure
https://pubchem.ncbi.nlm.nih.gov/compound/59502496#section=2D-Structure
https://pubchem.ncbi.nlm.nih.gov/compound/59502495#section=2D-Structure
https://pubchem.ncbi.nlm.nih.gov/compound/59502494#section=2D-Structure
https://pubchem.ncbi.nlm.nih.gov/compound/59502492#section=2D-Structure
https://pubchem.ncbi.nlm.nih.gov/compound/59469651#section=2D-Structure
https://pubchem.ncbi.nlm.nih.gov/compound/59469642#section=2D-Structure
https://pubchem.ncbi.nlm.nih.gov/compound/59469620#section=2D-Structure
https://pubchem.ncbi.nlm.nih.gov/compound/59469608#section=2D-Structure
https://pubchem.ncbi.nlm.nih.gov/compound/59467712#section=2D-Structure
https://pubchem.ncbi.nlm.nih.gov/compound/59467711#section=2D-Structure
https://pubchem.ncbi.nlm.nih.gov/compound/59467676#section=2D-Structure
https://pubchem.ncbi.nlm.nih.gov/compound/59467675#section=2D-Structure
https://pubchem.ncbi.nlm.nih.gov/compound/59467660#section=2D-Structure
https://pubchem.ncbi.nlm.nih.gov/compound/59467659#section=2D-Structure
https://pubchem.ncbi.nlm.nih.gov/compound/59467658#section=2D-Structure
https://pubchem.ncbi.nlm.nih.gov/compound/59467652#section=2D-Structure
https://pubchem.ncbi.nlm.nih.gov/compound/59467639#section=2D-Structure
https://pubchem.ncbi.nlm.nih.gov/compound/59467631#section=2D-Structure
https://pubchem.ncbi.nlm.nih.gov/compound/59467620#section=2D-Structure
https://pubchem.ncbi.nlm.nih.gov/compound/59467607#section=2D-Structure
https://pubchem.ncbi.nlm.nih.gov/compound/59467606#section=2D-Structure
https://pubchem.ncbi.nlm.nih.gov/compound/59459889#section=2D-Structure

https://pubchem.ncbi.nlm.nih.gov/compound/59422764#section=2D-Structure
https://pubchem.ncbi.nlm.nih.gov/compound/59422623#section=2D-Structure
https://pubchem.ncbi.nlm.nih.gov/compound/59412465#section=2D-Structure
https://pubchem.ncbi.nlm.nih.gov/compound/59403900#section=2D-Structure
https://pubchem.ncbi.nlm.nih.gov/compound/59403877#section=2D-Structure
https://pubchem.ncbi.nlm.nih.gov/compound/59403875#section=2D-Structure
https://pubchem.ncbi.nlm.nih.gov/compound/59403843#section=2D-Structure
https://pubchem.ncbi.nlm.nih.gov/compound/59403814#section=2D-Structure
https://pubchem.ncbi.nlm.nih.gov/compound/59398760#section=2D-Structure
https://pubchem.ncbi.nlm.nih.gov/compound/59383533#section=2D-Structure
https://pubchem.ncbi.nlm.nih.gov/compound/59378696#section=2D-Structure
https://pubchem.ncbi.nlm.nih.gov/compound/59365838#section=2D-Structure
https://pubchem.ncbi.nlm.nih.gov/compound/59352637#section=2D-Structure
https://pubchem.ncbi.nlm.nih.gov/compound/59348106#section=2D-Structure
https://pubchem.ncbi.nlm.nih.gov/compound/59348093#section=2D-Structure
https://pubchem.ncbi.nlm.nih.gov/compound/59345826#section=2D-Structure
https://pubchem.ncbi.nlm.nih.gov/compound/59340836#section=2D-Structure
https://pubchem.ncbi.nlm.nih.gov/compound/59340832#section=2D-Structure
https://pubchem.ncbi.nlm.nih.gov/compound/59336297#section=2D-Structure
https://pubchem.ncbi.nlm.nih.gov/compound/59336292#section=2D-Structure
https://pubchem.ncbi.nlm.nih.gov/compound/59319629#section=2D-Structure
https://pubchem.ncbi.nlm.nih.gov/compound/59302057#section=2D-Structure
https://pubchem.ncbi.nlm.nih.gov/compound/59260571#section=2D-Structure
https://pubchem.ncbi.nlm.nih.gov/compound/59256775#section=2D-Structure
https://pubchem.ncbi.nlm.nih.gov/compound/59256621#section=2D-Structure
https://pubchem.ncbi.nlm.nih.gov/compound/59256558#section=2D-Structure
https://pubchem.ncbi.nlm.nih.gov/compound/59197347#section=2D-Structure
https://pubchem.ncbi.nlm.nih.gov/compound/59184052#section=2D-Structure
https://pubchem.ncbi.nlm.nih.gov/compound/59167625#section=2D-Structure
https://pubchem.ncbi.nlm.nih.gov/compound/59167232#section=2D-Structure
https://pubchem.ncbi.nlm.nih.gov/compound/59121095#section=2D-Structure
https://pubchem.ncbi.nlm.nih.gov/compound/59092767#section=2D-Structure
https://pubchem.ncbi.nlm.nih.gov/compound/59029855#section=2D-Structure
https://pubchem.ncbi.nlm.nih.gov/compound/59016501#section=2D-Structure
https://pubchem.ncbi.nlm.nih.gov/compound/59016497#section=2D-Structure
https://pubchem.ncbi.nlm.nih.gov/compound/59016484#section=2D-Structure
https://pubchem.ncbi.nlm.nih.gov/compound/59012507#section=2D-Structure
https://pubchem.ncbi.nlm.nih.gov/compound/59005065#section=2D-Structure
https://pubchem.ncbi.nlm.nih.gov/compound/59003413#section=2D-Structure
https://pubchem.ncbi.nlm.nih.gov/compound/59002257#section=2D-Structure
https://pubchem.ncbi.nlm.nih.gov/compound/58986760#section=2D-Structure
https://pubchem.ncbi.nlm.nih.gov/compound/58971196#section=2D-Structure
https://pubchem.ncbi.nlm.nih.gov/compound/58949614#section=2D-Structure
https://pubchem.ncbi.nlm.nih.gov/compound/58923#section=2D-Structure
https://pubchem.ncbi.nlm.nih.gov/compound/58921#section=2D-Structure
https://pubchem.ncbi.nlm.nih.gov/compound/58918396#section=2D-Structure
https://pubchem.ncbi.nlm.nih.gov/compound/58915771#section=2D-Structure
https://pubchem.ncbi.nlm.nih.gov/compound/58915763#section=2D-Structure
https://pubchem.ncbi.nlm.nih.gov/compound/58915756#section=2D-Structure
https://pubchem.ncbi.nlm.nih.gov/compound/58915750#section=2D-Structure
https://pubchem.ncbi.nlm.nih.gov/compound/58915740#section=2D-Structure
https://pubchem.ncbi.nlm.nih.gov/compound/58915731#section=2D-Structure
https://pubchem.ncbi.nlm.nih.gov/compound/58905856#section=2D-Structure
https://pubchem.ncbi.nlm.nih.gov/compound/58898815#section=2D-Structure
https://pubchem.ncbi.nlm.nih.gov/compound/58898812#section=2D-Structure

https://pubchem.ncbi.nlm.nih.gov/compound/58898800#section=2D-Structure
https://pubchem.ncbi.nlm.nih.gov/compound/58898791#section=2D-Structure
https://pubchem.ncbi.nlm.nih.gov/compound/58898788#section=2D-Structure
https://pubchem.ncbi.nlm.nih.gov/compound/58898781#section=2D-Structure
https://pubchem.ncbi.nlm.nih.gov/compound/58898780#section=2D-Structure
https://pubchem.ncbi.nlm.nih.gov/compound/58898779#section=2D-Structure
https://pubchem.ncbi.nlm.nih.gov/compound/58898763#section=2D-Structure
https://pubchem.ncbi.nlm.nih.gov/compound/58898760#section=2D-Structure
https://pubchem.ncbi.nlm.nih.gov/compound/58898753#section=2D-Structure
https://pubchem.ncbi.nlm.nih.gov/compound/58898743#section=2D-Structure
https://pubchem.ncbi.nlm.nih.gov/compound/58898730#section=2D-Structure
https://pubchem.ncbi.nlm.nih.gov/compound/58898726#section=2D-Structure
https://pubchem.ncbi.nlm.nih.gov/compound/58898720#section=2D-Structure
https://pubchem.ncbi.nlm.nih.gov/compound/58881386#section=2D-Structure
https://pubchem.ncbi.nlm.nih.gov/compound/58875780#section=2D-Structure
https://pubchem.ncbi.nlm.nih.gov/compound/58831040#section=2D-Structure
https://pubchem.ncbi.nlm.nih.gov/compound/58830911#section=2D-Structure
https://pubchem.ncbi.nlm.nih.gov/compound/58827698#section=2D-Structure
https://pubchem.ncbi.nlm.nih.gov/compound/58827693#section=2D-Structure
https://pubchem.ncbi.nlm.nih.gov/compound/58827668#section=2D-Structure
https://pubchem.ncbi.nlm.nih.gov/compound/58827665#section=2D-Structure
https://pubchem.ncbi.nlm.nih.gov/compound/58827664#section=2D-Structure
https://pubchem.ncbi.nlm.nih.gov/compound/58825154#section=2D-Structure
https://pubchem.ncbi.nlm.nih.gov/compound/58814516#section=2D-Structure
https://pubchem.ncbi.nlm.nih.gov/compound/58813968#section=2D-Structure
https://pubchem.ncbi.nlm.nih.gov/compound/58813916#section=2D-Structure
https://pubchem.ncbi.nlm.nih.gov/compound/58813802#section=2D-Structure
https://pubchem.ncbi.nlm.nih.gov/compound/58813769#section=2D-Structure
https://pubchem.ncbi.nlm.nih.gov/compound/58813740#section=2D-Structure
https://pubchem.ncbi.nlm.nih.gov/compound/58813585#section=2D-Structure
https://pubchem.ncbi.nlm.nih.gov/compound/58805651#section=2D-Structure
https://pubchem.ncbi.nlm.nih.gov/compound/58803261#section=2D-Structure
https://pubchem.ncbi.nlm.nih.gov/compound/58800444#section=2D-Structure
https://pubchem.ncbi.nlm.nih.gov/compound/58792607#section=2D-Structure
https://pubchem.ncbi.nlm.nih.gov/compound/58792604#section=2D-Structure
https://pubchem.ncbi.nlm.nih.gov/compound/58785091#section=2D-Structure
https://pubchem.ncbi.nlm.nih.gov/compound/58779838#section=2D-Structure
https://pubchem.ncbi.nlm.nih.gov/compound/58779798#section=2D-Structure
https://pubchem.ncbi.nlm.nih.gov/compound/58738545#section=2D-Structure
https://pubchem.ncbi.nlm.nih.gov/compound/58734342#section=2D-Structure
https://pubchem.ncbi.nlm.nih.gov/compound/58731987#section=2D-Structure
https://pubchem.ncbi.nlm.nih.gov/compound/58730123#section=2D-Structure
https://pubchem.ncbi.nlm.nih.gov/compound/58722584#section=2D-Structure
https://pubchem.ncbi.nlm.nih.gov/compound/58715757#section=2D-Structure
https://pubchem.ncbi.nlm.nih.gov/compound/58690788#section=2D-Structure
https://pubchem.ncbi.nlm.nih.gov/compound/58681470#section=2D-Structure
https://pubchem.ncbi.nlm.nih.gov/compound/58680658#section=2D-Structure
https://pubchem.ncbi.nlm.nih.gov/compound/58676200#section=2D-Structure
https://pubchem.ncbi.nlm.nih.gov/compound/58673389#section=2D-Structure
https://pubchem.ncbi.nlm.nih.gov/compound/58669277#section=2D-Structure
https://pubchem.ncbi.nlm.nih.gov/compound/58668873#section=2D-Structure
https://pubchem.ncbi.nlm.nih.gov/compound/58663739#section=2D-Structure
https://pubchem.ncbi.nlm.nih.gov/compound/58625646#section=2D-Structure
https://pubchem.ncbi.nlm.nih.gov/compound/58623562#section=2D-Structure
https://pubchem.ncbi.nlm.nih.gov/compound/58536657#section=2D-Structure

https://pubchem.ncbi.nlm.nih.gov/compound/58517854#section=2D-Structure
https://pubchem.ncbi.nlm.nih.gov/compound/58490115#section=2D-Structure
https://pubchem.ncbi.nlm.nih.gov/compound/58462768#section=2D-Structure
https://pubchem.ncbi.nlm.nih.gov/compound/58435014#section=2D-Structure
https://pubchem.ncbi.nlm.nih.gov/compound/58426670#section=2D-Structure
https://pubchem.ncbi.nlm.nih.gov/compound/58411607#section=2D-Structure
https://pubchem.ncbi.nlm.nih.gov/compound/58411121#section=2D-Structure
https://pubchem.ncbi.nlm.nih.gov/compound/58411076#section=2D-Structure
https://pubchem.ncbi.nlm.nih.gov/compound/58410881#section=2D-Structure
https://pubchem.ncbi.nlm.nih.gov/compound/58403475#section=2D-Structure
https://pubchem.ncbi.nlm.nih.gov/compound/58403474#section=2D-Structure
https://pubchem.ncbi.nlm.nih.gov/compound/58391792#section=2D-Structure
https://pubchem.ncbi.nlm.nih.gov/compound/58391774#section=2D-Structure
https://pubchem.ncbi.nlm.nih.gov/compound/58390399#section=2D-Structure
https://pubchem.ncbi.nlm.nih.gov/compound/58389864#section=2D-Structure
https://pubchem.ncbi.nlm.nih.gov/compound/5838246#section=2D-Structure
https://pubchem.ncbi.nlm.nih.gov/compound/58381866#section=2D-Structure
https://pubchem.ncbi.nlm.nih.gov/compound/58381858#section=2D-Structure
https://pubchem.ncbi.nlm.nih.gov/compound/58372100#section=2D-Structure
https://pubchem.ncbi.nlm.nih.gov/compound/58370643#section=2D-Structure
https://pubchem.ncbi.nlm.nih.gov/compound/58321686#section=2D-Structure
https://pubchem.ncbi.nlm.nih.gov/compound/58321644#section=2D-Structure
https://pubchem.ncbi.nlm.nih.gov/compound/58321606#section=2D-Structure
https://pubchem.ncbi.nlm.nih.gov/compound/58320127#section=2D-Structure
https://pubchem.ncbi.nlm.nih.gov/compound/58309865#section=2D-Structure
https://pubchem.ncbi.nlm.nih.gov/compound/58281937#section=2D-Structure
https://pubchem.ncbi.nlm.nih.gov/compound/58280670#section=2D-Structure
https://pubchem.ncbi.nlm.nih.gov/compound/58260660#section=2D-Structure
https://pubchem.ncbi.nlm.nih.gov/compound/58260635#section=2D-Structure
https://pubchem.ncbi.nlm.nih.gov/compound/58251140#section=2D-Structure
https://pubchem.ncbi.nlm.nih.gov/compound/58238766#section=2D-Structure
https://pubchem.ncbi.nlm.nih.gov/compound/58224410#section=2D-Structure
https://pubchem.ncbi.nlm.nih.gov/compound/58224257#section=2D-Structure
https://pubchem.ncbi.nlm.nih.gov/compound/582226#section=2D-Structure
https://pubchem.ncbi.nlm.nih.gov/compound/58220553#section=2D-Structure
https://pubchem.ncbi.nlm.nih.gov/compound/58220528#section=2D-Structure
https://pubchem.ncbi.nlm.nih.gov/compound/58220524#section=2D-Structure
https://pubchem.ncbi.nlm.nih.gov/compound/58219952#section=2D-Structure
https://pubchem.ncbi.nlm.nih.gov/compound/58219949#section=2D-Structure
https://pubchem.ncbi.nlm.nih.gov/compound/58193405#section=2D-Structure
https://pubchem.ncbi.nlm.nih.gov/compound/58193389#section=2D-Structure
https://pubchem.ncbi.nlm.nih.gov/compound/58185056#section=2D-Structure
https://pubchem.ncbi.nlm.nih.gov/compound/58185025#section=2D-Structure
https://pubchem.ncbi.nlm.nih.gov/compound/58175346#section=2D-Structure
https://pubchem.ncbi.nlm.nih.gov/compound/58175310#section=2D-Structure
https://pubchem.ncbi.nlm.nih.gov/compound/58162512#section=2D-Structure
https://pubchem.ncbi.nlm.nih.gov/compound/58151504#section=2D-Structure
https://pubchem.ncbi.nlm.nih.gov/compound/58103139#section=2D-Structure
https://pubchem.ncbi.nlm.nih.gov/compound/58095#section=2D-Structure
https://pubchem.ncbi.nlm.nih.gov/compound/58084932#section=2D-Structure
https://pubchem.ncbi.nlm.nih.gov/compound/58084924#section=2D-Structure
https://pubchem.ncbi.nlm.nih.gov/compound/58084922#section=2D-Structure
https://pubchem.ncbi.nlm.nih.gov/compound/58084904#section=2D-Structure
https://pubchem.ncbi.nlm.nih.gov/compound/58084866#section=2D-Structure
https://pubchem.ncbi.nlm.nih.gov/compound/58084864#section=2D-Structure

https://pubchem.ncbi.nlm.nih.gov/compound/58058859#section=2D-Structure
https://pubchem.ncbi.nlm.nih.gov/compound/58049739#section=2D-Structure
https://pubchem.ncbi.nlm.nih.gov/compound/58049735#section=2D-Structure
https://pubchem.ncbi.nlm.nih.gov/compound/58013630#section=2D-Structure
https://pubchem.ncbi.nlm.nih.gov/compound/57998558#section=2D-Structure
https://pubchem.ncbi.nlm.nih.gov/compound/57972864#section=2D-Structure
https://pubchem.ncbi.nlm.nih.gov/compound/57961#section=2D-Structure
https://pubchem.ncbi.nlm.nih.gov/compound/57960#section=2D-Structure
https://pubchem.ncbi.nlm.nih.gov/compound/57959098#section=2D-Structure
https://pubchem.ncbi.nlm.nih.gov/compound/57957959#section=2D-Structure
https://pubchem.ncbi.nlm.nih.gov/compound/57957956#section=2D-Structure
https://pubchem.ncbi.nlm.nih.gov/compound/57957932#section=2D-Structure
https://pubchem.ncbi.nlm.nih.gov/compound/57957929#section=2D-Structure
https://pubchem.ncbi.nlm.nih.gov/compound/57957924#section=2D-Structure
https://pubchem.ncbi.nlm.nih.gov/compound/57945661#section=2D-Structure
https://pubchem.ncbi.nlm.nih.gov/compound/57913427#section=2D-Structure
https://pubchem.ncbi.nlm.nih.gov/compound/57857865#section=2D-Structure
https://pubchem.ncbi.nlm.nih.gov/compound/57852045#section=2D-Structure
https://pubchem.ncbi.nlm.nih.gov/compound/57838736#section=2D-Structure
https://pubchem.ncbi.nlm.nih.gov/compound/57838724#section=2D-Structure
https://pubchem.ncbi.nlm.nih.gov/compound/57838718#section=2D-Structure
https://pubchem.ncbi.nlm.nih.gov/compound/57838715#section=2D-Structure
https://pubchem.ncbi.nlm.nih.gov/compound/57796591#section=2D-Structure
https://pubchem.ncbi.nlm.nih.gov/compound/57796573#section=2D-Structure
https://pubchem.ncbi.nlm.nih.gov/compound/57774101#section=2D-Structure
https://pubchem.ncbi.nlm.nih.gov/compound/57773964#section=2D-Structure
https://pubchem.ncbi.nlm.nih.gov/compound/57773963#section=2D-Structure
https://pubchem.ncbi.nlm.nih.gov/compound/57773226#section=2D-Structure
https://pubchem.ncbi.nlm.nih.gov/compound/57763580#section=2D-Structure
https://pubchem.ncbi.nlm.nih.gov/compound/57759900#section=2D-Structure
https://pubchem.ncbi.nlm.nih.gov/compound/57759629#section=2D-Structure
https://pubchem.ncbi.nlm.nih.gov/compound/57759384#section=2D-Structure
https://pubchem.ncbi.nlm.nih.gov/compound/57759281#section=2D-Structure
https://pubchem.ncbi.nlm.nih.gov/compound/57757663#section=2D-Structure
https://pubchem.ncbi.nlm.nih.gov/compound/57725#section=2D-Structure
https://pubchem.ncbi.nlm.nih.gov/compound/57711745#section=2D-Structure
https://pubchem.ncbi.nlm.nih.gov/compound/57709563#section=2D-Structure
https://pubchem.ncbi.nlm.nih.gov/compound/57689034#section=2D-Structure
https://pubchem.ncbi.nlm.nih.gov/compound/57687907#section=2D-Structure
https://pubchem.ncbi.nlm.nih.gov/compound/57673626#section=2D-Structure
https://pubchem.ncbi.nlm.nih.gov/compound/57641798#section=2D-Structure
https://pubchem.ncbi.nlm.nih.gov/compound/57628273#section=2D-Structure
https://pubchem.ncbi.nlm.nih.gov/compound/57616814#section=2D-Structure
https://pubchem.ncbi.nlm.nih.gov/compound/57615802#section=2D-Structure
https://pubchem.ncbi.nlm.nih.gov/compound/57615773#section=2D-Structure
https://pubchem.ncbi.nlm.nih.gov/compound/57615221#section=2D-Structure
https://pubchem.ncbi.nlm.nih.gov/compound/57615219#section=2D-Structure
https://pubchem.ncbi.nlm.nih.gov/compound/57571339#section=2D-Structure
https://pubchem.ncbi.nlm.nih.gov/compound/57568023#section=2D-Structure
https://pubchem.ncbi.nlm.nih.gov/compound/57552933#section=2D-Structure
https://pubchem.ncbi.nlm.nih.gov/compound/57552856#section=2D-Structure
https://pubchem.ncbi.nlm.nih.gov/compound/57552853#section=2D-Structure
https://pubchem.ncbi.nlm.nih.gov/compound/57552835#section=2D-Structure
https://pubchem.ncbi.nlm.nih.gov/compound/57552832#section=2D-Structure
https://pubchem.ncbi.nlm.nih.gov/compound/57552815#section=2D-Structure

https://pubchem.ncbi.nlm.nih.gov/compound/57552808#section=2D-Structure
https://pubchem.ncbi.nlm.nih.gov/compound/57552751#section=2D-Structure
https://pubchem.ncbi.nlm.nih.gov/compound/57552435#section=2D-Structure
https://pubchem.ncbi.nlm.nih.gov/compound/57543584#section=2D-Structure
https://pubchem.ncbi.nlm.nih.gov/compound/57543581#section=2D-Structure
https://pubchem.ncbi.nlm.nih.gov/compound/57543579#section=2D-Structure
https://pubchem.ncbi.nlm.nih.gov/compound/57543576#section=2D-Structure
https://pubchem.ncbi.nlm.nih.gov/compound/57543574#section=2D-Structure
https://pubchem.ncbi.nlm.nih.gov/compound/57543572#section=2D-Structure
https://pubchem.ncbi.nlm.nih.gov/compound/57543571#section=2D-Structure
https://pubchem.ncbi.nlm.nih.gov/compound/57543567#section=2D-Structure
https://pubchem.ncbi.nlm.nih.gov/compound/57543562#section=2D-Structure
https://pubchem.ncbi.nlm.nih.gov/compound/57543559#section=2D-Structure
https://pubchem.ncbi.nlm.nih.gov/compound/57543550#section=2D-Structure
https://pubchem.ncbi.nlm.nih.gov/compound/57543548#section=2D-Structure
https://pubchem.ncbi.nlm.nih.gov/compound/57543541#section=2D-Structure
https://pubchem.ncbi.nlm.nih.gov/compound/57543536#section=2D-Structure
https://pubchem.ncbi.nlm.nih.gov/compound/57536587#section=2D-Structure
https://pubchem.ncbi.nlm.nih.gov/compound/57536584#section=2D-Structure
https://pubchem.ncbi.nlm.nih.gov/compound/57536444#section=2D-Structure
https://pubchem.ncbi.nlm.nih.gov/compound/57536441#section=2D-Structure
https://pubchem.ncbi.nlm.nih.gov/compound/57536437#section=2D-Structure
https://pubchem.ncbi.nlm.nih.gov/compound/57536402#section=2D-Structure
https://pubchem.ncbi.nlm.nih.gov/compound/57536387#section=2D-Structure
https://pubchem.ncbi.nlm.nih.gov/compound/57536374#section=2D-Structure
https://pubchem.ncbi.nlm.nih.gov/compound/57536360#section=2D-Structure
https://pubchem.ncbi.nlm.nih.gov/compound/57536358#section=2D-Structure
https://pubchem.ncbi.nlm.nih.gov/compound/57536066#section=2D-Structure
https://pubchem.ncbi.nlm.nih.gov/compound/57523979#section=2D-Structure
https://pubchem.ncbi.nlm.nih.gov/compound/57504#section=2D-Structure
https://pubchem.ncbi.nlm.nih.gov/compound/57458870#section=2D-Structure
https://pubchem.ncbi.nlm.nih.gov/compound/57418116#section=2D-Structure
https://pubchem.ncbi.nlm.nih.gov/compound/57417960#section=2D-Structure
https://pubchem.ncbi.nlm.nih.gov/compound/57417361#section=2D-Structure
https://pubchem.ncbi.nlm.nih.gov/compound/57417290#section=2D-Structure
https://pubchem.ncbi.nlm.nih.gov/compound/57380725#section=2D-Structure
https://pubchem.ncbi.nlm.nih.gov/compound/57376328#section=2D-Structure
https://pubchem.ncbi.nlm.nih.gov/compound/57375574#section=2D-Structure
https://pubchem.ncbi.nlm.nih.gov/compound/57375445#section=2D-Structure
https://pubchem.ncbi.nlm.nih.gov/compound/57372977#section=2D-Structure
https://pubchem.ncbi.nlm.nih.gov/compound/57370962#section=2D-Structure
https://pubchem.ncbi.nlm.nih.gov/compound/57370704#section=2D-Structure
https://pubchem.ncbi.nlm.nih.gov/compound/57370565#section=2D-Structure
https://pubchem.ncbi.nlm.nih.gov/compound/57370390#section=2D-Structure
https://pubchem.ncbi.nlm.nih.gov/compound/57357943#section=2D-Structure
https://pubchem.ncbi.nlm.nih.gov/compound/57357930#section=2D-Structure
https://pubchem.ncbi.nlm.nih.gov/compound/57353022#section=2D-Structure
https://pubchem.ncbi.nlm.nih.gov/compound/57343403#section=2D-Structure
https://pubchem.ncbi.nlm.nih.gov/compound/57316047#section=2D-Structure
https://pubchem.ncbi.nlm.nih.gov/compound/57295824#section=2D-Structure
https://pubchem.ncbi.nlm.nih.gov/compound/572912#section=2D-Structure
https://pubchem.ncbi.nlm.nih.gov/compound/57286801#section=2D-Structure
https://pubchem.ncbi.nlm.nih.gov/compound/57261868#section=2D-Structure
https://pubchem.ncbi.nlm.nih.gov/compound/57249530#section=2D-Structure
https://pubchem.ncbi.nlm.nih.gov/compound/57235245#section=2D-Structure

https://pubchem.ncbi.nlm.nih.gov/compound/57226759#section=2D-Structure
https://pubchem.ncbi.nlm.nih.gov/compound/57220812#section=2D-Structure
https://pubchem.ncbi.nlm.nih.gov/compound/57220566#section=2D-Structure
https://pubchem.ncbi.nlm.nih.gov/compound/57196#section=2D-Structure
https://pubchem.ncbi.nlm.nih.gov/compound/57183364#section=2D-Structure
https://pubchem.ncbi.nlm.nih.gov/compound/57181844#section=2D-Structure
https://pubchem.ncbi.nlm.nih.gov/compound/57158191#section=2D-Structure
https://pubchem.ncbi.nlm.nih.gov/compound/57146922#section=2D-Structure
https://pubchem.ncbi.nlm.nih.gov/compound/57142130#section=2D-Structure
https://pubchem.ncbi.nlm.nih.gov/compound/57138951#section=2D-Structure
https://pubchem.ncbi.nlm.nih.gov/compound/57130709#section=2D-Structure
https://pubchem.ncbi.nlm.nih.gov/compound/57127212#section=2D-Structure
https://pubchem.ncbi.nlm.nih.gov/compound/57100984#section=2D-Structure
https://pubchem.ncbi.nlm.nih.gov/compound/57093109#section=2D-Structure
https://pubchem.ncbi.nlm.nih.gov/compound/57090658#section=2D-Structure
https://pubchem.ncbi.nlm.nih.gov/compound/57087732#section=2D-Structure
https://pubchem.ncbi.nlm.nih.gov/compound/57075785#section=2D-Structure
https://pubchem.ncbi.nlm.nih.gov/compound/57070126#section=2D-Structure
https://pubchem.ncbi.nlm.nih.gov/compound/57069115#section=2D-Structure
https://pubchem.ncbi.nlm.nih.gov/compound/5705483#section=2D-Structure
https://pubchem.ncbi.nlm.nih.gov/compound/57053190#section=2D-Structure
https://pubchem.ncbi.nlm.nih.gov/compound/57052860#section=2D-Structure
https://pubchem.ncbi.nlm.nih.gov/compound/5704851#section=2D-Structure
https://pubchem.ncbi.nlm.nih.gov/compound/57048219#section=2D-Structure
https://pubchem.ncbi.nlm.nih.gov/compound/57036498#section=2D-Structure
https://pubchem.ncbi.nlm.nih.gov/compound/57025316#section=2D-Structure
https://pubchem.ncbi.nlm.nih.gov/compound/57009922#section=2D-Structure
https://pubchem.ncbi.nlm.nih.gov/compound/56981555#section=2D-Structure
https://pubchem.ncbi.nlm.nih.gov/compound/56979682#section=2D-Structure
https://pubchem.ncbi.nlm.nih.gov/compound/56957306#section=2D-Structure
https://pubchem.ncbi.nlm.nih.gov/compound/56957140#section=2D-Structure
https://pubchem.ncbi.nlm.nih.gov/compound/56947478#section=2D-Structure
https://pubchem.ncbi.nlm.nih.gov/compound/56947473#section=2D-Structure
https://pubchem.ncbi.nlm.nih.gov/compound/56946227#section=2D-Structure
https://pubchem.ncbi.nlm.nih.gov/compound/56932269#section=2D-Structure
https://pubchem.ncbi.nlm.nih.gov/compound/56931640#section=2D-Structure
https://pubchem.ncbi.nlm.nih.gov/compound/56924695#section=2D-Structure
https://pubchem.ncbi.nlm.nih.gov/compound/56845461#section=2D-Structure
https://pubchem.ncbi.nlm.nih.gov/compound/56842801#section=2D-Structure
https://pubchem.ncbi.nlm.nih.gov/compound/56744#section=2D-Structure
https://pubchem.ncbi.nlm.nih.gov/compound/56696517#section=2D-Structure
https://pubchem.ncbi.nlm.nih.gov/compound/56690268#section=2D-Structure
https://pubchem.ncbi.nlm.nih.gov/compound/56688814#section=2D-Structure
https://pubchem.ncbi.nlm.nih.gov/compound/56647955#section=2D-Structure
https://pubchem.ncbi.nlm.nih.gov/compound/56647954#section=2D-Structure
https://pubchem.ncbi.nlm.nih.gov/compound/56639274#section=2D-Structure
https://pubchem.ncbi.nlm.nih.gov/compound/56637141#section=2D-Structure
https://pubchem.ncbi.nlm.nih.gov/compound/56632413#section=2D-Structure
https://pubchem.ncbi.nlm.nih.gov/compound/56630746#section=2D-Structure
https://pubchem.ncbi.nlm.nih.gov/compound/56629869#section=2D-Structure
https://pubchem.ncbi.nlm.nih.gov/compound/56626614#section=2D-Structure
https://pubchem.ncbi.nlm.nih.gov/compound/56625779#section=2D-Structure
https://pubchem.ncbi.nlm.nih.gov/compound/56622004#section=2D-Structure
https://pubchem.ncbi.nlm.nih.gov/compound/56621677#section=2D-Structure
https://pubchem.ncbi.nlm.nih.gov/compound/56619448#section=2D-Structure

https://pubchem.ncbi.nlm.nih.gov/compound/56617803#section=2D-Structure
https://pubchem.ncbi.nlm.nih.gov/compound/56614901#section=2D-Structure
https://pubchem.ncbi.nlm.nih.gov/compound/56614640#section=2D-Structure
https://pubchem.ncbi.nlm.nih.gov/compound/56612228#section=2D-Structure
https://pubchem.ncbi.nlm.nih.gov/compound/56610564#section=2D-Structure
https://pubchem.ncbi.nlm.nih.gov/compound/56607879#section=2D-Structure
https://pubchem.ncbi.nlm.nih.gov/compound/56607673#section=2D-Structure
https://pubchem.ncbi.nlm.nih.gov/compound/56600844#section=2D-Structure
https://pubchem.ncbi.nlm.nih.gov/compound/564899#section=2D-Structure
https://pubchem.ncbi.nlm.nih.gov/compound/56343#section=2D-Structure
https://pubchem.ncbi.nlm.nih.gov/compound/560845#section=2D-Structure
https://pubchem.ncbi.nlm.nih.gov/compound/55576#section=2D-Structure
https://pubchem.ncbi.nlm.nih.gov/compound/55574#section=2D-Structure
https://pubchem.ncbi.nlm.nih.gov/compound/55268579#section=2D-Structure
https://pubchem.ncbi.nlm.nih.gov/compound/55051#section=2D-Structure
https://pubchem.ncbi.nlm.nih.gov/compound/54880#section=2D-Structure
https://pubchem.ncbi.nlm.nih.gov/compound/54879#section=2D-Structure
https://pubchem.ncbi.nlm.nih.gov/compound/54878#section=2D-Structure
https://pubchem.ncbi.nlm.nih.gov/compound/54733#section=2D-Structure
https://pubchem.ncbi.nlm.nih.gov/compound/54704137#section=2D-Structure
https://pubchem.ncbi.nlm.nih.gov/compound/5463258#section=2D-Structure
https://pubchem.ncbi.nlm.nih.gov/compound/54606361#section=2D-Structure
https://pubchem.ncbi.nlm.nih.gov/compound/54590506#section=2D-Structure
https://pubchem.ncbi.nlm.nih.gov/compound/54590502#section=2D-Structure
https://pubchem.ncbi.nlm.nih.gov/compound/54569003#section=2D-Structure
https://pubchem.ncbi.nlm.nih.gov/compound/54559519#section=2D-Structure
https://pubchem.ncbi.nlm.nih.gov/compound/54556901#section=2D-Structure
https://pubchem.ncbi.nlm.nih.gov/compound/54545117#section=2D-Structure
https://pubchem.ncbi.nlm.nih.gov/compound/54545#section=2D-Structure
https://pubchem.ncbi.nlm.nih.gov/compound/54544672#section=2D-Structure
https://pubchem.ncbi.nlm.nih.gov/compound/54543#section=2D-Structure
https://pubchem.ncbi.nlm.nih.gov/compound/54520966#section=2D-Structure
https://pubchem.ncbi.nlm.nih.gov/compound/54514698#section=2D-Structure
https://pubchem.ncbi.nlm.nih.gov/compound/54507255#section=2D-Structure
https://pubchem.ncbi.nlm.nih.gov/compound/54506758#section=2D-Structure
https://pubchem.ncbi.nlm.nih.gov/compound/54504698#section=2D-Structure
https://pubchem.ncbi.nlm.nih.gov/compound/54483141#section=2D-Structure
https://pubchem.ncbi.nlm.nih.gov/compound/54482555#section=2D-Structure
https://pubchem.ncbi.nlm.nih.gov/compound/54482238#section=2D-Structure
https://pubchem.ncbi.nlm.nih.gov/compound/54479019#section=2D-Structure
https://pubchem.ncbi.nlm.nih.gov/compound/54474312#section=2D-Structure
https://pubchem.ncbi.nlm.nih.gov/compound/54469828#section=2D-Structure
https://pubchem.ncbi.nlm.nih.gov/compound/54469125#section=2D-Structure
https://pubchem.ncbi.nlm.nih.gov/compound/54461681#section=2D-Structure
https://pubchem.ncbi.nlm.nih.gov/compound/54435524#section=2D-Structure
https://pubchem.ncbi.nlm.nih.gov/compound/54434326#section=2D-Structure
https://pubchem.ncbi.nlm.nih.gov/compound/54429442#section=2D-Structure
https://pubchem.ncbi.nlm.nih.gov/compound/54426862#section=2D-Structure
https://pubchem.ncbi.nlm.nih.gov/compound/54422369#section=2D-Structure
https://pubchem.ncbi.nlm.nih.gov/compound/54417012#section=2D-Structure
https://pubchem.ncbi.nlm.nih.gov/compound/54413837#section=2D-Structure
https://pubchem.ncbi.nlm.nih.gov/compound/54407305#section=2D-Structure
https://pubchem.ncbi.nlm.nih.gov/compound/54406373#section=2D-Structure
https://pubchem.ncbi.nlm.nih.gov/compound/54395690#section=2D-Structure
https://pubchem.ncbi.nlm.nih.gov/compound/54392957#section=2D-Structure

https://pubchem.ncbi.nlm.nih.gov/compound/54391575#section=2D-Structure
https://pubchem.ncbi.nlm.nih.gov/compound/54370894#section=2D-Structure
https://pubchem.ncbi.nlm.nih.gov/compound/54364881#section=2D-Structure
https://pubchem.ncbi.nlm.nih.gov/compound/54364012#section=2D-Structure
https://pubchem.ncbi.nlm.nih.gov/compound/54363922#section=2D-Structure
https://pubchem.ncbi.nlm.nih.gov/compound/54354101#section=2D-Structure
https://pubchem.ncbi.nlm.nih.gov/compound/54349734#section=2D-Structure
https://pubchem.ncbi.nlm.nih.gov/compound/54349402#section=2D-Structure
https://pubchem.ncbi.nlm.nih.gov/compound/54349067#section=2D-Structure
https://pubchem.ncbi.nlm.nih.gov/compound/54346647#section=2D-Structure
https://pubchem.ncbi.nlm.nih.gov/compound/54345421#section=2D-Structure
https://pubchem.ncbi.nlm.nih.gov/compound/54336280#section=2D-Structure
https://pubchem.ncbi.nlm.nih.gov/compound/54334998#section=2D-Structure
https://pubchem.ncbi.nlm.nih.gov/compound/54318476#section=2D-Structure
https://pubchem.ncbi.nlm.nih.gov/compound/54313771#section=2D-Structure
https://pubchem.ncbi.nlm.nih.gov/compound/54299687#section=2D-Structure
https://pubchem.ncbi.nlm.nih.gov/compound/54299538#section=2D-Structure
https://pubchem.ncbi.nlm.nih.gov/compound/54296210#section=2D-Structure
https://pubchem.ncbi.nlm.nih.gov/compound/54292#section=2D-Structure
https://pubchem.ncbi.nlm.nih.gov/compound/54291#section=2D-Structure
https://pubchem.ncbi.nlm.nih.gov/compound/54288684#section=2D-Structure
https://pubchem.ncbi.nlm.nih.gov/compound/54284585#section=2D-Structure
https://pubchem.ncbi.nlm.nih.gov/compound/54277783#section=2D-Structure
https://pubchem.ncbi.nlm.nih.gov/compound/54273685#section=2D-Structure
https://pubchem.ncbi.nlm.nih.gov/compound/54272825#section=2D-Structure
https://pubchem.ncbi.nlm.nih.gov/compound/54271852#section=2D-Structure
https://pubchem.ncbi.nlm.nih.gov/compound/54253429#section=2D-Structure
https://pubchem.ncbi.nlm.nih.gov/compound/54252982#section=2D-Structure
https://pubchem.ncbi.nlm.nih.gov/compound/54250922#section=2D-Structure
https://pubchem.ncbi.nlm.nih.gov/compound/54242562#section=2D-Structure
https://pubchem.ncbi.nlm.nih.gov/compound/54235055#section=2D-Structure
https://pubchem.ncbi.nlm.nih.gov/compound/54220816#section=2D-Structure
https://pubchem.ncbi.nlm.nih.gov/compound/54212653#section=2D-Structure
https://pubchem.ncbi.nlm.nih.gov/compound/54211532#section=2D-Structure
https://pubchem.ncbi.nlm.nih.gov/compound/54206939#section=2D-Structure
https://pubchem.ncbi.nlm.nih.gov/compound/54189954#section=2D-Structure
https://pubchem.ncbi.nlm.nih.gov/compound/54188020#section=2D-Structure
https://pubchem.ncbi.nlm.nih.gov/compound/54186076#section=2D-Structure
https://pubchem.ncbi.nlm.nih.gov/compound/54180634#section=2D-Structure
https://pubchem.ncbi.nlm.nih.gov/compound/54174574#section=2D-Structure
https://pubchem.ncbi.nlm.nih.gov/compound/54173757#section=2D-Structure
https://pubchem.ncbi.nlm.nih.gov/compound/54171867#section=2D-Structure
https://pubchem.ncbi.nlm.nih.gov/compound/54170636#section=2D-Structure
https://pubchem.ncbi.nlm.nih.gov/compound/54169218#section=2D-Structure
https://pubchem.ncbi.nlm.nih.gov/compound/54161328#section=2D-Structure
https://pubchem.ncbi.nlm.nih.gov/compound/54139631#section=2D-Structure
https://pubchem.ncbi.nlm.nih.gov/compound/54137574#section=2D-Structure
https://pubchem.ncbi.nlm.nih.gov/compound/54133116#section=2D-Structure
https://pubchem.ncbi.nlm.nih.gov/compound/54125697#section=2D-Structure
https://pubchem.ncbi.nlm.nih.gov/compound/54123187#section=2D-Structure
https://pubchem.ncbi.nlm.nih.gov/compound/54119343#section=2D-Structure
https://pubchem.ncbi.nlm.nih.gov/compound/54116602#section=2D-Structure
https://pubchem.ncbi.nlm.nih.gov/compound/54108113#section=2D-Structure
https://pubchem.ncbi.nlm.nih.gov/compound/54107119#section=2D-Structure
https://pubchem.ncbi.nlm.nih.gov/compound/54105047#section=2D-Structure

https://pubchem.ncbi.nlm.nih.gov/compound/54088140#section=2D-Structure
https://pubchem.ncbi.nlm.nih.gov/compound/54076186#section=2D-Structure
https://pubchem.ncbi.nlm.nih.gov/compound/54065971#section=2D-Structure
https://pubchem.ncbi.nlm.nih.gov/compound/54039058#section=2D-Structure
https://pubchem.ncbi.nlm.nih.gov/compound/54034489#section=2D-Structure
https://pubchem.ncbi.nlm.nih.gov/compound/54027631#section=2D-Structure
https://pubchem.ncbi.nlm.nih.gov/compound/54010498#section=2D-Structure
https://pubchem.ncbi.nlm.nih.gov/compound/54007360#section=2D-Structure
https://pubchem.ncbi.nlm.nih.gov/compound/54002015#section=2D-Structure
https://pubchem.ncbi.nlm.nih.gov/compound/53996273#section=2D-Structure
https://pubchem.ncbi.nlm.nih.gov/compound/53994026#section=2D-Structure
https://pubchem.ncbi.nlm.nih.gov/compound/53993333#section=2D-Structure
https://pubchem.ncbi.nlm.nih.gov/compound/53971163#section=2D-Structure
https://pubchem.ncbi.nlm.nih.gov/compound/53969589#section=2D-Structure
https://pubchem.ncbi.nlm.nih.gov/compound/53968979#section=2D-Structure
https://pubchem.ncbi.nlm.nih.gov/compound/53955039#section=2D-Structure
https://pubchem.ncbi.nlm.nih.gov/compound/53953765#section=2D-Structure
https://pubchem.ncbi.nlm.nih.gov/compound/53953560#section=2D-Structure
https://pubchem.ncbi.nlm.nih.gov/compound/53917670#section=2D-Structure
https://pubchem.ncbi.nlm.nih.gov/compound/53916745#section=2D-Structure
https://pubchem.ncbi.nlm.nih.gov/compound/53915733#section=2D-Structure
https://pubchem.ncbi.nlm.nih.gov/compound/53897864#section=2D-Structure
https://pubchem.ncbi.nlm.nih.gov/compound/53893327#section=2D-Structure
https://pubchem.ncbi.nlm.nih.gov/compound/53888938#section=2D-Structure
https://pubchem.ncbi.nlm.nih.gov/compound/53882246#section=2D-Structure
https://pubchem.ncbi.nlm.nih.gov/compound/53878310#section=2D-Structure
https://pubchem.ncbi.nlm.nih.gov/compound/53875095#section=2D-Structure
https://pubchem.ncbi.nlm.nih.gov/compound/53858239#section=2D-Structure
https://pubchem.ncbi.nlm.nih.gov/compound/53854003#section=2D-Structure
https://pubchem.ncbi.nlm.nih.gov/compound/53844349#section=2D-Structure
https://pubchem.ncbi.nlm.nih.gov/compound/53842575#section=2D-Structure
https://pubchem.ncbi.nlm.nih.gov/compound/53829103#section=2D-Structure
https://pubchem.ncbi.nlm.nih.gov/compound/53822905#section=2D-Structure
https://pubchem.ncbi.nlm.nih.gov/compound/53821761#section=2D-Structure
https://pubchem.ncbi.nlm.nih.gov/compound/53810471#section=2D-Structure
https://pubchem.ncbi.nlm.nih.gov/compound/5377838#section=2D-Structure
https://pubchem.ncbi.nlm.nih.gov/compound/53776707#section=2D-Structure
https://pubchem.ncbi.nlm.nih.gov/compound/53773074#section=2D-Structure
https://pubchem.ncbi.nlm.nih.gov/compound/53768244#section=2D-Structure
https://pubchem.ncbi.nlm.nih.gov/compound/53762028#section=2D-Structure
https://pubchem.ncbi.nlm.nih.gov/compound/5375369#section=2D-Structure
https://pubchem.ncbi.nlm.nih.gov/compound/5374023#section=2D-Structure
https://pubchem.ncbi.nlm.nih.gov/compound/53739688#section=2D-Structure
https://pubchem.ncbi.nlm.nih.gov/compound/53738785#section=2D-Structure
https://pubchem.ncbi.nlm.nih.gov/compound/53737397#section=2D-Structure
https://pubchem.ncbi.nlm.nih.gov/compound/53736818#section=2D-Structure
https://pubchem.ncbi.nlm.nih.gov/compound/53734095#section=2D-Structure
https://pubchem.ncbi.nlm.nih.gov/compound/53725353#section=2D-Structure
https://pubchem.ncbi.nlm.nih.gov/compound/53712206#section=2D-Structure
https://pubchem.ncbi.nlm.nih.gov/compound/53710397#section=2D-Structure
https://pubchem.ncbi.nlm.nih.gov/compound/53667089#section=2D-Structure
https://pubchem.ncbi.nlm.nih.gov/compound/53665811#section=2D-Structure
https://pubchem.ncbi.nlm.nih.gov/compound/53664206#section=2D-Structure
https://pubchem.ncbi.nlm.nih.gov/compound/53661529#section=2D-Structure
https://pubchem.ncbi.nlm.nih.gov/compound/53649916#section=2D-Structure

https://pubchem.ncbi.nlm.nih.gov/compound/53649491#section=2D-Structure
https://pubchem.ncbi.nlm.nih.gov/compound/53441006#section=2D-Structure
https://pubchem.ncbi.nlm.nih.gov/compound/53438435#section=2D-Structure
https://pubchem.ncbi.nlm.nih.gov/compound/53437953#section=2D-Structure
https://pubchem.ncbi.nlm.nih.gov/compound/53436019#section=2D-Structure
https://pubchem.ncbi.nlm.nih.gov/compound/53431378#section=2D-Structure
https://pubchem.ncbi.nlm.nih.gov/compound/534153#section=2D-Structure
https://pubchem.ncbi.nlm.nih.gov/compound/53400946#section=2D-Structure
https://pubchem.ncbi.nlm.nih.gov/compound/53315526#section=2D-Structure
https://pubchem.ncbi.nlm.nih.gov/compound/53310455#section=2D-Structure
https://pubchem.ncbi.nlm.nih.gov/compound/5330889#section=2D-Structure
https://pubchem.ncbi.nlm.nih.gov/compound/5330888#section=2D-Structure
https://pubchem.ncbi.nlm.nih.gov/compound/53297434#section=2D-Structure
https://pubchem.ncbi.nlm.nih.gov/compound/53254646#section=2D-Structure
https://pubchem.ncbi.nlm.nih.gov/compound/53249550#section=2D-Structure
https://pubchem.ncbi.nlm.nih.gov/compound/53230#section=2D-Structure
https://pubchem.ncbi.nlm.nih.gov/compound/53229#section=2D-Structure
https://pubchem.ncbi.nlm.nih.gov/compound/526645#section=2D-Structure
https://pubchem.ncbi.nlm.nih.gov/compound/5256594#section=2D-Structure
https://pubchem.ncbi.nlm.nih.gov/compound/5256498#section=2D-Structure
https://pubchem.ncbi.nlm.nih.gov/compound/524983#section=2D-Structure
https://pubchem.ncbi.nlm.nih.gov/compound/5239442#section=2D-Structure
https://pubchem.ncbi.nlm.nih.gov/compound/523200#section=2D-Structure
https://pubchem.ncbi.nlm.nih.gov/compound/522799#section=2D-Structure
https://pubchem.ncbi.nlm.nih.gov/compound/5225193#section=2D-Structure
https://pubchem.ncbi.nlm.nih.gov/compound/52218#section=2D-Structure
https://pubchem.ncbi.nlm.nih.gov/compound/52217#section=2D-Structure
https://pubchem.ncbi.nlm.nih.gov/compound/521820#section=2D-Structure
https://pubchem.ncbi.nlm.nih.gov/compound/5216861#section=2D-Structure
https://pubchem.ncbi.nlm.nih.gov/compound/52095744#section=2D-Structure
https://pubchem.ncbi.nlm.nih.gov/compound/51346159#section=2D-Structure
https://pubchem.ncbi.nlm.nih.gov/compound/51336#section=2D-Structure
https://pubchem.ncbi.nlm.nih.gov/compound/51255#section=2D-Structure
https://pubchem.ncbi.nlm.nih.gov/compound/51254#section=2D-Structure
https://pubchem.ncbi.nlm.nih.gov/compound/5123522#section=2D-Structure
https://pubchem.ncbi.nlm.nih.gov/compound/51117#section=2D-Structure
https://pubchem.ncbi.nlm.nih.gov/compound/50986100#section=2D-Structure
https://pubchem.ncbi.nlm.nih.gov/compound/50961#section=2D-Structure
https://pubchem.ncbi.nlm.nih.gov/compound/50958#section=2D-Structure
https://pubchem.ncbi.nlm.nih.gov/compound/5064524#section=2D-Structure
https://pubchem.ncbi.nlm.nih.gov/compound/5055736#section=2D-Structure
https://pubchem.ncbi.nlm.nih.gov/compound/5052385#section=2D-Structure
https://pubchem.ncbi.nlm.nih.gov/compound/4990776#section=2D-Structure
https://pubchem.ncbi.nlm.nih.gov/compound/49841197#section=2D-Structure
https://pubchem.ncbi.nlm.nih.gov/compound/49837503#section=2D-Structure
https://pubchem.ncbi.nlm.nih.gov/compound/494079#section=2D-Structure
https://pubchem.ncbi.nlm.nih.gov/compound/48034#section=2D-Structure
https://pubchem.ncbi.nlm.nih.gov/compound/47954#section=2D-Structure
https://pubchem.ncbi.nlm.nih.gov/compound/4757#section=2D-Structure
https://pubchem.ncbi.nlm.nih.gov/compound/47416#section=2D-Structure
https://pubchem.ncbi.nlm.nih.gov/compound/47415#section=2D-Structure
https://pubchem.ncbi.nlm.nih.gov/compound/46946305#section=2D-Structure
https://pubchem.ncbi.nlm.nih.gov/compound/46931020#section=2D-Structure
https://pubchem.ncbi.nlm.nih.gov/compound/46894595#section=2D-Structure
https://pubchem.ncbi.nlm.nih.gov/compound/46872427#section=2D-Structure

https://pubchem.ncbi.nlm.nih.gov/compound/46871891#section=2D-Structure
https://pubchem.ncbi.nlm.nih.gov/compound/46849172#section=2D-Structure
https://pubchem.ncbi.nlm.nih.gov/compound/46784535#section=2D-Structure
https://pubchem.ncbi.nlm.nih.gov/compound/46780462#section=2D-Structure
https://pubchem.ncbi.nlm.nih.gov/compound/4677071#section=2D-Structure
https://pubchem.ncbi.nlm.nih.gov/compound/4675840#section=2D-Structure
https://pubchem.ncbi.nlm.nih.gov/compound/46703947#section=2D-Structure
https://pubchem.ncbi.nlm.nih.gov/compound/46661#section=2D-Structure
https://pubchem.ncbi.nlm.nih.gov/compound/46500630#section=2D-Structure
https://pubchem.ncbi.nlm.nih.gov/compound/46500629#section=2D-Structure
https://pubchem.ncbi.nlm.nih.gov/compound/46500530#section=2D-Structure
https://pubchem.ncbi.nlm.nih.gov/compound/46500529#section=2D-Structure
https://pubchem.ncbi.nlm.nih.gov/compound/46500528#section=2D-Structure
https://pubchem.ncbi.nlm.nih.gov/compound/46222067#section=2D-Structure
https://pubchem.ncbi.nlm.nih.gov/compound/46187782#section=2D-Structure
https://pubchem.ncbi.nlm.nih.gov/compound/46187612#section=2D-Structure
https://pubchem.ncbi.nlm.nih.gov/compound/46187429#section=2D-Structure
https://pubchem.ncbi.nlm.nih.gov/compound/4606838#section=2D-Structure
https://pubchem.ncbi.nlm.nih.gov/compound/4568516#section=2D-Structure
https://pubchem.ncbi.nlm.nih.gov/compound/45490344#section=2D-Structure
https://pubchem.ncbi.nlm.nih.gov/compound/4543920#section=2D-Structure
https://pubchem.ncbi.nlm.nih.gov/compound/454147#section=2D-Structure
https://pubchem.ncbi.nlm.nih.gov/compound/45272779#section=2D-Structure
https://pubchem.ncbi.nlm.nih.gov/compound/452587#section=2D-Structure
https://pubchem.ncbi.nlm.nih.gov/compound/45117472#section=2D-Structure
https://pubchem.ncbi.nlm.nih.gov/compound/45116585#section=2D-Structure
https://pubchem.ncbi.nlm.nih.gov/compound/45116584#section=2D-Structure
https://pubchem.ncbi.nlm.nih.gov/compound/45116578#section=2D-Structure
https://pubchem.ncbi.nlm.nih.gov/compound/45097193#section=2D-Structure
https://pubchem.ncbi.nlm.nih.gov/compound/448005#section=2D-Structure
https://pubchem.ncbi.nlm.nih.gov/compound/447875#section=2D-Structure
https://pubchem.ncbi.nlm.nih.gov/compound/44753056#section=2D-Structure
https://pubchem.ncbi.nlm.nih.gov/compound/44725541#section=2D-Structure
https://pubchem.ncbi.nlm.nih.gov/compound/447052#section=2D-Structure
https://pubchem.ncbi.nlm.nih.gov/compound/44576110#section=2D-Structure
https://pubchem.ncbi.nlm.nih.gov/compound/445479#section=2D-Structure
https://pubchem.ncbi.nlm.nih.gov/compound/44537034#section=2D-Structure
https://pubchem.ncbi.nlm.nih.gov/compound/44435209#section=2D-Structure
https://pubchem.ncbi.nlm.nih.gov/compound/44435207#section=2D-Structure
https://pubchem.ncbi.nlm.nih.gov/compound/44435205#section=2D-Structure
https://pubchem.ncbi.nlm.nih.gov/compound/44427#section=2D-Structure
https://pubchem.ncbi.nlm.nih.gov/compound/44425166#section=2D-Structure
https://pubchem.ncbi.nlm.nih.gov/compound/44393#section=2D-Structure
https://pubchem.ncbi.nlm.nih.gov/compound/44392#section=2D-Structure
https://pubchem.ncbi.nlm.nih.gov/compound/44377811#section=2D-Structure
https://pubchem.ncbi.nlm.nih.gov/compound/44376#section=2D-Structure
https://pubchem.ncbi.nlm.nih.gov/compound/44368#section=2D-Structure
https://pubchem.ncbi.nlm.nih.gov/compound/44367#section=2D-Structure
https://pubchem.ncbi.nlm.nih.gov/compound/44364503#section=2D-Structure
https://pubchem.ncbi.nlm.nih.gov/compound/44349676#section=2D-Structure
https://pubchem.ncbi.nlm.nih.gov/compound/44311#section=2D-Structure
https://pubchem.ncbi.nlm.nih.gov/compound/44306#section=2D-Structure
https://pubchem.ncbi.nlm.nih.gov/compound/44274#section=2D-Structure
https://pubchem.ncbi.nlm.nih.gov/compound/44273#section=2D-Structure
https://pubchem.ncbi.nlm.nih.gov/compound/44271#section=2D-Structure

https://pubchem.ncbi.nlm.nih.gov/compound/441776#section=2D-Structure
https://pubchem.ncbi.nlm.nih.gov/compound/44154976#section=2D-Structure
https://pubchem.ncbi.nlm.nih.gov/compound/44151332#section=2D-Structure
https://pubchem.ncbi.nlm.nih.gov/compound/44147#section=2D-Structure
https://pubchem.ncbi.nlm.nih.gov/compound/44107#section=2D-Structure
https://pubchem.ncbi.nlm.nih.gov/compound/438794#section=2D-Structure
https://pubchem.ncbi.nlm.nih.gov/compound/438763#section=2D-Structure
https://pubchem.ncbi.nlm.nih.gov/compound/438755#section=2D-Structure
https://pubchem.ncbi.nlm.nih.gov/compound/4385768#section=2D-Structure
https://pubchem.ncbi.nlm.nih.gov/compound/43770#section=2D-Structure
https://pubchem.ncbi.nlm.nih.gov/compound/436975#section=2D-Structure
https://pubchem.ncbi.nlm.nih.gov/compound/435407#section=2D-Structure
https://pubchem.ncbi.nlm.nih.gov/compound/43513#section=2D-Structure
https://pubchem.ncbi.nlm.nih.gov/compound/43512#section=2D-Structure
https://pubchem.ncbi.nlm.nih.gov/compound/435038#section=2D-Structure
https://pubchem.ncbi.nlm.nih.gov/compound/43494#section=2D-Structure
https://pubchem.ncbi.nlm.nih.gov/compound/430348#section=2D-Structure
https://pubchem.ncbi.nlm.nih.gov/compound/430294#section=2D-Structure
https://pubchem.ncbi.nlm.nih.gov/compound/429792#section=2D-Structure
https://pubchem.ncbi.nlm.nih.gov/compound/429757#section=2D-Structure
https://pubchem.ncbi.nlm.nih.gov/compound/423064#section=2D-Structure
https://pubchem.ncbi.nlm.nih.gov/compound/423038#section=2D-Structure
https://pubchem.ncbi.nlm.nih.gov/compound/42267#section=2D-Structure
https://pubchem.ncbi.nlm.nih.gov/compound/4222770#section=2D-Structure
https://pubchem.ncbi.nlm.nih.gov/compound/42030#section=2D-Structure
https://pubchem.ncbi.nlm.nih.gov/compound/42029#section=2D-Structure
https://pubchem.ncbi.nlm.nih.gov/compound/42028#section=2D-Structure
https://pubchem.ncbi.nlm.nih.gov/compound/42027#section=2D-Structure
https://pubchem.ncbi.nlm.nih.gov/compound/4200562#section=2D-Structure
https://pubchem.ncbi.nlm.nih.gov/compound/419914#section=2D-Structure
https://pubchem.ncbi.nlm.nih.gov/compound/419082#section=2D-Structure
https://pubchem.ncbi.nlm.nih.gov/compound/418491#section=2D-Structure
https://pubchem.ncbi.nlm.nih.gov/compound/417151#section=2D-Structure
https://pubchem.ncbi.nlm.nih.gov/compound/4167056#section=2D-Structure
https://pubchem.ncbi.nlm.nih.gov/compound/4166526#section=2D-Structure
https://pubchem.ncbi.nlm.nih.gov/compound/416285#section=2D-Structure
https://pubchem.ncbi.nlm.nih.gov/compound/4154735#section=2D-Structure
https://pubchem.ncbi.nlm.nih.gov/compound/415201#section=2D-Structure
https://pubchem.ncbi.nlm.nih.gov/compound/414853#section=2D-Structure
https://pubchem.ncbi.nlm.nih.gov/compound/41165#section=2D-Structure
https://pubchem.ncbi.nlm.nih.gov/compound/4104523#section=2D-Structure
https://pubchem.ncbi.nlm.nih.gov/compound/4101471#section=2D-Structure
https://pubchem.ncbi.nlm.nih.gov/compound/410099#section=2D-Structure
https://pubchem.ncbi.nlm.nih.gov/compound/405331#section=2D-Structure
https://pubchem.ncbi.nlm.nih.gov/compound/40513#section=2D-Structure
https://pubchem.ncbi.nlm.nih.gov/compound/4038769#section=2D-Structure
https://pubchem.ncbi.nlm.nih.gov/compound/4035806#section=2D-Structure
https://pubchem.ncbi.nlm.nih.gov/compound/3988679#section=2D-Structure
https://pubchem.ncbi.nlm.nih.gov/compound/3971577#section=2D-Structure
https://pubchem.ncbi.nlm.nih.gov/compound/395931#section=2D-Structure
https://pubchem.ncbi.nlm.nih.gov/compound/395928#section=2D-Structure
https://pubchem.ncbi.nlm.nih.gov/compound/39354402#section=2D-Structure
https://pubchem.ncbi.nlm.nih.gov/compound/3912411#section=2D-Structure
https://pubchem.ncbi.nlm.nih.gov/compound/38520#section=2D-Structure
https://pubchem.ncbi.nlm.nih.gov/compound/38497#section=2D-Structure

https://pubchem.ncbi.nlm.nih.gov/compound/3835463#section=2D-Structure
https://pubchem.ncbi.nlm.nih.gov/compound/382590#section=2D-Structure
https://pubchem.ncbi.nlm.nih.gov/compound/3825719#section=2D-Structure
https://pubchem.ncbi.nlm.nih.gov/compound/3805899#section=2D-Structure
https://pubchem.ncbi.nlm.nih.gov/compound/379117#section=2D-Structure
https://pubchem.ncbi.nlm.nih.gov/compound/379115#section=2D-Structure
https://pubchem.ncbi.nlm.nih.gov/compound/379114#section=2D-Structure
https://pubchem.ncbi.nlm.nih.gov/compound/37880#section=2D-Structure
https://pubchem.ncbi.nlm.nih.gov/compound/3787266#section=2D-Structure
https://pubchem.ncbi.nlm.nih.gov/compound/378356#section=2D-Structure
https://pubchem.ncbi.nlm.nih.gov/compound/37787#section=2D-Structure
https://pubchem.ncbi.nlm.nih.gov/compound/3768681#section=2D-Structure
https://pubchem.ncbi.nlm.nih.gov/compound/3762072#section=2D-Structure
https://pubchem.ncbi.nlm.nih.gov/compound/375143#section=2D-Structure
https://pubchem.ncbi.nlm.nih.gov/compound/375025#section=2D-Structure
https://pubchem.ncbi.nlm.nih.gov/compound/37455#section=2D-Structure
https://pubchem.ncbi.nlm.nih.gov/compound/374034#section=2D-Structure
https://pubchem.ncbi.nlm.nih.gov/compound/372119#section=2D-Structure
https://pubchem.ncbi.nlm.nih.gov/compound/3716443#section=2D-Structure
https://pubchem.ncbi.nlm.nih.gov/compound/36918#section=2D-Structure
https://pubchem.ncbi.nlm.nih.gov/compound/3678092#section=2D-Structure
https://pubchem.ncbi.nlm.nih.gov/compound/36636#section=2D-Structure
https://pubchem.ncbi.nlm.nih.gov/compound/36631#section=2D-Structure
https://pubchem.ncbi.nlm.nih.gov/compound/36630#section=2D-Structure
https://pubchem.ncbi.nlm.nih.gov/compound/364062#section=2D-Structure
https://pubchem.ncbi.nlm.nih.gov/compound/363747#section=2D-Structure
https://pubchem.ncbi.nlm.nih.gov/compound/3635696#section=2D-Structure
https://pubchem.ncbi.nlm.nih.gov/compound/363004#section=2D-Structure
https://pubchem.ncbi.nlm.nih.gov/compound/3613107#section=2D-Structure
https://pubchem.ncbi.nlm.nih.gov/compound/361134#section=2D-Structure
https://pubchem.ncbi.nlm.nih.gov/compound/3605995#section=2D-Structure
https://pubchem.ncbi.nlm.nih.gov/compound/360398#section=2D-Structure
https://pubchem.ncbi.nlm.nih.gov/compound/35861#section=2D-Structure
https://pubchem.ncbi.nlm.nih.gov/compound/356231#section=2D-Structure
https://pubchem.ncbi.nlm.nih.gov/compound/353429#section=2D-Structure
https://pubchem.ncbi.nlm.nih.gov/compound/3515568#section=2D-Structure
https://pubchem.ncbi.nlm.nih.gov/compound/348142#section=2D-Structure
https://pubchem.ncbi.nlm.nih.gov/compound/34809#section=2D-Structure
https://pubchem.ncbi.nlm.nih.gov/compound/345313#section=2D-Structure
https://pubchem.ncbi.nlm.nih.gov/compound/344235#section=2D-Structure
https://pubchem.ncbi.nlm.nih.gov/compound/34341#section=2D-Structure
https://pubchem.ncbi.nlm.nih.gov/compound/34327#section=2D-Structure
https://pubchem.ncbi.nlm.nih.gov/compound/342649#section=2D-Structure
https://pubchem.ncbi.nlm.nih.gov/compound/34232#section=2D-Structure
https://pubchem.ncbi.nlm.nih.gov/compound/33929#section=2D-Structure
https://pubchem.ncbi.nlm.nih.gov/compound/33777663#section=2D-Structure
https://pubchem.ncbi.nlm.nih.gov/compound/337601#section=2D-Structure
https://pubchem.ncbi.nlm.nih.gov/compound/337259#section=2D-Structure
https://pubchem.ncbi.nlm.nih.gov/compound/3339960#section=2D-Structure
https://pubchem.ncbi.nlm.nih.gov/compound/330914#section=2D-Structure
https://pubchem.ncbi.nlm.nih.gov/compound/32751#section=2D-Structure
https://pubchem.ncbi.nlm.nih.gov/compound/325056#section=2D-Structure
https://pubchem.ncbi.nlm.nih.gov/compound/32463#section=2D-Structure
https://pubchem.ncbi.nlm.nih.gov/compound/3238042#section=2D-Structure
https://pubchem.ncbi.nlm.nih.gov/compound/32191#section=2D-Structure

https://pubchem.ncbi.nlm.nih.gov/compound/321796#section=2D-Structure
https://pubchem.ncbi.nlm.nih.gov/compound/319721#section=2D-Structure
https://pubchem.ncbi.nlm.nih.gov/compound/316765#section=2D-Structure
https://pubchem.ncbi.nlm.nih.gov/compound/315033#section=2D-Structure
https://pubchem.ncbi.nlm.nih.gov/compound/31412#section=2D-Structure
https://pubchem.ncbi.nlm.nih.gov/compound/313353#section=2D-Structure
https://pubchem.ncbi.nlm.nih.gov/compound/310601#section=2D-Structure
https://pubchem.ncbi.nlm.nih.gov/compound/310600#section=2D-Structure
https://pubchem.ncbi.nlm.nih.gov/compound/308381#section=2D-Structure
https://pubchem.ncbi.nlm.nih.gov/compound/3081928#section=2D-Structure
https://pubchem.ncbi.nlm.nih.gov/compound/3047213#section=2D-Structure
https://pubchem.ncbi.nlm.nih.gov/compound/3047212#section=2D-Structure
https://pubchem.ncbi.nlm.nih.gov/compound/303943#section=2D-Structure
https://pubchem.ncbi.nlm.nih.gov/compound/3037425#section=2D-Structure
https://pubchem.ncbi.nlm.nih.gov/compound/3036603#section=2D-Structure
https://pubchem.ncbi.nlm.nih.gov/compound/3035232#section=2D-Structure
https://pubchem.ncbi.nlm.nih.gov/compound/3033969#section=2D-Structure
https://pubchem.ncbi.nlm.nih.gov/compound/3033721#section=2D-Structure
https://pubchem.ncbi.nlm.nih.gov/compound/3032356#section=2D-Structure
https://pubchem.ncbi.nlm.nih.gov/compound/301511#section=2D-Structure
https://pubchem.ncbi.nlm.nih.gov/compound/3014012#section=2D-Structure
https://pubchem.ncbi.nlm.nih.gov/compound/3011792#section=2D-Structure
https://pubchem.ncbi.nlm.nih.gov/compound/3010850#section=2D-Structure
https://pubchem.ncbi.nlm.nih.gov/compound/300390#section=2D-Structure
https://pubchem.ncbi.nlm.nih.gov/compound/300386#section=2D-Structure
https://pubchem.ncbi.nlm.nih.gov/compound/299466#section=2D-Structure
https://pubchem.ncbi.nlm.nih.gov/compound/299132#section=2D-Structure
https://pubchem.ncbi.nlm.nih.gov/compound/298500#section=2D-Structure
https://pubchem.ncbi.nlm.nih.gov/compound/298497#section=2D-Structure
https://pubchem.ncbi.nlm.nih.gov/compound/298496#section=2D-Structure
https://pubchem.ncbi.nlm.nih.gov/compound/294677#section=2D-Structure
https://pubchem.ncbi.nlm.nih.gov/compound/290521#section=2D-Structure
https://pubchem.ncbi.nlm.nih.gov/compound/290520#section=2D-Structure
https://pubchem.ncbi.nlm.nih.gov/compound/290519#section=2D-Structure
https://pubchem.ncbi.nlm.nih.gov/compound/290518#section=2D-Structure
https://pubchem.ncbi.nlm.nih.gov/compound/290517#section=2D-Structure
https://pubchem.ncbi.nlm.nih.gov/compound/289476#section=2D-Structure
https://pubchem.ncbi.nlm.nih.gov/compound/288414#section=2D-Structure
https://pubchem.ncbi.nlm.nih.gov/compound/28809352#section=2D-Structure
https://pubchem.ncbi.nlm.nih.gov/compound/28599#section=2D-Structure
https://pubchem.ncbi.nlm.nih.gov/compound/28598#section=2D-Structure
https://pubchem.ncbi.nlm.nih.gov/compound/285956#section=2D-Structure
https://pubchem.ncbi.nlm.nih.gov/compound/2854495#section=2D-Structure
https://pubchem.ncbi.nlm.nih.gov/compound/2844783#section=2D-Structure
https://pubchem.ncbi.nlm.nih.gov/compound/2833910#section=2D-Structure
https://pubchem.ncbi.nlm.nih.gov/compound/2825690#section=2D-Structure
https://pubchem.ncbi.nlm.nih.gov/compound/28047#section=2D-Structure
https://pubchem.ncbi.nlm.nih.gov/compound/28046#section=2D-Structure
https://pubchem.ncbi.nlm.nih.gov/compound/28045#section=2D-Structure
https://pubchem.ncbi.nlm.nih.gov/compound/28044#section=2D-Structure
https://pubchem.ncbi.nlm.nih.gov/compound/28043#section=2D-Structure
https://pubchem.ncbi.nlm.nih.gov/compound/28042#section=2D-Structure
https://pubchem.ncbi.nlm.nih.gov/compound/28041#section=2D-Structure
https://pubchem.ncbi.nlm.nih.gov/compound/28040#section=2D-Structure
https://pubchem.ncbi.nlm.nih.gov/compound/28039#section=2D-Structure

https://pubchem.ncbi.nlm.nih.gov/compound/28038#section=2D-Structure
https://pubchem.ncbi.nlm.nih.gov/compound/28037#section=2D-Structure
https://pubchem.ncbi.nlm.nih.gov/compound/28036#section=2D-Structure
https://pubchem.ncbi.nlm.nih.gov/compound/279441#section=2D-Structure
https://pubchem.ncbi.nlm.nih.gov/compound/27931#section=2D-Structure
https://pubchem.ncbi.nlm.nih.gov/compound/27930#section=2D-Structure
https://pubchem.ncbi.nlm.nih.gov/compound/279247#section=2D-Structure
https://pubchem.ncbi.nlm.nih.gov/compound/279237#section=2D-Structure
https://pubchem.ncbi.nlm.nih.gov/compound/2784130#section=2D-Structure
https://pubchem.ncbi.nlm.nih.gov/compound/2763073#section=2D-Structure
https://pubchem.ncbi.nlm.nih.gov/compound/2753212#section=2D-Structure
https://pubchem.ncbi.nlm.nih.gov/compound/2752026#section=2D-Structure
https://pubchem.ncbi.nlm.nih.gov/compound/2752024#section=2D-Structure
https://pubchem.ncbi.nlm.nih.gov/compound/2752022#section=2D-Structure
https://pubchem.ncbi.nlm.nih.gov/compound/2751919#section=2D-Structure
https://pubchem.ncbi.nlm.nih.gov/compound/2749329#section=2D-Structure
https://pubchem.ncbi.nlm.nih.gov/compound/2748018#section=2D-Structure
https://pubchem.ncbi.nlm.nih.gov/compound/2724868#section=2D-Structure
https://pubchem.ncbi.nlm.nih.gov/compound/26592#section=2D-Structure
https://pubchem.ncbi.nlm.nih.gov/compound/264192#section=2D-Structure
https://pubchem.ncbi.nlm.nih.gov/compound/261404#section=2D-Structure
https://pubchem.ncbi.nlm.nih.gov/compound/261401#section=2D-Structure
https://pubchem.ncbi.nlm.nih.gov/compound/261385#section=2D-Structure
https://pubchem.ncbi.nlm.nih.gov/compound/259449#section=2D-Structure
https://pubchem.ncbi.nlm.nih.gov/compound/25893#section=2D-Structure
https://pubchem.ncbi.nlm.nih.gov/compound/25892#section=2D-Structure
https://pubchem.ncbi.nlm.nih.gov/compound/25891#section=2D-Structure
https://pubchem.ncbi.nlm.nih.gov/compound/25890#section=2D-Structure
https://pubchem.ncbi.nlm.nih.gov/compound/255771#section=2D-Structure
https://pubchem.ncbi.nlm.nih.gov/compound/254115#section=2D-Structure
https://pubchem.ncbi.nlm.nih.gov/compound/254112#section=2D-Structure
https://pubchem.ncbi.nlm.nih.gov/compound/254111#section=2D-Structure
https://pubchem.ncbi.nlm.nih.gov/compound/254109#section=2D-Structure
https://pubchem.ncbi.nlm.nih.gov/compound/25251873#section=2D-Structure
https://pubchem.ncbi.nlm.nih.gov/compound/25226449#section=2D-Structure
https://pubchem.ncbi.nlm.nih.gov/compound/25226081#section=2D-Structure
https://pubchem.ncbi.nlm.nih.gov/compound/25225324#section=2D-Structure
https://pubchem.ncbi.nlm.nih.gov/compound/25221889#section=2D-Structure
https://pubchem.ncbi.nlm.nih.gov/compound/25210346#section=2D-Structure
https://pubchem.ncbi.nlm.nih.gov/compound/25196100#section=2D-Structure
https://pubchem.ncbi.nlm.nih.gov/compound/25191#section=2D-Structure
https://pubchem.ncbi.nlm.nih.gov/compound/25185901#section=2D-Structure
https://pubchem.ncbi.nlm.nih.gov/compound/25146402#section=2D-Structure
https://pubchem.ncbi.nlm.nih.gov/compound/25141297#section=2D-Structure
https://pubchem.ncbi.nlm.nih.gov/compound/25141296#section=2D-Structure
https://pubchem.ncbi.nlm.nih.gov/compound/25137879#section=2D-Structure
https://pubchem.ncbi.nlm.nih.gov/compound/25132754#section=2D-Structure
https://pubchem.ncbi.nlm.nih.gov/compound/25126114#section=2D-Structure
https://pubchem.ncbi.nlm.nih.gov/compound/25093276#section=2D-Structure
https://pubchem.ncbi.nlm.nih.gov/compound/25093275#section=2D-Structure
https://pubchem.ncbi.nlm.nih.gov/compound/250770#section=2D-Structure
https://pubchem.ncbi.nlm.nih.gov/compound/25061002#section=2D-Structure
https://pubchem.ncbi.nlm.nih.gov/compound/24975586#section=2D-Structure
https://pubchem.ncbi.nlm.nih.gov/compound/24972506#section=2D-Structure
https://pubchem.ncbi.nlm.nih.gov/compound/24962332#section=2D-Structure

https://pubchem.ncbi.nlm.nih.gov/compound/24961442#section=2D-Structure
https://pubchem.ncbi.nlm.nih.gov/compound/249459#section=2D-Structure
https://pubchem.ncbi.nlm.nih.gov/compound/249457#section=2D-Structure
https://pubchem.ncbi.nlm.nih.gov/compound/24898901#section=2D-Structure
https://pubchem.ncbi.nlm.nih.gov/compound/24882705#section=2D-Structure
https://pubchem.ncbi.nlm.nih.gov/compound/24872712#section=2D-Structure
https://pubchem.ncbi.nlm.nih.gov/compound/24872606#section=2D-Structure
https://pubchem.ncbi.nlm.nih.gov/compound/24870811#section=2D-Structure
https://pubchem.ncbi.nlm.nih.gov/compound/248564#section=2D-Structure
https://pubchem.ncbi.nlm.nih.gov/compound/24854166#section=2D-Structure
https://pubchem.ncbi.nlm.nih.gov/compound/24837490#section=2D-Structure
https://pubchem.ncbi.nlm.nih.gov/compound/24837486#section=2D-Structure
https://pubchem.ncbi.nlm.nih.gov/compound/24829048#section=2D-Structure
https://pubchem.ncbi.nlm.nih.gov/compound/24828889#section=2D-Structure
https://pubchem.ncbi.nlm.nih.gov/compound/24828090#section=2D-Structure
https://pubchem.ncbi.nlm.nih.gov/compound/24809477#section=2D-Structure
https://pubchem.ncbi.nlm.nih.gov/compound/24809476#section=2D-Structure
https://pubchem.ncbi.nlm.nih.gov/compound/24808437#section=2D-Structure
https://pubchem.ncbi.nlm.nih.gov/compound/24787820#section=2D-Structure
https://pubchem.ncbi.nlm.nih.gov/compound/24787819#section=2D-Structure
https://pubchem.ncbi.nlm.nih.gov/compound/24787818#section=2D-Structure
https://pubchem.ncbi.nlm.nih.gov/compound/24765603#section=2D-Structure
https://pubchem.ncbi.nlm.nih.gov/compound/247409#section=2D-Structure
https://pubchem.ncbi.nlm.nih.gov/compound/24721598#section=2D-Structure
https://pubchem.ncbi.nlm.nih.gov/compound/24721155#section=2D-Structure
https://pubchem.ncbi.nlm.nih.gov/compound/246787#section=2D-Structure
https://pubchem.ncbi.nlm.nih.gov/compound/245880#section=2D-Structure
https://pubchem.ncbi.nlm.nih.gov/compound/24276230#section=2D-Structure
https://pubchem.ncbi.nlm.nih.gov/compound/24205099#section=2D-Structure
https://pubchem.ncbi.nlm.nih.gov/compound/24205096#section=2D-Structure
https://pubchem.ncbi.nlm.nih.gov/compound/24204693#section=2D-Structure
https://pubchem.ncbi.nlm.nih.gov/compound/24203784#section=2D-Structure
https://pubchem.ncbi.nlm.nih.gov/compound/24203780#section=2D-Structure
https://pubchem.ncbi.nlm.nih.gov/compound/24203778#section=2D-Structure
https://pubchem.ncbi.nlm.nih.gov/compound/24203777#section=2D-Structure
https://pubchem.ncbi.nlm.nih.gov/compound/23911#section=2D-Structure
https://pubchem.ncbi.nlm.nih.gov/compound/23633137#section=2D-Structure
https://pubchem.ncbi.nlm.nih.gov/compound/23625270#section=2D-Structure
https://pubchem.ncbi.nlm.nih.gov/compound/23625267#section=2D-Structure
https://pubchem.ncbi.nlm.nih.gov/compound/23623285#section=2D-Structure
https://pubchem.ncbi.nlm.nih.gov/compound/23623051#section=2D-Structure
https://pubchem.ncbi.nlm.nih.gov/compound/23623005#section=2D-Structure
https://pubchem.ncbi.nlm.nih.gov/compound/23622957#section=2D-Structure
https://pubchem.ncbi.nlm.nih.gov/compound/23622955#section=2D-Structure
https://pubchem.ncbi.nlm.nih.gov/compound/23622585#section=2D-Structure
https://pubchem.ncbi.nlm.nih.gov/compound/23622584#section=2D-Structure
https://pubchem.ncbi.nlm.nih.gov/compound/23621448#section=2D-Structure
https://pubchem.ncbi.nlm.nih.gov/compound/23619177#section=2D-Structure
https://pubchem.ncbi.nlm.nih.gov/compound/23619043#section=2D-Structure
https://pubchem.ncbi.nlm.nih.gov/compound/23617881#section=2D-Structure
https://pubchem.ncbi.nlm.nih.gov/compound/23617717#section=2D-Structure
https://pubchem.ncbi.nlm.nih.gov/compound/23616721#section=2D-Structure
https://pubchem.ncbi.nlm.nih.gov/compound/23616359#section=2D-Structure
https://pubchem.ncbi.nlm.nih.gov/compound/23616349#section=2D-Structure
https://pubchem.ncbi.nlm.nih.gov/compound/23576499#section=2D-Structure

https://pubchem.ncbi.nlm.nih.gov/compound/23573969#section=2D-Structure
https://pubchem.ncbi.nlm.nih.gov/compound/23547401#section=2D-Structure
https://pubchem.ncbi.nlm.nih.gov/compound/23539809#section=2D-Structure
https://pubchem.ncbi.nlm.nih.gov/compound/23530343#section=2D-Structure
https://pubchem.ncbi.nlm.nih.gov/compound/23521969#section=2D-Structure
https://pubchem.ncbi.nlm.nih.gov/compound/23521967#section=2D-Structure
https://pubchem.ncbi.nlm.nih.gov/compound/23520#section=2D-Structure
https://pubchem.ncbi.nlm.nih.gov/compound/23511680#section=2D-Structure
https://pubchem.ncbi.nlm.nih.gov/compound/23505254#section=2D-Structure
https://pubchem.ncbi.nlm.nih.gov/compound/23505253#section=2D-Structure
https://pubchem.ncbi.nlm.nih.gov/compound/23505246#section=2D-Structure
https://pubchem.ncbi.nlm.nih.gov/compound/23505243#section=2D-Structure
https://pubchem.ncbi.nlm.nih.gov/compound/23505242#section=2D-Structure
https://pubchem.ncbi.nlm.nih.gov/compound/23500244#section=2D-Structure
https://pubchem.ncbi.nlm.nih.gov/compound/23454174#section=2D-Structure
https://pubchem.ncbi.nlm.nih.gov/compound/23443751#section=2D-Structure
https://pubchem.ncbi.nlm.nih.gov/compound/23443379#section=2D-Structure
https://pubchem.ncbi.nlm.nih.gov/compound/23435228#section=2D-Structure
https://pubchem.ncbi.nlm.nih.gov/compound/23433647#section=2D-Structure
https://pubchem.ncbi.nlm.nih.gov/compound/23433593#section=2D-Structure
https://pubchem.ncbi.nlm.nih.gov/compound/23433522#section=2D-Structure
https://pubchem.ncbi.nlm.nih.gov/compound/23433518#section=2D-Structure
https://pubchem.ncbi.nlm.nih.gov/compound/23433490#section=2D-Structure
https://pubchem.ncbi.nlm.nih.gov/compound/23433445#section=2D-Structure
https://pubchem.ncbi.nlm.nih.gov/compound/23423814#section=2D-Structure
https://pubchem.ncbi.nlm.nih.gov/compound/23422095#section=2D-Structure
https://pubchem.ncbi.nlm.nih.gov/compound/23396588#section=2D-Structure
https://pubchem.ncbi.nlm.nih.gov/compound/23396586#section=2D-Structure
https://pubchem.ncbi.nlm.nih.gov/compound/23396585#section=2D-Structure
https://pubchem.ncbi.nlm.nih.gov/compound/23396583#section=2D-Structure
https://pubchem.ncbi.nlm.nih.gov/compound/23396581#section=2D-Structure
https://pubchem.ncbi.nlm.nih.gov/compound/23396579#section=2D-Structure
https://pubchem.ncbi.nlm.nih.gov/compound/23396576#section=2D-Structure
https://pubchem.ncbi.nlm.nih.gov/compound/23396575#section=2D-Structure
https://pubchem.ncbi.nlm.nih.gov/compound/23396568#section=2D-Structure
https://pubchem.ncbi.nlm.nih.gov/compound/23382645#section=2D-Structure
https://pubchem.ncbi.nlm.nih.gov/compound/2336#section=2D-Structure
https://pubchem.ncbi.nlm.nih.gov/compound/23358520#section=2D-Structure
https://pubchem.ncbi.nlm.nih.gov/compound/23358518#section=2D-Structure
https://pubchem.ncbi.nlm.nih.gov/compound/23340046#section=2D-Structure
https://pubchem.ncbi.nlm.nih.gov/compound/23337023#section=2D-Structure
https://pubchem.ncbi.nlm.nih.gov/compound/23335367#section=2D-Structure
https://pubchem.ncbi.nlm.nih.gov/compound/23333976#section=2D-Structure
https://pubchem.ncbi.nlm.nih.gov/compound/23322818#section=2D-Structure
https://pubchem.ncbi.nlm.nih.gov/compound/23322812#section=2D-Structure
https://pubchem.ncbi.nlm.nih.gov/compound/23310941#section=2D-Structure
https://pubchem.ncbi.nlm.nih.gov/compound/23310938#section=2D-Structure
https://pubchem.ncbi.nlm.nih.gov/compound/23301745#section=2D-Structure
https://pubchem.ncbi.nlm.nih.gov/compound/23293717#section=2D-Structure
https://pubchem.ncbi.nlm.nih.gov/compound/23282440#section=2D-Structure
https://pubchem.ncbi.nlm.nih.gov/compound/23270061#section=2D-Structure
https://pubchem.ncbi.nlm.nih.gov/compound/23270060#section=2D-Structure
https://pubchem.ncbi.nlm.nih.gov/compound/23261757#section=2D-Structure
https://pubchem.ncbi.nlm.nih.gov/compound/23251480#section=2D-Structure
https://pubchem.ncbi.nlm.nih.gov/compound/23239145#section=2D-Structure

https://pubchem.ncbi.nlm.nih.gov/compound/23235432#section=2D-Structure
https://pubchem.ncbi.nlm.nih.gov/compound/23235431#section=2D-Structure
https://pubchem.ncbi.nlm.nih.gov/compound/23218766#section=2D-Structure
https://pubchem.ncbi.nlm.nih.gov/compound/2321217#section=2D-Structure
https://pubchem.ncbi.nlm.nih.gov/compound/23185010#section=2D-Structure
https://pubchem.ncbi.nlm.nih.gov/compound/23185009#section=2D-Structure
https://pubchem.ncbi.nlm.nih.gov/compound/23168852#section=2D-Structure
https://pubchem.ncbi.nlm.nih.gov/compound/23154719#section=2D-Structure
https://pubchem.ncbi.nlm.nih.gov/compound/23152250#section=2D-Structure
https://pubchem.ncbi.nlm.nih.gov/compound/23144882#section=2D-Structure
https://pubchem.ncbi.nlm.nih.gov/compound/23131485#section=2D-Structure
https://pubchem.ncbi.nlm.nih.gov/compound/23131477#section=2D-Structure
https://pubchem.ncbi.nlm.nih.gov/compound/23131476#section=2D-Structure
https://pubchem.ncbi.nlm.nih.gov/compound/23131458#section=2D-Structure
https://pubchem.ncbi.nlm.nih.gov/compound/23131450#section=2D-Structure
https://pubchem.ncbi.nlm.nih.gov/compound/23131438#section=2D-Structure
https://pubchem.ncbi.nlm.nih.gov/compound/23131423#section=2D-Structure
https://pubchem.ncbi.nlm.nih.gov/compound/23131422#section=2D-Structure
https://pubchem.ncbi.nlm.nih.gov/compound/23131412#section=2D-Structure
https://pubchem.ncbi.nlm.nih.gov/compound/23131407#section=2D-Structure
https://pubchem.ncbi.nlm.nih.gov/compound/23131402#section=2D-Structure
https://pubchem.ncbi.nlm.nih.gov/compound/23131390#section=2D-Structure
https://pubchem.ncbi.nlm.nih.gov/compound/23131389#section=2D-Structure
https://pubchem.ncbi.nlm.nih.gov/compound/23131387#section=2D-Structure
https://pubchem.ncbi.nlm.nih.gov/compound/23131381#section=2D-Structure
https://pubchem.ncbi.nlm.nih.gov/compound/23131369#section=2D-Structure
https://pubchem.ncbi.nlm.nih.gov/compound/23131356#section=2D-Structure
https://pubchem.ncbi.nlm.nih.gov/compound/23131351#section=2D-Structure
https://pubchem.ncbi.nlm.nih.gov/compound/23131336#section=2D-Structure
https://pubchem.ncbi.nlm.nih.gov/compound/23131319#section=2D-Structure
https://pubchem.ncbi.nlm.nih.gov/compound/23131318#section=2D-Structure
https://pubchem.ncbi.nlm.nih.gov/compound/23131300#section=2D-Structure
https://pubchem.ncbi.nlm.nih.gov/compound/23131255#section=2D-Structure
https://pubchem.ncbi.nlm.nih.gov/compound/23131247#section=2D-Structure
https://pubchem.ncbi.nlm.nih.gov/compound/23131246#section=2D-Structure
https://pubchem.ncbi.nlm.nih.gov/compound/23131228#section=2D-Structure
https://pubchem.ncbi.nlm.nih.gov/compound/23131215#section=2D-Structure
https://pubchem.ncbi.nlm.nih.gov/compound/23110783#section=2D-Structure
https://pubchem.ncbi.nlm.nih.gov/compound/23110776#section=2D-Structure
https://pubchem.ncbi.nlm.nih.gov/compound/2309275#section=2D-Structure
https://pubchem.ncbi.nlm.nih.gov/compound/230906#section=2D-Structure
https://pubchem.ncbi.nlm.nih.gov/compound/23064644#section=2D-Structure
https://pubchem.ncbi.nlm.nih.gov/compound/23063603#section=2D-Structure
https://pubchem.ncbi.nlm.nih.gov/compound/23061528#section=2D-Structure
https://pubchem.ncbi.nlm.nih.gov/compound/23054#section=2D-Structure
https://pubchem.ncbi.nlm.nih.gov/compound/23051115#section=2D-Structure
https://pubchem.ncbi.nlm.nih.gov/compound/23044563#section=2D-Structure
https://pubchem.ncbi.nlm.nih.gov/compound/23032503#section=2D-Structure
https://pubchem.ncbi.nlm.nih.gov/compound/23032498#section=2D-Structure
https://pubchem.ncbi.nlm.nih.gov/compound/23018363#section=2D-Structure
https://pubchem.ncbi.nlm.nih.gov/compound/22999777#section=2D-Structure
https://pubchem.ncbi.nlm.nih.gov/compound/22966620#section=2D-Structure
https://pubchem.ncbi.nlm.nih.gov/compound/22966616#section=2D-Structure
https://pubchem.ncbi.nlm.nih.gov/compound/22964366#section=2D-Structure
https://pubchem.ncbi.nlm.nih.gov/compound/22951931#section=2D-Structure

https://pubchem.ncbi.nlm.nih.gov/compound/22950387#section=2D-Structure
https://pubchem.ncbi.nlm.nih.gov/compound/22950386#section=2D-Structure
https://pubchem.ncbi.nlm.nih.gov/compound/22950385#section=2D-Structure
https://pubchem.ncbi.nlm.nih.gov/compound/22950384#section=2D-Structure
https://pubchem.ncbi.nlm.nih.gov/compound/22950383#section=2D-Structure
https://pubchem.ncbi.nlm.nih.gov/compound/22950382#section=2D-Structure
https://pubchem.ncbi.nlm.nih.gov/compound/22950367#section=2D-Structure
https://pubchem.ncbi.nlm.nih.gov/compound/22950264#section=2D-Structure
https://pubchem.ncbi.nlm.nih.gov/compound/22896084#section=2D-Structure
https://pubchem.ncbi.nlm.nih.gov/compound/22890150#section=2D-Structure
https://pubchem.ncbi.nlm.nih.gov/compound/22886614#section=2D-Structure
https://pubchem.ncbi.nlm.nih.gov/compound/22765616#section=2D-Structure
https://pubchem.ncbi.nlm.nih.gov/compound/22757555#section=2D-Structure
https://pubchem.ncbi.nlm.nih.gov/compound/22754689#section=2D-Structure
https://pubchem.ncbi.nlm.nih.gov/compound/22750094#section=2D-Structure
https://pubchem.ncbi.nlm.nih.gov/compound/22743920#section=2D-Structure
https://pubchem.ncbi.nlm.nih.gov/compound/22720125#section=2D-Structure
https://pubchem.ncbi.nlm.nih.gov/compound/22720124#section=2D-Structure
https://pubchem.ncbi.nlm.nih.gov/compound/22720121#section=2D-Structure
https://pubchem.ncbi.nlm.nih.gov/compound/22675979#section=2D-Structure
https://pubchem.ncbi.nlm.nih.gov/compound/22672524#section=2D-Structure
https://pubchem.ncbi.nlm.nih.gov/compound/22660387#section=2D-Structure
https://pubchem.ncbi.nlm.nih.gov/compound/22660372#section=2D-Structure
https://pubchem.ncbi.nlm.nih.gov/compound/22646503#section=2D-Structure
https://pubchem.ncbi.nlm.nih.gov/compound/22646496#section=2D-Structure
https://pubchem.ncbi.nlm.nih.gov/compound/22646483#section=2D-Structure
https://pubchem.ncbi.nlm.nih.gov/compound/22646479#section=2D-Structure
https://pubchem.ncbi.nlm.nih.gov/compound/22646471#section=2D-Structure
https://pubchem.ncbi.nlm.nih.gov/compound/22625464#section=2D-Structure
https://pubchem.ncbi.nlm.nih.gov/compound/22625463#section=2D-Structure
https://pubchem.ncbi.nlm.nih.gov/compound/22625461#section=2D-Structure
https://pubchem.ncbi.nlm.nih.gov/compound/22624840#section=2D-Structure
https://pubchem.ncbi.nlm.nih.gov/compound/22618641#section=2D-Structure
https://pubchem.ncbi.nlm.nih.gov/compound/22616766#section=2D-Structure
https://pubchem.ncbi.nlm.nih.gov/compound/22599163#section=2D-Structure
https://pubchem.ncbi.nlm.nih.gov/compound/22599159#section=2D-Structure
https://pubchem.ncbi.nlm.nih.gov/compound/22599157#section=2D-Structure
https://pubchem.ncbi.nlm.nih.gov/compound/22599150#section=2D-Structure
https://pubchem.ncbi.nlm.nih.gov/compound/22599148#section=2D-Structure
https://pubchem.ncbi.nlm.nih.gov/compound/22573892#section=2D-Structure
https://pubchem.ncbi.nlm.nih.gov/compound/22568379#section=2D-Structure
https://pubchem.ncbi.nlm.nih.gov/compound/22501121#section=2D-Structure
https://pubchem.ncbi.nlm.nih.gov/compound/22483812#section=2D-Structure
https://pubchem.ncbi.nlm.nih.gov/compound/22457424#section=2D-Structure
https://pubchem.ncbi.nlm.nih.gov/compound/22448245#section=2D-Structure
https://pubchem.ncbi.nlm.nih.gov/compound/22395530#section=2D-Structure
https://pubchem.ncbi.nlm.nih.gov/compound/22395387#section=2D-Structure
https://pubchem.ncbi.nlm.nih.gov/compound/2238116#section=2D-Structure
https://pubchem.ncbi.nlm.nih.gov/compound/22356018#section=2D-Structure
https://pubchem.ncbi.nlm.nih.gov/compound/22349123#section=2D-Structure
https://pubchem.ncbi.nlm.nih.gov/compound/22349120#section=2D-Structure
https://pubchem.ncbi.nlm.nih.gov/compound/22349118#section=2D-Structure
https://pubchem.ncbi.nlm.nih.gov/compound/22345724#section=2D-Structure
https://pubchem.ncbi.nlm.nih.gov/compound/22315030#section=2D-Structure
https://pubchem.ncbi.nlm.nih.gov/compound/22295663#section=2D-Structure

https://pubchem.ncbi.nlm.nih.gov/compound/22294685#section=2D-Structure
https://pubchem.ncbi.nlm.nih.gov/compound/22294684#section=2D-Structure
https://pubchem.ncbi.nlm.nih.gov/compound/22290969#section=2D-Structure
https://pubchem.ncbi.nlm.nih.gov/compound/22269476#section=2D-Structure
https://pubchem.ncbi.nlm.nih.gov/compound/22269443#section=2D-Structure
https://pubchem.ncbi.nlm.nih.gov/compound/22262491#section=2D-Structure
https://pubchem.ncbi.nlm.nih.gov/compound/22259998#section=2D-Structure
https://pubchem.ncbi.nlm.nih.gov/compound/22245540#section=2D-Structure
https://pubchem.ncbi.nlm.nih.gov/compound/22245515#section=2D-Structure
https://pubchem.ncbi.nlm.nih.gov/compound/22222869#section=2D-Structure
https://pubchem.ncbi.nlm.nih.gov/compound/22222867#section=2D-Structure
https://pubchem.ncbi.nlm.nih.gov/compound/22218893#section=2D-Structure
https://pubchem.ncbi.nlm.nih.gov/compound/22211879#section=2D-Structure
https://pubchem.ncbi.nlm.nih.gov/compound/22178333#section=2D-Structure
https://pubchem.ncbi.nlm.nih.gov/compound/22178326#section=2D-Structure
https://pubchem.ncbi.nlm.nih.gov/compound/22178304#section=2D-Structure
https://pubchem.ncbi.nlm.nih.gov/compound/22178298#section=2D-Structure
https://pubchem.ncbi.nlm.nih.gov/compound/22178286#section=2D-Structure
https://pubchem.ncbi.nlm.nih.gov/compound/22178284#section=2D-Structure
https://pubchem.ncbi.nlm.nih.gov/compound/22178283#section=2D-Structure
https://pubchem.ncbi.nlm.nih.gov/compound/22178281#section=2D-Structure
https://pubchem.ncbi.nlm.nih.gov/compound/22178280#section=2D-Structure
https://pubchem.ncbi.nlm.nih.gov/compound/22178231#section=2D-Structure
https://pubchem.ncbi.nlm.nih.gov/compound/22178229#section=2D-Structure
https://pubchem.ncbi.nlm.nih.gov/compound/22178225#section=2D-Structure
https://pubchem.ncbi.nlm.nih.gov/compound/22151709#section=2D-Structure
https://pubchem.ncbi.nlm.nih.gov/compound/22151705#section=2D-Structure
https://pubchem.ncbi.nlm.nih.gov/compound/22151699#section=2D-Structure
https://pubchem.ncbi.nlm.nih.gov/compound/22151698#section=2D-Structure
https://pubchem.ncbi.nlm.nih.gov/compound/22151595#section=2D-Structure
https://pubchem.ncbi.nlm.nih.gov/compound/221502#section=2D-Structure
https://pubchem.ncbi.nlm.nih.gov/compound/22141566#section=2D-Structure
https://pubchem.ncbi.nlm.nih.gov/compound/22141563#section=2D-Structure
https://pubchem.ncbi.nlm.nih.gov/compound/22123973#section=2D-Structure
https://pubchem.ncbi.nlm.nih.gov/compound/22123488#section=2D-Structure
https://pubchem.ncbi.nlm.nih.gov/compound/22117953#section=2D-Structure
https://pubchem.ncbi.nlm.nih.gov/compound/22117579#section=2D-Structure
https://pubchem.ncbi.nlm.nih.gov/compound/22104332#section=2D-Structure
https://pubchem.ncbi.nlm.nih.gov/compound/221040#section=2D-Structure
https://pubchem.ncbi.nlm.nih.gov/compound/22090441#section=2D-Structure
https://pubchem.ncbi.nlm.nih.gov/compound/22064520#section=2D-Structure
https://pubchem.ncbi.nlm.nih.gov/compound/22062664#section=2D-Structure
https://pubchem.ncbi.nlm.nih.gov/compound/22051953#section=2D-Structure
https://pubchem.ncbi.nlm.nih.gov/compound/22051937#section=2D-Structure
https://pubchem.ncbi.nlm.nih.gov/compound/22046215#section=2D-Structure
https://pubchem.ncbi.nlm.nih.gov/compound/22034819#section=2D-Structure
https://pubchem.ncbi.nlm.nih.gov/compound/22034814#section=2D-Structure
https://pubchem.ncbi.nlm.nih.gov/compound/22034601#section=2D-Structure
https://pubchem.ncbi.nlm.nih.gov/compound/22023830#section=2D-Structure
https://pubchem.ncbi.nlm.nih.gov/compound/22022587#section=2D-Structure
https://pubchem.ncbi.nlm.nih.gov/compound/22013121#section=2D-Structure
https://pubchem.ncbi.nlm.nih.gov/compound/22006961#section=2D-Structure
https://pubchem.ncbi.nlm.nih.gov/compound/21999505#section=2D-Structure
https://pubchem.ncbi.nlm.nih.gov/compound/21999468#section=2D-Structure
https://pubchem.ncbi.nlm.nih.gov/compound/21983109#section=2D-Structure

https://pubchem.ncbi.nlm.nih.gov/compound/21983076#section=2D-Structure
https://pubchem.ncbi.nlm.nih.gov/compound/21982877#section=2D-Structure
https://pubchem.ncbi.nlm.nih.gov/compound/21978237#section=2D-Structure
https://pubchem.ncbi.nlm.nih.gov/compound/21960534#section=2D-Structure
https://pubchem.ncbi.nlm.nih.gov/compound/21960533#section=2D-Structure
https://pubchem.ncbi.nlm.nih.gov/compound/21956097#section=2D-Structure
https://pubchem.ncbi.nlm.nih.gov/compound/21955449#section=2D-Structure
https://pubchem.ncbi.nlm.nih.gov/compound/21949980#section=2D-Structure
https://pubchem.ncbi.nlm.nih.gov/compound/21946141#section=2D-Structure
https://pubchem.ncbi.nlm.nih.gov/compound/21946127#section=2D-Structure
https://pubchem.ncbi.nlm.nih.gov/compound/21946111#section=2D-Structure
https://pubchem.ncbi.nlm.nih.gov/compound/21946108#section=2D-Structure
https://pubchem.ncbi.nlm.nih.gov/compound/21944662#section=2D-Structure
https://pubchem.ncbi.nlm.nih.gov/compound/21930678#section=2D-Structure
https://pubchem.ncbi.nlm.nih.gov/compound/21918028#section=2D-Structure
https://pubchem.ncbi.nlm.nih.gov/compound/21907349#section=2D-Structure
https://pubchem.ncbi.nlm.nih.gov/compound/21901227#section=2D-Structure
https://pubchem.ncbi.nlm.nih.gov/compound/21896369#section=2D-Structure
https://pubchem.ncbi.nlm.nih.gov/compound/21892034#section=2D-Structure
https://pubchem.ncbi.nlm.nih.gov/compound/21891971#section=2D-Structure
https://pubchem.ncbi.nlm.nih.gov/compound/21888138#section=2D-Structure
https://pubchem.ncbi.nlm.nih.gov/compound/21888127#section=2D-Structure
https://pubchem.ncbi.nlm.nih.gov/compound/21888124#section=2D-Structure
https://pubchem.ncbi.nlm.nih.gov/compound/21888115#section=2D-Structure
https://pubchem.ncbi.nlm.nih.gov/compound/21888113#section=2D-Structure
https://pubchem.ncbi.nlm.nih.gov/compound/21888108#section=2D-Structure
https://pubchem.ncbi.nlm.nih.gov/compound/21888102#section=2D-Structure
https://pubchem.ncbi.nlm.nih.gov/compound/21888099#section=2D-Structure
https://pubchem.ncbi.nlm.nih.gov/compound/21888088#section=2D-Structure
https://pubchem.ncbi.nlm.nih.gov/compound/21888084#section=2D-Structure
https://pubchem.ncbi.nlm.nih.gov/compound/21888083#section=2D-Structure
https://pubchem.ncbi.nlm.nih.gov/compound/21888074#section=2D-Structure
https://pubchem.ncbi.nlm.nih.gov/compound/21888063#section=2D-Structure
https://pubchem.ncbi.nlm.nih.gov/compound/21888025#section=2D-Structure
https://pubchem.ncbi.nlm.nih.gov/compound/21888024#section=2D-Structure
https://pubchem.ncbi.nlm.nih.gov/compound/21888012#section=2D-Structure
https://pubchem.ncbi.nlm.nih.gov/compound/21888004#section=2D-Structure
https://pubchem.ncbi.nlm.nih.gov/compound/21888002#section=2D-Structure
https://pubchem.ncbi.nlm.nih.gov/compound/21888001#section=2D-Structure
https://pubchem.ncbi.nlm.nih.gov/compound/21887984#section=2D-Structure
https://pubchem.ncbi.nlm.nih.gov/compound/21887974#section=2D-Structure
https://pubchem.ncbi.nlm.nih.gov/compound/21887969#section=2D-Structure
https://pubchem.ncbi.nlm.nih.gov/compound/21887954#section=2D-Structure
https://pubchem.ncbi.nlm.nih.gov/compound/21887953#section=2D-Structure
https://pubchem.ncbi.nlm.nih.gov/compound/21887947#section=2D-Structure
https://pubchem.ncbi.nlm.nih.gov/compound/21887946#section=2D-Structure
https://pubchem.ncbi.nlm.nih.gov/compound/21887936#section=2D-Structure
https://pubchem.ncbi.nlm.nih.gov/compound/21887932#section=2D-Structure
https://pubchem.ncbi.nlm.nih.gov/compound/21887926#section=2D-Structure
https://pubchem.ncbi.nlm.nih.gov/compound/21887924#section=2D-Structure
https://pubchem.ncbi.nlm.nih.gov/compound/21887913#section=2D-Structure
https://pubchem.ncbi.nlm.nih.gov/compound/21887893#section=2D-Structure
https://pubchem.ncbi.nlm.nih.gov/compound/21887873#section=2D-Structure
https://pubchem.ncbi.nlm.nih.gov/compound/21887867#section=2D-Structure
https://pubchem.ncbi.nlm.nih.gov/compound/21887850#section=2D-Structure

https://pubchem.ncbi.nlm.nih.gov/compound/21887846#section=2D-Structure
https://pubchem.ncbi.nlm.nih.gov/compound/21887820#section=2D-Structure
https://pubchem.ncbi.nlm.nih.gov/compound/21887804#section=2D-Structure
https://pubchem.ncbi.nlm.nih.gov/compound/21887803#section=2D-Structure
https://pubchem.ncbi.nlm.nih.gov/compound/21887800#section=2D-Structure
https://pubchem.ncbi.nlm.nih.gov/compound/21887785#section=2D-Structure
https://pubchem.ncbi.nlm.nih.gov/compound/21887780#section=2D-Structure
https://pubchem.ncbi.nlm.nih.gov/compound/21887779#section=2D-Structure
https://pubchem.ncbi.nlm.nih.gov/compound/21887756#section=2D-Structure
https://pubchem.ncbi.nlm.nih.gov/compound/21887754#section=2D-Structure
https://pubchem.ncbi.nlm.nih.gov/compound/21887751#section=2D-Structure
https://pubchem.ncbi.nlm.nih.gov/compound/21887748#section=2D-Structure
https://pubchem.ncbi.nlm.nih.gov/compound/21887694#section=2D-Structure
https://pubchem.ncbi.nlm.nih.gov/compound/21887676#section=2D-Structure
https://pubchem.ncbi.nlm.nih.gov/compound/21887646#section=2D-Structure
https://pubchem.ncbi.nlm.nih.gov/compound/21887638#section=2D-Structure
https://pubchem.ncbi.nlm.nih.gov/compound/21887603#section=2D-Structure
https://pubchem.ncbi.nlm.nih.gov/compound/21887595#section=2D-Structure
https://pubchem.ncbi.nlm.nih.gov/compound/21887583#section=2D-Structure
https://pubchem.ncbi.nlm.nih.gov/compound/21887571#section=2D-Structure
https://pubchem.ncbi.nlm.nih.gov/compound/21887534#section=2D-Structure
https://pubchem.ncbi.nlm.nih.gov/compound/21887519#section=2D-Structure
https://pubchem.ncbi.nlm.nih.gov/compound/21887512#section=2D-Structure
https://pubchem.ncbi.nlm.nih.gov/compound/21887509#section=2D-Structure
https://pubchem.ncbi.nlm.nih.gov/compound/21887498#section=2D-Structure
https://pubchem.ncbi.nlm.nih.gov/compound/21887490#section=2D-Structure
https://pubchem.ncbi.nlm.nih.gov/compound/21887488#section=2D-Structure
https://pubchem.ncbi.nlm.nih.gov/compound/21887482#section=2D-Structure
https://pubchem.ncbi.nlm.nih.gov/compound/21887470#section=2D-Structure
https://pubchem.ncbi.nlm.nih.gov/compound/21887423#section=2D-Structure
https://pubchem.ncbi.nlm.nih.gov/compound/21887417#section=2D-Structure
https://pubchem.ncbi.nlm.nih.gov/compound/21887410#section=2D-Structure
https://pubchem.ncbi.nlm.nih.gov/compound/21887396#section=2D-Structure
https://pubchem.ncbi.nlm.nih.gov/compound/21887388#section=2D-Structure
https://pubchem.ncbi.nlm.nih.gov/compound/21887365#section=2D-Structure
https://pubchem.ncbi.nlm.nih.gov/compound/21887334#section=2D-Structure
https://pubchem.ncbi.nlm.nih.gov/compound/21887333#section=2D-Structure
https://pubchem.ncbi.nlm.nih.gov/compound/21887293#section=2D-Structure
https://pubchem.ncbi.nlm.nih.gov/compound/21887286#section=2D-Structure
https://pubchem.ncbi.nlm.nih.gov/compound/21887285#section=2D-Structure
https://pubchem.ncbi.nlm.nih.gov/compound/21887254#section=2D-Structure
https://pubchem.ncbi.nlm.nih.gov/compound/21887244#section=2D-Structure
https://pubchem.ncbi.nlm.nih.gov/compound/21887242#section=2D-Structure
https://pubchem.ncbi.nlm.nih.gov/compound/21887226#section=2D-Structure
https://pubchem.ncbi.nlm.nih.gov/compound/21887220#section=2D-Structure
https://pubchem.ncbi.nlm.nih.gov/compound/21887215#section=2D-Structure
https://pubchem.ncbi.nlm.nih.gov/compound/21887182#section=2D-Structure
https://pubchem.ncbi.nlm.nih.gov/compound/21887178#section=2D-Structure
https://pubchem.ncbi.nlm.nih.gov/compound/21887159#section=2D-Structure
https://pubchem.ncbi.nlm.nih.gov/compound/21887142#section=2D-Structure
https://pubchem.ncbi.nlm.nih.gov/compound/21887135#section=2D-Structure
https://pubchem.ncbi.nlm.nih.gov/compound/21887127#section=2D-Structure
https://pubchem.ncbi.nlm.nih.gov/compound/21887066#section=2D-Structure
https://pubchem.ncbi.nlm.nih.gov/compound/21887054#section=2D-Structure
https://pubchem.ncbi.nlm.nih.gov/compound/21887047#section=2D-Structure

https://pubchem.ncbi.nlm.nih.gov/compound/21886992#section=2D-Structure
https://pubchem.ncbi.nlm.nih.gov/compound/21886979#section=2D-Structure
https://pubchem.ncbi.nlm.nih.gov/compound/21886976#section=2D-Structure
https://pubchem.ncbi.nlm.nih.gov/compound/21886974#section=2D-Structure
https://pubchem.ncbi.nlm.nih.gov/compound/21886972#section=2D-Structure
https://pubchem.ncbi.nlm.nih.gov/compound/21886969#section=2D-Structure
https://pubchem.ncbi.nlm.nih.gov/compound/21886941#section=2D-Structure
https://pubchem.ncbi.nlm.nih.gov/compound/21886928#section=2D-Structure
https://pubchem.ncbi.nlm.nih.gov/compound/21886889#section=2D-Structure
https://pubchem.ncbi.nlm.nih.gov/compound/21886839#section=2D-Structure
https://pubchem.ncbi.nlm.nih.gov/compound/21886833#section=2D-Structure
https://pubchem.ncbi.nlm.nih.gov/compound/21886811#section=2D-Structure
https://pubchem.ncbi.nlm.nih.gov/compound/21886806#section=2D-Structure
https://pubchem.ncbi.nlm.nih.gov/compound/21886795#section=2D-Structure
https://pubchem.ncbi.nlm.nih.gov/compound/21886787#section=2D-Structure
https://pubchem.ncbi.nlm.nih.gov/compound/21882147#section=2D-Structure
https://pubchem.ncbi.nlm.nih.gov/compound/21863886#section=2D-Structure
https://pubchem.ncbi.nlm.nih.gov/compound/21863579#section=2D-Structure
https://pubchem.ncbi.nlm.nih.gov/compound/21819557#section=2D-Structure
https://pubchem.ncbi.nlm.nih.gov/compound/21818595#section=2D-Structure
https://pubchem.ncbi.nlm.nih.gov/compound/21818309#section=2D-Structure
https://pubchem.ncbi.nlm.nih.gov/compound/21817494#section=2D-Structure
https://pubchem.ncbi.nlm.nih.gov/compound/21817492#section=2D-Structure
https://pubchem.ncbi.nlm.nih.gov/compound/21815237#section=2D-Structure
https://pubchem.ncbi.nlm.nih.gov/compound/21813402#section=2D-Structure
https://pubchem.ncbi.nlm.nih.gov/compound/21795294#section=2D-Structure
https://pubchem.ncbi.nlm.nih.gov/compound/21795285#section=2D-Structure
https://pubchem.ncbi.nlm.nih.gov/compound/21782606#section=2D-Structure
https://pubchem.ncbi.nlm.nih.gov/compound/21782605#section=2D-Structure
https://pubchem.ncbi.nlm.nih.gov/compound/21779884#section=2D-Structure
https://pubchem.ncbi.nlm.nih.gov/compound/21779531#section=2D-Structure
https://pubchem.ncbi.nlm.nih.gov/compound/21719112#section=2D-Structure
https://pubchem.ncbi.nlm.nih.gov/compound/21709765#section=2D-Structure
https://pubchem.ncbi.nlm.nih.gov/compound/21709574#section=2D-Structure
https://pubchem.ncbi.nlm.nih.gov/compound/21700977#section=2D-Structure
https://pubchem.ncbi.nlm.nih.gov/compound/21698884#section=2D-Structure
https://pubchem.ncbi.nlm.nih.gov/compound/21698878#section=2D-Structure
https://pubchem.ncbi.nlm.nih.gov/compound/21698875#section=2D-Structure
https://pubchem.ncbi.nlm.nih.gov/compound/21694#section=2D-Structure
https://pubchem.ncbi.nlm.nih.gov/compound/21678166#section=2D-Structure
https://pubchem.ncbi.nlm.nih.gov/compound/21657672#section=2D-Structure
https://pubchem.ncbi.nlm.nih.gov/compound/21650044#section=2D-Structure
https://pubchem.ncbi.nlm.nih.gov/compound/216375#section=2D-Structure
https://pubchem.ncbi.nlm.nih.gov/compound/21628228#section=2D-Structure
https://pubchem.ncbi.nlm.nih.gov/compound/21617644#section=2D-Structure
https://pubchem.ncbi.nlm.nih.gov/compound/21617629#section=2D-Structure
https://pubchem.ncbi.nlm.nih.gov/compound/21617472#section=2D-Structure
https://pubchem.ncbi.nlm.nih.gov/compound/21617103#section=2D-Structure
https://pubchem.ncbi.nlm.nih.gov/compound/21616420#section=2D-Structure
https://pubchem.ncbi.nlm.nih.gov/compound/21611829#section=2D-Structure
https://pubchem.ncbi.nlm.nih.gov/compound/21599022#section=2D-Structure
https://pubchem.ncbi.nlm.nih.gov/compound/21577123#section=2D-Structure
https://pubchem.ncbi.nlm.nih.gov/compound/21527468#section=2D-Structure
https://pubchem.ncbi.nlm.nih.gov/compound/21518774#section=2D-Structure
https://pubchem.ncbi.nlm.nih.gov/compound/21513416#section=2D-Structure

https://pubchem.ncbi.nlm.nih.gov/compound/21495314#section=2D-Structure
https://pubchem.ncbi.nlm.nih.gov/compound/21480#section=2D-Structure
https://pubchem.ncbi.nlm.nih.gov/compound/21474919#section=2D-Structure
https://pubchem.ncbi.nlm.nih.gov/compound/21474904#section=2D-Structure
https://pubchem.ncbi.nlm.nih.gov/compound/21463813#section=2D-Structure
https://pubchem.ncbi.nlm.nih.gov/compound/21447175#section=2D-Structure
https://pubchem.ncbi.nlm.nih.gov/compound/21440306#section=2D-Structure
https://pubchem.ncbi.nlm.nih.gov/compound/21439888#section=2D-Structure
https://pubchem.ncbi.nlm.nih.gov/compound/21439868#section=2D-Structure
https://pubchem.ncbi.nlm.nih.gov/compound/21437451#section=2D-Structure
https://pubchem.ncbi.nlm.nih.gov/compound/21437448#section=2D-Structure
https://pubchem.ncbi.nlm.nih.gov/compound/21436827#section=2D-Structure
https://pubchem.ncbi.nlm.nih.gov/compound/21428477#section=2D-Structure
https://pubchem.ncbi.nlm.nih.gov/compound/21424377#section=2D-Structure
https://pubchem.ncbi.nlm.nih.gov/compound/21422906#section=2D-Structure
https://pubchem.ncbi.nlm.nih.gov/compound/21412865#section=2D-Structure
https://pubchem.ncbi.nlm.nih.gov/compound/21407026#section=2D-Structure
https://pubchem.ncbi.nlm.nih.gov/compound/21403134#section=2D-Structure
https://pubchem.ncbi.nlm.nih.gov/compound/21402#section=2D-Structure
https://pubchem.ncbi.nlm.nih.gov/compound/21395771#section=2D-Structure
https://pubchem.ncbi.nlm.nih.gov/compound/21351061#section=2D-Structure
https://pubchem.ncbi.nlm.nih.gov/compound/21335067#section=2D-Structure
https://pubchem.ncbi.nlm.nih.gov/compound/21290337#section=2D-Structure
https://pubchem.ncbi.nlm.nih.gov/compound/21282420#section=2D-Structure
https://pubchem.ncbi.nlm.nih.gov/compound/21258226#section=2D-Structure
https://pubchem.ncbi.nlm.nih.gov/compound/21245417#section=2D-Structure
https://pubchem.ncbi.nlm.nih.gov/compound/21226522#section=2D-Structure
https://pubchem.ncbi.nlm.nih.gov/compound/21219818#section=2D-Structure
https://pubchem.ncbi.nlm.nih.gov/compound/21203503#section=2D-Structure
https://pubchem.ncbi.nlm.nih.gov/compound/21197345#section=2D-Structure
https://pubchem.ncbi.nlm.nih.gov/compound/21197343#section=2D-Structure
https://pubchem.ncbi.nlm.nih.gov/compound/21197339#section=2D-Structure
https://pubchem.ncbi.nlm.nih.gov/compound/21197330#section=2D-Structure
https://pubchem.ncbi.nlm.nih.gov/compound/21197326#section=2D-Structure
https://pubchem.ncbi.nlm.nih.gov/compound/21197321#section=2D-Structure
https://pubchem.ncbi.nlm.nih.gov/compound/21187141#section=2D-Structure
https://pubchem.ncbi.nlm.nih.gov/compound/21183233#section=2D-Structure
https://pubchem.ncbi.nlm.nih.gov/compound/21179175#section=2D-Structure
https://pubchem.ncbi.nlm.nih.gov/compound/21155991#section=2D-Structure
https://pubchem.ncbi.nlm.nih.gov/compound/21152608#section=2D-Structure
https://pubchem.ncbi.nlm.nih.gov/compound/21146915#section=2D-Structure
https://pubchem.ncbi.nlm.nih.gov/compound/21144439#section=2D-Structure
https://pubchem.ncbi.nlm.nih.gov/compound/21140800#section=2D-Structure
https://pubchem.ncbi.nlm.nih.gov/compound/21140429#section=2D-Structure
https://pubchem.ncbi.nlm.nih.gov/compound/21138295#section=2D-Structure
https://pubchem.ncbi.nlm.nih.gov/compound/21138271#section=2D-Structure
https://pubchem.ncbi.nlm.nih.gov/compound/21126763#section=2D-Structure
https://pubchem.ncbi.nlm.nih.gov/compound/21126651#section=2D-Structure
https://pubchem.ncbi.nlm.nih.gov/compound/21125666#section=2D-Structure
https://pubchem.ncbi.nlm.nih.gov/compound/21125508#section=2D-Structure
https://pubchem.ncbi.nlm.nih.gov/compound/21125249#section=2D-Structure
https://pubchem.ncbi.nlm.nih.gov/compound/21123380#section=2D-Structure
https://pubchem.ncbi.nlm.nih.gov/compound/21123378#section=2D-Structure
https://pubchem.ncbi.nlm.nih.gov/compound/21123114#section=2D-Structure
https://pubchem.ncbi.nlm.nih.gov/compound/21122922#section=2D-Structure

https://pubchem.ncbi.nlm.nih.gov/compound/21122898#section=2D-Structure
https://pubchem.ncbi.nlm.nih.gov/compound/21122873#section=2D-Structure
https://pubchem.ncbi.nlm.nih.gov/compound/21122854#section=2D-Structure
https://pubchem.ncbi.nlm.nih.gov/compound/21122752#section=2D-Structure
https://pubchem.ncbi.nlm.nih.gov/compound/21122074#section=2D-Structure
https://pubchem.ncbi.nlm.nih.gov/compound/21122064#section=2D-Structure
https://pubchem.ncbi.nlm.nih.gov/compound/21121335#section=2D-Structure
https://pubchem.ncbi.nlm.nih.gov/compound/21121334#section=2D-Structure
https://pubchem.ncbi.nlm.nih.gov/compound/21121069#section=2D-Structure
https://pubchem.ncbi.nlm.nih.gov/compound/21120803#section=2D-Structure
https://pubchem.ncbi.nlm.nih.gov/compound/21120690#section=2D-Structure
https://pubchem.ncbi.nlm.nih.gov/compound/21120566#section=2D-Structure
https://pubchem.ncbi.nlm.nih.gov/compound/21120545#section=2D-Structure
https://pubchem.ncbi.nlm.nih.gov/compound/21120346#section=2D-Structure
https://pubchem.ncbi.nlm.nih.gov/compound/21119874#section=2D-Structure
https://pubchem.ncbi.nlm.nih.gov/compound/21119871#section=2D-Structure
https://pubchem.ncbi.nlm.nih.gov/compound/21119866#section=2D-Structure
https://pubchem.ncbi.nlm.nih.gov/compound/21117979#section=2D-Structure
https://pubchem.ncbi.nlm.nih.gov/compound/21116094#section=2D-Structure
https://pubchem.ncbi.nlm.nih.gov/compound/21115195#section=2D-Structure
https://pubchem.ncbi.nlm.nih.gov/compound/21088011#section=2D-Structure
https://pubchem.ncbi.nlm.nih.gov/compound/21088010#section=2D-Structure
https://pubchem.ncbi.nlm.nih.gov/compound/21088008#section=2D-Structure
https://pubchem.ncbi.nlm.nih.gov/compound/21088006#section=2D-Structure
https://pubchem.ncbi.nlm.nih.gov/compound/21088004#section=2D-Structure
https://pubchem.ncbi.nlm.nih.gov/compound/21088003#section=2D-Structure
https://pubchem.ncbi.nlm.nih.gov/compound/21087948#section=2D-Structure
https://pubchem.ncbi.nlm.nih.gov/compound/21087921#section=2D-Structure
https://pubchem.ncbi.nlm.nih.gov/compound/21087916#section=2D-Structure
https://pubchem.ncbi.nlm.nih.gov/compound/21087910#section=2D-Structure
https://pubchem.ncbi.nlm.nih.gov/compound/21087893#section=2D-Structure
https://pubchem.ncbi.nlm.nih.gov/compound/21087888#section=2D-Structure
https://pubchem.ncbi.nlm.nih.gov/compound/21087869#section=2D-Structure
https://pubchem.ncbi.nlm.nih.gov/compound/21087867#section=2D-Structure
https://pubchem.ncbi.nlm.nih.gov/compound/21087864#section=2D-Structure
https://pubchem.ncbi.nlm.nih.gov/compound/21087850#section=2D-Structure
https://pubchem.ncbi.nlm.nih.gov/compound/21087840#section=2D-Structure
https://pubchem.ncbi.nlm.nih.gov/compound/21087839#section=2D-Structure
https://pubchem.ncbi.nlm.nih.gov/compound/21087838#section=2D-Structure
https://pubchem.ncbi.nlm.nih.gov/compound/21087837#section=2D-Structure
https://pubchem.ncbi.nlm.nih.gov/compound/21087836#section=2D-Structure
https://pubchem.ncbi.nlm.nih.gov/compound/21087835#section=2D-Structure
https://pubchem.ncbi.nlm.nih.gov/compound/21087834#section=2D-Structure
https://pubchem.ncbi.nlm.nih.gov/compound/21087833#section=2D-Structure
https://pubchem.ncbi.nlm.nih.gov/compound/21087832#section=2D-Structure
https://pubchem.ncbi.nlm.nih.gov/compound/21087827#section=2D-Structure
https://pubchem.ncbi.nlm.nih.gov/compound/21083035#section=2D-Structure
https://pubchem.ncbi.nlm.nih.gov/compound/21083034#section=2D-Structure
https://pubchem.ncbi.nlm.nih.gov/compound/21082949#section=2D-Structure
https://pubchem.ncbi.nlm.nih.gov/compound/21082937#section=2D-Structure
https://pubchem.ncbi.nlm.nih.gov/compound/21082934#section=2D-Structure
https://pubchem.ncbi.nlm.nih.gov/compound/21082#section=2D-Structure
https://pubchem.ncbi.nlm.nih.gov/compound/21050939#section=2D-Structure
https://pubchem.ncbi.nlm.nih.gov/compound/21029095#section=2D-Structure
https://pubchem.ncbi.nlm.nih.gov/compound/21023214#section=2D-Structure

https://pubchem.ncbi.nlm.nih.gov/compound/20975518#section=2D-Structure
https://pubchem.ncbi.nlm.nih.gov/compound/20841697#section=2D-Structure
https://pubchem.ncbi.nlm.nih.gov/compound/20823236#section=2D-Structure
https://pubchem.ncbi.nlm.nih.gov/compound/20823235#section=2D-Structure
https://pubchem.ncbi.nlm.nih.gov/compound/20823234#section=2D-Structure
https://pubchem.ncbi.nlm.nih.gov/compound/20823231#section=2D-Structure
https://pubchem.ncbi.nlm.nih.gov/compound/20823229#section=2D-Structure
https://pubchem.ncbi.nlm.nih.gov/compound/20823228#section=2D-Structure
https://pubchem.ncbi.nlm.nih.gov/compound/20823227#section=2D-Structure
https://pubchem.ncbi.nlm.nih.gov/compound/20823224#section=2D-Structure
https://pubchem.ncbi.nlm.nih.gov/compound/20823223#section=2D-Structure
https://pubchem.ncbi.nlm.nih.gov/compound/20823220#section=2D-Structure
https://pubchem.ncbi.nlm.nih.gov/compound/20823219#section=2D-Structure
https://pubchem.ncbi.nlm.nih.gov/compound/20823218#section=2D-Structure
https://pubchem.ncbi.nlm.nih.gov/compound/20823217#section=2D-Structure
https://pubchem.ncbi.nlm.nih.gov/compound/20823211#section=2D-Structure
https://pubchem.ncbi.nlm.nih.gov/compound/20823209#section=2D-Structure
https://pubchem.ncbi.nlm.nih.gov/compound/20823206#section=2D-Structure
https://pubchem.ncbi.nlm.nih.gov/compound/20823201#section=2D-Structure
https://pubchem.ncbi.nlm.nih.gov/compound/20823200#section=2D-Structure
https://pubchem.ncbi.nlm.nih.gov/compound/20823199#section=2D-Structure
https://pubchem.ncbi.nlm.nih.gov/compound/20823198#section=2D-Structure
https://pubchem.ncbi.nlm.nih.gov/compound/20823194#section=2D-Structure
https://pubchem.ncbi.nlm.nih.gov/compound/20823193#section=2D-Structure
https://pubchem.ncbi.nlm.nih.gov/compound/20823192#section=2D-Structure
https://pubchem.ncbi.nlm.nih.gov/compound/20823191#section=2D-Structure
https://pubchem.ncbi.nlm.nih.gov/compound/20823189#section=2D-Structure
https://pubchem.ncbi.nlm.nih.gov/compound/20823186#section=2D-Structure
https://pubchem.ncbi.nlm.nih.gov/compound/20823185#section=2D-Structure
https://pubchem.ncbi.nlm.nih.gov/compound/20823184#section=2D-Structure
https://pubchem.ncbi.nlm.nih.gov/compound/20753564#section=2D-Structure
https://pubchem.ncbi.nlm.nih.gov/compound/20753563#section=2D-Structure
https://pubchem.ncbi.nlm.nih.gov/compound/20752006#section=2D-Structure
https://pubchem.ncbi.nlm.nih.gov/compound/20746349#section=2D-Structure
https://pubchem.ncbi.nlm.nih.gov/compound/20727609#section=2D-Structure
https://pubchem.ncbi.nlm.nih.gov/compound/20727113#section=2D-Structure
https://pubchem.ncbi.nlm.nih.gov/compound/20720318#section=2D-Structure
https://pubchem.ncbi.nlm.nih.gov/compound/20719613#section=2D-Structure
https://pubchem.ncbi.nlm.nih.gov/compound/20693961#section=2D-Structure
https://pubchem.ncbi.nlm.nih.gov/compound/20691875#section=2D-Structure
https://pubchem.ncbi.nlm.nih.gov/compound/20691873#section=2D-Structure
https://pubchem.ncbi.nlm.nih.gov/compound/20691844#section=2D-Structure
https://pubchem.ncbi.nlm.nih.gov/compound/20689897#section=2D-Structure
https://pubchem.ncbi.nlm.nih.gov/compound/20685056#section=2D-Structure
https://pubchem.ncbi.nlm.nih.gov/compound/20682967#section=2D-Structure
https://pubchem.ncbi.nlm.nih.gov/compound/20682966#section=2D-Structure
https://pubchem.ncbi.nlm.nih.gov/compound/20682965#section=2D-Structure
https://pubchem.ncbi.nlm.nih.gov/compound/20682963#section=2D-Structure
https://pubchem.ncbi.nlm.nih.gov/compound/20682957#section=2D-Structure
https://pubchem.ncbi.nlm.nih.gov/compound/20682930#section=2D-Structure
https://pubchem.ncbi.nlm.nih.gov/compound/20682899#section=2D-Structure
https://pubchem.ncbi.nlm.nih.gov/compound/20681744#section=2D-Structure
https://pubchem.ncbi.nlm.nih.gov/compound/20679825#section=2D-Structure
https://pubchem.ncbi.nlm.nih.gov/compound/20679806#section=2D-Structure
https://pubchem.ncbi.nlm.nih.gov/compound/20679763#section=2D-Structure

https://pubchem.ncbi.nlm.nih.gov/compound/20677060#section=2D-Structure
https://pubchem.ncbi.nlm.nih.gov/compound/20663890#section=2D-Structure
https://pubchem.ncbi.nlm.nih.gov/compound/20648410#section=2D-Structure
https://pubchem.ncbi.nlm.nih.gov/compound/20645755#section=2D-Structure
https://pubchem.ncbi.nlm.nih.gov/compound/20642878#section=2D-Structure
https://pubchem.ncbi.nlm.nih.gov/compound/20642875#section=2D-Structure
https://pubchem.ncbi.nlm.nih.gov/compound/20626745#section=2D-Structure
https://pubchem.ncbi.nlm.nih.gov/compound/20621551#section=2D-Structure
https://pubchem.ncbi.nlm.nih.gov/compound/20611#section=2D-Structure
https://pubchem.ncbi.nlm.nih.gov/compound/20610565#section=2D-Structure
https://pubchem.ncbi.nlm.nih.gov/compound/20603194#section=2D-Structure
https://pubchem.ncbi.nlm.nih.gov/compound/20591633#section=2D-Structure
https://pubchem.ncbi.nlm.nih.gov/compound/20589057#section=2D-Structure
https://pubchem.ncbi.nlm.nih.gov/compound/20587534#section=2D-Structure
https://pubchem.ncbi.nlm.nih.gov/compound/20583839#section=2D-Structure
https://pubchem.ncbi.nlm.nih.gov/compound/20583393#section=2D-Structure
https://pubchem.ncbi.nlm.nih.gov/compound/20583392#section=2D-Structure
https://pubchem.ncbi.nlm.nih.gov/compound/20571354#section=2D-Structure
https://pubchem.ncbi.nlm.nih.gov/compound/20543915#section=2D-Structure
https://pubchem.ncbi.nlm.nih.gov/compound/20539842#section=2D-Structure
https://pubchem.ncbi.nlm.nih.gov/compound/20523672#section=2D-Structure
https://pubchem.ncbi.nlm.nih.gov/compound/20497010#section=2D-Structure
https://pubchem.ncbi.nlm.nih.gov/compound/20474664#section=2D-Structure
https://pubchem.ncbi.nlm.nih.gov/compound/20457873#section=2D-Structure
https://pubchem.ncbi.nlm.nih.gov/compound/20456904#section=2D-Structure
https://pubchem.ncbi.nlm.nih.gov/compound/20456899#section=2D-Structure
https://pubchem.ncbi.nlm.nih.gov/compound/20456898#section=2D-Structure
https://pubchem.ncbi.nlm.nih.gov/compound/20456896#section=2D-Structure
https://pubchem.ncbi.nlm.nih.gov/compound/20456893#section=2D-Structure
https://pubchem.ncbi.nlm.nih.gov/compound/20456892#section=2D-Structure
https://pubchem.ncbi.nlm.nih.gov/compound/20456889#section=2D-Structure
https://pubchem.ncbi.nlm.nih.gov/compound/20456644#section=2D-Structure
https://pubchem.ncbi.nlm.nih.gov/compound/20450517#section=2D-Structure
https://pubchem.ncbi.nlm.nih.gov/compound/20445402#section=2D-Structure
https://pubchem.ncbi.nlm.nih.gov/compound/20443619#section=2D-Structure
https://pubchem.ncbi.nlm.nih.gov/compound/20443613#section=2D-Structure
https://pubchem.ncbi.nlm.nih.gov/compound/20443612#section=2D-Structure
https://pubchem.ncbi.nlm.nih.gov/compound/20443609#section=2D-Structure
https://pubchem.ncbi.nlm.nih.gov/compound/20443606#section=2D-Structure
https://pubchem.ncbi.nlm.nih.gov/compound/20443605#section=2D-Structure
https://pubchem.ncbi.nlm.nih.gov/compound/20436215#section=2D-Structure
https://pubchem.ncbi.nlm.nih.gov/compound/20406060#section=2D-Structure
https://pubchem.ncbi.nlm.nih.gov/compound/20404712#section=2D-Structure
https://pubchem.ncbi.nlm.nih.gov/compound/20404650#section=2D-Structure
https://pubchem.ncbi.nlm.nih.gov/compound/20404649#section=2D-Structure
https://pubchem.ncbi.nlm.nih.gov/compound/20404642#section=2D-Structure
https://pubchem.ncbi.nlm.nih.gov/compound/20373272#section=2D-Structure
https://pubchem.ncbi.nlm.nih.gov/compound/20367851#section=2D-Structure
https://pubchem.ncbi.nlm.nih.gov/compound/20366984#section=2D-Structure
https://pubchem.ncbi.nlm.nih.gov/compound/20366983#section=2D-Structure
https://pubchem.ncbi.nlm.nih.gov/compound/20361400#section=2D-Structure
https://pubchem.ncbi.nlm.nih.gov/compound/20356436#section=2D-Structure
https://pubchem.ncbi.nlm.nih.gov/compound/20349758#section=2D-Structure
https://pubchem.ncbi.nlm.nih.gov/compound/20343424#section=2D-Structure
https://pubchem.ncbi.nlm.nih.gov/compound/20333888#section=2D-Structure

https://pubchem.ncbi.nlm.nih.gov/compound/20329092#section=2D-Structure
https://pubchem.ncbi.nlm.nih.gov/compound/20304473#section=2D-Structure
https://pubchem.ncbi.nlm.nih.gov/compound/20298877#section=2D-Structure
https://pubchem.ncbi.nlm.nih.gov/compound/20295278#section=2D-Structure
https://pubchem.ncbi.nlm.nih.gov/compound/20294079#section=2D-Structure
https://pubchem.ncbi.nlm.nih.gov/compound/20292031#section=2D-Structure
https://pubchem.ncbi.nlm.nih.gov/compound/20287807#section=2D-Structure
https://pubchem.ncbi.nlm.nih.gov/compound/20279711#section=2D-Structure
https://pubchem.ncbi.nlm.nih.gov/compound/20279702#section=2D-Structure
https://pubchem.ncbi.nlm.nih.gov/compound/20279696#section=2D-Structure
https://pubchem.ncbi.nlm.nih.gov/compound/20279694#section=2D-Structure
https://pubchem.ncbi.nlm.nih.gov/compound/20279692#section=2D-Structure
https://pubchem.ncbi.nlm.nih.gov/compound/20279690#section=2D-Structure
https://pubchem.ncbi.nlm.nih.gov/compound/20279689#section=2D-Structure
https://pubchem.ncbi.nlm.nih.gov/compound/20267699#section=2D-Structure
https://pubchem.ncbi.nlm.nih.gov/compound/20242871#section=2D-Structure
https://pubchem.ncbi.nlm.nih.gov/compound/20233395#section=2D-Structure
https://pubchem.ncbi.nlm.nih.gov/compound/20233391#section=2D-Structure
https://pubchem.ncbi.nlm.nih.gov/compound/20233387#section=2D-Structure
https://pubchem.ncbi.nlm.nih.gov/compound/20233386#section=2D-Structure
https://pubchem.ncbi.nlm.nih.gov/compound/20233383#section=2D-Structure
https://pubchem.ncbi.nlm.nih.gov/compound/20230986#section=2D-Structure
https://pubchem.ncbi.nlm.nih.gov/compound/20228743#section=2D-Structure
https://pubchem.ncbi.nlm.nih.gov/compound/20221379#section=2D-Structure
https://pubchem.ncbi.nlm.nih.gov/compound/20221375#section=2D-Structure
https://pubchem.ncbi.nlm.nih.gov/compound/20209336#section=2D-Structure
https://pubchem.ncbi.nlm.nih.gov/compound/20208213#section=2D-Structure
https://pubchem.ncbi.nlm.nih.gov/compound/20208212#section=2D-Structure
https://pubchem.ncbi.nlm.nih.gov/compound/20208197#section=2D-Structure
https://pubchem.ncbi.nlm.nih.gov/compound/20208192#section=2D-Structure
https://pubchem.ncbi.nlm.nih.gov/compound/20201695#section=2D-Structure
https://pubchem.ncbi.nlm.nih.gov/compound/20196336#section=2D-Structure
https://pubchem.ncbi.nlm.nih.gov/compound/20196326#section=2D-Structure
https://pubchem.ncbi.nlm.nih.gov/compound/201928#section=2D-Structure
https://pubchem.ncbi.nlm.nih.gov/compound/20188162#section=2D-Structure
https://pubchem.ncbi.nlm.nih.gov/compound/20161713#section=2D-Structure
https://pubchem.ncbi.nlm.nih.gov/compound/20159245#section=2D-Structure
https://pubchem.ncbi.nlm.nih.gov/compound/20156593#section=2D-Structure
https://pubchem.ncbi.nlm.nih.gov/compound/20154387#section=2D-Structure
https://pubchem.ncbi.nlm.nih.gov/compound/20148787#section=2D-Structure
https://pubchem.ncbi.nlm.nih.gov/compound/20088571#section=2D-Structure
https://pubchem.ncbi.nlm.nih.gov/compound/20081894#section=2D-Structure
https://pubchem.ncbi.nlm.nih.gov/compound/20080709#section=2D-Structure
https://pubchem.ncbi.nlm.nih.gov/compound/20079023#section=2D-Structure
https://pubchem.ncbi.nlm.nih.gov/compound/20067586#section=2D-Structure
https://pubchem.ncbi.nlm.nih.gov/compound/20067583#section=2D-Structure
https://pubchem.ncbi.nlm.nih.gov/compound/20067582#section=2D-Structure
https://pubchem.ncbi.nlm.nih.gov/compound/20058866#section=2D-Structure
https://pubchem.ncbi.nlm.nih.gov/compound/20056486#section=2D-Structure
https://pubchem.ncbi.nlm.nih.gov/compound/20055371#section=2D-Structure
https://pubchem.ncbi.nlm.nih.gov/compound/20034890#section=2D-Structure
https://pubchem.ncbi.nlm.nih.gov/compound/19985631#section=2D-Structure
https://pubchem.ncbi.nlm.nih.gov/compound/19975039#section=2D-Structure
https://pubchem.ncbi.nlm.nih.gov/compound/19971632#section=2D-Structure
https://pubchem.ncbi.nlm.nih.gov/compound/19971607#section=2D-Structure

https://pubchem.ncbi.nlm.nih.gov/compound/19964382#section=2D-Structure
https://pubchem.ncbi.nlm.nih.gov/compound/19962649#section=2D-Structure
https://pubchem.ncbi.nlm.nih.gov/compound/19962646#section=2D-Structure
https://pubchem.ncbi.nlm.nih.gov/compound/19960016#section=2D-Structure
https://pubchem.ncbi.nlm.nih.gov/compound/19938548#section=2D-Structure
https://pubchem.ncbi.nlm.nih.gov/compound/19937411#section=2D-Structure
https://pubchem.ncbi.nlm.nih.gov/compound/19934858#section=2D-Structure
https://pubchem.ncbi.nlm.nih.gov/compound/19909425#section=2D-Structure
https://pubchem.ncbi.nlm.nih.gov/compound/19901850#section=2D-Structure
https://pubchem.ncbi.nlm.nih.gov/compound/19901698#section=2D-Structure
https://pubchem.ncbi.nlm.nih.gov/compound/19890102#section=2D-Structure
https://pubchem.ncbi.nlm.nih.gov/compound/19890099#section=2D-Structure
https://pubchem.ncbi.nlm.nih.gov/compound/19875332#section=2D-Structure
https://pubchem.ncbi.nlm.nih.gov/compound/198698#section=2D-Structure
https://pubchem.ncbi.nlm.nih.gov/compound/198697#section=2D-Structure
https://pubchem.ncbi.nlm.nih.gov/compound/198696#section=2D-Structure
https://pubchem.ncbi.nlm.nih.gov/compound/19862903#section=2D-Structure
https://pubchem.ncbi.nlm.nih.gov/compound/19862899#section=2D-Structure
https://pubchem.ncbi.nlm.nih.gov/compound/19847275#section=2D-Structure
https://pubchem.ncbi.nlm.nih.gov/compound/19845433#section=2D-Structure
https://pubchem.ncbi.nlm.nih.gov/compound/19843926#section=2D-Structure
https://pubchem.ncbi.nlm.nih.gov/compound/19843922#section=2D-Structure
https://pubchem.ncbi.nlm.nih.gov/compound/19833359#section=2D-Structure
https://pubchem.ncbi.nlm.nih.gov/compound/19832846#section=2D-Structure
https://pubchem.ncbi.nlm.nih.gov/compound/19832826#section=2D-Structure
https://pubchem.ncbi.nlm.nih.gov/compound/19826049#section=2D-Structure
https://pubchem.ncbi.nlm.nih.gov/compound/19826048#section=2D-Structure
https://pubchem.ncbi.nlm.nih.gov/compound/19825645#section=2D-Structure
https://pubchem.ncbi.nlm.nih.gov/compound/19824920#section=2D-Structure
https://pubchem.ncbi.nlm.nih.gov/compound/19824918#section=2D-Structure
https://pubchem.ncbi.nlm.nih.gov/compound/19824115#section=2D-Structure
https://pubchem.ncbi.nlm.nih.gov/compound/19822428#section=2D-Structure
https://pubchem.ncbi.nlm.nih.gov/compound/19820756#section=2D-Structure
https://pubchem.ncbi.nlm.nih.gov/compound/19820414#section=2D-Structure
https://pubchem.ncbi.nlm.nih.gov/compound/19811430#section=2D-Structure
https://pubchem.ncbi.nlm.nih.gov/compound/19808110#section=2D-Structure
https://pubchem.ncbi.nlm.nih.gov/compound/19806880#section=2D-Structure
https://pubchem.ncbi.nlm.nih.gov/compound/19805354#section=2D-Structure
https://pubchem.ncbi.nlm.nih.gov/compound/19786014#section=2D-Structure
https://pubchem.ncbi.nlm.nih.gov/compound/19781529#section=2D-Structure
https://pubchem.ncbi.nlm.nih.gov/compound/19771244#section=2D-Structure
https://pubchem.ncbi.nlm.nih.gov/compound/19757586#section=2D-Structure
https://pubchem.ncbi.nlm.nih.gov/compound/19753181#section=2D-Structure
https://pubchem.ncbi.nlm.nih.gov/compound/19748570#section=2D-Structure
https://pubchem.ncbi.nlm.nih.gov/compound/19744958#section=2D-Structure
https://pubchem.ncbi.nlm.nih.gov/compound/19699635#section=2D-Structure
https://pubchem.ncbi.nlm.nih.gov/compound/19698700#section=2D-Structure
https://pubchem.ncbi.nlm.nih.gov/compound/19696287#section=2D-Structure
https://pubchem.ncbi.nlm.nih.gov/compound/19693564#section=2D-Structure
https://pubchem.ncbi.nlm.nih.gov/compound/19689749#section=2D-Structure
https://pubchem.ncbi.nlm.nih.gov/compound/19610814#section=2D-Structure
https://pubchem.ncbi.nlm.nih.gov/compound/19603199#section=2D-Structure
https://pubchem.ncbi.nlm.nih.gov/compound/19603197#section=2D-Structure
https://pubchem.ncbi.nlm.nih.gov/compound/19597021#section=2D-Structure
https://pubchem.ncbi.nlm.nih.gov/compound/193908#section=2D-Structure

https://pubchem.ncbi.nlm.nih.gov/compound/19382932#section=2D-Structure
https://pubchem.ncbi.nlm.nih.gov/compound/19356142#section=2D-Structure
https://pubchem.ncbi.nlm.nih.gov/compound/19353321#section=2D-Structure
https://pubchem.ncbi.nlm.nih.gov/compound/19353271#section=2D-Structure
https://pubchem.ncbi.nlm.nih.gov/compound/19350150#section=2D-Structure
https://pubchem.ncbi.nlm.nih.gov/compound/19168#section=2D-Structure
https://pubchem.ncbi.nlm.nih.gov/compound/19098104#section=2D-Structure
https://pubchem.ncbi.nlm.nih.gov/compound/19084393#section=2D-Structure
https://pubchem.ncbi.nlm.nih.gov/compound/19084134#section=2D-Structure
https://pubchem.ncbi.nlm.nih.gov/compound/19077474#section=2D-Structure
https://pubchem.ncbi.nlm.nih.gov/compound/19077104#section=2D-Structure
https://pubchem.ncbi.nlm.nih.gov/compound/19064490#section=2D-Structure
https://pubchem.ncbi.nlm.nih.gov/compound/19047267#section=2D-Structure
https://pubchem.ncbi.nlm.nih.gov/compound/19043316#section=2D-Structure
https://pubchem.ncbi.nlm.nih.gov/compound/19043279#section=2D-Structure
https://pubchem.ncbi.nlm.nih.gov/compound/190418#section=2D-Structure
https://pubchem.ncbi.nlm.nih.gov/compound/19041020#section=2D-Structure
https://pubchem.ncbi.nlm.nih.gov/compound/190389#section=2D-Structure
https://pubchem.ncbi.nlm.nih.gov/compound/19020842#section=2D-Structure
https://pubchem.ncbi.nlm.nih.gov/compound/19019310#section=2D-Structure
https://pubchem.ncbi.nlm.nih.gov/compound/190163#section=2D-Structure
https://pubchem.ncbi.nlm.nih.gov/compound/190127#section=2D-Structure
https://pubchem.ncbi.nlm.nih.gov/compound/19010385#section=2D-Structure
https://pubchem.ncbi.nlm.nih.gov/compound/19009344#section=2D-Structure
https://pubchem.ncbi.nlm.nih.gov/compound/19009330#section=2D-Structure
https://pubchem.ncbi.nlm.nih.gov/compound/19007191#section=2D-Structure
https://pubchem.ncbi.nlm.nih.gov/compound/189987#section=2D-Structure
https://pubchem.ncbi.nlm.nih.gov/compound/189972#section=2D-Structure
https://pubchem.ncbi.nlm.nih.gov/compound/18992840#section=2D-Structure
https://pubchem.ncbi.nlm.nih.gov/compound/18980841#section=2D-Structure
https://pubchem.ncbi.nlm.nih.gov/compound/18977684#section=2D-Structure
https://pubchem.ncbi.nlm.nih.gov/compound/18977681#section=2D-Structure
https://pubchem.ncbi.nlm.nih.gov/compound/189713#section=2D-Structure
https://pubchem.ncbi.nlm.nih.gov/compound/18964707#section=2D-Structure
https://pubchem.ncbi.nlm.nih.gov/compound/18961245#section=2D-Structure
https://pubchem.ncbi.nlm.nih.gov/compound/18942831#section=2D-Structure
https://pubchem.ncbi.nlm.nih.gov/compound/18926701#section=2D-Structure
https://pubchem.ncbi.nlm.nih.gov/compound/188944#section=2D-Structure
https://pubchem.ncbi.nlm.nih.gov/compound/188875#section=2D-Structure
https://pubchem.ncbi.nlm.nih.gov/compound/188874#section=2D-Structure
https://pubchem.ncbi.nlm.nih.gov/compound/188865#section=2D-Structure
https://pubchem.ncbi.nlm.nih.gov/compound/188809#section=2D-Structure
https://pubchem.ncbi.nlm.nih.gov/compound/188581#section=2D-Structure
https://pubchem.ncbi.nlm.nih.gov/compound/188520#section=2D-Structure
https://pubchem.ncbi.nlm.nih.gov/compound/188180#section=2D-Structure
https://pubchem.ncbi.nlm.nih.gov/compound/188022#section=2D-Structure
https://pubchem.ncbi.nlm.nih.gov/compound/18757607#section=2D-Structure
https://pubchem.ncbi.nlm.nih.gov/compound/18757603#section=2D-Structure
https://pubchem.ncbi.nlm.nih.gov/compound/18755049#section=2D-Structure
https://pubchem.ncbi.nlm.nih.gov/compound/18755047#section=2D-Structure
https://pubchem.ncbi.nlm.nih.gov/compound/18755046#section=2D-Structure
https://pubchem.ncbi.nlm.nih.gov/compound/187536#section=2D-Structure
https://pubchem.ncbi.nlm.nih.gov/compound/187535#section=2D-Structure
https://pubchem.ncbi.nlm.nih.gov/compound/187466#section=2D-Structure
https://pubchem.ncbi.nlm.nih.gov/compound/18740145#section=2D-Structure

https://pubchem.ncbi.nlm.nih.gov/compound/18740090#section=2D-Structure
https://pubchem.ncbi.nlm.nih.gov/compound/18725796#section=2D-Structure
https://pubchem.ncbi.nlm.nih.gov/compound/18724029#section=2D-Structure
https://pubchem.ncbi.nlm.nih.gov/compound/18724026#section=2D-Structure
https://pubchem.ncbi.nlm.nih.gov/compound/18724022#section=2D-Structure
https://pubchem.ncbi.nlm.nih.gov/compound/18724017#section=2D-Structure
https://pubchem.ncbi.nlm.nih.gov/compound/18724014#section=2D-Structure
https://pubchem.ncbi.nlm.nih.gov/compound/18724002#section=2D-Structure
https://pubchem.ncbi.nlm.nih.gov/compound/187211#section=2D-Structure
https://pubchem.ncbi.nlm.nih.gov/compound/187169#section=2D-Structure
https://pubchem.ncbi.nlm.nih.gov/compound/187167#section=2D-Structure
https://pubchem.ncbi.nlm.nih.gov/compound/187166#section=2D-Structure
https://pubchem.ncbi.nlm.nih.gov/compound/18704031#section=2D-Structure
https://pubchem.ncbi.nlm.nih.gov/compound/18704030#section=2D-Structure
https://pubchem.ncbi.nlm.nih.gov/compound/18702387#section=2D-Structure
https://pubchem.ncbi.nlm.nih.gov/compound/187005#section=2D-Structure
https://pubchem.ncbi.nlm.nih.gov/compound/186998#section=2D-Structure
https://pubchem.ncbi.nlm.nih.gov/compound/18699515#section=2D-Structure
https://pubchem.ncbi.nlm.nih.gov/compound/186918#section=2D-Structure
https://pubchem.ncbi.nlm.nih.gov/compound/18684130#section=2D-Structure
https://pubchem.ncbi.nlm.nih.gov/compound/18684129#section=2D-Structure
https://pubchem.ncbi.nlm.nih.gov/compound/18683217#section=2D-Structure
https://pubchem.ncbi.nlm.nih.gov/compound/18676801#section=2D-Structure
https://pubchem.ncbi.nlm.nih.gov/compound/186764#section=2D-Structure
https://pubchem.ncbi.nlm.nih.gov/compound/186571#section=2D-Structure
https://pubchem.ncbi.nlm.nih.gov/compound/186555#section=2D-Structure
https://pubchem.ncbi.nlm.nih.gov/compound/186535#section=2D-Structure
https://pubchem.ncbi.nlm.nih.gov/compound/186534#section=2D-Structure
https://pubchem.ncbi.nlm.nih.gov/compound/186532#section=2D-Structure
https://pubchem.ncbi.nlm.nih.gov/compound/186437#section=2D-Structure
https://pubchem.ncbi.nlm.nih.gov/compound/186435#section=2D-Structure
https://pubchem.ncbi.nlm.nih.gov/compound/186434#section=2D-Structure
https://pubchem.ncbi.nlm.nih.gov/compound/186361#section=2D-Structure
https://pubchem.ncbi.nlm.nih.gov/compound/186360#section=2D-Structure
https://pubchem.ncbi.nlm.nih.gov/compound/186290#section=2D-Structure
https://pubchem.ncbi.nlm.nih.gov/compound/186289#section=2D-Structure
https://pubchem.ncbi.nlm.nih.gov/compound/186288#section=2D-Structure
https://pubchem.ncbi.nlm.nih.gov/compound/186287#section=2D-Structure
https://pubchem.ncbi.nlm.nih.gov/compound/186286#section=2D-Structure
https://pubchem.ncbi.nlm.nih.gov/compound/186285#section=2D-Structure
https://pubchem.ncbi.nlm.nih.gov/compound/186283#section=2D-Structure
https://pubchem.ncbi.nlm.nih.gov/compound/186251#section=2D-Structure
https://pubchem.ncbi.nlm.nih.gov/compound/186217#section=2D-Structure
https://pubchem.ncbi.nlm.nih.gov/compound/18621046#section=2D-Structure
https://pubchem.ncbi.nlm.nih.gov/compound/18621045#section=2D-Structure
https://pubchem.ncbi.nlm.nih.gov/compound/186057#section=2D-Structure
https://pubchem.ncbi.nlm.nih.gov/compound/185879#section=2D-Structure
https://pubchem.ncbi.nlm.nih.gov/compound/18548300#section=2D-Structure
https://pubchem.ncbi.nlm.nih.gov/compound/18539605#section=2D-Structure
https://pubchem.ncbi.nlm.nih.gov/compound/18475262#section=2D-Structure
https://pubchem.ncbi.nlm.nih.gov/compound/184752#section=2D-Structure
https://pubchem.ncbi.nlm.nih.gov/compound/18445982#section=2D-Structure
https://pubchem.ncbi.nlm.nih.gov/compound/184445#section=2D-Structure
https://pubchem.ncbi.nlm.nih.gov/compound/18443862#section=2D-Structure
https://pubchem.ncbi.nlm.nih.gov/compound/18429010#section=2D-Structure

https://pubchem.ncbi.nlm.nih.gov/compound/18428899#section=2D-Structure
https://pubchem.ncbi.nlm.nih.gov/compound/18424997#section=2D-Structure
https://pubchem.ncbi.nlm.nih.gov/compound/184069#section=2D-Structure
https://pubchem.ncbi.nlm.nih.gov/compound/184047#section=2D-Structure
https://pubchem.ncbi.nlm.nih.gov/compound/184046#section=2D-Structure
https://pubchem.ncbi.nlm.nih.gov/compound/18369670#section=2D-Structure
https://pubchem.ncbi.nlm.nih.gov/compound/18362475#section=2D-Structure
https://pubchem.ncbi.nlm.nih.gov/compound/18354100#section=2D-Structure
https://pubchem.ncbi.nlm.nih.gov/compound/18347756#section=2D-Structure
https://pubchem.ncbi.nlm.nih.gov/compound/183235#section=2D-Structure
https://pubchem.ncbi.nlm.nih.gov/compound/183231#section=2D-Structure
https://pubchem.ncbi.nlm.nih.gov/compound/183179#section=2D-Structure
https://pubchem.ncbi.nlm.nih.gov/compound/183178#section=2D-Structure
https://pubchem.ncbi.nlm.nih.gov/compound/18301#section=2D-Structure
https://pubchem.ncbi.nlm.nih.gov/compound/18300#section=2D-Structure
https://pubchem.ncbi.nlm.nih.gov/compound/18299#section=2D-Structure
https://pubchem.ncbi.nlm.nih.gov/compound/182955#section=2D-Structure
https://pubchem.ncbi.nlm.nih.gov/compound/182400#section=2D-Structure
https://pubchem.ncbi.nlm.nih.gov/compound/181017#section=2D-Structure
https://pubchem.ncbi.nlm.nih.gov/compound/180700#section=2D-Structure
https://pubchem.ncbi.nlm.nih.gov/compound/180512#section=2D-Structure
https://pubchem.ncbi.nlm.nih.gov/compound/17983220#section=2D-Structure
https://pubchem.ncbi.nlm.nih.gov/compound/17969627#section=2D-Structure
https://pubchem.ncbi.nlm.nih.gov/compound/17964506#section=2D-Structure
https://pubchem.ncbi.nlm.nih.gov/compound/17959018#section=2D-Structure
https://pubchem.ncbi.nlm.nih.gov/compound/17921892#section=2D-Structure
https://pubchem.ncbi.nlm.nih.gov/compound/17900781#section=2D-Structure
https://pubchem.ncbi.nlm.nih.gov/compound/17900775#section=2D-Structure
https://pubchem.ncbi.nlm.nih.gov/compound/17874#section=2D-Structure
https://pubchem.ncbi.nlm.nih.gov/compound/17873#section=2D-Structure
https://pubchem.ncbi.nlm.nih.gov/compound/178652#section=2D-Structure
https://pubchem.ncbi.nlm.nih.gov/compound/178229#section=2D-Structure
https://pubchem.ncbi.nlm.nih.gov/compound/178228#section=2D-Structure
https://pubchem.ncbi.nlm.nih.gov/compound/178227#section=2D-Structure
https://pubchem.ncbi.nlm.nih.gov/compound/17819864#section=2D-Structure
https://pubchem.ncbi.nlm.nih.gov/compound/178018#section=2D-Structure
https://pubchem.ncbi.nlm.nih.gov/compound/17799624#section=2D-Structure
https://pubchem.ncbi.nlm.nih.gov/compound/177758#section=2D-Structure
https://pubchem.ncbi.nlm.nih.gov/compound/17653203#section=2D-Structure
https://pubchem.ncbi.nlm.nih.gov/compound/175982#section=2D-Structure
https://pubchem.ncbi.nlm.nih.gov/compound/175877#section=2D-Structure
https://pubchem.ncbi.nlm.nih.gov/compound/174523#section=2D-Structure
https://pubchem.ncbi.nlm.nih.gov/compound/174492#section=2D-Structure
https://pubchem.ncbi.nlm.nih.gov/compound/169380#section=2D-Structure
https://pubchem.ncbi.nlm.nih.gov/compound/16935#section=2D-Structure
https://pubchem.ncbi.nlm.nih.gov/compound/168922#section=2D-Structure
https://pubchem.ncbi.nlm.nih.gov/compound/16820#section=2D-Structure
https://pubchem.ncbi.nlm.nih.gov/compound/168173#section=2D-Structure
https://pubchem.ncbi.nlm.nih.gov/compound/168159#section=2D-Structure
https://pubchem.ncbi.nlm.nih.gov/compound/16740178#section=2D-Structure
https://pubchem.ncbi.nlm.nih.gov/compound/167034#section=2D-Structure
https://pubchem.ncbi.nlm.nih.gov/compound/16680860#section=2D-Structure
https://pubchem.ncbi.nlm.nih.gov/compound/16664789#section=2D-Structure
https://pubchem.ncbi.nlm.nih.gov/compound/16664788#section=2D-Structure
https://pubchem.ncbi.nlm.nih.gov/compound/16658561#section=2D-Structure

https://pubchem.ncbi.nlm.nih.gov/compound/16658389#section=2D-Structure
https://pubchem.ncbi.nlm.nih.gov/compound/164568#section=2D-Structure
https://pubchem.ncbi.nlm.nih.gov/compound/164559#section=2D-Structure
https://pubchem.ncbi.nlm.nih.gov/compound/164555#section=2D-Structure
https://pubchem.ncbi.nlm.nih.gov/compound/164554#section=2D-Structure
https://pubchem.ncbi.nlm.nih.gov/compound/164492#section=2D-Structure
https://pubchem.ncbi.nlm.nih.gov/compound/164491#section=2D-Structure
https://pubchem.ncbi.nlm.nih.gov/compound/163488#section=2D-Structure
https://pubchem.ncbi.nlm.nih.gov/compound/161923#section=2D-Structure
https://pubchem.ncbi.nlm.nih.gov/compound/16080842#section=2D-Structure
https://pubchem.ncbi.nlm.nih.gov/compound/16066791#section=2D-Structure
https://pubchem.ncbi.nlm.nih.gov/compound/16066790#section=2D-Structure
https://pubchem.ncbi.nlm.nih.gov/compound/160658#section=2D-Structure
https://pubchem.ncbi.nlm.nih.gov/compound/160566#section=2D-Structure
https://pubchem.ncbi.nlm.nih.gov/compound/160350#section=2D-Structure
https://pubchem.ncbi.nlm.nih.gov/compound/160349#section=2D-Structure
https://pubchem.ncbi.nlm.nih.gov/compound/160334#section=2D-Structure
https://pubchem.ncbi.nlm.nih.gov/compound/160249#section=2D-Structure
https://pubchem.ncbi.nlm.nih.gov/compound/15963795#section=2D-Structure
https://pubchem.ncbi.nlm.nih.gov/compound/159620#section=2D-Structure
https://pubchem.ncbi.nlm.nih.gov/compound/159004#section=2D-Structure
https://pubchem.ncbi.nlm.nih.gov/compound/158989#section=2D-Structure
https://pubchem.ncbi.nlm.nih.gov/compound/15893273#section=2D-Structure
https://pubchem.ncbi.nlm.nih.gov/compound/15883471#section=2D-Structure
https://pubchem.ncbi.nlm.nih.gov/compound/15872732#section=2D-Structure
https://pubchem.ncbi.nlm.nih.gov/compound/158647#section=2D-Structure
https://pubchem.ncbi.nlm.nih.gov/compound/15862336#section=2D-Structure
https://pubchem.ncbi.nlm.nih.gov/compound/15859760#section=2D-Structure
https://pubchem.ncbi.nlm.nih.gov/compound/15839028#section=2D-Structure
https://pubchem.ncbi.nlm.nih.gov/compound/15836872#section=2D-Structure
https://pubchem.ncbi.nlm.nih.gov/compound/15828734#section=2D-Structure
https://pubchem.ncbi.nlm.nih.gov/compound/15820912#section=2D-Structure
https://pubchem.ncbi.nlm.nih.gov/compound/15820911#section=2D-Structure
https://pubchem.ncbi.nlm.nih.gov/compound/158199#section=2D-Structure
https://pubchem.ncbi.nlm.nih.gov/compound/15767933#section=2D-Structure
https://pubchem.ncbi.nlm.nih.gov/compound/157306#section=2D-Structure
https://pubchem.ncbi.nlm.nih.gov/compound/15727012#section=2D-Structure
https://pubchem.ncbi.nlm.nih.gov/compound/15727010#section=2D-Structure
https://pubchem.ncbi.nlm.nih.gov/compound/15726994#section=2D-Structure
https://pubchem.ncbi.nlm.nih.gov/compound/15717568#section=2D-Structure
https://pubchem.ncbi.nlm.nih.gov/compound/15717000#section=2D-Structure
https://pubchem.ncbi.nlm.nih.gov/compound/15716473#section=2D-Structure
https://pubchem.ncbi.nlm.nih.gov/compound/15716470#section=2D-Structure
https://pubchem.ncbi.nlm.nih.gov/compound/15711092#section=2D-Structure
https://pubchem.ncbi.nlm.nih.gov/compound/15699#section=2D-Structure
https://pubchem.ncbi.nlm.nih.gov/compound/15660753#section=2D-Structure
https://pubchem.ncbi.nlm.nih.gov/compound/15660752#section=2D-Structure
https://pubchem.ncbi.nlm.nih.gov/compound/15645608#section=2D-Structure
https://pubchem.ncbi.nlm.nih.gov/compound/15620659#section=2D-Structure
https://pubchem.ncbi.nlm.nih.gov/compound/15590256#section=2D-Structure
https://pubchem.ncbi.nlm.nih.gov/compound/15588300#section=2D-Structure
https://pubchem.ncbi.nlm.nih.gov/compound/15579019#section=2D-Structure
https://pubchem.ncbi.nlm.nih.gov/compound/15579018#section=2D-Structure
https://pubchem.ncbi.nlm.nih.gov/compound/15567828#section=2D-Structure
https://pubchem.ncbi.nlm.nih.gov/compound/15563496#section=2D-Structure

https://pubchem.ncbi.nlm.nih.gov/compound/155608#section=2D-Structure
https://pubchem.ncbi.nlm.nih.gov/compound/15559138#section=2D-Structure
https://pubchem.ncbi.nlm.nih.gov/compound/155464#section=2D-Structure
https://pubchem.ncbi.nlm.nih.gov/compound/15535087#section=2D-Structure
https://pubchem.ncbi.nlm.nih.gov/compound/15534533#section=2D-Structure
https://pubchem.ncbi.nlm.nih.gov/compound/15532373#section=2D-Structure
https://pubchem.ncbi.nlm.nih.gov/compound/15531421#section=2D-Structure
https://pubchem.ncbi.nlm.nih.gov/compound/15520000#section=2D-Structure
https://pubchem.ncbi.nlm.nih.gov/compound/15507130#section=2D-Structure
https://pubchem.ncbi.nlm.nih.gov/compound/15507129#section=2D-Structure
https://pubchem.ncbi.nlm.nih.gov/compound/15499404#section=2D-Structure
https://pubchem.ncbi.nlm.nih.gov/compound/154901#section=2D-Structure
https://pubchem.ncbi.nlm.nih.gov/compound/15488322#section=2D-Structure
https://pubchem.ncbi.nlm.nih.gov/compound/154881#section=2D-Structure
https://pubchem.ncbi.nlm.nih.gov/compound/154829#section=2D-Structure
https://pubchem.ncbi.nlm.nih.gov/compound/154750#section=2D-Structure
https://pubchem.ncbi.nlm.nih.gov/compound/154740#section=2D-Structure
https://pubchem.ncbi.nlm.nih.gov/compound/154696#section=2D-Structure
https://pubchem.ncbi.nlm.nih.gov/compound/154601#section=2D-Structure
https://pubchem.ncbi.nlm.nih.gov/compound/154521#section=2D-Structure
https://pubchem.ncbi.nlm.nih.gov/compound/154510#section=2D-Structure
https://pubchem.ncbi.nlm.nih.gov/compound/15447367#section=2D-Structure
https://pubchem.ncbi.nlm.nih.gov/compound/154431#section=2D-Structure
https://pubchem.ncbi.nlm.nih.gov/compound/154427#section=2D-Structure
https://pubchem.ncbi.nlm.nih.gov/compound/154426#section=2D-Structure
https://pubchem.ncbi.nlm.nih.gov/compound/154425#section=2D-Structure
https://pubchem.ncbi.nlm.nih.gov/compound/154423#section=2D-Structure
https://pubchem.ncbi.nlm.nih.gov/compound/154419#section=2D-Structure
https://pubchem.ncbi.nlm.nih.gov/compound/154330#section=2D-Structure
https://pubchem.ncbi.nlm.nih.gov/compound/15428435#section=2D-Structure
https://pubchem.ncbi.nlm.nih.gov/compound/15423252#section=2D-Structure
https://pubchem.ncbi.nlm.nih.gov/compound/15423251#section=2D-Structure
https://pubchem.ncbi.nlm.nih.gov/compound/15422109#section=2D-Structure
https://pubchem.ncbi.nlm.nih.gov/compound/15418687#section=2D-Structure
https://pubchem.ncbi.nlm.nih.gov/compound/154007#section=2D-Structure
https://pubchem.ncbi.nlm.nih.gov/compound/15399287#section=2D-Structure
https://pubchem.ncbi.nlm.nih.gov/compound/15388787#section=2D-Structure
https://pubchem.ncbi.nlm.nih.gov/compound/153841#section=2D-Structure
https://pubchem.ncbi.nlm.nih.gov/compound/15381525#section=2D-Structure
https://pubchem.ncbi.nlm.nih.gov/compound/153774#section=2D-Structure
https://pubchem.ncbi.nlm.nih.gov/compound/15372568#section=2D-Structure
https://pubchem.ncbi.nlm.nih.gov/compound/153696#section=2D-Structure
https://pubchem.ncbi.nlm.nih.gov/compound/15365395#section=2D-Structure
https://pubchem.ncbi.nlm.nih.gov/compound/15360106#section=2D-Structure
https://pubchem.ncbi.nlm.nih.gov/compound/15353220#section=2D-Structure
https://pubchem.ncbi.nlm.nih.gov/compound/15353219#section=2D-Structure
https://pubchem.ncbi.nlm.nih.gov/compound/15353218#section=2D-Structure
https://pubchem.ncbi.nlm.nih.gov/compound/153520#section=2D-Structure
https://pubchem.ncbi.nlm.nih.gov/compound/153396#section=2D-Structure
https://pubchem.ncbi.nlm.nih.gov/compound/153355#section=2D-Structure
https://pubchem.ncbi.nlm.nih.gov/compound/15330291#section=2D-Structure
https://pubchem.ncbi.nlm.nih.gov/compound/15322961#section=2D-Structure
https://pubchem.ncbi.nlm.nih.gov/compound/15322959#section=2D-Structure
https://pubchem.ncbi.nlm.nih.gov/compound/153037#section=2D-Structure
https://pubchem.ncbi.nlm.nih.gov/compound/153031#section=2D-Structure

https://pubchem.ncbi.nlm.nih.gov/compound/15287164#section=2D-Structure
https://pubchem.ncbi.nlm.nih.gov/compound/15277896#section=2D-Structure
https://pubchem.ncbi.nlm.nih.gov/compound/15267653#section=2D-Structure
https://pubchem.ncbi.nlm.nih.gov/compound/15267652#section=2D-Structure
https://pubchem.ncbi.nlm.nih.gov/compound/15267425#section=2D-Structure
https://pubchem.ncbi.nlm.nih.gov/compound/15266425#section=2D-Structure
https://pubchem.ncbi.nlm.nih.gov/compound/15257537#section=2D-Structure
https://pubchem.ncbi.nlm.nih.gov/compound/152522#section=2D-Structure
https://pubchem.ncbi.nlm.nih.gov/compound/15249681#section=2D-Structure
https://pubchem.ncbi.nlm.nih.gov/compound/15249677#section=2D-Structure
https://pubchem.ncbi.nlm.nih.gov/compound/15248279#section=2D-Structure
https://pubchem.ncbi.nlm.nih.gov/compound/15247773#section=2D-Structure
https://pubchem.ncbi.nlm.nih.gov/compound/15225518#section=2D-Structure
https://pubchem.ncbi.nlm.nih.gov/compound/15214246#section=2D-Structure
https://pubchem.ncbi.nlm.nih.gov/compound/15211623#section=2D-Structure
https://pubchem.ncbi.nlm.nih.gov/compound/152023#section=2D-Structure
https://pubchem.ncbi.nlm.nih.gov/compound/152019#section=2D-Structure
https://pubchem.ncbi.nlm.nih.gov/compound/151999#section=2D-Structure
https://pubchem.ncbi.nlm.nih.gov/compound/15183527#section=2D-Structure
https://pubchem.ncbi.nlm.nih.gov/compound/15178861#section=2D-Structure
https://pubchem.ncbi.nlm.nih.gov/compound/15156402#section=2D-Structure
https://pubchem.ncbi.nlm.nih.gov/compound/151557#section=2D-Structure
https://pubchem.ncbi.nlm.nih.gov/compound/15147298#section=2D-Structure
https://pubchem.ncbi.nlm.nih.gov/compound/15135286#section=2D-Structure
https://pubchem.ncbi.nlm.nih.gov/compound/15135285#section=2D-Structure
https://pubchem.ncbi.nlm.nih.gov/compound/15115274#section=2D-Structure
https://pubchem.ncbi.nlm.nih.gov/compound/15109438#section=2D-Structure
https://pubchem.ncbi.nlm.nih.gov/compound/150767#section=2D-Structure
https://pubchem.ncbi.nlm.nih.gov/compound/15075416#section=2D-Structure
https://pubchem.ncbi.nlm.nih.gov/compound/150742#section=2D-Structure
https://pubchem.ncbi.nlm.nih.gov/compound/150674#section=2D-Structure
https://pubchem.ncbi.nlm.nih.gov/compound/150672#section=2D-Structure
https://pubchem.ncbi.nlm.nih.gov/compound/150608#section=2D-Structure
https://pubchem.ncbi.nlm.nih.gov/compound/150521#section=2D-Structure
https://pubchem.ncbi.nlm.nih.gov/compound/150520#section=2D-Structure
https://pubchem.ncbi.nlm.nih.gov/compound/15048486#section=2D-Structure
https://pubchem.ncbi.nlm.nih.gov/compound/15048478#section=2D-Structure
https://pubchem.ncbi.nlm.nih.gov/compound/15046007#section=2D-Structure
https://pubchem.ncbi.nlm.nih.gov/compound/150380#section=2D-Structure
https://pubchem.ncbi.nlm.nih.gov/compound/150370#section=2D-Structure
https://pubchem.ncbi.nlm.nih.gov/compound/150358#section=2D-Structure
https://pubchem.ncbi.nlm.nih.gov/compound/150354#section=2D-Structure
https://pubchem.ncbi.nlm.nih.gov/compound/150353#section=2D-Structure
https://pubchem.ncbi.nlm.nih.gov/compound/150330#section=2D-Structure
https://pubchem.ncbi.nlm.nih.gov/compound/15025487#section=2D-Structure
https://pubchem.ncbi.nlm.nih.gov/compound/15025485#section=2D-Structure
https://pubchem.ncbi.nlm.nih.gov/compound/150155#section=2D-Structure
https://pubchem.ncbi.nlm.nih.gov/compound/150154#section=2D-Structure
https://pubchem.ncbi.nlm.nih.gov/compound/150126#section=2D-Structure
https://pubchem.ncbi.nlm.nih.gov/compound/150019#section=2D-Structure
https://pubchem.ncbi.nlm.nih.gov/compound/15001195#section=2D-Structure
https://pubchem.ncbi.nlm.nih.gov/compound/150008#section=2D-Structure
https://pubchem.ncbi.nlm.nih.gov/compound/149916#section=2D-Structure
https://pubchem.ncbi.nlm.nih.gov/compound/149858#section=2D-Structure
https://pubchem.ncbi.nlm.nih.gov/compound/149857#section=2D-Structure

https://pubchem.ncbi.nlm.nih.gov/compound/149830#section=2D-Structure
https://pubchem.ncbi.nlm.nih.gov/compound/149805#section=2D-Structure
https://pubchem.ncbi.nlm.nih.gov/compound/14980284#section=2D-Structure
https://pubchem.ncbi.nlm.nih.gov/compound/14980239#section=2D-Structure
https://pubchem.ncbi.nlm.nih.gov/compound/14980238#section=2D-Structure
https://pubchem.ncbi.nlm.nih.gov/compound/14980237#section=2D-Structure
https://pubchem.ncbi.nlm.nih.gov/compound/149709#section=2D-Structure
https://pubchem.ncbi.nlm.nih.gov/compound/149708#section=2D-Structure
https://pubchem.ncbi.nlm.nih.gov/compound/14967251#section=2D-Structure
https://pubchem.ncbi.nlm.nih.gov/compound/14965403#section=2D-Structure
https://pubchem.ncbi.nlm.nih.gov/compound/14957844#section=2D-Structure
https://pubchem.ncbi.nlm.nih.gov/compound/14954925#section=2D-Structure
https://pubchem.ncbi.nlm.nih.gov/compound/14950622#section=2D-Structure
https://pubchem.ncbi.nlm.nih.gov/compound/149474#section=2D-Structure
https://pubchem.ncbi.nlm.nih.gov/compound/149451#section=2D-Structure
https://pubchem.ncbi.nlm.nih.gov/compound/14944079#section=2D-Structure
https://pubchem.ncbi.nlm.nih.gov/compound/14942854#section=2D-Structure
https://pubchem.ncbi.nlm.nih.gov/compound/149421#section=2D-Structure
https://pubchem.ncbi.nlm.nih.gov/compound/149345#section=2D-Structure
https://pubchem.ncbi.nlm.nih.gov/compound/149344#section=2D-Structure
https://pubchem.ncbi.nlm.nih.gov/compound/14924160#section=2D-Structure
https://pubchem.ncbi.nlm.nih.gov/compound/14923222#section=2D-Structure
https://pubchem.ncbi.nlm.nih.gov/compound/149212#section=2D-Structure
https://pubchem.ncbi.nlm.nih.gov/compound/14920019#section=2D-Structure
https://pubchem.ncbi.nlm.nih.gov/compound/149184#section=2D-Structure
https://pubchem.ncbi.nlm.nih.gov/compound/149183#section=2D-Structure
https://pubchem.ncbi.nlm.nih.gov/compound/149182#section=2D-Structure
https://pubchem.ncbi.nlm.nih.gov/compound/14905943#section=2D-Structure
https://pubchem.ncbi.nlm.nih.gov/compound/14905895#section=2D-Structure
https://pubchem.ncbi.nlm.nih.gov/compound/14905193#section=2D-Structure
https://pubchem.ncbi.nlm.nih.gov/compound/14903527#section=2D-Structure
https://pubchem.ncbi.nlm.nih.gov/compound/14903510#section=2D-Structure
https://pubchem.ncbi.nlm.nih.gov/compound/148990#section=2D-Structure
https://pubchem.ncbi.nlm.nih.gov/compound/14897005#section=2D-Structure
https://pubchem.ncbi.nlm.nih.gov/compound/14897000#section=2D-Structure
https://pubchem.ncbi.nlm.nih.gov/compound/14896997#section=2D-Structure
https://pubchem.ncbi.nlm.nih.gov/compound/14896996#section=2D-Structure
https://pubchem.ncbi.nlm.nih.gov/compound/1488653#section=2D-Structure
https://pubchem.ncbi.nlm.nih.gov/compound/148658#section=2D-Structure
https://pubchem.ncbi.nlm.nih.gov/compound/148413#section=2D-Structure
https://pubchem.ncbi.nlm.nih.gov/compound/148349#section=2D-Structure
https://pubchem.ncbi.nlm.nih.gov/compound/14831899#section=2D-Structure
https://pubchem.ncbi.nlm.nih.gov/compound/148186#section=2D-Structure
https://pubchem.ncbi.nlm.nih.gov/compound/14811980#section=2D-Structure
https://pubchem.ncbi.nlm.nih.gov/compound/14811979#section=2D-Structure
https://pubchem.ncbi.nlm.nih.gov/compound/14811458#section=2D-Structure
https://pubchem.ncbi.nlm.nih.gov/compound/14811456#section=2D-Structure
https://pubchem.ncbi.nlm.nih.gov/compound/14786992#section=2D-Structure
https://pubchem.ncbi.nlm.nih.gov/compound/147857#section=2D-Structure
https://pubchem.ncbi.nlm.nih.gov/compound/14767717#section=2D-Structure
https://pubchem.ncbi.nlm.nih.gov/compound/14765876#section=2D-Structure
https://pubchem.ncbi.nlm.nih.gov/compound/1475114#section=2D-Structure
https://pubchem.ncbi.nlm.nih.gov/compound/147501#section=2D-Structure
https://pubchem.ncbi.nlm.nih.gov/compound/147483#section=2D-Structure
https://pubchem.ncbi.nlm.nih.gov/compound/147455#section=2D-Structure

https://pubchem.ncbi.nlm.nih.gov/compound/147450#section=2D-Structure
https://pubchem.ncbi.nlm.nih.gov/compound/147335#section=2D-Structure
https://pubchem.ncbi.nlm.nih.gov/compound/147333#section=2D-Structure
https://pubchem.ncbi.nlm.nih.gov/compound/147195#section=2D-Structure
https://pubchem.ncbi.nlm.nih.gov/compound/14716635#section=2D-Structure
https://pubchem.ncbi.nlm.nih.gov/compound/14690954#section=2D-Structure
https://pubchem.ncbi.nlm.nih.gov/compound/146732#section=2D-Structure
https://pubchem.ncbi.nlm.nih.gov/compound/146720#section=2D-Structure
https://pubchem.ncbi.nlm.nih.gov/compound/14671244#section=2D-Structure
https://pubchem.ncbi.nlm.nih.gov/compound/14671124#section=2D-Structure
https://pubchem.ncbi.nlm.nih.gov/compound/14668820#section=2D-Structure
https://pubchem.ncbi.nlm.nih.gov/compound/146677729#section=2D-Structure
https://pubchem.ncbi.nlm.nih.gov/compound/146657536#section=2D-Structure
https://pubchem.ncbi.nlm.nih.gov/compound/146597506#section=2D-Structure
https://pubchem.ncbi.nlm.nih.gov/compound/146589965#section=2D-Structure
https://pubchem.ncbi.nlm.nih.gov/compound/146588683#section=2D-Structure
https://pubchem.ncbi.nlm.nih.gov/compound/146579755#section=2D-Structure
https://pubchem.ncbi.nlm.nih.gov/compound/146562485#section=2D-Structure
https://pubchem.ncbi.nlm.nih.gov/compound/146562483#section=2D-Structure
https://pubchem.ncbi.nlm.nih.gov/compound/146539367#section=2D-Structure
https://pubchem.ncbi.nlm.nih.gov/compound/146536785#section=2D-Structure
https://pubchem.ncbi.nlm.nih.gov/compound/146536782#section=2D-Structure
https://pubchem.ncbi.nlm.nih.gov/compound/146531670#section=2D-Structure
https://pubchem.ncbi.nlm.nih.gov/compound/146492401#section=2D-Structure
https://pubchem.ncbi.nlm.nih.gov/compound/146445402#section=2D-Structure
https://pubchem.ncbi.nlm.nih.gov/compound/146444995#section=2D-Structure
https://pubchem.ncbi.nlm.nih.gov/compound/146444992#section=2D-Structure
https://pubchem.ncbi.nlm.nih.gov/compound/146444990#section=2D-Structure
https://pubchem.ncbi.nlm.nih.gov/compound/146443905#section=2D-Structure
https://pubchem.ncbi.nlm.nih.gov/compound/146443903#section=2D-Structure
https://pubchem.ncbi.nlm.nih.gov/compound/146443888#section=2D-Structure
https://pubchem.ncbi.nlm.nih.gov/compound/146443852#section=2D-Structure
https://pubchem.ncbi.nlm.nih.gov/compound/146437961#section=2D-Structure
https://pubchem.ncbi.nlm.nih.gov/compound/146421189#section=2D-Structure
https://pubchem.ncbi.nlm.nih.gov/compound/146415818#section=2D-Structure
https://pubchem.ncbi.nlm.nih.gov/compound/146414513#section=2D-Structure
https://pubchem.ncbi.nlm.nih.gov/compound/146343446#section=2D-Structure
https://pubchem.ncbi.nlm.nih.gov/compound/146343422#section=2D-Structure
https://pubchem.ncbi.nlm.nih.gov/compound/146343409#section=2D-Structure
https://pubchem.ncbi.nlm.nih.gov/compound/146337710#section=2D-Structure
https://pubchem.ncbi.nlm.nih.gov/compound/146336923#section=2D-Structure
https://pubchem.ncbi.nlm.nih.gov/compound/146329777#section=2D-Structure
https://pubchem.ncbi.nlm.nih.gov/compound/146327141#section=2D-Structure
https://pubchem.ncbi.nlm.nih.gov/compound/146319207#section=2D-Structure
https://pubchem.ncbi.nlm.nih.gov/compound/146319206#section=2D-Structure
https://pubchem.ncbi.nlm.nih.gov/compound/146317519#section=2D-Structure
https://pubchem.ncbi.nlm.nih.gov/compound/146307#section=2D-Structure
https://pubchem.ncbi.nlm.nih.gov/compound/146301#section=2D-Structure
https://pubchem.ncbi.nlm.nih.gov/compound/146295519#section=2D-Structure
https://pubchem.ncbi.nlm.nih.gov/compound/146268534#section=2D-Structure
https://pubchem.ncbi.nlm.nih.gov/compound/146268517#section=2D-Structure
https://pubchem.ncbi.nlm.nih.gov/compound/146262526#section=2D-Structure
https://pubchem.ncbi.nlm.nih.gov/compound/146260506#section=2D-Structure
https://pubchem.ncbi.nlm.nih.gov/compound/146243950#section=2D-Structure
https://pubchem.ncbi.nlm.nih.gov/compound/146199289#section=2D-Structure

https://pubchem.ncbi.nlm.nih.gov/compound/146189121#section=2D-Structure
https://pubchem.ncbi.nlm.nih.gov/compound/146186928#section=2D-Structure
https://pubchem.ncbi.nlm.nih.gov/compound/146186927#section=2D-Structure
https://pubchem.ncbi.nlm.nih.gov/compound/146181819#section=2D-Structure
https://pubchem.ncbi.nlm.nih.gov/compound/146172254#section=2D-Structure
https://pubchem.ncbi.nlm.nih.gov/compound/146170194#section=2D-Structure
https://pubchem.ncbi.nlm.nih.gov/compound/146169789#section=2D-Structure
https://pubchem.ncbi.nlm.nih.gov/compound/14613283#section=2D-Structure
https://pubchem.ncbi.nlm.nih.gov/compound/14612041#section=2D-Structure
https://pubchem.ncbi.nlm.nih.gov/compound/146036197#section=2D-Structure
https://pubchem.ncbi.nlm.nih.gov/compound/146021372#section=2D-Structure
https://pubchem.ncbi.nlm.nih.gov/compound/146020895#section=2D-Structure
https://pubchem.ncbi.nlm.nih.gov/compound/145925931#section=2D-Structure
https://pubchem.ncbi.nlm.nih.gov/compound/145925867#section=2D-Structure
https://pubchem.ncbi.nlm.nih.gov/compound/14591610#section=2D-Structure
https://pubchem.ncbi.nlm.nih.gov/compound/145916051#section=2D-Structure
https://pubchem.ncbi.nlm.nih.gov/compound/145915999#section=2D-Structure
https://pubchem.ncbi.nlm.nih.gov/compound/145865472#section=2D-Structure
https://pubchem.ncbi.nlm.nih.gov/compound/145865471#section=2D-Structure
https://pubchem.ncbi.nlm.nih.gov/compound/14586#section=2D-Structure
https://pubchem.ncbi.nlm.nih.gov/compound/145836900#section=2D-Structure
https://pubchem.ncbi.nlm.nih.gov/compound/145834712#section=2D-Structure
https://pubchem.ncbi.nlm.nih.gov/compound/145834710#section=2D-Structure
https://pubchem.ncbi.nlm.nih.gov/compound/145823751#section=2D-Structure
https://pubchem.ncbi.nlm.nih.gov/compound/145808415#section=2D-Structure
https://pubchem.ncbi.nlm.nih.gov/compound/145807186#section=2D-Structure
https://pubchem.ncbi.nlm.nih.gov/compound/145804277#section=2D-Structure
https://pubchem.ncbi.nlm.nih.gov/compound/145802309#section=2D-Structure
https://pubchem.ncbi.nlm.nih.gov/compound/145798996#section=2D-Structure
https://pubchem.ncbi.nlm.nih.gov/compound/145795830#section=2D-Structure
https://pubchem.ncbi.nlm.nih.gov/compound/145791489#section=2D-Structure
https://pubchem.ncbi.nlm.nih.gov/compound/145776279#section=2D-Structure
https://pubchem.ncbi.nlm.nih.gov/compound/145771381#section=2D-Structure
https://pubchem.ncbi.nlm.nih.gov/compound/145770653#section=2D-Structure
https://pubchem.ncbi.nlm.nih.gov/compound/145769346#section=2D-Structure
https://pubchem.ncbi.nlm.nih.gov/compound/145769327#section=2D-Structure
https://pubchem.ncbi.nlm.nih.gov/compound/145756444#section=2D-Structure
https://pubchem.ncbi.nlm.nih.gov/compound/145754969#section=2D-Structure
https://pubchem.ncbi.nlm.nih.gov/compound/145747480#section=2D-Structure
https://pubchem.ncbi.nlm.nih.gov/compound/145743847#section=2D-Structure
https://pubchem.ncbi.nlm.nih.gov/compound/145738290#section=2D-Structure
https://pubchem.ncbi.nlm.nih.gov/compound/145731817#section=2D-Structure
https://pubchem.ncbi.nlm.nih.gov/compound/14572742#section=2D-Structure
https://pubchem.ncbi.nlm.nih.gov/compound/145699431#section=2D-Structure
https://pubchem.ncbi.nlm.nih.gov/compound/145694940#section=2D-Structure
https://pubchem.ncbi.nlm.nih.gov/compound/145690#section=2D-Structure
https://pubchem.ncbi.nlm.nih.gov/compound/145677932#section=2D-Structure
https://pubchem.ncbi.nlm.nih.gov/compound/145670309#section=2D-Structure
https://pubchem.ncbi.nlm.nih.gov/compound/145649515#section=2D-Structure
https://pubchem.ncbi.nlm.nih.gov/compound/145641090#section=2D-Structure
https://pubchem.ncbi.nlm.nih.gov/compound/145628208#section=2D-Structure
https://pubchem.ncbi.nlm.nih.gov/compound/145620765#section=2D-Structure
https://pubchem.ncbi.nlm.nih.gov/compound/145609574#section=2D-Structure
https://pubchem.ncbi.nlm.nih.gov/compound/145609415#section=2D-Structure
https://pubchem.ncbi.nlm.nih.gov/compound/145609394#section=2D-Structure

https://pubchem.ncbi.nlm.nih.gov/compound/145601518#section=2D-Structure
https://pubchem.ncbi.nlm.nih.gov/compound/145599263#section=2D-Structure
https://pubchem.ncbi.nlm.nih.gov/compound/145597418#section=2D-Structure
https://pubchem.ncbi.nlm.nih.gov/compound/145593856#section=2D-Structure
https://pubchem.ncbi.nlm.nih.gov/compound/145585699#section=2D-Structure
https://pubchem.ncbi.nlm.nih.gov/compound/145581416#section=2D-Structure
https://pubchem.ncbi.nlm.nih.gov/compound/145567243#section=2D-Structure
https://pubchem.ncbi.nlm.nih.gov/compound/145566966#section=2D-Structure
https://pubchem.ncbi.nlm.nih.gov/compound/145549922#section=2D-Structure
https://pubchem.ncbi.nlm.nih.gov/compound/14553#section=2D-Structure
https://pubchem.ncbi.nlm.nih.gov/compound/145527357#section=2D-Structure
https://pubchem.ncbi.nlm.nih.gov/compound/145518839#section=2D-Structure
https://pubchem.ncbi.nlm.nih.gov/compound/145518793#section=2D-Structure
https://pubchem.ncbi.nlm.nih.gov/compound/145518718#section=2D-Structure
https://pubchem.ncbi.nlm.nih.gov/compound/145518682#section=2D-Structure
https://pubchem.ncbi.nlm.nih.gov/compound/145513802#section=2D-Structure
https://pubchem.ncbi.nlm.nih.gov/compound/145510050#section=2D-Structure
https://pubchem.ncbi.nlm.nih.gov/compound/145498366#section=2D-Structure
https://pubchem.ncbi.nlm.nih.gov/compound/145498340#section=2D-Structure
https://pubchem.ncbi.nlm.nih.gov/compound/145486306#section=2D-Structure
https://pubchem.ncbi.nlm.nih.gov/compound/145447476#section=2D-Structure
https://pubchem.ncbi.nlm.nih.gov/compound/145438787#section=2D-Structure
https://pubchem.ncbi.nlm.nih.gov/compound/145436654#section=2D-Structure
https://pubchem.ncbi.nlm.nih.gov/compound/145422022#section=2D-Structure
https://pubchem.ncbi.nlm.nih.gov/compound/145412472#section=2D-Structure
https://pubchem.ncbi.nlm.nih.gov/compound/145409901#section=2D-Structure
https://pubchem.ncbi.nlm.nih.gov/compound/145406937#section=2D-Structure
https://pubchem.ncbi.nlm.nih.gov/compound/145396265#section=2D-Structure
https://pubchem.ncbi.nlm.nih.gov/compound/145373338#section=2D-Structure
https://pubchem.ncbi.nlm.nih.gov/compound/145373329#section=2D-Structure
https://pubchem.ncbi.nlm.nih.gov/compound/145373319#section=2D-Structure
https://pubchem.ncbi.nlm.nih.gov/compound/145373314#section=2D-Structure
https://pubchem.ncbi.nlm.nih.gov/compound/145373307#section=2D-Structure
https://pubchem.ncbi.nlm.nih.gov/compound/145361851#section=2D-Structure
https://pubchem.ncbi.nlm.nih.gov/compound/145353980#section=2D-Structure
https://pubchem.ncbi.nlm.nih.gov/compound/145350377#section=2D-Structure
https://pubchem.ncbi.nlm.nih.gov/compound/145350372#section=2D-Structure
https://pubchem.ncbi.nlm.nih.gov/compound/145348049#section=2D-Structure
https://pubchem.ncbi.nlm.nih.gov/compound/145346997#section=2D-Structure
https://pubchem.ncbi.nlm.nih.gov/compound/145325437#section=2D-Structure
https://pubchem.ncbi.nlm.nih.gov/compound/145288319#section=2D-Structure
https://pubchem.ncbi.nlm.nih.gov/compound/145276770#section=2D-Structure
https://pubchem.ncbi.nlm.nih.gov/compound/145275031#section=2D-Structure
https://pubchem.ncbi.nlm.nih.gov/compound/145261600#section=2D-Structure
https://pubchem.ncbi.nlm.nih.gov/compound/145260443#section=2D-Structure
https://pubchem.ncbi.nlm.nih.gov/compound/145242728#section=2D-Structure
https://pubchem.ncbi.nlm.nih.gov/compound/145204039#section=2D-Structure
https://pubchem.ncbi.nlm.nih.gov/compound/145195464#section=2D-Structure
https://pubchem.ncbi.nlm.nih.gov/compound/145187819#section=2D-Structure
https://pubchem.ncbi.nlm.nih.gov/compound/145180175#section=2D-Structure
https://pubchem.ncbi.nlm.nih.gov/compound/145156205#section=2D-Structure
https://pubchem.ncbi.nlm.nih.gov/compound/145154137#section=2D-Structure
https://pubchem.ncbi.nlm.nih.gov/compound/145151364#section=2D-Structure
https://pubchem.ncbi.nlm.nih.gov/compound/145134864#section=2D-Structure
https://pubchem.ncbi.nlm.nih.gov/compound/145132775#section=2D-Structure

https://pubchem.ncbi.nlm.nih.gov/compound/145128673#section=2D-Structure
https://pubchem.ncbi.nlm.nih.gov/compound/145100468#section=2D-Structure
https://pubchem.ncbi.nlm.nih.gov/compound/145100459#section=2D-Structure
https://pubchem.ncbi.nlm.nih.gov/compound/145097112#section=2D-Structure
https://pubchem.ncbi.nlm.nih.gov/compound/145082687#section=2D-Structure
https://pubchem.ncbi.nlm.nih.gov/compound/145067125#section=2D-Structure
https://pubchem.ncbi.nlm.nih.gov/compound/145059812#section=2D-Structure
https://pubchem.ncbi.nlm.nih.gov/compound/145053470#section=2D-Structure
https://pubchem.ncbi.nlm.nih.gov/compound/145053173#section=2D-Structure
https://pubchem.ncbi.nlm.nih.gov/compound/145051438#section=2D-Structure
https://pubchem.ncbi.nlm.nih.gov/compound/145051437#section=2D-Structure
https://pubchem.ncbi.nlm.nih.gov/compound/145051435#section=2D-Structure
https://pubchem.ncbi.nlm.nih.gov/compound/145051433#section=2D-Structure
https://pubchem.ncbi.nlm.nih.gov/compound/145051422#section=2D-Structure
https://pubchem.ncbi.nlm.nih.gov/compound/145051410#section=2D-Structure
https://pubchem.ncbi.nlm.nih.gov/compound/145049935#section=2D-Structure
https://pubchem.ncbi.nlm.nih.gov/compound/145022983#section=2D-Structure
https://pubchem.ncbi.nlm.nih.gov/compound/145003899#section=2D-Structure
https://pubchem.ncbi.nlm.nih.gov/compound/144993403#section=2D-Structure
https://pubchem.ncbi.nlm.nih.gov/compound/144976013#section=2D-Structure
https://pubchem.ncbi.nlm.nih.gov/compound/144945932#section=2D-Structure
https://pubchem.ncbi.nlm.nih.gov/compound/144939709#section=2D-Structure
https://pubchem.ncbi.nlm.nih.gov/compound/144939671#section=2D-Structure
https://pubchem.ncbi.nlm.nih.gov/compound/144939046#section=2D-Structure
https://pubchem.ncbi.nlm.nih.gov/compound/144916815#section=2D-Structure
https://pubchem.ncbi.nlm.nih.gov/compound/144901973#section=2D-Structure
https://pubchem.ncbi.nlm.nih.gov/compound/144899071#section=2D-Structure
https://pubchem.ncbi.nlm.nih.gov/compound/144896069#section=2D-Structure
https://pubchem.ncbi.nlm.nih.gov/compound/144867943#section=2D-Structure
https://pubchem.ncbi.nlm.nih.gov/compound/144857055#section=2D-Structure
https://pubchem.ncbi.nlm.nih.gov/compound/144857054#section=2D-Structure
https://pubchem.ncbi.nlm.nih.gov/compound/144827293#section=2D-Structure
https://pubchem.ncbi.nlm.nih.gov/compound/144827107#section=2D-Structure
https://pubchem.ncbi.nlm.nih.gov/compound/144815368#section=2D-Structure
https://pubchem.ncbi.nlm.nih.gov/compound/144813066#section=2D-Structure
https://pubchem.ncbi.nlm.nih.gov/compound/144800419#section=2D-Structure
https://pubchem.ncbi.nlm.nih.gov/compound/144792368#section=2D-Structure
https://pubchem.ncbi.nlm.nih.gov/compound/144792367#section=2D-Structure
https://pubchem.ncbi.nlm.nih.gov/compound/144792365#section=2D-Structure
https://pubchem.ncbi.nlm.nih.gov/compound/144792353#section=2D-Structure
https://pubchem.ncbi.nlm.nih.gov/compound/144783537#section=2D-Structure
https://pubchem.ncbi.nlm.nih.gov/compound/144780127#section=2D-Structure
https://pubchem.ncbi.nlm.nih.gov/compound/144772427#section=2D-Structure
https://pubchem.ncbi.nlm.nih.gov/compound/144741190#section=2D-Structure
https://pubchem.ncbi.nlm.nih.gov/compound/144696289#section=2D-Structure
https://pubchem.ncbi.nlm.nih.gov/compound/14469294#section=2D-Structure
https://pubchem.ncbi.nlm.nih.gov/compound/14469293#section=2D-Structure
https://pubchem.ncbi.nlm.nih.gov/compound/14469282#section=2D-Structure
https://pubchem.ncbi.nlm.nih.gov/compound/144683543#section=2D-Structure
https://pubchem.ncbi.nlm.nih.gov/compound/144676863#section=2D-Structure
https://pubchem.ncbi.nlm.nih.gov/compound/144660178#section=2D-Structure
https://pubchem.ncbi.nlm.nih.gov/compound/144658515#section=2D-Structure
https://pubchem.ncbi.nlm.nih.gov/compound/144588567#section=2D-Structure
https://pubchem.ncbi.nlm.nih.gov/compound/144588150#section=2D-Structure
https://pubchem.ncbi.nlm.nih.gov/compound/144583417#section=2D-Structure

https://pubchem.ncbi.nlm.nih.gov/compound/144578873#section=2D-Structure
https://pubchem.ncbi.nlm.nih.gov/compound/144562613#section=2D-Structure
https://pubchem.ncbi.nlm.nih.gov/compound/144553208#section=2D-Structure
https://pubchem.ncbi.nlm.nih.gov/compound/14450453#section=2D-Structure
https://pubchem.ncbi.nlm.nih.gov/compound/144496311#section=2D-Structure
https://pubchem.ncbi.nlm.nih.gov/compound/144474961#section=2D-Structure
https://pubchem.ncbi.nlm.nih.gov/compound/144462835#section=2D-Structure
https://pubchem.ncbi.nlm.nih.gov/compound/144458986#section=2D-Structure
https://pubchem.ncbi.nlm.nih.gov/compound/144449701#section=2D-Structure
https://pubchem.ncbi.nlm.nih.gov/compound/144445509#section=2D-Structure
https://pubchem.ncbi.nlm.nih.gov/compound/144421069#section=2D-Structure
https://pubchem.ncbi.nlm.nih.gov/compound/144420426#section=2D-Structure
https://pubchem.ncbi.nlm.nih.gov/compound/144411326#section=2D-Structure
https://pubchem.ncbi.nlm.nih.gov/compound/144405524#section=2D-Structure
https://pubchem.ncbi.nlm.nih.gov/compound/144371668#section=2D-Structure
https://pubchem.ncbi.nlm.nih.gov/compound/144371662#section=2D-Structure
https://pubchem.ncbi.nlm.nih.gov/compound/144371660#section=2D-Structure
https://pubchem.ncbi.nlm.nih.gov/compound/144335320#section=2D-Structure
https://pubchem.ncbi.nlm.nih.gov/compound/144325061#section=2D-Structure
https://pubchem.ncbi.nlm.nih.gov/compound/144324776#section=2D-Structure
https://pubchem.ncbi.nlm.nih.gov/compound/144321141#section=2D-Structure
https://pubchem.ncbi.nlm.nih.gov/compound/144318072#section=2D-Structure
https://pubchem.ncbi.nlm.nih.gov/compound/144300576#section=2D-Structure
https://pubchem.ncbi.nlm.nih.gov/compound/144300551#section=2D-Structure
https://pubchem.ncbi.nlm.nih.gov/compound/144291001#section=2D-Structure
https://pubchem.ncbi.nlm.nih.gov/compound/144278277#section=2D-Structure
https://pubchem.ncbi.nlm.nih.gov/compound/144273191#section=2D-Structure
https://pubchem.ncbi.nlm.nih.gov/compound/144266743#section=2D-Structure
https://pubchem.ncbi.nlm.nih.gov/compound/144266738#section=2D-Structure
https://pubchem.ncbi.nlm.nih.gov/compound/144262390#section=2D-Structure
https://pubchem.ncbi.nlm.nih.gov/compound/144261528#section=2D-Structure
https://pubchem.ncbi.nlm.nih.gov/compound/144257750#section=2D-Structure
https://pubchem.ncbi.nlm.nih.gov/compound/144241413#section=2D-Structure
https://pubchem.ncbi.nlm.nih.gov/compound/144195785#section=2D-Structure
https://pubchem.ncbi.nlm.nih.gov/compound/144182048#section=2D-Structure
https://pubchem.ncbi.nlm.nih.gov/compound/144182042#section=2D-Structure
https://pubchem.ncbi.nlm.nih.gov/compound/144178794#section=2D-Structure
https://pubchem.ncbi.nlm.nih.gov/compound/144160981#section=2D-Structure
https://pubchem.ncbi.nlm.nih.gov/compound/144141348#section=2D-Structure
https://pubchem.ncbi.nlm.nih.gov/compound/144127321#section=2D-Structure
https://pubchem.ncbi.nlm.nih.gov/compound/144125163#section=2D-Structure
https://pubchem.ncbi.nlm.nih.gov/compound/144090326#section=2D-Structure
https://pubchem.ncbi.nlm.nih.gov/compound/144090254#section=2D-Structure
https://pubchem.ncbi.nlm.nih.gov/compound/144089892#section=2D-Structure
https://pubchem.ncbi.nlm.nih.gov/compound/144077383#section=2D-Structure
https://pubchem.ncbi.nlm.nih.gov/compound/144056077#section=2D-Structure
https://pubchem.ncbi.nlm.nih.gov/compound/144028146#section=2D-Structure
https://pubchem.ncbi.nlm.nih.gov/compound/144018731#section=2D-Structure
https://pubchem.ncbi.nlm.nih.gov/compound/144015183#section=2D-Structure
https://pubchem.ncbi.nlm.nih.gov/compound/144006700#section=2D-Structure
https://pubchem.ncbi.nlm.nih.gov/compound/144006656#section=2D-Structure
https://pubchem.ncbi.nlm.nih.gov/compound/144006210#section=2D-Structure
https://pubchem.ncbi.nlm.nih.gov/compound/144006008#section=2D-Structure
https://pubchem.ncbi.nlm.nih.gov/compound/144003696#section=2D-Structure
https://pubchem.ncbi.nlm.nih.gov/compound/144003649#section=2D-Structure

https://pubchem.ncbi.nlm.nih.gov/compound/144000411#section=2D-Structure
https://pubchem.ncbi.nlm.nih.gov/compound/144000327#section=2D-Structure
https://pubchem.ncbi.nlm.nih.gov/compound/143986525#section=2D-Structure
https://pubchem.ncbi.nlm.nih.gov/compound/143986462#section=2D-Structure
https://pubchem.ncbi.nlm.nih.gov/compound/143986098#section=2D-Structure
https://pubchem.ncbi.nlm.nih.gov/compound/143983906#section=2D-Structure
https://pubchem.ncbi.nlm.nih.gov/compound/143982064#section=2D-Structure
https://pubchem.ncbi.nlm.nih.gov/compound/143980214#section=2D-Structure
https://pubchem.ncbi.nlm.nih.gov/compound/143979720#section=2D-Structure
https://pubchem.ncbi.nlm.nih.gov/compound/143972769#section=2D-Structure
https://pubchem.ncbi.nlm.nih.gov/compound/143900961#section=2D-Structure
https://pubchem.ncbi.nlm.nih.gov/compound/143900672#section=2D-Structure
https://pubchem.ncbi.nlm.nih.gov/compound/143900667#section=2D-Structure
https://pubchem.ncbi.nlm.nih.gov/compound/143897031#section=2D-Structure
https://pubchem.ncbi.nlm.nih.gov/compound/143881604#section=2D-Structure
https://pubchem.ncbi.nlm.nih.gov/compound/14387325#section=2D-Structure
https://pubchem.ncbi.nlm.nih.gov/compound/14387324#section=2D-Structure
https://pubchem.ncbi.nlm.nih.gov/compound/143869501#section=2D-Structure
https://pubchem.ncbi.nlm.nih.gov/compound/143848361#section=2D-Structure
https://pubchem.ncbi.nlm.nih.gov/compound/143848257#section=2D-Structure
https://pubchem.ncbi.nlm.nih.gov/compound/143844081#section=2D-Structure
https://pubchem.ncbi.nlm.nih.gov/compound/143839449#section=2D-Structure
https://pubchem.ncbi.nlm.nih.gov/compound/143834878#section=2D-Structure
https://pubchem.ncbi.nlm.nih.gov/compound/14381679#section=2D-Structure
https://pubchem.ncbi.nlm.nih.gov/compound/143812818#section=2D-Structure
https://pubchem.ncbi.nlm.nih.gov/compound/143808940#section=2D-Structure
https://pubchem.ncbi.nlm.nih.gov/compound/143801763#section=2D-Structure
https://pubchem.ncbi.nlm.nih.gov/compound/143781901#section=2D-Structure
https://pubchem.ncbi.nlm.nih.gov/compound/143760671#section=2D-Structure
https://pubchem.ncbi.nlm.nih.gov/compound/143742536#section=2D-Structure
https://pubchem.ncbi.nlm.nih.gov/compound/143742497#section=2D-Structure
https://pubchem.ncbi.nlm.nih.gov/compound/143742311#section=2D-Structure
https://pubchem.ncbi.nlm.nih.gov/compound/143727580#section=2D-Structure
https://pubchem.ncbi.nlm.nih.gov/compound/143726788#section=2D-Structure
https://pubchem.ncbi.nlm.nih.gov/compound/143722496#section=2D-Structure
https://pubchem.ncbi.nlm.nih.gov/compound/143722473#section=2D-Structure
https://pubchem.ncbi.nlm.nih.gov/compound/143708552#section=2D-Structure
https://pubchem.ncbi.nlm.nih.gov/compound/143688550#section=2D-Structure
https://pubchem.ncbi.nlm.nih.gov/compound/143684670#section=2D-Structure
https://pubchem.ncbi.nlm.nih.gov/compound/143677057#section=2D-Structure
https://pubchem.ncbi.nlm.nih.gov/compound/143667780#section=2D-Structure
https://pubchem.ncbi.nlm.nih.gov/compound/143654284#section=2D-Structure
https://pubchem.ncbi.nlm.nih.gov/compound/143653350#section=2D-Structure
https://pubchem.ncbi.nlm.nih.gov/compound/143646396#section=2D-Structure
https://pubchem.ncbi.nlm.nih.gov/compound/143643#section=2D-Structure
https://pubchem.ncbi.nlm.nih.gov/compound/143637279#section=2D-Structure
https://pubchem.ncbi.nlm.nih.gov/compound/143637263#section=2D-Structure
https://pubchem.ncbi.nlm.nih.gov/compound/143637256#section=2D-Structure
https://pubchem.ncbi.nlm.nih.gov/compound/143634039#section=2D-Structure
https://pubchem.ncbi.nlm.nih.gov/compound/143620419#section=2D-Structure
https://pubchem.ncbi.nlm.nih.gov/compound/143620367#section=2D-Structure
https://pubchem.ncbi.nlm.nih.gov/compound/143620344#section=2D-Structure
https://pubchem.ncbi.nlm.nih.gov/compound/143619742#section=2D-Structure
https://pubchem.ncbi.nlm.nih.gov/compound/143619725#section=2D-Structure
https://pubchem.ncbi.nlm.nih.gov/compound/143617114#section=2D-Structure

https://pubchem.ncbi.nlm.nih.gov/compound/14360019#section=2D-Structure
https://pubchem.ncbi.nlm.nih.gov/compound/143597752#section=2D-Structure
https://pubchem.ncbi.nlm.nih.gov/compound/143597735#section=2D-Structure
https://pubchem.ncbi.nlm.nih.gov/compound/143582602#section=2D-Structure
https://pubchem.ncbi.nlm.nih.gov/compound/143572010#section=2D-Structure
https://pubchem.ncbi.nlm.nih.gov/compound/143566685#section=2D-Structure
https://pubchem.ncbi.nlm.nih.gov/compound/143566676#section=2D-Structure
https://pubchem.ncbi.nlm.nih.gov/compound/143566632#section=2D-Structure
https://pubchem.ncbi.nlm.nih.gov/compound/143559778#section=2D-Structure
https://pubchem.ncbi.nlm.nih.gov/compound/143539678#section=2D-Structure
https://pubchem.ncbi.nlm.nih.gov/compound/143532024#section=2D-Structure
https://pubchem.ncbi.nlm.nih.gov/compound/143530724#section=2D-Structure
https://pubchem.ncbi.nlm.nih.gov/compound/143519558#section=2D-Structure
https://pubchem.ncbi.nlm.nih.gov/compound/143516790#section=2D-Structure
https://pubchem.ncbi.nlm.nih.gov/compound/143516073#section=2D-Structure
https://pubchem.ncbi.nlm.nih.gov/compound/143504988#section=2D-Structure
https://pubchem.ncbi.nlm.nih.gov/compound/143500537#section=2D-Structure
https://pubchem.ncbi.nlm.nih.gov/compound/143465436#section=2D-Structure
https://pubchem.ncbi.nlm.nih.gov/compound/143463542#section=2D-Structure
https://pubchem.ncbi.nlm.nih.gov/compound/14344339#section=2D-Structure
https://pubchem.ncbi.nlm.nih.gov/compound/143380612#section=2D-Structure
https://pubchem.ncbi.nlm.nih.gov/compound/143380608#section=2D-Structure
https://pubchem.ncbi.nlm.nih.gov/compound/143369868#section=2D-Structure
https://pubchem.ncbi.nlm.nih.gov/compound/143364701#section=2D-Structure
https://pubchem.ncbi.nlm.nih.gov/compound/143339874#section=2D-Structure
https://pubchem.ncbi.nlm.nih.gov/compound/143335337#section=2D-Structure
https://pubchem.ncbi.nlm.nih.gov/compound/143335285#section=2D-Structure
https://pubchem.ncbi.nlm.nih.gov/compound/143324808#section=2D-Structure
https://pubchem.ncbi.nlm.nih.gov/compound/143308958#section=2D-Structure
https://pubchem.ncbi.nlm.nih.gov/compound/143289437#section=2D-Structure
https://pubchem.ncbi.nlm.nih.gov/compound/143232117#section=2D-Structure
https://pubchem.ncbi.nlm.nih.gov/compound/14322637#section=2D-Structure
https://pubchem.ncbi.nlm.nih.gov/compound/143224894#section=2D-Structure
https://pubchem.ncbi.nlm.nih.gov/compound/143194881#section=2D-Structure
https://pubchem.ncbi.nlm.nih.gov/compound/143168969#section=2D-Structure
https://pubchem.ncbi.nlm.nih.gov/compound/143151815#section=2D-Structure
https://pubchem.ncbi.nlm.nih.gov/compound/143126712#section=2D-Structure
https://pubchem.ncbi.nlm.nih.gov/compound/143100309#section=2D-Structure
https://pubchem.ncbi.nlm.nih.gov/compound/143081797#section=2D-Structure
https://pubchem.ncbi.nlm.nih.gov/compound/143081485#section=2D-Structure
https://pubchem.ncbi.nlm.nih.gov/compound/143064346#section=2D-Structure
https://pubchem.ncbi.nlm.nih.gov/compound/143060503#section=2D-Structure
https://pubchem.ncbi.nlm.nih.gov/compound/143060330#section=2D-Structure
https://pubchem.ncbi.nlm.nih.gov/compound/143058575#section=2D-Structure
https://pubchem.ncbi.nlm.nih.gov/compound/143058018#section=2D-Structure
https://pubchem.ncbi.nlm.nih.gov/compound/143056361#section=2D-Structure
https://pubchem.ncbi.nlm.nih.gov/compound/143056358#section=2D-Structure
https://pubchem.ncbi.nlm.nih.gov/compound/143056351#section=2D-Structure
https://pubchem.ncbi.nlm.nih.gov/compound/143047190#section=2D-Structure
https://pubchem.ncbi.nlm.nih.gov/compound/143044#section=2D-Structure
https://pubchem.ncbi.nlm.nih.gov/compound/143027718#section=2D-Structure
https://pubchem.ncbi.nlm.nih.gov/compound/143022559#section=2D-Structure
https://pubchem.ncbi.nlm.nih.gov/compound/142986252#section=2D-Structure
https://pubchem.ncbi.nlm.nih.gov/compound/142967710#section=2D-Structure
https://pubchem.ncbi.nlm.nih.gov/compound/142967440#section=2D-Structure

https://pubchem.ncbi.nlm.nih.gov/compound/142961394#section=2D-Structure
https://pubchem.ncbi.nlm.nih.gov/compound/142949734#section=2D-Structure
https://pubchem.ncbi.nlm.nih.gov/compound/14292604#section=2D-Structure
https://pubchem.ncbi.nlm.nih.gov/compound/142881925#section=2D-Structure
https://pubchem.ncbi.nlm.nih.gov/compound/142878674#section=2D-Structure
https://pubchem.ncbi.nlm.nih.gov/compound/142878671#section=2D-Structure
https://pubchem.ncbi.nlm.nih.gov/compound/142868#section=2D-Structure
https://pubchem.ncbi.nlm.nih.gov/compound/142867878#section=2D-Structure
https://pubchem.ncbi.nlm.nih.gov/compound/142867860#section=2D-Structure
https://pubchem.ncbi.nlm.nih.gov/compound/142859803#section=2D-Structure
https://pubchem.ncbi.nlm.nih.gov/compound/142853848#section=2D-Structure
https://pubchem.ncbi.nlm.nih.gov/compound/142853838#section=2D-Structure
https://pubchem.ncbi.nlm.nih.gov/compound/142853836#section=2D-Structure
https://pubchem.ncbi.nlm.nih.gov/compound/142848396#section=2D-Structure
https://pubchem.ncbi.nlm.nih.gov/compound/142848393#section=2D-Structure
https://pubchem.ncbi.nlm.nih.gov/compound/142848372#section=2D-Structure
https://pubchem.ncbi.nlm.nih.gov/compound/142848348#section=2D-Structure
https://pubchem.ncbi.nlm.nih.gov/compound/142846339#section=2D-Structure
https://pubchem.ncbi.nlm.nih.gov/compound/142842316#section=2D-Structure
https://pubchem.ncbi.nlm.nih.gov/compound/142822281#section=2D-Structure
https://pubchem.ncbi.nlm.nih.gov/compound/142765855#section=2D-Structure
https://pubchem.ncbi.nlm.nih.gov/compound/142757153#section=2D-Structure
https://pubchem.ncbi.nlm.nih.gov/compound/142755500#section=2D-Structure
https://pubchem.ncbi.nlm.nih.gov/compound/142755491#section=2D-Structure
https://pubchem.ncbi.nlm.nih.gov/compound/142755489#section=2D-Structure
https://pubchem.ncbi.nlm.nih.gov/compound/14275443#section=2D-Structure
https://pubchem.ncbi.nlm.nih.gov/compound/142750912#section=2D-Structure
https://pubchem.ncbi.nlm.nih.gov/compound/142750031#section=2D-Structure
https://pubchem.ncbi.nlm.nih.gov/compound/142750030#section=2D-Structure
https://pubchem.ncbi.nlm.nih.gov/compound/14274984#section=2D-Structure
https://pubchem.ncbi.nlm.nih.gov/compound/142742178#section=2D-Structure
https://pubchem.ncbi.nlm.nih.gov/compound/142740890#section=2D-Structure
https://pubchem.ncbi.nlm.nih.gov/compound/142733150#section=2D-Structure
https://pubchem.ncbi.nlm.nih.gov/compound/142729183#section=2D-Structure
https://pubchem.ncbi.nlm.nih.gov/compound/142728922#section=2D-Structure
https://pubchem.ncbi.nlm.nih.gov/compound/142728330#section=2D-Structure
https://pubchem.ncbi.nlm.nih.gov/compound/142728329#section=2D-Structure
https://pubchem.ncbi.nlm.nih.gov/compound/142722346#section=2D-Structure
https://pubchem.ncbi.nlm.nih.gov/compound/142720473#section=2D-Structure
https://pubchem.ncbi.nlm.nih.gov/compound/142720468#section=2D-Structure
https://pubchem.ncbi.nlm.nih.gov/compound/142720464#section=2D-Structure
https://pubchem.ncbi.nlm.nih.gov/compound/142720451#section=2D-Structure
https://pubchem.ncbi.nlm.nih.gov/compound/142720432#section=2D-Structure
https://pubchem.ncbi.nlm.nih.gov/compound/142720424#section=2D-Structure
https://pubchem.ncbi.nlm.nih.gov/compound/142715459#section=2D-Structure
https://pubchem.ncbi.nlm.nih.gov/compound/142713665#section=2D-Structure
https://pubchem.ncbi.nlm.nih.gov/compound/142712714#section=2D-Structure
https://pubchem.ncbi.nlm.nih.gov/compound/142711702#section=2D-Structure
https://pubchem.ncbi.nlm.nih.gov/compound/142694341#section=2D-Structure
https://pubchem.ncbi.nlm.nih.gov/compound/142681625#section=2D-Structure
https://pubchem.ncbi.nlm.nih.gov/compound/142671725#section=2D-Structure
https://pubchem.ncbi.nlm.nih.gov/compound/142669566#section=2D-Structure
https://pubchem.ncbi.nlm.nih.gov/compound/14265486#section=2D-Structure
https://pubchem.ncbi.nlm.nih.gov/compound/142652569#section=2D-Structure
https://pubchem.ncbi.nlm.nih.gov/compound/142650978#section=2D-Structure

https://pubchem.ncbi.nlm.nih.gov/compound/142597845#section=2D-Structure
https://pubchem.ncbi.nlm.nih.gov/compound/142569#section=2D-Structure
https://pubchem.ncbi.nlm.nih.gov/compound/142526108#section=2D-Structure
https://pubchem.ncbi.nlm.nih.gov/compound/142515579#section=2D-Structure
https://pubchem.ncbi.nlm.nih.gov/compound/142497127#section=2D-Structure
https://pubchem.ncbi.nlm.nih.gov/compound/142495844#section=2D-Structure
https://pubchem.ncbi.nlm.nih.gov/compound/14241793#section=2D-Structure
https://pubchem.ncbi.nlm.nih.gov/compound/142384787#section=2D-Structure
https://pubchem.ncbi.nlm.nih.gov/compound/142380407#section=2D-Structure
https://pubchem.ncbi.nlm.nih.gov/compound/142355484#section=2D-Structure
https://pubchem.ncbi.nlm.nih.gov/compound/142355478#section=2D-Structure
https://pubchem.ncbi.nlm.nih.gov/compound/142355469#section=2D-Structure
https://pubchem.ncbi.nlm.nih.gov/compound/142353105#section=2D-Structure
https://pubchem.ncbi.nlm.nih.gov/compound/142336406#section=2D-Structure
https://pubchem.ncbi.nlm.nih.gov/compound/142336391#section=2D-Structure
https://pubchem.ncbi.nlm.nih.gov/compound/142335686#section=2D-Structure
https://pubchem.ncbi.nlm.nih.gov/compound/142298122#section=2D-Structure
https://pubchem.ncbi.nlm.nih.gov/compound/142298034#section=2D-Structure
https://pubchem.ncbi.nlm.nih.gov/compound/142297911#section=2D-Structure
https://pubchem.ncbi.nlm.nih.gov/compound/142297648#section=2D-Structure
https://pubchem.ncbi.nlm.nih.gov/compound/14228202#section=2D-Structure
https://pubchem.ncbi.nlm.nih.gov/compound/142275348#section=2D-Structure
https://pubchem.ncbi.nlm.nih.gov/compound/142255741#section=2D-Structure
https://pubchem.ncbi.nlm.nih.gov/compound/142204276#section=2D-Structure
https://pubchem.ncbi.nlm.nih.gov/compound/142191148#section=2D-Structure
https://pubchem.ncbi.nlm.nih.gov/compound/142105981#section=2D-Structure
https://pubchem.ncbi.nlm.nih.gov/compound/142105180#section=2D-Structure
https://pubchem.ncbi.nlm.nih.gov/compound/142105163#section=2D-Structure
https://pubchem.ncbi.nlm.nih.gov/compound/142090433#section=2D-Structure
https://pubchem.ncbi.nlm.nih.gov/compound/142089814#section=2D-Structure
https://pubchem.ncbi.nlm.nih.gov/compound/142075408#section=2D-Structure
https://pubchem.ncbi.nlm.nih.gov/compound/142022025#section=2D-Structure
https://pubchem.ncbi.nlm.nih.gov/compound/141998214#section=2D-Structure
https://pubchem.ncbi.nlm.nih.gov/compound/141998192#section=2D-Structure
https://pubchem.ncbi.nlm.nih.gov/compound/141993494#section=2D-Structure
https://pubchem.ncbi.nlm.nih.gov/compound/14198212#section=2D-Structure
https://pubchem.ncbi.nlm.nih.gov/compound/141981319#section=2D-Structure
https://pubchem.ncbi.nlm.nih.gov/compound/141968286#section=2D-Structure
https://pubchem.ncbi.nlm.nih.gov/compound/141966431#section=2D-Structure
https://pubchem.ncbi.nlm.nih.gov/compound/141928352#section=2D-Structure
https://pubchem.ncbi.nlm.nih.gov/compound/141928350#section=2D-Structure
https://pubchem.ncbi.nlm.nih.gov/compound/141920540#section=2D-Structure
https://pubchem.ncbi.nlm.nih.gov/compound/141919722#section=2D-Structure
https://pubchem.ncbi.nlm.nih.gov/compound/141916729#section=2D-Structure
https://pubchem.ncbi.nlm.nih.gov/compound/141916#section=2D-Structure
https://pubchem.ncbi.nlm.nih.gov/compound/141901970#section=2D-Structure
https://pubchem.ncbi.nlm.nih.gov/compound/141885344#section=2D-Structure
https://pubchem.ncbi.nlm.nih.gov/compound/141883919#section=2D-Structure
https://pubchem.ncbi.nlm.nih.gov/compound/141881453#section=2D-Structure
https://pubchem.ncbi.nlm.nih.gov/compound/141868700#section=2D-Structure
https://pubchem.ncbi.nlm.nih.gov/compound/141854302#section=2D-Structure
https://pubchem.ncbi.nlm.nih.gov/compound/141845326#section=2D-Structure
https://pubchem.ncbi.nlm.nih.gov/compound/141838020#section=2D-Structure
https://pubchem.ncbi.nlm.nih.gov/compound/141820989#section=2D-Structure
https://pubchem.ncbi.nlm.nih.gov/compound/141809097#section=2D-Structure

https://pubchem.ncbi.nlm.nih.gov/compound/141794021#section=2D-Structure
https://pubchem.ncbi.nlm.nih.gov/compound/141792794#section=2D-Structure
https://pubchem.ncbi.nlm.nih.gov/compound/141791864#section=2D-Structure
https://pubchem.ncbi.nlm.nih.gov/compound/141786276#section=2D-Structure
https://pubchem.ncbi.nlm.nih.gov/compound/141782954#section=2D-Structure
https://pubchem.ncbi.nlm.nih.gov/compound/141775357#section=2D-Structure
https://pubchem.ncbi.nlm.nih.gov/compound/141775157#section=2D-Structure
https://pubchem.ncbi.nlm.nih.gov/compound/141772315#section=2D-Structure
https://pubchem.ncbi.nlm.nih.gov/compound/141765316#section=2D-Structure
https://pubchem.ncbi.nlm.nih.gov/compound/141764733#section=2D-Structure
https://pubchem.ncbi.nlm.nih.gov/compound/141750649#section=2D-Structure
https://pubchem.ncbi.nlm.nih.gov/compound/141750648#section=2D-Structure
https://pubchem.ncbi.nlm.nih.gov/compound/141750647#section=2D-Structure
https://pubchem.ncbi.nlm.nih.gov/compound/141750645#section=2D-Structure
https://pubchem.ncbi.nlm.nih.gov/compound/141739535#section=2D-Structure
https://pubchem.ncbi.nlm.nih.gov/compound/141731786#section=2D-Structure
https://pubchem.ncbi.nlm.nih.gov/compound/141729682#section=2D-Structure
https://pubchem.ncbi.nlm.nih.gov/compound/141719264#section=2D-Structure
https://pubchem.ncbi.nlm.nih.gov/compound/141718074#section=2D-Structure
https://pubchem.ncbi.nlm.nih.gov/compound/14166820#section=2D-Structure
https://pubchem.ncbi.nlm.nih.gov/compound/141657961#section=2D-Structure
https://pubchem.ncbi.nlm.nih.gov/compound/141657956#section=2D-Structure
https://pubchem.ncbi.nlm.nih.gov/compound/141645945#section=2D-Structure
https://pubchem.ncbi.nlm.nih.gov/compound/141610337#section=2D-Structure
https://pubchem.ncbi.nlm.nih.gov/compound/141605244#section=2D-Structure
https://pubchem.ncbi.nlm.nih.gov/compound/141604129#section=2D-Structure
https://pubchem.ncbi.nlm.nih.gov/compound/141593115#section=2D-Structure
https://pubchem.ncbi.nlm.nih.gov/compound/141586640#section=2D-Structure
https://pubchem.ncbi.nlm.nih.gov/compound/141578654#section=2D-Structure
https://pubchem.ncbi.nlm.nih.gov/compound/141578007#section=2D-Structure
https://pubchem.ncbi.nlm.nih.gov/compound/141576484#section=2D-Structure
https://pubchem.ncbi.nlm.nih.gov/compound/141559098#section=2D-Structure
https://pubchem.ncbi.nlm.nih.gov/compound/141555187#section=2D-Structure
https://pubchem.ncbi.nlm.nih.gov/compound/141547413#section=2D-Structure
https://pubchem.ncbi.nlm.nih.gov/compound/141543483#section=2D-Structure
https://pubchem.ncbi.nlm.nih.gov/compound/141535651#section=2D-Structure
https://pubchem.ncbi.nlm.nih.gov/compound/141532934#section=2D-Structure
https://pubchem.ncbi.nlm.nih.gov/compound/141505598#section=2D-Structure
https://pubchem.ncbi.nlm.nih.gov/compound/141504372#section=2D-Structure
https://pubchem.ncbi.nlm.nih.gov/compound/141496502#section=2D-Structure
https://pubchem.ncbi.nlm.nih.gov/compound/141496501#section=2D-Structure
https://pubchem.ncbi.nlm.nih.gov/compound/141496293#section=2D-Structure
https://pubchem.ncbi.nlm.nih.gov/compound/141496292#section=2D-Structure
https://pubchem.ncbi.nlm.nih.gov/compound/141488955#section=2D-Structure
https://pubchem.ncbi.nlm.nih.gov/compound/141487942#section=2D-Structure
https://pubchem.ncbi.nlm.nih.gov/compound/141487865#section=2D-Structure
https://pubchem.ncbi.nlm.nih.gov/compound/141487317#section=2D-Structure
https://pubchem.ncbi.nlm.nih.gov/compound/141483487#section=2D-Structure
https://pubchem.ncbi.nlm.nih.gov/compound/1414825#section=2D-Structure
https://pubchem.ncbi.nlm.nih.gov/compound/141471809#section=2D-Structure
https://pubchem.ncbi.nlm.nih.gov/compound/141467601#section=2D-Structure
https://pubchem.ncbi.nlm.nih.gov/compound/141464992#section=2D-Structure
https://pubchem.ncbi.nlm.nih.gov/compound/141454441#section=2D-Structure
https://pubchem.ncbi.nlm.nih.gov/compound/141451311#section=2D-Structure
https://pubchem.ncbi.nlm.nih.gov/compound/141448362#section=2D-Structure

https://pubchem.ncbi.nlm.nih.gov/compound/141446879#section=2D-Structure
https://pubchem.ncbi.nlm.nih.gov/compound/141446877#section=2D-Structure
https://pubchem.ncbi.nlm.nih.gov/compound/141446875#section=2D-Structure
https://pubchem.ncbi.nlm.nih.gov/compound/141423037#section=2D-Structure
https://pubchem.ncbi.nlm.nih.gov/compound/141422748#section=2D-Structure
https://pubchem.ncbi.nlm.nih.gov/compound/141421692#section=2D-Structure
https://pubchem.ncbi.nlm.nih.gov/compound/141417456#section=2D-Structure
https://pubchem.ncbi.nlm.nih.gov/compound/141416675#section=2D-Structure
https://pubchem.ncbi.nlm.nih.gov/compound/141414621#section=2D-Structure
https://pubchem.ncbi.nlm.nih.gov/compound/141412114#section=2D-Structure
https://pubchem.ncbi.nlm.nih.gov/compound/141412095#section=2D-Structure
https://pubchem.ncbi.nlm.nih.gov/compound/141401127#section=2D-Structure
https://pubchem.ncbi.nlm.nih.gov/compound/141393655#section=2D-Structure
https://pubchem.ncbi.nlm.nih.gov/compound/141387921#section=2D-Structure
https://pubchem.ncbi.nlm.nih.gov/compound/141384360#section=2D-Structure
https://pubchem.ncbi.nlm.nih.gov/compound/141383863#section=2D-Structure
https://pubchem.ncbi.nlm.nih.gov/compound/141378072#section=2D-Structure
https://pubchem.ncbi.nlm.nih.gov/compound/141376889#section=2D-Structure
https://pubchem.ncbi.nlm.nih.gov/compound/141375502#section=2D-Structure
https://pubchem.ncbi.nlm.nih.gov/compound/141363857#section=2D-Structure
https://pubchem.ncbi.nlm.nih.gov/compound/141362707#section=2D-Structure
https://pubchem.ncbi.nlm.nih.gov/compound/141358024#section=2D-Structure
https://pubchem.ncbi.nlm.nih.gov/compound/141335330#section=2D-Structure
https://pubchem.ncbi.nlm.nih.gov/compound/141334479#section=2D-Structure
https://pubchem.ncbi.nlm.nih.gov/compound/141329610#section=2D-Structure
https://pubchem.ncbi.nlm.nih.gov/compound/141328669#section=2D-Structure
https://pubchem.ncbi.nlm.nih.gov/compound/141325326#section=2D-Structure
https://pubchem.ncbi.nlm.nih.gov/compound/141320846#section=2D-Structure
https://pubchem.ncbi.nlm.nih.gov/compound/141320842#section=2D-Structure
https://pubchem.ncbi.nlm.nih.gov/compound/141319851#section=2D-Structure
https://pubchem.ncbi.nlm.nih.gov/compound/141319389#section=2D-Structure
https://pubchem.ncbi.nlm.nih.gov/compound/141319387#section=2D-Structure
https://pubchem.ncbi.nlm.nih.gov/compound/141318771#section=2D-Structure
https://pubchem.ncbi.nlm.nih.gov/compound/141318768#section=2D-Structure
https://pubchem.ncbi.nlm.nih.gov/compound/141318765#section=2D-Structure
https://pubchem.ncbi.nlm.nih.gov/compound/14131694#section=2D-Structure
https://pubchem.ncbi.nlm.nih.gov/compound/14131687#section=2D-Structure
https://pubchem.ncbi.nlm.nih.gov/compound/141315212#section=2D-Structure
https://pubchem.ncbi.nlm.nih.gov/compound/141312220#section=2D-Structure
https://pubchem.ncbi.nlm.nih.gov/compound/141295813#section=2D-Structure
https://pubchem.ncbi.nlm.nih.gov/compound/141294990#section=2D-Structure
https://pubchem.ncbi.nlm.nih.gov/compound/141292813#section=2D-Structure
https://pubchem.ncbi.nlm.nih.gov/compound/141291176#section=2D-Structure
https://pubchem.ncbi.nlm.nih.gov/compound/141282974#section=2D-Structure
https://pubchem.ncbi.nlm.nih.gov/compound/141269387#section=2D-Structure
https://pubchem.ncbi.nlm.nih.gov/compound/141269185#section=2D-Structure
https://pubchem.ncbi.nlm.nih.gov/compound/141269156#section=2D-Structure
https://pubchem.ncbi.nlm.nih.gov/compound/141268074#section=2D-Structure
https://pubchem.ncbi.nlm.nih.gov/compound/141254916#section=2D-Structure
https://pubchem.ncbi.nlm.nih.gov/compound/141252618#section=2D-Structure
https://pubchem.ncbi.nlm.nih.gov/compound/141251593#section=2D-Structure
https://pubchem.ncbi.nlm.nih.gov/compound/141247466#section=2D-Structure
https://pubchem.ncbi.nlm.nih.gov/compound/141243404#section=2D-Structure
https://pubchem.ncbi.nlm.nih.gov/compound/141212089#section=2D-Structure
https://pubchem.ncbi.nlm.nih.gov/compound/141208305#section=2D-Structure

https://pubchem.ncbi.nlm.nih.gov/compound/141199256#section=2D-Structure
https://pubchem.ncbi.nlm.nih.gov/compound/141189132#section=2D-Structure
https://pubchem.ncbi.nlm.nih.gov/compound/141188773#section=2D-Structure
https://pubchem.ncbi.nlm.nih.gov/compound/141177159#section=2D-Structure
https://pubchem.ncbi.nlm.nih.gov/compound/141176037#section=2D-Structure
https://pubchem.ncbi.nlm.nih.gov/compound/141175499#section=2D-Structure
https://pubchem.ncbi.nlm.nih.gov/compound/141171641#section=2D-Structure
https://pubchem.ncbi.nlm.nih.gov/compound/141171636#section=2D-Structure
https://pubchem.ncbi.nlm.nih.gov/compound/141164119#section=2D-Structure
https://pubchem.ncbi.nlm.nih.gov/compound/141162563#section=2D-Structure
https://pubchem.ncbi.nlm.nih.gov/compound/141162452#section=2D-Structure
https://pubchem.ncbi.nlm.nih.gov/compound/141162451#section=2D-Structure
https://pubchem.ncbi.nlm.nih.gov/compound/141158773#section=2D-Structure
https://pubchem.ncbi.nlm.nih.gov/compound/141146385#section=2D-Structure
https://pubchem.ncbi.nlm.nih.gov/compound/141144432#section=2D-Structure
https://pubchem.ncbi.nlm.nih.gov/compound/141142176#section=2D-Structure
https://pubchem.ncbi.nlm.nih.gov/compound/141142172#section=2D-Structure
https://pubchem.ncbi.nlm.nih.gov/compound/141140951#section=2D-Structure
https://pubchem.ncbi.nlm.nih.gov/compound/141138179#section=2D-Structure
https://pubchem.ncbi.nlm.nih.gov/compound/141134375#section=2D-Structure
https://pubchem.ncbi.nlm.nih.gov/compound/141130611#section=2D-Structure
https://pubchem.ncbi.nlm.nih.gov/compound/141130602#section=2D-Structure
https://pubchem.ncbi.nlm.nih.gov/compound/141130601#section=2D-Structure
https://pubchem.ncbi.nlm.nih.gov/compound/141125023#section=2D-Structure
https://pubchem.ncbi.nlm.nih.gov/compound/141123349#section=2D-Structure
https://pubchem.ncbi.nlm.nih.gov/compound/141120393#section=2D-Structure
https://pubchem.ncbi.nlm.nih.gov/compound/141118489#section=2D-Structure
https://pubchem.ncbi.nlm.nih.gov/compound/141114871#section=2D-Structure
https://pubchem.ncbi.nlm.nih.gov/compound/141114481#section=2D-Structure
https://pubchem.ncbi.nlm.nih.gov/compound/141113034#section=2D-Structure
https://pubchem.ncbi.nlm.nih.gov/compound/141101471#section=2D-Structure
https://pubchem.ncbi.nlm.nih.gov/compound/141088000#section=2D-Structure
https://pubchem.ncbi.nlm.nih.gov/compound/141087353#section=2D-Structure
https://pubchem.ncbi.nlm.nih.gov/compound/141084745#section=2D-Structure
https://pubchem.ncbi.nlm.nih.gov/compound/141083836#section=2D-Structure
https://pubchem.ncbi.nlm.nih.gov/compound/141079932#section=2D-Structure
https://pubchem.ncbi.nlm.nih.gov/compound/141077725#section=2D-Structure
https://pubchem.ncbi.nlm.nih.gov/compound/141073931#section=2D-Structure
https://pubchem.ncbi.nlm.nih.gov/compound/141072450#section=2D-Structure
https://pubchem.ncbi.nlm.nih.gov/compound/141072383#section=2D-Structure
https://pubchem.ncbi.nlm.nih.gov/compound/141072041#section=2D-Structure
https://pubchem.ncbi.nlm.nih.gov/compound/141071793#section=2D-Structure
https://pubchem.ncbi.nlm.nih.gov/compound/141069632#section=2D-Structure
https://pubchem.ncbi.nlm.nih.gov/compound/141069631#section=2D-Structure
https://pubchem.ncbi.nlm.nih.gov/compound/141066475#section=2D-Structure
https://pubchem.ncbi.nlm.nih.gov/compound/141059905#section=2D-Structure
https://pubchem.ncbi.nlm.nih.gov/compound/141059904#section=2D-Structure
https://pubchem.ncbi.nlm.nih.gov/compound/141057621#section=2D-Structure
https://pubchem.ncbi.nlm.nih.gov/compound/141055482#section=2D-Structure
https://pubchem.ncbi.nlm.nih.gov/compound/141053576#section=2D-Structure
https://pubchem.ncbi.nlm.nih.gov/compound/141049239#section=2D-Structure
https://pubchem.ncbi.nlm.nih.gov/compound/141038952#section=2D-Structure
https://pubchem.ncbi.nlm.nih.gov/compound/141038319#section=2D-Structure
https://pubchem.ncbi.nlm.nih.gov/compound/141038316#section=2D-Structure
https://pubchem.ncbi.nlm.nih.gov/compound/141034902#section=2D-Structure

https://pubchem.ncbi.nlm.nih.gov/compound/141034901#section=2D-Structure
https://pubchem.ncbi.nlm.nih.gov/compound/141031586#section=2D-Structure
https://pubchem.ncbi.nlm.nih.gov/compound/141022641#section=2D-Structure
https://pubchem.ncbi.nlm.nih.gov/compound/141022627#section=2D-Structure
https://pubchem.ncbi.nlm.nih.gov/compound/141022191#section=2D-Structure
https://pubchem.ncbi.nlm.nih.gov/compound/141017182#section=2D-Structure
https://pubchem.ncbi.nlm.nih.gov/compound/141017181#section=2D-Structure
https://pubchem.ncbi.nlm.nih.gov/compound/141014561#section=2D-Structure
https://pubchem.ncbi.nlm.nih.gov/compound/141009095#section=2D-Structure
https://pubchem.ncbi.nlm.nih.gov/compound/141000474#section=2D-Structure
https://pubchem.ncbi.nlm.nih.gov/compound/141000470#section=2D-Structure
https://pubchem.ncbi.nlm.nih.gov/compound/140990136#section=2D-Structure
https://pubchem.ncbi.nlm.nih.gov/compound/140987963#section=2D-Structure
https://pubchem.ncbi.nlm.nih.gov/compound/140987958#section=2D-Structure
https://pubchem.ncbi.nlm.nih.gov/compound/140983934#section=2D-Structure
https://pubchem.ncbi.nlm.nih.gov/compound/140982522#section=2D-Structure
https://pubchem.ncbi.nlm.nih.gov/compound/140967990#section=2D-Structure
https://pubchem.ncbi.nlm.nih.gov/compound/140957710#section=2D-Structure
https://pubchem.ncbi.nlm.nih.gov/compound/140957674#section=2D-Structure
https://pubchem.ncbi.nlm.nih.gov/compound/140956230#section=2D-Structure
https://pubchem.ncbi.nlm.nih.gov/compound/140956142#section=2D-Structure
https://pubchem.ncbi.nlm.nih.gov/compound/140956135#section=2D-Structure
https://pubchem.ncbi.nlm.nih.gov/compound/140956102#section=2D-Structure
https://pubchem.ncbi.nlm.nih.gov/compound/140956079#section=2D-Structure
https://pubchem.ncbi.nlm.nih.gov/compound/140956068#section=2D-Structure
https://pubchem.ncbi.nlm.nih.gov/compound/140956053#section=2D-Structure
https://pubchem.ncbi.nlm.nih.gov/compound/140956043#section=2D-Structure
https://pubchem.ncbi.nlm.nih.gov/compound/140956026#section=2D-Structure
https://pubchem.ncbi.nlm.nih.gov/compound/140956014#section=2D-Structure
https://pubchem.ncbi.nlm.nih.gov/compound/140955991#section=2D-Structure
https://pubchem.ncbi.nlm.nih.gov/compound/140955981#section=2D-Structure
https://pubchem.ncbi.nlm.nih.gov/compound/140955970#section=2D-Structure
https://pubchem.ncbi.nlm.nih.gov/compound/14093436#section=2D-Structure
https://pubchem.ncbi.nlm.nih.gov/compound/140893567#section=2D-Structure
https://pubchem.ncbi.nlm.nih.gov/compound/140863548#section=2D-Structure
https://pubchem.ncbi.nlm.nih.gov/compound/140798183#section=2D-Structure
https://pubchem.ncbi.nlm.nih.gov/compound/140743759#section=2D-Structure
https://pubchem.ncbi.nlm.nih.gov/compound/140734705#section=2D-Structure
https://pubchem.ncbi.nlm.nih.gov/compound/140706579#section=2D-Structure
https://pubchem.ncbi.nlm.nih.gov/compound/140702346#section=2D-Structure
https://pubchem.ncbi.nlm.nih.gov/compound/140684078#section=2D-Structure
https://pubchem.ncbi.nlm.nih.gov/compound/140675513#section=2D-Structure
https://pubchem.ncbi.nlm.nih.gov/compound/140675511#section=2D-Structure
https://pubchem.ncbi.nlm.nih.gov/compound/140675508#section=2D-Structure
https://pubchem.ncbi.nlm.nih.gov/compound/140674052#section=2D-Structure
https://pubchem.ncbi.nlm.nih.gov/compound/140674049#section=2D-Structure
https://pubchem.ncbi.nlm.nih.gov/compound/140656654#section=2D-Structure
https://pubchem.ncbi.nlm.nih.gov/compound/140650316#section=2D-Structure
https://pubchem.ncbi.nlm.nih.gov/compound/140650304#section=2D-Structure
https://pubchem.ncbi.nlm.nih.gov/compound/140634209#section=2D-Structure
https://pubchem.ncbi.nlm.nih.gov/compound/140632263#section=2D-Structure
https://pubchem.ncbi.nlm.nih.gov/compound/140617716#section=2D-Structure
https://pubchem.ncbi.nlm.nih.gov/compound/140617688#section=2D-Structure
https://pubchem.ncbi.nlm.nih.gov/compound/140617687#section=2D-Structure
https://pubchem.ncbi.nlm.nih.gov/compound/140610073#section=2D-Structure

https://pubchem.ncbi.nlm.nih.gov/compound/140589435#section=2D-Structure
https://pubchem.ncbi.nlm.nih.gov/compound/140581326#section=2D-Structure
https://pubchem.ncbi.nlm.nih.gov/compound/140574762#section=2D-Structure
https://pubchem.ncbi.nlm.nih.gov/compound/140572205#section=2D-Structure
https://pubchem.ncbi.nlm.nih.gov/compound/140563309#section=2D-Structure
https://pubchem.ncbi.nlm.nih.gov/compound/140556607#section=2D-Structure
https://pubchem.ncbi.nlm.nih.gov/compound/140548717#section=2D-Structure
https://pubchem.ncbi.nlm.nih.gov/compound/140541146#section=2D-Structure
https://pubchem.ncbi.nlm.nih.gov/compound/140533003#section=2D-Structure
https://pubchem.ncbi.nlm.nih.gov/compound/140520773#section=2D-Structure
https://pubchem.ncbi.nlm.nih.gov/compound/140474679#section=2D-Structure
https://pubchem.ncbi.nlm.nih.gov/compound/140462744#section=2D-Structure
https://pubchem.ncbi.nlm.nih.gov/compound/140461203#section=2D-Structure
https://pubchem.ncbi.nlm.nih.gov/compound/140461202#section=2D-Structure
https://pubchem.ncbi.nlm.nih.gov/compound/140461199#section=2D-Structure
https://pubchem.ncbi.nlm.nih.gov/compound/140461197#section=2D-Structure
https://pubchem.ncbi.nlm.nih.gov/compound/140461196#section=2D-Structure
https://pubchem.ncbi.nlm.nih.gov/compound/140461193#section=2D-Structure
https://pubchem.ncbi.nlm.nih.gov/compound/140461190#section=2D-Structure
https://pubchem.ncbi.nlm.nih.gov/compound/140461189#section=2D-Structure
https://pubchem.ncbi.nlm.nih.gov/compound/140459047#section=2D-Structure
https://pubchem.ncbi.nlm.nih.gov/compound/140435856#section=2D-Structure
https://pubchem.ncbi.nlm.nih.gov/compound/140435345#section=2D-Structure
https://pubchem.ncbi.nlm.nih.gov/compound/140423532#section=2D-Structure
https://pubchem.ncbi.nlm.nih.gov/compound/14042187#section=2D-Structure
https://pubchem.ncbi.nlm.nih.gov/compound/140406#section=2D-Structure
https://pubchem.ncbi.nlm.nih.gov/compound/140398200#section=2D-Structure
https://pubchem.ncbi.nlm.nih.gov/compound/140397887#section=2D-Structure
https://pubchem.ncbi.nlm.nih.gov/compound/140391741#section=2D-Structure
https://pubchem.ncbi.nlm.nih.gov/compound/140380104#section=2D-Structure
https://pubchem.ncbi.nlm.nih.gov/compound/140371048#section=2D-Structure
https://pubchem.ncbi.nlm.nih.gov/compound/140370079#section=2D-Structure
https://pubchem.ncbi.nlm.nih.gov/compound/140367837#section=2D-Structure
https://pubchem.ncbi.nlm.nih.gov/compound/140360188#section=2D-Structure
https://pubchem.ncbi.nlm.nih.gov/compound/1403571#section=2D-Structure
https://pubchem.ncbi.nlm.nih.gov/compound/140355024#section=2D-Structure
https://pubchem.ncbi.nlm.nih.gov/compound/140354538#section=2D-Structure
https://pubchem.ncbi.nlm.nih.gov/compound/140346903#section=2D-Structure
https://pubchem.ncbi.nlm.nih.gov/compound/140346901#section=2D-Structure
https://pubchem.ncbi.nlm.nih.gov/compound/140341802#section=2D-Structure
https://pubchem.ncbi.nlm.nih.gov/compound/140339700#section=2D-Structure
https://pubchem.ncbi.nlm.nih.gov/compound/140334536#section=2D-Structure
https://pubchem.ncbi.nlm.nih.gov/compound/140322395#section=2D-Structure
https://pubchem.ncbi.nlm.nih.gov/compound/140308818#section=2D-Structure
https://pubchem.ncbi.nlm.nih.gov/compound/140308120#section=2D-Structure
https://pubchem.ncbi.nlm.nih.gov/compound/140296646#section=2D-Structure
https://pubchem.ncbi.nlm.nih.gov/compound/140292946#section=2D-Structure
https://pubchem.ncbi.nlm.nih.gov/compound/14028896#section=2D-Structure
https://pubchem.ncbi.nlm.nih.gov/compound/140255810#section=2D-Structure
https://pubchem.ncbi.nlm.nih.gov/compound/140255805#section=2D-Structure
https://pubchem.ncbi.nlm.nih.gov/compound/140254793#section=2D-Structure
https://pubchem.ncbi.nlm.nih.gov/compound/140251889#section=2D-Structure
https://pubchem.ncbi.nlm.nih.gov/compound/140251127#section=2D-Structure
https://pubchem.ncbi.nlm.nih.gov/compound/140249140#section=2D-Structure
https://pubchem.ncbi.nlm.nih.gov/compound/140247956#section=2D-Structure

https://pubchem.ncbi.nlm.nih.gov/compound/140246959#section=2D-Structure
https://pubchem.ncbi.nlm.nih.gov/compound/140242905#section=2D-Structure
https://pubchem.ncbi.nlm.nih.gov/compound/140242384#section=2D-Structure
https://pubchem.ncbi.nlm.nih.gov/compound/140241063#section=2D-Structure
https://pubchem.ncbi.nlm.nih.gov/compound/140240870#section=2D-Structure
https://pubchem.ncbi.nlm.nih.gov/compound/140226844#section=2D-Structure
https://pubchem.ncbi.nlm.nih.gov/compound/140225952#section=2D-Structure
https://pubchem.ncbi.nlm.nih.gov/compound/140225912#section=2D-Structure
https://pubchem.ncbi.nlm.nih.gov/compound/140225850#section=2D-Structure
https://pubchem.ncbi.nlm.nih.gov/compound/140225764#section=2D-Structure
https://pubchem.ncbi.nlm.nih.gov/compound/140225506#section=2D-Structure
https://pubchem.ncbi.nlm.nih.gov/compound/140225443#section=2D-Structure
https://pubchem.ncbi.nlm.nih.gov/compound/140217077#section=2D-Structure
https://pubchem.ncbi.nlm.nih.gov/compound/140211512#section=2D-Structure
https://pubchem.ncbi.nlm.nih.gov/compound/140208250#section=2D-Structure
https://pubchem.ncbi.nlm.nih.gov/compound/140199333#section=2D-Structure
https://pubchem.ncbi.nlm.nih.gov/compound/14017876#section=2D-Structure
https://pubchem.ncbi.nlm.nih.gov/compound/140173006#section=2D-Structure
https://pubchem.ncbi.nlm.nih.gov/compound/140167758#section=2D-Structure
https://pubchem.ncbi.nlm.nih.gov/compound/140167743#section=2D-Structure
https://pubchem.ncbi.nlm.nih.gov/compound/140167731#section=2D-Structure
https://pubchem.ncbi.nlm.nih.gov/compound/140167722#section=2D-Structure
https://pubchem.ncbi.nlm.nih.gov/compound/140167721#section=2D-Structure
https://pubchem.ncbi.nlm.nih.gov/compound/140167719#section=2D-Structure
https://pubchem.ncbi.nlm.nih.gov/compound/140167711#section=2D-Structure
https://pubchem.ncbi.nlm.nih.gov/compound/140141661#section=2D-Structure
https://pubchem.ncbi.nlm.nih.gov/compound/1401371#section=2D-Structure
https://pubchem.ncbi.nlm.nih.gov/compound/140132069#section=2D-Structure
https://pubchem.ncbi.nlm.nih.gov/compound/140119322#section=2D-Structure
https://pubchem.ncbi.nlm.nih.gov/compound/140100268#section=2D-Structure
https://pubchem.ncbi.nlm.nih.gov/compound/14009568#section=2D-Structure
https://pubchem.ncbi.nlm.nih.gov/compound/14008926#section=2D-Structure
https://pubchem.ncbi.nlm.nih.gov/compound/140078547#section=2D-Structure
https://pubchem.ncbi.nlm.nih.gov/compound/140076837#section=2D-Structure
https://pubchem.ncbi.nlm.nih.gov/compound/140075248#section=2D-Structure
https://pubchem.ncbi.nlm.nih.gov/compound/140075237#section=2D-Structure
https://pubchem.ncbi.nlm.nih.gov/compound/140075219#section=2D-Structure
https://pubchem.ncbi.nlm.nih.gov/compound/140075199#section=2D-Structure
https://pubchem.ncbi.nlm.nih.gov/compound/140075166#section=2D-Structure
https://pubchem.ncbi.nlm.nih.gov/compound/140075165#section=2D-Structure
https://pubchem.ncbi.nlm.nih.gov/compound/140075155#section=2D-Structure
https://pubchem.ncbi.nlm.nih.gov/compound/140075139#section=2D-Structure
https://pubchem.ncbi.nlm.nih.gov/compound/140075137#section=2D-Structure
https://pubchem.ncbi.nlm.nih.gov/compound/140075117#section=2D-Structure
https://pubchem.ncbi.nlm.nih.gov/compound/140075115#section=2D-Structure
https://pubchem.ncbi.nlm.nih.gov/compound/140075100#section=2D-Structure
https://pubchem.ncbi.nlm.nih.gov/compound/140075074#section=2D-Structure
https://pubchem.ncbi.nlm.nih.gov/compound/140075063#section=2D-Structure
https://pubchem.ncbi.nlm.nih.gov/compound/140075054#section=2D-Structure
https://pubchem.ncbi.nlm.nih.gov/compound/140075052#section=2D-Structure
https://pubchem.ncbi.nlm.nih.gov/compound/140075039#section=2D-Structure
https://pubchem.ncbi.nlm.nih.gov/compound/140075031#section=2D-Structure
https://pubchem.ncbi.nlm.nih.gov/compound/140075028#section=2D-Structure
https://pubchem.ncbi.nlm.nih.gov/compound/140075017#section=2D-Structure
https://pubchem.ncbi.nlm.nih.gov/compound/140074983#section=2D-Structure

https://pubchem.ncbi.nlm.nih.gov/compound/140074969#section=2D-Structure
https://pubchem.ncbi.nlm.nih.gov/compound/140074947#section=2D-Structure
https://pubchem.ncbi.nlm.nih.gov/compound/140074920#section=2D-Structure
https://pubchem.ncbi.nlm.nih.gov/compound/140074894#section=2D-Structure
https://pubchem.ncbi.nlm.nih.gov/compound/140074887#section=2D-Structure
https://pubchem.ncbi.nlm.nih.gov/compound/140074877#section=2D-Structure
https://pubchem.ncbi.nlm.nih.gov/compound/140074862#section=2D-Structure
https://pubchem.ncbi.nlm.nih.gov/compound/140074861#section=2D-Structure
https://pubchem.ncbi.nlm.nih.gov/compound/140074835#section=2D-Structure
https://pubchem.ncbi.nlm.nih.gov/compound/140074827#section=2D-Structure
https://pubchem.ncbi.nlm.nih.gov/compound/140074821#section=2D-Structure
https://pubchem.ncbi.nlm.nih.gov/compound/140074811#section=2D-Structure
https://pubchem.ncbi.nlm.nih.gov/compound/140059930#section=2D-Structure
https://pubchem.ncbi.nlm.nih.gov/compound/140052997#section=2D-Structure
https://pubchem.ncbi.nlm.nih.gov/compound/140033820#section=2D-Structure
https://pubchem.ncbi.nlm.nih.gov/compound/140030935#section=2D-Structure
https://pubchem.ncbi.nlm.nih.gov/compound/140018976#section=2D-Structure
https://pubchem.ncbi.nlm.nih.gov/compound/140011885#section=2D-Structure
https://pubchem.ncbi.nlm.nih.gov/compound/140011884#section=2D-Structure
https://pubchem.ncbi.nlm.nih.gov/compound/139987014#section=2D-Structure
https://pubchem.ncbi.nlm.nih.gov/compound/139949733#section=2D-Structure
https://pubchem.ncbi.nlm.nih.gov/compound/139949686#section=2D-Structure
https://pubchem.ncbi.nlm.nih.gov/compound/139933856#section=2D-Structure
https://pubchem.ncbi.nlm.nih.gov/compound/139931367#section=2D-Structure
https://pubchem.ncbi.nlm.nih.gov/compound/139924014#section=2D-Structure
https://pubchem.ncbi.nlm.nih.gov/compound/139922534#section=2D-Structure
https://pubchem.ncbi.nlm.nih.gov/compound/139919525#section=2D-Structure
https://pubchem.ncbi.nlm.nih.gov/compound/139918489#section=2D-Structure
https://pubchem.ncbi.nlm.nih.gov/compound/139918488#section=2D-Structure
https://pubchem.ncbi.nlm.nih.gov/compound/139916564#section=2D-Structure
https://pubchem.ncbi.nlm.nih.gov/compound/139908919#section=2D-Structure
https://pubchem.ncbi.nlm.nih.gov/compound/139904221#section=2D-Structure
https://pubchem.ncbi.nlm.nih.gov/compound/139887420#section=2D-Structure
https://pubchem.ncbi.nlm.nih.gov/compound/139885892#section=2D-Structure
https://pubchem.ncbi.nlm.nih.gov/compound/139840838#section=2D-Structure
https://pubchem.ncbi.nlm.nih.gov/compound/139834817#section=2D-Structure
https://pubchem.ncbi.nlm.nih.gov/compound/139823527#section=2D-Structure
https://pubchem.ncbi.nlm.nih.gov/compound/139816942#section=2D-Structure
https://pubchem.ncbi.nlm.nih.gov/compound/139797674#section=2D-Structure
https://pubchem.ncbi.nlm.nih.gov/compound/139796250#section=2D-Structure
https://pubchem.ncbi.nlm.nih.gov/compound/139777268#section=2D-Structure
https://pubchem.ncbi.nlm.nih.gov/compound/139767824#section=2D-Structure
https://pubchem.ncbi.nlm.nih.gov/compound/139749081#section=2D-Structure
https://pubchem.ncbi.nlm.nih.gov/compound/139749079#section=2D-Structure
https://pubchem.ncbi.nlm.nih.gov/compound/139747087#section=2D-Structure
https://pubchem.ncbi.nlm.nih.gov/compound/13973935#section=2D-Structure
https://pubchem.ncbi.nlm.nih.gov/compound/139730743#section=2D-Structure
https://pubchem.ncbi.nlm.nih.gov/compound/13972344#section=2D-Structure
https://pubchem.ncbi.nlm.nih.gov/compound/13972340#section=2D-Structure
https://pubchem.ncbi.nlm.nih.gov/compound/139710411#section=2D-Structure
https://pubchem.ncbi.nlm.nih.gov/compound/139694853#section=2D-Structure
https://pubchem.ncbi.nlm.nih.gov/compound/139680668#section=2D-Structure
https://pubchem.ncbi.nlm.nih.gov/compound/139667381#section=2D-Structure
https://pubchem.ncbi.nlm.nih.gov/compound/139655609#section=2D-Structure
https://pubchem.ncbi.nlm.nih.gov/compound/139647442#section=2D-Structure

https://pubchem.ncbi.nlm.nih.gov/compound/139636060#section=2D-Structure
https://pubchem.ncbi.nlm.nih.gov/compound/139636052#section=2D-Structure
https://pubchem.ncbi.nlm.nih.gov/compound/139636051#section=2D-Structure
https://pubchem.ncbi.nlm.nih.gov/compound/139626401#section=2D-Structure
https://pubchem.ncbi.nlm.nih.gov/compound/139626362#section=2D-Structure
https://pubchem.ncbi.nlm.nih.gov/compound/139626361#section=2D-Structure
https://pubchem.ncbi.nlm.nih.gov/compound/139601143#section=2D-Structure
https://pubchem.ncbi.nlm.nih.gov/compound/139601138#section=2D-Structure
https://pubchem.ncbi.nlm.nih.gov/compound/139592971#section=2D-Structure
https://pubchem.ncbi.nlm.nih.gov/compound/139559894#section=2D-Structure
https://pubchem.ncbi.nlm.nih.gov/compound/139524878#section=2D-Structure
https://pubchem.ncbi.nlm.nih.gov/compound/139516129#section=2D-Structure
https://pubchem.ncbi.nlm.nih.gov/compound/139508671#section=2D-Structure
https://pubchem.ncbi.nlm.nih.gov/compound/13949790#section=2D-Structure
https://pubchem.ncbi.nlm.nih.gov/compound/139489385#section=2D-Structure
https://pubchem.ncbi.nlm.nih.gov/compound/139466388#section=2D-Structure
https://pubchem.ncbi.nlm.nih.gov/compound/139462112#section=2D-Structure
https://pubchem.ncbi.nlm.nih.gov/compound/13943966#section=2D-Structure
https://pubchem.ncbi.nlm.nih.gov/compound/139436429#section=2D-Structure
https://pubchem.ncbi.nlm.nih.gov/compound/139436428#section=2D-Structure
https://pubchem.ncbi.nlm.nih.gov/compound/139436392#section=2D-Structure
https://pubchem.ncbi.nlm.nih.gov/compound/139436390#section=2D-Structure
https://pubchem.ncbi.nlm.nih.gov/compound/139436389#section=2D-Structure
https://pubchem.ncbi.nlm.nih.gov/compound/139436359#section=2D-Structure
https://pubchem.ncbi.nlm.nih.gov/compound/139436336#section=2D-Structure
https://pubchem.ncbi.nlm.nih.gov/compound/139436328#section=2D-Structure
https://pubchem.ncbi.nlm.nih.gov/compound/139436035#section=2D-Structure
https://pubchem.ncbi.nlm.nih.gov/compound/139436034#section=2D-Structure
https://pubchem.ncbi.nlm.nih.gov/compound/139436026#section=2D-Structure
https://pubchem.ncbi.nlm.nih.gov/compound/139436025#section=2D-Structure
https://pubchem.ncbi.nlm.nih.gov/compound/139436024#section=2D-Structure
https://pubchem.ncbi.nlm.nih.gov/compound/139436021#section=2D-Structure
https://pubchem.ncbi.nlm.nih.gov/compound/139436019#section=2D-Structure
https://pubchem.ncbi.nlm.nih.gov/compound/139436018#section=2D-Structure
https://pubchem.ncbi.nlm.nih.gov/compound/139436016#section=2D-Structure
https://pubchem.ncbi.nlm.nih.gov/compound/139436014#section=2D-Structure
https://pubchem.ncbi.nlm.nih.gov/compound/139436009#section=2D-Structure
https://pubchem.ncbi.nlm.nih.gov/compound/139436007#section=2D-Structure
https://pubchem.ncbi.nlm.nih.gov/compound/139436005#section=2D-Structure
https://pubchem.ncbi.nlm.nih.gov/compound/139435985#section=2D-Structure
https://pubchem.ncbi.nlm.nih.gov/compound/139435976#section=2D-Structure
https://pubchem.ncbi.nlm.nih.gov/compound/139435973#section=2D-Structure
https://pubchem.ncbi.nlm.nih.gov/compound/139435969#section=2D-Structure
https://pubchem.ncbi.nlm.nih.gov/compound/139435968#section=2D-Structure
https://pubchem.ncbi.nlm.nih.gov/compound/139435966#section=2D-Structure
https://pubchem.ncbi.nlm.nih.gov/compound/139435965#section=2D-Structure
https://pubchem.ncbi.nlm.nih.gov/compound/139435892#section=2D-Structure
https://pubchem.ncbi.nlm.nih.gov/compound/139435861#section=2D-Structure
https://pubchem.ncbi.nlm.nih.gov/compound/139435859#section=2D-Structure
https://pubchem.ncbi.nlm.nih.gov/compound/139435857#section=2D-Structure
https://pubchem.ncbi.nlm.nih.gov/compound/139435856#section=2D-Structure
https://pubchem.ncbi.nlm.nih.gov/compound/139385089#section=2D-Structure
https://pubchem.ncbi.nlm.nih.gov/compound/139345011#section=2D-Structure
https://pubchem.ncbi.nlm.nih.gov/compound/139323648#section=2D-Structure
https://pubchem.ncbi.nlm.nih.gov/compound/13928168#section=2D-Structure

https://pubchem.ncbi.nlm.nih.gov/compound/13925849#section=2D-Structure
https://pubchem.ncbi.nlm.nih.gov/compound/13925832#section=2D-Structure
https://pubchem.ncbi.nlm.nih.gov/compound/13925831#section=2D-Structure
https://pubchem.ncbi.nlm.nih.gov/compound/139257662#section=2D-Structure
https://pubchem.ncbi.nlm.nih.gov/compound/139246388#section=2D-Structure
https://pubchem.ncbi.nlm.nih.gov/compound/139244996#section=2D-Structure
https://pubchem.ncbi.nlm.nih.gov/compound/139243140#section=2D-Structure
https://pubchem.ncbi.nlm.nih.gov/compound/139232805#section=2D-Structure
https://pubchem.ncbi.nlm.nih.gov/compound/139226683#section=2D-Structure
https://pubchem.ncbi.nlm.nih.gov/compound/139214555#section=2D-Structure
https://pubchem.ncbi.nlm.nih.gov/compound/139214030#section=2D-Structure
https://pubchem.ncbi.nlm.nih.gov/compound/139207860#section=2D-Structure
https://pubchem.ncbi.nlm.nih.gov/compound/139182206#section=2D-Structure
https://pubchem.ncbi.nlm.nih.gov/compound/139174620#section=2D-Structure
https://pubchem.ncbi.nlm.nih.gov/compound/139143#section=2D-Structure
https://pubchem.ncbi.nlm.nih.gov/compound/139126638#section=2D-Structure
https://pubchem.ncbi.nlm.nih.gov/compound/139072756#section=2D-Structure
https://pubchem.ncbi.nlm.nih.gov/compound/139065902#section=2D-Structure
https://pubchem.ncbi.nlm.nih.gov/compound/13906556#section=2D-Structure
https://pubchem.ncbi.nlm.nih.gov/compound/139053689#section=2D-Structure
https://pubchem.ncbi.nlm.nih.gov/compound/13905332#section=2D-Structure
https://pubchem.ncbi.nlm.nih.gov/compound/13905331#section=2D-Structure
https://pubchem.ncbi.nlm.nih.gov/compound/13905324#section=2D-Structure
https://pubchem.ncbi.nlm.nih.gov/compound/139038400#section=2D-Structure
https://pubchem.ncbi.nlm.nih.gov/compound/139030674#section=2D-Structure
https://pubchem.ncbi.nlm.nih.gov/compound/13902159#section=2D-Structure
https://pubchem.ncbi.nlm.nih.gov/compound/138963115#section=2D-Structure
https://pubchem.ncbi.nlm.nih.gov/compound/138857491#section=2D-Structure
https://pubchem.ncbi.nlm.nih.gov/compound/138756974#section=2D-Structure
https://pubchem.ncbi.nlm.nih.gov/compound/138756939#section=2D-Structure
https://pubchem.ncbi.nlm.nih.gov/compound/138756904#section=2D-Structure
https://pubchem.ncbi.nlm.nih.gov/compound/138753440#section=2D-Structure
https://pubchem.ncbi.nlm.nih.gov/compound/138704#section=2D-Structure
https://pubchem.ncbi.nlm.nih.gov/compound/138616462#section=2D-Structure
https://pubchem.ncbi.nlm.nih.gov/compound/138616414#section=2D-Structure
https://pubchem.ncbi.nlm.nih.gov/compound/138616363#section=2D-Structure
https://pubchem.ncbi.nlm.nih.gov/compound/138606344#section=2D-Structure
https://pubchem.ncbi.nlm.nih.gov/compound/138559320#section=2D-Structure
https://pubchem.ncbi.nlm.nih.gov/compound/138527764#section=2D-Structure
https://pubchem.ncbi.nlm.nih.gov/compound/138515691#section=2D-Structure
https://pubchem.ncbi.nlm.nih.gov/compound/138512272#section=2D-Structure
https://pubchem.ncbi.nlm.nih.gov/compound/13847995#section=2D-Structure
https://pubchem.ncbi.nlm.nih.gov/compound/138457563#section=2D-Structure
https://pubchem.ncbi.nlm.nih.gov/compound/138454453#section=2D-Structure
https://pubchem.ncbi.nlm.nih.gov/compound/138399111#section=2D-Structure
https://pubchem.ncbi.nlm.nih.gov/compound/13837860#section=2D-Structure
https://pubchem.ncbi.nlm.nih.gov/compound/13837859#section=2D-Structure
https://pubchem.ncbi.nlm.nih.gov/compound/13837856#section=2D-Structure
https://pubchem.ncbi.nlm.nih.gov/compound/13837854#section=2D-Structure
https://pubchem.ncbi.nlm.nih.gov/compound/13837849#section=2D-Structure
https://pubchem.ncbi.nlm.nih.gov/compound/13835032#section=2D-Structure
https://pubchem.ncbi.nlm.nih.gov/compound/13814388#section=2D-Structure
https://pubchem.ncbi.nlm.nih.gov/compound/138115531#section=2D-Structure
https://pubchem.ncbi.nlm.nih.gov/compound/138115529#section=2D-Structure
https://pubchem.ncbi.nlm.nih.gov/compound/13809326#section=2D-Structure

https://pubchem.ncbi.nlm.nih.gov/compound/13809325#section=2D-Structure
https://pubchem.ncbi.nlm.nih.gov/compound/13808971#section=2D-Structure
https://pubchem.ncbi.nlm.nih.gov/compound/13808966#section=2D-Structure
https://pubchem.ncbi.nlm.nih.gov/compound/137951415#section=2D-Structure
https://pubchem.ncbi.nlm.nih.gov/compound/13776238#section=2D-Structure
https://pubchem.ncbi.nlm.nih.gov/compound/13766980#section=2D-Structure
https://pubchem.ncbi.nlm.nih.gov/compound/13766965#section=2D-Structure
https://pubchem.ncbi.nlm.nih.gov/compound/13766961#section=2D-Structure
https://pubchem.ncbi.nlm.nih.gov/compound/13766957#section=2D-Structure
https://pubchem.ncbi.nlm.nih.gov/compound/13766955#section=2D-Structure
https://pubchem.ncbi.nlm.nih.gov/compound/13766947#section=2D-Structure
https://pubchem.ncbi.nlm.nih.gov/compound/13766946#section=2D-Structure
https://pubchem.ncbi.nlm.nih.gov/compound/13766945#section=2D-Structure
https://pubchem.ncbi.nlm.nih.gov/compound/13766942#section=2D-Structure
https://pubchem.ncbi.nlm.nih.gov/compound/13766936#section=2D-Structure
https://pubchem.ncbi.nlm.nih.gov/compound/13766933#section=2D-Structure
https://pubchem.ncbi.nlm.nih.gov/compound/13758705#section=2D-Structure
https://pubchem.ncbi.nlm.nih.gov/compound/137553231#section=2D-Structure
https://pubchem.ncbi.nlm.nih.gov/compound/137552863#section=2D-Structure
https://pubchem.ncbi.nlm.nih.gov/compound/137552858#section=2D-Structure
https://pubchem.ncbi.nlm.nih.gov/compound/137537919#section=2D-Structure
https://pubchem.ncbi.nlm.nih.gov/compound/137537918#section=2D-Structure
https://pubchem.ncbi.nlm.nih.gov/compound/137537916#section=2D-Structure
https://pubchem.ncbi.nlm.nih.gov/compound/137537914#section=2D-Structure
https://pubchem.ncbi.nlm.nih.gov/compound/137537909#section=2D-Structure
https://pubchem.ncbi.nlm.nih.gov/compound/137530240#section=2D-Structure
https://pubchem.ncbi.nlm.nih.gov/compound/137493161#section=2D-Structure
https://pubchem.ncbi.nlm.nih.gov/compound/137492954#section=2D-Structure
https://pubchem.ncbi.nlm.nih.gov/compound/137492604#section=2D-Structure
https://pubchem.ncbi.nlm.nih.gov/compound/137492603#section=2D-Structure
https://pubchem.ncbi.nlm.nih.gov/compound/137492602#section=2D-Structure
https://pubchem.ncbi.nlm.nih.gov/compound/137492601#section=2D-Structure
https://pubchem.ncbi.nlm.nih.gov/compound/137492575#section=2D-Structure
https://pubchem.ncbi.nlm.nih.gov/compound/137470384#section=2D-Structure
https://pubchem.ncbi.nlm.nih.gov/compound/137470382#section=2D-Structure
https://pubchem.ncbi.nlm.nih.gov/compound/137467611#section=2D-Structure
https://pubchem.ncbi.nlm.nih.gov/compound/137459041#section=2D-Structure
https://pubchem.ncbi.nlm.nih.gov/compound/137459009#section=2D-Structure
https://pubchem.ncbi.nlm.nih.gov/compound/137458815#section=2D-Structure
https://pubchem.ncbi.nlm.nih.gov/compound/137458814#section=2D-Structure
https://pubchem.ncbi.nlm.nih.gov/compound/137458813#section=2D-Structure
https://pubchem.ncbi.nlm.nih.gov/compound/137458812#section=2D-Structure
https://pubchem.ncbi.nlm.nih.gov/compound/137441225#section=2D-Structure
https://pubchem.ncbi.nlm.nih.gov/compound/137421938#section=2D-Structure
https://pubchem.ncbi.nlm.nih.gov/compound/137402781#section=2D-Structure
https://pubchem.ncbi.nlm.nih.gov/compound/137386521#section=2D-Structure
https://pubchem.ncbi.nlm.nih.gov/compound/137346185#section=2D-Structure
https://pubchem.ncbi.nlm.nih.gov/compound/13734043#section=2D-Structure
https://pubchem.ncbi.nlm.nih.gov/compound/13734030#section=2D-Structure
https://pubchem.ncbi.nlm.nih.gov/compound/137321889#section=2D-Structure
https://pubchem.ncbi.nlm.nih.gov/compound/137321508#section=2D-Structure
https://pubchem.ncbi.nlm.nih.gov/compound/137321331#section=2D-Structure
https://pubchem.ncbi.nlm.nih.gov/compound/137316003#section=2D-Structure
https://pubchem.ncbi.nlm.nih.gov/compound/137309029#section=2D-Structure
https://pubchem.ncbi.nlm.nih.gov/compound/137295458#section=2D-Structure

https://pubchem.ncbi.nlm.nih.gov/compound/137295457#section=2D-Structure
https://pubchem.ncbi.nlm.nih.gov/compound/137288272#section=2D-Structure
https://pubchem.ncbi.nlm.nih.gov/compound/137288268#section=2D-Structure
https://pubchem.ncbi.nlm.nih.gov/compound/137288265#section=2D-Structure
https://pubchem.ncbi.nlm.nih.gov/compound/137287576#section=2D-Structure
https://pubchem.ncbi.nlm.nih.gov/compound/137285014#section=2D-Structure
https://pubchem.ncbi.nlm.nih.gov/compound/137281662#section=2D-Structure
https://pubchem.ncbi.nlm.nih.gov/compound/137281646#section=2D-Structure
https://pubchem.ncbi.nlm.nih.gov/compound/137281639#section=2D-Structure
https://pubchem.ncbi.nlm.nih.gov/compound/137252148#section=2D-Structure
https://pubchem.ncbi.nlm.nih.gov/compound/137235469#section=2D-Structure
https://pubchem.ncbi.nlm.nih.gov/compound/137225803#section=2D-Structure
https://pubchem.ncbi.nlm.nih.gov/compound/137225245#section=2D-Structure
https://pubchem.ncbi.nlm.nih.gov/compound/137225199#section=2D-Structure
https://pubchem.ncbi.nlm.nih.gov/compound/137222993#section=2D-Structure
https://pubchem.ncbi.nlm.nih.gov/compound/137222895#section=2D-Structure
https://pubchem.ncbi.nlm.nih.gov/compound/137222894#section=2D-Structure
https://pubchem.ncbi.nlm.nih.gov/compound/137218422#section=2D-Structure
https://pubchem.ncbi.nlm.nih.gov/compound/137213827#section=2D-Structure
https://pubchem.ncbi.nlm.nih.gov/compound/137207583#section=2D-Structure
https://pubchem.ncbi.nlm.nih.gov/compound/137207567#section=2D-Structure
https://pubchem.ncbi.nlm.nih.gov/compound/137207502#section=2D-Structure
https://pubchem.ncbi.nlm.nih.gov/compound/137201249#section=2D-Structure
https://pubchem.ncbi.nlm.nih.gov/compound/137192635#section=2D-Structure
https://pubchem.ncbi.nlm.nih.gov/compound/137190266#section=2D-Structure
https://pubchem.ncbi.nlm.nih.gov/compound/137190265#section=2D-Structure
https://pubchem.ncbi.nlm.nih.gov/compound/137187100#section=2D-Structure
https://pubchem.ncbi.nlm.nih.gov/compound/137184005#section=2D-Structure
https://pubchem.ncbi.nlm.nih.gov/compound/137183769#section=2D-Structure
https://pubchem.ncbi.nlm.nih.gov/compound/137183205#section=2D-Structure
https://pubchem.ncbi.nlm.nih.gov/compound/137179382#section=2D-Structure
https://pubchem.ncbi.nlm.nih.gov/compound/137177319#section=2D-Structure
https://pubchem.ncbi.nlm.nih.gov/compound/137177312#section=2D-Structure
https://pubchem.ncbi.nlm.nih.gov/compound/137175931#section=2D-Structure
https://pubchem.ncbi.nlm.nih.gov/compound/137175858#section=2D-Structure
https://pubchem.ncbi.nlm.nih.gov/compound/137169230#section=2D-Structure
https://pubchem.ncbi.nlm.nih.gov/compound/137158302#section=2D-Structure
https://pubchem.ncbi.nlm.nih.gov/compound/137152788#section=2D-Structure
https://pubchem.ncbi.nlm.nih.gov/compound/137132073#section=2D-Structure
https://pubchem.ncbi.nlm.nih.gov/compound/137131935#section=2D-Structure
https://pubchem.ncbi.nlm.nih.gov/compound/137126089#section=2D-Structure
https://pubchem.ncbi.nlm.nih.gov/compound/137126088#section=2D-Structure
https://pubchem.ncbi.nlm.nih.gov/compound/137123860#section=2D-Structure
https://pubchem.ncbi.nlm.nih.gov/compound/137121844#section=2D-Structure
https://pubchem.ncbi.nlm.nih.gov/compound/137118607#section=2D-Structure
https://pubchem.ncbi.nlm.nih.gov/compound/137104143#section=2D-Structure
https://pubchem.ncbi.nlm.nih.gov/compound/137103749#section=2D-Structure
https://pubchem.ncbi.nlm.nih.gov/compound/137103497#section=2D-Structure
https://pubchem.ncbi.nlm.nih.gov/compound/137101019#section=2D-Structure
https://pubchem.ncbi.nlm.nih.gov/compound/137100456#section=2D-Structure
https://pubchem.ncbi.nlm.nih.gov/compound/137100251#section=2D-Structure
https://pubchem.ncbi.nlm.nih.gov/compound/137084077#section=2D-Structure
https://pubchem.ncbi.nlm.nih.gov/compound/137083172#section=2D-Structure
https://pubchem.ncbi.nlm.nih.gov/compound/137081615#section=2D-Structure
https://pubchem.ncbi.nlm.nih.gov/compound/137035286#section=2D-Structure

https://pubchem.ncbi.nlm.nih.gov/compound/137007122#section=2D-Structure
https://pubchem.ncbi.nlm.nih.gov/compound/136999213#section=2D-Structure
https://pubchem.ncbi.nlm.nih.gov/compound/136983673#section=2D-Structure
https://pubchem.ncbi.nlm.nih.gov/compound/136970216#section=2D-Structure
https://pubchem.ncbi.nlm.nih.gov/compound/136963268#section=2D-Structure
https://pubchem.ncbi.nlm.nih.gov/compound/136960289#section=2D-Structure
https://pubchem.ncbi.nlm.nih.gov/compound/136951738#section=2D-Structure
https://pubchem.ncbi.nlm.nih.gov/compound/136951317#section=2D-Structure
https://pubchem.ncbi.nlm.nih.gov/compound/136948129#section=2D-Structure
https://pubchem.ncbi.nlm.nih.gov/compound/136947754#section=2D-Structure
https://pubchem.ncbi.nlm.nih.gov/compound/136901458#section=2D-Structure
https://pubchem.ncbi.nlm.nih.gov/compound/136883583#section=2D-Structure
https://pubchem.ncbi.nlm.nih.gov/compound/136877341#section=2D-Structure
https://pubchem.ncbi.nlm.nih.gov/compound/136862626#section=2D-Structure
https://pubchem.ncbi.nlm.nih.gov/compound/136861122#section=2D-Structure
https://pubchem.ncbi.nlm.nih.gov/compound/13685248#section=2D-Structure
https://pubchem.ncbi.nlm.nih.gov/compound/136777062#section=2D-Structure
https://pubchem.ncbi.nlm.nih.gov/compound/136759131#section=2D-Structure
https://pubchem.ncbi.nlm.nih.gov/compound/136749867#section=2D-Structure
https://pubchem.ncbi.nlm.nih.gov/compound/136745192#section=2D-Structure
https://pubchem.ncbi.nlm.nih.gov/compound/136740030#section=2D-Structure
https://pubchem.ncbi.nlm.nih.gov/compound/136739967#section=2D-Structure
https://pubchem.ncbi.nlm.nih.gov/compound/136738486#section=2D-Structure
https://pubchem.ncbi.nlm.nih.gov/compound/136738277#section=2D-Structure
https://pubchem.ncbi.nlm.nih.gov/compound/136731901#section=2D-Structure
https://pubchem.ncbi.nlm.nih.gov/compound/136726720#section=2D-Structure
https://pubchem.ncbi.nlm.nih.gov/compound/136717587#section=2D-Structure
https://pubchem.ncbi.nlm.nih.gov/compound/136715198#section=2D-Structure
https://pubchem.ncbi.nlm.nih.gov/compound/136711248#section=2D-Structure
https://pubchem.ncbi.nlm.nih.gov/compound/136711245#section=2D-Structure
https://pubchem.ncbi.nlm.nih.gov/compound/136705121#section=2D-Structure
https://pubchem.ncbi.nlm.nih.gov/compound/136704350#section=2D-Structure
https://pubchem.ncbi.nlm.nih.gov/compound/136702816#section=2D-Structure
https://pubchem.ncbi.nlm.nih.gov/compound/136700374#section=2D-Structure
https://pubchem.ncbi.nlm.nih.gov/compound/136700373#section=2D-Structure
https://pubchem.ncbi.nlm.nih.gov/compound/136700372#section=2D-Structure
https://pubchem.ncbi.nlm.nih.gov/compound/136700371#section=2D-Structure
https://pubchem.ncbi.nlm.nih.gov/compound/136697#section=2D-Structure
https://pubchem.ncbi.nlm.nih.gov/compound/136688213#section=2D-Structure
https://pubchem.ncbi.nlm.nih.gov/compound/13668817#section=2D-Structure
https://pubchem.ncbi.nlm.nih.gov/compound/13668776#section=2D-Structure
https://pubchem.ncbi.nlm.nih.gov/compound/136683367#section=2D-Structure
https://pubchem.ncbi.nlm.nih.gov/compound/136672277#section=2D-Structure
https://pubchem.ncbi.nlm.nih.gov/compound/136660149#section=2D-Structure
https://pubchem.ncbi.nlm.nih.gov/compound/136659864#section=2D-Structure
https://pubchem.ncbi.nlm.nih.gov/compound/136657724#section=2D-Structure
https://pubchem.ncbi.nlm.nih.gov/compound/136657054#section=2D-Structure
https://pubchem.ncbi.nlm.nih.gov/compound/136656429#section=2D-Structure
https://pubchem.ncbi.nlm.nih.gov/compound/136651179#section=2D-Structure
https://pubchem.ncbi.nlm.nih.gov/compound/136650745#section=2D-Structure
https://pubchem.ncbi.nlm.nih.gov/compound/136650180#section=2D-Structure
https://pubchem.ncbi.nlm.nih.gov/compound/136644374#section=2D-Structure
https://pubchem.ncbi.nlm.nih.gov/compound/136640538#section=2D-Structure
https://pubchem.ncbi.nlm.nih.gov/compound/136639830#section=2D-Structure
https://pubchem.ncbi.nlm.nih.gov/compound/136635010#section=2D-Structure

https://pubchem.ncbi.nlm.nih.gov/compound/136632228#section=2D-Structure
https://pubchem.ncbi.nlm.nih.gov/compound/136629949#section=2D-Structure
https://pubchem.ncbi.nlm.nih.gov/compound/136629022#section=2D-Structure
https://pubchem.ncbi.nlm.nih.gov/compound/136626807#section=2D-Structure
https://pubchem.ncbi.nlm.nih.gov/compound/136623083#section=2D-Structure
https://pubchem.ncbi.nlm.nih.gov/compound/136622606#section=2D-Structure
https://pubchem.ncbi.nlm.nih.gov/compound/136621231#section=2D-Structure
https://pubchem.ncbi.nlm.nih.gov/compound/136613611#section=2D-Structure
https://pubchem.ncbi.nlm.nih.gov/compound/136611187#section=2D-Structure
https://pubchem.ncbi.nlm.nih.gov/compound/136611126#section=2D-Structure
https://pubchem.ncbi.nlm.nih.gov/compound/136609123#section=2D-Structure
https://pubchem.ncbi.nlm.nih.gov/compound/136593849#section=2D-Structure
https://pubchem.ncbi.nlm.nih.gov/compound/136564#section=2D-Structure
https://pubchem.ncbi.nlm.nih.gov/compound/136506710#section=2D-Structure
https://pubchem.ncbi.nlm.nih.gov/compound/136503553#section=2D-Structure
https://pubchem.ncbi.nlm.nih.gov/compound/136500273#section=2D-Structure
https://pubchem.ncbi.nlm.nih.gov/compound/136499650#section=2D-Structure
https://pubchem.ncbi.nlm.nih.gov/compound/136498321#section=2D-Structure
https://pubchem.ncbi.nlm.nih.gov/compound/136488865#section=2D-Structure
https://pubchem.ncbi.nlm.nih.gov/compound/136486575#section=2D-Structure
https://pubchem.ncbi.nlm.nih.gov/compound/136485405#section=2D-Structure
https://pubchem.ncbi.nlm.nih.gov/compound/136482556#section=2D-Structure
https://pubchem.ncbi.nlm.nih.gov/compound/136480754#section=2D-Structure
https://pubchem.ncbi.nlm.nih.gov/compound/136478073#section=2D-Structure
https://pubchem.ncbi.nlm.nih.gov/compound/136477318#section=2D-Structure
https://pubchem.ncbi.nlm.nih.gov/compound/136477170#section=2D-Structure
https://pubchem.ncbi.nlm.nih.gov/compound/136477167#section=2D-Structure
https://pubchem.ncbi.nlm.nih.gov/compound/136471115#section=2D-Structure
https://pubchem.ncbi.nlm.nih.gov/compound/136466754#section=2D-Structure
https://pubchem.ncbi.nlm.nih.gov/compound/136466626#section=2D-Structure
https://pubchem.ncbi.nlm.nih.gov/compound/136462796#section=2D-Structure
https://pubchem.ncbi.nlm.nih.gov/compound/136462567#section=2D-Structure
https://pubchem.ncbi.nlm.nih.gov/compound/136459949#section=2D-Structure
https://pubchem.ncbi.nlm.nih.gov/compound/136457440#section=2D-Structure
https://pubchem.ncbi.nlm.nih.gov/compound/136453179#section=2D-Structure
https://pubchem.ncbi.nlm.nih.gov/compound/136451929#section=2D-Structure
https://pubchem.ncbi.nlm.nih.gov/compound/136449984#section=2D-Structure
https://pubchem.ncbi.nlm.nih.gov/compound/136446807#section=2D-Structure
https://pubchem.ncbi.nlm.nih.gov/compound/136446804#section=2D-Structure
https://pubchem.ncbi.nlm.nih.gov/compound/136443024#section=2D-Structure
https://pubchem.ncbi.nlm.nih.gov/compound/136441339#section=2D-Structure
https://pubchem.ncbi.nlm.nih.gov/compound/136439176#section=2D-Structure
https://pubchem.ncbi.nlm.nih.gov/compound/136438313#section=2D-Structure
https://pubchem.ncbi.nlm.nih.gov/compound/136435552#section=2D-Structure
https://pubchem.ncbi.nlm.nih.gov/compound/136431327#section=2D-Structure
https://pubchem.ncbi.nlm.nih.gov/compound/136426942#section=2D-Structure
https://pubchem.ncbi.nlm.nih.gov/compound/136423328#section=2D-Structure
https://pubchem.ncbi.nlm.nih.gov/compound/136417010#section=2D-Structure
https://pubchem.ncbi.nlm.nih.gov/compound/136416780#section=2D-Structure
https://pubchem.ncbi.nlm.nih.gov/compound/136416455#section=2D-Structure
https://pubchem.ncbi.nlm.nih.gov/compound/136415792#section=2D-Structure
https://pubchem.ncbi.nlm.nih.gov/compound/136411432#section=2D-Structure
https://pubchem.ncbi.nlm.nih.gov/compound/136405580#section=2D-Structure
https://pubchem.ncbi.nlm.nih.gov/compound/136404676#section=2D-Structure
https://pubchem.ncbi.nlm.nih.gov/compound/136403262#section=2D-Structure

https://pubchem.ncbi.nlm.nih.gov/compound/136398867#section=2D-Structure
https://pubchem.ncbi.nlm.nih.gov/compound/136398715#section=2D-Structure
https://pubchem.ncbi.nlm.nih.gov/compound/136378286#section=2D-Structure
https://pubchem.ncbi.nlm.nih.gov/compound/136364496#section=2D-Structure
https://pubchem.ncbi.nlm.nih.gov/compound/136361130#section=2D-Structure
https://pubchem.ncbi.nlm.nih.gov/compound/13635641#section=2D-Structure
https://pubchem.ncbi.nlm.nih.gov/compound/136353543#section=2D-Structure
https://pubchem.ncbi.nlm.nih.gov/compound/136353514#section=2D-Structure
https://pubchem.ncbi.nlm.nih.gov/compound/136353454#section=2D-Structure
https://pubchem.ncbi.nlm.nih.gov/compound/136351870#section=2D-Structure
https://pubchem.ncbi.nlm.nih.gov/compound/136347#section=2D-Structure
https://pubchem.ncbi.nlm.nih.gov/compound/136345353#section=2D-Structure
https://pubchem.ncbi.nlm.nih.gov/compound/136344730#section=2D-Structure
https://pubchem.ncbi.nlm.nih.gov/compound/136343127#section=2D-Structure
https://pubchem.ncbi.nlm.nih.gov/compound/136342965#section=2D-Structure
https://pubchem.ncbi.nlm.nih.gov/compound/136341758#section=2D-Structure
https://pubchem.ncbi.nlm.nih.gov/compound/136335859#section=2D-Structure
https://pubchem.ncbi.nlm.nih.gov/compound/136334683#section=2D-Structure
https://pubchem.ncbi.nlm.nih.gov/compound/136330915#section=2D-Structure
https://pubchem.ncbi.nlm.nih.gov/compound/136277566#section=2D-Structure
https://pubchem.ncbi.nlm.nih.gov/compound/136272531#section=2D-Structure
https://pubchem.ncbi.nlm.nih.gov/compound/136270327#section=2D-Structure
https://pubchem.ncbi.nlm.nih.gov/compound/136266797#section=2D-Structure
https://pubchem.ncbi.nlm.nih.gov/compound/136266790#section=2D-Structure
https://pubchem.ncbi.nlm.nih.gov/compound/136260893#section=2D-Structure
https://pubchem.ncbi.nlm.nih.gov/compound/136253300#section=2D-Structure
https://pubchem.ncbi.nlm.nih.gov/compound/136247303#section=2D-Structure
https://pubchem.ncbi.nlm.nih.gov/compound/136236003#section=2D-Structure
https://pubchem.ncbi.nlm.nih.gov/compound/136227801#section=2D-Structure
https://pubchem.ncbi.nlm.nih.gov/compound/136219761#section=2D-Structure
https://pubchem.ncbi.nlm.nih.gov/compound/136217262#section=2D-Structure
https://pubchem.ncbi.nlm.nih.gov/compound/136210653#section=2D-Structure
https://pubchem.ncbi.nlm.nih.gov/compound/136203487#section=2D-Structure
https://pubchem.ncbi.nlm.nih.gov/compound/136202052#section=2D-Structure
https://pubchem.ncbi.nlm.nih.gov/compound/136197899#section=2D-Structure
https://pubchem.ncbi.nlm.nih.gov/compound/136196968#section=2D-Structure
https://pubchem.ncbi.nlm.nih.gov/compound/136196389#section=2D-Structure
https://pubchem.ncbi.nlm.nih.gov/compound/136195564#section=2D-Structure
https://pubchem.ncbi.nlm.nih.gov/compound/136186380#section=2D-Structure
https://pubchem.ncbi.nlm.nih.gov/compound/136185857#section=2D-Structure
https://pubchem.ncbi.nlm.nih.gov/compound/13617943#section=2D-Structure
https://pubchem.ncbi.nlm.nih.gov/compound/136176274#section=2D-Structure
https://pubchem.ncbi.nlm.nih.gov/compound/136174278#section=2D-Structure
https://pubchem.ncbi.nlm.nih.gov/compound/136169436#section=2D-Structure
https://pubchem.ncbi.nlm.nih.gov/compound/136162643#section=2D-Structure
https://pubchem.ncbi.nlm.nih.gov/compound/136162642#section=2D-Structure
https://pubchem.ncbi.nlm.nih.gov/compound/136162630#section=2D-Structure
https://pubchem.ncbi.nlm.nih.gov/compound/136159792#section=2D-Structure
https://pubchem.ncbi.nlm.nih.gov/compound/136157#section=2D-Structure
https://pubchem.ncbi.nlm.nih.gov/compound/136156806#section=2D-Structure
https://pubchem.ncbi.nlm.nih.gov/compound/136156#section=2D-Structure
https://pubchem.ncbi.nlm.nih.gov/compound/136155134#section=2D-Structure
https://pubchem.ncbi.nlm.nih.gov/compound/136145165#section=2D-Structure
https://pubchem.ncbi.nlm.nih.gov/compound/136145152#section=2D-Structure
https://pubchem.ncbi.nlm.nih.gov/compound/136145149#section=2D-Structure

https://pubchem.ncbi.nlm.nih.gov/compound/136145130#section=2D-Structure
https://pubchem.ncbi.nlm.nih.gov/compound/136145127#section=2D-Structure
https://pubchem.ncbi.nlm.nih.gov/compound/136145114#section=2D-Structure
https://pubchem.ncbi.nlm.nih.gov/compound/136145096#section=2D-Structure
https://pubchem.ncbi.nlm.nih.gov/compound/136140341#section=2D-Structure
https://pubchem.ncbi.nlm.nih.gov/compound/136140338#section=2D-Structure
https://pubchem.ncbi.nlm.nih.gov/compound/136139862#section=2D-Structure
https://pubchem.ncbi.nlm.nih.gov/compound/136117125#section=2D-Structure
https://pubchem.ncbi.nlm.nih.gov/compound/136102948#section=2D-Structure
https://pubchem.ncbi.nlm.nih.gov/compound/136102887#section=2D-Structure
https://pubchem.ncbi.nlm.nih.gov/compound/136090888#section=2D-Structure
https://pubchem.ncbi.nlm.nih.gov/compound/136088362#section=2D-Structure
https://pubchem.ncbi.nlm.nih.gov/compound/136086501#section=2D-Structure
https://pubchem.ncbi.nlm.nih.gov/compound/136081426#section=2D-Structure
https://pubchem.ncbi.nlm.nih.gov/compound/136069979#section=2D-Structure
https://pubchem.ncbi.nlm.nih.gov/compound/136064561#section=2D-Structure
https://pubchem.ncbi.nlm.nih.gov/compound/136057#section=2D-Structure
https://pubchem.ncbi.nlm.nih.gov/compound/136045313#section=2D-Structure
https://pubchem.ncbi.nlm.nih.gov/compound/136044237#section=2D-Structure
https://pubchem.ncbi.nlm.nih.gov/compound/136023204#section=2D-Structure
https://pubchem.ncbi.nlm.nih.gov/compound/136020#section=2D-Structure
https://pubchem.ncbi.nlm.nih.gov/compound/136019993#section=2D-Structure
https://pubchem.ncbi.nlm.nih.gov/compound/136006560#section=2D-Structure
https://pubchem.ncbi.nlm.nih.gov/compound/135993082#section=2D-Structure
https://pubchem.ncbi.nlm.nih.gov/compound/135991615#section=2D-Structure
https://pubchem.ncbi.nlm.nih.gov/compound/135984098#section=2D-Structure
https://pubchem.ncbi.nlm.nih.gov/compound/135983161#section=2D-Structure
https://pubchem.ncbi.nlm.nih.gov/compound/135979321#section=2D-Structure
https://pubchem.ncbi.nlm.nih.gov/compound/135977793#section=2D-Structure
https://pubchem.ncbi.nlm.nih.gov/compound/135976856#section=2D-Structure
https://pubchem.ncbi.nlm.nih.gov/compound/135976147#section=2D-Structure
https://pubchem.ncbi.nlm.nih.gov/compound/135973969#section=2D-Structure
https://pubchem.ncbi.nlm.nih.gov/compound/13596875#section=2D-Structure
https://pubchem.ncbi.nlm.nih.gov/compound/135967302#section=2D-Structure
https://pubchem.ncbi.nlm.nih.gov/compound/13596585#section=2D-Structure
https://pubchem.ncbi.nlm.nih.gov/compound/135956070#section=2D-Structure
https://pubchem.ncbi.nlm.nih.gov/compound/135900326#section=2D-Structure
https://pubchem.ncbi.nlm.nih.gov/compound/135883873#section=2D-Structure
https://pubchem.ncbi.nlm.nih.gov/compound/135870324#section=2D-Structure
https://pubchem.ncbi.nlm.nih.gov/compound/135856722#section=2D-Structure
https://pubchem.ncbi.nlm.nih.gov/compound/135839649#section=2D-Structure
https://pubchem.ncbi.nlm.nih.gov/compound/135838369#section=2D-Structure
https://pubchem.ncbi.nlm.nih.gov/compound/135837450#section=2D-Structure
https://pubchem.ncbi.nlm.nih.gov/compound/135837449#section=2D-Structure
https://pubchem.ncbi.nlm.nih.gov/compound/135828209#section=2D-Structure
https://pubchem.ncbi.nlm.nih.gov/compound/135822824#section=2D-Structure
https://pubchem.ncbi.nlm.nih.gov/compound/135822644#section=2D-Structure
https://pubchem.ncbi.nlm.nih.gov/compound/135819033#section=2D-Structure
https://pubchem.ncbi.nlm.nih.gov/compound/135818742#section=2D-Structure
https://pubchem.ncbi.nlm.nih.gov/compound/135818719#section=2D-Structure
https://pubchem.ncbi.nlm.nih.gov/compound/135818718#section=2D-Structure
https://pubchem.ncbi.nlm.nih.gov/compound/135818713#section=2D-Structure
https://pubchem.ncbi.nlm.nih.gov/compound/135818712#section=2D-Structure
https://pubchem.ncbi.nlm.nih.gov/compound/135817497#section=2D-Structure
https://pubchem.ncbi.nlm.nih.gov/compound/135817494#section=2D-Structure

https://pubchem.ncbi.nlm.nih.gov/compound/135802621#section=2D-Structure
https://pubchem.ncbi.nlm.nih.gov/compound/135802618#section=2D-Structure
https://pubchem.ncbi.nlm.nih.gov/compound/135797782#section=2D-Structure
https://pubchem.ncbi.nlm.nih.gov/compound/135794147#section=2D-Structure
https://pubchem.ncbi.nlm.nih.gov/compound/135782709#section=2D-Structure
https://pubchem.ncbi.nlm.nih.gov/compound/135782705#section=2D-Structure
https://pubchem.ncbi.nlm.nih.gov/compound/135780329#section=2D-Structure
https://pubchem.ncbi.nlm.nih.gov/compound/135778966#section=2D-Structure
https://pubchem.ncbi.nlm.nih.gov/compound/135778492#section=2D-Structure
https://pubchem.ncbi.nlm.nih.gov/compound/135767393#section=2D-Structure
https://pubchem.ncbi.nlm.nih.gov/compound/135766963#section=2D-Structure
https://pubchem.ncbi.nlm.nih.gov/compound/135766962#section=2D-Structure
https://pubchem.ncbi.nlm.nih.gov/compound/135746881#section=2D-Structure
https://pubchem.ncbi.nlm.nih.gov/compound/13574608#section=2D-Structure
https://pubchem.ncbi.nlm.nih.gov/compound/135743875#section=2D-Structure
https://pubchem.ncbi.nlm.nih.gov/compound/135741916#section=2D-Structure
https://pubchem.ncbi.nlm.nih.gov/compound/135741915#section=2D-Structure
https://pubchem.ncbi.nlm.nih.gov/compound/135741914#section=2D-Structure
https://pubchem.ncbi.nlm.nih.gov/compound/135741275#section=2D-Structure
https://pubchem.ncbi.nlm.nih.gov/compound/135736906#section=2D-Structure
https://pubchem.ncbi.nlm.nih.gov/compound/135736652#section=2D-Structure
https://pubchem.ncbi.nlm.nih.gov/compound/135736651#section=2D-Structure
https://pubchem.ncbi.nlm.nih.gov/compound/135736627#section=2D-Structure
https://pubchem.ncbi.nlm.nih.gov/compound/135735556#section=2D-Structure
https://pubchem.ncbi.nlm.nih.gov/compound/135735514#section=2D-Structure
https://pubchem.ncbi.nlm.nih.gov/compound/135735512#section=2D-Structure
https://pubchem.ncbi.nlm.nih.gov/compound/135735511#section=2D-Structure
https://pubchem.ncbi.nlm.nih.gov/compound/135732709#section=2D-Structure
https://pubchem.ncbi.nlm.nih.gov/compound/135732707#section=2D-Structure
https://pubchem.ncbi.nlm.nih.gov/compound/135732705#section=2D-Structure
https://pubchem.ncbi.nlm.nih.gov/compound/135711163#section=2D-Structure
https://pubchem.ncbi.nlm.nih.gov/compound/135704064#section=2D-Structure
https://pubchem.ncbi.nlm.nih.gov/compound/135697447#section=2D-Structure
https://pubchem.ncbi.nlm.nih.gov/compound/135697199#section=2D-Structure
https://pubchem.ncbi.nlm.nih.gov/compound/135692489#section=2D-Structure
https://pubchem.ncbi.nlm.nih.gov/compound/135692478#section=2D-Structure
https://pubchem.ncbi.nlm.nih.gov/compound/135689#section=2D-Structure
https://pubchem.ncbi.nlm.nih.gov/compound/135687065#section=2D-Structure
https://pubchem.ncbi.nlm.nih.gov/compound/135687064#section=2D-Structure
https://pubchem.ncbi.nlm.nih.gov/compound/135687002#section=2D-Structure
https://pubchem.ncbi.nlm.nih.gov/compound/135662#section=2D-Structure
https://pubchem.ncbi.nlm.nih.gov/compound/135632#section=2D-Structure
https://pubchem.ncbi.nlm.nih.gov/compound/135616208#section=2D-Structure
https://pubchem.ncbi.nlm.nih.gov/compound/135612989#section=2D-Structure
https://pubchem.ncbi.nlm.nih.gov/compound/135611148#section=2D-Structure
https://pubchem.ncbi.nlm.nih.gov/compound/135604464#section=2D-Structure
https://pubchem.ncbi.nlm.nih.gov/compound/135602114#section=2D-Structure
https://pubchem.ncbi.nlm.nih.gov/compound/135601411#section=2D-Structure
https://pubchem.ncbi.nlm.nih.gov/compound/135596#section=2D-Structure
https://pubchem.ncbi.nlm.nih.gov/compound/135590599#section=2D-Structure
https://pubchem.ncbi.nlm.nih.gov/compound/135590597#section=2D-Structure
https://pubchem.ncbi.nlm.nih.gov/compound/135583#section=2D-Structure
https://pubchem.ncbi.nlm.nih.gov/compound/135581323#section=2D-Structure
https://pubchem.ncbi.nlm.nih.gov/compound/135580926#section=2D-Structure
https://pubchem.ncbi.nlm.nih.gov/compound/135580#section=2D-Structure

https://pubchem.ncbi.nlm.nih.gov/compound/135566591#section=2D-Structure
https://pubchem.ncbi.nlm.nih.gov/compound/135563936#section=2D-Structure
https://pubchem.ncbi.nlm.nih.gov/compound/135563856#section=2D-Structure
https://pubchem.ncbi.nlm.nih.gov/compound/135556675#section=2D-Structure
https://pubchem.ncbi.nlm.nih.gov/compound/135556674#section=2D-Structure
https://pubchem.ncbi.nlm.nih.gov/compound/135552064#section=2D-Structure
https://pubchem.ncbi.nlm.nih.gov/compound/135546365#section=2D-Structure
https://pubchem.ncbi.nlm.nih.gov/compound/135544557#section=2D-Structure
https://pubchem.ncbi.nlm.nih.gov/compound/135533977#section=2D-Structure
https://pubchem.ncbi.nlm.nih.gov/compound/13553135#section=2D-Structure
https://pubchem.ncbi.nlm.nih.gov/compound/13553133#section=2D-Structure
https://pubchem.ncbi.nlm.nih.gov/compound/135531177#section=2D-Structure
https://pubchem.ncbi.nlm.nih.gov/compound/135528#section=2D-Structure
https://pubchem.ncbi.nlm.nih.gov/compound/135524467#section=2D-Structure
https://pubchem.ncbi.nlm.nih.gov/compound/135523701#section=2D-Structure
https://pubchem.ncbi.nlm.nih.gov/compound/135522061#section=2D-Structure
https://pubchem.ncbi.nlm.nih.gov/compound/135514780#section=2D-Structure
https://pubchem.ncbi.nlm.nih.gov/compound/135513248#section=2D-Structure
https://pubchem.ncbi.nlm.nih.gov/compound/135508589#section=2D-Structure
https://pubchem.ncbi.nlm.nih.gov/compound/135504679#section=2D-Structure
https://pubchem.ncbi.nlm.nih.gov/compound/135503521#section=2D-Structure
https://pubchem.ncbi.nlm.nih.gov/compound/135500728#section=2D-Structure
https://pubchem.ncbi.nlm.nih.gov/compound/135499785#section=2D-Structure
https://pubchem.ncbi.nlm.nih.gov/compound/135497195#section=2D-Structure
https://pubchem.ncbi.nlm.nih.gov/compound/135494975#section=2D-Structure
https://pubchem.ncbi.nlm.nih.gov/compound/135493763#section=2D-Structure
https://pubchem.ncbi.nlm.nih.gov/compound/135493762#section=2D-Structure
https://pubchem.ncbi.nlm.nih.gov/compound/135493761#section=2D-Structure
https://pubchem.ncbi.nlm.nih.gov/compound/135484967#section=2D-Structure
https://pubchem.ncbi.nlm.nih.gov/compound/135480545#section=2D-Structure
https://pubchem.ncbi.nlm.nih.gov/compound/135474167#section=2D-Structure
https://pubchem.ncbi.nlm.nih.gov/compound/135471514#section=2D-Structure
https://pubchem.ncbi.nlm.nih.gov/compound/135471393#section=2D-Structure
https://pubchem.ncbi.nlm.nih.gov/compound/135468954#section=2D-Structure
https://pubchem.ncbi.nlm.nih.gov/compound/135464258#section=2D-Structure
https://pubchem.ncbi.nlm.nih.gov/compound/135463214#section=2D-Structure
https://pubchem.ncbi.nlm.nih.gov/compound/135460250#section=2D-Structure
https://pubchem.ncbi.nlm.nih.gov/compound/135460249#section=2D-Structure
https://pubchem.ncbi.nlm.nih.gov/compound/135457031#section=2D-Structure
https://pubchem.ncbi.nlm.nih.gov/compound/135456890#section=2D-Structure
https://pubchem.ncbi.nlm.nih.gov/compound/135456764#section=2D-Structure
https://pubchem.ncbi.nlm.nih.gov/compound/135456678#section=2D-Structure
https://pubchem.ncbi.nlm.nih.gov/compound/135454059#section=2D-Structure
https://pubchem.ncbi.nlm.nih.gov/compound/135453893#section=2D-Structure
https://pubchem.ncbi.nlm.nih.gov/compound/135453643#section=2D-Structure
https://pubchem.ncbi.nlm.nih.gov/compound/135452590#section=2D-Structure
https://pubchem.ncbi.nlm.nih.gov/compound/135452589#section=2D-Structure
https://pubchem.ncbi.nlm.nih.gov/compound/135452459#section=2D-Structure
https://pubchem.ncbi.nlm.nih.gov/compound/135452433#section=2D-Structure
https://pubchem.ncbi.nlm.nih.gov/compound/135451648#section=2D-Structure
https://pubchem.ncbi.nlm.nih.gov/compound/135449044#section=2D-Structure
https://pubchem.ncbi.nlm.nih.gov/compound/135447042#section=2D-Structure
https://pubchem.ncbi.nlm.nih.gov/compound/135444589#section=2D-Structure
https://pubchem.ncbi.nlm.nih.gov/compound/135444363#section=2D-Structure
https://pubchem.ncbi.nlm.nih.gov/compound/135443583#section=2D-Structure

https://pubchem.ncbi.nlm.nih.gov/compound/135443283#section=2D-Structure
https://pubchem.ncbi.nlm.nih.gov/compound/135442801#section=2D-Structure
https://pubchem.ncbi.nlm.nih.gov/compound/135442614#section=2D-Structure
https://pubchem.ncbi.nlm.nih.gov/compound/135441800#section=2D-Structure
https://pubchem.ncbi.nlm.nih.gov/compound/135441779#section=2D-Structure
https://pubchem.ncbi.nlm.nih.gov/compound/135440604#section=2D-Structure
https://pubchem.ncbi.nlm.nih.gov/compound/135440594#section=2D-Structure
https://pubchem.ncbi.nlm.nih.gov/compound/135430298#section=2D-Structure
https://pubchem.ncbi.nlm.nih.gov/compound/135426814#section=2D-Structure
https://pubchem.ncbi.nlm.nih.gov/compound/135426380#section=2D-Structure
https://pubchem.ncbi.nlm.nih.gov/compound/135424411#section=2D-Structure
https://pubchem.ncbi.nlm.nih.gov/compound/135423664#section=2D-Structure
https://pubchem.ncbi.nlm.nih.gov/compound/135412648#section=2D-Structure
https://pubchem.ncbi.nlm.nih.gov/compound/135410226#section=2D-Structure
https://pubchem.ncbi.nlm.nih.gov/compound/135409921#section=2D-Structure
https://pubchem.ncbi.nlm.nih.gov/compound/135406748#section=2D-Structure
https://pubchem.ncbi.nlm.nih.gov/compound/135403802#section=2D-Structure
https://pubchem.ncbi.nlm.nih.gov/compound/135401184#section=2D-Structure
https://pubchem.ncbi.nlm.nih.gov/compound/135398181#section=2D-Structure
https://pubchem.ncbi.nlm.nih.gov/compound/135393659#section=2D-Structure
https://pubchem.ncbi.nlm.nih.gov/compound/135393611#section=2D-Structure
https://pubchem.ncbi.nlm.nih.gov/compound/135389272#section=2D-Structure
https://pubchem.ncbi.nlm.nih.gov/compound/135380342#section=2D-Structure
https://pubchem.ncbi.nlm.nih.gov/compound/135374076#section=2D-Structure
https://pubchem.ncbi.nlm.nih.gov/compound/135301487#section=2D-Structure
https://pubchem.ncbi.nlm.nih.gov/compound/135298353#section=2D-Structure
https://pubchem.ncbi.nlm.nih.gov/compound/135283186#section=2D-Structure
https://pubchem.ncbi.nlm.nih.gov/compound/135237233#section=2D-Structure
https://pubchem.ncbi.nlm.nih.gov/compound/135215198#section=2D-Structure
https://pubchem.ncbi.nlm.nih.gov/compound/135214994#section=2D-Structure
https://pubchem.ncbi.nlm.nih.gov/compound/135214992#section=2D-Structure
https://pubchem.ncbi.nlm.nih.gov/compound/135202330#section=2D-Structure
https://pubchem.ncbi.nlm.nih.gov/compound/135183664#section=2D-Structure
https://pubchem.ncbi.nlm.nih.gov/compound/135061542#section=2D-Structure
https://pubchem.ncbi.nlm.nih.gov/compound/135025141#section=2D-Structure
https://pubchem.ncbi.nlm.nih.gov/compound/135023453#section=2D-Structure
https://pubchem.ncbi.nlm.nih.gov/compound/135015159#section=2D-Structure
https://pubchem.ncbi.nlm.nih.gov/compound/135008730#section=2D-Structure
https://pubchem.ncbi.nlm.nih.gov/compound/134997069#section=2D-Structure
https://pubchem.ncbi.nlm.nih.gov/compound/134989724#section=2D-Structure
https://pubchem.ncbi.nlm.nih.gov/compound/134989464#section=2D-Structure
https://pubchem.ncbi.nlm.nih.gov/compound/134961413#section=2D-Structure
https://pubchem.ncbi.nlm.nih.gov/compound/134916239#section=2D-Structure
https://pubchem.ncbi.nlm.nih.gov/compound/134912799#section=2D-Structure
https://pubchem.ncbi.nlm.nih.gov/compound/134898103#section=2D-Structure
https://pubchem.ncbi.nlm.nih.gov/compound/134895144#section=2D-Structure
https://pubchem.ncbi.nlm.nih.gov/compound/134839403#section=2D-Structure
https://pubchem.ncbi.nlm.nih.gov/compound/134837255#section=2D-Structure
https://pubchem.ncbi.nlm.nih.gov/compound/134822205#section=2D-Structure
https://pubchem.ncbi.nlm.nih.gov/compound/134817901#section=2D-Structure
https://pubchem.ncbi.nlm.nih.gov/compound/134817892#section=2D-Structure
https://pubchem.ncbi.nlm.nih.gov/compound/134813943#section=2D-Structure
https://pubchem.ncbi.nlm.nih.gov/compound/134693381#section=2D-Structure
https://pubchem.ncbi.nlm.nih.gov/compound/134693361#section=2D-Structure
https://pubchem.ncbi.nlm.nih.gov/compound/134693360#section=2D-Structure

https://pubchem.ncbi.nlm.nih.gov/compound/13466096#section=2D-Structure
https://pubchem.ncbi.nlm.nih.gov/compound/13466090#section=2D-Structure
https://pubchem.ncbi.nlm.nih.gov/compound/13466083#section=2D-Structure
https://pubchem.ncbi.nlm.nih.gov/compound/13465292#section=2D-Structure
https://pubchem.ncbi.nlm.nih.gov/compound/13465251#section=2D-Structure
https://pubchem.ncbi.nlm.nih.gov/compound/13465249#section=2D-Structure
https://pubchem.ncbi.nlm.nih.gov/compound/134528399#section=2D-Structure
https://pubchem.ncbi.nlm.nih.gov/compound/134502369#section=2D-Structure
https://pubchem.ncbi.nlm.nih.gov/compound/134466761#section=2D-Structure
https://pubchem.ncbi.nlm.nih.gov/compound/13443210#section=2D-Structure
https://pubchem.ncbi.nlm.nih.gov/compound/13440492#section=2D-Structure
https://pubchem.ncbi.nlm.nih.gov/compound/134401377#section=2D-Structure
https://pubchem.ncbi.nlm.nih.gov/compound/134389457#section=2D-Structure
https://pubchem.ncbi.nlm.nih.gov/compound/134385988#section=2D-Structure
https://pubchem.ncbi.nlm.nih.gov/compound/134346932#section=2D-Structure
https://pubchem.ncbi.nlm.nih.gov/compound/13433933#section=2D-Structure
https://pubchem.ncbi.nlm.nih.gov/compound/134272491#section=2D-Structure
https://pubchem.ncbi.nlm.nih.gov/compound/13417960#section=2D-Structure
https://pubchem.ncbi.nlm.nih.gov/compound/134170651#section=2D-Structure
https://pubchem.ncbi.nlm.nih.gov/compound/134158551#section=2D-Structure
https://pubchem.ncbi.nlm.nih.gov/compound/13413071#section=2D-Structure
https://pubchem.ncbi.nlm.nih.gov/compound/13413067#section=2D-Structure
https://pubchem.ncbi.nlm.nih.gov/compound/134096170#section=2D-Structure
https://pubchem.ncbi.nlm.nih.gov/compound/134078#section=2D-Structure
https://pubchem.ncbi.nlm.nih.gov/compound/13407078#section=2D-Structure
https://pubchem.ncbi.nlm.nih.gov/compound/133590003#section=2D-Structure
https://pubchem.ncbi.nlm.nih.gov/compound/13333755#section=2D-Structure
https://pubchem.ncbi.nlm.nih.gov/compound/13332628#section=2D-Structure
https://pubchem.ncbi.nlm.nih.gov/compound/133188368#section=2D-Structure
https://pubchem.ncbi.nlm.nih.gov/compound/133083506#section=2D-Structure
https://pubchem.ncbi.nlm.nih.gov/compound/133083397#section=2D-Structure
https://pubchem.ncbi.nlm.nih.gov/compound/133064871#section=2D-Structure
https://pubchem.ncbi.nlm.nih.gov/compound/133064149#section=2D-Structure
https://pubchem.ncbi.nlm.nih.gov/compound/133063257#section=2D-Structure
https://pubchem.ncbi.nlm.nih.gov/compound/133061238#section=2D-Structure
https://pubchem.ncbi.nlm.nih.gov/compound/133054712#section=2D-Structure
https://pubchem.ncbi.nlm.nih.gov/compound/133054279#section=2D-Structure
https://pubchem.ncbi.nlm.nih.gov/compound/132991050#section=2D-Structure
https://pubchem.ncbi.nlm.nih.gov/compound/13295870#section=2D-Structure
https://pubchem.ncbi.nlm.nih.gov/compound/132941053#section=2D-Structure
https://pubchem.ncbi.nlm.nih.gov/compound/132916186#section=2D-Structure
https://pubchem.ncbi.nlm.nih.gov/compound/132916185#section=2D-Structure
https://pubchem.ncbi.nlm.nih.gov/compound/132915360#section=2D-Structure
https://pubchem.ncbi.nlm.nih.gov/compound/132915245#section=2D-Structure
https://pubchem.ncbi.nlm.nih.gov/compound/132539144#section=2D-Structure
https://pubchem.ncbi.nlm.nih.gov/compound/132533670#section=2D-Structure
https://pubchem.ncbi.nlm.nih.gov/compound/132512012#section=2D-Structure
https://pubchem.ncbi.nlm.nih.gov/compound/132511254#section=2D-Structure
https://pubchem.ncbi.nlm.nih.gov/compound/132507082#section=2D-Structure
https://pubchem.ncbi.nlm.nih.gov/compound/132493843#section=2D-Structure
https://pubchem.ncbi.nlm.nih.gov/compound/132489147#section=2D-Structure
https://pubchem.ncbi.nlm.nih.gov/compound/13244876#section=2D-Structure
https://pubchem.ncbi.nlm.nih.gov/compound/132427394#section=2D-Structure
https://pubchem.ncbi.nlm.nih.gov/compound/132426891#section=2D-Structure
https://pubchem.ncbi.nlm.nih.gov/compound/132281280#section=2D-Structure

https://pubchem.ncbi.nlm.nih.gov/compound/132280960#section=2D-Structure
https://pubchem.ncbi.nlm.nih.gov/compound/132280911#section=2D-Structure
https://pubchem.ncbi.nlm.nih.gov/compound/132279777#section=2D-Structure
https://pubchem.ncbi.nlm.nih.gov/compound/132279439#section=2D-Structure
https://pubchem.ncbi.nlm.nih.gov/compound/132277160#section=2D-Structure
https://pubchem.ncbi.nlm.nih.gov/compound/132275684#section=2D-Structure
https://pubchem.ncbi.nlm.nih.gov/compound/132275612#section=2D-Structure
https://pubchem.ncbi.nlm.nih.gov/compound/132264650#section=2D-Structure
https://pubchem.ncbi.nlm.nih.gov/compound/132236119#section=2D-Structure
https://pubchem.ncbi.nlm.nih.gov/compound/132204277#section=2D-Structure
https://pubchem.ncbi.nlm.nih.gov/compound/132204205#section=2D-Structure
https://pubchem.ncbi.nlm.nih.gov/compound/132204167#section=2D-Structure
https://pubchem.ncbi.nlm.nih.gov/compound/132204161#section=2D-Structure
https://pubchem.ncbi.nlm.nih.gov/compound/132204156#section=2D-Structure
https://pubchem.ncbi.nlm.nih.gov/compound/132204074#section=2D-Structure
https://pubchem.ncbi.nlm.nih.gov/compound/132203964#section=2D-Structure
https://pubchem.ncbi.nlm.nih.gov/compound/132203961#section=2D-Structure
https://pubchem.ncbi.nlm.nih.gov/compound/132203960#section=2D-Structure
https://pubchem.ncbi.nlm.nih.gov/compound/132203959#section=2D-Structure
https://pubchem.ncbi.nlm.nih.gov/compound/132203949#section=2D-Structure
https://pubchem.ncbi.nlm.nih.gov/compound/132203948#section=2D-Structure
https://pubchem.ncbi.nlm.nih.gov/compound/132203946#section=2D-Structure
https://pubchem.ncbi.nlm.nih.gov/compound/132203936#section=2D-Structure
https://pubchem.ncbi.nlm.nih.gov/compound/132051274#section=2D-Structure
https://pubchem.ncbi.nlm.nih.gov/compound/132049905#section=2D-Structure
https://pubchem.ncbi.nlm.nih.gov/compound/132016976#section=2D-Structure
https://pubchem.ncbi.nlm.nih.gov/compound/131994140#section=2D-Structure
https://pubchem.ncbi.nlm.nih.gov/compound/13199371#section=2D-Structure
https://pubchem.ncbi.nlm.nih.gov/compound/131953175#section=2D-Structure
https://pubchem.ncbi.nlm.nih.gov/compound/13194137#section=2D-Structure
https://pubchem.ncbi.nlm.nih.gov/compound/131708513#section=2D-Structure
https://pubchem.ncbi.nlm.nih.gov/compound/131674253#section=2D-Structure
https://pubchem.ncbi.nlm.nih.gov/compound/131668499#section=2D-Structure
https://pubchem.ncbi.nlm.nih.gov/compound/13155836#section=2D-Structure
https://pubchem.ncbi.nlm.nih.gov/compound/13143888#section=2D-Structure
https://pubchem.ncbi.nlm.nih.gov/compound/13143878#section=2D-Structure
https://pubchem.ncbi.nlm.nih.gov/compound/13142450#section=2D-Structure
https://pubchem.ncbi.nlm.nih.gov/compound/13076#section=2D-Structure
https://pubchem.ncbi.nlm.nih.gov/compound/13070131#section=2D-Structure
https://pubchem.ncbi.nlm.nih.gov/compound/13069#section=2D-Structure
https://pubchem.ncbi.nlm.nih.gov/compound/13068655#section=2D-Structure
https://pubchem.ncbi.nlm.nih.gov/compound/13068490#section=2D-Structure
https://pubchem.ncbi.nlm.nih.gov/compound/13068#section=2D-Structure
https://pubchem.ncbi.nlm.nih.gov/compound/13064692#section=2D-Structure
https://pubchem.ncbi.nlm.nih.gov/compound/13059841#section=2D-Structure
https://pubchem.ncbi.nlm.nih.gov/compound/13058789#section=2D-Structure
https://pubchem.ncbi.nlm.nih.gov/compound/13053337#section=2D-Structure
https://pubchem.ncbi.nlm.nih.gov/compound/130448732#section=2D-Structure
https://pubchem.ncbi.nlm.nih.gov/compound/130421036#section=2D-Structure
https://pubchem.ncbi.nlm.nih.gov/compound/130404599#section=2D-Structure
https://pubchem.ncbi.nlm.nih.gov/compound/130399991#section=2D-Structure
https://pubchem.ncbi.nlm.nih.gov/compound/130350724#section=2D-Structure
https://pubchem.ncbi.nlm.nih.gov/compound/130344737#section=2D-Structure
https://pubchem.ncbi.nlm.nih.gov/compound/13032860#section=2D-Structure
https://pubchem.ncbi.nlm.nih.gov/compound/130319136#section=2D-Structure

https://pubchem.ncbi.nlm.nih.gov/compound/130316198#section=2D-Structure
https://pubchem.ncbi.nlm.nih.gov/compound/130307738#section=2D-Structure
https://pubchem.ncbi.nlm.nih.gov/compound/130291444#section=2D-Structure
https://pubchem.ncbi.nlm.nih.gov/compound/130291102#section=2D-Structure
https://pubchem.ncbi.nlm.nih.gov/compound/130290942#section=2D-Structure
https://pubchem.ncbi.nlm.nih.gov/compound/130290929#section=2D-Structure
https://pubchem.ncbi.nlm.nih.gov/compound/130290900#section=2D-Structure
https://pubchem.ncbi.nlm.nih.gov/compound/130290887#section=2D-Structure
https://pubchem.ncbi.nlm.nih.gov/compound/130290833#section=2D-Structure
https://pubchem.ncbi.nlm.nih.gov/compound/130290803#section=2D-Structure
https://pubchem.ncbi.nlm.nih.gov/compound/130290775#section=2D-Structure
https://pubchem.ncbi.nlm.nih.gov/compound/130290664#section=2D-Structure
https://pubchem.ncbi.nlm.nih.gov/compound/130290509#section=2D-Structure
https://pubchem.ncbi.nlm.nih.gov/compound/130273957#section=2D-Structure
https://pubchem.ncbi.nlm.nih.gov/compound/130252#section=2D-Structure
https://pubchem.ncbi.nlm.nih.gov/compound/130241664#section=2D-Structure
https://pubchem.ncbi.nlm.nih.gov/compound/130221343#section=2D-Structure
https://pubchem.ncbi.nlm.nih.gov/compound/130209573#section=2D-Structure
https://pubchem.ncbi.nlm.nih.gov/compound/130209565#section=2D-Structure
https://pubchem.ncbi.nlm.nih.gov/compound/130209542#section=2D-Structure
https://pubchem.ncbi.nlm.nih.gov/compound/130177891#section=2D-Structure
https://pubchem.ncbi.nlm.nih.gov/compound/130177201#section=2D-Structure
https://pubchem.ncbi.nlm.nih.gov/compound/13012447#section=2D-Structure
https://pubchem.ncbi.nlm.nih.gov/compound/13011295#section=2D-Structure
https://pubchem.ncbi.nlm.nih.gov/compound/13008117#section=2D-Structure
https://pubchem.ncbi.nlm.nih.gov/compound/13008115#section=2D-Structure
https://pubchem.ncbi.nlm.nih.gov/compound/13001623#section=2D-Structure
https://pubchem.ncbi.nlm.nih.gov/compound/129906#section=2D-Structure
https://pubchem.ncbi.nlm.nih.gov/compound/129892585#section=2D-Structure
https://pubchem.ncbi.nlm.nih.gov/compound/129892260#section=2D-Structure
https://pubchem.ncbi.nlm.nih.gov/compound/129892041#section=2D-Structure
https://pubchem.ncbi.nlm.nih.gov/compound/129891049#section=2D-Structure
https://pubchem.ncbi.nlm.nih.gov/compound/129891014#section=2D-Structure
https://pubchem.ncbi.nlm.nih.gov/compound/129890059#section=2D-Structure
https://pubchem.ncbi.nlm.nih.gov/compound/129886314#section=2D-Structure
https://pubchem.ncbi.nlm.nih.gov/compound/129885823#section=2D-Structure
https://pubchem.ncbi.nlm.nih.gov/compound/129884785#section=2D-Structure
https://pubchem.ncbi.nlm.nih.gov/compound/129884706#section=2D-Structure
https://pubchem.ncbi.nlm.nih.gov/compound/129884552#section=2D-Structure
https://pubchem.ncbi.nlm.nih.gov/compound/129883915#section=2D-Structure
https://pubchem.ncbi.nlm.nih.gov/compound/129883101#section=2D-Structure
https://pubchem.ncbi.nlm.nih.gov/compound/129883063#section=2D-Structure
https://pubchem.ncbi.nlm.nih.gov/compound/129881678#section=2D-Structure
https://pubchem.ncbi.nlm.nih.gov/compound/129881441#section=2D-Structure
https://pubchem.ncbi.nlm.nih.gov/compound/129881428#section=2D-Structure
https://pubchem.ncbi.nlm.nih.gov/compound/129881158#section=2D-Structure
https://pubchem.ncbi.nlm.nih.gov/compound/129881132#section=2D-Structure
https://pubchem.ncbi.nlm.nih.gov/compound/129879369#section=2D-Structure
https://pubchem.ncbi.nlm.nih.gov/compound/129878367#section=2D-Structure
https://pubchem.ncbi.nlm.nih.gov/compound/129878338#section=2D-Structure
https://pubchem.ncbi.nlm.nih.gov/compound/129878213#section=2D-Structure
https://pubchem.ncbi.nlm.nih.gov/compound/129877585#section=2D-Structure
https://pubchem.ncbi.nlm.nih.gov/compound/129877548#section=2D-Structure
https://pubchem.ncbi.nlm.nih.gov/compound/129877453#section=2D-Structure
https://pubchem.ncbi.nlm.nih.gov/compound/129877083#section=2D-Structure

https://pubchem.ncbi.nlm.nih.gov/compound/129876847#section=2D-Structure
https://pubchem.ncbi.nlm.nih.gov/compound/129876735#section=2D-Structure
https://pubchem.ncbi.nlm.nih.gov/compound/129876423#section=2D-Structure
https://pubchem.ncbi.nlm.nih.gov/compound/129876295#section=2D-Structure
https://pubchem.ncbi.nlm.nih.gov/compound/129876133#section=2D-Structure
https://pubchem.ncbi.nlm.nih.gov/compound/129876123#section=2D-Structure
https://pubchem.ncbi.nlm.nih.gov/compound/129876023#section=2D-Structure
https://pubchem.ncbi.nlm.nih.gov/compound/129875783#section=2D-Structure
https://pubchem.ncbi.nlm.nih.gov/compound/129874938#section=2D-Structure
https://pubchem.ncbi.nlm.nih.gov/compound/129874402#section=2D-Structure
https://pubchem.ncbi.nlm.nih.gov/compound/129874235#section=2D-Structure
https://pubchem.ncbi.nlm.nih.gov/compound/129874166#section=2D-Structure
https://pubchem.ncbi.nlm.nih.gov/compound/129873895#section=2D-Structure
https://pubchem.ncbi.nlm.nih.gov/compound/129873766#section=2D-Structure
https://pubchem.ncbi.nlm.nih.gov/compound/129873597#section=2D-Structure
https://pubchem.ncbi.nlm.nih.gov/compound/129873565#section=2D-Structure
https://pubchem.ncbi.nlm.nih.gov/compound/129873562#section=2D-Structure
https://pubchem.ncbi.nlm.nih.gov/compound/129873475#section=2D-Structure
https://pubchem.ncbi.nlm.nih.gov/compound/129872731#section=2D-Structure
https://pubchem.ncbi.nlm.nih.gov/compound/129872551#section=2D-Structure
https://pubchem.ncbi.nlm.nih.gov/compound/129872146#section=2D-Structure
https://pubchem.ncbi.nlm.nih.gov/compound/129871943#section=2D-Structure
https://pubchem.ncbi.nlm.nih.gov/compound/129871806#section=2D-Structure
https://pubchem.ncbi.nlm.nih.gov/compound/129871759#section=2D-Structure
https://pubchem.ncbi.nlm.nih.gov/compound/129871724#section=2D-Structure
https://pubchem.ncbi.nlm.nih.gov/compound/129871721#section=2D-Structure
https://pubchem.ncbi.nlm.nih.gov/compound/129871600#section=2D-Structure
https://pubchem.ncbi.nlm.nih.gov/compound/129871358#section=2D-Structure
https://pubchem.ncbi.nlm.nih.gov/compound/129871329#section=2D-Structure
https://pubchem.ncbi.nlm.nih.gov/compound/129870705#section=2D-Structure
https://pubchem.ncbi.nlm.nih.gov/compound/129870703#section=2D-Structure
https://pubchem.ncbi.nlm.nih.gov/compound/129870641#section=2D-Structure
https://pubchem.ncbi.nlm.nih.gov/compound/129870266#section=2D-Structure
https://pubchem.ncbi.nlm.nih.gov/compound/129869970#section=2D-Structure
https://pubchem.ncbi.nlm.nih.gov/compound/129869175#section=2D-Structure
https://pubchem.ncbi.nlm.nih.gov/compound/129868240#section=2D-Structure
https://pubchem.ncbi.nlm.nih.gov/compound/129867754#section=2D-Structure
https://pubchem.ncbi.nlm.nih.gov/compound/129867708#section=2D-Structure
https://pubchem.ncbi.nlm.nih.gov/compound/129865027#section=2D-Structure
https://pubchem.ncbi.nlm.nih.gov/compound/129861656#section=2D-Structure
https://pubchem.ncbi.nlm.nih.gov/compound/129860689#section=2D-Structure
https://pubchem.ncbi.nlm.nih.gov/compound/129860292#section=2D-Structure
https://pubchem.ncbi.nlm.nih.gov/compound/129859151#section=2D-Structure
https://pubchem.ncbi.nlm.nih.gov/compound/129857954#section=2D-Structure
https://pubchem.ncbi.nlm.nih.gov/compound/129857662#section=2D-Structure
https://pubchem.ncbi.nlm.nih.gov/compound/129857652#section=2D-Structure
https://pubchem.ncbi.nlm.nih.gov/compound/129857253#section=2D-Structure
https://pubchem.ncbi.nlm.nih.gov/compound/129856559#section=2D-Structure
https://pubchem.ncbi.nlm.nih.gov/compound/129855761#section=2D-Structure
https://pubchem.ncbi.nlm.nih.gov/compound/129855698#section=2D-Structure
https://pubchem.ncbi.nlm.nih.gov/compound/129855575#section=2D-Structure
https://pubchem.ncbi.nlm.nih.gov/compound/129855567#section=2D-Structure
https://pubchem.ncbi.nlm.nih.gov/compound/129854420#section=2D-Structure
https://pubchem.ncbi.nlm.nih.gov/compound/129853340#section=2D-Structure
https://pubchem.ncbi.nlm.nih.gov/compound/129849900#section=2D-Structure

https://pubchem.ncbi.nlm.nih.gov/compound/129849247#section=2D-Structure
https://pubchem.ncbi.nlm.nih.gov/compound/129847854#section=2D-Structure
https://pubchem.ncbi.nlm.nih.gov/compound/129847736#section=2D-Structure
https://pubchem.ncbi.nlm.nih.gov/compound/129846568#section=2D-Structure
https://pubchem.ncbi.nlm.nih.gov/compound/129846138#section=2D-Structure
https://pubchem.ncbi.nlm.nih.gov/compound/129845974#section=2D-Structure
https://pubchem.ncbi.nlm.nih.gov/compound/129845465#section=2D-Structure
https://pubchem.ncbi.nlm.nih.gov/compound/129844163#section=2D-Structure
https://pubchem.ncbi.nlm.nih.gov/compound/129843769#section=2D-Structure
https://pubchem.ncbi.nlm.nih.gov/compound/129842570#section=2D-Structure
https://pubchem.ncbi.nlm.nih.gov/compound/129841554#section=2D-Structure
https://pubchem.ncbi.nlm.nih.gov/compound/129841047#section=2D-Structure
https://pubchem.ncbi.nlm.nih.gov/compound/129839622#section=2D-Structure
https://pubchem.ncbi.nlm.nih.gov/compound/129837945#section=2D-Structure
https://pubchem.ncbi.nlm.nih.gov/compound/129837777#section=2D-Structure
https://pubchem.ncbi.nlm.nih.gov/compound/129835603#section=2D-Structure
https://pubchem.ncbi.nlm.nih.gov/compound/129833930#section=2D-Structure
https://pubchem.ncbi.nlm.nih.gov/compound/129833724#section=2D-Structure
https://pubchem.ncbi.nlm.nih.gov/compound/129833227#section=2D-Structure
https://pubchem.ncbi.nlm.nih.gov/compound/129833101#section=2D-Structure
https://pubchem.ncbi.nlm.nih.gov/compound/129830124#section=2D-Structure
https://pubchem.ncbi.nlm.nih.gov/compound/129828941#section=2D-Structure
https://pubchem.ncbi.nlm.nih.gov/compound/129827254#section=2D-Structure
https://pubchem.ncbi.nlm.nih.gov/compound/129825276#section=2D-Structure
https://pubchem.ncbi.nlm.nih.gov/compound/129825219#section=2D-Structure
https://pubchem.ncbi.nlm.nih.gov/compound/129822681#section=2D-Structure
https://pubchem.ncbi.nlm.nih.gov/compound/129822007#section=2D-Structure
https://pubchem.ncbi.nlm.nih.gov/compound/129821968#section=2D-Structure
https://pubchem.ncbi.nlm.nih.gov/compound/129820836#section=2D-Structure
https://pubchem.ncbi.nlm.nih.gov/compound/129820537#section=2D-Structure
https://pubchem.ncbi.nlm.nih.gov/compound/129820520#section=2D-Structure
https://pubchem.ncbi.nlm.nih.gov/compound/129818499#section=2D-Structure
https://pubchem.ncbi.nlm.nih.gov/compound/129818204#section=2D-Structure
https://pubchem.ncbi.nlm.nih.gov/compound/129817778#section=2D-Structure
https://pubchem.ncbi.nlm.nih.gov/compound/129817628#section=2D-Structure
https://pubchem.ncbi.nlm.nih.gov/compound/129816615#section=2D-Structure
https://pubchem.ncbi.nlm.nih.gov/compound/129815725#section=2D-Structure
https://pubchem.ncbi.nlm.nih.gov/compound/129815360#section=2D-Structure
https://pubchem.ncbi.nlm.nih.gov/compound/129814243#section=2D-Structure
https://pubchem.ncbi.nlm.nih.gov/compound/129814179#section=2D-Structure
https://pubchem.ncbi.nlm.nih.gov/compound/129814064#section=2D-Structure
https://pubchem.ncbi.nlm.nih.gov/compound/129814015#section=2D-Structure
https://pubchem.ncbi.nlm.nih.gov/compound/129812844#section=2D-Structure
https://pubchem.ncbi.nlm.nih.gov/compound/129812685#section=2D-Structure
https://pubchem.ncbi.nlm.nih.gov/compound/129807252#section=2D-Structure
https://pubchem.ncbi.nlm.nih.gov/compound/129804767#section=2D-Structure
https://pubchem.ncbi.nlm.nih.gov/compound/129804675#section=2D-Structure
https://pubchem.ncbi.nlm.nih.gov/compound/129803970#section=2D-Structure
https://pubchem.ncbi.nlm.nih.gov/compound/129802986#section=2D-Structure
https://pubchem.ncbi.nlm.nih.gov/compound/129802512#section=2D-Structure
https://pubchem.ncbi.nlm.nih.gov/compound/129801689#section=2D-Structure
https://pubchem.ncbi.nlm.nih.gov/compound/129801544#section=2D-Structure
https://pubchem.ncbi.nlm.nih.gov/compound/129801262#section=2D-Structure
https://pubchem.ncbi.nlm.nih.gov/compound/129801084#section=2D-Structure
https://pubchem.ncbi.nlm.nih.gov/compound/129801074#section=2D-Structure

https://pubchem.ncbi.nlm.nih.gov/compound/129800884#section=2D-Structure
https://pubchem.ncbi.nlm.nih.gov/compound/129800475#section=2D-Structure
https://pubchem.ncbi.nlm.nih.gov/compound/129800462#section=2D-Structure
https://pubchem.ncbi.nlm.nih.gov/compound/129800319#section=2D-Structure
https://pubchem.ncbi.nlm.nih.gov/compound/129800206#section=2D-Structure
https://pubchem.ncbi.nlm.nih.gov/compound/129799612#section=2D-Structure
https://pubchem.ncbi.nlm.nih.gov/compound/129799516#section=2D-Structure
https://pubchem.ncbi.nlm.nih.gov/compound/129798837#section=2D-Structure
https://pubchem.ncbi.nlm.nih.gov/compound/129798713#section=2D-Structure
https://pubchem.ncbi.nlm.nih.gov/compound/129798149#section=2D-Structure
https://pubchem.ncbi.nlm.nih.gov/compound/129797965#section=2D-Structure
https://pubchem.ncbi.nlm.nih.gov/compound/129797594#section=2D-Structure
https://pubchem.ncbi.nlm.nih.gov/compound/129797431#section=2D-Structure
https://pubchem.ncbi.nlm.nih.gov/compound/129796689#section=2D-Structure
https://pubchem.ncbi.nlm.nih.gov/compound/129796269#section=2D-Structure
https://pubchem.ncbi.nlm.nih.gov/compound/129795837#section=2D-Structure
https://pubchem.ncbi.nlm.nih.gov/compound/129795820#section=2D-Structure
https://pubchem.ncbi.nlm.nih.gov/compound/129795716#section=2D-Structure
https://pubchem.ncbi.nlm.nih.gov/compound/129795648#section=2D-Structure
https://pubchem.ncbi.nlm.nih.gov/compound/129795449#section=2D-Structure
https://pubchem.ncbi.nlm.nih.gov/compound/129795189#section=2D-Structure
https://pubchem.ncbi.nlm.nih.gov/compound/129794362#section=2D-Structure
https://pubchem.ncbi.nlm.nih.gov/compound/129794301#section=2D-Structure
https://pubchem.ncbi.nlm.nih.gov/compound/129793622#section=2D-Structure
https://pubchem.ncbi.nlm.nih.gov/compound/129792941#section=2D-Structure
https://pubchem.ncbi.nlm.nih.gov/compound/129792744#section=2D-Structure
https://pubchem.ncbi.nlm.nih.gov/compound/129792282#section=2D-Structure
https://pubchem.ncbi.nlm.nih.gov/compound/129792132#section=2D-Structure
https://pubchem.ncbi.nlm.nih.gov/compound/129792064#section=2D-Structure
https://pubchem.ncbi.nlm.nih.gov/compound/129792028#section=2D-Structure
https://pubchem.ncbi.nlm.nih.gov/compound/129791817#section=2D-Structure
https://pubchem.ncbi.nlm.nih.gov/compound/129791796#section=2D-Structure
https://pubchem.ncbi.nlm.nih.gov/compound/129791665#section=2D-Structure
https://pubchem.ncbi.nlm.nih.gov/compound/129791329#section=2D-Structure
https://pubchem.ncbi.nlm.nih.gov/compound/129791285#section=2D-Structure
https://pubchem.ncbi.nlm.nih.gov/compound/129791217#section=2D-Structure
https://pubchem.ncbi.nlm.nih.gov/compound/129791103#section=2D-Structure
https://pubchem.ncbi.nlm.nih.gov/compound/129790524#section=2D-Structure
https://pubchem.ncbi.nlm.nih.gov/compound/129789792#section=2D-Structure
https://pubchem.ncbi.nlm.nih.gov/compound/129789706#section=2D-Structure
https://pubchem.ncbi.nlm.nih.gov/compound/129789649#section=2D-Structure
https://pubchem.ncbi.nlm.nih.gov/compound/129789015#section=2D-Structure
https://pubchem.ncbi.nlm.nih.gov/compound/129788932#section=2D-Structure
https://pubchem.ncbi.nlm.nih.gov/compound/129788545#section=2D-Structure
https://pubchem.ncbi.nlm.nih.gov/compound/129788257#section=2D-Structure
https://pubchem.ncbi.nlm.nih.gov/compound/129787996#section=2D-Structure
https://pubchem.ncbi.nlm.nih.gov/compound/129787891#section=2D-Structure
https://pubchem.ncbi.nlm.nih.gov/compound/129787739#section=2D-Structure
https://pubchem.ncbi.nlm.nih.gov/compound/129787498#section=2D-Structure
https://pubchem.ncbi.nlm.nih.gov/compound/129787341#section=2D-Structure
https://pubchem.ncbi.nlm.nih.gov/compound/129787240#section=2D-Structure
https://pubchem.ncbi.nlm.nih.gov/compound/129786945#section=2D-Structure
https://pubchem.ncbi.nlm.nih.gov/compound/129786893#section=2D-Structure
https://pubchem.ncbi.nlm.nih.gov/compound/129786556#section=2D-Structure
https://pubchem.ncbi.nlm.nih.gov/compound/129786525#section=2D-Structure

https://pubchem.ncbi.nlm.nih.gov/compound/129786427#section=2D-Structure
https://pubchem.ncbi.nlm.nih.gov/compound/129786356#section=2D-Structure
https://pubchem.ncbi.nlm.nih.gov/compound/129786179#section=2D-Structure
https://pubchem.ncbi.nlm.nih.gov/compound/129786159#section=2D-Structure
https://pubchem.ncbi.nlm.nih.gov/compound/129785990#section=2D-Structure
https://pubchem.ncbi.nlm.nih.gov/compound/129785943#section=2D-Structure
https://pubchem.ncbi.nlm.nih.gov/compound/129785520#section=2D-Structure
https://pubchem.ncbi.nlm.nih.gov/compound/129785364#section=2D-Structure
https://pubchem.ncbi.nlm.nih.gov/compound/129785284#section=2D-Structure
https://pubchem.ncbi.nlm.nih.gov/compound/129784910#section=2D-Structure
https://pubchem.ncbi.nlm.nih.gov/compound/129784652#section=2D-Structure
https://pubchem.ncbi.nlm.nih.gov/compound/129784592#section=2D-Structure
https://pubchem.ncbi.nlm.nih.gov/compound/129784440#section=2D-Structure
https://pubchem.ncbi.nlm.nih.gov/compound/129784401#section=2D-Structure
https://pubchem.ncbi.nlm.nih.gov/compound/129784004#section=2D-Structure
https://pubchem.ncbi.nlm.nih.gov/compound/129783051#section=2D-Structure
https://pubchem.ncbi.nlm.nih.gov/compound/129782927#section=2D-Structure
https://pubchem.ncbi.nlm.nih.gov/compound/129782523#section=2D-Structure
https://pubchem.ncbi.nlm.nih.gov/compound/129782488#section=2D-Structure
https://pubchem.ncbi.nlm.nih.gov/compound/129782033#section=2D-Structure
https://pubchem.ncbi.nlm.nih.gov/compound/129781892#section=2D-Structure
https://pubchem.ncbi.nlm.nih.gov/compound/129781783#section=2D-Structure
https://pubchem.ncbi.nlm.nih.gov/compound/129781551#section=2D-Structure
https://pubchem.ncbi.nlm.nih.gov/compound/129781463#section=2D-Structure
https://pubchem.ncbi.nlm.nih.gov/compound/129781435#section=2D-Structure
https://pubchem.ncbi.nlm.nih.gov/compound/129780729#section=2D-Structure
https://pubchem.ncbi.nlm.nih.gov/compound/129779498#section=2D-Structure
https://pubchem.ncbi.nlm.nih.gov/compound/129779304#section=2D-Structure
https://pubchem.ncbi.nlm.nih.gov/compound/129778212#section=2D-Structure
https://pubchem.ncbi.nlm.nih.gov/compound/129778175#section=2D-Structure
https://pubchem.ncbi.nlm.nih.gov/compound/129776532#section=2D-Structure
https://pubchem.ncbi.nlm.nih.gov/compound/129775373#section=2D-Structure
https://pubchem.ncbi.nlm.nih.gov/compound/129775166#section=2D-Structure
https://pubchem.ncbi.nlm.nih.gov/compound/129775067#section=2D-Structure
https://pubchem.ncbi.nlm.nih.gov/compound/129774543#section=2D-Structure
https://pubchem.ncbi.nlm.nih.gov/compound/129773555#section=2D-Structure
https://pubchem.ncbi.nlm.nih.gov/compound/129771259#section=2D-Structure
https://pubchem.ncbi.nlm.nih.gov/compound/129767390#section=2D-Structure
https://pubchem.ncbi.nlm.nih.gov/compound/129766731#section=2D-Structure
https://pubchem.ncbi.nlm.nih.gov/compound/129763916#section=2D-Structure
https://pubchem.ncbi.nlm.nih.gov/compound/129763877#section=2D-Structure
https://pubchem.ncbi.nlm.nih.gov/compound/129762518#section=2D-Structure
https://pubchem.ncbi.nlm.nih.gov/compound/129759925#section=2D-Structure
https://pubchem.ncbi.nlm.nih.gov/compound/129759255#section=2D-Structure
https://pubchem.ncbi.nlm.nih.gov/compound/129757121#section=2D-Structure
https://pubchem.ncbi.nlm.nih.gov/compound/129756125#section=2D-Structure
https://pubchem.ncbi.nlm.nih.gov/compound/129756115#section=2D-Structure
https://pubchem.ncbi.nlm.nih.gov/compound/129755995#section=2D-Structure
https://pubchem.ncbi.nlm.nih.gov/compound/129755666#section=2D-Structure
https://pubchem.ncbi.nlm.nih.gov/compound/129754946#section=2D-Structure
https://pubchem.ncbi.nlm.nih.gov/compound/129752382#section=2D-Structure
https://pubchem.ncbi.nlm.nih.gov/compound/129751957#section=2D-Structure
https://pubchem.ncbi.nlm.nih.gov/compound/129751533#section=2D-Structure
https://pubchem.ncbi.nlm.nih.gov/compound/129751106#section=2D-Structure
https://pubchem.ncbi.nlm.nih.gov/compound/129750029#section=2D-Structure

https://pubchem.ncbi.nlm.nih.gov/compound/129749992#section=2D-Structure
https://pubchem.ncbi.nlm.nih.gov/compound/129747628#section=2D-Structure
https://pubchem.ncbi.nlm.nih.gov/compound/129746796#section=2D-Structure
https://pubchem.ncbi.nlm.nih.gov/compound/129746778#section=2D-Structure
https://pubchem.ncbi.nlm.nih.gov/compound/129745695#section=2D-Structure
https://pubchem.ncbi.nlm.nih.gov/compound/129745638#section=2D-Structure
https://pubchem.ncbi.nlm.nih.gov/compound/129745056#section=2D-Structure
https://pubchem.ncbi.nlm.nih.gov/compound/129743575#section=2D-Structure
https://pubchem.ncbi.nlm.nih.gov/compound/129742536#section=2D-Structure
https://pubchem.ncbi.nlm.nih.gov/compound/129740670#section=2D-Structure
https://pubchem.ncbi.nlm.nih.gov/compound/129740633#section=2D-Structure
https://pubchem.ncbi.nlm.nih.gov/compound/129740447#section=2D-Structure
https://pubchem.ncbi.nlm.nih.gov/compound/129740153#section=2D-Structure
https://pubchem.ncbi.nlm.nih.gov/compound/129740116#section=2D-Structure
https://pubchem.ncbi.nlm.nih.gov/compound/129740103#section=2D-Structure
https://pubchem.ncbi.nlm.nih.gov/compound/129739984#section=2D-Structure
https://pubchem.ncbi.nlm.nih.gov/compound/129739783#section=2D-Structure
https://pubchem.ncbi.nlm.nih.gov/compound/129739771#section=2D-Structure
https://pubchem.ncbi.nlm.nih.gov/compound/129739566#section=2D-Structure
https://pubchem.ncbi.nlm.nih.gov/compound/129738816#section=2D-Structure
https://pubchem.ncbi.nlm.nih.gov/compound/129738609#section=2D-Structure
https://pubchem.ncbi.nlm.nih.gov/compound/129738336#section=2D-Structure
https://pubchem.ncbi.nlm.nih.gov/compound/129737805#section=2D-Structure
https://pubchem.ncbi.nlm.nih.gov/compound/129737768#section=2D-Structure
https://pubchem.ncbi.nlm.nih.gov/compound/129735590#section=2D-Structure
https://pubchem.ncbi.nlm.nih.gov/compound/129734948#section=2D-Structure
https://pubchem.ncbi.nlm.nih.gov/compound/129733237#section=2D-Structure
https://pubchem.ncbi.nlm.nih.gov/compound/129733217#section=2D-Structure
https://pubchem.ncbi.nlm.nih.gov/compound/129733207#section=2D-Structure
https://pubchem.ncbi.nlm.nih.gov/compound/129732871#section=2D-Structure
https://pubchem.ncbi.nlm.nih.gov/compound/129732496#section=2D-Structure
https://pubchem.ncbi.nlm.nih.gov/compound/129731811#section=2D-Structure
https://pubchem.ncbi.nlm.nih.gov/compound/129731290#section=2D-Structure
https://pubchem.ncbi.nlm.nih.gov/compound/129731053#section=2D-Structure
https://pubchem.ncbi.nlm.nih.gov/compound/129730068#section=2D-Structure
https://pubchem.ncbi.nlm.nih.gov/compound/129724149#section=2D-Structure
https://pubchem.ncbi.nlm.nih.gov/compound/129723681#section=2D-Structure
https://pubchem.ncbi.nlm.nih.gov/compound/129723028#section=2D-Structure
https://pubchem.ncbi.nlm.nih.gov/compound/129722969#section=2D-Structure
https://pubchem.ncbi.nlm.nih.gov/compound/129721093#section=2D-Structure
https://pubchem.ncbi.nlm.nih.gov/compound/129721092#section=2D-Structure
https://pubchem.ncbi.nlm.nih.gov/compound/129720979#section=2D-Structure
https://pubchem.ncbi.nlm.nih.gov/compound/129720382#section=2D-Structure
https://pubchem.ncbi.nlm.nih.gov/compound/129719875#section=2D-Structure
https://pubchem.ncbi.nlm.nih.gov/compound/129717567#section=2D-Structure
https://pubchem.ncbi.nlm.nih.gov/compound/129717322#section=2D-Structure
https://pubchem.ncbi.nlm.nih.gov/compound/129716798#section=2D-Structure
https://pubchem.ncbi.nlm.nih.gov/compound/129716713#section=2D-Structure
https://pubchem.ncbi.nlm.nih.gov/compound/129715348#section=2D-Structure
https://pubchem.ncbi.nlm.nih.gov/compound/129714471#section=2D-Structure
https://pubchem.ncbi.nlm.nih.gov/compound/129714445#section=2D-Structure
https://pubchem.ncbi.nlm.nih.gov/compound/129714341#section=2D-Structure
https://pubchem.ncbi.nlm.nih.gov/compound/129708869#section=2D-Structure
https://pubchem.ncbi.nlm.nih.gov/compound/129708655#section=2D-Structure
https://pubchem.ncbi.nlm.nih.gov/compound/129708292#section=2D-Structure

https://pubchem.ncbi.nlm.nih.gov/compound/129708220#section=2D-Structure
https://pubchem.ncbi.nlm.nih.gov/compound/129707845#section=2D-Structure
https://pubchem.ncbi.nlm.nih.gov/compound/129707698#section=2D-Structure
https://pubchem.ncbi.nlm.nih.gov/compound/129705976#section=2D-Structure
https://pubchem.ncbi.nlm.nih.gov/compound/129705545#section=2D-Structure
https://pubchem.ncbi.nlm.nih.gov/compound/129705544#section=2D-Structure
https://pubchem.ncbi.nlm.nih.gov/compound/129703877#section=2D-Structure
https://pubchem.ncbi.nlm.nih.gov/compound/129698357#section=2D-Structure
https://pubchem.ncbi.nlm.nih.gov/compound/129698289#section=2D-Structure
https://pubchem.ncbi.nlm.nih.gov/compound/129696828#section=2D-Structure
https://pubchem.ncbi.nlm.nih.gov/compound/129695822#section=2D-Structure
https://pubchem.ncbi.nlm.nih.gov/compound/129694578#section=2D-Structure
https://pubchem.ncbi.nlm.nih.gov/compound/129694296#section=2D-Structure
https://pubchem.ncbi.nlm.nih.gov/compound/129691340#section=2D-Structure
https://pubchem.ncbi.nlm.nih.gov/compound/129691003#section=2D-Structure
https://pubchem.ncbi.nlm.nih.gov/compound/129690747#section=2D-Structure
https://pubchem.ncbi.nlm.nih.gov/compound/129689007#section=2D-Structure
https://pubchem.ncbi.nlm.nih.gov/compound/129687166#section=2D-Structure
https://pubchem.ncbi.nlm.nih.gov/compound/129686597#section=2D-Structure
https://pubchem.ncbi.nlm.nih.gov/compound/129684775#section=2D-Structure
https://pubchem.ncbi.nlm.nih.gov/compound/129679712#section=2D-Structure
https://pubchem.ncbi.nlm.nih.gov/compound/129677584#section=2D-Structure
https://pubchem.ncbi.nlm.nih.gov/compound/129677230#section=2D-Structure
https://pubchem.ncbi.nlm.nih.gov/compound/129676842#section=2D-Structure
https://pubchem.ncbi.nlm.nih.gov/compound/129676829#section=2D-Structure
https://pubchem.ncbi.nlm.nih.gov/compound/129674180#section=2D-Structure
https://pubchem.ncbi.nlm.nih.gov/compound/129674066#section=2D-Structure
https://pubchem.ncbi.nlm.nih.gov/compound/129673801#section=2D-Structure
https://pubchem.ncbi.nlm.nih.gov/compound/129673781#section=2D-Structure
https://pubchem.ncbi.nlm.nih.gov/compound/129673235#section=2D-Structure
https://pubchem.ncbi.nlm.nih.gov/compound/129672956#section=2D-Structure
https://pubchem.ncbi.nlm.nih.gov/compound/129671881#section=2D-Structure
https://pubchem.ncbi.nlm.nih.gov/compound/129669701#section=2D-Structure
https://pubchem.ncbi.nlm.nih.gov/compound/129669155#section=2D-Structure
https://pubchem.ncbi.nlm.nih.gov/compound/129664052#section=2D-Structure
https://pubchem.ncbi.nlm.nih.gov/compound/129656001#section=2D-Structure
https://pubchem.ncbi.nlm.nih.gov/compound/129654675#section=2D-Structure
https://pubchem.ncbi.nlm.nih.gov/compound/129652472#section=2D-Structure
https://pubchem.ncbi.nlm.nih.gov/compound/129650651#section=2D-Structure
https://pubchem.ncbi.nlm.nih.gov/compound/129649254#section=2D-Structure
https://pubchem.ncbi.nlm.nih.gov/compound/129648869#section=2D-Structure
https://pubchem.ncbi.nlm.nih.gov/compound/129648655#section=2D-Structure
https://pubchem.ncbi.nlm.nih.gov/compound/129648194#section=2D-Structure
https://pubchem.ncbi.nlm.nih.gov/compound/129646580#section=2D-Structure
https://pubchem.ncbi.nlm.nih.gov/compound/129642394#section=2D-Structure
https://pubchem.ncbi.nlm.nih.gov/compound/129642087#section=2D-Structure
https://pubchem.ncbi.nlm.nih.gov/compound/129641318#section=2D-Structure
https://pubchem.ncbi.nlm.nih.gov/compound/129640004#section=2D-Structure
https://pubchem.ncbi.nlm.nih.gov/compound/129639704#section=2D-Structure
https://pubchem.ncbi.nlm.nih.gov/compound/129637932#section=2D-Structure
https://pubchem.ncbi.nlm.nih.gov/compound/129636703#section=2D-Structure
https://pubchem.ncbi.nlm.nih.gov/compound/129634950#section=2D-Structure
https://pubchem.ncbi.nlm.nih.gov/compound/129634711#section=2D-Structure
https://pubchem.ncbi.nlm.nih.gov/compound/129634620#section=2D-Structure
https://pubchem.ncbi.nlm.nih.gov/compound/129634365#section=2D-Structure

https://pubchem.ncbi.nlm.nih.gov/compound/129633639#section=2D-Structure
https://pubchem.ncbi.nlm.nih.gov/compound/12963293#section=2D-Structure
https://pubchem.ncbi.nlm.nih.gov/compound/129632354#section=2D-Structure
https://pubchem.ncbi.nlm.nih.gov/compound/129632286#section=2D-Structure
https://pubchem.ncbi.nlm.nih.gov/compound/129632115#section=2D-Structure
https://pubchem.ncbi.nlm.nih.gov/compound/129631905#section=2D-Structure
https://pubchem.ncbi.nlm.nih.gov/compound/129630859#section=2D-Structure
https://pubchem.ncbi.nlm.nih.gov/compound/129629936#section=2D-Structure
https://pubchem.ncbi.nlm.nih.gov/compound/129629584#section=2D-Structure
https://pubchem.ncbi.nlm.nih.gov/compound/129629580#section=2D-Structure
https://pubchem.ncbi.nlm.nih.gov/compound/129628903#section=2D-Structure
https://pubchem.ncbi.nlm.nih.gov/compound/129628872#section=2D-Structure
https://pubchem.ncbi.nlm.nih.gov/compound/129628745#section=2D-Structure
https://pubchem.ncbi.nlm.nih.gov/compound/129628693#section=2D-Structure
https://pubchem.ncbi.nlm.nih.gov/compound/129628669#section=2D-Structure
https://pubchem.ncbi.nlm.nih.gov/compound/129628662#section=2D-Structure
https://pubchem.ncbi.nlm.nih.gov/compound/129301257#section=2D-Structure
https://pubchem.ncbi.nlm.nih.gov/compound/129286373#section=2D-Structure
https://pubchem.ncbi.nlm.nih.gov/compound/129268922#section=2D-Structure
https://pubchem.ncbi.nlm.nih.gov/compound/129267981#section=2D-Structure
https://pubchem.ncbi.nlm.nih.gov/compound/129267978#section=2D-Structure
https://pubchem.ncbi.nlm.nih.gov/compound/129267862#section=2D-Structure
https://pubchem.ncbi.nlm.nih.gov/compound/129267856#section=2D-Structure
https://pubchem.ncbi.nlm.nih.gov/compound/129238341#section=2D-Structure
https://pubchem.ncbi.nlm.nih.gov/compound/129236732#section=2D-Structure
https://pubchem.ncbi.nlm.nih.gov/compound/129185056#section=2D-Structure
https://pubchem.ncbi.nlm.nih.gov/compound/129176187#section=2D-Structure
https://pubchem.ncbi.nlm.nih.gov/compound/129174545#section=2D-Structure
https://pubchem.ncbi.nlm.nih.gov/compound/129172898#section=2D-Structure
https://pubchem.ncbi.nlm.nih.gov/compound/129172849#section=2D-Structure
https://pubchem.ncbi.nlm.nih.gov/compound/129172792#section=2D-Structure
https://pubchem.ncbi.nlm.nih.gov/compound/129156173#section=2D-Structure
https://pubchem.ncbi.nlm.nih.gov/compound/129137197#section=2D-Structure
https://pubchem.ncbi.nlm.nih.gov/compound/129136793#section=2D-Structure
https://pubchem.ncbi.nlm.nih.gov/compound/129136681#section=2D-Structure
https://pubchem.ncbi.nlm.nih.gov/compound/129136505#section=2D-Structure
https://pubchem.ncbi.nlm.nih.gov/compound/129096310#section=2D-Structure
https://pubchem.ncbi.nlm.nih.gov/compound/129092233#section=2D-Structure
https://pubchem.ncbi.nlm.nih.gov/compound/129092232#section=2D-Structure
https://pubchem.ncbi.nlm.nih.gov/compound/129092230#section=2D-Structure
https://pubchem.ncbi.nlm.nih.gov/compound/129092228#section=2D-Structure
https://pubchem.ncbi.nlm.nih.gov/compound/129073713#section=2D-Structure
https://pubchem.ncbi.nlm.nih.gov/compound/129053525#section=2D-Structure
https://pubchem.ncbi.nlm.nih.gov/compound/12904432#section=2D-Structure
https://pubchem.ncbi.nlm.nih.gov/compound/12902518#section=2D-Structure
https://pubchem.ncbi.nlm.nih.gov/compound/12886698#section=2D-Structure
https://pubchem.ncbi.nlm.nih.gov/compound/128666#section=2D-Structure
https://pubchem.ncbi.nlm.nih.gov/compound/128665#section=2D-Structure
https://pubchem.ncbi.nlm.nih.gov/compound/12858754#section=2D-Structure
https://pubchem.ncbi.nlm.nih.gov/compound/12858750#section=2D-Structure
https://pubchem.ncbi.nlm.nih.gov/compound/12858739#section=2D-Structure
https://pubchem.ncbi.nlm.nih.gov/compound/12841864#section=2D-Structure
https://pubchem.ncbi.nlm.nih.gov/compound/12836508#section=2D-Structure
https://pubchem.ncbi.nlm.nih.gov/compound/12820772#section=2D-Structure
https://pubchem.ncbi.nlm.nih.gov/compound/12820463#section=2D-Structure

https://pubchem.ncbi.nlm.nih.gov/compound/12748935#section=2D-Structure
https://pubchem.ncbi.nlm.nih.gov/compound/12748932#section=2D-Structure
https://pubchem.ncbi.nlm.nih.gov/compound/12748929#section=2D-Structure
https://pubchem.ncbi.nlm.nih.gov/compound/12743735#section=2D-Structure
https://pubchem.ncbi.nlm.nih.gov/compound/12733648#section=2D-Structure
https://pubchem.ncbi.nlm.nih.gov/compound/12733638#section=2D-Structure
https://pubchem.ncbi.nlm.nih.gov/compound/12731233#section=2D-Structure
https://pubchem.ncbi.nlm.nih.gov/compound/12731226#section=2D-Structure
https://pubchem.ncbi.nlm.nih.gov/compound/127094#section=2D-Structure
https://pubchem.ncbi.nlm.nih.gov/compound/12709066#section=2D-Structure
https://pubchem.ncbi.nlm.nih.gov/compound/126848#section=2D-Structure
https://pubchem.ncbi.nlm.nih.gov/compound/126740820#section=2D-Structure
https://pubchem.ncbi.nlm.nih.gov/compound/126736280#section=2D-Structure
https://pubchem.ncbi.nlm.nih.gov/compound/126709677#section=2D-Structure
https://pubchem.ncbi.nlm.nih.gov/compound/126709676#section=2D-Structure
https://pubchem.ncbi.nlm.nih.gov/compound/126705444#section=2D-Structure
https://pubchem.ncbi.nlm.nih.gov/compound/126705436#section=2D-Structure
https://pubchem.ncbi.nlm.nih.gov/compound/126682585#section=2D-Structure
https://pubchem.ncbi.nlm.nih.gov/compound/12667399#section=2D-Structure
https://pubchem.ncbi.nlm.nih.gov/compound/12667390#section=2D-Structure
https://pubchem.ncbi.nlm.nih.gov/compound/126666500#section=2D-Structure
https://pubchem.ncbi.nlm.nih.gov/compound/126666495#section=2D-Structure
https://pubchem.ncbi.nlm.nih.gov/compound/126666489#section=2D-Structure
https://pubchem.ncbi.nlm.nih.gov/compound/126666467#section=2D-Structure
https://pubchem.ncbi.nlm.nih.gov/compound/126656465#section=2D-Structure
https://pubchem.ncbi.nlm.nih.gov/compound/126646777#section=2D-Structure
https://pubchem.ncbi.nlm.nih.gov/compound/126644091#section=2D-Structure
https://pubchem.ncbi.nlm.nih.gov/compound/126635934#section=2D-Structure
https://pubchem.ncbi.nlm.nih.gov/compound/126634993#section=2D-Structure
https://pubchem.ncbi.nlm.nih.gov/compound/126606634#section=2D-Structure
https://pubchem.ncbi.nlm.nih.gov/compound/126585513#section=2D-Structure
https://pubchem.ncbi.nlm.nih.gov/compound/126585512#section=2D-Structure
https://pubchem.ncbi.nlm.nih.gov/compound/12654469#section=2D-Structure
https://pubchem.ncbi.nlm.nih.gov/compound/126521762#section=2D-Structure
https://pubchem.ncbi.nlm.nih.gov/compound/126515176#section=2D-Structure
https://pubchem.ncbi.nlm.nih.gov/compound/126500993#section=2D-Structure
https://pubchem.ncbi.nlm.nih.gov/compound/126500988#section=2D-Structure
https://pubchem.ncbi.nlm.nih.gov/compound/126500981#section=2D-Structure
https://pubchem.ncbi.nlm.nih.gov/compound/126500968#section=2D-Structure
https://pubchem.ncbi.nlm.nih.gov/compound/126500#section=2D-Structure
https://pubchem.ncbi.nlm.nih.gov/compound/126491889#section=2D-Structure
https://pubchem.ncbi.nlm.nih.gov/compound/12634708#section=2D-Structure
https://pubchem.ncbi.nlm.nih.gov/compound/12631814#section=2D-Structure
https://pubchem.ncbi.nlm.nih.gov/compound/12628768#section=2D-Structure
https://pubchem.ncbi.nlm.nih.gov/compound/126245#section=2D-Structure
https://pubchem.ncbi.nlm.nih.gov/compound/126189#section=2D-Structure
https://pubchem.ncbi.nlm.nih.gov/compound/12617916#section=2D-Structure
https://pubchem.ncbi.nlm.nih.gov/compound/12612208#section=2D-Structure
https://pubchem.ncbi.nlm.nih.gov/compound/12605#section=2D-Structure
https://pubchem.ncbi.nlm.nih.gov/compound/12596493#section=2D-Structure
https://pubchem.ncbi.nlm.nih.gov/compound/12586670#section=2D-Structure
https://pubchem.ncbi.nlm.nih.gov/compound/12586665#section=2D-Structure
https://pubchem.ncbi.nlm.nih.gov/compound/12571581#section=2D-Structure
https://pubchem.ncbi.nlm.nih.gov/compound/12571578#section=2D-Structure
https://pubchem.ncbi.nlm.nih.gov/compound/12553000#section=2D-Structure

https://pubchem.ncbi.nlm.nih.gov/compound/125502692#section=2D-Structure
https://pubchem.ncbi.nlm.nih.gov/compound/125495474#section=2D-Structure
https://pubchem.ncbi.nlm.nih.gov/compound/125495460#section=2D-Structure
https://pubchem.ncbi.nlm.nih.gov/compound/125494884#section=2D-Structure
https://pubchem.ncbi.nlm.nih.gov/compound/125481272#section=2D-Structure
https://pubchem.ncbi.nlm.nih.gov/compound/125480832#section=2D-Structure
https://pubchem.ncbi.nlm.nih.gov/compound/125477902#section=2D-Structure
https://pubchem.ncbi.nlm.nih.gov/compound/12547701#section=2D-Structure
https://pubchem.ncbi.nlm.nih.gov/compound/125473974#section=2D-Structure
https://pubchem.ncbi.nlm.nih.gov/compound/125473971#section=2D-Structure
https://pubchem.ncbi.nlm.nih.gov/compound/125467900#section=2D-Structure
https://pubchem.ncbi.nlm.nih.gov/compound/125464497#section=2D-Structure
https://pubchem.ncbi.nlm.nih.gov/compound/12544557#section=2D-Structure
https://pubchem.ncbi.nlm.nih.gov/compound/125394#section=2D-Structure
https://pubchem.ncbi.nlm.nih.gov/compound/12526418#section=2D-Structure
https://pubchem.ncbi.nlm.nih.gov/compound/12525061#section=2D-Structure
https://pubchem.ncbi.nlm.nih.gov/compound/12519097#section=2D-Structure
https://pubchem.ncbi.nlm.nih.gov/compound/12502024#section=2D-Structure
https://pubchem.ncbi.nlm.nih.gov/compound/12502023#section=2D-Structure
https://pubchem.ncbi.nlm.nih.gov/compound/12502022#section=2D-Structure
https://pubchem.ncbi.nlm.nih.gov/compound/12502021#section=2D-Structure
https://pubchem.ncbi.nlm.nih.gov/compound/12498157#section=2D-Structure
https://pubchem.ncbi.nlm.nih.gov/compound/12490807#section=2D-Structure
https://pubchem.ncbi.nlm.nih.gov/compound/124834#section=2D-Structure
https://pubchem.ncbi.nlm.nih.gov/compound/12482157#section=2D-Structure
https://pubchem.ncbi.nlm.nih.gov/compound/12479994#section=2D-Structure
https://pubchem.ncbi.nlm.nih.gov/compound/124733#section=2D-Structure
https://pubchem.ncbi.nlm.nih.gov/compound/124722#section=2D-Structure
https://pubchem.ncbi.nlm.nih.gov/compound/12460123#section=2D-Structure
https://pubchem.ncbi.nlm.nih.gov/compound/12459799#section=2D-Structure
https://pubchem.ncbi.nlm.nih.gov/compound/12451498#section=2D-Structure
https://pubchem.ncbi.nlm.nih.gov/compound/12440963#section=2D-Structure
https://pubchem.ncbi.nlm.nih.gov/compound/12436253#section=2D-Structure
https://pubchem.ncbi.nlm.nih.gov/compound/12435805#section=2D-Structure
https://pubchem.ncbi.nlm.nih.gov/compound/12430542#section=2D-Structure
https://pubchem.ncbi.nlm.nih.gov/compound/12424448#section=2D-Structure
https://pubchem.ncbi.nlm.nih.gov/compound/12423164#section=2D-Structure
https://pubchem.ncbi.nlm.nih.gov/compound/124196257#section=2D-Structure
https://pubchem.ncbi.nlm.nih.gov/compound/124193402#section=2D-Structure
https://pubchem.ncbi.nlm.nih.gov/compound/124192904#section=2D-Structure
https://pubchem.ncbi.nlm.nih.gov/compound/124183632#section=2D-Structure
https://pubchem.ncbi.nlm.nih.gov/compound/124155591#section=2D-Structure
https://pubchem.ncbi.nlm.nih.gov/compound/124122087#section=2D-Structure
https://pubchem.ncbi.nlm.nih.gov/compound/124116380#section=2D-Structure
https://pubchem.ncbi.nlm.nih.gov/compound/12410131#section=2D-Structure
https://pubchem.ncbi.nlm.nih.gov/compound/124098411#section=2D-Structure
https://pubchem.ncbi.nlm.nih.gov/compound/12409753#section=2D-Structure
https://pubchem.ncbi.nlm.nih.gov/compound/124096562#section=2D-Structure
https://pubchem.ncbi.nlm.nih.gov/compound/124096544#section=2D-Structure
https://pubchem.ncbi.nlm.nih.gov/compound/124096542#section=2D-Structure
https://pubchem.ncbi.nlm.nih.gov/compound/124096535#section=2D-Structure
https://pubchem.ncbi.nlm.nih.gov/compound/124096534#section=2D-Structure
https://pubchem.ncbi.nlm.nih.gov/compound/124096530#section=2D-Structure
https://pubchem.ncbi.nlm.nih.gov/compound/124096529#section=2D-Structure
https://pubchem.ncbi.nlm.nih.gov/compound/124031685#section=2D-Structure

https://pubchem.ncbi.nlm.nih.gov/compound/123995266#section=2D-Structure
https://pubchem.ncbi.nlm.nih.gov/compound/123986741#section=2D-Structure
https://pubchem.ncbi.nlm.nih.gov/compound/123982406#section=2D-Structure
https://pubchem.ncbi.nlm.nih.gov/compound/123973676#section=2D-Structure
https://pubchem.ncbi.nlm.nih.gov/compound/123968599#section=2D-Structure
https://pubchem.ncbi.nlm.nih.gov/compound/123965393#section=2D-Structure
https://pubchem.ncbi.nlm.nih.gov/compound/123962284#section=2D-Structure
https://pubchem.ncbi.nlm.nih.gov/compound/123943168#section=2D-Structure
https://pubchem.ncbi.nlm.nih.gov/compound/123938641#section=2D-Structure
https://pubchem.ncbi.nlm.nih.gov/compound/123937144#section=2D-Structure
https://pubchem.ncbi.nlm.nih.gov/compound/123932274#section=2D-Structure
https://pubchem.ncbi.nlm.nih.gov/compound/123931700#section=2D-Structure
https://pubchem.ncbi.nlm.nih.gov/compound/123929196#section=2D-Structure
https://pubchem.ncbi.nlm.nih.gov/compound/12392611#section=2D-Structure
https://pubchem.ncbi.nlm.nih.gov/compound/123925051#section=2D-Structure
https://pubchem.ncbi.nlm.nih.gov/compound/123924790#section=2D-Structure
https://pubchem.ncbi.nlm.nih.gov/compound/123914089#section=2D-Structure
https://pubchem.ncbi.nlm.nih.gov/compound/123902300#section=2D-Structure
https://pubchem.ncbi.nlm.nih.gov/compound/123901990#section=2D-Structure
https://pubchem.ncbi.nlm.nih.gov/compound/123878771#section=2D-Structure
https://pubchem.ncbi.nlm.nih.gov/compound/123874426#section=2D-Structure
https://pubchem.ncbi.nlm.nih.gov/compound/123866151#section=2D-Structure
https://pubchem.ncbi.nlm.nih.gov/compound/123858203#section=2D-Structure
https://pubchem.ncbi.nlm.nih.gov/compound/123854694#section=2D-Structure
https://pubchem.ncbi.nlm.nih.gov/compound/123845284#section=2D-Structure
https://pubchem.ncbi.nlm.nih.gov/compound/123842604#section=2D-Structure
https://pubchem.ncbi.nlm.nih.gov/compound/123831489#section=2D-Structure
https://pubchem.ncbi.nlm.nih.gov/compound/123825014#section=2D-Structure
https://pubchem.ncbi.nlm.nih.gov/compound/123820922#section=2D-Structure
https://pubchem.ncbi.nlm.nih.gov/compound/123812559#section=2D-Structure
https://pubchem.ncbi.nlm.nih.gov/compound/123804260#section=2D-Structure
https://pubchem.ncbi.nlm.nih.gov/compound/123802668#section=2D-Structure
https://pubchem.ncbi.nlm.nih.gov/compound/123773299#section=2D-Structure
https://pubchem.ncbi.nlm.nih.gov/compound/123771553#section=2D-Structure
https://pubchem.ncbi.nlm.nih.gov/compound/123763660#section=2D-Structure
https://pubchem.ncbi.nlm.nih.gov/compound/123744266#section=2D-Structure
https://pubchem.ncbi.nlm.nih.gov/compound/123744159#section=2D-Structure
https://pubchem.ncbi.nlm.nih.gov/compound/123742668#section=2D-Structure
https://pubchem.ncbi.nlm.nih.gov/compound/123742494#section=2D-Structure
https://pubchem.ncbi.nlm.nih.gov/compound/123742297#section=2D-Structure
https://pubchem.ncbi.nlm.nih.gov/compound/123727152#section=2D-Structure
https://pubchem.ncbi.nlm.nih.gov/compound/123722683#section=2D-Structure
https://pubchem.ncbi.nlm.nih.gov/compound/123720684#section=2D-Structure
https://pubchem.ncbi.nlm.nih.gov/compound/123717118#section=2D-Structure
https://pubchem.ncbi.nlm.nih.gov/compound/123711328#section=2D-Structure
https://pubchem.ncbi.nlm.nih.gov/compound/123700409#section=2D-Structure
https://pubchem.ncbi.nlm.nih.gov/compound/123697017#section=2D-Structure
https://pubchem.ncbi.nlm.nih.gov/compound/12369460#section=2D-Structure
https://pubchem.ncbi.nlm.nih.gov/compound/123694224#section=2D-Structure
https://pubchem.ncbi.nlm.nih.gov/compound/123693139#section=2D-Structure
https://pubchem.ncbi.nlm.nih.gov/compound/12369252#section=2D-Structure
https://pubchem.ncbi.nlm.nih.gov/compound/123689737#section=2D-Structure
https://pubchem.ncbi.nlm.nih.gov/compound/123688828#section=2D-Structure
https://pubchem.ncbi.nlm.nih.gov/compound/123670146#section=2D-Structure
https://pubchem.ncbi.nlm.nih.gov/compound/123667488#section=2D-Structure

https://pubchem.ncbi.nlm.nih.gov/compound/123665032#section=2D-Structure
https://pubchem.ncbi.nlm.nih.gov/compound/123656635#section=2D-Structure
https://pubchem.ncbi.nlm.nih.gov/compound/123655048#section=2D-Structure
https://pubchem.ncbi.nlm.nih.gov/compound/123653932#section=2D-Structure
https://pubchem.ncbi.nlm.nih.gov/compound/12365247#section=2D-Structure
https://pubchem.ncbi.nlm.nih.gov/compound/123647382#section=2D-Structure
https://pubchem.ncbi.nlm.nih.gov/compound/123622594#section=2D-Structure
https://pubchem.ncbi.nlm.nih.gov/compound/123619979#section=2D-Structure
https://pubchem.ncbi.nlm.nih.gov/compound/12360436#section=2D-Structure
https://pubchem.ncbi.nlm.nih.gov/compound/123599010#section=2D-Structure
https://pubchem.ncbi.nlm.nih.gov/compound/123598353#section=2D-Structure
https://pubchem.ncbi.nlm.nih.gov/compound/123597930#section=2D-Structure
https://pubchem.ncbi.nlm.nih.gov/compound/123594947#section=2D-Structure
https://pubchem.ncbi.nlm.nih.gov/compound/123584476#section=2D-Structure
https://pubchem.ncbi.nlm.nih.gov/compound/123582314#section=2D-Structure
https://pubchem.ncbi.nlm.nih.gov/compound/123548625#section=2D-Structure
https://pubchem.ncbi.nlm.nih.gov/compound/123547598#section=2D-Structure
https://pubchem.ncbi.nlm.nih.gov/compound/123545421#section=2D-Structure
https://pubchem.ncbi.nlm.nih.gov/compound/123544012#section=2D-Structure
https://pubchem.ncbi.nlm.nih.gov/compound/12354255#section=2D-Structure
https://pubchem.ncbi.nlm.nih.gov/compound/123536959#section=2D-Structure
https://pubchem.ncbi.nlm.nih.gov/compound/123528818#section=2D-Structure
https://pubchem.ncbi.nlm.nih.gov/compound/123522657#section=2D-Structure
https://pubchem.ncbi.nlm.nih.gov/compound/123512234#section=2D-Structure
https://pubchem.ncbi.nlm.nih.gov/compound/123511661#section=2D-Structure
https://pubchem.ncbi.nlm.nih.gov/compound/12350802#section=2D-Structure
https://pubchem.ncbi.nlm.nih.gov/compound/12350801#section=2D-Structure
https://pubchem.ncbi.nlm.nih.gov/compound/123499520#section=2D-Structure
https://pubchem.ncbi.nlm.nih.gov/compound/123497088#section=2D-Structure
https://pubchem.ncbi.nlm.nih.gov/compound/123491308#section=2D-Structure
https://pubchem.ncbi.nlm.nih.gov/compound/123487015#section=2D-Structure
https://pubchem.ncbi.nlm.nih.gov/compound/123475523#section=2D-Structure
https://pubchem.ncbi.nlm.nih.gov/compound/123472258#section=2D-Structure
https://pubchem.ncbi.nlm.nih.gov/compound/123457963#section=2D-Structure
https://pubchem.ncbi.nlm.nih.gov/compound/123452757#section=2D-Structure
https://pubchem.ncbi.nlm.nih.gov/compound/123452365#section=2D-Structure
https://pubchem.ncbi.nlm.nih.gov/compound/123444540#section=2D-Structure
https://pubchem.ncbi.nlm.nih.gov/compound/12343961#section=2D-Structure
https://pubchem.ncbi.nlm.nih.gov/compound/123428423#section=2D-Structure
https://pubchem.ncbi.nlm.nih.gov/compound/123412012#section=2D-Structure
https://pubchem.ncbi.nlm.nih.gov/compound/123405113#section=2D-Structure
https://pubchem.ncbi.nlm.nih.gov/compound/123395912#section=2D-Structure
https://pubchem.ncbi.nlm.nih.gov/compound/123385559#section=2D-Structure
https://pubchem.ncbi.nlm.nih.gov/compound/123359308#section=2D-Structure
https://pubchem.ncbi.nlm.nih.gov/compound/123356893#section=2D-Structure
https://pubchem.ncbi.nlm.nih.gov/compound/123346470#section=2D-Structure
https://pubchem.ncbi.nlm.nih.gov/compound/123346114#section=2D-Structure
https://pubchem.ncbi.nlm.nih.gov/compound/12334577#section=2D-Structure
https://pubchem.ncbi.nlm.nih.gov/compound/123341729#section=2D-Structure
https://pubchem.ncbi.nlm.nih.gov/compound/123340750#section=2D-Structure
https://pubchem.ncbi.nlm.nih.gov/compound/12333737#section=2D-Structure
https://pubchem.ncbi.nlm.nih.gov/compound/12333736#section=2D-Structure
https://pubchem.ncbi.nlm.nih.gov/compound/12333735#section=2D-Structure
https://pubchem.ncbi.nlm.nih.gov/compound/123332478#section=2D-Structure
https://pubchem.ncbi.nlm.nih.gov/compound/123326669#section=2D-Structure

https://pubchem.ncbi.nlm.nih.gov/compound/123321471#section=2D-Structure
https://pubchem.ncbi.nlm.nih.gov/compound/123311168#section=2D-Structure
https://pubchem.ncbi.nlm.nih.gov/compound/123304402#section=2D-Structure
https://pubchem.ncbi.nlm.nih.gov/compound/123303605#section=2D-Structure
https://pubchem.ncbi.nlm.nih.gov/compound/123297409#section=2D-Structure
https://pubchem.ncbi.nlm.nih.gov/compound/123292196#section=2D-Structure
https://pubchem.ncbi.nlm.nih.gov/compound/123284031#section=2D-Structure
https://pubchem.ncbi.nlm.nih.gov/compound/123280994#section=2D-Structure
https://pubchem.ncbi.nlm.nih.gov/compound/123280462#section=2D-Structure
https://pubchem.ncbi.nlm.nih.gov/compound/123269075#section=2D-Structure
https://pubchem.ncbi.nlm.nih.gov/compound/123238505#section=2D-Structure
https://pubchem.ncbi.nlm.nih.gov/compound/123229518#section=2D-Structure
https://pubchem.ncbi.nlm.nih.gov/compound/123210237#section=2D-Structure
https://pubchem.ncbi.nlm.nih.gov/compound/123185271#section=2D-Structure
https://pubchem.ncbi.nlm.nih.gov/compound/123179200#section=2D-Structure
https://pubchem.ncbi.nlm.nih.gov/compound/123177135#section=2D-Structure
https://pubchem.ncbi.nlm.nih.gov/compound/123176049#section=2D-Structure
https://pubchem.ncbi.nlm.nih.gov/compound/12317102#section=2D-Structure
https://pubchem.ncbi.nlm.nih.gov/compound/123169904#section=2D-Structure
https://pubchem.ncbi.nlm.nih.gov/compound/123153821#section=2D-Structure
https://pubchem.ncbi.nlm.nih.gov/compound/12314797#section=2D-Structure
https://pubchem.ncbi.nlm.nih.gov/compound/123147156#section=2D-Structure
https://pubchem.ncbi.nlm.nih.gov/compound/12313103#section=2D-Structure
https://pubchem.ncbi.nlm.nih.gov/compound/12309611#section=2D-Structure
https://pubchem.ncbi.nlm.nih.gov/compound/123077#section=2D-Structure
https://pubchem.ncbi.nlm.nih.gov/compound/12307103#section=2D-Structure
https://pubchem.ncbi.nlm.nih.gov/compound/12303760#section=2D-Structure
https://pubchem.ncbi.nlm.nih.gov/compound/12303758#section=2D-Structure
https://pubchem.ncbi.nlm.nih.gov/compound/12301746#section=2D-Structure
https://pubchem.ncbi.nlm.nih.gov/compound/12295638#section=2D-Structure
https://pubchem.ncbi.nlm.nih.gov/compound/12295637#section=2D-Structure
https://pubchem.ncbi.nlm.nih.gov/compound/12295636#section=2D-Structure
https://pubchem.ncbi.nlm.nih.gov/compound/12295635#section=2D-Structure
https://pubchem.ncbi.nlm.nih.gov/compound/12292862#section=2D-Structure
https://pubchem.ncbi.nlm.nih.gov/compound/12292410#section=2D-Structure
https://pubchem.ncbi.nlm.nih.gov/compound/12278629#section=2D-Structure
https://pubchem.ncbi.nlm.nih.gov/compound/12278628#section=2D-Structure
https://pubchem.ncbi.nlm.nih.gov/compound/12278627#section=2D-Structure
https://pubchem.ncbi.nlm.nih.gov/compound/12278626#section=2D-Structure
https://pubchem.ncbi.nlm.nih.gov/compound/12265070#section=2D-Structure
https://pubchem.ncbi.nlm.nih.gov/compound/12260950#section=2D-Structure
https://pubchem.ncbi.nlm.nih.gov/compound/122555451#section=2D-Structure
https://pubchem.ncbi.nlm.nih.gov/compound/122555432#section=2D-Structure
https://pubchem.ncbi.nlm.nih.gov/compound/122545984#section=2D-Structure
https://pubchem.ncbi.nlm.nih.gov/compound/122545979#section=2D-Structure
https://pubchem.ncbi.nlm.nih.gov/compound/122543874#section=2D-Structure
https://pubchem.ncbi.nlm.nih.gov/compound/122508445#section=2D-Structure
https://pubchem.ncbi.nlm.nih.gov/compound/122486250#section=2D-Structure
https://pubchem.ncbi.nlm.nih.gov/compound/122448821#section=2D-Structure
https://pubchem.ncbi.nlm.nih.gov/compound/122448813#section=2D-Structure
https://pubchem.ncbi.nlm.nih.gov/compound/122398527#section=2D-Structure
https://pubchem.ncbi.nlm.nih.gov/compound/122392054#section=2D-Structure
https://pubchem.ncbi.nlm.nih.gov/compound/122384632#section=2D-Structure
https://pubchem.ncbi.nlm.nih.gov/compound/122381799#section=2D-Structure
https://pubchem.ncbi.nlm.nih.gov/compound/122381797#section=2D-Structure

https://pubchem.ncbi.nlm.nih.gov/compound/122375454#section=2D-Structure
https://pubchem.ncbi.nlm.nih.gov/compound/122375453#section=2D-Structure
https://pubchem.ncbi.nlm.nih.gov/compound/12237058#section=2D-Structure
https://pubchem.ncbi.nlm.nih.gov/compound/12233699#section=2D-Structure
https://pubchem.ncbi.nlm.nih.gov/compound/12232187#section=2D-Structure
https://pubchem.ncbi.nlm.nih.gov/compound/122220079#section=2D-Structure
https://pubchem.ncbi.nlm.nih.gov/compound/122212318#section=2D-Structure
https://pubchem.ncbi.nlm.nih.gov/compound/122203343#section=2D-Structure
https://pubchem.ncbi.nlm.nih.gov/compound/12220291#section=2D-Structure
https://pubchem.ncbi.nlm.nih.gov/compound/122202596#section=2D-Structure
https://pubchem.ncbi.nlm.nih.gov/compound/12207900#section=2D-Structure
https://pubchem.ncbi.nlm.nih.gov/compound/12163902#section=2D-Structure
https://pubchem.ncbi.nlm.nih.gov/compound/12148513#section=2D-Structure
https://pubchem.ncbi.nlm.nih.gov/compound/121474314#section=2D-Structure
https://pubchem.ncbi.nlm.nih.gov/compound/121417069#section=2D-Structure
https://pubchem.ncbi.nlm.nih.gov/compound/121374901#section=2D-Structure
https://pubchem.ncbi.nlm.nih.gov/compound/121364906#section=2D-Structure
https://pubchem.ncbi.nlm.nih.gov/compound/121352699#section=2D-Structure
https://pubchem.ncbi.nlm.nih.gov/compound/121329468#section=2D-Structure
https://pubchem.ncbi.nlm.nih.gov/compound/121292529#section=2D-Structure
https://pubchem.ncbi.nlm.nih.gov/compound/121288773#section=2D-Structure
https://pubchem.ncbi.nlm.nih.gov/compound/121255592#section=2D-Structure
https://pubchem.ncbi.nlm.nih.gov/compound/121226513#section=2D-Structure
https://pubchem.ncbi.nlm.nih.gov/compound/12103396#section=2D-Structure
https://pubchem.ncbi.nlm.nih.gov/compound/12102726#section=2D-Structure
https://pubchem.ncbi.nlm.nih.gov/compound/121011220#section=2D-Structure
https://pubchem.ncbi.nlm.nih.gov/compound/121009685#section=2D-Structure
https://pubchem.ncbi.nlm.nih.gov/compound/121009593#section=2D-Structure
https://pubchem.ncbi.nlm.nih.gov/compound/12063057#section=2D-Structure
https://pubchem.ncbi.nlm.nih.gov/compound/12054520#section=2D-Structure
https://pubchem.ncbi.nlm.nih.gov/compound/12054518#section=2D-Structure
https://pubchem.ncbi.nlm.nih.gov/compound/12049115#section=2D-Structure
https://pubchem.ncbi.nlm.nih.gov/compound/12028636#section=2D-Structure
https://pubchem.ncbi.nlm.nih.gov/compound/12025928#section=2D-Structure
https://pubchem.ncbi.nlm.nih.gov/compound/120193#section=2D-Structure
https://pubchem.ncbi.nlm.nih.gov/compound/11980585#section=2D-Structure
https://pubchem.ncbi.nlm.nih.gov/compound/11938#section=2D-Structure
https://pubchem.ncbi.nlm.nih.gov/compound/11913#section=2D-Structure
https://pubchem.ncbi.nlm.nih.gov/compound/119080326#section=2D-Structure
https://pubchem.ncbi.nlm.nih.gov/compound/119077605#section=2D-Structure
https://pubchem.ncbi.nlm.nih.gov/compound/118895211#section=2D-Structure
https://pubchem.ncbi.nlm.nih.gov/compound/118895210#section=2D-Structure
https://pubchem.ncbi.nlm.nih.gov/compound/118895209#section=2D-Structure
https://pubchem.ncbi.nlm.nih.gov/compound/118895206#section=2D-Structure
https://pubchem.ncbi.nlm.nih.gov/compound/118895205#section=2D-Structure
https://pubchem.ncbi.nlm.nih.gov/compound/118882207#section=2D-Structure
https://pubchem.ncbi.nlm.nih.gov/compound/11886#section=2D-Structure
https://pubchem.ncbi.nlm.nih.gov/compound/118855982#section=2D-Structure
https://pubchem.ncbi.nlm.nih.gov/compound/11885#section=2D-Structure
https://pubchem.ncbi.nlm.nih.gov/compound/118753718#section=2D-Structure
https://pubchem.ncbi.nlm.nih.gov/compound/118726780#section=2D-Structure
https://pubchem.ncbi.nlm.nih.gov/compound/118699211#section=2D-Structure
https://pubchem.ncbi.nlm.nih.gov/compound/118682689#section=2D-Structure
https://pubchem.ncbi.nlm.nih.gov/compound/118670116#section=2D-Structure
https://pubchem.ncbi.nlm.nih.gov/compound/118658809#section=2D-Structure

https://pubchem.ncbi.nlm.nih.gov/compound/118658808#section=2D-Structure
https://pubchem.ncbi.nlm.nih.gov/compound/118657405#section=2D-Structure
https://pubchem.ncbi.nlm.nih.gov/compound/118642207#section=2D-Structure
https://pubchem.ncbi.nlm.nih.gov/compound/118636790#section=2D-Structure
https://pubchem.ncbi.nlm.nih.gov/compound/118621033#section=2D-Structure
https://pubchem.ncbi.nlm.nih.gov/compound/118607613#section=2D-Structure
https://pubchem.ncbi.nlm.nih.gov/compound/118586779#section=2D-Structure
https://pubchem.ncbi.nlm.nih.gov/compound/118564046#section=2D-Structure
https://pubchem.ncbi.nlm.nih.gov/compound/118534063#section=2D-Structure
https://pubchem.ncbi.nlm.nih.gov/compound/118467945#section=2D-Structure
https://pubchem.ncbi.nlm.nih.gov/compound/118461124#section=2D-Structure
https://pubchem.ncbi.nlm.nih.gov/compound/118450341#section=2D-Structure
https://pubchem.ncbi.nlm.nih.gov/compound/118429056#section=2D-Structure
https://pubchem.ncbi.nlm.nih.gov/compound/118426783#section=2D-Structure
https://pubchem.ncbi.nlm.nih.gov/compound/118418533#section=2D-Structure
https://pubchem.ncbi.nlm.nih.gov/compound/118376910#section=2D-Structure
https://pubchem.ncbi.nlm.nih.gov/compound/118375253#section=2D-Structure
https://pubchem.ncbi.nlm.nih.gov/compound/118374156#section=2D-Structure
https://pubchem.ncbi.nlm.nih.gov/compound/118354543#section=2D-Structure
https://pubchem.ncbi.nlm.nih.gov/compound/118351303#section=2D-Structure
https://pubchem.ncbi.nlm.nih.gov/compound/118274437#section=2D-Structure
https://pubchem.ncbi.nlm.nih.gov/compound/118265475#section=2D-Structure
https://pubchem.ncbi.nlm.nih.gov/compound/118195545#section=2D-Structure
https://pubchem.ncbi.nlm.nih.gov/compound/118157786#section=2D-Structure
https://pubchem.ncbi.nlm.nih.gov/compound/118157785#section=2D-Structure
https://pubchem.ncbi.nlm.nih.gov/compound/118064210#section=2D-Structure
https://pubchem.ncbi.nlm.nih.gov/compound/118063894#section=2D-Structure
https://pubchem.ncbi.nlm.nih.gov/compound/118025086#section=2D-Structure
https://pubchem.ncbi.nlm.nih.gov/compound/117965392#section=2D-Structure
https://pubchem.ncbi.nlm.nih.gov/compound/117965117#section=2D-Structure
https://pubchem.ncbi.nlm.nih.gov/compound/117914579#section=2D-Structure
https://pubchem.ncbi.nlm.nih.gov/compound/117889661#section=2D-Structure
https://pubchem.ncbi.nlm.nih.gov/compound/11786362#section=2D-Structure
https://pubchem.ncbi.nlm.nih.gov/compound/117851904#section=2D-Structure
https://pubchem.ncbi.nlm.nih.gov/compound/117834494#section=2D-Structure
https://pubchem.ncbi.nlm.nih.gov/compound/11780559#section=2D-Structure
https://pubchem.ncbi.nlm.nih.gov/compound/117793758#section=2D-Structure
https://pubchem.ncbi.nlm.nih.gov/compound/11778532#section=2D-Structure
https://pubchem.ncbi.nlm.nih.gov/compound/117784898#section=2D-Structure
https://pubchem.ncbi.nlm.nih.gov/compound/11776519#section=2D-Structure
https://pubchem.ncbi.nlm.nih.gov/compound/117733015#section=2D-Structure
https://pubchem.ncbi.nlm.nih.gov/compound/117722754#section=2D-Structure
https://pubchem.ncbi.nlm.nih.gov/compound/117716083#section=2D-Structure
https://pubchem.ncbi.nlm.nih.gov/compound/11771154#section=2D-Structure
https://pubchem.ncbi.nlm.nih.gov/compound/117703394#section=2D-Structure
https://pubchem.ncbi.nlm.nih.gov/compound/117673301#section=2D-Structure
https://pubchem.ncbi.nlm.nih.gov/compound/117673249#section=2D-Structure
https://pubchem.ncbi.nlm.nih.gov/compound/117673245#section=2D-Structure
https://pubchem.ncbi.nlm.nih.gov/compound/117673244#section=2D-Structure
https://pubchem.ncbi.nlm.nih.gov/compound/117673235#section=2D-Structure
https://pubchem.ncbi.nlm.nih.gov/compound/11767#section=2D-Structure
https://pubchem.ncbi.nlm.nih.gov/compound/117630769#section=2D-Structure
https://pubchem.ncbi.nlm.nih.gov/compound/117599464#section=2D-Structure
https://pubchem.ncbi.nlm.nih.gov/compound/11739145#section=2D-Structure
https://pubchem.ncbi.nlm.nih.gov/compound/11737052#section=2D-Structure

https://pubchem.ncbi.nlm.nih.gov/compound/11694859#section=2D-Structure
https://pubchem.ncbi.nlm.nih.gov/compound/11659350#section=2D-Structure
https://pubchem.ncbi.nlm.nih.gov/compound/11630137#section=2D-Structure
https://pubchem.ncbi.nlm.nih.gov/compound/11622904#section=2D-Structure
https://pubchem.ncbi.nlm.nih.gov/compound/11609629#section=2D-Structure
https://pubchem.ncbi.nlm.nih.gov/compound/11544066#section=2D-Structure
https://pubchem.ncbi.nlm.nih.gov/compound/11499937#section=2D-Structure
https://pubchem.ncbi.nlm.nih.gov/compound/11493156#section=2D-Structure
https://pubchem.ncbi.nlm.nih.gov/compound/114910#section=2D-Structure
https://pubchem.ncbi.nlm.nih.gov/compound/114754#section=2D-Structure
https://pubchem.ncbi.nlm.nih.gov/compound/11470045#section=2D-Structure
https://pubchem.ncbi.nlm.nih.gov/compound/114645#section=2D-Structure
https://pubchem.ncbi.nlm.nih.gov/compound/11413143#section=2D-Structure
https://pubchem.ncbi.nlm.nih.gov/compound/11371481#section=2D-Structure
https://pubchem.ncbi.nlm.nih.gov/compound/11353#section=2D-Structure
https://pubchem.ncbi.nlm.nih.gov/compound/11352#section=2D-Structure
https://pubchem.ncbi.nlm.nih.gov/compound/11350263#section=2D-Structure
https://pubchem.ncbi.nlm.nih.gov/compound/11345981#section=2D-Structure
https://pubchem.ncbi.nlm.nih.gov/compound/11345295#section=2D-Structure
https://pubchem.ncbi.nlm.nih.gov/compound/11345285#section=2D-Structure
https://pubchem.ncbi.nlm.nih.gov/compound/11342#section=2D-Structure
https://pubchem.ncbi.nlm.nih.gov/compound/11298953#section=2D-Structure
https://pubchem.ncbi.nlm.nih.gov/compound/11276276#section=2D-Structure
https://pubchem.ncbi.nlm.nih.gov/compound/11254945#section=2D-Structure
https://pubchem.ncbi.nlm.nih.gov/compound/11253575#section=2D-Structure
https://pubchem.ncbi.nlm.nih.gov/compound/11242781#section=2D-Structure
https://pubchem.ncbi.nlm.nih.gov/compound/11242005#section=2D-Structure
https://pubchem.ncbi.nlm.nih.gov/compound/11231394#section=2D-Structure
https://pubchem.ncbi.nlm.nih.gov/compound/11218051#section=2D-Structure
https://pubchem.ncbi.nlm.nih.gov/compound/11207640#section=2D-Structure
https://pubchem.ncbi.nlm.nih.gov/compound/11176265#section=2D-Structure
https://pubchem.ncbi.nlm.nih.gov/compound/11160163#section=2D-Structure
https://pubchem.ncbi.nlm.nih.gov/compound/11151846#section=2D-Structure
https://pubchem.ncbi.nlm.nih.gov/compound/11150190#section=2D-Structure
https://pubchem.ncbi.nlm.nih.gov/compound/11149151#section=2D-Structure
https://pubchem.ncbi.nlm.nih.gov/compound/11138005#section=2D-Structure
https://pubchem.ncbi.nlm.nih.gov/compound/11118672#section=2D-Structure
https://pubchem.ncbi.nlm.nih.gov/compound/11118468#section=2D-Structure
https://pubchem.ncbi.nlm.nih.gov/compound/11097892#section=2D-Structure
https://pubchem.ncbi.nlm.nih.gov/compound/11086826#section=2D-Structure
https://pubchem.ncbi.nlm.nih.gov/compound/11075341#section=2D-Structure
https://pubchem.ncbi.nlm.nih.gov/compound/11063652#section=2D-Structure
https://pubchem.ncbi.nlm.nih.gov/compound/11053044#section=2D-Structure
https://pubchem.ncbi.nlm.nih.gov/compound/11053003#section=2D-Structure
https://pubchem.ncbi.nlm.nih.gov/compound/11032116#section=2D-Structure
https://pubchem.ncbi.nlm.nih.gov/compound/11020259#section=2D-Structure
https://pubchem.ncbi.nlm.nih.gov/compound/110173102#section=2D-Structure
https://pubchem.ncbi.nlm.nih.gov/compound/11010344#section=2D-Structure
https://pubchem.ncbi.nlm.nih.gov/compound/10978520#section=2D-Structure
https://pubchem.ncbi.nlm.nih.gov/compound/10968724#section=2D-Structure
https://pubchem.ncbi.nlm.nih.gov/compound/10966622#section=2D-Structure
https://pubchem.ncbi.nlm.nih.gov/compound/10954438#section=2D-Structure
https://pubchem.ncbi.nlm.nih.gov/compound/10950233#section=2D-Structure
https://pubchem.ncbi.nlm.nih.gov/compound/10933658#section=2D-Structure
https://pubchem.ncbi.nlm.nih.gov/compound/10923530#section=2D-Structure

https://pubchem.ncbi.nlm.nih.gov/compound/10900428#section=2D-Structure
https://pubchem.ncbi.nlm.nih.gov/compound/108891#section=2D-Structure
https://pubchem.ncbi.nlm.nih.gov/compound/10880095#section=2D-Structure
https://pubchem.ncbi.nlm.nih.gov/compound/10878450#section=2D-Structure
https://pubchem.ncbi.nlm.nih.gov/compound/10853413#section=2D-Structure
https://pubchem.ncbi.nlm.nih.gov/compound/10847287#section=2D-Structure
https://pubchem.ncbi.nlm.nih.gov/compound/10822382#section=2D-Structure
https://pubchem.ncbi.nlm.nih.gov/compound/108113#section=2D-Structure
https://pubchem.ncbi.nlm.nih.gov/compound/10799208#section=2D-Structure
https://pubchem.ncbi.nlm.nih.gov/compound/107945#section=2D-Structure
https://pubchem.ncbi.nlm.nih.gov/compound/107781#section=2D-Structure
https://pubchem.ncbi.nlm.nih.gov/compound/10775573#section=2D-Structure
https://pubchem.ncbi.nlm.nih.gov/compound/10774261#section=2D-Structure
https://pubchem.ncbi.nlm.nih.gov/compound/10774154#section=2D-Structure
https://pubchem.ncbi.nlm.nih.gov/compound/10750785#section=2D-Structure
https://pubchem.ncbi.nlm.nih.gov/compound/107447190#section=2D-Structure
https://pubchem.ncbi.nlm.nih.gov/compound/10731#section=2D-Structure
https://pubchem.ncbi.nlm.nih.gov/compound/10705692#section=2D-Structure
https://pubchem.ncbi.nlm.nih.gov/compound/10705591#section=2D-Structure
https://pubchem.ncbi.nlm.nih.gov/compound/10662605#section=2D-Structure
https://pubchem.ncbi.nlm.nih.gov/compound/10638969#section=2D-Structure
https://pubchem.ncbi.nlm.nih.gov/compound/10633433#section=2D-Structure
https://pubchem.ncbi.nlm.nih.gov/compound/10632444#section=2D-Structure
https://pubchem.ncbi.nlm.nih.gov/compound/10612774#section=2D-Structure
https://pubchem.ncbi.nlm.nih.gov/compound/10609727#section=2D-Structure
https://pubchem.ncbi.nlm.nih.gov/compound/10584909#section=2D-Structure
https://pubchem.ncbi.nlm.nih.gov/compound/10584604#section=2D-Structure
https://pubchem.ncbi.nlm.nih.gov/compound/10563550#section=2D-Structure
https://pubchem.ncbi.nlm.nih.gov/compound/10562355#section=2D-Structure
https://pubchem.ncbi.nlm.nih.gov/compound/10562128#section=2D-Structure
https://pubchem.ncbi.nlm.nih.gov/compound/10561172#section=2D-Structure
https://pubchem.ncbi.nlm.nih.gov/compound/10560762#section=2D-Structure
https://pubchem.ncbi.nlm.nih.gov/compound/10539437#section=2D-Structure
https://pubchem.ncbi.nlm.nih.gov/compound/10538615#section=2D-Structure
https://pubchem.ncbi.nlm.nih.gov/compound/10538485#section=2D-Structure
https://pubchem.ncbi.nlm.nih.gov/compound/10513903#section=2D-Structure
https://pubchem.ncbi.nlm.nih.gov/compound/105022#section=2D-Structure
https://pubchem.ncbi.nlm.nih.gov/compound/104988#section=2D-Structure
https://pubchem.ncbi.nlm.nih.gov/compound/10495813#section=2D-Structure
https://pubchem.ncbi.nlm.nih.gov/compound/10493167#section=2D-Structure
https://pubchem.ncbi.nlm.nih.gov/compound/10490627#section=2D-Structure
https://pubchem.ncbi.nlm.nih.gov/compound/104777#section=2D-Structure
https://pubchem.ncbi.nlm.nih.gov/compound/10465615#section=2D-Structure
https://pubchem.ncbi.nlm.nih.gov/compound/10422453#section=2D-Structure
https://pubchem.ncbi.nlm.nih.gov/compound/10401228#section=2D-Structure
https://pubchem.ncbi.nlm.nih.gov/compound/10378116#section=2D-Structure
https://pubchem.ncbi.nlm.nih.gov/compound/10331771#section=2D-Structure
https://pubchem.ncbi.nlm.nih.gov/compound/10308016#section=2D-Structure
https://pubchem.ncbi.nlm.nih.gov/compound/10288#section=2D-Structure
https://pubchem.ncbi.nlm.nih.gov/compound/10285940#section=2D-Structure
https://pubchem.ncbi.nlm.nih.gov/compound/10263500#section=2D-Structure
https://pubchem.ncbi.nlm.nih.gov/compound/102589905#section=2D-Structure
https://pubchem.ncbi.nlm.nih.gov/compound/102531024#section=2D-Structure
https://pubchem.ncbi.nlm.nih.gov/compound/102518946#section=2D-Structure
https://pubchem.ncbi.nlm.nih.gov/compound/102506826#section=2D-Structure

https://pubchem.ncbi.nlm.nih.gov/compound/102490123#section=2D-Structure
https://pubchem.ncbi.nlm.nih.gov/compound/10247175#section=2D-Structure
https://pubchem.ncbi.nlm.nih.gov/compound/102460999#section=2D-Structure
https://pubchem.ncbi.nlm.nih.gov/compound/102458883#section=2D-Structure
https://pubchem.ncbi.nlm.nih.gov/compound/102447235#section=2D-Structure
https://pubchem.ncbi.nlm.nih.gov/compound/102447234#section=2D-Structure
https://pubchem.ncbi.nlm.nih.gov/compound/102444189#section=2D-Structure
https://pubchem.ncbi.nlm.nih.gov/compound/102441883#section=2D-Structure
https://pubchem.ncbi.nlm.nih.gov/compound/102436785#section=2D-Structure
https://pubchem.ncbi.nlm.nih.gov/compound/10243654#section=2D-Structure
https://pubchem.ncbi.nlm.nih.gov/compound/102436019#section=2D-Structure
https://pubchem.ncbi.nlm.nih.gov/compound/102436018#section=2D-Structure
https://pubchem.ncbi.nlm.nih.gov/compound/102436016#section=2D-Structure
https://pubchem.ncbi.nlm.nih.gov/compound/102429943#section=2D-Structure
https://pubchem.ncbi.nlm.nih.gov/compound/102426751#section=2D-Structure
https://pubchem.ncbi.nlm.nih.gov/compound/102419834#section=2D-Structure
https://pubchem.ncbi.nlm.nih.gov/compound/102410246#section=2D-Structure
https://pubchem.ncbi.nlm.nih.gov/compound/102408840#section=2D-Structure
https://pubchem.ncbi.nlm.nih.gov/compound/102405455#section=2D-Structure
https://pubchem.ncbi.nlm.nih.gov/compound/102403997#section=2D-Structure
https://pubchem.ncbi.nlm.nih.gov/compound/102398010#section=2D-Structure
https://pubchem.ncbi.nlm.nih.gov/compound/102396433#section=2D-Structure
https://pubchem.ncbi.nlm.nih.gov/compound/102382625#section=2D-Structure
https://pubchem.ncbi.nlm.nih.gov/compound/102374833#section=2D-Structure
https://pubchem.ncbi.nlm.nih.gov/compound/102362197#section=2D-Structure
https://pubchem.ncbi.nlm.nih.gov/compound/102362196#section=2D-Structure
https://pubchem.ncbi.nlm.nih.gov/compound/102345314#section=2D-Structure
https://pubchem.ncbi.nlm.nih.gov/compound/102339152#section=2D-Structure
https://pubchem.ncbi.nlm.nih.gov/compound/102334849#section=2D-Structure
https://pubchem.ncbi.nlm.nih.gov/compound/102331123#section=2D-Structure
https://pubchem.ncbi.nlm.nih.gov/compound/102318273#section=2D-Structure
https://pubchem.ncbi.nlm.nih.gov/compound/102313770#section=2D-Structure
https://pubchem.ncbi.nlm.nih.gov/compound/102290570#section=2D-Structure
https://pubchem.ncbi.nlm.nih.gov/compound/102289373#section=2D-Structure
https://pubchem.ncbi.nlm.nih.gov/compound/102289372#section=2D-Structure
https://pubchem.ncbi.nlm.nih.gov/compound/102289370#section=2D-Structure
https://pubchem.ncbi.nlm.nih.gov/compound/102289369#section=2D-Structure
https://pubchem.ncbi.nlm.nih.gov/compound/102275013#section=2D-Structure
https://pubchem.ncbi.nlm.nih.gov/compound/102274972#section=2D-Structure
https://pubchem.ncbi.nlm.nih.gov/compound/102245257#section=2D-Structure
https://pubchem.ncbi.nlm.nih.gov/compound/102244136#section=2D-Structure
https://pubchem.ncbi.nlm.nih.gov/compound/102225311#section=2D-Structure
https://pubchem.ncbi.nlm.nih.gov/compound/102223831#section=2D-Structure
https://pubchem.ncbi.nlm.nih.gov/compound/102221969#section=2D-Structure
https://pubchem.ncbi.nlm.nih.gov/compound/102219927#section=2D-Structure
https://pubchem.ncbi.nlm.nih.gov/compound/102169934#section=2D-Structure
https://pubchem.ncbi.nlm.nih.gov/compound/102169933#section=2D-Structure
https://pubchem.ncbi.nlm.nih.gov/compound/102163449#section=2D-Structure
https://pubchem.ncbi.nlm.nih.gov/compound/102160551#section=2D-Structure
https://pubchem.ncbi.nlm.nih.gov/compound/102127433#section=2D-Structure
https://pubchem.ncbi.nlm.nih.gov/compound/102090738#section=2D-Structure
https://pubchem.ncbi.nlm.nih.gov/compound/102067260#section=2D-Structure
https://pubchem.ncbi.nlm.nih.gov/compound/102065214#section=2D-Structure
https://pubchem.ncbi.nlm.nih.gov/compound/102059165#section=2D-Structure
https://pubchem.ncbi.nlm.nih.gov/compound/102059164#section=2D-Structure

https://pubchem.ncbi.nlm.nih.gov/compound/102057756#section=2D-Structure
https://pubchem.ncbi.nlm.nih.gov/compound/102047514#section=2D-Structure
https://pubchem.ncbi.nlm.nih.gov/compound/102043752#section=2D-Structure
https://pubchem.ncbi.nlm.nih.gov/compound/102043607#section=2D-Structure
https://pubchem.ncbi.nlm.nih.gov/compound/102042004#section=2D-Structure
https://pubchem.ncbi.nlm.nih.gov/compound/102041770#section=2D-Structure
https://pubchem.ncbi.nlm.nih.gov/compound/102040648#section=2D-Structure
https://pubchem.ncbi.nlm.nih.gov/compound/102031400#section=2D-Structure
https://pubchem.ncbi.nlm.nih.gov/compound/102015694#section=2D-Structure
https://pubchem.ncbi.nlm.nih.gov/compound/102003012#section=2D-Structure
https://pubchem.ncbi.nlm.nih.gov/compound/101982230#section=2D-Structure
https://pubchem.ncbi.nlm.nih.gov/compound/101967222#section=2D-Structure
https://pubchem.ncbi.nlm.nih.gov/compound/101967220#section=2D-Structure
https://pubchem.ncbi.nlm.nih.gov/compound/101956168#section=2D-Structure
https://pubchem.ncbi.nlm.nih.gov/compound/101954572#section=2D-Structure
https://pubchem.ncbi.nlm.nih.gov/compound/101954512#section=2D-Structure
https://pubchem.ncbi.nlm.nih.gov/compound/101906784#section=2D-Structure
https://pubchem.ncbi.nlm.nih.gov/compound/101906568#section=2D-Structure
https://pubchem.ncbi.nlm.nih.gov/compound/101903121#section=2D-Structure
https://pubchem.ncbi.nlm.nih.gov/compound/101898565#section=2D-Structure
https://pubchem.ncbi.nlm.nih.gov/compound/101879865#section=2D-Structure
https://pubchem.ncbi.nlm.nih.gov/compound/101879862#section=2D-Structure
https://pubchem.ncbi.nlm.nih.gov/compound/10187#section=2D-Structure
https://pubchem.ncbi.nlm.nih.gov/compound/101864607#section=2D-Structure
https://pubchem.ncbi.nlm.nih.gov/compound/101864605#section=2D-Structure
https://pubchem.ncbi.nlm.nih.gov/compound/101853138#section=2D-Structure
https://pubchem.ncbi.nlm.nih.gov/compound/101853137#section=2D-Structure
https://pubchem.ncbi.nlm.nih.gov/compound/101790357#section=2D-Structure
https://pubchem.ncbi.nlm.nih.gov/compound/101789631#section=2D-Structure
https://pubchem.ncbi.nlm.nih.gov/compound/101788372#section=2D-Structure
https://pubchem.ncbi.nlm.nih.gov/compound/101769005#section=2D-Structure
https://pubchem.ncbi.nlm.nih.gov/compound/101745865#section=2D-Structure
https://pubchem.ncbi.nlm.nih.gov/compound/101743355#section=2D-Structure
https://pubchem.ncbi.nlm.nih.gov/compound/101700876#section=2D-Structure
https://pubchem.ncbi.nlm.nih.gov/compound/101682485#section=2D-Structure
https://pubchem.ncbi.nlm.nih.gov/compound/101674495#section=2D-Structure
https://pubchem.ncbi.nlm.nih.gov/compound/101673350#section=2D-Structure
https://pubchem.ncbi.nlm.nih.gov/compound/101672257#section=2D-Structure
https://pubchem.ncbi.nlm.nih.gov/compound/101605586#section=2D-Structure
https://pubchem.ncbi.nlm.nih.gov/compound/101600736#section=2D-Structure
https://pubchem.ncbi.nlm.nih.gov/compound/101600735#section=2D-Structure
https://pubchem.ncbi.nlm.nih.gov/compound/101597069#section=2D-Structure
https://pubchem.ncbi.nlm.nih.gov/compound/101586946#section=2D-Structure
https://pubchem.ncbi.nlm.nih.gov/compound/101584802#section=2D-Structure
https://pubchem.ncbi.nlm.nih.gov/compound/101584199#section=2D-Structure
https://pubchem.ncbi.nlm.nih.gov/compound/101578668#section=2D-Structure
https://pubchem.ncbi.nlm.nih.gov/compound/101569075#section=2D-Structure
https://pubchem.ncbi.nlm.nih.gov/compound/101569074#section=2D-Structure
https://pubchem.ncbi.nlm.nih.gov/compound/101557962#section=2D-Structure
https://pubchem.ncbi.nlm.nih.gov/compound/101557384#section=2D-Structure
https://pubchem.ncbi.nlm.nih.gov/compound/101557383#section=2D-Structure
https://pubchem.ncbi.nlm.nih.gov/compound/101514253#section=2D-Structure
https://pubchem.ncbi.nlm.nih.gov/compound/101499492#section=2D-Structure
https://pubchem.ncbi.nlm.nih.gov/compound/101499486#section=2D-Structure
https://pubchem.ncbi.nlm.nih.gov/compound/101497334#section=2D-Structure

https://pubchem.ncbi.nlm.nih.gov/compound/10149212#section=2D-Structure
https://pubchem.ncbi.nlm.nih.gov/compound/10149107#section=2D-Structure
https://pubchem.ncbi.nlm.nih.gov/compound/101489767#section=2D-Structure
https://pubchem.ncbi.nlm.nih.gov/compound/101465896#section=2D-Structure
https://pubchem.ncbi.nlm.nih.gov/compound/101465895#section=2D-Structure
https://pubchem.ncbi.nlm.nih.gov/compound/101457268#section=2D-Structure
https://pubchem.ncbi.nlm.nih.gov/compound/101449898#section=2D-Structure
https://pubchem.ncbi.nlm.nih.gov/compound/101442949#section=2D-Structure
https://pubchem.ncbi.nlm.nih.gov/compound/101440160#section=2D-Structure
https://pubchem.ncbi.nlm.nih.gov/compound/101426937#section=2D-Structure
https://pubchem.ncbi.nlm.nih.gov/compound/101342764#section=2D-Structure
https://pubchem.ncbi.nlm.nih.gov/compound/10130532#section=2D-Structure
https://pubchem.ncbi.nlm.nih.gov/compound/10130497#section=2D-Structure
https://pubchem.ncbi.nlm.nih.gov/compound/101273245#section=2D-Structure
https://pubchem.ncbi.nlm.nih.gov/compound/101262961#section=2D-Structure
https://pubchem.ncbi.nlm.nih.gov/compound/101260032#section=2D-Structure
https://pubchem.ncbi.nlm.nih.gov/compound/101252#section=2D-Structure
https://pubchem.ncbi.nlm.nih.gov/compound/101244294#section=2D-Structure
https://pubchem.ncbi.nlm.nih.gov/compound/101194299#section=2D-Structure
https://pubchem.ncbi.nlm.nih.gov/compound/101194298#section=2D-Structure
https://pubchem.ncbi.nlm.nih.gov/compound/101191567#section=2D-Structure
https://pubchem.ncbi.nlm.nih.gov/compound/101182421#section=2D-Structure
https://pubchem.ncbi.nlm.nih.gov/compound/101178549#section=2D-Structure
https://pubchem.ncbi.nlm.nih.gov/compound/101127358#section=2D-Structure
https://pubchem.ncbi.nlm.nih.gov/compound/101120092#section=2D-Structure
https://pubchem.ncbi.nlm.nih.gov/compound/101117638#section=2D-Structure
https://pubchem.ncbi.nlm.nih.gov/compound/101117637#section=2D-Structure
https://pubchem.ncbi.nlm.nih.gov/compound/101113694#section=2D-Structure
https://pubchem.ncbi.nlm.nih.gov/compound/101113692#section=2D-Structure
https://pubchem.ncbi.nlm.nih.gov/compound/101110721#section=2D-Structure
https://pubchem.ncbi.nlm.nih.gov/compound/101088269#section=2D-Structure
https://pubchem.ncbi.nlm.nih.gov/compound/101085334#section=2D-Structure
https://pubchem.ncbi.nlm.nih.gov/compound/101061208#section=2D-Structure
https://pubchem.ncbi.nlm.nih.gov/compound/10105654#section=2D-Structure
https://pubchem.ncbi.nlm.nih.gov/compound/101044395#section=2D-Structure
https://pubchem.ncbi.nlm.nih.gov/compound/101022943#section=2D-Structure
https://pubchem.ncbi.nlm.nih.gov/compound/100970047#section=2D-Structure
https://pubchem.ncbi.nlm.nih.gov/compound/100970046#section=2D-Structure
https://pubchem.ncbi.nlm.nih.gov/compound/100956568#section=2D-Structure
https://pubchem.ncbi.nlm.nih.gov/compound/100952886#section=2D-Structure
https://pubchem.ncbi.nlm.nih.gov/compound/100930981#section=2D-Structure
https://pubchem.ncbi.nlm.nih.gov/compound/100919218#section=2D-Structure
https://pubchem.ncbi.nlm.nih.gov/compound/10082406#section=2D-Structure
https://pubchem.ncbi.nlm.nih.gov/compound/10082104#section=2D-Structure
https://pubchem.ncbi.nlm.nih.gov/compound/10081288#section=2D-Structure
https://pubchem.ncbi.nlm.nih.gov/compound/10060149#section=2D-Structure
https://pubchem.ncbi.nlm.nih.gov/compound/9994760#section=2D-Structure
https://pubchem.ncbi.nlm.nih.gov/compound/9991383#section=2D-Structure
https://pubchem.ncbi.nlm.nih.gov/compound/9964435#section=2D-Structure
https://pubchem.ncbi.nlm.nih.gov/compound/9948110#section=2D-Structure
https://pubchem.ncbi.nlm.nih.gov/compound/9942954#section=2D-Structure
https://pubchem.ncbi.nlm.nih.gov/compound/9925608#section=2D-Structure
https://pubchem.ncbi.nlm.nih.gov/compound/9899021#section=2D-Structure
https://pubchem.ncbi.nlm.nih.gov/compound/9856653#section=2D-Structure
https://pubchem.ncbi.nlm.nih.gov/compound/9838992#section=2D-Structure

https://pubchem.ncbi.nlm.nih.gov/compound/9837334#section=2D-Structure
https://pubchem.ncbi.nlm.nih.gov/compound/98200053#section=2D-Structure
https://pubchem.ncbi.nlm.nih.gov/compound/9817510#section=2D-Structure
https://pubchem.ncbi.nlm.nih.gov/compound/98075858#section=2D-Structure
https://pubchem.ncbi.nlm.nih.gov/compound/98069140#section=2D-Structure
https://pubchem.ncbi.nlm.nih.gov/compound/9794356#section=2D-Structure
https://pubchem.ncbi.nlm.nih.gov/compound/97303753#section=2D-Structure
https://pubchem.ncbi.nlm.nih.gov/compound/97303724#section=2D-Structure
https://pubchem.ncbi.nlm.nih.gov/compound/97302434#section=2D-Structure
https://pubchem.ncbi.nlm.nih.gov/compound/97169990#section=2D-Structure
https://pubchem.ncbi.nlm.nih.gov/compound/97049648#section=2D-Structure
https://pubchem.ncbi.nlm.nih.gov/compound/96469368#section=2D-Structure
https://pubchem.ncbi.nlm.nih.gov/compound/96460#section=2D-Structure
https://pubchem.ncbi.nlm.nih.gov/compound/95843#section=2D-Structure
https://pubchem.ncbi.nlm.nih.gov/compound/9577013#section=2D-Structure
https://pubchem.ncbi.nlm.nih.gov/compound/9548821#section=2D-Structure
https://pubchem.ncbi.nlm.nih.gov/compound/9548747#section=2D-Structure
https://pubchem.ncbi.nlm.nih.gov/compound/9543005#section=2D-Structure
https://pubchem.ncbi.nlm.nih.gov/compound/95117963#section=2D-Structure
https://pubchem.ncbi.nlm.nih.gov/compound/95046937#section=2D-Structure
https://pubchem.ncbi.nlm.nih.gov/compound/94044673#section=2D-Structure
https://pubchem.ncbi.nlm.nih.gov/compound/94044671#section=2D-Structure
https://pubchem.ncbi.nlm.nih.gov/compound/9362#section=2D-Structure
https://pubchem.ncbi.nlm.nih.gov/compound/928025#section=2D-Structure
https://pubchem.ncbi.nlm.nih.gov/compound/921373#section=2D-Structure
https://pubchem.ncbi.nlm.nih.gov/compound/92030249#section=2D-Structure
https://pubchem.ncbi.nlm.nih.gov/compound/92018378#section=2D-Structure
https://pubchem.ncbi.nlm.nih.gov/compound/91927068#section=2D-Structure
https://pubchem.ncbi.nlm.nih.gov/compound/919150#section=2D-Structure
https://pubchem.ncbi.nlm.nih.gov/compound/91884024#section=2D-Structure
https://pubchem.ncbi.nlm.nih.gov/compound/91882450#section=2D-Structure
https://pubchem.ncbi.nlm.nih.gov/compound/91882449#section=2D-Structure
https://pubchem.ncbi.nlm.nih.gov/compound/91871080#section=2D-Structure
https://pubchem.ncbi.nlm.nih.gov/compound/91870478#section=2D-Structure
https://pubchem.ncbi.nlm.nih.gov/compound/91870477#section=2D-Structure
https://pubchem.ncbi.nlm.nih.gov/compound/91870229#section=2D-Structure
https://pubchem.ncbi.nlm.nih.gov/compound/91869098#section=2D-Structure
https://pubchem.ncbi.nlm.nih.gov/compound/91868786#section=2D-Structure
https://pubchem.ncbi.nlm.nih.gov/compound/91866349#section=2D-Structure
https://pubchem.ncbi.nlm.nih.gov/compound/91866340#section=2D-Structure
https://pubchem.ncbi.nlm.nih.gov/compound/91865703#section=2D-Structure
https://pubchem.ncbi.nlm.nih.gov/compound/91844761#section=2D-Structure
https://pubchem.ncbi.nlm.nih.gov/compound/91844749#section=2D-Structure
https://pubchem.ncbi.nlm.nih.gov/compound/91844576#section=2D-Structure
https://pubchem.ncbi.nlm.nih.gov/compound/91822992#section=2D-Structure
https://pubchem.ncbi.nlm.nih.gov/compound/91809266#section=2D-Structure
https://pubchem.ncbi.nlm.nih.gov/compound/91806511#section=2D-Structure
https://pubchem.ncbi.nlm.nih.gov/compound/91805271#section=2D-Structure
https://pubchem.ncbi.nlm.nih.gov/compound/91804770#section=2D-Structure
https://pubchem.ncbi.nlm.nih.gov/compound/91801988#section=2D-Structure
https://pubchem.ncbi.nlm.nih.gov/compound/91801931#section=2D-Structure
https://pubchem.ncbi.nlm.nih.gov/compound/91747385#section=2D-Structure
https://pubchem.ncbi.nlm.nih.gov/compound/91694317#section=2D-Structure
https://pubchem.ncbi.nlm.nih.gov/compound/91665818#section=2D-Structure
https://pubchem.ncbi.nlm.nih.gov/compound/91665754#section=2D-Structure

https://pubchem.ncbi.nlm.nih.gov/compound/91665544#section=2D-Structure
https://pubchem.ncbi.nlm.nih.gov/compound/91665543#section=2D-Structure
https://pubchem.ncbi.nlm.nih.gov/compound/91665542#section=2D-Structure
https://pubchem.ncbi.nlm.nih.gov/compound/91665525#section=2D-Structure
https://pubchem.ncbi.nlm.nih.gov/compound/91665520#section=2D-Structure
https://pubchem.ncbi.nlm.nih.gov/compound/91665514#section=2D-Structure
https://pubchem.ncbi.nlm.nih.gov/compound/91665513#section=2D-Structure
https://pubchem.ncbi.nlm.nih.gov/compound/91665462#section=2D-Structure
https://pubchem.ncbi.nlm.nih.gov/compound/91665258#section=2D-Structure
https://pubchem.ncbi.nlm.nih.gov/compound/91665243#section=2D-Structure
https://pubchem.ncbi.nlm.nih.gov/compound/91622123#section=2D-Structure
https://pubchem.ncbi.nlm.nih.gov/compound/91621497#section=2D-Structure
https://pubchem.ncbi.nlm.nih.gov/compound/91621341#section=2D-Structure
https://pubchem.ncbi.nlm.nih.gov/compound/91621219#section=2D-Structure
https://pubchem.ncbi.nlm.nih.gov/compound/91620187#section=2D-Structure
https://pubchem.ncbi.nlm.nih.gov/compound/91617035#section=2D-Structure
https://pubchem.ncbi.nlm.nih.gov/compound/91615238#section=2D-Structure
https://pubchem.ncbi.nlm.nih.gov/compound/91610378#section=2D-Structure
https://pubchem.ncbi.nlm.nih.gov/compound/91610110#section=2D-Structure
https://pubchem.ncbi.nlm.nih.gov/compound/91608823#section=2D-Structure
https://pubchem.ncbi.nlm.nih.gov/compound/91607940#section=2D-Structure
https://pubchem.ncbi.nlm.nih.gov/compound/91607256#section=2D-Structure
https://pubchem.ncbi.nlm.nih.gov/compound/91607009#section=2D-Structure
https://pubchem.ncbi.nlm.nih.gov/compound/91605273#section=2D-Structure
https://pubchem.ncbi.nlm.nih.gov/compound/91603765#section=2D-Structure
https://pubchem.ncbi.nlm.nih.gov/compound/91603117#section=2D-Structure
https://pubchem.ncbi.nlm.nih.gov/compound/91602557#section=2D-Structure
https://pubchem.ncbi.nlm.nih.gov/compound/91599723#section=2D-Structure
https://pubchem.ncbi.nlm.nih.gov/compound/91598288#section=2D-Structure
https://pubchem.ncbi.nlm.nih.gov/compound/91598248#section=2D-Structure
https://pubchem.ncbi.nlm.nih.gov/compound/91593064#section=2D-Structure
https://pubchem.ncbi.nlm.nih.gov/compound/91592325#section=2D-Structure
https://pubchem.ncbi.nlm.nih.gov/compound/91589877#section=2D-Structure
https://pubchem.ncbi.nlm.nih.gov/compound/91584180#section=2D-Structure
https://pubchem.ncbi.nlm.nih.gov/compound/91571244#section=2D-Structure
https://pubchem.ncbi.nlm.nih.gov/compound/91571052#section=2D-Structure
https://pubchem.ncbi.nlm.nih.gov/compound/91567549#section=2D-Structure
https://pubchem.ncbi.nlm.nih.gov/compound/91563327#section=2D-Structure
https://pubchem.ncbi.nlm.nih.gov/compound/91560554#section=2D-Structure
https://pubchem.ncbi.nlm.nih.gov/compound/91555244#section=2D-Structure
https://pubchem.ncbi.nlm.nih.gov/compound/91554759#section=2D-Structure
https://pubchem.ncbi.nlm.nih.gov/compound/91553473#section=2D-Structure
https://pubchem.ncbi.nlm.nih.gov/compound/91551030#section=2D-Structure
https://pubchem.ncbi.nlm.nih.gov/compound/91550504#section=2D-Structure
https://pubchem.ncbi.nlm.nih.gov/compound/91547585#section=2D-Structure
https://pubchem.ncbi.nlm.nih.gov/compound/91546475#section=2D-Structure
https://pubchem.ncbi.nlm.nih.gov/compound/91545241#section=2D-Structure
https://pubchem.ncbi.nlm.nih.gov/compound/91544674#section=2D-Structure
https://pubchem.ncbi.nlm.nih.gov/compound/91541155#section=2D-Structure
https://pubchem.ncbi.nlm.nih.gov/compound/91539577#section=2D-Structure
https://pubchem.ncbi.nlm.nih.gov/compound/91539100#section=2D-Structure
https://pubchem.ncbi.nlm.nih.gov/compound/91538419#section=2D-Structure
https://pubchem.ncbi.nlm.nih.gov/compound/91537534#section=2D-Structure
https://pubchem.ncbi.nlm.nih.gov/compound/91537528#section=2D-Structure
https://pubchem.ncbi.nlm.nih.gov/compound/915336#section=2D-Structure

https://pubchem.ncbi.nlm.nih.gov/compound/91532390#section=2D-Structure
https://pubchem.ncbi.nlm.nih.gov/compound/91528922#section=2D-Structure
https://pubchem.ncbi.nlm.nih.gov/compound/91527849#section=2D-Structure
https://pubchem.ncbi.nlm.nih.gov/compound/91527817#section=2D-Structure
https://pubchem.ncbi.nlm.nih.gov/compound/91526879#section=2D-Structure
https://pubchem.ncbi.nlm.nih.gov/compound/91524386#section=2D-Structure
https://pubchem.ncbi.nlm.nih.gov/compound/91519883#section=2D-Structure
https://pubchem.ncbi.nlm.nih.gov/compound/91516001#section=2D-Structure
https://pubchem.ncbi.nlm.nih.gov/compound/91512313#section=2D-Structure
https://pubchem.ncbi.nlm.nih.gov/compound/91510630#section=2D-Structure
https://pubchem.ncbi.nlm.nih.gov/compound/91509079#section=2D-Structure
https://pubchem.ncbi.nlm.nih.gov/compound/91504338#section=2D-Structure
https://pubchem.ncbi.nlm.nih.gov/compound/91496656#section=2D-Structure
https://pubchem.ncbi.nlm.nih.gov/compound/91494069#section=2D-Structure
https://pubchem.ncbi.nlm.nih.gov/compound/91493639#section=2D-Structure
https://pubchem.ncbi.nlm.nih.gov/compound/91493551#section=2D-Structure
https://pubchem.ncbi.nlm.nih.gov/compound/91490023#section=2D-Structure
https://pubchem.ncbi.nlm.nih.gov/compound/91483#section=2D-Structure
https://pubchem.ncbi.nlm.nih.gov/compound/91482637#section=2D-Structure
https://pubchem.ncbi.nlm.nih.gov/compound/91482604#section=2D-Structure
https://pubchem.ncbi.nlm.nih.gov/compound/91481875#section=2D-Structure
https://pubchem.ncbi.nlm.nih.gov/compound/91477925#section=2D-Structure
https://pubchem.ncbi.nlm.nih.gov/compound/91473716#section=2D-Structure
https://pubchem.ncbi.nlm.nih.gov/compound/91470186#section=2D-Structure
https://pubchem.ncbi.nlm.nih.gov/compound/91467377#section=2D-Structure
https://pubchem.ncbi.nlm.nih.gov/compound/91465400#section=2D-Structure
https://pubchem.ncbi.nlm.nih.gov/compound/91464771#section=2D-Structure
https://pubchem.ncbi.nlm.nih.gov/compound/91464340#section=2D-Structure
https://pubchem.ncbi.nlm.nih.gov/compound/91463063#section=2D-Structure
https://pubchem.ncbi.nlm.nih.gov/compound/91460699#section=2D-Structure
https://pubchem.ncbi.nlm.nih.gov/compound/91457986#section=2D-Structure
https://pubchem.ncbi.nlm.nih.gov/compound/91456742#section=2D-Structure
https://pubchem.ncbi.nlm.nih.gov/compound/91455019#section=2D-Structure
https://pubchem.ncbi.nlm.nih.gov/compound/91452770#section=2D-Structure
https://pubchem.ncbi.nlm.nih.gov/compound/91449658#section=2D-Structure
https://pubchem.ncbi.nlm.nih.gov/compound/91448263#section=2D-Structure
https://pubchem.ncbi.nlm.nih.gov/compound/91447513#section=2D-Structure
https://pubchem.ncbi.nlm.nih.gov/compound/91439754#section=2D-Structure
https://pubchem.ncbi.nlm.nih.gov/compound/91435189#section=2D-Structure
https://pubchem.ncbi.nlm.nih.gov/compound/91435049#section=2D-Structure
https://pubchem.ncbi.nlm.nih.gov/compound/91434474#section=2D-Structure
https://pubchem.ncbi.nlm.nih.gov/compound/91433915#section=2D-Structure
https://pubchem.ncbi.nlm.nih.gov/compound/91433090#section=2D-Structure
https://pubchem.ncbi.nlm.nih.gov/compound/91428428#section=2D-Structure
https://pubchem.ncbi.nlm.nih.gov/compound/91426924#section=2D-Structure
https://pubchem.ncbi.nlm.nih.gov/compound/91426763#section=2D-Structure
https://pubchem.ncbi.nlm.nih.gov/compound/91420427#section=2D-Structure
https://pubchem.ncbi.nlm.nih.gov/compound/91419207#section=2D-Structure
https://pubchem.ncbi.nlm.nih.gov/compound/91416814#section=2D-Structure
https://pubchem.ncbi.nlm.nih.gov/compound/91416034#section=2D-Structure
https://pubchem.ncbi.nlm.nih.gov/compound/91415570#section=2D-Structure
https://pubchem.ncbi.nlm.nih.gov/compound/91413441#section=2D-Structure
https://pubchem.ncbi.nlm.nih.gov/compound/91406994#section=2D-Structure
https://pubchem.ncbi.nlm.nih.gov/compound/91406905#section=2D-Structure
https://pubchem.ncbi.nlm.nih.gov/compound/91398657#section=2D-Structure

https://pubchem.ncbi.nlm.nih.gov/compound/91398429#section=2D-Structure
https://pubchem.ncbi.nlm.nih.gov/compound/91395188#section=2D-Structure
https://pubchem.ncbi.nlm.nih.gov/compound/91392933#section=2D-Structure
https://pubchem.ncbi.nlm.nih.gov/compound/91391052#section=2D-Structure
https://pubchem.ncbi.nlm.nih.gov/compound/91389866#section=2D-Structure
https://pubchem.ncbi.nlm.nih.gov/compound/91386663#section=2D-Structure
https://pubchem.ncbi.nlm.nih.gov/compound/91386058#section=2D-Structure
https://pubchem.ncbi.nlm.nih.gov/compound/91384860#section=2D-Structure
https://pubchem.ncbi.nlm.nih.gov/compound/91381303#section=2D-Structure
https://pubchem.ncbi.nlm.nih.gov/compound/91379316#section=2D-Structure
https://pubchem.ncbi.nlm.nih.gov/compound/91370491#section=2D-Structure
https://pubchem.ncbi.nlm.nih.gov/compound/91368249#section=2D-Structure
https://pubchem.ncbi.nlm.nih.gov/compound/91367924#section=2D-Structure
https://pubchem.ncbi.nlm.nih.gov/compound/91366266#section=2D-Structure
https://pubchem.ncbi.nlm.nih.gov/compound/91363362#section=2D-Structure
https://pubchem.ncbi.nlm.nih.gov/compound/91362959#section=2D-Structure
https://pubchem.ncbi.nlm.nih.gov/compound/91362289#section=2D-Structure
https://pubchem.ncbi.nlm.nih.gov/compound/91361162#section=2D-Structure
https://pubchem.ncbi.nlm.nih.gov/compound/91360541#section=2D-Structure
https://pubchem.ncbi.nlm.nih.gov/compound/91357412#section=2D-Structure
https://pubchem.ncbi.nlm.nih.gov/compound/91354619#section=2D-Structure
https://pubchem.ncbi.nlm.nih.gov/compound/91353835#section=2D-Structure
https://pubchem.ncbi.nlm.nih.gov/compound/91353761#section=2D-Structure
https://pubchem.ncbi.nlm.nih.gov/compound/91352842#section=2D-Structure
https://pubchem.ncbi.nlm.nih.gov/compound/91348246#section=2D-Structure
https://pubchem.ncbi.nlm.nih.gov/compound/91345960#section=2D-Structure
https://pubchem.ncbi.nlm.nih.gov/compound/91343811#section=2D-Structure
https://pubchem.ncbi.nlm.nih.gov/compound/91336533#section=2D-Structure
https://pubchem.ncbi.nlm.nih.gov/compound/91334256#section=2D-Structure
https://pubchem.ncbi.nlm.nih.gov/compound/91333225#section=2D-Structure
https://pubchem.ncbi.nlm.nih.gov/compound/91332389#section=2D-Structure
https://pubchem.ncbi.nlm.nih.gov/compound/91329453#section=2D-Structure
https://pubchem.ncbi.nlm.nih.gov/compound/91329444#section=2D-Structure
https://pubchem.ncbi.nlm.nih.gov/compound/91327759#section=2D-Structure
https://pubchem.ncbi.nlm.nih.gov/compound/91327594#section=2D-Structure
https://pubchem.ncbi.nlm.nih.gov/compound/91327161#section=2D-Structure
https://pubchem.ncbi.nlm.nih.gov/compound/91321315#section=2D-Structure
https://pubchem.ncbi.nlm.nih.gov/compound/91318987#section=2D-Structure
https://pubchem.ncbi.nlm.nih.gov/compound/91316980#section=2D-Structure
https://pubchem.ncbi.nlm.nih.gov/compound/91315233#section=2D-Structure
https://pubchem.ncbi.nlm.nih.gov/compound/91305121#section=2D-Structure
https://pubchem.ncbi.nlm.nih.gov/compound/91303768#section=2D-Structure
https://pubchem.ncbi.nlm.nih.gov/compound/91301628#section=2D-Structure
https://pubchem.ncbi.nlm.nih.gov/compound/91299445#section=2D-Structure
https://pubchem.ncbi.nlm.nih.gov/compound/91294468#section=2D-Structure
https://pubchem.ncbi.nlm.nih.gov/compound/91293639#section=2D-Structure
https://pubchem.ncbi.nlm.nih.gov/compound/91293056#section=2D-Structure
https://pubchem.ncbi.nlm.nih.gov/compound/91292224#section=2D-Structure
https://pubchem.ncbi.nlm.nih.gov/compound/91289501#section=2D-Structure
https://pubchem.ncbi.nlm.nih.gov/compound/91288824#section=2D-Structure
https://pubchem.ncbi.nlm.nih.gov/compound/91286067#section=2D-Structure
https://pubchem.ncbi.nlm.nih.gov/compound/91283599#section=2D-Structure
https://pubchem.ncbi.nlm.nih.gov/compound/91281842#section=2D-Structure
https://pubchem.ncbi.nlm.nih.gov/compound/91281054#section=2D-Structure
https://pubchem.ncbi.nlm.nih.gov/compound/91277727#section=2D-Structure

https://pubchem.ncbi.nlm.nih.gov/compound/91275805#section=2D-Structure
https://pubchem.ncbi.nlm.nih.gov/compound/91275499#section=2D-Structure
https://pubchem.ncbi.nlm.nih.gov/compound/91275202#section=2D-Structure
https://pubchem.ncbi.nlm.nih.gov/compound/91271455#section=2D-Structure
https://pubchem.ncbi.nlm.nih.gov/compound/91270254#section=2D-Structure
https://pubchem.ncbi.nlm.nih.gov/compound/91269233#section=2D-Structure
https://pubchem.ncbi.nlm.nih.gov/compound/91267301#section=2D-Structure
https://pubchem.ncbi.nlm.nih.gov/compound/91266198#section=2D-Structure
https://pubchem.ncbi.nlm.nih.gov/compound/91263635#section=2D-Structure
https://pubchem.ncbi.nlm.nih.gov/compound/91263468#section=2D-Structure
https://pubchem.ncbi.nlm.nih.gov/compound/91259842#section=2D-Structure
https://pubchem.ncbi.nlm.nih.gov/compound/91259305#section=2D-Structure
https://pubchem.ncbi.nlm.nih.gov/compound/91257235#section=2D-Structure
https://pubchem.ncbi.nlm.nih.gov/compound/91254179#section=2D-Structure
https://pubchem.ncbi.nlm.nih.gov/compound/91253649#section=2D-Structure
https://pubchem.ncbi.nlm.nih.gov/compound/91253592#section=2D-Structure
https://pubchem.ncbi.nlm.nih.gov/compound/91252918#section=2D-Structure
https://pubchem.ncbi.nlm.nih.gov/compound/91251546#section=2D-Structure
https://pubchem.ncbi.nlm.nih.gov/compound/91251228#section=2D-Structure
https://pubchem.ncbi.nlm.nih.gov/compound/91251042#section=2D-Structure
https://pubchem.ncbi.nlm.nih.gov/compound/91250019#section=2D-Structure
https://pubchem.ncbi.nlm.nih.gov/compound/91247829#section=2D-Structure
https://pubchem.ncbi.nlm.nih.gov/compound/91243009#section=2D-Structure
https://pubchem.ncbi.nlm.nih.gov/compound/91241228#section=2D-Structure
https://pubchem.ncbi.nlm.nih.gov/compound/91240707#section=2D-Structure
https://pubchem.ncbi.nlm.nih.gov/compound/91238377#section=2D-Structure
https://pubchem.ncbi.nlm.nih.gov/compound/91236375#section=2D-Structure
https://pubchem.ncbi.nlm.nih.gov/compound/91235946#section=2D-Structure
https://pubchem.ncbi.nlm.nih.gov/compound/91235732#section=2D-Structure
https://pubchem.ncbi.nlm.nih.gov/compound/91234785#section=2D-Structure
https://pubchem.ncbi.nlm.nih.gov/compound/91233666#section=2D-Structure
https://pubchem.ncbi.nlm.nih.gov/compound/91230476#section=2D-Structure
https://pubchem.ncbi.nlm.nih.gov/compound/91230251#section=2D-Structure
https://pubchem.ncbi.nlm.nih.gov/compound/91227420#section=2D-Structure
https://pubchem.ncbi.nlm.nih.gov/compound/91226789#section=2D-Structure
https://pubchem.ncbi.nlm.nih.gov/compound/91226724#section=2D-Structure
https://pubchem.ncbi.nlm.nih.gov/compound/91225692#section=2D-Structure
https://pubchem.ncbi.nlm.nih.gov/compound/91221411#section=2D-Structure
https://pubchem.ncbi.nlm.nih.gov/compound/91213439#section=2D-Structure
https://pubchem.ncbi.nlm.nih.gov/compound/91213373#section=2D-Structure
https://pubchem.ncbi.nlm.nih.gov/compound/91209882#section=2D-Structure
https://pubchem.ncbi.nlm.nih.gov/compound/91201298#section=2D-Structure
https://pubchem.ncbi.nlm.nih.gov/compound/91197563#section=2D-Structure
https://pubchem.ncbi.nlm.nih.gov/compound/91196679#section=2D-Structure
https://pubchem.ncbi.nlm.nih.gov/compound/91196146#section=2D-Structure
https://pubchem.ncbi.nlm.nih.gov/compound/91193258#section=2D-Structure
https://pubchem.ncbi.nlm.nih.gov/compound/91192322#section=2D-Structure
https://pubchem.ncbi.nlm.nih.gov/compound/91191206#section=2D-Structure
https://pubchem.ncbi.nlm.nih.gov/compound/91190098#section=2D-Structure
https://pubchem.ncbi.nlm.nih.gov/compound/91187152#section=2D-Structure
https://pubchem.ncbi.nlm.nih.gov/compound/91186536#section=2D-Structure
https://pubchem.ncbi.nlm.nih.gov/compound/91185584#section=2D-Structure
https://pubchem.ncbi.nlm.nih.gov/compound/91183051#section=2D-Structure
https://pubchem.ncbi.nlm.nih.gov/compound/91182328#section=2D-Structure
https://pubchem.ncbi.nlm.nih.gov/compound/91181429#section=2D-Structure

https://pubchem.ncbi.nlm.nih.gov/compound/91179849#section=2D-Structure
https://pubchem.ncbi.nlm.nih.gov/compound/91178973#section=2D-Structure
https://pubchem.ncbi.nlm.nih.gov/compound/91178513#section=2D-Structure
https://pubchem.ncbi.nlm.nih.gov/compound/91177477#section=2D-Structure
https://pubchem.ncbi.nlm.nih.gov/compound/91176491#section=2D-Structure
https://pubchem.ncbi.nlm.nih.gov/compound/91176376#section=2D-Structure
https://pubchem.ncbi.nlm.nih.gov/compound/91175854#section=2D-Structure
https://pubchem.ncbi.nlm.nih.gov/compound/91167341#section=2D-Structure
https://pubchem.ncbi.nlm.nih.gov/compound/91165144#section=2D-Structure
https://pubchem.ncbi.nlm.nih.gov/compound/91164891#section=2D-Structure
https://pubchem.ncbi.nlm.nih.gov/compound/91163793#section=2D-Structure
https://pubchem.ncbi.nlm.nih.gov/compound/91162091#section=2D-Structure
https://pubchem.ncbi.nlm.nih.gov/compound/91158672#section=2D-Structure
https://pubchem.ncbi.nlm.nih.gov/compound/91152673#section=2D-Structure
https://pubchem.ncbi.nlm.nih.gov/compound/91150386#section=2D-Structure
https://pubchem.ncbi.nlm.nih.gov/compound/91146915#section=2D-Structure
https://pubchem.ncbi.nlm.nih.gov/compound/91144350#section=2D-Structure
https://pubchem.ncbi.nlm.nih.gov/compound/91142700#section=2D-Structure
https://pubchem.ncbi.nlm.nih.gov/compound/91141533#section=2D-Structure
https://pubchem.ncbi.nlm.nih.gov/compound/91138572#section=2D-Structure
https://pubchem.ncbi.nlm.nih.gov/compound/91136112#section=2D-Structure
https://pubchem.ncbi.nlm.nih.gov/compound/91134284#section=2D-Structure
https://pubchem.ncbi.nlm.nih.gov/compound/91132231#section=2D-Structure
https://pubchem.ncbi.nlm.nih.gov/compound/91129785#section=2D-Structure
https://pubchem.ncbi.nlm.nih.gov/compound/91129745#section=2D-Structure
https://pubchem.ncbi.nlm.nih.gov/compound/91128582#section=2D-Structure
https://pubchem.ncbi.nlm.nih.gov/compound/91127892#section=2D-Structure
https://pubchem.ncbi.nlm.nih.gov/compound/91123912#section=2D-Structure
https://pubchem.ncbi.nlm.nih.gov/compound/91121499#section=2D-Structure
https://pubchem.ncbi.nlm.nih.gov/compound/91121121#section=2D-Structure
https://pubchem.ncbi.nlm.nih.gov/compound/91120980#section=2D-Structure
https://pubchem.ncbi.nlm.nih.gov/compound/91120373#section=2D-Structure
https://pubchem.ncbi.nlm.nih.gov/compound/91118462#section=2D-Structure
https://pubchem.ncbi.nlm.nih.gov/compound/91112452#section=2D-Structure
https://pubchem.ncbi.nlm.nih.gov/compound/91111646#section=2D-Structure
https://pubchem.ncbi.nlm.nih.gov/compound/91108234#section=2D-Structure
https://pubchem.ncbi.nlm.nih.gov/compound/91107916#section=2D-Structure
https://pubchem.ncbi.nlm.nih.gov/compound/91105845#section=2D-Structure
https://pubchem.ncbi.nlm.nih.gov/compound/91102410#section=2D-Structure
https://pubchem.ncbi.nlm.nih.gov/compound/91093592#section=2D-Structure
https://pubchem.ncbi.nlm.nih.gov/compound/91093326#section=2D-Structure
https://pubchem.ncbi.nlm.nih.gov/compound/91092592#section=2D-Structure
https://pubchem.ncbi.nlm.nih.gov/compound/91089129#section=2D-Structure
https://pubchem.ncbi.nlm.nih.gov/compound/91087728#section=2D-Structure
https://pubchem.ncbi.nlm.nih.gov/compound/91086986#section=2D-Structure
https://pubchem.ncbi.nlm.nih.gov/compound/91086080#section=2D-Structure
https://pubchem.ncbi.nlm.nih.gov/compound/91084897#section=2D-Structure
https://pubchem.ncbi.nlm.nih.gov/compound/91084393#section=2D-Structure
https://pubchem.ncbi.nlm.nih.gov/compound/91074168#section=2D-Structure
https://pubchem.ncbi.nlm.nih.gov/compound/91071820#section=2D-Structure
https://pubchem.ncbi.nlm.nih.gov/compound/91067073#section=2D-Structure
https://pubchem.ncbi.nlm.nih.gov/compound/91061887#section=2D-Structure
https://pubchem.ncbi.nlm.nih.gov/compound/91052180#section=2D-Structure
https://pubchem.ncbi.nlm.nih.gov/compound/91050521#section=2D-Structure
https://pubchem.ncbi.nlm.nih.gov/compound/91049855#section=2D-Structure

https://pubchem.ncbi.nlm.nih.gov/compound/91037686#section=2D-Structure
https://pubchem.ncbi.nlm.nih.gov/compound/91033182#section=2D-Structure
https://pubchem.ncbi.nlm.nih.gov/compound/91029854#section=2D-Structure
https://pubchem.ncbi.nlm.nih.gov/compound/91029016#section=2D-Structure
https://pubchem.ncbi.nlm.nih.gov/compound/91026379#section=2D-Structure
https://pubchem.ncbi.nlm.nih.gov/compound/91024706#section=2D-Structure
https://pubchem.ncbi.nlm.nih.gov/compound/91017142#section=2D-Structure
https://pubchem.ncbi.nlm.nih.gov/compound/91005415#section=2D-Structure
https://pubchem.ncbi.nlm.nih.gov/compound/91004519#section=2D-Structure
https://pubchem.ncbi.nlm.nih.gov/compound/91001079#section=2D-Structure
https://pubchem.ncbi.nlm.nih.gov/compound/90998231#section=2D-Structure
https://pubchem.ncbi.nlm.nih.gov/compound/90996991#section=2D-Structure
https://pubchem.ncbi.nlm.nih.gov/compound/90990129#section=2D-Structure
https://pubchem.ncbi.nlm.nih.gov/compound/90989910#section=2D-Structure
https://pubchem.ncbi.nlm.nih.gov/compound/90989033#section=2D-Structure
https://pubchem.ncbi.nlm.nih.gov/compound/90983910#section=2D-Structure
https://pubchem.ncbi.nlm.nih.gov/compound/90983779#section=2D-Structure
https://pubchem.ncbi.nlm.nih.gov/compound/90982911#section=2D-Structure
https://pubchem.ncbi.nlm.nih.gov/compound/90982059#section=2D-Structure
https://pubchem.ncbi.nlm.nih.gov/compound/90980354#section=2D-Structure
https://pubchem.ncbi.nlm.nih.gov/compound/90976421#section=2D-Structure
https://pubchem.ncbi.nlm.nih.gov/compound/90976176#section=2D-Structure
https://pubchem.ncbi.nlm.nih.gov/compound/90976071#section=2D-Structure
https://pubchem.ncbi.nlm.nih.gov/compound/90976004#section=2D-Structure
https://pubchem.ncbi.nlm.nih.gov/compound/90972491#section=2D-Structure
https://pubchem.ncbi.nlm.nih.gov/compound/90970400#section=2D-Structure
https://pubchem.ncbi.nlm.nih.gov/compound/90966220#section=2D-Structure
https://pubchem.ncbi.nlm.nih.gov/compound/90961594#section=2D-Structure
https://pubchem.ncbi.nlm.nih.gov/compound/90959311#section=2D-Structure
https://pubchem.ncbi.nlm.nih.gov/compound/90953475#section=2D-Structure
https://pubchem.ncbi.nlm.nih.gov/compound/90948612#section=2D-Structure
https://pubchem.ncbi.nlm.nih.gov/compound/90947803#section=2D-Structure
https://pubchem.ncbi.nlm.nih.gov/compound/90946057#section=2D-Structure
https://pubchem.ncbi.nlm.nih.gov/compound/90945867#section=2D-Structure
https://pubchem.ncbi.nlm.nih.gov/compound/90945267#section=2D-Structure
https://pubchem.ncbi.nlm.nih.gov/compound/90944820#section=2D-Structure
https://pubchem.ncbi.nlm.nih.gov/compound/90941361#section=2D-Structure
https://pubchem.ncbi.nlm.nih.gov/compound/90940858#section=2D-Structure
https://pubchem.ncbi.nlm.nih.gov/compound/90938062#section=2D-Structure
https://pubchem.ncbi.nlm.nih.gov/compound/90937933#section=2D-Structure
https://pubchem.ncbi.nlm.nih.gov/compound/90937619#section=2D-Structure
https://pubchem.ncbi.nlm.nih.gov/compound/90936003#section=2D-Structure
https://pubchem.ncbi.nlm.nih.gov/compound/90935897#section=2D-Structure
https://pubchem.ncbi.nlm.nih.gov/compound/90935279#section=2D-Structure
https://pubchem.ncbi.nlm.nih.gov/compound/90930141#section=2D-Structure
https://pubchem.ncbi.nlm.nih.gov/compound/90927513#section=2D-Structure
https://pubchem.ncbi.nlm.nih.gov/compound/90920998#section=2D-Structure
https://pubchem.ncbi.nlm.nih.gov/compound/90917717#section=2D-Structure
https://pubchem.ncbi.nlm.nih.gov/compound/90910845#section=2D-Structure
https://pubchem.ncbi.nlm.nih.gov/compound/90908781#section=2D-Structure
https://pubchem.ncbi.nlm.nih.gov/compound/90907581#section=2D-Structure
https://pubchem.ncbi.nlm.nih.gov/compound/90907509#section=2D-Structure
https://pubchem.ncbi.nlm.nih.gov/compound/90906197#section=2D-Structure
https://pubchem.ncbi.nlm.nih.gov/compound/90904928#section=2D-Structure
https://pubchem.ncbi.nlm.nih.gov/compound/90903948#section=2D-Structure

https://pubchem.ncbi.nlm.nih.gov/compound/90898342#section=2D-Structure
https://pubchem.ncbi.nlm.nih.gov/compound/90897660#section=2D-Structure
https://pubchem.ncbi.nlm.nih.gov/compound/90896318#section=2D-Structure
https://pubchem.ncbi.nlm.nih.gov/compound/90887036#section=2D-Structure
https://pubchem.ncbi.nlm.nih.gov/compound/90886209#section=2D-Structure
https://pubchem.ncbi.nlm.nih.gov/compound/90881706#section=2D-Structure
https://pubchem.ncbi.nlm.nih.gov/compound/90877010#section=2D-Structure
https://pubchem.ncbi.nlm.nih.gov/compound/90875987#section=2D-Structure
https://pubchem.ncbi.nlm.nih.gov/compound/90875933#section=2D-Structure
https://pubchem.ncbi.nlm.nih.gov/compound/90873060#section=2D-Structure
https://pubchem.ncbi.nlm.nih.gov/compound/90872123#section=2D-Structure
https://pubchem.ncbi.nlm.nih.gov/compound/90871730#section=2D-Structure
https://pubchem.ncbi.nlm.nih.gov/compound/90865755#section=2D-Structure
https://pubchem.ncbi.nlm.nih.gov/compound/90860593#section=2D-Structure
https://pubchem.ncbi.nlm.nih.gov/compound/90859824#section=2D-Structure
https://pubchem.ncbi.nlm.nih.gov/compound/90859340#section=2D-Structure
https://pubchem.ncbi.nlm.nih.gov/compound/90857940#section=2D-Structure
https://pubchem.ncbi.nlm.nih.gov/compound/90857366#section=2D-Structure
https://pubchem.ncbi.nlm.nih.gov/compound/90856802#section=2D-Structure
https://pubchem.ncbi.nlm.nih.gov/compound/90856787#section=2D-Structure
https://pubchem.ncbi.nlm.nih.gov/compound/90854531#section=2D-Structure
https://pubchem.ncbi.nlm.nih.gov/compound/90852821#section=2D-Structure
https://pubchem.ncbi.nlm.nih.gov/compound/90851900#section=2D-Structure
https://pubchem.ncbi.nlm.nih.gov/compound/90851721#section=2D-Structure
https://pubchem.ncbi.nlm.nih.gov/compound/90850254#section=2D-Structure
https://pubchem.ncbi.nlm.nih.gov/compound/90849659#section=2D-Structure
https://pubchem.ncbi.nlm.nih.gov/compound/90848626#section=2D-Structure
https://pubchem.ncbi.nlm.nih.gov/compound/90846222#section=2D-Structure
https://pubchem.ncbi.nlm.nih.gov/compound/90846024#section=2D-Structure
https://pubchem.ncbi.nlm.nih.gov/compound/90842950#section=2D-Structure
https://pubchem.ncbi.nlm.nih.gov/compound/90841098#section=2D-Structure
https://pubchem.ncbi.nlm.nih.gov/compound/90828445#section=2D-Structure
https://pubchem.ncbi.nlm.nih.gov/compound/90822137#section=2D-Structure
https://pubchem.ncbi.nlm.nih.gov/compound/90821076#section=2D-Structure
https://pubchem.ncbi.nlm.nih.gov/compound/90817120#section=2D-Structure
https://pubchem.ncbi.nlm.nih.gov/compound/90813733#section=2D-Structure
https://pubchem.ncbi.nlm.nih.gov/compound/90811808#section=2D-Structure
https://pubchem.ncbi.nlm.nih.gov/compound/90811517#section=2D-Structure
https://pubchem.ncbi.nlm.nih.gov/compound/90811281#section=2D-Structure
https://pubchem.ncbi.nlm.nih.gov/compound/90811047#section=2D-Structure
https://pubchem.ncbi.nlm.nih.gov/compound/90810347#section=2D-Structure
https://pubchem.ncbi.nlm.nih.gov/compound/90809487#section=2D-Structure
https://pubchem.ncbi.nlm.nih.gov/compound/90807289#section=2D-Structure
https://pubchem.ncbi.nlm.nih.gov/compound/90804239#section=2D-Structure
https://pubchem.ncbi.nlm.nih.gov/compound/90801310#section=2D-Structure
https://pubchem.ncbi.nlm.nih.gov/compound/90800922#section=2D-Structure
https://pubchem.ncbi.nlm.nih.gov/compound/90797566#section=2D-Structure
https://pubchem.ncbi.nlm.nih.gov/compound/90797553#section=2D-Structure
https://pubchem.ncbi.nlm.nih.gov/compound/90796608#section=2D-Structure
https://pubchem.ncbi.nlm.nih.gov/compound/90796407#section=2D-Structure
https://pubchem.ncbi.nlm.nih.gov/compound/90790060#section=2D-Structure
https://pubchem.ncbi.nlm.nih.gov/compound/90789133#section=2D-Structure
https://pubchem.ncbi.nlm.nih.gov/compound/90786657#section=2D-Structure
https://pubchem.ncbi.nlm.nih.gov/compound/90785123#section=2D-Structure
https://pubchem.ncbi.nlm.nih.gov/compound/90782894#section=2D-Structure

https://pubchem.ncbi.nlm.nih.gov/compound/90781736#section=2D-Structure
https://pubchem.ncbi.nlm.nih.gov/compound/90780831#section=2D-Structure
https://pubchem.ncbi.nlm.nih.gov/compound/90777666#section=2D-Structure
https://pubchem.ncbi.nlm.nih.gov/compound/90773412#section=2D-Structure
https://pubchem.ncbi.nlm.nih.gov/compound/90770720#section=2D-Structure
https://pubchem.ncbi.nlm.nih.gov/compound/90768295#section=2D-Structure
https://pubchem.ncbi.nlm.nih.gov/compound/90767960#section=2D-Structure
https://pubchem.ncbi.nlm.nih.gov/compound/90766995#section=2D-Structure
https://pubchem.ncbi.nlm.nih.gov/compound/90765970#section=2D-Structure
https://pubchem.ncbi.nlm.nih.gov/compound/90761700#section=2D-Structure
https://pubchem.ncbi.nlm.nih.gov/compound/90757766#section=2D-Structure
https://pubchem.ncbi.nlm.nih.gov/compound/90755317#section=2D-Structure
https://pubchem.ncbi.nlm.nih.gov/compound/90752861#section=2D-Structure
https://pubchem.ncbi.nlm.nih.gov/compound/90747193#section=2D-Structure
https://pubchem.ncbi.nlm.nih.gov/compound/90747063#section=2D-Structure
https://pubchem.ncbi.nlm.nih.gov/compound/90744728#section=2D-Structure
https://pubchem.ncbi.nlm.nih.gov/compound/90742669#section=2D-Structure
https://pubchem.ncbi.nlm.nih.gov/compound/90741337#section=2D-Structure
https://pubchem.ncbi.nlm.nih.gov/compound/90740560#section=2D-Structure
https://pubchem.ncbi.nlm.nih.gov/compound/90738891#section=2D-Structure
https://pubchem.ncbi.nlm.nih.gov/compound/90736922#section=2D-Structure
https://pubchem.ncbi.nlm.nih.gov/compound/90736772#section=2D-Structure
https://pubchem.ncbi.nlm.nih.gov/compound/90736006#section=2D-Structure
https://pubchem.ncbi.nlm.nih.gov/compound/90732379#section=2D-Structure
https://pubchem.ncbi.nlm.nih.gov/compound/90732332#section=2D-Structure
https://pubchem.ncbi.nlm.nih.gov/compound/90730960#section=2D-Structure
https://pubchem.ncbi.nlm.nih.gov/compound/90728000#section=2D-Structure
https://pubchem.ncbi.nlm.nih.gov/compound/90727837#section=2D-Structure
https://pubchem.ncbi.nlm.nih.gov/compound/90727464#section=2D-Structure
https://pubchem.ncbi.nlm.nih.gov/compound/90721449#section=2D-Structure
https://pubchem.ncbi.nlm.nih.gov/compound/90721137#section=2D-Structure
https://pubchem.ncbi.nlm.nih.gov/compound/90720589#section=2D-Structure
https://pubchem.ncbi.nlm.nih.gov/compound/90720002#section=2D-Structure
https://pubchem.ncbi.nlm.nih.gov/compound/90715251#section=2D-Structure
https://pubchem.ncbi.nlm.nih.gov/compound/90713624#section=2D-Structure
https://pubchem.ncbi.nlm.nih.gov/compound/90712963#section=2D-Structure
https://pubchem.ncbi.nlm.nih.gov/compound/90703288#section=2D-Structure
https://pubchem.ncbi.nlm.nih.gov/compound/90701951#section=2D-Structure
https://pubchem.ncbi.nlm.nih.gov/compound/90697982#section=2D-Structure
https://pubchem.ncbi.nlm.nih.gov/compound/90697879#section=2D-Structure
https://pubchem.ncbi.nlm.nih.gov/compound/90697792#section=2D-Structure
https://pubchem.ncbi.nlm.nih.gov/compound/90693171#section=2D-Structure
https://pubchem.ncbi.nlm.nih.gov/compound/90691443#section=2D-Structure
https://pubchem.ncbi.nlm.nih.gov/compound/90685183#section=2D-Structure
https://pubchem.ncbi.nlm.nih.gov/compound/90487925#section=2D-Structure
https://pubchem.ncbi.nlm.nih.gov/compound/90487809#section=2D-Structure
https://pubchem.ncbi.nlm.nih.gov/compound/90487698#section=2D-Structure
https://pubchem.ncbi.nlm.nih.gov/compound/90487638#section=2D-Structure
https://pubchem.ncbi.nlm.nih.gov/compound/90487522#section=2D-Structure
https://pubchem.ncbi.nlm.nih.gov/compound/90482663#section=2D-Structure
https://pubchem.ncbi.nlm.nih.gov/compound/90482654#section=2D-Structure
https://pubchem.ncbi.nlm.nih.gov/compound/90482343#section=2D-Structure
https://pubchem.ncbi.nlm.nih.gov/compound/90473894#section=2D-Structure
https://pubchem.ncbi.nlm.nih.gov/compound/90473472#section=2D-Structure
https://pubchem.ncbi.nlm.nih.gov/compound/90473094#section=2D-Structure

https://pubchem.ncbi.nlm.nih.gov/compound/90472786#section=2D-Structure
https://pubchem.ncbi.nlm.nih.gov/compound/90471704#section=2D-Structure
https://pubchem.ncbi.nlm.nih.gov/compound/90470777#section=2D-Structure
https://pubchem.ncbi.nlm.nih.gov/compound/90469985#section=2D-Structure
https://pubchem.ncbi.nlm.nih.gov/compound/90462868#section=2D-Structure
https://pubchem.ncbi.nlm.nih.gov/compound/90462866#section=2D-Structure
https://pubchem.ncbi.nlm.nih.gov/compound/90455831#section=2D-Structure
https://pubchem.ncbi.nlm.nih.gov/compound/90455287#section=2D-Structure
https://pubchem.ncbi.nlm.nih.gov/compound/90455274#section=2D-Structure
https://pubchem.ncbi.nlm.nih.gov/compound/90443792#section=2D-Structure
https://pubchem.ncbi.nlm.nih.gov/compound/90435272#section=2D-Structure
https://pubchem.ncbi.nlm.nih.gov/compound/90399150#section=2D-Structure
https://pubchem.ncbi.nlm.nih.gov/compound/90399132#section=2D-Structure
https://pubchem.ncbi.nlm.nih.gov/compound/90375143#section=2D-Structure
https://pubchem.ncbi.nlm.nih.gov/compound/90359340#section=2D-Structure
https://pubchem.ncbi.nlm.nih.gov/compound/90358506#section=2D-Structure
https://pubchem.ncbi.nlm.nih.gov/compound/90357835#section=2D-Structure
https://pubchem.ncbi.nlm.nih.gov/compound/90354355#section=2D-Structure
https://pubchem.ncbi.nlm.nih.gov/compound/90350832#section=2D-Structure
https://pubchem.ncbi.nlm.nih.gov/compound/90334628#section=2D-Structure
https://pubchem.ncbi.nlm.nih.gov/compound/90328964#section=2D-Structure
https://pubchem.ncbi.nlm.nih.gov/compound/90328574#section=2D-Structure
https://pubchem.ncbi.nlm.nih.gov/compound/90328568#section=2D-Structure
https://pubchem.ncbi.nlm.nih.gov/compound/90328566#section=2D-Structure
https://pubchem.ncbi.nlm.nih.gov/compound/90327360#section=2D-Structure
https://pubchem.ncbi.nlm.nih.gov/compound/90327359#section=2D-Structure
https://pubchem.ncbi.nlm.nih.gov/compound/90325341#section=2D-Structure
https://pubchem.ncbi.nlm.nih.gov/compound/90322109#section=2D-Structure
https://pubchem.ncbi.nlm.nih.gov/compound/90321089#section=2D-Structure
https://pubchem.ncbi.nlm.nih.gov/compound/90317355#section=2D-Structure
https://pubchem.ncbi.nlm.nih.gov/compound/90315707#section=2D-Structure
https://pubchem.ncbi.nlm.nih.gov/compound/90315702#section=2D-Structure
https://pubchem.ncbi.nlm.nih.gov/compound/90307898#section=2D-Structure
https://pubchem.ncbi.nlm.nih.gov/compound/90298429#section=2D-Structure
https://pubchem.ncbi.nlm.nih.gov/compound/90295322#section=2D-Structure
https://pubchem.ncbi.nlm.nih.gov/compound/90289988#section=2D-Structure
https://pubchem.ncbi.nlm.nih.gov/compound/90281624#section=2D-Structure
https://pubchem.ncbi.nlm.nih.gov/compound/90281618#section=2D-Structure
https://pubchem.ncbi.nlm.nih.gov/compound/90269055#section=2D-Structure
https://pubchem.ncbi.nlm.nih.gov/compound/90259986#section=2D-Structure
https://pubchem.ncbi.nlm.nih.gov/compound/90254144#section=2D-Structure
https://pubchem.ncbi.nlm.nih.gov/compound/90251004#section=2D-Structure
https://pubchem.ncbi.nlm.nih.gov/compound/90230305#section=2D-Structure
https://pubchem.ncbi.nlm.nih.gov/compound/90225337#section=2D-Structure
https://pubchem.ncbi.nlm.nih.gov/compound/90219376#section=2D-Structure
https://pubchem.ncbi.nlm.nih.gov/compound/90207005#section=2D-Structure
https://pubchem.ncbi.nlm.nih.gov/compound/90206883#section=2D-Structure
https://pubchem.ncbi.nlm.nih.gov/compound/90202100#section=2D-Structure
https://pubchem.ncbi.nlm.nih.gov/compound/90195802#section=2D-Structure
https://pubchem.ncbi.nlm.nih.gov/compound/90189979#section=2D-Structure
https://pubchem.ncbi.nlm.nih.gov/compound/90189622#section=2D-Structure
https://pubchem.ncbi.nlm.nih.gov/compound/90167756#section=2D-Structure
https://pubchem.ncbi.nlm.nih.gov/compound/90167742#section=2D-Structure
https://pubchem.ncbi.nlm.nih.gov/compound/90158108#section=2D-Structure
https://pubchem.ncbi.nlm.nih.gov/compound/90153387#section=2D-Structure

https://pubchem.ncbi.nlm.nih.gov/compound/90148870#section=2D-Structure
https://pubchem.ncbi.nlm.nih.gov/compound/90147196#section=2D-Structure
https://pubchem.ncbi.nlm.nih.gov/compound/90141895#section=2D-Structure
https://pubchem.ncbi.nlm.nih.gov/compound/90133718#section=2D-Structure
https://pubchem.ncbi.nlm.nih.gov/compound/90133694#section=2D-Structure
https://pubchem.ncbi.nlm.nih.gov/compound/90133584#section=2D-Structure
https://pubchem.ncbi.nlm.nih.gov/compound/90103923#section=2D-Structure
https://pubchem.ncbi.nlm.nih.gov/compound/90103921#section=2D-Structure
https://pubchem.ncbi.nlm.nih.gov/compound/90101568#section=2D-Structure
https://pubchem.ncbi.nlm.nih.gov/compound/90097367#section=2D-Structure
https://pubchem.ncbi.nlm.nih.gov/compound/90091163#section=2D-Structure
https://pubchem.ncbi.nlm.nih.gov/compound/90091153#section=2D-Structure
https://pubchem.ncbi.nlm.nih.gov/compound/90091151#section=2D-Structure
https://pubchem.ncbi.nlm.nih.gov/compound/90091150#section=2D-Structure
https://pubchem.ncbi.nlm.nih.gov/compound/90090573#section=2D-Structure
https://pubchem.ncbi.nlm.nih.gov/compound/90084901#section=2D-Structure
https://pubchem.ncbi.nlm.nih.gov/compound/90084896#section=2D-Structure
https://pubchem.ncbi.nlm.nih.gov/compound/90084672#section=2D-Structure
https://pubchem.ncbi.nlm.nih.gov/compound/90084391#section=2D-Structure
https://pubchem.ncbi.nlm.nih.gov/compound/90084190#section=2D-Structure
https://pubchem.ncbi.nlm.nih.gov/compound/90079408#section=2D-Structure
https://pubchem.ncbi.nlm.nih.gov/compound/90079152#section=2D-Structure
https://pubchem.ncbi.nlm.nih.gov/compound/90073204#section=2D-Structure
https://pubchem.ncbi.nlm.nih.gov/compound/90070959#section=2D-Structure
https://pubchem.ncbi.nlm.nih.gov/compound/90048310#section=2D-Structure
https://pubchem.ncbi.nlm.nih.gov/compound/90043264#section=2D-Structure
https://pubchem.ncbi.nlm.nih.gov/compound/90028829#section=2D-Structure
https://pubchem.ncbi.nlm.nih.gov/compound/90014803#section=2D-Structure
https://pubchem.ncbi.nlm.nih.gov/compound/90008905#section=2D-Structure
https://pubchem.ncbi.nlm.nih.gov/compound/90003835#section=2D-Structure
https://pubchem.ncbi.nlm.nih.gov/compound/90003823#section=2D-Structure
https://pubchem.ncbi.nlm.nih.gov/compound/90003739#section=2D-Structure
https://pubchem.ncbi.nlm.nih.gov/compound/90003647#section=2D-Structure
https://pubchem.ncbi.nlm.nih.gov/compound/90003520#section=2D-Structure
https://pubchem.ncbi.nlm.nih.gov/compound/90003327#section=2D-Structure
https://pubchem.ncbi.nlm.nih.gov/compound/90001610#section=2D-Structure
https://pubchem.ncbi.nlm.nih.gov/compound/90000481#section=2D-Structure
https://pubchem.ncbi.nlm.nih.gov/compound/89997165#section=2D-Structure
https://pubchem.ncbi.nlm.nih.gov/compound/89981502#section=2D-Structure
https://pubchem.ncbi.nlm.nih.gov/compound/89972262#section=2D-Structure
https://pubchem.ncbi.nlm.nih.gov/compound/89972204#section=2D-Structure
https://pubchem.ncbi.nlm.nih.gov/compound/89964995#section=2D-Structure
https://pubchem.ncbi.nlm.nih.gov/compound/89947085#section=2D-Structure
https://pubchem.ncbi.nlm.nih.gov/compound/89935337#section=2D-Structure
https://pubchem.ncbi.nlm.nih.gov/compound/89935333#section=2D-Structure
https://pubchem.ncbi.nlm.nih.gov/compound/89932486#section=2D-Structure
https://pubchem.ncbi.nlm.nih.gov/compound/89916757#section=2D-Structure
https://pubchem.ncbi.nlm.nih.gov/compound/89904789#section=2D-Structure
https://pubchem.ncbi.nlm.nih.gov/compound/89904788#section=2D-Structure
https://pubchem.ncbi.nlm.nih.gov/compound/89900804#section=2D-Structure
https://pubchem.ncbi.nlm.nih.gov/compound/89888273#section=2D-Structure
https://pubchem.ncbi.nlm.nih.gov/compound/89884033#section=2D-Structure
https://pubchem.ncbi.nlm.nih.gov/compound/89858932#section=2D-Structure
https://pubchem.ncbi.nlm.nih.gov/compound/89854653#section=2D-Structure
https://pubchem.ncbi.nlm.nih.gov/compound/89854208#section=2D-Structure

https://pubchem.ncbi.nlm.nih.gov/compound/89852652#section=2D-Structure
https://pubchem.ncbi.nlm.nih.gov/compound/89849121#section=2D-Structure
https://pubchem.ncbi.nlm.nih.gov/compound/89849120#section=2D-Structure
https://pubchem.ncbi.nlm.nih.gov/compound/89849119#section=2D-Structure
https://pubchem.ncbi.nlm.nih.gov/compound/89836535#section=2D-Structure
https://pubchem.ncbi.nlm.nih.gov/compound/89823026#section=2D-Structure
https://pubchem.ncbi.nlm.nih.gov/compound/89821465#section=2D-Structure
https://pubchem.ncbi.nlm.nih.gov/compound/89820008#section=2D-Structure
https://pubchem.ncbi.nlm.nih.gov/compound/89809139#section=2D-Structure
https://pubchem.ncbi.nlm.nih.gov/compound/89798144#section=2D-Structure
https://pubchem.ncbi.nlm.nih.gov/compound/89795953#section=2D-Structure
https://pubchem.ncbi.nlm.nih.gov/compound/89774149#section=2D-Structure
https://pubchem.ncbi.nlm.nih.gov/compound/89771372#section=2D-Structure
https://pubchem.ncbi.nlm.nih.gov/compound/89766951#section=2D-Structure
https://pubchem.ncbi.nlm.nih.gov/compound/89761088#section=2D-Structure
https://pubchem.ncbi.nlm.nih.gov/compound/89752233#section=2D-Structure
https://pubchem.ncbi.nlm.nih.gov/compound/89729448#section=2D-Structure
https://pubchem.ncbi.nlm.nih.gov/compound/89718151#section=2D-Structure
https://pubchem.ncbi.nlm.nih.gov/compound/89701472#section=2D-Structure
https://pubchem.ncbi.nlm.nih.gov/compound/89678132#section=2D-Structure
https://pubchem.ncbi.nlm.nih.gov/compound/89670464#section=2D-Structure
https://pubchem.ncbi.nlm.nih.gov/compound/89657394#section=2D-Structure
https://pubchem.ncbi.nlm.nih.gov/compound/89646434#section=2D-Structure
https://pubchem.ncbi.nlm.nih.gov/compound/89643673#section=2D-Structure
https://pubchem.ncbi.nlm.nih.gov/compound/89643624#section=2D-Structure
https://pubchem.ncbi.nlm.nih.gov/compound/89643460#section=2D-Structure
https://pubchem.ncbi.nlm.nih.gov/compound/89643448#section=2D-Structure
https://pubchem.ncbi.nlm.nih.gov/compound/89633488#section=2D-Structure
https://pubchem.ncbi.nlm.nih.gov/compound/89618190#section=2D-Structure
https://pubchem.ncbi.nlm.nih.gov/compound/89608429#section=2D-Structure
https://pubchem.ncbi.nlm.nih.gov/compound/89607107#section=2D-Structure
https://pubchem.ncbi.nlm.nih.gov/compound/89607101#section=2D-Structure
https://pubchem.ncbi.nlm.nih.gov/compound/89607100#section=2D-Structure
https://pubchem.ncbi.nlm.nih.gov/compound/89607099#section=2D-Structure
https://pubchem.ncbi.nlm.nih.gov/compound/89606268#section=2D-Structure
https://pubchem.ncbi.nlm.nih.gov/compound/89604269#section=2D-Structure
https://pubchem.ncbi.nlm.nih.gov/compound/89590338#section=2D-Structure
https://pubchem.ncbi.nlm.nih.gov/compound/89590321#section=2D-Structure
https://pubchem.ncbi.nlm.nih.gov/compound/89585577#section=2D-Structure
https://pubchem.ncbi.nlm.nih.gov/compound/89575533#section=2D-Structure
https://pubchem.ncbi.nlm.nih.gov/compound/89574526#section=2D-Structure
https://pubchem.ncbi.nlm.nih.gov/compound/89573955#section=2D-Structure
https://pubchem.ncbi.nlm.nih.gov/compound/89571315#section=2D-Structure
https://pubchem.ncbi.nlm.nih.gov/compound/89570362#section=2D-Structure
https://pubchem.ncbi.nlm.nih.gov/compound/89569189#section=2D-Structure
https://pubchem.ncbi.nlm.nih.gov/compound/89562177#section=2D-Structure
https://pubchem.ncbi.nlm.nih.gov/compound/89562045#section=2D-Structure
https://pubchem.ncbi.nlm.nih.gov/compound/89546195#section=2D-Structure
https://pubchem.ncbi.nlm.nih.gov/compound/89546182#section=2D-Structure
https://pubchem.ncbi.nlm.nih.gov/compound/89546179#section=2D-Structure
https://pubchem.ncbi.nlm.nih.gov/compound/89534176#section=2D-Structure
https://pubchem.ncbi.nlm.nih.gov/compound/89526459#section=2D-Structure
https://pubchem.ncbi.nlm.nih.gov/compound/89510039#section=2D-Structure
https://pubchem.ncbi.nlm.nih.gov/compound/89501964#section=2D-Structure
https://pubchem.ncbi.nlm.nih.gov/compound/89495253#section=2D-Structure

https://pubchem.ncbi.nlm.nih.gov/compound/89495239#section=2D-Structure
https://pubchem.ncbi.nlm.nih.gov/compound/89495230#section=2D-Structure
https://pubchem.ncbi.nlm.nih.gov/compound/89495227#section=2D-Structure
https://pubchem.ncbi.nlm.nih.gov/compound/89495226#section=2D-Structure
https://pubchem.ncbi.nlm.nih.gov/compound/89495223#section=2D-Structure
https://pubchem.ncbi.nlm.nih.gov/compound/89495217#section=2D-Structure
https://pubchem.ncbi.nlm.nih.gov/compound/89495216#section=2D-Structure
https://pubchem.ncbi.nlm.nih.gov/compound/89495213#section=2D-Structure
https://pubchem.ncbi.nlm.nih.gov/compound/89495212#section=2D-Structure
https://pubchem.ncbi.nlm.nih.gov/compound/89495207#section=2D-Structure
https://pubchem.ncbi.nlm.nih.gov/compound/89495204#section=2D-Structure
https://pubchem.ncbi.nlm.nih.gov/compound/89495198#section=2D-Structure
https://pubchem.ncbi.nlm.nih.gov/compound/89495197#section=2D-Structure
https://pubchem.ncbi.nlm.nih.gov/compound/89495196#section=2D-Structure
https://pubchem.ncbi.nlm.nih.gov/compound/89495186#section=2D-Structure
https://pubchem.ncbi.nlm.nih.gov/compound/89495184#section=2D-Structure
https://pubchem.ncbi.nlm.nih.gov/compound/89493316#section=2D-Structure
https://pubchem.ncbi.nlm.nih.gov/compound/89464257#section=2D-Structure
https://pubchem.ncbi.nlm.nih.gov/compound/89463344#section=2D-Structure
https://pubchem.ncbi.nlm.nih.gov/compound/89457343#section=2D-Structure
https://pubchem.ncbi.nlm.nih.gov/compound/89422554#section=2D-Structure
https://pubchem.ncbi.nlm.nih.gov/compound/89420138#section=2D-Structure
https://pubchem.ncbi.nlm.nih.gov/compound/89418744#section=2D-Structure
https://pubchem.ncbi.nlm.nih.gov/compound/89417256#section=2D-Structure
https://pubchem.ncbi.nlm.nih.gov/compound/89414976#section=2D-Structure
https://pubchem.ncbi.nlm.nih.gov/compound/89407616#section=2D-Structure
https://pubchem.ncbi.nlm.nih.gov/compound/89399704#section=2D-Structure
https://pubchem.ncbi.nlm.nih.gov/compound/89398295#section=2D-Structure
https://pubchem.ncbi.nlm.nih.gov/compound/89398293#section=2D-Structure
https://pubchem.ncbi.nlm.nih.gov/compound/89386847#section=2D-Structure
https://pubchem.ncbi.nlm.nih.gov/compound/89386304#section=2D-Structure
https://pubchem.ncbi.nlm.nih.gov/compound/89378333#section=2D-Structure
https://pubchem.ncbi.nlm.nih.gov/compound/89376313#section=2D-Structure
https://pubchem.ncbi.nlm.nih.gov/compound/89376300#section=2D-Structure
https://pubchem.ncbi.nlm.nih.gov/compound/89369128#section=2D-Structure
https://pubchem.ncbi.nlm.nih.gov/compound/89361413#section=2D-Structure
https://pubchem.ncbi.nlm.nih.gov/compound/89360410#section=2D-Structure
https://pubchem.ncbi.nlm.nih.gov/compound/89359626#section=2D-Structure
https://pubchem.ncbi.nlm.nih.gov/compound/89358895#section=2D-Structure
https://pubchem.ncbi.nlm.nih.gov/compound/89357299#section=2D-Structure
https://pubchem.ncbi.nlm.nih.gov/compound/89355027#section=2D-Structure
https://pubchem.ncbi.nlm.nih.gov/compound/89350883#section=2D-Structure
https://pubchem.ncbi.nlm.nih.gov/compound/89350095#section=2D-Structure
https://pubchem.ncbi.nlm.nih.gov/compound/89347149#section=2D-Structure
https://pubchem.ncbi.nlm.nih.gov/compound/89335206#section=2D-Structure
https://pubchem.ncbi.nlm.nih.gov/compound/89331991#section=2D-Structure
https://pubchem.ncbi.nlm.nih.gov/compound/89329450#section=2D-Structure
https://pubchem.ncbi.nlm.nih.gov/compound/89327807#section=2D-Structure
https://pubchem.ncbi.nlm.nih.gov/compound/89325638#section=2D-Structure
https://pubchem.ncbi.nlm.nih.gov/compound/89324675#section=2D-Structure
https://pubchem.ncbi.nlm.nih.gov/compound/89322444#section=2D-Structure
https://pubchem.ncbi.nlm.nih.gov/compound/89320861#section=2D-Structure
https://pubchem.ncbi.nlm.nih.gov/compound/89317106#section=2D-Structure
https://pubchem.ncbi.nlm.nih.gov/compound/89304573#section=2D-Structure
https://pubchem.ncbi.nlm.nih.gov/compound/89304022#section=2D-Structure

https://pubchem.ncbi.nlm.nih.gov/compound/89302809#section=2D-Structure
https://pubchem.ncbi.nlm.nih.gov/compound/89302448#section=2D-Structure
https://pubchem.ncbi.nlm.nih.gov/compound/89298772#section=2D-Structure
https://pubchem.ncbi.nlm.nih.gov/compound/89297718#section=2D-Structure
https://pubchem.ncbi.nlm.nih.gov/compound/89297533#section=2D-Structure
https://pubchem.ncbi.nlm.nih.gov/compound/89297047#section=2D-Structure
https://pubchem.ncbi.nlm.nih.gov/compound/89297044#section=2D-Structure
https://pubchem.ncbi.nlm.nih.gov/compound/89296529#section=2D-Structure
https://pubchem.ncbi.nlm.nih.gov/compound/89292623#section=2D-Structure
https://pubchem.ncbi.nlm.nih.gov/compound/89292621#section=2D-Structure
https://pubchem.ncbi.nlm.nih.gov/compound/89292614#section=2D-Structure
https://pubchem.ncbi.nlm.nih.gov/compound/89290923#section=2D-Structure
https://pubchem.ncbi.nlm.nih.gov/compound/89283881#section=2D-Structure
https://pubchem.ncbi.nlm.nih.gov/compound/89283769#section=2D-Structure
https://pubchem.ncbi.nlm.nih.gov/compound/89282804#section=2D-Structure
https://pubchem.ncbi.nlm.nih.gov/compound/89282623#section=2D-Structure
https://pubchem.ncbi.nlm.nih.gov/compound/89278992#section=2D-Structure
https://pubchem.ncbi.nlm.nih.gov/compound/89278974#section=2D-Structure
https://pubchem.ncbi.nlm.nih.gov/compound/89277544#section=2D-Structure
https://pubchem.ncbi.nlm.nih.gov/compound/89269946#section=2D-Structure
https://pubchem.ncbi.nlm.nih.gov/compound/89263459#section=2D-Structure
https://pubchem.ncbi.nlm.nih.gov/compound/89263431#section=2D-Structure
https://pubchem.ncbi.nlm.nih.gov/compound/89262955#section=2D-Structure
https://pubchem.ncbi.nlm.nih.gov/compound/89260641#section=2D-Structure
https://pubchem.ncbi.nlm.nih.gov/compound/89255212#section=2D-Structure
https://pubchem.ncbi.nlm.nih.gov/compound/89255042#section=2D-Structure
https://pubchem.ncbi.nlm.nih.gov/compound/89253091#section=2D-Structure
https://pubchem.ncbi.nlm.nih.gov/compound/89252309#section=2D-Structure
https://pubchem.ncbi.nlm.nih.gov/compound/89249950#section=2D-Structure
https://pubchem.ncbi.nlm.nih.gov/compound/89248923#section=2D-Structure
https://pubchem.ncbi.nlm.nih.gov/compound/89239096#section=2D-Structure
https://pubchem.ncbi.nlm.nih.gov/compound/89231125#section=2D-Structure
https://pubchem.ncbi.nlm.nih.gov/compound/89230101#section=2D-Structure
https://pubchem.ncbi.nlm.nih.gov/compound/89226843#section=2D-Structure
https://pubchem.ncbi.nlm.nih.gov/compound/89225472#section=2D-Structure
https://pubchem.ncbi.nlm.nih.gov/compound/89221839#section=2D-Structure
https://pubchem.ncbi.nlm.nih.gov/compound/89221134#section=2D-Structure
https://pubchem.ncbi.nlm.nih.gov/compound/89220569#section=2D-Structure
https://pubchem.ncbi.nlm.nih.gov/compound/89202609#section=2D-Structure
https://pubchem.ncbi.nlm.nih.gov/compound/89201197#section=2D-Structure
https://pubchem.ncbi.nlm.nih.gov/compound/89200531#section=2D-Structure
https://pubchem.ncbi.nlm.nih.gov/compound/89200123#section=2D-Structure
https://pubchem.ncbi.nlm.nih.gov/compound/89198916#section=2D-Structure
https://pubchem.ncbi.nlm.nih.gov/compound/89190019#section=2D-Structure
https://pubchem.ncbi.nlm.nih.gov/compound/89189092#section=2D-Structure
https://pubchem.ncbi.nlm.nih.gov/compound/89181407#section=2D-Structure
https://pubchem.ncbi.nlm.nih.gov/compound/89174840#section=2D-Structure
https://pubchem.ncbi.nlm.nih.gov/compound/89174221#section=2D-Structure
https://pubchem.ncbi.nlm.nih.gov/compound/89172473#section=2D-Structure
https://pubchem.ncbi.nlm.nih.gov/compound/89167841#section=2D-Structure
https://pubchem.ncbi.nlm.nih.gov/compound/89166169#section=2D-Structure
https://pubchem.ncbi.nlm.nih.gov/compound/89163845#section=2D-Structure
https://pubchem.ncbi.nlm.nih.gov/compound/89157583#section=2D-Structure
https://pubchem.ncbi.nlm.nih.gov/compound/89155171#section=2D-Structure
https://pubchem.ncbi.nlm.nih.gov/compound/89153007#section=2D-Structure

https://pubchem.ncbi.nlm.nih.gov/compound/89147961#section=2D-Structure
https://pubchem.ncbi.nlm.nih.gov/compound/89143761#section=2D-Structure
https://pubchem.ncbi.nlm.nih.gov/compound/89143707#section=2D-Structure
https://pubchem.ncbi.nlm.nih.gov/compound/89143705#section=2D-Structure
https://pubchem.ncbi.nlm.nih.gov/compound/89143698#section=2D-Structure
https://pubchem.ncbi.nlm.nih.gov/compound/89140934#section=2D-Structure
https://pubchem.ncbi.nlm.nih.gov/compound/89140247#section=2D-Structure
https://pubchem.ncbi.nlm.nih.gov/compound/89134134#section=2D-Structure
https://pubchem.ncbi.nlm.nih.gov/compound/89130009#section=2D-Structure
https://pubchem.ncbi.nlm.nih.gov/compound/89129896#section=2D-Structure
https://pubchem.ncbi.nlm.nih.gov/compound/89127000#section=2D-Structure
https://pubchem.ncbi.nlm.nih.gov/compound/89116332#section=2D-Structure
https://pubchem.ncbi.nlm.nih.gov/compound/89099332#section=2D-Structure
https://pubchem.ncbi.nlm.nih.gov/compound/89089462#section=2D-Structure
https://pubchem.ncbi.nlm.nih.gov/compound/89088486#section=2D-Structure
https://pubchem.ncbi.nlm.nih.gov/compound/89075690#section=2D-Structure
https://pubchem.ncbi.nlm.nih.gov/compound/89070251#section=2D-Structure
https://pubchem.ncbi.nlm.nih.gov/compound/89068472#section=2D-Structure
https://pubchem.ncbi.nlm.nih.gov/compound/89062201#section=2D-Structure
https://pubchem.ncbi.nlm.nih.gov/compound/89056454#section=2D-Structure
https://pubchem.ncbi.nlm.nih.gov/compound/89053997#section=2D-Structure
https://pubchem.ncbi.nlm.nih.gov/compound/89041740#section=2D-Structure
https://pubchem.ncbi.nlm.nih.gov/compound/89038768#section=2D-Structure
https://pubchem.ncbi.nlm.nih.gov/compound/89029289#section=2D-Structure
https://pubchem.ncbi.nlm.nih.gov/compound/89026494#section=2D-Structure
https://pubchem.ncbi.nlm.nih.gov/compound/89025834#section=2D-Structure
https://pubchem.ncbi.nlm.nih.gov/compound/89025212#section=2D-Structure
https://pubchem.ncbi.nlm.nih.gov/compound/89022865#section=2D-Structure
https://pubchem.ncbi.nlm.nih.gov/compound/89018641#section=2D-Structure
https://pubchem.ncbi.nlm.nih.gov/compound/89012856#section=2D-Structure
https://pubchem.ncbi.nlm.nih.gov/compound/89004356#section=2D-Structure
https://pubchem.ncbi.nlm.nih.gov/compound/89003703#section=2D-Structure
https://pubchem.ncbi.nlm.nih.gov/compound/88997175#section=2D-Structure
https://pubchem.ncbi.nlm.nih.gov/compound/88997060#section=2D-Structure
https://pubchem.ncbi.nlm.nih.gov/compound/88988414#section=2D-Structure
https://pubchem.ncbi.nlm.nih.gov/compound/88983063#section=2D-Structure
https://pubchem.ncbi.nlm.nih.gov/compound/88972084#section=2D-Structure
https://pubchem.ncbi.nlm.nih.gov/compound/88972082#section=2D-Structure
https://pubchem.ncbi.nlm.nih.gov/compound/88972081#section=2D-Structure
https://pubchem.ncbi.nlm.nih.gov/compound/88969682#section=2D-Structure
https://pubchem.ncbi.nlm.nih.gov/compound/88966411#section=2D-Structure
https://pubchem.ncbi.nlm.nih.gov/compound/88956528#section=2D-Structure
https://pubchem.ncbi.nlm.nih.gov/compound/88955268#section=2D-Structure
https://pubchem.ncbi.nlm.nih.gov/compound/88951926#section=2D-Structure
https://pubchem.ncbi.nlm.nih.gov/compound/88949439#section=2D-Structure
https://pubchem.ncbi.nlm.nih.gov/compound/88946778#section=2D-Structure
https://pubchem.ncbi.nlm.nih.gov/compound/88946679#section=2D-Structure
https://pubchem.ncbi.nlm.nih.gov/compound/88945837#section=2D-Structure
https://pubchem.ncbi.nlm.nih.gov/compound/88945836#section=2D-Structure
https://pubchem.ncbi.nlm.nih.gov/compound/88945834#section=2D-Structure
https://pubchem.ncbi.nlm.nih.gov/compound/88938121#section=2D-Structure
https://pubchem.ncbi.nlm.nih.gov/compound/88937584#section=2D-Structure
https://pubchem.ncbi.nlm.nih.gov/compound/88933699#section=2D-Structure
https://pubchem.ncbi.nlm.nih.gov/compound/88926445#section=2D-Structure
https://pubchem.ncbi.nlm.nih.gov/compound/88926329#section=2D-Structure

https://pubchem.ncbi.nlm.nih.gov/compound/88924463#section=2D-Structure
https://pubchem.ncbi.nlm.nih.gov/compound/88924344#section=2D-Structure
https://pubchem.ncbi.nlm.nih.gov/compound/88924236#section=2D-Structure
https://pubchem.ncbi.nlm.nih.gov/compound/88924186#section=2D-Structure
https://pubchem.ncbi.nlm.nih.gov/compound/88924165#section=2D-Structure
https://pubchem.ncbi.nlm.nih.gov/compound/88922946#section=2D-Structure
https://pubchem.ncbi.nlm.nih.gov/compound/88920652#section=2D-Structure
https://pubchem.ncbi.nlm.nih.gov/compound/88913762#section=2D-Structure
https://pubchem.ncbi.nlm.nih.gov/compound/88912343#section=2D-Structure
https://pubchem.ncbi.nlm.nih.gov/compound/88903430#section=2D-Structure
https://pubchem.ncbi.nlm.nih.gov/compound/88903429#section=2D-Structure
https://pubchem.ncbi.nlm.nih.gov/compound/88898455#section=2D-Structure
https://pubchem.ncbi.nlm.nih.gov/compound/88898454#section=2D-Structure
https://pubchem.ncbi.nlm.nih.gov/compound/88893793#section=2D-Structure
https://pubchem.ncbi.nlm.nih.gov/compound/88886490#section=2D-Structure
https://pubchem.ncbi.nlm.nih.gov/compound/88886152#section=2D-Structure
https://pubchem.ncbi.nlm.nih.gov/compound/88884594#section=2D-Structure
https://pubchem.ncbi.nlm.nih.gov/compound/88884589#section=2D-Structure
https://pubchem.ncbi.nlm.nih.gov/compound/88881745#section=2D-Structure
https://pubchem.ncbi.nlm.nih.gov/compound/88881741#section=2D-Structure
https://pubchem.ncbi.nlm.nih.gov/compound/88880481#section=2D-Structure
https://pubchem.ncbi.nlm.nih.gov/compound/88875847#section=2D-Structure
https://pubchem.ncbi.nlm.nih.gov/compound/88870628#section=2D-Structure
https://pubchem.ncbi.nlm.nih.gov/compound/88865810#section=2D-Structure
https://pubchem.ncbi.nlm.nih.gov/compound/88861828#section=2D-Structure
https://pubchem.ncbi.nlm.nih.gov/compound/88858017#section=2D-Structure
https://pubchem.ncbi.nlm.nih.gov/compound/88853186#section=2D-Structure
https://pubchem.ncbi.nlm.nih.gov/compound/88853104#section=2D-Structure
https://pubchem.ncbi.nlm.nih.gov/compound/88853103#section=2D-Structure
https://pubchem.ncbi.nlm.nih.gov/compound/88853101#section=2D-Structure
https://pubchem.ncbi.nlm.nih.gov/compound/88849460#section=2D-Structure
https://pubchem.ncbi.nlm.nih.gov/compound/88848567#section=2D-Structure
https://pubchem.ncbi.nlm.nih.gov/compound/88848450#section=2D-Structure
https://pubchem.ncbi.nlm.nih.gov/compound/88847931#section=2D-Structure
https://pubchem.ncbi.nlm.nih.gov/compound/88845997#section=2D-Structure
https://pubchem.ncbi.nlm.nih.gov/compound/88845836#section=2D-Structure
https://pubchem.ncbi.nlm.nih.gov/compound/88844063#section=2D-Structure
https://pubchem.ncbi.nlm.nih.gov/compound/88842726#section=2D-Structure
https://pubchem.ncbi.nlm.nih.gov/compound/88841752#section=2D-Structure
https://pubchem.ncbi.nlm.nih.gov/compound/88841751#section=2D-Structure
https://pubchem.ncbi.nlm.nih.gov/compound/88838226#section=2D-Structure
https://pubchem.ncbi.nlm.nih.gov/compound/88834157#section=2D-Structure
https://pubchem.ncbi.nlm.nih.gov/compound/88834155#section=2D-Structure
https://pubchem.ncbi.nlm.nih.gov/compound/88833410#section=2D-Structure
https://pubchem.ncbi.nlm.nih.gov/compound/88831606#section=2D-Structure
https://pubchem.ncbi.nlm.nih.gov/compound/88831604#section=2D-Structure
https://pubchem.ncbi.nlm.nih.gov/compound/88829818#section=2D-Structure
https://pubchem.ncbi.nlm.nih.gov/compound/88829762#section=2D-Structure
https://pubchem.ncbi.nlm.nih.gov/compound/88829157#section=2D-Structure
https://pubchem.ncbi.nlm.nih.gov/compound/88828783#section=2D-Structure
https://pubchem.ncbi.nlm.nih.gov/compound/88828462#section=2D-Structure
https://pubchem.ncbi.nlm.nih.gov/compound/88828298#section=2D-Structure
https://pubchem.ncbi.nlm.nih.gov/compound/88828125#section=2D-Structure
https://pubchem.ncbi.nlm.nih.gov/compound/88827051#section=2D-Structure
https://pubchem.ncbi.nlm.nih.gov/compound/88826842#section=2D-Structure

https://pubchem.ncbi.nlm.nih.gov/compound/88826519#section=2D-Structure
https://pubchem.ncbi.nlm.nih.gov/compound/88823469#section=2D-Structure
https://pubchem.ncbi.nlm.nih.gov/compound/88821773#section=2D-Structure
https://pubchem.ncbi.nlm.nih.gov/compound/88820659#section=2D-Structure
https://pubchem.ncbi.nlm.nih.gov/compound/88817793#section=2D-Structure
https://pubchem.ncbi.nlm.nih.gov/compound/88817080#section=2D-Structure
https://pubchem.ncbi.nlm.nih.gov/compound/88817016#section=2D-Structure
https://pubchem.ncbi.nlm.nih.gov/compound/88816668#section=2D-Structure
https://pubchem.ncbi.nlm.nih.gov/compound/88814220#section=2D-Structure
https://pubchem.ncbi.nlm.nih.gov/compound/88813954#section=2D-Structure
https://pubchem.ncbi.nlm.nih.gov/compound/88813953#section=2D-Structure
https://pubchem.ncbi.nlm.nih.gov/compound/88813769#section=2D-Structure
https://pubchem.ncbi.nlm.nih.gov/compound/88810942#section=2D-Structure
https://pubchem.ncbi.nlm.nih.gov/compound/88810590#section=2D-Structure
https://pubchem.ncbi.nlm.nih.gov/compound/88807493#section=2D-Structure
https://pubchem.ncbi.nlm.nih.gov/compound/88805004#section=2D-Structure
https://pubchem.ncbi.nlm.nih.gov/compound/88804790#section=2D-Structure
https://pubchem.ncbi.nlm.nih.gov/compound/88803894#section=2D-Structure
https://pubchem.ncbi.nlm.nih.gov/compound/88801644#section=2D-Structure
https://pubchem.ncbi.nlm.nih.gov/compound/88800293#section=2D-Structure
https://pubchem.ncbi.nlm.nih.gov/compound/88798857#section=2D-Structure
https://pubchem.ncbi.nlm.nih.gov/compound/88796804#section=2D-Structure
https://pubchem.ncbi.nlm.nih.gov/compound/88795225#section=2D-Structure
https://pubchem.ncbi.nlm.nih.gov/compound/88792824#section=2D-Structure
https://pubchem.ncbi.nlm.nih.gov/compound/88792355#section=2D-Structure
https://pubchem.ncbi.nlm.nih.gov/compound/88789001#section=2D-Structure
https://pubchem.ncbi.nlm.nih.gov/compound/88784009#section=2D-Structure
https://pubchem.ncbi.nlm.nih.gov/compound/88783763#section=2D-Structure
https://pubchem.ncbi.nlm.nih.gov/compound/88783526#section=2D-Structure
https://pubchem.ncbi.nlm.nih.gov/compound/88782631#section=2D-Structure
https://pubchem.ncbi.nlm.nih.gov/compound/88780948#section=2D-Structure
https://pubchem.ncbi.nlm.nih.gov/compound/88780584#section=2D-Structure
https://pubchem.ncbi.nlm.nih.gov/compound/88780313#section=2D-Structure
https://pubchem.ncbi.nlm.nih.gov/compound/88779455#section=2D-Structure
https://pubchem.ncbi.nlm.nih.gov/compound/88775580#section=2D-Structure
https://pubchem.ncbi.nlm.nih.gov/compound/88773962#section=2D-Structure
https://pubchem.ncbi.nlm.nih.gov/compound/88772864#section=2D-Structure
https://pubchem.ncbi.nlm.nih.gov/compound/88772129#section=2D-Structure
https://pubchem.ncbi.nlm.nih.gov/compound/88771950#section=2D-Structure
https://pubchem.ncbi.nlm.nih.gov/compound/88769902#section=2D-Structure
https://pubchem.ncbi.nlm.nih.gov/compound/88768287#section=2D-Structure
https://pubchem.ncbi.nlm.nih.gov/compound/88767960#section=2D-Structure
https://pubchem.ncbi.nlm.nih.gov/compound/88767861#section=2D-Structure
https://pubchem.ncbi.nlm.nih.gov/compound/88766589#section=2D-Structure
https://pubchem.ncbi.nlm.nih.gov/compound/88766134#section=2D-Structure
https://pubchem.ncbi.nlm.nih.gov/compound/88758610#section=2D-Structure
https://pubchem.ncbi.nlm.nih.gov/compound/88758053#section=2D-Structure
https://pubchem.ncbi.nlm.nih.gov/compound/88757493#section=2D-Structure
https://pubchem.ncbi.nlm.nih.gov/compound/88756565#section=2D-Structure
https://pubchem.ncbi.nlm.nih.gov/compound/88752628#section=2D-Structure
https://pubchem.ncbi.nlm.nih.gov/compound/88751248#section=2D-Structure
https://pubchem.ncbi.nlm.nih.gov/compound/88746354#section=2D-Structure
https://pubchem.ncbi.nlm.nih.gov/compound/88744612#section=2D-Structure
https://pubchem.ncbi.nlm.nih.gov/compound/88739493#section=2D-Structure
https://pubchem.ncbi.nlm.nih.gov/compound/88733765#section=2D-Structure

https://pubchem.ncbi.nlm.nih.gov/compound/88732010#section=2D-Structure
https://pubchem.ncbi.nlm.nih.gov/compound/88729588#section=2D-Structure
https://pubchem.ncbi.nlm.nih.gov/compound/88727825#section=2D-Structure
https://pubchem.ncbi.nlm.nih.gov/compound/88725574#section=2D-Structure
https://pubchem.ncbi.nlm.nih.gov/compound/88725333#section=2D-Structure
https://pubchem.ncbi.nlm.nih.gov/compound/88723027#section=2D-Structure
https://pubchem.ncbi.nlm.nih.gov/compound/88720920#section=2D-Structure
https://pubchem.ncbi.nlm.nih.gov/compound/88716166#section=2D-Structure
https://pubchem.ncbi.nlm.nih.gov/compound/88715149#section=2D-Structure
https://pubchem.ncbi.nlm.nih.gov/compound/88714120#section=2D-Structure
https://pubchem.ncbi.nlm.nih.gov/compound/88712479#section=2D-Structure
https://pubchem.ncbi.nlm.nih.gov/compound/88712350#section=2D-Structure
https://pubchem.ncbi.nlm.nih.gov/compound/88711364#section=2D-Structure
https://pubchem.ncbi.nlm.nih.gov/compound/88710599#section=2D-Structure
https://pubchem.ncbi.nlm.nih.gov/compound/88709580#section=2D-Structure
https://pubchem.ncbi.nlm.nih.gov/compound/88707496#section=2D-Structure
https://pubchem.ncbi.nlm.nih.gov/compound/88705735#section=2D-Structure
https://pubchem.ncbi.nlm.nih.gov/compound/88705468#section=2D-Structure
https://pubchem.ncbi.nlm.nih.gov/compound/88705467#section=2D-Structure
https://pubchem.ncbi.nlm.nih.gov/compound/88701621#section=2D-Structure
https://pubchem.ncbi.nlm.nih.gov/compound/88701522#section=2D-Structure
https://pubchem.ncbi.nlm.nih.gov/compound/88699840#section=2D-Structure
https://pubchem.ncbi.nlm.nih.gov/compound/88698061#section=2D-Structure
https://pubchem.ncbi.nlm.nih.gov/compound/88697965#section=2D-Structure
https://pubchem.ncbi.nlm.nih.gov/compound/88696694#section=2D-Structure
https://pubchem.ncbi.nlm.nih.gov/compound/88694746#section=2D-Structure
https://pubchem.ncbi.nlm.nih.gov/compound/88692217#section=2D-Structure
https://pubchem.ncbi.nlm.nih.gov/compound/88692169#section=2D-Structure
https://pubchem.ncbi.nlm.nih.gov/compound/88691808#section=2D-Structure
https://pubchem.ncbi.nlm.nih.gov/compound/88688605#section=2D-Structure
https://pubchem.ncbi.nlm.nih.gov/compound/88686581#section=2D-Structure
https://pubchem.ncbi.nlm.nih.gov/compound/88686354#section=2D-Structure
https://pubchem.ncbi.nlm.nih.gov/compound/88686335#section=2D-Structure
https://pubchem.ncbi.nlm.nih.gov/compound/88680013#section=2D-Structure
https://pubchem.ncbi.nlm.nih.gov/compound/88675992#section=2D-Structure
https://pubchem.ncbi.nlm.nih.gov/compound/88675985#section=2D-Structure
https://pubchem.ncbi.nlm.nih.gov/compound/88673449#section=2D-Structure
https://pubchem.ncbi.nlm.nih.gov/compound/88673447#section=2D-Structure
https://pubchem.ncbi.nlm.nih.gov/compound/88672704#section=2D-Structure
https://pubchem.ncbi.nlm.nih.gov/compound/88671410#section=2D-Structure
https://pubchem.ncbi.nlm.nih.gov/compound/88670347#section=2D-Structure
https://pubchem.ncbi.nlm.nih.gov/compound/88666112#section=2D-Structure
https://pubchem.ncbi.nlm.nih.gov/compound/88664126#section=2D-Structure
https://pubchem.ncbi.nlm.nih.gov/compound/88661661#section=2D-Structure
https://pubchem.ncbi.nlm.nih.gov/compound/88661553#section=2D-Structure
https://pubchem.ncbi.nlm.nih.gov/compound/88657498#section=2D-Structure
https://pubchem.ncbi.nlm.nih.gov/compound/88657494#section=2D-Structure
https://pubchem.ncbi.nlm.nih.gov/compound/88655132#section=2D-Structure
https://pubchem.ncbi.nlm.nih.gov/compound/88654179#section=2D-Structure
https://pubchem.ncbi.nlm.nih.gov/compound/88653735#section=2D-Structure
https://pubchem.ncbi.nlm.nih.gov/compound/88651340#section=2D-Structure
https://pubchem.ncbi.nlm.nih.gov/compound/88651293#section=2D-Structure
https://pubchem.ncbi.nlm.nih.gov/compound/88650955#section=2D-Structure
https://pubchem.ncbi.nlm.nih.gov/compound/88650415#section=2D-Structure
https://pubchem.ncbi.nlm.nih.gov/compound/88645671#section=2D-Structure

https://pubchem.ncbi.nlm.nih.gov/compound/88645388#section=2D-Structure
https://pubchem.ncbi.nlm.nih.gov/compound/88645324#section=2D-Structure
https://pubchem.ncbi.nlm.nih.gov/compound/88644840#section=2D-Structure
https://pubchem.ncbi.nlm.nih.gov/compound/88640260#section=2D-Structure
https://pubchem.ncbi.nlm.nih.gov/compound/88633722#section=2D-Structure
https://pubchem.ncbi.nlm.nih.gov/compound/88631500#section=2D-Structure
https://pubchem.ncbi.nlm.nih.gov/compound/88630565#section=2D-Structure
https://pubchem.ncbi.nlm.nih.gov/compound/88623123#section=2D-Structure
https://pubchem.ncbi.nlm.nih.gov/compound/88622112#section=2D-Structure
https://pubchem.ncbi.nlm.nih.gov/compound/88621897#section=2D-Structure
https://pubchem.ncbi.nlm.nih.gov/compound/88621501#section=2D-Structure
https://pubchem.ncbi.nlm.nih.gov/compound/88611711#section=2D-Structure
https://pubchem.ncbi.nlm.nih.gov/compound/88609365#section=2D-Structure
https://pubchem.ncbi.nlm.nih.gov/compound/88597552#section=2D-Structure
https://pubchem.ncbi.nlm.nih.gov/compound/88596642#section=2D-Structure
https://pubchem.ncbi.nlm.nih.gov/compound/88590160#section=2D-Structure
https://pubchem.ncbi.nlm.nih.gov/compound/88576383#section=2D-Structure
https://pubchem.ncbi.nlm.nih.gov/compound/88576215#section=2D-Structure
https://pubchem.ncbi.nlm.nih.gov/compound/88572395#section=2D-Structure
https://pubchem.ncbi.nlm.nih.gov/compound/88569833#section=2D-Structure
https://pubchem.ncbi.nlm.nih.gov/compound/88563061#section=2D-Structure
https://pubchem.ncbi.nlm.nih.gov/compound/88562351#section=2D-Structure
https://pubchem.ncbi.nlm.nih.gov/compound/88560236#section=2D-Structure
https://pubchem.ncbi.nlm.nih.gov/compound/88559888#section=2D-Structure
https://pubchem.ncbi.nlm.nih.gov/compound/88559370#section=2D-Structure
https://pubchem.ncbi.nlm.nih.gov/compound/88556598#section=2D-Structure
https://pubchem.ncbi.nlm.nih.gov/compound/88554247#section=2D-Structure
https://pubchem.ncbi.nlm.nih.gov/compound/88554222#section=2D-Structure
https://pubchem.ncbi.nlm.nih.gov/compound/88553228#section=2D-Structure
https://pubchem.ncbi.nlm.nih.gov/compound/88542754#section=2D-Structure
https://pubchem.ncbi.nlm.nih.gov/compound/88542497#section=2D-Structure
https://pubchem.ncbi.nlm.nih.gov/compound/88537935#section=2D-Structure
https://pubchem.ncbi.nlm.nih.gov/compound/88536446#section=2D-Structure
https://pubchem.ncbi.nlm.nih.gov/compound/88534885#section=2D-Structure
https://pubchem.ncbi.nlm.nih.gov/compound/88533766#section=2D-Structure
https://pubchem.ncbi.nlm.nih.gov/compound/88533764#section=2D-Structure
https://pubchem.ncbi.nlm.nih.gov/compound/88528510#section=2D-Structure
https://pubchem.ncbi.nlm.nih.gov/compound/88521331#section=2D-Structure
https://pubchem.ncbi.nlm.nih.gov/compound/88519204#section=2D-Structure
https://pubchem.ncbi.nlm.nih.gov/compound/88517455#section=2D-Structure
https://pubchem.ncbi.nlm.nih.gov/compound/88516498#section=2D-Structure
https://pubchem.ncbi.nlm.nih.gov/compound/88503580#section=2D-Structure
https://pubchem.ncbi.nlm.nih.gov/compound/88501038#section=2D-Structure
https://pubchem.ncbi.nlm.nih.gov/compound/88497651#section=2D-Structure
https://pubchem.ncbi.nlm.nih.gov/compound/88497574#section=2D-Structure
https://pubchem.ncbi.nlm.nih.gov/compound/88497393#section=2D-Structure
https://pubchem.ncbi.nlm.nih.gov/compound/88496973#section=2D-Structure
https://pubchem.ncbi.nlm.nih.gov/compound/88496073#section=2D-Structure
https://pubchem.ncbi.nlm.nih.gov/compound/88492637#section=2D-Structure
https://pubchem.ncbi.nlm.nih.gov/compound/88492636#section=2D-Structure
https://pubchem.ncbi.nlm.nih.gov/compound/88492624#section=2D-Structure
https://pubchem.ncbi.nlm.nih.gov/compound/88490436#section=2D-Structure
https://pubchem.ncbi.nlm.nih.gov/compound/88490185#section=2D-Structure
https://pubchem.ncbi.nlm.nih.gov/compound/88490183#section=2D-Structure
https://pubchem.ncbi.nlm.nih.gov/compound/88489922#section=2D-Structure

https://pubchem.ncbi.nlm.nih.gov/compound/88487653#section=2D-Structure
https://pubchem.ncbi.nlm.nih.gov/compound/88485121#section=2D-Structure
https://pubchem.ncbi.nlm.nih.gov/compound/88485091#section=2D-Structure
https://pubchem.ncbi.nlm.nih.gov/compound/88484740#section=2D-Structure
https://pubchem.ncbi.nlm.nih.gov/compound/88479642#section=2D-Structure
https://pubchem.ncbi.nlm.nih.gov/compound/88479609#section=2D-Structure
https://pubchem.ncbi.nlm.nih.gov/compound/88476956#section=2D-Structure
https://pubchem.ncbi.nlm.nih.gov/compound/88474117#section=2D-Structure
https://pubchem.ncbi.nlm.nih.gov/compound/88471286#section=2D-Structure
https://pubchem.ncbi.nlm.nih.gov/compound/88468405#section=2D-Structure
https://pubchem.ncbi.nlm.nih.gov/compound/88468076#section=2D-Structure
https://pubchem.ncbi.nlm.nih.gov/compound/88466717#section=2D-Structure
https://pubchem.ncbi.nlm.nih.gov/compound/88462907#section=2D-Structure
https://pubchem.ncbi.nlm.nih.gov/compound/88460292#section=2D-Structure
https://pubchem.ncbi.nlm.nih.gov/compound/88459119#section=2D-Structure
https://pubchem.ncbi.nlm.nih.gov/compound/88457965#section=2D-Structure
https://pubchem.ncbi.nlm.nih.gov/compound/88454585#section=2D-Structure
https://pubchem.ncbi.nlm.nih.gov/compound/88454330#section=2D-Structure
https://pubchem.ncbi.nlm.nih.gov/compound/88453980#section=2D-Structure
https://pubchem.ncbi.nlm.nih.gov/compound/88453597#section=2D-Structure
https://pubchem.ncbi.nlm.nih.gov/compound/88453500#section=2D-Structure
https://pubchem.ncbi.nlm.nih.gov/compound/88451509#section=2D-Structure
https://pubchem.ncbi.nlm.nih.gov/compound/88447528#section=2D-Structure
https://pubchem.ncbi.nlm.nih.gov/compound/88447412#section=2D-Structure
https://pubchem.ncbi.nlm.nih.gov/compound/88446734#section=2D-Structure
https://pubchem.ncbi.nlm.nih.gov/compound/88445332#section=2D-Structure
https://pubchem.ncbi.nlm.nih.gov/compound/88442385#section=2D-Structure
https://pubchem.ncbi.nlm.nih.gov/compound/88440404#section=2D-Structure
https://pubchem.ncbi.nlm.nih.gov/compound/88439719#section=2D-Structure
https://pubchem.ncbi.nlm.nih.gov/compound/88438638#section=2D-Structure
https://pubchem.ncbi.nlm.nih.gov/compound/88436608#section=2D-Structure
https://pubchem.ncbi.nlm.nih.gov/compound/88430719#section=2D-Structure
https://pubchem.ncbi.nlm.nih.gov/compound/88428577#section=2D-Structure
https://pubchem.ncbi.nlm.nih.gov/compound/88428561#section=2D-Structure
https://pubchem.ncbi.nlm.nih.gov/compound/88428560#section=2D-Structure
https://pubchem.ncbi.nlm.nih.gov/compound/88426346#section=2D-Structure
https://pubchem.ncbi.nlm.nih.gov/compound/88424922#section=2D-Structure
https://pubchem.ncbi.nlm.nih.gov/compound/88423392#section=2D-Structure
https://pubchem.ncbi.nlm.nih.gov/compound/88421432#section=2D-Structure
https://pubchem.ncbi.nlm.nih.gov/compound/88421298#section=2D-Structure
https://pubchem.ncbi.nlm.nih.gov/compound/88421107#section=2D-Structure
https://pubchem.ncbi.nlm.nih.gov/compound/88417601#section=2D-Structure
https://pubchem.ncbi.nlm.nih.gov/compound/88413727#section=2D-Structure
https://pubchem.ncbi.nlm.nih.gov/compound/88412046#section=2D-Structure
https://pubchem.ncbi.nlm.nih.gov/compound/88409967#section=2D-Structure
https://pubchem.ncbi.nlm.nih.gov/compound/88406269#section=2D-Structure
https://pubchem.ncbi.nlm.nih.gov/compound/88406229#section=2D-Structure
https://pubchem.ncbi.nlm.nih.gov/compound/88400930#section=2D-Structure
https://pubchem.ncbi.nlm.nih.gov/compound/88400874#section=2D-Structure
https://pubchem.ncbi.nlm.nih.gov/compound/88398826#section=2D-Structure
https://pubchem.ncbi.nlm.nih.gov/compound/88398824#section=2D-Structure
https://pubchem.ncbi.nlm.nih.gov/compound/88398817#section=2D-Structure
https://pubchem.ncbi.nlm.nih.gov/compound/88398805#section=2D-Structure
https://pubchem.ncbi.nlm.nih.gov/compound/88398800#section=2D-Structure
https://pubchem.ncbi.nlm.nih.gov/compound/88398797#section=2D-Structure

https://pubchem.ncbi.nlm.nih.gov/compound/88398791#section=2D-Structure
https://pubchem.ncbi.nlm.nih.gov/compound/88398790#section=2D-Structure
https://pubchem.ncbi.nlm.nih.gov/compound/88393311#section=2D-Structure
https://pubchem.ncbi.nlm.nih.gov/compound/88391541#section=2D-Structure
https://pubchem.ncbi.nlm.nih.gov/compound/88389680#section=2D-Structure
https://pubchem.ncbi.nlm.nih.gov/compound/88388685#section=2D-Structure
https://pubchem.ncbi.nlm.nih.gov/compound/88388289#section=2D-Structure
https://pubchem.ncbi.nlm.nih.gov/compound/88387057#section=2D-Structure
https://pubchem.ncbi.nlm.nih.gov/compound/88386595#section=2D-Structure
https://pubchem.ncbi.nlm.nih.gov/compound/88385482#section=2D-Structure
https://pubchem.ncbi.nlm.nih.gov/compound/88384955#section=2D-Structure
https://pubchem.ncbi.nlm.nih.gov/compound/88384863#section=2D-Structure
https://pubchem.ncbi.nlm.nih.gov/compound/88383655#section=2D-Structure
https://pubchem.ncbi.nlm.nih.gov/compound/88382065#section=2D-Structure
https://pubchem.ncbi.nlm.nih.gov/compound/88382038#section=2D-Structure
https://pubchem.ncbi.nlm.nih.gov/compound/88382037#section=2D-Structure
https://pubchem.ncbi.nlm.nih.gov/compound/88382032#section=2D-Structure
https://pubchem.ncbi.nlm.nih.gov/compound/88378911#section=2D-Structure
https://pubchem.ncbi.nlm.nih.gov/compound/88378380#section=2D-Structure
https://pubchem.ncbi.nlm.nih.gov/compound/88378134#section=2D-Structure
https://pubchem.ncbi.nlm.nih.gov/compound/88377763#section=2D-Structure
https://pubchem.ncbi.nlm.nih.gov/compound/88371411#section=2D-Structure
https://pubchem.ncbi.nlm.nih.gov/compound/88369348#section=2D-Structure
https://pubchem.ncbi.nlm.nih.gov/compound/88368697#section=2D-Structure
https://pubchem.ncbi.nlm.nih.gov/compound/88367122#section=2D-Structure
https://pubchem.ncbi.nlm.nih.gov/compound/88366364#section=2D-Structure
https://pubchem.ncbi.nlm.nih.gov/compound/88364169#section=2D-Structure
https://pubchem.ncbi.nlm.nih.gov/compound/88362126#section=2D-Structure
https://pubchem.ncbi.nlm.nih.gov/compound/88354561#section=2D-Structure
https://pubchem.ncbi.nlm.nih.gov/compound/88345071#section=2D-Structure
https://pubchem.ncbi.nlm.nih.gov/compound/88342370#section=2D-Structure
https://pubchem.ncbi.nlm.nih.gov/compound/88340500#section=2D-Structure
https://pubchem.ncbi.nlm.nih.gov/compound/88329071#section=2D-Structure
https://pubchem.ncbi.nlm.nih.gov/compound/88327806#section=2D-Structure
https://pubchem.ncbi.nlm.nih.gov/compound/88323838#section=2D-Structure
https://pubchem.ncbi.nlm.nih.gov/compound/88322838#section=2D-Structure
https://pubchem.ncbi.nlm.nih.gov/compound/88318033#section=2D-Structure
https://pubchem.ncbi.nlm.nih.gov/compound/88316851#section=2D-Structure
https://pubchem.ncbi.nlm.nih.gov/compound/88313832#section=2D-Structure
https://pubchem.ncbi.nlm.nih.gov/compound/88307173#section=2D-Structure
https://pubchem.ncbi.nlm.nih.gov/compound/88306641#section=2D-Structure
https://pubchem.ncbi.nlm.nih.gov/compound/88305626#section=2D-Structure
https://pubchem.ncbi.nlm.nih.gov/compound/88305625#section=2D-Structure
https://pubchem.ncbi.nlm.nih.gov/compound/88305145#section=2D-Structure
https://pubchem.ncbi.nlm.nih.gov/compound/88304664#section=2D-Structure
https://pubchem.ncbi.nlm.nih.gov/compound/88302493#section=2D-Structure
https://pubchem.ncbi.nlm.nih.gov/compound/88296095#section=2D-Structure
https://pubchem.ncbi.nlm.nih.gov/compound/88296070#section=2D-Structure
https://pubchem.ncbi.nlm.nih.gov/compound/88287380#section=2D-Structure
https://pubchem.ncbi.nlm.nih.gov/compound/88279761#section=2D-Structure
https://pubchem.ncbi.nlm.nih.gov/compound/88279063#section=2D-Structure
https://pubchem.ncbi.nlm.nih.gov/compound/88278711#section=2D-Structure
https://pubchem.ncbi.nlm.nih.gov/compound/88278622#section=2D-Structure
https://pubchem.ncbi.nlm.nih.gov/compound/88277995#section=2D-Structure
https://pubchem.ncbi.nlm.nih.gov/compound/88265879#section=2D-Structure

https://pubchem.ncbi.nlm.nih.gov/compound/88262658#section=2D-Structure
https://pubchem.ncbi.nlm.nih.gov/compound/88252760#section=2D-Structure
https://pubchem.ncbi.nlm.nih.gov/compound/88251585#section=2D-Structure
https://pubchem.ncbi.nlm.nih.gov/compound/88251446#section=2D-Structure
https://pubchem.ncbi.nlm.nih.gov/compound/88249306#section=2D-Structure
https://pubchem.ncbi.nlm.nih.gov/compound/88248581#section=2D-Structure
https://pubchem.ncbi.nlm.nih.gov/compound/88248006#section=2D-Structure
https://pubchem.ncbi.nlm.nih.gov/compound/88248#section=2D-Structure
https://pubchem.ncbi.nlm.nih.gov/compound/88243443#section=2D-Structure
https://pubchem.ncbi.nlm.nih.gov/compound/88235888#section=2D-Structure
https://pubchem.ncbi.nlm.nih.gov/compound/88232857#section=2D-Structure
https://pubchem.ncbi.nlm.nih.gov/compound/88225138#section=2D-Structure
https://pubchem.ncbi.nlm.nih.gov/compound/88222449#section=2D-Structure
https://pubchem.ncbi.nlm.nih.gov/compound/88221062#section=2D-Structure
https://pubchem.ncbi.nlm.nih.gov/compound/88217439#section=2D-Structure
https://pubchem.ncbi.nlm.nih.gov/compound/88213747#section=2D-Structure
https://pubchem.ncbi.nlm.nih.gov/compound/88213418#section=2D-Structure
https://pubchem.ncbi.nlm.nih.gov/compound/88208296#section=2D-Structure
https://pubchem.ncbi.nlm.nih.gov/compound/88206395#section=2D-Structure
https://pubchem.ncbi.nlm.nih.gov/compound/88205217#section=2D-Structure
https://pubchem.ncbi.nlm.nih.gov/compound/88196871#section=2D-Structure
https://pubchem.ncbi.nlm.nih.gov/compound/88196870#section=2D-Structure
https://pubchem.ncbi.nlm.nih.gov/compound/88196869#section=2D-Structure
https://pubchem.ncbi.nlm.nih.gov/compound/88189473#section=2D-Structure
https://pubchem.ncbi.nlm.nih.gov/compound/88187706#section=2D-Structure
https://pubchem.ncbi.nlm.nih.gov/compound/88187559#section=2D-Structure
https://pubchem.ncbi.nlm.nih.gov/compound/88186662#section=2D-Structure
https://pubchem.ncbi.nlm.nih.gov/compound/88186533#section=2D-Structure
https://pubchem.ncbi.nlm.nih.gov/compound/88184323#section=2D-Structure
https://pubchem.ncbi.nlm.nih.gov/compound/88183600#section=2D-Structure
https://pubchem.ncbi.nlm.nih.gov/compound/88178954#section=2D-Structure
https://pubchem.ncbi.nlm.nih.gov/compound/88177718#section=2D-Structure
https://pubchem.ncbi.nlm.nih.gov/compound/88177717#section=2D-Structure
https://pubchem.ncbi.nlm.nih.gov/compound/88176655#section=2D-Structure
https://pubchem.ncbi.nlm.nih.gov/compound/88175924#section=2D-Structure
https://pubchem.ncbi.nlm.nih.gov/compound/88174060#section=2D-Structure
https://pubchem.ncbi.nlm.nih.gov/compound/88172264#section=2D-Structure
https://pubchem.ncbi.nlm.nih.gov/compound/88164351#section=2D-Structure
https://pubchem.ncbi.nlm.nih.gov/compound/88162307#section=2D-Structure
https://pubchem.ncbi.nlm.nih.gov/compound/88161089#section=2D-Structure
https://pubchem.ncbi.nlm.nih.gov/compound/88160266#section=2D-Structure
https://pubchem.ncbi.nlm.nih.gov/compound/88160265#section=2D-Structure
https://pubchem.ncbi.nlm.nih.gov/compound/88156110#section=2D-Structure
https://pubchem.ncbi.nlm.nih.gov/compound/88155671#section=2D-Structure
https://pubchem.ncbi.nlm.nih.gov/compound/88153472#section=2D-Structure
https://pubchem.ncbi.nlm.nih.gov/compound/88151940#section=2D-Structure
https://pubchem.ncbi.nlm.nih.gov/compound/88144704#section=2D-Structure
https://pubchem.ncbi.nlm.nih.gov/compound/88141588#section=2D-Structure
https://pubchem.ncbi.nlm.nih.gov/compound/88139486#section=2D-Structure
https://pubchem.ncbi.nlm.nih.gov/compound/88138340#section=2D-Structure
https://pubchem.ncbi.nlm.nih.gov/compound/88138181#section=2D-Structure
https://pubchem.ncbi.nlm.nih.gov/compound/88130883#section=2D-Structure
https://pubchem.ncbi.nlm.nih.gov/compound/88129528#section=2D-Structure
https://pubchem.ncbi.nlm.nih.gov/compound/88129202#section=2D-Structure
https://pubchem.ncbi.nlm.nih.gov/compound/88128303#section=2D-Structure

https://pubchem.ncbi.nlm.nih.gov/compound/88127315#section=2D-Structure
https://pubchem.ncbi.nlm.nih.gov/compound/88122888#section=2D-Structure
https://pubchem.ncbi.nlm.nih.gov/compound/88121891#section=2D-Structure
https://pubchem.ncbi.nlm.nih.gov/compound/88120458#section=2D-Structure
https://pubchem.ncbi.nlm.nih.gov/compound/88108578#section=2D-Structure
https://pubchem.ncbi.nlm.nih.gov/compound/88104124#section=2D-Structure
https://pubchem.ncbi.nlm.nih.gov/compound/88103047#section=2D-Structure
https://pubchem.ncbi.nlm.nih.gov/compound/88099960#section=2D-Structure
https://pubchem.ncbi.nlm.nih.gov/compound/88094310#section=2D-Structure
https://pubchem.ncbi.nlm.nih.gov/compound/88092012#section=2D-Structure
https://pubchem.ncbi.nlm.nih.gov/compound/88089783#section=2D-Structure
https://pubchem.ncbi.nlm.nih.gov/compound/88088406#section=2D-Structure
https://pubchem.ncbi.nlm.nih.gov/compound/88087777#section=2D-Structure
https://pubchem.ncbi.nlm.nih.gov/compound/88087681#section=2D-Structure
https://pubchem.ncbi.nlm.nih.gov/compound/88085074#section=2D-Structure
https://pubchem.ncbi.nlm.nih.gov/compound/88078190#section=2D-Structure
https://pubchem.ncbi.nlm.nih.gov/compound/88077905#section=2D-Structure
https://pubchem.ncbi.nlm.nih.gov/compound/88077373#section=2D-Structure
https://pubchem.ncbi.nlm.nih.gov/compound/88074762#section=2D-Structure
https://pubchem.ncbi.nlm.nih.gov/compound/88072644#section=2D-Structure
https://pubchem.ncbi.nlm.nih.gov/compound/88068392#section=2D-Structure
https://pubchem.ncbi.nlm.nih.gov/compound/88058365#section=2D-Structure
https://pubchem.ncbi.nlm.nih.gov/compound/88052751#section=2D-Structure
https://pubchem.ncbi.nlm.nih.gov/compound/88044578#section=2D-Structure
https://pubchem.ncbi.nlm.nih.gov/compound/88036151#section=2D-Structure
https://pubchem.ncbi.nlm.nih.gov/compound/88036029#section=2D-Structure
https://pubchem.ncbi.nlm.nih.gov/compound/88035609#section=2D-Structure
https://pubchem.ncbi.nlm.nih.gov/compound/88035417#section=2D-Structure
https://pubchem.ncbi.nlm.nih.gov/compound/88030873#section=2D-Structure
https://pubchem.ncbi.nlm.nih.gov/compound/88023276#section=2D-Structure
https://pubchem.ncbi.nlm.nih.gov/compound/88021839#section=2D-Structure
https://pubchem.ncbi.nlm.nih.gov/compound/88021713#section=2D-Structure
https://pubchem.ncbi.nlm.nih.gov/compound/88021691#section=2D-Structure
https://pubchem.ncbi.nlm.nih.gov/compound/88020659#section=2D-Structure
https://pubchem.ncbi.nlm.nih.gov/compound/88020658#section=2D-Structure
https://pubchem.ncbi.nlm.nih.gov/compound/88020229#section=2D-Structure
https://pubchem.ncbi.nlm.nih.gov/compound/88007289#section=2D-Structure
https://pubchem.ncbi.nlm.nih.gov/compound/87998284#section=2D-Structure
https://pubchem.ncbi.nlm.nih.gov/compound/87997485#section=2D-Structure
https://pubchem.ncbi.nlm.nih.gov/compound/87994460#section=2D-Structure
https://pubchem.ncbi.nlm.nih.gov/compound/87994142#section=2D-Structure
https://pubchem.ncbi.nlm.nih.gov/compound/87993365#section=2D-Structure
https://pubchem.ncbi.nlm.nih.gov/compound/87991361#section=2D-Structure
https://pubchem.ncbi.nlm.nih.gov/compound/87990944#section=2D-Structure
https://pubchem.ncbi.nlm.nih.gov/compound/87990529#section=2D-Structure
https://pubchem.ncbi.nlm.nih.gov/compound/87990076#section=2D-Structure
https://pubchem.ncbi.nlm.nih.gov/compound/87989703#section=2D-Structure
https://pubchem.ncbi.nlm.nih.gov/compound/87988474#section=2D-Structure
https://pubchem.ncbi.nlm.nih.gov/compound/87985550#section=2D-Structure
https://pubchem.ncbi.nlm.nih.gov/compound/87984856#section=2D-Structure
https://pubchem.ncbi.nlm.nih.gov/compound/87982416#section=2D-Structure
https://pubchem.ncbi.nlm.nih.gov/compound/87968905#section=2D-Structure
https://pubchem.ncbi.nlm.nih.gov/compound/87968670#section=2D-Structure
https://pubchem.ncbi.nlm.nih.gov/compound/87968469#section=2D-Structure
https://pubchem.ncbi.nlm.nih.gov/compound/87965681#section=2D-Structure

https://pubchem.ncbi.nlm.nih.gov/compound/87965227#section=2D-Structure
https://pubchem.ncbi.nlm.nih.gov/compound/87964482#section=2D-Structure
https://pubchem.ncbi.nlm.nih.gov/compound/87961963#section=2D-Structure
https://pubchem.ncbi.nlm.nih.gov/compound/87958597#section=2D-Structure
https://pubchem.ncbi.nlm.nih.gov/compound/87957417#section=2D-Structure
https://pubchem.ncbi.nlm.nih.gov/compound/87956997#section=2D-Structure
https://pubchem.ncbi.nlm.nih.gov/compound/87956777#section=2D-Structure
https://pubchem.ncbi.nlm.nih.gov/compound/87956542#section=2D-Structure
https://pubchem.ncbi.nlm.nih.gov/compound/87956217#section=2D-Structure
https://pubchem.ncbi.nlm.nih.gov/compound/87956211#section=2D-Structure
https://pubchem.ncbi.nlm.nih.gov/compound/87955122#section=2D-Structure
https://pubchem.ncbi.nlm.nih.gov/compound/87952724#section=2D-Structure
https://pubchem.ncbi.nlm.nih.gov/compound/87952526#section=2D-Structure
https://pubchem.ncbi.nlm.nih.gov/compound/87952173#section=2D-Structure
https://pubchem.ncbi.nlm.nih.gov/compound/87949965#section=2D-Structure
https://pubchem.ncbi.nlm.nih.gov/compound/87946176#section=2D-Structure
https://pubchem.ncbi.nlm.nih.gov/compound/87944722#section=2D-Structure
https://pubchem.ncbi.nlm.nih.gov/compound/87941785#section=2D-Structure
https://pubchem.ncbi.nlm.nih.gov/compound/87940714#section=2D-Structure
https://pubchem.ncbi.nlm.nih.gov/compound/87933630#section=2D-Structure
https://pubchem.ncbi.nlm.nih.gov/compound/87932746#section=2D-Structure
https://pubchem.ncbi.nlm.nih.gov/compound/87931863#section=2D-Structure
https://pubchem.ncbi.nlm.nih.gov/compound/87930325#section=2D-Structure
https://pubchem.ncbi.nlm.nih.gov/compound/87929324#section=2D-Structure
https://pubchem.ncbi.nlm.nih.gov/compound/87926438#section=2D-Structure
https://pubchem.ncbi.nlm.nih.gov/compound/87923110#section=2D-Structure
https://pubchem.ncbi.nlm.nih.gov/compound/87918470#section=2D-Structure
https://pubchem.ncbi.nlm.nih.gov/compound/87907035#section=2D-Structure
https://pubchem.ncbi.nlm.nih.gov/compound/87907024#section=2D-Structure
https://pubchem.ncbi.nlm.nih.gov/compound/87907017#section=2D-Structure
https://pubchem.ncbi.nlm.nih.gov/compound/87907014#section=2D-Structure
https://pubchem.ncbi.nlm.nih.gov/compound/87907011#section=2D-Structure
https://pubchem.ncbi.nlm.nih.gov/compound/87907009#section=2D-Structure
https://pubchem.ncbi.nlm.nih.gov/compound/87907002#section=2D-Structure
https://pubchem.ncbi.nlm.nih.gov/compound/87907000#section=2D-Structure
https://pubchem.ncbi.nlm.nih.gov/compound/87906997#section=2D-Structure
https://pubchem.ncbi.nlm.nih.gov/compound/87906996#section=2D-Structure
https://pubchem.ncbi.nlm.nih.gov/compound/87906987#section=2D-Structure
https://pubchem.ncbi.nlm.nih.gov/compound/87906983#section=2D-Structure
https://pubchem.ncbi.nlm.nih.gov/compound/87906982#section=2D-Structure
https://pubchem.ncbi.nlm.nih.gov/compound/87906980#section=2D-Structure
https://pubchem.ncbi.nlm.nih.gov/compound/87906962#section=2D-Structure
https://pubchem.ncbi.nlm.nih.gov/compound/87906960#section=2D-Structure
https://pubchem.ncbi.nlm.nih.gov/compound/87906953#section=2D-Structure
https://pubchem.ncbi.nlm.nih.gov/compound/87906940#section=2D-Structure
https://pubchem.ncbi.nlm.nih.gov/compound/87906926#section=2D-Structure
https://pubchem.ncbi.nlm.nih.gov/compound/87906907#section=2D-Structure
https://pubchem.ncbi.nlm.nih.gov/compound/87906897#section=2D-Structure
https://pubchem.ncbi.nlm.nih.gov/compound/87905008#section=2D-Structure
https://pubchem.ncbi.nlm.nih.gov/compound/87902907#section=2D-Structure
https://pubchem.ncbi.nlm.nih.gov/compound/87902892#section=2D-Structure
https://pubchem.ncbi.nlm.nih.gov/compound/87900589#section=2D-Structure
https://pubchem.ncbi.nlm.nih.gov/compound/87894394#section=2D-Structure
https://pubchem.ncbi.nlm.nih.gov/compound/87892356#section=2D-Structure
https://pubchem.ncbi.nlm.nih.gov/compound/87887352#section=2D-Structure

https://pubchem.ncbi.nlm.nih.gov/compound/87886653#section=2D-Structure
https://pubchem.ncbi.nlm.nih.gov/compound/87886463#section=2D-Structure
https://pubchem.ncbi.nlm.nih.gov/compound/87881340#section=2D-Structure
https://pubchem.ncbi.nlm.nih.gov/compound/87879947#section=2D-Structure
https://pubchem.ncbi.nlm.nih.gov/compound/87879216#section=2D-Structure
https://pubchem.ncbi.nlm.nih.gov/compound/87871492#section=2D-Structure
https://pubchem.ncbi.nlm.nih.gov/compound/87865915#section=2D-Structure
https://pubchem.ncbi.nlm.nih.gov/compound/87864100#section=2D-Structure
https://pubchem.ncbi.nlm.nih.gov/compound/87861918#section=2D-Structure
https://pubchem.ncbi.nlm.nih.gov/compound/87857299#section=2D-Structure
https://pubchem.ncbi.nlm.nih.gov/compound/87856937#section=2D-Structure
https://pubchem.ncbi.nlm.nih.gov/compound/87855245#section=2D-Structure
https://pubchem.ncbi.nlm.nih.gov/compound/87849396#section=2D-Structure
https://pubchem.ncbi.nlm.nih.gov/compound/87849168#section=2D-Structure
https://pubchem.ncbi.nlm.nih.gov/compound/87846491#section=2D-Structure
https://pubchem.ncbi.nlm.nih.gov/compound/87842856#section=2D-Structure
https://pubchem.ncbi.nlm.nih.gov/compound/87828188#section=2D-Structure
https://pubchem.ncbi.nlm.nih.gov/compound/87827350#section=2D-Structure
https://pubchem.ncbi.nlm.nih.gov/compound/87825171#section=2D-Structure
https://pubchem.ncbi.nlm.nih.gov/compound/87817058#section=2D-Structure
https://pubchem.ncbi.nlm.nih.gov/compound/87813693#section=2D-Structure
https://pubchem.ncbi.nlm.nih.gov/compound/87803600#section=2D-Structure
https://pubchem.ncbi.nlm.nih.gov/compound/87802549#section=2D-Structure
https://pubchem.ncbi.nlm.nih.gov/compound/87802448#section=2D-Structure
https://pubchem.ncbi.nlm.nih.gov/compound/87794582#section=2D-Structure
https://pubchem.ncbi.nlm.nih.gov/compound/87794574#section=2D-Structure
https://pubchem.ncbi.nlm.nih.gov/compound/87794516#section=2D-Structure
https://pubchem.ncbi.nlm.nih.gov/compound/87793416#section=2D-Structure
https://pubchem.ncbi.nlm.nih.gov/compound/87792981#section=2D-Structure
https://pubchem.ncbi.nlm.nih.gov/compound/87787488#section=2D-Structure
https://pubchem.ncbi.nlm.nih.gov/compound/87784468#section=2D-Structure
https://pubchem.ncbi.nlm.nih.gov/compound/87782231#section=2D-Structure
https://pubchem.ncbi.nlm.nih.gov/compound/87781995#section=2D-Structure
https://pubchem.ncbi.nlm.nih.gov/compound/87780992#section=2D-Structure
https://pubchem.ncbi.nlm.nih.gov/compound/87779928#section=2D-Structure
https://pubchem.ncbi.nlm.nih.gov/compound/87779916#section=2D-Structure
https://pubchem.ncbi.nlm.nih.gov/compound/87779213#section=2D-Structure
https://pubchem.ncbi.nlm.nih.gov/compound/87775408#section=2D-Structure
https://pubchem.ncbi.nlm.nih.gov/compound/87772566#section=2D-Structure
https://pubchem.ncbi.nlm.nih.gov/compound/87770743#section=2D-Structure
https://pubchem.ncbi.nlm.nih.gov/compound/87769772#section=2D-Structure
https://pubchem.ncbi.nlm.nih.gov/compound/87769742#section=2D-Structure
https://pubchem.ncbi.nlm.nih.gov/compound/87768862#section=2D-Structure
https://pubchem.ncbi.nlm.nih.gov/compound/87766750#section=2D-Structure
https://pubchem.ncbi.nlm.nih.gov/compound/87766675#section=2D-Structure
https://pubchem.ncbi.nlm.nih.gov/compound/87765980#section=2D-Structure
https://pubchem.ncbi.nlm.nih.gov/compound/87765120#section=2D-Structure
https://pubchem.ncbi.nlm.nih.gov/compound/87763076#section=2D-Structure
https://pubchem.ncbi.nlm.nih.gov/compound/87763075#section=2D-Structure
https://pubchem.ncbi.nlm.nih.gov/compound/87755863#section=2D-Structure
https://pubchem.ncbi.nlm.nih.gov/compound/87755089#section=2D-Structure
https://pubchem.ncbi.nlm.nih.gov/compound/87752639#section=2D-Structure
https://pubchem.ncbi.nlm.nih.gov/compound/87752382#section=2D-Structure
https://pubchem.ncbi.nlm.nih.gov/compound/87752163#section=2D-Structure
https://pubchem.ncbi.nlm.nih.gov/compound/87735#section=2D-Structure

https://pubchem.ncbi.nlm.nih.gov/compound/87732636#section=2D-Structure
https://pubchem.ncbi.nlm.nih.gov/compound/87727623#section=2D-Structure
https://pubchem.ncbi.nlm.nih.gov/compound/87726285#section=2D-Structure
https://pubchem.ncbi.nlm.nih.gov/compound/87724934#section=2D-Structure
https://pubchem.ncbi.nlm.nih.gov/compound/87724682#section=2D-Structure
https://pubchem.ncbi.nlm.nih.gov/compound/87724590#section=2D-Structure
https://pubchem.ncbi.nlm.nih.gov/compound/87724484#section=2D-Structure
https://pubchem.ncbi.nlm.nih.gov/compound/87713398#section=2D-Structure
https://pubchem.ncbi.nlm.nih.gov/compound/87708310#section=2D-Structure
https://pubchem.ncbi.nlm.nih.gov/compound/87705818#section=2D-Structure
https://pubchem.ncbi.nlm.nih.gov/compound/87704074#section=2D-Structure
https://pubchem.ncbi.nlm.nih.gov/compound/87701685#section=2D-Structure
https://pubchem.ncbi.nlm.nih.gov/compound/87701673#section=2D-Structure
https://pubchem.ncbi.nlm.nih.gov/compound/87700379#section=2D-Structure
https://pubchem.ncbi.nlm.nih.gov/compound/87696619#section=2D-Structure
https://pubchem.ncbi.nlm.nih.gov/compound/87693941#section=2D-Structure
https://pubchem.ncbi.nlm.nih.gov/compound/87691308#section=2D-Structure
https://pubchem.ncbi.nlm.nih.gov/compound/87679177#section=2D-Structure
https://pubchem.ncbi.nlm.nih.gov/compound/87677337#section=2D-Structure
https://pubchem.ncbi.nlm.nih.gov/compound/87676507#section=2D-Structure
https://pubchem.ncbi.nlm.nih.gov/compound/87675351#section=2D-Structure
https://pubchem.ncbi.nlm.nih.gov/compound/87674993#section=2D-Structure
https://pubchem.ncbi.nlm.nih.gov/compound/87668511#section=2D-Structure
https://pubchem.ncbi.nlm.nih.gov/compound/87668405#section=2D-Structure
https://pubchem.ncbi.nlm.nih.gov/compound/87668298#section=2D-Structure
https://pubchem.ncbi.nlm.nih.gov/compound/87668046#section=2D-Structure
https://pubchem.ncbi.nlm.nih.gov/compound/87667440#section=2D-Structure
https://pubchem.ncbi.nlm.nih.gov/compound/87667150#section=2D-Structure
https://pubchem.ncbi.nlm.nih.gov/compound/87667079#section=2D-Structure
https://pubchem.ncbi.nlm.nih.gov/compound/87664673#section=2D-Structure
https://pubchem.ncbi.nlm.nih.gov/compound/87663759#section=2D-Structure
https://pubchem.ncbi.nlm.nih.gov/compound/87660241#section=2D-Structure
https://pubchem.ncbi.nlm.nih.gov/compound/87660154#section=2D-Structure
https://pubchem.ncbi.nlm.nih.gov/compound/87656029#section=2D-Structure
https://pubchem.ncbi.nlm.nih.gov/compound/87652757#section=2D-Structure
https://pubchem.ncbi.nlm.nih.gov/compound/87650251#section=2D-Structure
https://pubchem.ncbi.nlm.nih.gov/compound/87649882#section=2D-Structure
https://pubchem.ncbi.nlm.nih.gov/compound/87647486#section=2D-Structure
https://pubchem.ncbi.nlm.nih.gov/compound/87647378#section=2D-Structure
https://pubchem.ncbi.nlm.nih.gov/compound/87645505#section=2D-Structure
https://pubchem.ncbi.nlm.nih.gov/compound/87644040#section=2D-Structure
https://pubchem.ncbi.nlm.nih.gov/compound/87639303#section=2D-Structure
https://pubchem.ncbi.nlm.nih.gov/compound/87638589#section=2D-Structure
https://pubchem.ncbi.nlm.nih.gov/compound/87637787#section=2D-Structure
https://pubchem.ncbi.nlm.nih.gov/compound/87631774#section=2D-Structure
https://pubchem.ncbi.nlm.nih.gov/compound/87631066#section=2D-Structure
https://pubchem.ncbi.nlm.nih.gov/compound/87630426#section=2D-Structure
https://pubchem.ncbi.nlm.nih.gov/compound/87625170#section=2D-Structure
https://pubchem.ncbi.nlm.nih.gov/compound/87625071#section=2D-Structure
https://pubchem.ncbi.nlm.nih.gov/compound/87624371#section=2D-Structure
https://pubchem.ncbi.nlm.nih.gov/compound/87621035#section=2D-Structure
https://pubchem.ncbi.nlm.nih.gov/compound/87617112#section=2D-Structure
https://pubchem.ncbi.nlm.nih.gov/compound/87612655#section=2D-Structure
https://pubchem.ncbi.nlm.nih.gov/compound/87604864#section=2D-Structure
https://pubchem.ncbi.nlm.nih.gov/compound/87604710#section=2D-Structure

https://pubchem.ncbi.nlm.nih.gov/compound/87595023#section=2D-Structure
https://pubchem.ncbi.nlm.nih.gov/compound/87592046#section=2D-Structure
https://pubchem.ncbi.nlm.nih.gov/compound/87585373#section=2D-Structure
https://pubchem.ncbi.nlm.nih.gov/compound/87581807#section=2D-Structure
https://pubchem.ncbi.nlm.nih.gov/compound/87581806#section=2D-Structure
https://pubchem.ncbi.nlm.nih.gov/compound/87579484#section=2D-Structure
https://pubchem.ncbi.nlm.nih.gov/compound/87578240#section=2D-Structure
https://pubchem.ncbi.nlm.nih.gov/compound/87567725#section=2D-Structure
https://pubchem.ncbi.nlm.nih.gov/compound/87565208#section=2D-Structure
https://pubchem.ncbi.nlm.nih.gov/compound/87565124#section=2D-Structure
https://pubchem.ncbi.nlm.nih.gov/compound/87563605#section=2D-Structure
https://pubchem.ncbi.nlm.nih.gov/compound/87554976#section=2D-Structure
https://pubchem.ncbi.nlm.nih.gov/compound/87554696#section=2D-Structure
https://pubchem.ncbi.nlm.nih.gov/compound/87551408#section=2D-Structure
https://pubchem.ncbi.nlm.nih.gov/compound/87550236#section=2D-Structure
https://pubchem.ncbi.nlm.nih.gov/compound/87544759#section=2D-Structure
https://pubchem.ncbi.nlm.nih.gov/compound/87544098#section=2D-Structure
https://pubchem.ncbi.nlm.nih.gov/compound/87541817#section=2D-Structure
https://pubchem.ncbi.nlm.nih.gov/compound/87537339#section=2D-Structure
https://pubchem.ncbi.nlm.nih.gov/compound/87536945#section=2D-Structure
https://pubchem.ncbi.nlm.nih.gov/compound/87535893#section=2D-Structure
https://pubchem.ncbi.nlm.nih.gov/compound/87534563#section=2D-Structure
https://pubchem.ncbi.nlm.nih.gov/compound/87531479#section=2D-Structure
https://pubchem.ncbi.nlm.nih.gov/compound/87531307#section=2D-Structure
https://pubchem.ncbi.nlm.nih.gov/compound/87529671#section=2D-Structure
https://pubchem.ncbi.nlm.nih.gov/compound/87528467#section=2D-Structure
https://pubchem.ncbi.nlm.nih.gov/compound/87527579#section=2D-Structure
https://pubchem.ncbi.nlm.nih.gov/compound/87527096#section=2D-Structure
https://pubchem.ncbi.nlm.nih.gov/compound/87525619#section=2D-Structure
https://pubchem.ncbi.nlm.nih.gov/compound/87522063#section=2D-Structure
https://pubchem.ncbi.nlm.nih.gov/compound/87521654#section=2D-Structure
https://pubchem.ncbi.nlm.nih.gov/compound/87521286#section=2D-Structure
https://pubchem.ncbi.nlm.nih.gov/compound/87520933#section=2D-Structure
https://pubchem.ncbi.nlm.nih.gov/compound/87519722#section=2D-Structure
https://pubchem.ncbi.nlm.nih.gov/compound/87519171#section=2D-Structure
https://pubchem.ncbi.nlm.nih.gov/compound/87518066#section=2D-Structure
https://pubchem.ncbi.nlm.nih.gov/compound/87515056#section=2D-Structure
https://pubchem.ncbi.nlm.nih.gov/compound/87508416#section=2D-Structure
https://pubchem.ncbi.nlm.nih.gov/compound/87495530#section=2D-Structure
https://pubchem.ncbi.nlm.nih.gov/compound/87494349#section=2D-Structure
https://pubchem.ncbi.nlm.nih.gov/compound/87494174#section=2D-Structure
https://pubchem.ncbi.nlm.nih.gov/compound/87492765#section=2D-Structure
https://pubchem.ncbi.nlm.nih.gov/compound/87491670#section=2D-Structure
https://pubchem.ncbi.nlm.nih.gov/compound/87491538#section=2D-Structure
https://pubchem.ncbi.nlm.nih.gov/compound/87491065#section=2D-Structure
https://pubchem.ncbi.nlm.nih.gov/compound/87490799#section=2D-Structure
https://pubchem.ncbi.nlm.nih.gov/compound/87490288#section=2D-Structure
https://pubchem.ncbi.nlm.nih.gov/compound/87490076#section=2D-Structure
https://pubchem.ncbi.nlm.nih.gov/compound/87489562#section=2D-Structure
https://pubchem.ncbi.nlm.nih.gov/compound/87489425#section=2D-Structure
https://pubchem.ncbi.nlm.nih.gov/compound/87484974#section=2D-Structure
https://pubchem.ncbi.nlm.nih.gov/compound/87479394#section=2D-Structure
https://pubchem.ncbi.nlm.nih.gov/compound/87478790#section=2D-Structure
https://pubchem.ncbi.nlm.nih.gov/compound/87477910#section=2D-Structure
https://pubchem.ncbi.nlm.nih.gov/compound/87477182#section=2D-Structure

https://pubchem.ncbi.nlm.nih.gov/compound/87476572#section=2D-Structure
https://pubchem.ncbi.nlm.nih.gov/compound/87476488#section=2D-Structure
https://pubchem.ncbi.nlm.nih.gov/compound/87476177#section=2D-Structure
https://pubchem.ncbi.nlm.nih.gov/compound/87475109#section=2D-Structure
https://pubchem.ncbi.nlm.nih.gov/compound/87474879#section=2D-Structure
https://pubchem.ncbi.nlm.nih.gov/compound/87474758#section=2D-Structure
https://pubchem.ncbi.nlm.nih.gov/compound/87474405#section=2D-Structure
https://pubchem.ncbi.nlm.nih.gov/compound/87471709#section=2D-Structure
https://pubchem.ncbi.nlm.nih.gov/compound/87470806#section=2D-Structure
https://pubchem.ncbi.nlm.nih.gov/compound/87463690#section=2D-Structure
https://pubchem.ncbi.nlm.nih.gov/compound/87457049#section=2D-Structure
https://pubchem.ncbi.nlm.nih.gov/compound/87456249#section=2D-Structure
https://pubchem.ncbi.nlm.nih.gov/compound/87454103#section=2D-Structure
https://pubchem.ncbi.nlm.nih.gov/compound/87450376#section=2D-Structure
https://pubchem.ncbi.nlm.nih.gov/compound/87447035#section=2D-Structure
https://pubchem.ncbi.nlm.nih.gov/compound/87445814#section=2D-Structure
https://pubchem.ncbi.nlm.nih.gov/compound/87442631#section=2D-Structure
https://pubchem.ncbi.nlm.nih.gov/compound/87441730#section=2D-Structure
https://pubchem.ncbi.nlm.nih.gov/compound/87432093#section=2D-Structure
https://pubchem.ncbi.nlm.nih.gov/compound/87429729#section=2D-Structure
https://pubchem.ncbi.nlm.nih.gov/compound/87428141#section=2D-Structure
https://pubchem.ncbi.nlm.nih.gov/compound/87423895#section=2D-Structure
https://pubchem.ncbi.nlm.nih.gov/compound/87423877#section=2D-Structure
https://pubchem.ncbi.nlm.nih.gov/compound/87420115#section=2D-Structure
https://pubchem.ncbi.nlm.nih.gov/compound/87416925#section=2D-Structure
https://pubchem.ncbi.nlm.nih.gov/compound/87416761#section=2D-Structure
https://pubchem.ncbi.nlm.nih.gov/compound/87407906#section=2D-Structure
https://pubchem.ncbi.nlm.nih.gov/compound/87407134#section=2D-Structure
https://pubchem.ncbi.nlm.nih.gov/compound/87400395#section=2D-Structure
https://pubchem.ncbi.nlm.nih.gov/compound/87399813#section=2D-Structure
https://pubchem.ncbi.nlm.nih.gov/compound/87399547#section=2D-Structure
https://pubchem.ncbi.nlm.nih.gov/compound/87385037#section=2D-Structure
https://pubchem.ncbi.nlm.nih.gov/compound/87383392#section=2D-Structure
https://pubchem.ncbi.nlm.nih.gov/compound/87381853#section=2D-Structure
https://pubchem.ncbi.nlm.nih.gov/compound/87381299#section=2D-Structure
https://pubchem.ncbi.nlm.nih.gov/compound/87369041#section=2D-Structure
https://pubchem.ncbi.nlm.nih.gov/compound/87367183#section=2D-Structure
https://pubchem.ncbi.nlm.nih.gov/compound/87364754#section=2D-Structure
https://pubchem.ncbi.nlm.nih.gov/compound/87361649#section=2D-Structure
https://pubchem.ncbi.nlm.nih.gov/compound/87360835#section=2D-Structure
https://pubchem.ncbi.nlm.nih.gov/compound/87359342#section=2D-Structure
https://pubchem.ncbi.nlm.nih.gov/compound/87356925#section=2D-Structure
https://pubchem.ncbi.nlm.nih.gov/compound/87350051#section=2D-Structure
https://pubchem.ncbi.nlm.nih.gov/compound/87347278#section=2D-Structure
https://pubchem.ncbi.nlm.nih.gov/compound/87343681#section=2D-Structure
https://pubchem.ncbi.nlm.nih.gov/compound/87330823#section=2D-Structure
https://pubchem.ncbi.nlm.nih.gov/compound/87329071#section=2D-Structure
https://pubchem.ncbi.nlm.nih.gov/compound/87328920#section=2D-Structure
https://pubchem.ncbi.nlm.nih.gov/compound/87327824#section=2D-Structure
https://pubchem.ncbi.nlm.nih.gov/compound/87327246#section=2D-Structure
https://pubchem.ncbi.nlm.nih.gov/compound/87324747#section=2D-Structure
https://pubchem.ncbi.nlm.nih.gov/compound/87314922#section=2D-Structure
https://pubchem.ncbi.nlm.nih.gov/compound/87311878#section=2D-Structure
https://pubchem.ncbi.nlm.nih.gov/compound/87304617#section=2D-Structure
https://pubchem.ncbi.nlm.nih.gov/compound/87303983#section=2D-Structure

https://pubchem.ncbi.nlm.nih.gov/compound/87303058#section=2D-Structure
https://pubchem.ncbi.nlm.nih.gov/compound/87298764#section=2D-Structure
https://pubchem.ncbi.nlm.nih.gov/compound/87297165#section=2D-Structure
https://pubchem.ncbi.nlm.nih.gov/compound/87290000#section=2D-Structure
https://pubchem.ncbi.nlm.nih.gov/compound/87287610#section=2D-Structure
https://pubchem.ncbi.nlm.nih.gov/compound/87287050#section=2D-Structure
https://pubchem.ncbi.nlm.nih.gov/compound/87283671#section=2D-Structure
https://pubchem.ncbi.nlm.nih.gov/compound/87271350#section=2D-Structure
https://pubchem.ncbi.nlm.nih.gov/compound/872663#section=2D-Structure
https://pubchem.ncbi.nlm.nih.gov/compound/87257093#section=2D-Structure
https://pubchem.ncbi.nlm.nih.gov/compound/87256756#section=2D-Structure
https://pubchem.ncbi.nlm.nih.gov/compound/87256751#section=2D-Structure
https://pubchem.ncbi.nlm.nih.gov/compound/87253930#section=2D-Structure
https://pubchem.ncbi.nlm.nih.gov/compound/87253465#section=2D-Structure
https://pubchem.ncbi.nlm.nih.gov/compound/87244525#section=2D-Structure
https://pubchem.ncbi.nlm.nih.gov/compound/87237808#section=2D-Structure
https://pubchem.ncbi.nlm.nih.gov/compound/87228201#section=2D-Structure
https://pubchem.ncbi.nlm.nih.gov/compound/87226084#section=2D-Structure
https://pubchem.ncbi.nlm.nih.gov/compound/87218255#section=2D-Structure
https://pubchem.ncbi.nlm.nih.gov/compound/87218254#section=2D-Structure
https://pubchem.ncbi.nlm.nih.gov/compound/87216495#section=2D-Structure
https://pubchem.ncbi.nlm.nih.gov/compound/87216269#section=2D-Structure
https://pubchem.ncbi.nlm.nih.gov/compound/87208150#section=2D-Structure
https://pubchem.ncbi.nlm.nih.gov/compound/87206190#section=2D-Structure
https://pubchem.ncbi.nlm.nih.gov/compound/87205151#section=2D-Structure
https://pubchem.ncbi.nlm.nih.gov/compound/87205136#section=2D-Structure
https://pubchem.ncbi.nlm.nih.gov/compound/87201693#section=2D-Structure
https://pubchem.ncbi.nlm.nih.gov/compound/87201619#section=2D-Structure
https://pubchem.ncbi.nlm.nih.gov/compound/87195222#section=2D-Structure
https://pubchem.ncbi.nlm.nih.gov/compound/87194852#section=2D-Structure
https://pubchem.ncbi.nlm.nih.gov/compound/87177121#section=2D-Structure
https://pubchem.ncbi.nlm.nih.gov/compound/87175290#section=2D-Structure
https://pubchem.ncbi.nlm.nih.gov/compound/87165947#section=2D-Structure
https://pubchem.ncbi.nlm.nih.gov/compound/87158330#section=2D-Structure
https://pubchem.ncbi.nlm.nih.gov/compound/87158#section=2D-Structure
https://pubchem.ncbi.nlm.nih.gov/compound/87139601#section=2D-Structure
https://pubchem.ncbi.nlm.nih.gov/compound/87131920#section=2D-Structure
https://pubchem.ncbi.nlm.nih.gov/compound/87131369#section=2D-Structure
https://pubchem.ncbi.nlm.nih.gov/compound/87127676#section=2D-Structure
https://pubchem.ncbi.nlm.nih.gov/compound/87126344#section=2D-Structure
https://pubchem.ncbi.nlm.nih.gov/compound/87119839#section=2D-Structure
https://pubchem.ncbi.nlm.nih.gov/compound/87119549#section=2D-Structure
https://pubchem.ncbi.nlm.nih.gov/compound/87111168#section=2D-Structure
https://pubchem.ncbi.nlm.nih.gov/compound/87109202#section=2D-Structure
https://pubchem.ncbi.nlm.nih.gov/compound/87107973#section=2D-Structure
https://pubchem.ncbi.nlm.nih.gov/compound/87107289#section=2D-Structure
https://pubchem.ncbi.nlm.nih.gov/compound/87106394#section=2D-Structure
https://pubchem.ncbi.nlm.nih.gov/compound/87106294#section=2D-Structure
https://pubchem.ncbi.nlm.nih.gov/compound/87106202#section=2D-Structure
https://pubchem.ncbi.nlm.nih.gov/compound/87106084#section=2D-Structure
https://pubchem.ncbi.nlm.nih.gov/compound/87104537#section=2D-Structure
https://pubchem.ncbi.nlm.nih.gov/compound/87097744#section=2D-Structure
https://pubchem.ncbi.nlm.nih.gov/compound/87091738#section=2D-Structure
https://pubchem.ncbi.nlm.nih.gov/compound/87087142#section=2D-Structure
https://pubchem.ncbi.nlm.nih.gov/compound/87074307#section=2D-Structure

https://pubchem.ncbi.nlm.nih.gov/compound/87072684#section=2D-Structure
https://pubchem.ncbi.nlm.nih.gov/compound/87072007#section=2D-Structure
https://pubchem.ncbi.nlm.nih.gov/compound/87068579#section=2D-Structure
https://pubchem.ncbi.nlm.nih.gov/compound/87064713#section=2D-Structure
https://pubchem.ncbi.nlm.nih.gov/compound/87061101#section=2D-Structure
https://pubchem.ncbi.nlm.nih.gov/compound/87057790#section=2D-Structure
https://pubchem.ncbi.nlm.nih.gov/compound/86757226#section=2D-Structure
https://pubchem.ncbi.nlm.nih.gov/compound/86754514#section=2D-Structure
https://pubchem.ncbi.nlm.nih.gov/compound/86750165#section=2D-Structure
https://pubchem.ncbi.nlm.nih.gov/compound/86744960#section=2D-Structure
https://pubchem.ncbi.nlm.nih.gov/compound/86718837#section=2D-Structure
https://pubchem.ncbi.nlm.nih.gov/compound/86674037#section=2D-Structure
https://pubchem.ncbi.nlm.nih.gov/compound/86633129#section=2D-Structure
https://pubchem.ncbi.nlm.nih.gov/compound/86610186#section=2D-Structure
https://pubchem.ncbi.nlm.nih.gov/compound/86588978#section=2D-Structure
https://pubchem.ncbi.nlm.nih.gov/compound/86587025#section=2D-Structure
https://pubchem.ncbi.nlm.nih.gov/compound/86586492#section=2D-Structure
https://pubchem.ncbi.nlm.nih.gov/compound/86569022#section=2D-Structure
https://pubchem.ncbi.nlm.nih.gov/compound/865236#section=2D-Structure
https://pubchem.ncbi.nlm.nih.gov/compound/865235#section=2D-Structure
https://pubchem.ncbi.nlm.nih.gov/compound/865234#section=2D-Structure
https://pubchem.ncbi.nlm.nih.gov/compound/86340837#section=2D-Structure
https://pubchem.ncbi.nlm.nih.gov/compound/86340824#section=2D-Structure
https://pubchem.ncbi.nlm.nih.gov/compound/86310205#section=2D-Structure
https://pubchem.ncbi.nlm.nih.gov/compound/86308963#section=2D-Structure
https://pubchem.ncbi.nlm.nih.gov/compound/86280394#section=2D-Structure
https://pubchem.ncbi.nlm.nih.gov/compound/86270510#section=2D-Structure
https://pubchem.ncbi.nlm.nih.gov/compound/86270509#section=2D-Structure
https://pubchem.ncbi.nlm.nih.gov/compound/86268643#section=2D-Structure
https://pubchem.ncbi.nlm.nih.gov/compound/86268642#section=2D-Structure
https://pubchem.ncbi.nlm.nih.gov/compound/86268439#section=2D-Structure
https://pubchem.ncbi.nlm.nih.gov/compound/86237759#section=2D-Structure
https://pubchem.ncbi.nlm.nih.gov/compound/86230518#section=2D-Structure
https://pubchem.ncbi.nlm.nih.gov/compound/86223317#section=2D-Structure
https://pubchem.ncbi.nlm.nih.gov/compound/86212943#section=2D-Structure
https://pubchem.ncbi.nlm.nih.gov/compound/86212931#section=2D-Structure
https://pubchem.ncbi.nlm.nih.gov/compound/86206016#section=2D-Structure
https://pubchem.ncbi.nlm.nih.gov/compound/86205965#section=2D-Structure
https://pubchem.ncbi.nlm.nih.gov/compound/86203786#section=2D-Structure
https://pubchem.ncbi.nlm.nih.gov/compound/86200800#section=2D-Structure
https://pubchem.ncbi.nlm.nih.gov/compound/86187161#section=2D-Structure
https://pubchem.ncbi.nlm.nih.gov/compound/86185380#section=2D-Structure
https://pubchem.ncbi.nlm.nih.gov/compound/86175329#section=2D-Structure
https://pubchem.ncbi.nlm.nih.gov/compound/86175120#section=2D-Structure
https://pubchem.ncbi.nlm.nih.gov/compound/86173787#section=2D-Structure
https://pubchem.ncbi.nlm.nih.gov/compound/86169630#section=2D-Structure
https://pubchem.ncbi.nlm.nih.gov/compound/86157358#section=2D-Structure
https://pubchem.ncbi.nlm.nih.gov/compound/86114644#section=2D-Structure
https://pubchem.ncbi.nlm.nih.gov/compound/86109570#section=2D-Structure
https://pubchem.ncbi.nlm.nih.gov/compound/86104211#section=2D-Structure
https://pubchem.ncbi.nlm.nih.gov/compound/86104009#section=2D-Structure
https://pubchem.ncbi.nlm.nih.gov/compound/86103873#section=2D-Structure
https://pubchem.ncbi.nlm.nih.gov/compound/86103869#section=2D-Structure
https://pubchem.ncbi.nlm.nih.gov/compound/86103865#section=2D-Structure
https://pubchem.ncbi.nlm.nih.gov/compound/86103805#section=2D-Structure

https://pubchem.ncbi.nlm.nih.gov/compound/86103715#section=2D-Structure
https://pubchem.ncbi.nlm.nih.gov/compound/86102129#section=2D-Structure
https://pubchem.ncbi.nlm.nih.gov/compound/86094692#section=2D-Structure
https://pubchem.ncbi.nlm.nih.gov/compound/86083273#section=2D-Structure
https://pubchem.ncbi.nlm.nih.gov/compound/86059676#section=2D-Structure
https://pubchem.ncbi.nlm.nih.gov/compound/86056750#section=2D-Structure
https://pubchem.ncbi.nlm.nih.gov/compound/86052653#section=2D-Structure
https://pubchem.ncbi.nlm.nih.gov/compound/86037965#section=2D-Structure
https://pubchem.ncbi.nlm.nih.gov/compound/86036330#section=2D-Structure
https://pubchem.ncbi.nlm.nih.gov/compound/86036196#section=2D-Structure
https://pubchem.ncbi.nlm.nih.gov/compound/86022671#section=2D-Structure
https://pubchem.ncbi.nlm.nih.gov/compound/86021763#section=2D-Structure
https://pubchem.ncbi.nlm.nih.gov/compound/86021636#section=2D-Structure
https://pubchem.ncbi.nlm.nih.gov/compound/86021635#section=2D-Structure
https://pubchem.ncbi.nlm.nih.gov/compound/86017292#section=2D-Structure
https://pubchem.ncbi.nlm.nih.gov/compound/86017138#section=2D-Structure
https://pubchem.ncbi.nlm.nih.gov/compound/86010305#section=2D-Structure
https://pubchem.ncbi.nlm.nih.gov/compound/86007959#section=2D-Structure
https://pubchem.ncbi.nlm.nih.gov/compound/86007783#section=2D-Structure
https://pubchem.ncbi.nlm.nih.gov/compound/86007305#section=2D-Structure
https://pubchem.ncbi.nlm.nih.gov/compound/86001115#section=2D-Structure
https://pubchem.ncbi.nlm.nih.gov/compound/85999116#section=2D-Structure
https://pubchem.ncbi.nlm.nih.gov/compound/85992356#section=2D-Structure
https://pubchem.ncbi.nlm.nih.gov/compound/85980516#section=2D-Structure
https://pubchem.ncbi.nlm.nih.gov/compound/85976244#section=2D-Structure
https://pubchem.ncbi.nlm.nih.gov/compound/85975379#section=2D-Structure
https://pubchem.ncbi.nlm.nih.gov/compound/85972581#section=2D-Structure
https://pubchem.ncbi.nlm.nih.gov/compound/85953966#section=2D-Structure
https://pubchem.ncbi.nlm.nih.gov/compound/85951436#section=2D-Structure
https://pubchem.ncbi.nlm.nih.gov/compound/85942597#section=2D-Structure
https://pubchem.ncbi.nlm.nih.gov/compound/85942593#section=2D-Structure
https://pubchem.ncbi.nlm.nih.gov/compound/85939756#section=2D-Structure
https://pubchem.ncbi.nlm.nih.gov/compound/85935473#section=2D-Structure
https://pubchem.ncbi.nlm.nih.gov/compound/85927509#section=2D-Structure
https://pubchem.ncbi.nlm.nih.gov/compound/85910617#section=2D-Structure
https://pubchem.ncbi.nlm.nih.gov/compound/85907718#section=2D-Structure
https://pubchem.ncbi.nlm.nih.gov/compound/85882497#section=2D-Structure
https://pubchem.ncbi.nlm.nih.gov/compound/85879084#section=2D-Structure
https://pubchem.ncbi.nlm.nih.gov/compound/85877031#section=2D-Structure
https://pubchem.ncbi.nlm.nih.gov/compound/85865540#section=2D-Structure
https://pubchem.ncbi.nlm.nih.gov/compound/85861090#section=2D-Structure
https://pubchem.ncbi.nlm.nih.gov/compound/85861036#section=2D-Structure
https://pubchem.ncbi.nlm.nih.gov/compound/85854494#section=2D-Structure
https://pubchem.ncbi.nlm.nih.gov/compound/85854492#section=2D-Structure
https://pubchem.ncbi.nlm.nih.gov/compound/85854489#section=2D-Structure
https://pubchem.ncbi.nlm.nih.gov/compound/85854439#section=2D-Structure
https://pubchem.ncbi.nlm.nih.gov/compound/85851187#section=2D-Structure
https://pubchem.ncbi.nlm.nih.gov/compound/85835368#section=2D-Structure
https://pubchem.ncbi.nlm.nih.gov/compound/85821157#section=2D-Structure
https://pubchem.ncbi.nlm.nih.gov/compound/85819043#section=2D-Structure
https://pubchem.ncbi.nlm.nih.gov/compound/85817584#section=2D-Structure
https://pubchem.ncbi.nlm.nih.gov/compound/85803251#section=2D-Structure
https://pubchem.ncbi.nlm.nih.gov/compound/85800294#section=2D-Structure
https://pubchem.ncbi.nlm.nih.gov/compound/85795149#section=2D-Structure
https://pubchem.ncbi.nlm.nih.gov/compound/85795147#section=2D-Structure

https://pubchem.ncbi.nlm.nih.gov/compound/85791252#section=2D-Structure
https://pubchem.ncbi.nlm.nih.gov/compound/85790319#section=2D-Structure
https://pubchem.ncbi.nlm.nih.gov/compound/85790317#section=2D-Structure
https://pubchem.ncbi.nlm.nih.gov/compound/85788315#section=2D-Structure
https://pubchem.ncbi.nlm.nih.gov/compound/85787070#section=2D-Structure
https://pubchem.ncbi.nlm.nih.gov/compound/85786223#section=2D-Structure
https://pubchem.ncbi.nlm.nih.gov/compound/85786183#section=2D-Structure
https://pubchem.ncbi.nlm.nih.gov/compound/85784251#section=2D-Structure
https://pubchem.ncbi.nlm.nih.gov/compound/85784247#section=2D-Structure
https://pubchem.ncbi.nlm.nih.gov/compound/85784233#section=2D-Structure
https://pubchem.ncbi.nlm.nih.gov/compound/85776872#section=2D-Structure
https://pubchem.ncbi.nlm.nih.gov/compound/85771746#section=2D-Structure
https://pubchem.ncbi.nlm.nih.gov/compound/85769607#section=2D-Structure
https://pubchem.ncbi.nlm.nih.gov/compound/85766765#section=2D-Structure
https://pubchem.ncbi.nlm.nih.gov/compound/85766763#section=2D-Structure
https://pubchem.ncbi.nlm.nih.gov/compound/85766683#section=2D-Structure
https://pubchem.ncbi.nlm.nih.gov/compound/85766679#section=2D-Structure
https://pubchem.ncbi.nlm.nih.gov/compound/85766678#section=2D-Structure
https://pubchem.ncbi.nlm.nih.gov/compound/85766677#section=2D-Structure
https://pubchem.ncbi.nlm.nih.gov/compound/85756952#section=2D-Structure
https://pubchem.ncbi.nlm.nih.gov/compound/85755791#section=2D-Structure
https://pubchem.ncbi.nlm.nih.gov/compound/85753496#section=2D-Structure
https://pubchem.ncbi.nlm.nih.gov/compound/85745764#section=2D-Structure
https://pubchem.ncbi.nlm.nih.gov/compound/85744503#section=2D-Structure
https://pubchem.ncbi.nlm.nih.gov/compound/85741543#section=2D-Structure
https://pubchem.ncbi.nlm.nih.gov/compound/85737128#section=2D-Structure
https://pubchem.ncbi.nlm.nih.gov/compound/85734033#section=2D-Structure
https://pubchem.ncbi.nlm.nih.gov/compound/85732568#section=2D-Structure
https://pubchem.ncbi.nlm.nih.gov/compound/85723355#section=2D-Structure
https://pubchem.ncbi.nlm.nih.gov/compound/85723238#section=2D-Structure
https://pubchem.ncbi.nlm.nih.gov/compound/85718518#section=2D-Structure
https://pubchem.ncbi.nlm.nih.gov/compound/85709466#section=2D-Structure
https://pubchem.ncbi.nlm.nih.gov/compound/85706837#section=2D-Structure
https://pubchem.ncbi.nlm.nih.gov/compound/85702142#section=2D-Structure
https://pubchem.ncbi.nlm.nih.gov/compound/85702122#section=2D-Structure
https://pubchem.ncbi.nlm.nih.gov/compound/85687455#section=2D-Structure
https://pubchem.ncbi.nlm.nih.gov/compound/85686706#section=2D-Structure
https://pubchem.ncbi.nlm.nih.gov/compound/85667437#section=2D-Structure
https://pubchem.ncbi.nlm.nih.gov/compound/85655051#section=2D-Structure
https://pubchem.ncbi.nlm.nih.gov/compound/85652304#section=2D-Structure
https://pubchem.ncbi.nlm.nih.gov/compound/85651677#section=2D-Structure
https://pubchem.ncbi.nlm.nih.gov/compound/85642896#section=2D-Structure
https://pubchem.ncbi.nlm.nih.gov/compound/85638983#section=2D-Structure
https://pubchem.ncbi.nlm.nih.gov/compound/85633007#section=2D-Structure
https://pubchem.ncbi.nlm.nih.gov/compound/85630311#section=2D-Structure
https://pubchem.ncbi.nlm.nih.gov/compound/85625289#section=2D-Structure
https://pubchem.ncbi.nlm.nih.gov/compound/85616640#section=2D-Structure
https://pubchem.ncbi.nlm.nih.gov/compound/85616066#section=2D-Structure
https://pubchem.ncbi.nlm.nih.gov/compound/85614772#section=2D-Structure
https://pubchem.ncbi.nlm.nih.gov/compound/85612801#section=2D-Structure
https://pubchem.ncbi.nlm.nih.gov/compound/85611358#section=2D-Structure
https://pubchem.ncbi.nlm.nih.gov/compound/85594274#section=2D-Structure
https://pubchem.ncbi.nlm.nih.gov/compound/85579226#section=2D-Structure
https://pubchem.ncbi.nlm.nih.gov/compound/85578657#section=2D-Structure
https://pubchem.ncbi.nlm.nih.gov/compound/85574243#section=2D-Structure

https://pubchem.ncbi.nlm.nih.gov/compound/85563136#section=2D-Structure
https://pubchem.ncbi.nlm.nih.gov/compound/85560130#section=2D-Structure
https://pubchem.ncbi.nlm.nih.gov/compound/85538729#section=2D-Structure
https://pubchem.ncbi.nlm.nih.gov/compound/85533052#section=2D-Structure
https://pubchem.ncbi.nlm.nih.gov/compound/85444442#section=2D-Structure
https://pubchem.ncbi.nlm.nih.gov/compound/85444440#section=2D-Structure
https://pubchem.ncbi.nlm.nih.gov/compound/85440400#section=2D-Structure
https://pubchem.ncbi.nlm.nih.gov/compound/85438530#section=2D-Structure
https://pubchem.ncbi.nlm.nih.gov/compound/85438529#section=2D-Structure
https://pubchem.ncbi.nlm.nih.gov/compound/85437049#section=2D-Structure
https://pubchem.ncbi.nlm.nih.gov/compound/85435446#section=2D-Structure
https://pubchem.ncbi.nlm.nih.gov/compound/85345#section=2D-Structure
https://pubchem.ncbi.nlm.nih.gov/compound/85280065#section=2D-Structure
https://pubchem.ncbi.nlm.nih.gov/compound/85271768#section=2D-Structure
https://pubchem.ncbi.nlm.nih.gov/compound/85263679#section=2D-Structure
https://pubchem.ncbi.nlm.nih.gov/compound/85185824#section=2D-Structure
https://pubchem.ncbi.nlm.nih.gov/compound/85150969#section=2D-Structure
https://pubchem.ncbi.nlm.nih.gov/compound/85132201#section=2D-Structure
https://pubchem.ncbi.nlm.nih.gov/compound/84819741#section=2D-Structure
https://pubchem.ncbi.nlm.nih.gov/compound/84819627#section=2D-Structure
https://pubchem.ncbi.nlm.nih.gov/compound/84655427#section=2D-Structure
https://pubchem.ncbi.nlm.nih.gov/compound/84628#section=2D-Structure
https://pubchem.ncbi.nlm.nih.gov/compound/84627#section=2D-Structure
https://pubchem.ncbi.nlm.nih.gov/compound/84625#section=2D-Structure
https://pubchem.ncbi.nlm.nih.gov/compound/843904#section=2D-Structure
https://pubchem.ncbi.nlm.nih.gov/compound/84195#section=2D-Structure
https://pubchem.ncbi.nlm.nih.gov/compound/84194#section=2D-Structure
https://pubchem.ncbi.nlm.nih.gov/compound/83681646#section=2D-Structure
https://pubchem.ncbi.nlm.nih.gov/compound/83680631#section=2D-Structure
https://pubchem.ncbi.nlm.nih.gov/compound/83646#section=2D-Structure
https://pubchem.ncbi.nlm.nih.gov/compound/823682#section=2D-Structure
https://pubchem.ncbi.nlm.nih.gov/compound/822798#section=2D-Structure
https://pubchem.ncbi.nlm.nih.gov/compound/821129#section=2D-Structure
https://pubchem.ncbi.nlm.nih.gov/compound/82085#section=2D-Structure
https://pubchem.ncbi.nlm.nih.gov/compound/82036380#section=2D-Structure
https://pubchem.ncbi.nlm.nih.gov/compound/81935#section=2D-Structure
https://pubchem.ncbi.nlm.nih.gov/compound/819000#section=2D-Structure
https://pubchem.ncbi.nlm.nih.gov/compound/81547#section=2D-Structure
https://pubchem.ncbi.nlm.nih.gov/compound/813946#section=2D-Structure
https://pubchem.ncbi.nlm.nih.gov/compound/81144#section=2D-Structure
https://pubchem.ncbi.nlm.nih.gov/compound/810842#section=2D-Structure
https://pubchem.ncbi.nlm.nih.gov/compound/78802#section=2D-Structure
https://pubchem.ncbi.nlm.nih.gov/compound/78621#section=2D-Structure
https://pubchem.ncbi.nlm.nih.gov/compound/78545#section=2D-Structure
https://pubchem.ncbi.nlm.nih.gov/compound/78410427#section=2D-Structure
https://pubchem.ncbi.nlm.nih.gov/compound/78400050#section=2D-Structure
https://pubchem.ncbi.nlm.nih.gov/compound/78385875#section=2D-Structure
https://pubchem.ncbi.nlm.nih.gov/compound/78384561#section=2D-Structure
https://pubchem.ncbi.nlm.nih.gov/compound/78379871#section=2D-Structure
https://pubchem.ncbi.nlm.nih.gov/compound/78363934#section=2D-Structure
https://pubchem.ncbi.nlm.nih.gov/compound/78338561#section=2D-Structure
https://pubchem.ncbi.nlm.nih.gov/compound/78335485#section=2D-Structure
https://pubchem.ncbi.nlm.nih.gov/compound/78334470#section=2D-Structure
https://pubchem.ncbi.nlm.nih.gov/compound/78333561#section=2D-Structure
https://pubchem.ncbi.nlm.nih.gov/compound/78326845#section=2D-Structure

https://pubchem.ncbi.nlm.nih.gov/compound/78326551#section=2D-Structure
https://pubchem.ncbi.nlm.nih.gov/compound/78304257#section=2D-Structure
https://pubchem.ncbi.nlm.nih.gov/compound/78296968#section=2D-Structure
https://pubchem.ncbi.nlm.nih.gov/compound/78253435#section=2D-Structure
https://pubchem.ncbi.nlm.nih.gov/compound/78223996#section=2D-Structure
https://pubchem.ncbi.nlm.nih.gov/compound/78207341#section=2D-Structure
https://pubchem.ncbi.nlm.nih.gov/compound/78204452#section=2D-Structure
https://pubchem.ncbi.nlm.nih.gov/compound/78204109#section=2D-Structure
https://pubchem.ncbi.nlm.nih.gov/compound/78202256#section=2D-Structure
https://pubchem.ncbi.nlm.nih.gov/compound/78199524#section=2D-Structure
https://pubchem.ncbi.nlm.nih.gov/compound/78199486#section=2D-Structure
https://pubchem.ncbi.nlm.nih.gov/compound/78199344#section=2D-Structure
https://pubchem.ncbi.nlm.nih.gov/compound/78183197#section=2D-Structure
https://pubchem.ncbi.nlm.nih.gov/compound/78179441#section=2D-Structure
https://pubchem.ncbi.nlm.nih.gov/compound/78179440#section=2D-Structure
https://pubchem.ncbi.nlm.nih.gov/compound/78177279#section=2D-Structure
https://pubchem.ncbi.nlm.nih.gov/compound/78176153#section=2D-Structure
https://pubchem.ncbi.nlm.nih.gov/compound/78175319#section=2D-Structure
https://pubchem.ncbi.nlm.nih.gov/compound/78174616#section=2D-Structure
https://pubchem.ncbi.nlm.nih.gov/compound/78173796#section=2D-Structure
https://pubchem.ncbi.nlm.nih.gov/compound/78171991#section=2D-Structure
https://pubchem.ncbi.nlm.nih.gov/compound/78170550#section=2D-Structure
https://pubchem.ncbi.nlm.nih.gov/compound/78166512#section=2D-Structure
https://pubchem.ncbi.nlm.nih.gov/compound/78103673#section=2D-Structure
https://pubchem.ncbi.nlm.nih.gov/compound/78067850#section=2D-Structure
https://pubchem.ncbi.nlm.nih.gov/compound/77942398#section=2D-Structure
https://pubchem.ncbi.nlm.nih.gov/compound/77844694#section=2D-Structure
https://pubchem.ncbi.nlm.nih.gov/compound/77844693#section=2D-Structure
https://pubchem.ncbi.nlm.nih.gov/compound/77844692#section=2D-Structure
https://pubchem.ncbi.nlm.nih.gov/compound/77835027#section=2D-Structure
https://pubchem.ncbi.nlm.nih.gov/compound/77621081#section=2D-Structure
https://pubchem.ncbi.nlm.nih.gov/compound/77620786#section=2D-Structure
https://pubchem.ncbi.nlm.nih.gov/compound/77620779#section=2D-Structure
https://pubchem.ncbi.nlm.nih.gov/compound/77620605#section=2D-Structure
https://pubchem.ncbi.nlm.nih.gov/compound/77620592#section=2D-Structure
https://pubchem.ncbi.nlm.nih.gov/compound/77620567#section=2D-Structure
https://pubchem.ncbi.nlm.nih.gov/compound/77608812#section=2D-Structure
https://pubchem.ncbi.nlm.nih.gov/compound/77605376#section=2D-Structure
https://pubchem.ncbi.nlm.nih.gov/compound/77520392#section=2D-Structure
https://pubchem.ncbi.nlm.nih.gov/compound/77519926#section=2D-Structure
https://pubchem.ncbi.nlm.nih.gov/compound/77519909#section=2D-Structure
https://pubchem.ncbi.nlm.nih.gov/compound/77161586#section=2D-Structure
https://pubchem.ncbi.nlm.nih.gov/compound/77155903#section=2D-Structure
https://pubchem.ncbi.nlm.nih.gov/compound/76851638#section=2D-Structure
https://pubchem.ncbi.nlm.nih.gov/compound/76851609#section=2D-Structure
https://pubchem.ncbi.nlm.nih.gov/compound/76851456#section=2D-Structure
https://pubchem.ncbi.nlm.nih.gov/compound/76851369#section=2D-Structure
https://pubchem.ncbi.nlm.nih.gov/compound/76851278#section=2D-Structure
https://pubchem.ncbi.nlm.nih.gov/compound/76851213#section=2D-Structure
https://pubchem.ncbi.nlm.nih.gov/compound/76851207#section=2D-Structure
https://pubchem.ncbi.nlm.nih.gov/compound/76851203#section=2D-Structure
https://pubchem.ncbi.nlm.nih.gov/compound/76851189#section=2D-Structure
https://pubchem.ncbi.nlm.nih.gov/compound/76851169#section=2D-Structure
https://pubchem.ncbi.nlm.nih.gov/compound/76851168#section=2D-Structure
https://pubchem.ncbi.nlm.nih.gov/compound/76849753#section=2D-Structure

https://pubchem.ncbi.nlm.nih.gov/compound/76848604#section=2D-Structure
https://pubchem.ncbi.nlm.nih.gov/compound/76848446#section=2D-Structure
https://pubchem.ncbi.nlm.nih.gov/compound/76848294#section=2D-Structure
https://pubchem.ncbi.nlm.nih.gov/compound/76848291#section=2D-Structure
https://pubchem.ncbi.nlm.nih.gov/compound/76847064#section=2D-Structure
https://pubchem.ncbi.nlm.nih.gov/compound/76845720#section=2D-Structure
https://pubchem.ncbi.nlm.nih.gov/compound/76845687#section=2D-Structure
https://pubchem.ncbi.nlm.nih.gov/compound/76845643#section=2D-Structure
https://pubchem.ncbi.nlm.nih.gov/compound/76845631#section=2D-Structure
https://pubchem.ncbi.nlm.nih.gov/compound/76845397#section=2D-Structure
https://pubchem.ncbi.nlm.nih.gov/compound/76845094#section=2D-Structure
https://pubchem.ncbi.nlm.nih.gov/compound/76844803#section=2D-Structure
https://pubchem.ncbi.nlm.nih.gov/compound/76844721#section=2D-Structure
https://pubchem.ncbi.nlm.nih.gov/compound/76844690#section=2D-Structure
https://pubchem.ncbi.nlm.nih.gov/compound/76844684#section=2D-Structure
https://pubchem.ncbi.nlm.nih.gov/compound/76844611#section=2D-Structure
https://pubchem.ncbi.nlm.nih.gov/compound/76844596#section=2D-Structure
https://pubchem.ncbi.nlm.nih.gov/compound/76844539#section=2D-Structure
https://pubchem.ncbi.nlm.nih.gov/compound/76844534#section=2D-Structure
https://pubchem.ncbi.nlm.nih.gov/compound/76681384#section=2D-Structure
https://pubchem.ncbi.nlm.nih.gov/compound/76680947#section=2D-Structure
https://pubchem.ncbi.nlm.nih.gov/compound/76626520#section=2D-Structure
https://pubchem.ncbi.nlm.nih.gov/compound/76601675#section=2D-Structure
https://pubchem.ncbi.nlm.nih.gov/compound/76601671#section=2D-Structure
https://pubchem.ncbi.nlm.nih.gov/compound/765984#section=2D-Structure
https://pubchem.ncbi.nlm.nih.gov/compound/76590836#section=2D-Structure
https://pubchem.ncbi.nlm.nih.gov/compound/76538849#section=2D-Structure
https://pubchem.ncbi.nlm.nih.gov/compound/76533241#section=2D-Structure
https://pubchem.ncbi.nlm.nih.gov/compound/76532929#section=2D-Structure
https://pubchem.ncbi.nlm.nih.gov/compound/76522805#section=2D-Structure
https://pubchem.ncbi.nlm.nih.gov/compound/76518727#section=2D-Structure
https://pubchem.ncbi.nlm.nih.gov/compound/763237#section=2D-Structure
https://pubchem.ncbi.nlm.nih.gov/compound/762736#section=2D-Structure
https://pubchem.ncbi.nlm.nih.gov/compound/76197281#section=2D-Structure
https://pubchem.ncbi.nlm.nih.gov/compound/76168267#section=2D-Structure
https://pubchem.ncbi.nlm.nih.gov/compound/76134031#section=2D-Structure
https://pubchem.ncbi.nlm.nih.gov/compound/76123489#section=2D-Structure
https://pubchem.ncbi.nlm.nih.gov/compound/76109045#section=2D-Structure
https://pubchem.ncbi.nlm.nih.gov/compound/75997745#section=2D-Structure
https://pubchem.ncbi.nlm.nih.gov/compound/75997734#section=2D-Structure
https://pubchem.ncbi.nlm.nih.gov/compound/75996100#section=2D-Structure
https://pubchem.ncbi.nlm.nih.gov/compound/75996095#section=2D-Structure
https://pubchem.ncbi.nlm.nih.gov/compound/75996092#section=2D-Structure
https://pubchem.ncbi.nlm.nih.gov/compound/75996089#section=2D-Structure
https://pubchem.ncbi.nlm.nih.gov/compound/75679220#section=2D-Structure
https://pubchem.ncbi.nlm.nih.gov/compound/755456#section=2D-Structure
https://pubchem.ncbi.nlm.nih.gov/compound/75493009#section=2D-Structure
https://pubchem.ncbi.nlm.nih.gov/compound/75361264#section=2D-Structure
https://pubchem.ncbi.nlm.nih.gov/compound/75273621#section=2D-Structure
https://pubchem.ncbi.nlm.nih.gov/compound/75094969#section=2D-Structure
https://pubchem.ncbi.nlm.nih.gov/compound/75094922#section=2D-Structure
https://pubchem.ncbi.nlm.nih.gov/compound/75094583#section=2D-Structure
https://pubchem.ncbi.nlm.nih.gov/compound/75089628#section=2D-Structure
https://pubchem.ncbi.nlm.nih.gov/compound/75089346#section=2D-Structure
https://pubchem.ncbi.nlm.nih.gov/compound/75088738#section=2D-Structure

https://pubchem.ncbi.nlm.nih.gov/compound/75046875#section=2D-Structure
https://pubchem.ncbi.nlm.nih.gov/compound/74995#section=2D-Structure
https://pubchem.ncbi.nlm.nih.gov/compound/74938208#section=2D-Structure
https://pubchem.ncbi.nlm.nih.gov/compound/74936526#section=2D-Structure
https://pubchem.ncbi.nlm.nih.gov/compound/74936483#section=2D-Structure
https://pubchem.ncbi.nlm.nih.gov/compound/74819600#section=2D-Structure
https://pubchem.ncbi.nlm.nih.gov/compound/74819596#section=2D-Structure
https://pubchem.ncbi.nlm.nih.gov/compound/74659851#section=2D-Structure
https://pubchem.ncbi.nlm.nih.gov/compound/74603085#section=2D-Structure
https://pubchem.ncbi.nlm.nih.gov/compound/74389064#section=2D-Structure
https://pubchem.ncbi.nlm.nih.gov/compound/74377236#section=2D-Structure
https://pubchem.ncbi.nlm.nih.gov/compound/74259383#section=2D-Structure
https://pubchem.ncbi.nlm.nih.gov/compound/74050881#section=2D-Structure
https://pubchem.ncbi.nlm.nih.gov/compound/74009400#section=2D-Structure
https://pubchem.ncbi.nlm.nih.gov/compound/74006940#section=2D-Structure
https://pubchem.ncbi.nlm.nih.gov/compound/74006939#section=2D-Structure
https://pubchem.ncbi.nlm.nih.gov/compound/73994937#section=2D-Structure
https://pubchem.ncbi.nlm.nih.gov/compound/73994936#section=2D-Structure
https://pubchem.ncbi.nlm.nih.gov/compound/73994934#section=2D-Structure
https://pubchem.ncbi.nlm.nih.gov/compound/73994933#section=2D-Structure
https://pubchem.ncbi.nlm.nih.gov/compound/73994931#section=2D-Structure
https://pubchem.ncbi.nlm.nih.gov/compound/73989632#section=2D-Structure
https://pubchem.ncbi.nlm.nih.gov/compound/73951806#section=2D-Structure
https://pubchem.ncbi.nlm.nih.gov/compound/73949173#section=2D-Structure
https://pubchem.ncbi.nlm.nih.gov/compound/73893721#section=2D-Structure
https://pubchem.ncbi.nlm.nih.gov/compound/73882380#section=2D-Structure
https://pubchem.ncbi.nlm.nih.gov/compound/73882259#section=2D-Structure
https://pubchem.ncbi.nlm.nih.gov/compound/73866769#section=2D-Structure
https://pubchem.ncbi.nlm.nih.gov/compound/73853157#section=2D-Structure
https://pubchem.ncbi.nlm.nih.gov/compound/73828363#section=2D-Structure
https://pubchem.ncbi.nlm.nih.gov/compound/73812958#section=2D-Structure
https://pubchem.ncbi.nlm.nih.gov/compound/73795355#section=2D-Structure
https://pubchem.ncbi.nlm.nih.gov/compound/73794589#section=2D-Structure
https://pubchem.ncbi.nlm.nih.gov/compound/73737090#section=2D-Structure
https://pubchem.ncbi.nlm.nih.gov/compound/73729602#section=2D-Structure
https://pubchem.ncbi.nlm.nih.gov/compound/73711159#section=2D-Structure
https://pubchem.ncbi.nlm.nih.gov/compound/73697#section=2D-Structure
https://pubchem.ncbi.nlm.nih.gov/compound/73695216#section=2D-Structure
https://pubchem.ncbi.nlm.nih.gov/compound/73692739#section=2D-Structure
https://pubchem.ncbi.nlm.nih.gov/compound/73677013#section=2D-Structure
https://pubchem.ncbi.nlm.nih.gov/compound/73676748#section=2D-Structure
https://pubchem.ncbi.nlm.nih.gov/compound/73668288#section=2D-Structure
https://pubchem.ncbi.nlm.nih.gov/compound/736468#section=2D-Structure
https://pubchem.ncbi.nlm.nih.gov/compound/73629426#section=2D-Structure
https://pubchem.ncbi.nlm.nih.gov/compound/73474064#section=2D-Structure
https://pubchem.ncbi.nlm.nih.gov/compound/73388730#section=2D-Structure
https://pubchem.ncbi.nlm.nih.gov/compound/73297315#section=2D-Structure
https://pubchem.ncbi.nlm.nih.gov/compound/73189009#section=2D-Structure
https://pubchem.ncbi.nlm.nih.gov/compound/73188947#section=2D-Structure
https://pubchem.ncbi.nlm.nih.gov/compound/73188413#section=2D-Structure
https://pubchem.ncbi.nlm.nih.gov/compound/73183693#section=2D-Structure
https://pubchem.ncbi.nlm.nih.gov/compound/73176674#section=2D-Structure
https://pubchem.ncbi.nlm.nih.gov/compound/73071378#section=2D-Structure
https://pubchem.ncbi.nlm.nih.gov/compound/73045058#section=2D-Structure
https://pubchem.ncbi.nlm.nih.gov/compound/73037763#section=2D-Structure

https://pubchem.ncbi.nlm.nih.gov/compound/73025606#section=2D-Structure
https://pubchem.ncbi.nlm.nih.gov/compound/73013061#section=2D-Structure
https://pubchem.ncbi.nlm.nih.gov/compound/73012685#section=2D-Structure
https://pubchem.ncbi.nlm.nih.gov/compound/73011166#section=2D-Structure
https://pubchem.ncbi.nlm.nih.gov/compound/73011139#section=2D-Structure
https://pubchem.ncbi.nlm.nih.gov/compound/73011004#section=2D-Structure
https://pubchem.ncbi.nlm.nih.gov/compound/72998661#section=2D-Structure
https://pubchem.ncbi.nlm.nih.gov/compound/728298#section=2D-Structure
https://pubchem.ncbi.nlm.nih.gov/compound/72826#section=2D-Structure
https://pubchem.ncbi.nlm.nih.gov/compound/72810164#section=2D-Structure
https://pubchem.ncbi.nlm.nih.gov/compound/72744252#section=2D-Structure
https://pubchem.ncbi.nlm.nih.gov/compound/72733785#section=2D-Structure
https://pubchem.ncbi.nlm.nih.gov/compound/72725#section=2D-Structure
https://pubchem.ncbi.nlm.nih.gov/compound/72721#section=2D-Structure
https://pubchem.ncbi.nlm.nih.gov/compound/72698261#section=2D-Structure
https://pubchem.ncbi.nlm.nih.gov/compound/72659#section=2D-Structure
https://pubchem.ncbi.nlm.nih.gov/compound/72603500#section=2D-Structure
https://pubchem.ncbi.nlm.nih.gov/compound/72577554#section=2D-Structure
https://pubchem.ncbi.nlm.nih.gov/compound/72572184#section=2D-Structure
https://pubchem.ncbi.nlm.nih.gov/compound/72572182#section=2D-Structure
https://pubchem.ncbi.nlm.nih.gov/compound/72572181#section=2D-Structure
https://pubchem.ncbi.nlm.nih.gov/compound/72554674#section=2D-Structure
https://pubchem.ncbi.nlm.nih.gov/compound/72554662#section=2D-Structure
https://pubchem.ncbi.nlm.nih.gov/compound/72546932#section=2D-Structure
https://pubchem.ncbi.nlm.nih.gov/compound/72546931#section=2D-Structure
https://pubchem.ncbi.nlm.nih.gov/compound/72546930#section=2D-Structure
https://pubchem.ncbi.nlm.nih.gov/compound/72539488#section=2D-Structure
https://pubchem.ncbi.nlm.nih.gov/compound/72475894#section=2D-Structure
https://pubchem.ncbi.nlm.nih.gov/compound/72457088#section=2D-Structure
https://pubchem.ncbi.nlm.nih.gov/compound/72359283#section=2D-Structure
https://pubchem.ncbi.nlm.nih.gov/compound/72347585#section=2D-Structure
https://pubchem.ncbi.nlm.nih.gov/compound/72330401#section=2D-Structure
https://pubchem.ncbi.nlm.nih.gov/compound/72330395#section=2D-Structure
https://pubchem.ncbi.nlm.nih.gov/compound/723231#section=2D-Structure
https://pubchem.ncbi.nlm.nih.gov/compound/72286106#section=2D-Structure
https://pubchem.ncbi.nlm.nih.gov/compound/72279876#section=2D-Structure
https://pubchem.ncbi.nlm.nih.gov/compound/72279874#section=2D-Structure
https://pubchem.ncbi.nlm.nih.gov/compound/72279868#section=2D-Structure
https://pubchem.ncbi.nlm.nih.gov/compound/72255306#section=2D-Structure
https://pubchem.ncbi.nlm.nih.gov/compound/72239786#section=2D-Structure
https://pubchem.ncbi.nlm.nih.gov/compound/72239776#section=2D-Structure
https://pubchem.ncbi.nlm.nih.gov/compound/72213624#section=2D-Structure
https://pubchem.ncbi.nlm.nih.gov/compound/72213623#section=2D-Structure
https://pubchem.ncbi.nlm.nih.gov/compound/72212541#section=2D-Structure
https://pubchem.ncbi.nlm.nih.gov/compound/72208321#section=2D-Structure
https://pubchem.ncbi.nlm.nih.gov/compound/72207653#section=2D-Structure
https://pubchem.ncbi.nlm.nih.gov/compound/72206613#section=2D-Structure
https://pubchem.ncbi.nlm.nih.gov/compound/722057#section=2D-Structure
https://pubchem.ncbi.nlm.nih.gov/compound/72073#section=2D-Structure
https://pubchem.ncbi.nlm.nih.gov/compound/718874#section=2D-Structure
https://pubchem.ncbi.nlm.nih.gov/compound/71815096#section=2D-Structure
https://pubchem.ncbi.nlm.nih.gov/compound/71774041#section=2D-Structure
https://pubchem.ncbi.nlm.nih.gov/compound/71773273#section=2D-Structure
https://pubchem.ncbi.nlm.nih.gov/compound/71773259#section=2D-Structure
https://pubchem.ncbi.nlm.nih.gov/compound/71772882#section=2D-Structure

https://pubchem.ncbi.nlm.nih.gov/compound/71738033#section=2D-Structure
https://pubchem.ncbi.nlm.nih.gov/compound/71718015#section=2D-Structure
https://pubchem.ncbi.nlm.nih.gov/compound/71676672#section=2D-Structure
https://pubchem.ncbi.nlm.nih.gov/compound/71668504#section=2D-Structure
https://pubchem.ncbi.nlm.nih.gov/compound/71668469#section=2D-Structure
https://pubchem.ncbi.nlm.nih.gov/compound/71650944#section=2D-Structure
https://pubchem.ncbi.nlm.nih.gov/compound/71650484#section=2D-Structure
https://pubchem.ncbi.nlm.nih.gov/compound/71650475#section=2D-Structure
https://pubchem.ncbi.nlm.nih.gov/compound/71650474#section=2D-Structure
https://pubchem.ncbi.nlm.nih.gov/compound/71650473#section=2D-Structure
https://pubchem.ncbi.nlm.nih.gov/compound/71650341#section=2D-Structure
https://pubchem.ncbi.nlm.nih.gov/compound/71625520#section=2D-Structure
https://pubchem.ncbi.nlm.nih.gov/compound/71625411#section=2D-Structure
https://pubchem.ncbi.nlm.nih.gov/compound/71625410#section=2D-Structure
https://pubchem.ncbi.nlm.nih.gov/compound/71593719#section=2D-Structure
https://pubchem.ncbi.nlm.nih.gov/compound/71593718#section=2D-Structure
https://pubchem.ncbi.nlm.nih.gov/compound/71593717#section=2D-Structure
https://pubchem.ncbi.nlm.nih.gov/compound/71537710#section=2D-Structure
https://pubchem.ncbi.nlm.nih.gov/compound/71514438#section=2D-Structure
https://pubchem.ncbi.nlm.nih.gov/compound/71514437#section=2D-Structure
https://pubchem.ncbi.nlm.nih.gov/compound/71514436#section=2D-Structure
https://pubchem.ncbi.nlm.nih.gov/compound/71514435#section=2D-Structure
https://pubchem.ncbi.nlm.nih.gov/compound/71505191#section=2D-Structure
https://pubchem.ncbi.nlm.nih.gov/compound/71464309#section=2D-Structure
https://pubchem.ncbi.nlm.nih.gov/compound/71460480#section=2D-Structure
https://pubchem.ncbi.nlm.nih.gov/compound/71456795#section=2D-Structure
https://pubchem.ncbi.nlm.nih.gov/compound/71442657#section=2D-Structure
https://pubchem.ncbi.nlm.nih.gov/compound/71441753#section=2D-Structure
https://pubchem.ncbi.nlm.nih.gov/compound/71439852#section=2D-Structure
https://pubchem.ncbi.nlm.nih.gov/compound/71438418#section=2D-Structure
https://pubchem.ncbi.nlm.nih.gov/compound/71437981#section=2D-Structure
https://pubchem.ncbi.nlm.nih.gov/compound/71437852#section=2D-Structure
https://pubchem.ncbi.nlm.nih.gov/compound/71437657#section=2D-Structure
https://pubchem.ncbi.nlm.nih.gov/compound/71437512#section=2D-Structure
https://pubchem.ncbi.nlm.nih.gov/compound/71436849#section=2D-Structure
https://pubchem.ncbi.nlm.nih.gov/compound/71436214#section=2D-Structure
https://pubchem.ncbi.nlm.nih.gov/compound/71435622#section=2D-Structure
https://pubchem.ncbi.nlm.nih.gov/compound/71435538#section=2D-Structure
https://pubchem.ncbi.nlm.nih.gov/compound/71430755#section=2D-Structure
https://pubchem.ncbi.nlm.nih.gov/compound/71426862#section=2D-Structure
https://pubchem.ncbi.nlm.nih.gov/compound/71426856#section=2D-Structure
https://pubchem.ncbi.nlm.nih.gov/compound/71426531#section=2D-Structure
https://pubchem.ncbi.nlm.nih.gov/compound/71422231#section=2D-Structure
https://pubchem.ncbi.nlm.nih.gov/compound/71419631#section=2D-Structure
https://pubchem.ncbi.nlm.nih.gov/compound/71419603#section=2D-Structure
https://pubchem.ncbi.nlm.nih.gov/compound/71415764#section=2D-Structure
https://pubchem.ncbi.nlm.nih.gov/compound/71414499#section=2D-Structure
https://pubchem.ncbi.nlm.nih.gov/compound/71408555#section=2D-Structure
https://pubchem.ncbi.nlm.nih.gov/compound/71407521#section=2D-Structure
https://pubchem.ncbi.nlm.nih.gov/compound/71406360#section=2D-Structure
https://pubchem.ncbi.nlm.nih.gov/compound/71404880#section=2D-Structure
https://pubchem.ncbi.nlm.nih.gov/compound/71403973#section=2D-Structure
https://pubchem.ncbi.nlm.nih.gov/compound/71401706#section=2D-Structure
https://pubchem.ncbi.nlm.nih.gov/compound/71401547#section=2D-Structure
https://pubchem.ncbi.nlm.nih.gov/compound/71400288#section=2D-Structure

https://pubchem.ncbi.nlm.nih.gov/compound/71396591#section=2D-Structure
https://pubchem.ncbi.nlm.nih.gov/compound/71394014#section=2D-Structure
https://pubchem.ncbi.nlm.nih.gov/compound/71392460#section=2D-Structure
https://pubchem.ncbi.nlm.nih.gov/compound/71385928#section=2D-Structure
https://pubchem.ncbi.nlm.nih.gov/compound/71385021#section=2D-Structure
https://pubchem.ncbi.nlm.nih.gov/compound/71377828#section=2D-Structure
https://pubchem.ncbi.nlm.nih.gov/compound/71373365#section=2D-Structure
https://pubchem.ncbi.nlm.nih.gov/compound/71373363#section=2D-Structure
https://pubchem.ncbi.nlm.nih.gov/compound/71368272#section=2D-Structure
https://pubchem.ncbi.nlm.nih.gov/compound/71362430#section=2D-Structure
https://pubchem.ncbi.nlm.nih.gov/compound/71361141#section=2D-Structure
https://pubchem.ncbi.nlm.nih.gov/compound/71359515#section=2D-Structure
https://pubchem.ncbi.nlm.nih.gov/compound/71359015#section=2D-Structure
https://pubchem.ncbi.nlm.nih.gov/compound/71359014#section=2D-Structure
https://pubchem.ncbi.nlm.nih.gov/compound/71358975#section=2D-Structure
https://pubchem.ncbi.nlm.nih.gov/compound/71358967#section=2D-Structure
https://pubchem.ncbi.nlm.nih.gov/compound/71358939#section=2D-Structure
https://pubchem.ncbi.nlm.nih.gov/compound/71358938#section=2D-Structure
https://pubchem.ncbi.nlm.nih.gov/compound/71358905#section=2D-Structure
https://pubchem.ncbi.nlm.nih.gov/compound/71358855#section=2D-Structure
https://pubchem.ncbi.nlm.nih.gov/compound/71358835#section=2D-Structure
https://pubchem.ncbi.nlm.nih.gov/compound/71358692#section=2D-Structure
https://pubchem.ncbi.nlm.nih.gov/compound/71356358#section=2D-Structure
https://pubchem.ncbi.nlm.nih.gov/compound/71356321#section=2D-Structure
https://pubchem.ncbi.nlm.nih.gov/compound/71356022#section=2D-Structure
https://pubchem.ncbi.nlm.nih.gov/compound/71356000#section=2D-Structure
https://pubchem.ncbi.nlm.nih.gov/compound/71354290#section=2D-Structure
https://pubchem.ncbi.nlm.nih.gov/compound/71353446#section=2D-Structure
https://pubchem.ncbi.nlm.nih.gov/compound/71351299#section=2D-Structure
https://pubchem.ncbi.nlm.nih.gov/compound/71346896#section=2D-Structure
https://pubchem.ncbi.nlm.nih.gov/compound/71346883#section=2D-Structure
https://pubchem.ncbi.nlm.nih.gov/compound/71346189#section=2D-Structure
https://pubchem.ncbi.nlm.nih.gov/compound/71345955#section=2D-Structure
https://pubchem.ncbi.nlm.nih.gov/compound/71344755#section=2D-Structure
https://pubchem.ncbi.nlm.nih.gov/compound/71341492#section=2D-Structure
https://pubchem.ncbi.nlm.nih.gov/compound/71341269#section=2D-Structure
https://pubchem.ncbi.nlm.nih.gov/compound/71338922#section=2D-Structure
https://pubchem.ncbi.nlm.nih.gov/compound/71338441#section=2D-Structure
https://pubchem.ncbi.nlm.nih.gov/compound/71337555#section=2D-Structure
https://pubchem.ncbi.nlm.nih.gov/compound/71335973#section=2D-Structure
https://pubchem.ncbi.nlm.nih.gov/compound/71335529#section=2D-Structure
https://pubchem.ncbi.nlm.nih.gov/compound/71334390#section=2D-Structure
https://pubchem.ncbi.nlm.nih.gov/compound/71333142#section=2D-Structure
https://pubchem.ncbi.nlm.nih.gov/compound/71329490#section=2D-Structure
https://pubchem.ncbi.nlm.nih.gov/compound/71329161#section=2D-Structure
https://pubchem.ncbi.nlm.nih.gov/compound/71328827#section=2D-Structure
https://pubchem.ncbi.nlm.nih.gov/compound/71327638#section=2D-Structure
https://pubchem.ncbi.nlm.nih.gov/compound/71327274#section=2D-Structure
https://pubchem.ncbi.nlm.nih.gov/compound/71326204#section=2D-Structure
https://pubchem.ncbi.nlm.nih.gov/compound/71326071#section=2D-Structure
https://pubchem.ncbi.nlm.nih.gov/compound/71324903#section=2D-Structure
https://pubchem.ncbi.nlm.nih.gov/compound/71322261#section=2D-Structure
https://pubchem.ncbi.nlm.nih.gov/compound/71322259#section=2D-Structure
https://pubchem.ncbi.nlm.nih.gov/compound/71322257#section=2D-Structure
https://pubchem.ncbi.nlm.nih.gov/compound/71304585#section=2D-Structure

https://pubchem.ncbi.nlm.nih.gov/compound/71304104#section=2D-Structure
https://pubchem.ncbi.nlm.nih.gov/compound/71304097#section=2D-Structure
https://pubchem.ncbi.nlm.nih.gov/compound/71304069#section=2D-Structure
https://pubchem.ncbi.nlm.nih.gov/compound/71304041#section=2D-Structure
https://pubchem.ncbi.nlm.nih.gov/compound/71303986#section=2D-Structure
https://pubchem.ncbi.nlm.nih.gov/compound/71303969#section=2D-Structure
https://pubchem.ncbi.nlm.nih.gov/compound/71303889#section=2D-Structure
https://pubchem.ncbi.nlm.nih.gov/compound/71303754#section=2D-Structure
https://pubchem.ncbi.nlm.nih.gov/compound/71303010#section=2D-Structure
https://pubchem.ncbi.nlm.nih.gov/compound/71302972#section=2D-Structure
https://pubchem.ncbi.nlm.nih.gov/compound/71302883#section=2D-Structure
https://pubchem.ncbi.nlm.nih.gov/compound/71302596#section=2D-Structure
https://pubchem.ncbi.nlm.nih.gov/compound/71302434#section=2D-Structure
https://pubchem.ncbi.nlm.nih.gov/compound/71301387#section=2D-Structure
https://pubchem.ncbi.nlm.nih.gov/compound/71272752#section=2D-Structure
https://pubchem.ncbi.nlm.nih.gov/compound/71261435#section=2D-Structure
https://pubchem.ncbi.nlm.nih.gov/compound/71261416#section=2D-Structure
https://pubchem.ncbi.nlm.nih.gov/compound/71248877#section=2D-Structure
https://pubchem.ncbi.nlm.nih.gov/compound/71242158#section=2D-Structure
https://pubchem.ncbi.nlm.nih.gov/compound/71242154#section=2D-Structure
https://pubchem.ncbi.nlm.nih.gov/compound/71242151#section=2D-Structure
https://pubchem.ncbi.nlm.nih.gov/compound/71242148#section=2D-Structure
https://pubchem.ncbi.nlm.nih.gov/compound/71234797#section=2D-Structure
https://pubchem.ncbi.nlm.nih.gov/compound/71222252#section=2D-Structure
https://pubchem.ncbi.nlm.nih.gov/compound/71209831#section=2D-Structure
https://pubchem.ncbi.nlm.nih.gov/compound/71147947#section=2D-Structure
https://pubchem.ncbi.nlm.nih.gov/compound/71128505#section=2D-Structure
https://pubchem.ncbi.nlm.nih.gov/compound/71128504#section=2D-Structure
https://pubchem.ncbi.nlm.nih.gov/compound/71128503#section=2D-Structure
https://pubchem.ncbi.nlm.nih.gov/compound/71128501#section=2D-Structure
https://pubchem.ncbi.nlm.nih.gov/compound/71128500#section=2D-Structure
https://pubchem.ncbi.nlm.nih.gov/compound/71079559#section=2D-Structure
https://pubchem.ncbi.nlm.nih.gov/compound/71062471#section=2D-Structure
https://pubchem.ncbi.nlm.nih.gov/compound/71062404#section=2D-Structure
https://pubchem.ncbi.nlm.nih.gov/compound/71058496#section=2D-Structure
https://pubchem.ncbi.nlm.nih.gov/compound/71054079#section=2D-Structure
https://pubchem.ncbi.nlm.nih.gov/compound/71052462#section=2D-Structure
https://pubchem.ncbi.nlm.nih.gov/compound/7099159#section=2D-Structure
https://pubchem.ncbi.nlm.nih.gov/compound/70984919#section=2D-Structure
https://pubchem.ncbi.nlm.nih.gov/compound/70979917#section=2D-Structure
https://pubchem.ncbi.nlm.nih.gov/compound/70935064#section=2D-Structure
https://pubchem.ncbi.nlm.nih.gov/compound/70908349#section=2D-Structure
https://pubchem.ncbi.nlm.nih.gov/compound/70863329#section=2D-Structure
https://pubchem.ncbi.nlm.nih.gov/compound/70863251#section=2D-Structure
https://pubchem.ncbi.nlm.nih.gov/compound/70823064#section=2D-Structure
https://pubchem.ncbi.nlm.nih.gov/compound/70809517#section=2D-Structure
https://pubchem.ncbi.nlm.nih.gov/compound/70806158#section=2D-Structure
https://pubchem.ncbi.nlm.nih.gov/compound/70700716#section=2D-Structure
https://pubchem.ncbi.nlm.nih.gov/compound/70658735#section=2D-Structure
https://pubchem.ncbi.nlm.nih.gov/compound/70651783#section=2D-Structure
https://pubchem.ncbi.nlm.nih.gov/compound/70651590#section=2D-Structure
https://pubchem.ncbi.nlm.nih.gov/compound/70629609#section=2D-Structure
https://pubchem.ncbi.nlm.nih.gov/compound/70629381#section=2D-Structure
https://pubchem.ncbi.nlm.nih.gov/compound/70629358#section=2D-Structure
https://pubchem.ncbi.nlm.nih.gov/compound/70628010#section=2D-Structure

https://pubchem.ncbi.nlm.nih.gov/compound/70623457#section=2D-Structure
https://pubchem.ncbi.nlm.nih.gov/compound/70614081#section=2D-Structure
https://pubchem.ncbi.nlm.nih.gov/compound/70612307#section=2D-Structure
https://pubchem.ncbi.nlm.nih.gov/compound/70605745#section=2D-Structure
https://pubchem.ncbi.nlm.nih.gov/compound/70598908#section=2D-Structure
https://pubchem.ncbi.nlm.nih.gov/compound/70595849#section=2D-Structure
https://pubchem.ncbi.nlm.nih.gov/compound/70586183#section=2D-Structure
https://pubchem.ncbi.nlm.nih.gov/compound/70583134#section=2D-Structure
https://pubchem.ncbi.nlm.nih.gov/compound/70580685#section=2D-Structure
https://pubchem.ncbi.nlm.nih.gov/compound/70579873#section=2D-Structure
https://pubchem.ncbi.nlm.nih.gov/compound/70578501#section=2D-Structure
https://pubchem.ncbi.nlm.nih.gov/compound/70575047#section=2D-Structure
https://pubchem.ncbi.nlm.nih.gov/compound/70569848#section=2D-Structure
https://pubchem.ncbi.nlm.nih.gov/compound/70568263#section=2D-Structure
https://pubchem.ncbi.nlm.nih.gov/compound/70566891#section=2D-Structure
https://pubchem.ncbi.nlm.nih.gov/compound/70564748#section=2D-Structure
https://pubchem.ncbi.nlm.nih.gov/compound/70564451#section=2D-Structure
https://pubchem.ncbi.nlm.nih.gov/compound/70560872#section=2D-Structure
https://pubchem.ncbi.nlm.nih.gov/compound/70554162#section=2D-Structure
https://pubchem.ncbi.nlm.nih.gov/compound/70551684#section=2D-Structure
https://pubchem.ncbi.nlm.nih.gov/compound/70549247#section=2D-Structure
https://pubchem.ncbi.nlm.nih.gov/compound/70546197#section=2D-Structure
https://pubchem.ncbi.nlm.nih.gov/compound/70545274#section=2D-Structure
https://pubchem.ncbi.nlm.nih.gov/compound/70538061#section=2D-Structure
https://pubchem.ncbi.nlm.nih.gov/compound/70537573#section=2D-Structure
https://pubchem.ncbi.nlm.nih.gov/compound/70537485#section=2D-Structure
https://pubchem.ncbi.nlm.nih.gov/compound/70534190#section=2D-Structure
https://pubchem.ncbi.nlm.nih.gov/compound/70531182#section=2D-Structure
https://pubchem.ncbi.nlm.nih.gov/compound/70530258#section=2D-Structure
https://pubchem.ncbi.nlm.nih.gov/compound/70528379#section=2D-Structure
https://pubchem.ncbi.nlm.nih.gov/compound/70527947#section=2D-Structure
https://pubchem.ncbi.nlm.nih.gov/compound/70517627#section=2D-Structure
https://pubchem.ncbi.nlm.nih.gov/compound/70515381#section=2D-Structure
https://pubchem.ncbi.nlm.nih.gov/compound/70511422#section=2D-Structure
https://pubchem.ncbi.nlm.nih.gov/compound/70511016#section=2D-Structure
https://pubchem.ncbi.nlm.nih.gov/compound/70509038#section=2D-Structure
https://pubchem.ncbi.nlm.nih.gov/compound/70507279#section=2D-Structure
https://pubchem.ncbi.nlm.nih.gov/compound/70506444#section=2D-Structure
https://pubchem.ncbi.nlm.nih.gov/compound/70501612#section=2D-Structure
https://pubchem.ncbi.nlm.nih.gov/compound/70500217#section=2D-Structure
https://pubchem.ncbi.nlm.nih.gov/compound/70499014#section=2D-Structure
https://pubchem.ncbi.nlm.nih.gov/compound/70498590#section=2D-Structure
https://pubchem.ncbi.nlm.nih.gov/compound/70495708#section=2D-Structure
https://pubchem.ncbi.nlm.nih.gov/compound/70491256#section=2D-Structure
https://pubchem.ncbi.nlm.nih.gov/compound/70485587#section=2D-Structure
https://pubchem.ncbi.nlm.nih.gov/compound/70483374#section=2D-Structure
https://pubchem.ncbi.nlm.nih.gov/compound/70483347#section=2D-Structure
https://pubchem.ncbi.nlm.nih.gov/compound/70483043#section=2D-Structure
https://pubchem.ncbi.nlm.nih.gov/compound/70482694#section=2D-Structure
https://pubchem.ncbi.nlm.nih.gov/compound/70481440#section=2D-Structure
https://pubchem.ncbi.nlm.nih.gov/compound/70478181#section=2D-Structure
https://pubchem.ncbi.nlm.nih.gov/compound/70476536#section=2D-Structure
https://pubchem.ncbi.nlm.nih.gov/compound/70475090#section=2D-Structure
https://pubchem.ncbi.nlm.nih.gov/compound/70472420#section=2D-Structure
https://pubchem.ncbi.nlm.nih.gov/compound/70471851#section=2D-Structure

https://pubchem.ncbi.nlm.nih.gov/compound/70470144#section=2D-Structure
https://pubchem.ncbi.nlm.nih.gov/compound/70468654#section=2D-Structure
https://pubchem.ncbi.nlm.nih.gov/compound/70468548#section=2D-Structure
https://pubchem.ncbi.nlm.nih.gov/compound/70468245#section=2D-Structure
https://pubchem.ncbi.nlm.nih.gov/compound/70467284#section=2D-Structure
https://pubchem.ncbi.nlm.nih.gov/compound/70465637#section=2D-Structure
https://pubchem.ncbi.nlm.nih.gov/compound/70460375#section=2D-Structure
https://pubchem.ncbi.nlm.nih.gov/compound/70460261#section=2D-Structure
https://pubchem.ncbi.nlm.nih.gov/compound/70450074#section=2D-Structure
https://pubchem.ncbi.nlm.nih.gov/compound/70449288#section=2D-Structure
https://pubchem.ncbi.nlm.nih.gov/compound/70448098#section=2D-Structure
https://pubchem.ncbi.nlm.nih.gov/compound/70447304#section=2D-Structure
https://pubchem.ncbi.nlm.nih.gov/compound/70447236#section=2D-Structure
https://pubchem.ncbi.nlm.nih.gov/compound/70443488#section=2D-Structure
https://pubchem.ncbi.nlm.nih.gov/compound/70441740#section=2D-Structure
https://pubchem.ncbi.nlm.nih.gov/compound/70439420#section=2D-Structure
https://pubchem.ncbi.nlm.nih.gov/compound/70432655#section=2D-Structure
https://pubchem.ncbi.nlm.nih.gov/compound/70429693#section=2D-Structure
https://pubchem.ncbi.nlm.nih.gov/compound/70427528#section=2D-Structure
https://pubchem.ncbi.nlm.nih.gov/compound/70427079#section=2D-Structure
https://pubchem.ncbi.nlm.nih.gov/compound/70426348#section=2D-Structure
https://pubchem.ncbi.nlm.nih.gov/compound/70425188#section=2D-Structure
https://pubchem.ncbi.nlm.nih.gov/compound/70421288#section=2D-Structure
https://pubchem.ncbi.nlm.nih.gov/compound/70420569#section=2D-Structure
https://pubchem.ncbi.nlm.nih.gov/compound/70420125#section=2D-Structure
https://pubchem.ncbi.nlm.nih.gov/compound/70416786#section=2D-Structure
https://pubchem.ncbi.nlm.nih.gov/compound/70414762#section=2D-Structure
https://pubchem.ncbi.nlm.nih.gov/compound/70414056#section=2D-Structure
https://pubchem.ncbi.nlm.nih.gov/compound/70414053#section=2D-Structure
https://pubchem.ncbi.nlm.nih.gov/compound/70413588#section=2D-Structure
https://pubchem.ncbi.nlm.nih.gov/compound/70410425#section=2D-Structure
https://pubchem.ncbi.nlm.nih.gov/compound/70409780#section=2D-Structure
https://pubchem.ncbi.nlm.nih.gov/compound/70409407#section=2D-Structure
https://pubchem.ncbi.nlm.nih.gov/compound/70409264#section=2D-Structure
https://pubchem.ncbi.nlm.nih.gov/compound/70403934#section=2D-Structure
https://pubchem.ncbi.nlm.nih.gov/compound/70400810#section=2D-Structure
https://pubchem.ncbi.nlm.nih.gov/compound/70398225#section=2D-Structure
https://pubchem.ncbi.nlm.nih.gov/compound/70395181#section=2D-Structure
https://pubchem.ncbi.nlm.nih.gov/compound/70392198#section=2D-Structure
https://pubchem.ncbi.nlm.nih.gov/compound/70379135#section=2D-Structure
https://pubchem.ncbi.nlm.nih.gov/compound/70377937#section=2D-Structure
https://pubchem.ncbi.nlm.nih.gov/compound/70377936#section=2D-Structure
https://pubchem.ncbi.nlm.nih.gov/compound/70377807#section=2D-Structure
https://pubchem.ncbi.nlm.nih.gov/compound/70374532#section=2D-Structure
https://pubchem.ncbi.nlm.nih.gov/compound/70374137#section=2D-Structure
https://pubchem.ncbi.nlm.nih.gov/compound/70374116#section=2D-Structure
https://pubchem.ncbi.nlm.nih.gov/compound/70372666#section=2D-Structure
https://pubchem.ncbi.nlm.nih.gov/compound/70369513#section=2D-Structure
https://pubchem.ncbi.nlm.nih.gov/compound/70367355#section=2D-Structure
https://pubchem.ncbi.nlm.nih.gov/compound/70367354#section=2D-Structure
https://pubchem.ncbi.nlm.nih.gov/compound/70366959#section=2D-Structure
https://pubchem.ncbi.nlm.nih.gov/compound/70364051#section=2D-Structure
https://pubchem.ncbi.nlm.nih.gov/compound/70362881#section=2D-Structure
https://pubchem.ncbi.nlm.nih.gov/compound/70362569#section=2D-Structure
https://pubchem.ncbi.nlm.nih.gov/compound/70362568#section=2D-Structure

https://pubchem.ncbi.nlm.nih.gov/compound/70361217#section=2D-Structure
https://pubchem.ncbi.nlm.nih.gov/compound/70358313#section=2D-Structure
https://pubchem.ncbi.nlm.nih.gov/compound/70357429#section=2D-Structure
https://pubchem.ncbi.nlm.nih.gov/compound/70356227#section=2D-Structure
https://pubchem.ncbi.nlm.nih.gov/compound/70353867#section=2D-Structure
https://pubchem.ncbi.nlm.nih.gov/compound/70353622#section=2D-Structure
https://pubchem.ncbi.nlm.nih.gov/compound/70352915#section=2D-Structure
https://pubchem.ncbi.nlm.nih.gov/compound/70352787#section=2D-Structure
https://pubchem.ncbi.nlm.nih.gov/compound/70348693#section=2D-Structure
https://pubchem.ncbi.nlm.nih.gov/compound/70346083#section=2D-Structure
https://pubchem.ncbi.nlm.nih.gov/compound/70336425#section=2D-Structure
https://pubchem.ncbi.nlm.nih.gov/compound/70323249#section=2D-Structure
https://pubchem.ncbi.nlm.nih.gov/compound/70320931#section=2D-Structure
https://pubchem.ncbi.nlm.nih.gov/compound/70316666#section=2D-Structure
https://pubchem.ncbi.nlm.nih.gov/compound/70305294#section=2D-Structure
https://pubchem.ncbi.nlm.nih.gov/compound/70304286#section=2D-Structure
https://pubchem.ncbi.nlm.nih.gov/compound/70303232#section=2D-Structure
https://pubchem.ncbi.nlm.nih.gov/compound/70302279#section=2D-Structure
https://pubchem.ncbi.nlm.nih.gov/compound/70292459#section=2D-Structure
https://pubchem.ncbi.nlm.nih.gov/compound/70291399#section=2D-Structure
https://pubchem.ncbi.nlm.nih.gov/compound/70290495#section=2D-Structure
https://pubchem.ncbi.nlm.nih.gov/compound/70290201#section=2D-Structure
https://pubchem.ncbi.nlm.nih.gov/compound/70282250#section=2D-Structure
https://pubchem.ncbi.nlm.nih.gov/compound/70281031#section=2D-Structure
https://pubchem.ncbi.nlm.nih.gov/compound/70280664#section=2D-Structure
https://pubchem.ncbi.nlm.nih.gov/compound/70280169#section=2D-Structure
https://pubchem.ncbi.nlm.nih.gov/compound/70272819#section=2D-Structure
https://pubchem.ncbi.nlm.nih.gov/compound/70263916#section=2D-Structure
https://pubchem.ncbi.nlm.nih.gov/compound/70261790#section=2D-Structure
https://pubchem.ncbi.nlm.nih.gov/compound/70257494#section=2D-Structure
https://pubchem.ncbi.nlm.nih.gov/compound/70252106#section=2D-Structure
https://pubchem.ncbi.nlm.nih.gov/compound/70245507#section=2D-Structure
https://pubchem.ncbi.nlm.nih.gov/compound/70233652#section=2D-Structure
https://pubchem.ncbi.nlm.nih.gov/compound/70233593#section=2D-Structure
https://pubchem.ncbi.nlm.nih.gov/compound/70216417#section=2D-Structure
https://pubchem.ncbi.nlm.nih.gov/compound/7021466#section=2D-Structure
https://pubchem.ncbi.nlm.nih.gov/compound/70212872#section=2D-Structure
https://pubchem.ncbi.nlm.nih.gov/compound/70206088#section=2D-Structure
https://pubchem.ncbi.nlm.nih.gov/compound/70203070#section=2D-Structure
https://pubchem.ncbi.nlm.nih.gov/compound/70200377#section=2D-Structure
https://pubchem.ncbi.nlm.nih.gov/compound/70196219#section=2D-Structure
https://pubchem.ncbi.nlm.nih.gov/compound/70189617#section=2D-Structure
https://pubchem.ncbi.nlm.nih.gov/compound/70182365#section=2D-Structure
https://pubchem.ncbi.nlm.nih.gov/compound/70171497#section=2D-Structure
https://pubchem.ncbi.nlm.nih.gov/compound/70155153#section=2D-Structure
https://pubchem.ncbi.nlm.nih.gov/compound/70153180#section=2D-Structure
https://pubchem.ncbi.nlm.nih.gov/compound/70151630#section=2D-Structure
https://pubchem.ncbi.nlm.nih.gov/compound/70138831#section=2D-Structure
https://pubchem.ncbi.nlm.nih.gov/compound/70133005#section=2D-Structure
https://pubchem.ncbi.nlm.nih.gov/compound/70132305#section=2D-Structure
https://pubchem.ncbi.nlm.nih.gov/compound/70123457#section=2D-Structure
https://pubchem.ncbi.nlm.nih.gov/compound/70122398#section=2D-Structure
https://pubchem.ncbi.nlm.nih.gov/compound/70120595#section=2D-Structure
https://pubchem.ncbi.nlm.nih.gov/compound/70114824#section=2D-Structure
https://pubchem.ncbi.nlm.nih.gov/compound/70111652#section=2D-Structure

https://pubchem.ncbi.nlm.nih.gov/compound/70105236#section=2D-Structure
https://pubchem.ncbi.nlm.nih.gov/compound/70104958#section=2D-Structure
https://pubchem.ncbi.nlm.nih.gov/compound/70104575#section=2D-Structure
https://pubchem.ncbi.nlm.nih.gov/compound/70102714#section=2D-Structure
https://pubchem.ncbi.nlm.nih.gov/compound/70093312#section=2D-Structure
https://pubchem.ncbi.nlm.nih.gov/compound/70090571#section=2D-Structure
https://pubchem.ncbi.nlm.nih.gov/compound/70089549#section=2D-Structure
https://pubchem.ncbi.nlm.nih.gov/compound/70087110#section=2D-Structure
https://pubchem.ncbi.nlm.nih.gov/compound/70084835#section=2D-Structure
https://pubchem.ncbi.nlm.nih.gov/compound/70082747#section=2D-Structure
https://pubchem.ncbi.nlm.nih.gov/compound/70082239#section=2D-Structure
https://pubchem.ncbi.nlm.nih.gov/compound/70068199#section=2D-Structure
https://pubchem.ncbi.nlm.nih.gov/compound/70066652#section=2D-Structure
https://pubchem.ncbi.nlm.nih.gov/compound/70058286#section=2D-Structure
https://pubchem.ncbi.nlm.nih.gov/compound/70043787#section=2D-Structure
https://pubchem.ncbi.nlm.nih.gov/compound/70043227#section=2D-Structure
https://pubchem.ncbi.nlm.nih.gov/compound/70024078#section=2D-Structure
https://pubchem.ncbi.nlm.nih.gov/compound/70021711#section=2D-Structure
https://pubchem.ncbi.nlm.nih.gov/compound/70020260#section=2D-Structure
https://pubchem.ncbi.nlm.nih.gov/compound/70008451#section=2D-Structure
https://pubchem.ncbi.nlm.nih.gov/compound/70002842#section=2D-Structure
https://pubchem.ncbi.nlm.nih.gov/compound/70001591#section=2D-Structure
https://pubchem.ncbi.nlm.nih.gov/compound/70000478#section=2D-Structure
https://pubchem.ncbi.nlm.nih.gov/compound/69998077#section=2D-Structure
https://pubchem.ncbi.nlm.nih.gov/compound/69997470#section=2D-Structure
https://pubchem.ncbi.nlm.nih.gov/compound/69995517#section=2D-Structure
https://pubchem.ncbi.nlm.nih.gov/compound/69991802#section=2D-Structure
https://pubchem.ncbi.nlm.nih.gov/compound/69989685#section=2D-Structure
https://pubchem.ncbi.nlm.nih.gov/compound/69989680#section=2D-Structure
https://pubchem.ncbi.nlm.nih.gov/compound/69987734#section=2D-Structure
https://pubchem.ncbi.nlm.nih.gov/compound/69970981#section=2D-Structure
https://pubchem.ncbi.nlm.nih.gov/compound/69970359#section=2D-Structure
https://pubchem.ncbi.nlm.nih.gov/compound/69967956#section=2D-Structure
https://pubchem.ncbi.nlm.nih.gov/compound/69962115#section=2D-Structure
https://pubchem.ncbi.nlm.nih.gov/compound/69961591#section=2D-Structure
https://pubchem.ncbi.nlm.nih.gov/compound/69956507#section=2D-Structure
https://pubchem.ncbi.nlm.nih.gov/compound/69941487#section=2D-Structure
https://pubchem.ncbi.nlm.nih.gov/compound/69933456#section=2D-Structure
https://pubchem.ncbi.nlm.nih.gov/compound/69932826#section=2D-Structure
https://pubchem.ncbi.nlm.nih.gov/compound/69929926#section=2D-Structure
https://pubchem.ncbi.nlm.nih.gov/compound/69929574#section=2D-Structure
https://pubchem.ncbi.nlm.nih.gov/compound/69922448#section=2D-Structure
https://pubchem.ncbi.nlm.nih.gov/compound/69915710#section=2D-Structure
https://pubchem.ncbi.nlm.nih.gov/compound/69909060#section=2D-Structure
https://pubchem.ncbi.nlm.nih.gov/compound/69908459#section=2D-Structure
https://pubchem.ncbi.nlm.nih.gov/compound/69907701#section=2D-Structure
https://pubchem.ncbi.nlm.nih.gov/compound/69901747#section=2D-Structure
https://pubchem.ncbi.nlm.nih.gov/compound/69891811#section=2D-Structure
https://pubchem.ncbi.nlm.nih.gov/compound/69891026#section=2D-Structure
https://pubchem.ncbi.nlm.nih.gov/compound/69885757#section=2D-Structure
https://pubchem.ncbi.nlm.nih.gov/compound/69879810#section=2D-Structure
https://pubchem.ncbi.nlm.nih.gov/compound/69875754#section=2D-Structure
https://pubchem.ncbi.nlm.nih.gov/compound/69873950#section=2D-Structure
https://pubchem.ncbi.nlm.nih.gov/compound/69868391#section=2D-Structure
https://pubchem.ncbi.nlm.nih.gov/compound/69854729#section=2D-Structure

https://pubchem.ncbi.nlm.nih.gov/compound/69853735#section=2D-Structure
https://pubchem.ncbi.nlm.nih.gov/compound/69852121#section=2D-Structure
https://pubchem.ncbi.nlm.nih.gov/compound/69850826#section=2D-Structure
https://pubchem.ncbi.nlm.nih.gov/compound/69843539#section=2D-Structure
https://pubchem.ncbi.nlm.nih.gov/compound/69841493#section=2D-Structure
https://pubchem.ncbi.nlm.nih.gov/compound/69840782#section=2D-Structure
https://pubchem.ncbi.nlm.nih.gov/compound/69828591#section=2D-Structure
https://pubchem.ncbi.nlm.nih.gov/compound/69820890#section=2D-Structure
https://pubchem.ncbi.nlm.nih.gov/compound/69797804#section=2D-Structure
https://pubchem.ncbi.nlm.nih.gov/compound/69782063#section=2D-Structure
https://pubchem.ncbi.nlm.nih.gov/compound/69777934#section=2D-Structure
https://pubchem.ncbi.nlm.nih.gov/compound/69771586#section=2D-Structure
https://pubchem.ncbi.nlm.nih.gov/compound/69767361#section=2D-Structure
https://pubchem.ncbi.nlm.nih.gov/compound/69757646#section=2D-Structure
https://pubchem.ncbi.nlm.nih.gov/compound/69756146#section=2D-Structure
https://pubchem.ncbi.nlm.nih.gov/compound/69754744#section=2D-Structure
https://pubchem.ncbi.nlm.nih.gov/compound/69753968#section=2D-Structure
https://pubchem.ncbi.nlm.nih.gov/compound/69737331#section=2D-Structure
https://pubchem.ncbi.nlm.nih.gov/compound/69737067#section=2D-Structure
https://pubchem.ncbi.nlm.nih.gov/compound/69736900#section=2D-Structure
https://pubchem.ncbi.nlm.nih.gov/compound/69736688#section=2D-Structure
https://pubchem.ncbi.nlm.nih.gov/compound/69726197#section=2D-Structure
https://pubchem.ncbi.nlm.nih.gov/compound/69725814#section=2D-Structure
https://pubchem.ncbi.nlm.nih.gov/compound/69725022#section=2D-Structure
https://pubchem.ncbi.nlm.nih.gov/compound/69725021#section=2D-Structure
https://pubchem.ncbi.nlm.nih.gov/compound/69724643#section=2D-Structure
https://pubchem.ncbi.nlm.nih.gov/compound/69721233#section=2D-Structure
https://pubchem.ncbi.nlm.nih.gov/compound/69720366#section=2D-Structure
https://pubchem.ncbi.nlm.nih.gov/compound/69718607#section=2D-Structure
https://pubchem.ncbi.nlm.nih.gov/compound/69715440#section=2D-Structure
https://pubchem.ncbi.nlm.nih.gov/compound/69705275#section=2D-Structure
https://pubchem.ncbi.nlm.nih.gov/compound/69703734#section=2D-Structure
https://pubchem.ncbi.nlm.nih.gov/compound/69703466#section=2D-Structure
https://pubchem.ncbi.nlm.nih.gov/compound/69693631#section=2D-Structure
https://pubchem.ncbi.nlm.nih.gov/compound/69676231#section=2D-Structure
https://pubchem.ncbi.nlm.nih.gov/compound/69675539#section=2D-Structure
https://pubchem.ncbi.nlm.nih.gov/compound/69670848#section=2D-Structure
https://pubchem.ncbi.nlm.nih.gov/compound/69669048#section=2D-Structure
https://pubchem.ncbi.nlm.nih.gov/compound/69665929#section=2D-Structure
https://pubchem.ncbi.nlm.nih.gov/compound/69657213#section=2D-Structure
https://pubchem.ncbi.nlm.nih.gov/compound/69648974#section=2D-Structure
https://pubchem.ncbi.nlm.nih.gov/compound/69648951#section=2D-Structure
https://pubchem.ncbi.nlm.nih.gov/compound/69647161#section=2D-Structure
https://pubchem.ncbi.nlm.nih.gov/compound/69644657#section=2D-Structure
https://pubchem.ncbi.nlm.nih.gov/compound/69639967#section=2D-Structure
https://pubchem.ncbi.nlm.nih.gov/compound/69629541#section=2D-Structure
https://pubchem.ncbi.nlm.nih.gov/compound/69629142#section=2D-Structure
https://pubchem.ncbi.nlm.nih.gov/compound/69616450#section=2D-Structure
https://pubchem.ncbi.nlm.nih.gov/compound/69613154#section=2D-Structure
https://pubchem.ncbi.nlm.nih.gov/compound/69613117#section=2D-Structure
https://pubchem.ncbi.nlm.nih.gov/compound/69613078#section=2D-Structure
https://pubchem.ncbi.nlm.nih.gov/compound/69612324#section=2D-Structure
https://pubchem.ncbi.nlm.nih.gov/compound/69609088#section=2D-Structure
https://pubchem.ncbi.nlm.nih.gov/compound/69607802#section=2D-Structure
https://pubchem.ncbi.nlm.nih.gov/compound/69607737#section=2D-Structure

https://pubchem.ncbi.nlm.nih.gov/compound/69598669#section=2D-Structure
https://pubchem.ncbi.nlm.nih.gov/compound/69598664#section=2D-Structure
https://pubchem.ncbi.nlm.nih.gov/compound/69598647#section=2D-Structure
https://pubchem.ncbi.nlm.nih.gov/compound/69598637#section=2D-Structure
https://pubchem.ncbi.nlm.nih.gov/compound/69598634#section=2D-Structure
https://pubchem.ncbi.nlm.nih.gov/compound/69598629#section=2D-Structure
https://pubchem.ncbi.nlm.nih.gov/compound/69598606#section=2D-Structure
https://pubchem.ncbi.nlm.nih.gov/compound/69598591#section=2D-Structure
https://pubchem.ncbi.nlm.nih.gov/compound/69598584#section=2D-Structure
https://pubchem.ncbi.nlm.nih.gov/compound/69598583#section=2D-Structure
https://pubchem.ncbi.nlm.nih.gov/compound/69598571#section=2D-Structure
https://pubchem.ncbi.nlm.nih.gov/compound/69598568#section=2D-Structure
https://pubchem.ncbi.nlm.nih.gov/compound/69598561#section=2D-Structure
https://pubchem.ncbi.nlm.nih.gov/compound/69598549#section=2D-Structure
https://pubchem.ncbi.nlm.nih.gov/compound/69598548#section=2D-Structure
https://pubchem.ncbi.nlm.nih.gov/compound/69598539#section=2D-Structure
https://pubchem.ncbi.nlm.nih.gov/compound/69598534#section=2D-Structure
https://pubchem.ncbi.nlm.nih.gov/compound/69598523#section=2D-Structure
https://pubchem.ncbi.nlm.nih.gov/compound/69598517#section=2D-Structure
https://pubchem.ncbi.nlm.nih.gov/compound/69598516#section=2D-Structure
https://pubchem.ncbi.nlm.nih.gov/compound/69598467#section=2D-Structure
https://pubchem.ncbi.nlm.nih.gov/compound/69598447#section=2D-Structure
https://pubchem.ncbi.nlm.nih.gov/compound/69598443#section=2D-Structure
https://pubchem.ncbi.nlm.nih.gov/compound/69598439#section=2D-Structure
https://pubchem.ncbi.nlm.nih.gov/compound/69598424#section=2D-Structure
https://pubchem.ncbi.nlm.nih.gov/compound/69598413#section=2D-Structure
https://pubchem.ncbi.nlm.nih.gov/compound/69598406#section=2D-Structure
https://pubchem.ncbi.nlm.nih.gov/compound/69598402#section=2D-Structure
https://pubchem.ncbi.nlm.nih.gov/compound/69598399#section=2D-Structure
https://pubchem.ncbi.nlm.nih.gov/compound/69598398#section=2D-Structure
https://pubchem.ncbi.nlm.nih.gov/compound/69598394#section=2D-Structure
https://pubchem.ncbi.nlm.nih.gov/compound/69598386#section=2D-Structure
https://pubchem.ncbi.nlm.nih.gov/compound/69598363#section=2D-Structure
https://pubchem.ncbi.nlm.nih.gov/compound/69598343#section=2D-Structure
https://pubchem.ncbi.nlm.nih.gov/compound/69598326#section=2D-Structure
https://pubchem.ncbi.nlm.nih.gov/compound/69598318#section=2D-Structure
https://pubchem.ncbi.nlm.nih.gov/compound/69598281#section=2D-Structure
https://pubchem.ncbi.nlm.nih.gov/compound/69598276#section=2D-Structure
https://pubchem.ncbi.nlm.nih.gov/compound/69598254#section=2D-Structure
https://pubchem.ncbi.nlm.nih.gov/compound/69598251#section=2D-Structure
https://pubchem.ncbi.nlm.nih.gov/compound/69598234#section=2D-Structure
https://pubchem.ncbi.nlm.nih.gov/compound/69598220#section=2D-Structure
https://pubchem.ncbi.nlm.nih.gov/compound/69598210#section=2D-Structure
https://pubchem.ncbi.nlm.nih.gov/compound/69598199#section=2D-Structure
https://pubchem.ncbi.nlm.nih.gov/compound/69598176#section=2D-Structure
https://pubchem.ncbi.nlm.nih.gov/compound/69596941#section=2D-Structure
https://pubchem.ncbi.nlm.nih.gov/compound/69596925#section=2D-Structure
https://pubchem.ncbi.nlm.nih.gov/compound/69591429#section=2D-Structure
https://pubchem.ncbi.nlm.nih.gov/compound/69588120#section=2D-Structure
https://pubchem.ncbi.nlm.nih.gov/compound/69572418#section=2D-Structure
https://pubchem.ncbi.nlm.nih.gov/compound/69569485#section=2D-Structure
https://pubchem.ncbi.nlm.nih.gov/compound/69562504#section=2D-Structure
https://pubchem.ncbi.nlm.nih.gov/compound/69559088#section=2D-Structure
https://pubchem.ncbi.nlm.nih.gov/compound/69557155#section=2D-Structure
https://pubchem.ncbi.nlm.nih.gov/compound/69550728#section=2D-Structure

https://pubchem.ncbi.nlm.nih.gov/compound/69548963#section=2D-Structure
https://pubchem.ncbi.nlm.nih.gov/compound/69546271#section=2D-Structure
https://pubchem.ncbi.nlm.nih.gov/compound/69538666#section=2D-Structure
https://pubchem.ncbi.nlm.nih.gov/compound/69527589#section=2D-Structure
https://pubchem.ncbi.nlm.nih.gov/compound/69522641#section=2D-Structure
https://pubchem.ncbi.nlm.nih.gov/compound/69520291#section=2D-Structure
https://pubchem.ncbi.nlm.nih.gov/compound/69510979#section=2D-Structure
https://pubchem.ncbi.nlm.nih.gov/compound/69505374#section=2D-Structure
https://pubchem.ncbi.nlm.nih.gov/compound/69499659#section=2D-Structure
https://pubchem.ncbi.nlm.nih.gov/compound/69497208#section=2D-Structure
https://pubchem.ncbi.nlm.nih.gov/compound/69488791#section=2D-Structure
https://pubchem.ncbi.nlm.nih.gov/compound/69488785#section=2D-Structure
https://pubchem.ncbi.nlm.nih.gov/compound/69486140#section=2D-Structure
https://pubchem.ncbi.nlm.nih.gov/compound/69479746#section=2D-Structure
https://pubchem.ncbi.nlm.nih.gov/compound/69477271#section=2D-Structure
https://pubchem.ncbi.nlm.nih.gov/compound/69472375#section=2D-Structure
https://pubchem.ncbi.nlm.nih.gov/compound/69469281#section=2D-Structure
https://pubchem.ncbi.nlm.nih.gov/compound/69462#section=2D-Structure
https://pubchem.ncbi.nlm.nih.gov/compound/69461657#section=2D-Structure
https://pubchem.ncbi.nlm.nih.gov/compound/69458728#section=2D-Structure
https://pubchem.ncbi.nlm.nih.gov/compound/69450160#section=2D-Structure
https://pubchem.ncbi.nlm.nih.gov/compound/69439267#section=2D-Structure
https://pubchem.ncbi.nlm.nih.gov/compound/69432054#section=2D-Structure
https://pubchem.ncbi.nlm.nih.gov/compound/69429377#section=2D-Structure
https://pubchem.ncbi.nlm.nih.gov/compound/69416189#section=2D-Structure
https://pubchem.ncbi.nlm.nih.gov/compound/69412913#section=2D-Structure
https://pubchem.ncbi.nlm.nih.gov/compound/69410507#section=2D-Structure
https://pubchem.ncbi.nlm.nih.gov/compound/69405928#section=2D-Structure
https://pubchem.ncbi.nlm.nih.gov/compound/69405559#section=2D-Structure
https://pubchem.ncbi.nlm.nih.gov/compound/69402487#section=2D-Structure
https://pubchem.ncbi.nlm.nih.gov/compound/69378989#section=2D-Structure
https://pubchem.ncbi.nlm.nih.gov/compound/69378045#section=2D-Structure
https://pubchem.ncbi.nlm.nih.gov/compound/69375690#section=2D-Structure
https://pubchem.ncbi.nlm.nih.gov/compound/69368114#section=2D-Structure
https://pubchem.ncbi.nlm.nih.gov/compound/69367842#section=2D-Structure
https://pubchem.ncbi.nlm.nih.gov/compound/69362970#section=2D-Structure
https://pubchem.ncbi.nlm.nih.gov/compound/69350456#section=2D-Structure
https://pubchem.ncbi.nlm.nih.gov/compound/69349938#section=2D-Structure
https://pubchem.ncbi.nlm.nih.gov/compound/69348047#section=2D-Structure
https://pubchem.ncbi.nlm.nih.gov/compound/69346566#section=2D-Structure
https://pubchem.ncbi.nlm.nih.gov/compound/69345756#section=2D-Structure
https://pubchem.ncbi.nlm.nih.gov/compound/69345662#section=2D-Structure
https://pubchem.ncbi.nlm.nih.gov/compound/69342864#section=2D-Structure
https://pubchem.ncbi.nlm.nih.gov/compound/69339815#section=2D-Structure
https://pubchem.ncbi.nlm.nih.gov/compound/69336569#section=2D-Structure
https://pubchem.ncbi.nlm.nih.gov/compound/69324817#section=2D-Structure
https://pubchem.ncbi.nlm.nih.gov/compound/69322669#section=2D-Structure
https://pubchem.ncbi.nlm.nih.gov/compound/69303509#section=2D-Structure
https://pubchem.ncbi.nlm.nih.gov/compound/69302209#section=2D-Structure
https://pubchem.ncbi.nlm.nih.gov/compound/69301064#section=2D-Structure
https://pubchem.ncbi.nlm.nih.gov/compound/69291375#section=2D-Structure
https://pubchem.ncbi.nlm.nih.gov/compound/69288403#section=2D-Structure
https://pubchem.ncbi.nlm.nih.gov/compound/69280525#section=2D-Structure
https://pubchem.ncbi.nlm.nih.gov/compound/69278912#section=2D-Structure
https://pubchem.ncbi.nlm.nih.gov/compound/69265584#section=2D-Structure

https://pubchem.ncbi.nlm.nih.gov/compound/69258867#section=2D-Structure
https://pubchem.ncbi.nlm.nih.gov/compound/69254775#section=2D-Structure
https://pubchem.ncbi.nlm.nih.gov/compound/69252361#section=2D-Structure
https://pubchem.ncbi.nlm.nih.gov/compound/69252072#section=2D-Structure
https://pubchem.ncbi.nlm.nih.gov/compound/69251230#section=2D-Structure
https://pubchem.ncbi.nlm.nih.gov/compound/69239192#section=2D-Structure
https://pubchem.ncbi.nlm.nih.gov/compound/69233172#section=2D-Structure
https://pubchem.ncbi.nlm.nih.gov/compound/69224613#section=2D-Structure
https://pubchem.ncbi.nlm.nih.gov/compound/69224317#section=2D-Structure
https://pubchem.ncbi.nlm.nih.gov/compound/69223480#section=2D-Structure
https://pubchem.ncbi.nlm.nih.gov/compound/69221655#section=2D-Structure
https://pubchem.ncbi.nlm.nih.gov/compound/69215177#section=2D-Structure
https://pubchem.ncbi.nlm.nih.gov/compound/69214598#section=2D-Structure
https://pubchem.ncbi.nlm.nih.gov/compound/69211913#section=2D-Structure
https://pubchem.ncbi.nlm.nih.gov/compound/69210036#section=2D-Structure
https://pubchem.ncbi.nlm.nih.gov/compound/6919885#section=2D-Structure
https://pubchem.ncbi.nlm.nih.gov/compound/69192727#section=2D-Structure
https://pubchem.ncbi.nlm.nih.gov/compound/69191637#section=2D-Structure
https://pubchem.ncbi.nlm.nih.gov/compound/69189548#section=2D-Structure
https://pubchem.ncbi.nlm.nih.gov/compound/69187757#section=2D-Structure
https://pubchem.ncbi.nlm.nih.gov/compound/69187503#section=2D-Structure
https://pubchem.ncbi.nlm.nih.gov/compound/69187083#section=2D-Structure
https://pubchem.ncbi.nlm.nih.gov/compound/69186425#section=2D-Structure
https://pubchem.ncbi.nlm.nih.gov/compound/69183921#section=2D-Structure
https://pubchem.ncbi.nlm.nih.gov/compound/69181946#section=2D-Structure
https://pubchem.ncbi.nlm.nih.gov/compound/69180629#section=2D-Structure
https://pubchem.ncbi.nlm.nih.gov/compound/69178831#section=2D-Structure
https://pubchem.ncbi.nlm.nih.gov/compound/69177964#section=2D-Structure
https://pubchem.ncbi.nlm.nih.gov/compound/69176447#section=2D-Structure
https://pubchem.ncbi.nlm.nih.gov/compound/69176426#section=2D-Structure
https://pubchem.ncbi.nlm.nih.gov/compound/69175011#section=2D-Structure
https://pubchem.ncbi.nlm.nih.gov/compound/69173896#section=2D-Structure
https://pubchem.ncbi.nlm.nih.gov/compound/69163999#section=2D-Structure
https://pubchem.ncbi.nlm.nih.gov/compound/69163665#section=2D-Structure
https://pubchem.ncbi.nlm.nih.gov/compound/69163567#section=2D-Structure
https://pubchem.ncbi.nlm.nih.gov/compound/69163186#section=2D-Structure
https://pubchem.ncbi.nlm.nih.gov/compound/69154169#section=2D-Structure
https://pubchem.ncbi.nlm.nih.gov/compound/6914501#section=2D-Structure
https://pubchem.ncbi.nlm.nih.gov/compound/6914296#section=2D-Structure
https://pubchem.ncbi.nlm.nih.gov/compound/69142392#section=2D-Structure
https://pubchem.ncbi.nlm.nih.gov/compound/69131791#section=2D-Structure
https://pubchem.ncbi.nlm.nih.gov/compound/69130767#section=2D-Structure
https://pubchem.ncbi.nlm.nih.gov/compound/69123951#section=2D-Structure
https://pubchem.ncbi.nlm.nih.gov/compound/69106177#section=2D-Structure
https://pubchem.ncbi.nlm.nih.gov/compound/69106159#section=2D-Structure
https://pubchem.ncbi.nlm.nih.gov/compound/69090327#section=2D-Structure
https://pubchem.ncbi.nlm.nih.gov/compound/69088809#section=2D-Structure
https://pubchem.ncbi.nlm.nih.gov/compound/69079878#section=2D-Structure
https://pubchem.ncbi.nlm.nih.gov/compound/69068461#section=2D-Structure
https://pubchem.ncbi.nlm.nih.gov/compound/69061570#section=2D-Structure
https://pubchem.ncbi.nlm.nih.gov/compound/69060669#section=2D-Structure
https://pubchem.ncbi.nlm.nih.gov/compound/69058597#section=2D-Structure
https://pubchem.ncbi.nlm.nih.gov/compound/69057366#section=2D-Structure
https://pubchem.ncbi.nlm.nih.gov/compound/69052613#section=2D-Structure
https://pubchem.ncbi.nlm.nih.gov/compound/69045078#section=2D-Structure

https://pubchem.ncbi.nlm.nih.gov/compound/69044312#section=2D-Structure
https://pubchem.ncbi.nlm.nih.gov/compound/69040606#section=2D-Structure
https://pubchem.ncbi.nlm.nih.gov/compound/69040223#section=2D-Structure
https://pubchem.ncbi.nlm.nih.gov/compound/69036656#section=2D-Structure
https://pubchem.ncbi.nlm.nih.gov/compound/69031466#section=2D-Structure
https://pubchem.ncbi.nlm.nih.gov/compound/69022475#section=2D-Structure
https://pubchem.ncbi.nlm.nih.gov/compound/69016174#section=2D-Structure
https://pubchem.ncbi.nlm.nih.gov/compound/68994199#section=2D-Structure
https://pubchem.ncbi.nlm.nih.gov/compound/68978773#section=2D-Structure
https://pubchem.ncbi.nlm.nih.gov/compound/68969732#section=2D-Structure
https://pubchem.ncbi.nlm.nih.gov/compound/68953157#section=2D-Structure
https://pubchem.ncbi.nlm.nih.gov/compound/68947878#section=2D-Structure
https://pubchem.ncbi.nlm.nih.gov/compound/68947844#section=2D-Structure
https://pubchem.ncbi.nlm.nih.gov/compound/68947736#section=2D-Structure
https://pubchem.ncbi.nlm.nih.gov/compound/68945343#section=2D-Structure
https://pubchem.ncbi.nlm.nih.gov/compound/68943201#section=2D-Structure
https://pubchem.ncbi.nlm.nih.gov/compound/68940503#section=2D-Structure
https://pubchem.ncbi.nlm.nih.gov/compound/68931743#section=2D-Structure
https://pubchem.ncbi.nlm.nih.gov/compound/68931483#section=2D-Structure
https://pubchem.ncbi.nlm.nih.gov/compound/68924582#section=2D-Structure
https://pubchem.ncbi.nlm.nih.gov/compound/68879262#section=2D-Structure
https://pubchem.ncbi.nlm.nih.gov/compound/68870743#section=2D-Structure
https://pubchem.ncbi.nlm.nih.gov/compound/68865416#section=2D-Structure
https://pubchem.ncbi.nlm.nih.gov/compound/68864105#section=2D-Structure
https://pubchem.ncbi.nlm.nih.gov/compound/68862303#section=2D-Structure
https://pubchem.ncbi.nlm.nih.gov/compound/68861245#section=2D-Structure
https://pubchem.ncbi.nlm.nih.gov/compound/68860257#section=2D-Structure
https://pubchem.ncbi.nlm.nih.gov/compound/68853780#section=2D-Structure
https://pubchem.ncbi.nlm.nih.gov/compound/68847825#section=2D-Structure
https://pubchem.ncbi.nlm.nih.gov/compound/68843997#section=2D-Structure
https://pubchem.ncbi.nlm.nih.gov/compound/68842660#section=2D-Structure
https://pubchem.ncbi.nlm.nih.gov/compound/68839798#section=2D-Structure
https://pubchem.ncbi.nlm.nih.gov/compound/68835035#section=2D-Structure
https://pubchem.ncbi.nlm.nih.gov/compound/68824948#section=2D-Structure
https://pubchem.ncbi.nlm.nih.gov/compound/68820655#section=2D-Structure
https://pubchem.ncbi.nlm.nih.gov/compound/68814964#section=2D-Structure
https://pubchem.ncbi.nlm.nih.gov/compound/68801840#section=2D-Structure
https://pubchem.ncbi.nlm.nih.gov/compound/68782051#section=2D-Structure
https://pubchem.ncbi.nlm.nih.gov/compound/68776835#section=2D-Structure
https://pubchem.ncbi.nlm.nih.gov/compound/68775748#section=2D-Structure
https://pubchem.ncbi.nlm.nih.gov/compound/68771875#section=2D-Structure
https://pubchem.ncbi.nlm.nih.gov/compound/68740851#section=2D-Structure
https://pubchem.ncbi.nlm.nih.gov/compound/68736329#section=2D-Structure
https://pubchem.ncbi.nlm.nih.gov/compound/68727534#section=2D-Structure
https://pubchem.ncbi.nlm.nih.gov/compound/68722404#section=2D-Structure
https://pubchem.ncbi.nlm.nih.gov/compound/68720074#section=2D-Structure
https://pubchem.ncbi.nlm.nih.gov/compound/68719520#section=2D-Structure
https://pubchem.ncbi.nlm.nih.gov/compound/68716508#section=2D-Structure
https://pubchem.ncbi.nlm.nih.gov/compound/68715261#section=2D-Structure
https://pubchem.ncbi.nlm.nih.gov/compound/68706097#section=2D-Structure
https://pubchem.ncbi.nlm.nih.gov/compound/68700270#section=2D-Structure
https://pubchem.ncbi.nlm.nih.gov/compound/68699805#section=2D-Structure
https://pubchem.ncbi.nlm.nih.gov/compound/68680236#section=2D-Structure
https://pubchem.ncbi.nlm.nih.gov/compound/68680161#section=2D-Structure
https://pubchem.ncbi.nlm.nih.gov/compound/68672916#section=2D-Structure

https://pubchem.ncbi.nlm.nih.gov/compound/68664293#section=2D-Structure
https://pubchem.ncbi.nlm.nih.gov/compound/68661467#section=2D-Structure
https://pubchem.ncbi.nlm.nih.gov/compound/68658097#section=2D-Structure
https://pubchem.ncbi.nlm.nih.gov/compound/68656243#section=2D-Structure
https://pubchem.ncbi.nlm.nih.gov/compound/68655731#section=2D-Structure
https://pubchem.ncbi.nlm.nih.gov/compound/68650046#section=2D-Structure
https://pubchem.ncbi.nlm.nih.gov/compound/68638191#section=2D-Structure
https://pubchem.ncbi.nlm.nih.gov/compound/68636997#section=2D-Structure
https://pubchem.ncbi.nlm.nih.gov/compound/68634703#section=2D-Structure
https://pubchem.ncbi.nlm.nih.gov/compound/68634408#section=2D-Structure
https://pubchem.ncbi.nlm.nih.gov/compound/68634028#section=2D-Structure
https://pubchem.ncbi.nlm.nih.gov/compound/68632147#section=2D-Structure
https://pubchem.ncbi.nlm.nih.gov/compound/68622639#section=2D-Structure
https://pubchem.ncbi.nlm.nih.gov/compound/68611020#section=2D-Structure
https://pubchem.ncbi.nlm.nih.gov/compound/68607565#section=2D-Structure
https://pubchem.ncbi.nlm.nih.gov/compound/68606889#section=2D-Structure
https://pubchem.ncbi.nlm.nih.gov/compound/68602667#section=2D-Structure
https://pubchem.ncbi.nlm.nih.gov/compound/68601250#section=2D-Structure
https://pubchem.ncbi.nlm.nih.gov/compound/68583944#section=2D-Structure
https://pubchem.ncbi.nlm.nih.gov/compound/68573136#section=2D-Structure
https://pubchem.ncbi.nlm.nih.gov/compound/68563294#section=2D-Structure
https://pubchem.ncbi.nlm.nih.gov/compound/68563250#section=2D-Structure
https://pubchem.ncbi.nlm.nih.gov/compound/68562329#section=2D-Structure
https://pubchem.ncbi.nlm.nih.gov/compound/68559299#section=2D-Structure
https://pubchem.ncbi.nlm.nih.gov/compound/68558068#section=2D-Structure
https://pubchem.ncbi.nlm.nih.gov/compound/68557275#section=2D-Structure
https://pubchem.ncbi.nlm.nih.gov/compound/68557084#section=2D-Structure
https://pubchem.ncbi.nlm.nih.gov/compound/68550528#section=2D-Structure
https://pubchem.ncbi.nlm.nih.gov/compound/68550505#section=2D-Structure
https://pubchem.ncbi.nlm.nih.gov/compound/68549659#section=2D-Structure
https://pubchem.ncbi.nlm.nih.gov/compound/68549169#section=2D-Structure
https://pubchem.ncbi.nlm.nih.gov/compound/68546453#section=2D-Structure
https://pubchem.ncbi.nlm.nih.gov/compound/68546394#section=2D-Structure
https://pubchem.ncbi.nlm.nih.gov/compound/68546027#section=2D-Structure
https://pubchem.ncbi.nlm.nih.gov/compound/68544834#section=2D-Structure
https://pubchem.ncbi.nlm.nih.gov/compound/68544410#section=2D-Structure
https://pubchem.ncbi.nlm.nih.gov/compound/68544103#section=2D-Structure
https://pubchem.ncbi.nlm.nih.gov/compound/68543666#section=2D-Structure
https://pubchem.ncbi.nlm.nih.gov/compound/68543155#section=2D-Structure
https://pubchem.ncbi.nlm.nih.gov/compound/68543152#section=2D-Structure
https://pubchem.ncbi.nlm.nih.gov/compound/68542462#section=2D-Structure
https://pubchem.ncbi.nlm.nih.gov/compound/68541333#section=2D-Structure
https://pubchem.ncbi.nlm.nih.gov/compound/68539071#section=2D-Structure
https://pubchem.ncbi.nlm.nih.gov/compound/68538399#section=2D-Structure
https://pubchem.ncbi.nlm.nih.gov/compound/68526277#section=2D-Structure
https://pubchem.ncbi.nlm.nih.gov/compound/68522826#section=2D-Structure
https://pubchem.ncbi.nlm.nih.gov/compound/68522367#section=2D-Structure
https://pubchem.ncbi.nlm.nih.gov/compound/68520593#section=2D-Structure
https://pubchem.ncbi.nlm.nih.gov/compound/68519754#section=2D-Structure
https://pubchem.ncbi.nlm.nih.gov/compound/68506858#section=2D-Structure
https://pubchem.ncbi.nlm.nih.gov/compound/68506070#section=2D-Structure
https://pubchem.ncbi.nlm.nih.gov/compound/68504927#section=2D-Structure
https://pubchem.ncbi.nlm.nih.gov/compound/68504267#section=2D-Structure
https://pubchem.ncbi.nlm.nih.gov/compound/68503602#section=2D-Structure
https://pubchem.ncbi.nlm.nih.gov/compound/6850311#section=2D-Structure

https://pubchem.ncbi.nlm.nih.gov/compound/68502711#section=2D-Structure
https://pubchem.ncbi.nlm.nih.gov/compound/68502710#section=2D-Structure
https://pubchem.ncbi.nlm.nih.gov/compound/68501296#section=2D-Structure
https://pubchem.ncbi.nlm.nih.gov/compound/6850073#section=2D-Structure
https://pubchem.ncbi.nlm.nih.gov/compound/68500384#section=2D-Structure
https://pubchem.ncbi.nlm.nih.gov/compound/68500383#section=2D-Structure
https://pubchem.ncbi.nlm.nih.gov/compound/68492879#section=2D-Structure
https://pubchem.ncbi.nlm.nih.gov/compound/68488833#section=2D-Structure
https://pubchem.ncbi.nlm.nih.gov/compound/68488832#section=2D-Structure
https://pubchem.ncbi.nlm.nih.gov/compound/68488319#section=2D-Structure
https://pubchem.ncbi.nlm.nih.gov/compound/68487934#section=2D-Structure
https://pubchem.ncbi.nlm.nih.gov/compound/68485#section=2D-Structure
https://pubchem.ncbi.nlm.nih.gov/compound/68483993#section=2D-Structure
https://pubchem.ncbi.nlm.nih.gov/compound/68483470#section=2D-Structure
https://pubchem.ncbi.nlm.nih.gov/compound/68447974#section=2D-Structure
https://pubchem.ncbi.nlm.nih.gov/compound/68447289#section=2D-Structure
https://pubchem.ncbi.nlm.nih.gov/compound/68421139#section=2D-Structure
https://pubchem.ncbi.nlm.nih.gov/compound/68415096#section=2D-Structure
https://pubchem.ncbi.nlm.nih.gov/compound/68407483#section=2D-Structure
https://pubchem.ncbi.nlm.nih.gov/compound/68385012#section=2D-Structure
https://pubchem.ncbi.nlm.nih.gov/compound/68383846#section=2D-Structure
https://pubchem.ncbi.nlm.nih.gov/compound/68373323#section=2D-Structure
https://pubchem.ncbi.nlm.nih.gov/compound/68359067#section=2D-Structure
https://pubchem.ncbi.nlm.nih.gov/compound/68348635#section=2D-Structure
https://pubchem.ncbi.nlm.nih.gov/compound/68345740#section=2D-Structure
https://pubchem.ncbi.nlm.nih.gov/compound/68343555#section=2D-Structure
https://pubchem.ncbi.nlm.nih.gov/compound/68336299#section=2D-Structure
https://pubchem.ncbi.nlm.nih.gov/compound/68328829#section=2D-Structure
https://pubchem.ncbi.nlm.nih.gov/compound/68326505#section=2D-Structure
https://pubchem.ncbi.nlm.nih.gov/compound/68326264#section=2D-Structure
https://pubchem.ncbi.nlm.nih.gov/compound/68326263#section=2D-Structure
https://pubchem.ncbi.nlm.nih.gov/compound/68319445#section=2D-Structure
https://pubchem.ncbi.nlm.nih.gov/compound/68292403#section=2D-Structure
https://pubchem.ncbi.nlm.nih.gov/compound/68281758#section=2D-Structure
https://pubchem.ncbi.nlm.nih.gov/compound/68278#section=2D-Structure
https://pubchem.ncbi.nlm.nih.gov/compound/68274032#section=2D-Structure
https://pubchem.ncbi.nlm.nih.gov/compound/68271413#section=2D-Structure
https://pubchem.ncbi.nlm.nih.gov/compound/68264817#section=2D-Structure
https://pubchem.ncbi.nlm.nih.gov/compound/68264362#section=2D-Structure
https://pubchem.ncbi.nlm.nih.gov/compound/68264255#section=2D-Structure
https://pubchem.ncbi.nlm.nih.gov/compound/68250020#section=2D-Structure
https://pubchem.ncbi.nlm.nih.gov/compound/68244947#section=2D-Structure
https://pubchem.ncbi.nlm.nih.gov/compound/68240631#section=2D-Structure
https://pubchem.ncbi.nlm.nih.gov/compound/68227572#section=2D-Structure
https://pubchem.ncbi.nlm.nih.gov/compound/68214849#section=2D-Structure
https://pubchem.ncbi.nlm.nih.gov/compound/68201186#section=2D-Structure
https://pubchem.ncbi.nlm.nih.gov/compound/68194511#section=2D-Structure
https://pubchem.ncbi.nlm.nih.gov/compound/68191746#section=2D-Structure
https://pubchem.ncbi.nlm.nih.gov/compound/68189521#section=2D-Structure
https://pubchem.ncbi.nlm.nih.gov/compound/68178842#section=2D-Structure
https://pubchem.ncbi.nlm.nih.gov/compound/68177973#section=2D-Structure
https://pubchem.ncbi.nlm.nih.gov/compound/68171021#section=2D-Structure
https://pubchem.ncbi.nlm.nih.gov/compound/68169663#section=2D-Structure
https://pubchem.ncbi.nlm.nih.gov/compound/68149930#section=2D-Structure
https://pubchem.ncbi.nlm.nih.gov/compound/68149755#section=2D-Structure

https://pubchem.ncbi.nlm.nih.gov/compound/68148980#section=2D-Structure
https://pubchem.ncbi.nlm.nih.gov/compound/68148480#section=2D-Structure
https://pubchem.ncbi.nlm.nih.gov/compound/68145156#section=2D-Structure
https://pubchem.ncbi.nlm.nih.gov/compound/68144553#section=2D-Structure
https://pubchem.ncbi.nlm.nih.gov/compound/68143486#section=2D-Structure
https://pubchem.ncbi.nlm.nih.gov/compound/68143274#section=2D-Structure
https://pubchem.ncbi.nlm.nih.gov/compound/68141465#section=2D-Structure
https://pubchem.ncbi.nlm.nih.gov/compound/68140954#section=2D-Structure
https://pubchem.ncbi.nlm.nih.gov/compound/68140580#section=2D-Structure
https://pubchem.ncbi.nlm.nih.gov/compound/68136737#section=2D-Structure
https://pubchem.ncbi.nlm.nih.gov/compound/68134593#section=2D-Structure
https://pubchem.ncbi.nlm.nih.gov/compound/68131193#section=2D-Structure
https://pubchem.ncbi.nlm.nih.gov/compound/68124553#section=2D-Structure
https://pubchem.ncbi.nlm.nih.gov/compound/68121224#section=2D-Structure
https://pubchem.ncbi.nlm.nih.gov/compound/68118299#section=2D-Structure
https://pubchem.ncbi.nlm.nih.gov/compound/68114295#section=2D-Structure
https://pubchem.ncbi.nlm.nih.gov/compound/68094#section=2D-Structure
https://pubchem.ncbi.nlm.nih.gov/compound/68092327#section=2D-Structure
https://pubchem.ncbi.nlm.nih.gov/compound/68090304#section=2D-Structure
https://pubchem.ncbi.nlm.nih.gov/compound/68075#section=2D-Structure
https://pubchem.ncbi.nlm.nih.gov/compound/68071784#section=2D-Structure
https://pubchem.ncbi.nlm.nih.gov/compound/68069042#section=2D-Structure
https://pubchem.ncbi.nlm.nih.gov/compound/68061309#section=2D-Structure
https://pubchem.ncbi.nlm.nih.gov/compound/68059808#section=2D-Structure
https://pubchem.ncbi.nlm.nih.gov/compound/68058392#section=2D-Structure
https://pubchem.ncbi.nlm.nih.gov/compound/68058388#section=2D-Structure
https://pubchem.ncbi.nlm.nih.gov/compound/68052704#section=2D-Structure
https://pubchem.ncbi.nlm.nih.gov/compound/68021362#section=2D-Structure
https://pubchem.ncbi.nlm.nih.gov/compound/68013254#section=2D-Structure
https://pubchem.ncbi.nlm.nih.gov/compound/68009492#section=2D-Structure
https://pubchem.ncbi.nlm.nih.gov/compound/67994276#section=2D-Structure
https://pubchem.ncbi.nlm.nih.gov/compound/67994253#section=2D-Structure
https://pubchem.ncbi.nlm.nih.gov/compound/67994139#section=2D-Structure
https://pubchem.ncbi.nlm.nih.gov/compound/67994072#section=2D-Structure
https://pubchem.ncbi.nlm.nih.gov/compound/67994056#section=2D-Structure
https://pubchem.ncbi.nlm.nih.gov/compound/67993986#section=2D-Structure
https://pubchem.ncbi.nlm.nih.gov/compound/67993781#section=2D-Structure
https://pubchem.ncbi.nlm.nih.gov/compound/67991293#section=2D-Structure
https://pubchem.ncbi.nlm.nih.gov/compound/67991292#section=2D-Structure
https://pubchem.ncbi.nlm.nih.gov/compound/67982447#section=2D-Structure
https://pubchem.ncbi.nlm.nih.gov/compound/67981342#section=2D-Structure
https://pubchem.ncbi.nlm.nih.gov/compound/67980328#section=2D-Structure
https://pubchem.ncbi.nlm.nih.gov/compound/67958971#section=2D-Structure
https://pubchem.ncbi.nlm.nih.gov/compound/67958853#section=2D-Structure
https://pubchem.ncbi.nlm.nih.gov/compound/67957864#section=2D-Structure
https://pubchem.ncbi.nlm.nih.gov/compound/67939022#section=2D-Structure
https://pubchem.ncbi.nlm.nih.gov/compound/67938558#section=2D-Structure
https://pubchem.ncbi.nlm.nih.gov/compound/67935666#section=2D-Structure
https://pubchem.ncbi.nlm.nih.gov/compound/67934078#section=2D-Structure
https://pubchem.ncbi.nlm.nih.gov/compound/67932577#section=2D-Structure
https://pubchem.ncbi.nlm.nih.gov/compound/67919580#section=2D-Structure
https://pubchem.ncbi.nlm.nih.gov/compound/67918595#section=2D-Structure
https://pubchem.ncbi.nlm.nih.gov/compound/67915990#section=2D-Structure
https://pubchem.ncbi.nlm.nih.gov/compound/67915731#section=2D-Structure
https://pubchem.ncbi.nlm.nih.gov/compound/67910998#section=2D-Structure

https://pubchem.ncbi.nlm.nih.gov/compound/67907432#section=2D-Structure
https://pubchem.ncbi.nlm.nih.gov/compound/67904080#section=2D-Structure
https://pubchem.ncbi.nlm.nih.gov/compound/67902282#section=2D-Structure
https://pubchem.ncbi.nlm.nih.gov/compound/67894084#section=2D-Structure
https://pubchem.ncbi.nlm.nih.gov/compound/67893619#section=2D-Structure
https://pubchem.ncbi.nlm.nih.gov/compound/67892802#section=2D-Structure
https://pubchem.ncbi.nlm.nih.gov/compound/67885998#section=2D-Structure
https://pubchem.ncbi.nlm.nih.gov/compound/67885991#section=2D-Structure
https://pubchem.ncbi.nlm.nih.gov/compound/67880762#section=2D-Structure
https://pubchem.ncbi.nlm.nih.gov/compound/67878880#section=2D-Structure
https://pubchem.ncbi.nlm.nih.gov/compound/67872874#section=2D-Structure
https://pubchem.ncbi.nlm.nih.gov/compound/67867836#section=2D-Structure
https://pubchem.ncbi.nlm.nih.gov/compound/67858820#section=2D-Structure
https://pubchem.ncbi.nlm.nih.gov/compound/67858401#section=2D-Structure
https://pubchem.ncbi.nlm.nih.gov/compound/67854465#section=2D-Structure
https://pubchem.ncbi.nlm.nih.gov/compound/67854098#section=2D-Structure
https://pubchem.ncbi.nlm.nih.gov/compound/67851688#section=2D-Structure
https://pubchem.ncbi.nlm.nih.gov/compound/67851473#section=2D-Structure
https://pubchem.ncbi.nlm.nih.gov/compound/67848872#section=2D-Structure
https://pubchem.ncbi.nlm.nih.gov/compound/67847947#section=2D-Structure
https://pubchem.ncbi.nlm.nih.gov/compound/67845634#section=2D-Structure
https://pubchem.ncbi.nlm.nih.gov/compound/67843409#section=2D-Structure
https://pubchem.ncbi.nlm.nih.gov/compound/67839569#section=2D-Structure
https://pubchem.ncbi.nlm.nih.gov/compound/67839242#section=2D-Structure
https://pubchem.ncbi.nlm.nih.gov/compound/67838209#section=2D-Structure
https://pubchem.ncbi.nlm.nih.gov/compound/67835818#section=2D-Structure
https://pubchem.ncbi.nlm.nih.gov/compound/67835501#section=2D-Structure
https://pubchem.ncbi.nlm.nih.gov/compound/67828500#section=2D-Structure
https://pubchem.ncbi.nlm.nih.gov/compound/67827675#section=2D-Structure
https://pubchem.ncbi.nlm.nih.gov/compound/67825781#section=2D-Structure
https://pubchem.ncbi.nlm.nih.gov/compound/67823571#section=2D-Structure
https://pubchem.ncbi.nlm.nih.gov/compound/67821907#section=2D-Structure
https://pubchem.ncbi.nlm.nih.gov/compound/67815094#section=2D-Structure
https://pubchem.ncbi.nlm.nih.gov/compound/67811136#section=2D-Structure
https://pubchem.ncbi.nlm.nih.gov/compound/67810965#section=2D-Structure
https://pubchem.ncbi.nlm.nih.gov/compound/67809652#section=2D-Structure
https://pubchem.ncbi.nlm.nih.gov/compound/67809345#section=2D-Structure
https://pubchem.ncbi.nlm.nih.gov/compound/67802011#section=2D-Structure
https://pubchem.ncbi.nlm.nih.gov/compound/67800159#section=2D-Structure
https://pubchem.ncbi.nlm.nih.gov/compound/67799541#section=2D-Structure
https://pubchem.ncbi.nlm.nih.gov/compound/67793346#section=2D-Structure
https://pubchem.ncbi.nlm.nih.gov/compound/67781869#section=2D-Structure
https://pubchem.ncbi.nlm.nih.gov/compound/67780081#section=2D-Structure
https://pubchem.ncbi.nlm.nih.gov/compound/67770360#section=2D-Structure
https://pubchem.ncbi.nlm.nih.gov/compound/67770325#section=2D-Structure
https://pubchem.ncbi.nlm.nih.gov/compound/67767526#section=2D-Structure
https://pubchem.ncbi.nlm.nih.gov/compound/67760843#section=2D-Structure
https://pubchem.ncbi.nlm.nih.gov/compound/67760526#section=2D-Structure
https://pubchem.ncbi.nlm.nih.gov/compound/67749665#section=2D-Structure
https://pubchem.ncbi.nlm.nih.gov/compound/67740346#section=2D-Structure
https://pubchem.ncbi.nlm.nih.gov/compound/67736002#section=2D-Structure
https://pubchem.ncbi.nlm.nih.gov/compound/67731834#section=2D-Structure
https://pubchem.ncbi.nlm.nih.gov/compound/67730165#section=2D-Structure
https://pubchem.ncbi.nlm.nih.gov/compound/67726965#section=2D-Structure
https://pubchem.ncbi.nlm.nih.gov/compound/67723995#section=2D-Structure

https://pubchem.ncbi.nlm.nih.gov/compound/67723546#section=2D-Structure
https://pubchem.ncbi.nlm.nih.gov/compound/67721185#section=2D-Structure
https://pubchem.ncbi.nlm.nih.gov/compound/67717488#section=2D-Structure
https://pubchem.ncbi.nlm.nih.gov/compound/67717094#section=2D-Structure
https://pubchem.ncbi.nlm.nih.gov/compound/67715130#section=2D-Structure
https://pubchem.ncbi.nlm.nih.gov/compound/67713480#section=2D-Structure
https://pubchem.ncbi.nlm.nih.gov/compound/67706128#section=2D-Structure
https://pubchem.ncbi.nlm.nih.gov/compound/67703985#section=2D-Structure
https://pubchem.ncbi.nlm.nih.gov/compound/67698111#section=2D-Structure
https://pubchem.ncbi.nlm.nih.gov/compound/67697978#section=2D-Structure
https://pubchem.ncbi.nlm.nih.gov/compound/67697881#section=2D-Structure
https://pubchem.ncbi.nlm.nih.gov/compound/67697455#section=2D-Structure
https://pubchem.ncbi.nlm.nih.gov/compound/67696840#section=2D-Structure
https://pubchem.ncbi.nlm.nih.gov/compound/67695160#section=2D-Structure
https://pubchem.ncbi.nlm.nih.gov/compound/67694163#section=2D-Structure
https://pubchem.ncbi.nlm.nih.gov/compound/67693892#section=2D-Structure
https://pubchem.ncbi.nlm.nih.gov/compound/67690809#section=2D-Structure
https://pubchem.ncbi.nlm.nih.gov/compound/67688592#section=2D-Structure
https://pubchem.ncbi.nlm.nih.gov/compound/67688417#section=2D-Structure
https://pubchem.ncbi.nlm.nih.gov/compound/67674079#section=2D-Structure
https://pubchem.ncbi.nlm.nih.gov/compound/67664085#section=2D-Structure
https://pubchem.ncbi.nlm.nih.gov/compound/67656252#section=2D-Structure
https://pubchem.ncbi.nlm.nih.gov/compound/67655215#section=2D-Structure
https://pubchem.ncbi.nlm.nih.gov/compound/67649809#section=2D-Structure
https://pubchem.ncbi.nlm.nih.gov/compound/67646633#section=2D-Structure
https://pubchem.ncbi.nlm.nih.gov/compound/67645522#section=2D-Structure
https://pubchem.ncbi.nlm.nih.gov/compound/676447#section=2D-Structure
https://pubchem.ncbi.nlm.nih.gov/compound/676446#section=2D-Structure
https://pubchem.ncbi.nlm.nih.gov/compound/67639737#section=2D-Structure
https://pubchem.ncbi.nlm.nih.gov/compound/67634625#section=2D-Structure
https://pubchem.ncbi.nlm.nih.gov/compound/67634624#section=2D-Structure
https://pubchem.ncbi.nlm.nih.gov/compound/67633373#section=2D-Structure
https://pubchem.ncbi.nlm.nih.gov/compound/67632972#section=2D-Structure
https://pubchem.ncbi.nlm.nih.gov/compound/67632717#section=2D-Structure
https://pubchem.ncbi.nlm.nih.gov/compound/67631116#section=2D-Structure
https://pubchem.ncbi.nlm.nih.gov/compound/67626617#section=2D-Structure
https://pubchem.ncbi.nlm.nih.gov/compound/67626183#section=2D-Structure
https://pubchem.ncbi.nlm.nih.gov/compound/67624755#section=2D-Structure
https://pubchem.ncbi.nlm.nih.gov/compound/67618772#section=2D-Structure
https://pubchem.ncbi.nlm.nih.gov/compound/67613036#section=2D-Structure
https://pubchem.ncbi.nlm.nih.gov/compound/67612600#section=2D-Structure
https://pubchem.ncbi.nlm.nih.gov/compound/67610275#section=2D-Structure
https://pubchem.ncbi.nlm.nih.gov/compound/67590922#section=2D-Structure
https://pubchem.ncbi.nlm.nih.gov/compound/67590577#section=2D-Structure
https://pubchem.ncbi.nlm.nih.gov/compound/67582832#section=2D-Structure
https://pubchem.ncbi.nlm.nih.gov/compound/67575187#section=2D-Structure
https://pubchem.ncbi.nlm.nih.gov/compound/67569412#section=2D-Structure
https://pubchem.ncbi.nlm.nih.gov/compound/67559344#section=2D-Structure
https://pubchem.ncbi.nlm.nih.gov/compound/67557331#section=2D-Structure
https://pubchem.ncbi.nlm.nih.gov/compound/67551313#section=2D-Structure
https://pubchem.ncbi.nlm.nih.gov/compound/67534820#section=2D-Structure
https://pubchem.ncbi.nlm.nih.gov/compound/67531248#section=2D-Structure
https://pubchem.ncbi.nlm.nih.gov/compound/67527642#section=2D-Structure
https://pubchem.ncbi.nlm.nih.gov/compound/67522931#section=2D-Structure
https://pubchem.ncbi.nlm.nih.gov/compound/67521265#section=2D-Structure

https://pubchem.ncbi.nlm.nih.gov/compound/67520979#section=2D-Structure
https://pubchem.ncbi.nlm.nih.gov/compound/67520562#section=2D-Structure
https://pubchem.ncbi.nlm.nih.gov/compound/67518544#section=2D-Structure
https://pubchem.ncbi.nlm.nih.gov/compound/67513094#section=2D-Structure
https://pubchem.ncbi.nlm.nih.gov/compound/67504705#section=2D-Structure
https://pubchem.ncbi.nlm.nih.gov/compound/67504704#section=2D-Structure
https://pubchem.ncbi.nlm.nih.gov/compound/67503228#section=2D-Structure
https://pubchem.ncbi.nlm.nih.gov/compound/67490158#section=2D-Structure
https://pubchem.ncbi.nlm.nih.gov/compound/67481290#section=2D-Structure
https://pubchem.ncbi.nlm.nih.gov/compound/67480947#section=2D-Structure
https://pubchem.ncbi.nlm.nih.gov/compound/67474583#section=2D-Structure
https://pubchem.ncbi.nlm.nih.gov/compound/67468141#section=2D-Structure
https://pubchem.ncbi.nlm.nih.gov/compound/67457771#section=2D-Structure
https://pubchem.ncbi.nlm.nih.gov/compound/67456831#section=2D-Structure
https://pubchem.ncbi.nlm.nih.gov/compound/67456382#section=2D-Structure
https://pubchem.ncbi.nlm.nih.gov/compound/67455972#section=2D-Structure
https://pubchem.ncbi.nlm.nih.gov/compound/67455910#section=2D-Structure
https://pubchem.ncbi.nlm.nih.gov/compound/67455620#section=2D-Structure
https://pubchem.ncbi.nlm.nih.gov/compound/67454316#section=2D-Structure
https://pubchem.ncbi.nlm.nih.gov/compound/67452718#section=2D-Structure
https://pubchem.ncbi.nlm.nih.gov/compound/67446981#section=2D-Structure
https://pubchem.ncbi.nlm.nih.gov/compound/67443593#section=2D-Structure
https://pubchem.ncbi.nlm.nih.gov/compound/67440690#section=2D-Structure
https://pubchem.ncbi.nlm.nih.gov/compound/67432473#section=2D-Structure
https://pubchem.ncbi.nlm.nih.gov/compound/67423609#section=2D-Structure
https://pubchem.ncbi.nlm.nih.gov/compound/67420796#section=2D-Structure
https://pubchem.ncbi.nlm.nih.gov/compound/67414241#section=2D-Structure
https://pubchem.ncbi.nlm.nih.gov/compound/67403008#section=2D-Structure
https://pubchem.ncbi.nlm.nih.gov/compound/67401617#section=2D-Structure
https://pubchem.ncbi.nlm.nih.gov/compound/67385062#section=2D-Structure
https://pubchem.ncbi.nlm.nih.gov/compound/67379720#section=2D-Structure
https://pubchem.ncbi.nlm.nih.gov/compound/67378211#section=2D-Structure
https://pubchem.ncbi.nlm.nih.gov/compound/67377596#section=2D-Structure
https://pubchem.ncbi.nlm.nih.gov/compound/673744#section=2D-Structure
https://pubchem.ncbi.nlm.nih.gov/compound/67372541#section=2D-Structure
https://pubchem.ncbi.nlm.nih.gov/compound/67362718#section=2D-Structure
https://pubchem.ncbi.nlm.nih.gov/compound/67351551#section=2D-Structure
https://pubchem.ncbi.nlm.nih.gov/compound/67328964#section=2D-Structure
https://pubchem.ncbi.nlm.nih.gov/compound/67324244#section=2D-Structure
https://pubchem.ncbi.nlm.nih.gov/compound/67311488#section=2D-Structure
https://pubchem.ncbi.nlm.nih.gov/compound/67309399#section=2D-Structure
https://pubchem.ncbi.nlm.nih.gov/compound/67307113#section=2D-Structure
https://pubchem.ncbi.nlm.nih.gov/compound/67302894#section=2D-Structure
https://pubchem.ncbi.nlm.nih.gov/compound/67299026#section=2D-Structure
https://pubchem.ncbi.nlm.nih.gov/compound/67298784#section=2D-Structure
https://pubchem.ncbi.nlm.nih.gov/compound/67284822#section=2D-Structure
https://pubchem.ncbi.nlm.nih.gov/compound/67281666#section=2D-Structure
https://pubchem.ncbi.nlm.nih.gov/compound/67281483#section=2D-Structure
https://pubchem.ncbi.nlm.nih.gov/compound/67279202#section=2D-Structure
https://pubchem.ncbi.nlm.nih.gov/compound/67278870#section=2D-Structure
https://pubchem.ncbi.nlm.nih.gov/compound/67278017#section=2D-Structure
https://pubchem.ncbi.nlm.nih.gov/compound/67278016#section=2D-Structure
https://pubchem.ncbi.nlm.nih.gov/compound/67262045#section=2D-Structure
https://pubchem.ncbi.nlm.nih.gov/compound/67260576#section=2D-Structure
https://pubchem.ncbi.nlm.nih.gov/compound/67260480#section=2D-Structure

https://pubchem.ncbi.nlm.nih.gov/compound/67260177#section=2D-Structure
https://pubchem.ncbi.nlm.nih.gov/compound/67259985#section=2D-Structure
https://pubchem.ncbi.nlm.nih.gov/compound/67259474#section=2D-Structure
https://pubchem.ncbi.nlm.nih.gov/compound/67259194#section=2D-Structure
https://pubchem.ncbi.nlm.nih.gov/compound/67259147#section=2D-Structure
https://pubchem.ncbi.nlm.nih.gov/compound/67258855#section=2D-Structure
https://pubchem.ncbi.nlm.nih.gov/compound/67258838#section=2D-Structure
https://pubchem.ncbi.nlm.nih.gov/compound/67258548#section=2D-Structure
https://pubchem.ncbi.nlm.nih.gov/compound/67258464#section=2D-Structure
https://pubchem.ncbi.nlm.nih.gov/compound/67257757#section=2D-Structure
https://pubchem.ncbi.nlm.nih.gov/compound/67254226#section=2D-Structure
https://pubchem.ncbi.nlm.nih.gov/compound/67245344#section=2D-Structure
https://pubchem.ncbi.nlm.nih.gov/compound/67236650#section=2D-Structure
https://pubchem.ncbi.nlm.nih.gov/compound/67236308#section=2D-Structure
https://pubchem.ncbi.nlm.nih.gov/compound/67216692#section=2D-Structure
https://pubchem.ncbi.nlm.nih.gov/compound/67210489#section=2D-Structure
https://pubchem.ncbi.nlm.nih.gov/compound/67204701#section=2D-Structure
https://pubchem.ncbi.nlm.nih.gov/compound/67202991#section=2D-Structure
https://pubchem.ncbi.nlm.nih.gov/compound/67199051#section=2D-Structure
https://pubchem.ncbi.nlm.nih.gov/compound/67187901#section=2D-Structure
https://pubchem.ncbi.nlm.nih.gov/compound/67176184#section=2D-Structure
https://pubchem.ncbi.nlm.nih.gov/compound/67169610#section=2D-Structure
https://pubchem.ncbi.nlm.nih.gov/compound/67143171#section=2D-Structure
https://pubchem.ncbi.nlm.nih.gov/compound/67135344#section=2D-Structure
https://pubchem.ncbi.nlm.nih.gov/compound/6713158#section=2D-Structure
https://pubchem.ncbi.nlm.nih.gov/compound/67130986#section=2D-Structure
https://pubchem.ncbi.nlm.nih.gov/compound/67127553#section=2D-Structure
https://pubchem.ncbi.nlm.nih.gov/compound/67120228#section=2D-Structure
https://pubchem.ncbi.nlm.nih.gov/compound/67115895#section=2D-Structure
https://pubchem.ncbi.nlm.nih.gov/compound/6711495#section=2D-Structure
https://pubchem.ncbi.nlm.nih.gov/compound/67105956#section=2D-Structure
https://pubchem.ncbi.nlm.nih.gov/compound/67099148#section=2D-Structure
https://pubchem.ncbi.nlm.nih.gov/compound/67094015#section=2D-Structure
https://pubchem.ncbi.nlm.nih.gov/compound/67091661#section=2D-Structure
https://pubchem.ncbi.nlm.nih.gov/compound/67084015#section=2D-Structure
https://pubchem.ncbi.nlm.nih.gov/compound/67071254#section=2D-Structure
https://pubchem.ncbi.nlm.nih.gov/compound/67069002#section=2D-Structure
https://pubchem.ncbi.nlm.nih.gov/compound/67059706#section=2D-Structure
https://pubchem.ncbi.nlm.nih.gov/compound/67058501#section=2D-Structure
https://pubchem.ncbi.nlm.nih.gov/compound/67057785#section=2D-Structure
https://pubchem.ncbi.nlm.nih.gov/compound/67054642#section=2D-Structure
https://pubchem.ncbi.nlm.nih.gov/compound/67048502#section=2D-Structure
https://pubchem.ncbi.nlm.nih.gov/compound/67039107#section=2D-Structure
https://pubchem.ncbi.nlm.nih.gov/compound/67030130#section=2D-Structure
https://pubchem.ncbi.nlm.nih.gov/compound/67023850#section=2D-Structure
https://pubchem.ncbi.nlm.nih.gov/compound/67022850#section=2D-Structure
https://pubchem.ncbi.nlm.nih.gov/compound/66996878#section=2D-Structure
https://pubchem.ncbi.nlm.nih.gov/compound/66988410#section=2D-Structure
https://pubchem.ncbi.nlm.nih.gov/compound/66982928#section=2D-Structure
https://pubchem.ncbi.nlm.nih.gov/compound/66980009#section=2D-Structure
https://pubchem.ncbi.nlm.nih.gov/compound/66979217#section=2D-Structure
https://pubchem.ncbi.nlm.nih.gov/compound/66978486#section=2D-Structure
https://pubchem.ncbi.nlm.nih.gov/compound/66976705#section=2D-Structure
https://pubchem.ncbi.nlm.nih.gov/compound/66969644#section=2D-Structure
https://pubchem.ncbi.nlm.nih.gov/compound/66968229#section=2D-Structure

https://pubchem.ncbi.nlm.nih.gov/compound/66958672#section=2D-Structure
https://pubchem.ncbi.nlm.nih.gov/compound/66958645#section=2D-Structure
https://pubchem.ncbi.nlm.nih.gov/compound/66933389#section=2D-Structure
https://pubchem.ncbi.nlm.nih.gov/compound/66933239#section=2D-Structure
https://pubchem.ncbi.nlm.nih.gov/compound/66926121#section=2D-Structure
https://pubchem.ncbi.nlm.nih.gov/compound/66919674#section=2D-Structure
https://pubchem.ncbi.nlm.nih.gov/compound/66916701#section=2D-Structure
https://pubchem.ncbi.nlm.nih.gov/compound/66911587#section=2D-Structure
https://pubchem.ncbi.nlm.nih.gov/compound/66911586#section=2D-Structure
https://pubchem.ncbi.nlm.nih.gov/compound/66911418#section=2D-Structure
https://pubchem.ncbi.nlm.nih.gov/compound/66908635#section=2D-Structure
https://pubchem.ncbi.nlm.nih.gov/compound/66905781#section=2D-Structure
https://pubchem.ncbi.nlm.nih.gov/compound/66898255#section=2D-Structure
https://pubchem.ncbi.nlm.nih.gov/compound/66888895#section=2D-Structure
https://pubchem.ncbi.nlm.nih.gov/compound/66886825#section=2D-Structure
https://pubchem.ncbi.nlm.nih.gov/compound/66886359#section=2D-Structure
https://pubchem.ncbi.nlm.nih.gov/compound/66883578#section=2D-Structure
https://pubchem.ncbi.nlm.nih.gov/compound/66873938#section=2D-Structure
https://pubchem.ncbi.nlm.nih.gov/compound/66873478#section=2D-Structure
https://pubchem.ncbi.nlm.nih.gov/compound/66873283#section=2D-Structure
https://pubchem.ncbi.nlm.nih.gov/compound/66872699#section=2D-Structure
https://pubchem.ncbi.nlm.nih.gov/compound/66872597#section=2D-Structure
https://pubchem.ncbi.nlm.nih.gov/compound/66868347#section=2D-Structure
https://pubchem.ncbi.nlm.nih.gov/compound/66842015#section=2D-Structure
https://pubchem.ncbi.nlm.nih.gov/compound/66841971#section=2D-Structure
https://pubchem.ncbi.nlm.nih.gov/compound/66841354#section=2D-Structure
https://pubchem.ncbi.nlm.nih.gov/compound/66838086#section=2D-Structure
https://pubchem.ncbi.nlm.nih.gov/compound/66826493#section=2D-Structure
https://pubchem.ncbi.nlm.nih.gov/compound/66825836#section=2D-Structure
https://pubchem.ncbi.nlm.nih.gov/compound/66823966#section=2D-Structure
https://pubchem.ncbi.nlm.nih.gov/compound/66823950#section=2D-Structure
https://pubchem.ncbi.nlm.nih.gov/compound/66823512#section=2D-Structure
https://pubchem.ncbi.nlm.nih.gov/compound/66806135#section=2D-Structure
https://pubchem.ncbi.nlm.nih.gov/compound/66793103#section=2D-Structure
https://pubchem.ncbi.nlm.nih.gov/compound/66790732#section=2D-Structure
https://pubchem.ncbi.nlm.nih.gov/compound/66788165#section=2D-Structure
https://pubchem.ncbi.nlm.nih.gov/compound/66787659#section=2D-Structure
https://pubchem.ncbi.nlm.nih.gov/compound/66769975#section=2D-Structure
https://pubchem.ncbi.nlm.nih.gov/compound/66764726#section=2D-Structure
https://pubchem.ncbi.nlm.nih.gov/compound/66763871#section=2D-Structure
https://pubchem.ncbi.nlm.nih.gov/compound/667632#section=2D-Structure
https://pubchem.ncbi.nlm.nih.gov/compound/66749275#section=2D-Structure
https://pubchem.ncbi.nlm.nih.gov/compound/66746910#section=2D-Structure
https://pubchem.ncbi.nlm.nih.gov/compound/66734115#section=2D-Structure
https://pubchem.ncbi.nlm.nih.gov/compound/66733499#section=2D-Structure
https://pubchem.ncbi.nlm.nih.gov/compound/66726955#section=2D-Structure
https://pubchem.ncbi.nlm.nih.gov/compound/66719001#section=2D-Structure
https://pubchem.ncbi.nlm.nih.gov/compound/66718456#section=2D-Structure
https://pubchem.ncbi.nlm.nih.gov/compound/66717889#section=2D-Structure
https://pubchem.ncbi.nlm.nih.gov/compound/66715966#section=2D-Structure
https://pubchem.ncbi.nlm.nih.gov/compound/66711717#section=2D-Structure
https://pubchem.ncbi.nlm.nih.gov/compound/66700952#section=2D-Structure
https://pubchem.ncbi.nlm.nih.gov/compound/66700294#section=2D-Structure
https://pubchem.ncbi.nlm.nih.gov/compound/66700290#section=2D-Structure
https://pubchem.ncbi.nlm.nih.gov/compound/66697549#section=2D-Structure

https://pubchem.ncbi.nlm.nih.gov/compound/66694480#section=2D-Structure
https://pubchem.ncbi.nlm.nih.gov/compound/66694476#section=2D-Structure
https://pubchem.ncbi.nlm.nih.gov/compound/66690157#section=2D-Structure
https://pubchem.ncbi.nlm.nih.gov/compound/66690042#section=2D-Structure
https://pubchem.ncbi.nlm.nih.gov/compound/66689934#section=2D-Structure
https://pubchem.ncbi.nlm.nih.gov/compound/66689254#section=2D-Structure
https://pubchem.ncbi.nlm.nih.gov/compound/66686218#section=2D-Structure
https://pubchem.ncbi.nlm.nih.gov/compound/66679879#section=2D-Structure
https://pubchem.ncbi.nlm.nih.gov/compound/66671349#section=2D-Structure
https://pubchem.ncbi.nlm.nih.gov/compound/66668822#section=2D-Structure
https://pubchem.ncbi.nlm.nih.gov/compound/66668100#section=2D-Structure
https://pubchem.ncbi.nlm.nih.gov/compound/66666119#section=2D-Structure
https://pubchem.ncbi.nlm.nih.gov/compound/66665757#section=2D-Structure
https://pubchem.ncbi.nlm.nih.gov/compound/66654704#section=2D-Structure
https://pubchem.ncbi.nlm.nih.gov/compound/66654654#section=2D-Structure
https://pubchem.ncbi.nlm.nih.gov/compound/66650381#section=2D-Structure
https://pubchem.ncbi.nlm.nih.gov/compound/66649818#section=2D-Structure
https://pubchem.ncbi.nlm.nih.gov/compound/66642716#section=2D-Structure
https://pubchem.ncbi.nlm.nih.gov/compound/66641512#section=2D-Structure
https://pubchem.ncbi.nlm.nih.gov/compound/66641490#section=2D-Structure
https://pubchem.ncbi.nlm.nih.gov/compound/66641479#section=2D-Structure
https://pubchem.ncbi.nlm.nih.gov/compound/66633359#section=2D-Structure
https://pubchem.ncbi.nlm.nih.gov/compound/66624235#section=2D-Structure
https://pubchem.ncbi.nlm.nih.gov/compound/66623719#section=2D-Structure
https://pubchem.ncbi.nlm.nih.gov/compound/66623496#section=2D-Structure
https://pubchem.ncbi.nlm.nih.gov/compound/66616202#section=2D-Structure
https://pubchem.ncbi.nlm.nih.gov/compound/66606603#section=2D-Structure
https://pubchem.ncbi.nlm.nih.gov/compound/66602201#section=2D-Structure
https://pubchem.ncbi.nlm.nih.gov/compound/66589681#section=2D-Structure
https://pubchem.ncbi.nlm.nih.gov/compound/66588839#section=2D-Structure
https://pubchem.ncbi.nlm.nih.gov/compound/66584625#section=2D-Structure
https://pubchem.ncbi.nlm.nih.gov/compound/66583170#section=2D-Structure
https://pubchem.ncbi.nlm.nih.gov/compound/66577304#section=2D-Structure
https://pubchem.ncbi.nlm.nih.gov/compound/66575536#section=2D-Structure
https://pubchem.ncbi.nlm.nih.gov/compound/66489962#section=2D-Structure
https://pubchem.ncbi.nlm.nih.gov/compound/658543#section=2D-Structure
https://pubchem.ncbi.nlm.nih.gov/compound/657611#section=2D-Structure
https://pubchem.ncbi.nlm.nih.gov/compound/65335741#section=2D-Structure
https://pubchem.ncbi.nlm.nih.gov/compound/6511533#section=2D-Structure
https://pubchem.ncbi.nlm.nih.gov/compound/65044#section=2D-Structure
https://pubchem.ncbi.nlm.nih.gov/compound/6453553#section=2D-Structure
https://pubchem.ncbi.nlm.nih.gov/compound/6451530#section=2D-Structure
https://pubchem.ncbi.nlm.nih.gov/compound/6451439#section=2D-Structure
https://pubchem.ncbi.nlm.nih.gov/compound/6451412#section=2D-Structure
https://pubchem.ncbi.nlm.nih.gov/compound/6451388#section=2D-Structure
https://pubchem.ncbi.nlm.nih.gov/compound/64468#section=2D-Structure
https://pubchem.ncbi.nlm.nih.gov/compound/643548#section=2D-Structure
https://pubchem.ncbi.nlm.nih.gov/compound/6430130#section=2D-Structure
https://pubchem.ncbi.nlm.nih.gov/compound/6418401#section=2D-Structure
https://pubchem.ncbi.nlm.nih.gov/compound/6413040#section=2D-Structure
https://pubchem.ncbi.nlm.nih.gov/compound/6411549#section=2D-Structure
https://pubchem.ncbi.nlm.nih.gov/compound/6410858#section=2D-Structure
https://pubchem.ncbi.nlm.nih.gov/compound/6404763#section=2D-Structure
https://pubchem.ncbi.nlm.nih.gov/compound/6401665#section=2D-Structure
https://pubchem.ncbi.nlm.nih.gov/compound/6395985#section=2D-Structure

https://pubchem.ncbi.nlm.nih.gov/compound/639380#section=2D-Structure
https://pubchem.ncbi.nlm.nih.gov/compound/639361#section=2D-Structure
https://pubchem.ncbi.nlm.nih.gov/compound/6390498#section=2D-Structure
https://pubchem.ncbi.nlm.nih.gov/compound/638710#section=2D-Structure
https://pubchem.ncbi.nlm.nih.gov/compound/6312004#section=2D-Structure
https://pubchem.ncbi.nlm.nih.gov/compound/62785340#section=2D-Structure
https://pubchem.ncbi.nlm.nih.gov/compound/625410#section=2D-Structure
https://pubchem.ncbi.nlm.nih.gov/compound/621615#section=2D-Structure
https://pubchem.ncbi.nlm.nih.gov/compound/620812#section=2D-Structure
https://pubchem.ncbi.nlm.nih.gov/compound/620168#section=2D-Structure
https://pubchem.ncbi.nlm.nih.gov/compound/620167#section=2D-Structure
https://pubchem.ncbi.nlm.nih.gov/compound/619337#section=2D-Structure
https://pubchem.ncbi.nlm.nih.gov/compound/619135#section=2D-Structure
https://pubchem.ncbi.nlm.nih.gov/compound/617803#section=2D-Structure
https://pubchem.ncbi.nlm.nih.gov/compound/6169276#section=2D-Structure
https://pubchem.ncbi.nlm.nih.gov/compound/616740#section=2D-Structure
https://pubchem.ncbi.nlm.nih.gov/compound/616047#section=2D-Structure
https://pubchem.ncbi.nlm.nih.gov/compound/615243#section=2D-Structure
https://pubchem.ncbi.nlm.nih.gov/compound/615165#section=2D-Structure
https://pubchem.ncbi.nlm.nih.gov/compound/614888#section=2D-Structure
https://pubchem.ncbi.nlm.nih.gov/compound/614473#section=2D-Structure
https://pubchem.ncbi.nlm.nih.gov/compound/613659#section=2D-Structure
https://pubchem.ncbi.nlm.nih.gov/compound/610909#section=2D-Structure
https://pubchem.ncbi.nlm.nih.gov/compound/6105970#section=2D-Structure
https://pubchem.ncbi.nlm.nih.gov/compound/610421#section=2D-Structure
https://pubchem.ncbi.nlm.nih.gov/compound/6102399#section=2D-Structure
https://pubchem.ncbi.nlm.nih.gov/compound/610127#section=2D-Structure
https://pubchem.ncbi.nlm.nih.gov/compound/609888#section=2D-Structure
https://pubchem.ncbi.nlm.nih.gov/compound/6095736#section=2D-Structure
https://pubchem.ncbi.nlm.nih.gov/compound/609007#section=2D-Structure
https://pubchem.ncbi.nlm.nih.gov/compound/608500#section=2D-Structure
https://pubchem.ncbi.nlm.nih.gov/compound/608329#section=2D-Structure
https://pubchem.ncbi.nlm.nih.gov/compound/607269#section=2D-Structure
https://pubchem.ncbi.nlm.nih.gov/compound/607187#section=2D-Structure
https://pubchem.ncbi.nlm.nih.gov/compound/607182#section=2D-Structure
https://pubchem.ncbi.nlm.nih.gov/compound/606303#section=2D-Structure
https://pubchem.ncbi.nlm.nih.gov/compound/605873#section=2D-Structure
https://pubchem.ncbi.nlm.nih.gov/compound/605796#section=2D-Structure
https://pubchem.ncbi.nlm.nih.gov/compound/605448#section=2D-Structure
https://pubchem.ncbi.nlm.nih.gov/compound/605377#section=2D-Structure
https://pubchem.ncbi.nlm.nih.gov/compound/605334#section=2D-Structure
https://pubchem.ncbi.nlm.nih.gov/compound/605303#section=2D-Structure
https://pubchem.ncbi.nlm.nih.gov/compound/605263#section=2D-Structure
https://pubchem.ncbi.nlm.nih.gov/compound/604125#section=2D-Structure
https://pubchem.ncbi.nlm.nih.gov/compound/603531#section=2D-Structure
https://pubchem.ncbi.nlm.nih.gov/compound/602830#section=2D-Structure
https://pubchem.ncbi.nlm.nih.gov/compound/602674#section=2D-Structure
https://pubchem.ncbi.nlm.nih.gov/compound/602524#section=2D-Structure
https://pubchem.ncbi.nlm.nih.gov/compound/602238#section=2D-Structure
https://pubchem.ncbi.nlm.nih.gov/compound/60205840#section=2D-Structure
https://pubchem.ncbi.nlm.nih.gov/compound/601975#section=2D-Structure
https://pubchem.ncbi.nlm.nih.gov/compound/601894#section=2D-Structure
https://pubchem.ncbi.nlm.nih.gov/compound/601892#section=2D-Structure
https://pubchem.ncbi.nlm.nih.gov/compound/601856#section=2D-Structure
https://pubchem.ncbi.nlm.nih.gov/compound/60168190#section=2D-Structure

https://pubchem.ncbi.nlm.nih.gov/compound/60160793#section=2D-Structure
https://pubchem.ncbi.nlm.nih.gov/compound/60145843#section=2D-Structure
https://pubchem.ncbi.nlm.nih.gov/compound/60138572#section=2D-Structure
https://pubchem.ncbi.nlm.nih.gov/compound/60133598#section=2D-Structure
https://pubchem.ncbi.nlm.nih.gov/compound/60133597#section=2D-Structure
https://pubchem.ncbi.nlm.nih.gov/compound/60132549#section=2D-Structure
https://pubchem.ncbi.nlm.nih.gov/compound/60123831#section=2D-Structure
https://pubchem.ncbi.nlm.nih.gov/compound/60119342#section=2D-Structure
https://pubchem.ncbi.nlm.nih.gov/compound/60116285#section=2D-Structure
https://pubchem.ncbi.nlm.nih.gov/compound/60103367#section=2D-Structure
https://pubchem.ncbi.nlm.nih.gov/compound/60100179#section=2D-Structure
https://pubchem.ncbi.nlm.nih.gov/compound/60079321#section=2D-Structure
https://pubchem.ncbi.nlm.nih.gov/compound/600594#section=2D-Structure
https://pubchem.ncbi.nlm.nih.gov/compound/60057473#section=2D-Structure
https://pubchem.ncbi.nlm.nih.gov/compound/60055069#section=2D-Structure
https://pubchem.ncbi.nlm.nih.gov/compound/60042392#section=2D-Structure
https://pubchem.ncbi.nlm.nih.gov/compound/60042307#section=2D-Structure
https://pubchem.ncbi.nlm.nih.gov/compound/60033551#section=2D-Structure
https://pubchem.ncbi.nlm.nih.gov/compound/60012342#section=2D-Structure
https://pubchem.ncbi.nlm.nih.gov/compound/60011151#section=2D-Structure
https://pubchem.ncbi.nlm.nih.gov/compound/60001234#section=2D-Structure
https://pubchem.ncbi.nlm.nih.gov/compound/59990691#section=2D-Structure
https://pubchem.ncbi.nlm.nih.gov/compound/59990690#section=2D-Structure
https://pubchem.ncbi.nlm.nih.gov/compound/59989238#section=2D-Structure
https://pubchem.ncbi.nlm.nih.gov/compound/59950572#section=2D-Structure
https://pubchem.ncbi.nlm.nih.gov/compound/59946653#section=2D-Structure
https://pubchem.ncbi.nlm.nih.gov/compound/59946640#section=2D-Structure
https://pubchem.ncbi.nlm.nih.gov/compound/59944755#section=2D-Structure
https://pubchem.ncbi.nlm.nih.gov/compound/59944751#section=2D-Structure
https://pubchem.ncbi.nlm.nih.gov/compound/59944708#section=2D-Structure
https://pubchem.ncbi.nlm.nih.gov/compound/59944321#section=2D-Structure
https://pubchem.ncbi.nlm.nih.gov/compound/59944320#section=2D-Structure
https://pubchem.ncbi.nlm.nih.gov/compound/59944319#section=2D-Structure
https://pubchem.ncbi.nlm.nih.gov/compound/59944314#section=2D-Structure
https://pubchem.ncbi.nlm.nih.gov/compound/59944313#section=2D-Structure
https://pubchem.ncbi.nlm.nih.gov/compound/59944311#section=2D-Structure
https://pubchem.ncbi.nlm.nih.gov/compound/59944310#section=2D-Structure
https://pubchem.ncbi.nlm.nih.gov/compound/59944306#section=2D-Structure
https://pubchem.ncbi.nlm.nih.gov/compound/59944305#section=2D-Structure
https://pubchem.ncbi.nlm.nih.gov/compound/59944303#section=2D-Structure
https://pubchem.ncbi.nlm.nih.gov/compound/59944301#section=2D-Structure
https://pubchem.ncbi.nlm.nih.gov/compound/59944300#section=2D-Structure
https://pubchem.ncbi.nlm.nih.gov/compound/59944297#section=2D-Structure
https://pubchem.ncbi.nlm.nih.gov/compound/59944296#section=2D-Structure
https://pubchem.ncbi.nlm.nih.gov/compound/59944295#section=2D-Structure
https://pubchem.ncbi.nlm.nih.gov/compound/59944294#section=2D-Structure
https://pubchem.ncbi.nlm.nih.gov/compound/59944292#section=2D-Structure
https://pubchem.ncbi.nlm.nih.gov/compound/59944290#section=2D-Structure
https://pubchem.ncbi.nlm.nih.gov/compound/59944288#section=2D-Structure
https://pubchem.ncbi.nlm.nih.gov/compound/59944286#section=2D-Structure
https://pubchem.ncbi.nlm.nih.gov/compound/59944282#section=2D-Structure
https://pubchem.ncbi.nlm.nih.gov/compound/59944281#section=2D-Structure
https://pubchem.ncbi.nlm.nih.gov/compound/59944280#section=2D-Structure
https://pubchem.ncbi.nlm.nih.gov/compound/59944279#section=2D-Structure
https://pubchem.ncbi.nlm.nih.gov/compound/59944278#section=2D-Structure

https://pubchem.ncbi.nlm.nih.gov/compound/59944275#section=2D-Structure
https://pubchem.ncbi.nlm.nih.gov/compound/59944272#section=2D-Structure
https://pubchem.ncbi.nlm.nih.gov/compound/59944268#section=2D-Structure
https://pubchem.ncbi.nlm.nih.gov/compound/59944267#section=2D-Structure
https://pubchem.ncbi.nlm.nih.gov/compound/59944264#section=2D-Structure
https://pubchem.ncbi.nlm.nih.gov/compound/59944263#section=2D-Structure
https://pubchem.ncbi.nlm.nih.gov/compound/59944261#section=2D-Structure
https://pubchem.ncbi.nlm.nih.gov/compound/59944260#section=2D-Structure
https://pubchem.ncbi.nlm.nih.gov/compound/59944258#section=2D-Structure
https://pubchem.ncbi.nlm.nih.gov/compound/59944257#section=2D-Structure
https://pubchem.ncbi.nlm.nih.gov/compound/59944256#section=2D-Structure
https://pubchem.ncbi.nlm.nih.gov/compound/59944255#section=2D-Structure
https://pubchem.ncbi.nlm.nih.gov/compound/59944249#section=2D-Structure
https://pubchem.ncbi.nlm.nih.gov/compound/59944248#section=2D-Structure
https://pubchem.ncbi.nlm.nih.gov/compound/59944247#section=2D-Structure
https://pubchem.ncbi.nlm.nih.gov/compound/59944246#section=2D-Structure
https://pubchem.ncbi.nlm.nih.gov/compound/59944244#section=2D-Structure
https://pubchem.ncbi.nlm.nih.gov/compound/59944243#section=2D-Structure
https://pubchem.ncbi.nlm.nih.gov/compound/59944241#section=2D-Structure
https://pubchem.ncbi.nlm.nih.gov/compound/59944239#section=2D-Structure
https://pubchem.ncbi.nlm.nih.gov/compound/59944236#section=2D-Structure
https://pubchem.ncbi.nlm.nih.gov/compound/59944235#section=2D-Structure
https://pubchem.ncbi.nlm.nih.gov/compound/59944234#section=2D-Structure
https://pubchem.ncbi.nlm.nih.gov/compound/59944233#section=2D-Structure
https://pubchem.ncbi.nlm.nih.gov/compound/59944232#section=2D-Structure
https://pubchem.ncbi.nlm.nih.gov/compound/59944231#section=2D-Structure
https://pubchem.ncbi.nlm.nih.gov/compound/59944230#section=2D-Structure
https://pubchem.ncbi.nlm.nih.gov/compound/59944229#section=2D-Structure
https://pubchem.ncbi.nlm.nih.gov/compound/59944227#section=2D-Structure
https://pubchem.ncbi.nlm.nih.gov/compound/59944226#section=2D-Structure
https://pubchem.ncbi.nlm.nih.gov/compound/59944224#section=2D-Structure
https://pubchem.ncbi.nlm.nih.gov/compound/59944223#section=2D-Structure
https://pubchem.ncbi.nlm.nih.gov/compound/59944222#section=2D-Structure
https://pubchem.ncbi.nlm.nih.gov/compound/59944220#section=2D-Structure
https://pubchem.ncbi.nlm.nih.gov/compound/59944218#section=2D-Structure
https://pubchem.ncbi.nlm.nih.gov/compound/59944217#section=2D-Structure
https://pubchem.ncbi.nlm.nih.gov/compound/59944215#section=2D-Structure
https://pubchem.ncbi.nlm.nih.gov/compound/59944213#section=2D-Structure
https://pubchem.ncbi.nlm.nih.gov/compound/59944212#section=2D-Structure
https://pubchem.ncbi.nlm.nih.gov/compound/59944210#section=2D-Structure
https://pubchem.ncbi.nlm.nih.gov/compound/59944209#section=2D-Structure
https://pubchem.ncbi.nlm.nih.gov/compound/59944208#section=2D-Structure
https://pubchem.ncbi.nlm.nih.gov/compound/59944207#section=2D-Structure
https://pubchem.ncbi.nlm.nih.gov/compound/59944203#section=2D-Structure
https://pubchem.ncbi.nlm.nih.gov/compound/59944202#section=2D-Structure
https://pubchem.ncbi.nlm.nih.gov/compound/59944198#section=2D-Structure
https://pubchem.ncbi.nlm.nih.gov/compound/59944193#section=2D-Structure
https://pubchem.ncbi.nlm.nih.gov/compound/59944192#section=2D-Structure
https://pubchem.ncbi.nlm.nih.gov/compound/59944190#section=2D-Structure
https://pubchem.ncbi.nlm.nih.gov/compound/59944189#section=2D-Structure
https://pubchem.ncbi.nlm.nih.gov/compound/59944188#section=2D-Structure
https://pubchem.ncbi.nlm.nih.gov/compound/59944186#section=2D-Structure
https://pubchem.ncbi.nlm.nih.gov/compound/59944185#section=2D-Structure
https://pubchem.ncbi.nlm.nih.gov/compound/59942537#section=2D-Structure
https://pubchem.ncbi.nlm.nih.gov/compound/59941273#section=2D-Structure

https://pubchem.ncbi.nlm.nih.gov/compound/59931163#section=2D-Structure
https://pubchem.ncbi.nlm.nih.gov/compound/59931162#section=2D-Structure
https://pubchem.ncbi.nlm.nih.gov/compound/59925714#section=2D-Structure
https://pubchem.ncbi.nlm.nih.gov/compound/59910305#section=2D-Structure
https://pubchem.ncbi.nlm.nih.gov/compound/59903291#section=2D-Structure
https://pubchem.ncbi.nlm.nih.gov/compound/59894081#section=2D-Structure
https://pubchem.ncbi.nlm.nih.gov/compound/59879101#section=2D-Structure
https://pubchem.ncbi.nlm.nih.gov/compound/59873754#section=2D-Structure
https://pubchem.ncbi.nlm.nih.gov/compound/598703#section=2D-Structure
https://pubchem.ncbi.nlm.nih.gov/compound/59861135#section=2D-Structure
https://pubchem.ncbi.nlm.nih.gov/compound/59858066#section=2D-Structure
https://pubchem.ncbi.nlm.nih.gov/compound/59854148#section=2D-Structure
https://pubchem.ncbi.nlm.nih.gov/compound/59854132#section=2D-Structure
https://pubchem.ncbi.nlm.nih.gov/compound/598529#section=2D-Structure
https://pubchem.ncbi.nlm.nih.gov/compound/59847941#section=2D-Structure
https://pubchem.ncbi.nlm.nih.gov/compound/59844085#section=2D-Structure
https://pubchem.ncbi.nlm.nih.gov/compound/598389#section=2D-Structure
https://pubchem.ncbi.nlm.nih.gov/compound/59827482#section=2D-Structure
https://pubchem.ncbi.nlm.nih.gov/compound/59827478#section=2D-Structure
https://pubchem.ncbi.nlm.nih.gov/compound/59827473#section=2D-Structure
https://pubchem.ncbi.nlm.nih.gov/compound/59827160#section=2D-Structure
https://pubchem.ncbi.nlm.nih.gov/compound/59827158#section=2D-Structure
https://pubchem.ncbi.nlm.nih.gov/compound/59816805#section=2D-Structure
https://pubchem.ncbi.nlm.nih.gov/compound/59815343#section=2D-Structure
https://pubchem.ncbi.nlm.nih.gov/compound/59811841#section=2D-Structure
https://pubchem.ncbi.nlm.nih.gov/compound/59811840#section=2D-Structure
https://pubchem.ncbi.nlm.nih.gov/compound/59811821#section=2D-Structure
https://pubchem.ncbi.nlm.nih.gov/compound/59811818#section=2D-Structure
https://pubchem.ncbi.nlm.nih.gov/compound/59811817#section=2D-Structure
https://pubchem.ncbi.nlm.nih.gov/compound/59789008#section=2D-Structure
https://pubchem.ncbi.nlm.nih.gov/compound/59779930#section=2D-Structure
https://pubchem.ncbi.nlm.nih.gov/compound/59775750#section=2D-Structure
https://pubchem.ncbi.nlm.nih.gov/compound/59775748#section=2D-Structure
https://pubchem.ncbi.nlm.nih.gov/compound/59775747#section=2D-Structure
https://pubchem.ncbi.nlm.nih.gov/compound/59775746#section=2D-Structure
https://pubchem.ncbi.nlm.nih.gov/compound/59766474#section=2D-Structure
https://pubchem.ncbi.nlm.nih.gov/compound/597611#section=2D-Structure
https://pubchem.ncbi.nlm.nih.gov/compound/59760795#section=2D-Structure
https://pubchem.ncbi.nlm.nih.gov/compound/59760794#section=2D-Structure
https://pubchem.ncbi.nlm.nih.gov/compound/59760726#section=2D-Structure
https://pubchem.ncbi.nlm.nih.gov/compound/59760725#section=2D-Structure
https://pubchem.ncbi.nlm.nih.gov/compound/59760723#section=2D-Structure
https://pubchem.ncbi.nlm.nih.gov/compound/597568#section=2D-Structure
https://pubchem.ncbi.nlm.nih.gov/compound/59752049#section=2D-Structure
https://pubchem.ncbi.nlm.nih.gov/compound/59747980#section=2D-Structure
https://pubchem.ncbi.nlm.nih.gov/compound/59746597#section=2D-Structure
https://pubchem.ncbi.nlm.nih.gov/compound/59712568#section=2D-Structure
https://pubchem.ncbi.nlm.nih.gov/compound/59696063#section=2D-Structure
https://pubchem.ncbi.nlm.nih.gov/compound/596893#section=2D-Structure
https://pubchem.ncbi.nlm.nih.gov/compound/59650217#section=2D-Structure
https://pubchem.ncbi.nlm.nih.gov/compound/59650214#section=2D-Structure
https://pubchem.ncbi.nlm.nih.gov/compound/59646223#section=2D-Structure
https://pubchem.ncbi.nlm.nih.gov/compound/59646218#section=2D-Structure
https://pubchem.ncbi.nlm.nih.gov/compound/59601156#section=2D-Structure
https://pubchem.ncbi.nlm.nih.gov/compound/59601148#section=2D-Structure

https://pubchem.ncbi.nlm.nih.gov/compound/59601138#section=2D-Structure
https://pubchem.ncbi.nlm.nih.gov/compound/59601122#section=2D-Structure
https://pubchem.ncbi.nlm.nih.gov/compound/59601114#section=2D-Structure
https://pubchem.ncbi.nlm.nih.gov/compound/59601112#section=2D-Structure
https://pubchem.ncbi.nlm.nih.gov/compound/59599969#section=2D-Structure
https://pubchem.ncbi.nlm.nih.gov/compound/59599967#section=2D-Structure
https://pubchem.ncbi.nlm.nih.gov/compound/59599965#section=2D-Structure
https://pubchem.ncbi.nlm.nih.gov/compound/59599963#section=2D-Structure
https://pubchem.ncbi.nlm.nih.gov/compound/59599957#section=2D-Structure
https://pubchem.ncbi.nlm.nih.gov/compound/59593609#section=2D-Structure
https://pubchem.ncbi.nlm.nih.gov/compound/59575046#section=2D-Structure
https://pubchem.ncbi.nlm.nih.gov/compound/59574229#section=2D-Structure
https://pubchem.ncbi.nlm.nih.gov/compound/59566416#section=2D-Structure
https://pubchem.ncbi.nlm.nih.gov/compound/59563902#section=2D-Structure
https://pubchem.ncbi.nlm.nih.gov/compound/59554035#section=2D-Structure
https://pubchem.ncbi.nlm.nih.gov/compound/595540#section=2D-Structure
https://pubchem.ncbi.nlm.nih.gov/compound/5955#section=2D-Structure
https://pubchem.ncbi.nlm.nih.gov/compound/595234#section=2D-Structure
https://pubchem.ncbi.nlm.nih.gov/compound/59516802#section=2D-Structure
https://pubchem.ncbi.nlm.nih.gov/compound/59497979#section=2D-Structure
https://pubchem.ncbi.nlm.nih.gov/compound/59491924#section=2D-Structure
https://pubchem.ncbi.nlm.nih.gov/compound/594769#section=2D-Structure
https://pubchem.ncbi.nlm.nih.gov/compound/59476722#section=2D-Structure
https://pubchem.ncbi.nlm.nih.gov/compound/59471196#section=2D-Structure
https://pubchem.ncbi.nlm.nih.gov/compound/59471195#section=2D-Structure
https://pubchem.ncbi.nlm.nih.gov/compound/59467405#section=2D-Structure
https://pubchem.ncbi.nlm.nih.gov/compound/59457696#section=2D-Structure
https://pubchem.ncbi.nlm.nih.gov/compound/59450478#section=2D-Structure
https://pubchem.ncbi.nlm.nih.gov/compound/59435156#section=2D-Structure
https://pubchem.ncbi.nlm.nih.gov/compound/59434951#section=2D-Structure
https://pubchem.ncbi.nlm.nih.gov/compound/59422636#section=2D-Structure
https://pubchem.ncbi.nlm.nih.gov/compound/59422634#section=2D-Structure
https://pubchem.ncbi.nlm.nih.gov/compound/59422630#section=2D-Structure
https://pubchem.ncbi.nlm.nih.gov/compound/59422624#section=2D-Structure
https://pubchem.ncbi.nlm.nih.gov/compound/59407999#section=2D-Structure
https://pubchem.ncbi.nlm.nih.gov/compound/59403579#section=2D-Structure
https://pubchem.ncbi.nlm.nih.gov/compound/59401339#section=2D-Structure
https://pubchem.ncbi.nlm.nih.gov/compound/59360853#section=2D-Structure
https://pubchem.ncbi.nlm.nih.gov/compound/59350047#section=2D-Structure
https://pubchem.ncbi.nlm.nih.gov/compound/59336295#section=2D-Structure
https://pubchem.ncbi.nlm.nih.gov/compound/59336290#section=2D-Structure
https://pubchem.ncbi.nlm.nih.gov/compound/59334145#section=2D-Structure
https://pubchem.ncbi.nlm.nih.gov/compound/59302059#section=2D-Structure
https://pubchem.ncbi.nlm.nih.gov/compound/592994#section=2D-Structure
https://pubchem.ncbi.nlm.nih.gov/compound/59284286#section=2D-Structure
https://pubchem.ncbi.nlm.nih.gov/compound/59283379#section=2D-Structure
https://pubchem.ncbi.nlm.nih.gov/compound/59274943#section=2D-Structure
https://pubchem.ncbi.nlm.nih.gov/compound/59256862#section=2D-Structure
https://pubchem.ncbi.nlm.nih.gov/compound/59236030#section=2D-Structure
https://pubchem.ncbi.nlm.nih.gov/compound/59236018#section=2D-Structure
https://pubchem.ncbi.nlm.nih.gov/compound/59235947#section=2D-Structure
https://pubchem.ncbi.nlm.nih.gov/compound/59235938#section=2D-Structure
https://pubchem.ncbi.nlm.nih.gov/compound/59227472#section=2D-Structure
https://pubchem.ncbi.nlm.nih.gov/compound/59207613#section=2D-Structure
https://pubchem.ncbi.nlm.nih.gov/compound/59165310#section=2D-Structure

https://pubchem.ncbi.nlm.nih.gov/compound/591590#section=2D-Structure
https://pubchem.ncbi.nlm.nih.gov/compound/59145901#section=2D-Structure
https://pubchem.ncbi.nlm.nih.gov/compound/59145891#section=2D-Structure
https://pubchem.ncbi.nlm.nih.gov/compound/591366#section=2D-Structure
https://pubchem.ncbi.nlm.nih.gov/compound/59135335#section=2D-Structure
https://pubchem.ncbi.nlm.nih.gov/compound/59126078#section=2D-Structure
https://pubchem.ncbi.nlm.nih.gov/compound/59125802#section=2D-Structure
https://pubchem.ncbi.nlm.nih.gov/compound/59107609#section=2D-Structure
https://pubchem.ncbi.nlm.nih.gov/compound/59087629#section=2D-Structure
https://pubchem.ncbi.nlm.nih.gov/compound/59069692#section=2D-Structure
https://pubchem.ncbi.nlm.nih.gov/compound/590685#section=2D-Structure
https://pubchem.ncbi.nlm.nih.gov/compound/59068349#section=2D-Structure
https://pubchem.ncbi.nlm.nih.gov/compound/59057775#section=2D-Structure
https://pubchem.ncbi.nlm.nih.gov/compound/59018653#section=2D-Structure
https://pubchem.ncbi.nlm.nih.gov/compound/59017735#section=2D-Structure
https://pubchem.ncbi.nlm.nih.gov/compound/59016494#section=2D-Structure
https://pubchem.ncbi.nlm.nih.gov/compound/59008447#section=2D-Structure
https://pubchem.ncbi.nlm.nih.gov/compound/59008445#section=2D-Structure
https://pubchem.ncbi.nlm.nih.gov/compound/59008443#section=2D-Structure
https://pubchem.ncbi.nlm.nih.gov/compound/59008442#section=2D-Structure
https://pubchem.ncbi.nlm.nih.gov/compound/59000372#section=2D-Structure
https://pubchem.ncbi.nlm.nih.gov/compound/58998073#section=2D-Structure
https://pubchem.ncbi.nlm.nih.gov/compound/58995326#section=2D-Structure
https://pubchem.ncbi.nlm.nih.gov/compound/58991180#section=2D-Structure
https://pubchem.ncbi.nlm.nih.gov/compound/58988440#section=2D-Structure
https://pubchem.ncbi.nlm.nih.gov/compound/58988416#section=2D-Structure
https://pubchem.ncbi.nlm.nih.gov/compound/58986587#section=2D-Structure
https://pubchem.ncbi.nlm.nih.gov/compound/58986583#section=2D-Structure
https://pubchem.ncbi.nlm.nih.gov/compound/589495#section=2D-Structure
https://pubchem.ncbi.nlm.nih.gov/compound/58949420#section=2D-Structure
https://pubchem.ncbi.nlm.nih.gov/compound/58948421#section=2D-Structure
https://pubchem.ncbi.nlm.nih.gov/compound/58944373#section=2D-Structure
https://pubchem.ncbi.nlm.nih.gov/compound/58944313#section=2D-Structure
https://pubchem.ncbi.nlm.nih.gov/compound/58944292#section=2D-Structure
https://pubchem.ncbi.nlm.nih.gov/compound/58944215#section=2D-Structure
https://pubchem.ncbi.nlm.nih.gov/compound/58943873#section=2D-Structure
https://pubchem.ncbi.nlm.nih.gov/compound/58922320#section=2D-Structure
https://pubchem.ncbi.nlm.nih.gov/compound/58895037#section=2D-Structure
https://pubchem.ncbi.nlm.nih.gov/compound/58890229#section=2D-Structure
https://pubchem.ncbi.nlm.nih.gov/compound/58880396#section=2D-Structure
https://pubchem.ncbi.nlm.nih.gov/compound/588784#section=2D-Structure
https://pubchem.ncbi.nlm.nih.gov/compound/58875985#section=2D-Structure
https://pubchem.ncbi.nlm.nih.gov/compound/58875934#section=2D-Structure
https://pubchem.ncbi.nlm.nih.gov/compound/58870903#section=2D-Structure
https://pubchem.ncbi.nlm.nih.gov/compound/58839828#section=2D-Structure
https://pubchem.ncbi.nlm.nih.gov/compound/58824499#section=2D-Structure
https://pubchem.ncbi.nlm.nih.gov/compound/58823892#section=2D-Structure
https://pubchem.ncbi.nlm.nih.gov/compound/58823885#section=2D-Structure
https://pubchem.ncbi.nlm.nih.gov/compound/58823835#section=2D-Structure
https://pubchem.ncbi.nlm.nih.gov/compound/58801158#section=2D-Structure
https://pubchem.ncbi.nlm.nih.gov/compound/58789549#section=2D-Structure
https://pubchem.ncbi.nlm.nih.gov/compound/58789548#section=2D-Structure
https://pubchem.ncbi.nlm.nih.gov/compound/58762185#section=2D-Structure
https://pubchem.ncbi.nlm.nih.gov/compound/58756295#section=2D-Structure
https://pubchem.ncbi.nlm.nih.gov/compound/58754643#section=2D-Structure

https://pubchem.ncbi.nlm.nih.gov/compound/58750801#section=2D-Structure
https://pubchem.ncbi.nlm.nih.gov/compound/58745966#section=2D-Structure
https://pubchem.ncbi.nlm.nih.gov/compound/58738319#section=2D-Structure
https://pubchem.ncbi.nlm.nih.gov/compound/58731939#section=2D-Structure
https://pubchem.ncbi.nlm.nih.gov/compound/58729606#section=2D-Structure
https://pubchem.ncbi.nlm.nih.gov/compound/58717366#section=2D-Structure
https://pubchem.ncbi.nlm.nih.gov/compound/58701457#section=2D-Structure
https://pubchem.ncbi.nlm.nih.gov/compound/58701456#section=2D-Structure
https://pubchem.ncbi.nlm.nih.gov/compound/58701455#section=2D-Structure
https://pubchem.ncbi.nlm.nih.gov/compound/58690944#section=2D-Structure
https://pubchem.ncbi.nlm.nih.gov/compound/58681469#section=2D-Structure
https://pubchem.ncbi.nlm.nih.gov/compound/58673372#section=2D-Structure
https://pubchem.ncbi.nlm.nih.gov/compound/58671709#section=2D-Structure
https://pubchem.ncbi.nlm.nih.gov/compound/58669288#section=2D-Structure
https://pubchem.ncbi.nlm.nih.gov/compound/58664738#section=2D-Structure
https://pubchem.ncbi.nlm.nih.gov/compound/58663917#section=2D-Structure
https://pubchem.ncbi.nlm.nih.gov/compound/5866141#section=2D-Structure
https://pubchem.ncbi.nlm.nih.gov/compound/58623544#section=2D-Structure
https://pubchem.ncbi.nlm.nih.gov/compound/58620803#section=2D-Structure
https://pubchem.ncbi.nlm.nih.gov/compound/58609310#section=2D-Structure
https://pubchem.ncbi.nlm.nih.gov/compound/58567867#section=2D-Structure
https://pubchem.ncbi.nlm.nih.gov/compound/58556007#section=2D-Structure
https://pubchem.ncbi.nlm.nih.gov/compound/58544713#section=2D-Structure
https://pubchem.ncbi.nlm.nih.gov/compound/58544711#section=2D-Structure
https://pubchem.ncbi.nlm.nih.gov/compound/58526586#section=2D-Structure
https://pubchem.ncbi.nlm.nih.gov/compound/58517597#section=2D-Structure
https://pubchem.ncbi.nlm.nih.gov/compound/58490164#section=2D-Structure
https://pubchem.ncbi.nlm.nih.gov/compound/58490148#section=2D-Structure
https://pubchem.ncbi.nlm.nih.gov/compound/584570#section=2D-Structure
https://pubchem.ncbi.nlm.nih.gov/compound/58430135#section=2D-Structure
https://pubchem.ncbi.nlm.nih.gov/compound/58420885#section=2D-Structure
https://pubchem.ncbi.nlm.nih.gov/compound/584114#section=2D-Structure
https://pubchem.ncbi.nlm.nih.gov/compound/58371059#section=2D-Structure
https://pubchem.ncbi.nlm.nih.gov/compound/58361837#section=2D-Structure
https://pubchem.ncbi.nlm.nih.gov/compound/583362#section=2D-Structure
https://pubchem.ncbi.nlm.nih.gov/compound/58310521#section=2D-Structure
https://pubchem.ncbi.nlm.nih.gov/compound/58289197#section=2D-Structure
https://pubchem.ncbi.nlm.nih.gov/compound/58281904#section=2D-Structure
https://pubchem.ncbi.nlm.nih.gov/compound/58279881#section=2D-Structure
https://pubchem.ncbi.nlm.nih.gov/compound/58279850#section=2D-Structure
https://pubchem.ncbi.nlm.nih.gov/compound/582560#section=2D-Structure
https://pubchem.ncbi.nlm.nih.gov/compound/58247289#section=2D-Structure
https://pubchem.ncbi.nlm.nih.gov/compound/58230855#section=2D-Structure
https://pubchem.ncbi.nlm.nih.gov/compound/58205390#section=2D-Structure
https://pubchem.ncbi.nlm.nih.gov/compound/58196027#section=2D-Structure
https://pubchem.ncbi.nlm.nih.gov/compound/58195996#section=2D-Structure
https://pubchem.ncbi.nlm.nih.gov/compound/581543#section=2D-Structure
https://pubchem.ncbi.nlm.nih.gov/compound/58152741#section=2D-Structure
https://pubchem.ncbi.nlm.nih.gov/compound/58106257#section=2D-Structure
https://pubchem.ncbi.nlm.nih.gov/compound/58099771#section=2D-Structure
https://pubchem.ncbi.nlm.nih.gov/compound/58098949#section=2D-Structure
https://pubchem.ncbi.nlm.nih.gov/compound/58084157#section=2D-Structure
https://pubchem.ncbi.nlm.nih.gov/compound/58049736#section=2D-Structure
https://pubchem.ncbi.nlm.nih.gov/compound/58049733#section=2D-Structure
https://pubchem.ncbi.nlm.nih.gov/compound/58049731#section=2D-Structure

https://pubchem.ncbi.nlm.nih.gov/compound/58040963#section=2D-Structure
https://pubchem.ncbi.nlm.nih.gov/compound/58037521#section=2D-Structure
https://pubchem.ncbi.nlm.nih.gov/compound/58037518#section=2D-Structure
https://pubchem.ncbi.nlm.nih.gov/compound/58037508#section=2D-Structure
https://pubchem.ncbi.nlm.nih.gov/compound/58010203#section=2D-Structure
https://pubchem.ncbi.nlm.nih.gov/compound/58009952#section=2D-Structure
https://pubchem.ncbi.nlm.nih.gov/compound/57998409#section=2D-Structure
https://pubchem.ncbi.nlm.nih.gov/compound/57983839#section=2D-Structure
https://pubchem.ncbi.nlm.nih.gov/compound/579832#section=2D-Structure
https://pubchem.ncbi.nlm.nih.gov/compound/57914807#section=2D-Structure
https://pubchem.ncbi.nlm.nih.gov/compound/57913419#section=2D-Structure
https://pubchem.ncbi.nlm.nih.gov/compound/57910840#section=2D-Structure
https://pubchem.ncbi.nlm.nih.gov/compound/57895740#section=2D-Structure
https://pubchem.ncbi.nlm.nih.gov/compound/578867#section=2D-Structure
https://pubchem.ncbi.nlm.nih.gov/compound/57886642#section=2D-Structure
https://pubchem.ncbi.nlm.nih.gov/compound/57864870#section=2D-Structure
https://pubchem.ncbi.nlm.nih.gov/compound/57864866#section=2D-Structure
https://pubchem.ncbi.nlm.nih.gov/compound/57859560#section=2D-Structure
https://pubchem.ncbi.nlm.nih.gov/compound/57859252#section=2D-Structure
https://pubchem.ncbi.nlm.nih.gov/compound/57858194#section=2D-Structure
https://pubchem.ncbi.nlm.nih.gov/compound/57857273#section=2D-Structure
https://pubchem.ncbi.nlm.nih.gov/compound/57857246#section=2D-Structure
https://pubchem.ncbi.nlm.nih.gov/compound/57857243#section=2D-Structure
https://pubchem.ncbi.nlm.nih.gov/compound/57853760#section=2D-Structure
https://pubchem.ncbi.nlm.nih.gov/compound/57852611#section=2D-Structure
https://pubchem.ncbi.nlm.nih.gov/compound/578397#section=2D-Structure
https://pubchem.ncbi.nlm.nih.gov/compound/57834178#section=2D-Structure
https://pubchem.ncbi.nlm.nih.gov/compound/57817472#section=2D-Structure
https://pubchem.ncbi.nlm.nih.gov/compound/57817471#section=2D-Structure
https://pubchem.ncbi.nlm.nih.gov/compound/57815215#section=2D-Structure
https://pubchem.ncbi.nlm.nih.gov/compound/57781253#section=2D-Structure
https://pubchem.ncbi.nlm.nih.gov/compound/577792#section=2D-Structure
https://pubchem.ncbi.nlm.nih.gov/compound/57773325#section=2D-Structure
https://pubchem.ncbi.nlm.nih.gov/compound/57773269#section=2D-Structure
https://pubchem.ncbi.nlm.nih.gov/compound/57773250#section=2D-Structure
https://pubchem.ncbi.nlm.nih.gov/compound/57754487#section=2D-Structure
https://pubchem.ncbi.nlm.nih.gov/compound/57751736#section=2D-Structure
https://pubchem.ncbi.nlm.nih.gov/compound/57730924#section=2D-Structure
https://pubchem.ncbi.nlm.nih.gov/compound/57729781#section=2D-Structure
https://pubchem.ncbi.nlm.nih.gov/compound/57711737#section=2D-Structure
https://pubchem.ncbi.nlm.nih.gov/compound/57709567#section=2D-Structure
https://pubchem.ncbi.nlm.nih.gov/compound/57709565#section=2D-Structure
https://pubchem.ncbi.nlm.nih.gov/compound/57709562#section=2D-Structure
https://pubchem.ncbi.nlm.nih.gov/compound/57687128#section=2D-Structure
https://pubchem.ncbi.nlm.nih.gov/compound/57684226#section=2D-Structure
https://pubchem.ncbi.nlm.nih.gov/compound/57681687#section=2D-Structure
https://pubchem.ncbi.nlm.nih.gov/compound/57675575#section=2D-Structure
https://pubchem.ncbi.nlm.nih.gov/compound/57672039#section=2D-Structure
https://pubchem.ncbi.nlm.nih.gov/compound/57672037#section=2D-Structure
https://pubchem.ncbi.nlm.nih.gov/compound/57653296#section=2D-Structure
https://pubchem.ncbi.nlm.nih.gov/compound/57641800#section=2D-Structure
https://pubchem.ncbi.nlm.nih.gov/compound/57641797#section=2D-Structure
https://pubchem.ncbi.nlm.nih.gov/compound/57638586#section=2D-Structure
https://pubchem.ncbi.nlm.nih.gov/compound/57616815#section=2D-Structure
https://pubchem.ncbi.nlm.nih.gov/compound/57611341#section=2D-Structure

https://pubchem.ncbi.nlm.nih.gov/compound/576083#section=2D-Structure
https://pubchem.ncbi.nlm.nih.gov/compound/57605322#section=2D-Structure
https://pubchem.ncbi.nlm.nih.gov/compound/57596177#section=2D-Structure
https://pubchem.ncbi.nlm.nih.gov/compound/57578272#section=2D-Structure
https://pubchem.ncbi.nlm.nih.gov/compound/57578243#section=2D-Structure
https://pubchem.ncbi.nlm.nih.gov/compound/57553277#section=2D-Structure
https://pubchem.ncbi.nlm.nih.gov/compound/57543580#section=2D-Structure
https://pubchem.ncbi.nlm.nih.gov/compound/57536231#section=2D-Structure
https://pubchem.ncbi.nlm.nih.gov/compound/57529469#section=2D-Structure
https://pubchem.ncbi.nlm.nih.gov/compound/57522734#section=2D-Structure
https://pubchem.ncbi.nlm.nih.gov/compound/57500471#section=2D-Structure
https://pubchem.ncbi.nlm.nih.gov/compound/57495515#section=2D-Structure
https://pubchem.ncbi.nlm.nih.gov/compound/57494212#section=2D-Structure
https://pubchem.ncbi.nlm.nih.gov/compound/57470832#section=2D-Structure
https://pubchem.ncbi.nlm.nih.gov/compound/57469589#section=2D-Structure
https://pubchem.ncbi.nlm.nih.gov/compound/57469474#section=2D-Structure
https://pubchem.ncbi.nlm.nih.gov/compound/57461865#section=2D-Structure
https://pubchem.ncbi.nlm.nih.gov/compound/57461864#section=2D-Structure
https://pubchem.ncbi.nlm.nih.gov/compound/57461743#section=2D-Structure
https://pubchem.ncbi.nlm.nih.gov/compound/57451974#section=2D-Structure
https://pubchem.ncbi.nlm.nih.gov/compound/57450635#section=2D-Structure
https://pubchem.ncbi.nlm.nih.gov/compound/57444127#section=2D-Structure
https://pubchem.ncbi.nlm.nih.gov/compound/57444098#section=2D-Structure
https://pubchem.ncbi.nlm.nih.gov/compound/57435931#section=2D-Structure
https://pubchem.ncbi.nlm.nih.gov/compound/57433430#section=2D-Structure
https://pubchem.ncbi.nlm.nih.gov/compound/57421637#section=2D-Structure
https://pubchem.ncbi.nlm.nih.gov/compound/57417572#section=2D-Structure
https://pubchem.ncbi.nlm.nih.gov/compound/57380507#section=2D-Structure
https://pubchem.ncbi.nlm.nih.gov/compound/57376932#section=2D-Structure
https://pubchem.ncbi.nlm.nih.gov/compound/57376327#section=2D-Structure
https://pubchem.ncbi.nlm.nih.gov/compound/57376306#section=2D-Structure
https://pubchem.ncbi.nlm.nih.gov/compound/57375896#section=2D-Structure
https://pubchem.ncbi.nlm.nih.gov/compound/57374606#section=2D-Structure
https://pubchem.ncbi.nlm.nih.gov/compound/57372961#section=2D-Structure
https://pubchem.ncbi.nlm.nih.gov/compound/57370801#section=2D-Structure
https://pubchem.ncbi.nlm.nih.gov/compound/57370316#section=2D-Structure
https://pubchem.ncbi.nlm.nih.gov/compound/57370283#section=2D-Structure
https://pubchem.ncbi.nlm.nih.gov/compound/57364363#section=2D-Structure
https://pubchem.ncbi.nlm.nih.gov/compound/57358873#section=2D-Structure
https://pubchem.ncbi.nlm.nih.gov/compound/57358783#section=2D-Structure
https://pubchem.ncbi.nlm.nih.gov/compound/57358685#section=2D-Structure
https://pubchem.ncbi.nlm.nih.gov/compound/57358663#section=2D-Structure
https://pubchem.ncbi.nlm.nih.gov/compound/57358593#section=2D-Structure
https://pubchem.ncbi.nlm.nih.gov/compound/57358466#section=2D-Structure
https://pubchem.ncbi.nlm.nih.gov/compound/57358101#section=2D-Structure
https://pubchem.ncbi.nlm.nih.gov/compound/57358100#section=2D-Structure
https://pubchem.ncbi.nlm.nih.gov/compound/57358051#section=2D-Structure
https://pubchem.ncbi.nlm.nih.gov/compound/57357956#section=2D-Structure
https://pubchem.ncbi.nlm.nih.gov/compound/57357844#section=2D-Structure
https://pubchem.ncbi.nlm.nih.gov/compound/57357843#section=2D-Structure
https://pubchem.ncbi.nlm.nih.gov/compound/57357811#section=2D-Structure
https://pubchem.ncbi.nlm.nih.gov/compound/57357758#section=2D-Structure
https://pubchem.ncbi.nlm.nih.gov/compound/57357749#section=2D-Structure
https://pubchem.ncbi.nlm.nih.gov/compound/57357682#section=2D-Structure
https://pubchem.ncbi.nlm.nih.gov/compound/57357572#section=2D-Structure

https://pubchem.ncbi.nlm.nih.gov/compound/57357559#section=2D-Structure
https://pubchem.ncbi.nlm.nih.gov/compound/57357522#section=2D-Structure
https://pubchem.ncbi.nlm.nih.gov/compound/57357383#section=2D-Structure
https://pubchem.ncbi.nlm.nih.gov/compound/57357341#section=2D-Structure
https://pubchem.ncbi.nlm.nih.gov/compound/57355519#section=2D-Structure
https://pubchem.ncbi.nlm.nih.gov/compound/57355165#section=2D-Structure
https://pubchem.ncbi.nlm.nih.gov/compound/57354145#section=2D-Structure
https://pubchem.ncbi.nlm.nih.gov/compound/57353111#section=2D-Structure
https://pubchem.ncbi.nlm.nih.gov/compound/57352477#section=2D-Structure
https://pubchem.ncbi.nlm.nih.gov/compound/57352246#section=2D-Structure
https://pubchem.ncbi.nlm.nih.gov/compound/57351887#section=2D-Structure
https://pubchem.ncbi.nlm.nih.gov/compound/57351601#section=2D-Structure
https://pubchem.ncbi.nlm.nih.gov/compound/57350730#section=2D-Structure
https://pubchem.ncbi.nlm.nih.gov/compound/57349913#section=2D-Structure
https://pubchem.ncbi.nlm.nih.gov/compound/57349912#section=2D-Structure
https://pubchem.ncbi.nlm.nih.gov/compound/57349555#section=2D-Structure
https://pubchem.ncbi.nlm.nih.gov/compound/57348845#section=2D-Structure
https://pubchem.ncbi.nlm.nih.gov/compound/57348516#section=2D-Structure
https://pubchem.ncbi.nlm.nih.gov/compound/57347925#section=2D-Structure
https://pubchem.ncbi.nlm.nih.gov/compound/57347419#section=2D-Structure
https://pubchem.ncbi.nlm.nih.gov/compound/57347297#section=2D-Structure
https://pubchem.ncbi.nlm.nih.gov/compound/57347296#section=2D-Structure
https://pubchem.ncbi.nlm.nih.gov/compound/57336669#section=2D-Structure
https://pubchem.ncbi.nlm.nih.gov/compound/57332003#section=2D-Structure
https://pubchem.ncbi.nlm.nih.gov/compound/57322914#section=2D-Structure
https://pubchem.ncbi.nlm.nih.gov/compound/573156#section=2D-Structure
https://pubchem.ncbi.nlm.nih.gov/compound/57313595#section=2D-Structure
https://pubchem.ncbi.nlm.nih.gov/compound/57301150#section=2D-Structure
https://pubchem.ncbi.nlm.nih.gov/compound/57300954#section=2D-Structure
https://pubchem.ncbi.nlm.nih.gov/compound/57298362#section=2D-Structure
https://pubchem.ncbi.nlm.nih.gov/compound/57293262#section=2D-Structure
https://pubchem.ncbi.nlm.nih.gov/compound/57292138#section=2D-Structure
https://pubchem.ncbi.nlm.nih.gov/compound/57292102#section=2D-Structure
https://pubchem.ncbi.nlm.nih.gov/compound/57291597#section=2D-Structure
https://pubchem.ncbi.nlm.nih.gov/compound/57289964#section=2D-Structure
https://pubchem.ncbi.nlm.nih.gov/compound/57284317#section=2D-Structure
https://pubchem.ncbi.nlm.nih.gov/compound/57282709#section=2D-Structure
https://pubchem.ncbi.nlm.nih.gov/compound/57282519#section=2D-Structure
https://pubchem.ncbi.nlm.nih.gov/compound/57281252#section=2D-Structure
https://pubchem.ncbi.nlm.nih.gov/compound/57280515#section=2D-Structure
https://pubchem.ncbi.nlm.nih.gov/compound/57280118#section=2D-Structure
https://pubchem.ncbi.nlm.nih.gov/compound/57270762#section=2D-Structure
https://pubchem.ncbi.nlm.nih.gov/compound/57270211#section=2D-Structure
https://pubchem.ncbi.nlm.nih.gov/compound/57268492#section=2D-Structure
https://pubchem.ncbi.nlm.nih.gov/compound/57265468#section=2D-Structure
https://pubchem.ncbi.nlm.nih.gov/compound/572653#section=2D-Structure
https://pubchem.ncbi.nlm.nih.gov/compound/57264785#section=2D-Structure
https://pubchem.ncbi.nlm.nih.gov/compound/57264202#section=2D-Structure
https://pubchem.ncbi.nlm.nih.gov/compound/57261060#section=2D-Structure
https://pubchem.ncbi.nlm.nih.gov/compound/57258506#section=2D-Structure
https://pubchem.ncbi.nlm.nih.gov/compound/57251542#section=2D-Structure
https://pubchem.ncbi.nlm.nih.gov/compound/57248913#section=2D-Structure
https://pubchem.ncbi.nlm.nih.gov/compound/57244412#section=2D-Structure
https://pubchem.ncbi.nlm.nih.gov/compound/57242977#section=2D-Structure
https://pubchem.ncbi.nlm.nih.gov/compound/57240257#section=2D-Structure

https://pubchem.ncbi.nlm.nih.gov/compound/57240146#section=2D-Structure
https://pubchem.ncbi.nlm.nih.gov/compound/57239844#section=2D-Structure
https://pubchem.ncbi.nlm.nih.gov/compound/57239397#section=2D-Structure
https://pubchem.ncbi.nlm.nih.gov/compound/57238768#section=2D-Structure
https://pubchem.ncbi.nlm.nih.gov/compound/57237259#section=2D-Structure
https://pubchem.ncbi.nlm.nih.gov/compound/57237015#section=2D-Structure
https://pubchem.ncbi.nlm.nih.gov/compound/57236524#section=2D-Structure
https://pubchem.ncbi.nlm.nih.gov/compound/57235232#section=2D-Structure
https://pubchem.ncbi.nlm.nih.gov/compound/57232374#section=2D-Structure
https://pubchem.ncbi.nlm.nih.gov/compound/57224582#section=2D-Structure
https://pubchem.ncbi.nlm.nih.gov/compound/57222719#section=2D-Structure
https://pubchem.ncbi.nlm.nih.gov/compound/57221964#section=2D-Structure
https://pubchem.ncbi.nlm.nih.gov/compound/57220733#section=2D-Structure
https://pubchem.ncbi.nlm.nih.gov/compound/57218060#section=2D-Structure
https://pubchem.ncbi.nlm.nih.gov/compound/57217773#section=2D-Structure
https://pubchem.ncbi.nlm.nih.gov/compound/57217050#section=2D-Structure
https://pubchem.ncbi.nlm.nih.gov/compound/57210844#section=2D-Structure
https://pubchem.ncbi.nlm.nih.gov/compound/57210088#section=2D-Structure
https://pubchem.ncbi.nlm.nih.gov/compound/57209646#section=2D-Structure
https://pubchem.ncbi.nlm.nih.gov/compound/57207223#section=2D-Structure
https://pubchem.ncbi.nlm.nih.gov/compound/57201531#section=2D-Structure
https://pubchem.ncbi.nlm.nih.gov/compound/57199410#section=2D-Structure
https://pubchem.ncbi.nlm.nih.gov/compound/57198009#section=2D-Structure
https://pubchem.ncbi.nlm.nih.gov/compound/57195549#section=2D-Structure
https://pubchem.ncbi.nlm.nih.gov/compound/57191331#section=2D-Structure
https://pubchem.ncbi.nlm.nih.gov/compound/57189059#section=2D-Structure
https://pubchem.ncbi.nlm.nih.gov/compound/57186667#section=2D-Structure
https://pubchem.ncbi.nlm.nih.gov/compound/57185932#section=2D-Structure
https://pubchem.ncbi.nlm.nih.gov/compound/57183226#section=2D-Structure
https://pubchem.ncbi.nlm.nih.gov/compound/57180665#section=2D-Structure
https://pubchem.ncbi.nlm.nih.gov/compound/57179906#section=2D-Structure
https://pubchem.ncbi.nlm.nih.gov/compound/57179876#section=2D-Structure
https://pubchem.ncbi.nlm.nih.gov/compound/57176543#section=2D-Structure
https://pubchem.ncbi.nlm.nih.gov/compound/57172534#section=2D-Structure
https://pubchem.ncbi.nlm.nih.gov/compound/57169914#section=2D-Structure
https://pubchem.ncbi.nlm.nih.gov/compound/57167529#section=2D-Structure
https://pubchem.ncbi.nlm.nih.gov/compound/57164831#section=2D-Structure
https://pubchem.ncbi.nlm.nih.gov/compound/57160146#section=2D-Structure
https://pubchem.ncbi.nlm.nih.gov/compound/57158479#section=2D-Structure
https://pubchem.ncbi.nlm.nih.gov/compound/57158293#section=2D-Structure
https://pubchem.ncbi.nlm.nih.gov/compound/57157741#section=2D-Structure
https://pubchem.ncbi.nlm.nih.gov/compound/57153900#section=2D-Structure
https://pubchem.ncbi.nlm.nih.gov/compound/57153036#section=2D-Structure
https://pubchem.ncbi.nlm.nih.gov/compound/57152082#section=2D-Structure
https://pubchem.ncbi.nlm.nih.gov/compound/57148956#section=2D-Structure
https://pubchem.ncbi.nlm.nih.gov/compound/57148835#section=2D-Structure
https://pubchem.ncbi.nlm.nih.gov/compound/57141459#section=2D-Structure
https://pubchem.ncbi.nlm.nih.gov/compound/57140065#section=2D-Structure
https://pubchem.ncbi.nlm.nih.gov/compound/57134691#section=2D-Structure
https://pubchem.ncbi.nlm.nih.gov/compound/57133864#section=2D-Structure
https://pubchem.ncbi.nlm.nih.gov/compound/57129642#section=2D-Structure
https://pubchem.ncbi.nlm.nih.gov/compound/57127816#section=2D-Structure
https://pubchem.ncbi.nlm.nih.gov/compound/57124699#section=2D-Structure
https://pubchem.ncbi.nlm.nih.gov/compound/57119038#section=2D-Structure
https://pubchem.ncbi.nlm.nih.gov/compound/57118589#section=2D-Structure

https://pubchem.ncbi.nlm.nih.gov/compound/57116115#section=2D-Structure
https://pubchem.ncbi.nlm.nih.gov/compound/57115356#section=2D-Structure
https://pubchem.ncbi.nlm.nih.gov/compound/57115245#section=2D-Structure
https://pubchem.ncbi.nlm.nih.gov/compound/57114855#section=2D-Structure
https://pubchem.ncbi.nlm.nih.gov/compound/57109138#section=2D-Structure
https://pubchem.ncbi.nlm.nih.gov/compound/57109128#section=2D-Structure
https://pubchem.ncbi.nlm.nih.gov/compound/57108754#section=2D-Structure
https://pubchem.ncbi.nlm.nih.gov/compound/57108691#section=2D-Structure
https://pubchem.ncbi.nlm.nih.gov/compound/57107312#section=2D-Structure
https://pubchem.ncbi.nlm.nih.gov/compound/57102823#section=2D-Structure
https://pubchem.ncbi.nlm.nih.gov/compound/57098504#section=2D-Structure
https://pubchem.ncbi.nlm.nih.gov/compound/57098219#section=2D-Structure
https://pubchem.ncbi.nlm.nih.gov/compound/57096365#section=2D-Structure
https://pubchem.ncbi.nlm.nih.gov/compound/57092555#section=2D-Structure
https://pubchem.ncbi.nlm.nih.gov/compound/57086454#section=2D-Structure
https://pubchem.ncbi.nlm.nih.gov/compound/57084630#section=2D-Structure
https://pubchem.ncbi.nlm.nih.gov/compound/57081434#section=2D-Structure
https://pubchem.ncbi.nlm.nih.gov/compound/57077106#section=2D-Structure
https://pubchem.ncbi.nlm.nih.gov/compound/57072129#section=2D-Structure
https://pubchem.ncbi.nlm.nih.gov/compound/57069473#section=2D-Structure
https://pubchem.ncbi.nlm.nih.gov/compound/57066674#section=2D-Structure
https://pubchem.ncbi.nlm.nih.gov/compound/57064905#section=2D-Structure
https://pubchem.ncbi.nlm.nih.gov/compound/57057543#section=2D-Structure
https://pubchem.ncbi.nlm.nih.gov/compound/57050529#section=2D-Structure
https://pubchem.ncbi.nlm.nih.gov/compound/57049172#section=2D-Structure
https://pubchem.ncbi.nlm.nih.gov/compound/57048447#section=2D-Structure
https://pubchem.ncbi.nlm.nih.gov/compound/57047390#section=2D-Structure
https://pubchem.ncbi.nlm.nih.gov/compound/57045158#section=2D-Structure
https://pubchem.ncbi.nlm.nih.gov/compound/57042169#section=2D-Structure
https://pubchem.ncbi.nlm.nih.gov/compound/57039976#section=2D-Structure
https://pubchem.ncbi.nlm.nih.gov/compound/57035422#section=2D-Structure
https://pubchem.ncbi.nlm.nih.gov/compound/57034641#section=2D-Structure
https://pubchem.ncbi.nlm.nih.gov/compound/57034318#section=2D-Structure
https://pubchem.ncbi.nlm.nih.gov/compound/57031190#section=2D-Structure
https://pubchem.ncbi.nlm.nih.gov/compound/57027101#section=2D-Structure
https://pubchem.ncbi.nlm.nih.gov/compound/57026792#section=2D-Structure
https://pubchem.ncbi.nlm.nih.gov/compound/57026716#section=2D-Structure
https://pubchem.ncbi.nlm.nih.gov/compound/57023871#section=2D-Structure
https://pubchem.ncbi.nlm.nih.gov/compound/57018152#section=2D-Structure
https://pubchem.ncbi.nlm.nih.gov/compound/57016149#section=2D-Structure
https://pubchem.ncbi.nlm.nih.gov/compound/57007164#section=2D-Structure
https://pubchem.ncbi.nlm.nih.gov/compound/57004019#section=2D-Structure
https://pubchem.ncbi.nlm.nih.gov/compound/56999754#section=2D-Structure
https://pubchem.ncbi.nlm.nih.gov/compound/56999153#section=2D-Structure
https://pubchem.ncbi.nlm.nih.gov/compound/56998631#section=2D-Structure
https://pubchem.ncbi.nlm.nih.gov/compound/56997182#section=2D-Structure
https://pubchem.ncbi.nlm.nih.gov/compound/56992700#section=2D-Structure
https://pubchem.ncbi.nlm.nih.gov/compound/56982525#section=2D-Structure
https://pubchem.ncbi.nlm.nih.gov/compound/56981217#section=2D-Structure
https://pubchem.ncbi.nlm.nih.gov/compound/56978916#section=2D-Structure
https://pubchem.ncbi.nlm.nih.gov/compound/56975039#section=2D-Structure
https://pubchem.ncbi.nlm.nih.gov/compound/56972666#section=2D-Structure
https://pubchem.ncbi.nlm.nih.gov/compound/56972648#section=2D-Structure
https://pubchem.ncbi.nlm.nih.gov/compound/56972634#section=2D-Structure
https://pubchem.ncbi.nlm.nih.gov/compound/56972633#section=2D-Structure

https://pubchem.ncbi.nlm.nih.gov/compound/56972632#section=2D-Structure
https://pubchem.ncbi.nlm.nih.gov/compound/56972630#section=2D-Structure
https://pubchem.ncbi.nlm.nih.gov/compound/56972380#section=2D-Structure
https://pubchem.ncbi.nlm.nih.gov/compound/56972319#section=2D-Structure
https://pubchem.ncbi.nlm.nih.gov/compound/568978#section=2D-Structure
https://pubchem.ncbi.nlm.nih.gov/compound/56834906#section=2D-Structure
https://pubchem.ncbi.nlm.nih.gov/compound/56695034#section=2D-Structure
https://pubchem.ncbi.nlm.nih.gov/compound/56692257#section=2D-Structure
https://pubchem.ncbi.nlm.nih.gov/compound/56638532#section=2D-Structure
https://pubchem.ncbi.nlm.nih.gov/compound/56636296#section=2D-Structure
https://pubchem.ncbi.nlm.nih.gov/compound/56634811#section=2D-Structure
https://pubchem.ncbi.nlm.nih.gov/compound/56634324#section=2D-Structure
https://pubchem.ncbi.nlm.nih.gov/compound/56633852#section=2D-Structure
https://pubchem.ncbi.nlm.nih.gov/compound/56632208#section=2D-Structure
https://pubchem.ncbi.nlm.nih.gov/compound/56632128#section=2D-Structure
https://pubchem.ncbi.nlm.nih.gov/compound/56631850#section=2D-Structure
https://pubchem.ncbi.nlm.nih.gov/compound/56628890#section=2D-Structure
https://pubchem.ncbi.nlm.nih.gov/compound/56628192#section=2D-Structure
https://pubchem.ncbi.nlm.nih.gov/compound/56625553#section=2D-Structure
https://pubchem.ncbi.nlm.nih.gov/compound/56625474#section=2D-Structure
https://pubchem.ncbi.nlm.nih.gov/compound/56623997#section=2D-Structure
https://pubchem.ncbi.nlm.nih.gov/compound/56623228#section=2D-Structure
https://pubchem.ncbi.nlm.nih.gov/compound/56620558#section=2D-Structure
https://pubchem.ncbi.nlm.nih.gov/compound/56617165#section=2D-Structure
https://pubchem.ncbi.nlm.nih.gov/compound/56616229#section=2D-Structure
https://pubchem.ncbi.nlm.nih.gov/compound/56615601#section=2D-Structure
https://pubchem.ncbi.nlm.nih.gov/compound/56615129#section=2D-Structure
https://pubchem.ncbi.nlm.nih.gov/compound/56614147#section=2D-Structure
https://pubchem.ncbi.nlm.nih.gov/compound/56613411#section=2D-Structure
https://pubchem.ncbi.nlm.nih.gov/compound/56612013#section=2D-Structure
https://pubchem.ncbi.nlm.nih.gov/compound/56611504#section=2D-Structure
https://pubchem.ncbi.nlm.nih.gov/compound/56610688#section=2D-Structure
https://pubchem.ncbi.nlm.nih.gov/compound/56609019#section=2D-Structure
https://pubchem.ncbi.nlm.nih.gov/compound/56608791#section=2D-Structure
https://pubchem.ncbi.nlm.nih.gov/compound/56605812#section=2D-Structure
https://pubchem.ncbi.nlm.nih.gov/compound/564660#section=2D-Structure
https://pubchem.ncbi.nlm.nih.gov/compound/564381#section=2D-Structure
https://pubchem.ncbi.nlm.nih.gov/compound/564238#section=2D-Structure
https://pubchem.ncbi.nlm.nih.gov/compound/556491#section=2D-Structure
https://pubchem.ncbi.nlm.nih.gov/compound/55487#section=2D-Structure
https://pubchem.ncbi.nlm.nih.gov/compound/55299820#section=2D-Structure
https://pubchem.ncbi.nlm.nih.gov/compound/55296570#section=2D-Structure
https://pubchem.ncbi.nlm.nih.gov/compound/55296249#section=2D-Structure
https://pubchem.ncbi.nlm.nih.gov/compound/55288848#section=2D-Structure
https://pubchem.ncbi.nlm.nih.gov/compound/55288689#section=2D-Structure
https://pubchem.ncbi.nlm.nih.gov/compound/55281080#section=2D-Structure
https://pubchem.ncbi.nlm.nih.gov/compound/55253791#section=2D-Structure
https://pubchem.ncbi.nlm.nih.gov/compound/55250597#section=2D-Structure
https://pubchem.ncbi.nlm.nih.gov/compound/550252#section=2D-Structure
https://pubchem.ncbi.nlm.nih.gov/compound/549232#section=2D-Structure
https://pubchem.ncbi.nlm.nih.gov/compound/548978#section=2D-Structure
https://pubchem.ncbi.nlm.nih.gov/compound/548518#section=2D-Structure
https://pubchem.ncbi.nlm.nih.gov/compound/5476997#section=2D-Structure
https://pubchem.ncbi.nlm.nih.gov/compound/54763228#section=2D-Structure
https://pubchem.ncbi.nlm.nih.gov/compound/5476141#section=2D-Structure

https://pubchem.ncbi.nlm.nih.gov/compound/5474684#section=2D-Structure
https://pubchem.ncbi.nlm.nih.gov/compound/54717150#section=2D-Structure
https://pubchem.ncbi.nlm.nih.gov/compound/54717146#section=2D-Structure
https://pubchem.ncbi.nlm.nih.gov/compound/54717139#section=2D-Structure
https://pubchem.ncbi.nlm.nih.gov/compound/54693803#section=2D-Structure
https://pubchem.ncbi.nlm.nih.gov/compound/54689224#section=2D-Structure
https://pubchem.ncbi.nlm.nih.gov/compound/5466882#section=2D-Structure
https://pubchem.ncbi.nlm.nih.gov/compound/5460734#section=2D-Structure
https://pubchem.ncbi.nlm.nih.gov/compound/5460726#section=2D-Structure
https://pubchem.ncbi.nlm.nih.gov/compound/54563511#section=2D-Structure
https://pubchem.ncbi.nlm.nih.gov/compound/54560805#section=2D-Structure
https://pubchem.ncbi.nlm.nih.gov/compound/54545031#section=2D-Structure
https://pubchem.ncbi.nlm.nih.gov/compound/54542864#section=2D-Structure
https://pubchem.ncbi.nlm.nih.gov/compound/54542644#section=2D-Structure
https://pubchem.ncbi.nlm.nih.gov/compound/54537181#section=2D-Structure
https://pubchem.ncbi.nlm.nih.gov/compound/54527377#section=2D-Structure
https://pubchem.ncbi.nlm.nih.gov/compound/54525840#section=2D-Structure
https://pubchem.ncbi.nlm.nih.gov/compound/54517402#section=2D-Structure
https://pubchem.ncbi.nlm.nih.gov/compound/54504639#section=2D-Structure
https://pubchem.ncbi.nlm.nih.gov/compound/54499318#section=2D-Structure
https://pubchem.ncbi.nlm.nih.gov/compound/54494392#section=2D-Structure
https://pubchem.ncbi.nlm.nih.gov/compound/54494275#section=2D-Structure
https://pubchem.ncbi.nlm.nih.gov/compound/54494099#section=2D-Structure
https://pubchem.ncbi.nlm.nih.gov/compound/54491356#section=2D-Structure
https://pubchem.ncbi.nlm.nih.gov/compound/54491094#section=2D-Structure
https://pubchem.ncbi.nlm.nih.gov/compound/54487064#section=2D-Structure
https://pubchem.ncbi.nlm.nih.gov/compound/54478018#section=2D-Structure
https://pubchem.ncbi.nlm.nih.gov/compound/54470948#section=2D-Structure
https://pubchem.ncbi.nlm.nih.gov/compound/54458500#section=2D-Structure
https://pubchem.ncbi.nlm.nih.gov/compound/54458033#section=2D-Structure
https://pubchem.ncbi.nlm.nih.gov/compound/54457645#section=2D-Structure
https://pubchem.ncbi.nlm.nih.gov/compound/54455503#section=2D-Structure
https://pubchem.ncbi.nlm.nih.gov/compound/54453049#section=2D-Structure
https://pubchem.ncbi.nlm.nih.gov/compound/54449736#section=2D-Structure
https://pubchem.ncbi.nlm.nih.gov/compound/54446673#section=2D-Structure
https://pubchem.ncbi.nlm.nih.gov/compound/54440655#section=2D-Structure
https://pubchem.ncbi.nlm.nih.gov/compound/54439389#section=2D-Structure
https://pubchem.ncbi.nlm.nih.gov/compound/54427985#section=2D-Structure
https://pubchem.ncbi.nlm.nih.gov/compound/54424796#section=2D-Structure
https://pubchem.ncbi.nlm.nih.gov/compound/54424226#section=2D-Structure
https://pubchem.ncbi.nlm.nih.gov/compound/54418888#section=2D-Structure
https://pubchem.ncbi.nlm.nih.gov/compound/54416719#section=2D-Structure
https://pubchem.ncbi.nlm.nih.gov/compound/54409936#section=2D-Structure
https://pubchem.ncbi.nlm.nih.gov/compound/54408073#section=2D-Structure
https://pubchem.ncbi.nlm.nih.gov/compound/54407186#section=2D-Structure
https://pubchem.ncbi.nlm.nih.gov/compound/54406986#section=2D-Structure
https://pubchem.ncbi.nlm.nih.gov/compound/54405697#section=2D-Structure
https://pubchem.ncbi.nlm.nih.gov/compound/54405611#section=2D-Structure
https://pubchem.ncbi.nlm.nih.gov/compound/54404489#section=2D-Structure
https://pubchem.ncbi.nlm.nih.gov/compound/54396365#section=2D-Structure
https://pubchem.ncbi.nlm.nih.gov/compound/54374541#section=2D-Structure
https://pubchem.ncbi.nlm.nih.gov/compound/54367030#section=2D-Structure
https://pubchem.ncbi.nlm.nih.gov/compound/54363980#section=2D-Structure
https://pubchem.ncbi.nlm.nih.gov/compound/54357120#section=2D-Structure
https://pubchem.ncbi.nlm.nih.gov/compound/54356889#section=2D-Structure

https://pubchem.ncbi.nlm.nih.gov/compound/54343651#section=2D-Structure
https://pubchem.ncbi.nlm.nih.gov/compound/54342937#section=2D-Structure
https://pubchem.ncbi.nlm.nih.gov/compound/54342378#section=2D-Structure
https://pubchem.ncbi.nlm.nih.gov/compound/54338256#section=2D-Structure
https://pubchem.ncbi.nlm.nih.gov/compound/54335979#section=2D-Structure
https://pubchem.ncbi.nlm.nih.gov/compound/54335908#section=2D-Structure
https://pubchem.ncbi.nlm.nih.gov/compound/54325832#section=2D-Structure
https://pubchem.ncbi.nlm.nih.gov/compound/54323653#section=2D-Structure
https://pubchem.ncbi.nlm.nih.gov/compound/54316117#section=2D-Structure
https://pubchem.ncbi.nlm.nih.gov/compound/54314193#section=2D-Structure
https://pubchem.ncbi.nlm.nih.gov/compound/54312799#section=2D-Structure
https://pubchem.ncbi.nlm.nih.gov/compound/54309499#section=2D-Structure
https://pubchem.ncbi.nlm.nih.gov/compound/54308151#section=2D-Structure
https://pubchem.ncbi.nlm.nih.gov/compound/54296254#section=2D-Structure
https://pubchem.ncbi.nlm.nih.gov/compound/54295785#section=2D-Structure
https://pubchem.ncbi.nlm.nih.gov/compound/54292025#section=2D-Structure
https://pubchem.ncbi.nlm.nih.gov/compound/54286612#section=2D-Structure
https://pubchem.ncbi.nlm.nih.gov/compound/542786#section=2D-Structure
https://pubchem.ncbi.nlm.nih.gov/compound/54274495#section=2D-Structure
https://pubchem.ncbi.nlm.nih.gov/compound/54273132#section=2D-Structure
https://pubchem.ncbi.nlm.nih.gov/compound/54273048#section=2D-Structure
https://pubchem.ncbi.nlm.nih.gov/compound/54271589#section=2D-Structure
https://pubchem.ncbi.nlm.nih.gov/compound/54271564#section=2D-Structure
https://pubchem.ncbi.nlm.nih.gov/compound/542639#section=2D-Structure
https://pubchem.ncbi.nlm.nih.gov/compound/54263309#section=2D-Structure
https://pubchem.ncbi.nlm.nih.gov/compound/54259042#section=2D-Structure
https://pubchem.ncbi.nlm.nih.gov/compound/54254601#section=2D-Structure
https://pubchem.ncbi.nlm.nih.gov/compound/54253715#section=2D-Structure
https://pubchem.ncbi.nlm.nih.gov/compound/54252523#section=2D-Structure
https://pubchem.ncbi.nlm.nih.gov/compound/54252407#section=2D-Structure
https://pubchem.ncbi.nlm.nih.gov/compound/54251527#section=2D-Structure
https://pubchem.ncbi.nlm.nih.gov/compound/54248390#section=2D-Structure
https://pubchem.ncbi.nlm.nih.gov/compound/54246136#section=2D-Structure
https://pubchem.ncbi.nlm.nih.gov/compound/54241957#section=2D-Structure
https://pubchem.ncbi.nlm.nih.gov/compound/54239542#section=2D-Structure
https://pubchem.ncbi.nlm.nih.gov/compound/54231321#section=2D-Structure
https://pubchem.ncbi.nlm.nih.gov/compound/54227804#section=2D-Structure
https://pubchem.ncbi.nlm.nih.gov/compound/54226955#section=2D-Structure
https://pubchem.ncbi.nlm.nih.gov/compound/54225441#section=2D-Structure
https://pubchem.ncbi.nlm.nih.gov/compound/54220801#section=2D-Structure
https://pubchem.ncbi.nlm.nih.gov/compound/54213635#section=2D-Structure
https://pubchem.ncbi.nlm.nih.gov/compound/54213230#section=2D-Structure
https://pubchem.ncbi.nlm.nih.gov/compound/54208249#section=2D-Structure
https://pubchem.ncbi.nlm.nih.gov/compound/54205515#section=2D-Structure
https://pubchem.ncbi.nlm.nih.gov/compound/54199749#section=2D-Structure
https://pubchem.ncbi.nlm.nih.gov/compound/54195629#section=2D-Structure
https://pubchem.ncbi.nlm.nih.gov/compound/54189383#section=2D-Structure
https://pubchem.ncbi.nlm.nih.gov/compound/54188234#section=2D-Structure
https://pubchem.ncbi.nlm.nih.gov/compound/54185489#section=2D-Structure
https://pubchem.ncbi.nlm.nih.gov/compound/54184204#section=2D-Structure
https://pubchem.ncbi.nlm.nih.gov/compound/54171261#section=2D-Structure
https://pubchem.ncbi.nlm.nih.gov/compound/54170451#section=2D-Structure
https://pubchem.ncbi.nlm.nih.gov/compound/54169346#section=2D-Structure
https://pubchem.ncbi.nlm.nih.gov/compound/54166985#section=2D-Structure
https://pubchem.ncbi.nlm.nih.gov/compound/54157735#section=2D-Structure

https://pubchem.ncbi.nlm.nih.gov/compound/54153964#section=2D-Structure
https://pubchem.ncbi.nlm.nih.gov/compound/54150424#section=2D-Structure
https://pubchem.ncbi.nlm.nih.gov/compound/54145411#section=2D-Structure
https://pubchem.ncbi.nlm.nih.gov/compound/54140692#section=2D-Structure
https://pubchem.ncbi.nlm.nih.gov/compound/54124950#section=2D-Structure
https://pubchem.ncbi.nlm.nih.gov/compound/54117195#section=2D-Structure
https://pubchem.ncbi.nlm.nih.gov/compound/54109654#section=2D-Structure
https://pubchem.ncbi.nlm.nih.gov/compound/54108076#section=2D-Structure
https://pubchem.ncbi.nlm.nih.gov/compound/54105587#section=2D-Structure
https://pubchem.ncbi.nlm.nih.gov/compound/5409526#section=2D-Structure
https://pubchem.ncbi.nlm.nih.gov/compound/54092732#section=2D-Structure
https://pubchem.ncbi.nlm.nih.gov/compound/54085890#section=2D-Structure
https://pubchem.ncbi.nlm.nih.gov/compound/54085464#section=2D-Structure
https://pubchem.ncbi.nlm.nih.gov/compound/54069466#section=2D-Structure
https://pubchem.ncbi.nlm.nih.gov/compound/54066051#section=2D-Structure
https://pubchem.ncbi.nlm.nih.gov/compound/54065269#section=2D-Structure
https://pubchem.ncbi.nlm.nih.gov/compound/54061398#section=2D-Structure
https://pubchem.ncbi.nlm.nih.gov/compound/54059262#section=2D-Structure
https://pubchem.ncbi.nlm.nih.gov/compound/54058859#section=2D-Structure
https://pubchem.ncbi.nlm.nih.gov/compound/54057896#section=2D-Structure
https://pubchem.ncbi.nlm.nih.gov/compound/54055928#section=2D-Structure
https://pubchem.ncbi.nlm.nih.gov/compound/54047071#section=2D-Structure
https://pubchem.ncbi.nlm.nih.gov/compound/54040076#section=2D-Structure
https://pubchem.ncbi.nlm.nih.gov/compound/54037601#section=2D-Structure
https://pubchem.ncbi.nlm.nih.gov/compound/54032095#section=2D-Structure
https://pubchem.ncbi.nlm.nih.gov/compound/54020179#section=2D-Structure
https://pubchem.ncbi.nlm.nih.gov/compound/54016621#section=2D-Structure
https://pubchem.ncbi.nlm.nih.gov/compound/54012974#section=2D-Structure
https://pubchem.ncbi.nlm.nih.gov/compound/54011103#section=2D-Structure
https://pubchem.ncbi.nlm.nih.gov/compound/54006931#section=2D-Structure
https://pubchem.ncbi.nlm.nih.gov/compound/54004237#section=2D-Structure
https://pubchem.ncbi.nlm.nih.gov/compound/54001359#section=2D-Structure
https://pubchem.ncbi.nlm.nih.gov/compound/53995246#section=2D-Structure
https://pubchem.ncbi.nlm.nih.gov/compound/53991988#section=2D-Structure
https://pubchem.ncbi.nlm.nih.gov/compound/53991673#section=2D-Structure
https://pubchem.ncbi.nlm.nih.gov/compound/53990564#section=2D-Structure
https://pubchem.ncbi.nlm.nih.gov/compound/53984828#section=2D-Structure
https://pubchem.ncbi.nlm.nih.gov/compound/53978627#section=2D-Structure
https://pubchem.ncbi.nlm.nih.gov/compound/5397448#section=2D-Structure
https://pubchem.ncbi.nlm.nih.gov/compound/53957209#section=2D-Structure
https://pubchem.ncbi.nlm.nih.gov/compound/53957013#section=2D-Structure
https://pubchem.ncbi.nlm.nih.gov/compound/53954769#section=2D-Structure
https://pubchem.ncbi.nlm.nih.gov/compound/53954165#section=2D-Structure
https://pubchem.ncbi.nlm.nih.gov/compound/53941406#section=2D-Structure
https://pubchem.ncbi.nlm.nih.gov/compound/53938649#section=2D-Structure
https://pubchem.ncbi.nlm.nih.gov/compound/53931676#section=2D-Structure
https://pubchem.ncbi.nlm.nih.gov/compound/53929965#section=2D-Structure
https://pubchem.ncbi.nlm.nih.gov/compound/53928674#section=2D-Structure
https://pubchem.ncbi.nlm.nih.gov/compound/53927158#section=2D-Structure
https://pubchem.ncbi.nlm.nih.gov/compound/53922537#section=2D-Structure
https://pubchem.ncbi.nlm.nih.gov/compound/53918000#section=2D-Structure
https://pubchem.ncbi.nlm.nih.gov/compound/53916166#section=2D-Structure
https://pubchem.ncbi.nlm.nih.gov/compound/53912813#section=2D-Structure
https://pubchem.ncbi.nlm.nih.gov/compound/53909154#section=2D-Structure
https://pubchem.ncbi.nlm.nih.gov/compound/53906718#section=2D-Structure

https://pubchem.ncbi.nlm.nih.gov/compound/53905409#section=2D-Structure
https://pubchem.ncbi.nlm.nih.gov/compound/53901762#section=2D-Structure
https://pubchem.ncbi.nlm.nih.gov/compound/53898450#section=2D-Structure
https://pubchem.ncbi.nlm.nih.gov/compound/53894216#section=2D-Structure
https://pubchem.ncbi.nlm.nih.gov/compound/53883788#section=2D-Structure
https://pubchem.ncbi.nlm.nih.gov/compound/53880230#section=2D-Structure
https://pubchem.ncbi.nlm.nih.gov/compound/53876232#section=2D-Structure
https://pubchem.ncbi.nlm.nih.gov/compound/53867643#section=2D-Structure
https://pubchem.ncbi.nlm.nih.gov/compound/53864316#section=2D-Structure
https://pubchem.ncbi.nlm.nih.gov/compound/53863470#section=2D-Structure
https://pubchem.ncbi.nlm.nih.gov/compound/53861187#section=2D-Structure
https://pubchem.ncbi.nlm.nih.gov/compound/53860837#section=2D-Structure
https://pubchem.ncbi.nlm.nih.gov/compound/53855209#section=2D-Structure
https://pubchem.ncbi.nlm.nih.gov/compound/53844581#section=2D-Structure
https://pubchem.ncbi.nlm.nih.gov/compound/53844166#section=2D-Structure
https://pubchem.ncbi.nlm.nih.gov/compound/53839315#section=2D-Structure
https://pubchem.ncbi.nlm.nih.gov/compound/53838966#section=2D-Structure
https://pubchem.ncbi.nlm.nih.gov/compound/53838411#section=2D-Structure
https://pubchem.ncbi.nlm.nih.gov/compound/5383762#section=2D-Structure
https://pubchem.ncbi.nlm.nih.gov/compound/53835802#section=2D-Structure
https://pubchem.ncbi.nlm.nih.gov/compound/53835720#section=2D-Structure
https://pubchem.ncbi.nlm.nih.gov/compound/53823945#section=2D-Structure
https://pubchem.ncbi.nlm.nih.gov/compound/53819635#section=2D-Structure
https://pubchem.ncbi.nlm.nih.gov/compound/53815679#section=2D-Structure
https://pubchem.ncbi.nlm.nih.gov/compound/53815016#section=2D-Structure
https://pubchem.ncbi.nlm.nih.gov/compound/53805102#section=2D-Structure
https://pubchem.ncbi.nlm.nih.gov/compound/53792966#section=2D-Structure
https://pubchem.ncbi.nlm.nih.gov/compound/53788449#section=2D-Structure
https://pubchem.ncbi.nlm.nih.gov/compound/53783183#section=2D-Structure
https://pubchem.ncbi.nlm.nih.gov/compound/53783086#section=2D-Structure
https://pubchem.ncbi.nlm.nih.gov/compound/53777050#section=2D-Structure
https://pubchem.ncbi.nlm.nih.gov/compound/53775577#section=2D-Structure
https://pubchem.ncbi.nlm.nih.gov/compound/53764979#section=2D-Structure
https://pubchem.ncbi.nlm.nih.gov/compound/53763457#section=2D-Structure
https://pubchem.ncbi.nlm.nih.gov/compound/5375990#section=2D-Structure
https://pubchem.ncbi.nlm.nih.gov/compound/53755965#section=2D-Structure
https://pubchem.ncbi.nlm.nih.gov/compound/53752135#section=2D-Structure
https://pubchem.ncbi.nlm.nih.gov/compound/53751763#section=2D-Structure
https://pubchem.ncbi.nlm.nih.gov/compound/53749898#section=2D-Structure
https://pubchem.ncbi.nlm.nih.gov/compound/53744636#section=2D-Structure
https://pubchem.ncbi.nlm.nih.gov/compound/53744574#section=2D-Structure
https://pubchem.ncbi.nlm.nih.gov/compound/53738536#section=2D-Structure
https://pubchem.ncbi.nlm.nih.gov/compound/53728233#section=2D-Structure
https://pubchem.ncbi.nlm.nih.gov/compound/53721913#section=2D-Structure
https://pubchem.ncbi.nlm.nih.gov/compound/53719678#section=2D-Structure
https://pubchem.ncbi.nlm.nih.gov/compound/53710089#section=2D-Structure
https://pubchem.ncbi.nlm.nih.gov/compound/53707316#section=2D-Structure
https://pubchem.ncbi.nlm.nih.gov/compound/53706897#section=2D-Structure
https://pubchem.ncbi.nlm.nih.gov/compound/53706337#section=2D-Structure
https://pubchem.ncbi.nlm.nih.gov/compound/53703861#section=2D-Structure
https://pubchem.ncbi.nlm.nih.gov/compound/53699458#section=2D-Structure
https://pubchem.ncbi.nlm.nih.gov/compound/53697056#section=2D-Structure
https://pubchem.ncbi.nlm.nih.gov/compound/53693495#section=2D-Structure
https://pubchem.ncbi.nlm.nih.gov/compound/53692130#section=2D-Structure
https://pubchem.ncbi.nlm.nih.gov/compound/53691664#section=2D-Structure

https://pubchem.ncbi.nlm.nih.gov/compound/53690773#section=2D-Structure
https://pubchem.ncbi.nlm.nih.gov/compound/53688907#section=2D-Structure
https://pubchem.ncbi.nlm.nih.gov/compound/53687773#section=2D-Structure
https://pubchem.ncbi.nlm.nih.gov/compound/53681777#section=2D-Structure
https://pubchem.ncbi.nlm.nih.gov/compound/53681517#section=2D-Structure
https://pubchem.ncbi.nlm.nih.gov/compound/53680315#section=2D-Structure
https://pubchem.ncbi.nlm.nih.gov/compound/53677691#section=2D-Structure
https://pubchem.ncbi.nlm.nih.gov/compound/53677501#section=2D-Structure
https://pubchem.ncbi.nlm.nih.gov/compound/53675025#section=2D-Structure
https://pubchem.ncbi.nlm.nih.gov/compound/5367363#section=2D-Structure
https://pubchem.ncbi.nlm.nih.gov/compound/53670378#section=2D-Structure
https://pubchem.ncbi.nlm.nih.gov/compound/53666974#section=2D-Structure
https://pubchem.ncbi.nlm.nih.gov/compound/53666084#section=2D-Structure
https://pubchem.ncbi.nlm.nih.gov/compound/53665246#section=2D-Structure
https://pubchem.ncbi.nlm.nih.gov/compound/53661130#section=2D-Structure
https://pubchem.ncbi.nlm.nih.gov/compound/53659320#section=2D-Structure
https://pubchem.ncbi.nlm.nih.gov/compound/53658263#section=2D-Structure
https://pubchem.ncbi.nlm.nih.gov/compound/53649127#section=2D-Structure
https://pubchem.ncbi.nlm.nih.gov/compound/53648942#section=2D-Structure
https://pubchem.ncbi.nlm.nih.gov/compound/53643127#section=2D-Structure
https://pubchem.ncbi.nlm.nih.gov/compound/53642552#section=2D-Structure
https://pubchem.ncbi.nlm.nih.gov/compound/53641845#section=2D-Structure
https://pubchem.ncbi.nlm.nih.gov/compound/53635185#section=2D-Structure
https://pubchem.ncbi.nlm.nih.gov/compound/535982#section=2D-Structure
https://pubchem.ncbi.nlm.nih.gov/compound/5356767#section=2D-Structure
https://pubchem.ncbi.nlm.nih.gov/compound/53494355#section=2D-Structure
https://pubchem.ncbi.nlm.nih.gov/compound/53470894#section=2D-Structure
https://pubchem.ncbi.nlm.nih.gov/compound/53470609#section=2D-Structure
https://pubchem.ncbi.nlm.nih.gov/compound/53470568#section=2D-Structure
https://pubchem.ncbi.nlm.nih.gov/compound/534588#section=2D-Structure
https://pubchem.ncbi.nlm.nih.gov/compound/53441027#section=2D-Structure
https://pubchem.ncbi.nlm.nih.gov/compound/53440623#section=2D-Structure
https://pubchem.ncbi.nlm.nih.gov/compound/53438523#section=2D-Structure
https://pubchem.ncbi.nlm.nih.gov/compound/53438135#section=2D-Structure
https://pubchem.ncbi.nlm.nih.gov/compound/53436347#section=2D-Structure
https://pubchem.ncbi.nlm.nih.gov/compound/53435919#section=2D-Structure
https://pubchem.ncbi.nlm.nih.gov/compound/53435401#section=2D-Structure
https://pubchem.ncbi.nlm.nih.gov/compound/53431173#section=2D-Structure
https://pubchem.ncbi.nlm.nih.gov/compound/53424822#section=2D-Structure
https://pubchem.ncbi.nlm.nih.gov/compound/53424746#section=2D-Structure
https://pubchem.ncbi.nlm.nih.gov/compound/53423963#section=2D-Structure
https://pubchem.ncbi.nlm.nih.gov/compound/53423911#section=2D-Structure
https://pubchem.ncbi.nlm.nih.gov/compound/53420644#section=2D-Structure
https://pubchem.ncbi.nlm.nih.gov/compound/534185#section=2D-Structure
https://pubchem.ncbi.nlm.nih.gov/compound/5341377#section=2D-Structure
https://pubchem.ncbi.nlm.nih.gov/compound/53400461#section=2D-Structure
https://pubchem.ncbi.nlm.nih.gov/compound/53400400#section=2D-Structure
https://pubchem.ncbi.nlm.nih.gov/compound/53400278#section=2D-Structure
https://pubchem.ncbi.nlm.nih.gov/compound/533844#section=2D-Structure
https://pubchem.ncbi.nlm.nih.gov/compound/53379246#section=2D-Structure
https://pubchem.ncbi.nlm.nih.gov/compound/5325318#section=2D-Structure
https://pubchem.ncbi.nlm.nih.gov/compound/5325317#section=2D-Structure
https://pubchem.ncbi.nlm.nih.gov/compound/5311750#section=2D-Structure
https://pubchem.ncbi.nlm.nih.gov/compound/5311564#section=2D-Structure
https://pubchem.ncbi.nlm.nih.gov/compound/5306823#section=2D-Structure

https://pubchem.ncbi.nlm.nih.gov/compound/52988053#section=2D-Structure
https://pubchem.ncbi.nlm.nih.gov/compound/52963572#section=2D-Structure
https://pubchem.ncbi.nlm.nih.gov/compound/52944377#section=2D-Structure
https://pubchem.ncbi.nlm.nih.gov/compound/5257566#section=2D-Structure
https://pubchem.ncbi.nlm.nih.gov/compound/5256999#section=2D-Structure
https://pubchem.ncbi.nlm.nih.gov/compound/5255908#section=2D-Structure
https://pubchem.ncbi.nlm.nih.gov/compound/5255840#section=2D-Structure
https://pubchem.ncbi.nlm.nih.gov/compound/5246393#section=2D-Structure
https://pubchem.ncbi.nlm.nih.gov/compound/5242875#section=2D-Structure
https://pubchem.ncbi.nlm.nih.gov/compound/5232433#section=2D-Structure
https://pubchem.ncbi.nlm.nih.gov/compound/5231634#section=2D-Structure
https://pubchem.ncbi.nlm.nih.gov/compound/5231351#section=2D-Structure
https://pubchem.ncbi.nlm.nih.gov/compound/5225463#section=2D-Structure
https://pubchem.ncbi.nlm.nih.gov/compound/52232108#section=2D-Structure
https://pubchem.ncbi.nlm.nih.gov/compound/5220371#section=2D-Structure
https://pubchem.ncbi.nlm.nih.gov/compound/5214706#section=2D-Structure
https://pubchem.ncbi.nlm.nih.gov/compound/521200#section=2D-Structure
https://pubchem.ncbi.nlm.nih.gov/compound/51412952#section=2D-Structure
https://pubchem.ncbi.nlm.nih.gov/compound/51350331#section=2D-Structure
https://pubchem.ncbi.nlm.nih.gov/compound/51056395#section=2D-Structure
https://pubchem.ncbi.nlm.nih.gov/compound/50989474#section=2D-Structure
https://pubchem.ncbi.nlm.nih.gov/compound/50919272#section=2D-Structure
https://pubchem.ncbi.nlm.nih.gov/compound/4996739#section=2D-Structure
https://pubchem.ncbi.nlm.nih.gov/compound/4992792#section=2D-Structure
https://pubchem.ncbi.nlm.nih.gov/compound/4991822#section=2D-Structure
https://pubchem.ncbi.nlm.nih.gov/compound/49873536#section=2D-Structure
https://pubchem.ncbi.nlm.nih.gov/compound/49845918#section=2D-Structure
https://pubchem.ncbi.nlm.nih.gov/compound/49842552#section=2D-Structure
https://pubchem.ncbi.nlm.nih.gov/compound/49841083#section=2D-Structure
https://pubchem.ncbi.nlm.nih.gov/compound/49836493#section=2D-Structure
https://pubchem.ncbi.nlm.nih.gov/compound/49836492#section=2D-Structure
https://pubchem.ncbi.nlm.nih.gov/compound/494411#section=2D-Structure
https://pubchem.ncbi.nlm.nih.gov/compound/494410#section=2D-Structure
https://pubchem.ncbi.nlm.nih.gov/compound/4895990#section=2D-Structure
https://pubchem.ncbi.nlm.nih.gov/compound/4868274#section=2D-Structure
https://pubchem.ncbi.nlm.nih.gov/compound/484719#section=2D-Structure
https://pubchem.ncbi.nlm.nih.gov/compound/484715#section=2D-Structure
https://pubchem.ncbi.nlm.nih.gov/compound/484713#section=2D-Structure
https://pubchem.ncbi.nlm.nih.gov/compound/484707#section=2D-Structure
https://pubchem.ncbi.nlm.nih.gov/compound/484702#section=2D-Structure
https://pubchem.ncbi.nlm.nih.gov/compound/484700#section=2D-Structure
https://pubchem.ncbi.nlm.nih.gov/compound/46739861#section=2D-Structure
https://pubchem.ncbi.nlm.nih.gov/compound/46739859#section=2D-Structure
https://pubchem.ncbi.nlm.nih.gov/compound/46739858#section=2D-Structure
https://pubchem.ncbi.nlm.nih.gov/compound/46739854#section=2D-Structure
https://pubchem.ncbi.nlm.nih.gov/compound/46739853#section=2D-Structure
https://pubchem.ncbi.nlm.nih.gov/compound/46739847#section=2D-Structure
https://pubchem.ncbi.nlm.nih.gov/compound/46739844#section=2D-Structure
https://pubchem.ncbi.nlm.nih.gov/compound/46739841#section=2D-Structure
https://pubchem.ncbi.nlm.nih.gov/compound/4671514#section=2D-Structure
https://pubchem.ncbi.nlm.nih.gov/compound/4635105#section=2D-Structure
https://pubchem.ncbi.nlm.nih.gov/compound/46232479#section=2D-Structure
https://pubchem.ncbi.nlm.nih.gov/compound/46228150#section=2D-Structure
https://pubchem.ncbi.nlm.nih.gov/compound/46173012#section=2D-Structure
https://pubchem.ncbi.nlm.nih.gov/compound/4601010#section=2D-Structure

https://pubchem.ncbi.nlm.nih.gov/compound/4558355#section=2D-Structure
https://pubchem.ncbi.nlm.nih.gov/compound/45382144#section=2D-Structure
https://pubchem.ncbi.nlm.nih.gov/compound/4520255#section=2D-Structure
https://pubchem.ncbi.nlm.nih.gov/compound/4519244#section=2D-Structure
https://pubchem.ncbi.nlm.nih.gov/compound/45122595#section=2D-Structure
https://pubchem.ncbi.nlm.nih.gov/compound/45122565#section=2D-Structure
https://pubchem.ncbi.nlm.nih.gov/compound/45122561#section=2D-Structure
https://pubchem.ncbi.nlm.nih.gov/compound/45122550#section=2D-Structure
https://pubchem.ncbi.nlm.nih.gov/compound/45122549#section=2D-Structure
https://pubchem.ncbi.nlm.nih.gov/compound/45121713#section=2D-Structure
https://pubchem.ncbi.nlm.nih.gov/compound/45121711#section=2D-Structure
https://pubchem.ncbi.nlm.nih.gov/compound/45121710#section=2D-Structure
https://pubchem.ncbi.nlm.nih.gov/compound/45121708#section=2D-Structure
https://pubchem.ncbi.nlm.nih.gov/compound/45121707#section=2D-Structure
https://pubchem.ncbi.nlm.nih.gov/compound/45121602#section=2D-Structure
https://pubchem.ncbi.nlm.nih.gov/compound/45121601#section=2D-Structure
https://pubchem.ncbi.nlm.nih.gov/compound/45121598#section=2D-Structure
https://pubchem.ncbi.nlm.nih.gov/compound/45121593#section=2D-Structure
https://pubchem.ncbi.nlm.nih.gov/compound/45121589#section=2D-Structure
https://pubchem.ncbi.nlm.nih.gov/compound/45121587#section=2D-Structure
https://pubchem.ncbi.nlm.nih.gov/compound/45121571#section=2D-Structure
https://pubchem.ncbi.nlm.nih.gov/compound/45121557#section=2D-Structure
https://pubchem.ncbi.nlm.nih.gov/compound/45121550#section=2D-Structure
https://pubchem.ncbi.nlm.nih.gov/compound/45121159#section=2D-Structure
https://pubchem.ncbi.nlm.nih.gov/compound/45121150#section=2D-Structure
https://pubchem.ncbi.nlm.nih.gov/compound/45121139#section=2D-Structure
https://pubchem.ncbi.nlm.nih.gov/compound/45121137#section=2D-Structure
https://pubchem.ncbi.nlm.nih.gov/compound/45121132#section=2D-Structure
https://pubchem.ncbi.nlm.nih.gov/compound/45121129#section=2D-Structure
https://pubchem.ncbi.nlm.nih.gov/compound/45121126#section=2D-Structure
https://pubchem.ncbi.nlm.nih.gov/compound/45121125#section=2D-Structure
https://pubchem.ncbi.nlm.nih.gov/compound/45121124#section=2D-Structure
https://pubchem.ncbi.nlm.nih.gov/compound/45120988#section=2D-Structure
https://pubchem.ncbi.nlm.nih.gov/compound/45120860#section=2D-Structure
https://pubchem.ncbi.nlm.nih.gov/compound/45120825#section=2D-Structure
https://pubchem.ncbi.nlm.nih.gov/compound/45120765#section=2D-Structure
https://pubchem.ncbi.nlm.nih.gov/compound/45120652#section=2D-Structure
https://pubchem.ncbi.nlm.nih.gov/compound/45120289#section=2D-Structure
https://pubchem.ncbi.nlm.nih.gov/compound/45120288#section=2D-Structure
https://pubchem.ncbi.nlm.nih.gov/compound/45120285#section=2D-Structure
https://pubchem.ncbi.nlm.nih.gov/compound/45120283#section=2D-Structure
https://pubchem.ncbi.nlm.nih.gov/compound/45120281#section=2D-Structure
https://pubchem.ncbi.nlm.nih.gov/compound/45120280#section=2D-Structure
https://pubchem.ncbi.nlm.nih.gov/compound/45120278#section=2D-Structure
https://pubchem.ncbi.nlm.nih.gov/compound/45120276#section=2D-Structure
https://pubchem.ncbi.nlm.nih.gov/compound/45120274#section=2D-Structure
https://pubchem.ncbi.nlm.nih.gov/compound/45118100#section=2D-Structure
https://pubchem.ncbi.nlm.nih.gov/compound/45115648#section=2D-Structure
https://pubchem.ncbi.nlm.nih.gov/compound/45108281#section=2D-Structure
https://pubchem.ncbi.nlm.nih.gov/compound/45099662#section=2D-Structure
https://pubchem.ncbi.nlm.nih.gov/compound/45099233#section=2D-Structure
https://pubchem.ncbi.nlm.nih.gov/compound/45099225#section=2D-Structure
https://pubchem.ncbi.nlm.nih.gov/compound/45099214#section=2D-Structure
https://pubchem.ncbi.nlm.nih.gov/compound/45099213#section=2D-Structure
https://pubchem.ncbi.nlm.nih.gov/compound/45099204#section=2D-Structure

https://pubchem.ncbi.nlm.nih.gov/compound/45099203#section=2D-Structure
https://pubchem.ncbi.nlm.nih.gov/compound/45099193#section=2D-Structure
https://pubchem.ncbi.nlm.nih.gov/compound/45099183#section=2D-Structure
https://pubchem.ncbi.nlm.nih.gov/compound/45099179#section=2D-Structure
https://pubchem.ncbi.nlm.nih.gov/compound/45099169#section=2D-Structure
https://pubchem.ncbi.nlm.nih.gov/compound/45099166#section=2D-Structure
https://pubchem.ncbi.nlm.nih.gov/compound/45099161#section=2D-Structure
https://pubchem.ncbi.nlm.nih.gov/compound/45099157#section=2D-Structure
https://pubchem.ncbi.nlm.nih.gov/compound/45099154#section=2D-Structure
https://pubchem.ncbi.nlm.nih.gov/compound/45099145#section=2D-Structure
https://pubchem.ncbi.nlm.nih.gov/compound/45099135#section=2D-Structure
https://pubchem.ncbi.nlm.nih.gov/compound/45099041#section=2D-Structure
https://pubchem.ncbi.nlm.nih.gov/compound/45099022#section=2D-Structure
https://pubchem.ncbi.nlm.nih.gov/compound/45097591#section=2D-Structure
https://pubchem.ncbi.nlm.nih.gov/compound/45097212#section=2D-Structure
https://pubchem.ncbi.nlm.nih.gov/compound/45092970#section=2D-Structure
https://pubchem.ncbi.nlm.nih.gov/compound/45092690#section=2D-Structure
https://pubchem.ncbi.nlm.nih.gov/compound/45092520#section=2D-Structure
https://pubchem.ncbi.nlm.nih.gov/compound/45090385#section=2D-Structure
https://pubchem.ncbi.nlm.nih.gov/compound/45090352#section=2D-Structure
https://pubchem.ncbi.nlm.nih.gov/compound/45088162#section=2D-Structure
https://pubchem.ncbi.nlm.nih.gov/compound/45088093#section=2D-Structure
https://pubchem.ncbi.nlm.nih.gov/compound/45088065#section=2D-Structure
https://pubchem.ncbi.nlm.nih.gov/compound/45088058#section=2D-Structure
https://pubchem.ncbi.nlm.nih.gov/compound/45088055#section=2D-Structure
https://pubchem.ncbi.nlm.nih.gov/compound/45088005#section=2D-Structure
https://pubchem.ncbi.nlm.nih.gov/compound/45087969#section=2D-Structure
https://pubchem.ncbi.nlm.nih.gov/compound/45087961#section=2D-Structure
https://pubchem.ncbi.nlm.nih.gov/compound/45087948#section=2D-Structure
https://pubchem.ncbi.nlm.nih.gov/compound/45087608#section=2D-Structure
https://pubchem.ncbi.nlm.nih.gov/compound/45087601#section=2D-Structure
https://pubchem.ncbi.nlm.nih.gov/compound/45087565#section=2D-Structure
https://pubchem.ncbi.nlm.nih.gov/compound/45087560#section=2D-Structure
https://pubchem.ncbi.nlm.nih.gov/compound/45087472#section=2D-Structure
https://pubchem.ncbi.nlm.nih.gov/compound/45087444#section=2D-Structure
https://pubchem.ncbi.nlm.nih.gov/compound/45087439#section=2D-Structure
https://pubchem.ncbi.nlm.nih.gov/compound/45080515#section=2D-Structure
https://pubchem.ncbi.nlm.nih.gov/compound/45080512#section=2D-Structure
https://pubchem.ncbi.nlm.nih.gov/compound/45080510#section=2D-Structure
https://pubchem.ncbi.nlm.nih.gov/compound/45080488#section=2D-Structure
https://pubchem.ncbi.nlm.nih.gov/compound/45080388#section=2D-Structure
https://pubchem.ncbi.nlm.nih.gov/compound/45080345#section=2D-Structure
https://pubchem.ncbi.nlm.nih.gov/compound/45080340#section=2D-Structure
https://pubchem.ncbi.nlm.nih.gov/compound/45080333#section=2D-Structure
https://pubchem.ncbi.nlm.nih.gov/compound/45080303#section=2D-Structure
https://pubchem.ncbi.nlm.nih.gov/compound/45080268#section=2D-Structure
https://pubchem.ncbi.nlm.nih.gov/compound/45080244#section=2D-Structure
https://pubchem.ncbi.nlm.nih.gov/compound/45080157#section=2D-Structure
https://pubchem.ncbi.nlm.nih.gov/compound/45080154#section=2D-Structure
https://pubchem.ncbi.nlm.nih.gov/compound/45080091#section=2D-Structure
https://pubchem.ncbi.nlm.nih.gov/compound/45080023#section=2D-Structure
https://pubchem.ncbi.nlm.nih.gov/compound/45079983#section=2D-Structure
https://pubchem.ncbi.nlm.nih.gov/compound/45079978#section=2D-Structure
https://pubchem.ncbi.nlm.nih.gov/compound/45079970#section=2D-Structure
https://pubchem.ncbi.nlm.nih.gov/compound/45079951#section=2D-Structure

https://pubchem.ncbi.nlm.nih.gov/compound/45079939#section=2D-Structure
https://pubchem.ncbi.nlm.nih.gov/compound/45079920#section=2D-Structure
https://pubchem.ncbi.nlm.nih.gov/compound/45079832#section=2D-Structure
https://pubchem.ncbi.nlm.nih.gov/compound/45079789#section=2D-Structure
https://pubchem.ncbi.nlm.nih.gov/compound/45079778#section=2D-Structure
https://pubchem.ncbi.nlm.nih.gov/compound/45079761#section=2D-Structure
https://pubchem.ncbi.nlm.nih.gov/compound/45079760#section=2D-Structure
https://pubchem.ncbi.nlm.nih.gov/compound/45079721#section=2D-Structure
https://pubchem.ncbi.nlm.nih.gov/compound/45079678#section=2D-Structure
https://pubchem.ncbi.nlm.nih.gov/compound/45079492#section=2D-Structure
https://pubchem.ncbi.nlm.nih.gov/compound/45079446#section=2D-Structure
https://pubchem.ncbi.nlm.nih.gov/compound/45079143#section=2D-Structure
https://pubchem.ncbi.nlm.nih.gov/compound/45078661#section=2D-Structure
https://pubchem.ncbi.nlm.nih.gov/compound/45078561#section=2D-Structure
https://pubchem.ncbi.nlm.nih.gov/compound/45078560#section=2D-Structure
https://pubchem.ncbi.nlm.nih.gov/compound/45078539#section=2D-Structure
https://pubchem.ncbi.nlm.nih.gov/compound/45078522#section=2D-Structure
https://pubchem.ncbi.nlm.nih.gov/compound/45078503#section=2D-Structure
https://pubchem.ncbi.nlm.nih.gov/compound/45078486#section=2D-Structure
https://pubchem.ncbi.nlm.nih.gov/compound/45078450#section=2D-Structure
https://pubchem.ncbi.nlm.nih.gov/compound/45078411#section=2D-Structure
https://pubchem.ncbi.nlm.nih.gov/compound/45078404#section=2D-Structure
https://pubchem.ncbi.nlm.nih.gov/compound/45078387#section=2D-Structure
https://pubchem.ncbi.nlm.nih.gov/compound/45078158#section=2D-Structure
https://pubchem.ncbi.nlm.nih.gov/compound/45078034#section=2D-Structure
https://pubchem.ncbi.nlm.nih.gov/compound/45077953#section=2D-Structure
https://pubchem.ncbi.nlm.nih.gov/compound/45077912#section=2D-Structure
https://pubchem.ncbi.nlm.nih.gov/compound/45077908#section=2D-Structure
https://pubchem.ncbi.nlm.nih.gov/compound/45077826#section=2D-Structure
https://pubchem.ncbi.nlm.nih.gov/compound/45077798#section=2D-Structure
https://pubchem.ncbi.nlm.nih.gov/compound/45077700#section=2D-Structure
https://pubchem.ncbi.nlm.nih.gov/compound/45077697#section=2D-Structure
https://pubchem.ncbi.nlm.nih.gov/compound/45077684#section=2D-Structure
https://pubchem.ncbi.nlm.nih.gov/compound/45077595#section=2D-Structure
https://pubchem.ncbi.nlm.nih.gov/compound/45077556#section=2D-Structure
https://pubchem.ncbi.nlm.nih.gov/compound/45077555#section=2D-Structure
https://pubchem.ncbi.nlm.nih.gov/compound/45077554#section=2D-Structure
https://pubchem.ncbi.nlm.nih.gov/compound/45077552#section=2D-Structure
https://pubchem.ncbi.nlm.nih.gov/compound/45077551#section=2D-Structure
https://pubchem.ncbi.nlm.nih.gov/compound/45077546#section=2D-Structure
https://pubchem.ncbi.nlm.nih.gov/compound/45077471#section=2D-Structure
https://pubchem.ncbi.nlm.nih.gov/compound/45077401#section=2D-Structure
https://pubchem.ncbi.nlm.nih.gov/compound/45077372#section=2D-Structure
https://pubchem.ncbi.nlm.nih.gov/compound/45077210#section=2D-Structure
https://pubchem.ncbi.nlm.nih.gov/compound/45077102#section=2D-Structure
https://pubchem.ncbi.nlm.nih.gov/compound/45077074#section=2D-Structure
https://pubchem.ncbi.nlm.nih.gov/compound/45077050#section=2D-Structure
https://pubchem.ncbi.nlm.nih.gov/compound/45077049#section=2D-Structure
https://pubchem.ncbi.nlm.nih.gov/compound/45077039#section=2D-Structure
https://pubchem.ncbi.nlm.nih.gov/compound/45077038#section=2D-Structure
https://pubchem.ncbi.nlm.nih.gov/compound/45077037#section=2D-Structure
https://pubchem.ncbi.nlm.nih.gov/compound/45077036#section=2D-Structure
https://pubchem.ncbi.nlm.nih.gov/compound/45077035#section=2D-Structure
https://pubchem.ncbi.nlm.nih.gov/compound/45076955#section=2D-Structure
https://pubchem.ncbi.nlm.nih.gov/compound/45074556#section=2D-Structure

https://pubchem.ncbi.nlm.nih.gov/compound/45050288#section=2D-Structure
https://pubchem.ncbi.nlm.nih.gov/compound/45039516#section=2D-Structure
https://pubchem.ncbi.nlm.nih.gov/compound/45036147#section=2D-Structure
https://pubchem.ncbi.nlm.nih.gov/compound/45025980#section=2D-Structure
https://pubchem.ncbi.nlm.nih.gov/compound/45025978#section=2D-Structure
https://pubchem.ncbi.nlm.nih.gov/compound/45025758#section=2D-Structure
https://pubchem.ncbi.nlm.nih.gov/compound/45025452#section=2D-Structure
https://pubchem.ncbi.nlm.nih.gov/compound/45024318#section=2D-Structure
https://pubchem.ncbi.nlm.nih.gov/compound/45023718#section=2D-Structure
https://pubchem.ncbi.nlm.nih.gov/compound/44826101#section=2D-Structure
https://pubchem.ncbi.nlm.nih.gov/compound/44556520#section=2D-Structure
https://pubchem.ncbi.nlm.nih.gov/compound/44483312#section=2D-Structure
https://pubchem.ncbi.nlm.nih.gov/compound/444211#section=2D-Structure
https://pubchem.ncbi.nlm.nih.gov/compound/444084#section=2D-Structure
https://pubchem.ncbi.nlm.nih.gov/compound/44388235#section=2D-Structure
https://pubchem.ncbi.nlm.nih.gov/compound/44347218#section=2D-Structure
https://pubchem.ncbi.nlm.nih.gov/compound/44268587#section=2D-Structure
https://pubchem.ncbi.nlm.nih.gov/compound/44227411#section=2D-Structure
https://pubchem.ncbi.nlm.nih.gov/compound/44226989#section=2D-Structure
https://pubchem.ncbi.nlm.nih.gov/compound/4421616#section=2D-Structure
https://pubchem.ncbi.nlm.nih.gov/compound/44181984#section=2D-Structure
https://pubchem.ncbi.nlm.nih.gov/compound/440728#section=2D-Structure
https://pubchem.ncbi.nlm.nih.gov/compound/4401630#section=2D-Structure
https://pubchem.ncbi.nlm.nih.gov/compound/439016#section=2D-Structure
https://pubchem.ncbi.nlm.nih.gov/compound/438981#section=2D-Structure
https://pubchem.ncbi.nlm.nih.gov/compound/438284#section=2D-Structure
https://pubchem.ncbi.nlm.nih.gov/compound/43828337#section=2D-Structure
https://pubchem.ncbi.nlm.nih.gov/compound/4372587#section=2D-Structure
https://pubchem.ncbi.nlm.nih.gov/compound/437049#section=2D-Structure
https://pubchem.ncbi.nlm.nih.gov/compound/436854#section=2D-Structure
https://pubchem.ncbi.nlm.nih.gov/compound/4347237#section=2D-Structure
https://pubchem.ncbi.nlm.nih.gov/compound/433577#section=2D-Structure
https://pubchem.ncbi.nlm.nih.gov/compound/4312984#section=2D-Structure
https://pubchem.ncbi.nlm.nih.gov/compound/4306964#section=2D-Structure
https://pubchem.ncbi.nlm.nih.gov/compound/430558#section=2D-Structure
https://pubchem.ncbi.nlm.nih.gov/compound/428955#section=2D-Structure
https://pubchem.ncbi.nlm.nih.gov/compound/4281287#section=2D-Structure
https://pubchem.ncbi.nlm.nih.gov/compound/426699#section=2D-Structure
https://pubchem.ncbi.nlm.nih.gov/compound/42605710#section=2D-Structure
https://pubchem.ncbi.nlm.nih.gov/compound/421754#section=2D-Structure
https://pubchem.ncbi.nlm.nih.gov/compound/4212173#section=2D-Structure
https://pubchem.ncbi.nlm.nih.gov/compound/420971#section=2D-Structure
https://pubchem.ncbi.nlm.nih.gov/compound/419668#section=2D-Structure
https://pubchem.ncbi.nlm.nih.gov/compound/419605#section=2D-Structure
https://pubchem.ncbi.nlm.nih.gov/compound/4170769#section=2D-Structure
https://pubchem.ncbi.nlm.nih.gov/compound/416303#section=2D-Structure
https://pubchem.ncbi.nlm.nih.gov/compound/416302#section=2D-Structure
https://pubchem.ncbi.nlm.nih.gov/compound/4127804#section=2D-Structure
https://pubchem.ncbi.nlm.nih.gov/compound/4121342#section=2D-Structure
https://pubchem.ncbi.nlm.nih.gov/compound/411278#section=2D-Structure
https://pubchem.ncbi.nlm.nih.gov/compound/4101807#section=2D-Structure
https://pubchem.ncbi.nlm.nih.gov/compound/409149#section=2D-Structure
https://pubchem.ncbi.nlm.nih.gov/compound/408333#section=2D-Structure
https://pubchem.ncbi.nlm.nih.gov/compound/4080350#section=2D-Structure
https://pubchem.ncbi.nlm.nih.gov/compound/4067664#section=2D-Structure

https://pubchem.ncbi.nlm.nih.gov/compound/406028#section=2D-Structure
https://pubchem.ncbi.nlm.nih.gov/compound/39733513#section=2D-Structure
https://pubchem.ncbi.nlm.nih.gov/compound/39732182#section=2D-Structure
https://pubchem.ncbi.nlm.nih.gov/compound/395809#section=2D-Structure
https://pubchem.ncbi.nlm.nih.gov/compound/39493#section=2D-Structure
https://pubchem.ncbi.nlm.nih.gov/compound/394342#section=2D-Structure
https://pubchem.ncbi.nlm.nih.gov/compound/39368#section=2D-Structure
https://pubchem.ncbi.nlm.nih.gov/compound/3891512#section=2D-Structure
https://pubchem.ncbi.nlm.nih.gov/compound/388097#section=2D-Structure
https://pubchem.ncbi.nlm.nih.gov/compound/387475#section=2D-Structure
https://pubchem.ncbi.nlm.nih.gov/compound/386998#section=2D-Structure
https://pubchem.ncbi.nlm.nih.gov/compound/385100#section=2D-Structure
https://pubchem.ncbi.nlm.nih.gov/compound/3840595#section=2D-Structure
https://pubchem.ncbi.nlm.nih.gov/compound/3824753#section=2D-Structure
https://pubchem.ncbi.nlm.nih.gov/compound/3815759#section=2D-Structure
https://pubchem.ncbi.nlm.nih.gov/compound/379727#section=2D-Structure
https://pubchem.ncbi.nlm.nih.gov/compound/379714#section=2D-Structure
https://pubchem.ncbi.nlm.nih.gov/compound/379417#section=2D-Structure
https://pubchem.ncbi.nlm.nih.gov/compound/378710#section=2D-Structure
https://pubchem.ncbi.nlm.nih.gov/compound/3782080#section=2D-Structure
https://pubchem.ncbi.nlm.nih.gov/compound/375636#section=2D-Structure
https://pubchem.ncbi.nlm.nih.gov/compound/3755009#section=2D-Structure
https://pubchem.ncbi.nlm.nih.gov/compound/3735986#section=2D-Structure
https://pubchem.ncbi.nlm.nih.gov/compound/3735416#section=2D-Structure
https://pubchem.ncbi.nlm.nih.gov/compound/3725167#section=2D-Structure
https://pubchem.ncbi.nlm.nih.gov/compound/3723025#section=2D-Structure
https://pubchem.ncbi.nlm.nih.gov/compound/371020#section=2D-Structure
https://pubchem.ncbi.nlm.nih.gov/compound/3709660#section=2D-Structure
https://pubchem.ncbi.nlm.nih.gov/compound/3701502#section=2D-Structure
https://pubchem.ncbi.nlm.nih.gov/compound/369392#section=2D-Structure
https://pubchem.ncbi.nlm.nih.gov/compound/369391#section=2D-Structure
https://pubchem.ncbi.nlm.nih.gov/compound/369390#section=2D-Structure
https://pubchem.ncbi.nlm.nih.gov/compound/369389#section=2D-Structure
https://pubchem.ncbi.nlm.nih.gov/compound/369388#section=2D-Structure
https://pubchem.ncbi.nlm.nih.gov/compound/369387#section=2D-Structure
https://pubchem.ncbi.nlm.nih.gov/compound/36917#section=2D-Structure
https://pubchem.ncbi.nlm.nih.gov/compound/368744#section=2D-Structure
https://pubchem.ncbi.nlm.nih.gov/compound/368585#section=2D-Structure
https://pubchem.ncbi.nlm.nih.gov/compound/367728#section=2D-Structure
https://pubchem.ncbi.nlm.nih.gov/compound/367482#section=2D-Structure
https://pubchem.ncbi.nlm.nih.gov/compound/366821#section=2D-Structure
https://pubchem.ncbi.nlm.nih.gov/compound/363742#section=2D-Structure
https://pubchem.ncbi.nlm.nih.gov/compound/362769#section=2D-Structure
https://pubchem.ncbi.nlm.nih.gov/compound/362381#section=2D-Structure
https://pubchem.ncbi.nlm.nih.gov/compound/3583588#section=2D-Structure
https://pubchem.ncbi.nlm.nih.gov/compound/354840#section=2D-Structure
https://pubchem.ncbi.nlm.nih.gov/compound/3540653#section=2D-Structure
https://pubchem.ncbi.nlm.nih.gov/compound/3526710#section=2D-Structure
https://pubchem.ncbi.nlm.nih.gov/compound/3521820#section=2D-Structure
https://pubchem.ncbi.nlm.nih.gov/compound/350894#section=2D-Structure
https://pubchem.ncbi.nlm.nih.gov/compound/346483#section=2D-Structure
https://pubchem.ncbi.nlm.nih.gov/compound/3452617#section=2D-Structure
https://pubchem.ncbi.nlm.nih.gov/compound/3443569#section=2D-Structure
https://pubchem.ncbi.nlm.nih.gov/compound/343245#section=2D-Structure
https://pubchem.ncbi.nlm.nih.gov/compound/342343#section=2D-Structure

https://pubchem.ncbi.nlm.nih.gov/compound/3397381#section=2D-Structure
https://pubchem.ncbi.nlm.nih.gov/compound/339232#section=2D-Structure
https://pubchem.ncbi.nlm.nih.gov/compound/338001#section=2D-Structure
https://pubchem.ncbi.nlm.nih.gov/compound/3365515#section=2D-Structure
https://pubchem.ncbi.nlm.nih.gov/compound/334433#section=2D-Structure
https://pubchem.ncbi.nlm.nih.gov/compound/331847#section=2D-Structure
https://pubchem.ncbi.nlm.nih.gov/compound/3315732#section=2D-Structure
https://pubchem.ncbi.nlm.nih.gov/compound/330839#section=2D-Structure
https://pubchem.ncbi.nlm.nih.gov/compound/328944#section=2D-Structure
https://pubchem.ncbi.nlm.nih.gov/compound/3276928#section=2D-Structure
https://pubchem.ncbi.nlm.nih.gov/compound/3273117#section=2D-Structure
https://pubchem.ncbi.nlm.nih.gov/compound/327058#section=2D-Structure
https://pubchem.ncbi.nlm.nih.gov/compound/327037#section=2D-Structure
https://pubchem.ncbi.nlm.nih.gov/compound/327035#section=2D-Structure
https://pubchem.ncbi.nlm.nih.gov/compound/3269144#section=2D-Structure
https://pubchem.ncbi.nlm.nih.gov/compound/3241830#section=2D-Structure
https://pubchem.ncbi.nlm.nih.gov/compound/3239837#section=2D-Structure
https://pubchem.ncbi.nlm.nih.gov/compound/3238284#section=2D-Structure
https://pubchem.ncbi.nlm.nih.gov/compound/320652#section=2D-Structure
https://pubchem.ncbi.nlm.nih.gov/compound/318085#section=2D-Structure
https://pubchem.ncbi.nlm.nih.gov/compound/314945#section=2D-Structure
https://pubchem.ncbi.nlm.nih.gov/compound/313608#section=2D-Structure
https://pubchem.ncbi.nlm.nih.gov/compound/312775#section=2D-Structure
https://pubchem.ncbi.nlm.nih.gov/compound/3075494#section=2D-Structure
https://pubchem.ncbi.nlm.nih.gov/compound/3075493#section=2D-Structure
https://pubchem.ncbi.nlm.nih.gov/compound/3075492#section=2D-Structure
https://pubchem.ncbi.nlm.nih.gov/compound/3075490#section=2D-Structure
https://pubchem.ncbi.nlm.nih.gov/compound/3075489#section=2D-Structure
https://pubchem.ncbi.nlm.nih.gov/compound/3075488#section=2D-Structure
https://pubchem.ncbi.nlm.nih.gov/compound/3041164#section=2D-Structure
https://pubchem.ncbi.nlm.nih.gov/compound/3034120#section=2D-Structure
https://pubchem.ncbi.nlm.nih.gov/compound/3017169#section=2D-Structure
https://pubchem.ncbi.nlm.nih.gov/compound/3015673#section=2D-Structure
https://pubchem.ncbi.nlm.nih.gov/compound/3013077#section=2D-Structure
https://pubchem.ncbi.nlm.nih.gov/compound/3013076#section=2D-Structure
https://pubchem.ncbi.nlm.nih.gov/compound/3004193#section=2D-Structure
https://pubchem.ncbi.nlm.nih.gov/compound/3003952#section=2D-Structure
https://pubchem.ncbi.nlm.nih.gov/compound/29972023#section=2D-Structure
https://pubchem.ncbi.nlm.nih.gov/compound/299520#section=2D-Structure
https://pubchem.ncbi.nlm.nih.gov/compound/299519#section=2D-Structure
https://pubchem.ncbi.nlm.nih.gov/compound/29949692#section=2D-Structure
https://pubchem.ncbi.nlm.nih.gov/compound/297897#section=2D-Structure
https://pubchem.ncbi.nlm.nih.gov/compound/293612#section=2D-Structure
https://pubchem.ncbi.nlm.nih.gov/compound/292773#section=2D-Structure
https://pubchem.ncbi.nlm.nih.gov/compound/292772#section=2D-Structure
https://pubchem.ncbi.nlm.nih.gov/compound/29244#section=2D-Structure
https://pubchem.ncbi.nlm.nih.gov/compound/292387#section=2D-Structure
https://pubchem.ncbi.nlm.nih.gov/compound/29049#section=2D-Structure
https://pubchem.ncbi.nlm.nih.gov/compound/288392#section=2D-Structure
https://pubchem.ncbi.nlm.nih.gov/compound/288151#section=2D-Structure
https://pubchem.ncbi.nlm.nih.gov/compound/28809373#section=2D-Structure
https://pubchem.ncbi.nlm.nih.gov/compound/28809349#section=2D-Structure
https://pubchem.ncbi.nlm.nih.gov/compound/28809338#section=2D-Structure
https://pubchem.ncbi.nlm.nih.gov/compound/28809336#section=2D-Structure
https://pubchem.ncbi.nlm.nih.gov/compound/28809335#section=2D-Structure

https://pubchem.ncbi.nlm.nih.gov/compound/28809334#section=2D-Structure
https://pubchem.ncbi.nlm.nih.gov/compound/28809333#section=2D-Structure
https://pubchem.ncbi.nlm.nih.gov/compound/28809332#section=2D-Structure
https://pubchem.ncbi.nlm.nih.gov/compound/28809329#section=2D-Structure
https://pubchem.ncbi.nlm.nih.gov/compound/28809326#section=2D-Structure
https://pubchem.ncbi.nlm.nih.gov/compound/28809320#section=2D-Structure
https://pubchem.ncbi.nlm.nih.gov/compound/287397#section=2D-Structure
https://pubchem.ncbi.nlm.nih.gov/compound/2849444#section=2D-Structure
https://pubchem.ncbi.nlm.nih.gov/compound/28470535#section=2D-Structure
https://pubchem.ncbi.nlm.nih.gov/compound/2794543#section=2D-Structure
https://pubchem.ncbi.nlm.nih.gov/compound/279439#section=2D-Structure
https://pubchem.ncbi.nlm.nih.gov/compound/27814#section=2D-Structure
https://pubchem.ncbi.nlm.nih.gov/compound/2777964#section=2D-Structure
https://pubchem.ncbi.nlm.nih.gov/compound/27752#section=2D-Structure
https://pubchem.ncbi.nlm.nih.gov/compound/27722884#section=2D-Structure
https://pubchem.ncbi.nlm.nih.gov/compound/2751990#section=2D-Structure
https://pubchem.ncbi.nlm.nih.gov/compound/2751922#section=2D-Structure
https://pubchem.ncbi.nlm.nih.gov/compound/2751879#section=2D-Structure
https://pubchem.ncbi.nlm.nih.gov/compound/2748984#section=2D-Structure
https://pubchem.ncbi.nlm.nih.gov/compound/2737100#section=2D-Structure
https://pubchem.ncbi.nlm.nih.gov/compound/2736920#section=2D-Structure
https://pubchem.ncbi.nlm.nih.gov/compound/273664#section=2D-Structure
https://pubchem.ncbi.nlm.nih.gov/compound/2735385#section=2D-Structure
https://pubchem.ncbi.nlm.nih.gov/compound/2728508#section=2D-Structure
https://pubchem.ncbi.nlm.nih.gov/compound/2721946#section=2D-Structure
https://pubchem.ncbi.nlm.nih.gov/compound/2721945#section=2D-Structure
https://pubchem.ncbi.nlm.nih.gov/compound/27036331#section=2D-Structure
https://pubchem.ncbi.nlm.nih.gov/compound/26728#section=2D-Structure
https://pubchem.ncbi.nlm.nih.gov/compound/26446#section=2D-Structure
https://pubchem.ncbi.nlm.nih.gov/compound/264285#section=2D-Structure
https://pubchem.ncbi.nlm.nih.gov/compound/258131#section=2D-Structure
https://pubchem.ncbi.nlm.nih.gov/compound/255114#section=2D-Structure
https://pubchem.ncbi.nlm.nih.gov/compound/253579#section=2D-Structure
https://pubchem.ncbi.nlm.nih.gov/compound/25225323#section=2D-Structure
https://pubchem.ncbi.nlm.nih.gov/compound/25225143#section=2D-Structure
https://pubchem.ncbi.nlm.nih.gov/compound/25224773#section=2D-Structure
https://pubchem.ncbi.nlm.nih.gov/compound/25224589#section=2D-Structure
https://pubchem.ncbi.nlm.nih.gov/compound/25154441#section=2D-Structure
https://pubchem.ncbi.nlm.nih.gov/compound/25138707#section=2D-Structure
https://pubchem.ncbi.nlm.nih.gov/compound/25136603#section=2D-Structure
https://pubchem.ncbi.nlm.nih.gov/compound/25135249#section=2D-Structure
https://pubchem.ncbi.nlm.nih.gov/compound/25135248#section=2D-Structure
https://pubchem.ncbi.nlm.nih.gov/compound/25044854#section=2D-Structure
https://pubchem.ncbi.nlm.nih.gov/compound/25022001#section=2D-Structure
https://pubchem.ncbi.nlm.nih.gov/compound/25021591#section=2D-Structure
https://pubchem.ncbi.nlm.nih.gov/compound/24977578#section=2D-Structure
https://pubchem.ncbi.nlm.nih.gov/compound/24977287#section=2D-Structure
https://pubchem.ncbi.nlm.nih.gov/compound/24974326#section=2D-Structure
https://pubchem.ncbi.nlm.nih.gov/compound/248808#section=2D-Structure
https://pubchem.ncbi.nlm.nih.gov/compound/24869788#section=2D-Structure
https://pubchem.ncbi.nlm.nih.gov/compound/24867599#section=2D-Structure
https://pubchem.ncbi.nlm.nih.gov/compound/24812680#section=2D-Structure
https://pubchem.ncbi.nlm.nih.gov/compound/24800382#section=2D-Structure
https://pubchem.ncbi.nlm.nih.gov/compound/24796434#section=2D-Structure
https://pubchem.ncbi.nlm.nih.gov/compound/24796433#section=2D-Structure

https://pubchem.ncbi.nlm.nih.gov/compound/24773136#section=2D-Structure
https://pubchem.ncbi.nlm.nih.gov/compound/24752218#section=2D-Structure
https://pubchem.ncbi.nlm.nih.gov/compound/245990#section=2D-Structure
https://pubchem.ncbi.nlm.nih.gov/compound/245674#section=2D-Structure
https://pubchem.ncbi.nlm.nih.gov/compound/24276229#section=2D-Structure
https://pubchem.ncbi.nlm.nih.gov/compound/24276227#section=2D-Structure
https://pubchem.ncbi.nlm.nih.gov/compound/24276226#section=2D-Structure
https://pubchem.ncbi.nlm.nih.gov/compound/24200563#section=2D-Structure
https://pubchem.ncbi.nlm.nih.gov/compound/24183780#section=2D-Structure
https://pubchem.ncbi.nlm.nih.gov/compound/241778#section=2D-Structure
https://pubchem.ncbi.nlm.nih.gov/compound/24158690#section=2D-Structure
https://pubchem.ncbi.nlm.nih.gov/compound/24045767#section=2D-Structure
https://pubchem.ncbi.nlm.nih.gov/compound/23991491#section=2D-Structure
https://pubchem.ncbi.nlm.nih.gov/compound/23964457#section=2D-Structure
https://pubchem.ncbi.nlm.nih.gov/compound/23882121#section=2D-Structure
https://pubchem.ncbi.nlm.nih.gov/compound/236710#section=2D-Structure
https://pubchem.ncbi.nlm.nih.gov/compound/23644010#section=2D-Structure
https://pubchem.ncbi.nlm.nih.gov/compound/23638151#section=2D-Structure
https://pubchem.ncbi.nlm.nih.gov/compound/23637711#section=2D-Structure
https://pubchem.ncbi.nlm.nih.gov/compound/23620297#section=2D-Structure
https://pubchem.ncbi.nlm.nih.gov/compound/23619769#section=2D-Structure
https://pubchem.ncbi.nlm.nih.gov/compound/23619683#section=2D-Structure
https://pubchem.ncbi.nlm.nih.gov/compound/23619369#section=2D-Structure
https://pubchem.ncbi.nlm.nih.gov/compound/23618291#section=2D-Structure
https://pubchem.ncbi.nlm.nih.gov/compound/23617693#section=2D-Structure
https://pubchem.ncbi.nlm.nih.gov/compound/23616197#section=2D-Structure
https://pubchem.ncbi.nlm.nih.gov/compound/23596803#section=2D-Structure
https://pubchem.ncbi.nlm.nih.gov/compound/23568527#section=2D-Structure
https://pubchem.ncbi.nlm.nih.gov/compound/23568001#section=2D-Structure
https://pubchem.ncbi.nlm.nih.gov/compound/23555571#section=2D-Structure
https://pubchem.ncbi.nlm.nih.gov/compound/23555570#section=2D-Structure
https://pubchem.ncbi.nlm.nih.gov/compound/23542596#section=2D-Structure
https://pubchem.ncbi.nlm.nih.gov/compound/23541594#section=2D-Structure
https://pubchem.ncbi.nlm.nih.gov/compound/23534557#section=2D-Structure
https://pubchem.ncbi.nlm.nih.gov/compound/23511539#section=2D-Structure
https://pubchem.ncbi.nlm.nih.gov/compound/23503296#section=2D-Structure
https://pubchem.ncbi.nlm.nih.gov/compound/23500243#section=2D-Structure
https://pubchem.ncbi.nlm.nih.gov/compound/23500242#section=2D-Structure
https://pubchem.ncbi.nlm.nih.gov/compound/23498543#section=2D-Structure
https://pubchem.ncbi.nlm.nih.gov/compound/23498208#section=2D-Structure
https://pubchem.ncbi.nlm.nih.gov/compound/23498199#section=2D-Structure
https://pubchem.ncbi.nlm.nih.gov/compound/234722#section=2D-Structure
https://pubchem.ncbi.nlm.nih.gov/compound/23470829#section=2D-Structure
https://pubchem.ncbi.nlm.nih.gov/compound/23467291#section=2D-Structure
https://pubchem.ncbi.nlm.nih.gov/compound/23459860#section=2D-Structure
https://pubchem.ncbi.nlm.nih.gov/compound/23456794#section=2D-Structure
https://pubchem.ncbi.nlm.nih.gov/compound/23442908#section=2D-Structure
https://pubchem.ncbi.nlm.nih.gov/compound/23433547#section=2D-Structure
https://pubchem.ncbi.nlm.nih.gov/compound/23431560#section=2D-Structure
https://pubchem.ncbi.nlm.nih.gov/compound/234270#section=2D-Structure
https://pubchem.ncbi.nlm.nih.gov/compound/23423830#section=2D-Structure
https://pubchem.ncbi.nlm.nih.gov/compound/23423829#section=2D-Structure
https://pubchem.ncbi.nlm.nih.gov/compound/23422232#section=2D-Structure
https://pubchem.ncbi.nlm.nih.gov/compound/23422231#section=2D-Structure
https://pubchem.ncbi.nlm.nih.gov/compound/23422153#section=2D-Structure

https://pubchem.ncbi.nlm.nih.gov/compound/23422143#section=2D-Structure
https://pubchem.ncbi.nlm.nih.gov/compound/23422115#section=2D-Structure
https://pubchem.ncbi.nlm.nih.gov/compound/23422114#section=2D-Structure
https://pubchem.ncbi.nlm.nih.gov/compound/23403165#section=2D-Structure
https://pubchem.ncbi.nlm.nih.gov/compound/23402225#section=2D-Structure
https://pubchem.ncbi.nlm.nih.gov/compound/23395159#section=2D-Structure
https://pubchem.ncbi.nlm.nih.gov/compound/23393435#section=2D-Structure
https://pubchem.ncbi.nlm.nih.gov/compound/23392133#section=2D-Structure
https://pubchem.ncbi.nlm.nih.gov/compound/23389121#section=2D-Structure
https://pubchem.ncbi.nlm.nih.gov/compound/23379913#section=2D-Structure
https://pubchem.ncbi.nlm.nih.gov/compound/23379902#section=2D-Structure
https://pubchem.ncbi.nlm.nih.gov/compound/23368952#section=2D-Structure
https://pubchem.ncbi.nlm.nih.gov/compound/23363345#section=2D-Structure
https://pubchem.ncbi.nlm.nih.gov/compound/23362699#section=2D-Structure
https://pubchem.ncbi.nlm.nih.gov/compound/23361993#section=2D-Structure
https://pubchem.ncbi.nlm.nih.gov/compound/23361685#section=2D-Structure
https://pubchem.ncbi.nlm.nih.gov/compound/23355019#section=2D-Structure
https://pubchem.ncbi.nlm.nih.gov/compound/23350232#section=2D-Structure
https://pubchem.ncbi.nlm.nih.gov/compound/23337252#section=2D-Structure
https://pubchem.ncbi.nlm.nih.gov/compound/23337151#section=2D-Structure
https://pubchem.ncbi.nlm.nih.gov/compound/23336415#section=2D-Structure
https://pubchem.ncbi.nlm.nih.gov/compound/23335368#section=2D-Structure
https://pubchem.ncbi.nlm.nih.gov/compound/23335357#section=2D-Structure
https://pubchem.ncbi.nlm.nih.gov/compound/23333580#section=2D-Structure
https://pubchem.ncbi.nlm.nih.gov/compound/23323007#section=2D-Structure
https://pubchem.ncbi.nlm.nih.gov/compound/23321505#section=2D-Structure
https://pubchem.ncbi.nlm.nih.gov/compound/23315661#section=2D-Structure
https://pubchem.ncbi.nlm.nih.gov/compound/23312028#section=2D-Structure
https://pubchem.ncbi.nlm.nih.gov/compound/23303932#section=2D-Structure
https://pubchem.ncbi.nlm.nih.gov/compound/23296989#section=2D-Structure
https://pubchem.ncbi.nlm.nih.gov/compound/23295414#section=2D-Structure
https://pubchem.ncbi.nlm.nih.gov/compound/23295256#section=2D-Structure
https://pubchem.ncbi.nlm.nih.gov/compound/23295169#section=2D-Structure
https://pubchem.ncbi.nlm.nih.gov/compound/23294064#section=2D-Structure
https://pubchem.ncbi.nlm.nih.gov/compound/23291061#section=2D-Structure
https://pubchem.ncbi.nlm.nih.gov/compound/23290725#section=2D-Structure
https://pubchem.ncbi.nlm.nih.gov/compound/23288978#section=2D-Structure
https://pubchem.ncbi.nlm.nih.gov/compound/23288949#section=2D-Structure
https://pubchem.ncbi.nlm.nih.gov/compound/23287913#section=2D-Structure
https://pubchem.ncbi.nlm.nih.gov/compound/23279584#section=2D-Structure
https://pubchem.ncbi.nlm.nih.gov/compound/23278249#section=2D-Structure
https://pubchem.ncbi.nlm.nih.gov/compound/23272359#section=2D-Structure
https://pubchem.ncbi.nlm.nih.gov/compound/23271168#section=2D-Structure
https://pubchem.ncbi.nlm.nih.gov/compound/23271087#section=2D-Structure
https://pubchem.ncbi.nlm.nih.gov/compound/23270277#section=2D-Structure
https://pubchem.ncbi.nlm.nih.gov/compound/23270272#section=2D-Structure
https://pubchem.ncbi.nlm.nih.gov/compound/23270271#section=2D-Structure
https://pubchem.ncbi.nlm.nih.gov/compound/23270270#section=2D-Structure
https://pubchem.ncbi.nlm.nih.gov/compound/23270269#section=2D-Structure
https://pubchem.ncbi.nlm.nih.gov/compound/23270268#section=2D-Structure
https://pubchem.ncbi.nlm.nih.gov/compound/23270267#section=2D-Structure
https://pubchem.ncbi.nlm.nih.gov/compound/23270264#section=2D-Structure
https://pubchem.ncbi.nlm.nih.gov/compound/23270263#section=2D-Structure
https://pubchem.ncbi.nlm.nih.gov/compound/23270261#section=2D-Structure
https://pubchem.ncbi.nlm.nih.gov/compound/23268297#section=2D-Structure

https://pubchem.ncbi.nlm.nih.gov/compound/23268278#section=2D-Structure
https://pubchem.ncbi.nlm.nih.gov/compound/23262676#section=2D-Structure
https://pubchem.ncbi.nlm.nih.gov/compound/23257193#section=2D-Structure
https://pubchem.ncbi.nlm.nih.gov/compound/23252058#section=2D-Structure
https://pubchem.ncbi.nlm.nih.gov/compound/23252057#section=2D-Structure
https://pubchem.ncbi.nlm.nih.gov/compound/23251476#section=2D-Structure
https://pubchem.ncbi.nlm.nih.gov/compound/23251473#section=2D-Structure
https://pubchem.ncbi.nlm.nih.gov/compound/23248724#section=2D-Structure
https://pubchem.ncbi.nlm.nih.gov/compound/23248082#section=2D-Structure
https://pubchem.ncbi.nlm.nih.gov/compound/23248074#section=2D-Structure
https://pubchem.ncbi.nlm.nih.gov/compound/23247998#section=2D-Structure
https://pubchem.ncbi.nlm.nih.gov/compound/23224549#section=2D-Structure
https://pubchem.ncbi.nlm.nih.gov/compound/23200200#section=2D-Structure
https://pubchem.ncbi.nlm.nih.gov/compound/23192565#section=2D-Structure
https://pubchem.ncbi.nlm.nih.gov/compound/23176845#section=2D-Structure
https://pubchem.ncbi.nlm.nih.gov/compound/23163761#section=2D-Structure
https://pubchem.ncbi.nlm.nih.gov/compound/23163336#section=2D-Structure
https://pubchem.ncbi.nlm.nih.gov/compound/23161283#section=2D-Structure
https://pubchem.ncbi.nlm.nih.gov/compound/23135729#section=2D-Structure
https://pubchem.ncbi.nlm.nih.gov/compound/23134092#section=2D-Structure
https://pubchem.ncbi.nlm.nih.gov/compound/23128011#section=2D-Structure
https://pubchem.ncbi.nlm.nih.gov/compound/23124554#section=2D-Structure
https://pubchem.ncbi.nlm.nih.gov/compound/23115220#section=2D-Structure
https://pubchem.ncbi.nlm.nih.gov/compound/23112583#section=2D-Structure
https://pubchem.ncbi.nlm.nih.gov/compound/23109829#section=2D-Structure
https://pubchem.ncbi.nlm.nih.gov/compound/23103937#section=2D-Structure
https://pubchem.ncbi.nlm.nih.gov/compound/23102998#section=2D-Structure
https://pubchem.ncbi.nlm.nih.gov/compound/23077332#section=2D-Structure
https://pubchem.ncbi.nlm.nih.gov/compound/23077325#section=2D-Structure
https://pubchem.ncbi.nlm.nih.gov/compound/23074857#section=2D-Structure
https://pubchem.ncbi.nlm.nih.gov/compound/23051876#section=2D-Structure
https://pubchem.ncbi.nlm.nih.gov/compound/23051126#section=2D-Structure
https://pubchem.ncbi.nlm.nih.gov/compound/23049988#section=2D-Structure
https://pubchem.ncbi.nlm.nih.gov/compound/23048255#section=2D-Structure
https://pubchem.ncbi.nlm.nih.gov/compound/23046822#section=2D-Structure
https://pubchem.ncbi.nlm.nih.gov/compound/23046821#section=2D-Structure
https://pubchem.ncbi.nlm.nih.gov/compound/23046673#section=2D-Structure
https://pubchem.ncbi.nlm.nih.gov/compound/23046670#section=2D-Structure
https://pubchem.ncbi.nlm.nih.gov/compound/23036804#section=2D-Structure
https://pubchem.ncbi.nlm.nih.gov/compound/23035071#section=2D-Structure
https://pubchem.ncbi.nlm.nih.gov/compound/23030891#section=2D-Structure
https://pubchem.ncbi.nlm.nih.gov/compound/23030883#section=2D-Structure
https://pubchem.ncbi.nlm.nih.gov/compound/23030855#section=2D-Structure
https://pubchem.ncbi.nlm.nih.gov/compound/23030846#section=2D-Structure
https://pubchem.ncbi.nlm.nih.gov/compound/23007989#section=2D-Structure
https://pubchem.ncbi.nlm.nih.gov/compound/23007986#section=2D-Structure
https://pubchem.ncbi.nlm.nih.gov/compound/22983435#section=2D-Structure
https://pubchem.ncbi.nlm.nih.gov/compound/22980677#section=2D-Structure
https://pubchem.ncbi.nlm.nih.gov/compound/22979278#section=2D-Structure
https://pubchem.ncbi.nlm.nih.gov/compound/22976879#section=2D-Structure
https://pubchem.ncbi.nlm.nih.gov/compound/22976024#section=2D-Structure
https://pubchem.ncbi.nlm.nih.gov/compound/22972251#section=2D-Structure
https://pubchem.ncbi.nlm.nih.gov/compound/22968970#section=2D-Structure
https://pubchem.ncbi.nlm.nih.gov/compound/22964199#section=2D-Structure
https://pubchem.ncbi.nlm.nih.gov/compound/22964196#section=2D-Structure

https://pubchem.ncbi.nlm.nih.gov/compound/22946114#section=2D-Structure
https://pubchem.ncbi.nlm.nih.gov/compound/22925536#section=2D-Structure
https://pubchem.ncbi.nlm.nih.gov/compound/22921711#section=2D-Structure
https://pubchem.ncbi.nlm.nih.gov/compound/22918061#section=2D-Structure
https://pubchem.ncbi.nlm.nih.gov/compound/22908948#section=2D-Structure
https://pubchem.ncbi.nlm.nih.gov/compound/22901873#section=2D-Structure
https://pubchem.ncbi.nlm.nih.gov/compound/22899771#section=2D-Structure
https://pubchem.ncbi.nlm.nih.gov/compound/22899748#section=2D-Structure
https://pubchem.ncbi.nlm.nih.gov/compound/22899719#section=2D-Structure
https://pubchem.ncbi.nlm.nih.gov/compound/22897973#section=2D-Structure
https://pubchem.ncbi.nlm.nih.gov/compound/228833#section=2D-Structure
https://pubchem.ncbi.nlm.nih.gov/compound/228613#section=2D-Structure
https://pubchem.ncbi.nlm.nih.gov/compound/227746#section=2D-Structure
https://pubchem.ncbi.nlm.nih.gov/compound/22764380#section=2D-Structure
https://pubchem.ncbi.nlm.nih.gov/compound/22754947#section=2D-Structure
https://pubchem.ncbi.nlm.nih.gov/compound/22750358#section=2D-Structure
https://pubchem.ncbi.nlm.nih.gov/compound/22750357#section=2D-Structure
https://pubchem.ncbi.nlm.nih.gov/compound/22750356#section=2D-Structure
https://pubchem.ncbi.nlm.nih.gov/compound/22749998#section=2D-Structure
https://pubchem.ncbi.nlm.nih.gov/compound/22747797#section=2D-Structure
https://pubchem.ncbi.nlm.nih.gov/compound/22747786#section=2D-Structure
https://pubchem.ncbi.nlm.nih.gov/compound/22740342#section=2D-Structure
https://pubchem.ncbi.nlm.nih.gov/compound/22740094#section=2D-Structure
https://pubchem.ncbi.nlm.nih.gov/compound/22737532#section=2D-Structure
https://pubchem.ncbi.nlm.nih.gov/compound/22722285#section=2D-Structure
https://pubchem.ncbi.nlm.nih.gov/compound/22721969#section=2D-Structure
https://pubchem.ncbi.nlm.nih.gov/compound/22721479#section=2D-Structure
https://pubchem.ncbi.nlm.nih.gov/compound/22714981#section=2D-Structure
https://pubchem.ncbi.nlm.nih.gov/compound/2268494#section=2D-Structure
https://pubchem.ncbi.nlm.nih.gov/compound/226655#section=2D-Structure
https://pubchem.ncbi.nlm.nih.gov/compound/22665415#section=2D-Structure
https://pubchem.ncbi.nlm.nih.gov/compound/22665413#section=2D-Structure
https://pubchem.ncbi.nlm.nih.gov/compound/22660386#section=2D-Structure
https://pubchem.ncbi.nlm.nih.gov/compound/22660384#section=2D-Structure
https://pubchem.ncbi.nlm.nih.gov/compound/22660379#section=2D-Structure
https://pubchem.ncbi.nlm.nih.gov/compound/22639930#section=2D-Structure
https://pubchem.ncbi.nlm.nih.gov/compound/22639647#section=2D-Structure
https://pubchem.ncbi.nlm.nih.gov/compound/22623931#section=2D-Structure
https://pubchem.ncbi.nlm.nih.gov/compound/22620832#section=2D-Structure
https://pubchem.ncbi.nlm.nih.gov/compound/22610993#section=2D-Structure
https://pubchem.ncbi.nlm.nih.gov/compound/22609702#section=2D-Structure
https://pubchem.ncbi.nlm.nih.gov/compound/22598619#section=2D-Structure
https://pubchem.ncbi.nlm.nih.gov/compound/22598424#section=2D-Structure
https://pubchem.ncbi.nlm.nih.gov/compound/22597294#section=2D-Structure
https://pubchem.ncbi.nlm.nih.gov/compound/22571372#section=2D-Structure
https://pubchem.ncbi.nlm.nih.gov/compound/22566308#section=2D-Structure
https://pubchem.ncbi.nlm.nih.gov/compound/22566295#section=2D-Structure
https://pubchem.ncbi.nlm.nih.gov/compound/22562131#section=2D-Structure
https://pubchem.ncbi.nlm.nih.gov/compound/22561294#section=2D-Structure
https://pubchem.ncbi.nlm.nih.gov/compound/22559980#section=2D-Structure
https://pubchem.ncbi.nlm.nih.gov/compound/22556345#section=2D-Structure
https://pubchem.ncbi.nlm.nih.gov/compound/2253428#section=2D-Structure
https://pubchem.ncbi.nlm.nih.gov/compound/22509386#section=2D-Structure
https://pubchem.ncbi.nlm.nih.gov/compound/22491692#section=2D-Structure
https://pubchem.ncbi.nlm.nih.gov/compound/22489712#section=2D-Structure

https://pubchem.ncbi.nlm.nih.gov/compound/22484459#section=2D-Structure
https://pubchem.ncbi.nlm.nih.gov/compound/22480612#section=2D-Structure
https://pubchem.ncbi.nlm.nih.gov/compound/22475738#section=2D-Structure
https://pubchem.ncbi.nlm.nih.gov/compound/2247082#section=2D-Structure
https://pubchem.ncbi.nlm.nih.gov/compound/22460773#section=2D-Structure
https://pubchem.ncbi.nlm.nih.gov/compound/22452072#section=2D-Structure
https://pubchem.ncbi.nlm.nih.gov/compound/22442198#section=2D-Structure
https://pubchem.ncbi.nlm.nih.gov/compound/22434114#section=2D-Structure
https://pubchem.ncbi.nlm.nih.gov/compound/22419835#section=2D-Structure
https://pubchem.ncbi.nlm.nih.gov/compound/22416933#section=2D-Structure
https://pubchem.ncbi.nlm.nih.gov/compound/22411360#section=2D-Structure
https://pubchem.ncbi.nlm.nih.gov/compound/22397721#section=2D-Structure
https://pubchem.ncbi.nlm.nih.gov/compound/22397713#section=2D-Structure
https://pubchem.ncbi.nlm.nih.gov/compound/22397710#section=2D-Structure
https://pubchem.ncbi.nlm.nih.gov/compound/22397697#section=2D-Structure
https://pubchem.ncbi.nlm.nih.gov/compound/22397680#section=2D-Structure
https://pubchem.ncbi.nlm.nih.gov/compound/22395755#section=2D-Structure
https://pubchem.ncbi.nlm.nih.gov/compound/22392037#section=2D-Structure
https://pubchem.ncbi.nlm.nih.gov/compound/22375080#section=2D-Structure
https://pubchem.ncbi.nlm.nih.gov/compound/22373444#section=2D-Structure
https://pubchem.ncbi.nlm.nih.gov/compound/22371656#section=2D-Structure
https://pubchem.ncbi.nlm.nih.gov/compound/22361982#section=2D-Structure
https://pubchem.ncbi.nlm.nih.gov/compound/22355505#section=2D-Structure
https://pubchem.ncbi.nlm.nih.gov/compound/22352967#section=2D-Structure
https://pubchem.ncbi.nlm.nih.gov/compound/22349137#section=2D-Structure
https://pubchem.ncbi.nlm.nih.gov/compound/22345691#section=2D-Structure
https://pubchem.ncbi.nlm.nih.gov/compound/22339864#section=2D-Structure
https://pubchem.ncbi.nlm.nih.gov/compound/22338880#section=2D-Structure
https://pubchem.ncbi.nlm.nih.gov/compound/22337603#section=2D-Structure
https://pubchem.ncbi.nlm.nih.gov/compound/22317647#section=2D-Structure
https://pubchem.ncbi.nlm.nih.gov/compound/22313871#section=2D-Structure
https://pubchem.ncbi.nlm.nih.gov/compound/22313535#section=2D-Structure
https://pubchem.ncbi.nlm.nih.gov/compound/22311913#section=2D-Structure
https://pubchem.ncbi.nlm.nih.gov/compound/22311479#section=2D-Structure
https://pubchem.ncbi.nlm.nih.gov/compound/22295440#section=2D-Structure
https://pubchem.ncbi.nlm.nih.gov/compound/22294701#section=2D-Structure
https://pubchem.ncbi.nlm.nih.gov/compound/22294688#section=2D-Structure
https://pubchem.ncbi.nlm.nih.gov/compound/22294518#section=2D-Structure
https://pubchem.ncbi.nlm.nih.gov/compound/22294292#section=2D-Structure
https://pubchem.ncbi.nlm.nih.gov/compound/22291337#section=2D-Structure
https://pubchem.ncbi.nlm.nih.gov/compound/22288794#section=2D-Structure
https://pubchem.ncbi.nlm.nih.gov/compound/22286767#section=2D-Structure
https://pubchem.ncbi.nlm.nih.gov/compound/22286721#section=2D-Structure
https://pubchem.ncbi.nlm.nih.gov/compound/22282101#section=2D-Structure
https://pubchem.ncbi.nlm.nih.gov/compound/22281840#section=2D-Structure
https://pubchem.ncbi.nlm.nih.gov/compound/22281833#section=2D-Structure
https://pubchem.ncbi.nlm.nih.gov/compound/22281011#section=2D-Structure
https://pubchem.ncbi.nlm.nih.gov/compound/22270306#section=2D-Structure
https://pubchem.ncbi.nlm.nih.gov/compound/22269650#section=2D-Structure
https://pubchem.ncbi.nlm.nih.gov/compound/22266648#section=2D-Structure
https://pubchem.ncbi.nlm.nih.gov/compound/22266482#section=2D-Structure
https://pubchem.ncbi.nlm.nih.gov/compound/22262493#section=2D-Structure
https://pubchem.ncbi.nlm.nih.gov/compound/22259649#section=2D-Structure
https://pubchem.ncbi.nlm.nih.gov/compound/22259544#section=2D-Structure
https://pubchem.ncbi.nlm.nih.gov/compound/22257834#section=2D-Structure

https://pubchem.ncbi.nlm.nih.gov/compound/22257462#section=2D-Structure
https://pubchem.ncbi.nlm.nih.gov/compound/22253894#section=2D-Structure
https://pubchem.ncbi.nlm.nih.gov/compound/22250977#section=2D-Structure
https://pubchem.ncbi.nlm.nih.gov/compound/22250219#section=2D-Structure
https://pubchem.ncbi.nlm.nih.gov/compound/22250100#section=2D-Structure
https://pubchem.ncbi.nlm.nih.gov/compound/22243853#section=2D-Structure
https://pubchem.ncbi.nlm.nih.gov/compound/22243685#section=2D-Structure
https://pubchem.ncbi.nlm.nih.gov/compound/22227760#section=2D-Structure
https://pubchem.ncbi.nlm.nih.gov/compound/22227517#section=2D-Structure
https://pubchem.ncbi.nlm.nih.gov/compound/22223162#section=2D-Structure
https://pubchem.ncbi.nlm.nih.gov/compound/22218144#section=2D-Structure
https://pubchem.ncbi.nlm.nih.gov/compound/22217622#section=2D-Structure
https://pubchem.ncbi.nlm.nih.gov/compound/22209898#section=2D-Structure
https://pubchem.ncbi.nlm.nih.gov/compound/22182538#section=2D-Structure
https://pubchem.ncbi.nlm.nih.gov/compound/22181905#section=2D-Structure
https://pubchem.ncbi.nlm.nih.gov/compound/22175148#section=2D-Structure
https://pubchem.ncbi.nlm.nih.gov/compound/22171862#section=2D-Structure
https://pubchem.ncbi.nlm.nih.gov/compound/22169237#section=2D-Structure
https://pubchem.ncbi.nlm.nih.gov/compound/22162090#section=2D-Structure
https://pubchem.ncbi.nlm.nih.gov/compound/22159672#section=2D-Structure
https://pubchem.ncbi.nlm.nih.gov/compound/22155144#section=2D-Structure
https://pubchem.ncbi.nlm.nih.gov/compound/22138384#section=2D-Structure
https://pubchem.ncbi.nlm.nih.gov/compound/22138284#section=2D-Structure
https://pubchem.ncbi.nlm.nih.gov/compound/22131515#section=2D-Structure
https://pubchem.ncbi.nlm.nih.gov/compound/22117583#section=2D-Structure
https://pubchem.ncbi.nlm.nih.gov/compound/22114368#section=2D-Structure
https://pubchem.ncbi.nlm.nih.gov/compound/22105241#section=2D-Structure
https://pubchem.ncbi.nlm.nih.gov/compound/22102198#section=2D-Structure
https://pubchem.ncbi.nlm.nih.gov/compound/22100319#section=2D-Structure
https://pubchem.ncbi.nlm.nih.gov/compound/22099958#section=2D-Structure
https://pubchem.ncbi.nlm.nih.gov/compound/22098179#section=2D-Structure
https://pubchem.ncbi.nlm.nih.gov/compound/22098003#section=2D-Structure
https://pubchem.ncbi.nlm.nih.gov/compound/22097109#section=2D-Structure
https://pubchem.ncbi.nlm.nih.gov/compound/22091257#section=2D-Structure
https://pubchem.ncbi.nlm.nih.gov/compound/22090638#section=2D-Structure
https://pubchem.ncbi.nlm.nih.gov/compound/22085020#section=2D-Structure
https://pubchem.ncbi.nlm.nih.gov/compound/220848#section=2D-Structure
https://pubchem.ncbi.nlm.nih.gov/compound/22081154#section=2D-Structure
https://pubchem.ncbi.nlm.nih.gov/compound/22081150#section=2D-Structure
https://pubchem.ncbi.nlm.nih.gov/compound/22065833#section=2D-Structure
https://pubchem.ncbi.nlm.nih.gov/compound/22058241#section=2D-Structure
https://pubchem.ncbi.nlm.nih.gov/compound/22056790#section=2D-Structure
https://pubchem.ncbi.nlm.nih.gov/compound/22051601#section=2D-Structure
https://pubchem.ncbi.nlm.nih.gov/compound/22046620#section=2D-Structure
https://pubchem.ncbi.nlm.nih.gov/compound/22042547#section=2D-Structure
https://pubchem.ncbi.nlm.nih.gov/compound/22037565#section=2D-Structure
https://pubchem.ncbi.nlm.nih.gov/compound/22034818#section=2D-Structure
https://pubchem.ncbi.nlm.nih.gov/compound/22027752#section=2D-Structure
https://pubchem.ncbi.nlm.nih.gov/compound/22023257#section=2D-Structure
https://pubchem.ncbi.nlm.nih.gov/compound/22022782#section=2D-Structure
https://pubchem.ncbi.nlm.nih.gov/compound/22020361#section=2D-Structure
https://pubchem.ncbi.nlm.nih.gov/compound/22016405#section=2D-Structure
https://pubchem.ncbi.nlm.nih.gov/compound/22013657#section=2D-Structure
https://pubchem.ncbi.nlm.nih.gov/compound/22013150#section=2D-Structure
https://pubchem.ncbi.nlm.nih.gov/compound/22012288#section=2D-Structure

https://pubchem.ncbi.nlm.nih.gov/compound/22011660#section=2D-Structure
https://pubchem.ncbi.nlm.nih.gov/compound/22008237#section=2D-Structure
https://pubchem.ncbi.nlm.nih.gov/compound/22007280#section=2D-Structure
https://pubchem.ncbi.nlm.nih.gov/compound/22003319#section=2D-Structure
https://pubchem.ncbi.nlm.nih.gov/compound/21999559#section=2D-Structure
https://pubchem.ncbi.nlm.nih.gov/compound/21995638#section=2D-Structure
https://pubchem.ncbi.nlm.nih.gov/compound/21989323#section=2D-Structure
https://pubchem.ncbi.nlm.nih.gov/compound/21987933#section=2D-Structure
https://pubchem.ncbi.nlm.nih.gov/compound/21983472#section=2D-Structure
https://pubchem.ncbi.nlm.nih.gov/compound/21983279#section=2D-Structure
https://pubchem.ncbi.nlm.nih.gov/compound/21982761#section=2D-Structure
https://pubchem.ncbi.nlm.nih.gov/compound/21979274#section=2D-Structure
https://pubchem.ncbi.nlm.nih.gov/compound/21977525#section=2D-Structure
https://pubchem.ncbi.nlm.nih.gov/compound/21973223#section=2D-Structure
https://pubchem.ncbi.nlm.nih.gov/compound/21966246#section=2D-Structure
https://pubchem.ncbi.nlm.nih.gov/compound/21965378#section=2D-Structure
https://pubchem.ncbi.nlm.nih.gov/compound/21964347#section=2D-Structure
https://pubchem.ncbi.nlm.nih.gov/compound/21956213#section=2D-Structure
https://pubchem.ncbi.nlm.nih.gov/compound/21953560#section=2D-Structure
https://pubchem.ncbi.nlm.nih.gov/compound/21946153#section=2D-Structure
https://pubchem.ncbi.nlm.nih.gov/compound/21941497#section=2D-Structure
https://pubchem.ncbi.nlm.nih.gov/compound/21940733#section=2D-Structure
https://pubchem.ncbi.nlm.nih.gov/compound/21940699#section=2D-Structure
https://pubchem.ncbi.nlm.nih.gov/compound/21940664#section=2D-Structure
https://pubchem.ncbi.nlm.nih.gov/compound/21939718#section=2D-Structure
https://pubchem.ncbi.nlm.nih.gov/compound/21936832#section=2D-Structure
https://pubchem.ncbi.nlm.nih.gov/compound/21936507#section=2D-Structure
https://pubchem.ncbi.nlm.nih.gov/compound/21930930#section=2D-Structure
https://pubchem.ncbi.nlm.nih.gov/compound/21920537#section=2D-Structure
https://pubchem.ncbi.nlm.nih.gov/compound/21909719#section=2D-Structure
https://pubchem.ncbi.nlm.nih.gov/compound/21909717#section=2D-Structure
https://pubchem.ncbi.nlm.nih.gov/compound/21908937#section=2D-Structure
https://pubchem.ncbi.nlm.nih.gov/compound/21907087#section=2D-Structure
https://pubchem.ncbi.nlm.nih.gov/compound/21907046#section=2D-Structure
https://pubchem.ncbi.nlm.nih.gov/compound/21907020#section=2D-Structure
https://pubchem.ncbi.nlm.nih.gov/compound/21907012#section=2D-Structure
https://pubchem.ncbi.nlm.nih.gov/compound/21907006#section=2D-Structure
https://pubchem.ncbi.nlm.nih.gov/compound/21906995#section=2D-Structure
https://pubchem.ncbi.nlm.nih.gov/compound/21896384#section=2D-Structure
https://pubchem.ncbi.nlm.nih.gov/compound/21889413#section=2D-Structure
https://pubchem.ncbi.nlm.nih.gov/compound/21889205#section=2D-Structure
https://pubchem.ncbi.nlm.nih.gov/compound/21888141#section=2D-Structure
https://pubchem.ncbi.nlm.nih.gov/compound/21888131#section=2D-Structure
https://pubchem.ncbi.nlm.nih.gov/compound/21888126#section=2D-Structure
https://pubchem.ncbi.nlm.nih.gov/compound/21888079#section=2D-Structure
https://pubchem.ncbi.nlm.nih.gov/compound/21888078#section=2D-Structure
https://pubchem.ncbi.nlm.nih.gov/compound/21887990#section=2D-Structure
https://pubchem.ncbi.nlm.nih.gov/compound/21887956#section=2D-Structure
https://pubchem.ncbi.nlm.nih.gov/compound/21887760#section=2D-Structure
https://pubchem.ncbi.nlm.nih.gov/compound/21887742#section=2D-Structure
https://pubchem.ncbi.nlm.nih.gov/compound/21887686#section=2D-Structure
https://pubchem.ncbi.nlm.nih.gov/compound/21887617#section=2D-Structure
https://pubchem.ncbi.nlm.nih.gov/compound/21887557#section=2D-Structure
https://pubchem.ncbi.nlm.nih.gov/compound/21887528#section=2D-Structure
https://pubchem.ncbi.nlm.nih.gov/compound/21887508#section=2D-Structure

https://pubchem.ncbi.nlm.nih.gov/compound/21887483#section=2D-Structure
https://pubchem.ncbi.nlm.nih.gov/compound/21887478#section=2D-Structure
https://pubchem.ncbi.nlm.nih.gov/compound/21887416#section=2D-Structure
https://pubchem.ncbi.nlm.nih.gov/compound/21887328#section=2D-Structure
https://pubchem.ncbi.nlm.nih.gov/compound/21887311#section=2D-Structure
https://pubchem.ncbi.nlm.nih.gov/compound/21887294#section=2D-Structure
https://pubchem.ncbi.nlm.nih.gov/compound/21887273#section=2D-Structure
https://pubchem.ncbi.nlm.nih.gov/compound/21887231#section=2D-Structure
https://pubchem.ncbi.nlm.nih.gov/compound/21887219#section=2D-Structure
https://pubchem.ncbi.nlm.nih.gov/compound/21887130#section=2D-Structure
https://pubchem.ncbi.nlm.nih.gov/compound/21887129#section=2D-Structure
https://pubchem.ncbi.nlm.nih.gov/compound/21887113#section=2D-Structure
https://pubchem.ncbi.nlm.nih.gov/compound/21887007#section=2D-Structure
https://pubchem.ncbi.nlm.nih.gov/compound/21886900#section=2D-Structure
https://pubchem.ncbi.nlm.nih.gov/compound/21886869#section=2D-Structure
https://pubchem.ncbi.nlm.nih.gov/compound/21886814#section=2D-Structure
https://pubchem.ncbi.nlm.nih.gov/compound/21886777#section=2D-Structure
https://pubchem.ncbi.nlm.nih.gov/compound/21885942#section=2D-Structure
https://pubchem.ncbi.nlm.nih.gov/compound/21881922#section=2D-Structure
https://pubchem.ncbi.nlm.nih.gov/compound/21881612#section=2D-Structure
https://pubchem.ncbi.nlm.nih.gov/compound/21880592#section=2D-Structure
https://pubchem.ncbi.nlm.nih.gov/compound/21880529#section=2D-Structure
https://pubchem.ncbi.nlm.nih.gov/compound/21874020#section=2D-Structure
https://pubchem.ncbi.nlm.nih.gov/compound/21873569#section=2D-Structure
https://pubchem.ncbi.nlm.nih.gov/compound/21869151#section=2D-Structure
https://pubchem.ncbi.nlm.nih.gov/compound/21868309#section=2D-Structure
https://pubchem.ncbi.nlm.nih.gov/compound/21868029#section=2D-Structure
https://pubchem.ncbi.nlm.nih.gov/compound/21864639#section=2D-Structure
https://pubchem.ncbi.nlm.nih.gov/compound/21864376#section=2D-Structure
https://pubchem.ncbi.nlm.nih.gov/compound/21863423#section=2D-Structure
https://pubchem.ncbi.nlm.nih.gov/compound/21859815#section=2D-Structure
https://pubchem.ncbi.nlm.nih.gov/compound/21859734#section=2D-Structure
https://pubchem.ncbi.nlm.nih.gov/compound/21859243#section=2D-Structure
https://pubchem.ncbi.nlm.nih.gov/compound/21858681#section=2D-Structure
https://pubchem.ncbi.nlm.nih.gov/compound/21854141#section=2D-Structure
https://pubchem.ncbi.nlm.nih.gov/compound/21853854#section=2D-Structure
https://pubchem.ncbi.nlm.nih.gov/compound/21853793#section=2D-Structure
https://pubchem.ncbi.nlm.nih.gov/compound/21850459#section=2D-Structure
https://pubchem.ncbi.nlm.nih.gov/compound/21847777#section=2D-Structure
https://pubchem.ncbi.nlm.nih.gov/compound/21839581#section=2D-Structure
https://pubchem.ncbi.nlm.nih.gov/compound/21838780#section=2D-Structure
https://pubchem.ncbi.nlm.nih.gov/compound/21837554#section=2D-Structure
https://pubchem.ncbi.nlm.nih.gov/compound/21834832#section=2D-Structure
https://pubchem.ncbi.nlm.nih.gov/compound/21828193#section=2D-Structure
https://pubchem.ncbi.nlm.nih.gov/compound/21828152#section=2D-Structure
https://pubchem.ncbi.nlm.nih.gov/compound/21828148#section=2D-Structure
https://pubchem.ncbi.nlm.nih.gov/compound/21828120#section=2D-Structure
https://pubchem.ncbi.nlm.nih.gov/compound/21825451#section=2D-Structure
https://pubchem.ncbi.nlm.nih.gov/compound/21825445#section=2D-Structure
https://pubchem.ncbi.nlm.nih.gov/compound/21825437#section=2D-Structure
https://pubchem.ncbi.nlm.nih.gov/compound/21825434#section=2D-Structure
https://pubchem.ncbi.nlm.nih.gov/compound/21824117#section=2D-Structure
https://pubchem.ncbi.nlm.nih.gov/compound/21823198#section=2D-Structure
https://pubchem.ncbi.nlm.nih.gov/compound/21822225#section=2D-Structure
https://pubchem.ncbi.nlm.nih.gov/compound/21821479#section=2D-Structure

https://pubchem.ncbi.nlm.nih.gov/compound/21819779#section=2D-Structure
https://pubchem.ncbi.nlm.nih.gov/compound/21819778#section=2D-Structure
https://pubchem.ncbi.nlm.nih.gov/compound/21819777#section=2D-Structure
https://pubchem.ncbi.nlm.nih.gov/compound/21818603#section=2D-Structure
https://pubchem.ncbi.nlm.nih.gov/compound/21815704#section=2D-Structure
https://pubchem.ncbi.nlm.nih.gov/compound/21801618#section=2D-Structure
https://pubchem.ncbi.nlm.nih.gov/compound/21792360#section=2D-Structure
https://pubchem.ncbi.nlm.nih.gov/compound/21787752#section=2D-Structure
https://pubchem.ncbi.nlm.nih.gov/compound/21787751#section=2D-Structure
https://pubchem.ncbi.nlm.nih.gov/compound/21787749#section=2D-Structure
https://pubchem.ncbi.nlm.nih.gov/compound/21783405#section=2D-Structure
https://pubchem.ncbi.nlm.nih.gov/compound/21781305#section=2D-Structure
https://pubchem.ncbi.nlm.nih.gov/compound/21780786#section=2D-Structure
https://pubchem.ncbi.nlm.nih.gov/compound/21780785#section=2D-Structure
https://pubchem.ncbi.nlm.nih.gov/compound/21780784#section=2D-Structure
https://pubchem.ncbi.nlm.nih.gov/compound/21780783#section=2D-Structure
https://pubchem.ncbi.nlm.nih.gov/compound/21780782#section=2D-Structure
https://pubchem.ncbi.nlm.nih.gov/compound/21780781#section=2D-Structure
https://pubchem.ncbi.nlm.nih.gov/compound/21780469#section=2D-Structure
https://pubchem.ncbi.nlm.nih.gov/compound/21779317#section=2D-Structure
https://pubchem.ncbi.nlm.nih.gov/compound/21779316#section=2D-Structure
https://pubchem.ncbi.nlm.nih.gov/compound/21767360#section=2D-Structure
https://pubchem.ncbi.nlm.nih.gov/compound/21767359#section=2D-Structure
https://pubchem.ncbi.nlm.nih.gov/compound/21767355#section=2D-Structure
https://pubchem.ncbi.nlm.nih.gov/compound/21761455#section=2D-Structure
https://pubchem.ncbi.nlm.nih.gov/compound/21760986#section=2D-Structure
https://pubchem.ncbi.nlm.nih.gov/compound/21755360#section=2D-Structure
https://pubchem.ncbi.nlm.nih.gov/compound/21754528#section=2D-Structure
https://pubchem.ncbi.nlm.nih.gov/compound/21753385#section=2D-Structure
https://pubchem.ncbi.nlm.nih.gov/compound/21752676#section=2D-Structure
https://pubchem.ncbi.nlm.nih.gov/compound/21752672#section=2D-Structure
https://pubchem.ncbi.nlm.nih.gov/compound/21749136#section=2D-Structure
https://pubchem.ncbi.nlm.nih.gov/compound/21746959#section=2D-Structure
https://pubchem.ncbi.nlm.nih.gov/compound/21732130#section=2D-Structure
https://pubchem.ncbi.nlm.nih.gov/compound/21728679#section=2D-Structure
https://pubchem.ncbi.nlm.nih.gov/compound/21721135#section=2D-Structure
https://pubchem.ncbi.nlm.nih.gov/compound/21721133#section=2D-Structure
https://pubchem.ncbi.nlm.nih.gov/compound/21721132#section=2D-Structure
https://pubchem.ncbi.nlm.nih.gov/compound/21712583#section=2D-Structure
https://pubchem.ncbi.nlm.nih.gov/compound/21709798#section=2D-Structure
https://pubchem.ncbi.nlm.nih.gov/compound/21709739#section=2D-Structure
https://pubchem.ncbi.nlm.nih.gov/compound/21709671#section=2D-Structure
https://pubchem.ncbi.nlm.nih.gov/compound/21707304#section=2D-Structure
https://pubchem.ncbi.nlm.nih.gov/compound/21695573#section=2D-Structure
https://pubchem.ncbi.nlm.nih.gov/compound/21678714#section=2D-Structure
https://pubchem.ncbi.nlm.nih.gov/compound/21676797#section=2D-Structure
https://pubchem.ncbi.nlm.nih.gov/compound/21661291#section=2D-Structure
https://pubchem.ncbi.nlm.nih.gov/compound/21652883#section=2D-Structure
https://pubchem.ncbi.nlm.nih.gov/compound/21651078#section=2D-Structure
https://pubchem.ncbi.nlm.nih.gov/compound/21646649#section=2D-Structure
https://pubchem.ncbi.nlm.nih.gov/compound/21646283#section=2D-Structure
https://pubchem.ncbi.nlm.nih.gov/compound/21645863#section=2D-Structure
https://pubchem.ncbi.nlm.nih.gov/compound/21641559#section=2D-Structure
https://pubchem.ncbi.nlm.nih.gov/compound/21631335#section=2D-Structure
https://pubchem.ncbi.nlm.nih.gov/compound/21624017#section=2D-Structure

https://pubchem.ncbi.nlm.nih.gov/compound/21623218#section=2D-Structure
https://pubchem.ncbi.nlm.nih.gov/compound/21623175#section=2D-Structure
https://pubchem.ncbi.nlm.nih.gov/compound/21617836#section=2D-Structure
https://pubchem.ncbi.nlm.nih.gov/compound/21615149#section=2D-Structure
https://pubchem.ncbi.nlm.nih.gov/compound/21613441#section=2D-Structure
https://pubchem.ncbi.nlm.nih.gov/compound/21613428#section=2D-Structure
https://pubchem.ncbi.nlm.nih.gov/compound/21610904#section=2D-Structure
https://pubchem.ncbi.nlm.nih.gov/compound/21608021#section=2D-Structure
https://pubchem.ncbi.nlm.nih.gov/compound/21603700#section=2D-Structure
https://pubchem.ncbi.nlm.nih.gov/compound/21602280#section=2D-Structure
https://pubchem.ncbi.nlm.nih.gov/compound/21595061#section=2D-Structure
https://pubchem.ncbi.nlm.nih.gov/compound/21572084#section=2D-Structure
https://pubchem.ncbi.nlm.nih.gov/compound/21564780#section=2D-Structure
https://pubchem.ncbi.nlm.nih.gov/compound/21551961#section=2D-Structure
https://pubchem.ncbi.nlm.nih.gov/compound/21551629#section=2D-Structure
https://pubchem.ncbi.nlm.nih.gov/compound/21551628#section=2D-Structure
https://pubchem.ncbi.nlm.nih.gov/compound/21551623#section=2D-Structure
https://pubchem.ncbi.nlm.nih.gov/compound/21551614#section=2D-Structure
https://pubchem.ncbi.nlm.nih.gov/compound/21549879#section=2D-Structure
https://pubchem.ncbi.nlm.nih.gov/compound/21547657#section=2D-Structure
https://pubchem.ncbi.nlm.nih.gov/compound/21533331#section=2D-Structure
https://pubchem.ncbi.nlm.nih.gov/compound/21530759#section=2D-Structure
https://pubchem.ncbi.nlm.nih.gov/compound/21530174#section=2D-Structure
https://pubchem.ncbi.nlm.nih.gov/compound/21528786#section=2D-Structure
https://pubchem.ncbi.nlm.nih.gov/compound/21523528#section=2D-Structure
https://pubchem.ncbi.nlm.nih.gov/compound/21522596#section=2D-Structure
https://pubchem.ncbi.nlm.nih.gov/compound/21517248#section=2D-Structure
https://pubchem.ncbi.nlm.nih.gov/compound/21514875#section=2D-Structure
https://pubchem.ncbi.nlm.nih.gov/compound/21514629#section=2D-Structure
https://pubchem.ncbi.nlm.nih.gov/compound/21503772#section=2D-Structure
https://pubchem.ncbi.nlm.nih.gov/compound/21502217#section=2D-Structure
https://pubchem.ncbi.nlm.nih.gov/compound/21499769#section=2D-Structure
https://pubchem.ncbi.nlm.nih.gov/compound/21499447#section=2D-Structure
https://pubchem.ncbi.nlm.nih.gov/compound/21499404#section=2D-Structure
https://pubchem.ncbi.nlm.nih.gov/compound/21494666#section=2D-Structure
https://pubchem.ncbi.nlm.nih.gov/compound/21492250#section=2D-Structure
https://pubchem.ncbi.nlm.nih.gov/compound/21489918#section=2D-Structure
https://pubchem.ncbi.nlm.nih.gov/compound/21489222#section=2D-Structure
https://pubchem.ncbi.nlm.nih.gov/compound/21477845#section=2D-Structure
https://pubchem.ncbi.nlm.nih.gov/compound/21477271#section=2D-Structure
https://pubchem.ncbi.nlm.nih.gov/compound/21476777#section=2D-Structure
https://pubchem.ncbi.nlm.nih.gov/compound/21465305#section=2D-Structure
https://pubchem.ncbi.nlm.nih.gov/compound/21455257#section=2D-Structure
https://pubchem.ncbi.nlm.nih.gov/compound/21455224#section=2D-Structure
https://pubchem.ncbi.nlm.nih.gov/compound/21453712#section=2D-Structure
https://pubchem.ncbi.nlm.nih.gov/compound/21448199#section=2D-Structure
https://pubchem.ncbi.nlm.nih.gov/compound/21447657#section=2D-Structure
https://pubchem.ncbi.nlm.nih.gov/compound/21447294#section=2D-Structure
https://pubchem.ncbi.nlm.nih.gov/compound/21446371#section=2D-Structure
https://pubchem.ncbi.nlm.nih.gov/compound/21426961#section=2D-Structure
https://pubchem.ncbi.nlm.nih.gov/compound/21425928#section=2D-Structure
https://pubchem.ncbi.nlm.nih.gov/compound/21425393#section=2D-Structure
https://pubchem.ncbi.nlm.nih.gov/compound/21423164#section=2D-Structure
https://pubchem.ncbi.nlm.nih.gov/compound/21409992#section=2D-Structure
https://pubchem.ncbi.nlm.nih.gov/compound/21409978#section=2D-Structure

https://pubchem.ncbi.nlm.nih.gov/compound/21408150#section=2D-Structure
https://pubchem.ncbi.nlm.nih.gov/compound/21399386#section=2D-Structure
https://pubchem.ncbi.nlm.nih.gov/compound/21395657#section=2D-Structure
https://pubchem.ncbi.nlm.nih.gov/compound/21393735#section=2D-Structure
https://pubchem.ncbi.nlm.nih.gov/compound/21363405#section=2D-Structure
https://pubchem.ncbi.nlm.nih.gov/compound/21363400#section=2D-Structure
https://pubchem.ncbi.nlm.nih.gov/compound/21363393#section=2D-Structure
https://pubchem.ncbi.nlm.nih.gov/compound/21360758#section=2D-Structure
https://pubchem.ncbi.nlm.nih.gov/compound/21360441#section=2D-Structure
https://pubchem.ncbi.nlm.nih.gov/compound/21344347#section=2D-Structure
https://pubchem.ncbi.nlm.nih.gov/compound/21332332#section=2D-Structure
https://pubchem.ncbi.nlm.nih.gov/compound/21328028#section=2D-Structure
https://pubchem.ncbi.nlm.nih.gov/compound/21327975#section=2D-Structure
https://pubchem.ncbi.nlm.nih.gov/compound/21314187#section=2D-Structure
https://pubchem.ncbi.nlm.nih.gov/compound/21303730#section=2D-Structure
https://pubchem.ncbi.nlm.nih.gov/compound/21293479#section=2D-Structure
https://pubchem.ncbi.nlm.nih.gov/compound/21289271#section=2D-Structure
https://pubchem.ncbi.nlm.nih.gov/compound/21285739#section=2D-Structure
https://pubchem.ncbi.nlm.nih.gov/compound/21285727#section=2D-Structure
https://pubchem.ncbi.nlm.nih.gov/compound/21280577#section=2D-Structure
https://pubchem.ncbi.nlm.nih.gov/compound/21276161#section=2D-Structure
https://pubchem.ncbi.nlm.nih.gov/compound/21276156#section=2D-Structure
https://pubchem.ncbi.nlm.nih.gov/compound/21269122#section=2D-Structure
https://pubchem.ncbi.nlm.nih.gov/compound/21266640#section=2D-Structure
https://pubchem.ncbi.nlm.nih.gov/compound/21257368#section=2D-Structure
https://pubchem.ncbi.nlm.nih.gov/compound/21253081#section=2D-Structure
https://pubchem.ncbi.nlm.nih.gov/compound/21251950#section=2D-Structure
https://pubchem.ncbi.nlm.nih.gov/compound/21234685#section=2D-Structure
https://pubchem.ncbi.nlm.nih.gov/compound/21234683#section=2D-Structure
https://pubchem.ncbi.nlm.nih.gov/compound/21218275#section=2D-Structure
https://pubchem.ncbi.nlm.nih.gov/compound/21218138#section=2D-Structure
https://pubchem.ncbi.nlm.nih.gov/compound/21217179#section=2D-Structure
https://pubchem.ncbi.nlm.nih.gov/compound/21201186#section=2D-Structure
https://pubchem.ncbi.nlm.nih.gov/compound/21195234#section=2D-Structure
https://pubchem.ncbi.nlm.nih.gov/compound/21190031#section=2D-Structure
https://pubchem.ncbi.nlm.nih.gov/compound/21190012#section=2D-Structure
https://pubchem.ncbi.nlm.nih.gov/compound/21189936#section=2D-Structure
https://pubchem.ncbi.nlm.nih.gov/compound/21163914#section=2D-Structure
https://pubchem.ncbi.nlm.nih.gov/compound/21162934#section=2D-Structure
https://pubchem.ncbi.nlm.nih.gov/compound/21158684#section=2D-Structure
https://pubchem.ncbi.nlm.nih.gov/compound/21158682#section=2D-Structure
https://pubchem.ncbi.nlm.nih.gov/compound/21155575#section=2D-Structure
https://pubchem.ncbi.nlm.nih.gov/compound/21147680#section=2D-Structure
https://pubchem.ncbi.nlm.nih.gov/compound/21147047#section=2D-Structure
https://pubchem.ncbi.nlm.nih.gov/compound/21146047#section=2D-Structure
https://pubchem.ncbi.nlm.nih.gov/compound/21145866#section=2D-Structure
https://pubchem.ncbi.nlm.nih.gov/compound/21145806#section=2D-Structure
https://pubchem.ncbi.nlm.nih.gov/compound/21140431#section=2D-Structure
https://pubchem.ncbi.nlm.nih.gov/compound/21135661#section=2D-Structure
https://pubchem.ncbi.nlm.nih.gov/compound/21126221#section=2D-Structure
https://pubchem.ncbi.nlm.nih.gov/compound/21122456#section=2D-Structure
https://pubchem.ncbi.nlm.nih.gov/compound/21122175#section=2D-Structure
https://pubchem.ncbi.nlm.nih.gov/compound/21121031#section=2D-Structure
https://pubchem.ncbi.nlm.nih.gov/compound/21117118#section=2D-Structure
https://pubchem.ncbi.nlm.nih.gov/compound/21115849#section=2D-Structure

https://pubchem.ncbi.nlm.nih.gov/compound/21115632#section=2D-Structure
https://pubchem.ncbi.nlm.nih.gov/compound/21113427#section=2D-Structure
https://pubchem.ncbi.nlm.nih.gov/compound/21111729#section=2D-Structure
https://pubchem.ncbi.nlm.nih.gov/compound/21106423#section=2D-Structure
https://pubchem.ncbi.nlm.nih.gov/compound/21095854#section=2D-Structure
https://pubchem.ncbi.nlm.nih.gov/compound/21095850#section=2D-Structure
https://pubchem.ncbi.nlm.nih.gov/compound/21095803#section=2D-Structure
https://pubchem.ncbi.nlm.nih.gov/compound/21095801#section=2D-Structure
https://pubchem.ncbi.nlm.nih.gov/compound/21095564#section=2D-Structure
https://pubchem.ncbi.nlm.nih.gov/compound/21095557#section=2D-Structure
https://pubchem.ncbi.nlm.nih.gov/compound/21095372#section=2D-Structure
https://pubchem.ncbi.nlm.nih.gov/compound/21095368#section=2D-Structure
https://pubchem.ncbi.nlm.nih.gov/compound/21095215#section=2D-Structure
https://pubchem.ncbi.nlm.nih.gov/compound/21094332#section=2D-Structure
https://pubchem.ncbi.nlm.nih.gov/compound/21094331#section=2D-Structure
https://pubchem.ncbi.nlm.nih.gov/compound/21061100#section=2D-Structure
https://pubchem.ncbi.nlm.nih.gov/compound/21049753#section=2D-Structure
https://pubchem.ncbi.nlm.nih.gov/compound/21043376#section=2D-Structure
https://pubchem.ncbi.nlm.nih.gov/compound/21039960#section=2D-Structure
https://pubchem.ncbi.nlm.nih.gov/compound/21034549#section=2D-Structure
https://pubchem.ncbi.nlm.nih.gov/compound/21022305#section=2D-Structure
https://pubchem.ncbi.nlm.nih.gov/compound/21019565#section=2D-Structure
https://pubchem.ncbi.nlm.nih.gov/compound/21018131#section=2D-Structure
https://pubchem.ncbi.nlm.nih.gov/compound/20981185#section=2D-Structure
https://pubchem.ncbi.nlm.nih.gov/compound/20979253#section=2D-Structure
https://pubchem.ncbi.nlm.nih.gov/compound/20979231#section=2D-Structure
https://pubchem.ncbi.nlm.nih.gov/compound/20977611#section=2D-Structure
https://pubchem.ncbi.nlm.nih.gov/compound/20976115#section=2D-Structure
https://pubchem.ncbi.nlm.nih.gov/compound/209041#section=2D-Structure
https://pubchem.ncbi.nlm.nih.gov/compound/20871243#section=2D-Structure
https://pubchem.ncbi.nlm.nih.gov/compound/20831750#section=2D-Structure
https://pubchem.ncbi.nlm.nih.gov/compound/20813605#section=2D-Structure
https://pubchem.ncbi.nlm.nih.gov/compound/20812386#section=2D-Structure
https://pubchem.ncbi.nlm.nih.gov/compound/20802505#section=2D-Structure
https://pubchem.ncbi.nlm.nih.gov/compound/207778#section=2D-Structure
https://pubchem.ncbi.nlm.nih.gov/compound/20767394#section=2D-Structure
https://pubchem.ncbi.nlm.nih.gov/compound/20767131#section=2D-Structure
https://pubchem.ncbi.nlm.nih.gov/compound/20753152#section=2D-Structure
https://pubchem.ncbi.nlm.nih.gov/compound/20750667#section=2D-Structure
https://pubchem.ncbi.nlm.nih.gov/compound/20740752#section=2D-Structure
https://pubchem.ncbi.nlm.nih.gov/compound/20740675#section=2D-Structure
https://pubchem.ncbi.nlm.nih.gov/compound/20713192#section=2D-Structure
https://pubchem.ncbi.nlm.nih.gov/compound/20712640#section=2D-Structure
https://pubchem.ncbi.nlm.nih.gov/compound/20707726#section=2D-Structure
https://pubchem.ncbi.nlm.nih.gov/compound/20702778#section=2D-Structure
https://pubchem.ncbi.nlm.nih.gov/compound/20695679#section=2D-Structure
https://pubchem.ncbi.nlm.nih.gov/compound/20695179#section=2D-Structure
https://pubchem.ncbi.nlm.nih.gov/compound/20693964#section=2D-Structure
https://pubchem.ncbi.nlm.nih.gov/compound/20687100#section=2D-Structure
https://pubchem.ncbi.nlm.nih.gov/compound/20687099#section=2D-Structure
https://pubchem.ncbi.nlm.nih.gov/compound/20686298#section=2D-Structure
https://pubchem.ncbi.nlm.nih.gov/compound/20679770#section=2D-Structure
https://pubchem.ncbi.nlm.nih.gov/compound/20679754#section=2D-Structure
https://pubchem.ncbi.nlm.nih.gov/compound/20674978#section=2D-Structure
https://pubchem.ncbi.nlm.nih.gov/compound/20673268#section=2D-Structure

https://pubchem.ncbi.nlm.nih.gov/compound/20670465#section=2D-Structure
https://pubchem.ncbi.nlm.nih.gov/compound/20663925#section=2D-Structure
https://pubchem.ncbi.nlm.nih.gov/compound/20652728#section=2D-Structure
https://pubchem.ncbi.nlm.nih.gov/compound/20650276#section=2D-Structure
https://pubchem.ncbi.nlm.nih.gov/compound/20650092#section=2D-Structure
https://pubchem.ncbi.nlm.nih.gov/compound/20643064#section=2D-Structure
https://pubchem.ncbi.nlm.nih.gov/compound/2063960#section=2D-Structure
https://pubchem.ncbi.nlm.nih.gov/compound/20633891#section=2D-Structure
https://pubchem.ncbi.nlm.nih.gov/compound/20633888#section=2D-Structure
https://pubchem.ncbi.nlm.nih.gov/compound/20603351#section=2D-Structure
https://pubchem.ncbi.nlm.nih.gov/compound/20595617#section=2D-Structure
https://pubchem.ncbi.nlm.nih.gov/compound/20595616#section=2D-Structure
https://pubchem.ncbi.nlm.nih.gov/compound/20588552#section=2D-Structure
https://pubchem.ncbi.nlm.nih.gov/compound/20587209#section=2D-Structure
https://pubchem.ncbi.nlm.nih.gov/compound/20587172#section=2D-Structure
https://pubchem.ncbi.nlm.nih.gov/compound/20587170#section=2D-Structure
https://pubchem.ncbi.nlm.nih.gov/compound/20583496#section=2D-Structure
https://pubchem.ncbi.nlm.nih.gov/compound/20581920#section=2D-Structure
https://pubchem.ncbi.nlm.nih.gov/compound/20578484#section=2D-Structure
https://pubchem.ncbi.nlm.nih.gov/compound/20572658#section=2D-Structure
https://pubchem.ncbi.nlm.nih.gov/compound/20569859#section=2D-Structure
https://pubchem.ncbi.nlm.nih.gov/compound/20563349#section=2D-Structure
https://pubchem.ncbi.nlm.nih.gov/compound/20562075#section=2D-Structure
https://pubchem.ncbi.nlm.nih.gov/compound/20551377#section=2D-Structure
https://pubchem.ncbi.nlm.nih.gov/compound/20550020#section=2D-Structure
https://pubchem.ncbi.nlm.nih.gov/compound/20537722#section=2D-Structure
https://pubchem.ncbi.nlm.nih.gov/compound/20531454#section=2D-Structure
https://pubchem.ncbi.nlm.nih.gov/compound/20524686#section=2D-Structure
https://pubchem.ncbi.nlm.nih.gov/compound/20523274#section=2D-Structure
https://pubchem.ncbi.nlm.nih.gov/compound/20519175#section=2D-Structure
https://pubchem.ncbi.nlm.nih.gov/compound/20519174#section=2D-Structure
https://pubchem.ncbi.nlm.nih.gov/compound/20515626#section=2D-Structure
https://pubchem.ncbi.nlm.nih.gov/compound/20515274#section=2D-Structure
https://pubchem.ncbi.nlm.nih.gov/compound/20506605#section=2D-Structure
https://pubchem.ncbi.nlm.nih.gov/compound/20502086#section=2D-Structure
https://pubchem.ncbi.nlm.nih.gov/compound/20495738#section=2D-Structure
https://pubchem.ncbi.nlm.nih.gov/compound/20490855#section=2D-Structure
https://pubchem.ncbi.nlm.nih.gov/compound/20480257#section=2D-Structure
https://pubchem.ncbi.nlm.nih.gov/compound/20475005#section=2D-Structure
https://pubchem.ncbi.nlm.nih.gov/compound/20469210#section=2D-Structure
https://pubchem.ncbi.nlm.nih.gov/compound/20454005#section=2D-Structure
https://pubchem.ncbi.nlm.nih.gov/compound/20453160#section=2D-Structure
https://pubchem.ncbi.nlm.nih.gov/compound/20450787#section=2D-Structure
https://pubchem.ncbi.nlm.nih.gov/compound/20443842#section=2D-Structure
https://pubchem.ncbi.nlm.nih.gov/compound/20443551#section=2D-Structure
https://pubchem.ncbi.nlm.nih.gov/compound/20443107#section=2D-Structure
https://pubchem.ncbi.nlm.nih.gov/compound/20443105#section=2D-Structure
https://pubchem.ncbi.nlm.nih.gov/compound/20442577#section=2D-Structure
https://pubchem.ncbi.nlm.nih.gov/compound/20436212#section=2D-Structure
https://pubchem.ncbi.nlm.nih.gov/compound/20430857#section=2D-Structure
https://pubchem.ncbi.nlm.nih.gov/compound/20401254#section=2D-Structure
https://pubchem.ncbi.nlm.nih.gov/compound/20395792#section=2D-Structure
https://pubchem.ncbi.nlm.nih.gov/compound/20386358#section=2D-Structure
https://pubchem.ncbi.nlm.nih.gov/compound/20377211#section=2D-Structure
https://pubchem.ncbi.nlm.nih.gov/compound/20374995#section=2D-Structure

https://pubchem.ncbi.nlm.nih.gov/compound/20353196#section=2D-Structure
https://pubchem.ncbi.nlm.nih.gov/compound/20351808#section=2D-Structure
https://pubchem.ncbi.nlm.nih.gov/compound/20346208#section=2D-Structure
https://pubchem.ncbi.nlm.nih.gov/compound/20336181#section=2D-Structure
https://pubchem.ncbi.nlm.nih.gov/compound/20334970#section=2D-Structure
https://pubchem.ncbi.nlm.nih.gov/compound/20332431#section=2D-Structure
https://pubchem.ncbi.nlm.nih.gov/compound/20313006#section=2D-Structure
https://pubchem.ncbi.nlm.nih.gov/compound/20309857#section=2D-Structure
https://pubchem.ncbi.nlm.nih.gov/compound/20309429#section=2D-Structure
https://pubchem.ncbi.nlm.nih.gov/compound/20307234#section=2D-Structure
https://pubchem.ncbi.nlm.nih.gov/compound/20298306#section=2D-Structure
https://pubchem.ncbi.nlm.nih.gov/compound/20296811#section=2D-Structure
https://pubchem.ncbi.nlm.nih.gov/compound/20294936#section=2D-Structure
https://pubchem.ncbi.nlm.nih.gov/compound/20286925#section=2D-Structure
https://pubchem.ncbi.nlm.nih.gov/compound/20286440#section=2D-Structure
https://pubchem.ncbi.nlm.nih.gov/compound/20284807#section=2D-Structure
https://pubchem.ncbi.nlm.nih.gov/compound/20280918#section=2D-Structure
https://pubchem.ncbi.nlm.nih.gov/compound/20275116#section=2D-Structure
https://pubchem.ncbi.nlm.nih.gov/compound/20269008#section=2D-Structure
https://pubchem.ncbi.nlm.nih.gov/compound/20264410#section=2D-Structure
https://pubchem.ncbi.nlm.nih.gov/compound/20262830#section=2D-Structure
https://pubchem.ncbi.nlm.nih.gov/compound/20262826#section=2D-Structure
https://pubchem.ncbi.nlm.nih.gov/compound/20262825#section=2D-Structure
https://pubchem.ncbi.nlm.nih.gov/compound/20261340#section=2D-Structure
https://pubchem.ncbi.nlm.nih.gov/compound/20256804#section=2D-Structure
https://pubchem.ncbi.nlm.nih.gov/compound/20256803#section=2D-Structure
https://pubchem.ncbi.nlm.nih.gov/compound/20244493#section=2D-Structure
https://pubchem.ncbi.nlm.nih.gov/compound/20239825#section=2D-Structure
https://pubchem.ncbi.nlm.nih.gov/compound/20235936#section=2D-Structure
https://pubchem.ncbi.nlm.nih.gov/compound/20234974#section=2D-Structure
https://pubchem.ncbi.nlm.nih.gov/compound/20232710#section=2D-Structure
https://pubchem.ncbi.nlm.nih.gov/compound/20232264#section=2D-Structure
https://pubchem.ncbi.nlm.nih.gov/compound/20231025#section=2D-Structure
https://pubchem.ncbi.nlm.nih.gov/compound/20227272#section=2D-Structure
https://pubchem.ncbi.nlm.nih.gov/compound/20225915#section=2D-Structure
https://pubchem.ncbi.nlm.nih.gov/compound/20225764#section=2D-Structure
https://pubchem.ncbi.nlm.nih.gov/compound/20225587#section=2D-Structure
https://pubchem.ncbi.nlm.nih.gov/compound/20221382#section=2D-Structure
https://pubchem.ncbi.nlm.nih.gov/compound/20221008#section=2D-Structure
https://pubchem.ncbi.nlm.nih.gov/compound/20208215#section=2D-Structure
https://pubchem.ncbi.nlm.nih.gov/compound/20203864#section=2D-Structure
https://pubchem.ncbi.nlm.nih.gov/compound/20203863#section=2D-Structure
https://pubchem.ncbi.nlm.nih.gov/compound/20202082#section=2D-Structure
https://pubchem.ncbi.nlm.nih.gov/compound/20201281#section=2D-Structure
https://pubchem.ncbi.nlm.nih.gov/compound/20199014#section=2D-Structure
https://pubchem.ncbi.nlm.nih.gov/compound/20195160#section=2D-Structure
https://pubchem.ncbi.nlm.nih.gov/compound/20192716#section=2D-Structure
https://pubchem.ncbi.nlm.nih.gov/compound/20164134#section=2D-Structure
https://pubchem.ncbi.nlm.nih.gov/compound/20158587#section=2D-Structure
https://pubchem.ncbi.nlm.nih.gov/compound/20156592#section=2D-Structure
https://pubchem.ncbi.nlm.nih.gov/compound/20154053#section=2D-Structure
https://pubchem.ncbi.nlm.nih.gov/compound/20146588#section=2D-Structure
https://pubchem.ncbi.nlm.nih.gov/compound/20141772#section=2D-Structure
https://pubchem.ncbi.nlm.nih.gov/compound/20140047#section=2D-Structure
https://pubchem.ncbi.nlm.nih.gov/compound/20137556#section=2D-Structure

https://pubchem.ncbi.nlm.nih.gov/compound/20137544#section=2D-Structure
https://pubchem.ncbi.nlm.nih.gov/compound/20137538#section=2D-Structure
https://pubchem.ncbi.nlm.nih.gov/compound/20131544#section=2D-Structure
https://pubchem.ncbi.nlm.nih.gov/compound/20098137#section=2D-Structure
https://pubchem.ncbi.nlm.nih.gov/compound/20096843#section=2D-Structure
https://pubchem.ncbi.nlm.nih.gov/compound/20093456#section=2D-Structure
https://pubchem.ncbi.nlm.nih.gov/compound/20089402#section=2D-Structure
https://pubchem.ncbi.nlm.nih.gov/compound/20088072#section=2D-Structure
https://pubchem.ncbi.nlm.nih.gov/compound/20087566#section=2D-Structure
https://pubchem.ncbi.nlm.nih.gov/compound/20077782#section=2D-Structure
https://pubchem.ncbi.nlm.nih.gov/compound/20072783#section=2D-Structure
https://pubchem.ncbi.nlm.nih.gov/compound/20067069#section=2D-Structure
https://pubchem.ncbi.nlm.nih.gov/compound/20064366#section=2D-Structure
https://pubchem.ncbi.nlm.nih.gov/compound/200456#section=2D-Structure
https://pubchem.ncbi.nlm.nih.gov/compound/20039855#section=2D-Structure
https://pubchem.ncbi.nlm.nih.gov/compound/20039851#section=2D-Structure
https://pubchem.ncbi.nlm.nih.gov/compound/20039847#section=2D-Structure
https://pubchem.ncbi.nlm.nih.gov/compound/20036523#section=2D-Structure
https://pubchem.ncbi.nlm.nih.gov/compound/20035549#section=2D-Structure
https://pubchem.ncbi.nlm.nih.gov/compound/20032866#section=2D-Structure
https://pubchem.ncbi.nlm.nih.gov/compound/20031100#section=2D-Structure
https://pubchem.ncbi.nlm.nih.gov/compound/19996745#section=2D-Structure
https://pubchem.ncbi.nlm.nih.gov/compound/19996714#section=2D-Structure
https://pubchem.ncbi.nlm.nih.gov/compound/19996712#section=2D-Structure
https://pubchem.ncbi.nlm.nih.gov/compound/19996705#section=2D-Structure
https://pubchem.ncbi.nlm.nih.gov/compound/19995716#section=2D-Structure
https://pubchem.ncbi.nlm.nih.gov/compound/19985672#section=2D-Structure
https://pubchem.ncbi.nlm.nih.gov/compound/19980090#section=2D-Structure
https://pubchem.ncbi.nlm.nih.gov/compound/19979578#section=2D-Structure
https://pubchem.ncbi.nlm.nih.gov/compound/19975663#section=2D-Structure
https://pubchem.ncbi.nlm.nih.gov/compound/19972193#section=2D-Structure
https://pubchem.ncbi.nlm.nih.gov/compound/19971598#section=2D-Structure
https://pubchem.ncbi.nlm.nih.gov/compound/19969783#section=2D-Structure
https://pubchem.ncbi.nlm.nih.gov/compound/19969464#section=2D-Structure
https://pubchem.ncbi.nlm.nih.gov/compound/19969459#section=2D-Structure
https://pubchem.ncbi.nlm.nih.gov/compound/19969453#section=2D-Structure
https://pubchem.ncbi.nlm.nih.gov/compound/19968#section=2D-Structure
https://pubchem.ncbi.nlm.nih.gov/compound/19959602#section=2D-Structure
https://pubchem.ncbi.nlm.nih.gov/compound/19959592#section=2D-Structure
https://pubchem.ncbi.nlm.nih.gov/compound/19959591#section=2D-Structure
https://pubchem.ncbi.nlm.nih.gov/compound/19959590#section=2D-Structure
https://pubchem.ncbi.nlm.nih.gov/compound/19959586#section=2D-Structure
https://pubchem.ncbi.nlm.nih.gov/compound/19959585#section=2D-Structure
https://pubchem.ncbi.nlm.nih.gov/compound/19958894#section=2D-Structure
https://pubchem.ncbi.nlm.nih.gov/compound/19958892#section=2D-Structure
https://pubchem.ncbi.nlm.nih.gov/compound/19958602#section=2D-Structure
https://pubchem.ncbi.nlm.nih.gov/compound/19958563#section=2D-Structure
https://pubchem.ncbi.nlm.nih.gov/compound/19955569#section=2D-Structure
https://pubchem.ncbi.nlm.nih.gov/compound/19935234#section=2D-Structure
https://pubchem.ncbi.nlm.nih.gov/compound/19935233#section=2D-Structure
https://pubchem.ncbi.nlm.nih.gov/compound/19935232#section=2D-Structure
https://pubchem.ncbi.nlm.nih.gov/compound/19931713#section=2D-Structure
https://pubchem.ncbi.nlm.nih.gov/compound/19919690#section=2D-Structure
https://pubchem.ncbi.nlm.nih.gov/compound/19918842#section=2D-Structure
https://pubchem.ncbi.nlm.nih.gov/compound/19918670#section=2D-Structure

https://pubchem.ncbi.nlm.nih.gov/compound/19911676#section=2D-Structure
https://pubchem.ncbi.nlm.nih.gov/compound/19905606#section=2D-Structure
https://pubchem.ncbi.nlm.nih.gov/compound/19902715#section=2D-Structure
https://pubchem.ncbi.nlm.nih.gov/compound/19902711#section=2D-Structure
https://pubchem.ncbi.nlm.nih.gov/compound/19899665#section=2D-Structure
https://pubchem.ncbi.nlm.nih.gov/compound/19898646#section=2D-Structure
https://pubchem.ncbi.nlm.nih.gov/compound/19898518#section=2D-Structure
https://pubchem.ncbi.nlm.nih.gov/compound/19898510#section=2D-Structure
https://pubchem.ncbi.nlm.nih.gov/compound/19898460#section=2D-Structure
https://pubchem.ncbi.nlm.nih.gov/compound/19896762#section=2D-Structure
https://pubchem.ncbi.nlm.nih.gov/compound/19885595#section=2D-Structure
https://pubchem.ncbi.nlm.nih.gov/compound/19883871#section=2D-Structure
https://pubchem.ncbi.nlm.nih.gov/compound/19881897#section=2D-Structure
https://pubchem.ncbi.nlm.nih.gov/compound/19880538#section=2D-Structure
https://pubchem.ncbi.nlm.nih.gov/compound/19880533#section=2D-Structure
https://pubchem.ncbi.nlm.nih.gov/compound/19879807#section=2D-Structure
https://pubchem.ncbi.nlm.nih.gov/compound/19874661#section=2D-Structure
https://pubchem.ncbi.nlm.nih.gov/compound/19874627#section=2D-Structure
https://pubchem.ncbi.nlm.nih.gov/compound/19873270#section=2D-Structure
https://pubchem.ncbi.nlm.nih.gov/compound/19862953#section=2D-Structure
https://pubchem.ncbi.nlm.nih.gov/compound/19861007#section=2D-Structure
https://pubchem.ncbi.nlm.nih.gov/compound/19857437#section=2D-Structure
https://pubchem.ncbi.nlm.nih.gov/compound/19851309#section=2D-Structure
https://pubchem.ncbi.nlm.nih.gov/compound/19847252#section=2D-Structure
https://pubchem.ncbi.nlm.nih.gov/compound/19844541#section=2D-Structure
https://pubchem.ncbi.nlm.nih.gov/compound/19841381#section=2D-Structure
https://pubchem.ncbi.nlm.nih.gov/compound/19837879#section=2D-Structure
https://pubchem.ncbi.nlm.nih.gov/compound/19835666#section=2D-Structure
https://pubchem.ncbi.nlm.nih.gov/compound/19832890#section=2D-Structure
https://pubchem.ncbi.nlm.nih.gov/compound/19830578#section=2D-Structure
https://pubchem.ncbi.nlm.nih.gov/compound/19828016#section=2D-Structure
https://pubchem.ncbi.nlm.nih.gov/compound/19828013#section=2D-Structure
https://pubchem.ncbi.nlm.nih.gov/compound/19824931#section=2D-Structure
https://pubchem.ncbi.nlm.nih.gov/compound/19820843#section=2D-Structure
https://pubchem.ncbi.nlm.nih.gov/compound/19820439#section=2D-Structure
https://pubchem.ncbi.nlm.nih.gov/compound/19816510#section=2D-Structure
https://pubchem.ncbi.nlm.nih.gov/compound/19815765#section=2D-Structure
https://pubchem.ncbi.nlm.nih.gov/compound/19815743#section=2D-Structure
https://pubchem.ncbi.nlm.nih.gov/compound/19815709#section=2D-Structure
https://pubchem.ncbi.nlm.nih.gov/compound/19812726#section=2D-Structure
https://pubchem.ncbi.nlm.nih.gov/compound/19810500#section=2D-Structure
https://pubchem.ncbi.nlm.nih.gov/compound/19806581#section=2D-Structure
https://pubchem.ncbi.nlm.nih.gov/compound/19806578#section=2D-Structure
https://pubchem.ncbi.nlm.nih.gov/compound/19801734#section=2D-Structure
https://pubchem.ncbi.nlm.nih.gov/compound/19801698#section=2D-Structure
https://pubchem.ncbi.nlm.nih.gov/compound/19800485#section=2D-Structure
https://pubchem.ncbi.nlm.nih.gov/compound/19800206#section=2D-Structure
https://pubchem.ncbi.nlm.nih.gov/compound/19800194#section=2D-Structure
https://pubchem.ncbi.nlm.nih.gov/compound/19795966#section=2D-Structure
https://pubchem.ncbi.nlm.nih.gov/compound/19795265#section=2D-Structure
https://pubchem.ncbi.nlm.nih.gov/compound/19786476#section=2D-Structure
https://pubchem.ncbi.nlm.nih.gov/compound/19782484#section=2D-Structure
https://pubchem.ncbi.nlm.nih.gov/compound/19781538#section=2D-Structure
https://pubchem.ncbi.nlm.nih.gov/compound/19778214#section=2D-Structure
https://pubchem.ncbi.nlm.nih.gov/compound/19774398#section=2D-Structure

https://pubchem.ncbi.nlm.nih.gov/compound/19766745#section=2D-Structure
https://pubchem.ncbi.nlm.nih.gov/compound/19765016#section=2D-Structure
https://pubchem.ncbi.nlm.nih.gov/compound/19764080#section=2D-Structure
https://pubchem.ncbi.nlm.nih.gov/compound/19763352#section=2D-Structure
https://pubchem.ncbi.nlm.nih.gov/compound/19757525#section=2D-Structure
https://pubchem.ncbi.nlm.nih.gov/compound/19748666#section=2D-Structure
https://pubchem.ncbi.nlm.nih.gov/compound/19748605#section=2D-Structure
https://pubchem.ncbi.nlm.nih.gov/compound/19748576#section=2D-Structure
https://pubchem.ncbi.nlm.nih.gov/compound/19710549#section=2D-Structure
https://pubchem.ncbi.nlm.nih.gov/compound/19702209#section=2D-Structure
https://pubchem.ncbi.nlm.nih.gov/compound/19702091#section=2D-Structure
https://pubchem.ncbi.nlm.nih.gov/compound/19701076#section=2D-Structure
https://pubchem.ncbi.nlm.nih.gov/compound/19699118#section=2D-Structure
https://pubchem.ncbi.nlm.nih.gov/compound/19691932#section=2D-Structure
https://pubchem.ncbi.nlm.nih.gov/compound/19608956#section=2D-Structure
https://pubchem.ncbi.nlm.nih.gov/compound/19603193#section=2D-Structure
https://pubchem.ncbi.nlm.nih.gov/compound/19601191#section=2D-Structure
https://pubchem.ncbi.nlm.nih.gov/compound/19594190#section=2D-Structure
https://pubchem.ncbi.nlm.nih.gov/compound/19437327#section=2D-Structure
https://pubchem.ncbi.nlm.nih.gov/compound/19432859#section=2D-Structure
https://pubchem.ncbi.nlm.nih.gov/compound/19432083#section=2D-Structure
https://pubchem.ncbi.nlm.nih.gov/compound/19425983#section=2D-Structure
https://pubchem.ncbi.nlm.nih.gov/compound/19422860#section=2D-Structure
https://pubchem.ncbi.nlm.nih.gov/compound/19422374#section=2D-Structure
https://pubchem.ncbi.nlm.nih.gov/compound/19417810#section=2D-Structure
https://pubchem.ncbi.nlm.nih.gov/compound/19387240#section=2D-Structure
https://pubchem.ncbi.nlm.nih.gov/compound/19385898#section=2D-Structure
https://pubchem.ncbi.nlm.nih.gov/compound/19384587#section=2D-Structure
https://pubchem.ncbi.nlm.nih.gov/compound/19377758#section=2D-Structure
https://pubchem.ncbi.nlm.nih.gov/compound/19376056#section=2D-Structure
https://pubchem.ncbi.nlm.nih.gov/compound/19371539#section=2D-Structure
https://pubchem.ncbi.nlm.nih.gov/compound/19368228#section=2D-Structure
https://pubchem.ncbi.nlm.nih.gov/compound/19360791#section=2D-Structure
https://pubchem.ncbi.nlm.nih.gov/compound/19360520#section=2D-Structure
https://pubchem.ncbi.nlm.nih.gov/compound/19352224#section=2D-Structure
https://pubchem.ncbi.nlm.nih.gov/compound/193287#section=2D-Structure
https://pubchem.ncbi.nlm.nih.gov/compound/192821#section=2D-Structure
https://pubchem.ncbi.nlm.nih.gov/compound/19261615#section=2D-Structure
https://pubchem.ncbi.nlm.nih.gov/compound/19245295#section=2D-Structure
https://pubchem.ncbi.nlm.nih.gov/compound/191671#section=2D-Structure
https://pubchem.ncbi.nlm.nih.gov/compound/1911832#section=2D-Structure
https://pubchem.ncbi.nlm.nih.gov/compound/19104814#section=2D-Structure
https://pubchem.ncbi.nlm.nih.gov/compound/19104811#section=2D-Structure
https://pubchem.ncbi.nlm.nih.gov/compound/19102560#section=2D-Structure
https://pubchem.ncbi.nlm.nih.gov/compound/19098919#section=2D-Structure
https://pubchem.ncbi.nlm.nih.gov/compound/19097556#section=2D-Structure
https://pubchem.ncbi.nlm.nih.gov/compound/19093339#section=2D-Structure
https://pubchem.ncbi.nlm.nih.gov/compound/19092219#section=2D-Structure
https://pubchem.ncbi.nlm.nih.gov/compound/19090414#section=2D-Structure
https://pubchem.ncbi.nlm.nih.gov/compound/19086887#section=2D-Structure
https://pubchem.ncbi.nlm.nih.gov/compound/19079986#section=2D-Structure
https://pubchem.ncbi.nlm.nih.gov/compound/19079983#section=2D-Structure
https://pubchem.ncbi.nlm.nih.gov/compound/19079981#section=2D-Structure
https://pubchem.ncbi.nlm.nih.gov/compound/19077232#section=2D-Structure
https://pubchem.ncbi.nlm.nih.gov/compound/19077089#section=2D-Structure

https://pubchem.ncbi.nlm.nih.gov/compound/19075073#section=2D-Structure
https://pubchem.ncbi.nlm.nih.gov/compound/19073814#section=2D-Structure
https://pubchem.ncbi.nlm.nih.gov/compound/19073526#section=2D-Structure
https://pubchem.ncbi.nlm.nih.gov/compound/19070995#section=2D-Structure
https://pubchem.ncbi.nlm.nih.gov/compound/19068933#section=2D-Structure
https://pubchem.ncbi.nlm.nih.gov/compound/19068402#section=2D-Structure
https://pubchem.ncbi.nlm.nih.gov/compound/19067856#section=2D-Structure
https://pubchem.ncbi.nlm.nih.gov/compound/19067747#section=2D-Structure
https://pubchem.ncbi.nlm.nih.gov/compound/19067665#section=2D-Structure
https://pubchem.ncbi.nlm.nih.gov/compound/19049859#section=2D-Structure
https://pubchem.ncbi.nlm.nih.gov/compound/19048702#section=2D-Structure
https://pubchem.ncbi.nlm.nih.gov/compound/19048680#section=2D-Structure
https://pubchem.ncbi.nlm.nih.gov/compound/19048102#section=2D-Structure
https://pubchem.ncbi.nlm.nih.gov/compound/19038785#section=2D-Structure
https://pubchem.ncbi.nlm.nih.gov/compound/19037542#section=2D-Structure
https://pubchem.ncbi.nlm.nih.gov/compound/19035669#section=2D-Structure
https://pubchem.ncbi.nlm.nih.gov/compound/19031642#section=2D-Structure
https://pubchem.ncbi.nlm.nih.gov/compound/19029265#section=2D-Structure
https://pubchem.ncbi.nlm.nih.gov/compound/19026265#section=2D-Structure
https://pubchem.ncbi.nlm.nih.gov/compound/19021824#section=2D-Structure
https://pubchem.ncbi.nlm.nih.gov/compound/19017548#section=2D-Structure
https://pubchem.ncbi.nlm.nih.gov/compound/19013751#section=2D-Structure
https://pubchem.ncbi.nlm.nih.gov/compound/19013146#section=2D-Structure
https://pubchem.ncbi.nlm.nih.gov/compound/19009370#section=2D-Structure
https://pubchem.ncbi.nlm.nih.gov/compound/19006097#section=2D-Structure
https://pubchem.ncbi.nlm.nih.gov/compound/19003072#section=2D-Structure
https://pubchem.ncbi.nlm.nih.gov/compound/19001541#section=2D-Structure
https://pubchem.ncbi.nlm.nih.gov/compound/18994621#section=2D-Structure
https://pubchem.ncbi.nlm.nih.gov/compound/18992749#section=2D-Structure
https://pubchem.ncbi.nlm.nih.gov/compound/18992563#section=2D-Structure
https://pubchem.ncbi.nlm.nih.gov/compound/18992505#section=2D-Structure
https://pubchem.ncbi.nlm.nih.gov/compound/18988106#section=2D-Structure
https://pubchem.ncbi.nlm.nih.gov/compound/18970466#section=2D-Structure
https://pubchem.ncbi.nlm.nih.gov/compound/18968491#section=2D-Structure
https://pubchem.ncbi.nlm.nih.gov/compound/1896427#section=2D-Structure
https://pubchem.ncbi.nlm.nih.gov/compound/18956727#section=2D-Structure
https://pubchem.ncbi.nlm.nih.gov/compound/18955300#section=2D-Structure
https://pubchem.ncbi.nlm.nih.gov/compound/18952429#section=2D-Structure
https://pubchem.ncbi.nlm.nih.gov/compound/18952074#section=2D-Structure
https://pubchem.ncbi.nlm.nih.gov/compound/18942142#section=2D-Structure
https://pubchem.ncbi.nlm.nih.gov/compound/18941541#section=2D-Structure
https://pubchem.ncbi.nlm.nih.gov/compound/18941525#section=2D-Structure
https://pubchem.ncbi.nlm.nih.gov/compound/18940758#section=2D-Structure
https://pubchem.ncbi.nlm.nih.gov/compound/18933198#section=2D-Structure
https://pubchem.ncbi.nlm.nih.gov/compound/1893302#section=2D-Structure
https://pubchem.ncbi.nlm.nih.gov/compound/18930017#section=2D-Structure
https://pubchem.ncbi.nlm.nih.gov/compound/18928400#section=2D-Structure
https://pubchem.ncbi.nlm.nih.gov/compound/18928234#section=2D-Structure
https://pubchem.ncbi.nlm.nih.gov/compound/18925321#section=2D-Structure
https://pubchem.ncbi.nlm.nih.gov/compound/18925113#section=2D-Structure
https://pubchem.ncbi.nlm.nih.gov/compound/18919291#section=2D-Structure
https://pubchem.ncbi.nlm.nih.gov/compound/18917836#section=2D-Structure
https://pubchem.ncbi.nlm.nih.gov/compound/188071#section=2D-Structure
https://pubchem.ncbi.nlm.nih.gov/compound/18799103#section=2D-Structure
https://pubchem.ncbi.nlm.nih.gov/compound/18791921#section=2D-Structure

https://pubchem.ncbi.nlm.nih.gov/compound/18788140#section=2D-Structure
https://pubchem.ncbi.nlm.nih.gov/compound/18785560#section=2D-Structure
https://pubchem.ncbi.nlm.nih.gov/compound/18782100#section=2D-Structure
https://pubchem.ncbi.nlm.nih.gov/compound/1876787#section=2D-Structure
https://pubchem.ncbi.nlm.nih.gov/compound/18755303#section=2D-Structure
https://pubchem.ncbi.nlm.nih.gov/compound/18739017#section=2D-Structure
https://pubchem.ncbi.nlm.nih.gov/compound/18737576#section=2D-Structure
https://pubchem.ncbi.nlm.nih.gov/compound/18730669#section=2D-Structure
https://pubchem.ncbi.nlm.nih.gov/compound/18730119#section=2D-Structure
https://pubchem.ncbi.nlm.nih.gov/compound/18724003#section=2D-Structure
https://pubchem.ncbi.nlm.nih.gov/compound/18723999#section=2D-Structure
https://pubchem.ncbi.nlm.nih.gov/compound/18723998#section=2D-Structure
https://pubchem.ncbi.nlm.nih.gov/compound/18723997#section=2D-Structure
https://pubchem.ncbi.nlm.nih.gov/compound/18716344#section=2D-Structure
https://pubchem.ncbi.nlm.nih.gov/compound/18714394#section=2D-Structure
https://pubchem.ncbi.nlm.nih.gov/compound/18706419#section=2D-Structure
https://pubchem.ncbi.nlm.nih.gov/compound/18704058#section=2D-Structure
https://pubchem.ncbi.nlm.nih.gov/compound/18704056#section=2D-Structure
https://pubchem.ncbi.nlm.nih.gov/compound/18694962#section=2D-Structure
https://pubchem.ncbi.nlm.nih.gov/compound/18684773#section=2D-Structure
https://pubchem.ncbi.nlm.nih.gov/compound/18676598#section=2D-Structure
https://pubchem.ncbi.nlm.nih.gov/compound/18670037#section=2D-Structure
https://pubchem.ncbi.nlm.nih.gov/compound/186694#section=2D-Structure
https://pubchem.ncbi.nlm.nih.gov/compound/18627784#section=2D-Structure
https://pubchem.ncbi.nlm.nih.gov/compound/18623171#section=2D-Structure
https://pubchem.ncbi.nlm.nih.gov/compound/18622940#section=2D-Structure
https://pubchem.ncbi.nlm.nih.gov/compound/18616870#section=2D-Structure
https://pubchem.ncbi.nlm.nih.gov/compound/18616864#section=2D-Structure
https://pubchem.ncbi.nlm.nih.gov/compound/18616205#section=2D-Structure
https://pubchem.ncbi.nlm.nih.gov/compound/18612470#section=2D-Structure
https://pubchem.ncbi.nlm.nih.gov/compound/18612332#section=2D-Structure
https://pubchem.ncbi.nlm.nih.gov/compound/18539628#section=2D-Structure
https://pubchem.ncbi.nlm.nih.gov/compound/18538199#section=2D-Structure
https://pubchem.ncbi.nlm.nih.gov/compound/18533593#section=2D-Structure
https://pubchem.ncbi.nlm.nih.gov/compound/18533355#section=2D-Structure
https://pubchem.ncbi.nlm.nih.gov/compound/18522577#section=2D-Structure
https://pubchem.ncbi.nlm.nih.gov/compound/18509251#section=2D-Structure
https://pubchem.ncbi.nlm.nih.gov/compound/18471893#section=2D-Structure
https://pubchem.ncbi.nlm.nih.gov/compound/18469897#section=2D-Structure
https://pubchem.ncbi.nlm.nih.gov/compound/18459618#section=2D-Structure
https://pubchem.ncbi.nlm.nih.gov/compound/18445838#section=2D-Structure
https://pubchem.ncbi.nlm.nih.gov/compound/18445836#section=2D-Structure
https://pubchem.ncbi.nlm.nih.gov/compound/18442195#section=2D-Structure
https://pubchem.ncbi.nlm.nih.gov/compound/18435511#section=2D-Structure
https://pubchem.ncbi.nlm.nih.gov/compound/18416204#section=2D-Structure
https://pubchem.ncbi.nlm.nih.gov/compound/18416186#section=2D-Structure
https://pubchem.ncbi.nlm.nih.gov/compound/18410033#section=2D-Structure
https://pubchem.ncbi.nlm.nih.gov/compound/18401488#section=2D-Structure
https://pubchem.ncbi.nlm.nih.gov/compound/18401110#section=2D-Structure
https://pubchem.ncbi.nlm.nih.gov/compound/18387425#section=2D-Structure
https://pubchem.ncbi.nlm.nih.gov/compound/18377852#section=2D-Structure
https://pubchem.ncbi.nlm.nih.gov/compound/18376415#section=2D-Structure
https://pubchem.ncbi.nlm.nih.gov/compound/18376413#section=2D-Structure
https://pubchem.ncbi.nlm.nih.gov/compound/18376408#section=2D-Structure
https://pubchem.ncbi.nlm.nih.gov/compound/18375721#section=2D-Structure

https://pubchem.ncbi.nlm.nih.gov/compound/18362943#section=2D-Structure
https://pubchem.ncbi.nlm.nih.gov/compound/18361956#section=2D-Structure
https://pubchem.ncbi.nlm.nih.gov/compound/18354516#section=2D-Structure
https://pubchem.ncbi.nlm.nih.gov/compound/18347243#section=2D-Structure
https://pubchem.ncbi.nlm.nih.gov/compound/18341060#section=2D-Structure
https://pubchem.ncbi.nlm.nih.gov/compound/18336127#section=2D-Structure
https://pubchem.ncbi.nlm.nih.gov/compound/18326642#section=2D-Structure
https://pubchem.ncbi.nlm.nih.gov/compound/182144#section=2D-Structure
https://pubchem.ncbi.nlm.nih.gov/compound/18187422#section=2D-Structure
https://pubchem.ncbi.nlm.nih.gov/compound/18187031#section=2D-Structure
https://pubchem.ncbi.nlm.nih.gov/compound/18184371#section=2D-Structure
https://pubchem.ncbi.nlm.nih.gov/compound/18183250#section=2D-Structure
https://pubchem.ncbi.nlm.nih.gov/compound/18177598#section=2D-Structure
https://pubchem.ncbi.nlm.nih.gov/compound/181529#section=2D-Structure
https://pubchem.ncbi.nlm.nih.gov/compound/180157#section=2D-Structure
https://pubchem.ncbi.nlm.nih.gov/compound/18006508#section=2D-Structure
https://pubchem.ncbi.nlm.nih.gov/compound/18004599#section=2D-Structure
https://pubchem.ncbi.nlm.nih.gov/compound/18003950#section=2D-Structure
https://pubchem.ncbi.nlm.nih.gov/compound/18000385#section=2D-Structure
https://pubchem.ncbi.nlm.nih.gov/compound/17993275#section=2D-Structure
https://pubchem.ncbi.nlm.nih.gov/compound/17993229#section=2D-Structure
https://pubchem.ncbi.nlm.nih.gov/compound/17993198#section=2D-Structure
https://pubchem.ncbi.nlm.nih.gov/compound/17992407#section=2D-Structure
https://pubchem.ncbi.nlm.nih.gov/compound/17986764#section=2D-Structure
https://pubchem.ncbi.nlm.nih.gov/compound/17986498#section=2D-Structure
https://pubchem.ncbi.nlm.nih.gov/compound/17979851#section=2D-Structure
https://pubchem.ncbi.nlm.nih.gov/compound/17979849#section=2D-Structure
https://pubchem.ncbi.nlm.nih.gov/compound/17979838#section=2D-Structure
https://pubchem.ncbi.nlm.nih.gov/compound/17974461#section=2D-Structure
https://pubchem.ncbi.nlm.nih.gov/compound/17967584#section=2D-Structure
https://pubchem.ncbi.nlm.nih.gov/compound/17965575#section=2D-Structure
https://pubchem.ncbi.nlm.nih.gov/compound/17963410#section=2D-Structure
https://pubchem.ncbi.nlm.nih.gov/compound/17961012#section=2D-Structure
https://pubchem.ncbi.nlm.nih.gov/compound/17953902#section=2D-Structure
https://pubchem.ncbi.nlm.nih.gov/compound/17949164#section=2D-Structure
https://pubchem.ncbi.nlm.nih.gov/compound/17947130#section=2D-Structure
https://pubchem.ncbi.nlm.nih.gov/compound/17943896#section=2D-Structure
https://pubchem.ncbi.nlm.nih.gov/compound/17941562#section=2D-Structure
https://pubchem.ncbi.nlm.nih.gov/compound/17938646#section=2D-Structure
https://pubchem.ncbi.nlm.nih.gov/compound/17937711#section=2D-Structure
https://pubchem.ncbi.nlm.nih.gov/compound/17936041#section=2D-Structure
https://pubchem.ncbi.nlm.nih.gov/compound/17932476#section=2D-Structure
https://pubchem.ncbi.nlm.nih.gov/compound/17932398#section=2D-Structure
https://pubchem.ncbi.nlm.nih.gov/compound/17928804#section=2D-Structure
https://pubchem.ncbi.nlm.nih.gov/compound/17921883#section=2D-Structure
https://pubchem.ncbi.nlm.nih.gov/compound/17912094#section=2D-Structure
https://pubchem.ncbi.nlm.nih.gov/compound/17911139#section=2D-Structure
https://pubchem.ncbi.nlm.nih.gov/compound/17904787#section=2D-Structure
https://pubchem.ncbi.nlm.nih.gov/compound/17898003#section=2D-Structure
https://pubchem.ncbi.nlm.nih.gov/compound/17889187#section=2D-Structure
https://pubchem.ncbi.nlm.nih.gov/compound/17883295#section=2D-Structure
https://pubchem.ncbi.nlm.nih.gov/compound/17876085#section=2D-Structure
https://pubchem.ncbi.nlm.nih.gov/compound/178753#section=2D-Structure
https://pubchem.ncbi.nlm.nih.gov/compound/17875278#section=2D-Structure
https://pubchem.ncbi.nlm.nih.gov/compound/17874416#section=2D-Structure

https://pubchem.ncbi.nlm.nih.gov/compound/17872331#section=2D-Structure
https://pubchem.ncbi.nlm.nih.gov/compound/17868368#section=2D-Structure
https://pubchem.ncbi.nlm.nih.gov/compound/17865062#section=2D-Structure
https://pubchem.ncbi.nlm.nih.gov/compound/17861185#section=2D-Structure
https://pubchem.ncbi.nlm.nih.gov/compound/17859716#section=2D-Structure
https://pubchem.ncbi.nlm.nih.gov/compound/17858333#section=2D-Structure
https://pubchem.ncbi.nlm.nih.gov/compound/17858179#section=2D-Structure
https://pubchem.ncbi.nlm.nih.gov/compound/17856947#section=2D-Structure
https://pubchem.ncbi.nlm.nih.gov/compound/17855717#section=2D-Structure
https://pubchem.ncbi.nlm.nih.gov/compound/17855662#section=2D-Structure
https://pubchem.ncbi.nlm.nih.gov/compound/17845415#section=2D-Structure
https://pubchem.ncbi.nlm.nih.gov/compound/17844695#section=2D-Structure
https://pubchem.ncbi.nlm.nih.gov/compound/17843990#section=2D-Structure
https://pubchem.ncbi.nlm.nih.gov/compound/17818593#section=2D-Structure
https://pubchem.ncbi.nlm.nih.gov/compound/17804911#section=2D-Structure
https://pubchem.ncbi.nlm.nih.gov/compound/17804720#section=2D-Structure
https://pubchem.ncbi.nlm.nih.gov/compound/17803698#section=2D-Structure
https://pubchem.ncbi.nlm.nih.gov/compound/17803465#section=2D-Structure
https://pubchem.ncbi.nlm.nih.gov/compound/17803292#section=2D-Structure
https://pubchem.ncbi.nlm.nih.gov/compound/17800807#section=2D-Structure
https://pubchem.ncbi.nlm.nih.gov/compound/17800402#section=2D-Structure
https://pubchem.ncbi.nlm.nih.gov/compound/17793025#section=2D-Structure
https://pubchem.ncbi.nlm.nih.gov/compound/17784010#section=2D-Structure
https://pubchem.ncbi.nlm.nih.gov/compound/17777333#section=2D-Structure
https://pubchem.ncbi.nlm.nih.gov/compound/17773988#section=2D-Structure
https://pubchem.ncbi.nlm.nih.gov/compound/17769767#section=2D-Structure
https://pubchem.ncbi.nlm.nih.gov/compound/17763059#section=2D-Structure
https://pubchem.ncbi.nlm.nih.gov/compound/16876902#section=2D-Structure
https://pubchem.ncbi.nlm.nih.gov/compound/168170#section=2D-Structure
https://pubchem.ncbi.nlm.nih.gov/compound/16762004#section=2D-Structure
https://pubchem.ncbi.nlm.nih.gov/compound/16727013#section=2D-Structure
https://pubchem.ncbi.nlm.nih.gov/compound/167#section=2D-Structure
https://pubchem.ncbi.nlm.nih.gov/compound/166878#section=2D-Structure
https://pubchem.ncbi.nlm.nih.gov/compound/166734#section=2D-Structure
https://pubchem.ncbi.nlm.nih.gov/compound/16639781#section=2D-Structure
https://pubchem.ncbi.nlm.nih.gov/compound/16639195#section=2D-Structure
https://pubchem.ncbi.nlm.nih.gov/compound/16457388#section=2D-Structure
https://pubchem.ncbi.nlm.nih.gov/compound/16414177#section=2D-Structure
https://pubchem.ncbi.nlm.nih.gov/compound/162542#section=2D-Structure
https://pubchem.ncbi.nlm.nih.gov/compound/16206018#section=2D-Structure
https://pubchem.ncbi.nlm.nih.gov/compound/15971363#section=2D-Structure
https://pubchem.ncbi.nlm.nih.gov/compound/15966252#section=2D-Structure
https://pubchem.ncbi.nlm.nih.gov/compound/15961788#section=2D-Structure
https://pubchem.ncbi.nlm.nih.gov/compound/15960218#section=2D-Structure
https://pubchem.ncbi.nlm.nih.gov/compound/15939513#section=2D-Structure
https://pubchem.ncbi.nlm.nih.gov/compound/15935821#section=2D-Structure
https://pubchem.ncbi.nlm.nih.gov/compound/1590789#section=2D-Structure
https://pubchem.ncbi.nlm.nih.gov/compound/15897929#section=2D-Structure
https://pubchem.ncbi.nlm.nih.gov/compound/15892787#section=2D-Structure
https://pubchem.ncbi.nlm.nih.gov/compound/158834#section=2D-Structure
https://pubchem.ncbi.nlm.nih.gov/compound/15832065#section=2D-Structure
https://pubchem.ncbi.nlm.nih.gov/compound/15811645#section=2D-Structure
https://pubchem.ncbi.nlm.nih.gov/compound/15810462#section=2D-Structure
https://pubchem.ncbi.nlm.nih.gov/compound/15793117#section=2D-Structure
https://pubchem.ncbi.nlm.nih.gov/compound/15791992#section=2D-Structure

https://pubchem.ncbi.nlm.nih.gov/compound/15783124#section=2D-Structure
https://pubchem.ncbi.nlm.nih.gov/compound/15781774#section=2D-Structure
https://pubchem.ncbi.nlm.nih.gov/compound/15778976#section=2D-Structure
https://pubchem.ncbi.nlm.nih.gov/compound/15761308#section=2D-Structure
https://pubchem.ncbi.nlm.nih.gov/compound/15749754#section=2D-Structure
https://pubchem.ncbi.nlm.nih.gov/compound/15745038#section=2D-Structure
https://pubchem.ncbi.nlm.nih.gov/compound/15744371#section=2D-Structure
https://pubchem.ncbi.nlm.nih.gov/compound/15733178#section=2D-Structure
https://pubchem.ncbi.nlm.nih.gov/compound/15720713#section=2D-Structure
https://pubchem.ncbi.nlm.nih.gov/compound/15715931#section=2D-Structure
https://pubchem.ncbi.nlm.nih.gov/compound/15711331#section=2D-Structure
https://pubchem.ncbi.nlm.nih.gov/compound/15711329#section=2D-Structure
https://pubchem.ncbi.nlm.nih.gov/compound/15711075#section=2D-Structure
https://pubchem.ncbi.nlm.nih.gov/compound/15711073#section=2D-Structure
https://pubchem.ncbi.nlm.nih.gov/compound/15711068#section=2D-Structure
https://pubchem.ncbi.nlm.nih.gov/compound/15690351#section=2D-Structure
https://pubchem.ncbi.nlm.nih.gov/compound/15683242#section=2D-Structure
https://pubchem.ncbi.nlm.nih.gov/compound/15680526#section=2D-Structure
https://pubchem.ncbi.nlm.nih.gov/compound/15668048#section=2D-Structure
https://pubchem.ncbi.nlm.nih.gov/compound/15668045#section=2D-Structure
https://pubchem.ncbi.nlm.nih.gov/compound/15649067#section=2D-Structure
https://pubchem.ncbi.nlm.nih.gov/compound/15648092#section=2D-Structure
https://pubchem.ncbi.nlm.nih.gov/compound/15618943#section=2D-Structure
https://pubchem.ncbi.nlm.nih.gov/compound/15578809#section=2D-Structure
https://pubchem.ncbi.nlm.nih.gov/compound/15570693#section=2D-Structure
https://pubchem.ncbi.nlm.nih.gov/compound/15561145#section=2D-Structure
https://pubchem.ncbi.nlm.nih.gov/compound/155610#section=2D-Structure
https://pubchem.ncbi.nlm.nih.gov/compound/155609#section=2D-Structure
https://pubchem.ncbi.nlm.nih.gov/compound/15554847#section=2D-Structure
https://pubchem.ncbi.nlm.nih.gov/compound/15537495#section=2D-Structure
https://pubchem.ncbi.nlm.nih.gov/compound/15528581#section=2D-Structure
https://pubchem.ncbi.nlm.nih.gov/compound/15528580#section=2D-Structure
https://pubchem.ncbi.nlm.nih.gov/compound/15520186#section=2D-Structure
https://pubchem.ncbi.nlm.nih.gov/compound/15491825#section=2D-Structure
https://pubchem.ncbi.nlm.nih.gov/compound/15480207#section=2D-Structure
https://pubchem.ncbi.nlm.nih.gov/compound/154772#section=2D-Structure
https://pubchem.ncbi.nlm.nih.gov/compound/154771#section=2D-Structure
https://pubchem.ncbi.nlm.nih.gov/compound/15449312#section=2D-Structure
https://pubchem.ncbi.nlm.nih.gov/compound/15449031#section=2D-Structure
https://pubchem.ncbi.nlm.nih.gov/compound/154258#section=2D-Structure
https://pubchem.ncbi.nlm.nih.gov/compound/154245#section=2D-Structure
https://pubchem.ncbi.nlm.nih.gov/compound/15418379#section=2D-Structure
https://pubchem.ncbi.nlm.nih.gov/compound/15402461#section=2D-Structure
https://pubchem.ncbi.nlm.nih.gov/compound/15373437#section=2D-Structure
https://pubchem.ncbi.nlm.nih.gov/compound/15366034#section=2D-Structure
https://pubchem.ncbi.nlm.nih.gov/compound/15366#section=2D-Structure
https://pubchem.ncbi.nlm.nih.gov/compound/15357981#section=2D-Structure
https://pubchem.ncbi.nlm.nih.gov/compound/15353497#section=2D-Structure
https://pubchem.ncbi.nlm.nih.gov/compound/15346030#section=2D-Structure
https://pubchem.ncbi.nlm.nih.gov/compound/15339777#section=2D-Structure
https://pubchem.ncbi.nlm.nih.gov/compound/15325848#section=2D-Structure
https://pubchem.ncbi.nlm.nih.gov/compound/15321802#section=2D-Structure
https://pubchem.ncbi.nlm.nih.gov/compound/15321367#section=2D-Structure
https://pubchem.ncbi.nlm.nih.gov/compound/15321363#section=2D-Structure
https://pubchem.ncbi.nlm.nih.gov/compound/15290749#section=2D-Structure

https://pubchem.ncbi.nlm.nih.gov/compound/15273723#section=2D-Structure
https://pubchem.ncbi.nlm.nih.gov/compound/15261149#section=2D-Structure
https://pubchem.ncbi.nlm.nih.gov/compound/15260748#section=2D-Structure
https://pubchem.ncbi.nlm.nih.gov/compound/15260744#section=2D-Structure
https://pubchem.ncbi.nlm.nih.gov/compound/15260742#section=2D-Structure
https://pubchem.ncbi.nlm.nih.gov/compound/15258763#section=2D-Structure
https://pubchem.ncbi.nlm.nih.gov/compound/15258762#section=2D-Structure
https://pubchem.ncbi.nlm.nih.gov/compound/15258761#section=2D-Structure
https://pubchem.ncbi.nlm.nih.gov/compound/15252526#section=2D-Structure
https://pubchem.ncbi.nlm.nih.gov/compound/15250547#section=2D-Structure
https://pubchem.ncbi.nlm.nih.gov/compound/15250082#section=2D-Structure
https://pubchem.ncbi.nlm.nih.gov/compound/152352#section=2D-Structure
https://pubchem.ncbi.nlm.nih.gov/compound/15234753#section=2D-Structure
https://pubchem.ncbi.nlm.nih.gov/compound/15230913#section=2D-Structure
https://pubchem.ncbi.nlm.nih.gov/compound/15230911#section=2D-Structure
https://pubchem.ncbi.nlm.nih.gov/compound/152272#section=2D-Structure
https://pubchem.ncbi.nlm.nih.gov/compound/15214240#section=2D-Structure
https://pubchem.ncbi.nlm.nih.gov/compound/15206507#section=2D-Structure
https://pubchem.ncbi.nlm.nih.gov/compound/15152018#section=2D-Structure
https://pubchem.ncbi.nlm.nih.gov/compound/15142105#section=2D-Structure
https://pubchem.ncbi.nlm.nih.gov/compound/15110654#section=2D-Structure
https://pubchem.ncbi.nlm.nih.gov/compound/15109159#section=2D-Structure
https://pubchem.ncbi.nlm.nih.gov/compound/15105540#section=2D-Structure
https://pubchem.ncbi.nlm.nih.gov/compound/151035#section=2D-Structure
https://pubchem.ncbi.nlm.nih.gov/compound/15096388#section=2D-Structure
https://pubchem.ncbi.nlm.nih.gov/compound/15094759#section=2D-Structure
https://pubchem.ncbi.nlm.nih.gov/compound/15033708#section=2D-Structure
https://pubchem.ncbi.nlm.nih.gov/compound/15033674#section=2D-Structure
https://pubchem.ncbi.nlm.nih.gov/compound/15033672#section=2D-Structure
https://pubchem.ncbi.nlm.nih.gov/compound/15033669#section=2D-Structure
https://pubchem.ncbi.nlm.nih.gov/compound/15033668#section=2D-Structure
https://pubchem.ncbi.nlm.nih.gov/compound/15033666#section=2D-Structure
https://pubchem.ncbi.nlm.nih.gov/compound/15031567#section=2D-Structure
https://pubchem.ncbi.nlm.nih.gov/compound/15027773#section=2D-Structure
https://pubchem.ncbi.nlm.nih.gov/compound/15025486#section=2D-Structure
https://pubchem.ncbi.nlm.nih.gov/compound/15013607#section=2D-Structure
https://pubchem.ncbi.nlm.nih.gov/compound/15004838#section=2D-Structure
https://pubchem.ncbi.nlm.nih.gov/compound/14984945#section=2D-Structure
https://pubchem.ncbi.nlm.nih.gov/compound/14973632#section=2D-Structure
https://pubchem.ncbi.nlm.nih.gov/compound/14973539#section=2D-Structure
https://pubchem.ncbi.nlm.nih.gov/compound/149603#section=2D-Structure
https://pubchem.ncbi.nlm.nih.gov/compound/14950261#section=2D-Structure
https://pubchem.ncbi.nlm.nih.gov/compound/14950258#section=2D-Structure
https://pubchem.ncbi.nlm.nih.gov/compound/14950257#section=2D-Structure
https://pubchem.ncbi.nlm.nih.gov/compound/14945130#section=2D-Structure
https://pubchem.ncbi.nlm.nih.gov/compound/14945129#section=2D-Structure
https://pubchem.ncbi.nlm.nih.gov/compound/14945128#section=2D-Structure
https://pubchem.ncbi.nlm.nih.gov/compound/14945127#section=2D-Structure
https://pubchem.ncbi.nlm.nih.gov/compound/14945126#section=2D-Structure
https://pubchem.ncbi.nlm.nih.gov/compound/14945125#section=2D-Structure
https://pubchem.ncbi.nlm.nih.gov/compound/14945124#section=2D-Structure
https://pubchem.ncbi.nlm.nih.gov/compound/14945123#section=2D-Structure
https://pubchem.ncbi.nlm.nih.gov/compound/14945122#section=2D-Structure
https://pubchem.ncbi.nlm.nih.gov/compound/14945121#section=2D-Structure
https://pubchem.ncbi.nlm.nih.gov/compound/14945120#section=2D-Structure

https://pubchem.ncbi.nlm.nih.gov/compound/14945119#section=2D-Structure
https://pubchem.ncbi.nlm.nih.gov/compound/14945118#section=2D-Structure
https://pubchem.ncbi.nlm.nih.gov/compound/14945117#section=2D-Structure
https://pubchem.ncbi.nlm.nih.gov/compound/14945116#section=2D-Structure
https://pubchem.ncbi.nlm.nih.gov/compound/14945115#section=2D-Structure
https://pubchem.ncbi.nlm.nih.gov/compound/14945114#section=2D-Structure
https://pubchem.ncbi.nlm.nih.gov/compound/14945112#section=2D-Structure
https://pubchem.ncbi.nlm.nih.gov/compound/14945110#section=2D-Structure
https://pubchem.ncbi.nlm.nih.gov/compound/14945108#section=2D-Structure
https://pubchem.ncbi.nlm.nih.gov/compound/14945106#section=2D-Structure
https://pubchem.ncbi.nlm.nih.gov/compound/14945102#section=2D-Structure
https://pubchem.ncbi.nlm.nih.gov/compound/14944110#section=2D-Structure
https://pubchem.ncbi.nlm.nih.gov/compound/14942230#section=2D-Structure
https://pubchem.ncbi.nlm.nih.gov/compound/14935214#section=2D-Structure
https://pubchem.ncbi.nlm.nih.gov/compound/14932781#section=2D-Structure
https://pubchem.ncbi.nlm.nih.gov/compound/14931494#section=2D-Structure
https://pubchem.ncbi.nlm.nih.gov/compound/14931491#section=2D-Structure
https://pubchem.ncbi.nlm.nih.gov/compound/14929555#section=2D-Structure
https://pubchem.ncbi.nlm.nih.gov/compound/14903118#section=2D-Structure
https://pubchem.ncbi.nlm.nih.gov/compound/14899686#section=2D-Structure
https://pubchem.ncbi.nlm.nih.gov/compound/14899616#section=2D-Structure
https://pubchem.ncbi.nlm.nih.gov/compound/14897002#section=2D-Structure
https://pubchem.ncbi.nlm.nih.gov/compound/14896998#section=2D-Structure
https://pubchem.ncbi.nlm.nih.gov/compound/14896110#section=2D-Structure
https://pubchem.ncbi.nlm.nih.gov/compound/14867063#section=2D-Structure
https://pubchem.ncbi.nlm.nih.gov/compound/14822479#section=2D-Structure
https://pubchem.ncbi.nlm.nih.gov/compound/14822477#section=2D-Structure
https://pubchem.ncbi.nlm.nih.gov/compound/14822476#section=2D-Structure
https://pubchem.ncbi.nlm.nih.gov/compound/14792118#section=2D-Structure
https://pubchem.ncbi.nlm.nih.gov/compound/147442#section=2D-Structure
https://pubchem.ncbi.nlm.nih.gov/compound/147344#section=2D-Structure
https://pubchem.ncbi.nlm.nih.gov/compound/147196#section=2D-Structure
https://pubchem.ncbi.nlm.nih.gov/compound/14717987#section=2D-Structure
https://pubchem.ncbi.nlm.nih.gov/compound/146687#section=2D-Structure
https://pubchem.ncbi.nlm.nih.gov/compound/146679463#section=2D-Structure
https://pubchem.ncbi.nlm.nih.gov/compound/146679403#section=2D-Structure
https://pubchem.ncbi.nlm.nih.gov/compound/146665677#section=2D-Structure
https://pubchem.ncbi.nlm.nih.gov/compound/146651685#section=2D-Structure
https://pubchem.ncbi.nlm.nih.gov/compound/146651487#section=2D-Structure
https://pubchem.ncbi.nlm.nih.gov/compound/14664826#section=2D-Structure
https://pubchem.ncbi.nlm.nih.gov/compound/14663056#section=2D-Structure
https://pubchem.ncbi.nlm.nih.gov/compound/14663053#section=2D-Structure
https://pubchem.ncbi.nlm.nih.gov/compound/14661706#section=2D-Structure
https://pubchem.ncbi.nlm.nih.gov/compound/14661705#section=2D-Structure
https://pubchem.ncbi.nlm.nih.gov/compound/14661703#section=2D-Structure
https://pubchem.ncbi.nlm.nih.gov/compound/146595241#section=2D-Structure
https://pubchem.ncbi.nlm.nih.gov/compound/146595238#section=2D-Structure
https://pubchem.ncbi.nlm.nih.gov/compound/146591905#section=2D-Structure
https://pubchem.ncbi.nlm.nih.gov/compound/146590366#section=2D-Structure
https://pubchem.ncbi.nlm.nih.gov/compound/146590360#section=2D-Structure
https://pubchem.ncbi.nlm.nih.gov/compound/146589484#section=2D-Structure
https://pubchem.ncbi.nlm.nih.gov/compound/146576736#section=2D-Structure
https://pubchem.ncbi.nlm.nih.gov/compound/146576735#section=2D-Structure
https://pubchem.ncbi.nlm.nih.gov/compound/146561216#section=2D-Structure
https://pubchem.ncbi.nlm.nih.gov/compound/146560634#section=2D-Structure

https://pubchem.ncbi.nlm.nih.gov/compound/146555320#section=2D-Structure
https://pubchem.ncbi.nlm.nih.gov/compound/146552174#section=2D-Structure
https://pubchem.ncbi.nlm.nih.gov/compound/146535347#section=2D-Structure
https://pubchem.ncbi.nlm.nih.gov/compound/146529632#section=2D-Structure
https://pubchem.ncbi.nlm.nih.gov/compound/146522559#section=2D-Structure
https://pubchem.ncbi.nlm.nih.gov/compound/146515409#section=2D-Structure
https://pubchem.ncbi.nlm.nih.gov/compound/146509336#section=2D-Structure
https://pubchem.ncbi.nlm.nih.gov/compound/146491#section=2D-Structure
https://pubchem.ncbi.nlm.nih.gov/compound/146486233#section=2D-Structure
https://pubchem.ncbi.nlm.nih.gov/compound/146483993#section=2D-Structure
https://pubchem.ncbi.nlm.nih.gov/compound/146451390#section=2D-Structure
https://pubchem.ncbi.nlm.nih.gov/compound/146429935#section=2D-Structure
https://pubchem.ncbi.nlm.nih.gov/compound/14642811#section=2D-Structure
https://pubchem.ncbi.nlm.nih.gov/compound/14642804#section=2D-Structure
https://pubchem.ncbi.nlm.nih.gov/compound/146427961#section=2D-Structure
https://pubchem.ncbi.nlm.nih.gov/compound/14642051#section=2D-Structure
https://pubchem.ncbi.nlm.nih.gov/compound/146416559#section=2D-Structure
https://pubchem.ncbi.nlm.nih.gov/compound/14641653#section=2D-Structure
https://pubchem.ncbi.nlm.nih.gov/compound/146414642#section=2D-Structure
https://pubchem.ncbi.nlm.nih.gov/compound/14639457#section=2D-Structure
https://pubchem.ncbi.nlm.nih.gov/compound/14639455#section=2D-Structure
https://pubchem.ncbi.nlm.nih.gov/compound/146356454#section=2D-Structure
https://pubchem.ncbi.nlm.nih.gov/compound/146351704#section=2D-Structure
https://pubchem.ncbi.nlm.nih.gov/compound/146351701#section=2D-Structure
https://pubchem.ncbi.nlm.nih.gov/compound/146351614#section=2D-Structure
https://pubchem.ncbi.nlm.nih.gov/compound/146347502#section=2D-Structure
https://pubchem.ncbi.nlm.nih.gov/compound/146319577#section=2D-Structure
https://pubchem.ncbi.nlm.nih.gov/compound/146319438#section=2D-Structure
https://pubchem.ncbi.nlm.nih.gov/compound/146278276#section=2D-Structure
https://pubchem.ncbi.nlm.nih.gov/compound/146275025#section=2D-Structure
https://pubchem.ncbi.nlm.nih.gov/compound/146275011#section=2D-Structure
https://pubchem.ncbi.nlm.nih.gov/compound/146268259#section=2D-Structure
https://pubchem.ncbi.nlm.nih.gov/compound/146255222#section=2D-Structure
https://pubchem.ncbi.nlm.nih.gov/compound/146248415#section=2D-Structure
https://pubchem.ncbi.nlm.nih.gov/compound/146244301#section=2D-Structure
https://pubchem.ncbi.nlm.nih.gov/compound/146244205#section=2D-Structure
https://pubchem.ncbi.nlm.nih.gov/compound/146240518#section=2D-Structure
https://pubchem.ncbi.nlm.nih.gov/compound/146232935#section=2D-Structure
https://pubchem.ncbi.nlm.nih.gov/compound/146232929#section=2D-Structure
https://pubchem.ncbi.nlm.nih.gov/compound/146232519#section=2D-Structure
https://pubchem.ncbi.nlm.nih.gov/compound/146232188#section=2D-Structure
https://pubchem.ncbi.nlm.nih.gov/compound/146227334#section=2D-Structure
https://pubchem.ncbi.nlm.nih.gov/compound/146225385#section=2D-Structure
https://pubchem.ncbi.nlm.nih.gov/compound/146214158#section=2D-Structure
https://pubchem.ncbi.nlm.nih.gov/compound/146214156#section=2D-Structure
https://pubchem.ncbi.nlm.nih.gov/compound/146191580#section=2D-Structure
https://pubchem.ncbi.nlm.nih.gov/compound/146177103#section=2D-Structure
https://pubchem.ncbi.nlm.nih.gov/compound/146170130#section=2D-Structure
https://pubchem.ncbi.nlm.nih.gov/compound/14617007#section=2D-Structure
https://pubchem.ncbi.nlm.nih.gov/compound/146168603#section=2D-Structure
https://pubchem.ncbi.nlm.nih.gov/compound/146163472#section=2D-Structure
https://pubchem.ncbi.nlm.nih.gov/compound/14612052#section=2D-Structure
https://pubchem.ncbi.nlm.nih.gov/compound/146037682#section=2D-Structure
https://pubchem.ncbi.nlm.nih.gov/compound/146037677#section=2D-Structure
https://pubchem.ncbi.nlm.nih.gov/compound/146037666#section=2D-Structure

https://pubchem.ncbi.nlm.nih.gov/compound/146036092#section=2D-Structure
https://pubchem.ncbi.nlm.nih.gov/compound/146029225#section=2D-Structure
https://pubchem.ncbi.nlm.nih.gov/compound/146020777#section=2D-Structure
https://pubchem.ncbi.nlm.nih.gov/compound/146020765#section=2D-Structure
https://pubchem.ncbi.nlm.nih.gov/compound/146020755#section=2D-Structure
https://pubchem.ncbi.nlm.nih.gov/compound/146020733#section=2D-Structure
https://pubchem.ncbi.nlm.nih.gov/compound/146018413#section=2D-Structure
https://pubchem.ncbi.nlm.nih.gov/compound/145953133#section=2D-Structure
https://pubchem.ncbi.nlm.nih.gov/compound/145926233#section=2D-Structure
https://pubchem.ncbi.nlm.nih.gov/compound/145918835#section=2D-Structure
https://pubchem.ncbi.nlm.nih.gov/compound/145863595#section=2D-Structure
https://pubchem.ncbi.nlm.nih.gov/compound/145860478#section=2D-Structure
https://pubchem.ncbi.nlm.nih.gov/compound/145857575#section=2D-Structure
https://pubchem.ncbi.nlm.nih.gov/compound/145855340#section=2D-Structure
https://pubchem.ncbi.nlm.nih.gov/compound/145850737#section=2D-Structure
https://pubchem.ncbi.nlm.nih.gov/compound/145849641#section=2D-Structure
https://pubchem.ncbi.nlm.nih.gov/compound/145837271#section=2D-Structure
https://pubchem.ncbi.nlm.nih.gov/compound/145833089#section=2D-Structure
https://pubchem.ncbi.nlm.nih.gov/compound/145821720#section=2D-Structure
https://pubchem.ncbi.nlm.nih.gov/compound/145821432#section=2D-Structure
https://pubchem.ncbi.nlm.nih.gov/compound/145821429#section=2D-Structure
https://pubchem.ncbi.nlm.nih.gov/compound/145821428#section=2D-Structure
https://pubchem.ncbi.nlm.nih.gov/compound/145820321#section=2D-Structure
https://pubchem.ncbi.nlm.nih.gov/compound/145820320#section=2D-Structure
https://pubchem.ncbi.nlm.nih.gov/compound/145815088#section=2D-Structure
https://pubchem.ncbi.nlm.nih.gov/compound/145812476#section=2D-Structure
https://pubchem.ncbi.nlm.nih.gov/compound/145809921#section=2D-Structure
https://pubchem.ncbi.nlm.nih.gov/compound/145809200#section=2D-Structure
https://pubchem.ncbi.nlm.nih.gov/compound/145809011#section=2D-Structure
https://pubchem.ncbi.nlm.nih.gov/compound/145808286#section=2D-Structure
https://pubchem.ncbi.nlm.nih.gov/compound/145802769#section=2D-Structure
https://pubchem.ncbi.nlm.nih.gov/compound/145800603#section=2D-Structure
https://pubchem.ncbi.nlm.nih.gov/compound/145793927#section=2D-Structure
https://pubchem.ncbi.nlm.nih.gov/compound/145791494#section=2D-Structure
https://pubchem.ncbi.nlm.nih.gov/compound/145779594#section=2D-Structure
https://pubchem.ncbi.nlm.nih.gov/compound/145776021#section=2D-Structure
https://pubchem.ncbi.nlm.nih.gov/compound/145733090#section=2D-Structure
https://pubchem.ncbi.nlm.nih.gov/compound/145733077#section=2D-Structure
https://pubchem.ncbi.nlm.nih.gov/compound/145733047#section=2D-Structure
https://pubchem.ncbi.nlm.nih.gov/compound/145727998#section=2D-Structure
https://pubchem.ncbi.nlm.nih.gov/compound/145722169#section=2D-Structure
https://pubchem.ncbi.nlm.nih.gov/compound/145722118#section=2D-Structure
https://pubchem.ncbi.nlm.nih.gov/compound/145713955#section=2D-Structure
https://pubchem.ncbi.nlm.nih.gov/compound/145711680#section=2D-Structure
https://pubchem.ncbi.nlm.nih.gov/compound/145707135#section=2D-Structure
https://pubchem.ncbi.nlm.nih.gov/compound/145707124#section=2D-Structure
https://pubchem.ncbi.nlm.nih.gov/compound/145705663#section=2D-Structure
https://pubchem.ncbi.nlm.nih.gov/compound/145701701#section=2D-Structure
https://pubchem.ncbi.nlm.nih.gov/compound/145700214#section=2D-Structure
https://pubchem.ncbi.nlm.nih.gov/compound/145699997#section=2D-Structure
https://pubchem.ncbi.nlm.nih.gov/compound/145697542#section=2D-Structure
https://pubchem.ncbi.nlm.nih.gov/compound/145697540#section=2D-Structure
https://pubchem.ncbi.nlm.nih.gov/compound/145694206#section=2D-Structure
https://pubchem.ncbi.nlm.nih.gov/compound/145686204#section=2D-Structure
https://pubchem.ncbi.nlm.nih.gov/compound/145685253#section=2D-Structure

https://pubchem.ncbi.nlm.nih.gov/compound/145678992#section=2D-Structure
https://pubchem.ncbi.nlm.nih.gov/compound/145678576#section=2D-Structure
https://pubchem.ncbi.nlm.nih.gov/compound/145678328#section=2D-Structure
https://pubchem.ncbi.nlm.nih.gov/compound/145675737#section=2D-Structure
https://pubchem.ncbi.nlm.nih.gov/compound/145673478#section=2D-Structure
https://pubchem.ncbi.nlm.nih.gov/compound/145673471#section=2D-Structure
https://pubchem.ncbi.nlm.nih.gov/compound/145673463#section=2D-Structure
https://pubchem.ncbi.nlm.nih.gov/compound/145673462#section=2D-Structure
https://pubchem.ncbi.nlm.nih.gov/compound/145673459#section=2D-Structure
https://pubchem.ncbi.nlm.nih.gov/compound/145654954#section=2D-Structure
https://pubchem.ncbi.nlm.nih.gov/compound/145647750#section=2D-Structure
https://pubchem.ncbi.nlm.nih.gov/compound/145639470#section=2D-Structure
https://pubchem.ncbi.nlm.nih.gov/compound/145625160#section=2D-Structure
https://pubchem.ncbi.nlm.nih.gov/compound/145625#section=2D-Structure
https://pubchem.ncbi.nlm.nih.gov/compound/145605919#section=2D-Structure
https://pubchem.ncbi.nlm.nih.gov/compound/145599266#section=2D-Structure
https://pubchem.ncbi.nlm.nih.gov/compound/145598876#section=2D-Structure
https://pubchem.ncbi.nlm.nih.gov/compound/145598109#section=2D-Structure
https://pubchem.ncbi.nlm.nih.gov/compound/145598106#section=2D-Structure
https://pubchem.ncbi.nlm.nih.gov/compound/145598095#section=2D-Structure
https://pubchem.ncbi.nlm.nih.gov/compound/145598093#section=2D-Structure
https://pubchem.ncbi.nlm.nih.gov/compound/145598054#section=2D-Structure
https://pubchem.ncbi.nlm.nih.gov/compound/145596201#section=2D-Structure
https://pubchem.ncbi.nlm.nih.gov/compound/145590399#section=2D-Structure
https://pubchem.ncbi.nlm.nih.gov/compound/145583234#section=2D-Structure
https://pubchem.ncbi.nlm.nih.gov/compound/145578686#section=2D-Structure
https://pubchem.ncbi.nlm.nih.gov/compound/145574622#section=2D-Structure
https://pubchem.ncbi.nlm.nih.gov/compound/145572250#section=2D-Structure
https://pubchem.ncbi.nlm.nih.gov/compound/145571511#section=2D-Structure
https://pubchem.ncbi.nlm.nih.gov/compound/145570555#section=2D-Structure
https://pubchem.ncbi.nlm.nih.gov/compound/145567235#section=2D-Structure
https://pubchem.ncbi.nlm.nih.gov/compound/145550295#section=2D-Structure
https://pubchem.ncbi.nlm.nih.gov/compound/145541415#section=2D-Structure
https://pubchem.ncbi.nlm.nih.gov/compound/145539173#section=2D-Structure
https://pubchem.ncbi.nlm.nih.gov/compound/145533867#section=2D-Structure
https://pubchem.ncbi.nlm.nih.gov/compound/145528699#section=2D-Structure
https://pubchem.ncbi.nlm.nih.gov/compound/145520960#section=2D-Structure
https://pubchem.ncbi.nlm.nih.gov/compound/145518744#section=2D-Structure
https://pubchem.ncbi.nlm.nih.gov/compound/145507026#section=2D-Structure
https://pubchem.ncbi.nlm.nih.gov/compound/145498432#section=2D-Structure
https://pubchem.ncbi.nlm.nih.gov/compound/145498334#section=2D-Structure
https://pubchem.ncbi.nlm.nih.gov/compound/145497504#section=2D-Structure
https://pubchem.ncbi.nlm.nih.gov/compound/145497359#section=2D-Structure
https://pubchem.ncbi.nlm.nih.gov/compound/145493638#section=2D-Structure
https://pubchem.ncbi.nlm.nih.gov/compound/145490559#section=2D-Structure
https://pubchem.ncbi.nlm.nih.gov/compound/145489923#section=2D-Structure
https://pubchem.ncbi.nlm.nih.gov/compound/145485349#section=2D-Structure
https://pubchem.ncbi.nlm.nih.gov/compound/145477563#section=2D-Structure
https://pubchem.ncbi.nlm.nih.gov/compound/145475289#section=2D-Structure
https://pubchem.ncbi.nlm.nih.gov/compound/14546396#section=2D-Structure
https://pubchem.ncbi.nlm.nih.gov/compound/145450191#section=2D-Structure
https://pubchem.ncbi.nlm.nih.gov/compound/145446496#section=2D-Structure
https://pubchem.ncbi.nlm.nih.gov/compound/145430876#section=2D-Structure
https://pubchem.ncbi.nlm.nih.gov/compound/145423042#section=2D-Structure
https://pubchem.ncbi.nlm.nih.gov/compound/145421941#section=2D-Structure

https://pubchem.ncbi.nlm.nih.gov/compound/145419542#section=2D-Structure
https://pubchem.ncbi.nlm.nih.gov/compound/145405248#section=2D-Structure
https://pubchem.ncbi.nlm.nih.gov/compound/145396861#section=2D-Structure
https://pubchem.ncbi.nlm.nih.gov/compound/145390153#section=2D-Structure
https://pubchem.ncbi.nlm.nih.gov/compound/145367660#section=2D-Structure
https://pubchem.ncbi.nlm.nih.gov/compound/145360835#section=2D-Structure
https://pubchem.ncbi.nlm.nih.gov/compound/145358005#section=2D-Structure
https://pubchem.ncbi.nlm.nih.gov/compound/145356624#section=2D-Structure
https://pubchem.ncbi.nlm.nih.gov/compound/145356536#section=2D-Structure
https://pubchem.ncbi.nlm.nih.gov/compound/145345736#section=2D-Structure
https://pubchem.ncbi.nlm.nih.gov/compound/145340057#section=2D-Structure
https://pubchem.ncbi.nlm.nih.gov/compound/145332955#section=2D-Structure
https://pubchem.ncbi.nlm.nih.gov/compound/145332952#section=2D-Structure
https://pubchem.ncbi.nlm.nih.gov/compound/145322684#section=2D-Structure
https://pubchem.ncbi.nlm.nih.gov/compound/145322447#section=2D-Structure
https://pubchem.ncbi.nlm.nih.gov/compound/145322439#section=2D-Structure
https://pubchem.ncbi.nlm.nih.gov/compound/145312347#section=2D-Structure
https://pubchem.ncbi.nlm.nih.gov/compound/145312243#section=2D-Structure
https://pubchem.ncbi.nlm.nih.gov/compound/145292029#section=2D-Structure
https://pubchem.ncbi.nlm.nih.gov/compound/145274751#section=2D-Structure
https://pubchem.ncbi.nlm.nih.gov/compound/145272155#section=2D-Structure
https://pubchem.ncbi.nlm.nih.gov/compound/145257654#section=2D-Structure
https://pubchem.ncbi.nlm.nih.gov/compound/145233540#section=2D-Structure
https://pubchem.ncbi.nlm.nih.gov/compound/145221643#section=2D-Structure
https://pubchem.ncbi.nlm.nih.gov/compound/145195501#section=2D-Structure
https://pubchem.ncbi.nlm.nih.gov/compound/145192891#section=2D-Structure
https://pubchem.ncbi.nlm.nih.gov/compound/145191938#section=2D-Structure
https://pubchem.ncbi.nlm.nih.gov/compound/145179369#section=2D-Structure
https://pubchem.ncbi.nlm.nih.gov/compound/145174665#section=2D-Structure
https://pubchem.ncbi.nlm.nih.gov/compound/145173414#section=2D-Structure
https://pubchem.ncbi.nlm.nih.gov/compound/145161989#section=2D-Structure
https://pubchem.ncbi.nlm.nih.gov/compound/145158909#section=2D-Structure
https://pubchem.ncbi.nlm.nih.gov/compound/145156109#section=2D-Structure
https://pubchem.ncbi.nlm.nih.gov/compound/145156097#section=2D-Structure
https://pubchem.ncbi.nlm.nih.gov/compound/145153695#section=2D-Structure
https://pubchem.ncbi.nlm.nih.gov/compound/145152222#section=2D-Structure
https://pubchem.ncbi.nlm.nih.gov/compound/145130224#section=2D-Structure
https://pubchem.ncbi.nlm.nih.gov/compound/145123007#section=2D-Structure
https://pubchem.ncbi.nlm.nih.gov/compound/145119998#section=2D-Structure
https://pubchem.ncbi.nlm.nih.gov/compound/145119941#section=2D-Structure
https://pubchem.ncbi.nlm.nih.gov/compound/145119087#section=2D-Structure
https://pubchem.ncbi.nlm.nih.gov/compound/145118817#section=2D-Structure
https://pubchem.ncbi.nlm.nih.gov/compound/14511465#section=2D-Structure
https://pubchem.ncbi.nlm.nih.gov/compound/145098424#section=2D-Structure
https://pubchem.ncbi.nlm.nih.gov/compound/145092078#section=2D-Structure
https://pubchem.ncbi.nlm.nih.gov/compound/145077814#section=2D-Structure
https://pubchem.ncbi.nlm.nih.gov/compound/145065299#section=2D-Structure
https://pubchem.ncbi.nlm.nih.gov/compound/145065258#section=2D-Structure
https://pubchem.ncbi.nlm.nih.gov/compound/145063805#section=2D-Structure
https://pubchem.ncbi.nlm.nih.gov/compound/145048159#section=2D-Structure
https://pubchem.ncbi.nlm.nih.gov/compound/145010767#section=2D-Structure
https://pubchem.ncbi.nlm.nih.gov/compound/144989126#section=2D-Structure
https://pubchem.ncbi.nlm.nih.gov/compound/144987893#section=2D-Structure
https://pubchem.ncbi.nlm.nih.gov/compound/144983268#section=2D-Structure
https://pubchem.ncbi.nlm.nih.gov/compound/144961413#section=2D-Structure

https://pubchem.ncbi.nlm.nih.gov/compound/144956262#section=2D-Structure
https://pubchem.ncbi.nlm.nih.gov/compound/144956253#section=2D-Structure
https://pubchem.ncbi.nlm.nih.gov/compound/144956251#section=2D-Structure
https://pubchem.ncbi.nlm.nih.gov/compound/144942178#section=2D-Structure
https://pubchem.ncbi.nlm.nih.gov/compound/144934693#section=2D-Structure
https://pubchem.ncbi.nlm.nih.gov/compound/144931095#section=2D-Structure
https://pubchem.ncbi.nlm.nih.gov/compound/144920480#section=2D-Structure
https://pubchem.ncbi.nlm.nih.gov/compound/144918704#section=2D-Structure
https://pubchem.ncbi.nlm.nih.gov/compound/144896054#section=2D-Structure
https://pubchem.ncbi.nlm.nih.gov/compound/144896052#section=2D-Structure
https://pubchem.ncbi.nlm.nih.gov/compound/144887064#section=2D-Structure
https://pubchem.ncbi.nlm.nih.gov/compound/144887061#section=2D-Structure
https://pubchem.ncbi.nlm.nih.gov/compound/144887052#section=2D-Structure
https://pubchem.ncbi.nlm.nih.gov/compound/144865738#section=2D-Structure
https://pubchem.ncbi.nlm.nih.gov/compound/144856563#section=2D-Structure
https://pubchem.ncbi.nlm.nih.gov/compound/144853791#section=2D-Structure
https://pubchem.ncbi.nlm.nih.gov/compound/144846870#section=2D-Structure
https://pubchem.ncbi.nlm.nih.gov/compound/144846864#section=2D-Structure
https://pubchem.ncbi.nlm.nih.gov/compound/144844049#section=2D-Structure
https://pubchem.ncbi.nlm.nih.gov/compound/144839314#section=2D-Structure
https://pubchem.ncbi.nlm.nih.gov/compound/144816048#section=2D-Structure
https://pubchem.ncbi.nlm.nih.gov/compound/144799826#section=2D-Structure
https://pubchem.ncbi.nlm.nih.gov/compound/144796958#section=2D-Structure
https://pubchem.ncbi.nlm.nih.gov/compound/144790125#section=2D-Structure
https://pubchem.ncbi.nlm.nih.gov/compound/144790124#section=2D-Structure
https://pubchem.ncbi.nlm.nih.gov/compound/144790116#section=2D-Structure
https://pubchem.ncbi.nlm.nih.gov/compound/144790115#section=2D-Structure
https://pubchem.ncbi.nlm.nih.gov/compound/144788689#section=2D-Structure
https://pubchem.ncbi.nlm.nih.gov/compound/144775363#section=2D-Structure
https://pubchem.ncbi.nlm.nih.gov/compound/144769212#section=2D-Structure
https://pubchem.ncbi.nlm.nih.gov/compound/144768811#section=2D-Structure
https://pubchem.ncbi.nlm.nih.gov/compound/144766026#section=2D-Structure
https://pubchem.ncbi.nlm.nih.gov/compound/144764792#section=2D-Structure
https://pubchem.ncbi.nlm.nih.gov/compound/144761807#section=2D-Structure
https://pubchem.ncbi.nlm.nih.gov/compound/144761030#section=2D-Structure
https://pubchem.ncbi.nlm.nih.gov/compound/144761028#section=2D-Structure
https://pubchem.ncbi.nlm.nih.gov/compound/144761009#section=2D-Structure
https://pubchem.ncbi.nlm.nih.gov/compound/144761003#section=2D-Structure
https://pubchem.ncbi.nlm.nih.gov/compound/144760996#section=2D-Structure
https://pubchem.ncbi.nlm.nih.gov/compound/144750353#section=2D-Structure
https://pubchem.ncbi.nlm.nih.gov/compound/144749416#section=2D-Structure
https://pubchem.ncbi.nlm.nih.gov/compound/144745153#section=2D-Structure
https://pubchem.ncbi.nlm.nih.gov/compound/144725572#section=2D-Structure
https://pubchem.ncbi.nlm.nih.gov/compound/144724957#section=2D-Structure
https://pubchem.ncbi.nlm.nih.gov/compound/144724919#section=2D-Structure
https://pubchem.ncbi.nlm.nih.gov/compound/144723204#section=2D-Structure
https://pubchem.ncbi.nlm.nih.gov/compound/14471519#section=2D-Structure
https://pubchem.ncbi.nlm.nih.gov/compound/144711#section=2D-Structure
https://pubchem.ncbi.nlm.nih.gov/compound/144706039#section=2D-Structure
https://pubchem.ncbi.nlm.nih.gov/compound/144696635#section=2D-Structure
https://pubchem.ncbi.nlm.nih.gov/compound/144687920#section=2D-Structure
https://pubchem.ncbi.nlm.nih.gov/compound/144672359#section=2D-Structure
https://pubchem.ncbi.nlm.nih.gov/compound/144670648#section=2D-Structure
https://pubchem.ncbi.nlm.nih.gov/compound/144659288#section=2D-Structure
https://pubchem.ncbi.nlm.nih.gov/compound/144649477#section=2D-Structure

https://pubchem.ncbi.nlm.nih.gov/compound/144648560#section=2D-Structure
https://pubchem.ncbi.nlm.nih.gov/compound/144648209#section=2D-Structure
https://pubchem.ncbi.nlm.nih.gov/compound/144646204#section=2D-Structure
https://pubchem.ncbi.nlm.nih.gov/compound/144642800#section=2D-Structure
https://pubchem.ncbi.nlm.nih.gov/compound/144636024#section=2D-Structure
https://pubchem.ncbi.nlm.nih.gov/compound/144636023#section=2D-Structure
https://pubchem.ncbi.nlm.nih.gov/compound/144636022#section=2D-Structure
https://pubchem.ncbi.nlm.nih.gov/compound/144636003#section=2D-Structure
https://pubchem.ncbi.nlm.nih.gov/compound/144635991#section=2D-Structure
https://pubchem.ncbi.nlm.nih.gov/compound/144635984#section=2D-Structure
https://pubchem.ncbi.nlm.nih.gov/compound/144627059#section=2D-Structure
https://pubchem.ncbi.nlm.nih.gov/compound/144626438#section=2D-Structure
https://pubchem.ncbi.nlm.nih.gov/compound/144607039#section=2D-Structure
https://pubchem.ncbi.nlm.nih.gov/compound/144602354#section=2D-Structure
https://pubchem.ncbi.nlm.nih.gov/compound/144599511#section=2D-Structure
https://pubchem.ncbi.nlm.nih.gov/compound/144598204#section=2D-Structure
https://pubchem.ncbi.nlm.nih.gov/compound/144595382#section=2D-Structure
https://pubchem.ncbi.nlm.nih.gov/compound/144594801#section=2D-Structure
https://pubchem.ncbi.nlm.nih.gov/compound/144591208#section=2D-Structure
https://pubchem.ncbi.nlm.nih.gov/compound/144590072#section=2D-Structure
https://pubchem.ncbi.nlm.nih.gov/compound/144588033#section=2D-Structure
https://pubchem.ncbi.nlm.nih.gov/compound/144587671#section=2D-Structure
https://pubchem.ncbi.nlm.nih.gov/compound/144577349#section=2D-Structure
https://pubchem.ncbi.nlm.nih.gov/compound/144548742#section=2D-Structure
https://pubchem.ncbi.nlm.nih.gov/compound/144546366#section=2D-Structure
https://pubchem.ncbi.nlm.nih.gov/compound/144546144#section=2D-Structure
https://pubchem.ncbi.nlm.nih.gov/compound/144546005#section=2D-Structure
https://pubchem.ncbi.nlm.nih.gov/compound/144545385#section=2D-Structure
https://pubchem.ncbi.nlm.nih.gov/compound/144544372#section=2D-Structure
https://pubchem.ncbi.nlm.nih.gov/compound/144544295#section=2D-Structure
https://pubchem.ncbi.nlm.nih.gov/compound/144543491#section=2D-Structure
https://pubchem.ncbi.nlm.nih.gov/compound/144536857#section=2D-Structure
https://pubchem.ncbi.nlm.nih.gov/compound/144532652#section=2D-Structure
https://pubchem.ncbi.nlm.nih.gov/compound/144532617#section=2D-Structure
https://pubchem.ncbi.nlm.nih.gov/compound/144532179#section=2D-Structure
https://pubchem.ncbi.nlm.nih.gov/compound/144521580#section=2D-Structure
https://pubchem.ncbi.nlm.nih.gov/compound/14451981#section=2D-Structure
https://pubchem.ncbi.nlm.nih.gov/compound/144516985#section=2D-Structure
https://pubchem.ncbi.nlm.nih.gov/compound/144509315#section=2D-Structure
https://pubchem.ncbi.nlm.nih.gov/compound/144506422#section=2D-Structure
https://pubchem.ncbi.nlm.nih.gov/compound/144498470#section=2D-Structure
https://pubchem.ncbi.nlm.nih.gov/compound/144497083#section=2D-Structure
https://pubchem.ncbi.nlm.nih.gov/compound/144490305#section=2D-Structure
https://pubchem.ncbi.nlm.nih.gov/compound/144490301#section=2D-Structure
https://pubchem.ncbi.nlm.nih.gov/compound/144487661#section=2D-Structure
https://pubchem.ncbi.nlm.nih.gov/compound/144480847#section=2D-Structure
https://pubchem.ncbi.nlm.nih.gov/compound/144479600#section=2D-Structure
https://pubchem.ncbi.nlm.nih.gov/compound/144477497#section=2D-Structure
https://pubchem.ncbi.nlm.nih.gov/compound/144476193#section=2D-Structure
https://pubchem.ncbi.nlm.nih.gov/compound/144475886#section=2D-Structure
https://pubchem.ncbi.nlm.nih.gov/compound/144467490#section=2D-Structure
https://pubchem.ncbi.nlm.nih.gov/compound/144465855#section=2D-Structure
https://pubchem.ncbi.nlm.nih.gov/compound/144464025#section=2D-Structure
https://pubchem.ncbi.nlm.nih.gov/compound/144455645#section=2D-Structure
https://pubchem.ncbi.nlm.nih.gov/compound/144446112#section=2D-Structure

https://pubchem.ncbi.nlm.nih.gov/compound/144445995#section=2D-Structure
https://pubchem.ncbi.nlm.nih.gov/compound/144443943#section=2D-Structure
https://pubchem.ncbi.nlm.nih.gov/compound/144427648#section=2D-Structure
https://pubchem.ncbi.nlm.nih.gov/compound/144427435#section=2D-Structure
https://pubchem.ncbi.nlm.nih.gov/compound/144420741#section=2D-Structure
https://pubchem.ncbi.nlm.nih.gov/compound/144404923#section=2D-Structure
https://pubchem.ncbi.nlm.nih.gov/compound/144402775#section=2D-Structure
https://pubchem.ncbi.nlm.nih.gov/compound/144401310#section=2D-Structure
https://pubchem.ncbi.nlm.nih.gov/compound/144398955#section=2D-Structure
https://pubchem.ncbi.nlm.nih.gov/compound/144398935#section=2D-Structure
https://pubchem.ncbi.nlm.nih.gov/compound/144383490#section=2D-Structure
https://pubchem.ncbi.nlm.nih.gov/compound/14438031#section=2D-Structure
https://pubchem.ncbi.nlm.nih.gov/compound/144376823#section=2D-Structure
https://pubchem.ncbi.nlm.nih.gov/compound/144359655#section=2D-Structure
https://pubchem.ncbi.nlm.nih.gov/compound/144359584#section=2D-Structure
https://pubchem.ncbi.nlm.nih.gov/compound/144357166#section=2D-Structure
https://pubchem.ncbi.nlm.nih.gov/compound/144342998#section=2D-Structure
https://pubchem.ncbi.nlm.nih.gov/compound/144336786#section=2D-Structure
https://pubchem.ncbi.nlm.nih.gov/compound/144318082#section=2D-Structure
https://pubchem.ncbi.nlm.nih.gov/compound/144315095#section=2D-Structure
https://pubchem.ncbi.nlm.nih.gov/compound/144313235#section=2D-Structure
https://pubchem.ncbi.nlm.nih.gov/compound/144313125#section=2D-Structure
https://pubchem.ncbi.nlm.nih.gov/compound/144308278#section=2D-Structure
https://pubchem.ncbi.nlm.nih.gov/compound/144308204#section=2D-Structure
https://pubchem.ncbi.nlm.nih.gov/compound/144307981#section=2D-Structure
https://pubchem.ncbi.nlm.nih.gov/compound/144285550#section=2D-Structure
https://pubchem.ncbi.nlm.nih.gov/compound/144280744#section=2D-Structure
https://pubchem.ncbi.nlm.nih.gov/compound/144279#section=2D-Structure
https://pubchem.ncbi.nlm.nih.gov/compound/144276613#section=2D-Structure
https://pubchem.ncbi.nlm.nih.gov/compound/144276380#section=2D-Structure
https://pubchem.ncbi.nlm.nih.gov/compound/144262350#section=2D-Structure
https://pubchem.ncbi.nlm.nih.gov/compound/144261673#section=2D-Structure
https://pubchem.ncbi.nlm.nih.gov/compound/144261581#section=2D-Structure
https://pubchem.ncbi.nlm.nih.gov/compound/144259#section=2D-Structure
https://pubchem.ncbi.nlm.nih.gov/compound/144257429#section=2D-Structure
https://pubchem.ncbi.nlm.nih.gov/compound/144257064#section=2D-Structure
https://pubchem.ncbi.nlm.nih.gov/compound/144243955#section=2D-Structure
https://pubchem.ncbi.nlm.nih.gov/compound/144242008#section=2D-Structure
https://pubchem.ncbi.nlm.nih.gov/compound/144241490#section=2D-Structure
https://pubchem.ncbi.nlm.nih.gov/compound/144236371#section=2D-Structure
https://pubchem.ncbi.nlm.nih.gov/compound/144236286#section=2D-Structure
https://pubchem.ncbi.nlm.nih.gov/compound/144231152#section=2D-Structure
https://pubchem.ncbi.nlm.nih.gov/compound/144226737#section=2D-Structure
https://pubchem.ncbi.nlm.nih.gov/compound/144222180#section=2D-Structure
https://pubchem.ncbi.nlm.nih.gov/compound/144220228#section=2D-Structure
https://pubchem.ncbi.nlm.nih.gov/compound/144211757#section=2D-Structure
https://pubchem.ncbi.nlm.nih.gov/compound/144201196#section=2D-Structure
https://pubchem.ncbi.nlm.nih.gov/compound/144196887#section=2D-Structure
https://pubchem.ncbi.nlm.nih.gov/compound/144188633#section=2D-Structure
https://pubchem.ncbi.nlm.nih.gov/compound/14418250#section=2D-Structure
https://pubchem.ncbi.nlm.nih.gov/compound/144178978#section=2D-Structure
https://pubchem.ncbi.nlm.nih.gov/compound/144178820#section=2D-Structure
https://pubchem.ncbi.nlm.nih.gov/compound/144177848#section=2D-Structure
https://pubchem.ncbi.nlm.nih.gov/compound/144174950#section=2D-Structure
https://pubchem.ncbi.nlm.nih.gov/compound/14417391#section=2D-Structure

https://pubchem.ncbi.nlm.nih.gov/compound/144171832#section=2D-Structure
https://pubchem.ncbi.nlm.nih.gov/compound/144168343#section=2D-Structure
https://pubchem.ncbi.nlm.nih.gov/compound/144160304#section=2D-Structure
https://pubchem.ncbi.nlm.nih.gov/compound/144160237#section=2D-Structure
https://pubchem.ncbi.nlm.nih.gov/compound/144155482#section=2D-Structure
https://pubchem.ncbi.nlm.nih.gov/compound/144155366#section=2D-Structure
https://pubchem.ncbi.nlm.nih.gov/compound/144144417#section=2D-Structure
https://pubchem.ncbi.nlm.nih.gov/compound/144136987#section=2D-Structure
https://pubchem.ncbi.nlm.nih.gov/compound/144130401#section=2D-Structure
https://pubchem.ncbi.nlm.nih.gov/compound/144125451#section=2D-Structure
https://pubchem.ncbi.nlm.nih.gov/compound/144125318#section=2D-Structure
https://pubchem.ncbi.nlm.nih.gov/compound/144125136#section=2D-Structure
https://pubchem.ncbi.nlm.nih.gov/compound/144119133#section=2D-Structure
https://pubchem.ncbi.nlm.nih.gov/compound/144118466#section=2D-Structure
https://pubchem.ncbi.nlm.nih.gov/compound/144104038#section=2D-Structure
https://pubchem.ncbi.nlm.nih.gov/compound/144101781#section=2D-Structure
https://pubchem.ncbi.nlm.nih.gov/compound/144095717#section=2D-Structure
https://pubchem.ncbi.nlm.nih.gov/compound/144090050#section=2D-Structure
https://pubchem.ncbi.nlm.nih.gov/compound/144089875#section=2D-Structure
https://pubchem.ncbi.nlm.nih.gov/compound/144089810#section=2D-Structure
https://pubchem.ncbi.nlm.nih.gov/compound/144089599#section=2D-Structure
https://pubchem.ncbi.nlm.nih.gov/compound/144084144#section=2D-Structure
https://pubchem.ncbi.nlm.nih.gov/compound/144080453#section=2D-Structure
https://pubchem.ncbi.nlm.nih.gov/compound/144078934#section=2D-Structure
https://pubchem.ncbi.nlm.nih.gov/compound/144077363#section=2D-Structure
https://pubchem.ncbi.nlm.nih.gov/compound/144075140#section=2D-Structure
https://pubchem.ncbi.nlm.nih.gov/compound/144075093#section=2D-Structure
https://pubchem.ncbi.nlm.nih.gov/compound/144059632#section=2D-Structure
https://pubchem.ncbi.nlm.nih.gov/compound/144056218#section=2D-Structure
https://pubchem.ncbi.nlm.nih.gov/compound/144055400#section=2D-Structure
https://pubchem.ncbi.nlm.nih.gov/compound/144054447#section=2D-Structure
https://pubchem.ncbi.nlm.nih.gov/compound/144045429#section=2D-Structure
https://pubchem.ncbi.nlm.nih.gov/compound/144045428#section=2D-Structure
https://pubchem.ncbi.nlm.nih.gov/compound/144045425#section=2D-Structure
https://pubchem.ncbi.nlm.nih.gov/compound/144044437#section=2D-Structure
https://pubchem.ncbi.nlm.nih.gov/compound/144031586#section=2D-Structure
https://pubchem.ncbi.nlm.nih.gov/compound/144029851#section=2D-Structure
https://pubchem.ncbi.nlm.nih.gov/compound/144025167#section=2D-Structure
https://pubchem.ncbi.nlm.nih.gov/compound/144023034#section=2D-Structure
https://pubchem.ncbi.nlm.nih.gov/compound/144021528#section=2D-Structure
https://pubchem.ncbi.nlm.nih.gov/compound/144021291#section=2D-Structure
https://pubchem.ncbi.nlm.nih.gov/compound/144017947#section=2D-Structure
https://pubchem.ncbi.nlm.nih.gov/compound/144009624#section=2D-Structure
https://pubchem.ncbi.nlm.nih.gov/compound/144007445#section=2D-Structure
https://pubchem.ncbi.nlm.nih.gov/compound/144007413#section=2D-Structure
https://pubchem.ncbi.nlm.nih.gov/compound/144005303#section=2D-Structure
https://pubchem.ncbi.nlm.nih.gov/compound/144003629#section=2D-Structure
https://pubchem.ncbi.nlm.nih.gov/compound/143996328#section=2D-Structure
https://pubchem.ncbi.nlm.nih.gov/compound/143993752#section=2D-Structure
https://pubchem.ncbi.nlm.nih.gov/compound/143983374#section=2D-Structure
https://pubchem.ncbi.nlm.nih.gov/compound/143982974#section=2D-Structure
https://pubchem.ncbi.nlm.nih.gov/compound/143966066#section=2D-Structure
https://pubchem.ncbi.nlm.nih.gov/compound/143964137#section=2D-Structure
https://pubchem.ncbi.nlm.nih.gov/compound/143964134#section=2D-Structure
https://pubchem.ncbi.nlm.nih.gov/compound/143936176#section=2D-Structure

https://pubchem.ncbi.nlm.nih.gov/compound/143917535#section=2D-Structure
https://pubchem.ncbi.nlm.nih.gov/compound/143902532#section=2D-Structure
https://pubchem.ncbi.nlm.nih.gov/compound/143890945#section=2D-Structure
https://pubchem.ncbi.nlm.nih.gov/compound/143877770#section=2D-Structure
https://pubchem.ncbi.nlm.nih.gov/compound/143870462#section=2D-Structure
https://pubchem.ncbi.nlm.nih.gov/compound/143866490#section=2D-Structure
https://pubchem.ncbi.nlm.nih.gov/compound/143861159#section=2D-Structure
https://pubchem.ncbi.nlm.nih.gov/compound/143861114#section=2D-Structure
https://pubchem.ncbi.nlm.nih.gov/compound/143860115#section=2D-Structure
https://pubchem.ncbi.nlm.nih.gov/compound/143859019#section=2D-Structure
https://pubchem.ncbi.nlm.nih.gov/compound/143858547#section=2D-Structure
https://pubchem.ncbi.nlm.nih.gov/compound/143854550#section=2D-Structure
https://pubchem.ncbi.nlm.nih.gov/compound/143846935#section=2D-Structure
https://pubchem.ncbi.nlm.nih.gov/compound/143830233#section=2D-Structure
https://pubchem.ncbi.nlm.nih.gov/compound/143830227#section=2D-Structure
https://pubchem.ncbi.nlm.nih.gov/compound/143829571#section=2D-Structure
https://pubchem.ncbi.nlm.nih.gov/compound/143812621#section=2D-Structure
https://pubchem.ncbi.nlm.nih.gov/compound/143811126#section=2D-Structure
https://pubchem.ncbi.nlm.nih.gov/compound/143802904#section=2D-Structure
https://pubchem.ncbi.nlm.nih.gov/compound/143796#section=2D-Structure
https://pubchem.ncbi.nlm.nih.gov/compound/143792685#section=2D-Structure
https://pubchem.ncbi.nlm.nih.gov/compound/143786739#section=2D-Structure
https://pubchem.ncbi.nlm.nih.gov/compound/143776426#section=2D-Structure
https://pubchem.ncbi.nlm.nih.gov/compound/143775410#section=2D-Structure
https://pubchem.ncbi.nlm.nih.gov/compound/143769334#section=2D-Structure
https://pubchem.ncbi.nlm.nih.gov/compound/143765482#section=2D-Structure
https://pubchem.ncbi.nlm.nih.gov/compound/143765480#section=2D-Structure
https://pubchem.ncbi.nlm.nih.gov/compound/143765473#section=2D-Structure
https://pubchem.ncbi.nlm.nih.gov/compound/143764766#section=2D-Structure
https://pubchem.ncbi.nlm.nih.gov/compound/143760722#section=2D-Structure
https://pubchem.ncbi.nlm.nih.gov/compound/143760690#section=2D-Structure
https://pubchem.ncbi.nlm.nih.gov/compound/143753613#section=2D-Structure
https://pubchem.ncbi.nlm.nih.gov/compound/143749243#section=2D-Structure
https://pubchem.ncbi.nlm.nih.gov/compound/143743650#section=2D-Structure
https://pubchem.ncbi.nlm.nih.gov/compound/143742529#section=2D-Structure
https://pubchem.ncbi.nlm.nih.gov/compound/143742391#section=2D-Structure
https://pubchem.ncbi.nlm.nih.gov/compound/143742329#section=2D-Structure
https://pubchem.ncbi.nlm.nih.gov/compound/143742281#section=2D-Structure
https://pubchem.ncbi.nlm.nih.gov/compound/143742280#section=2D-Structure
https://pubchem.ncbi.nlm.nih.gov/compound/143731094#section=2D-Structure
https://pubchem.ncbi.nlm.nih.gov/compound/143722515#section=2D-Structure
https://pubchem.ncbi.nlm.nih.gov/compound/143716588#section=2D-Structure
https://pubchem.ncbi.nlm.nih.gov/compound/143716562#section=2D-Structure
https://pubchem.ncbi.nlm.nih.gov/compound/143716332#section=2D-Structure
https://pubchem.ncbi.nlm.nih.gov/compound/143715859#section=2D-Structure
https://pubchem.ncbi.nlm.nih.gov/compound/143715768#section=2D-Structure
https://pubchem.ncbi.nlm.nih.gov/compound/143715749#section=2D-Structure
https://pubchem.ncbi.nlm.nih.gov/compound/143715531#section=2D-Structure
https://pubchem.ncbi.nlm.nih.gov/compound/143711042#section=2D-Structure
https://pubchem.ncbi.nlm.nih.gov/compound/143694469#section=2D-Structure
https://pubchem.ncbi.nlm.nih.gov/compound/143693665#section=2D-Structure
https://pubchem.ncbi.nlm.nih.gov/compound/143688364#section=2D-Structure
https://pubchem.ncbi.nlm.nih.gov/compound/143684631#section=2D-Structure
https://pubchem.ncbi.nlm.nih.gov/compound/143672524#section=2D-Structure
https://pubchem.ncbi.nlm.nih.gov/compound/143662038#section=2D-Structure

https://pubchem.ncbi.nlm.nih.gov/compound/143660285#section=2D-Structure
https://pubchem.ncbi.nlm.nih.gov/compound/143660271#section=2D-Structure
https://pubchem.ncbi.nlm.nih.gov/compound/143660258#section=2D-Structure
https://pubchem.ncbi.nlm.nih.gov/compound/143658743#section=2D-Structure
https://pubchem.ncbi.nlm.nih.gov/compound/143657188#section=2D-Structure
https://pubchem.ncbi.nlm.nih.gov/compound/143652763#section=2D-Structure
https://pubchem.ncbi.nlm.nih.gov/compound/143640993#section=2D-Structure
https://pubchem.ncbi.nlm.nih.gov/compound/143637061#section=2D-Structure
https://pubchem.ncbi.nlm.nih.gov/compound/143624670#section=2D-Structure
https://pubchem.ncbi.nlm.nih.gov/compound/143620726#section=2D-Structure
https://pubchem.ncbi.nlm.nih.gov/compound/143620502#section=2D-Structure
https://pubchem.ncbi.nlm.nih.gov/compound/143619998#section=2D-Structure
https://pubchem.ncbi.nlm.nih.gov/compound/143619442#section=2D-Structure
https://pubchem.ncbi.nlm.nih.gov/compound/143618827#section=2D-Structure
https://pubchem.ncbi.nlm.nih.gov/compound/143618814#section=2D-Structure
https://pubchem.ncbi.nlm.nih.gov/compound/143604795#section=2D-Structure
https://pubchem.ncbi.nlm.nih.gov/compound/143599914#section=2D-Structure
https://pubchem.ncbi.nlm.nih.gov/compound/143594408#section=2D-Structure
https://pubchem.ncbi.nlm.nih.gov/compound/143592387#section=2D-Structure
https://pubchem.ncbi.nlm.nih.gov/compound/143590936#section=2D-Structure
https://pubchem.ncbi.nlm.nih.gov/compound/14359079#section=2D-Structure
https://pubchem.ncbi.nlm.nih.gov/compound/14359077#section=2D-Structure
https://pubchem.ncbi.nlm.nih.gov/compound/14359073#section=2D-Structure
https://pubchem.ncbi.nlm.nih.gov/compound/143587#section=2D-Structure
https://pubchem.ncbi.nlm.nih.gov/compound/143582927#section=2D-Structure
https://pubchem.ncbi.nlm.nih.gov/compound/143582665#section=2D-Structure
https://pubchem.ncbi.nlm.nih.gov/compound/143578#section=2D-Structure
https://pubchem.ncbi.nlm.nih.gov/compound/143572376#section=2D-Structure
https://pubchem.ncbi.nlm.nih.gov/compound/143570421#section=2D-Structure
https://pubchem.ncbi.nlm.nih.gov/compound/143568231#section=2D-Structure
https://pubchem.ncbi.nlm.nih.gov/compound/143563#section=2D-Structure
https://pubchem.ncbi.nlm.nih.gov/compound/143539684#section=2D-Structure
https://pubchem.ncbi.nlm.nih.gov/compound/143535649#section=2D-Structure
https://pubchem.ncbi.nlm.nih.gov/compound/143532382#section=2D-Structure
https://pubchem.ncbi.nlm.nih.gov/compound/143530676#section=2D-Structure
https://pubchem.ncbi.nlm.nih.gov/compound/143519594#section=2D-Structure
https://pubchem.ncbi.nlm.nih.gov/compound/143504992#section=2D-Structure
https://pubchem.ncbi.nlm.nih.gov/compound/143501410#section=2D-Structure
https://pubchem.ncbi.nlm.nih.gov/compound/143499964#section=2D-Structure
https://pubchem.ncbi.nlm.nih.gov/compound/143482880#section=2D-Structure
https://pubchem.ncbi.nlm.nih.gov/compound/143481361#section=2D-Structure
https://pubchem.ncbi.nlm.nih.gov/compound/143477726#section=2D-Structure
https://pubchem.ncbi.nlm.nih.gov/compound/143477005#section=2D-Structure
https://pubchem.ncbi.nlm.nih.gov/compound/143469770#section=2D-Structure
https://pubchem.ncbi.nlm.nih.gov/compound/143467321#section=2D-Structure
https://pubchem.ncbi.nlm.nih.gov/compound/143460989#section=2D-Structure
https://pubchem.ncbi.nlm.nih.gov/compound/143452944#section=2D-Structure
https://pubchem.ncbi.nlm.nih.gov/compound/143448247#section=2D-Structure
https://pubchem.ncbi.nlm.nih.gov/compound/143448240#section=2D-Structure
https://pubchem.ncbi.nlm.nih.gov/compound/14343272#section=2D-Structure
https://pubchem.ncbi.nlm.nih.gov/compound/143427984#section=2D-Structure
https://pubchem.ncbi.nlm.nih.gov/compound/143418934#section=2D-Structure
https://pubchem.ncbi.nlm.nih.gov/compound/143408849#section=2D-Structure
https://pubchem.ncbi.nlm.nih.gov/compound/143403419#section=2D-Structure
https://pubchem.ncbi.nlm.nih.gov/compound/143400474#section=2D-Structure

https://pubchem.ncbi.nlm.nih.gov/compound/143400464#section=2D-Structure
https://pubchem.ncbi.nlm.nih.gov/compound/143400350#section=2D-Structure
https://pubchem.ncbi.nlm.nih.gov/compound/143396152#section=2D-Structure
https://pubchem.ncbi.nlm.nih.gov/compound/143393418#section=2D-Structure
https://pubchem.ncbi.nlm.nih.gov/compound/143385882#section=2D-Structure
https://pubchem.ncbi.nlm.nih.gov/compound/143379#section=2D-Structure
https://pubchem.ncbi.nlm.nih.gov/compound/143370263#section=2D-Structure
https://pubchem.ncbi.nlm.nih.gov/compound/143365667#section=2D-Structure
https://pubchem.ncbi.nlm.nih.gov/compound/143353039#section=2D-Structure
https://pubchem.ncbi.nlm.nih.gov/compound/143349571#section=2D-Structure
https://pubchem.ncbi.nlm.nih.gov/compound/143348618#section=2D-Structure
https://pubchem.ncbi.nlm.nih.gov/compound/143341733#section=2D-Structure
https://pubchem.ncbi.nlm.nih.gov/compound/143338872#section=2D-Structure
https://pubchem.ncbi.nlm.nih.gov/compound/143336397#section=2D-Structure
https://pubchem.ncbi.nlm.nih.gov/compound/143335088#section=2D-Structure
https://pubchem.ncbi.nlm.nih.gov/compound/143333770#section=2D-Structure
https://pubchem.ncbi.nlm.nih.gov/compound/143324988#section=2D-Structure
https://pubchem.ncbi.nlm.nih.gov/compound/143310556#section=2D-Structure
https://pubchem.ncbi.nlm.nih.gov/compound/143309604#section=2D-Structure
https://pubchem.ncbi.nlm.nih.gov/compound/143305651#section=2D-Structure
https://pubchem.ncbi.nlm.nih.gov/compound/143305007#section=2D-Structure
https://pubchem.ncbi.nlm.nih.gov/compound/143295593#section=2D-Structure
https://pubchem.ncbi.nlm.nih.gov/compound/143295133#section=2D-Structure
https://pubchem.ncbi.nlm.nih.gov/compound/143291584#section=2D-Structure
https://pubchem.ncbi.nlm.nih.gov/compound/143289408#section=2D-Structure
https://pubchem.ncbi.nlm.nih.gov/compound/143288927#section=2D-Structure
https://pubchem.ncbi.nlm.nih.gov/compound/143287925#section=2D-Structure
https://pubchem.ncbi.nlm.nih.gov/compound/143283966#section=2D-Structure
https://pubchem.ncbi.nlm.nih.gov/compound/143282420#section=2D-Structure
https://pubchem.ncbi.nlm.nih.gov/compound/143278482#section=2D-Structure
https://pubchem.ncbi.nlm.nih.gov/compound/143269006#section=2D-Structure
https://pubchem.ncbi.nlm.nih.gov/compound/143261899#section=2D-Structure
https://pubchem.ncbi.nlm.nih.gov/compound/143261257#section=2D-Structure
https://pubchem.ncbi.nlm.nih.gov/compound/143261256#section=2D-Structure
https://pubchem.ncbi.nlm.nih.gov/compound/143261255#section=2D-Structure
https://pubchem.ncbi.nlm.nih.gov/compound/143260231#section=2D-Structure
https://pubchem.ncbi.nlm.nih.gov/compound/143259587#section=2D-Structure
https://pubchem.ncbi.nlm.nih.gov/compound/143259497#section=2D-Structure
https://pubchem.ncbi.nlm.nih.gov/compound/143256720#section=2D-Structure
https://pubchem.ncbi.nlm.nih.gov/compound/143254267#section=2D-Structure
https://pubchem.ncbi.nlm.nih.gov/compound/143253962#section=2D-Structure
https://pubchem.ncbi.nlm.nih.gov/compound/143253759#section=2D-Structure
https://pubchem.ncbi.nlm.nih.gov/compound/143253725#section=2D-Structure
https://pubchem.ncbi.nlm.nih.gov/compound/143253652#section=2D-Structure
https://pubchem.ncbi.nlm.nih.gov/compound/143249223#section=2D-Structure
https://pubchem.ncbi.nlm.nih.gov/compound/143248677#section=2D-Structure
https://pubchem.ncbi.nlm.nih.gov/compound/143248628#section=2D-Structure
https://pubchem.ncbi.nlm.nih.gov/compound/143248490#section=2D-Structure
https://pubchem.ncbi.nlm.nih.gov/compound/143248482#section=2D-Structure
https://pubchem.ncbi.nlm.nih.gov/compound/143245665#section=2D-Structure
https://pubchem.ncbi.nlm.nih.gov/compound/143232200#section=2D-Structure
https://pubchem.ncbi.nlm.nih.gov/compound/143228397#section=2D-Structure
https://pubchem.ncbi.nlm.nih.gov/compound/143221928#section=2D-Structure
https://pubchem.ncbi.nlm.nih.gov/compound/143221437#section=2D-Structure
https://pubchem.ncbi.nlm.nih.gov/compound/143216568#section=2D-Structure

https://pubchem.ncbi.nlm.nih.gov/compound/143204838#section=2D-Structure
https://pubchem.ncbi.nlm.nih.gov/compound/143203799#section=2D-Structure
https://pubchem.ncbi.nlm.nih.gov/compound/143200619#section=2D-Structure
https://pubchem.ncbi.nlm.nih.gov/compound/143195380#section=2D-Structure
https://pubchem.ncbi.nlm.nih.gov/compound/143169025#section=2D-Structure
https://pubchem.ncbi.nlm.nih.gov/compound/143166318#section=2D-Structure
https://pubchem.ncbi.nlm.nih.gov/compound/143165385#section=2D-Structure
https://pubchem.ncbi.nlm.nih.gov/compound/143157893#section=2D-Structure
https://pubchem.ncbi.nlm.nih.gov/compound/143149701#section=2D-Structure
https://pubchem.ncbi.nlm.nih.gov/compound/143140024#section=2D-Structure
https://pubchem.ncbi.nlm.nih.gov/compound/143135194#section=2D-Structure
https://pubchem.ncbi.nlm.nih.gov/compound/143125864#section=2D-Structure
https://pubchem.ncbi.nlm.nih.gov/compound/143124701#section=2D-Structure
https://pubchem.ncbi.nlm.nih.gov/compound/143123247#section=2D-Structure
https://pubchem.ncbi.nlm.nih.gov/compound/14312#section=2D-Structure
https://pubchem.ncbi.nlm.nih.gov/compound/143116832#section=2D-Structure
https://pubchem.ncbi.nlm.nih.gov/compound/143110008#section=2D-Structure
https://pubchem.ncbi.nlm.nih.gov/compound/143107071#section=2D-Structure
https://pubchem.ncbi.nlm.nih.gov/compound/143104049#section=2D-Structure
https://pubchem.ncbi.nlm.nih.gov/compound/143097058#section=2D-Structure
https://pubchem.ncbi.nlm.nih.gov/compound/143096893#section=2D-Structure
https://pubchem.ncbi.nlm.nih.gov/compound/143096878#section=2D-Structure
https://pubchem.ncbi.nlm.nih.gov/compound/143096639#section=2D-Structure
https://pubchem.ncbi.nlm.nih.gov/compound/143091633#section=2D-Structure
https://pubchem.ncbi.nlm.nih.gov/compound/143091414#section=2D-Structure
https://pubchem.ncbi.nlm.nih.gov/compound/143088900#section=2D-Structure
https://pubchem.ncbi.nlm.nih.gov/compound/143087208#section=2D-Structure
https://pubchem.ncbi.nlm.nih.gov/compound/143069194#section=2D-Structure
https://pubchem.ncbi.nlm.nih.gov/compound/143066322#section=2D-Structure
https://pubchem.ncbi.nlm.nih.gov/compound/143063678#section=2D-Structure
https://pubchem.ncbi.nlm.nih.gov/compound/143057915#section=2D-Structure
https://pubchem.ncbi.nlm.nih.gov/compound/143056223#section=2D-Structure
https://pubchem.ncbi.nlm.nih.gov/compound/143051330#section=2D-Structure
https://pubchem.ncbi.nlm.nih.gov/compound/143050023#section=2D-Structure
https://pubchem.ncbi.nlm.nih.gov/compound/143048751#section=2D-Structure
https://pubchem.ncbi.nlm.nih.gov/compound/143045689#section=2D-Structure
https://pubchem.ncbi.nlm.nih.gov/compound/143041699#section=2D-Structure
https://pubchem.ncbi.nlm.nih.gov/compound/143041691#section=2D-Structure
https://pubchem.ncbi.nlm.nih.gov/compound/143041270#section=2D-Structure
https://pubchem.ncbi.nlm.nih.gov/compound/143038625#section=2D-Structure
https://pubchem.ncbi.nlm.nih.gov/compound/143037106#section=2D-Structure
https://pubchem.ncbi.nlm.nih.gov/compound/143020195#section=2D-Structure
https://pubchem.ncbi.nlm.nih.gov/compound/143013516#section=2D-Structure
https://pubchem.ncbi.nlm.nih.gov/compound/143010186#section=2D-Structure
https://pubchem.ncbi.nlm.nih.gov/compound/142986256#section=2D-Structure
https://pubchem.ncbi.nlm.nih.gov/compound/142972771#section=2D-Structure
https://pubchem.ncbi.nlm.nih.gov/compound/142968947#section=2D-Structure
https://pubchem.ncbi.nlm.nih.gov/compound/142968891#section=2D-Structure
https://pubchem.ncbi.nlm.nih.gov/compound/142967420#section=2D-Structure
https://pubchem.ncbi.nlm.nih.gov/compound/142964290#section=2D-Structure
https://pubchem.ncbi.nlm.nih.gov/compound/142964288#section=2D-Structure
https://pubchem.ncbi.nlm.nih.gov/compound/142961465#section=2D-Structure
https://pubchem.ncbi.nlm.nih.gov/compound/142961357#section=2D-Structure
https://pubchem.ncbi.nlm.nih.gov/compound/142956931#section=2D-Structure
https://pubchem.ncbi.nlm.nih.gov/compound/142950814#section=2D-Structure

https://pubchem.ncbi.nlm.nih.gov/compound/142950780#section=2D-Structure
https://pubchem.ncbi.nlm.nih.gov/compound/142947680#section=2D-Structure
https://pubchem.ncbi.nlm.nih.gov/compound/142944786#section=2D-Structure
https://pubchem.ncbi.nlm.nih.gov/compound/142940467#section=2D-Structure
https://pubchem.ncbi.nlm.nih.gov/compound/142939715#section=2D-Structure
https://pubchem.ncbi.nlm.nih.gov/compound/142934799#section=2D-Structure
https://pubchem.ncbi.nlm.nih.gov/compound/142933227#section=2D-Structure
https://pubchem.ncbi.nlm.nih.gov/compound/142928072#section=2D-Structure
https://pubchem.ncbi.nlm.nih.gov/compound/142925620#section=2D-Structure
https://pubchem.ncbi.nlm.nih.gov/compound/142901266#section=2D-Structure
https://pubchem.ncbi.nlm.nih.gov/compound/142899173#section=2D-Structure
https://pubchem.ncbi.nlm.nih.gov/compound/142893895#section=2D-Structure
https://pubchem.ncbi.nlm.nih.gov/compound/142875350#section=2D-Structure
https://pubchem.ncbi.nlm.nih.gov/compound/142875348#section=2D-Structure
https://pubchem.ncbi.nlm.nih.gov/compound/142869228#section=2D-Structure
https://pubchem.ncbi.nlm.nih.gov/compound/142867887#section=2D-Structure
https://pubchem.ncbi.nlm.nih.gov/compound/14286031#section=2D-Structure
https://pubchem.ncbi.nlm.nih.gov/compound/142857799#section=2D-Structure
https://pubchem.ncbi.nlm.nih.gov/compound/142857717#section=2D-Structure
https://pubchem.ncbi.nlm.nih.gov/compound/142854302#section=2D-Structure
https://pubchem.ncbi.nlm.nih.gov/compound/142853557#section=2D-Structure
https://pubchem.ncbi.nlm.nih.gov/compound/142849592#section=2D-Structure
https://pubchem.ncbi.nlm.nih.gov/compound/142848417#section=2D-Structure
https://pubchem.ncbi.nlm.nih.gov/compound/142848383#section=2D-Structure
https://pubchem.ncbi.nlm.nih.gov/compound/142848346#section=2D-Structure
https://pubchem.ncbi.nlm.nih.gov/compound/142838746#section=2D-Structure
https://pubchem.ncbi.nlm.nih.gov/compound/142838733#section=2D-Structure
https://pubchem.ncbi.nlm.nih.gov/compound/142838449#section=2D-Structure
https://pubchem.ncbi.nlm.nih.gov/compound/142825885#section=2D-Structure
https://pubchem.ncbi.nlm.nih.gov/compound/142819043#section=2D-Structure
https://pubchem.ncbi.nlm.nih.gov/compound/142812505#section=2D-Structure
https://pubchem.ncbi.nlm.nih.gov/compound/142811334#section=2D-Structure
https://pubchem.ncbi.nlm.nih.gov/compound/142810848#section=2D-Structure
https://pubchem.ncbi.nlm.nih.gov/compound/142805390#section=2D-Structure
https://pubchem.ncbi.nlm.nih.gov/compound/142805298#section=2D-Structure
https://pubchem.ncbi.nlm.nih.gov/compound/142800585#section=2D-Structure
https://pubchem.ncbi.nlm.nih.gov/compound/142791266#section=2D-Structure
https://pubchem.ncbi.nlm.nih.gov/compound/142790005#section=2D-Structure
https://pubchem.ncbi.nlm.nih.gov/compound/142784239#section=2D-Structure
https://pubchem.ncbi.nlm.nih.gov/compound/142780186#section=2D-Structure
https://pubchem.ncbi.nlm.nih.gov/compound/142779970#section=2D-Structure
https://pubchem.ncbi.nlm.nih.gov/compound/142779519#section=2D-Structure
https://pubchem.ncbi.nlm.nih.gov/compound/142779516#section=2D-Structure
https://pubchem.ncbi.nlm.nih.gov/compound/142771798#section=2D-Structure
https://pubchem.ncbi.nlm.nih.gov/compound/142769124#section=2D-Structure
https://pubchem.ncbi.nlm.nih.gov/compound/142766722#section=2D-Structure
https://pubchem.ncbi.nlm.nih.gov/compound/142766334#section=2D-Structure
https://pubchem.ncbi.nlm.nih.gov/compound/142765187#section=2D-Structure
https://pubchem.ncbi.nlm.nih.gov/compound/142764706#section=2D-Structure
https://pubchem.ncbi.nlm.nih.gov/compound/142764648#section=2D-Structure
https://pubchem.ncbi.nlm.nih.gov/compound/142762277#section=2D-Structure
https://pubchem.ncbi.nlm.nih.gov/compound/14274778#section=2D-Structure
https://pubchem.ncbi.nlm.nih.gov/compound/142746947#section=2D-Structure
https://pubchem.ncbi.nlm.nih.gov/compound/142743615#section=2D-Structure
https://pubchem.ncbi.nlm.nih.gov/compound/142743396#section=2D-Structure

https://pubchem.ncbi.nlm.nih.gov/compound/142742638#section=2D-Structure
https://pubchem.ncbi.nlm.nih.gov/compound/142737794#section=2D-Structure
https://pubchem.ncbi.nlm.nih.gov/compound/142735891#section=2D-Structure
https://pubchem.ncbi.nlm.nih.gov/compound/142732989#section=2D-Structure
https://pubchem.ncbi.nlm.nih.gov/compound/142728963#section=2D-Structure
https://pubchem.ncbi.nlm.nih.gov/compound/14272060#section=2D-Structure
https://pubchem.ncbi.nlm.nih.gov/compound/142713596#section=2D-Structure
https://pubchem.ncbi.nlm.nih.gov/compound/142707777#section=2D-Structure
https://pubchem.ncbi.nlm.nih.gov/compound/142707776#section=2D-Structure
https://pubchem.ncbi.nlm.nih.gov/compound/142707775#section=2D-Structure
https://pubchem.ncbi.nlm.nih.gov/compound/142707772#section=2D-Structure
https://pubchem.ncbi.nlm.nih.gov/compound/142707769#section=2D-Structure
https://pubchem.ncbi.nlm.nih.gov/compound/142707768#section=2D-Structure
https://pubchem.ncbi.nlm.nih.gov/compound/142707766#section=2D-Structure
https://pubchem.ncbi.nlm.nih.gov/compound/142707763#section=2D-Structure
https://pubchem.ncbi.nlm.nih.gov/compound/142698506#section=2D-Structure
https://pubchem.ncbi.nlm.nih.gov/compound/142696123#section=2D-Structure
https://pubchem.ncbi.nlm.nih.gov/compound/142694976#section=2D-Structure
https://pubchem.ncbi.nlm.nih.gov/compound/142690219#section=2D-Structure
https://pubchem.ncbi.nlm.nih.gov/compound/142685040#section=2D-Structure
https://pubchem.ncbi.nlm.nih.gov/compound/142682392#section=2D-Structure
https://pubchem.ncbi.nlm.nih.gov/compound/142678793#section=2D-Structure
https://pubchem.ncbi.nlm.nih.gov/compound/142676031#section=2D-Structure
https://pubchem.ncbi.nlm.nih.gov/compound/142673684#section=2D-Structure
https://pubchem.ncbi.nlm.nih.gov/compound/142668410#section=2D-Structure
https://pubchem.ncbi.nlm.nih.gov/compound/142667352#section=2D-Structure
https://pubchem.ncbi.nlm.nih.gov/compound/142667351#section=2D-Structure
https://pubchem.ncbi.nlm.nih.gov/compound/142662025#section=2D-Structure
https://pubchem.ncbi.nlm.nih.gov/compound/142654649#section=2D-Structure
https://pubchem.ncbi.nlm.nih.gov/compound/142654645#section=2D-Structure
https://pubchem.ncbi.nlm.nih.gov/compound/142652377#section=2D-Structure
https://pubchem.ncbi.nlm.nih.gov/compound/142649972#section=2D-Structure
https://pubchem.ncbi.nlm.nih.gov/compound/142649025#section=2D-Structure
https://pubchem.ncbi.nlm.nih.gov/compound/142649024#section=2D-Structure
https://pubchem.ncbi.nlm.nih.gov/compound/142645924#section=2D-Structure
https://pubchem.ncbi.nlm.nih.gov/compound/142642814#section=2D-Structure
https://pubchem.ncbi.nlm.nih.gov/compound/142642521#section=2D-Structure
https://pubchem.ncbi.nlm.nih.gov/compound/142634230#section=2D-Structure
https://pubchem.ncbi.nlm.nih.gov/compound/142632006#section=2D-Structure
https://pubchem.ncbi.nlm.nih.gov/compound/142628338#section=2D-Structure
https://pubchem.ncbi.nlm.nih.gov/compound/142626662#section=2D-Structure
https://pubchem.ncbi.nlm.nih.gov/compound/142626007#section=2D-Structure
https://pubchem.ncbi.nlm.nih.gov/compound/14261625#section=2D-Structure
https://pubchem.ncbi.nlm.nih.gov/compound/142608902#section=2D-Structure
https://pubchem.ncbi.nlm.nih.gov/compound/142608901#section=2D-Structure
https://pubchem.ncbi.nlm.nih.gov/compound/142604116#section=2D-Structure
https://pubchem.ncbi.nlm.nih.gov/compound/142599385#section=2D-Structure
https://pubchem.ncbi.nlm.nih.gov/compound/142599356#section=2D-Structure
https://pubchem.ncbi.nlm.nih.gov/compound/142587339#section=2D-Structure
https://pubchem.ncbi.nlm.nih.gov/compound/142582786#section=2D-Structure
https://pubchem.ncbi.nlm.nih.gov/compound/142548539#section=2D-Structure
https://pubchem.ncbi.nlm.nih.gov/compound/142519667#section=2D-Structure
https://pubchem.ncbi.nlm.nih.gov/compound/142519151#section=2D-Structure
https://pubchem.ncbi.nlm.nih.gov/compound/142515536#section=2D-Structure
https://pubchem.ncbi.nlm.nih.gov/compound/142482458#section=2D-Structure

https://pubchem.ncbi.nlm.nih.gov/compound/142449208#section=2D-Structure
https://pubchem.ncbi.nlm.nih.gov/compound/142446541#section=2D-Structure
https://pubchem.ncbi.nlm.nih.gov/compound/142446419#section=2D-Structure
https://pubchem.ncbi.nlm.nih.gov/compound/142430074#section=2D-Structure
https://pubchem.ncbi.nlm.nih.gov/compound/142426550#section=2D-Structure
https://pubchem.ncbi.nlm.nih.gov/compound/142419380#section=2D-Structure
https://pubchem.ncbi.nlm.nih.gov/compound/142416259#section=2D-Structure
https://pubchem.ncbi.nlm.nih.gov/compound/142416238#section=2D-Structure
https://pubchem.ncbi.nlm.nih.gov/compound/142416141#section=2D-Structure
https://pubchem.ncbi.nlm.nih.gov/compound/142415963#section=2D-Structure
https://pubchem.ncbi.nlm.nih.gov/compound/142397871#section=2D-Structure
https://pubchem.ncbi.nlm.nih.gov/compound/142394193#section=2D-Structure
https://pubchem.ncbi.nlm.nih.gov/compound/142393425#section=2D-Structure
https://pubchem.ncbi.nlm.nih.gov/compound/142393422#section=2D-Structure
https://pubchem.ncbi.nlm.nih.gov/compound/142393333#section=2D-Structure
https://pubchem.ncbi.nlm.nih.gov/compound/142393241#section=2D-Structure
https://pubchem.ncbi.nlm.nih.gov/compound/142393234#section=2D-Structure
https://pubchem.ncbi.nlm.nih.gov/compound/142383738#section=2D-Structure
https://pubchem.ncbi.nlm.nih.gov/compound/142353255#section=2D-Structure
https://pubchem.ncbi.nlm.nih.gov/compound/142347634#section=2D-Structure
https://pubchem.ncbi.nlm.nih.gov/compound/142347338#section=2D-Structure
https://pubchem.ncbi.nlm.nih.gov/compound/142342566#section=2D-Structure
https://pubchem.ncbi.nlm.nih.gov/compound/142326#section=2D-Structure
https://pubchem.ncbi.nlm.nih.gov/compound/142324719#section=2D-Structure
https://pubchem.ncbi.nlm.nih.gov/compound/142322490#section=2D-Structure
https://pubchem.ncbi.nlm.nih.gov/compound/142322488#section=2D-Structure
https://pubchem.ncbi.nlm.nih.gov/compound/142319251#section=2D-Structure
https://pubchem.ncbi.nlm.nih.gov/compound/142319#section=2D-Structure
https://pubchem.ncbi.nlm.nih.gov/compound/142318799#section=2D-Structure
https://pubchem.ncbi.nlm.nih.gov/compound/142318768#section=2D-Structure
https://pubchem.ncbi.nlm.nih.gov/compound/142316#section=2D-Structure
https://pubchem.ncbi.nlm.nih.gov/compound/142312564#section=2D-Structure
https://pubchem.ncbi.nlm.nih.gov/compound/142312562#section=2D-Structure
https://pubchem.ncbi.nlm.nih.gov/compound/142298089#section=2D-Structure
https://pubchem.ncbi.nlm.nih.gov/compound/142298061#section=2D-Structure
https://pubchem.ncbi.nlm.nih.gov/compound/142298007#section=2D-Structure
https://pubchem.ncbi.nlm.nih.gov/compound/142297993#section=2D-Structure
https://pubchem.ncbi.nlm.nih.gov/compound/142296747#section=2D-Structure
https://pubchem.ncbi.nlm.nih.gov/compound/142296041#section=2D-Structure
https://pubchem.ncbi.nlm.nih.gov/compound/142292721#section=2D-Structure
https://pubchem.ncbi.nlm.nih.gov/compound/142292539#section=2D-Structure
https://pubchem.ncbi.nlm.nih.gov/compound/142288022#section=2D-Structure
https://pubchem.ncbi.nlm.nih.gov/compound/14228263#section=2D-Structure
https://pubchem.ncbi.nlm.nih.gov/compound/142266851#section=2D-Structure
https://pubchem.ncbi.nlm.nih.gov/compound/142265723#section=2D-Structure
https://pubchem.ncbi.nlm.nih.gov/compound/142257846#section=2D-Structure
https://pubchem.ncbi.nlm.nih.gov/compound/142246259#section=2D-Structure
https://pubchem.ncbi.nlm.nih.gov/compound/142243701#section=2D-Structure
https://pubchem.ncbi.nlm.nih.gov/compound/142240534#section=2D-Structure
https://pubchem.ncbi.nlm.nih.gov/compound/142239005#section=2D-Structure
https://pubchem.ncbi.nlm.nih.gov/compound/142234329#section=2D-Structure
https://pubchem.ncbi.nlm.nih.gov/compound/142222442#section=2D-Structure
https://pubchem.ncbi.nlm.nih.gov/compound/142221369#section=2D-Structure
https://pubchem.ncbi.nlm.nih.gov/compound/142220354#section=2D-Structure
https://pubchem.ncbi.nlm.nih.gov/compound/142205638#section=2D-Structure

https://pubchem.ncbi.nlm.nih.gov/compound/142202585#section=2D-Structure
https://pubchem.ncbi.nlm.nih.gov/compound/142201775#section=2D-Structure
https://pubchem.ncbi.nlm.nih.gov/compound/142201042#section=2D-Structure
https://pubchem.ncbi.nlm.nih.gov/compound/142198819#section=2D-Structure
https://pubchem.ncbi.nlm.nih.gov/compound/142191561#section=2D-Structure
https://pubchem.ncbi.nlm.nih.gov/compound/142184128#section=2D-Structure
https://pubchem.ncbi.nlm.nih.gov/compound/142183343#section=2D-Structure
https://pubchem.ncbi.nlm.nih.gov/compound/142182866#section=2D-Structure
https://pubchem.ncbi.nlm.nih.gov/compound/142181440#section=2D-Structure
https://pubchem.ncbi.nlm.nih.gov/compound/142176611#section=2D-Structure
https://pubchem.ncbi.nlm.nih.gov/compound/142175567#section=2D-Structure
https://pubchem.ncbi.nlm.nih.gov/compound/142165612#section=2D-Structure
https://pubchem.ncbi.nlm.nih.gov/compound/142164524#section=2D-Structure
https://pubchem.ncbi.nlm.nih.gov/compound/142157712#section=2D-Structure
https://pubchem.ncbi.nlm.nih.gov/compound/142155635#section=2D-Structure
https://pubchem.ncbi.nlm.nih.gov/compound/142147867#section=2D-Structure
https://pubchem.ncbi.nlm.nih.gov/compound/142145033#section=2D-Structure
https://pubchem.ncbi.nlm.nih.gov/compound/142143318#section=2D-Structure
https://pubchem.ncbi.nlm.nih.gov/compound/142143207#section=2D-Structure
https://pubchem.ncbi.nlm.nih.gov/compound/142141748#section=2D-Structure
https://pubchem.ncbi.nlm.nih.gov/compound/142140915#section=2D-Structure
https://pubchem.ncbi.nlm.nih.gov/compound/142135403#section=2D-Structure
https://pubchem.ncbi.nlm.nih.gov/compound/142135270#section=2D-Structure
https://pubchem.ncbi.nlm.nih.gov/compound/142133969#section=2D-Structure
https://pubchem.ncbi.nlm.nih.gov/compound/142131498#section=2D-Structure
https://pubchem.ncbi.nlm.nih.gov/compound/142129728#section=2D-Structure
https://pubchem.ncbi.nlm.nih.gov/compound/142129557#section=2D-Structure
https://pubchem.ncbi.nlm.nih.gov/compound/142115543#section=2D-Structure
https://pubchem.ncbi.nlm.nih.gov/compound/142115366#section=2D-Structure
https://pubchem.ncbi.nlm.nih.gov/compound/142088413#section=2D-Structure
https://pubchem.ncbi.nlm.nih.gov/compound/142083820#section=2D-Structure
https://pubchem.ncbi.nlm.nih.gov/compound/142083678#section=2D-Structure
https://pubchem.ncbi.nlm.nih.gov/compound/142083391#section=2D-Structure
https://pubchem.ncbi.nlm.nih.gov/compound/142076545#section=2D-Structure
https://pubchem.ncbi.nlm.nih.gov/compound/142076396#section=2D-Structure
https://pubchem.ncbi.nlm.nih.gov/compound/142064672#section=2D-Structure
https://pubchem.ncbi.nlm.nih.gov/compound/142063077#section=2D-Structure
https://pubchem.ncbi.nlm.nih.gov/compound/142063039#section=2D-Structure
https://pubchem.ncbi.nlm.nih.gov/compound/142056122#section=2D-Structure
https://pubchem.ncbi.nlm.nih.gov/compound/142053614#section=2D-Structure
https://pubchem.ncbi.nlm.nih.gov/compound/142052000#section=2D-Structure
https://pubchem.ncbi.nlm.nih.gov/compound/142042189#section=2D-Structure
https://pubchem.ncbi.nlm.nih.gov/compound/142034394#section=2D-Structure
https://pubchem.ncbi.nlm.nih.gov/compound/142028408#section=2D-Structure
https://pubchem.ncbi.nlm.nih.gov/compound/142023548#section=2D-Structure
https://pubchem.ncbi.nlm.nih.gov/compound/142022013#section=2D-Structure
https://pubchem.ncbi.nlm.nih.gov/compound/142009985#section=2D-Structure
https://pubchem.ncbi.nlm.nih.gov/compound/141996191#section=2D-Structure
https://pubchem.ncbi.nlm.nih.gov/compound/141996183#section=2D-Structure
https://pubchem.ncbi.nlm.nih.gov/compound/141996161#section=2D-Structure
https://pubchem.ncbi.nlm.nih.gov/compound/141992590#section=2D-Structure
https://pubchem.ncbi.nlm.nih.gov/compound/141990400#section=2D-Structure
https://pubchem.ncbi.nlm.nih.gov/compound/141981519#section=2D-Structure
https://pubchem.ncbi.nlm.nih.gov/compound/141980600#section=2D-Structure
https://pubchem.ncbi.nlm.nih.gov/compound/141979337#section=2D-Structure

https://pubchem.ncbi.nlm.nih.gov/compound/141979321#section=2D-Structure
https://pubchem.ncbi.nlm.nih.gov/compound/141974559#section=2D-Structure
https://pubchem.ncbi.nlm.nih.gov/compound/141974424#section=2D-Structure
https://pubchem.ncbi.nlm.nih.gov/compound/141973885#section=2D-Structure
https://pubchem.ncbi.nlm.nih.gov/compound/141973345#section=2D-Structure
https://pubchem.ncbi.nlm.nih.gov/compound/141971960#section=2D-Structure
https://pubchem.ncbi.nlm.nih.gov/compound/141971364#section=2D-Structure
https://pubchem.ncbi.nlm.nih.gov/compound/141970358#section=2D-Structure
https://pubchem.ncbi.nlm.nih.gov/compound/141970305#section=2D-Structure
https://pubchem.ncbi.nlm.nih.gov/compound/141967460#section=2D-Structure
https://pubchem.ncbi.nlm.nih.gov/compound/141966455#section=2D-Structure
https://pubchem.ncbi.nlm.nih.gov/compound/141965853#section=2D-Structure
https://pubchem.ncbi.nlm.nih.gov/compound/141959081#section=2D-Structure
https://pubchem.ncbi.nlm.nih.gov/compound/141958568#section=2D-Structure
https://pubchem.ncbi.nlm.nih.gov/compound/141952884#section=2D-Structure
https://pubchem.ncbi.nlm.nih.gov/compound/141948912#section=2D-Structure
https://pubchem.ncbi.nlm.nih.gov/compound/141946769#section=2D-Structure
https://pubchem.ncbi.nlm.nih.gov/compound/141944779#section=2D-Structure
https://pubchem.ncbi.nlm.nih.gov/compound/141943085#section=2D-Structure
https://pubchem.ncbi.nlm.nih.gov/compound/141942679#section=2D-Structure
https://pubchem.ncbi.nlm.nih.gov/compound/141940942#section=2D-Structure
https://pubchem.ncbi.nlm.nih.gov/compound/141938299#section=2D-Structure
https://pubchem.ncbi.nlm.nih.gov/compound/141937944#section=2D-Structure
https://pubchem.ncbi.nlm.nih.gov/compound/141937214#section=2D-Structure
https://pubchem.ncbi.nlm.nih.gov/compound/14193305#section=2D-Structure
https://pubchem.ncbi.nlm.nih.gov/compound/141929328#section=2D-Structure
https://pubchem.ncbi.nlm.nih.gov/compound/141929323#section=2D-Structure
https://pubchem.ncbi.nlm.nih.gov/compound/141923584#section=2D-Structure
https://pubchem.ncbi.nlm.nih.gov/compound/141919721#section=2D-Structure
https://pubchem.ncbi.nlm.nih.gov/compound/141917274#section=2D-Structure
https://pubchem.ncbi.nlm.nih.gov/compound/141913915#section=2D-Structure
https://pubchem.ncbi.nlm.nih.gov/compound/141913134#section=2D-Structure
https://pubchem.ncbi.nlm.nih.gov/compound/141911597#section=2D-Structure
https://pubchem.ncbi.nlm.nih.gov/compound/141905296#section=2D-Structure
https://pubchem.ncbi.nlm.nih.gov/compound/141905130#section=2D-Structure
https://pubchem.ncbi.nlm.nih.gov/compound/141904842#section=2D-Structure
https://pubchem.ncbi.nlm.nih.gov/compound/141894813#section=2D-Structure
https://pubchem.ncbi.nlm.nih.gov/compound/141893982#section=2D-Structure
https://pubchem.ncbi.nlm.nih.gov/compound/141890348#section=2D-Structure
https://pubchem.ncbi.nlm.nih.gov/compound/141890250#section=2D-Structure
https://pubchem.ncbi.nlm.nih.gov/compound/141890181#section=2D-Structure
https://pubchem.ncbi.nlm.nih.gov/compound/141888298#section=2D-Structure
https://pubchem.ncbi.nlm.nih.gov/compound/141880451#section=2D-Structure
https://pubchem.ncbi.nlm.nih.gov/compound/141879865#section=2D-Structure
https://pubchem.ncbi.nlm.nih.gov/compound/141878781#section=2D-Structure
https://pubchem.ncbi.nlm.nih.gov/compound/141872439#section=2D-Structure
https://pubchem.ncbi.nlm.nih.gov/compound/141868709#section=2D-Structure
https://pubchem.ncbi.nlm.nih.gov/compound/141859918#section=2D-Structure
https://pubchem.ncbi.nlm.nih.gov/compound/141858102#section=2D-Structure
https://pubchem.ncbi.nlm.nih.gov/compound/14185409#section=2D-Structure
https://pubchem.ncbi.nlm.nih.gov/compound/141851696#section=2D-Structure
https://pubchem.ncbi.nlm.nih.gov/compound/141848288#section=2D-Structure
https://pubchem.ncbi.nlm.nih.gov/compound/141846025#section=2D-Structure
https://pubchem.ncbi.nlm.nih.gov/compound/141840114#section=2D-Structure
https://pubchem.ncbi.nlm.nih.gov/compound/141836443#section=2D-Structure

https://pubchem.ncbi.nlm.nih.gov/compound/141829407#section=2D-Structure
https://pubchem.ncbi.nlm.nih.gov/compound/141827548#section=2D-Structure
https://pubchem.ncbi.nlm.nih.gov/compound/141822715#section=2D-Structure
https://pubchem.ncbi.nlm.nih.gov/compound/141821954#section=2D-Structure
https://pubchem.ncbi.nlm.nih.gov/compound/141818676#section=2D-Structure
https://pubchem.ncbi.nlm.nih.gov/compound/141817673#section=2D-Structure
https://pubchem.ncbi.nlm.nih.gov/compound/141816255#section=2D-Structure
https://pubchem.ncbi.nlm.nih.gov/compound/141812070#section=2D-Structure
https://pubchem.ncbi.nlm.nih.gov/compound/141810896#section=2D-Structure
https://pubchem.ncbi.nlm.nih.gov/compound/141807473#section=2D-Structure
https://pubchem.ncbi.nlm.nih.gov/compound/141801451#section=2D-Structure
https://pubchem.ncbi.nlm.nih.gov/compound/141797549#section=2D-Structure
https://pubchem.ncbi.nlm.nih.gov/compound/141797355#section=2D-Structure
https://pubchem.ncbi.nlm.nih.gov/compound/141797166#section=2D-Structure
https://pubchem.ncbi.nlm.nih.gov/compound/141795739#section=2D-Structure
https://pubchem.ncbi.nlm.nih.gov/compound/141795673#section=2D-Structure
https://pubchem.ncbi.nlm.nih.gov/compound/141795422#section=2D-Structure
https://pubchem.ncbi.nlm.nih.gov/compound/141795236#section=2D-Structure
https://pubchem.ncbi.nlm.nih.gov/compound/141794861#section=2D-Structure
https://pubchem.ncbi.nlm.nih.gov/compound/141792205#section=2D-Structure
https://pubchem.ncbi.nlm.nih.gov/compound/141792200#section=2D-Structure
https://pubchem.ncbi.nlm.nih.gov/compound/141790965#section=2D-Structure
https://pubchem.ncbi.nlm.nih.gov/compound/141790738#section=2D-Structure
https://pubchem.ncbi.nlm.nih.gov/compound/141790733#section=2D-Structure
https://pubchem.ncbi.nlm.nih.gov/compound/141787839#section=2D-Structure
https://pubchem.ncbi.nlm.nih.gov/compound/141780248#section=2D-Structure
https://pubchem.ncbi.nlm.nih.gov/compound/141779823#section=2D-Structure
https://pubchem.ncbi.nlm.nih.gov/compound/141778941#section=2D-Structure
https://pubchem.ncbi.nlm.nih.gov/compound/141778495#section=2D-Structure
https://pubchem.ncbi.nlm.nih.gov/compound/141778237#section=2D-Structure
https://pubchem.ncbi.nlm.nih.gov/compound/141776516#section=2D-Structure
https://pubchem.ncbi.nlm.nih.gov/compound/141774831#section=2D-Structure
https://pubchem.ncbi.nlm.nih.gov/compound/141771443#section=2D-Structure
https://pubchem.ncbi.nlm.nih.gov/compound/141770884#section=2D-Structure
https://pubchem.ncbi.nlm.nih.gov/compound/141768114#section=2D-Structure
https://pubchem.ncbi.nlm.nih.gov/compound/141767326#section=2D-Structure
https://pubchem.ncbi.nlm.nih.gov/compound/141767302#section=2D-Structure
https://pubchem.ncbi.nlm.nih.gov/compound/141767268#section=2D-Structure
https://pubchem.ncbi.nlm.nih.gov/compound/141765659#section=2D-Structure
https://pubchem.ncbi.nlm.nih.gov/compound/141764923#section=2D-Structure
https://pubchem.ncbi.nlm.nih.gov/compound/141761167#section=2D-Structure
https://pubchem.ncbi.nlm.nih.gov/compound/141756820#section=2D-Structure
https://pubchem.ncbi.nlm.nih.gov/compound/141755826#section=2D-Structure
https://pubchem.ncbi.nlm.nih.gov/compound/141753654#section=2D-Structure
https://pubchem.ncbi.nlm.nih.gov/compound/141751302#section=2D-Structure
https://pubchem.ncbi.nlm.nih.gov/compound/14174519#section=2D-Structure
https://pubchem.ncbi.nlm.nih.gov/compound/14174518#section=2D-Structure
https://pubchem.ncbi.nlm.nih.gov/compound/14174469#section=2D-Structure
https://pubchem.ncbi.nlm.nih.gov/compound/141736031#section=2D-Structure
https://pubchem.ncbi.nlm.nih.gov/compound/141735#section=2D-Structure
https://pubchem.ncbi.nlm.nih.gov/compound/141734780#section=2D-Structure
https://pubchem.ncbi.nlm.nih.gov/compound/141734779#section=2D-Structure
https://pubchem.ncbi.nlm.nih.gov/compound/141734390#section=2D-Structure
https://pubchem.ncbi.nlm.nih.gov/compound/141734344#section=2D-Structure
https://pubchem.ncbi.nlm.nih.gov/compound/141733307#section=2D-Structure

https://pubchem.ncbi.nlm.nih.gov/compound/141733304#section=2D-Structure
https://pubchem.ncbi.nlm.nih.gov/compound/141732809#section=2D-Structure
https://pubchem.ncbi.nlm.nih.gov/compound/14173154#section=2D-Structure
https://pubchem.ncbi.nlm.nih.gov/compound/141730521#section=2D-Structure
https://pubchem.ncbi.nlm.nih.gov/compound/141729845#section=2D-Structure
https://pubchem.ncbi.nlm.nih.gov/compound/141725817#section=2D-Structure
https://pubchem.ncbi.nlm.nih.gov/compound/141711523#section=2D-Structure
https://pubchem.ncbi.nlm.nih.gov/compound/141707369#section=2D-Structure
https://pubchem.ncbi.nlm.nih.gov/compound/141655609#section=2D-Structure
https://pubchem.ncbi.nlm.nih.gov/compound/141636562#section=2D-Structure
https://pubchem.ncbi.nlm.nih.gov/compound/141636089#section=2D-Structure
https://pubchem.ncbi.nlm.nih.gov/compound/141625536#section=2D-Structure
https://pubchem.ncbi.nlm.nih.gov/compound/141623638#section=2D-Structure
https://pubchem.ncbi.nlm.nih.gov/compound/141617773#section=2D-Structure
https://pubchem.ncbi.nlm.nih.gov/compound/141615005#section=2D-Structure
https://pubchem.ncbi.nlm.nih.gov/compound/141607935#section=2D-Structure
https://pubchem.ncbi.nlm.nih.gov/compound/141605844#section=2D-Structure
https://pubchem.ncbi.nlm.nih.gov/compound/141600938#section=2D-Structure
https://pubchem.ncbi.nlm.nih.gov/compound/141598254#section=2D-Structure
https://pubchem.ncbi.nlm.nih.gov/compound/141598224#section=2D-Structure
https://pubchem.ncbi.nlm.nih.gov/compound/141597214#section=2D-Structure
https://pubchem.ncbi.nlm.nih.gov/compound/141593711#section=2D-Structure
https://pubchem.ncbi.nlm.nih.gov/compound/141592947#section=2D-Structure
https://pubchem.ncbi.nlm.nih.gov/compound/141592014#section=2D-Structure
https://pubchem.ncbi.nlm.nih.gov/compound/141591992#section=2D-Structure
https://pubchem.ncbi.nlm.nih.gov/compound/141583514#section=2D-Structure
https://pubchem.ncbi.nlm.nih.gov/compound/141583183#section=2D-Structure
https://pubchem.ncbi.nlm.nih.gov/compound/141572849#section=2D-Structure
https://pubchem.ncbi.nlm.nih.gov/compound/141569420#section=2D-Structure
https://pubchem.ncbi.nlm.nih.gov/compound/141569356#section=2D-Structure
https://pubchem.ncbi.nlm.nih.gov/compound/141569355#section=2D-Structure
https://pubchem.ncbi.nlm.nih.gov/compound/141564453#section=2D-Structure
https://pubchem.ncbi.nlm.nih.gov/compound/141559197#section=2D-Structure
https://pubchem.ncbi.nlm.nih.gov/compound/141556974#section=2D-Structure
https://pubchem.ncbi.nlm.nih.gov/compound/141555796#section=2D-Structure
https://pubchem.ncbi.nlm.nih.gov/compound/141552023#section=2D-Structure
https://pubchem.ncbi.nlm.nih.gov/compound/141548779#section=2D-Structure
https://pubchem.ncbi.nlm.nih.gov/compound/141547034#section=2D-Structure
https://pubchem.ncbi.nlm.nih.gov/compound/141542590#section=2D-Structure
https://pubchem.ncbi.nlm.nih.gov/compound/141540060#section=2D-Structure
https://pubchem.ncbi.nlm.nih.gov/compound/141538651#section=2D-Structure
https://pubchem.ncbi.nlm.nih.gov/compound/141538033#section=2D-Structure
https://pubchem.ncbi.nlm.nih.gov/compound/141537531#section=2D-Structure
https://pubchem.ncbi.nlm.nih.gov/compound/141537233#section=2D-Structure
https://pubchem.ncbi.nlm.nih.gov/compound/141535806#section=2D-Structure
https://pubchem.ncbi.nlm.nih.gov/compound/141531102#section=2D-Structure
https://pubchem.ncbi.nlm.nih.gov/compound/141531040#section=2D-Structure
https://pubchem.ncbi.nlm.nih.gov/compound/141529484#section=2D-Structure
https://pubchem.ncbi.nlm.nih.gov/compound/141523324#section=2D-Structure
https://pubchem.ncbi.nlm.nih.gov/compound/141519289#section=2D-Structure
https://pubchem.ncbi.nlm.nih.gov/compound/141512726#section=2D-Structure
https://pubchem.ncbi.nlm.nih.gov/compound/141512597#section=2D-Structure
https://pubchem.ncbi.nlm.nih.gov/compound/141512231#section=2D-Structure
https://pubchem.ncbi.nlm.nih.gov/compound/141509505#section=2D-Structure
https://pubchem.ncbi.nlm.nih.gov/compound/141506997#section=2D-Structure

https://pubchem.ncbi.nlm.nih.gov/compound/141504568#section=2D-Structure
https://pubchem.ncbi.nlm.nih.gov/compound/141504373#section=2D-Structure
https://pubchem.ncbi.nlm.nih.gov/compound/141499493#section=2D-Structure
https://pubchem.ncbi.nlm.nih.gov/compound/141496919#section=2D-Structure
https://pubchem.ncbi.nlm.nih.gov/compound/141483917#section=2D-Structure
https://pubchem.ncbi.nlm.nih.gov/compound/141481387#section=2D-Structure
https://pubchem.ncbi.nlm.nih.gov/compound/141481152#section=2D-Structure
https://pubchem.ncbi.nlm.nih.gov/compound/141478201#section=2D-Structure
https://pubchem.ncbi.nlm.nih.gov/compound/141478200#section=2D-Structure
https://pubchem.ncbi.nlm.nih.gov/compound/141468602#section=2D-Structure
https://pubchem.ncbi.nlm.nih.gov/compound/141466487#section=2D-Structure
https://pubchem.ncbi.nlm.nih.gov/compound/141464382#section=2D-Structure
https://pubchem.ncbi.nlm.nih.gov/compound/141463941#section=2D-Structure
https://pubchem.ncbi.nlm.nih.gov/compound/141463587#section=2D-Structure
https://pubchem.ncbi.nlm.nih.gov/compound/141463585#section=2D-Structure
https://pubchem.ncbi.nlm.nih.gov/compound/141459469#section=2D-Structure
https://pubchem.ncbi.nlm.nih.gov/compound/141457553#section=2D-Structure
https://pubchem.ncbi.nlm.nih.gov/compound/141457550#section=2D-Structure
https://pubchem.ncbi.nlm.nih.gov/compound/141453269#section=2D-Structure
https://pubchem.ncbi.nlm.nih.gov/compound/141452310#section=2D-Structure
https://pubchem.ncbi.nlm.nih.gov/compound/141450703#section=2D-Structure
https://pubchem.ncbi.nlm.nih.gov/compound/141450477#section=2D-Structure
https://pubchem.ncbi.nlm.nih.gov/compound/141449721#section=2D-Structure
https://pubchem.ncbi.nlm.nih.gov/compound/141449202#section=2D-Structure
https://pubchem.ncbi.nlm.nih.gov/compound/141447185#section=2D-Structure
https://pubchem.ncbi.nlm.nih.gov/compound/141443065#section=2D-Structure
https://pubchem.ncbi.nlm.nih.gov/compound/141440908#section=2D-Structure
https://pubchem.ncbi.nlm.nih.gov/compound/141440474#section=2D-Structure
https://pubchem.ncbi.nlm.nih.gov/compound/141440003#section=2D-Structure
https://pubchem.ncbi.nlm.nih.gov/compound/141439150#section=2D-Structure
https://pubchem.ncbi.nlm.nih.gov/compound/141438386#section=2D-Structure
https://pubchem.ncbi.nlm.nih.gov/compound/141437697#section=2D-Structure
https://pubchem.ncbi.nlm.nih.gov/compound/141436101#section=2D-Structure
https://pubchem.ncbi.nlm.nih.gov/compound/141431050#section=2D-Structure
https://pubchem.ncbi.nlm.nih.gov/compound/141428430#section=2D-Structure
https://pubchem.ncbi.nlm.nih.gov/compound/141425951#section=2D-Structure
https://pubchem.ncbi.nlm.nih.gov/compound/141417254#section=2D-Structure
https://pubchem.ncbi.nlm.nih.gov/compound/141414595#section=2D-Structure
https://pubchem.ncbi.nlm.nih.gov/compound/141413076#section=2D-Structure
https://pubchem.ncbi.nlm.nih.gov/compound/141412702#section=2D-Structure
https://pubchem.ncbi.nlm.nih.gov/compound/141412108#section=2D-Structure
https://pubchem.ncbi.nlm.nih.gov/compound/141412099#section=2D-Structure
https://pubchem.ncbi.nlm.nih.gov/compound/141411144#section=2D-Structure
https://pubchem.ncbi.nlm.nih.gov/compound/141410950#section=2D-Structure
https://pubchem.ncbi.nlm.nih.gov/compound/141410611#section=2D-Structure
https://pubchem.ncbi.nlm.nih.gov/compound/141408467#section=2D-Structure
https://pubchem.ncbi.nlm.nih.gov/compound/141404144#section=2D-Structure
https://pubchem.ncbi.nlm.nih.gov/compound/141403172#section=2D-Structure
https://pubchem.ncbi.nlm.nih.gov/compound/141401824#section=2D-Structure
https://pubchem.ncbi.nlm.nih.gov/compound/141400223#section=2D-Structure
https://pubchem.ncbi.nlm.nih.gov/compound/141400142#section=2D-Structure
https://pubchem.ncbi.nlm.nih.gov/compound/141399947#section=2D-Structure
https://pubchem.ncbi.nlm.nih.gov/compound/141396163#section=2D-Structure
https://pubchem.ncbi.nlm.nih.gov/compound/141391769#section=2D-Structure
https://pubchem.ncbi.nlm.nih.gov/compound/141391468#section=2D-Structure

https://pubchem.ncbi.nlm.nih.gov/compound/141391393#section=2D-Structure
https://pubchem.ncbi.nlm.nih.gov/compound/141389952#section=2D-Structure
https://pubchem.ncbi.nlm.nih.gov/compound/141389940#section=2D-Structure
https://pubchem.ncbi.nlm.nih.gov/compound/141389153#section=2D-Structure
https://pubchem.ncbi.nlm.nih.gov/compound/141389150#section=2D-Structure
https://pubchem.ncbi.nlm.nih.gov/compound/141389147#section=2D-Structure
https://pubchem.ncbi.nlm.nih.gov/compound/141389145#section=2D-Structure
https://pubchem.ncbi.nlm.nih.gov/compound/141389144#section=2D-Structure
https://pubchem.ncbi.nlm.nih.gov/compound/141389137#section=2D-Structure
https://pubchem.ncbi.nlm.nih.gov/compound/141388838#section=2D-Structure
https://pubchem.ncbi.nlm.nih.gov/compound/141388837#section=2D-Structure
https://pubchem.ncbi.nlm.nih.gov/compound/141387683#section=2D-Structure
https://pubchem.ncbi.nlm.nih.gov/compound/141387535#section=2D-Structure
https://pubchem.ncbi.nlm.nih.gov/compound/141385749#section=2D-Structure
https://pubchem.ncbi.nlm.nih.gov/compound/141385580#section=2D-Structure
https://pubchem.ncbi.nlm.nih.gov/compound/141383991#section=2D-Structure
https://pubchem.ncbi.nlm.nih.gov/compound/141382003#section=2D-Structure
https://pubchem.ncbi.nlm.nih.gov/compound/141381583#section=2D-Structure
https://pubchem.ncbi.nlm.nih.gov/compound/141379818#section=2D-Structure
https://pubchem.ncbi.nlm.nih.gov/compound/141376778#section=2D-Structure
https://pubchem.ncbi.nlm.nih.gov/compound/141373529#section=2D-Structure
https://pubchem.ncbi.nlm.nih.gov/compound/141370223#section=2D-Structure
https://pubchem.ncbi.nlm.nih.gov/compound/141369737#section=2D-Structure
https://pubchem.ncbi.nlm.nih.gov/compound/141369585#section=2D-Structure
https://pubchem.ncbi.nlm.nih.gov/compound/141364444#section=2D-Structure
https://pubchem.ncbi.nlm.nih.gov/compound/141363927#section=2D-Structure
https://pubchem.ncbi.nlm.nih.gov/compound/141361100#section=2D-Structure
https://pubchem.ncbi.nlm.nih.gov/compound/141360428#section=2D-Structure
https://pubchem.ncbi.nlm.nih.gov/compound/141356082#section=2D-Structure
https://pubchem.ncbi.nlm.nih.gov/compound/141354832#section=2D-Structure
https://pubchem.ncbi.nlm.nih.gov/compound/141353398#section=2D-Structure
https://pubchem.ncbi.nlm.nih.gov/compound/141353051#section=2D-Structure
https://pubchem.ncbi.nlm.nih.gov/compound/141353050#section=2D-Structure
https://pubchem.ncbi.nlm.nih.gov/compound/141353048#section=2D-Structure
https://pubchem.ncbi.nlm.nih.gov/compound/141352670#section=2D-Structure
https://pubchem.ncbi.nlm.nih.gov/compound/141351666#section=2D-Structure
https://pubchem.ncbi.nlm.nih.gov/compound/141351167#section=2D-Structure
https://pubchem.ncbi.nlm.nih.gov/compound/141350679#section=2D-Structure
https://pubchem.ncbi.nlm.nih.gov/compound/141349061#section=2D-Structure
https://pubchem.ncbi.nlm.nih.gov/compound/141348871#section=2D-Structure
https://pubchem.ncbi.nlm.nih.gov/compound/141347615#section=2D-Structure
https://pubchem.ncbi.nlm.nih.gov/compound/141345484#section=2D-Structure
https://pubchem.ncbi.nlm.nih.gov/compound/141343582#section=2D-Structure
https://pubchem.ncbi.nlm.nih.gov/compound/141343573#section=2D-Structure
https://pubchem.ncbi.nlm.nih.gov/compound/141343064#section=2D-Structure
https://pubchem.ncbi.nlm.nih.gov/compound/141340000#section=2D-Structure
https://pubchem.ncbi.nlm.nih.gov/compound/141339018#section=2D-Structure
https://pubchem.ncbi.nlm.nih.gov/compound/141338687#section=2D-Structure
https://pubchem.ncbi.nlm.nih.gov/compound/141336413#section=2D-Structure
https://pubchem.ncbi.nlm.nih.gov/compound/141334692#section=2D-Structure
https://pubchem.ncbi.nlm.nih.gov/compound/141334002#section=2D-Structure
https://pubchem.ncbi.nlm.nih.gov/compound/141333783#section=2D-Structure
https://pubchem.ncbi.nlm.nih.gov/compound/141331472#section=2D-Structure
https://pubchem.ncbi.nlm.nih.gov/compound/141331469#section=2D-Structure
https://pubchem.ncbi.nlm.nih.gov/compound/141331407#section=2D-Structure

https://pubchem.ncbi.nlm.nih.gov/compound/141327017#section=2D-Structure
https://pubchem.ncbi.nlm.nih.gov/compound/141326556#section=2D-Structure
https://pubchem.ncbi.nlm.nih.gov/compound/141325252#section=2D-Structure
https://pubchem.ncbi.nlm.nih.gov/compound/141322223#section=2D-Structure
https://pubchem.ncbi.nlm.nih.gov/compound/141321806#section=2D-Structure
https://pubchem.ncbi.nlm.nih.gov/compound/141319712#section=2D-Structure
https://pubchem.ncbi.nlm.nih.gov/compound/141319375#section=2D-Structure
https://pubchem.ncbi.nlm.nih.gov/compound/141318347#section=2D-Structure
https://pubchem.ncbi.nlm.nih.gov/compound/141318164#section=2D-Structure
https://pubchem.ncbi.nlm.nih.gov/compound/141318042#section=2D-Structure
https://pubchem.ncbi.nlm.nih.gov/compound/141314591#section=2D-Structure
https://pubchem.ncbi.nlm.nih.gov/compound/141312684#section=2D-Structure
https://pubchem.ncbi.nlm.nih.gov/compound/141311137#section=2D-Structure
https://pubchem.ncbi.nlm.nih.gov/compound/141310836#section=2D-Structure
https://pubchem.ncbi.nlm.nih.gov/compound/141310141#section=2D-Structure
https://pubchem.ncbi.nlm.nih.gov/compound/141309997#section=2D-Structure
https://pubchem.ncbi.nlm.nih.gov/compound/141301152#section=2D-Structure
https://pubchem.ncbi.nlm.nih.gov/compound/141298154#section=2D-Structure
https://pubchem.ncbi.nlm.nih.gov/compound/141297584#section=2D-Structure
https://pubchem.ncbi.nlm.nih.gov/compound/141295004#section=2D-Structure
https://pubchem.ncbi.nlm.nih.gov/compound/141293433#section=2D-Structure
https://pubchem.ncbi.nlm.nih.gov/compound/141293425#section=2D-Structure
https://pubchem.ncbi.nlm.nih.gov/compound/141290808#section=2D-Structure
https://pubchem.ncbi.nlm.nih.gov/compound/141289941#section=2D-Structure
https://pubchem.ncbi.nlm.nih.gov/compound/141289920#section=2D-Structure
https://pubchem.ncbi.nlm.nih.gov/compound/141288031#section=2D-Structure
https://pubchem.ncbi.nlm.nih.gov/compound/141285230#section=2D-Structure
https://pubchem.ncbi.nlm.nih.gov/compound/141284225#section=2D-Structure
https://pubchem.ncbi.nlm.nih.gov/compound/141283889#section=2D-Structure
https://pubchem.ncbi.nlm.nih.gov/compound/141283486#section=2D-Structure
https://pubchem.ncbi.nlm.nih.gov/compound/141278147#section=2D-Structure
https://pubchem.ncbi.nlm.nih.gov/compound/141277329#section=2D-Structure
https://pubchem.ncbi.nlm.nih.gov/compound/141276715#section=2D-Structure
https://pubchem.ncbi.nlm.nih.gov/compound/141275333#section=2D-Structure
https://pubchem.ncbi.nlm.nih.gov/compound/141274902#section=2D-Structure
https://pubchem.ncbi.nlm.nih.gov/compound/141274645#section=2D-Structure
https://pubchem.ncbi.nlm.nih.gov/compound/141274632#section=2D-Structure
https://pubchem.ncbi.nlm.nih.gov/compound/141274070#section=2D-Structure
https://pubchem.ncbi.nlm.nih.gov/compound/141270987#section=2D-Structure
https://pubchem.ncbi.nlm.nih.gov/compound/141270378#section=2D-Structure
https://pubchem.ncbi.nlm.nih.gov/compound/141269734#section=2D-Structure
https://pubchem.ncbi.nlm.nih.gov/compound/141267681#section=2D-Structure
https://pubchem.ncbi.nlm.nih.gov/compound/141267680#section=2D-Structure
https://pubchem.ncbi.nlm.nih.gov/compound/141267679#section=2D-Structure
https://pubchem.ncbi.nlm.nih.gov/compound/141267678#section=2D-Structure
https://pubchem.ncbi.nlm.nih.gov/compound/141248591#section=2D-Structure
https://pubchem.ncbi.nlm.nih.gov/compound/141248287#section=2D-Structure
https://pubchem.ncbi.nlm.nih.gov/compound/141247339#section=2D-Structure
https://pubchem.ncbi.nlm.nih.gov/compound/141244513#section=2D-Structure
https://pubchem.ncbi.nlm.nih.gov/compound/141243405#section=2D-Structure
https://pubchem.ncbi.nlm.nih.gov/compound/141242895#section=2D-Structure
https://pubchem.ncbi.nlm.nih.gov/compound/141239130#section=2D-Structure
https://pubchem.ncbi.nlm.nih.gov/compound/141232604#section=2D-Structure
https://pubchem.ncbi.nlm.nih.gov/compound/141228988#section=2D-Structure
https://pubchem.ncbi.nlm.nih.gov/compound/141226154#section=2D-Structure

https://pubchem.ncbi.nlm.nih.gov/compound/141223278#section=2D-Structure
https://pubchem.ncbi.nlm.nih.gov/compound/141218176#section=2D-Structure
https://pubchem.ncbi.nlm.nih.gov/compound/141210760#section=2D-Structure
https://pubchem.ncbi.nlm.nih.gov/compound/141208359#section=2D-Structure
https://pubchem.ncbi.nlm.nih.gov/compound/141205609#section=2D-Structure
https://pubchem.ncbi.nlm.nih.gov/compound/141205343#section=2D-Structure
https://pubchem.ncbi.nlm.nih.gov/compound/141205313#section=2D-Structure
https://pubchem.ncbi.nlm.nih.gov/compound/141205013#section=2D-Structure
https://pubchem.ncbi.nlm.nih.gov/compound/141204226#section=2D-Structure
https://pubchem.ncbi.nlm.nih.gov/compound/141204053#section=2D-Structure
https://pubchem.ncbi.nlm.nih.gov/compound/141202618#section=2D-Structure
https://pubchem.ncbi.nlm.nih.gov/compound/141199065#section=2D-Structure
https://pubchem.ncbi.nlm.nih.gov/compound/141197860#section=2D-Structure
https://pubchem.ncbi.nlm.nih.gov/compound/141197856#section=2D-Structure
https://pubchem.ncbi.nlm.nih.gov/compound/141197375#section=2D-Structure
https://pubchem.ncbi.nlm.nih.gov/compound/141196144#section=2D-Structure
https://pubchem.ncbi.nlm.nih.gov/compound/141196134#section=2D-Structure
https://pubchem.ncbi.nlm.nih.gov/compound/141195888#section=2D-Structure
https://pubchem.ncbi.nlm.nih.gov/compound/141194957#section=2D-Structure
https://pubchem.ncbi.nlm.nih.gov/compound/141194492#section=2D-Structure
https://pubchem.ncbi.nlm.nih.gov/compound/141187837#section=2D-Structure
https://pubchem.ncbi.nlm.nih.gov/compound/141186708#section=2D-Structure
https://pubchem.ncbi.nlm.nih.gov/compound/141186365#section=2D-Structure
https://pubchem.ncbi.nlm.nih.gov/compound/141185747#section=2D-Structure
https://pubchem.ncbi.nlm.nih.gov/compound/141183355#section=2D-Structure
https://pubchem.ncbi.nlm.nih.gov/compound/141181368#section=2D-Structure
https://pubchem.ncbi.nlm.nih.gov/compound/14118031#section=2D-Structure
https://pubchem.ncbi.nlm.nih.gov/compound/141174348#section=2D-Structure
https://pubchem.ncbi.nlm.nih.gov/compound/141170179#section=2D-Structure
https://pubchem.ncbi.nlm.nih.gov/compound/141169173#section=2D-Structure
https://pubchem.ncbi.nlm.nih.gov/compound/141169156#section=2D-Structure
https://pubchem.ncbi.nlm.nih.gov/compound/141164086#section=2D-Structure
https://pubchem.ncbi.nlm.nih.gov/compound/141163686#section=2D-Structure
https://pubchem.ncbi.nlm.nih.gov/compound/141163669#section=2D-Structure
https://pubchem.ncbi.nlm.nih.gov/compound/141163663#section=2D-Structure
https://pubchem.ncbi.nlm.nih.gov/compound/141163635#section=2D-Structure
https://pubchem.ncbi.nlm.nih.gov/compound/141163386#section=2D-Structure
https://pubchem.ncbi.nlm.nih.gov/compound/141163032#section=2D-Structure
https://pubchem.ncbi.nlm.nih.gov/compound/141162710#section=2D-Structure
https://pubchem.ncbi.nlm.nih.gov/compound/141161016#section=2D-Structure
https://pubchem.ncbi.nlm.nih.gov/compound/141160806#section=2D-Structure
https://pubchem.ncbi.nlm.nih.gov/compound/141160715#section=2D-Structure
https://pubchem.ncbi.nlm.nih.gov/compound/141160713#section=2D-Structure
https://pubchem.ncbi.nlm.nih.gov/compound/141160293#section=2D-Structure
https://pubchem.ncbi.nlm.nih.gov/compound/141158526#section=2D-Structure
https://pubchem.ncbi.nlm.nih.gov/compound/141156383#section=2D-Structure
https://pubchem.ncbi.nlm.nih.gov/compound/141156#section=2D-Structure
https://pubchem.ncbi.nlm.nih.gov/compound/141153153#section=2D-Structure
https://pubchem.ncbi.nlm.nih.gov/compound/141151046#section=2D-Structure
https://pubchem.ncbi.nlm.nih.gov/compound/141148642#section=2D-Structure
https://pubchem.ncbi.nlm.nih.gov/compound/141147806#section=2D-Structure
https://pubchem.ncbi.nlm.nih.gov/compound/141147804#section=2D-Structure
https://pubchem.ncbi.nlm.nih.gov/compound/141146356#section=2D-Structure
https://pubchem.ncbi.nlm.nih.gov/compound/141145177#section=2D-Structure
https://pubchem.ncbi.nlm.nih.gov/compound/141144364#section=2D-Structure

https://pubchem.ncbi.nlm.nih.gov/compound/141143575#section=2D-Structure
https://pubchem.ncbi.nlm.nih.gov/compound/141143466#section=2D-Structure
https://pubchem.ncbi.nlm.nih.gov/compound/141142814#section=2D-Structure
https://pubchem.ncbi.nlm.nih.gov/compound/141136605#section=2D-Structure
https://pubchem.ncbi.nlm.nih.gov/compound/141136195#section=2D-Structure
https://pubchem.ncbi.nlm.nih.gov/compound/141135888#section=2D-Structure
https://pubchem.ncbi.nlm.nih.gov/compound/141134900#section=2D-Structure
https://pubchem.ncbi.nlm.nih.gov/compound/141134899#section=2D-Structure
https://pubchem.ncbi.nlm.nih.gov/compound/141132431#section=2D-Structure
https://pubchem.ncbi.nlm.nih.gov/compound/141130716#section=2D-Structure
https://pubchem.ncbi.nlm.nih.gov/compound/141130608#section=2D-Structure
https://pubchem.ncbi.nlm.nih.gov/compound/141130606#section=2D-Structure
https://pubchem.ncbi.nlm.nih.gov/compound/141129019#section=2D-Structure
https://pubchem.ncbi.nlm.nih.gov/compound/141128997#section=2D-Structure
https://pubchem.ncbi.nlm.nih.gov/compound/141125509#section=2D-Structure
https://pubchem.ncbi.nlm.nih.gov/compound/141125508#section=2D-Structure
https://pubchem.ncbi.nlm.nih.gov/compound/141124392#section=2D-Structure
https://pubchem.ncbi.nlm.nih.gov/compound/141124281#section=2D-Structure
https://pubchem.ncbi.nlm.nih.gov/compound/141122476#section=2D-Structure
https://pubchem.ncbi.nlm.nih.gov/compound/141121590#section=2D-Structure
https://pubchem.ncbi.nlm.nih.gov/compound/141120378#section=2D-Structure
https://pubchem.ncbi.nlm.nih.gov/compound/141116279#section=2D-Structure
https://pubchem.ncbi.nlm.nih.gov/compound/141115958#section=2D-Structure
https://pubchem.ncbi.nlm.nih.gov/compound/141115727#section=2D-Structure
https://pubchem.ncbi.nlm.nih.gov/compound/141114654#section=2D-Structure
https://pubchem.ncbi.nlm.nih.gov/compound/141114367#section=2D-Structure
https://pubchem.ncbi.nlm.nih.gov/compound/141113245#section=2D-Structure
https://pubchem.ncbi.nlm.nih.gov/compound/141113044#section=2D-Structure
https://pubchem.ncbi.nlm.nih.gov/compound/141111595#section=2D-Structure
https://pubchem.ncbi.nlm.nih.gov/compound/141111593#section=2D-Structure
https://pubchem.ncbi.nlm.nih.gov/compound/141111582#section=2D-Structure
https://pubchem.ncbi.nlm.nih.gov/compound/141111580#section=2D-Structure
https://pubchem.ncbi.nlm.nih.gov/compound/141110#section=2D-Structure
https://pubchem.ncbi.nlm.nih.gov/compound/141105839#section=2D-Structure
https://pubchem.ncbi.nlm.nih.gov/compound/141104281#section=2D-Structure
https://pubchem.ncbi.nlm.nih.gov/compound/141104278#section=2D-Structure
https://pubchem.ncbi.nlm.nih.gov/compound/141104082#section=2D-Structure
https://pubchem.ncbi.nlm.nih.gov/compound/141103659#section=2D-Structure
https://pubchem.ncbi.nlm.nih.gov/compound/141103223#section=2D-Structure
https://pubchem.ncbi.nlm.nih.gov/compound/141101458#section=2D-Structure
https://pubchem.ncbi.nlm.nih.gov/compound/141100546#section=2D-Structure
https://pubchem.ncbi.nlm.nih.gov/compound/141091028#section=2D-Structure
https://pubchem.ncbi.nlm.nih.gov/compound/141090949#section=2D-Structure
https://pubchem.ncbi.nlm.nih.gov/compound/141090625#section=2D-Structure
https://pubchem.ncbi.nlm.nih.gov/compound/141088942#section=2D-Structure
https://pubchem.ncbi.nlm.nih.gov/compound/141088006#section=2D-Structure
https://pubchem.ncbi.nlm.nih.gov/compound/141084731#section=2D-Structure
https://pubchem.ncbi.nlm.nih.gov/compound/141081034#section=2D-Structure
https://pubchem.ncbi.nlm.nih.gov/compound/141080973#section=2D-Structure
https://pubchem.ncbi.nlm.nih.gov/compound/141078890#section=2D-Structure
https://pubchem.ncbi.nlm.nih.gov/compound/141078842#section=2D-Structure
https://pubchem.ncbi.nlm.nih.gov/compound/141076919#section=2D-Structure
https://pubchem.ncbi.nlm.nih.gov/compound/141069635#section=2D-Structure
https://pubchem.ncbi.nlm.nih.gov/compound/141069427#section=2D-Structure
https://pubchem.ncbi.nlm.nih.gov/compound/141069235#section=2D-Structure

https://pubchem.ncbi.nlm.nih.gov/compound/141068953#section=2D-Structure
https://pubchem.ncbi.nlm.nih.gov/compound/141068488#section=2D-Structure
https://pubchem.ncbi.nlm.nih.gov/compound/141066869#section=2D-Structure
https://pubchem.ncbi.nlm.nih.gov/compound/141066597#section=2D-Structure
https://pubchem.ncbi.nlm.nih.gov/compound/141064512#section=2D-Structure
https://pubchem.ncbi.nlm.nih.gov/compound/141064211#section=2D-Structure
https://pubchem.ncbi.nlm.nih.gov/compound/141062601#section=2D-Structure
https://pubchem.ncbi.nlm.nih.gov/compound/141062108#section=2D-Structure
https://pubchem.ncbi.nlm.nih.gov/compound/141062100#section=2D-Structure
https://pubchem.ncbi.nlm.nih.gov/compound/141061830#section=2D-Structure
https://pubchem.ncbi.nlm.nih.gov/compound/141060298#section=2D-Structure
https://pubchem.ncbi.nlm.nih.gov/compound/141060174#section=2D-Structure
https://pubchem.ncbi.nlm.nih.gov/compound/141059026#section=2D-Structure
https://pubchem.ncbi.nlm.nih.gov/compound/141057618#section=2D-Structure
https://pubchem.ncbi.nlm.nih.gov/compound/141055470#section=2D-Structure
https://pubchem.ncbi.nlm.nih.gov/compound/141055451#section=2D-Structure
https://pubchem.ncbi.nlm.nih.gov/compound/141055450#section=2D-Structure
https://pubchem.ncbi.nlm.nih.gov/compound/141055445#section=2D-Structure
https://pubchem.ncbi.nlm.nih.gov/compound/141055442#section=2D-Structure
https://pubchem.ncbi.nlm.nih.gov/compound/141055021#section=2D-Structure
https://pubchem.ncbi.nlm.nih.gov/compound/141051728#section=2D-Structure
https://pubchem.ncbi.nlm.nih.gov/compound/141050593#section=2D-Structure
https://pubchem.ncbi.nlm.nih.gov/compound/141050012#section=2D-Structure
https://pubchem.ncbi.nlm.nih.gov/compound/141050010#section=2D-Structure
https://pubchem.ncbi.nlm.nih.gov/compound/141047326#section=2D-Structure
https://pubchem.ncbi.nlm.nih.gov/compound/141046774#section=2D-Structure
https://pubchem.ncbi.nlm.nih.gov/compound/141045009#section=2D-Structure
https://pubchem.ncbi.nlm.nih.gov/compound/141041228#section=2D-Structure
https://pubchem.ncbi.nlm.nih.gov/compound/141041129#section=2D-Structure
https://pubchem.ncbi.nlm.nih.gov/compound/141041127#section=2D-Structure
https://pubchem.ncbi.nlm.nih.gov/compound/141041107#section=2D-Structure
https://pubchem.ncbi.nlm.nih.gov/compound/141039423#section=2D-Structure
https://pubchem.ncbi.nlm.nih.gov/compound/141039174#section=2D-Structure
https://pubchem.ncbi.nlm.nih.gov/compound/141039098#section=2D-Structure
https://pubchem.ncbi.nlm.nih.gov/compound/141036851#section=2D-Structure
https://pubchem.ncbi.nlm.nih.gov/compound/141036506#section=2D-Structure
https://pubchem.ncbi.nlm.nih.gov/compound/141036499#section=2D-Structure
https://pubchem.ncbi.nlm.nih.gov/compound/141032647#section=2D-Structure
https://pubchem.ncbi.nlm.nih.gov/compound/141030631#section=2D-Structure
https://pubchem.ncbi.nlm.nih.gov/compound/141030607#section=2D-Structure
https://pubchem.ncbi.nlm.nih.gov/compound/141028860#section=2D-Structure
https://pubchem.ncbi.nlm.nih.gov/compound/141028840#section=2D-Structure
https://pubchem.ncbi.nlm.nih.gov/compound/141025562#section=2D-Structure
https://pubchem.ncbi.nlm.nih.gov/compound/141024625#section=2D-Structure
https://pubchem.ncbi.nlm.nih.gov/compound/141023803#section=2D-Structure
https://pubchem.ncbi.nlm.nih.gov/compound/141023801#section=2D-Structure
https://pubchem.ncbi.nlm.nih.gov/compound/141023693#section=2D-Structure
https://pubchem.ncbi.nlm.nih.gov/compound/141022642#section=2D-Structure
https://pubchem.ncbi.nlm.nih.gov/compound/141022635#section=2D-Structure
https://pubchem.ncbi.nlm.nih.gov/compound/141022117#section=2D-Structure
https://pubchem.ncbi.nlm.nih.gov/compound/141021478#section=2D-Structure
https://pubchem.ncbi.nlm.nih.gov/compound/141021182#section=2D-Structure
https://pubchem.ncbi.nlm.nih.gov/compound/141019432#section=2D-Structure
https://pubchem.ncbi.nlm.nih.gov/compound/141015300#section=2D-Structure
https://pubchem.ncbi.nlm.nih.gov/compound/141013481#section=2D-Structure

https://pubchem.ncbi.nlm.nih.gov/compound/141012797#section=2D-Structure
https://pubchem.ncbi.nlm.nih.gov/compound/141010810#section=2D-Structure
https://pubchem.ncbi.nlm.nih.gov/compound/141010808#section=2D-Structure
https://pubchem.ncbi.nlm.nih.gov/compound/141010742#section=2D-Structure
https://pubchem.ncbi.nlm.nih.gov/compound/141008502#section=2D-Structure
https://pubchem.ncbi.nlm.nih.gov/compound/141006793#section=2D-Structure
https://pubchem.ncbi.nlm.nih.gov/compound/141006568#section=2D-Structure
https://pubchem.ncbi.nlm.nih.gov/compound/141004407#section=2D-Structure
https://pubchem.ncbi.nlm.nih.gov/compound/141002818#section=2D-Structure
https://pubchem.ncbi.nlm.nih.gov/compound/141002363#section=2D-Structure
https://pubchem.ncbi.nlm.nih.gov/compound/141001396#section=2D-Structure
https://pubchem.ncbi.nlm.nih.gov/compound/141001027#section=2D-Structure
https://pubchem.ncbi.nlm.nih.gov/compound/141000319#section=2D-Structure
https://pubchem.ncbi.nlm.nih.gov/compound/141000195#section=2D-Structure
https://pubchem.ncbi.nlm.nih.gov/compound/140999526#section=2D-Structure
https://pubchem.ncbi.nlm.nih.gov/compound/140996734#section=2D-Structure
https://pubchem.ncbi.nlm.nih.gov/compound/140994845#section=2D-Structure
https://pubchem.ncbi.nlm.nih.gov/compound/140994842#section=2D-Structure
https://pubchem.ncbi.nlm.nih.gov/compound/140994841#section=2D-Structure
https://pubchem.ncbi.nlm.nih.gov/compound/140994732#section=2D-Structure
https://pubchem.ncbi.nlm.nih.gov/compound/140994561#section=2D-Structure
https://pubchem.ncbi.nlm.nih.gov/compound/140994203#section=2D-Structure
https://pubchem.ncbi.nlm.nih.gov/compound/140994195#section=2D-Structure
https://pubchem.ncbi.nlm.nih.gov/compound/140993835#section=2D-Structure
https://pubchem.ncbi.nlm.nih.gov/compound/140991499#section=2D-Structure
https://pubchem.ncbi.nlm.nih.gov/compound/140988316#section=2D-Structure
https://pubchem.ncbi.nlm.nih.gov/compound/140988311#section=2D-Structure
https://pubchem.ncbi.nlm.nih.gov/compound/140987570#section=2D-Structure
https://pubchem.ncbi.nlm.nih.gov/compound/140986736#section=2D-Structure
https://pubchem.ncbi.nlm.nih.gov/compound/140985757#section=2D-Structure
https://pubchem.ncbi.nlm.nih.gov/compound/140982977#section=2D-Structure
https://pubchem.ncbi.nlm.nih.gov/compound/140982956#section=2D-Structure
https://pubchem.ncbi.nlm.nih.gov/compound/140982955#section=2D-Structure
https://pubchem.ncbi.nlm.nih.gov/compound/140982562#section=2D-Structure
https://pubchem.ncbi.nlm.nih.gov/compound/140981914#section=2D-Structure
https://pubchem.ncbi.nlm.nih.gov/compound/140981913#section=2D-Structure
https://pubchem.ncbi.nlm.nih.gov/compound/140981910#section=2D-Structure
https://pubchem.ncbi.nlm.nih.gov/compound/140979053#section=2D-Structure
https://pubchem.ncbi.nlm.nih.gov/compound/140977841#section=2D-Structure
https://pubchem.ncbi.nlm.nih.gov/compound/140977085#section=2D-Structure
https://pubchem.ncbi.nlm.nih.gov/compound/140977060#section=2D-Structure
https://pubchem.ncbi.nlm.nih.gov/compound/140976851#section=2D-Structure
https://pubchem.ncbi.nlm.nih.gov/compound/140976849#section=2D-Structure
https://pubchem.ncbi.nlm.nih.gov/compound/140975885#section=2D-Structure
https://pubchem.ncbi.nlm.nih.gov/compound/140974903#section=2D-Structure
https://pubchem.ncbi.nlm.nih.gov/compound/140974594#section=2D-Structure
https://pubchem.ncbi.nlm.nih.gov/compound/140974525#section=2D-Structure
https://pubchem.ncbi.nlm.nih.gov/compound/140974520#section=2D-Structure
https://pubchem.ncbi.nlm.nih.gov/compound/140973266#section=2D-Structure
https://pubchem.ncbi.nlm.nih.gov/compound/140973262#section=2D-Structure
https://pubchem.ncbi.nlm.nih.gov/compound/140972994#section=2D-Structure
https://pubchem.ncbi.nlm.nih.gov/compound/140971719#section=2D-Structure
https://pubchem.ncbi.nlm.nih.gov/compound/140970945#section=2D-Structure
https://pubchem.ncbi.nlm.nih.gov/compound/140969854#section=2D-Structure
https://pubchem.ncbi.nlm.nih.gov/compound/140968629#section=2D-Structure

https://pubchem.ncbi.nlm.nih.gov/compound/140968436#section=2D-Structure
https://pubchem.ncbi.nlm.nih.gov/compound/140968142#section=2D-Structure
https://pubchem.ncbi.nlm.nih.gov/compound/140968140#section=2D-Structure
https://pubchem.ncbi.nlm.nih.gov/compound/140956219#section=2D-Structure
https://pubchem.ncbi.nlm.nih.gov/compound/140956203#section=2D-Structure
https://pubchem.ncbi.nlm.nih.gov/compound/140956194#section=2D-Structure
https://pubchem.ncbi.nlm.nih.gov/compound/140956189#section=2D-Structure
https://pubchem.ncbi.nlm.nih.gov/compound/140956169#section=2D-Structure
https://pubchem.ncbi.nlm.nih.gov/compound/140956144#section=2D-Structure
https://pubchem.ncbi.nlm.nih.gov/compound/140956143#section=2D-Structure
https://pubchem.ncbi.nlm.nih.gov/compound/140956133#section=2D-Structure
https://pubchem.ncbi.nlm.nih.gov/compound/140956118#section=2D-Structure
https://pubchem.ncbi.nlm.nih.gov/compound/140956093#section=2D-Structure
https://pubchem.ncbi.nlm.nih.gov/compound/140956072#section=2D-Structure
https://pubchem.ncbi.nlm.nih.gov/compound/140956057#section=2D-Structure
https://pubchem.ncbi.nlm.nih.gov/compound/140956054#section=2D-Structure
https://pubchem.ncbi.nlm.nih.gov/compound/140956009#section=2D-Structure
https://pubchem.ncbi.nlm.nih.gov/compound/14092593#section=2D-Structure
https://pubchem.ncbi.nlm.nih.gov/compound/140922688#section=2D-Structure
https://pubchem.ncbi.nlm.nih.gov/compound/140920717#section=2D-Structure
https://pubchem.ncbi.nlm.nih.gov/compound/140896070#section=2D-Structure
https://pubchem.ncbi.nlm.nih.gov/compound/14088957#section=2D-Structure
https://pubchem.ncbi.nlm.nih.gov/compound/140885618#section=2D-Structure
https://pubchem.ncbi.nlm.nih.gov/compound/140885605#section=2D-Structure
https://pubchem.ncbi.nlm.nih.gov/compound/140885601#section=2D-Structure
https://pubchem.ncbi.nlm.nih.gov/compound/140885600#section=2D-Structure
https://pubchem.ncbi.nlm.nih.gov/compound/140885595#section=2D-Structure
https://pubchem.ncbi.nlm.nih.gov/compound/140885591#section=2D-Structure
https://pubchem.ncbi.nlm.nih.gov/compound/140885590#section=2D-Structure
https://pubchem.ncbi.nlm.nih.gov/compound/140885587#section=2D-Structure
https://pubchem.ncbi.nlm.nih.gov/compound/140885559#section=2D-Structure
https://pubchem.ncbi.nlm.nih.gov/compound/140885555#section=2D-Structure
https://pubchem.ncbi.nlm.nih.gov/compound/140885551#section=2D-Structure
https://pubchem.ncbi.nlm.nih.gov/compound/140885546#section=2D-Structure
https://pubchem.ncbi.nlm.nih.gov/compound/140885531#section=2D-Structure
https://pubchem.ncbi.nlm.nih.gov/compound/140873623#section=2D-Structure
https://pubchem.ncbi.nlm.nih.gov/compound/140868970#section=2D-Structure
https://pubchem.ncbi.nlm.nih.gov/compound/140866789#section=2D-Structure
https://pubchem.ncbi.nlm.nih.gov/compound/140843898#section=2D-Structure
https://pubchem.ncbi.nlm.nih.gov/compound/140824#section=2D-Structure
https://pubchem.ncbi.nlm.nih.gov/compound/140818267#section=2D-Structure
https://pubchem.ncbi.nlm.nih.gov/compound/140814685#section=2D-Structure
https://pubchem.ncbi.nlm.nih.gov/compound/140801627#section=2D-Structure
https://pubchem.ncbi.nlm.nih.gov/compound/140790208#section=2D-Structure
https://pubchem.ncbi.nlm.nih.gov/compound/140764532#section=2D-Structure
https://pubchem.ncbi.nlm.nih.gov/compound/140761376#section=2D-Structure
https://pubchem.ncbi.nlm.nih.gov/compound/140759994#section=2D-Structure
https://pubchem.ncbi.nlm.nih.gov/compound/140750341#section=2D-Structure
https://pubchem.ncbi.nlm.nih.gov/compound/140699432#section=2D-Structure
https://pubchem.ncbi.nlm.nih.gov/compound/140696339#section=2D-Structure
https://pubchem.ncbi.nlm.nih.gov/compound/140678466#section=2D-Structure
https://pubchem.ncbi.nlm.nih.gov/compound/140675505#section=2D-Structure
https://pubchem.ncbi.nlm.nih.gov/compound/140674054#section=2D-Structure
https://pubchem.ncbi.nlm.nih.gov/compound/140674053#section=2D-Structure
https://pubchem.ncbi.nlm.nih.gov/compound/140665977#section=2D-Structure

https://pubchem.ncbi.nlm.nih.gov/compound/140652609#section=2D-Structure
https://pubchem.ncbi.nlm.nih.gov/compound/140648300#section=2D-Structure
https://pubchem.ncbi.nlm.nih.gov/compound/140646046#section=2D-Structure
https://pubchem.ncbi.nlm.nih.gov/compound/140643697#section=2D-Structure
https://pubchem.ncbi.nlm.nih.gov/compound/14064339#section=2D-Structure
https://pubchem.ncbi.nlm.nih.gov/compound/140643194#section=2D-Structure
https://pubchem.ncbi.nlm.nih.gov/compound/14063921#section=2D-Structure
https://pubchem.ncbi.nlm.nih.gov/compound/14062921#section=2D-Structure
https://pubchem.ncbi.nlm.nih.gov/compound/140622964#section=2D-Structure
https://pubchem.ncbi.nlm.nih.gov/compound/140617724#section=2D-Structure
https://pubchem.ncbi.nlm.nih.gov/compound/140609872#section=2D-Structure
https://pubchem.ncbi.nlm.nih.gov/compound/140609086#section=2D-Structure
https://pubchem.ncbi.nlm.nih.gov/compound/140608255#section=2D-Structure
https://pubchem.ncbi.nlm.nih.gov/compound/140606563#section=2D-Structure
https://pubchem.ncbi.nlm.nih.gov/compound/14060368#section=2D-Structure
https://pubchem.ncbi.nlm.nih.gov/compound/140602698#section=2D-Structure
https://pubchem.ncbi.nlm.nih.gov/compound/140592129#section=2D-Structure
https://pubchem.ncbi.nlm.nih.gov/compound/140590843#section=2D-Structure
https://pubchem.ncbi.nlm.nih.gov/compound/140586622#section=2D-Structure
https://pubchem.ncbi.nlm.nih.gov/compound/140583851#section=2D-Structure
https://pubchem.ncbi.nlm.nih.gov/compound/140574764#section=2D-Structure
https://pubchem.ncbi.nlm.nih.gov/compound/140566203#section=2D-Structure
https://pubchem.ncbi.nlm.nih.gov/compound/140564449#section=2D-Structure
https://pubchem.ncbi.nlm.nih.gov/compound/140563927#section=2D-Structure
https://pubchem.ncbi.nlm.nih.gov/compound/140560886#section=2D-Structure
https://pubchem.ncbi.nlm.nih.gov/compound/140553893#section=2D-Structure
https://pubchem.ncbi.nlm.nih.gov/compound/140551183#section=2D-Structure
https://pubchem.ncbi.nlm.nih.gov/compound/140551144#section=2D-Structure
https://pubchem.ncbi.nlm.nih.gov/compound/140548453#section=2D-Structure
https://pubchem.ncbi.nlm.nih.gov/compound/140547621#section=2D-Structure
https://pubchem.ncbi.nlm.nih.gov/compound/140539963#section=2D-Structure
https://pubchem.ncbi.nlm.nih.gov/compound/140537345#section=2D-Structure
https://pubchem.ncbi.nlm.nih.gov/compound/140536845#section=2D-Structure
https://pubchem.ncbi.nlm.nih.gov/compound/140530273#section=2D-Structure
https://pubchem.ncbi.nlm.nih.gov/compound/140525935#section=2D-Structure
https://pubchem.ncbi.nlm.nih.gov/compound/140519285#section=2D-Structure
https://pubchem.ncbi.nlm.nih.gov/compound/140518010#section=2D-Structure
https://pubchem.ncbi.nlm.nih.gov/compound/140517100#section=2D-Structure
https://pubchem.ncbi.nlm.nih.gov/compound/140516206#section=2D-Structure
https://pubchem.ncbi.nlm.nih.gov/compound/140515326#section=2D-Structure
https://pubchem.ncbi.nlm.nih.gov/compound/140512895#section=2D-Structure
https://pubchem.ncbi.nlm.nih.gov/compound/140510037#section=2D-Structure
https://pubchem.ncbi.nlm.nih.gov/compound/140509291#section=2D-Structure
https://pubchem.ncbi.nlm.nih.gov/compound/140506450#section=2D-Structure
https://pubchem.ncbi.nlm.nih.gov/compound/140503843#section=2D-Structure
https://pubchem.ncbi.nlm.nih.gov/compound/140497888#section=2D-Structure
https://pubchem.ncbi.nlm.nih.gov/compound/140493894#section=2D-Structure
https://pubchem.ncbi.nlm.nih.gov/compound/140486#section=2D-Structure
https://pubchem.ncbi.nlm.nih.gov/compound/140485378#section=2D-Structure
https://pubchem.ncbi.nlm.nih.gov/compound/140481134#section=2D-Structure
https://pubchem.ncbi.nlm.nih.gov/compound/140477303#section=2D-Structure
https://pubchem.ncbi.nlm.nih.gov/compound/140474956#section=2D-Structure
https://pubchem.ncbi.nlm.nih.gov/compound/140457297#section=2D-Structure
https://pubchem.ncbi.nlm.nih.gov/compound/140451657#section=2D-Structure
https://pubchem.ncbi.nlm.nih.gov/compound/140449245#section=2D-Structure

https://pubchem.ncbi.nlm.nih.gov/compound/140446381#section=2D-Structure
https://pubchem.ncbi.nlm.nih.gov/compound/140445998#section=2D-Structure
https://pubchem.ncbi.nlm.nih.gov/compound/140438347#section=2D-Structure
https://pubchem.ncbi.nlm.nih.gov/compound/140422036#section=2D-Structure
https://pubchem.ncbi.nlm.nih.gov/compound/140413479#section=2D-Structure
https://pubchem.ncbi.nlm.nih.gov/compound/140412995#section=2D-Structure
https://pubchem.ncbi.nlm.nih.gov/compound/140407842#section=2D-Structure
https://pubchem.ncbi.nlm.nih.gov/compound/140405615#section=2D-Structure
https://pubchem.ncbi.nlm.nih.gov/compound/140404481#section=2D-Structure
https://pubchem.ncbi.nlm.nih.gov/compound/140404479#section=2D-Structure
https://pubchem.ncbi.nlm.nih.gov/compound/140404187#section=2D-Structure
https://pubchem.ncbi.nlm.nih.gov/compound/14040329#section=2D-Structure
https://pubchem.ncbi.nlm.nih.gov/compound/140402753#section=2D-Structure
https://pubchem.ncbi.nlm.nih.gov/compound/140399691#section=2D-Structure
https://pubchem.ncbi.nlm.nih.gov/compound/140398768#section=2D-Structure
https://pubchem.ncbi.nlm.nih.gov/compound/140398050#section=2D-Structure
https://pubchem.ncbi.nlm.nih.gov/compound/140396104#section=2D-Structure
https://pubchem.ncbi.nlm.nih.gov/compound/140394496#section=2D-Structure
https://pubchem.ncbi.nlm.nih.gov/compound/140389912#section=2D-Structure
https://pubchem.ncbi.nlm.nih.gov/compound/140386543#section=2D-Structure
https://pubchem.ncbi.nlm.nih.gov/compound/140385254#section=2D-Structure
https://pubchem.ncbi.nlm.nih.gov/compound/140379593#section=2D-Structure
https://pubchem.ncbi.nlm.nih.gov/compound/140379218#section=2D-Structure
https://pubchem.ncbi.nlm.nih.gov/compound/140377536#section=2D-Structure
https://pubchem.ncbi.nlm.nih.gov/compound/140376079#section=2D-Structure
https://pubchem.ncbi.nlm.nih.gov/compound/140370641#section=2D-Structure
https://pubchem.ncbi.nlm.nih.gov/compound/140369364#section=2D-Structure
https://pubchem.ncbi.nlm.nih.gov/compound/140367477#section=2D-Structure
https://pubchem.ncbi.nlm.nih.gov/compound/140364293#section=2D-Structure
https://pubchem.ncbi.nlm.nih.gov/compound/140363904#section=2D-Structure
https://pubchem.ncbi.nlm.nih.gov/compound/140361734#section=2D-Structure
https://pubchem.ncbi.nlm.nih.gov/compound/140361047#section=2D-Structure
https://pubchem.ncbi.nlm.nih.gov/compound/140360663#section=2D-Structure
https://pubchem.ncbi.nlm.nih.gov/compound/140359217#section=2D-Structure
https://pubchem.ncbi.nlm.nih.gov/compound/140354141#section=2D-Structure
https://pubchem.ncbi.nlm.nih.gov/compound/140354022#section=2D-Structure
https://pubchem.ncbi.nlm.nih.gov/compound/140353206#section=2D-Structure
https://pubchem.ncbi.nlm.nih.gov/compound/140349704#section=2D-Structure
https://pubchem.ncbi.nlm.nih.gov/compound/140346845#section=2D-Structure
https://pubchem.ncbi.nlm.nih.gov/compound/140345576#section=2D-Structure
https://pubchem.ncbi.nlm.nih.gov/compound/140345110#section=2D-Structure
https://pubchem.ncbi.nlm.nih.gov/compound/140344635#section=2D-Structure
https://pubchem.ncbi.nlm.nih.gov/compound/140342935#section=2D-Structure
https://pubchem.ncbi.nlm.nih.gov/compound/140341055#section=2D-Structure
https://pubchem.ncbi.nlm.nih.gov/compound/140340663#section=2D-Structure
https://pubchem.ncbi.nlm.nih.gov/compound/140340593#section=2D-Structure
https://pubchem.ncbi.nlm.nih.gov/compound/140340108#section=2D-Structure
https://pubchem.ncbi.nlm.nih.gov/compound/140338525#section=2D-Structure
https://pubchem.ncbi.nlm.nih.gov/compound/140337774#section=2D-Structure
https://pubchem.ncbi.nlm.nih.gov/compound/140337772#section=2D-Structure
https://pubchem.ncbi.nlm.nih.gov/compound/140332480#section=2D-Structure
https://pubchem.ncbi.nlm.nih.gov/compound/140332183#section=2D-Structure
https://pubchem.ncbi.nlm.nih.gov/compound/140331833#section=2D-Structure
https://pubchem.ncbi.nlm.nih.gov/compound/140329516#section=2D-Structure
https://pubchem.ncbi.nlm.nih.gov/compound/140323857#section=2D-Structure

https://pubchem.ncbi.nlm.nih.gov/compound/140322977#section=2D-Structure
https://pubchem.ncbi.nlm.nih.gov/compound/140322631#section=2D-Structure
https://pubchem.ncbi.nlm.nih.gov/compound/140311186#section=2D-Structure
https://pubchem.ncbi.nlm.nih.gov/compound/140299033#section=2D-Structure
https://pubchem.ncbi.nlm.nih.gov/compound/140299009#section=2D-Structure
https://pubchem.ncbi.nlm.nih.gov/compound/140299007#section=2D-Structure
https://pubchem.ncbi.nlm.nih.gov/compound/140293594#section=2D-Structure
https://pubchem.ncbi.nlm.nih.gov/compound/140288819#section=2D-Structure
https://pubchem.ncbi.nlm.nih.gov/compound/14028815#section=2D-Structure
https://pubchem.ncbi.nlm.nih.gov/compound/1402668#section=2D-Structure
https://pubchem.ncbi.nlm.nih.gov/compound/140266051#section=2D-Structure
https://pubchem.ncbi.nlm.nih.gov/compound/140262277#section=2D-Structure
https://pubchem.ncbi.nlm.nih.gov/compound/140256327#section=2D-Structure
https://pubchem.ncbi.nlm.nih.gov/compound/140254608#section=2D-Structure
https://pubchem.ncbi.nlm.nih.gov/compound/140242060#section=2D-Structure
https://pubchem.ncbi.nlm.nih.gov/compound/140226943#section=2D-Structure
https://pubchem.ncbi.nlm.nih.gov/compound/140201534#section=2D-Structure
https://pubchem.ncbi.nlm.nih.gov/compound/140173346#section=2D-Structure
https://pubchem.ncbi.nlm.nih.gov/compound/140173#section=2D-Structure
https://pubchem.ncbi.nlm.nih.gov/compound/140171474#section=2D-Structure
https://pubchem.ncbi.nlm.nih.gov/compound/140167738#section=2D-Structure
https://pubchem.ncbi.nlm.nih.gov/compound/140153021#section=2D-Structure
https://pubchem.ncbi.nlm.nih.gov/compound/140152082#section=2D-Structure
https://pubchem.ncbi.nlm.nih.gov/compound/140147489#section=2D-Structure
https://pubchem.ncbi.nlm.nih.gov/compound/140140525#section=2D-Structure
https://pubchem.ncbi.nlm.nih.gov/compound/140131019#section=2D-Structure
https://pubchem.ncbi.nlm.nih.gov/compound/140130981#section=2D-Structure
https://pubchem.ncbi.nlm.nih.gov/compound/140129894#section=2D-Structure
https://pubchem.ncbi.nlm.nih.gov/compound/140124219#section=2D-Structure
https://pubchem.ncbi.nlm.nih.gov/compound/140119776#section=2D-Structure
https://pubchem.ncbi.nlm.nih.gov/compound/140118638#section=2D-Structure
https://pubchem.ncbi.nlm.nih.gov/compound/140117724#section=2D-Structure
https://pubchem.ncbi.nlm.nih.gov/compound/140109811#section=2D-Structure
https://pubchem.ncbi.nlm.nih.gov/compound/140106099#section=2D-Structure
https://pubchem.ncbi.nlm.nih.gov/compound/140093672#section=2D-Structure
https://pubchem.ncbi.nlm.nih.gov/compound/140093546#section=2D-Structure
https://pubchem.ncbi.nlm.nih.gov/compound/140089084#section=2D-Structure
https://pubchem.ncbi.nlm.nih.gov/compound/140079474#section=2D-Structure
https://pubchem.ncbi.nlm.nih.gov/compound/140063523#section=2D-Structure
https://pubchem.ncbi.nlm.nih.gov/compound/140059897#section=2D-Structure
https://pubchem.ncbi.nlm.nih.gov/compound/140048815#section=2D-Structure
https://pubchem.ncbi.nlm.nih.gov/compound/140033541#section=2D-Structure
https://pubchem.ncbi.nlm.nih.gov/compound/140032473#section=2D-Structure
https://pubchem.ncbi.nlm.nih.gov/compound/140030041#section=2D-Structure
https://pubchem.ncbi.nlm.nih.gov/compound/140030021#section=2D-Structure
https://pubchem.ncbi.nlm.nih.gov/compound/1400245#section=2D-Structure
https://pubchem.ncbi.nlm.nih.gov/compound/140015533#section=2D-Structure
https://pubchem.ncbi.nlm.nih.gov/compound/140013257#section=2D-Structure
https://pubchem.ncbi.nlm.nih.gov/compound/140004462#section=2D-Structure
https://pubchem.ncbi.nlm.nih.gov/compound/140004461#section=2D-Structure
https://pubchem.ncbi.nlm.nih.gov/compound/139993060#section=2D-Structure
https://pubchem.ncbi.nlm.nih.gov/compound/139991243#section=2D-Structure
https://pubchem.ncbi.nlm.nih.gov/compound/139984171#section=2D-Structure
https://pubchem.ncbi.nlm.nih.gov/compound/139970716#section=2D-Structure
https://pubchem.ncbi.nlm.nih.gov/compound/139966334#section=2D-Structure

https://pubchem.ncbi.nlm.nih.gov/compound/139951484#section=2D-Structure
https://pubchem.ncbi.nlm.nih.gov/compound/139949700#section=2D-Structure
https://pubchem.ncbi.nlm.nih.gov/compound/139947618#section=2D-Structure
https://pubchem.ncbi.nlm.nih.gov/compound/139944555#section=2D-Structure
https://pubchem.ncbi.nlm.nih.gov/compound/139943148#section=2D-Structure
https://pubchem.ncbi.nlm.nih.gov/compound/139934620#section=2D-Structure
https://pubchem.ncbi.nlm.nih.gov/compound/139934609#section=2D-Structure
https://pubchem.ncbi.nlm.nih.gov/compound/139933662#section=2D-Structure
https://pubchem.ncbi.nlm.nih.gov/compound/139929362#section=2D-Structure
https://pubchem.ncbi.nlm.nih.gov/compound/139916553#section=2D-Structure
https://pubchem.ncbi.nlm.nih.gov/compound/139898357#section=2D-Structure
https://pubchem.ncbi.nlm.nih.gov/compound/139894353#section=2D-Structure
https://pubchem.ncbi.nlm.nih.gov/compound/139886308#section=2D-Structure
https://pubchem.ncbi.nlm.nih.gov/compound/139846301#section=2D-Structure
https://pubchem.ncbi.nlm.nih.gov/compound/139834818#section=2D-Structure
https://pubchem.ncbi.nlm.nih.gov/compound/139830249#section=2D-Structure
https://pubchem.ncbi.nlm.nih.gov/compound/139828812#section=2D-Structure
https://pubchem.ncbi.nlm.nih.gov/compound/139823504#section=2D-Structure
https://pubchem.ncbi.nlm.nih.gov/compound/139822026#section=2D-Structure
https://pubchem.ncbi.nlm.nih.gov/compound/139809988#section=2D-Structure
https://pubchem.ncbi.nlm.nih.gov/compound/139807006#section=2D-Structure
https://pubchem.ncbi.nlm.nih.gov/compound/139791384#section=2D-Structure
https://pubchem.ncbi.nlm.nih.gov/compound/139786973#section=2D-Structure
https://pubchem.ncbi.nlm.nih.gov/compound/139785568#section=2D-Structure
https://pubchem.ncbi.nlm.nih.gov/compound/139780484#section=2D-Structure
https://pubchem.ncbi.nlm.nih.gov/compound/139779933#section=2D-Structure
https://pubchem.ncbi.nlm.nih.gov/compound/139776980#section=2D-Structure
https://pubchem.ncbi.nlm.nih.gov/compound/139776948#section=2D-Structure
https://pubchem.ncbi.nlm.nih.gov/compound/139775794#section=2D-Structure
https://pubchem.ncbi.nlm.nih.gov/compound/139773333#section=2D-Structure
https://pubchem.ncbi.nlm.nih.gov/compound/139772630#section=2D-Structure
https://pubchem.ncbi.nlm.nih.gov/compound/13976194#section=2D-Structure
https://pubchem.ncbi.nlm.nih.gov/compound/13976192#section=2D-Structure
https://pubchem.ncbi.nlm.nih.gov/compound/139756102#section=2D-Structure
https://pubchem.ncbi.nlm.nih.gov/compound/139755512#section=2D-Structure
https://pubchem.ncbi.nlm.nih.gov/compound/139721920#section=2D-Structure
https://pubchem.ncbi.nlm.nih.gov/compound/139716000#section=2D-Structure
https://pubchem.ncbi.nlm.nih.gov/compound/139709086#section=2D-Structure
https://pubchem.ncbi.nlm.nih.gov/compound/139704508#section=2D-Structure
https://pubchem.ncbi.nlm.nih.gov/compound/139692328#section=2D-Structure
https://pubchem.ncbi.nlm.nih.gov/compound/139686403#section=2D-Structure
https://pubchem.ncbi.nlm.nih.gov/compound/139680665#section=2D-Structure
https://pubchem.ncbi.nlm.nih.gov/compound/139674399#section=2D-Structure
https://pubchem.ncbi.nlm.nih.gov/compound/139662276#section=2D-Structure
https://pubchem.ncbi.nlm.nih.gov/compound/139655258#section=2D-Structure
https://pubchem.ncbi.nlm.nih.gov/compound/139642341#section=2D-Structure
https://pubchem.ncbi.nlm.nih.gov/compound/139642041#section=2D-Structure
https://pubchem.ncbi.nlm.nih.gov/compound/139637057#section=2D-Structure
https://pubchem.ncbi.nlm.nih.gov/compound/139620542#section=2D-Structure
https://pubchem.ncbi.nlm.nih.gov/compound/139619727#section=2D-Structure
https://pubchem.ncbi.nlm.nih.gov/compound/139617827#section=2D-Structure
https://pubchem.ncbi.nlm.nih.gov/compound/139616650#section=2D-Structure
https://pubchem.ncbi.nlm.nih.gov/compound/139614416#section=2D-Structure
https://pubchem.ncbi.nlm.nih.gov/compound/139601985#section=2D-Structure
https://pubchem.ncbi.nlm.nih.gov/compound/139593697#section=2D-Structure

https://pubchem.ncbi.nlm.nih.gov/compound/139593686#section=2D-Structure
https://pubchem.ncbi.nlm.nih.gov/compound/139592953#section=2D-Structure
https://pubchem.ncbi.nlm.nih.gov/compound/139585801#section=2D-Structure
https://pubchem.ncbi.nlm.nih.gov/compound/139583700#section=2D-Structure
https://pubchem.ncbi.nlm.nih.gov/compound/139565684#section=2D-Structure
https://pubchem.ncbi.nlm.nih.gov/compound/139565604#section=2D-Structure
https://pubchem.ncbi.nlm.nih.gov/compound/139559459#section=2D-Structure
https://pubchem.ncbi.nlm.nih.gov/compound/139557674#section=2D-Structure
https://pubchem.ncbi.nlm.nih.gov/compound/139557631#section=2D-Structure
https://pubchem.ncbi.nlm.nih.gov/compound/139557627#section=2D-Structure
https://pubchem.ncbi.nlm.nih.gov/compound/139518977#section=2D-Structure
https://pubchem.ncbi.nlm.nih.gov/compound/139518506#section=2D-Structure
https://pubchem.ncbi.nlm.nih.gov/compound/139505109#section=2D-Structure
https://pubchem.ncbi.nlm.nih.gov/compound/139502550#section=2D-Structure
https://pubchem.ncbi.nlm.nih.gov/compound/139491626#section=2D-Structure
https://pubchem.ncbi.nlm.nih.gov/compound/139469852#section=2D-Structure
https://pubchem.ncbi.nlm.nih.gov/compound/139451820#section=2D-Structure
https://pubchem.ncbi.nlm.nih.gov/compound/139445425#section=2D-Structure
https://pubchem.ncbi.nlm.nih.gov/compound/139442563#section=2D-Structure
https://pubchem.ncbi.nlm.nih.gov/compound/139436257#section=2D-Structure
https://pubchem.ncbi.nlm.nih.gov/compound/139436248#section=2D-Structure
https://pubchem.ncbi.nlm.nih.gov/compound/139436247#section=2D-Structure
https://pubchem.ncbi.nlm.nih.gov/compound/139436246#section=2D-Structure
https://pubchem.ncbi.nlm.nih.gov/compound/139436022#section=2D-Structure
https://pubchem.ncbi.nlm.nih.gov/compound/139436020#section=2D-Structure
https://pubchem.ncbi.nlm.nih.gov/compound/139436015#section=2D-Structure
https://pubchem.ncbi.nlm.nih.gov/compound/139435987#section=2D-Structure
https://pubchem.ncbi.nlm.nih.gov/compound/139435986#section=2D-Structure
https://pubchem.ncbi.nlm.nih.gov/compound/139435979#section=2D-Structure
https://pubchem.ncbi.nlm.nih.gov/compound/139435972#section=2D-Structure
https://pubchem.ncbi.nlm.nih.gov/compound/139435971#section=2D-Structure
https://pubchem.ncbi.nlm.nih.gov/compound/139435970#section=2D-Structure
https://pubchem.ncbi.nlm.nih.gov/compound/139435967#section=2D-Structure
https://pubchem.ncbi.nlm.nih.gov/compound/139435964#section=2D-Structure
https://pubchem.ncbi.nlm.nih.gov/compound/139435895#section=2D-Structure
https://pubchem.ncbi.nlm.nih.gov/compound/139435894#section=2D-Structure
https://pubchem.ncbi.nlm.nih.gov/compound/139435889#section=2D-Structure
https://pubchem.ncbi.nlm.nih.gov/compound/139435887#section=2D-Structure
https://pubchem.ncbi.nlm.nih.gov/compound/139435885#section=2D-Structure
https://pubchem.ncbi.nlm.nih.gov/compound/139435884#section=2D-Structure
https://pubchem.ncbi.nlm.nih.gov/compound/139435883#section=2D-Structure
https://pubchem.ncbi.nlm.nih.gov/compound/139435882#section=2D-Structure
https://pubchem.ncbi.nlm.nih.gov/compound/139435881#section=2D-Structure
https://pubchem.ncbi.nlm.nih.gov/compound/139435874#section=2D-Structure
https://pubchem.ncbi.nlm.nih.gov/compound/139435872#section=2D-Structure
https://pubchem.ncbi.nlm.nih.gov/compound/139435855#section=2D-Structure
https://pubchem.ncbi.nlm.nih.gov/compound/139435852#section=2D-Structure
https://pubchem.ncbi.nlm.nih.gov/compound/139435851#section=2D-Structure
https://pubchem.ncbi.nlm.nih.gov/compound/139435831#section=2D-Structure
https://pubchem.ncbi.nlm.nih.gov/compound/139435827#section=2D-Structure
https://pubchem.ncbi.nlm.nih.gov/compound/139427757#section=2D-Structure
https://pubchem.ncbi.nlm.nih.gov/compound/139421831#section=2D-Structure
https://pubchem.ncbi.nlm.nih.gov/compound/139419386#section=2D-Structure
https://pubchem.ncbi.nlm.nih.gov/compound/139417008#section=2D-Structure
https://pubchem.ncbi.nlm.nih.gov/compound/139416288#section=2D-Structure

https://pubchem.ncbi.nlm.nih.gov/compound/139381153#section=2D-Structure
https://pubchem.ncbi.nlm.nih.gov/compound/139378631#section=2D-Structure
https://pubchem.ncbi.nlm.nih.gov/compound/139374528#section=2D-Structure
https://pubchem.ncbi.nlm.nih.gov/compound/139360729#section=2D-Structure
https://pubchem.ncbi.nlm.nih.gov/compound/139334994#section=2D-Structure
https://pubchem.ncbi.nlm.nih.gov/compound/139329693#section=2D-Structure
https://pubchem.ncbi.nlm.nih.gov/compound/139318067#section=2D-Structure
https://pubchem.ncbi.nlm.nih.gov/compound/139316701#section=2D-Structure
https://pubchem.ncbi.nlm.nih.gov/compound/139297478#section=2D-Structure
https://pubchem.ncbi.nlm.nih.gov/compound/139266956#section=2D-Structure
https://pubchem.ncbi.nlm.nih.gov/compound/139266921#section=2D-Structure
https://pubchem.ncbi.nlm.nih.gov/compound/139266919#section=2D-Structure
https://pubchem.ncbi.nlm.nih.gov/compound/139266891#section=2D-Structure
https://pubchem.ncbi.nlm.nih.gov/compound/139266862#section=2D-Structure
https://pubchem.ncbi.nlm.nih.gov/compound/139263011#section=2D-Structure
https://pubchem.ncbi.nlm.nih.gov/compound/139253875#section=2D-Structure
https://pubchem.ncbi.nlm.nih.gov/compound/139250117#section=2D-Structure
https://pubchem.ncbi.nlm.nih.gov/compound/139249937#section=2D-Structure
https://pubchem.ncbi.nlm.nih.gov/compound/139249931#section=2D-Structure
https://pubchem.ncbi.nlm.nih.gov/compound/139249250#section=2D-Structure
https://pubchem.ncbi.nlm.nih.gov/compound/139246496#section=2D-Structure
https://pubchem.ncbi.nlm.nih.gov/compound/139245203#section=2D-Structure
https://pubchem.ncbi.nlm.nih.gov/compound/139245196#section=2D-Structure
https://pubchem.ncbi.nlm.nih.gov/compound/139245185#section=2D-Structure
https://pubchem.ncbi.nlm.nih.gov/compound/139244871#section=2D-Structure
https://pubchem.ncbi.nlm.nih.gov/compound/139244675#section=2D-Structure
https://pubchem.ncbi.nlm.nih.gov/compound/139243766#section=2D-Structure
https://pubchem.ncbi.nlm.nih.gov/compound/139243536#section=2D-Structure
https://pubchem.ncbi.nlm.nih.gov/compound/139243535#section=2D-Structure
https://pubchem.ncbi.nlm.nih.gov/compound/139243534#section=2D-Structure
https://pubchem.ncbi.nlm.nih.gov/compound/139242769#section=2D-Structure
https://pubchem.ncbi.nlm.nih.gov/compound/139242687#section=2D-Structure
https://pubchem.ncbi.nlm.nih.gov/compound/139239011#section=2D-Structure
https://pubchem.ncbi.nlm.nih.gov/compound/139232562#section=2D-Structure
https://pubchem.ncbi.nlm.nih.gov/compound/139231402#section=2D-Structure
https://pubchem.ncbi.nlm.nih.gov/compound/139230897#section=2D-Structure
https://pubchem.ncbi.nlm.nih.gov/compound/139224992#section=2D-Structure
https://pubchem.ncbi.nlm.nih.gov/compound/139224990#section=2D-Structure
https://pubchem.ncbi.nlm.nih.gov/compound/139219616#section=2D-Structure
https://pubchem.ncbi.nlm.nih.gov/compound/139215547#section=2D-Structure
https://pubchem.ncbi.nlm.nih.gov/compound/139215053#section=2D-Structure
https://pubchem.ncbi.nlm.nih.gov/compound/139214687#section=2D-Structure
https://pubchem.ncbi.nlm.nih.gov/compound/139207808#section=2D-Structure
https://pubchem.ncbi.nlm.nih.gov/compound/139181328#section=2D-Structure
https://pubchem.ncbi.nlm.nih.gov/compound/139178661#section=2D-Structure
https://pubchem.ncbi.nlm.nih.gov/compound/139173742#section=2D-Structure
https://pubchem.ncbi.nlm.nih.gov/compound/139114519#section=2D-Structure
https://pubchem.ncbi.nlm.nih.gov/compound/139103148#section=2D-Structure
https://pubchem.ncbi.nlm.nih.gov/compound/139101709#section=2D-Structure
https://pubchem.ncbi.nlm.nih.gov/compound/139096158#section=2D-Structure
https://pubchem.ncbi.nlm.nih.gov/compound/139066244#section=2D-Structure
https://pubchem.ncbi.nlm.nih.gov/compound/139049082#section=2D-Structure
https://pubchem.ncbi.nlm.nih.gov/compound/139034813#section=2D-Structure
https://pubchem.ncbi.nlm.nih.gov/compound/13902866#section=2D-Structure
https://pubchem.ncbi.nlm.nih.gov/compound/13902864#section=2D-Structure

https://pubchem.ncbi.nlm.nih.gov/compound/139024388#section=2D-Structure
https://pubchem.ncbi.nlm.nih.gov/compound/13899145#section=2D-Structure
https://pubchem.ncbi.nlm.nih.gov/compound/13899136#section=2D-Structure
https://pubchem.ncbi.nlm.nih.gov/compound/138986898#section=2D-Structure
https://pubchem.ncbi.nlm.nih.gov/compound/138976344#section=2D-Structure
https://pubchem.ncbi.nlm.nih.gov/compound/138970765#section=2D-Structure
https://pubchem.ncbi.nlm.nih.gov/compound/138963076#section=2D-Structure
https://pubchem.ncbi.nlm.nih.gov/compound/138963001#section=2D-Structure
https://pubchem.ncbi.nlm.nih.gov/compound/138961440#section=2D-Structure
https://pubchem.ncbi.nlm.nih.gov/compound/138779#section=2D-Structure
https://pubchem.ncbi.nlm.nih.gov/compound/138756899#section=2D-Structure
https://pubchem.ncbi.nlm.nih.gov/compound/138756856#section=2D-Structure
https://pubchem.ncbi.nlm.nih.gov/compound/138756304#section=2D-Structure
https://pubchem.ncbi.nlm.nih.gov/compound/138756297#section=2D-Structure
https://pubchem.ncbi.nlm.nih.gov/compound/138753445#section=2D-Structure
https://pubchem.ncbi.nlm.nih.gov/compound/138753430#section=2D-Structure
https://pubchem.ncbi.nlm.nih.gov/compound/138753411#section=2D-Structure
https://pubchem.ncbi.nlm.nih.gov/compound/138753410#section=2D-Structure
https://pubchem.ncbi.nlm.nih.gov/compound/138753219#section=2D-Structure
https://pubchem.ncbi.nlm.nih.gov/compound/138748980#section=2D-Structure
https://pubchem.ncbi.nlm.nih.gov/compound/138748456#section=2D-Structure
https://pubchem.ncbi.nlm.nih.gov/compound/138747694#section=2D-Structure
https://pubchem.ncbi.nlm.nih.gov/compound/138741024#section=2D-Structure
https://pubchem.ncbi.nlm.nih.gov/compound/138737895#section=2D-Structure
https://pubchem.ncbi.nlm.nih.gov/compound/138709029#section=2D-Structure
https://pubchem.ncbi.nlm.nih.gov/compound/138708569#section=2D-Structure
https://pubchem.ncbi.nlm.nih.gov/compound/138706625#section=2D-Structure
https://pubchem.ncbi.nlm.nih.gov/compound/138687159#section=2D-Structure
https://pubchem.ncbi.nlm.nih.gov/compound/13868660#section=2D-Structure
https://pubchem.ncbi.nlm.nih.gov/compound/138676876#section=2D-Structure
https://pubchem.ncbi.nlm.nih.gov/compound/138674212#section=2D-Structure
https://pubchem.ncbi.nlm.nih.gov/compound/138671766#section=2D-Structure
https://pubchem.ncbi.nlm.nih.gov/compound/13866593#section=2D-Structure
https://pubchem.ncbi.nlm.nih.gov/compound/138664516#section=2D-Structure
https://pubchem.ncbi.nlm.nih.gov/compound/138660921#section=2D-Structure
https://pubchem.ncbi.nlm.nih.gov/compound/138660912#section=2D-Structure
https://pubchem.ncbi.nlm.nih.gov/compound/138658848#section=2D-Structure
https://pubchem.ncbi.nlm.nih.gov/compound/138658846#section=2D-Structure
https://pubchem.ncbi.nlm.nih.gov/compound/138642011#section=2D-Structure
https://pubchem.ncbi.nlm.nih.gov/compound/138631749#section=2D-Structure
https://pubchem.ncbi.nlm.nih.gov/compound/138614543#section=2D-Structure
https://pubchem.ncbi.nlm.nih.gov/compound/138600269#section=2D-Structure
https://pubchem.ncbi.nlm.nih.gov/compound/138598764#section=2D-Structure
https://pubchem.ncbi.nlm.nih.gov/compound/138598725#section=2D-Structure
https://pubchem.ncbi.nlm.nih.gov/compound/138584151#section=2D-Structure
https://pubchem.ncbi.nlm.nih.gov/compound/138582724#section=2D-Structure
https://pubchem.ncbi.nlm.nih.gov/compound/138563327#section=2D-Structure
https://pubchem.ncbi.nlm.nih.gov/compound/138548320#section=2D-Structure
https://pubchem.ncbi.nlm.nih.gov/compound/13853725#section=2D-Structure
https://pubchem.ncbi.nlm.nih.gov/compound/138510821#section=2D-Structure
https://pubchem.ncbi.nlm.nih.gov/compound/138502555#section=2D-Structure
https://pubchem.ncbi.nlm.nih.gov/compound/138481103#section=2D-Structure
https://pubchem.ncbi.nlm.nih.gov/compound/138481100#section=2D-Structure
https://pubchem.ncbi.nlm.nih.gov/compound/138481098#section=2D-Structure
https://pubchem.ncbi.nlm.nih.gov/compound/138478292#section=2D-Structure

https://pubchem.ncbi.nlm.nih.gov/compound/138402088#section=2D-Structure
https://pubchem.ncbi.nlm.nih.gov/compound/138400829#section=2D-Structure
https://pubchem.ncbi.nlm.nih.gov/compound/138396855#section=2D-Structure
https://pubchem.ncbi.nlm.nih.gov/compound/138376773#section=2D-Structure
https://pubchem.ncbi.nlm.nih.gov/compound/138373836#section=2D-Structure
https://pubchem.ncbi.nlm.nih.gov/compound/138373805#section=2D-Structure
https://pubchem.ncbi.nlm.nih.gov/compound/13835279#section=2D-Structure
https://pubchem.ncbi.nlm.nih.gov/compound/13834862#section=2D-Structure
https://pubchem.ncbi.nlm.nih.gov/compound/13834860#section=2D-Structure
https://pubchem.ncbi.nlm.nih.gov/compound/13827938#section=2D-Structure
https://pubchem.ncbi.nlm.nih.gov/compound/13827937#section=2D-Structure
https://pubchem.ncbi.nlm.nih.gov/compound/13827102#section=2D-Structure
https://pubchem.ncbi.nlm.nih.gov/compound/138115457#section=2D-Structure
https://pubchem.ncbi.nlm.nih.gov/compound/138059371#section=2D-Structure
https://pubchem.ncbi.nlm.nih.gov/compound/138059285#section=2D-Structure
https://pubchem.ncbi.nlm.nih.gov/compound/138058914#section=2D-Structure
https://pubchem.ncbi.nlm.nih.gov/compound/138058907#section=2D-Structure
https://pubchem.ncbi.nlm.nih.gov/compound/138058902#section=2D-Structure
https://pubchem.ncbi.nlm.nih.gov/compound/138058801#section=2D-Structure
https://pubchem.ncbi.nlm.nih.gov/compound/13783592#section=2D-Structure
https://pubchem.ncbi.nlm.nih.gov/compound/13781809#section=2D-Structure
https://pubchem.ncbi.nlm.nih.gov/compound/13781592#section=2D-Structure
https://pubchem.ncbi.nlm.nih.gov/compound/137797120#section=2D-Structure
https://pubchem.ncbi.nlm.nih.gov/compound/137754#section=2D-Structure
https://pubchem.ncbi.nlm.nih.gov/compound/137698664#section=2D-Structure
https://pubchem.ncbi.nlm.nih.gov/compound/137698624#section=2D-Structure
https://pubchem.ncbi.nlm.nih.gov/compound/137698461#section=2D-Structure
https://pubchem.ncbi.nlm.nih.gov/compound/137632031#section=2D-Structure
https://pubchem.ncbi.nlm.nih.gov/compound/13762205#section=2D-Structure
https://pubchem.ncbi.nlm.nih.gov/compound/137526842#section=2D-Structure
https://pubchem.ncbi.nlm.nih.gov/compound/137515946#section=2D-Structure
https://pubchem.ncbi.nlm.nih.gov/compound/137506183#section=2D-Structure
https://pubchem.ncbi.nlm.nih.gov/compound/137502035#section=2D-Structure
https://pubchem.ncbi.nlm.nih.gov/compound/137501798#section=2D-Structure
https://pubchem.ncbi.nlm.nih.gov/compound/137501514#section=2D-Structure
https://pubchem.ncbi.nlm.nih.gov/compound/137501432#section=2D-Structure
https://pubchem.ncbi.nlm.nih.gov/compound/137477608#section=2D-Structure
https://pubchem.ncbi.nlm.nih.gov/compound/137466756#section=2D-Structure
https://pubchem.ncbi.nlm.nih.gov/compound/137465078#section=2D-Structure
https://pubchem.ncbi.nlm.nih.gov/compound/137446813#section=2D-Structure
https://pubchem.ncbi.nlm.nih.gov/compound/137427590#section=2D-Structure
https://pubchem.ncbi.nlm.nih.gov/compound/137427423#section=2D-Structure
https://pubchem.ncbi.nlm.nih.gov/compound/137426776#section=2D-Structure
https://pubchem.ncbi.nlm.nih.gov/compound/137422000#section=2D-Structure
https://pubchem.ncbi.nlm.nih.gov/compound/137421996#section=2D-Structure
https://pubchem.ncbi.nlm.nih.gov/compound/137419604#section=2D-Structure
https://pubchem.ncbi.nlm.nih.gov/compound/137417555#section=2D-Structure
https://pubchem.ncbi.nlm.nih.gov/compound/137417491#section=2D-Structure
https://pubchem.ncbi.nlm.nih.gov/compound/137417374#section=2D-Structure
https://pubchem.ncbi.nlm.nih.gov/compound/13739856#section=2D-Structure
https://pubchem.ncbi.nlm.nih.gov/compound/137389560#section=2D-Structure
https://pubchem.ncbi.nlm.nih.gov/compound/137379190#section=2D-Structure
https://pubchem.ncbi.nlm.nih.gov/compound/137372115#section=2D-Structure
https://pubchem.ncbi.nlm.nih.gov/compound/137350255#section=2D-Structure
https://pubchem.ncbi.nlm.nih.gov/compound/137346105#section=2D-Structure

https://pubchem.ncbi.nlm.nih.gov/compound/137346089#section=2D-Structure
https://pubchem.ncbi.nlm.nih.gov/compound/137346088#section=2D-Structure
https://pubchem.ncbi.nlm.nih.gov/compound/137332143#section=2D-Structure
https://pubchem.ncbi.nlm.nih.gov/compound/137321272#section=2D-Structure
https://pubchem.ncbi.nlm.nih.gov/compound/137310050#section=2D-Structure
https://pubchem.ncbi.nlm.nih.gov/compound/137305441#section=2D-Structure
https://pubchem.ncbi.nlm.nih.gov/compound/137295716#section=2D-Structure
https://pubchem.ncbi.nlm.nih.gov/compound/137294#section=2D-Structure
https://pubchem.ncbi.nlm.nih.gov/compound/137292528#section=2D-Structure
https://pubchem.ncbi.nlm.nih.gov/compound/137290998#section=2D-Structure
https://pubchem.ncbi.nlm.nih.gov/compound/137286993#section=2D-Structure
https://pubchem.ncbi.nlm.nih.gov/compound/137284474#section=2D-Structure
https://pubchem.ncbi.nlm.nih.gov/compound/137277411#section=2D-Structure
https://pubchem.ncbi.nlm.nih.gov/compound/137277399#section=2D-Structure
https://pubchem.ncbi.nlm.nih.gov/compound/137277312#section=2D-Structure
https://pubchem.ncbi.nlm.nih.gov/compound/137276594#section=2D-Structure
https://pubchem.ncbi.nlm.nih.gov/compound/137270238#section=2D-Structure
https://pubchem.ncbi.nlm.nih.gov/compound/137265607#section=2D-Structure
https://pubchem.ncbi.nlm.nih.gov/compound/137263665#section=2D-Structure
https://pubchem.ncbi.nlm.nih.gov/compound/137258605#section=2D-Structure
https://pubchem.ncbi.nlm.nih.gov/compound/137254993#section=2D-Structure
https://pubchem.ncbi.nlm.nih.gov/compound/137254202#section=2D-Structure
https://pubchem.ncbi.nlm.nih.gov/compound/137253904#section=2D-Structure
https://pubchem.ncbi.nlm.nih.gov/compound/137253689#section=2D-Structure
https://pubchem.ncbi.nlm.nih.gov/compound/137253349#section=2D-Structure
https://pubchem.ncbi.nlm.nih.gov/compound/137250232#section=2D-Structure
https://pubchem.ncbi.nlm.nih.gov/compound/137241216#section=2D-Structure
https://pubchem.ncbi.nlm.nih.gov/compound/137240528#section=2D-Structure
https://pubchem.ncbi.nlm.nih.gov/compound/137240508#section=2D-Structure
https://pubchem.ncbi.nlm.nih.gov/compound/137240494#section=2D-Structure
https://pubchem.ncbi.nlm.nih.gov/compound/137228033#section=2D-Structure
https://pubchem.ncbi.nlm.nih.gov/compound/137226062#section=2D-Structure
https://pubchem.ncbi.nlm.nih.gov/compound/137226043#section=2D-Structure
https://pubchem.ncbi.nlm.nih.gov/compound/137225927#section=2D-Structure
https://pubchem.ncbi.nlm.nih.gov/compound/137224668#section=2D-Structure
https://pubchem.ncbi.nlm.nih.gov/compound/137224633#section=2D-Structure
https://pubchem.ncbi.nlm.nih.gov/compound/137224469#section=2D-Structure
https://pubchem.ncbi.nlm.nih.gov/compound/137223027#section=2D-Structure
https://pubchem.ncbi.nlm.nih.gov/compound/137222966#section=2D-Structure
https://pubchem.ncbi.nlm.nih.gov/compound/137222920#section=2D-Structure
https://pubchem.ncbi.nlm.nih.gov/compound/137222290#section=2D-Structure
https://pubchem.ncbi.nlm.nih.gov/compound/137221104#section=2D-Structure
https://pubchem.ncbi.nlm.nih.gov/compound/137219529#section=2D-Structure
https://pubchem.ncbi.nlm.nih.gov/compound/137219476#section=2D-Structure
https://pubchem.ncbi.nlm.nih.gov/compound/137219474#section=2D-Structure
https://pubchem.ncbi.nlm.nih.gov/compound/137219306#section=2D-Structure
https://pubchem.ncbi.nlm.nih.gov/compound/137219305#section=2D-Structure
https://pubchem.ncbi.nlm.nih.gov/compound/137215199#section=2D-Structure
https://pubchem.ncbi.nlm.nih.gov/compound/137214410#section=2D-Structure
https://pubchem.ncbi.nlm.nih.gov/compound/137214323#section=2D-Structure
https://pubchem.ncbi.nlm.nih.gov/compound/137213811#section=2D-Structure
https://pubchem.ncbi.nlm.nih.gov/compound/137213810#section=2D-Structure
https://pubchem.ncbi.nlm.nih.gov/compound/137213615#section=2D-Structure
https://pubchem.ncbi.nlm.nih.gov/compound/137212226#section=2D-Structure
https://pubchem.ncbi.nlm.nih.gov/compound/137211591#section=2D-Structure

https://pubchem.ncbi.nlm.nih.gov/compound/137211581#section=2D-Structure
https://pubchem.ncbi.nlm.nih.gov/compound/137211568#section=2D-Structure
https://pubchem.ncbi.nlm.nih.gov/compound/137211555#section=2D-Structure
https://pubchem.ncbi.nlm.nih.gov/compound/137210190#section=2D-Structure
https://pubchem.ncbi.nlm.nih.gov/compound/137210185#section=2D-Structure
https://pubchem.ncbi.nlm.nih.gov/compound/137209967#section=2D-Structure
https://pubchem.ncbi.nlm.nih.gov/compound/137209922#section=2D-Structure
https://pubchem.ncbi.nlm.nih.gov/compound/137209856#section=2D-Structure
https://pubchem.ncbi.nlm.nih.gov/compound/137207588#section=2D-Structure
https://pubchem.ncbi.nlm.nih.gov/compound/137206748#section=2D-Structure
https://pubchem.ncbi.nlm.nih.gov/compound/137205822#section=2D-Structure
https://pubchem.ncbi.nlm.nih.gov/compound/137205752#section=2D-Structure
https://pubchem.ncbi.nlm.nih.gov/compound/137205451#section=2D-Structure
https://pubchem.ncbi.nlm.nih.gov/compound/137205209#section=2D-Structure
https://pubchem.ncbi.nlm.nih.gov/compound/137204251#section=2D-Structure
https://pubchem.ncbi.nlm.nih.gov/compound/137203317#section=2D-Structure
https://pubchem.ncbi.nlm.nih.gov/compound/137202726#section=2D-Structure
https://pubchem.ncbi.nlm.nih.gov/compound/137202722#section=2D-Structure
https://pubchem.ncbi.nlm.nih.gov/compound/137202639#section=2D-Structure
https://pubchem.ncbi.nlm.nih.gov/compound/137202472#section=2D-Structure
https://pubchem.ncbi.nlm.nih.gov/compound/137201418#section=2D-Structure
https://pubchem.ncbi.nlm.nih.gov/compound/137201398#section=2D-Structure
https://pubchem.ncbi.nlm.nih.gov/compound/137201290#section=2D-Structure
https://pubchem.ncbi.nlm.nih.gov/compound/137201258#section=2D-Structure
https://pubchem.ncbi.nlm.nih.gov/compound/137201071#section=2D-Structure
https://pubchem.ncbi.nlm.nih.gov/compound/137201070#section=2D-Structure
https://pubchem.ncbi.nlm.nih.gov/compound/137200584#section=2D-Structure
https://pubchem.ncbi.nlm.nih.gov/compound/137199492#section=2D-Structure
https://pubchem.ncbi.nlm.nih.gov/compound/137199277#section=2D-Structure
https://pubchem.ncbi.nlm.nih.gov/compound/137199274#section=2D-Structure
https://pubchem.ncbi.nlm.nih.gov/compound/137198808#section=2D-Structure
https://pubchem.ncbi.nlm.nih.gov/compound/137197373#section=2D-Structure
https://pubchem.ncbi.nlm.nih.gov/compound/137197333#section=2D-Structure
https://pubchem.ncbi.nlm.nih.gov/compound/137196324#section=2D-Structure
https://pubchem.ncbi.nlm.nih.gov/compound/137196135#section=2D-Structure
https://pubchem.ncbi.nlm.nih.gov/compound/137195149#section=2D-Structure
https://pubchem.ncbi.nlm.nih.gov/compound/137195126#section=2D-Structure
https://pubchem.ncbi.nlm.nih.gov/compound/137194385#section=2D-Structure
https://pubchem.ncbi.nlm.nih.gov/compound/137194105#section=2D-Structure
https://pubchem.ncbi.nlm.nih.gov/compound/137193533#section=2D-Structure
https://pubchem.ncbi.nlm.nih.gov/compound/137193265#section=2D-Structure
https://pubchem.ncbi.nlm.nih.gov/compound/137193255#section=2D-Structure
https://pubchem.ncbi.nlm.nih.gov/compound/137192853#section=2D-Structure
https://pubchem.ncbi.nlm.nih.gov/compound/137192734#section=2D-Structure
https://pubchem.ncbi.nlm.nih.gov/compound/137192720#section=2D-Structure
https://pubchem.ncbi.nlm.nih.gov/compound/137192709#section=2D-Structure
https://pubchem.ncbi.nlm.nih.gov/compound/137192667#section=2D-Structure
https://pubchem.ncbi.nlm.nih.gov/compound/137190705#section=2D-Structure
https://pubchem.ncbi.nlm.nih.gov/compound/137190703#section=2D-Structure
https://pubchem.ncbi.nlm.nih.gov/compound/137187623#section=2D-Structure
https://pubchem.ncbi.nlm.nih.gov/compound/137187496#section=2D-Structure
https://pubchem.ncbi.nlm.nih.gov/compound/137187482#section=2D-Structure
https://pubchem.ncbi.nlm.nih.gov/compound/137187449#section=2D-Structure
https://pubchem.ncbi.nlm.nih.gov/compound/137187446#section=2D-Structure
https://pubchem.ncbi.nlm.nih.gov/compound/137187377#section=2D-Structure

https://pubchem.ncbi.nlm.nih.gov/compound/137187375#section=2D-Structure
https://pubchem.ncbi.nlm.nih.gov/compound/137187248#section=2D-Structure
https://pubchem.ncbi.nlm.nih.gov/compound/137186382#section=2D-Structure
https://pubchem.ncbi.nlm.nih.gov/compound/137186323#section=2D-Structure
https://pubchem.ncbi.nlm.nih.gov/compound/137186297#section=2D-Structure
https://pubchem.ncbi.nlm.nih.gov/compound/137186293#section=2D-Structure
https://pubchem.ncbi.nlm.nih.gov/compound/137186267#section=2D-Structure
https://pubchem.ncbi.nlm.nih.gov/compound/137185813#section=2D-Structure
https://pubchem.ncbi.nlm.nih.gov/compound/137185439#section=2D-Structure
https://pubchem.ncbi.nlm.nih.gov/compound/137184715#section=2D-Structure
https://pubchem.ncbi.nlm.nih.gov/compound/137184665#section=2D-Structure
https://pubchem.ncbi.nlm.nih.gov/compound/137184582#section=2D-Structure
https://pubchem.ncbi.nlm.nih.gov/compound/137184460#section=2D-Structure
https://pubchem.ncbi.nlm.nih.gov/compound/137184459#section=2D-Structure
https://pubchem.ncbi.nlm.nih.gov/compound/137184353#section=2D-Structure
https://pubchem.ncbi.nlm.nih.gov/compound/137184348#section=2D-Structure
https://pubchem.ncbi.nlm.nih.gov/compound/137183922#section=2D-Structure
https://pubchem.ncbi.nlm.nih.gov/compound/137183896#section=2D-Structure
https://pubchem.ncbi.nlm.nih.gov/compound/137182840#section=2D-Structure
https://pubchem.ncbi.nlm.nih.gov/compound/137182763#section=2D-Structure
https://pubchem.ncbi.nlm.nih.gov/compound/137182739#section=2D-Structure
https://pubchem.ncbi.nlm.nih.gov/compound/137182614#section=2D-Structure
https://pubchem.ncbi.nlm.nih.gov/compound/137182570#section=2D-Structure
https://pubchem.ncbi.nlm.nih.gov/compound/137182477#section=2D-Structure
https://pubchem.ncbi.nlm.nih.gov/compound/137182398#section=2D-Structure
https://pubchem.ncbi.nlm.nih.gov/compound/137181248#section=2D-Structure
https://pubchem.ncbi.nlm.nih.gov/compound/137180948#section=2D-Structure
https://pubchem.ncbi.nlm.nih.gov/compound/137180918#section=2D-Structure
https://pubchem.ncbi.nlm.nih.gov/compound/137179308#section=2D-Structure
https://pubchem.ncbi.nlm.nih.gov/compound/137178523#section=2D-Structure
https://pubchem.ncbi.nlm.nih.gov/compound/137178482#section=2D-Structure
https://pubchem.ncbi.nlm.nih.gov/compound/137176113#section=2D-Structure
https://pubchem.ncbi.nlm.nih.gov/compound/137176064#section=2D-Structure
https://pubchem.ncbi.nlm.nih.gov/compound/137176037#section=2D-Structure
https://pubchem.ncbi.nlm.nih.gov/compound/137176021#section=2D-Structure
https://pubchem.ncbi.nlm.nih.gov/compound/137175995#section=2D-Structure
https://pubchem.ncbi.nlm.nih.gov/compound/137170241#section=2D-Structure
https://pubchem.ncbi.nlm.nih.gov/compound/137153918#section=2D-Structure
https://pubchem.ncbi.nlm.nih.gov/compound/137153699#section=2D-Structure
https://pubchem.ncbi.nlm.nih.gov/compound/137143146#section=2D-Structure
https://pubchem.ncbi.nlm.nih.gov/compound/137140#section=2D-Structure
https://pubchem.ncbi.nlm.nih.gov/compound/137132878#section=2D-Structure
https://pubchem.ncbi.nlm.nih.gov/compound/137131564#section=2D-Structure
https://pubchem.ncbi.nlm.nih.gov/compound/137128913#section=2D-Structure
https://pubchem.ncbi.nlm.nih.gov/compound/137128712#section=2D-Structure
https://pubchem.ncbi.nlm.nih.gov/compound/137127118#section=2D-Structure
https://pubchem.ncbi.nlm.nih.gov/compound/137126572#section=2D-Structure
https://pubchem.ncbi.nlm.nih.gov/compound/137126325#section=2D-Structure
https://pubchem.ncbi.nlm.nih.gov/compound/137125503#section=2D-Structure
https://pubchem.ncbi.nlm.nih.gov/compound/137125467#section=2D-Structure
https://pubchem.ncbi.nlm.nih.gov/compound/137123966#section=2D-Structure
https://pubchem.ncbi.nlm.nih.gov/compound/137122587#section=2D-Structure
https://pubchem.ncbi.nlm.nih.gov/compound/137108885#section=2D-Structure
https://pubchem.ncbi.nlm.nih.gov/compound/137103397#section=2D-Structure
https://pubchem.ncbi.nlm.nih.gov/compound/137101565#section=2D-Structure

https://pubchem.ncbi.nlm.nih.gov/compound/137079413#section=2D-Structure
https://pubchem.ncbi.nlm.nih.gov/compound/137072921#section=2D-Structure
https://pubchem.ncbi.nlm.nih.gov/compound/137066609#section=2D-Structure
https://pubchem.ncbi.nlm.nih.gov/compound/137062006#section=2D-Structure
https://pubchem.ncbi.nlm.nih.gov/compound/137060358#section=2D-Structure
https://pubchem.ncbi.nlm.nih.gov/compound/137053600#section=2D-Structure
https://pubchem.ncbi.nlm.nih.gov/compound/137045353#section=2D-Structure
https://pubchem.ncbi.nlm.nih.gov/compound/137030950#section=2D-Structure
https://pubchem.ncbi.nlm.nih.gov/compound/137028764#section=2D-Structure
https://pubchem.ncbi.nlm.nih.gov/compound/137015138#section=2D-Structure
https://pubchem.ncbi.nlm.nih.gov/compound/137015137#section=2D-Structure
https://pubchem.ncbi.nlm.nih.gov/compound/137015136#section=2D-Structure
https://pubchem.ncbi.nlm.nih.gov/compound/137015135#section=2D-Structure
https://pubchem.ncbi.nlm.nih.gov/compound/137015134#section=2D-Structure
https://pubchem.ncbi.nlm.nih.gov/compound/137010335#section=2D-Structure
https://pubchem.ncbi.nlm.nih.gov/compound/137004405#section=2D-Structure
https://pubchem.ncbi.nlm.nih.gov/compound/136999828#section=2D-Structure
https://pubchem.ncbi.nlm.nih.gov/compound/136999641#section=2D-Structure
https://pubchem.ncbi.nlm.nih.gov/compound/136999640#section=2D-Structure
https://pubchem.ncbi.nlm.nih.gov/compound/136999639#section=2D-Structure
https://pubchem.ncbi.nlm.nih.gov/compound/136999636#section=2D-Structure
https://pubchem.ncbi.nlm.nih.gov/compound/136999634#section=2D-Structure
https://pubchem.ncbi.nlm.nih.gov/compound/136999633#section=2D-Structure
https://pubchem.ncbi.nlm.nih.gov/compound/136987554#section=2D-Structure
https://pubchem.ncbi.nlm.nih.gov/compound/136984234#section=2D-Structure
https://pubchem.ncbi.nlm.nih.gov/compound/136982182#section=2D-Structure
https://pubchem.ncbi.nlm.nih.gov/compound/136982179#section=2D-Structure
https://pubchem.ncbi.nlm.nih.gov/compound/136982178#section=2D-Structure
https://pubchem.ncbi.nlm.nih.gov/compound/136980671#section=2D-Structure
https://pubchem.ncbi.nlm.nih.gov/compound/136980600#section=2D-Structure
https://pubchem.ncbi.nlm.nih.gov/compound/136964365#section=2D-Structure
https://pubchem.ncbi.nlm.nih.gov/compound/136960066#section=2D-Structure
https://pubchem.ncbi.nlm.nih.gov/compound/136959589#section=2D-Structure
https://pubchem.ncbi.nlm.nih.gov/compound/136952168#section=2D-Structure
https://pubchem.ncbi.nlm.nih.gov/compound/136947688#section=2D-Structure
https://pubchem.ncbi.nlm.nih.gov/compound/136931195#section=2D-Structure
https://pubchem.ncbi.nlm.nih.gov/compound/136930530#section=2D-Structure
https://pubchem.ncbi.nlm.nih.gov/compound/136929376#section=2D-Structure
https://pubchem.ncbi.nlm.nih.gov/compound/136927833#section=2D-Structure
https://pubchem.ncbi.nlm.nih.gov/compound/136900302#section=2D-Structure
https://pubchem.ncbi.nlm.nih.gov/compound/136900123#section=2D-Structure
https://pubchem.ncbi.nlm.nih.gov/compound/136889986#section=2D-Structure
https://pubchem.ncbi.nlm.nih.gov/compound/136881011#section=2D-Structure
https://pubchem.ncbi.nlm.nih.gov/compound/136862702#section=2D-Structure
https://pubchem.ncbi.nlm.nih.gov/compound/136861754#section=2D-Structure
https://pubchem.ncbi.nlm.nih.gov/compound/136846020#section=2D-Structure
https://pubchem.ncbi.nlm.nih.gov/compound/136817926#section=2D-Structure
https://pubchem.ncbi.nlm.nih.gov/compound/136808180#section=2D-Structure
https://pubchem.ncbi.nlm.nih.gov/compound/136772502#section=2D-Structure
https://pubchem.ncbi.nlm.nih.gov/compound/136765386#section=2D-Structure
https://pubchem.ncbi.nlm.nih.gov/compound/136762339#section=2D-Structure
https://pubchem.ncbi.nlm.nih.gov/compound/136760120#section=2D-Structure
https://pubchem.ncbi.nlm.nih.gov/compound/136752192#section=2D-Structure
https://pubchem.ncbi.nlm.nih.gov/compound/136746555#section=2D-Structure
https://pubchem.ncbi.nlm.nih.gov/compound/13674165#section=2D-Structure

https://pubchem.ncbi.nlm.nih.gov/compound/136740545#section=2D-Structure
https://pubchem.ncbi.nlm.nih.gov/compound/136738281#section=2D-Structure
https://pubchem.ncbi.nlm.nih.gov/compound/136726337#section=2D-Structure
https://pubchem.ncbi.nlm.nih.gov/compound/136705119#section=2D-Structure
https://pubchem.ncbi.nlm.nih.gov/compound/136700209#section=2D-Structure
https://pubchem.ncbi.nlm.nih.gov/compound/136693570#section=2D-Structure
https://pubchem.ncbi.nlm.nih.gov/compound/136688211#section=2D-Structure
https://pubchem.ncbi.nlm.nih.gov/compound/136679963#section=2D-Structure
https://pubchem.ncbi.nlm.nih.gov/compound/136662873#section=2D-Structure
https://pubchem.ncbi.nlm.nih.gov/compound/136662789#section=2D-Structure
https://pubchem.ncbi.nlm.nih.gov/compound/136659816#section=2D-Structure
https://pubchem.ncbi.nlm.nih.gov/compound/136657943#section=2D-Structure
https://pubchem.ncbi.nlm.nih.gov/compound/136657384#section=2D-Structure
https://pubchem.ncbi.nlm.nih.gov/compound/136655332#section=2D-Structure
https://pubchem.ncbi.nlm.nih.gov/compound/136654516#section=2D-Structure
https://pubchem.ncbi.nlm.nih.gov/compound/136654408#section=2D-Structure
https://pubchem.ncbi.nlm.nih.gov/compound/136653780#section=2D-Structure
https://pubchem.ncbi.nlm.nih.gov/compound/136652242#section=2D-Structure
https://pubchem.ncbi.nlm.nih.gov/compound/136651536#section=2D-Structure
https://pubchem.ncbi.nlm.nih.gov/compound/136651447#section=2D-Structure
https://pubchem.ncbi.nlm.nih.gov/compound/136651306#section=2D-Structure
https://pubchem.ncbi.nlm.nih.gov/compound/136650483#section=2D-Structure
https://pubchem.ncbi.nlm.nih.gov/compound/136644822#section=2D-Structure
https://pubchem.ncbi.nlm.nih.gov/compound/136644411#section=2D-Structure
https://pubchem.ncbi.nlm.nih.gov/compound/136638665#section=2D-Structure
https://pubchem.ncbi.nlm.nih.gov/compound/136636036#section=2D-Structure
https://pubchem.ncbi.nlm.nih.gov/compound/136635631#section=2D-Structure
https://pubchem.ncbi.nlm.nih.gov/compound/136635333#section=2D-Structure
https://pubchem.ncbi.nlm.nih.gov/compound/136634948#section=2D-Structure
https://pubchem.ncbi.nlm.nih.gov/compound/136629763#section=2D-Structure
https://pubchem.ncbi.nlm.nih.gov/compound/136629693#section=2D-Structure
https://pubchem.ncbi.nlm.nih.gov/compound/136628514#section=2D-Structure
https://pubchem.ncbi.nlm.nih.gov/compound/136628012#section=2D-Structure
https://pubchem.ncbi.nlm.nih.gov/compound/136627750#section=2D-Structure
https://pubchem.ncbi.nlm.nih.gov/compound/136624454#section=2D-Structure
https://pubchem.ncbi.nlm.nih.gov/compound/136620622#section=2D-Structure
https://pubchem.ncbi.nlm.nih.gov/compound/136619686#section=2D-Structure
https://pubchem.ncbi.nlm.nih.gov/compound/136618074#section=2D-Structure
https://pubchem.ncbi.nlm.nih.gov/compound/136600491#section=2D-Structure
https://pubchem.ncbi.nlm.nih.gov/compound/136598378#section=2D-Structure
https://pubchem.ncbi.nlm.nih.gov/compound/136598338#section=2D-Structure
https://pubchem.ncbi.nlm.nih.gov/compound/136596840#section=2D-Structure
https://pubchem.ncbi.nlm.nih.gov/compound/136594301#section=2D-Structure
https://pubchem.ncbi.nlm.nih.gov/compound/136593559#section=2D-Structure
https://pubchem.ncbi.nlm.nih.gov/compound/136510284#section=2D-Structure
https://pubchem.ncbi.nlm.nih.gov/compound/136509900#section=2D-Structure
https://pubchem.ncbi.nlm.nih.gov/compound/136505105#section=2D-Structure
https://pubchem.ncbi.nlm.nih.gov/compound/136504863#section=2D-Structure
https://pubchem.ncbi.nlm.nih.gov/compound/136504744#section=2D-Structure
https://pubchem.ncbi.nlm.nih.gov/compound/136503742#section=2D-Structure
https://pubchem.ncbi.nlm.nih.gov/compound/136500186#section=2D-Structure
https://pubchem.ncbi.nlm.nih.gov/compound/136499844#section=2D-Structure
https://pubchem.ncbi.nlm.nih.gov/compound/136496397#section=2D-Structure
https://pubchem.ncbi.nlm.nih.gov/compound/136493893#section=2D-Structure
https://pubchem.ncbi.nlm.nih.gov/compound/136491017#section=2D-Structure

https://pubchem.ncbi.nlm.nih.gov/compound/136490419#section=2D-Structure
https://pubchem.ncbi.nlm.nih.gov/compound/136489088#section=2D-Structure
https://pubchem.ncbi.nlm.nih.gov/compound/136488932#section=2D-Structure
https://pubchem.ncbi.nlm.nih.gov/compound/136488464#section=2D-Structure
https://pubchem.ncbi.nlm.nih.gov/compound/136487327#section=2D-Structure
https://pubchem.ncbi.nlm.nih.gov/compound/136486904#section=2D-Structure
https://pubchem.ncbi.nlm.nih.gov/compound/136486578#section=2D-Structure
https://pubchem.ncbi.nlm.nih.gov/compound/136485361#section=2D-Structure
https://pubchem.ncbi.nlm.nih.gov/compound/136485065#section=2D-Structure
https://pubchem.ncbi.nlm.nih.gov/compound/136481867#section=2D-Structure
https://pubchem.ncbi.nlm.nih.gov/compound/136481073#section=2D-Structure
https://pubchem.ncbi.nlm.nih.gov/compound/136479779#section=2D-Structure
https://pubchem.ncbi.nlm.nih.gov/compound/136476773#section=2D-Structure
https://pubchem.ncbi.nlm.nih.gov/compound/136476059#section=2D-Structure
https://pubchem.ncbi.nlm.nih.gov/compound/136474800#section=2D-Structure
https://pubchem.ncbi.nlm.nih.gov/compound/136474151#section=2D-Structure
https://pubchem.ncbi.nlm.nih.gov/compound/136471706#section=2D-Structure
https://pubchem.ncbi.nlm.nih.gov/compound/136469880#section=2D-Structure
https://pubchem.ncbi.nlm.nih.gov/compound/136469827#section=2D-Structure
https://pubchem.ncbi.nlm.nih.gov/compound/136466625#section=2D-Structure
https://pubchem.ncbi.nlm.nih.gov/compound/136465588#section=2D-Structure
https://pubchem.ncbi.nlm.nih.gov/compound/136462953#section=2D-Structure
https://pubchem.ncbi.nlm.nih.gov/compound/136462559#section=2D-Structure
https://pubchem.ncbi.nlm.nih.gov/compound/136462546#section=2D-Structure
https://pubchem.ncbi.nlm.nih.gov/compound/136462461#section=2D-Structure
https://pubchem.ncbi.nlm.nih.gov/compound/136462293#section=2D-Structure
https://pubchem.ncbi.nlm.nih.gov/compound/136460928#section=2D-Structure
https://pubchem.ncbi.nlm.nih.gov/compound/136460920#section=2D-Structure
https://pubchem.ncbi.nlm.nih.gov/compound/136460654#section=2D-Structure
https://pubchem.ncbi.nlm.nih.gov/compound/136459792#section=2D-Structure
https://pubchem.ncbi.nlm.nih.gov/compound/136457849#section=2D-Structure
https://pubchem.ncbi.nlm.nih.gov/compound/136457090#section=2D-Structure
https://pubchem.ncbi.nlm.nih.gov/compound/136456200#section=2D-Structure
https://pubchem.ncbi.nlm.nih.gov/compound/136455735#section=2D-Structure
https://pubchem.ncbi.nlm.nih.gov/compound/136455178#section=2D-Structure
https://pubchem.ncbi.nlm.nih.gov/compound/136454610#section=2D-Structure
https://pubchem.ncbi.nlm.nih.gov/compound/136454146#section=2D-Structure
https://pubchem.ncbi.nlm.nih.gov/compound/136453501#section=2D-Structure
https://pubchem.ncbi.nlm.nih.gov/compound/136453435#section=2D-Structure
https://pubchem.ncbi.nlm.nih.gov/compound/136453422#section=2D-Structure
https://pubchem.ncbi.nlm.nih.gov/compound/136453352#section=2D-Structure
https://pubchem.ncbi.nlm.nih.gov/compound/136452542#section=2D-Structure
https://pubchem.ncbi.nlm.nih.gov/compound/136451900#section=2D-Structure
https://pubchem.ncbi.nlm.nih.gov/compound/136451897#section=2D-Structure
https://pubchem.ncbi.nlm.nih.gov/compound/136450831#section=2D-Structure
https://pubchem.ncbi.nlm.nih.gov/compound/136449960#section=2D-Structure
https://pubchem.ncbi.nlm.nih.gov/compound/136448547#section=2D-Structure
https://pubchem.ncbi.nlm.nih.gov/compound/136447470#section=2D-Structure
https://pubchem.ncbi.nlm.nih.gov/compound/136446505#section=2D-Structure
https://pubchem.ncbi.nlm.nih.gov/compound/136444602#section=2D-Structure
https://pubchem.ncbi.nlm.nih.gov/compound/136444578#section=2D-Structure
https://pubchem.ncbi.nlm.nih.gov/compound/136442669#section=2D-Structure
https://pubchem.ncbi.nlm.nih.gov/compound/136442195#section=2D-Structure
https://pubchem.ncbi.nlm.nih.gov/compound/136441835#section=2D-Structure
https://pubchem.ncbi.nlm.nih.gov/compound/136441268#section=2D-Structure

https://pubchem.ncbi.nlm.nih.gov/compound/136439267#section=2D-Structure
https://pubchem.ncbi.nlm.nih.gov/compound/136438779#section=2D-Structure
https://pubchem.ncbi.nlm.nih.gov/compound/136438348#section=2D-Structure
https://pubchem.ncbi.nlm.nih.gov/compound/136437590#section=2D-Structure
https://pubchem.ncbi.nlm.nih.gov/compound/136437333#section=2D-Structure
https://pubchem.ncbi.nlm.nih.gov/compound/136435358#section=2D-Structure
https://pubchem.ncbi.nlm.nih.gov/compound/136435104#section=2D-Structure
https://pubchem.ncbi.nlm.nih.gov/compound/136434528#section=2D-Structure
https://pubchem.ncbi.nlm.nih.gov/compound/136433062#section=2D-Structure
https://pubchem.ncbi.nlm.nih.gov/compound/136433021#section=2D-Structure
https://pubchem.ncbi.nlm.nih.gov/compound/136432860#section=2D-Structure
https://pubchem.ncbi.nlm.nih.gov/compound/136432084#section=2D-Structure
https://pubchem.ncbi.nlm.nih.gov/compound/136430464#section=2D-Structure
https://pubchem.ncbi.nlm.nih.gov/compound/136429495#section=2D-Structure
https://pubchem.ncbi.nlm.nih.gov/compound/136429132#section=2D-Structure
https://pubchem.ncbi.nlm.nih.gov/compound/136428941#section=2D-Structure
https://pubchem.ncbi.nlm.nih.gov/compound/136427644#section=2D-Structure
https://pubchem.ncbi.nlm.nih.gov/compound/136427427#section=2D-Structure
https://pubchem.ncbi.nlm.nih.gov/compound/136427010#section=2D-Structure
https://pubchem.ncbi.nlm.nih.gov/compound/136425782#section=2D-Structure
https://pubchem.ncbi.nlm.nih.gov/compound/136424166#section=2D-Structure
https://pubchem.ncbi.nlm.nih.gov/compound/136424159#section=2D-Structure
https://pubchem.ncbi.nlm.nih.gov/compound/136424158#section=2D-Structure
https://pubchem.ncbi.nlm.nih.gov/compound/136424154#section=2D-Structure
https://pubchem.ncbi.nlm.nih.gov/compound/136424150#section=2D-Structure
https://pubchem.ncbi.nlm.nih.gov/compound/136424144#section=2D-Structure
https://pubchem.ncbi.nlm.nih.gov/compound/136424136#section=2D-Structure
https://pubchem.ncbi.nlm.nih.gov/compound/136423828#section=2D-Structure
https://pubchem.ncbi.nlm.nih.gov/compound/136422431#section=2D-Structure
https://pubchem.ncbi.nlm.nih.gov/compound/136420053#section=2D-Structure
https://pubchem.ncbi.nlm.nih.gov/compound/136419423#section=2D-Structure
https://pubchem.ncbi.nlm.nih.gov/compound/136418310#section=2D-Structure
https://pubchem.ncbi.nlm.nih.gov/compound/136417501#section=2D-Structure
https://pubchem.ncbi.nlm.nih.gov/compound/136417107#section=2D-Structure
https://pubchem.ncbi.nlm.nih.gov/compound/136416762#section=2D-Structure
https://pubchem.ncbi.nlm.nih.gov/compound/136416477#section=2D-Structure
https://pubchem.ncbi.nlm.nih.gov/compound/136415887#section=2D-Structure
https://pubchem.ncbi.nlm.nih.gov/compound/13641541#section=2D-Structure
https://pubchem.ncbi.nlm.nih.gov/compound/13641540#section=2D-Structure
https://pubchem.ncbi.nlm.nih.gov/compound/136411399#section=2D-Structure
https://pubchem.ncbi.nlm.nih.gov/compound/136411016#section=2D-Structure
https://pubchem.ncbi.nlm.nih.gov/compound/136407962#section=2D-Structure
https://pubchem.ncbi.nlm.nih.gov/compound/136407798#section=2D-Structure
https://pubchem.ncbi.nlm.nih.gov/compound/136405443#section=2D-Structure
https://pubchem.ncbi.nlm.nih.gov/compound/136404999#section=2D-Structure
https://pubchem.ncbi.nlm.nih.gov/compound/136404861#section=2D-Structure
https://pubchem.ncbi.nlm.nih.gov/compound/136404823#section=2D-Structure
https://pubchem.ncbi.nlm.nih.gov/compound/136401146#section=2D-Structure
https://pubchem.ncbi.nlm.nih.gov/compound/136400743#section=2D-Structure
https://pubchem.ncbi.nlm.nih.gov/compound/136400551#section=2D-Structure
https://pubchem.ncbi.nlm.nih.gov/compound/136400364#section=2D-Structure
https://pubchem.ncbi.nlm.nih.gov/compound/13640031#section=2D-Structure
https://pubchem.ncbi.nlm.nih.gov/compound/136399784#section=2D-Structure
https://pubchem.ncbi.nlm.nih.gov/compound/136397751#section=2D-Structure
https://pubchem.ncbi.nlm.nih.gov/compound/136397612#section=2D-Structure

https://pubchem.ncbi.nlm.nih.gov/compound/136396645#section=2D-Structure
https://pubchem.ncbi.nlm.nih.gov/compound/136396412#section=2D-Structure
https://pubchem.ncbi.nlm.nih.gov/compound/136393194#section=2D-Structure
https://pubchem.ncbi.nlm.nih.gov/compound/136391399#section=2D-Structure
https://pubchem.ncbi.nlm.nih.gov/compound/136391345#section=2D-Structure
https://pubchem.ncbi.nlm.nih.gov/compound/136390972#section=2D-Structure
https://pubchem.ncbi.nlm.nih.gov/compound/136390134#section=2D-Structure
https://pubchem.ncbi.nlm.nih.gov/compound/136388341#section=2D-Structure
https://pubchem.ncbi.nlm.nih.gov/compound/136386535#section=2D-Structure
https://pubchem.ncbi.nlm.nih.gov/compound/136386031#section=2D-Structure
https://pubchem.ncbi.nlm.nih.gov/compound/136386030#section=2D-Structure
https://pubchem.ncbi.nlm.nih.gov/compound/13638602#section=2D-Structure
https://pubchem.ncbi.nlm.nih.gov/compound/136385905#section=2D-Structure
https://pubchem.ncbi.nlm.nih.gov/compound/136385438#section=2D-Structure
https://pubchem.ncbi.nlm.nih.gov/compound/136385435#section=2D-Structure
https://pubchem.ncbi.nlm.nih.gov/compound/136385431#section=2D-Structure
https://pubchem.ncbi.nlm.nih.gov/compound/136385428#section=2D-Structure
https://pubchem.ncbi.nlm.nih.gov/compound/136385425#section=2D-Structure
https://pubchem.ncbi.nlm.nih.gov/compound/136385424#section=2D-Structure
https://pubchem.ncbi.nlm.nih.gov/compound/136385421#section=2D-Structure
https://pubchem.ncbi.nlm.nih.gov/compound/136385420#section=2D-Structure
https://pubchem.ncbi.nlm.nih.gov/compound/136385415#section=2D-Structure
https://pubchem.ncbi.nlm.nih.gov/compound/136385414#section=2D-Structure
https://pubchem.ncbi.nlm.nih.gov/compound/136385411#section=2D-Structure
https://pubchem.ncbi.nlm.nih.gov/compound/136385408#section=2D-Structure
https://pubchem.ncbi.nlm.nih.gov/compound/136385406#section=2D-Structure
https://pubchem.ncbi.nlm.nih.gov/compound/136385401#section=2D-Structure
https://pubchem.ncbi.nlm.nih.gov/compound/136385400#section=2D-Structure
https://pubchem.ncbi.nlm.nih.gov/compound/136385397#section=2D-Structure
https://pubchem.ncbi.nlm.nih.gov/compound/136385394#section=2D-Structure
https://pubchem.ncbi.nlm.nih.gov/compound/136385390#section=2D-Structure
https://pubchem.ncbi.nlm.nih.gov/compound/136383945#section=2D-Structure
https://pubchem.ncbi.nlm.nih.gov/compound/136381026#section=2D-Structure
https://pubchem.ncbi.nlm.nih.gov/compound/136380030#section=2D-Structure
https://pubchem.ncbi.nlm.nih.gov/compound/136379967#section=2D-Structure
https://pubchem.ncbi.nlm.nih.gov/compound/136378837#section=2D-Structure
https://pubchem.ncbi.nlm.nih.gov/compound/136378725#section=2D-Structure
https://pubchem.ncbi.nlm.nih.gov/compound/136378042#section=2D-Structure
https://pubchem.ncbi.nlm.nih.gov/compound/136378039#section=2D-Structure
https://pubchem.ncbi.nlm.nih.gov/compound/136377998#section=2D-Structure
https://pubchem.ncbi.nlm.nih.gov/compound/136377980#section=2D-Structure
https://pubchem.ncbi.nlm.nih.gov/compound/136375781#section=2D-Structure
https://pubchem.ncbi.nlm.nih.gov/compound/136375648#section=2D-Structure
https://pubchem.ncbi.nlm.nih.gov/compound/136375554#section=2D-Structure
https://pubchem.ncbi.nlm.nih.gov/compound/136372985#section=2D-Structure
https://pubchem.ncbi.nlm.nih.gov/compound/136372210#section=2D-Structure
https://pubchem.ncbi.nlm.nih.gov/compound/136372209#section=2D-Structure
https://pubchem.ncbi.nlm.nih.gov/compound/136365795#section=2D-Structure
https://pubchem.ncbi.nlm.nih.gov/compound/136365579#section=2D-Structure
https://pubchem.ncbi.nlm.nih.gov/compound/136364081#section=2D-Structure
https://pubchem.ncbi.nlm.nih.gov/compound/136363797#section=2D-Structure
https://pubchem.ncbi.nlm.nih.gov/compound/136360363#section=2D-Structure
https://pubchem.ncbi.nlm.nih.gov/compound/136355348#section=2D-Structure
https://pubchem.ncbi.nlm.nih.gov/compound/136355173#section=2D-Structure
https://pubchem.ncbi.nlm.nih.gov/compound/136353424#section=2D-Structure

https://pubchem.ncbi.nlm.nih.gov/compound/136352165#section=2D-Structure
https://pubchem.ncbi.nlm.nih.gov/compound/136352103#section=2D-Structure
https://pubchem.ncbi.nlm.nih.gov/compound/136351981#section=2D-Structure
https://pubchem.ncbi.nlm.nih.gov/compound/136351980#section=2D-Structure
https://pubchem.ncbi.nlm.nih.gov/compound/136349623#section=2D-Structure
https://pubchem.ncbi.nlm.nih.gov/compound/136347748#section=2D-Structure
https://pubchem.ncbi.nlm.nih.gov/compound/13634653#section=2D-Structure
https://pubchem.ncbi.nlm.nih.gov/compound/136342421#section=2D-Structure
https://pubchem.ncbi.nlm.nih.gov/compound/136341764#section=2D-Structure
https://pubchem.ncbi.nlm.nih.gov/compound/136341680#section=2D-Structure
https://pubchem.ncbi.nlm.nih.gov/compound/136341486#section=2D-Structure
https://pubchem.ncbi.nlm.nih.gov/compound/136341284#section=2D-Structure
https://pubchem.ncbi.nlm.nih.gov/compound/136339032#section=2D-Structure
https://pubchem.ncbi.nlm.nih.gov/compound/136336923#section=2D-Structure
https://pubchem.ncbi.nlm.nih.gov/compound/136336110#section=2D-Structure
https://pubchem.ncbi.nlm.nih.gov/compound/136334540#section=2D-Structure
https://pubchem.ncbi.nlm.nih.gov/compound/136332830#section=2D-Structure
https://pubchem.ncbi.nlm.nih.gov/compound/136331381#section=2D-Structure
https://pubchem.ncbi.nlm.nih.gov/compound/136305571#section=2D-Structure
https://pubchem.ncbi.nlm.nih.gov/compound/136288889#section=2D-Structure
https://pubchem.ncbi.nlm.nih.gov/compound/136275337#section=2D-Structure
https://pubchem.ncbi.nlm.nih.gov/compound/136272986#section=2D-Structure
https://pubchem.ncbi.nlm.nih.gov/compound/136272668#section=2D-Structure
https://pubchem.ncbi.nlm.nih.gov/compound/136272547#section=2D-Structure
https://pubchem.ncbi.nlm.nih.gov/compound/136263025#section=2D-Structure
https://pubchem.ncbi.nlm.nih.gov/compound/136263006#section=2D-Structure
https://pubchem.ncbi.nlm.nih.gov/compound/136262995#section=2D-Structure
https://pubchem.ncbi.nlm.nih.gov/compound/136262972#section=2D-Structure
https://pubchem.ncbi.nlm.nih.gov/compound/136262387#section=2D-Structure
https://pubchem.ncbi.nlm.nih.gov/compound/136249881#section=2D-Structure
https://pubchem.ncbi.nlm.nih.gov/compound/136249040#section=2D-Structure
https://pubchem.ncbi.nlm.nih.gov/compound/136247510#section=2D-Structure
https://pubchem.ncbi.nlm.nih.gov/compound/136247487#section=2D-Structure
https://pubchem.ncbi.nlm.nih.gov/compound/136247352#section=2D-Structure
https://pubchem.ncbi.nlm.nih.gov/compound/136247329#section=2D-Structure
https://pubchem.ncbi.nlm.nih.gov/compound/136242701#section=2D-Structure
https://pubchem.ncbi.nlm.nih.gov/compound/13624229#section=2D-Structure
https://pubchem.ncbi.nlm.nih.gov/compound/136239713#section=2D-Structure
https://pubchem.ncbi.nlm.nih.gov/compound/136238032#section=2D-Structure
https://pubchem.ncbi.nlm.nih.gov/compound/136236424#section=2D-Structure
https://pubchem.ncbi.nlm.nih.gov/compound/136234681#section=2D-Structure
https://pubchem.ncbi.nlm.nih.gov/compound/136230285#section=2D-Structure
https://pubchem.ncbi.nlm.nih.gov/compound/136230214#section=2D-Structure
https://pubchem.ncbi.nlm.nih.gov/compound/136229941#section=2D-Structure
https://pubchem.ncbi.nlm.nih.gov/compound/136229064#section=2D-Structure
https://pubchem.ncbi.nlm.nih.gov/compound/136229062#section=2D-Structure
https://pubchem.ncbi.nlm.nih.gov/compound/136229061#section=2D-Structure
https://pubchem.ncbi.nlm.nih.gov/compound/136227171#section=2D-Structure
https://pubchem.ncbi.nlm.nih.gov/compound/136227161#section=2D-Structure
https://pubchem.ncbi.nlm.nih.gov/compound/136219612#section=2D-Structure
https://pubchem.ncbi.nlm.nih.gov/compound/136209710#section=2D-Structure
https://pubchem.ncbi.nlm.nih.gov/compound/136209572#section=2D-Structure
https://pubchem.ncbi.nlm.nih.gov/compound/136208153#section=2D-Structure
https://pubchem.ncbi.nlm.nih.gov/compound/136207708#section=2D-Structure
https://pubchem.ncbi.nlm.nih.gov/compound/136207384#section=2D-Structure

https://pubchem.ncbi.nlm.nih.gov/compound/136207382#section=2D-Structure
https://pubchem.ncbi.nlm.nih.gov/compound/136197878#section=2D-Structure
https://pubchem.ncbi.nlm.nih.gov/compound/136196939#section=2D-Structure
https://pubchem.ncbi.nlm.nih.gov/compound/136190662#section=2D-Structure
https://pubchem.ncbi.nlm.nih.gov/compound/136188307#section=2D-Structure
https://pubchem.ncbi.nlm.nih.gov/compound/136187769#section=2D-Structure
https://pubchem.ncbi.nlm.nih.gov/compound/136186367#section=2D-Structure
https://pubchem.ncbi.nlm.nih.gov/compound/13617786#section=2D-Structure
https://pubchem.ncbi.nlm.nih.gov/compound/136169345#section=2D-Structure
https://pubchem.ncbi.nlm.nih.gov/compound/136168286#section=2D-Structure
https://pubchem.ncbi.nlm.nih.gov/compound/136165984#section=2D-Structure
https://pubchem.ncbi.nlm.nih.gov/compound/136165983#section=2D-Structure
https://pubchem.ncbi.nlm.nih.gov/compound/136165982#section=2D-Structure
https://pubchem.ncbi.nlm.nih.gov/compound/136161820#section=2D-Structure
https://pubchem.ncbi.nlm.nih.gov/compound/136160751#section=2D-Structure
https://pubchem.ncbi.nlm.nih.gov/compound/136160729#section=2D-Structure
https://pubchem.ncbi.nlm.nih.gov/compound/136160087#section=2D-Structure
https://pubchem.ncbi.nlm.nih.gov/compound/136151633#section=2D-Structure
https://pubchem.ncbi.nlm.nih.gov/compound/136149467#section=2D-Structure
https://pubchem.ncbi.nlm.nih.gov/compound/136147030#section=2D-Structure
https://pubchem.ncbi.nlm.nih.gov/compound/13614111#section=2D-Structure
https://pubchem.ncbi.nlm.nih.gov/compound/136133756#section=2D-Structure
https://pubchem.ncbi.nlm.nih.gov/compound/136129161#section=2D-Structure
https://pubchem.ncbi.nlm.nih.gov/compound/136128659#section=2D-Structure
https://pubchem.ncbi.nlm.nih.gov/compound/136116521#section=2D-Structure
https://pubchem.ncbi.nlm.nih.gov/compound/136114813#section=2D-Structure
https://pubchem.ncbi.nlm.nih.gov/compound/136113999#section=2D-Structure
https://pubchem.ncbi.nlm.nih.gov/compound/136109965#section=2D-Structure
https://pubchem.ncbi.nlm.nih.gov/compound/13610941#section=2D-Structure
https://pubchem.ncbi.nlm.nih.gov/compound/13610939#section=2D-Structure
https://pubchem.ncbi.nlm.nih.gov/compound/136107598#section=2D-Structure
https://pubchem.ncbi.nlm.nih.gov/compound/136103500#section=2D-Structure
https://pubchem.ncbi.nlm.nih.gov/compound/136102339#section=2D-Structure
https://pubchem.ncbi.nlm.nih.gov/compound/136094230#section=2D-Structure
https://pubchem.ncbi.nlm.nih.gov/compound/136094228#section=2D-Structure
https://pubchem.ncbi.nlm.nih.gov/compound/136094227#section=2D-Structure
https://pubchem.ncbi.nlm.nih.gov/compound/136093738#section=2D-Structure
https://pubchem.ncbi.nlm.nih.gov/compound/136093737#section=2D-Structure
https://pubchem.ncbi.nlm.nih.gov/compound/136093736#section=2D-Structure
https://pubchem.ncbi.nlm.nih.gov/compound/136092329#section=2D-Structure
https://pubchem.ncbi.nlm.nih.gov/compound/136092327#section=2D-Structure
https://pubchem.ncbi.nlm.nih.gov/compound/136082991#section=2D-Structure
https://pubchem.ncbi.nlm.nih.gov/compound/136075#section=2D-Structure
https://pubchem.ncbi.nlm.nih.gov/compound/136074644#section=2D-Structure
https://pubchem.ncbi.nlm.nih.gov/compound/136067582#section=2D-Structure
https://pubchem.ncbi.nlm.nih.gov/compound/136065259#section=2D-Structure
https://pubchem.ncbi.nlm.nih.gov/compound/136062990#section=2D-Structure
https://pubchem.ncbi.nlm.nih.gov/compound/136045369#section=2D-Structure
https://pubchem.ncbi.nlm.nih.gov/compound/136041387#section=2D-Structure
https://pubchem.ncbi.nlm.nih.gov/compound/136030477#section=2D-Structure
https://pubchem.ncbi.nlm.nih.gov/compound/136020600#section=2D-Structure
https://pubchem.ncbi.nlm.nih.gov/compound/136020584#section=2D-Structure
https://pubchem.ncbi.nlm.nih.gov/compound/136019020#section=2D-Structure
https://pubchem.ncbi.nlm.nih.gov/compound/136018058#section=2D-Structure
https://pubchem.ncbi.nlm.nih.gov/compound/136017498#section=2D-Structure

https://pubchem.ncbi.nlm.nih.gov/compound/136014045#section=2D-Structure
https://pubchem.ncbi.nlm.nih.gov/compound/136013473#section=2D-Structure
https://pubchem.ncbi.nlm.nih.gov/compound/136013472#section=2D-Structure
https://pubchem.ncbi.nlm.nih.gov/compound/13601070#section=2D-Structure
https://pubchem.ncbi.nlm.nih.gov/compound/136010341#section=2D-Structure
https://pubchem.ncbi.nlm.nih.gov/compound/136006599#section=2D-Structure
https://pubchem.ncbi.nlm.nih.gov/compound/135997673#section=2D-Structure
https://pubchem.ncbi.nlm.nih.gov/compound/135997344#section=2D-Structure
https://pubchem.ncbi.nlm.nih.gov/compound/135996334#section=2D-Structure
https://pubchem.ncbi.nlm.nih.gov/compound/135991669#section=2D-Structure
https://pubchem.ncbi.nlm.nih.gov/compound/135991631#section=2D-Structure
https://pubchem.ncbi.nlm.nih.gov/compound/135985008#section=2D-Structure
https://pubchem.ncbi.nlm.nih.gov/compound/135984325#section=2D-Structure
https://pubchem.ncbi.nlm.nih.gov/compound/135978935#section=2D-Structure
https://pubchem.ncbi.nlm.nih.gov/compound/135966908#section=2D-Structure
https://pubchem.ncbi.nlm.nih.gov/compound/135966713#section=2D-Structure
https://pubchem.ncbi.nlm.nih.gov/compound/135937412#section=2D-Structure
https://pubchem.ncbi.nlm.nih.gov/compound/135899568#section=2D-Structure
https://pubchem.ncbi.nlm.nih.gov/compound/135874025#section=2D-Structure
https://pubchem.ncbi.nlm.nih.gov/compound/135856981#section=2D-Structure
https://pubchem.ncbi.nlm.nih.gov/compound/135839859#section=2D-Structure
https://pubchem.ncbi.nlm.nih.gov/compound/135838397#section=2D-Structure
https://pubchem.ncbi.nlm.nih.gov/compound/135833960#section=2D-Structure
https://pubchem.ncbi.nlm.nih.gov/compound/135833959#section=2D-Structure
https://pubchem.ncbi.nlm.nih.gov/compound/135833871#section=2D-Structure
https://pubchem.ncbi.nlm.nih.gov/compound/135829567#section=2D-Structure
https://pubchem.ncbi.nlm.nih.gov/compound/135829455#section=2D-Structure
https://pubchem.ncbi.nlm.nih.gov/compound/135827499#section=2D-Structure
https://pubchem.ncbi.nlm.nih.gov/compound/135822584#section=2D-Structure
https://pubchem.ncbi.nlm.nih.gov/compound/13581636#section=2D-Structure
https://pubchem.ncbi.nlm.nih.gov/compound/135815288#section=2D-Structure
https://pubchem.ncbi.nlm.nih.gov/compound/135815021#section=2D-Structure
https://pubchem.ncbi.nlm.nih.gov/compound/135813180#section=2D-Structure
https://pubchem.ncbi.nlm.nih.gov/compound/135813177#section=2D-Structure
https://pubchem.ncbi.nlm.nih.gov/compound/135812192#section=2D-Structure
https://pubchem.ncbi.nlm.nih.gov/compound/135802572#section=2D-Structure
https://pubchem.ncbi.nlm.nih.gov/compound/135802571#section=2D-Structure
https://pubchem.ncbi.nlm.nih.gov/compound/135802535#section=2D-Structure
https://pubchem.ncbi.nlm.nih.gov/compound/135798524#section=2D-Structure
https://pubchem.ncbi.nlm.nih.gov/compound/135797574#section=2D-Structure
https://pubchem.ncbi.nlm.nih.gov/compound/135797571#section=2D-Structure
https://pubchem.ncbi.nlm.nih.gov/compound/135797567#section=2D-Structure
https://pubchem.ncbi.nlm.nih.gov/compound/135788037#section=2D-Structure
https://pubchem.ncbi.nlm.nih.gov/compound/135778938#section=2D-Structure
https://pubchem.ncbi.nlm.nih.gov/compound/135778137#section=2D-Structure
https://pubchem.ncbi.nlm.nih.gov/compound/135759471#section=2D-Structure
https://pubchem.ncbi.nlm.nih.gov/compound/135756395#section=2D-Structure
https://pubchem.ncbi.nlm.nih.gov/compound/135755468#section=2D-Structure
https://pubchem.ncbi.nlm.nih.gov/compound/135755392#section=2D-Structure
https://pubchem.ncbi.nlm.nih.gov/compound/135755391#section=2D-Structure
https://pubchem.ncbi.nlm.nih.gov/compound/135753913#section=2D-Structure
https://pubchem.ncbi.nlm.nih.gov/compound/135747057#section=2D-Structure
https://pubchem.ncbi.nlm.nih.gov/compound/135746029#section=2D-Structure
https://pubchem.ncbi.nlm.nih.gov/compound/135745168#section=2D-Structure
https://pubchem.ncbi.nlm.nih.gov/compound/135743680#section=2D-Structure

https://pubchem.ncbi.nlm.nih.gov/compound/135742208#section=2D-Structure
https://pubchem.ncbi.nlm.nih.gov/compound/135742165#section=2D-Structure
https://pubchem.ncbi.nlm.nih.gov/compound/135735532#section=2D-Structure
https://pubchem.ncbi.nlm.nih.gov/compound/135735502#section=2D-Structure
https://pubchem.ncbi.nlm.nih.gov/compound/135729094#section=2D-Structure
https://pubchem.ncbi.nlm.nih.gov/compound/135725029#section=2D-Structure
https://pubchem.ncbi.nlm.nih.gov/compound/135723898#section=2D-Structure
https://pubchem.ncbi.nlm.nih.gov/compound/135723010#section=2D-Structure
https://pubchem.ncbi.nlm.nih.gov/compound/135722048#section=2D-Structure
https://pubchem.ncbi.nlm.nih.gov/compound/135713112#section=2D-Structure
https://pubchem.ncbi.nlm.nih.gov/compound/135708813#section=2D-Structure
https://pubchem.ncbi.nlm.nih.gov/compound/135706827#section=2D-Structure
https://pubchem.ncbi.nlm.nih.gov/compound/135703143#section=2D-Structure
https://pubchem.ncbi.nlm.nih.gov/compound/135700406#section=2D-Structure
https://pubchem.ncbi.nlm.nih.gov/compound/135699201#section=2D-Structure
https://pubchem.ncbi.nlm.nih.gov/compound/135697159#section=2D-Structure
https://pubchem.ncbi.nlm.nih.gov/compound/135697158#section=2D-Structure
https://pubchem.ncbi.nlm.nih.gov/compound/135692518#section=2D-Structure
https://pubchem.ncbi.nlm.nih.gov/compound/135692516#section=2D-Structure
https://pubchem.ncbi.nlm.nih.gov/compound/135690275#section=2D-Structure
https://pubchem.ncbi.nlm.nih.gov/compound/135688034#section=2D-Structure
https://pubchem.ncbi.nlm.nih.gov/compound/135679881#section=2D-Structure
https://pubchem.ncbi.nlm.nih.gov/compound/135675013#section=2D-Structure
https://pubchem.ncbi.nlm.nih.gov/compound/135672041#section=2D-Structure
https://pubchem.ncbi.nlm.nih.gov/compound/135672039#section=2D-Structure
https://pubchem.ncbi.nlm.nih.gov/compound/135665261#section=2D-Structure
https://pubchem.ncbi.nlm.nih.gov/compound/135665145#section=2D-Structure
https://pubchem.ncbi.nlm.nih.gov/compound/135624326#section=2D-Structure
https://pubchem.ncbi.nlm.nih.gov/compound/135623227#section=2D-Structure
https://pubchem.ncbi.nlm.nih.gov/compound/135622989#section=2D-Structure
https://pubchem.ncbi.nlm.nih.gov/compound/135621801#section=2D-Structure
https://pubchem.ncbi.nlm.nih.gov/compound/135611757#section=2D-Structure
https://pubchem.ncbi.nlm.nih.gov/compound/135604505#section=2D-Structure
https://pubchem.ncbi.nlm.nih.gov/compound/135604504#section=2D-Structure
https://pubchem.ncbi.nlm.nih.gov/compound/135600840#section=2D-Structure
https://pubchem.ncbi.nlm.nih.gov/compound/135599173#section=2D-Structure
https://pubchem.ncbi.nlm.nih.gov/compound/135593097#section=2D-Structure
https://pubchem.ncbi.nlm.nih.gov/compound/135593046#section=2D-Structure
https://pubchem.ncbi.nlm.nih.gov/compound/135585305#section=2D-Structure
https://pubchem.ncbi.nlm.nih.gov/compound/135583122#section=2D-Structure
https://pubchem.ncbi.nlm.nih.gov/compound/135583121#section=2D-Structure
https://pubchem.ncbi.nlm.nih.gov/compound/135581587#section=2D-Structure
https://pubchem.ncbi.nlm.nih.gov/compound/135580092#section=2D-Structure
https://pubchem.ncbi.nlm.nih.gov/compound/135576263#section=2D-Structure
https://pubchem.ncbi.nlm.nih.gov/compound/135556879#section=2D-Structure
https://pubchem.ncbi.nlm.nih.gov/compound/135555524#section=2D-Structure
https://pubchem.ncbi.nlm.nih.gov/compound/13554802#section=2D-Structure
https://pubchem.ncbi.nlm.nih.gov/compound/135547003#section=2D-Structure
https://pubchem.ncbi.nlm.nih.gov/compound/135542663#section=2D-Structure
https://pubchem.ncbi.nlm.nih.gov/compound/135542477#section=2D-Structure
https://pubchem.ncbi.nlm.nih.gov/compound/135542401#section=2D-Structure
https://pubchem.ncbi.nlm.nih.gov/compound/135541289#section=2D-Structure
https://pubchem.ncbi.nlm.nih.gov/compound/135537087#section=2D-Structure
https://pubchem.ncbi.nlm.nih.gov/compound/135536529#section=2D-Structure
https://pubchem.ncbi.nlm.nih.gov/compound/135535906#section=2D-Structure

https://pubchem.ncbi.nlm.nih.gov/compound/135533041#section=2D-Structure
https://pubchem.ncbi.nlm.nih.gov/compound/135528319#section=2D-Structure
https://pubchem.ncbi.nlm.nih.gov/compound/135527511#section=2D-Structure
https://pubchem.ncbi.nlm.nih.gov/compound/135527509#section=2D-Structure
https://pubchem.ncbi.nlm.nih.gov/compound/135526953#section=2D-Structure
https://pubchem.ncbi.nlm.nih.gov/compound/135526537#section=2D-Structure
https://pubchem.ncbi.nlm.nih.gov/compound/135525212#section=2D-Structure
https://pubchem.ncbi.nlm.nih.gov/compound/135524974#section=2D-Structure
https://pubchem.ncbi.nlm.nih.gov/compound/13552410#section=2D-Structure
https://pubchem.ncbi.nlm.nih.gov/compound/13552407#section=2D-Structure
https://pubchem.ncbi.nlm.nih.gov/compound/13552399#section=2D-Structure
https://pubchem.ncbi.nlm.nih.gov/compound/135522544#section=2D-Structure
https://pubchem.ncbi.nlm.nih.gov/compound/135522269#section=2D-Structure
https://pubchem.ncbi.nlm.nih.gov/compound/135521950#section=2D-Structure
https://pubchem.ncbi.nlm.nih.gov/compound/135520344#section=2D-Structure
https://pubchem.ncbi.nlm.nih.gov/compound/135506853#section=2D-Structure
https://pubchem.ncbi.nlm.nih.gov/compound/135506754#section=2D-Structure
https://pubchem.ncbi.nlm.nih.gov/compound/135502411#section=2D-Structure
https://pubchem.ncbi.nlm.nih.gov/compound/135492326#section=2D-Structure
https://pubchem.ncbi.nlm.nih.gov/compound/135489814#section=2D-Structure
https://pubchem.ncbi.nlm.nih.gov/compound/135485947#section=2D-Structure
https://pubchem.ncbi.nlm.nih.gov/compound/135485164#section=2D-Structure
https://pubchem.ncbi.nlm.nih.gov/compound/135483971#section=2D-Structure
https://pubchem.ncbi.nlm.nih.gov/compound/135481413#section=2D-Structure
https://pubchem.ncbi.nlm.nih.gov/compound/135476115#section=2D-Structure
https://pubchem.ncbi.nlm.nih.gov/compound/135475514#section=2D-Structure
https://pubchem.ncbi.nlm.nih.gov/compound/135474607#section=2D-Structure
https://pubchem.ncbi.nlm.nih.gov/compound/135473537#section=2D-Structure
https://pubchem.ncbi.nlm.nih.gov/compound/135471325#section=2D-Structure
https://pubchem.ncbi.nlm.nih.gov/compound/135471301#section=2D-Structure
https://pubchem.ncbi.nlm.nih.gov/compound/135470275#section=2D-Structure
https://pubchem.ncbi.nlm.nih.gov/compound/135469297#section=2D-Structure
https://pubchem.ncbi.nlm.nih.gov/compound/135469292#section=2D-Structure
https://pubchem.ncbi.nlm.nih.gov/compound/135468210#section=2D-Structure
https://pubchem.ncbi.nlm.nih.gov/compound/135467695#section=2D-Structure
https://pubchem.ncbi.nlm.nih.gov/compound/135467057#section=2D-Structure
https://pubchem.ncbi.nlm.nih.gov/compound/135464764#section=2D-Structure
https://pubchem.ncbi.nlm.nih.gov/compound/135463794#section=2D-Structure
https://pubchem.ncbi.nlm.nih.gov/compound/135462538#section=2D-Structure
https://pubchem.ncbi.nlm.nih.gov/compound/135457960#section=2D-Structure
https://pubchem.ncbi.nlm.nih.gov/compound/135456836#section=2D-Structure
https://pubchem.ncbi.nlm.nih.gov/compound/135455641#section=2D-Structure
https://pubchem.ncbi.nlm.nih.gov/compound/135455538#section=2D-Structure
https://pubchem.ncbi.nlm.nih.gov/compound/135455537#section=2D-Structure
https://pubchem.ncbi.nlm.nih.gov/compound/135455447#section=2D-Structure
https://pubchem.ncbi.nlm.nih.gov/compound/135450931#section=2D-Structure
https://pubchem.ncbi.nlm.nih.gov/compound/135449570#section=2D-Structure
https://pubchem.ncbi.nlm.nih.gov/compound/135449218#section=2D-Structure
https://pubchem.ncbi.nlm.nih.gov/compound/135449214#section=2D-Structure
https://pubchem.ncbi.nlm.nih.gov/compound/135443984#section=2D-Structure
https://pubchem.ncbi.nlm.nih.gov/compound/135443722#section=2D-Structure
https://pubchem.ncbi.nlm.nih.gov/compound/135443703#section=2D-Structure
https://pubchem.ncbi.nlm.nih.gov/compound/135443574#section=2D-Structure
https://pubchem.ncbi.nlm.nih.gov/compound/135440570#section=2D-Structure
https://pubchem.ncbi.nlm.nih.gov/compound/135432610#section=2D-Structure

https://pubchem.ncbi.nlm.nih.gov/compound/135432339#section=2D-Structure
https://pubchem.ncbi.nlm.nih.gov/compound/135429923#section=2D-Structure
https://pubchem.ncbi.nlm.nih.gov/compound/135427573#section=2D-Structure
https://pubchem.ncbi.nlm.nih.gov/compound/135427114#section=2D-Structure
https://pubchem.ncbi.nlm.nih.gov/compound/135426746#section=2D-Structure
https://pubchem.ncbi.nlm.nih.gov/compound/135426498#section=2D-Structure
https://pubchem.ncbi.nlm.nih.gov/compound/135423601#section=2D-Structure
https://pubchem.ncbi.nlm.nih.gov/compound/135423155#section=2D-Structure
https://pubchem.ncbi.nlm.nih.gov/compound/135413852#section=2D-Structure
https://pubchem.ncbi.nlm.nih.gov/compound/135413511#section=2D-Structure
https://pubchem.ncbi.nlm.nih.gov/compound/135411953#section=2D-Structure
https://pubchem.ncbi.nlm.nih.gov/compound/135411839#section=2D-Structure
https://pubchem.ncbi.nlm.nih.gov/compound/135410202#section=2D-Structure
https://pubchem.ncbi.nlm.nih.gov/compound/135410016#section=2D-Structure
https://pubchem.ncbi.nlm.nih.gov/compound/135407228#section=2D-Structure
https://pubchem.ncbi.nlm.nih.gov/compound/135404296#section=2D-Structure
https://pubchem.ncbi.nlm.nih.gov/compound/135399646#section=2D-Structure
https://pubchem.ncbi.nlm.nih.gov/compound/13539897#section=2D-Structure
https://pubchem.ncbi.nlm.nih.gov/compound/135393619#section=2D-Structure
https://pubchem.ncbi.nlm.nih.gov/compound/135393599#section=2D-Structure
https://pubchem.ncbi.nlm.nih.gov/compound/135393570#section=2D-Structure
https://pubchem.ncbi.nlm.nih.gov/compound/135393554#section=2D-Structure
https://pubchem.ncbi.nlm.nih.gov/compound/135362429#section=2D-Structure
https://pubchem.ncbi.nlm.nih.gov/compound/135338919#section=2D-Structure
https://pubchem.ncbi.nlm.nih.gov/compound/135338910#section=2D-Structure
https://pubchem.ncbi.nlm.nih.gov/compound/135331791#section=2D-Structure
https://pubchem.ncbi.nlm.nih.gov/compound/135324868#section=2D-Structure
https://pubchem.ncbi.nlm.nih.gov/compound/13530913#section=2D-Structure
https://pubchem.ncbi.nlm.nih.gov/compound/135303857#section=2D-Structure
https://pubchem.ncbi.nlm.nih.gov/compound/135277898#section=2D-Structure
https://pubchem.ncbi.nlm.nih.gov/compound/135263668#section=2D-Structure
https://pubchem.ncbi.nlm.nih.gov/compound/135242335#section=2D-Structure
https://pubchem.ncbi.nlm.nih.gov/compound/135234909#section=2D-Structure
https://pubchem.ncbi.nlm.nih.gov/compound/135207337#section=2D-Structure
https://pubchem.ncbi.nlm.nih.gov/compound/135202339#section=2D-Structure
https://pubchem.ncbi.nlm.nih.gov/compound/135201120#section=2D-Structure
https://pubchem.ncbi.nlm.nih.gov/compound/135199196#section=2D-Structure
https://pubchem.ncbi.nlm.nih.gov/compound/135194796#section=2D-Structure
https://pubchem.ncbi.nlm.nih.gov/compound/135194655#section=2D-Structure
https://pubchem.ncbi.nlm.nih.gov/compound/135194294#section=2D-Structure
https://pubchem.ncbi.nlm.nih.gov/compound/135162689#section=2D-Structure
https://pubchem.ncbi.nlm.nih.gov/compound/135159463#section=2D-Structure
https://pubchem.ncbi.nlm.nih.gov/compound/135121990#section=2D-Structure
https://pubchem.ncbi.nlm.nih.gov/compound/135121977#section=2D-Structure
https://pubchem.ncbi.nlm.nih.gov/compound/135084658#section=2D-Structure
https://pubchem.ncbi.nlm.nih.gov/compound/135083124#section=2D-Structure
https://pubchem.ncbi.nlm.nih.gov/compound/135082585#section=2D-Structure
https://pubchem.ncbi.nlm.nih.gov/compound/135082353#section=2D-Structure
https://pubchem.ncbi.nlm.nih.gov/compound/135082282#section=2D-Structure
https://pubchem.ncbi.nlm.nih.gov/compound/135082198#section=2D-Structure
https://pubchem.ncbi.nlm.nih.gov/compound/13507522#section=2D-Structure
https://pubchem.ncbi.nlm.nih.gov/compound/13507520#section=2D-Structure
https://pubchem.ncbi.nlm.nih.gov/compound/135065836#section=2D-Structure
https://pubchem.ncbi.nlm.nih.gov/compound/135060714#section=2D-Structure
https://pubchem.ncbi.nlm.nih.gov/compound/135060361#section=2D-Structure

https://pubchem.ncbi.nlm.nih.gov/compound/135053087#section=2D-Structure
https://pubchem.ncbi.nlm.nih.gov/compound/135052996#section=2D-Structure
https://pubchem.ncbi.nlm.nih.gov/compound/135043592#section=2D-Structure
https://pubchem.ncbi.nlm.nih.gov/compound/135039128#section=2D-Structure
https://pubchem.ncbi.nlm.nih.gov/compound/135030106#section=2D-Structure
https://pubchem.ncbi.nlm.nih.gov/compound/135024590#section=2D-Structure
https://pubchem.ncbi.nlm.nih.gov/compound/135022372#section=2D-Structure
https://pubchem.ncbi.nlm.nih.gov/compound/135015787#section=2D-Structure
https://pubchem.ncbi.nlm.nih.gov/compound/135007954#section=2D-Structure
https://pubchem.ncbi.nlm.nih.gov/compound/135007671#section=2D-Structure
https://pubchem.ncbi.nlm.nih.gov/compound/135007670#section=2D-Structure
https://pubchem.ncbi.nlm.nih.gov/compound/134995238#section=2D-Structure
https://pubchem.ncbi.nlm.nih.gov/compound/134993664#section=2D-Structure
https://pubchem.ncbi.nlm.nih.gov/compound/134990030#section=2D-Structure
https://pubchem.ncbi.nlm.nih.gov/compound/134989678#section=2D-Structure
https://pubchem.ncbi.nlm.nih.gov/compound/134989267#section=2D-Structure
https://pubchem.ncbi.nlm.nih.gov/compound/134987129#section=2D-Structure
https://pubchem.ncbi.nlm.nih.gov/compound/134987032#section=2D-Structure
https://pubchem.ncbi.nlm.nih.gov/compound/134982445#section=2D-Structure
https://pubchem.ncbi.nlm.nih.gov/compound/134976007#section=2D-Structure
https://pubchem.ncbi.nlm.nih.gov/compound/134975787#section=2D-Structure
https://pubchem.ncbi.nlm.nih.gov/compound/134975502#section=2D-Structure
https://pubchem.ncbi.nlm.nih.gov/compound/134972168#section=2D-Structure
https://pubchem.ncbi.nlm.nih.gov/compound/134966537#section=2D-Structure
https://pubchem.ncbi.nlm.nih.gov/compound/134938459#section=2D-Structure
https://pubchem.ncbi.nlm.nih.gov/compound/134937277#section=2D-Structure
https://pubchem.ncbi.nlm.nih.gov/compound/134916223#section=2D-Structure
https://pubchem.ncbi.nlm.nih.gov/compound/134915403#section=2D-Structure
https://pubchem.ncbi.nlm.nih.gov/compound/134914637#section=2D-Structure
https://pubchem.ncbi.nlm.nih.gov/compound/134914384#section=2D-Structure
https://pubchem.ncbi.nlm.nih.gov/compound/134911637#section=2D-Structure
https://pubchem.ncbi.nlm.nih.gov/compound/134909831#section=2D-Structure
https://pubchem.ncbi.nlm.nih.gov/compound/134909544#section=2D-Structure
https://pubchem.ncbi.nlm.nih.gov/compound/134907939#section=2D-Structure
https://pubchem.ncbi.nlm.nih.gov/compound/134891365#section=2D-Structure
https://pubchem.ncbi.nlm.nih.gov/compound/134891347#section=2D-Structure
https://pubchem.ncbi.nlm.nih.gov/compound/134891345#section=2D-Structure
https://pubchem.ncbi.nlm.nih.gov/compound/134891332#section=2D-Structure
https://pubchem.ncbi.nlm.nih.gov/compound/134891313#section=2D-Structure
https://pubchem.ncbi.nlm.nih.gov/compound/134891281#section=2D-Structure
https://pubchem.ncbi.nlm.nih.gov/compound/134890898#section=2D-Structure
https://pubchem.ncbi.nlm.nih.gov/compound/134887819#section=2D-Structure
https://pubchem.ncbi.nlm.nih.gov/compound/134877822#section=2D-Structure
https://pubchem.ncbi.nlm.nih.gov/compound/134874619#section=2D-Structure
https://pubchem.ncbi.nlm.nih.gov/compound/134873956#section=2D-Structure
https://pubchem.ncbi.nlm.nih.gov/compound/13487236#section=2D-Structure
https://pubchem.ncbi.nlm.nih.gov/compound/134870002#section=2D-Structure
https://pubchem.ncbi.nlm.nih.gov/compound/134869031#section=2D-Structure
https://pubchem.ncbi.nlm.nih.gov/compound/134868246#section=2D-Structure
https://pubchem.ncbi.nlm.nih.gov/compound/134868193#section=2D-Structure
https://pubchem.ncbi.nlm.nih.gov/compound/134868141#section=2D-Structure
https://pubchem.ncbi.nlm.nih.gov/compound/134858532#section=2D-Structure
https://pubchem.ncbi.nlm.nih.gov/compound/134858274#section=2D-Structure
https://pubchem.ncbi.nlm.nih.gov/compound/134854275#section=2D-Structure
https://pubchem.ncbi.nlm.nih.gov/compound/13484333#section=2D-Structure

https://pubchem.ncbi.nlm.nih.gov/compound/134837545#section=2D-Structure
https://pubchem.ncbi.nlm.nih.gov/compound/134834968#section=2D-Structure
https://pubchem.ncbi.nlm.nih.gov/compound/134824428#section=2D-Structure
https://pubchem.ncbi.nlm.nih.gov/compound/134822094#section=2D-Structure
https://pubchem.ncbi.nlm.nih.gov/compound/134822056#section=2D-Structure
https://pubchem.ncbi.nlm.nih.gov/compound/134817867#section=2D-Structure
https://pubchem.ncbi.nlm.nih.gov/compound/134817851#section=2D-Structure
https://pubchem.ncbi.nlm.nih.gov/compound/134817831#section=2D-Structure
https://pubchem.ncbi.nlm.nih.gov/compound/134814442#section=2D-Structure
https://pubchem.ncbi.nlm.nih.gov/compound/134813939#section=2D-Structure
https://pubchem.ncbi.nlm.nih.gov/compound/134811500#section=2D-Structure
https://pubchem.ncbi.nlm.nih.gov/compound/134716161#section=2D-Structure
https://pubchem.ncbi.nlm.nih.gov/compound/134715929#section=2D-Structure
https://pubchem.ncbi.nlm.nih.gov/compound/134691338#section=2D-Structure
https://pubchem.ncbi.nlm.nih.gov/compound/13466413#section=2D-Structure
https://pubchem.ncbi.nlm.nih.gov/compound/134612897#section=2D-Structure
https://pubchem.ncbi.nlm.nih.gov/compound/134603965#section=2D-Structure
https://pubchem.ncbi.nlm.nih.gov/compound/13459945#section=2D-Structure
https://pubchem.ncbi.nlm.nih.gov/compound/134593475#section=2D-Structure
https://pubchem.ncbi.nlm.nih.gov/compound/134591790#section=2D-Structure
https://pubchem.ncbi.nlm.nih.gov/compound/134591783#section=2D-Structure
https://pubchem.ncbi.nlm.nih.gov/compound/134572256#section=2D-Structure
https://pubchem.ncbi.nlm.nih.gov/compound/134562732#section=2D-Structure
https://pubchem.ncbi.nlm.nih.gov/compound/134560679#section=2D-Structure
https://pubchem.ncbi.nlm.nih.gov/compound/134560156#section=2D-Structure
https://pubchem.ncbi.nlm.nih.gov/compound/134556520#section=2D-Structure
https://pubchem.ncbi.nlm.nih.gov/compound/134550896#section=2D-Structure
https://pubchem.ncbi.nlm.nih.gov/compound/134528398#section=2D-Structure
https://pubchem.ncbi.nlm.nih.gov/compound/134527509#section=2D-Structure
https://pubchem.ncbi.nlm.nih.gov/compound/134527364#section=2D-Structure
https://pubchem.ncbi.nlm.nih.gov/compound/134526743#section=2D-Structure
https://pubchem.ncbi.nlm.nih.gov/compound/134526719#section=2D-Structure
https://pubchem.ncbi.nlm.nih.gov/compound/134518599#section=2D-Structure
https://pubchem.ncbi.nlm.nih.gov/compound/134518535#section=2D-Structure
https://pubchem.ncbi.nlm.nih.gov/compound/134507693#section=2D-Structure
https://pubchem.ncbi.nlm.nih.gov/compound/134487307#section=2D-Structure
https://pubchem.ncbi.nlm.nih.gov/compound/134485423#section=2D-Structure
https://pubchem.ncbi.nlm.nih.gov/compound/134477393#section=2D-Structure
https://pubchem.ncbi.nlm.nih.gov/compound/134476428#section=2D-Structure
https://pubchem.ncbi.nlm.nih.gov/compound/134474711#section=2D-Structure
https://pubchem.ncbi.nlm.nih.gov/compound/13447424#section=2D-Structure
https://pubchem.ncbi.nlm.nih.gov/compound/134464348#section=2D-Structure
https://pubchem.ncbi.nlm.nih.gov/compound/134451781#section=2D-Structure
https://pubchem.ncbi.nlm.nih.gov/compound/134444198#section=2D-Structure
https://pubchem.ncbi.nlm.nih.gov/compound/134442760#section=2D-Structure
https://pubchem.ncbi.nlm.nih.gov/compound/134437429#section=2D-Structure
https://pubchem.ncbi.nlm.nih.gov/compound/134417912#section=2D-Structure
https://pubchem.ncbi.nlm.nih.gov/compound/134417911#section=2D-Structure
https://pubchem.ncbi.nlm.nih.gov/compound/134409733#section=2D-Structure
https://pubchem.ncbi.nlm.nih.gov/compound/134409660#section=2D-Structure
https://pubchem.ncbi.nlm.nih.gov/compound/134409470#section=2D-Structure
https://pubchem.ncbi.nlm.nih.gov/compound/134387974#section=2D-Structure
https://pubchem.ncbi.nlm.nih.gov/compound/134387821#section=2D-Structure
https://pubchem.ncbi.nlm.nih.gov/compound/134368850#section=2D-Structure
https://pubchem.ncbi.nlm.nih.gov/compound/134368156#section=2D-Structure

https://pubchem.ncbi.nlm.nih.gov/compound/134345717#section=2D-Structure
https://pubchem.ncbi.nlm.nih.gov/compound/13433674#section=2D-Structure
https://pubchem.ncbi.nlm.nih.gov/compound/13433673#section=2D-Structure
https://pubchem.ncbi.nlm.nih.gov/compound/13433623#section=2D-Structure
https://pubchem.ncbi.nlm.nih.gov/compound/134324141#section=2D-Structure
https://pubchem.ncbi.nlm.nih.gov/compound/134324133#section=2D-Structure
https://pubchem.ncbi.nlm.nih.gov/compound/134312142#section=2D-Structure
https://pubchem.ncbi.nlm.nih.gov/compound/134288755#section=2D-Structure
https://pubchem.ncbi.nlm.nih.gov/compound/134278021#section=2D-Structure
https://pubchem.ncbi.nlm.nih.gov/compound/134275057#section=2D-Structure
https://pubchem.ncbi.nlm.nih.gov/compound/134266766#section=2D-Structure
https://pubchem.ncbi.nlm.nih.gov/compound/134239242#section=2D-Structure
https://pubchem.ncbi.nlm.nih.gov/compound/134232094#section=2D-Structure
https://pubchem.ncbi.nlm.nih.gov/compound/134170045#section=2D-Structure
https://pubchem.ncbi.nlm.nih.gov/compound/134158642#section=2D-Structure
https://pubchem.ncbi.nlm.nih.gov/compound/13412123#section=2D-Structure
https://pubchem.ncbi.nlm.nih.gov/compound/134120337#section=2D-Structure
https://pubchem.ncbi.nlm.nih.gov/compound/134118562#section=2D-Structure
https://pubchem.ncbi.nlm.nih.gov/compound/134115119#section=2D-Structure
https://pubchem.ncbi.nlm.nih.gov/compound/13409899#section=2D-Structure
https://pubchem.ncbi.nlm.nih.gov/compound/134095944#section=2D-Structure
https://pubchem.ncbi.nlm.nih.gov/compound/134093764#section=2D-Structure
https://pubchem.ncbi.nlm.nih.gov/compound/134091534#section=2D-Structure
https://pubchem.ncbi.nlm.nih.gov/compound/134088155#section=2D-Structure
https://pubchem.ncbi.nlm.nih.gov/compound/13402040#section=2D-Structure
https://pubchem.ncbi.nlm.nih.gov/compound/13379723#section=2D-Structure
https://pubchem.ncbi.nlm.nih.gov/compound/13373802#section=2D-Structure
https://pubchem.ncbi.nlm.nih.gov/compound/13371714#section=2D-Structure
https://pubchem.ncbi.nlm.nih.gov/compound/133664206#section=2D-Structure
https://pubchem.ncbi.nlm.nih.gov/compound/133633105#section=2D-Structure
https://pubchem.ncbi.nlm.nih.gov/compound/133630749#section=2D-Structure
https://pubchem.ncbi.nlm.nih.gov/compound/133629331#section=2D-Structure
https://pubchem.ncbi.nlm.nih.gov/compound/133628125#section=2D-Structure
https://pubchem.ncbi.nlm.nih.gov/compound/133627804#section=2D-Structure
https://pubchem.ncbi.nlm.nih.gov/compound/133625728#section=2D-Structure
https://pubchem.ncbi.nlm.nih.gov/compound/133616754#section=2D-Structure
https://pubchem.ncbi.nlm.nih.gov/compound/13355482#section=2D-Structure
https://pubchem.ncbi.nlm.nih.gov/compound/13355397#section=2D-Structure
https://pubchem.ncbi.nlm.nih.gov/compound/13349656#section=2D-Structure
https://pubchem.ncbi.nlm.nih.gov/compound/13347915#section=2D-Structure
https://pubchem.ncbi.nlm.nih.gov/compound/13340707#section=2D-Structure
https://pubchem.ncbi.nlm.nih.gov/compound/133084024#section=2D-Structure
https://pubchem.ncbi.nlm.nih.gov/compound/133083759#section=2D-Structure
https://pubchem.ncbi.nlm.nih.gov/compound/133065013#section=2D-Structure
https://pubchem.ncbi.nlm.nih.gov/compound/133064764#section=2D-Structure
https://pubchem.ncbi.nlm.nih.gov/compound/133064668#section=2D-Structure
https://pubchem.ncbi.nlm.nih.gov/compound/133064120#section=2D-Structure
https://pubchem.ncbi.nlm.nih.gov/compound/133054555#section=2D-Structure
https://pubchem.ncbi.nlm.nih.gov/compound/13299747#section=2D-Structure
https://pubchem.ncbi.nlm.nih.gov/compound/132993015#section=2D-Structure
https://pubchem.ncbi.nlm.nih.gov/compound/132991323#section=2D-Structure
https://pubchem.ncbi.nlm.nih.gov/compound/132990696#section=2D-Structure
https://pubchem.ncbi.nlm.nih.gov/compound/132988408#section=2D-Structure
https://pubchem.ncbi.nlm.nih.gov/compound/132968985#section=2D-Structure
https://pubchem.ncbi.nlm.nih.gov/compound/132968983#section=2D-Structure

https://pubchem.ncbi.nlm.nih.gov/compound/13278086#section=2D-Structure
https://pubchem.ncbi.nlm.nih.gov/compound/13260660#section=2D-Structure
https://pubchem.ncbi.nlm.nih.gov/compound/132566763#section=2D-Structure
https://pubchem.ncbi.nlm.nih.gov/compound/132542059#section=2D-Structure
https://pubchem.ncbi.nlm.nih.gov/compound/132513567#section=2D-Structure
https://pubchem.ncbi.nlm.nih.gov/compound/132512221#section=2D-Structure
https://pubchem.ncbi.nlm.nih.gov/compound/132506855#section=2D-Structure
https://pubchem.ncbi.nlm.nih.gov/compound/13250029#section=2D-Structure
https://pubchem.ncbi.nlm.nih.gov/compound/132499429#section=2D-Structure
https://pubchem.ncbi.nlm.nih.gov/compound/132493858#section=2D-Structure
https://pubchem.ncbi.nlm.nih.gov/compound/132470850#section=2D-Structure
https://pubchem.ncbi.nlm.nih.gov/compound/132469950#section=2D-Structure
https://pubchem.ncbi.nlm.nih.gov/compound/132468865#section=2D-Structure
https://pubchem.ncbi.nlm.nih.gov/compound/132459578#section=2D-Structure
https://pubchem.ncbi.nlm.nih.gov/compound/13244324#section=2D-Structure
https://pubchem.ncbi.nlm.nih.gov/compound/132427542#section=2D-Structure
https://pubchem.ncbi.nlm.nih.gov/compound/132427244#section=2D-Structure
https://pubchem.ncbi.nlm.nih.gov/compound/13230862#section=2D-Structure
https://pubchem.ncbi.nlm.nih.gov/compound/132281750#section=2D-Structure
https://pubchem.ncbi.nlm.nih.gov/compound/132281377#section=2D-Structure
https://pubchem.ncbi.nlm.nih.gov/compound/132281278#section=2D-Structure
https://pubchem.ncbi.nlm.nih.gov/compound/132281239#section=2D-Structure
https://pubchem.ncbi.nlm.nih.gov/compound/132280651#section=2D-Structure
https://pubchem.ncbi.nlm.nih.gov/compound/132279932#section=2D-Structure
https://pubchem.ncbi.nlm.nih.gov/compound/132278978#section=2D-Structure
https://pubchem.ncbi.nlm.nih.gov/compound/132278794#section=2D-Structure
https://pubchem.ncbi.nlm.nih.gov/compound/132278747#section=2D-Structure
https://pubchem.ncbi.nlm.nih.gov/compound/132277995#section=2D-Structure
https://pubchem.ncbi.nlm.nih.gov/compound/132277487#section=2D-Structure
https://pubchem.ncbi.nlm.nih.gov/compound/132277305#section=2D-Structure
https://pubchem.ncbi.nlm.nih.gov/compound/132277157#section=2D-Structure
https://pubchem.ncbi.nlm.nih.gov/compound/132264767#section=2D-Structure
https://pubchem.ncbi.nlm.nih.gov/compound/132264762#section=2D-Structure
https://pubchem.ncbi.nlm.nih.gov/compound/132264680#section=2D-Structure
https://pubchem.ncbi.nlm.nih.gov/compound/132264652#section=2D-Structure
https://pubchem.ncbi.nlm.nih.gov/compound/132264405#section=2D-Structure
https://pubchem.ncbi.nlm.nih.gov/compound/132241380#section=2D-Structure
https://pubchem.ncbi.nlm.nih.gov/compound/132236115#section=2D-Structure
https://pubchem.ncbi.nlm.nih.gov/compound/13221214#section=2D-Structure
https://pubchem.ncbi.nlm.nih.gov/compound/132196698#section=2D-Structure
https://pubchem.ncbi.nlm.nih.gov/compound/132195673#section=2D-Structure
https://pubchem.ncbi.nlm.nih.gov/compound/132191674#section=2D-Structure
https://pubchem.ncbi.nlm.nih.gov/compound/132179086#section=2D-Structure
https://pubchem.ncbi.nlm.nih.gov/compound/132176449#section=2D-Structure
https://pubchem.ncbi.nlm.nih.gov/compound/132151204#section=2D-Structure
https://pubchem.ncbi.nlm.nih.gov/compound/132132547#section=2D-Structure
https://pubchem.ncbi.nlm.nih.gov/compound/132127056#section=2D-Structure
https://pubchem.ncbi.nlm.nih.gov/compound/132126920#section=2D-Structure
https://pubchem.ncbi.nlm.nih.gov/compound/132125566#section=2D-Structure
https://pubchem.ncbi.nlm.nih.gov/compound/132125459#section=2D-Structure
https://pubchem.ncbi.nlm.nih.gov/compound/132124876#section=2D-Structure
https://pubchem.ncbi.nlm.nih.gov/compound/132120474#section=2D-Structure
https://pubchem.ncbi.nlm.nih.gov/compound/13211065#section=2D-Structure
https://pubchem.ncbi.nlm.nih.gov/compound/132104010#section=2D-Structure
https://pubchem.ncbi.nlm.nih.gov/compound/132096006#section=2D-Structure

https://pubchem.ncbi.nlm.nih.gov/compound/13208428#section=2D-Structure
https://pubchem.ncbi.nlm.nih.gov/compound/132081343#section=2D-Structure
https://pubchem.ncbi.nlm.nih.gov/compound/132077787#section=2D-Structure
https://pubchem.ncbi.nlm.nih.gov/compound/132066218#section=2D-Structure
https://pubchem.ncbi.nlm.nih.gov/compound/132066031#section=2D-Structure
https://pubchem.ncbi.nlm.nih.gov/compound/132064535#section=2D-Structure
https://pubchem.ncbi.nlm.nih.gov/compound/132058500#section=2D-Structure
https://pubchem.ncbi.nlm.nih.gov/compound/132058116#section=2D-Structure
https://pubchem.ncbi.nlm.nih.gov/compound/132054053#section=2D-Structure
https://pubchem.ncbi.nlm.nih.gov/compound/132018#section=2D-Structure
https://pubchem.ncbi.nlm.nih.gov/compound/13199307#section=2D-Structure
https://pubchem.ncbi.nlm.nih.gov/compound/131992989#section=2D-Structure
https://pubchem.ncbi.nlm.nih.gov/compound/131987079#section=2D-Structure
https://pubchem.ncbi.nlm.nih.gov/compound/131978162#section=2D-Structure
https://pubchem.ncbi.nlm.nih.gov/compound/131966673#section=2D-Structure
https://pubchem.ncbi.nlm.nih.gov/compound/131966672#section=2D-Structure
https://pubchem.ncbi.nlm.nih.gov/compound/131966634#section=2D-Structure
https://pubchem.ncbi.nlm.nih.gov/compound/131876770#section=2D-Structure
https://pubchem.ncbi.nlm.nih.gov/compound/131864177#section=2D-Structure
https://pubchem.ncbi.nlm.nih.gov/compound/131860159#section=2D-Structure
https://pubchem.ncbi.nlm.nih.gov/compound/131857489#section=2D-Structure
https://pubchem.ncbi.nlm.nih.gov/compound/131857177#section=2D-Structure
https://pubchem.ncbi.nlm.nih.gov/compound/131855332#section=2D-Structure
https://pubchem.ncbi.nlm.nih.gov/compound/131853888#section=2D-Structure
https://pubchem.ncbi.nlm.nih.gov/compound/131852954#section=2D-Structure
https://pubchem.ncbi.nlm.nih.gov/compound/131852921#section=2D-Structure
https://pubchem.ncbi.nlm.nih.gov/compound/131852885#section=2D-Structure
https://pubchem.ncbi.nlm.nih.gov/compound/131852399#section=2D-Structure
https://pubchem.ncbi.nlm.nih.gov/compound/131852376#section=2D-Structure
https://pubchem.ncbi.nlm.nih.gov/compound/131851021#section=2D-Structure
https://pubchem.ncbi.nlm.nih.gov/compound/131849717#section=2D-Structure
https://pubchem.ncbi.nlm.nih.gov/compound/131847326#section=2D-Structure
https://pubchem.ncbi.nlm.nih.gov/compound/131845929#section=2D-Structure
https://pubchem.ncbi.nlm.nih.gov/compound/131845124#section=2D-Structure
https://pubchem.ncbi.nlm.nih.gov/compound/131843938#section=2D-Structure
https://pubchem.ncbi.nlm.nih.gov/compound/131841864#section=2D-Structure
https://pubchem.ncbi.nlm.nih.gov/compound/13171767#section=2D-Structure
https://pubchem.ncbi.nlm.nih.gov/compound/131716649#section=2D-Structure
https://pubchem.ncbi.nlm.nih.gov/compound/131698510#section=2D-Structure
https://pubchem.ncbi.nlm.nih.gov/compound/131614474#section=2D-Structure
https://pubchem.ncbi.nlm.nih.gov/compound/131612912#section=2D-Structure
https://pubchem.ncbi.nlm.nih.gov/compound/13160798#section=2D-Structure
https://pubchem.ncbi.nlm.nih.gov/compound/131590146#section=2D-Structure
https://pubchem.ncbi.nlm.nih.gov/compound/131583050#section=2D-Structure
https://pubchem.ncbi.nlm.nih.gov/compound/131576542#section=2D-Structure
https://pubchem.ncbi.nlm.nih.gov/compound/131576541#section=2D-Structure
https://pubchem.ncbi.nlm.nih.gov/compound/13156884#section=2D-Structure
https://pubchem.ncbi.nlm.nih.gov/compound/13156502#section=2D-Structure
https://pubchem.ncbi.nlm.nih.gov/compound/13156501#section=2D-Structure
https://pubchem.ncbi.nlm.nih.gov/compound/13156496#section=2D-Structure
https://pubchem.ncbi.nlm.nih.gov/compound/131558210#section=2D-Structure
https://pubchem.ncbi.nlm.nih.gov/compound/131553098#section=2D-Structure
https://pubchem.ncbi.nlm.nih.gov/compound/131552221#section=2D-Structure
https://pubchem.ncbi.nlm.nih.gov/compound/131541414#section=2D-Structure
https://pubchem.ncbi.nlm.nih.gov/compound/131511647#section=2D-Structure

https://pubchem.ncbi.nlm.nih.gov/compound/131496835#section=2D-Structure
https://pubchem.ncbi.nlm.nih.gov/compound/131493130#section=2D-Structure
https://pubchem.ncbi.nlm.nih.gov/compound/131480959#section=2D-Structure
https://pubchem.ncbi.nlm.nih.gov/compound/13145399#section=2D-Structure
https://pubchem.ncbi.nlm.nih.gov/compound/131452284#section=2D-Structure
https://pubchem.ncbi.nlm.nih.gov/compound/131448661#section=2D-Structure
https://pubchem.ncbi.nlm.nih.gov/compound/131441105#section=2D-Structure
https://pubchem.ncbi.nlm.nih.gov/compound/131438836#section=2D-Structure
https://pubchem.ncbi.nlm.nih.gov/compound/131430030#section=2D-Structure
https://pubchem.ncbi.nlm.nih.gov/compound/131428339#section=2D-Structure
https://pubchem.ncbi.nlm.nih.gov/compound/131428333#section=2D-Structure
https://pubchem.ncbi.nlm.nih.gov/compound/131425012#section=2D-Structure
https://pubchem.ncbi.nlm.nih.gov/compound/131423490#section=2D-Structure
https://pubchem.ncbi.nlm.nih.gov/compound/131421209#section=2D-Structure
https://pubchem.ncbi.nlm.nih.gov/compound/131420890#section=2D-Structure
https://pubchem.ncbi.nlm.nih.gov/compound/131419812#section=2D-Structure
https://pubchem.ncbi.nlm.nih.gov/compound/131407647#section=2D-Structure
https://pubchem.ncbi.nlm.nih.gov/compound/131400190#section=2D-Structure
https://pubchem.ncbi.nlm.nih.gov/compound/131400189#section=2D-Structure
https://pubchem.ncbi.nlm.nih.gov/compound/131391306#section=2D-Structure
https://pubchem.ncbi.nlm.nih.gov/compound/131383047#section=2D-Structure
https://pubchem.ncbi.nlm.nih.gov/compound/131359644#section=2D-Structure
https://pubchem.ncbi.nlm.nih.gov/compound/13133814#section=2D-Structure
https://pubchem.ncbi.nlm.nih.gov/compound/131332404#section=2D-Structure
https://pubchem.ncbi.nlm.nih.gov/compound/131331377#section=2D-Structure
https://pubchem.ncbi.nlm.nih.gov/compound/131330281#section=2D-Structure
https://pubchem.ncbi.nlm.nih.gov/compound/131328403#section=2D-Structure
https://pubchem.ncbi.nlm.nih.gov/compound/131326085#section=2D-Structure
https://pubchem.ncbi.nlm.nih.gov/compound/131324418#section=2D-Structure
https://pubchem.ncbi.nlm.nih.gov/compound/131294224#section=2D-Structure
https://pubchem.ncbi.nlm.nih.gov/compound/131283762#section=2D-Structure
https://pubchem.ncbi.nlm.nih.gov/compound/131277132#section=2D-Structure
https://pubchem.ncbi.nlm.nih.gov/compound/131265805#section=2D-Structure
https://pubchem.ncbi.nlm.nih.gov/compound/131257574#section=2D-Structure
https://pubchem.ncbi.nlm.nih.gov/compound/131256812#section=2D-Structure
https://pubchem.ncbi.nlm.nih.gov/compound/131255743#section=2D-Structure
https://pubchem.ncbi.nlm.nih.gov/compound/131255740#section=2D-Structure
https://pubchem.ncbi.nlm.nih.gov/compound/131254137#section=2D-Structure
https://pubchem.ncbi.nlm.nih.gov/compound/131241532#section=2D-Structure
https://pubchem.ncbi.nlm.nih.gov/compound/131240771#section=2D-Structure
https://pubchem.ncbi.nlm.nih.gov/compound/13119069#section=2D-Structure
https://pubchem.ncbi.nlm.nih.gov/compound/131190531#section=2D-Structure
https://pubchem.ncbi.nlm.nih.gov/compound/131187641#section=2D-Structure
https://pubchem.ncbi.nlm.nih.gov/compound/131182172#section=2D-Structure
https://pubchem.ncbi.nlm.nih.gov/compound/131166813#section=2D-Structure
https://pubchem.ncbi.nlm.nih.gov/compound/13115664#section=2D-Structure
https://pubchem.ncbi.nlm.nih.gov/compound/13115663#section=2D-Structure
https://pubchem.ncbi.nlm.nih.gov/compound/131154404#section=2D-Structure
https://pubchem.ncbi.nlm.nih.gov/compound/131152720#section=2D-Structure
https://pubchem.ncbi.nlm.nih.gov/compound/131151122#section=2D-Structure
https://pubchem.ncbi.nlm.nih.gov/compound/131127729#section=2D-Structure
https://pubchem.ncbi.nlm.nih.gov/compound/131125575#section=2D-Structure
https://pubchem.ncbi.nlm.nih.gov/compound/131093852#section=2D-Structure
https://pubchem.ncbi.nlm.nih.gov/compound/131082812#section=2D-Structure
https://pubchem.ncbi.nlm.nih.gov/compound/131082531#section=2D-Structure

https://pubchem.ncbi.nlm.nih.gov/compound/131068248#section=2D-Structure
https://pubchem.ncbi.nlm.nih.gov/compound/131058237#section=2D-Structure
https://pubchem.ncbi.nlm.nih.gov/compound/131049235#section=2D-Structure
https://pubchem.ncbi.nlm.nih.gov/compound/131028647#section=2D-Structure
https://pubchem.ncbi.nlm.nih.gov/compound/131027885#section=2D-Structure
https://pubchem.ncbi.nlm.nih.gov/compound/131010308#section=2D-Structure
https://pubchem.ncbi.nlm.nih.gov/compound/130982352#section=2D-Structure
https://pubchem.ncbi.nlm.nih.gov/compound/130956024#section=2D-Structure
https://pubchem.ncbi.nlm.nih.gov/compound/13095460#section=2D-Structure
https://pubchem.ncbi.nlm.nih.gov/compound/130934822#section=2D-Structure
https://pubchem.ncbi.nlm.nih.gov/compound/130906148#section=2D-Structure
https://pubchem.ncbi.nlm.nih.gov/compound/130867210#section=2D-Structure
https://pubchem.ncbi.nlm.nih.gov/compound/130864962#section=2D-Structure
https://pubchem.ncbi.nlm.nih.gov/compound/13082872#section=2D-Structure
https://pubchem.ncbi.nlm.nih.gov/compound/130822482#section=2D-Structure
https://pubchem.ncbi.nlm.nih.gov/compound/130786720#section=2D-Structure
https://pubchem.ncbi.nlm.nih.gov/compound/130784950#section=2D-Structure
https://pubchem.ncbi.nlm.nih.gov/compound/13077698#section=2D-Structure
https://pubchem.ncbi.nlm.nih.gov/compound/130770695#section=2D-Structure
https://pubchem.ncbi.nlm.nih.gov/compound/130764236#section=2D-Structure
https://pubchem.ncbi.nlm.nih.gov/compound/13074536#section=2D-Structure
https://pubchem.ncbi.nlm.nih.gov/compound/13074534#section=2D-Structure
https://pubchem.ncbi.nlm.nih.gov/compound/130740385#section=2D-Structure
https://pubchem.ncbi.nlm.nih.gov/compound/130737261#section=2D-Structure
https://pubchem.ncbi.nlm.nih.gov/compound/130696626#section=2D-Structure
https://pubchem.ncbi.nlm.nih.gov/compound/13069568#section=2D-Structure
https://pubchem.ncbi.nlm.nih.gov/compound/130688936#section=2D-Structure
https://pubchem.ncbi.nlm.nih.gov/compound/130672699#section=2D-Structure
https://pubchem.ncbi.nlm.nih.gov/compound/13065888#section=2D-Structure
https://pubchem.ncbi.nlm.nih.gov/compound/130656515#section=2D-Structure
https://pubchem.ncbi.nlm.nih.gov/compound/130651411#section=2D-Structure
https://pubchem.ncbi.nlm.nih.gov/compound/130649170#section=2D-Structure
https://pubchem.ncbi.nlm.nih.gov/compound/130639565#section=2D-Structure
https://pubchem.ncbi.nlm.nih.gov/compound/130455339#section=2D-Structure
https://pubchem.ncbi.nlm.nih.gov/compound/130442464#section=2D-Structure
https://pubchem.ncbi.nlm.nih.gov/compound/130436639#section=2D-Structure
https://pubchem.ncbi.nlm.nih.gov/compound/130432924#section=2D-Structure
https://pubchem.ncbi.nlm.nih.gov/compound/130425400#section=2D-Structure
https://pubchem.ncbi.nlm.nih.gov/compound/13040808#section=2D-Structure
https://pubchem.ncbi.nlm.nih.gov/compound/13040711#section=2D-Structure
https://pubchem.ncbi.nlm.nih.gov/compound/130394832#section=2D-Structure
https://pubchem.ncbi.nlm.nih.gov/compound/130392240#section=2D-Structure
https://pubchem.ncbi.nlm.nih.gov/compound/130388999#section=2D-Structure
https://pubchem.ncbi.nlm.nih.gov/compound/130382356#section=2D-Structure
https://pubchem.ncbi.nlm.nih.gov/compound/130370241#section=2D-Structure
https://pubchem.ncbi.nlm.nih.gov/compound/130354798#section=2D-Structure
https://pubchem.ncbi.nlm.nih.gov/compound/130349644#section=2D-Structure
https://pubchem.ncbi.nlm.nih.gov/compound/130348737#section=2D-Structure
https://pubchem.ncbi.nlm.nih.gov/compound/130332546#section=2D-Structure
https://pubchem.ncbi.nlm.nih.gov/compound/130321653#section=2D-Structure
https://pubchem.ncbi.nlm.nih.gov/compound/130316652#section=2D-Structure
https://pubchem.ncbi.nlm.nih.gov/compound/130296904#section=2D-Structure
https://pubchem.ncbi.nlm.nih.gov/compound/130294885#section=2D-Structure
https://pubchem.ncbi.nlm.nih.gov/compound/130294784#section=2D-Structure
https://pubchem.ncbi.nlm.nih.gov/compound/130294570#section=2D-Structure

https://pubchem.ncbi.nlm.nih.gov/compound/130291489#section=2D-Structure
https://pubchem.ncbi.nlm.nih.gov/compound/130290940#section=2D-Structure
https://pubchem.ncbi.nlm.nih.gov/compound/130290834#section=2D-Structure
https://pubchem.ncbi.nlm.nih.gov/compound/130280690#section=2D-Structure
https://pubchem.ncbi.nlm.nih.gov/compound/13027553#section=2D-Structure
https://pubchem.ncbi.nlm.nih.gov/compound/130244593#section=2D-Structure
https://pubchem.ncbi.nlm.nih.gov/compound/130243348#section=2D-Structure
https://pubchem.ncbi.nlm.nih.gov/compound/130241843#section=2D-Structure
https://pubchem.ncbi.nlm.nih.gov/compound/130238843#section=2D-Structure
https://pubchem.ncbi.nlm.nih.gov/compound/130233380#section=2D-Structure
https://pubchem.ncbi.nlm.nih.gov/compound/130231293#section=2D-Structure
https://pubchem.ncbi.nlm.nih.gov/compound/130224942#section=2D-Structure
https://pubchem.ncbi.nlm.nih.gov/compound/130223711#section=2D-Structure
https://pubchem.ncbi.nlm.nih.gov/compound/130221277#section=2D-Structure
https://pubchem.ncbi.nlm.nih.gov/compound/130219415#section=2D-Structure
https://pubchem.ncbi.nlm.nih.gov/compound/130219156#section=2D-Structure
https://pubchem.ncbi.nlm.nih.gov/compound/13021008#section=2D-Structure
https://pubchem.ncbi.nlm.nih.gov/compound/130209575#section=2D-Structure
https://pubchem.ncbi.nlm.nih.gov/compound/130209574#section=2D-Structure
https://pubchem.ncbi.nlm.nih.gov/compound/130209557#section=2D-Structure
https://pubchem.ncbi.nlm.nih.gov/compound/130209556#section=2D-Structure
https://pubchem.ncbi.nlm.nih.gov/compound/130209555#section=2D-Structure
https://pubchem.ncbi.nlm.nih.gov/compound/130209554#section=2D-Structure
https://pubchem.ncbi.nlm.nih.gov/compound/130209553#section=2D-Structure
https://pubchem.ncbi.nlm.nih.gov/compound/130209552#section=2D-Structure
https://pubchem.ncbi.nlm.nih.gov/compound/130209551#section=2D-Structure
https://pubchem.ncbi.nlm.nih.gov/compound/130209548#section=2D-Structure
https://pubchem.ncbi.nlm.nih.gov/compound/130209547#section=2D-Structure
https://pubchem.ncbi.nlm.nih.gov/compound/130209544#section=2D-Structure
https://pubchem.ncbi.nlm.nih.gov/compound/130209543#section=2D-Structure
https://pubchem.ncbi.nlm.nih.gov/compound/130207751#section=2D-Structure
https://pubchem.ncbi.nlm.nih.gov/compound/130205651#section=2D-Structure
https://pubchem.ncbi.nlm.nih.gov/compound/130182476#section=2D-Structure
https://pubchem.ncbi.nlm.nih.gov/compound/130173126#section=2D-Structure
https://pubchem.ncbi.nlm.nih.gov/compound/130172070#section=2D-Structure
https://pubchem.ncbi.nlm.nih.gov/compound/130170190#section=2D-Structure
https://pubchem.ncbi.nlm.nih.gov/compound/130168530#section=2D-Structure
https://pubchem.ncbi.nlm.nih.gov/compound/130168525#section=2D-Structure
https://pubchem.ncbi.nlm.nih.gov/compound/130168519#section=2D-Structure
https://pubchem.ncbi.nlm.nih.gov/compound/130167181#section=2D-Structure
https://pubchem.ncbi.nlm.nih.gov/compound/130167180#section=2D-Structure
https://pubchem.ncbi.nlm.nih.gov/compound/130166303#section=2D-Structure
https://pubchem.ncbi.nlm.nih.gov/compound/130164372#section=2D-Structure
https://pubchem.ncbi.nlm.nih.gov/compound/130164369#section=2D-Structure
https://pubchem.ncbi.nlm.nih.gov/compound/130164359#section=2D-Structure
https://pubchem.ncbi.nlm.nih.gov/compound/130162433#section=2D-Structure
https://pubchem.ncbi.nlm.nih.gov/compound/130162432#section=2D-Structure
https://pubchem.ncbi.nlm.nih.gov/compound/130160511#section=2D-Structure
https://pubchem.ncbi.nlm.nih.gov/compound/130160300#section=2D-Structure
https://pubchem.ncbi.nlm.nih.gov/compound/130160244#section=2D-Structure
https://pubchem.ncbi.nlm.nih.gov/compound/130159804#section=2D-Structure
https://pubchem.ncbi.nlm.nih.gov/compound/130159803#section=2D-Structure
https://pubchem.ncbi.nlm.nih.gov/compound/130159791#section=2D-Structure
https://pubchem.ncbi.nlm.nih.gov/compound/130159789#section=2D-Structure
https://pubchem.ncbi.nlm.nih.gov/compound/130159788#section=2D-Structure

https://pubchem.ncbi.nlm.nih.gov/compound/130158424#section=2D-Structure
https://pubchem.ncbi.nlm.nih.gov/compound/130157566#section=2D-Structure
https://pubchem.ncbi.nlm.nih.gov/compound/130157551#section=2D-Structure
https://pubchem.ncbi.nlm.nih.gov/compound/130157320#section=2D-Structure
https://pubchem.ncbi.nlm.nih.gov/compound/130156912#section=2D-Structure
https://pubchem.ncbi.nlm.nih.gov/compound/130150838#section=2D-Structure
https://pubchem.ncbi.nlm.nih.gov/compound/130149762#section=2D-Structure
https://pubchem.ncbi.nlm.nih.gov/compound/130149019#section=2D-Structure
https://pubchem.ncbi.nlm.nih.gov/compound/130147811#section=2D-Structure
https://pubchem.ncbi.nlm.nih.gov/compound/130146281#section=2D-Structure
https://pubchem.ncbi.nlm.nih.gov/compound/130145114#section=2D-Structure
https://pubchem.ncbi.nlm.nih.gov/compound/130143711#section=2D-Structure
https://pubchem.ncbi.nlm.nih.gov/compound/130142672#section=2D-Structure
https://pubchem.ncbi.nlm.nih.gov/compound/130141360#section=2D-Structure
https://pubchem.ncbi.nlm.nih.gov/compound/130141336#section=2D-Structure
https://pubchem.ncbi.nlm.nih.gov/compound/130141332#section=2D-Structure
https://pubchem.ncbi.nlm.nih.gov/compound/130141325#section=2D-Structure
https://pubchem.ncbi.nlm.nih.gov/compound/130141324#section=2D-Structure
https://pubchem.ncbi.nlm.nih.gov/compound/130141312#section=2D-Structure
https://pubchem.ncbi.nlm.nih.gov/compound/130141310#section=2D-Structure
https://pubchem.ncbi.nlm.nih.gov/compound/130141297#section=2D-Structure
https://pubchem.ncbi.nlm.nih.gov/compound/130141287#section=2D-Structure
https://pubchem.ncbi.nlm.nih.gov/compound/130141283#section=2D-Structure
https://pubchem.ncbi.nlm.nih.gov/compound/130141281#section=2D-Structure
https://pubchem.ncbi.nlm.nih.gov/compound/130141242#section=2D-Structure
https://pubchem.ncbi.nlm.nih.gov/compound/130141220#section=2D-Structure
https://pubchem.ncbi.nlm.nih.gov/compound/130141210#section=2D-Structure
https://pubchem.ncbi.nlm.nih.gov/compound/130141202#section=2D-Structure
https://pubchem.ncbi.nlm.nih.gov/compound/130141020#section=2D-Structure
https://pubchem.ncbi.nlm.nih.gov/compound/130136223#section=2D-Structure
https://pubchem.ncbi.nlm.nih.gov/compound/130129447#section=2D-Structure
https://pubchem.ncbi.nlm.nih.gov/compound/130129368#section=2D-Structure
https://pubchem.ncbi.nlm.nih.gov/compound/130123751#section=2D-Structure
https://pubchem.ncbi.nlm.nih.gov/compound/130120472#section=2D-Structure
https://pubchem.ncbi.nlm.nih.gov/compound/130120439#section=2D-Structure
https://pubchem.ncbi.nlm.nih.gov/compound/130120177#section=2D-Structure
https://pubchem.ncbi.nlm.nih.gov/compound/130120155#section=2D-Structure
https://pubchem.ncbi.nlm.nih.gov/compound/130119879#section=2D-Structure
https://pubchem.ncbi.nlm.nih.gov/compound/130119316#section=2D-Structure
https://pubchem.ncbi.nlm.nih.gov/compound/130117145#section=2D-Structure
https://pubchem.ncbi.nlm.nih.gov/compound/130117144#section=2D-Structure
https://pubchem.ncbi.nlm.nih.gov/compound/130117142#section=2D-Structure
https://pubchem.ncbi.nlm.nih.gov/compound/130117118#section=2D-Structure
https://pubchem.ncbi.nlm.nih.gov/compound/130117117#section=2D-Structure
https://pubchem.ncbi.nlm.nih.gov/compound/130117043#section=2D-Structure
https://pubchem.ncbi.nlm.nih.gov/compound/130116581#section=2D-Structure
https://pubchem.ncbi.nlm.nih.gov/compound/130116579#section=2D-Structure
https://pubchem.ncbi.nlm.nih.gov/compound/130115988#section=2D-Structure
https://pubchem.ncbi.nlm.nih.gov/compound/130115987#section=2D-Structure
https://pubchem.ncbi.nlm.nih.gov/compound/130115974#section=2D-Structure
https://pubchem.ncbi.nlm.nih.gov/compound/130113526#section=2D-Structure
https://pubchem.ncbi.nlm.nih.gov/compound/130067283#section=2D-Structure
https://pubchem.ncbi.nlm.nih.gov/compound/130058704#section=2D-Structure
https://pubchem.ncbi.nlm.nih.gov/compound/130056007#section=2D-Structure
https://pubchem.ncbi.nlm.nih.gov/compound/130053739#section=2D-Structure

https://pubchem.ncbi.nlm.nih.gov/compound/130038261#section=2D-Structure
https://pubchem.ncbi.nlm.nih.gov/compound/130038158#section=2D-Structure
https://pubchem.ncbi.nlm.nih.gov/compound/130038156#section=2D-Structure
https://pubchem.ncbi.nlm.nih.gov/compound/130038057#section=2D-Structure
https://pubchem.ncbi.nlm.nih.gov/compound/130037952#section=2D-Structure
https://pubchem.ncbi.nlm.nih.gov/compound/130037918#section=2D-Structure
https://pubchem.ncbi.nlm.nih.gov/compound/130037730#section=2D-Structure
https://pubchem.ncbi.nlm.nih.gov/compound/130037156#section=2D-Structure
https://pubchem.ncbi.nlm.nih.gov/compound/130034267#section=2D-Structure
https://pubchem.ncbi.nlm.nih.gov/compound/130032416#section=2D-Structure
https://pubchem.ncbi.nlm.nih.gov/compound/130032189#section=2D-Structure
https://pubchem.ncbi.nlm.nih.gov/compound/130031683#section=2D-Structure
https://pubchem.ncbi.nlm.nih.gov/compound/130031624#section=2D-Structure
https://pubchem.ncbi.nlm.nih.gov/compound/130031612#section=2D-Structure
https://pubchem.ncbi.nlm.nih.gov/compound/130031480#section=2D-Structure
https://pubchem.ncbi.nlm.nih.gov/compound/130030638#section=2D-Structure
https://pubchem.ncbi.nlm.nih.gov/compound/130030597#section=2D-Structure
https://pubchem.ncbi.nlm.nih.gov/compound/130030574#section=2D-Structure
https://pubchem.ncbi.nlm.nih.gov/compound/130027918#section=2D-Structure
https://pubchem.ncbi.nlm.nih.gov/compound/130027885#section=2D-Structure
https://pubchem.ncbi.nlm.nih.gov/compound/130027808#section=2D-Structure
https://pubchem.ncbi.nlm.nih.gov/compound/130027464#section=2D-Structure
https://pubchem.ncbi.nlm.nih.gov/compound/130026936#section=2D-Structure
https://pubchem.ncbi.nlm.nih.gov/compound/130026926#section=2D-Structure
https://pubchem.ncbi.nlm.nih.gov/compound/130018173#section=2D-Structure
https://pubchem.ncbi.nlm.nih.gov/compound/130017975#section=2D-Structure
https://pubchem.ncbi.nlm.nih.gov/compound/130017301#section=2D-Structure
https://pubchem.ncbi.nlm.nih.gov/compound/130015458#section=2D-Structure
https://pubchem.ncbi.nlm.nih.gov/compound/130015457#section=2D-Structure
https://pubchem.ncbi.nlm.nih.gov/compound/130015456#section=2D-Structure
https://pubchem.ncbi.nlm.nih.gov/compound/130015455#section=2D-Structure
https://pubchem.ncbi.nlm.nih.gov/compound/130015454#section=2D-Structure
https://pubchem.ncbi.nlm.nih.gov/compound/130015453#section=2D-Structure
https://pubchem.ncbi.nlm.nih.gov/compound/130015452#section=2D-Structure
https://pubchem.ncbi.nlm.nih.gov/compound/130015451#section=2D-Structure
https://pubchem.ncbi.nlm.nih.gov/compound/130015450#section=2D-Structure
https://pubchem.ncbi.nlm.nih.gov/compound/130015449#section=2D-Structure
https://pubchem.ncbi.nlm.nih.gov/compound/130015448#section=2D-Structure
https://pubchem.ncbi.nlm.nih.gov/compound/130015447#section=2D-Structure
https://pubchem.ncbi.nlm.nih.gov/compound/130015446#section=2D-Structure
https://pubchem.ncbi.nlm.nih.gov/compound/130015445#section=2D-Structure
https://pubchem.ncbi.nlm.nih.gov/compound/130015444#section=2D-Structure
https://pubchem.ncbi.nlm.nih.gov/compound/130015443#section=2D-Structure
https://pubchem.ncbi.nlm.nih.gov/compound/130015441#section=2D-Structure
https://pubchem.ncbi.nlm.nih.gov/compound/130015439#section=2D-Structure
https://pubchem.ncbi.nlm.nih.gov/compound/130015434#section=2D-Structure
https://pubchem.ncbi.nlm.nih.gov/compound/130015431#section=2D-Structure
https://pubchem.ncbi.nlm.nih.gov/compound/130015430#section=2D-Structure
https://pubchem.ncbi.nlm.nih.gov/compound/130015426#section=2D-Structure
https://pubchem.ncbi.nlm.nih.gov/compound/130015423#section=2D-Structure
https://pubchem.ncbi.nlm.nih.gov/compound/130015422#section=2D-Structure
https://pubchem.ncbi.nlm.nih.gov/compound/130015413#section=2D-Structure
https://pubchem.ncbi.nlm.nih.gov/compound/130015412#section=2D-Structure
https://pubchem.ncbi.nlm.nih.gov/compound/130015411#section=2D-Structure
https://pubchem.ncbi.nlm.nih.gov/compound/130015410#section=2D-Structure

https://pubchem.ncbi.nlm.nih.gov/compound/130015409#section=2D-Structure
https://pubchem.ncbi.nlm.nih.gov/compound/130015407#section=2D-Structure
https://pubchem.ncbi.nlm.nih.gov/compound/130015403#section=2D-Structure
https://pubchem.ncbi.nlm.nih.gov/compound/130015215#section=2D-Structure
https://pubchem.ncbi.nlm.nih.gov/compound/130014752#section=2D-Structure
https://pubchem.ncbi.nlm.nih.gov/compound/130013945#section=2D-Structure
https://pubchem.ncbi.nlm.nih.gov/compound/130013852#section=2D-Structure
https://pubchem.ncbi.nlm.nih.gov/compound/130013724#section=2D-Structure
https://pubchem.ncbi.nlm.nih.gov/compound/130013722#section=2D-Structure
https://pubchem.ncbi.nlm.nih.gov/compound/130013720#section=2D-Structure
https://pubchem.ncbi.nlm.nih.gov/compound/130013557#section=2D-Structure
https://pubchem.ncbi.nlm.nih.gov/compound/130013521#section=2D-Structure
https://pubchem.ncbi.nlm.nih.gov/compound/130013167#section=2D-Structure
https://pubchem.ncbi.nlm.nih.gov/compound/130012777#section=2D-Structure
https://pubchem.ncbi.nlm.nih.gov/compound/130012755#section=2D-Structure
https://pubchem.ncbi.nlm.nih.gov/compound/130012730#section=2D-Structure
https://pubchem.ncbi.nlm.nih.gov/compound/13001161#section=2D-Structure
https://pubchem.ncbi.nlm.nih.gov/compound/130010492#section=2D-Structure
https://pubchem.ncbi.nlm.nih.gov/compound/130010485#section=2D-Structure
https://pubchem.ncbi.nlm.nih.gov/compound/130009896#section=2D-Structure
https://pubchem.ncbi.nlm.nih.gov/compound/130009781#section=2D-Structure
https://pubchem.ncbi.nlm.nih.gov/compound/130009761#section=2D-Structure
https://pubchem.ncbi.nlm.nih.gov/compound/130009747#section=2D-Structure
https://pubchem.ncbi.nlm.nih.gov/compound/130009586#section=2D-Structure
https://pubchem.ncbi.nlm.nih.gov/compound/130009524#section=2D-Structure
https://pubchem.ncbi.nlm.nih.gov/compound/130009458#section=2D-Structure
https://pubchem.ncbi.nlm.nih.gov/compound/130007084#section=2D-Structure
https://pubchem.ncbi.nlm.nih.gov/compound/130006640#section=2D-Structure
https://pubchem.ncbi.nlm.nih.gov/compound/130006627#section=2D-Structure
https://pubchem.ncbi.nlm.nih.gov/compound/130006616#section=2D-Structure
https://pubchem.ncbi.nlm.nih.gov/compound/130006539#section=2D-Structure
https://pubchem.ncbi.nlm.nih.gov/compound/130006536#section=2D-Structure
https://pubchem.ncbi.nlm.nih.gov/compound/130006500#section=2D-Structure
https://pubchem.ncbi.nlm.nih.gov/compound/130006499#section=2D-Structure
https://pubchem.ncbi.nlm.nih.gov/compound/130006421#section=2D-Structure
https://pubchem.ncbi.nlm.nih.gov/compound/130006144#section=2D-Structure
https://pubchem.ncbi.nlm.nih.gov/compound/130006132#section=2D-Structure
https://pubchem.ncbi.nlm.nih.gov/compound/130006050#section=2D-Structure
https://pubchem.ncbi.nlm.nih.gov/compound/130005857#section=2D-Structure
https://pubchem.ncbi.nlm.nih.gov/compound/130005562#section=2D-Structure
https://pubchem.ncbi.nlm.nih.gov/compound/130005505#section=2D-Structure
https://pubchem.ncbi.nlm.nih.gov/compound/130005435#section=2D-Structure
https://pubchem.ncbi.nlm.nih.gov/compound/130005434#section=2D-Structure
https://pubchem.ncbi.nlm.nih.gov/compound/130005409#section=2D-Structure
https://pubchem.ncbi.nlm.nih.gov/compound/130005408#section=2D-Structure
https://pubchem.ncbi.nlm.nih.gov/compound/130005401#section=2D-Structure
https://pubchem.ncbi.nlm.nih.gov/compound/130005399#section=2D-Structure
https://pubchem.ncbi.nlm.nih.gov/compound/130005240#section=2D-Structure
https://pubchem.ncbi.nlm.nih.gov/compound/130004853#section=2D-Structure
https://pubchem.ncbi.nlm.nih.gov/compound/12999139#section=2D-Structure
https://pubchem.ncbi.nlm.nih.gov/compound/12998954#section=2D-Structure
https://pubchem.ncbi.nlm.nih.gov/compound/129980903#section=2D-Structure
https://pubchem.ncbi.nlm.nih.gov/compound/129978836#section=2D-Structure
https://pubchem.ncbi.nlm.nih.gov/compound/129978140#section=2D-Structure
https://pubchem.ncbi.nlm.nih.gov/compound/129977844#section=2D-Structure

https://pubchem.ncbi.nlm.nih.gov/compound/129977391#section=2D-Structure
https://pubchem.ncbi.nlm.nih.gov/compound/129972896#section=2D-Structure
https://pubchem.ncbi.nlm.nih.gov/compound/129972895#section=2D-Structure
https://pubchem.ncbi.nlm.nih.gov/compound/129972835#section=2D-Structure
https://pubchem.ncbi.nlm.nih.gov/compound/129972423#section=2D-Structure
https://pubchem.ncbi.nlm.nih.gov/compound/129972422#section=2D-Structure
https://pubchem.ncbi.nlm.nih.gov/compound/129972421#section=2D-Structure
https://pubchem.ncbi.nlm.nih.gov/compound/129972413#section=2D-Structure
https://pubchem.ncbi.nlm.nih.gov/compound/129972258#section=2D-Structure
https://pubchem.ncbi.nlm.nih.gov/compound/129968819#section=2D-Structure
https://pubchem.ncbi.nlm.nih.gov/compound/129968709#section=2D-Structure
https://pubchem.ncbi.nlm.nih.gov/compound/129965725#section=2D-Structure
https://pubchem.ncbi.nlm.nih.gov/compound/129956308#section=2D-Structure
https://pubchem.ncbi.nlm.nih.gov/compound/129956304#section=2D-Structure
https://pubchem.ncbi.nlm.nih.gov/compound/129956217#section=2D-Structure
https://pubchem.ncbi.nlm.nih.gov/compound/129950282#section=2D-Structure
https://pubchem.ncbi.nlm.nih.gov/compound/129940581#section=2D-Structure
https://pubchem.ncbi.nlm.nih.gov/compound/129940393#section=2D-Structure
https://pubchem.ncbi.nlm.nih.gov/compound/129939462#section=2D-Structure
https://pubchem.ncbi.nlm.nih.gov/compound/129939342#section=2D-Structure
https://pubchem.ncbi.nlm.nih.gov/compound/129938919#section=2D-Structure
https://pubchem.ncbi.nlm.nih.gov/compound/129938107#section=2D-Structure
https://pubchem.ncbi.nlm.nih.gov/compound/129938102#section=2D-Structure
https://pubchem.ncbi.nlm.nih.gov/compound/129937893#section=2D-Structure
https://pubchem.ncbi.nlm.nih.gov/compound/129935972#section=2D-Structure
https://pubchem.ncbi.nlm.nih.gov/compound/129935081#section=2D-Structure
https://pubchem.ncbi.nlm.nih.gov/compound/129935013#section=2D-Structure
https://pubchem.ncbi.nlm.nih.gov/compound/129934628#section=2D-Structure
https://pubchem.ncbi.nlm.nih.gov/compound/12993412#section=2D-Structure
https://pubchem.ncbi.nlm.nih.gov/compound/129934102#section=2D-Structure
https://pubchem.ncbi.nlm.nih.gov/compound/129933496#section=2D-Structure
https://pubchem.ncbi.nlm.nih.gov/compound/129933307#section=2D-Structure
https://pubchem.ncbi.nlm.nih.gov/compound/129933165#section=2D-Structure
https://pubchem.ncbi.nlm.nih.gov/compound/129933099#section=2D-Structure
https://pubchem.ncbi.nlm.nih.gov/compound/129932937#section=2D-Structure
https://pubchem.ncbi.nlm.nih.gov/compound/129932188#section=2D-Structure
https://pubchem.ncbi.nlm.nih.gov/compound/129932187#section=2D-Structure
https://pubchem.ncbi.nlm.nih.gov/compound/129931438#section=2D-Structure
https://pubchem.ncbi.nlm.nih.gov/compound/129931416#section=2D-Structure
https://pubchem.ncbi.nlm.nih.gov/compound/129920753#section=2D-Structure
https://pubchem.ncbi.nlm.nih.gov/compound/129920751#section=2D-Structure
https://pubchem.ncbi.nlm.nih.gov/compound/129920737#section=2D-Structure
https://pubchem.ncbi.nlm.nih.gov/compound/129920729#section=2D-Structure
https://pubchem.ncbi.nlm.nih.gov/compound/129920728#section=2D-Structure
https://pubchem.ncbi.nlm.nih.gov/compound/129920727#section=2D-Structure
https://pubchem.ncbi.nlm.nih.gov/compound/129920720#section=2D-Structure
https://pubchem.ncbi.nlm.nih.gov/compound/129920710#section=2D-Structure
https://pubchem.ncbi.nlm.nih.gov/compound/129920663#section=2D-Structure
https://pubchem.ncbi.nlm.nih.gov/compound/129920661#section=2D-Structure
https://pubchem.ncbi.nlm.nih.gov/compound/129920660#section=2D-Structure
https://pubchem.ncbi.nlm.nih.gov/compound/129920659#section=2D-Structure
https://pubchem.ncbi.nlm.nih.gov/compound/129919690#section=2D-Structure
https://pubchem.ncbi.nlm.nih.gov/compound/129918511#section=2D-Structure
https://pubchem.ncbi.nlm.nih.gov/compound/129918341#section=2D-Structure
https://pubchem.ncbi.nlm.nih.gov/compound/129918339#section=2D-Structure

https://pubchem.ncbi.nlm.nih.gov/compound/129918007#section=2D-Structure
https://pubchem.ncbi.nlm.nih.gov/compound/129918002#section=2D-Structure
https://pubchem.ncbi.nlm.nih.gov/compound/129917801#section=2D-Structure
https://pubchem.ncbi.nlm.nih.gov/compound/129917663#section=2D-Structure
https://pubchem.ncbi.nlm.nih.gov/compound/129917273#section=2D-Structure
https://pubchem.ncbi.nlm.nih.gov/compound/129917108#section=2D-Structure
https://pubchem.ncbi.nlm.nih.gov/compound/129916929#section=2D-Structure
https://pubchem.ncbi.nlm.nih.gov/compound/129916776#section=2D-Structure
https://pubchem.ncbi.nlm.nih.gov/compound/129916615#section=2D-Structure
https://pubchem.ncbi.nlm.nih.gov/compound/129915807#section=2D-Structure
https://pubchem.ncbi.nlm.nih.gov/compound/129915647#section=2D-Structure
https://pubchem.ncbi.nlm.nih.gov/compound/129914482#section=2D-Structure
https://pubchem.ncbi.nlm.nih.gov/compound/129914279#section=2D-Structure
https://pubchem.ncbi.nlm.nih.gov/compound/129914158#section=2D-Structure
https://pubchem.ncbi.nlm.nih.gov/compound/129913901#section=2D-Structure
https://pubchem.ncbi.nlm.nih.gov/compound/129913894#section=2D-Structure
https://pubchem.ncbi.nlm.nih.gov/compound/129913893#section=2D-Structure
https://pubchem.ncbi.nlm.nih.gov/compound/129913890#section=2D-Structure
https://pubchem.ncbi.nlm.nih.gov/compound/129913857#section=2D-Structure
https://pubchem.ncbi.nlm.nih.gov/compound/129912435#section=2D-Structure
https://pubchem.ncbi.nlm.nih.gov/compound/129911171#section=2D-Structure
https://pubchem.ncbi.nlm.nih.gov/compound/129911103#section=2D-Structure
https://pubchem.ncbi.nlm.nih.gov/compound/129910950#section=2D-Structure
https://pubchem.ncbi.nlm.nih.gov/compound/129910928#section=2D-Structure
https://pubchem.ncbi.nlm.nih.gov/compound/129910927#section=2D-Structure
https://pubchem.ncbi.nlm.nih.gov/compound/129910877#section=2D-Structure
https://pubchem.ncbi.nlm.nih.gov/compound/129910853#section=2D-Structure
https://pubchem.ncbi.nlm.nih.gov/compound/129910830#section=2D-Structure
https://pubchem.ncbi.nlm.nih.gov/compound/129910829#section=2D-Structure
https://pubchem.ncbi.nlm.nih.gov/compound/129910779#section=2D-Structure
https://pubchem.ncbi.nlm.nih.gov/compound/129910702#section=2D-Structure
https://pubchem.ncbi.nlm.nih.gov/compound/129910697#section=2D-Structure
https://pubchem.ncbi.nlm.nih.gov/compound/129910675#section=2D-Structure
https://pubchem.ncbi.nlm.nih.gov/compound/129910419#section=2D-Structure
https://pubchem.ncbi.nlm.nih.gov/compound/129910192#section=2D-Structure
https://pubchem.ncbi.nlm.nih.gov/compound/129892387#section=2D-Structure
https://pubchem.ncbi.nlm.nih.gov/compound/129892384#section=2D-Structure
https://pubchem.ncbi.nlm.nih.gov/compound/129891720#section=2D-Structure
https://pubchem.ncbi.nlm.nih.gov/compound/129891669#section=2D-Structure
https://pubchem.ncbi.nlm.nih.gov/compound/129888313#section=2D-Structure
https://pubchem.ncbi.nlm.nih.gov/compound/129888095#section=2D-Structure
https://pubchem.ncbi.nlm.nih.gov/compound/129888055#section=2D-Structure
https://pubchem.ncbi.nlm.nih.gov/compound/129887989#section=2D-Structure
https://pubchem.ncbi.nlm.nih.gov/compound/129886068#section=2D-Structure
https://pubchem.ncbi.nlm.nih.gov/compound/129885858#section=2D-Structure
https://pubchem.ncbi.nlm.nih.gov/compound/129885356#section=2D-Structure
https://pubchem.ncbi.nlm.nih.gov/compound/129884760#section=2D-Structure
https://pubchem.ncbi.nlm.nih.gov/compound/129884719#section=2D-Structure
https://pubchem.ncbi.nlm.nih.gov/compound/129884652#section=2D-Structure
https://pubchem.ncbi.nlm.nih.gov/compound/129884483#section=2D-Structure
https://pubchem.ncbi.nlm.nih.gov/compound/129884416#section=2D-Structure
https://pubchem.ncbi.nlm.nih.gov/compound/129884348#section=2D-Structure
https://pubchem.ncbi.nlm.nih.gov/compound/129884329#section=2D-Structure
https://pubchem.ncbi.nlm.nih.gov/compound/129883397#section=2D-Structure
https://pubchem.ncbi.nlm.nih.gov/compound/129883194#section=2D-Structure

https://pubchem.ncbi.nlm.nih.gov/compound/129882072#section=2D-Structure
https://pubchem.ncbi.nlm.nih.gov/compound/129881425#section=2D-Structure
https://pubchem.ncbi.nlm.nih.gov/compound/129881420#section=2D-Structure
https://pubchem.ncbi.nlm.nih.gov/compound/129880115#section=2D-Structure
https://pubchem.ncbi.nlm.nih.gov/compound/129880104#section=2D-Structure
https://pubchem.ncbi.nlm.nih.gov/compound/129880094#section=2D-Structure
https://pubchem.ncbi.nlm.nih.gov/compound/129880056#section=2D-Structure
https://pubchem.ncbi.nlm.nih.gov/compound/129880042#section=2D-Structure
https://pubchem.ncbi.nlm.nih.gov/compound/129880012#section=2D-Structure
https://pubchem.ncbi.nlm.nih.gov/compound/129879760#section=2D-Structure
https://pubchem.ncbi.nlm.nih.gov/compound/129879745#section=2D-Structure
https://pubchem.ncbi.nlm.nih.gov/compound/129879730#section=2D-Structure
https://pubchem.ncbi.nlm.nih.gov/compound/129879647#section=2D-Structure
https://pubchem.ncbi.nlm.nih.gov/compound/129879338#section=2D-Structure
https://pubchem.ncbi.nlm.nih.gov/compound/129879324#section=2D-Structure
https://pubchem.ncbi.nlm.nih.gov/compound/129878426#section=2D-Structure
https://pubchem.ncbi.nlm.nih.gov/compound/129878416#section=2D-Structure
https://pubchem.ncbi.nlm.nih.gov/compound/129878375#section=2D-Structure
https://pubchem.ncbi.nlm.nih.gov/compound/129877899#section=2D-Structure
https://pubchem.ncbi.nlm.nih.gov/compound/129877887#section=2D-Structure
https://pubchem.ncbi.nlm.nih.gov/compound/129877709#section=2D-Structure
https://pubchem.ncbi.nlm.nih.gov/compound/129877283#section=2D-Structure
https://pubchem.ncbi.nlm.nih.gov/compound/129877080#section=2D-Structure
https://pubchem.ncbi.nlm.nih.gov/compound/129877052#section=2D-Structure
https://pubchem.ncbi.nlm.nih.gov/compound/129876752#section=2D-Structure
https://pubchem.ncbi.nlm.nih.gov/compound/129876738#section=2D-Structure
https://pubchem.ncbi.nlm.nih.gov/compound/129876726#section=2D-Structure
https://pubchem.ncbi.nlm.nih.gov/compound/129876712#section=2D-Structure
https://pubchem.ncbi.nlm.nih.gov/compound/129876517#section=2D-Structure
https://pubchem.ncbi.nlm.nih.gov/compound/129876391#section=2D-Structure
https://pubchem.ncbi.nlm.nih.gov/compound/129875957#section=2D-Structure
https://pubchem.ncbi.nlm.nih.gov/compound/129875790#section=2D-Structure
https://pubchem.ncbi.nlm.nih.gov/compound/129875554#section=2D-Structure
https://pubchem.ncbi.nlm.nih.gov/compound/129875545#section=2D-Structure
https://pubchem.ncbi.nlm.nih.gov/compound/129875208#section=2D-Structure
https://pubchem.ncbi.nlm.nih.gov/compound/129875136#section=2D-Structure
https://pubchem.ncbi.nlm.nih.gov/compound/129874530#section=2D-Structure
https://pubchem.ncbi.nlm.nih.gov/compound/129874114#section=2D-Structure
https://pubchem.ncbi.nlm.nih.gov/compound/129873870#section=2D-Structure
https://pubchem.ncbi.nlm.nih.gov/compound/129873830#section=2D-Structure
https://pubchem.ncbi.nlm.nih.gov/compound/129873812#section=2D-Structure
https://pubchem.ncbi.nlm.nih.gov/compound/129873752#section=2D-Structure
https://pubchem.ncbi.nlm.nih.gov/compound/129873567#section=2D-Structure
https://pubchem.ncbi.nlm.nih.gov/compound/129873483#section=2D-Structure
https://pubchem.ncbi.nlm.nih.gov/compound/129873346#section=2D-Structure
https://pubchem.ncbi.nlm.nih.gov/compound/129873007#section=2D-Structure
https://pubchem.ncbi.nlm.nih.gov/compound/129872827#section=2D-Structure
https://pubchem.ncbi.nlm.nih.gov/compound/129872472#section=2D-Structure
https://pubchem.ncbi.nlm.nih.gov/compound/129872445#section=2D-Structure
https://pubchem.ncbi.nlm.nih.gov/compound/129872306#section=2D-Structure
https://pubchem.ncbi.nlm.nih.gov/compound/129872302#section=2D-Structure
https://pubchem.ncbi.nlm.nih.gov/compound/129872187#section=2D-Structure
https://pubchem.ncbi.nlm.nih.gov/compound/129872035#section=2D-Structure
https://pubchem.ncbi.nlm.nih.gov/compound/129871882#section=2D-Structure
https://pubchem.ncbi.nlm.nih.gov/compound/129871771#section=2D-Structure

https://pubchem.ncbi.nlm.nih.gov/compound/129871614#section=2D-Structure
https://pubchem.ncbi.nlm.nih.gov/compound/129871592#section=2D-Structure
https://pubchem.ncbi.nlm.nih.gov/compound/129871501#section=2D-Structure
https://pubchem.ncbi.nlm.nih.gov/compound/129871296#section=2D-Structure
https://pubchem.ncbi.nlm.nih.gov/compound/129871291#section=2D-Structure
https://pubchem.ncbi.nlm.nih.gov/compound/129871137#section=2D-Structure
https://pubchem.ncbi.nlm.nih.gov/compound/129871063#section=2D-Structure
https://pubchem.ncbi.nlm.nih.gov/compound/129870932#section=2D-Structure
https://pubchem.ncbi.nlm.nih.gov/compound/129870873#section=2D-Structure
https://pubchem.ncbi.nlm.nih.gov/compound/129870799#section=2D-Structure
https://pubchem.ncbi.nlm.nih.gov/compound/129870792#section=2D-Structure
https://pubchem.ncbi.nlm.nih.gov/compound/129870655#section=2D-Structure
https://pubchem.ncbi.nlm.nih.gov/compound/129869574#section=2D-Structure
https://pubchem.ncbi.nlm.nih.gov/compound/129869022#section=2D-Structure
https://pubchem.ncbi.nlm.nih.gov/compound/129868540#section=2D-Structure
https://pubchem.ncbi.nlm.nih.gov/compound/129868044#section=2D-Structure
https://pubchem.ncbi.nlm.nih.gov/compound/129868031#section=2D-Structure
https://pubchem.ncbi.nlm.nih.gov/compound/129867993#section=2D-Structure
https://pubchem.ncbi.nlm.nih.gov/compound/129867348#section=2D-Structure
https://pubchem.ncbi.nlm.nih.gov/compound/129866315#section=2D-Structure
https://pubchem.ncbi.nlm.nih.gov/compound/129866068#section=2D-Structure
https://pubchem.ncbi.nlm.nih.gov/compound/129865021#section=2D-Structure
https://pubchem.ncbi.nlm.nih.gov/compound/129864704#section=2D-Structure
https://pubchem.ncbi.nlm.nih.gov/compound/129864413#section=2D-Structure
https://pubchem.ncbi.nlm.nih.gov/compound/129864412#section=2D-Structure
https://pubchem.ncbi.nlm.nih.gov/compound/129864406#section=2D-Structure
https://pubchem.ncbi.nlm.nih.gov/compound/129864378#section=2D-Structure
https://pubchem.ncbi.nlm.nih.gov/compound/129864367#section=2D-Structure
https://pubchem.ncbi.nlm.nih.gov/compound/129864159#section=2D-Structure
https://pubchem.ncbi.nlm.nih.gov/compound/129862031#section=2D-Structure
https://pubchem.ncbi.nlm.nih.gov/compound/129861635#section=2D-Structure
https://pubchem.ncbi.nlm.nih.gov/compound/129860738#section=2D-Structure
https://pubchem.ncbi.nlm.nih.gov/compound/129860547#section=2D-Structure
https://pubchem.ncbi.nlm.nih.gov/compound/129860534#section=2D-Structure
https://pubchem.ncbi.nlm.nih.gov/compound/129860341#section=2D-Structure
https://pubchem.ncbi.nlm.nih.gov/compound/129860211#section=2D-Structure
https://pubchem.ncbi.nlm.nih.gov/compound/129860193#section=2D-Structure
https://pubchem.ncbi.nlm.nih.gov/compound/129860084#section=2D-Structure
https://pubchem.ncbi.nlm.nih.gov/compound/129860013#section=2D-Structure
https://pubchem.ncbi.nlm.nih.gov/compound/129860002#section=2D-Structure
https://pubchem.ncbi.nlm.nih.gov/compound/129859575#section=2D-Structure
https://pubchem.ncbi.nlm.nih.gov/compound/129859392#section=2D-Structure
https://pubchem.ncbi.nlm.nih.gov/compound/129858756#section=2D-Structure
https://pubchem.ncbi.nlm.nih.gov/compound/129858520#section=2D-Structure
https://pubchem.ncbi.nlm.nih.gov/compound/129858489#section=2D-Structure
https://pubchem.ncbi.nlm.nih.gov/compound/129858010#section=2D-Structure
https://pubchem.ncbi.nlm.nih.gov/compound/129857953#section=2D-Structure
https://pubchem.ncbi.nlm.nih.gov/compound/129857610#section=2D-Structure
https://pubchem.ncbi.nlm.nih.gov/compound/129857284#section=2D-Structure
https://pubchem.ncbi.nlm.nih.gov/compound/129857133#section=2D-Structure
https://pubchem.ncbi.nlm.nih.gov/compound/129856546#section=2D-Structure
https://pubchem.ncbi.nlm.nih.gov/compound/129856473#section=2D-Structure
https://pubchem.ncbi.nlm.nih.gov/compound/129855875#section=2D-Structure
https://pubchem.ncbi.nlm.nih.gov/compound/129855844#section=2D-Structure
https://pubchem.ncbi.nlm.nih.gov/compound/129855755#section=2D-Structure

https://pubchem.ncbi.nlm.nih.gov/compound/129855727#section=2D-Structure
https://pubchem.ncbi.nlm.nih.gov/compound/129855669#section=2D-Structure
https://pubchem.ncbi.nlm.nih.gov/compound/129855653#section=2D-Structure
https://pubchem.ncbi.nlm.nih.gov/compound/129855338#section=2D-Structure
https://pubchem.ncbi.nlm.nih.gov/compound/129854852#section=2D-Structure
https://pubchem.ncbi.nlm.nih.gov/compound/129853110#section=2D-Structure
https://pubchem.ncbi.nlm.nih.gov/compound/129853052#section=2D-Structure
https://pubchem.ncbi.nlm.nih.gov/compound/129852955#section=2D-Structure
https://pubchem.ncbi.nlm.nih.gov/compound/129852928#section=2D-Structure
https://pubchem.ncbi.nlm.nih.gov/compound/129852706#section=2D-Structure
https://pubchem.ncbi.nlm.nih.gov/compound/129851331#section=2D-Structure
https://pubchem.ncbi.nlm.nih.gov/compound/129850485#section=2D-Structure
https://pubchem.ncbi.nlm.nih.gov/compound/129850279#section=2D-Structure
https://pubchem.ncbi.nlm.nih.gov/compound/129849634#section=2D-Structure
https://pubchem.ncbi.nlm.nih.gov/compound/129849632#section=2D-Structure
https://pubchem.ncbi.nlm.nih.gov/compound/129848730#section=2D-Structure
https://pubchem.ncbi.nlm.nih.gov/compound/129848351#section=2D-Structure
https://pubchem.ncbi.nlm.nih.gov/compound/129848262#section=2D-Structure
https://pubchem.ncbi.nlm.nih.gov/compound/129847881#section=2D-Structure
https://pubchem.ncbi.nlm.nih.gov/compound/129847548#section=2D-Structure
https://pubchem.ncbi.nlm.nih.gov/compound/129846023#section=2D-Structure
https://pubchem.ncbi.nlm.nih.gov/compound/129845542#section=2D-Structure
https://pubchem.ncbi.nlm.nih.gov/compound/129845519#section=2D-Structure
https://pubchem.ncbi.nlm.nih.gov/compound/129845236#section=2D-Structure
https://pubchem.ncbi.nlm.nih.gov/compound/129845043#section=2D-Structure
https://pubchem.ncbi.nlm.nih.gov/compound/129842611#section=2D-Structure
https://pubchem.ncbi.nlm.nih.gov/compound/129842022#section=2D-Structure
https://pubchem.ncbi.nlm.nih.gov/compound/129841932#section=2D-Structure
https://pubchem.ncbi.nlm.nih.gov/compound/129841614#section=2D-Structure
https://pubchem.ncbi.nlm.nih.gov/compound/129841151#section=2D-Structure
https://pubchem.ncbi.nlm.nih.gov/compound/129840863#section=2D-Structure
https://pubchem.ncbi.nlm.nih.gov/compound/129837528#section=2D-Structure
https://pubchem.ncbi.nlm.nih.gov/compound/129837526#section=2D-Structure
https://pubchem.ncbi.nlm.nih.gov/compound/129837508#section=2D-Structure
https://pubchem.ncbi.nlm.nih.gov/compound/129836037#section=2D-Structure
https://pubchem.ncbi.nlm.nih.gov/compound/129834698#section=2D-Structure
https://pubchem.ncbi.nlm.nih.gov/compound/129834650#section=2D-Structure
https://pubchem.ncbi.nlm.nih.gov/compound/129834062#section=2D-Structure
https://pubchem.ncbi.nlm.nih.gov/compound/129833657#section=2D-Structure
https://pubchem.ncbi.nlm.nih.gov/compound/129832669#section=2D-Structure
https://pubchem.ncbi.nlm.nih.gov/compound/129832654#section=2D-Structure
https://pubchem.ncbi.nlm.nih.gov/compound/129832585#section=2D-Structure
https://pubchem.ncbi.nlm.nih.gov/compound/129832561#section=2D-Structure
https://pubchem.ncbi.nlm.nih.gov/compound/129832243#section=2D-Structure
https://pubchem.ncbi.nlm.nih.gov/compound/129830385#section=2D-Structure
https://pubchem.ncbi.nlm.nih.gov/compound/129829440#section=2D-Structure
https://pubchem.ncbi.nlm.nih.gov/compound/129829435#section=2D-Structure
https://pubchem.ncbi.nlm.nih.gov/compound/129829335#section=2D-Structure
https://pubchem.ncbi.nlm.nih.gov/compound/129828956#section=2D-Structure
https://pubchem.ncbi.nlm.nih.gov/compound/129828927#section=2D-Structure
https://pubchem.ncbi.nlm.nih.gov/compound/129827937#section=2D-Structure
https://pubchem.ncbi.nlm.nih.gov/compound/129826099#section=2D-Structure
https://pubchem.ncbi.nlm.nih.gov/compound/129826088#section=2D-Structure
https://pubchem.ncbi.nlm.nih.gov/compound/129826016#section=2D-Structure
https://pubchem.ncbi.nlm.nih.gov/compound/129825850#section=2D-Structure

https://pubchem.ncbi.nlm.nih.gov/compound/129825668#section=2D-Structure
https://pubchem.ncbi.nlm.nih.gov/compound/129825667#section=2D-Structure
https://pubchem.ncbi.nlm.nih.gov/compound/129825400#section=2D-Structure
https://pubchem.ncbi.nlm.nih.gov/compound/129825383#section=2D-Structure
https://pubchem.ncbi.nlm.nih.gov/compound/129824389#section=2D-Structure
https://pubchem.ncbi.nlm.nih.gov/compound/129824001#section=2D-Structure
https://pubchem.ncbi.nlm.nih.gov/compound/129822687#section=2D-Structure
https://pubchem.ncbi.nlm.nih.gov/compound/129822665#section=2D-Structure
https://pubchem.ncbi.nlm.nih.gov/compound/129820811#section=2D-Structure
https://pubchem.ncbi.nlm.nih.gov/compound/129819653#section=2D-Structure
https://pubchem.ncbi.nlm.nih.gov/compound/129819047#section=2D-Structure
https://pubchem.ncbi.nlm.nih.gov/compound/129818086#section=2D-Structure
https://pubchem.ncbi.nlm.nih.gov/compound/129818046#section=2D-Structure
https://pubchem.ncbi.nlm.nih.gov/compound/129817981#section=2D-Structure
https://pubchem.ncbi.nlm.nih.gov/compound/129817965#section=2D-Structure
https://pubchem.ncbi.nlm.nih.gov/compound/129817079#section=2D-Structure
https://pubchem.ncbi.nlm.nih.gov/compound/129816529#section=2D-Structure
https://pubchem.ncbi.nlm.nih.gov/compound/129816504#section=2D-Structure
https://pubchem.ncbi.nlm.nih.gov/compound/129816394#section=2D-Structure
https://pubchem.ncbi.nlm.nih.gov/compound/129816333#section=2D-Structure
https://pubchem.ncbi.nlm.nih.gov/compound/129816307#section=2D-Structure
https://pubchem.ncbi.nlm.nih.gov/compound/129815902#section=2D-Structure
https://pubchem.ncbi.nlm.nih.gov/compound/129815355#section=2D-Structure
https://pubchem.ncbi.nlm.nih.gov/compound/129815297#section=2D-Structure
https://pubchem.ncbi.nlm.nih.gov/compound/129815264#section=2D-Structure
https://pubchem.ncbi.nlm.nih.gov/compound/129815039#section=2D-Structure
https://pubchem.ncbi.nlm.nih.gov/compound/129814690#section=2D-Structure
https://pubchem.ncbi.nlm.nih.gov/compound/129814626#section=2D-Structure
https://pubchem.ncbi.nlm.nih.gov/compound/129814535#section=2D-Structure
https://pubchem.ncbi.nlm.nih.gov/compound/129814436#section=2D-Structure
https://pubchem.ncbi.nlm.nih.gov/compound/129814088#section=2D-Structure
https://pubchem.ncbi.nlm.nih.gov/compound/129814042#section=2D-Structure
https://pubchem.ncbi.nlm.nih.gov/compound/129813924#section=2D-Structure
https://pubchem.ncbi.nlm.nih.gov/compound/129813745#section=2D-Structure
https://pubchem.ncbi.nlm.nih.gov/compound/129813740#section=2D-Structure
https://pubchem.ncbi.nlm.nih.gov/compound/129813713#section=2D-Structure
https://pubchem.ncbi.nlm.nih.gov/compound/129813648#section=2D-Structure
https://pubchem.ncbi.nlm.nih.gov/compound/129811873#section=2D-Structure
https://pubchem.ncbi.nlm.nih.gov/compound/129811568#section=2D-Structure
https://pubchem.ncbi.nlm.nih.gov/compound/129811517#section=2D-Structure
https://pubchem.ncbi.nlm.nih.gov/compound/129807911#section=2D-Structure
https://pubchem.ncbi.nlm.nih.gov/compound/129806545#section=2D-Structure
https://pubchem.ncbi.nlm.nih.gov/compound/129805960#section=2D-Structure
https://pubchem.ncbi.nlm.nih.gov/compound/129803354#section=2D-Structure
https://pubchem.ncbi.nlm.nih.gov/compound/129802966#section=2D-Structure
https://pubchem.ncbi.nlm.nih.gov/compound/129802668#section=2D-Structure
https://pubchem.ncbi.nlm.nih.gov/compound/129802464#section=2D-Structure
https://pubchem.ncbi.nlm.nih.gov/compound/129802255#section=2D-Structure
https://pubchem.ncbi.nlm.nih.gov/compound/129801913#section=2D-Structure
https://pubchem.ncbi.nlm.nih.gov/compound/129801781#section=2D-Structure
https://pubchem.ncbi.nlm.nih.gov/compound/129801613#section=2D-Structure
https://pubchem.ncbi.nlm.nih.gov/compound/129801408#section=2D-Structure
https://pubchem.ncbi.nlm.nih.gov/compound/129801283#section=2D-Structure
https://pubchem.ncbi.nlm.nih.gov/compound/129801241#section=2D-Structure
https://pubchem.ncbi.nlm.nih.gov/compound/129801180#section=2D-Structure

https://pubchem.ncbi.nlm.nih.gov/compound/129801088#section=2D-Structure
https://pubchem.ncbi.nlm.nih.gov/compound/129801040#section=2D-Structure
https://pubchem.ncbi.nlm.nih.gov/compound/129801028#section=2D-Structure
https://pubchem.ncbi.nlm.nih.gov/compound/129801021#section=2D-Structure
https://pubchem.ncbi.nlm.nih.gov/compound/129801010#section=2D-Structure
https://pubchem.ncbi.nlm.nih.gov/compound/129800561#section=2D-Structure
https://pubchem.ncbi.nlm.nih.gov/compound/129800425#section=2D-Structure
https://pubchem.ncbi.nlm.nih.gov/compound/129800313#section=2D-Structure
https://pubchem.ncbi.nlm.nih.gov/compound/129800260#section=2D-Structure
https://pubchem.ncbi.nlm.nih.gov/compound/129800237#section=2D-Structure
https://pubchem.ncbi.nlm.nih.gov/compound/129800126#section=2D-Structure
https://pubchem.ncbi.nlm.nih.gov/compound/129799987#section=2D-Structure
https://pubchem.ncbi.nlm.nih.gov/compound/129799967#section=2D-Structure
https://pubchem.ncbi.nlm.nih.gov/compound/129799767#section=2D-Structure
https://pubchem.ncbi.nlm.nih.gov/compound/129799717#section=2D-Structure
https://pubchem.ncbi.nlm.nih.gov/compound/129799442#section=2D-Structure
https://pubchem.ncbi.nlm.nih.gov/compound/129799258#section=2D-Structure
https://pubchem.ncbi.nlm.nih.gov/compound/129799178#section=2D-Structure
https://pubchem.ncbi.nlm.nih.gov/compound/129799044#section=2D-Structure
https://pubchem.ncbi.nlm.nih.gov/compound/129799004#section=2D-Structure
https://pubchem.ncbi.nlm.nih.gov/compound/129798958#section=2D-Structure
https://pubchem.ncbi.nlm.nih.gov/compound/129798820#section=2D-Structure
https://pubchem.ncbi.nlm.nih.gov/compound/129798695#section=2D-Structure
https://pubchem.ncbi.nlm.nih.gov/compound/129798586#section=2D-Structure
https://pubchem.ncbi.nlm.nih.gov/compound/129798579#section=2D-Structure
https://pubchem.ncbi.nlm.nih.gov/compound/129798403#section=2D-Structure
https://pubchem.ncbi.nlm.nih.gov/compound/129798361#section=2D-Structure
https://pubchem.ncbi.nlm.nih.gov/compound/129798120#section=2D-Structure
https://pubchem.ncbi.nlm.nih.gov/compound/129797911#section=2D-Structure
https://pubchem.ncbi.nlm.nih.gov/compound/129797886#section=2D-Structure
https://pubchem.ncbi.nlm.nih.gov/compound/129797761#section=2D-Structure
https://pubchem.ncbi.nlm.nih.gov/compound/129797741#section=2D-Structure
https://pubchem.ncbi.nlm.nih.gov/compound/129797386#section=2D-Structure
https://pubchem.ncbi.nlm.nih.gov/compound/129797308#section=2D-Structure
https://pubchem.ncbi.nlm.nih.gov/compound/129797286#section=2D-Structure
https://pubchem.ncbi.nlm.nih.gov/compound/129797169#section=2D-Structure
https://pubchem.ncbi.nlm.nih.gov/compound/129797087#section=2D-Structure
https://pubchem.ncbi.nlm.nih.gov/compound/129796984#section=2D-Structure
https://pubchem.ncbi.nlm.nih.gov/compound/129796580#section=2D-Structure
https://pubchem.ncbi.nlm.nih.gov/compound/129796435#section=2D-Structure
https://pubchem.ncbi.nlm.nih.gov/compound/129796415#section=2D-Structure
https://pubchem.ncbi.nlm.nih.gov/compound/129796091#section=2D-Structure
https://pubchem.ncbi.nlm.nih.gov/compound/129795907#section=2D-Structure
https://pubchem.ncbi.nlm.nih.gov/compound/129795822#section=2D-Structure
https://pubchem.ncbi.nlm.nih.gov/compound/129795792#section=2D-Structure
https://pubchem.ncbi.nlm.nih.gov/compound/129795444#section=2D-Structure
https://pubchem.ncbi.nlm.nih.gov/compound/129795377#section=2D-Structure
https://pubchem.ncbi.nlm.nih.gov/compound/129794609#section=2D-Structure
https://pubchem.ncbi.nlm.nih.gov/compound/129794169#section=2D-Structure
https://pubchem.ncbi.nlm.nih.gov/compound/129794116#section=2D-Structure
https://pubchem.ncbi.nlm.nih.gov/compound/129793990#section=2D-Structure
https://pubchem.ncbi.nlm.nih.gov/compound/129793754#section=2D-Structure
https://pubchem.ncbi.nlm.nih.gov/compound/129793602#section=2D-Structure
https://pubchem.ncbi.nlm.nih.gov/compound/129793551#section=2D-Structure
https://pubchem.ncbi.nlm.nih.gov/compound/129793471#section=2D-Structure

https://pubchem.ncbi.nlm.nih.gov/compound/129793215#section=2D-Structure
https://pubchem.ncbi.nlm.nih.gov/compound/129793042#section=2D-Structure
https://pubchem.ncbi.nlm.nih.gov/compound/129792812#section=2D-Structure
https://pubchem.ncbi.nlm.nih.gov/compound/129792771#section=2D-Structure
https://pubchem.ncbi.nlm.nih.gov/compound/129792679#section=2D-Structure
https://pubchem.ncbi.nlm.nih.gov/compound/129792651#section=2D-Structure
https://pubchem.ncbi.nlm.nih.gov/compound/129792462#section=2D-Structure
https://pubchem.ncbi.nlm.nih.gov/compound/129792349#section=2D-Structure
https://pubchem.ncbi.nlm.nih.gov/compound/129792183#section=2D-Structure
https://pubchem.ncbi.nlm.nih.gov/compound/129792027#section=2D-Structure
https://pubchem.ncbi.nlm.nih.gov/compound/129791797#section=2D-Structure
https://pubchem.ncbi.nlm.nih.gov/compound/129791648#section=2D-Structure
https://pubchem.ncbi.nlm.nih.gov/compound/129791643#section=2D-Structure
https://pubchem.ncbi.nlm.nih.gov/compound/129791560#section=2D-Structure
https://pubchem.ncbi.nlm.nih.gov/compound/129791557#section=2D-Structure
https://pubchem.ncbi.nlm.nih.gov/compound/129791395#section=2D-Structure
https://pubchem.ncbi.nlm.nih.gov/compound/129791300#section=2D-Structure
https://pubchem.ncbi.nlm.nih.gov/compound/129791269#section=2D-Structure
https://pubchem.ncbi.nlm.nih.gov/compound/129791065#section=2D-Structure
https://pubchem.ncbi.nlm.nih.gov/compound/129791053#section=2D-Structure
https://pubchem.ncbi.nlm.nih.gov/compound/129791050#section=2D-Structure
https://pubchem.ncbi.nlm.nih.gov/compound/129790881#section=2D-Structure
https://pubchem.ncbi.nlm.nih.gov/compound/129790774#section=2D-Structure
https://pubchem.ncbi.nlm.nih.gov/compound/129790578#section=2D-Structure
https://pubchem.ncbi.nlm.nih.gov/compound/129790528#section=2D-Structure
https://pubchem.ncbi.nlm.nih.gov/compound/129790486#section=2D-Structure
https://pubchem.ncbi.nlm.nih.gov/compound/129790465#section=2D-Structure
https://pubchem.ncbi.nlm.nih.gov/compound/129790442#section=2D-Structure
https://pubchem.ncbi.nlm.nih.gov/compound/129790427#section=2D-Structure
https://pubchem.ncbi.nlm.nih.gov/compound/129790303#section=2D-Structure
https://pubchem.ncbi.nlm.nih.gov/compound/129790225#section=2D-Structure
https://pubchem.ncbi.nlm.nih.gov/compound/129790170#section=2D-Structure
https://pubchem.ncbi.nlm.nih.gov/compound/129790084#section=2D-Structure
https://pubchem.ncbi.nlm.nih.gov/compound/129790031#section=2D-Structure
https://pubchem.ncbi.nlm.nih.gov/compound/129789905#section=2D-Structure
https://pubchem.ncbi.nlm.nih.gov/compound/129789853#section=2D-Structure
https://pubchem.ncbi.nlm.nih.gov/compound/129789805#section=2D-Structure
https://pubchem.ncbi.nlm.nih.gov/compound/129789627#section=2D-Structure
https://pubchem.ncbi.nlm.nih.gov/compound/129789245#section=2D-Structure
https://pubchem.ncbi.nlm.nih.gov/compound/129789167#section=2D-Structure
https://pubchem.ncbi.nlm.nih.gov/compound/129789151#section=2D-Structure
https://pubchem.ncbi.nlm.nih.gov/compound/129788783#section=2D-Structure
https://pubchem.ncbi.nlm.nih.gov/compound/129788726#section=2D-Structure
https://pubchem.ncbi.nlm.nih.gov/compound/129788521#section=2D-Structure
https://pubchem.ncbi.nlm.nih.gov/compound/129788449#section=2D-Structure
https://pubchem.ncbi.nlm.nih.gov/compound/129788153#section=2D-Structure
https://pubchem.ncbi.nlm.nih.gov/compound/129788138#section=2D-Structure
https://pubchem.ncbi.nlm.nih.gov/compound/129787888#section=2D-Structure
https://pubchem.ncbi.nlm.nih.gov/compound/129787779#section=2D-Structure
https://pubchem.ncbi.nlm.nih.gov/compound/129787704#section=2D-Structure
https://pubchem.ncbi.nlm.nih.gov/compound/129787656#section=2D-Structure
https://pubchem.ncbi.nlm.nih.gov/compound/129787572#section=2D-Structure
https://pubchem.ncbi.nlm.nih.gov/compound/129787492#section=2D-Structure
https://pubchem.ncbi.nlm.nih.gov/compound/129787324#section=2D-Structure
https://pubchem.ncbi.nlm.nih.gov/compound/129787316#section=2D-Structure

https://pubchem.ncbi.nlm.nih.gov/compound/129787263#section=2D-Structure
https://pubchem.ncbi.nlm.nih.gov/compound/129787215#section=2D-Structure
https://pubchem.ncbi.nlm.nih.gov/compound/129787204#section=2D-Structure
https://pubchem.ncbi.nlm.nih.gov/compound/129786984#section=2D-Structure
https://pubchem.ncbi.nlm.nih.gov/compound/129786930#section=2D-Structure
https://pubchem.ncbi.nlm.nih.gov/compound/129786668#section=2D-Structure
https://pubchem.ncbi.nlm.nih.gov/compound/129786493#section=2D-Structure
https://pubchem.ncbi.nlm.nih.gov/compound/129786449#section=2D-Structure
https://pubchem.ncbi.nlm.nih.gov/compound/129786197#section=2D-Structure
https://pubchem.ncbi.nlm.nih.gov/compound/129786154#section=2D-Structure
https://pubchem.ncbi.nlm.nih.gov/compound/129786140#section=2D-Structure
https://pubchem.ncbi.nlm.nih.gov/compound/129786033#section=2D-Structure
https://pubchem.ncbi.nlm.nih.gov/compound/129785985#section=2D-Structure
https://pubchem.ncbi.nlm.nih.gov/compound/129785899#section=2D-Structure
https://pubchem.ncbi.nlm.nih.gov/compound/129785826#section=2D-Structure
https://pubchem.ncbi.nlm.nih.gov/compound/129785800#section=2D-Structure
https://pubchem.ncbi.nlm.nih.gov/compound/129785757#section=2D-Structure
https://pubchem.ncbi.nlm.nih.gov/compound/129785670#section=2D-Structure
https://pubchem.ncbi.nlm.nih.gov/compound/129785633#section=2D-Structure
https://pubchem.ncbi.nlm.nih.gov/compound/129785490#section=2D-Structure
https://pubchem.ncbi.nlm.nih.gov/compound/129785441#section=2D-Structure
https://pubchem.ncbi.nlm.nih.gov/compound/129785158#section=2D-Structure
https://pubchem.ncbi.nlm.nih.gov/compound/129785117#section=2D-Structure
https://pubchem.ncbi.nlm.nih.gov/compound/129785097#section=2D-Structure
https://pubchem.ncbi.nlm.nih.gov/compound/129785079#section=2D-Structure
https://pubchem.ncbi.nlm.nih.gov/compound/129785018#section=2D-Structure
https://pubchem.ncbi.nlm.nih.gov/compound/129784963#section=2D-Structure
https://pubchem.ncbi.nlm.nih.gov/compound/129784951#section=2D-Structure
https://pubchem.ncbi.nlm.nih.gov/compound/129784649#section=2D-Structure
https://pubchem.ncbi.nlm.nih.gov/compound/129784500#section=2D-Structure
https://pubchem.ncbi.nlm.nih.gov/compound/129784350#section=2D-Structure
https://pubchem.ncbi.nlm.nih.gov/compound/129784332#section=2D-Structure
https://pubchem.ncbi.nlm.nih.gov/compound/129784053#section=2D-Structure
https://pubchem.ncbi.nlm.nih.gov/compound/129783925#section=2D-Structure
https://pubchem.ncbi.nlm.nih.gov/compound/129783550#section=2D-Structure
https://pubchem.ncbi.nlm.nih.gov/compound/129783521#section=2D-Structure
https://pubchem.ncbi.nlm.nih.gov/compound/129783482#section=2D-Structure
https://pubchem.ncbi.nlm.nih.gov/compound/129783172#section=2D-Structure
https://pubchem.ncbi.nlm.nih.gov/compound/129783037#section=2D-Structure
https://pubchem.ncbi.nlm.nih.gov/compound/129783006#section=2D-Structure
https://pubchem.ncbi.nlm.nih.gov/compound/129782975#section=2D-Structure
https://pubchem.ncbi.nlm.nih.gov/compound/129782681#section=2D-Structure
https://pubchem.ncbi.nlm.nih.gov/compound/129782537#section=2D-Structure
https://pubchem.ncbi.nlm.nih.gov/compound/129782486#section=2D-Structure
https://pubchem.ncbi.nlm.nih.gov/compound/129782474#section=2D-Structure
https://pubchem.ncbi.nlm.nih.gov/compound/129782465#section=2D-Structure
https://pubchem.ncbi.nlm.nih.gov/compound/129782358#section=2D-Structure
https://pubchem.ncbi.nlm.nih.gov/compound/129782230#section=2D-Structure
https://pubchem.ncbi.nlm.nih.gov/compound/129782213#section=2D-Structure
https://pubchem.ncbi.nlm.nih.gov/compound/129782169#section=2D-Structure
https://pubchem.ncbi.nlm.nih.gov/compound/129781803#section=2D-Structure
https://pubchem.ncbi.nlm.nih.gov/compound/129781759#section=2D-Structure
https://pubchem.ncbi.nlm.nih.gov/compound/129781536#section=2D-Structure
https://pubchem.ncbi.nlm.nih.gov/compound/129781457#section=2D-Structure
https://pubchem.ncbi.nlm.nih.gov/compound/129781416#section=2D-Structure

https://pubchem.ncbi.nlm.nih.gov/compound/129781225#section=2D-Structure
https://pubchem.ncbi.nlm.nih.gov/compound/129781211#section=2D-Structure
https://pubchem.ncbi.nlm.nih.gov/compound/129781166#section=2D-Structure
https://pubchem.ncbi.nlm.nih.gov/compound/129781145#section=2D-Structure
https://pubchem.ncbi.nlm.nih.gov/compound/129781083#section=2D-Structure
https://pubchem.ncbi.nlm.nih.gov/compound/129780899#section=2D-Structure
https://pubchem.ncbi.nlm.nih.gov/compound/129780891#section=2D-Structure
https://pubchem.ncbi.nlm.nih.gov/compound/129780853#section=2D-Structure
https://pubchem.ncbi.nlm.nih.gov/compound/129780777#section=2D-Structure
https://pubchem.ncbi.nlm.nih.gov/compound/129780198#section=2D-Structure
https://pubchem.ncbi.nlm.nih.gov/compound/129780060#section=2D-Structure
https://pubchem.ncbi.nlm.nih.gov/compound/129779843#section=2D-Structure
https://pubchem.ncbi.nlm.nih.gov/compound/129779823#section=2D-Structure
https://pubchem.ncbi.nlm.nih.gov/compound/129779188#section=2D-Structure
https://pubchem.ncbi.nlm.nih.gov/compound/129779130#section=2D-Structure
https://pubchem.ncbi.nlm.nih.gov/compound/129779110#section=2D-Structure
https://pubchem.ncbi.nlm.nih.gov/compound/129779042#section=2D-Structure
https://pubchem.ncbi.nlm.nih.gov/compound/129778320#section=2D-Structure
https://pubchem.ncbi.nlm.nih.gov/compound/129777943#section=2D-Structure
https://pubchem.ncbi.nlm.nih.gov/compound/129777770#section=2D-Structure
https://pubchem.ncbi.nlm.nih.gov/compound/129777546#section=2D-Structure
https://pubchem.ncbi.nlm.nih.gov/compound/129777153#section=2D-Structure
https://pubchem.ncbi.nlm.nih.gov/compound/129776938#section=2D-Structure
https://pubchem.ncbi.nlm.nih.gov/compound/129775150#section=2D-Structure
https://pubchem.ncbi.nlm.nih.gov/compound/129774029#section=2D-Structure
https://pubchem.ncbi.nlm.nih.gov/compound/129773627#section=2D-Structure
https://pubchem.ncbi.nlm.nih.gov/compound/129773008#section=2D-Structure
https://pubchem.ncbi.nlm.nih.gov/compound/129773001#section=2D-Structure
https://pubchem.ncbi.nlm.nih.gov/compound/129772468#section=2D-Structure
https://pubchem.ncbi.nlm.nih.gov/compound/129772457#section=2D-Structure
https://pubchem.ncbi.nlm.nih.gov/compound/129771965#section=2D-Structure
https://pubchem.ncbi.nlm.nih.gov/compound/129771868#section=2D-Structure
https://pubchem.ncbi.nlm.nih.gov/compound/129771686#section=2D-Structure
https://pubchem.ncbi.nlm.nih.gov/compound/129770059#section=2D-Structure
https://pubchem.ncbi.nlm.nih.gov/compound/129769760#section=2D-Structure
https://pubchem.ncbi.nlm.nih.gov/compound/129769043#section=2D-Structure
https://pubchem.ncbi.nlm.nih.gov/compound/129768201#section=2D-Structure
https://pubchem.ncbi.nlm.nih.gov/compound/129768060#section=2D-Structure
https://pubchem.ncbi.nlm.nih.gov/compound/129767271#section=2D-Structure
https://pubchem.ncbi.nlm.nih.gov/compound/129765402#section=2D-Structure
https://pubchem.ncbi.nlm.nih.gov/compound/129765346#section=2D-Structure
https://pubchem.ncbi.nlm.nih.gov/compound/129764861#section=2D-Structure
https://pubchem.ncbi.nlm.nih.gov/compound/129763086#section=2D-Structure
https://pubchem.ncbi.nlm.nih.gov/compound/129761920#section=2D-Structure
https://pubchem.ncbi.nlm.nih.gov/compound/129761484#section=2D-Structure
https://pubchem.ncbi.nlm.nih.gov/compound/129761138#section=2D-Structure
https://pubchem.ncbi.nlm.nih.gov/compound/129759880#section=2D-Structure
https://pubchem.ncbi.nlm.nih.gov/compound/129759774#section=2D-Structure
https://pubchem.ncbi.nlm.nih.gov/compound/129758414#section=2D-Structure
https://pubchem.ncbi.nlm.nih.gov/compound/129758278#section=2D-Structure
https://pubchem.ncbi.nlm.nih.gov/compound/129757394#section=2D-Structure
https://pubchem.ncbi.nlm.nih.gov/compound/129756988#section=2D-Structure
https://pubchem.ncbi.nlm.nih.gov/compound/129756617#section=2D-Structure
https://pubchem.ncbi.nlm.nih.gov/compound/129756546#section=2D-Structure
https://pubchem.ncbi.nlm.nih.gov/compound/129756485#section=2D-Structure

https://pubchem.ncbi.nlm.nih.gov/compound/129756239#section=2D-Structure
https://pubchem.ncbi.nlm.nih.gov/compound/129755782#section=2D-Structure
https://pubchem.ncbi.nlm.nih.gov/compound/129754444#section=2D-Structure
https://pubchem.ncbi.nlm.nih.gov/compound/129754437#section=2D-Structure
https://pubchem.ncbi.nlm.nih.gov/compound/129754379#section=2D-Structure
https://pubchem.ncbi.nlm.nih.gov/compound/129753602#section=2D-Structure
https://pubchem.ncbi.nlm.nih.gov/compound/129753346#section=2D-Structure
https://pubchem.ncbi.nlm.nih.gov/compound/129752840#section=2D-Structure
https://pubchem.ncbi.nlm.nih.gov/compound/129752664#section=2D-Structure
https://pubchem.ncbi.nlm.nih.gov/compound/129752244#section=2D-Structure
https://pubchem.ncbi.nlm.nih.gov/compound/129751832#section=2D-Structure
https://pubchem.ncbi.nlm.nih.gov/compound/129751293#section=2D-Structure
https://pubchem.ncbi.nlm.nih.gov/compound/129751184#section=2D-Structure
https://pubchem.ncbi.nlm.nih.gov/compound/129751171#section=2D-Structure
https://pubchem.ncbi.nlm.nih.gov/compound/129751113#section=2D-Structure
https://pubchem.ncbi.nlm.nih.gov/compound/129751073#section=2D-Structure
https://pubchem.ncbi.nlm.nih.gov/compound/129750782#section=2D-Structure
https://pubchem.ncbi.nlm.nih.gov/compound/129746449#section=2D-Structure
https://pubchem.ncbi.nlm.nih.gov/compound/129746067#section=2D-Structure
https://pubchem.ncbi.nlm.nih.gov/compound/129745290#section=2D-Structure
https://pubchem.ncbi.nlm.nih.gov/compound/129744738#section=2D-Structure
https://pubchem.ncbi.nlm.nih.gov/compound/129743699#section=2D-Structure
https://pubchem.ncbi.nlm.nih.gov/compound/129743142#section=2D-Structure
https://pubchem.ncbi.nlm.nih.gov/compound/129742945#section=2D-Structure
https://pubchem.ncbi.nlm.nih.gov/compound/129742505#section=2D-Structure
https://pubchem.ncbi.nlm.nih.gov/compound/129741743#section=2D-Structure
https://pubchem.ncbi.nlm.nih.gov/compound/129740721#section=2D-Structure
https://pubchem.ncbi.nlm.nih.gov/compound/129740237#section=2D-Structure
https://pubchem.ncbi.nlm.nih.gov/compound/129740212#section=2D-Structure
https://pubchem.ncbi.nlm.nih.gov/compound/129739869#section=2D-Structure
https://pubchem.ncbi.nlm.nih.gov/compound/129739463#section=2D-Structure
https://pubchem.ncbi.nlm.nih.gov/compound/129739123#section=2D-Structure
https://pubchem.ncbi.nlm.nih.gov/compound/129739042#section=2D-Structure
https://pubchem.ncbi.nlm.nih.gov/compound/129738821#section=2D-Structure
https://pubchem.ncbi.nlm.nih.gov/compound/129738522#section=2D-Structure
https://pubchem.ncbi.nlm.nih.gov/compound/129738493#section=2D-Structure
https://pubchem.ncbi.nlm.nih.gov/compound/129738444#section=2D-Structure
https://pubchem.ncbi.nlm.nih.gov/compound/129738275#section=2D-Structure
https://pubchem.ncbi.nlm.nih.gov/compound/129738086#section=2D-Structure
https://pubchem.ncbi.nlm.nih.gov/compound/129737589#section=2D-Structure
https://pubchem.ncbi.nlm.nih.gov/compound/129737367#section=2D-Structure
https://pubchem.ncbi.nlm.nih.gov/compound/129736969#section=2D-Structure
https://pubchem.ncbi.nlm.nih.gov/compound/129736963#section=2D-Structure
https://pubchem.ncbi.nlm.nih.gov/compound/129736858#section=2D-Structure
https://pubchem.ncbi.nlm.nih.gov/compound/129736573#section=2D-Structure
https://pubchem.ncbi.nlm.nih.gov/compound/129736451#section=2D-Structure
https://pubchem.ncbi.nlm.nih.gov/compound/129736434#section=2D-Structure
https://pubchem.ncbi.nlm.nih.gov/compound/129736324#section=2D-Structure
https://pubchem.ncbi.nlm.nih.gov/compound/129735799#section=2D-Structure
https://pubchem.ncbi.nlm.nih.gov/compound/129735211#section=2D-Structure
https://pubchem.ncbi.nlm.nih.gov/compound/129734419#section=2D-Structure
https://pubchem.ncbi.nlm.nih.gov/compound/129734206#section=2D-Structure
https://pubchem.ncbi.nlm.nih.gov/compound/129733939#section=2D-Structure
https://pubchem.ncbi.nlm.nih.gov/compound/129733753#section=2D-Structure
https://pubchem.ncbi.nlm.nih.gov/compound/129733246#section=2D-Structure

https://pubchem.ncbi.nlm.nih.gov/compound/129732849#section=2D-Structure
https://pubchem.ncbi.nlm.nih.gov/compound/129732714#section=2D-Structure
https://pubchem.ncbi.nlm.nih.gov/compound/129732635#section=2D-Structure
https://pubchem.ncbi.nlm.nih.gov/compound/129732532#section=2D-Structure
https://pubchem.ncbi.nlm.nih.gov/compound/129732430#section=2D-Structure
https://pubchem.ncbi.nlm.nih.gov/compound/129732351#section=2D-Structure
https://pubchem.ncbi.nlm.nih.gov/compound/129732303#section=2D-Structure
https://pubchem.ncbi.nlm.nih.gov/compound/129732063#section=2D-Structure
https://pubchem.ncbi.nlm.nih.gov/compound/129730448#section=2D-Structure
https://pubchem.ncbi.nlm.nih.gov/compound/129729650#section=2D-Structure
https://pubchem.ncbi.nlm.nih.gov/compound/129724623#section=2D-Structure
https://pubchem.ncbi.nlm.nih.gov/compound/129721416#section=2D-Structure
https://pubchem.ncbi.nlm.nih.gov/compound/129721091#section=2D-Structure
https://pubchem.ncbi.nlm.nih.gov/compound/129718908#section=2D-Structure
https://pubchem.ncbi.nlm.nih.gov/compound/129718728#section=2D-Structure
https://pubchem.ncbi.nlm.nih.gov/compound/129718308#section=2D-Structure
https://pubchem.ncbi.nlm.nih.gov/compound/129717795#section=2D-Structure
https://pubchem.ncbi.nlm.nih.gov/compound/129715632#section=2D-Structure
https://pubchem.ncbi.nlm.nih.gov/compound/129715466#section=2D-Structure
https://pubchem.ncbi.nlm.nih.gov/compound/129713513#section=2D-Structure
https://pubchem.ncbi.nlm.nih.gov/compound/129713430#section=2D-Structure
https://pubchem.ncbi.nlm.nih.gov/compound/129713420#section=2D-Structure
https://pubchem.ncbi.nlm.nih.gov/compound/129713413#section=2D-Structure
https://pubchem.ncbi.nlm.nih.gov/compound/129713405#section=2D-Structure
https://pubchem.ncbi.nlm.nih.gov/compound/129713339#section=2D-Structure
https://pubchem.ncbi.nlm.nih.gov/compound/129713056#section=2D-Structure
https://pubchem.ncbi.nlm.nih.gov/compound/129712801#section=2D-Structure
https://pubchem.ncbi.nlm.nih.gov/compound/129712711#section=2D-Structure
https://pubchem.ncbi.nlm.nih.gov/compound/129712421#section=2D-Structure
https://pubchem.ncbi.nlm.nih.gov/compound/129710875#section=2D-Structure
https://pubchem.ncbi.nlm.nih.gov/compound/129710694#section=2D-Structure
https://pubchem.ncbi.nlm.nih.gov/compound/129710688#section=2D-Structure
https://pubchem.ncbi.nlm.nih.gov/compound/129710526#section=2D-Structure
https://pubchem.ncbi.nlm.nih.gov/compound/129710375#section=2D-Structure
https://pubchem.ncbi.nlm.nih.gov/compound/129710373#section=2D-Structure
https://pubchem.ncbi.nlm.nih.gov/compound/129710340#section=2D-Structure
https://pubchem.ncbi.nlm.nih.gov/compound/129710235#section=2D-Structure
https://pubchem.ncbi.nlm.nih.gov/compound/129709676#section=2D-Structure
https://pubchem.ncbi.nlm.nih.gov/compound/129708815#section=2D-Structure
https://pubchem.ncbi.nlm.nih.gov/compound/129708621#section=2D-Structure
https://pubchem.ncbi.nlm.nih.gov/compound/129708524#section=2D-Structure
https://pubchem.ncbi.nlm.nih.gov/compound/129708289#section=2D-Structure
https://pubchem.ncbi.nlm.nih.gov/compound/129708276#section=2D-Structure
https://pubchem.ncbi.nlm.nih.gov/compound/129707857#section=2D-Structure
https://pubchem.ncbi.nlm.nih.gov/compound/129707827#section=2D-Structure
https://pubchem.ncbi.nlm.nih.gov/compound/129707621#section=2D-Structure
https://pubchem.ncbi.nlm.nih.gov/compound/129707600#section=2D-Structure
https://pubchem.ncbi.nlm.nih.gov/compound/129707510#section=2D-Structure
https://pubchem.ncbi.nlm.nih.gov/compound/129707398#section=2D-Structure
https://pubchem.ncbi.nlm.nih.gov/compound/129707027#section=2D-Structure
https://pubchem.ncbi.nlm.nih.gov/compound/129706615#section=2D-Structure
https://pubchem.ncbi.nlm.nih.gov/compound/129706419#section=2D-Structure
https://pubchem.ncbi.nlm.nih.gov/compound/129706333#section=2D-Structure
https://pubchem.ncbi.nlm.nih.gov/compound/129706252#section=2D-Structure
https://pubchem.ncbi.nlm.nih.gov/compound/129706230#section=2D-Structure

https://pubchem.ncbi.nlm.nih.gov/compound/129706075#section=2D-Structure
https://pubchem.ncbi.nlm.nih.gov/compound/129705995#section=2D-Structure
https://pubchem.ncbi.nlm.nih.gov/compound/129705837#section=2D-Structure
https://pubchem.ncbi.nlm.nih.gov/compound/129705729#section=2D-Structure
https://pubchem.ncbi.nlm.nih.gov/compound/129704984#section=2D-Structure
https://pubchem.ncbi.nlm.nih.gov/compound/129704222#section=2D-Structure
https://pubchem.ncbi.nlm.nih.gov/compound/129701966#section=2D-Structure
https://pubchem.ncbi.nlm.nih.gov/compound/129701789#section=2D-Structure
https://pubchem.ncbi.nlm.nih.gov/compound/129700771#section=2D-Structure
https://pubchem.ncbi.nlm.nih.gov/compound/129699889#section=2D-Structure
https://pubchem.ncbi.nlm.nih.gov/compound/129699780#section=2D-Structure
https://pubchem.ncbi.nlm.nih.gov/compound/129696654#section=2D-Structure
https://pubchem.ncbi.nlm.nih.gov/compound/129695706#section=2D-Structure
https://pubchem.ncbi.nlm.nih.gov/compound/129694662#section=2D-Structure
https://pubchem.ncbi.nlm.nih.gov/compound/129694261#section=2D-Structure
https://pubchem.ncbi.nlm.nih.gov/compound/129693256#section=2D-Structure
https://pubchem.ncbi.nlm.nih.gov/compound/129692361#section=2D-Structure
https://pubchem.ncbi.nlm.nih.gov/compound/129691884#section=2D-Structure
https://pubchem.ncbi.nlm.nih.gov/compound/129691857#section=2D-Structure
https://pubchem.ncbi.nlm.nih.gov/compound/129691291#section=2D-Structure
https://pubchem.ncbi.nlm.nih.gov/compound/129690966#section=2D-Structure
https://pubchem.ncbi.nlm.nih.gov/compound/129687500#section=2D-Structure
https://pubchem.ncbi.nlm.nih.gov/compound/129687193#section=2D-Structure
https://pubchem.ncbi.nlm.nih.gov/compound/129686796#section=2D-Structure
https://pubchem.ncbi.nlm.nih.gov/compound/129686552#section=2D-Structure
https://pubchem.ncbi.nlm.nih.gov/compound/129686415#section=2D-Structure
https://pubchem.ncbi.nlm.nih.gov/compound/129681759#section=2D-Structure
https://pubchem.ncbi.nlm.nih.gov/compound/129679939#section=2D-Structure
https://pubchem.ncbi.nlm.nih.gov/compound/129678503#section=2D-Structure
https://pubchem.ncbi.nlm.nih.gov/compound/129677264#section=2D-Structure
https://pubchem.ncbi.nlm.nih.gov/compound/129677181#section=2D-Structure
https://pubchem.ncbi.nlm.nih.gov/compound/129676861#section=2D-Structure
https://pubchem.ncbi.nlm.nih.gov/compound/129676826#section=2D-Structure
https://pubchem.ncbi.nlm.nih.gov/compound/129676816#section=2D-Structure
https://pubchem.ncbi.nlm.nih.gov/compound/129676812#section=2D-Structure
https://pubchem.ncbi.nlm.nih.gov/compound/129676375#section=2D-Structure
https://pubchem.ncbi.nlm.nih.gov/compound/129675893#section=2D-Structure
https://pubchem.ncbi.nlm.nih.gov/compound/129674962#section=2D-Structure
https://pubchem.ncbi.nlm.nih.gov/compound/129673795#section=2D-Structure
https://pubchem.ncbi.nlm.nih.gov/compound/129672216#section=2D-Structure
https://pubchem.ncbi.nlm.nih.gov/compound/129672004#section=2D-Structure
https://pubchem.ncbi.nlm.nih.gov/compound/129671304#section=2D-Structure
https://pubchem.ncbi.nlm.nih.gov/compound/129670468#section=2D-Structure
https://pubchem.ncbi.nlm.nih.gov/compound/129670406#section=2D-Structure
https://pubchem.ncbi.nlm.nih.gov/compound/129669754#section=2D-Structure
https://pubchem.ncbi.nlm.nih.gov/compound/129669104#section=2D-Structure
https://pubchem.ncbi.nlm.nih.gov/compound/129668101#section=2D-Structure
https://pubchem.ncbi.nlm.nih.gov/compound/129667870#section=2D-Structure
https://pubchem.ncbi.nlm.nih.gov/compound/129665727#section=2D-Structure
https://pubchem.ncbi.nlm.nih.gov/compound/129664963#section=2D-Structure
https://pubchem.ncbi.nlm.nih.gov/compound/129664649#section=2D-Structure
https://pubchem.ncbi.nlm.nih.gov/compound/129664605#section=2D-Structure
https://pubchem.ncbi.nlm.nih.gov/compound/129657930#section=2D-Structure
https://pubchem.ncbi.nlm.nih.gov/compound/129656502#section=2D-Structure
https://pubchem.ncbi.nlm.nih.gov/compound/129655833#section=2D-Structure

https://pubchem.ncbi.nlm.nih.gov/compound/129655773#section=2D-Structure
https://pubchem.ncbi.nlm.nih.gov/compound/129655251#section=2D-Structure
https://pubchem.ncbi.nlm.nih.gov/compound/129654238#section=2D-Structure
https://pubchem.ncbi.nlm.nih.gov/compound/129653816#section=2D-Structure
https://pubchem.ncbi.nlm.nih.gov/compound/129653508#section=2D-Structure
https://pubchem.ncbi.nlm.nih.gov/compound/129652905#section=2D-Structure
https://pubchem.ncbi.nlm.nih.gov/compound/129652791#section=2D-Structure
https://pubchem.ncbi.nlm.nih.gov/compound/129652751#section=2D-Structure
https://pubchem.ncbi.nlm.nih.gov/compound/129652740#section=2D-Structure
https://pubchem.ncbi.nlm.nih.gov/compound/129652375#section=2D-Structure
https://pubchem.ncbi.nlm.nih.gov/compound/129651813#section=2D-Structure
https://pubchem.ncbi.nlm.nih.gov/compound/129651533#section=2D-Structure
https://pubchem.ncbi.nlm.nih.gov/compound/129651512#section=2D-Structure
https://pubchem.ncbi.nlm.nih.gov/compound/129651052#section=2D-Structure
https://pubchem.ncbi.nlm.nih.gov/compound/129651047#section=2D-Structure
https://pubchem.ncbi.nlm.nih.gov/compound/129650989#section=2D-Structure
https://pubchem.ncbi.nlm.nih.gov/compound/129650567#section=2D-Structure
https://pubchem.ncbi.nlm.nih.gov/compound/129649555#section=2D-Structure
https://pubchem.ncbi.nlm.nih.gov/compound/129649484#section=2D-Structure
https://pubchem.ncbi.nlm.nih.gov/compound/129648666#section=2D-Structure
https://pubchem.ncbi.nlm.nih.gov/compound/129648137#section=2D-Structure
https://pubchem.ncbi.nlm.nih.gov/compound/129648117#section=2D-Structure
https://pubchem.ncbi.nlm.nih.gov/compound/129647816#section=2D-Structure
https://pubchem.ncbi.nlm.nih.gov/compound/129647693#section=2D-Structure
https://pubchem.ncbi.nlm.nih.gov/compound/129646995#section=2D-Structure
https://pubchem.ncbi.nlm.nih.gov/compound/129646973#section=2D-Structure
https://pubchem.ncbi.nlm.nih.gov/compound/129646741#section=2D-Structure
https://pubchem.ncbi.nlm.nih.gov/compound/129646726#section=2D-Structure
https://pubchem.ncbi.nlm.nih.gov/compound/129646440#section=2D-Structure
https://pubchem.ncbi.nlm.nih.gov/compound/129646389#section=2D-Structure
https://pubchem.ncbi.nlm.nih.gov/compound/129645459#section=2D-Structure
https://pubchem.ncbi.nlm.nih.gov/compound/129644291#section=2D-Structure
https://pubchem.ncbi.nlm.nih.gov/compound/129643970#section=2D-Structure
https://pubchem.ncbi.nlm.nih.gov/compound/129643833#section=2D-Structure
https://pubchem.ncbi.nlm.nih.gov/compound/129643222#section=2D-Structure
https://pubchem.ncbi.nlm.nih.gov/compound/129642533#section=2D-Structure
https://pubchem.ncbi.nlm.nih.gov/compound/129642151#section=2D-Structure
https://pubchem.ncbi.nlm.nih.gov/compound/129642113#section=2D-Structure
https://pubchem.ncbi.nlm.nih.gov/compound/129642097#section=2D-Structure
https://pubchem.ncbi.nlm.nih.gov/compound/129638268#section=2D-Structure
https://pubchem.ncbi.nlm.nih.gov/compound/129636957#section=2D-Structure
https://pubchem.ncbi.nlm.nih.gov/compound/129634464#section=2D-Structure
https://pubchem.ncbi.nlm.nih.gov/compound/129632179#section=2D-Structure
https://pubchem.ncbi.nlm.nih.gov/compound/129631749#section=2D-Structure
https://pubchem.ncbi.nlm.nih.gov/compound/129631468#section=2D-Structure
https://pubchem.ncbi.nlm.nih.gov/compound/129631421#section=2D-Structure
https://pubchem.ncbi.nlm.nih.gov/compound/129630965#section=2D-Structure
https://pubchem.ncbi.nlm.nih.gov/compound/129630784#section=2D-Structure
https://pubchem.ncbi.nlm.nih.gov/compound/129628989#section=2D-Structure
https://pubchem.ncbi.nlm.nih.gov/compound/129627838#section=2D-Structure
https://pubchem.ncbi.nlm.nih.gov/compound/129601470#section=2D-Structure
https://pubchem.ncbi.nlm.nih.gov/compound/12955918#section=2D-Structure
https://pubchem.ncbi.nlm.nih.gov/compound/12948612#section=2D-Structure
https://pubchem.ncbi.nlm.nih.gov/compound/12936350#section=2D-Structure
https://pubchem.ncbi.nlm.nih.gov/compound/129317059#section=2D-Structure

https://pubchem.ncbi.nlm.nih.gov/compound/129314727#section=2D-Structure
https://pubchem.ncbi.nlm.nih.gov/compound/129311838#section=2D-Structure
https://pubchem.ncbi.nlm.nih.gov/compound/129309016#section=2D-Structure
https://pubchem.ncbi.nlm.nih.gov/compound/129305297#section=2D-Structure
https://pubchem.ncbi.nlm.nih.gov/compound/129301079#section=2D-Structure
https://pubchem.ncbi.nlm.nih.gov/compound/129275615#section=2D-Structure
https://pubchem.ncbi.nlm.nih.gov/compound/129258708#section=2D-Structure
https://pubchem.ncbi.nlm.nih.gov/compound/129257378#section=2D-Structure
https://pubchem.ncbi.nlm.nih.gov/compound/129254004#section=2D-Structure
https://pubchem.ncbi.nlm.nih.gov/compound/129253990#section=2D-Structure
https://pubchem.ncbi.nlm.nih.gov/compound/129252647#section=2D-Structure
https://pubchem.ncbi.nlm.nih.gov/compound/129249521#section=2D-Structure
https://pubchem.ncbi.nlm.nih.gov/compound/129246256#section=2D-Structure
https://pubchem.ncbi.nlm.nih.gov/compound/129234797#section=2D-Structure
https://pubchem.ncbi.nlm.nih.gov/compound/129219632#section=2D-Structure
https://pubchem.ncbi.nlm.nih.gov/compound/129215728#section=2D-Structure
https://pubchem.ncbi.nlm.nih.gov/compound/129214831#section=2D-Structure
https://pubchem.ncbi.nlm.nih.gov/compound/129212578#section=2D-Structure
https://pubchem.ncbi.nlm.nih.gov/compound/129203127#section=2D-Structure
https://pubchem.ncbi.nlm.nih.gov/compound/129201516#section=2D-Structure
https://pubchem.ncbi.nlm.nih.gov/compound/129197539#section=2D-Structure
https://pubchem.ncbi.nlm.nih.gov/compound/129196365#section=2D-Structure
https://pubchem.ncbi.nlm.nih.gov/compound/129195589#section=2D-Structure
https://pubchem.ncbi.nlm.nih.gov/compound/129192994#section=2D-Structure
https://pubchem.ncbi.nlm.nih.gov/compound/129192231#section=2D-Structure
https://pubchem.ncbi.nlm.nih.gov/compound/129191238#section=2D-Structure
https://pubchem.ncbi.nlm.nih.gov/compound/129191201#section=2D-Structure
https://pubchem.ncbi.nlm.nih.gov/compound/129185471#section=2D-Structure
https://pubchem.ncbi.nlm.nih.gov/compound/129185459#section=2D-Structure
https://pubchem.ncbi.nlm.nih.gov/compound/129178398#section=2D-Structure
https://pubchem.ncbi.nlm.nih.gov/compound/129178003#section=2D-Structure
https://pubchem.ncbi.nlm.nih.gov/compound/129177995#section=2D-Structure
https://pubchem.ncbi.nlm.nih.gov/compound/129177622#section=2D-Structure
https://pubchem.ncbi.nlm.nih.gov/compound/129163716#section=2D-Structure
https://pubchem.ncbi.nlm.nih.gov/compound/129162184#section=2D-Structure
https://pubchem.ncbi.nlm.nih.gov/compound/129161149#section=2D-Structure
https://pubchem.ncbi.nlm.nih.gov/compound/129151831#section=2D-Structure
https://pubchem.ncbi.nlm.nih.gov/compound/129146656#section=2D-Structure
https://pubchem.ncbi.nlm.nih.gov/compound/129146571#section=2D-Structure
https://pubchem.ncbi.nlm.nih.gov/compound/129146222#section=2D-Structure
https://pubchem.ncbi.nlm.nih.gov/compound/129136506#section=2D-Structure
https://pubchem.ncbi.nlm.nih.gov/compound/129119743#section=2D-Structure
https://pubchem.ncbi.nlm.nih.gov/compound/129119742#section=2D-Structure
https://pubchem.ncbi.nlm.nih.gov/compound/129119739#section=2D-Structure
https://pubchem.ncbi.nlm.nih.gov/compound/129119738#section=2D-Structure
https://pubchem.ncbi.nlm.nih.gov/compound/129112676#section=2D-Structure
https://pubchem.ncbi.nlm.nih.gov/compound/129106605#section=2D-Structure
https://pubchem.ncbi.nlm.nih.gov/compound/12910555#section=2D-Structure
https://pubchem.ncbi.nlm.nih.gov/compound/129103670#section=2D-Structure
https://pubchem.ncbi.nlm.nih.gov/compound/129076542#section=2D-Structure
https://pubchem.ncbi.nlm.nih.gov/compound/129068252#section=2D-Structure
https://pubchem.ncbi.nlm.nih.gov/compound/129060224#section=2D-Structure
https://pubchem.ncbi.nlm.nih.gov/compound/129057100#section=2D-Structure
https://pubchem.ncbi.nlm.nih.gov/compound/129044407#section=2D-Structure
https://pubchem.ncbi.nlm.nih.gov/compound/129034736#section=2D-Structure

https://pubchem.ncbi.nlm.nih.gov/compound/129033243#section=2D-Structure
https://pubchem.ncbi.nlm.nih.gov/compound/12868612#section=2D-Structure
https://pubchem.ncbi.nlm.nih.gov/compound/12857734#section=2D-Structure
https://pubchem.ncbi.nlm.nih.gov/compound/12828912#section=2D-Structure
https://pubchem.ncbi.nlm.nih.gov/compound/12792706#section=2D-Structure
https://pubchem.ncbi.nlm.nih.gov/compound/12792702#section=2D-Structure
https://pubchem.ncbi.nlm.nih.gov/compound/1278834#section=2D-Structure
https://pubchem.ncbi.nlm.nih.gov/compound/12782234#section=2D-Structure
https://pubchem.ncbi.nlm.nih.gov/compound/12782222#section=2D-Structure
https://pubchem.ncbi.nlm.nih.gov/compound/12778534#section=2D-Structure
https://pubchem.ncbi.nlm.nih.gov/compound/12778533#section=2D-Structure
https://pubchem.ncbi.nlm.nih.gov/compound/12768971#section=2D-Structure
https://pubchem.ncbi.nlm.nih.gov/compound/12764776#section=2D-Structure
https://pubchem.ncbi.nlm.nih.gov/compound/12764775#section=2D-Structure
https://pubchem.ncbi.nlm.nih.gov/compound/12761532#section=2D-Structure
https://pubchem.ncbi.nlm.nih.gov/compound/12761293#section=2D-Structure
https://pubchem.ncbi.nlm.nih.gov/compound/12761290#section=2D-Structure
https://pubchem.ncbi.nlm.nih.gov/compound/12759679#section=2D-Structure
https://pubchem.ncbi.nlm.nih.gov/compound/12745687#section=2D-Structure
https://pubchem.ncbi.nlm.nih.gov/compound/12745686#section=2D-Structure
https://pubchem.ncbi.nlm.nih.gov/compound/12718125#section=2D-Structure
https://pubchem.ncbi.nlm.nih.gov/compound/12716463#section=2D-Structure
https://pubchem.ncbi.nlm.nih.gov/compound/12707344#section=2D-Structure
https://pubchem.ncbi.nlm.nih.gov/compound/12705282#section=2D-Structure
https://pubchem.ncbi.nlm.nih.gov/compound/12704485#section=2D-Structure
https://pubchem.ncbi.nlm.nih.gov/compound/12699825#section=2D-Structure
https://pubchem.ncbi.nlm.nih.gov/compound/12694154#section=2D-Structure
https://pubchem.ncbi.nlm.nih.gov/compound/12693489#section=2D-Structure
https://pubchem.ncbi.nlm.nih.gov/compound/12693488#section=2D-Structure
https://pubchem.ncbi.nlm.nih.gov/compound/12686970#section=2D-Structure
https://pubchem.ncbi.nlm.nih.gov/compound/126810#section=2D-Structure
https://pubchem.ncbi.nlm.nih.gov/compound/126746621#section=2D-Structure
https://pubchem.ncbi.nlm.nih.gov/compound/12674257#section=2D-Structure
https://pubchem.ncbi.nlm.nih.gov/compound/12674254#section=2D-Structure
https://pubchem.ncbi.nlm.nih.gov/compound/12674253#section=2D-Structure
https://pubchem.ncbi.nlm.nih.gov/compound/12674125#section=2D-Structure
https://pubchem.ncbi.nlm.nih.gov/compound/126709833#section=2D-Structure
https://pubchem.ncbi.nlm.nih.gov/compound/126709809#section=2D-Structure
https://pubchem.ncbi.nlm.nih.gov/compound/126707335#section=2D-Structure
https://pubchem.ncbi.nlm.nih.gov/compound/126681380#section=2D-Structure
https://pubchem.ncbi.nlm.nih.gov/compound/126673221#section=2D-Structure
https://pubchem.ncbi.nlm.nih.gov/compound/126661636#section=2D-Structure
https://pubchem.ncbi.nlm.nih.gov/compound/126660824#section=2D-Structure
https://pubchem.ncbi.nlm.nih.gov/compound/12665143#section=2D-Structure
https://pubchem.ncbi.nlm.nih.gov/compound/12665142#section=2D-Structure
https://pubchem.ncbi.nlm.nih.gov/compound/126640775#section=2D-Structure
https://pubchem.ncbi.nlm.nih.gov/compound/126635955#section=2D-Structure
https://pubchem.ncbi.nlm.nih.gov/compound/126632640#section=2D-Structure
https://pubchem.ncbi.nlm.nih.gov/compound/126628293#section=2D-Structure
https://pubchem.ncbi.nlm.nih.gov/compound/126611818#section=2D-Structure
https://pubchem.ncbi.nlm.nih.gov/compound/126605199#section=2D-Structure
https://pubchem.ncbi.nlm.nih.gov/compound/126589577#section=2D-Structure
https://pubchem.ncbi.nlm.nih.gov/compound/126589467#section=2D-Structure
https://pubchem.ncbi.nlm.nih.gov/compound/126573773#section=2D-Structure
https://pubchem.ncbi.nlm.nih.gov/compound/126564982#section=2D-Structure

https://pubchem.ncbi.nlm.nih.gov/compound/12654471#section=2D-Structure
https://pubchem.ncbi.nlm.nih.gov/compound/12654467#section=2D-Structure
https://pubchem.ncbi.nlm.nih.gov/compound/126536215#section=2D-Structure
https://pubchem.ncbi.nlm.nih.gov/compound/12652489#section=2D-Structure
https://pubchem.ncbi.nlm.nih.gov/compound/126516841#section=2D-Structure
https://pubchem.ncbi.nlm.nih.gov/compound/126509587#section=2D-Structure
https://pubchem.ncbi.nlm.nih.gov/compound/12646123#section=2D-Structure
https://pubchem.ncbi.nlm.nih.gov/compound/126455713#section=2D-Structure
https://pubchem.ncbi.nlm.nih.gov/compound/126422159#section=2D-Structure
https://pubchem.ncbi.nlm.nih.gov/compound/126421832#section=2D-Structure
https://pubchem.ncbi.nlm.nih.gov/compound/126421759#section=2D-Structure
https://pubchem.ncbi.nlm.nih.gov/compound/126419427#section=2D-Structure
https://pubchem.ncbi.nlm.nih.gov/compound/126419417#section=2D-Structure
https://pubchem.ncbi.nlm.nih.gov/compound/126419187#section=2D-Structure
https://pubchem.ncbi.nlm.nih.gov/compound/12637361#section=2D-Structure
https://pubchem.ncbi.nlm.nih.gov/compound/12636157#section=2D-Structure
https://pubchem.ncbi.nlm.nih.gov/compound/12629297#section=2D-Structure
https://pubchem.ncbi.nlm.nih.gov/compound/12627853#section=2D-Structure
https://pubchem.ncbi.nlm.nih.gov/compound/12627852#section=2D-Structure
https://pubchem.ncbi.nlm.nih.gov/compound/12621647#section=2D-Structure
https://pubchem.ncbi.nlm.nih.gov/compound/12614921#section=2D-Structure
https://pubchem.ncbi.nlm.nih.gov/compound/12601291#section=2D-Structure
https://pubchem.ncbi.nlm.nih.gov/compound/12594651#section=2D-Structure
https://pubchem.ncbi.nlm.nih.gov/compound/12594650#section=2D-Structure
https://pubchem.ncbi.nlm.nih.gov/compound/12588709#section=2D-Structure
https://pubchem.ncbi.nlm.nih.gov/compound/12588708#section=2D-Structure
https://pubchem.ncbi.nlm.nih.gov/compound/12588706#section=2D-Structure
https://pubchem.ncbi.nlm.nih.gov/compound/12588705#section=2D-Structure
https://pubchem.ncbi.nlm.nih.gov/compound/12588520#section=2D-Structure
https://pubchem.ncbi.nlm.nih.gov/compound/12586187#section=2D-Structure
https://pubchem.ncbi.nlm.nih.gov/compound/12579855#section=2D-Structure
https://pubchem.ncbi.nlm.nih.gov/compound/12576428#section=2D-Structure
https://pubchem.ncbi.nlm.nih.gov/compound/12576424#section=2D-Structure
https://pubchem.ncbi.nlm.nih.gov/compound/12571232#section=2D-Structure
https://pubchem.ncbi.nlm.nih.gov/compound/12557732#section=2D-Structure
https://pubchem.ncbi.nlm.nih.gov/compound/12553862#section=2D-Structure
https://pubchem.ncbi.nlm.nih.gov/compound/12553860#section=2D-Structure
https://pubchem.ncbi.nlm.nih.gov/compound/12553008#section=2D-Structure
https://pubchem.ncbi.nlm.nih.gov/compound/125507957#section=2D-Structure
https://pubchem.ncbi.nlm.nih.gov/compound/125504122#section=2D-Structure
https://pubchem.ncbi.nlm.nih.gov/compound/125489554#section=2D-Structure
https://pubchem.ncbi.nlm.nih.gov/compound/125477911#section=2D-Structure
https://pubchem.ncbi.nlm.nih.gov/compound/12545035#section=2D-Structure
https://pubchem.ncbi.nlm.nih.gov/compound/125421373#section=2D-Structure
https://pubchem.ncbi.nlm.nih.gov/compound/12541811#section=2D-Structure
https://pubchem.ncbi.nlm.nih.gov/compound/125411961#section=2D-Structure
https://pubchem.ncbi.nlm.nih.gov/compound/125390909#section=2D-Structure
https://pubchem.ncbi.nlm.nih.gov/compound/12532434#section=2D-Structure
https://pubchem.ncbi.nlm.nih.gov/compound/12531936#section=2D-Structure
https://pubchem.ncbi.nlm.nih.gov/compound/125288357#section=2D-Structure
https://pubchem.ncbi.nlm.nih.gov/compound/12527056#section=2D-Structure
https://pubchem.ncbi.nlm.nih.gov/compound/12517532#section=2D-Structure
https://pubchem.ncbi.nlm.nih.gov/compound/12502614#section=2D-Structure
https://pubchem.ncbi.nlm.nih.gov/compound/12502613#section=2D-Structure
https://pubchem.ncbi.nlm.nih.gov/compound/124927809#section=2D-Structure

https://pubchem.ncbi.nlm.nih.gov/compound/12488418#section=2D-Structure
https://pubchem.ncbi.nlm.nih.gov/compound/12488339#section=2D-Structure
https://pubchem.ncbi.nlm.nih.gov/compound/12481832#section=2D-Structure
https://pubchem.ncbi.nlm.nih.gov/compound/12481007#section=2D-Structure
https://pubchem.ncbi.nlm.nih.gov/compound/12473099#section=2D-Structure
https://pubchem.ncbi.nlm.nih.gov/compound/12473093#section=2D-Structure
https://pubchem.ncbi.nlm.nih.gov/compound/124707499#section=2D-Structure
https://pubchem.ncbi.nlm.nih.gov/compound/12469601#section=2D-Structure
https://pubchem.ncbi.nlm.nih.gov/compound/12469600#section=2D-Structure
https://pubchem.ncbi.nlm.nih.gov/compound/12462289#section=2D-Structure
https://pubchem.ncbi.nlm.nih.gov/compound/12461985#section=2D-Structure
https://pubchem.ncbi.nlm.nih.gov/compound/12459585#section=2D-Structure
https://pubchem.ncbi.nlm.nih.gov/compound/124576414#section=2D-Structure
https://pubchem.ncbi.nlm.nih.gov/compound/12450739#section=2D-Structure
https://pubchem.ncbi.nlm.nih.gov/compound/12450730#section=2D-Structure
https://pubchem.ncbi.nlm.nih.gov/compound/124504471#section=2D-Structure
https://pubchem.ncbi.nlm.nih.gov/compound/124504446#section=2D-Structure
https://pubchem.ncbi.nlm.nih.gov/compound/12447840#section=2D-Structure
https://pubchem.ncbi.nlm.nih.gov/compound/12446688#section=2D-Structure
https://pubchem.ncbi.nlm.nih.gov/compound/124415556#section=2D-Structure
https://pubchem.ncbi.nlm.nih.gov/compound/124413453#section=2D-Structure
https://pubchem.ncbi.nlm.nih.gov/compound/124305710#section=2D-Structure
https://pubchem.ncbi.nlm.nih.gov/compound/12429266#section=2D-Structure
https://pubchem.ncbi.nlm.nih.gov/compound/124288594#section=2D-Structure
https://pubchem.ncbi.nlm.nih.gov/compound/124288572#section=2D-Structure
https://pubchem.ncbi.nlm.nih.gov/compound/124288571#section=2D-Structure
https://pubchem.ncbi.nlm.nih.gov/compound/124288570#section=2D-Structure
https://pubchem.ncbi.nlm.nih.gov/compound/124287848#section=2D-Structure
https://pubchem.ncbi.nlm.nih.gov/compound/124287433#section=2D-Structure
https://pubchem.ncbi.nlm.nih.gov/compound/12427801#section=2D-Structure
https://pubchem.ncbi.nlm.nih.gov/compound/124253441#section=2D-Structure
https://pubchem.ncbi.nlm.nih.gov/compound/12425231#section=2D-Structure
https://pubchem.ncbi.nlm.nih.gov/compound/12424449#section=2D-Structure
https://pubchem.ncbi.nlm.nih.gov/compound/124226117#section=2D-Structure
https://pubchem.ncbi.nlm.nih.gov/compound/124224988#section=2D-Structure
https://pubchem.ncbi.nlm.nih.gov/compound/12421109#section=2D-Structure
https://pubchem.ncbi.nlm.nih.gov/compound/12421108#section=2D-Structure
https://pubchem.ncbi.nlm.nih.gov/compound/12421106#section=2D-Structure
https://pubchem.ncbi.nlm.nih.gov/compound/12421096#section=2D-Structure
https://pubchem.ncbi.nlm.nih.gov/compound/12421090#section=2D-Structure
https://pubchem.ncbi.nlm.nih.gov/compound/124185355#section=2D-Structure
https://pubchem.ncbi.nlm.nih.gov/compound/124175684#section=2D-Structure
https://pubchem.ncbi.nlm.nih.gov/compound/124149768#section=2D-Structure
https://pubchem.ncbi.nlm.nih.gov/compound/124144887#section=2D-Structure
https://pubchem.ncbi.nlm.nih.gov/compound/124142735#section=2D-Structure
https://pubchem.ncbi.nlm.nih.gov/compound/124141339#section=2D-Structure
https://pubchem.ncbi.nlm.nih.gov/compound/124130429#section=2D-Structure
https://pubchem.ncbi.nlm.nih.gov/compound/124128360#section=2D-Structure
https://pubchem.ncbi.nlm.nih.gov/compound/124128025#section=2D-Structure
https://pubchem.ncbi.nlm.nih.gov/compound/124089007#section=2D-Structure
https://pubchem.ncbi.nlm.nih.gov/compound/124034976#section=2D-Structure
https://pubchem.ncbi.nlm.nih.gov/compound/124030334#section=2D-Structure
https://pubchem.ncbi.nlm.nih.gov/compound/124027223#section=2D-Structure
https://pubchem.ncbi.nlm.nih.gov/compound/124024332#section=2D-Structure
https://pubchem.ncbi.nlm.nih.gov/compound/124020726#section=2D-Structure

https://pubchem.ncbi.nlm.nih.gov/compound/124020162#section=2D-Structure
https://pubchem.ncbi.nlm.nih.gov/compound/124005379#section=2D-Structure
https://pubchem.ncbi.nlm.nih.gov/compound/12399924#section=2D-Structure
https://pubchem.ncbi.nlm.nih.gov/compound/123997905#section=2D-Structure
https://pubchem.ncbi.nlm.nih.gov/compound/123995135#section=2D-Structure
https://pubchem.ncbi.nlm.nih.gov/compound/123987817#section=2D-Structure
https://pubchem.ncbi.nlm.nih.gov/compound/12398497#section=2D-Structure
https://pubchem.ncbi.nlm.nih.gov/compound/123982569#section=2D-Structure
https://pubchem.ncbi.nlm.nih.gov/compound/123981000#section=2D-Structure
https://pubchem.ncbi.nlm.nih.gov/compound/123980266#section=2D-Structure
https://pubchem.ncbi.nlm.nih.gov/compound/123976374#section=2D-Structure
https://pubchem.ncbi.nlm.nih.gov/compound/123975146#section=2D-Structure
https://pubchem.ncbi.nlm.nih.gov/compound/123974610#section=2D-Structure
https://pubchem.ncbi.nlm.nih.gov/compound/12396674#section=2D-Structure
https://pubchem.ncbi.nlm.nih.gov/compound/123964325#section=2D-Structure
https://pubchem.ncbi.nlm.nih.gov/compound/123962320#section=2D-Structure
https://pubchem.ncbi.nlm.nih.gov/compound/123957752#section=2D-Structure
https://pubchem.ncbi.nlm.nih.gov/compound/123955108#section=2D-Structure
https://pubchem.ncbi.nlm.nih.gov/compound/123954066#section=2D-Structure
https://pubchem.ncbi.nlm.nih.gov/compound/12395395#section=2D-Structure
https://pubchem.ncbi.nlm.nih.gov/compound/12395392#section=2D-Structure
https://pubchem.ncbi.nlm.nih.gov/compound/123946475#section=2D-Structure
https://pubchem.ncbi.nlm.nih.gov/compound/123943811#section=2D-Structure
https://pubchem.ncbi.nlm.nih.gov/compound/123936609#section=2D-Structure
https://pubchem.ncbi.nlm.nih.gov/compound/123935586#section=2D-Structure
https://pubchem.ncbi.nlm.nih.gov/compound/123935390#section=2D-Structure
https://pubchem.ncbi.nlm.nih.gov/compound/123934865#section=2D-Structure
https://pubchem.ncbi.nlm.nih.gov/compound/123932004#section=2D-Structure
https://pubchem.ncbi.nlm.nih.gov/compound/123930877#section=2D-Structure
https://pubchem.ncbi.nlm.nih.gov/compound/123930035#section=2D-Structure
https://pubchem.ncbi.nlm.nih.gov/compound/123929775#section=2D-Structure
https://pubchem.ncbi.nlm.nih.gov/compound/123922930#section=2D-Structure
https://pubchem.ncbi.nlm.nih.gov/compound/123921752#section=2D-Structure
https://pubchem.ncbi.nlm.nih.gov/compound/123918697#section=2D-Structure
https://pubchem.ncbi.nlm.nih.gov/compound/123906867#section=2D-Structure
https://pubchem.ncbi.nlm.nih.gov/compound/123904556#section=2D-Structure
https://pubchem.ncbi.nlm.nih.gov/compound/123899804#section=2D-Structure
https://pubchem.ncbi.nlm.nih.gov/compound/123890173#section=2D-Structure
https://pubchem.ncbi.nlm.nih.gov/compound/123889231#section=2D-Structure
https://pubchem.ncbi.nlm.nih.gov/compound/123884740#section=2D-Structure
https://pubchem.ncbi.nlm.nih.gov/compound/123883887#section=2D-Structure
https://pubchem.ncbi.nlm.nih.gov/compound/123877268#section=2D-Structure
https://pubchem.ncbi.nlm.nih.gov/compound/123876655#section=2D-Structure
https://pubchem.ncbi.nlm.nih.gov/compound/123864885#section=2D-Structure
https://pubchem.ncbi.nlm.nih.gov/compound/123862158#section=2D-Structure
https://pubchem.ncbi.nlm.nih.gov/compound/12386192#section=2D-Structure
https://pubchem.ncbi.nlm.nih.gov/compound/123859511#section=2D-Structure
https://pubchem.ncbi.nlm.nih.gov/compound/123855295#section=2D-Structure
https://pubchem.ncbi.nlm.nih.gov/compound/123853270#section=2D-Structure
https://pubchem.ncbi.nlm.nih.gov/compound/123850950#section=2D-Structure
https://pubchem.ncbi.nlm.nih.gov/compound/123850578#section=2D-Structure
https://pubchem.ncbi.nlm.nih.gov/compound/123848833#section=2D-Structure
https://pubchem.ncbi.nlm.nih.gov/compound/123841245#section=2D-Structure
https://pubchem.ncbi.nlm.nih.gov/compound/123838562#section=2D-Structure
https://pubchem.ncbi.nlm.nih.gov/compound/123833439#section=2D-Structure

https://pubchem.ncbi.nlm.nih.gov/compound/123833117#section=2D-Structure
https://pubchem.ncbi.nlm.nih.gov/compound/123831820#section=2D-Structure
https://pubchem.ncbi.nlm.nih.gov/compound/123831613#section=2D-Structure
https://pubchem.ncbi.nlm.nih.gov/compound/123831193#section=2D-Structure
https://pubchem.ncbi.nlm.nih.gov/compound/123830396#section=2D-Structure
https://pubchem.ncbi.nlm.nih.gov/compound/123830232#section=2D-Structure
https://pubchem.ncbi.nlm.nih.gov/compound/123829027#section=2D-Structure
https://pubchem.ncbi.nlm.nih.gov/compound/123819272#section=2D-Structure
https://pubchem.ncbi.nlm.nih.gov/compound/123814008#section=2D-Structure
https://pubchem.ncbi.nlm.nih.gov/compound/123808995#section=2D-Structure
https://pubchem.ncbi.nlm.nih.gov/compound/123808992#section=2D-Structure
https://pubchem.ncbi.nlm.nih.gov/compound/123806879#section=2D-Structure
https://pubchem.ncbi.nlm.nih.gov/compound/123803363#section=2D-Structure
https://pubchem.ncbi.nlm.nih.gov/compound/123797548#section=2D-Structure
https://pubchem.ncbi.nlm.nih.gov/compound/123795624#section=2D-Structure
https://pubchem.ncbi.nlm.nih.gov/compound/123793345#section=2D-Structure
https://pubchem.ncbi.nlm.nih.gov/compound/123790519#section=2D-Structure
https://pubchem.ncbi.nlm.nih.gov/compound/123785546#section=2D-Structure
https://pubchem.ncbi.nlm.nih.gov/compound/123785418#section=2D-Structure
https://pubchem.ncbi.nlm.nih.gov/compound/123781369#section=2D-Structure
https://pubchem.ncbi.nlm.nih.gov/compound/123780140#section=2D-Structure
https://pubchem.ncbi.nlm.nih.gov/compound/123778169#section=2D-Structure
https://pubchem.ncbi.nlm.nih.gov/compound/123777502#section=2D-Structure
https://pubchem.ncbi.nlm.nih.gov/compound/12377656#section=2D-Structure
https://pubchem.ncbi.nlm.nih.gov/compound/123775416#section=2D-Structure
https://pubchem.ncbi.nlm.nih.gov/compound/123769543#section=2D-Structure
https://pubchem.ncbi.nlm.nih.gov/compound/123769496#section=2D-Structure
https://pubchem.ncbi.nlm.nih.gov/compound/123764880#section=2D-Structure
https://pubchem.ncbi.nlm.nih.gov/compound/123764747#section=2D-Structure
https://pubchem.ncbi.nlm.nih.gov/compound/123761267#section=2D-Structure
https://pubchem.ncbi.nlm.nih.gov/compound/123748416#section=2D-Structure
https://pubchem.ncbi.nlm.nih.gov/compound/12374815#section=2D-Structure
https://pubchem.ncbi.nlm.nih.gov/compound/123746144#section=2D-Structure
https://pubchem.ncbi.nlm.nih.gov/compound/123744564#section=2D-Structure
https://pubchem.ncbi.nlm.nih.gov/compound/123737560#section=2D-Structure
https://pubchem.ncbi.nlm.nih.gov/compound/123736868#section=2D-Structure
https://pubchem.ncbi.nlm.nih.gov/compound/123734549#section=2D-Structure
https://pubchem.ncbi.nlm.nih.gov/compound/123726122#section=2D-Structure
https://pubchem.ncbi.nlm.nih.gov/compound/123722580#section=2D-Structure
https://pubchem.ncbi.nlm.nih.gov/compound/123720856#section=2D-Structure
https://pubchem.ncbi.nlm.nih.gov/compound/123717778#section=2D-Structure
https://pubchem.ncbi.nlm.nih.gov/compound/12371629#section=2D-Structure
https://pubchem.ncbi.nlm.nih.gov/compound/12371628#section=2D-Structure
https://pubchem.ncbi.nlm.nih.gov/compound/123711766#section=2D-Structure
https://pubchem.ncbi.nlm.nih.gov/compound/123710600#section=2D-Structure
https://pubchem.ncbi.nlm.nih.gov/compound/12370914#section=2D-Structure
https://pubchem.ncbi.nlm.nih.gov/compound/123708394#section=2D-Structure
https://pubchem.ncbi.nlm.nih.gov/compound/123701637#section=2D-Structure
https://pubchem.ncbi.nlm.nih.gov/compound/123700824#section=2D-Structure
https://pubchem.ncbi.nlm.nih.gov/compound/123696054#section=2D-Structure
https://pubchem.ncbi.nlm.nih.gov/compound/123689685#section=2D-Structure
https://pubchem.ncbi.nlm.nih.gov/compound/123680389#section=2D-Structure
https://pubchem.ncbi.nlm.nih.gov/compound/12368013#section=2D-Structure
https://pubchem.ncbi.nlm.nih.gov/compound/123676562#section=2D-Structure
https://pubchem.ncbi.nlm.nih.gov/compound/12367324#section=2D-Structure

https://pubchem.ncbi.nlm.nih.gov/compound/123672747#section=2D-Structure
https://pubchem.ncbi.nlm.nih.gov/compound/123662065#section=2D-Structure
https://pubchem.ncbi.nlm.nih.gov/compound/123661071#section=2D-Structure
https://pubchem.ncbi.nlm.nih.gov/compound/123660867#section=2D-Structure
https://pubchem.ncbi.nlm.nih.gov/compound/123657015#section=2D-Structure
https://pubchem.ncbi.nlm.nih.gov/compound/123655655#section=2D-Structure
https://pubchem.ncbi.nlm.nih.gov/compound/123655642#section=2D-Structure
https://pubchem.ncbi.nlm.nih.gov/compound/12365024#section=2D-Structure
https://pubchem.ncbi.nlm.nih.gov/compound/123646521#section=2D-Structure
https://pubchem.ncbi.nlm.nih.gov/compound/123646308#section=2D-Structure
https://pubchem.ncbi.nlm.nih.gov/compound/123642970#section=2D-Structure
https://pubchem.ncbi.nlm.nih.gov/compound/123639114#section=2D-Structure
https://pubchem.ncbi.nlm.nih.gov/compound/123634298#section=2D-Structure
https://pubchem.ncbi.nlm.nih.gov/compound/123633481#section=2D-Structure
https://pubchem.ncbi.nlm.nih.gov/compound/123631398#section=2D-Structure
https://pubchem.ncbi.nlm.nih.gov/compound/123630855#section=2D-Structure
https://pubchem.ncbi.nlm.nih.gov/compound/123630184#section=2D-Structure
https://pubchem.ncbi.nlm.nih.gov/compound/123626981#section=2D-Structure
https://pubchem.ncbi.nlm.nih.gov/compound/123623422#section=2D-Structure
https://pubchem.ncbi.nlm.nih.gov/compound/123619905#section=2D-Structure
https://pubchem.ncbi.nlm.nih.gov/compound/123617922#section=2D-Structure
https://pubchem.ncbi.nlm.nih.gov/compound/123611218#section=2D-Structure
https://pubchem.ncbi.nlm.nih.gov/compound/123611149#section=2D-Structure
https://pubchem.ncbi.nlm.nih.gov/compound/123603556#section=2D-Structure
https://pubchem.ncbi.nlm.nih.gov/compound/123590456#section=2D-Structure
https://pubchem.ncbi.nlm.nih.gov/compound/123589336#section=2D-Structure
https://pubchem.ncbi.nlm.nih.gov/compound/123587501#section=2D-Structure
https://pubchem.ncbi.nlm.nih.gov/compound/123584194#section=2D-Structure
https://pubchem.ncbi.nlm.nih.gov/compound/123570291#section=2D-Structure
https://pubchem.ncbi.nlm.nih.gov/compound/123569884#section=2D-Structure
https://pubchem.ncbi.nlm.nih.gov/compound/123569521#section=2D-Structure
https://pubchem.ncbi.nlm.nih.gov/compound/123566241#section=2D-Structure
https://pubchem.ncbi.nlm.nih.gov/compound/123565607#section=2D-Structure
https://pubchem.ncbi.nlm.nih.gov/compound/123564195#section=2D-Structure
https://pubchem.ncbi.nlm.nih.gov/compound/123558959#section=2D-Structure
https://pubchem.ncbi.nlm.nih.gov/compound/123554395#section=2D-Structure
https://pubchem.ncbi.nlm.nih.gov/compound/123542204#section=2D-Structure
https://pubchem.ncbi.nlm.nih.gov/compound/123540723#section=2D-Structure
https://pubchem.ncbi.nlm.nih.gov/compound/12353657#section=2D-Structure
https://pubchem.ncbi.nlm.nih.gov/compound/123533224#section=2D-Structure
https://pubchem.ncbi.nlm.nih.gov/compound/123532394#section=2D-Structure
https://pubchem.ncbi.nlm.nih.gov/compound/123529105#section=2D-Structure
https://pubchem.ncbi.nlm.nih.gov/compound/12352254#section=2D-Structure
https://pubchem.ncbi.nlm.nih.gov/compound/123519352#section=2D-Structure
https://pubchem.ncbi.nlm.nih.gov/compound/123517892#section=2D-Structure
https://pubchem.ncbi.nlm.nih.gov/compound/123515737#section=2D-Structure
https://pubchem.ncbi.nlm.nih.gov/compound/123508584#section=2D-Structure
https://pubchem.ncbi.nlm.nih.gov/compound/123495467#section=2D-Structure
https://pubchem.ncbi.nlm.nih.gov/compound/123495222#section=2D-Structure
https://pubchem.ncbi.nlm.nih.gov/compound/123494829#section=2D-Structure
https://pubchem.ncbi.nlm.nih.gov/compound/12349346#section=2D-Structure
https://pubchem.ncbi.nlm.nih.gov/compound/123481523#section=2D-Structure
https://pubchem.ncbi.nlm.nih.gov/compound/123477900#section=2D-Structure
https://pubchem.ncbi.nlm.nih.gov/compound/123475907#section=2D-Structure
https://pubchem.ncbi.nlm.nih.gov/compound/123474264#section=2D-Structure

https://pubchem.ncbi.nlm.nih.gov/compound/123468650#section=2D-Structure
https://pubchem.ncbi.nlm.nih.gov/compound/123456200#section=2D-Structure
https://pubchem.ncbi.nlm.nih.gov/compound/123454896#section=2D-Structure
https://pubchem.ncbi.nlm.nih.gov/compound/123453048#section=2D-Structure
https://pubchem.ncbi.nlm.nih.gov/compound/123450761#section=2D-Structure
https://pubchem.ncbi.nlm.nih.gov/compound/123444449#section=2D-Structure
https://pubchem.ncbi.nlm.nih.gov/compound/123443893#section=2D-Structure
https://pubchem.ncbi.nlm.nih.gov/compound/123443830#section=2D-Structure
https://pubchem.ncbi.nlm.nih.gov/compound/123432738#section=2D-Structure
https://pubchem.ncbi.nlm.nih.gov/compound/123423789#section=2D-Structure
https://pubchem.ncbi.nlm.nih.gov/compound/123420713#section=2D-Structure
https://pubchem.ncbi.nlm.nih.gov/compound/123417255#section=2D-Structure
https://pubchem.ncbi.nlm.nih.gov/compound/123404215#section=2D-Structure
https://pubchem.ncbi.nlm.nih.gov/compound/123401588#section=2D-Structure
https://pubchem.ncbi.nlm.nih.gov/compound/123400622#section=2D-Structure
https://pubchem.ncbi.nlm.nih.gov/compound/12339780#section=2D-Structure
https://pubchem.ncbi.nlm.nih.gov/compound/12339760#section=2D-Structure
https://pubchem.ncbi.nlm.nih.gov/compound/123396652#section=2D-Structure
https://pubchem.ncbi.nlm.nih.gov/compound/123393359#section=2D-Structure
https://pubchem.ncbi.nlm.nih.gov/compound/123389628#section=2D-Structure
https://pubchem.ncbi.nlm.nih.gov/compound/123387997#section=2D-Structure
https://pubchem.ncbi.nlm.nih.gov/compound/123382262#section=2D-Structure
https://pubchem.ncbi.nlm.nih.gov/compound/123376230#section=2D-Structure
https://pubchem.ncbi.nlm.nih.gov/compound/123374826#section=2D-Structure
https://pubchem.ncbi.nlm.nih.gov/compound/123372625#section=2D-Structure
https://pubchem.ncbi.nlm.nih.gov/compound/123370633#section=2D-Structure
https://pubchem.ncbi.nlm.nih.gov/compound/123370118#section=2D-Structure
https://pubchem.ncbi.nlm.nih.gov/compound/123369799#section=2D-Structure
https://pubchem.ncbi.nlm.nih.gov/compound/123368328#section=2D-Structure
https://pubchem.ncbi.nlm.nih.gov/compound/123366772#section=2D-Structure
https://pubchem.ncbi.nlm.nih.gov/compound/123366696#section=2D-Structure
https://pubchem.ncbi.nlm.nih.gov/compound/123362589#section=2D-Structure
https://pubchem.ncbi.nlm.nih.gov/compound/123355673#section=2D-Structure
https://pubchem.ncbi.nlm.nih.gov/compound/123352503#section=2D-Structure
https://pubchem.ncbi.nlm.nih.gov/compound/123349985#section=2D-Structure
https://pubchem.ncbi.nlm.nih.gov/compound/123343182#section=2D-Structure
https://pubchem.ncbi.nlm.nih.gov/compound/12333823#section=2D-Structure
https://pubchem.ncbi.nlm.nih.gov/compound/123337087#section=2D-Structure
https://pubchem.ncbi.nlm.nih.gov/compound/123336000#section=2D-Structure
https://pubchem.ncbi.nlm.nih.gov/compound/123335845#section=2D-Structure
https://pubchem.ncbi.nlm.nih.gov/compound/123335510#section=2D-Structure
https://pubchem.ncbi.nlm.nih.gov/compound/123334248#section=2D-Structure
https://pubchem.ncbi.nlm.nih.gov/compound/123333944#section=2D-Structure
https://pubchem.ncbi.nlm.nih.gov/compound/123333647#section=2D-Structure
https://pubchem.ncbi.nlm.nih.gov/compound/123328296#section=2D-Structure
https://pubchem.ncbi.nlm.nih.gov/compound/123323887#section=2D-Structure
https://pubchem.ncbi.nlm.nih.gov/compound/123322051#section=2D-Structure
https://pubchem.ncbi.nlm.nih.gov/compound/123319739#section=2D-Structure
https://pubchem.ncbi.nlm.nih.gov/compound/123316164#section=2D-Structure
https://pubchem.ncbi.nlm.nih.gov/compound/123313342#section=2D-Structure
https://pubchem.ncbi.nlm.nih.gov/compound/123308206#section=2D-Structure
https://pubchem.ncbi.nlm.nih.gov/compound/123307313#section=2D-Structure
https://pubchem.ncbi.nlm.nih.gov/compound/123303820#section=2D-Structure
https://pubchem.ncbi.nlm.nih.gov/compound/123297259#section=2D-Structure
https://pubchem.ncbi.nlm.nih.gov/compound/123296379#section=2D-Structure

https://pubchem.ncbi.nlm.nih.gov/compound/123291772#section=2D-Structure
https://pubchem.ncbi.nlm.nih.gov/compound/123291210#section=2D-Structure
https://pubchem.ncbi.nlm.nih.gov/compound/123289155#section=2D-Structure
https://pubchem.ncbi.nlm.nih.gov/compound/123286892#section=2D-Structure
https://pubchem.ncbi.nlm.nih.gov/compound/123284874#section=2D-Structure
https://pubchem.ncbi.nlm.nih.gov/compound/123279646#section=2D-Structure
https://pubchem.ncbi.nlm.nih.gov/compound/12327822#section=2D-Structure
https://pubchem.ncbi.nlm.nih.gov/compound/123275146#section=2D-Structure
https://pubchem.ncbi.nlm.nih.gov/compound/123275005#section=2D-Structure
https://pubchem.ncbi.nlm.nih.gov/compound/123271714#section=2D-Structure
https://pubchem.ncbi.nlm.nih.gov/compound/123270720#section=2D-Structure
https://pubchem.ncbi.nlm.nih.gov/compound/123269243#section=2D-Structure
https://pubchem.ncbi.nlm.nih.gov/compound/123266831#section=2D-Structure
https://pubchem.ncbi.nlm.nih.gov/compound/123266203#section=2D-Structure
https://pubchem.ncbi.nlm.nih.gov/compound/123265493#section=2D-Structure
https://pubchem.ncbi.nlm.nih.gov/compound/123264383#section=2D-Structure
https://pubchem.ncbi.nlm.nih.gov/compound/123263468#section=2D-Structure
https://pubchem.ncbi.nlm.nih.gov/compound/123259748#section=2D-Structure
https://pubchem.ncbi.nlm.nih.gov/compound/123258661#section=2D-Structure
https://pubchem.ncbi.nlm.nih.gov/compound/12324972#section=2D-Structure
https://pubchem.ncbi.nlm.nih.gov/compound/123236379#section=2D-Structure
https://pubchem.ncbi.nlm.nih.gov/compound/123235294#section=2D-Structure
https://pubchem.ncbi.nlm.nih.gov/compound/123232947#section=2D-Structure
https://pubchem.ncbi.nlm.nih.gov/compound/123230402#section=2D-Structure
https://pubchem.ncbi.nlm.nih.gov/compound/123228486#section=2D-Structure
https://pubchem.ncbi.nlm.nih.gov/compound/123228381#section=2D-Structure
https://pubchem.ncbi.nlm.nih.gov/compound/123220361#section=2D-Structure
https://pubchem.ncbi.nlm.nih.gov/compound/123219353#section=2D-Structure
https://pubchem.ncbi.nlm.nih.gov/compound/123216177#section=2D-Structure
https://pubchem.ncbi.nlm.nih.gov/compound/123213104#section=2D-Structure
https://pubchem.ncbi.nlm.nih.gov/compound/123212382#section=2D-Structure
https://pubchem.ncbi.nlm.nih.gov/compound/123211620#section=2D-Structure
https://pubchem.ncbi.nlm.nih.gov/compound/123210539#section=2D-Structure
https://pubchem.ncbi.nlm.nih.gov/compound/123210417#section=2D-Structure
https://pubchem.ncbi.nlm.nih.gov/compound/123209852#section=2D-Structure
https://pubchem.ncbi.nlm.nih.gov/compound/123208689#section=2D-Structure
https://pubchem.ncbi.nlm.nih.gov/compound/123205032#section=2D-Structure
https://pubchem.ncbi.nlm.nih.gov/compound/12320255#section=2D-Structure
https://pubchem.ncbi.nlm.nih.gov/compound/12320254#section=2D-Structure
https://pubchem.ncbi.nlm.nih.gov/compound/12320253#section=2D-Structure
https://pubchem.ncbi.nlm.nih.gov/compound/12320100#section=2D-Structure
https://pubchem.ncbi.nlm.nih.gov/compound/123197898#section=2D-Structure
https://pubchem.ncbi.nlm.nih.gov/compound/123191057#section=2D-Structure
https://pubchem.ncbi.nlm.nih.gov/compound/123184678#section=2D-Structure
https://pubchem.ncbi.nlm.nih.gov/compound/123174444#section=2D-Structure
https://pubchem.ncbi.nlm.nih.gov/compound/123173623#section=2D-Structure
https://pubchem.ncbi.nlm.nih.gov/compound/123167512#section=2D-Structure
https://pubchem.ncbi.nlm.nih.gov/compound/123164948#section=2D-Structure
https://pubchem.ncbi.nlm.nih.gov/compound/123164924#section=2D-Structure
https://pubchem.ncbi.nlm.nih.gov/compound/123163271#section=2D-Structure
https://pubchem.ncbi.nlm.nih.gov/compound/123158875#section=2D-Structure
https://pubchem.ncbi.nlm.nih.gov/compound/123158741#section=2D-Structure
https://pubchem.ncbi.nlm.nih.gov/compound/123157450#section=2D-Structure
https://pubchem.ncbi.nlm.nih.gov/compound/123157372#section=2D-Structure
https://pubchem.ncbi.nlm.nih.gov/compound/123155868#section=2D-Structure

https://pubchem.ncbi.nlm.nih.gov/compound/12313759#section=2D-Structure
https://pubchem.ncbi.nlm.nih.gov/compound/123135066#section=2D-Structure
https://pubchem.ncbi.nlm.nih.gov/compound/123134517#section=2D-Structure
https://pubchem.ncbi.nlm.nih.gov/compound/123134492#section=2D-Structure
https://pubchem.ncbi.nlm.nih.gov/compound/12312974#section=2D-Structure
https://pubchem.ncbi.nlm.nih.gov/compound/12312963#section=2D-Structure
https://pubchem.ncbi.nlm.nih.gov/compound/12311214#section=2D-Structure
https://pubchem.ncbi.nlm.nih.gov/compound/123045#section=2D-Structure
https://pubchem.ncbi.nlm.nih.gov/compound/12296716#section=2D-Structure
https://pubchem.ncbi.nlm.nih.gov/compound/12288467#section=2D-Structure
https://pubchem.ncbi.nlm.nih.gov/compound/12284589#section=2D-Structure
https://pubchem.ncbi.nlm.nih.gov/compound/12278633#section=2D-Structure
https://pubchem.ncbi.nlm.nih.gov/compound/12278631#section=2D-Structure
https://pubchem.ncbi.nlm.nih.gov/compound/12278625#section=2D-Structure
https://pubchem.ncbi.nlm.nih.gov/compound/12274823#section=2D-Structure
https://pubchem.ncbi.nlm.nih.gov/compound/122673341#section=2D-Structure
https://pubchem.ncbi.nlm.nih.gov/compound/122662894#section=2D-Structure
https://pubchem.ncbi.nlm.nih.gov/compound/12264783#section=2D-Structure
https://pubchem.ncbi.nlm.nih.gov/compound/122625911#section=2D-Structure
https://pubchem.ncbi.nlm.nih.gov/compound/12262074#section=2D-Structure
https://pubchem.ncbi.nlm.nih.gov/compound/122615159#section=2D-Structure
https://pubchem.ncbi.nlm.nih.gov/compound/122604534#section=2D-Structure
https://pubchem.ncbi.nlm.nih.gov/compound/122592630#section=2D-Structure
https://pubchem.ncbi.nlm.nih.gov/compound/122591446#section=2D-Structure
https://pubchem.ncbi.nlm.nih.gov/compound/12258104#section=2D-Structure
https://pubchem.ncbi.nlm.nih.gov/compound/122573019#section=2D-Structure
https://pubchem.ncbi.nlm.nih.gov/compound/122552366#section=2D-Structure
https://pubchem.ncbi.nlm.nih.gov/compound/122542111#section=2D-Structure
https://pubchem.ncbi.nlm.nih.gov/compound/12253847#section=2D-Structure
https://pubchem.ncbi.nlm.nih.gov/compound/122534398#section=2D-Structure
https://pubchem.ncbi.nlm.nih.gov/compound/122521970#section=2D-Structure
https://pubchem.ncbi.nlm.nih.gov/compound/12251311#section=2D-Structure
https://pubchem.ncbi.nlm.nih.gov/compound/122502606#section=2D-Structure
https://pubchem.ncbi.nlm.nih.gov/compound/122502605#section=2D-Structure
https://pubchem.ncbi.nlm.nih.gov/compound/122502598#section=2D-Structure
https://pubchem.ncbi.nlm.nih.gov/compound/122502595#section=2D-Structure
https://pubchem.ncbi.nlm.nih.gov/compound/122502594#section=2D-Structure
https://pubchem.ncbi.nlm.nih.gov/compound/122500867#section=2D-Structure
https://pubchem.ncbi.nlm.nih.gov/compound/122483204#section=2D-Structure
https://pubchem.ncbi.nlm.nih.gov/compound/12248317#section=2D-Structure
https://pubchem.ncbi.nlm.nih.gov/compound/12248316#section=2D-Structure
https://pubchem.ncbi.nlm.nih.gov/compound/122473431#section=2D-Structure
https://pubchem.ncbi.nlm.nih.gov/compound/12247037#section=2D-Structure
https://pubchem.ncbi.nlm.nih.gov/compound/12247036#section=2D-Structure
https://pubchem.ncbi.nlm.nih.gov/compound/1224685#section=2D-Structure
https://pubchem.ncbi.nlm.nih.gov/compound/12246297#section=2D-Structure
https://pubchem.ncbi.nlm.nih.gov/compound/122452825#section=2D-Structure
https://pubchem.ncbi.nlm.nih.gov/compound/122449523#section=2D-Structure
https://pubchem.ncbi.nlm.nih.gov/compound/122449282#section=2D-Structure
https://pubchem.ncbi.nlm.nih.gov/compound/122446131#section=2D-Structure
https://pubchem.ncbi.nlm.nih.gov/compound/122444608#section=2D-Structure
https://pubchem.ncbi.nlm.nih.gov/compound/12242900#section=2D-Structure
https://pubchem.ncbi.nlm.nih.gov/compound/122398464#section=2D-Structure
https://pubchem.ncbi.nlm.nih.gov/compound/122397568#section=2D-Structure
https://pubchem.ncbi.nlm.nih.gov/compound/122395765#section=2D-Structure

https://pubchem.ncbi.nlm.nih.gov/compound/122395574#section=2D-Structure
https://pubchem.ncbi.nlm.nih.gov/compound/122392908#section=2D-Structure
https://pubchem.ncbi.nlm.nih.gov/compound/122385333#section=2D-Structure
https://pubchem.ncbi.nlm.nih.gov/compound/122384215#section=2D-Structure
https://pubchem.ncbi.nlm.nih.gov/compound/122383181#section=2D-Structure
https://pubchem.ncbi.nlm.nih.gov/compound/122383180#section=2D-Structure
https://pubchem.ncbi.nlm.nih.gov/compound/122383179#section=2D-Structure
https://pubchem.ncbi.nlm.nih.gov/compound/122383178#section=2D-Structure
https://pubchem.ncbi.nlm.nih.gov/compound/122383177#section=2D-Structure
https://pubchem.ncbi.nlm.nih.gov/compound/122376130#section=2D-Structure
https://pubchem.ncbi.nlm.nih.gov/compound/12237057#section=2D-Structure
https://pubchem.ncbi.nlm.nih.gov/compound/122369795#section=2D-Structure
https://pubchem.ncbi.nlm.nih.gov/compound/122369793#section=2D-Structure
https://pubchem.ncbi.nlm.nih.gov/compound/122365900#section=2D-Structure
https://pubchem.ncbi.nlm.nih.gov/compound/122364013#section=2D-Structure
https://pubchem.ncbi.nlm.nih.gov/compound/12230400#section=2D-Structure
https://pubchem.ncbi.nlm.nih.gov/compound/12229466#section=2D-Structure
https://pubchem.ncbi.nlm.nih.gov/compound/122229074#section=2D-Structure
https://pubchem.ncbi.nlm.nih.gov/compound/122227340#section=2D-Structure
https://pubchem.ncbi.nlm.nih.gov/compound/122226226#section=2D-Structure
https://pubchem.ncbi.nlm.nih.gov/compound/122204333#section=2D-Structure
https://pubchem.ncbi.nlm.nih.gov/compound/12214719#section=2D-Structure
https://pubchem.ncbi.nlm.nih.gov/compound/122130638#section=2D-Structure
https://pubchem.ncbi.nlm.nih.gov/compound/122123#section=2D-Structure
https://pubchem.ncbi.nlm.nih.gov/compound/12211800#section=2D-Structure
https://pubchem.ncbi.nlm.nih.gov/compound/12197003#section=2D-Structure
https://pubchem.ncbi.nlm.nih.gov/compound/12197000#section=2D-Structure
https://pubchem.ncbi.nlm.nih.gov/compound/12196998#section=2D-Structure
https://pubchem.ncbi.nlm.nih.gov/compound/12196997#section=2D-Structure
https://pubchem.ncbi.nlm.nih.gov/compound/12196964#section=2D-Structure
https://pubchem.ncbi.nlm.nih.gov/compound/12196963#section=2D-Structure
https://pubchem.ncbi.nlm.nih.gov/compound/12196962#section=2D-Structure
https://pubchem.ncbi.nlm.nih.gov/compound/12196960#section=2D-Structure
https://pubchem.ncbi.nlm.nih.gov/compound/12196958#section=2D-Structure
https://pubchem.ncbi.nlm.nih.gov/compound/12196952#section=2D-Structure
https://pubchem.ncbi.nlm.nih.gov/compound/12196951#section=2D-Structure
https://pubchem.ncbi.nlm.nih.gov/compound/12196947#section=2D-Structure
https://pubchem.ncbi.nlm.nih.gov/compound/12196869#section=2D-Structure
https://pubchem.ncbi.nlm.nih.gov/compound/12196868#section=2D-Structure
https://pubchem.ncbi.nlm.nih.gov/compound/12195093#section=2D-Structure
https://pubchem.ncbi.nlm.nih.gov/compound/12181208#section=2D-Structure
https://pubchem.ncbi.nlm.nih.gov/compound/12181207#section=2D-Structure
https://pubchem.ncbi.nlm.nih.gov/compound/12169361#section=2D-Structure
https://pubchem.ncbi.nlm.nih.gov/compound/12168022#section=2D-Structure
https://pubchem.ncbi.nlm.nih.gov/compound/12168019#section=2D-Structure
https://pubchem.ncbi.nlm.nih.gov/compound/12163924#section=2D-Structure
https://pubchem.ncbi.nlm.nih.gov/compound/121472678#section=2D-Structure
https://pubchem.ncbi.nlm.nih.gov/compound/121459085#section=2D-Structure
https://pubchem.ncbi.nlm.nih.gov/compound/121456152#section=2D-Structure
https://pubchem.ncbi.nlm.nih.gov/compound/12145402#section=2D-Structure
https://pubchem.ncbi.nlm.nih.gov/compound/121440045#section=2D-Structure
https://pubchem.ncbi.nlm.nih.gov/compound/121425200#section=2D-Structure
https://pubchem.ncbi.nlm.nih.gov/compound/121410989#section=2D-Structure
https://pubchem.ncbi.nlm.nih.gov/compound/121406043#section=2D-Structure
https://pubchem.ncbi.nlm.nih.gov/compound/121395462#section=2D-Structure

https://pubchem.ncbi.nlm.nih.gov/compound/12138324#section=2D-Structure
https://pubchem.ncbi.nlm.nih.gov/compound/12137593#section=2D-Structure
https://pubchem.ncbi.nlm.nih.gov/compound/121375459#section=2D-Structure
https://pubchem.ncbi.nlm.nih.gov/compound/121366145#section=2D-Structure
https://pubchem.ncbi.nlm.nih.gov/compound/121350516#section=2D-Structure
https://pubchem.ncbi.nlm.nih.gov/compound/121334029#section=2D-Structure
https://pubchem.ncbi.nlm.nih.gov/compound/121305938#section=2D-Structure
https://pubchem.ncbi.nlm.nih.gov/compound/121292511#section=2D-Structure
https://pubchem.ncbi.nlm.nih.gov/compound/121278734#section=2D-Structure
https://pubchem.ncbi.nlm.nih.gov/compound/121275273#section=2D-Structure
https://pubchem.ncbi.nlm.nih.gov/compound/121267976#section=2D-Structure
https://pubchem.ncbi.nlm.nih.gov/compound/121240520#section=2D-Structure
https://pubchem.ncbi.nlm.nih.gov/compound/121226711#section=2D-Structure
https://pubchem.ncbi.nlm.nih.gov/compound/121226563#section=2D-Structure
https://pubchem.ncbi.nlm.nih.gov/compound/121220536#section=2D-Structure
https://pubchem.ncbi.nlm.nih.gov/compound/121218764#section=2D-Structure
https://pubchem.ncbi.nlm.nih.gov/compound/121217628#section=2D-Structure
https://pubchem.ncbi.nlm.nih.gov/compound/121217619#section=2D-Structure
https://pubchem.ncbi.nlm.nih.gov/compound/121217468#section=2D-Structure
https://pubchem.ncbi.nlm.nih.gov/compound/121217466#section=2D-Structure
https://pubchem.ncbi.nlm.nih.gov/compound/121216445#section=2D-Structure
https://pubchem.ncbi.nlm.nih.gov/compound/121215739#section=2D-Structure
https://pubchem.ncbi.nlm.nih.gov/compound/121014169#section=2D-Structure
https://pubchem.ncbi.nlm.nih.gov/compound/121012383#section=2D-Structure
https://pubchem.ncbi.nlm.nih.gov/compound/121010439#section=2D-Structure
https://pubchem.ncbi.nlm.nih.gov/compound/121009263#section=2D-Structure
https://pubchem.ncbi.nlm.nih.gov/compound/121007650#section=2D-Structure
https://pubchem.ncbi.nlm.nih.gov/compound/121007172#section=2D-Structure
https://pubchem.ncbi.nlm.nih.gov/compound/121007070#section=2D-Structure
https://pubchem.ncbi.nlm.nih.gov/compound/121006988#section=2D-Structure
https://pubchem.ncbi.nlm.nih.gov/compound/121006463#section=2D-Structure
https://pubchem.ncbi.nlm.nih.gov/compound/12078387#section=2D-Structure
https://pubchem.ncbi.nlm.nih.gov/compound/12059093#section=2D-Structure
https://pubchem.ncbi.nlm.nih.gov/compound/12039801#section=2D-Structure
https://pubchem.ncbi.nlm.nih.gov/compound/12032372#section=2D-Structure
https://pubchem.ncbi.nlm.nih.gov/compound/12031987#section=2D-Structure
https://pubchem.ncbi.nlm.nih.gov/compound/12027254#section=2D-Structure
https://pubchem.ncbi.nlm.nih.gov/compound/12027252#section=2D-Structure
https://pubchem.ncbi.nlm.nih.gov/compound/12011867#section=2D-Structure
https://pubchem.ncbi.nlm.nih.gov/compound/12007129#section=2D-Structure
https://pubchem.ncbi.nlm.nih.gov/compound/11966231#section=2D-Structure
https://pubchem.ncbi.nlm.nih.gov/compound/11951641#section=2D-Structure
https://pubchem.ncbi.nlm.nih.gov/compound/11951640#section=2D-Structure
https://pubchem.ncbi.nlm.nih.gov/compound/119208#section=2D-Structure
https://pubchem.ncbi.nlm.nih.gov/compound/119098720#section=2D-Structure
https://pubchem.ncbi.nlm.nih.gov/compound/119098595#section=2D-Structure
https://pubchem.ncbi.nlm.nih.gov/compound/119098479#section=2D-Structure
https://pubchem.ncbi.nlm.nih.gov/compound/119098419#section=2D-Structure
https://pubchem.ncbi.nlm.nih.gov/compound/119097421#section=2D-Structure
https://pubchem.ncbi.nlm.nih.gov/compound/119097308#section=2D-Structure
https://pubchem.ncbi.nlm.nih.gov/compound/119096694#section=2D-Structure
https://pubchem.ncbi.nlm.nih.gov/compound/119093034#section=2D-Structure
https://pubchem.ncbi.nlm.nih.gov/compound/119090679#section=2D-Structure
https://pubchem.ncbi.nlm.nih.gov/compound/119090417#section=2D-Structure
https://pubchem.ncbi.nlm.nih.gov/compound/119090079#section=2D-Structure

https://pubchem.ncbi.nlm.nih.gov/compound/119088349#section=2D-Structure
https://pubchem.ncbi.nlm.nih.gov/compound/119086068#section=2D-Structure
https://pubchem.ncbi.nlm.nih.gov/compound/119079939#section=2D-Structure
https://pubchem.ncbi.nlm.nih.gov/compound/119057457#section=2D-Structure
https://pubchem.ncbi.nlm.nih.gov/compound/118982107#section=2D-Structure
https://pubchem.ncbi.nlm.nih.gov/compound/118978182#section=2D-Structure
https://pubchem.ncbi.nlm.nih.gov/compound/118978181#section=2D-Structure
https://pubchem.ncbi.nlm.nih.gov/compound/118978039#section=2D-Structure
https://pubchem.ncbi.nlm.nih.gov/compound/118963550#section=2D-Structure
https://pubchem.ncbi.nlm.nih.gov/compound/118949676#section=2D-Structure
https://pubchem.ncbi.nlm.nih.gov/compound/118946212#section=2D-Structure
https://pubchem.ncbi.nlm.nih.gov/compound/118913372#section=2D-Structure
https://pubchem.ncbi.nlm.nih.gov/compound/118905887#section=2D-Structure
https://pubchem.ncbi.nlm.nih.gov/compound/118902720#section=2D-Structure
https://pubchem.ncbi.nlm.nih.gov/compound/118895263#section=2D-Structure
https://pubchem.ncbi.nlm.nih.gov/compound/118877669#section=2D-Structure
https://pubchem.ncbi.nlm.nih.gov/compound/118877667#section=2D-Structure
https://pubchem.ncbi.nlm.nih.gov/compound/118873510#section=2D-Structure
https://pubchem.ncbi.nlm.nih.gov/compound/118873126#section=2D-Structure
https://pubchem.ncbi.nlm.nih.gov/compound/118867387#section=2D-Structure
https://pubchem.ncbi.nlm.nih.gov/compound/118679150#section=2D-Structure
https://pubchem.ncbi.nlm.nih.gov/compound/118653102#section=2D-Structure
https://pubchem.ncbi.nlm.nih.gov/compound/118643170#section=2D-Structure
https://pubchem.ncbi.nlm.nih.gov/compound/118636235#section=2D-Structure
https://pubchem.ncbi.nlm.nih.gov/compound/118609799#section=2D-Structure
https://pubchem.ncbi.nlm.nih.gov/compound/118603521#section=2D-Structure
https://pubchem.ncbi.nlm.nih.gov/compound/118595389#section=2D-Structure
https://pubchem.ncbi.nlm.nih.gov/compound/118588840#section=2D-Structure
https://pubchem.ncbi.nlm.nih.gov/compound/118561747#section=2D-Structure
https://pubchem.ncbi.nlm.nih.gov/compound/118547292#section=2D-Structure
https://pubchem.ncbi.nlm.nih.gov/compound/118530670#section=2D-Structure
https://pubchem.ncbi.nlm.nih.gov/compound/118525262#section=2D-Structure
https://pubchem.ncbi.nlm.nih.gov/compound/118524979#section=2D-Structure
https://pubchem.ncbi.nlm.nih.gov/compound/118520963#section=2D-Structure
https://pubchem.ncbi.nlm.nih.gov/compound/118514533#section=2D-Structure
https://pubchem.ncbi.nlm.nih.gov/compound/118495758#section=2D-Structure
https://pubchem.ncbi.nlm.nih.gov/compound/118495757#section=2D-Structure
https://pubchem.ncbi.nlm.nih.gov/compound/118491867#section=2D-Structure
https://pubchem.ncbi.nlm.nih.gov/compound/118485772#section=2D-Structure
https://pubchem.ncbi.nlm.nih.gov/compound/118482385#section=2D-Structure
https://pubchem.ncbi.nlm.nih.gov/compound/118478906#section=2D-Structure
https://pubchem.ncbi.nlm.nih.gov/compound/118472605#section=2D-Structure
https://pubchem.ncbi.nlm.nih.gov/compound/118461146#section=2D-Structure
https://pubchem.ncbi.nlm.nih.gov/compound/118443254#section=2D-Structure
https://pubchem.ncbi.nlm.nih.gov/compound/118436774#section=2D-Structure
https://pubchem.ncbi.nlm.nih.gov/compound/11843335#section=2D-Structure
https://pubchem.ncbi.nlm.nih.gov/compound/11843334#section=2D-Structure
https://pubchem.ncbi.nlm.nih.gov/compound/11843333#section=2D-Structure
https://pubchem.ncbi.nlm.nih.gov/compound/118422252#section=2D-Structure
https://pubchem.ncbi.nlm.nih.gov/compound/118420227#section=2D-Structure
https://pubchem.ncbi.nlm.nih.gov/compound/118390874#section=2D-Structure
https://pubchem.ncbi.nlm.nih.gov/compound/118384426#section=2D-Structure
https://pubchem.ncbi.nlm.nih.gov/compound/118364550#section=2D-Structure
https://pubchem.ncbi.nlm.nih.gov/compound/118360001#section=2D-Structure
https://pubchem.ncbi.nlm.nih.gov/compound/118335317#section=2D-Structure

https://pubchem.ncbi.nlm.nih.gov/compound/118334614#section=2D-Structure
https://pubchem.ncbi.nlm.nih.gov/compound/118325155#section=2D-Structure
https://pubchem.ncbi.nlm.nih.gov/compound/118314913#section=2D-Structure
https://pubchem.ncbi.nlm.nih.gov/compound/11831271#section=2D-Structure
https://pubchem.ncbi.nlm.nih.gov/compound/118307124#section=2D-Structure
https://pubchem.ncbi.nlm.nih.gov/compound/118303098#section=2D-Structure
https://pubchem.ncbi.nlm.nih.gov/compound/11829735#section=2D-Structure
https://pubchem.ncbi.nlm.nih.gov/compound/118296306#section=2D-Structure
https://pubchem.ncbi.nlm.nih.gov/compound/118291573#section=2D-Structure
https://pubchem.ncbi.nlm.nih.gov/compound/118288414#section=2D-Structure
https://pubchem.ncbi.nlm.nih.gov/compound/118273973#section=2D-Structure
https://pubchem.ncbi.nlm.nih.gov/compound/118272223#section=2D-Structure
https://pubchem.ncbi.nlm.nih.gov/compound/118257602#section=2D-Structure
https://pubchem.ncbi.nlm.nih.gov/compound/118239391#section=2D-Structure
https://pubchem.ncbi.nlm.nih.gov/compound/118239388#section=2D-Structure
https://pubchem.ncbi.nlm.nih.gov/compound/118239386#section=2D-Structure
https://pubchem.ncbi.nlm.nih.gov/compound/118236166#section=2D-Structure
https://pubchem.ncbi.nlm.nih.gov/compound/118234208#section=2D-Structure
https://pubchem.ncbi.nlm.nih.gov/compound/118209761#section=2D-Structure
https://pubchem.ncbi.nlm.nih.gov/compound/118208421#section=2D-Structure
https://pubchem.ncbi.nlm.nih.gov/compound/118208406#section=2D-Structure
https://pubchem.ncbi.nlm.nih.gov/compound/11819721#section=2D-Structure
https://pubchem.ncbi.nlm.nih.gov/compound/118186644#section=2D-Structure
https://pubchem.ncbi.nlm.nih.gov/compound/118184971#section=2D-Structure
https://pubchem.ncbi.nlm.nih.gov/compound/118184970#section=2D-Structure
https://pubchem.ncbi.nlm.nih.gov/compound/118174549#section=2D-Structure
https://pubchem.ncbi.nlm.nih.gov/compound/118174499#section=2D-Structure
https://pubchem.ncbi.nlm.nih.gov/compound/118174479#section=2D-Structure
https://pubchem.ncbi.nlm.nih.gov/compound/118174379#section=2D-Structure
https://pubchem.ncbi.nlm.nih.gov/compound/118173537#section=2D-Structure
https://pubchem.ncbi.nlm.nih.gov/compound/118165001#section=2D-Structure
https://pubchem.ncbi.nlm.nih.gov/compound/118164425#section=2D-Structure
https://pubchem.ncbi.nlm.nih.gov/compound/118138956#section=2D-Structure
https://pubchem.ncbi.nlm.nih.gov/compound/118130241#section=2D-Structure
https://pubchem.ncbi.nlm.nih.gov/compound/118130240#section=2D-Structure
https://pubchem.ncbi.nlm.nih.gov/compound/118130239#section=2D-Structure
https://pubchem.ncbi.nlm.nih.gov/compound/118130237#section=2D-Structure
https://pubchem.ncbi.nlm.nih.gov/compound/118130130#section=2D-Structure
https://pubchem.ncbi.nlm.nih.gov/compound/118113845#section=2D-Structure
https://pubchem.ncbi.nlm.nih.gov/compound/118113842#section=2D-Structure
https://pubchem.ncbi.nlm.nih.gov/compound/118101356#section=2D-Structure
https://pubchem.ncbi.nlm.nih.gov/compound/118097719#section=2D-Structure
https://pubchem.ncbi.nlm.nih.gov/compound/118096739#section=2D-Structure
https://pubchem.ncbi.nlm.nih.gov/compound/118091279#section=2D-Structure
https://pubchem.ncbi.nlm.nih.gov/compound/118067714#section=2D-Structure
https://pubchem.ncbi.nlm.nih.gov/compound/118067713#section=2D-Structure
https://pubchem.ncbi.nlm.nih.gov/compound/11806265#section=2D-Structure
https://pubchem.ncbi.nlm.nih.gov/compound/118062263#section=2D-Structure
https://pubchem.ncbi.nlm.nih.gov/compound/118049158#section=2D-Structure
https://pubchem.ncbi.nlm.nih.gov/compound/118034357#section=2D-Structure
https://pubchem.ncbi.nlm.nih.gov/compound/118033989#section=2D-Structure
https://pubchem.ncbi.nlm.nih.gov/compound/117981270#section=2D-Structure
https://pubchem.ncbi.nlm.nih.gov/compound/117979601#section=2D-Structure
https://pubchem.ncbi.nlm.nih.gov/compound/117977034#section=2D-Structure
https://pubchem.ncbi.nlm.nih.gov/compound/117969449#section=2D-Structure

https://pubchem.ncbi.nlm.nih.gov/compound/117944472#section=2D-Structure
https://pubchem.ncbi.nlm.nih.gov/compound/117930055#section=2D-Structure
https://pubchem.ncbi.nlm.nih.gov/compound/117919356#section=2D-Structure
https://pubchem.ncbi.nlm.nih.gov/compound/117916617#section=2D-Structure
https://pubchem.ncbi.nlm.nih.gov/compound/117910858#section=2D-Structure
https://pubchem.ncbi.nlm.nih.gov/compound/117905672#section=2D-Structure
https://pubchem.ncbi.nlm.nih.gov/compound/11790515#section=2D-Structure
https://pubchem.ncbi.nlm.nih.gov/compound/117865681#section=2D-Structure
https://pubchem.ncbi.nlm.nih.gov/compound/117847673#section=2D-Structure
https://pubchem.ncbi.nlm.nih.gov/compound/117835372#section=2D-Structure
https://pubchem.ncbi.nlm.nih.gov/compound/117812641#section=2D-Structure
https://pubchem.ncbi.nlm.nih.gov/compound/117799040#section=2D-Structure
https://pubchem.ncbi.nlm.nih.gov/compound/117789442#section=2D-Structure
https://pubchem.ncbi.nlm.nih.gov/compound/117778389#section=2D-Structure
https://pubchem.ncbi.nlm.nih.gov/compound/117775768#section=2D-Structure
https://pubchem.ncbi.nlm.nih.gov/compound/11775830#section=2D-Structure
https://pubchem.ncbi.nlm.nih.gov/compound/117750349#section=2D-Structure
https://pubchem.ncbi.nlm.nih.gov/compound/117749321#section=2D-Structure
https://pubchem.ncbi.nlm.nih.gov/compound/117737847#section=2D-Structure
https://pubchem.ncbi.nlm.nih.gov/compound/117724372#section=2D-Structure
https://pubchem.ncbi.nlm.nih.gov/compound/117722732#section=2D-Structure
https://pubchem.ncbi.nlm.nih.gov/compound/117715456#section=2D-Structure
https://pubchem.ncbi.nlm.nih.gov/compound/117713587#section=2D-Structure
https://pubchem.ncbi.nlm.nih.gov/compound/117708816#section=2D-Structure
https://pubchem.ncbi.nlm.nih.gov/compound/117708794#section=2D-Structure
https://pubchem.ncbi.nlm.nih.gov/compound/117705367#section=2D-Structure
https://pubchem.ncbi.nlm.nih.gov/compound/11769737#section=2D-Structure
https://pubchem.ncbi.nlm.nih.gov/compound/117686428#section=2D-Structure
https://pubchem.ncbi.nlm.nih.gov/compound/117680189#section=2D-Structure
https://pubchem.ncbi.nlm.nih.gov/compound/117673259#section=2D-Structure
https://pubchem.ncbi.nlm.nih.gov/compound/117669906#section=2D-Structure
https://pubchem.ncbi.nlm.nih.gov/compound/117661083#section=2D-Structure
https://pubchem.ncbi.nlm.nih.gov/compound/117661082#section=2D-Structure
https://pubchem.ncbi.nlm.nih.gov/compound/117661080#section=2D-Structure
https://pubchem.ncbi.nlm.nih.gov/compound/117661074#section=2D-Structure
https://pubchem.ncbi.nlm.nih.gov/compound/117656110#section=2D-Structure
https://pubchem.ncbi.nlm.nih.gov/compound/117656008#section=2D-Structure
https://pubchem.ncbi.nlm.nih.gov/compound/117638827#section=2D-Structure
https://pubchem.ncbi.nlm.nih.gov/compound/117638825#section=2D-Structure
https://pubchem.ncbi.nlm.nih.gov/compound/117633098#section=2D-Structure
https://pubchem.ncbi.nlm.nih.gov/compound/117631621#section=2D-Structure
https://pubchem.ncbi.nlm.nih.gov/compound/117620631#section=2D-Structure
https://pubchem.ncbi.nlm.nih.gov/compound/11759246#section=2D-Structure
https://pubchem.ncbi.nlm.nih.gov/compound/11745256#section=2D-Structure
https://pubchem.ncbi.nlm.nih.gov/compound/11708211#section=2D-Structure
https://pubchem.ncbi.nlm.nih.gov/compound/117070536#section=2D-Structure
https://pubchem.ncbi.nlm.nih.gov/compound/117065553#section=2D-Structure
https://pubchem.ncbi.nlm.nih.gov/compound/117063329#section=2D-Structure
https://pubchem.ncbi.nlm.nih.gov/compound/11658656#section=2D-Structure
https://pubchem.ncbi.nlm.nih.gov/compound/11658496#section=2D-Structure
https://pubchem.ncbi.nlm.nih.gov/compound/11644155#section=2D-Structure
https://pubchem.ncbi.nlm.nih.gov/compound/11629843#section=2D-Structure
https://pubchem.ncbi.nlm.nih.gov/compound/11564663#section=2D-Structure
https://pubchem.ncbi.nlm.nih.gov/compound/11550271#section=2D-Structure
https://pubchem.ncbi.nlm.nih.gov/compound/115404241#section=2D-Structure

https://pubchem.ncbi.nlm.nih.gov/compound/115267#section=2D-Structure
https://pubchem.ncbi.nlm.nih.gov/compound/115100255#section=2D-Structure
https://pubchem.ncbi.nlm.nih.gov/compound/115021640#section=2D-Structure
https://pubchem.ncbi.nlm.nih.gov/compound/114834#section=2D-Structure
https://pubchem.ncbi.nlm.nih.gov/compound/11470508#section=2D-Structure
https://pubchem.ncbi.nlm.nih.gov/compound/11469229#section=2D-Structure
https://pubchem.ncbi.nlm.nih.gov/compound/11465516#section=2D-Structure
https://pubchem.ncbi.nlm.nih.gov/compound/11459516#section=2D-Structure
https://pubchem.ncbi.nlm.nih.gov/compound/11399115#section=2D-Structure
https://pubchem.ncbi.nlm.nih.gov/compound/11365890#section=2D-Structure
https://pubchem.ncbi.nlm.nih.gov/compound/11347493#section=2D-Structure
https://pubchem.ncbi.nlm.nih.gov/compound/11345378#section=2D-Structure
https://pubchem.ncbi.nlm.nih.gov/compound/11333049#section=2D-Structure
https://pubchem.ncbi.nlm.nih.gov/compound/1133285#section=2D-Structure
https://pubchem.ncbi.nlm.nih.gov/compound/1133283#section=2D-Structure
https://pubchem.ncbi.nlm.nih.gov/compound/11321373#section=2D-Structure
https://pubchem.ncbi.nlm.nih.gov/compound/11309618#section=2D-Structure
https://pubchem.ncbi.nlm.nih.gov/compound/11298783#section=2D-Structure
https://pubchem.ncbi.nlm.nih.gov/compound/11296#section=2D-Structure
https://pubchem.ncbi.nlm.nih.gov/compound/11287900#section=2D-Structure
https://pubchem.ncbi.nlm.nih.gov/compound/11287111#section=2D-Structure
https://pubchem.ncbi.nlm.nih.gov/compound/11275193#section=2D-Structure
https://pubchem.ncbi.nlm.nih.gov/compound/11253597#section=2D-Structure
https://pubchem.ncbi.nlm.nih.gov/compound/11240568#section=2D-Structure
https://pubchem.ncbi.nlm.nih.gov/compound/11217357#section=2D-Structure
https://pubchem.ncbi.nlm.nih.gov/compound/11184075#section=2D-Structure
https://pubchem.ncbi.nlm.nih.gov/compound/11139396#section=2D-Structure
https://pubchem.ncbi.nlm.nih.gov/compound/11138569#section=2D-Structure
https://pubchem.ncbi.nlm.nih.gov/compound/11137842#section=2D-Structure
https://pubchem.ncbi.nlm.nih.gov/compound/11137285#section=2D-Structure
https://pubchem.ncbi.nlm.nih.gov/compound/11128433#section=2D-Structure
https://pubchem.ncbi.nlm.nih.gov/compound/11127722#section=2D-Structure
https://pubchem.ncbi.nlm.nih.gov/compound/11107144#section=2D-Structure
https://pubchem.ncbi.nlm.nih.gov/compound/11105623#section=2D-Structure
https://pubchem.ncbi.nlm.nih.gov/compound/11094993#section=2D-Structure
https://pubchem.ncbi.nlm.nih.gov/compound/11094595#section=2D-Structure
https://pubchem.ncbi.nlm.nih.gov/compound/11084504#section=2D-Structure
https://pubchem.ncbi.nlm.nih.gov/compound/11077#section=2D-Structure
https://pubchem.ncbi.nlm.nih.gov/compound/11075748#section=2D-Structure
https://pubchem.ncbi.nlm.nih.gov/compound/11062511#section=2D-Structure
https://pubchem.ncbi.nlm.nih.gov/compound/11061346#section=2D-Structure
https://pubchem.ncbi.nlm.nih.gov/compound/11053926#section=2D-Structure
https://pubchem.ncbi.nlm.nih.gov/compound/11053556#section=2D-Structure
https://pubchem.ncbi.nlm.nih.gov/compound/11052627#section=2D-Structure
https://pubchem.ncbi.nlm.nih.gov/compound/11043383#section=2D-Structure
https://pubchem.ncbi.nlm.nih.gov/compound/11042652#section=2D-Structure
https://pubchem.ncbi.nlm.nih.gov/compound/11042540#section=2D-Structure
https://pubchem.ncbi.nlm.nih.gov/compound/11031068#section=2D-Structure
https://pubchem.ncbi.nlm.nih.gov/compound/11029816#section=2D-Structure
https://pubchem.ncbi.nlm.nih.gov/compound/11021486#section=2D-Structure
https://pubchem.ncbi.nlm.nih.gov/compound/110204814#section=2D-Structure
https://pubchem.ncbi.nlm.nih.gov/compound/110193753#section=2D-Structure
https://pubchem.ncbi.nlm.nih.gov/compound/110167672#section=2D-Structure
https://pubchem.ncbi.nlm.nih.gov/compound/10997191#section=2D-Structure
https://pubchem.ncbi.nlm.nih.gov/compound/10977144#section=2D-Structure

https://pubchem.ncbi.nlm.nih.gov/compound/10976606#section=2D-Structure
https://pubchem.ncbi.nlm.nih.gov/compound/10976431#section=2D-Structure
https://pubchem.ncbi.nlm.nih.gov/compound/10975908#section=2D-Structure
https://pubchem.ncbi.nlm.nih.gov/compound/10975475#section=2D-Structure
https://pubchem.ncbi.nlm.nih.gov/compound/10967743#section=2D-Structure
https://pubchem.ncbi.nlm.nih.gov/compound/10964909#section=2D-Structure
https://pubchem.ncbi.nlm.nih.gov/compound/10954637#section=2D-Structure
https://pubchem.ncbi.nlm.nih.gov/compound/10943142#section=2D-Structure
https://pubchem.ncbi.nlm.nih.gov/compound/10921228#section=2D-Structure
https://pubchem.ncbi.nlm.nih.gov/compound/10913934#section=2D-Structure
https://pubchem.ncbi.nlm.nih.gov/compound/10912058#section=2D-Structure
https://pubchem.ncbi.nlm.nih.gov/compound/10909510#section=2D-Structure
https://pubchem.ncbi.nlm.nih.gov/compound/10900478#section=2D-Structure
https://pubchem.ncbi.nlm.nih.gov/compound/10879706#section=2D-Structure
https://pubchem.ncbi.nlm.nih.gov/compound/10878384#section=2D-Structure
https://pubchem.ncbi.nlm.nih.gov/compound/10878094#section=2D-Structure
https://pubchem.ncbi.nlm.nih.gov/compound/10868135#section=2D-Structure
https://pubchem.ncbi.nlm.nih.gov/compound/10867718#section=2D-Structure
https://pubchem.ncbi.nlm.nih.gov/compound/10866368#section=2D-Structure
https://pubchem.ncbi.nlm.nih.gov/compound/10858186#section=2D-Structure
https://pubchem.ncbi.nlm.nih.gov/compound/10857128#section=2D-Structure
https://pubchem.ncbi.nlm.nih.gov/compound/10857095#section=2D-Structure
https://pubchem.ncbi.nlm.nih.gov/compound/10846924#section=2D-Structure
https://pubchem.ncbi.nlm.nih.gov/compound/10846335#section=2D-Structure
https://pubchem.ncbi.nlm.nih.gov/compound/10844579#section=2D-Structure
https://pubchem.ncbi.nlm.nih.gov/compound/10825155#section=2D-Structure
https://pubchem.ncbi.nlm.nih.gov/compound/10823682#section=2D-Structure
https://pubchem.ncbi.nlm.nih.gov/compound/10798814#section=2D-Structure
https://pubchem.ncbi.nlm.nih.gov/compound/10798079#section=2D-Structure
https://pubchem.ncbi.nlm.nih.gov/compound/10797486#section=2D-Structure
https://pubchem.ncbi.nlm.nih.gov/compound/10797474#section=2D-Structure
https://pubchem.ncbi.nlm.nih.gov/compound/10789#section=2D-Structure
https://pubchem.ncbi.nlm.nih.gov/compound/10777111#section=2D-Structure
https://pubchem.ncbi.nlm.nih.gov/compound/10776608#section=2D-Structure
https://pubchem.ncbi.nlm.nih.gov/compound/10774430#section=2D-Structure
https://pubchem.ncbi.nlm.nih.gov/compound/10774391#section=2D-Structure
https://pubchem.ncbi.nlm.nih.gov/compound/10773697#section=2D-Structure
https://pubchem.ncbi.nlm.nih.gov/compound/10773519#section=2D-Structure
https://pubchem.ncbi.nlm.nih.gov/compound/10773252#section=2D-Structure
https://pubchem.ncbi.nlm.nih.gov/compound/10773217#section=2D-Structure
https://pubchem.ncbi.nlm.nih.gov/compound/10752209#section=2D-Structure
https://pubchem.ncbi.nlm.nih.gov/compound/10749358#section=2D-Structure
https://pubchem.ncbi.nlm.nih.gov/compound/10706398#section=2D-Structure
https://pubchem.ncbi.nlm.nih.gov/compound/10703868#section=2D-Structure
https://pubchem.ncbi.nlm.nih.gov/compound/10702691#section=2D-Structure
https://pubchem.ncbi.nlm.nih.gov/compound/10702096#section=2D-Structure
https://pubchem.ncbi.nlm.nih.gov/compound/10679556#section=2D-Structure
https://pubchem.ncbi.nlm.nih.gov/compound/10679516#section=2D-Structure
https://pubchem.ncbi.nlm.nih.gov/compound/10679471#section=2D-Structure
https://pubchem.ncbi.nlm.nih.gov/compound/10678974#section=2D-Structure
https://pubchem.ncbi.nlm.nih.gov/compound/10657180#section=2D-Structure
https://pubchem.ncbi.nlm.nih.gov/compound/10654259#section=2D-Structure
https://pubchem.ncbi.nlm.nih.gov/compound/10635544#section=2D-Structure
https://pubchem.ncbi.nlm.nih.gov/compound/10633462#section=2D-Structure
https://pubchem.ncbi.nlm.nih.gov/compound/10632954#section=2D-Structure

https://pubchem.ncbi.nlm.nih.gov/compound/10631928#section=2D-Structure
https://pubchem.ncbi.nlm.nih.gov/compound/10631134#section=2D-Structure
https://pubchem.ncbi.nlm.nih.gov/compound/10630853#section=2D-Structure
https://pubchem.ncbi.nlm.nih.gov/compound/10630644#section=2D-Structure
https://pubchem.ncbi.nlm.nih.gov/compound/10610397#section=2D-Structure
https://pubchem.ncbi.nlm.nih.gov/compound/10587532#section=2D-Structure
https://pubchem.ncbi.nlm.nih.gov/compound/10584872#section=2D-Structure
https://pubchem.ncbi.nlm.nih.gov/compound/10584553#section=2D-Structure
https://pubchem.ncbi.nlm.nih.gov/compound/10584254#section=2D-Structure
https://pubchem.ncbi.nlm.nih.gov/compound/10560026#section=2D-Structure
https://pubchem.ncbi.nlm.nih.gov/compound/10559939#section=2D-Structure
https://pubchem.ncbi.nlm.nih.gov/compound/105522769#section=2D-Structure
https://pubchem.ncbi.nlm.nih.gov/compound/105522767#section=2D-Structure
https://pubchem.ncbi.nlm.nih.gov/compound/105490439#section=2D-Structure
https://pubchem.ncbi.nlm.nih.gov/compound/105487475#section=2D-Structure
https://pubchem.ncbi.nlm.nih.gov/compound/105487474#section=2D-Structure
https://pubchem.ncbi.nlm.nih.gov/compound/105486228#section=2D-Structure
https://pubchem.ncbi.nlm.nih.gov/compound/105486227#section=2D-Structure
https://pubchem.ncbi.nlm.nih.gov/compound/105455899#section=2D-Structure
https://pubchem.ncbi.nlm.nih.gov/compound/105455898#section=2D-Structure
https://pubchem.ncbi.nlm.nih.gov/compound/105454967#section=2D-Structure
https://pubchem.ncbi.nlm.nih.gov/compound/105430015#section=2D-Structure
https://pubchem.ncbi.nlm.nih.gov/compound/105430014#section=2D-Structure
https://pubchem.ncbi.nlm.nih.gov/compound/105429500#section=2D-Structure
https://pubchem.ncbi.nlm.nih.gov/compound/105429498#section=2D-Structure
https://pubchem.ncbi.nlm.nih.gov/compound/10539520#section=2D-Structure
https://pubchem.ncbi.nlm.nih.gov/compound/10536595#section=2D-Structure
https://pubchem.ncbi.nlm.nih.gov/compound/10536351#section=2D-Structure
https://pubchem.ncbi.nlm.nih.gov/compound/10535292#section=2D-Structure
https://pubchem.ncbi.nlm.nih.gov/compound/10514490#section=2D-Structure
https://pubchem.ncbi.nlm.nih.gov/compound/10512751#section=2D-Structure
https://pubchem.ncbi.nlm.nih.gov/compound/10512671#section=2D-Structure
https://pubchem.ncbi.nlm.nih.gov/compound/10512589#section=2D-Structure
https://pubchem.ncbi.nlm.nih.gov/compound/10512268#section=2D-Structure
https://pubchem.ncbi.nlm.nih.gov/compound/10511787#section=2D-Structure
https://pubchem.ncbi.nlm.nih.gov/compound/10493171#section=2D-Structure
https://pubchem.ncbi.nlm.nih.gov/compound/10490336#section=2D-Structure
https://pubchem.ncbi.nlm.nih.gov/compound/10488966#section=2D-Structure
https://pubchem.ncbi.nlm.nih.gov/compound/10488357#section=2D-Structure
https://pubchem.ncbi.nlm.nih.gov/compound/10466392#section=2D-Structure
https://pubchem.ncbi.nlm.nih.gov/compound/10465033#section=2D-Structure
https://pubchem.ncbi.nlm.nih.gov/compound/10444912#section=2D-Structure
https://pubchem.ncbi.nlm.nih.gov/compound/10442941#section=2D-Structure
https://pubchem.ncbi.nlm.nih.gov/compound/10442034#section=2D-Structure
https://pubchem.ncbi.nlm.nih.gov/compound/10422313#section=2D-Structure
https://pubchem.ncbi.nlm.nih.gov/compound/10419211#section=2D-Structure
https://pubchem.ncbi.nlm.nih.gov/compound/10400425#section=2D-Structure
https://pubchem.ncbi.nlm.nih.gov/compound/10399947#section=2D-Structure
https://pubchem.ncbi.nlm.nih.gov/compound/10398821#section=2D-Structure
https://pubchem.ncbi.nlm.nih.gov/compound/10397037#section=2D-Structure
https://pubchem.ncbi.nlm.nih.gov/compound/10377513#section=2D-Structure
https://pubchem.ncbi.nlm.nih.gov/compound/10353642#section=2D-Structure
https://pubchem.ncbi.nlm.nih.gov/compound/10352991#section=2D-Structure
https://pubchem.ncbi.nlm.nih.gov/compound/10352532#section=2D-Structure
https://pubchem.ncbi.nlm.nih.gov/compound/10352038#section=2D-Structure

https://pubchem.ncbi.nlm.nih.gov/compound/10351979#section=2D-Structure
https://pubchem.ncbi.nlm.nih.gov/compound/103351527#section=2D-Structure
https://pubchem.ncbi.nlm.nih.gov/compound/103351511#section=2D-Structure
https://pubchem.ncbi.nlm.nih.gov/compound/10310#section=2D-Structure
https://pubchem.ncbi.nlm.nih.gov/compound/102937#section=2D-Structure
https://pubchem.ncbi.nlm.nih.gov/compound/10264624#section=2D-Structure
https://pubchem.ncbi.nlm.nih.gov/compound/10264206#section=2D-Structure
https://pubchem.ncbi.nlm.nih.gov/compound/10262315#section=2D-Structure
https://pubchem.ncbi.nlm.nih.gov/compound/10261306#section=2D-Structure
https://pubchem.ncbi.nlm.nih.gov/compound/10261256#section=2D-Structure
https://pubchem.ncbi.nlm.nih.gov/compound/10261086#section=2D-Structure
https://pubchem.ncbi.nlm.nih.gov/compound/102593810#section=2D-Structure
https://pubchem.ncbi.nlm.nih.gov/compound/102585800#section=2D-Structure
https://pubchem.ncbi.nlm.nih.gov/compound/102573060#section=2D-Structure
https://pubchem.ncbi.nlm.nih.gov/compound/102531022#section=2D-Structure
https://pubchem.ncbi.nlm.nih.gov/compound/102531021#section=2D-Structure
https://pubchem.ncbi.nlm.nih.gov/compound/102509614#section=2D-Structure
https://pubchem.ncbi.nlm.nih.gov/compound/102502047#section=2D-Structure
https://pubchem.ncbi.nlm.nih.gov/compound/102499#section=2D-Structure
https://pubchem.ncbi.nlm.nih.gov/compound/102493706#section=2D-Structure
https://pubchem.ncbi.nlm.nih.gov/compound/102490122#section=2D-Structure
https://pubchem.ncbi.nlm.nih.gov/compound/102474087#section=2D-Structure
https://pubchem.ncbi.nlm.nih.gov/compound/102465443#section=2D-Structure
https://pubchem.ncbi.nlm.nih.gov/compound/102464506#section=2D-Structure
https://pubchem.ncbi.nlm.nih.gov/compound/102463082#section=2D-Structure
https://pubchem.ncbi.nlm.nih.gov/compound/102457222#section=2D-Structure
https://pubchem.ncbi.nlm.nih.gov/compound/102442598#section=2D-Structure
https://pubchem.ncbi.nlm.nih.gov/compound/102425178#section=2D-Structure
https://pubchem.ncbi.nlm.nih.gov/compound/10240957#section=2D-Structure
https://pubchem.ncbi.nlm.nih.gov/compound/102397607#section=2D-Structure
https://pubchem.ncbi.nlm.nih.gov/compound/102396813#section=2D-Structure
https://pubchem.ncbi.nlm.nih.gov/compound/102393575#section=2D-Structure
https://pubchem.ncbi.nlm.nih.gov/compound/102382281#section=2D-Structure
https://pubchem.ncbi.nlm.nih.gov/compound/102380398#section=2D-Structure
https://pubchem.ncbi.nlm.nih.gov/compound/102369755#section=2D-Structure
https://pubchem.ncbi.nlm.nih.gov/compound/102359467#section=2D-Structure
https://pubchem.ncbi.nlm.nih.gov/compound/102355889#section=2D-Structure
https://pubchem.ncbi.nlm.nih.gov/compound/102355465#section=2D-Structure
https://pubchem.ncbi.nlm.nih.gov/compound/102355464#section=2D-Structure
https://pubchem.ncbi.nlm.nih.gov/compound/102340619#section=2D-Structure
https://pubchem.ncbi.nlm.nih.gov/compound/102319468#section=2D-Structure
https://pubchem.ncbi.nlm.nih.gov/compound/102319466#section=2D-Structure
https://pubchem.ncbi.nlm.nih.gov/compound/102319464#section=2D-Structure
https://pubchem.ncbi.nlm.nih.gov/compound/102319385#section=2D-Structure
https://pubchem.ncbi.nlm.nih.gov/compound/102318613#section=2D-Structure
https://pubchem.ncbi.nlm.nih.gov/compound/102314308#section=2D-Structure
https://pubchem.ncbi.nlm.nih.gov/compound/102314306#section=2D-Structure
https://pubchem.ncbi.nlm.nih.gov/compound/102307903#section=2D-Structure
https://pubchem.ncbi.nlm.nih.gov/compound/102304569#section=2D-Structure
https://pubchem.ncbi.nlm.nih.gov/compound/102303742#section=2D-Structure
https://pubchem.ncbi.nlm.nih.gov/compound/102302918#section=2D-Structure
https://pubchem.ncbi.nlm.nih.gov/compound/102302150#section=2D-Structure
https://pubchem.ncbi.nlm.nih.gov/compound/102295325#section=2D-Structure
https://pubchem.ncbi.nlm.nih.gov/compound/102286203#section=2D-Structure
https://pubchem.ncbi.nlm.nih.gov/compound/102285820#section=2D-Structure

https://pubchem.ncbi.nlm.nih.gov/compound/102284347#section=2D-Structure
https://pubchem.ncbi.nlm.nih.gov/compound/102281878#section=2D-Structure
https://pubchem.ncbi.nlm.nih.gov/compound/102279245#section=2D-Structure
https://pubchem.ncbi.nlm.nih.gov/compound/102277141#section=2D-Structure
https://pubchem.ncbi.nlm.nih.gov/compound/102271310#section=2D-Structure
https://pubchem.ncbi.nlm.nih.gov/compound/102270539#section=2D-Structure
https://pubchem.ncbi.nlm.nih.gov/compound/102266909#section=2D-Structure
https://pubchem.ncbi.nlm.nih.gov/compound/102265537#section=2D-Structure
https://pubchem.ncbi.nlm.nih.gov/compound/102263896#section=2D-Structure
https://pubchem.ncbi.nlm.nih.gov/compound/102256177#section=2D-Structure
https://pubchem.ncbi.nlm.nih.gov/compound/102256172#section=2D-Structure
https://pubchem.ncbi.nlm.nih.gov/compound/102256170#section=2D-Structure
https://pubchem.ncbi.nlm.nih.gov/compound/102247400#section=2D-Structure
https://pubchem.ncbi.nlm.nih.gov/compound/102245858#section=2D-Structure
https://pubchem.ncbi.nlm.nih.gov/compound/102241193#section=2D-Structure
https://pubchem.ncbi.nlm.nih.gov/compound/102241133#section=2D-Structure
https://pubchem.ncbi.nlm.nih.gov/compound/102226965#section=2D-Structure
https://pubchem.ncbi.nlm.nih.gov/compound/102225308#section=2D-Structure
https://pubchem.ncbi.nlm.nih.gov/compound/102219417#section=2D-Structure
https://pubchem.ncbi.nlm.nih.gov/compound/102216120#section=2D-Structure
https://pubchem.ncbi.nlm.nih.gov/compound/102202516#section=2D-Structure
https://pubchem.ncbi.nlm.nih.gov/compound/102201901#section=2D-Structure
https://pubchem.ncbi.nlm.nih.gov/compound/102201898#section=2D-Structure
https://pubchem.ncbi.nlm.nih.gov/compound/102188272#section=2D-Structure
https://pubchem.ncbi.nlm.nih.gov/compound/102175662#section=2D-Structure
https://pubchem.ncbi.nlm.nih.gov/compound/102170912#section=2D-Structure
https://pubchem.ncbi.nlm.nih.gov/compound/102165918#section=2D-Structure
https://pubchem.ncbi.nlm.nih.gov/compound/102161376#section=2D-Structure
https://pubchem.ncbi.nlm.nih.gov/compound/102148866#section=2D-Structure
https://pubchem.ncbi.nlm.nih.gov/compound/102139524#section=2D-Structure
https://pubchem.ncbi.nlm.nih.gov/compound/102133773#section=2D-Structure
https://pubchem.ncbi.nlm.nih.gov/compound/102122428#section=2D-Structure
https://pubchem.ncbi.nlm.nih.gov/compound/102115932#section=2D-Structure
https://pubchem.ncbi.nlm.nih.gov/compound/102098703#section=2D-Structure
https://pubchem.ncbi.nlm.nih.gov/compound/102098702#section=2D-Structure
https://pubchem.ncbi.nlm.nih.gov/compound/102097985#section=2D-Structure
https://pubchem.ncbi.nlm.nih.gov/compound/102093590#section=2D-Structure
https://pubchem.ncbi.nlm.nih.gov/compound/102085008#section=2D-Structure
https://pubchem.ncbi.nlm.nih.gov/compound/102085007#section=2D-Structure
https://pubchem.ncbi.nlm.nih.gov/compound/102074480#section=2D-Structure
https://pubchem.ncbi.nlm.nih.gov/compound/102074195#section=2D-Structure
https://pubchem.ncbi.nlm.nih.gov/compound/102070804#section=2D-Structure
https://pubchem.ncbi.nlm.nih.gov/compound/102069602#section=2D-Structure
https://pubchem.ncbi.nlm.nih.gov/compound/102066297#section=2D-Structure
https://pubchem.ncbi.nlm.nih.gov/compound/102053453#section=2D-Structure
https://pubchem.ncbi.nlm.nih.gov/compound/102042075#section=2D-Structure
https://pubchem.ncbi.nlm.nih.gov/compound/102042009#section=2D-Structure
https://pubchem.ncbi.nlm.nih.gov/compound/102035093#section=2D-Structure
https://pubchem.ncbi.nlm.nih.gov/compound/102033681#section=2D-Structure
https://pubchem.ncbi.nlm.nih.gov/compound/102016826#section=2D-Structure
https://pubchem.ncbi.nlm.nih.gov/compound/102015697#section=2D-Structure
https://pubchem.ncbi.nlm.nih.gov/compound/102008808#section=2D-Structure
https://pubchem.ncbi.nlm.nih.gov/compound/102005616#section=2D-Structure
https://pubchem.ncbi.nlm.nih.gov/compound/102005615#section=2D-Structure
https://pubchem.ncbi.nlm.nih.gov/compound/101997629#section=2D-Structure

https://pubchem.ncbi.nlm.nih.gov/compound/101990473#section=2D-Structure
https://pubchem.ncbi.nlm.nih.gov/compound/101973314#section=2D-Structure
https://pubchem.ncbi.nlm.nih.gov/compound/101966846#section=2D-Structure
https://pubchem.ncbi.nlm.nih.gov/compound/101948633#section=2D-Structure
https://pubchem.ncbi.nlm.nih.gov/compound/101946074#section=2D-Structure
https://pubchem.ncbi.nlm.nih.gov/compound/101939891#section=2D-Structure
https://pubchem.ncbi.nlm.nih.gov/compound/101931886#section=2D-Structure
https://pubchem.ncbi.nlm.nih.gov/compound/101931882#section=2D-Structure
https://pubchem.ncbi.nlm.nih.gov/compound/101931880#section=2D-Structure
https://pubchem.ncbi.nlm.nih.gov/compound/101924032#section=2D-Structure
https://pubchem.ncbi.nlm.nih.gov/compound/101924029#section=2D-Structure
https://pubchem.ncbi.nlm.nih.gov/compound/101924027#section=2D-Structure
https://pubchem.ncbi.nlm.nih.gov/compound/101924025#section=2D-Structure
https://pubchem.ncbi.nlm.nih.gov/compound/101923583#section=2D-Structure
https://pubchem.ncbi.nlm.nih.gov/compound/101923582#section=2D-Structure
https://pubchem.ncbi.nlm.nih.gov/compound/101923581#section=2D-Structure
https://pubchem.ncbi.nlm.nih.gov/compound/101923579#section=2D-Structure
https://pubchem.ncbi.nlm.nih.gov/compound/101923578#section=2D-Structure
https://pubchem.ncbi.nlm.nih.gov/compound/101923577#section=2D-Structure
https://pubchem.ncbi.nlm.nih.gov/compound/101920552#section=2D-Structure
https://pubchem.ncbi.nlm.nih.gov/compound/101915176#section=2D-Structure
https://pubchem.ncbi.nlm.nih.gov/compound/101913864#section=2D-Structure
https://pubchem.ncbi.nlm.nih.gov/compound/101913863#section=2D-Structure
https://pubchem.ncbi.nlm.nih.gov/compound/101913861#section=2D-Structure
https://pubchem.ncbi.nlm.nih.gov/compound/101902360#section=2D-Structure
https://pubchem.ncbi.nlm.nih.gov/compound/101883260#section=2D-Structure
https://pubchem.ncbi.nlm.nih.gov/compound/101879864#section=2D-Structure
https://pubchem.ncbi.nlm.nih.gov/compound/101873530#section=2D-Structure
https://pubchem.ncbi.nlm.nih.gov/compound/101859662#section=2D-Structure
https://pubchem.ncbi.nlm.nih.gov/compound/101858347#section=2D-Structure
https://pubchem.ncbi.nlm.nih.gov/compound/101857073#section=2D-Structure
https://pubchem.ncbi.nlm.nih.gov/compound/101856458#section=2D-Structure
https://pubchem.ncbi.nlm.nih.gov/compound/101856455#section=2D-Structure
https://pubchem.ncbi.nlm.nih.gov/compound/101852242#section=2D-Structure
https://pubchem.ncbi.nlm.nih.gov/compound/101842642#section=2D-Structure
https://pubchem.ncbi.nlm.nih.gov/compound/101831516#section=2D-Structure
https://pubchem.ncbi.nlm.nih.gov/compound/101797771#section=2D-Structure
https://pubchem.ncbi.nlm.nih.gov/compound/101796833#section=2D-Structure
https://pubchem.ncbi.nlm.nih.gov/compound/101796341#section=2D-Structure
https://pubchem.ncbi.nlm.nih.gov/compound/101779649#section=2D-Structure
https://pubchem.ncbi.nlm.nih.gov/compound/101775511#section=2D-Structure
https://pubchem.ncbi.nlm.nih.gov/compound/101769393#section=2D-Structure
https://pubchem.ncbi.nlm.nih.gov/compound/101768327#section=2D-Structure
https://pubchem.ncbi.nlm.nih.gov/compound/101758595#section=2D-Structure
https://pubchem.ncbi.nlm.nih.gov/compound/101755937#section=2D-Structure
https://pubchem.ncbi.nlm.nih.gov/compound/101755546#section=2D-Structure
https://pubchem.ncbi.nlm.nih.gov/compound/101754273#section=2D-Structure
https://pubchem.ncbi.nlm.nih.gov/compound/101747560#section=2D-Structure
https://pubchem.ncbi.nlm.nih.gov/compound/101747003#section=2D-Structure
https://pubchem.ncbi.nlm.nih.gov/compound/101739411#section=2D-Structure
https://pubchem.ncbi.nlm.nih.gov/compound/101731396#section=2D-Structure
https://pubchem.ncbi.nlm.nih.gov/compound/101727618#section=2D-Structure
https://pubchem.ncbi.nlm.nih.gov/compound/101722067#section=2D-Structure
https://pubchem.ncbi.nlm.nih.gov/compound/101721183#section=2D-Structure
https://pubchem.ncbi.nlm.nih.gov/compound/101713890#section=2D-Structure

https://pubchem.ncbi.nlm.nih.gov/compound/101711745#section=2D-Structure
https://pubchem.ncbi.nlm.nih.gov/compound/101709386#section=2D-Structure
https://pubchem.ncbi.nlm.nih.gov/compound/101705742#section=2D-Structure
https://pubchem.ncbi.nlm.nih.gov/compound/101705043#section=2D-Structure
https://pubchem.ncbi.nlm.nih.gov/compound/101705042#section=2D-Structure
https://pubchem.ncbi.nlm.nih.gov/compound/101687411#section=2D-Structure
https://pubchem.ncbi.nlm.nih.gov/compound/101679114#section=2D-Structure
https://pubchem.ncbi.nlm.nih.gov/compound/101679113#section=2D-Structure
https://pubchem.ncbi.nlm.nih.gov/compound/101672519#section=2D-Structure
https://pubchem.ncbi.nlm.nih.gov/compound/101670329#section=2D-Structure
https://pubchem.ncbi.nlm.nih.gov/compound/101639591#section=2D-Structure
https://pubchem.ncbi.nlm.nih.gov/compound/101637305#section=2D-Structure
https://pubchem.ncbi.nlm.nih.gov/compound/101629773#section=2D-Structure
https://pubchem.ncbi.nlm.nih.gov/compound/101625207#section=2D-Structure
https://pubchem.ncbi.nlm.nih.gov/compound/101621796#section=2D-Structure
https://pubchem.ncbi.nlm.nih.gov/compound/101619741#section=2D-Structure
https://pubchem.ncbi.nlm.nih.gov/compound/101619504#section=2D-Structure
https://pubchem.ncbi.nlm.nih.gov/compound/101612704#section=2D-Structure
https://pubchem.ncbi.nlm.nih.gov/compound/101612654#section=2D-Structure
https://pubchem.ncbi.nlm.nih.gov/compound/101610907#section=2D-Structure
https://pubchem.ncbi.nlm.nih.gov/compound/101609931#section=2D-Structure
https://pubchem.ncbi.nlm.nih.gov/compound/101608861#section=2D-Structure
https://pubchem.ncbi.nlm.nih.gov/compound/101590082#section=2D-Structure
https://pubchem.ncbi.nlm.nih.gov/compound/101565192#section=2D-Structure
https://pubchem.ncbi.nlm.nih.gov/compound/101559489#section=2D-Structure
https://pubchem.ncbi.nlm.nih.gov/compound/101547144#section=2D-Structure
https://pubchem.ncbi.nlm.nih.gov/compound/10154343#section=2D-Structure
https://pubchem.ncbi.nlm.nih.gov/compound/101542362#section=2D-Structure
https://pubchem.ncbi.nlm.nih.gov/compound/101542359#section=2D-Structure
https://pubchem.ncbi.nlm.nih.gov/compound/10153995#section=2D-Structure
https://pubchem.ncbi.nlm.nih.gov/compound/101534643#section=2D-Structure
https://pubchem.ncbi.nlm.nih.gov/compound/101526414#section=2D-Structure
https://pubchem.ncbi.nlm.nih.gov/compound/101526036#section=2D-Structure
https://pubchem.ncbi.nlm.nih.gov/compound/101522162#section=2D-Structure
https://pubchem.ncbi.nlm.nih.gov/compound/101520118#section=2D-Structure
https://pubchem.ncbi.nlm.nih.gov/compound/101515908#section=2D-Structure
https://pubchem.ncbi.nlm.nih.gov/compound/101511101#section=2D-Structure
https://pubchem.ncbi.nlm.nih.gov/compound/101503793#section=2D-Structure
https://pubchem.ncbi.nlm.nih.gov/compound/101499485#section=2D-Structure
https://pubchem.ncbi.nlm.nih.gov/compound/101463836#section=2D-Structure
https://pubchem.ncbi.nlm.nih.gov/compound/101459342#section=2D-Structure
https://pubchem.ncbi.nlm.nih.gov/compound/101449998#section=2D-Structure
https://pubchem.ncbi.nlm.nih.gov/compound/101449997#section=2D-Structure
https://pubchem.ncbi.nlm.nih.gov/compound/101448445#section=2D-Structure
https://pubchem.ncbi.nlm.nih.gov/compound/101434855#section=2D-Structure
https://pubchem.ncbi.nlm.nih.gov/compound/101418284#section=2D-Structure
https://pubchem.ncbi.nlm.nih.gov/compound/101414491#section=2D-Structure
https://pubchem.ncbi.nlm.nih.gov/compound/101410254#section=2D-Structure
https://pubchem.ncbi.nlm.nih.gov/compound/101410252#section=2D-Structure
https://pubchem.ncbi.nlm.nih.gov/compound/101407804#section=2D-Structure
https://pubchem.ncbi.nlm.nih.gov/compound/101405296#section=2D-Structure
https://pubchem.ncbi.nlm.nih.gov/compound/101404883#section=2D-Structure
https://pubchem.ncbi.nlm.nih.gov/compound/101404539#section=2D-Structure
https://pubchem.ncbi.nlm.nih.gov/compound/101396162#section=2D-Structure
https://pubchem.ncbi.nlm.nih.gov/compound/101392731#section=2D-Structure

https://pubchem.ncbi.nlm.nih.gov/compound/101372191#section=2D-Structure
https://pubchem.ncbi.nlm.nih.gov/compound/101362649#section=2D-Structure
https://pubchem.ncbi.nlm.nih.gov/compound/101360604#section=2D-Structure
https://pubchem.ncbi.nlm.nih.gov/compound/101360374#section=2D-Structure
https://pubchem.ncbi.nlm.nih.gov/compound/101358942#section=2D-Structure
https://pubchem.ncbi.nlm.nih.gov/compound/101356166#section=2D-Structure
https://pubchem.ncbi.nlm.nih.gov/compound/101356165#section=2D-Structure
https://pubchem.ncbi.nlm.nih.gov/compound/101350324#section=2D-Structure
https://pubchem.ncbi.nlm.nih.gov/compound/101346876#section=2D-Structure
https://pubchem.ncbi.nlm.nih.gov/compound/101346874#section=2D-Structure
https://pubchem.ncbi.nlm.nih.gov/compound/101341967#section=2D-Structure
https://pubchem.ncbi.nlm.nih.gov/compound/101341965#section=2D-Structure
https://pubchem.ncbi.nlm.nih.gov/compound/101341964#section=2D-Structure
https://pubchem.ncbi.nlm.nih.gov/compound/101318478#section=2D-Structure
https://pubchem.ncbi.nlm.nih.gov/compound/101281794#section=2D-Structure
https://pubchem.ncbi.nlm.nih.gov/compound/101274369#section=2D-Structure
https://pubchem.ncbi.nlm.nih.gov/compound/101274367#section=2D-Structure
https://pubchem.ncbi.nlm.nih.gov/compound/101272043#section=2D-Structure
https://pubchem.ncbi.nlm.nih.gov/compound/101267496#section=2D-Structure
https://pubchem.ncbi.nlm.nih.gov/compound/101265298#section=2D-Structure
https://pubchem.ncbi.nlm.nih.gov/compound/101265296#section=2D-Structure
https://pubchem.ncbi.nlm.nih.gov/compound/101263914#section=2D-Structure
https://pubchem.ncbi.nlm.nih.gov/compound/101260113#section=2D-Structure
https://pubchem.ncbi.nlm.nih.gov/compound/101259311#section=2D-Structure
https://pubchem.ncbi.nlm.nih.gov/compound/101255354#section=2D-Structure
https://pubchem.ncbi.nlm.nih.gov/compound/101245093#section=2D-Structure
https://pubchem.ncbi.nlm.nih.gov/compound/101242378#section=2D-Structure
https://pubchem.ncbi.nlm.nih.gov/compound/101230559#section=2D-Structure
https://pubchem.ncbi.nlm.nih.gov/compound/101220633#section=2D-Structure
https://pubchem.ncbi.nlm.nih.gov/compound/101220571#section=2D-Structure
https://pubchem.ncbi.nlm.nih.gov/compound/101220570#section=2D-Structure
https://pubchem.ncbi.nlm.nih.gov/compound/101218213#section=2D-Structure
https://pubchem.ncbi.nlm.nih.gov/compound/101216924#section=2D-Structure
https://pubchem.ncbi.nlm.nih.gov/compound/101216917#section=2D-Structure
https://pubchem.ncbi.nlm.nih.gov/compound/101211877#section=2D-Structure
https://pubchem.ncbi.nlm.nih.gov/compound/101211875#section=2D-Structure
https://pubchem.ncbi.nlm.nih.gov/compound/101210294#section=2D-Structure
https://pubchem.ncbi.nlm.nih.gov/compound/101206933#section=2D-Structure
https://pubchem.ncbi.nlm.nih.gov/compound/101205987#section=2D-Structure
https://pubchem.ncbi.nlm.nih.gov/compound/101205985#section=2D-Structure
https://pubchem.ncbi.nlm.nih.gov/compound/101205984#section=2D-Structure
https://pubchem.ncbi.nlm.nih.gov/compound/101202288#section=2D-Structure
https://pubchem.ncbi.nlm.nih.gov/compound/101199438#section=2D-Structure
https://pubchem.ncbi.nlm.nih.gov/compound/101199437#section=2D-Structure
https://pubchem.ncbi.nlm.nih.gov/compound/101199101#section=2D-Structure
https://pubchem.ncbi.nlm.nih.gov/compound/101194405#section=2D-Structure
https://pubchem.ncbi.nlm.nih.gov/compound/101190147#section=2D-Structure
https://pubchem.ncbi.nlm.nih.gov/compound/101190144#section=2D-Structure
https://pubchem.ncbi.nlm.nih.gov/compound/101187366#section=2D-Structure
https://pubchem.ncbi.nlm.nih.gov/compound/101184681#section=2D-Structure
https://pubchem.ncbi.nlm.nih.gov/compound/101172776#section=2D-Structure
https://pubchem.ncbi.nlm.nih.gov/compound/101170853#section=2D-Structure
https://pubchem.ncbi.nlm.nih.gov/compound/101164660#section=2D-Structure
https://pubchem.ncbi.nlm.nih.gov/compound/101162452#section=2D-Structure
https://pubchem.ncbi.nlm.nih.gov/compound/101158161#section=2D-Structure

https://pubchem.ncbi.nlm.nih.gov/compound/101152737#section=2D-Structure
https://pubchem.ncbi.nlm.nih.gov/compound/101146729#section=2D-Structure
https://pubchem.ncbi.nlm.nih.gov/compound/101144661#section=2D-Structure
https://pubchem.ncbi.nlm.nih.gov/compound/101144660#section=2D-Structure
https://pubchem.ncbi.nlm.nih.gov/compound/101142904#section=2D-Structure
https://pubchem.ncbi.nlm.nih.gov/compound/101127232#section=2D-Structure
https://pubchem.ncbi.nlm.nih.gov/compound/101125919#section=2D-Structure
https://pubchem.ncbi.nlm.nih.gov/compound/101121960#section=2D-Structure
https://pubchem.ncbi.nlm.nih.gov/compound/101117837#section=2D-Structure
https://pubchem.ncbi.nlm.nih.gov/compound/101117684#section=2D-Structure
https://pubchem.ncbi.nlm.nih.gov/compound/101108378#section=2D-Structure
https://pubchem.ncbi.nlm.nih.gov/compound/101099464#section=2D-Structure
https://pubchem.ncbi.nlm.nih.gov/compound/101099214#section=2D-Structure
https://pubchem.ncbi.nlm.nih.gov/compound/101090788#section=2D-Structure
https://pubchem.ncbi.nlm.nih.gov/compound/101083012#section=2D-Structure
https://pubchem.ncbi.nlm.nih.gov/compound/101081692#section=2D-Structure
https://pubchem.ncbi.nlm.nih.gov/compound/101081691#section=2D-Structure
https://pubchem.ncbi.nlm.nih.gov/compound/101065151#section=2D-Structure
https://pubchem.ncbi.nlm.nih.gov/compound/101063671#section=2D-Structure
https://pubchem.ncbi.nlm.nih.gov/compound/101063670#section=2D-Structure
https://pubchem.ncbi.nlm.nih.gov/compound/101063665#section=2D-Structure
https://pubchem.ncbi.nlm.nih.gov/compound/10106236#section=2D-Structure
https://pubchem.ncbi.nlm.nih.gov/compound/101062059#section=2D-Structure
https://pubchem.ncbi.nlm.nih.gov/compound/101032884#section=2D-Structure
https://pubchem.ncbi.nlm.nih.gov/compound/101029865#section=2D-Structure
https://pubchem.ncbi.nlm.nih.gov/compound/101029864#section=2D-Structure
https://pubchem.ncbi.nlm.nih.gov/compound/101025272#section=2D-Structure
https://pubchem.ncbi.nlm.nih.gov/compound/101021274#section=2D-Structure
https://pubchem.ncbi.nlm.nih.gov/compound/101014635#section=2D-Structure
https://pubchem.ncbi.nlm.nih.gov/compound/101007978#section=2D-Structure
https://pubchem.ncbi.nlm.nih.gov/compound/101004123#section=2D-Structure
https://pubchem.ncbi.nlm.nih.gov/compound/100993361#section=2D-Structure
https://pubchem.ncbi.nlm.nih.gov/compound/100985901#section=2D-Structure
https://pubchem.ncbi.nlm.nih.gov/compound/100978596#section=2D-Structure
https://pubchem.ncbi.nlm.nih.gov/compound/100973758#section=2D-Structure
https://pubchem.ncbi.nlm.nih.gov/compound/100972119#section=2D-Structure
https://pubchem.ncbi.nlm.nih.gov/compound/100968469#section=2D-Structure
https://pubchem.ncbi.nlm.nih.gov/compound/100944523#section=2D-Structure
https://pubchem.ncbi.nlm.nih.gov/compound/100936820#section=2D-Structure
https://pubchem.ncbi.nlm.nih.gov/compound/100936819#section=2D-Structure
https://pubchem.ncbi.nlm.nih.gov/compound/100936818#section=2D-Structure
https://pubchem.ncbi.nlm.nih.gov/compound/100933534#section=2D-Structure
https://pubchem.ncbi.nlm.nih.gov/compound/100925558#section=2D-Structure
https://pubchem.ncbi.nlm.nih.gov/compound/100920056#section=2D-Structure
https://pubchem.ncbi.nlm.nih.gov/compound/100913757#section=2D-Structure
https://pubchem.ncbi.nlm.nih.gov/compound/10083083#section=2D-Structure
https://pubchem.ncbi.nlm.nih.gov/compound/10081894#section=2D-Structure
https://pubchem.ncbi.nlm.nih.gov/compound/10081558#section=2D-Structure
https://pubchem.ncbi.nlm.nih.gov/compound/10011906#section=2D-Structure
